# Discovery of Two Highly Selective Structurally Orthogonal Chemical Probes for Activin Receptor-like Kinases 1 and 2

**DOI:** 10.1021/acs.jmedchem.4c00629

**Published:** 2024-07-18

**Authors:** Václav Němec, Marek Remeš, Petr Beňovský, Michael C. Böck, Eliška Šranková, Jong Fu Wong, Julien Cros, Eleanor Williams, Lap Hang Tse, David Smil, Deeba Ensan, Methvin B. Isaac, Rima Al-Awar, Regina Gomolková, Vlad-Constantin Ursachi, Bohumil Fafílek, Zuzana Kahounová, Ráchel Víchová, Ondřej Vacek, Benedict-Tilman Berger, Carrow I. Wells, Cesear R. Corona, James D. Vasta, Matthew B. Robers, Pavel Krejci, Karel Souček, Alex N. Bullock, Stefan Knapp, Kamil Paruch

**Affiliations:** † Institute for Pharmaceutical Chemistry, Structural Genomics Consortium, Johann Wolfgang Goethe-University, Max-von-Laue-Strasse 9, Frankfurt am Main, 60438, Germany; ‡ Department of Chemistry, Masaryk University, Brno 625 00, Czech Republic; § Centre for Medicines Discovery, Nuffield Department of Medicine, University of Oxford, Oxford OX3 7FZ, U.K.; ∥ Drug Discovery Program, Ontario Institute for Cancer Research, 661 University Avenue, Toronto, Ontario M5G 0A3, Canada; ⬡ Department of Pharmacology and Toxicology, University of Toronto, Toronto, Ontario M5S 1A8, Canada; & Department of Biology, Faculty of Medicine, Masaryk University, 625 00 Brno, Czech Republic; $ Institute of Animal Physiology and Genetics of the Czech Academy of Sciences, 602 00 Brno, Czech Republic; ▲ International Clinical Research Center, St. Anne’s University Hospital, 602 00 Brno, Czech Republic; ¶ Institute of Biophysics of the Czech Academy of Sciences, Královopolská 135, 612 00 Brno Czech Republic; ⊥ Structural Genomics Consortium, UNC Eshelman School of Pharmacy, The University of North Carolina at Chapel Hill, Chapel Hill, North Carolina 27599, United States; ▽ Promega Corporation, Madison, Wisconsin 53716, United States

## Abstract

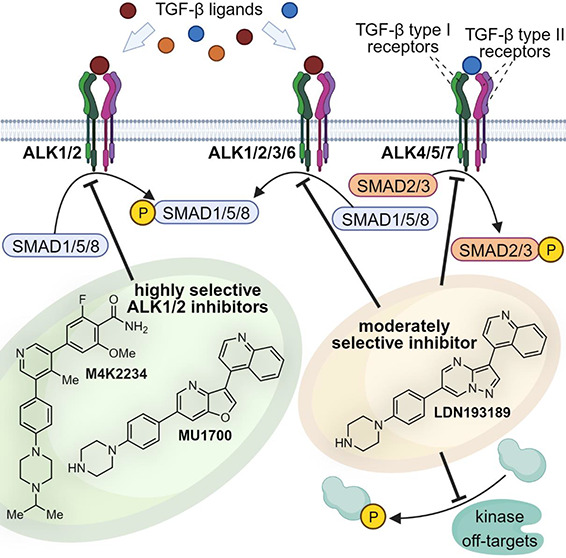

Activin receptor-like kinases 1–7 (ALK1–7) regulate a complex network of SMAD-independent as well as SMAD-dependent signaling pathways. One of the widely used inhibitors for functional investigations of these processes, in particular for bone morphogenetic protein (BMP) signaling, is **LDN-193189**. However, **LDN-193189** has insufficient kinome-wide selectivity complicating its use in cellular target validation assays. Herein, we report the identification and comprehensive characterization of two chemically distinct highly selective inhibitors of ALK1 and ALK2, **M4K2234** and **MU1700**, along with their negative controls. We show that both **MU1700** and **M4K2234** efficiently block the BMP pathway via selective in cellulo inhibition of ALK1/2 kinases and exhibit favorable in vivo profiles in mice. **MU1700** is highly brain penetrant and shows remarkably high accumulation in the brain. These high-quality orthogonal chemical probes offer the selectivity required to become widely used tools for in vitro and in vivo investigation of BMP signaling.

## Introduction

Protein kinases have become prominent drug targets over the past three decades.^1−3^ Currently, FDA-approved kinase inhibitors target approximately 20% of the human kinome,^4^ but there are still many protein kinases whose biological function and their roles in disease development are poorly understood.^5,6^ One of the reasons for this is the lack of high-quality chemical tools, so-called chemical probes, that would allow exploration of the corresponding biological processes at molecular level.^7−9^ To minimize the compound-specific off-target effects, it is thus highly advantageous to use at least two different chemical probes, along with their (structurally similar) negative control compounds.^7−9^

ALK1–7 are receptor serine/threonine protein kinases belonging to the group of tyrosine kinase-like kinases (TLKs), encoded by the *ACVRL1*, *ACVR1*, *BMPR1A*, *ACVR1B*, *TGFBR1*, *BMPR1B* and *ACVR1C* genes, respectively. ALK1–7 consist of extracellular, transmembrane, glycine-serine rich (GS), and kinase domains.^10,11^ These kinases belong to the transforming growth factor β (TGF-β) type I receptor family and function in heterotetrameric complexes with TGF-β type II receptors stabilized by the binding of ligands of the TGF-β superfamily. Upon formation of the heterotetrameric complex and ligand binding, the constitutively active TGF-β type II receptors phosphorylate TGF-β type I receptors (ALK1–7) on several Ser/Thr residues located in their GS domains resulting in their kinase activation.^11,12^ ALK1–7 receptors mediate SMAD-independent as well as SMAD-dependent signaling pathways. SMAD-dependent signaling has two distinct branches: one is canonically mediated by ALK4/5/7 kinases (TGFβ-activin-nodal branch) that phosphorylate SMAD2 and SMAD3, while the other pathway, called bone morphogenetic protein (BMP) signaling, relies on ALK1/2/3/6-mediated phosphorylation of SMAD1, SMAD5 and SMAD8. ALK family members phosphorylate SMAD2/3 or SMAD1/5/8 at two Ser residues located at the C termini. This phosphorylation events trigger complex formation with SMAD4. These complexes translocate into the nucleus where they regulate the transcription of target genes.^11,13^ Of note, the two branches of SMAD-dependent signaling are not completely separated, as there are ligands (e.g., activins) that are able to bind and activate receptors of both subtypes under certain conditions.^14^ Furthermore, TGF-β-induced epithelial-to-mesenchymal transition requires signaling via both the SMAD3 and SMAD1/5 pathways.^15^ In this context, TGF-β stimulates its canonical receptor ALK5 to phosphorylate ALK2 leading to ALK2-mediated phosphorylation of SMAD1/5/8 as well as ALK5-mediated phosphorylation of SMAD2/3.^15^ On the other hand, ALK2 upregulates ALK1 in endothelial cells in response to high-density lipoproteins, which subsequently promotes survival by inducing expression of VEGF-A via ALK1 signaling.^16^ Clearly, there is a certain level of interplay and compensatory crosstalk between ALK1- and ALK2-mediated signaling, as well as between the two branches of SMAD signaling. As discussed below, understanding of the crosstalk between the branches of ALK1–7 mediated signaling, as well as understanding the influence of individual ALK1–7 kinases to the signaling pathways, is fundamental for the development of rational treatment strategies.

ALK1 exhibits high cell-type specificity, particularly for arterial endothelial cells,^17^ and ALK1 signaling activates the proliferation of endothelial cells and orchestrates angiogenesis and maintenance of vascular quiescence. Dysregulation of this pathway is known to be linked to cardiovascular diseases. For instance, heterozygous loss-of-function mutations in ALK1 or ENG result in the development of hereditary hemorrhagic telangiectasia (HHT).^18^ Furthermore, targeting the LDL/ALK1 interaction has been suggested as a viable approach for prevention of atherosclerosis.^19^ Since activation of ALK1 is involved in the development of blood vessels, ALK1 inhibition is currently being evaluated in antiangiogenic therapy.^20^ In addition, ALK1 regulates extracellular matrix deposition which, upon dysregulation, can promote the development of fibrosis.^21^ Deeper understanding of ALK1 functions may thus contribute to more efficient treatment of cardiovascular diseases, cancers, and other diseases driven by ALK1-mediated signaling.^17,22,23^

ALK2 has emerged as a promising therapeutic target for the treatment of fibrodysplasia ossificans progressiva (FOP) and diffuse midline glioma (DMG).^10,24^ Gain of function mutations in ALK2 have been linked to the pathogenesis of both FOP and DMG.^10,24,25^ These mutations in the GS and kinase domain disrupt the inactive conformation of ALK2, as shown by its reduced interaction with the negative regulator FKBP12.^12^ Therefore, the disease mutations enhance activation of the ALK2 kinase by type II receptors.^26,12^ Whole genome sequencing revealed seven ALK2 (*ACVR1*) somatic mutations in DMG patients,^27−30^ including two mutations in the GS domain (R206H, Q207E) and another five identified in the kinase domain (R258G, G328E, G328 V, G328W and G356D). Approximately 25–30% of H3K27-altered DMG patients carry ALK2 (*ACVR1*) mutations, which makes ALK2 one of the most frequently mutated genes in DMG.^30−33^ It has been shown that shRNA knockdown of ALK2 R206H mutants inhibits proliferation of the HSJD-DIPG-007 cell line.^32^ Similarly, 13 gain of function ALK2 mutations have been identified in FOP patients, including six that are identical to those observed in DMG. Five of the mutations are located in the GS domain while eight mutations occur in the kinase domain.^10,34^ The most common mutation in FOP patients is ALK2 R206H (located in the GS domain), occurring in 95% of cases. It has been shown that ALK2 R206H knock-in mice develop a phenotype that is characteristic for FOP, including great toe malformation and heterotopic ossification, which indicates that the ALK2 R206H mutation drives the development of FOP.^35^ To date, a large number of clinical trials targeting DMG with radiotherapy, biologics, chemotherapeutics and their combinations have failed.^36,37^ This indicates that there is a clear demand for an innovative therapeutic approach such as the utilization of small molecules with a novel mechanism of action.^38,39^

Dorsomorphin has been the first widely used tool compound for investigation of BMP signaling.^40^ Although it has been published as an inhibitor of ALK2/3/6 and AMPK, it inhibits numerous additional kinases across the kinome.^41^ Several dorsomorphin analogues with a conserved central pyrazolo[1,5-*a*]pyrimidine scaffold have been identified with improved potency and selectivity.^42^ Of those, **LDN-193189** and **LDN-212854** are widely used tool compounds based on either greatly increased potency toward type I receptors ALK1/2/3/6 (**LDN-193189**) or improved selectivity for BMP type I receptors over ALK4/5 (**LDN-212854**).^43^ However, the kinome-wide selectivity profiles of **LDN-193189** and **LDN-212854** are not sufficient for mechanistic studies on BMP dependent signaling as a significant number of off-targets remain.^9,44^ Herein, we used LDN-193189 as a starting point for the development of a highly selective ALK1/2 inhibitor as well as a matching negative control. In addition, we comprehensively characterized an earlier inhibitor series derived from the pyridine-based scaffold of K02288 to define **M4K2234** as another highly quality ALK1/2 inhibitor that is structurally diverse.^42,45−47^ We demonstrated that the developed chemical probes are highly selective using a panel of TGF-β family receptor kinases mediated in vitro and cellular signaling assays. The presented inhibitors represent a compound set that allows mechanistic studies on ALK1/2 receptors and support their exploitation as targets for new ALK1/2 treatment strategies.

## RESULTS AND DISCUSSION

### Development of **MU1700**

**Figure 1:**
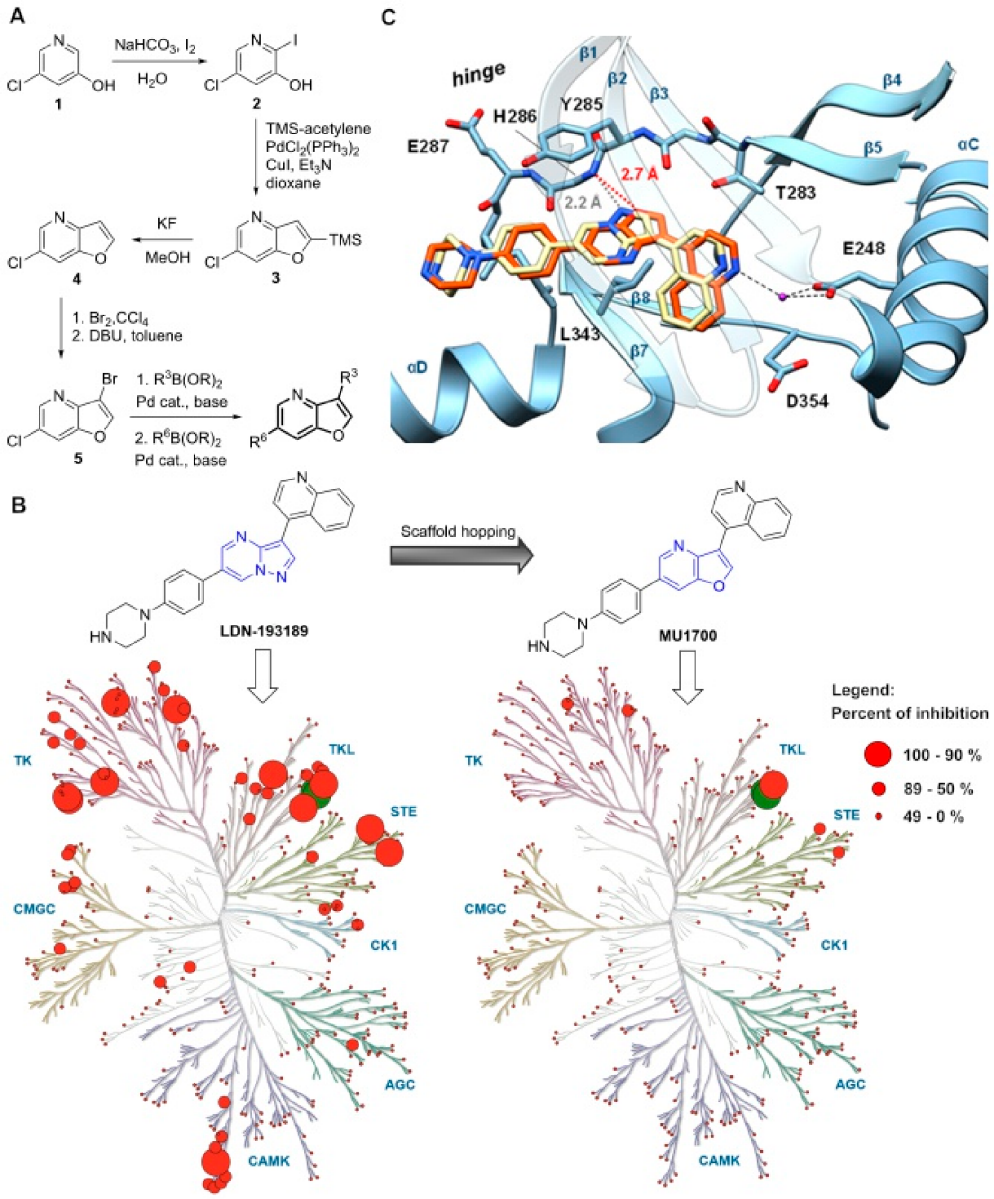
(**A**) Synthetic route leading to **MU1700** and its analogues. (**B**) Bioisosteric replacement of furo[3,2-*b*]pyridine for pyrazolo[1,5-*a*]pyrimidine resulted in a remarkably improved selectivity profile. **LDN-193189** and **MU1700** were screened in a panel of 369 protein kinases at 1 μM concentration (Reaction Biology). Filled circles represent percentage of inhibition; ALK1 and ALK2 kinases are marked in green (the only significant red circle for **MU1700** is BMP receptor ALK6). (**C**) Superposition of the X-ray cocrystal structures of **LDN-193189** (green, PDB code: 3Q4U) and MU1700 (magenta, PDB code: 8POD) bound to the active site of ALK2.^42^

The previously available crystal structure of **LDN-193189** and ALK2^42^ revealed interaction between the pyrazole nitrogen atom of the pyrazolo[1,5-*a*]pyrimidine central scaffold and the hinge region (Figure 1). We hypothesized that bioisosteric replacement of the pyrazolo[1,5-*a*]pyrimidine with furo[3,2-*b*]pyridine might result in a weaker interaction with the highly conserved hinge region^48^ and thus provide an improvement in the kinome-wide selectivity. Interactions with the less conserved amino acid residues of the catalytic site should be preserved, including the water mediated hydrogen bond with the conserved lysine (K225) and the αC glutamate (E248) salt bridge.

In order to prepare the furo[3,2-*b*]pyridine analogue of **LDN-193189**, we developed a new synthetic route allowing for modular synthesis of 3,6-disubstituted furo[3,2-*b*]pyridines (Figure 1A). The key versatile intermediate **5** was prepared on gram scale in five steps from commercially available 5-chloropyridin-3-ol (**1**). **5** was then subjected to the two-step sequence of consecutive chemoselective Suzuki couplings, which allowed for a flexible elaboration of the position 3 of the furo[3,2-*b*]pyridine scaffold, and the following substitution of the position 6 (Figure 1A). This synthetic route was used to prepare the direct furo[3,2-*b*]pyridine based bioisostere of **LDN-193189** – compound **MU1700**, as well as its analogues described below.

Both compounds **MU1700** and **LDN-193189** were subjected to kinome-wide profiling in a panel of 369 human protein kinases at 1 μM concentration (Reaction Biology). While **LDN-193189** exhibited a rather promiscuous profile, **MU1700** showed a remarkably improved selectivity profile in which only ALK1/2 kinases were highly potently inhibited, with some weaker inhibition of BMP receptor ALK6 (Figure 1B and Table S1). We therefore conducted crystallization screens to obtain a structure of the **MU1700**-ALK2 complex. Diffracting crystals and a structure were obtained upon stabilization of ALK2 via formation of a complex with FKBP12 (PDB-ID: 8POD and Table S2). Superimposition of the cocrystal structures of **LDN-193189** and **MU1700** in ALK2 (Figure 1C), revealed that both inhibitors occupy the active site with similar binding modes. As predicted, one of the notable differences was the longer distance between the backbone nitrogen of His286 and the oxygen atom of **MU1700** (2.7 Å), compared to that of the analogous nitrogen atom of **LDN-193189** (2.2 Å). This structural difference indicated comparatively weaker hydrogen bonding between the hinge region of ALK2 and the central furopyridine scaffold of **MU1700**, which was in line with our design hypothesis.

Taking advantage of the modular synthetic route depicted in Figure 1, we prepared numerous analogues of **MU1700**, with the primary aim to identify a suitable negative control compound. As summarized in Table 1, modification/bioisosteric replacement of the quinoline moiety had a profound effect on the inhibitory activity against ALK1–6. Specifically, proper positioning of the nitrogen atom in the quinoline ring proved to be important for achieving optimal potency. This is in accordance with its observed water molecule-mediated interaction with the amino acid residues K235 and E248 (Figure 1C).

**Table 1:**
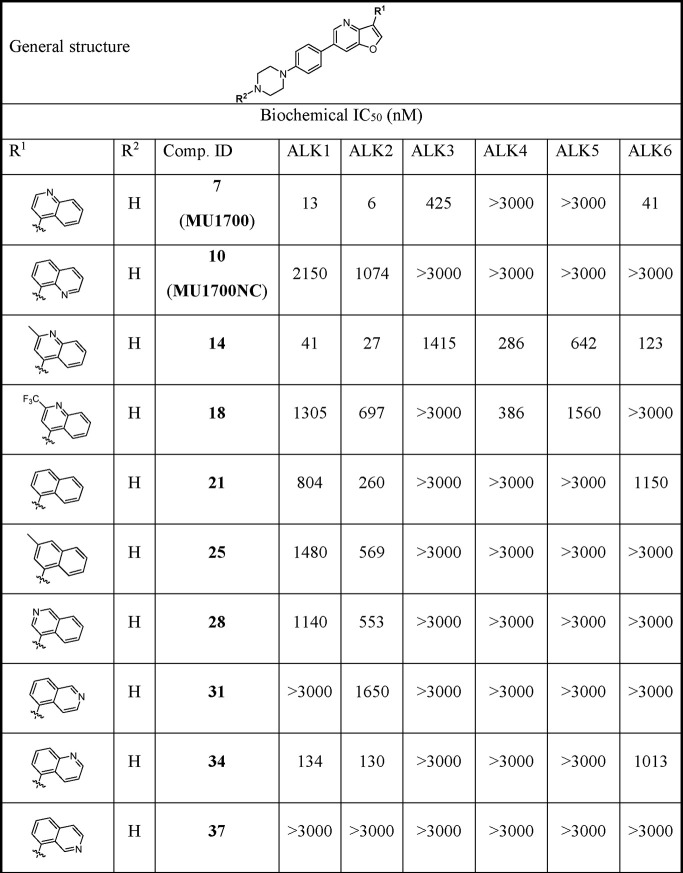

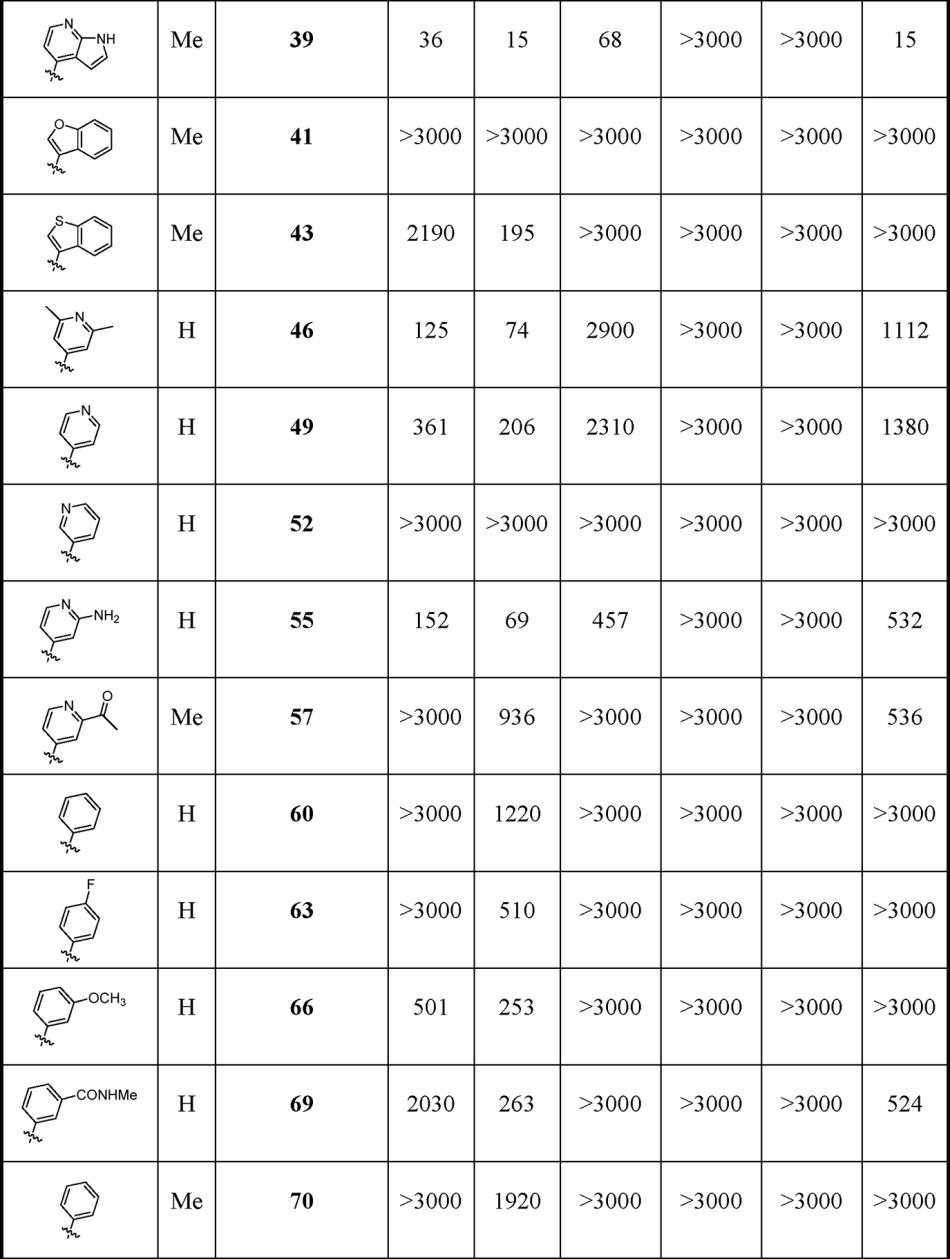

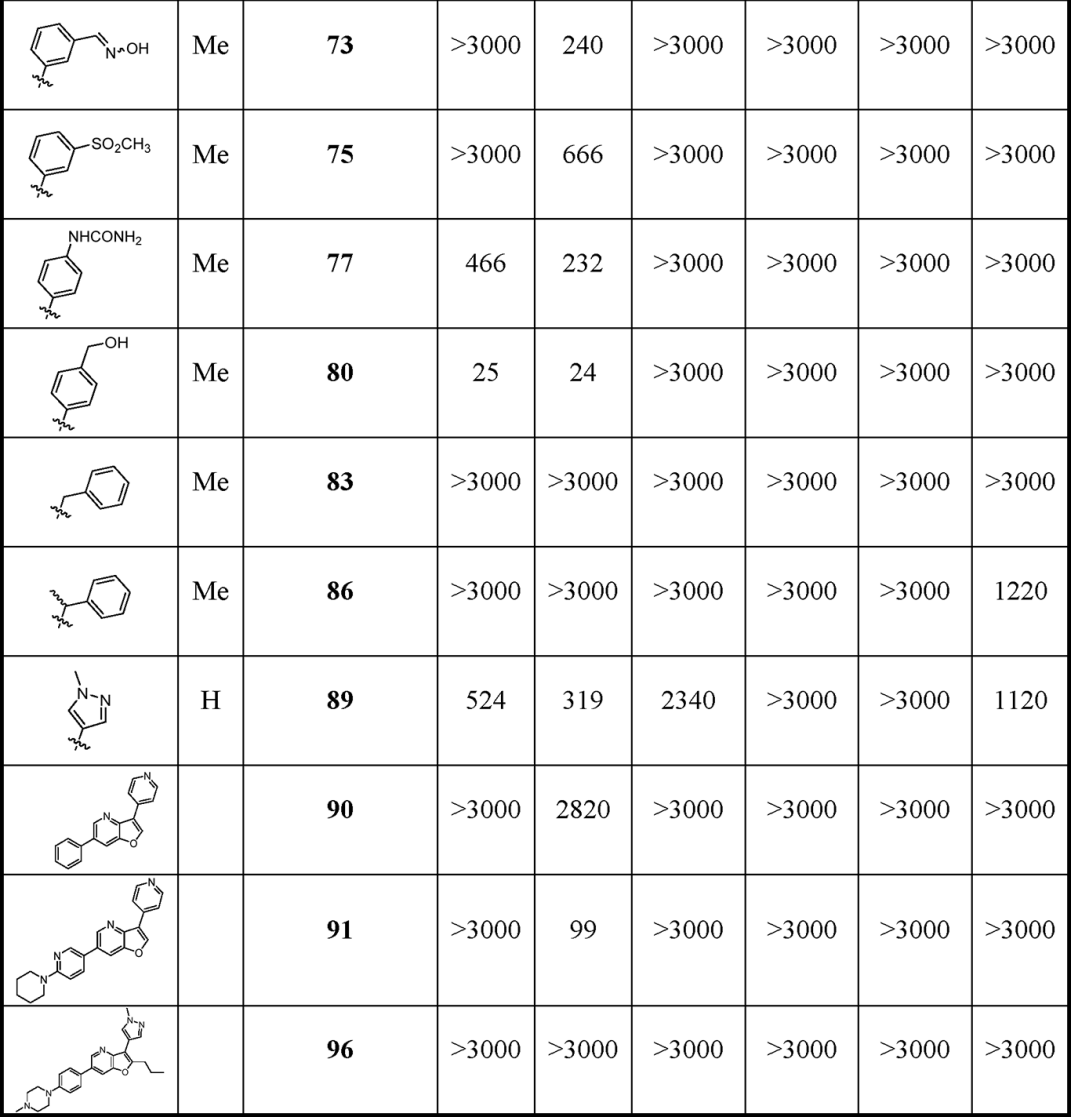
IC_50_ Values for ALK1-6 of **MU1700** and its Analogues^a^

a The IC_50_ values were determined by radiometric assays at an ATP concentration of 10 μM (Reaction Biology).

However, profiling of the compounds revealed several less obvious trends and rather unpredictable changes in the selectivity profile for individual ALK family members, perhaps best illustrated by the comparison of the compounds discussed below with the relatively inactive compound **60** possessing unsubstituted benzene ring at position 3 (Table 1). Fusion of an additional benzene ring led to the naphthyl analogue **21**, which showed good selectively and improved potency against ALK2. Interestingly, some isomers of **MU1700** with the (iso)quinoline nitrogen atom put at alternative positions (**MU1700NC**, **31** and **37**) were practically inactive. Installation of a methyl group onto the quinoline moiety of **MU1700** (**14**) with significantly improved potency for ALK4 and ALK5. This shift in potency may be attributed to the presence of a smaller serine gatekeeper residue in ALK4/5 (versus the threonine in ALK1/2/3/6),^49^ providing a larger cavity that can be occupied by the methyl substituent. Along this line, the direct trifluoromethylated analogue **18** was considerably less potent against ALK1 and ALK6 while keeping the potency against ALK4 (Table 1).

Bioisosteric replacement of the benzene moiety by the 4-pyridyl motif (**49**) significantly improved the activity against ALK1 and ALK2, which was further improved by dimethylation of the pyridine, exemplified by **46**. For this inhibitor, the proper positioning of the pyridine nitrogen atom was key as the 3-pyridyl isomer **52** was found to be inactive. Substitution of the benzene part of **60** with properly chosen substituents also afforded analogues with interesting selectivity. Specifically, fluorinated analogue **63**, oxime **73**, and methyl sulfone **75** were selective and had improved potency for ALK2; while the urea **77** and (especially) hydroxymethylated analogue **80** showed dual potency for ALK1 and ALK2. Installation of the methylene spacer at position 3 provided the inactive compound **83**. However, the analogue **86** harboring a methylated spacer showed moderately selectively and improved activity against ALK6 (Table 1). Finally, we realized that modification of other positions of the furopyridine scaffold can also lead to interesting preferential inhibition of certain ALK1–6 family members: the analogue of **49** with alternative polar R6, i.e. compound **91**, was selective for ALK2; and the installation of the propyl group at position 2 led to general loss of activity (cf. the compounds **89** and **96** in Table 1). Based on enzyme kinetic data, we selected **7** (**MU1700**) as the active chemical probe for ALK1/2/6 and **10** (**MU1700NC**) as the negative control for further biochemical and biological profiling.

### Characterization of **M4K2234**

**Figure 2:**
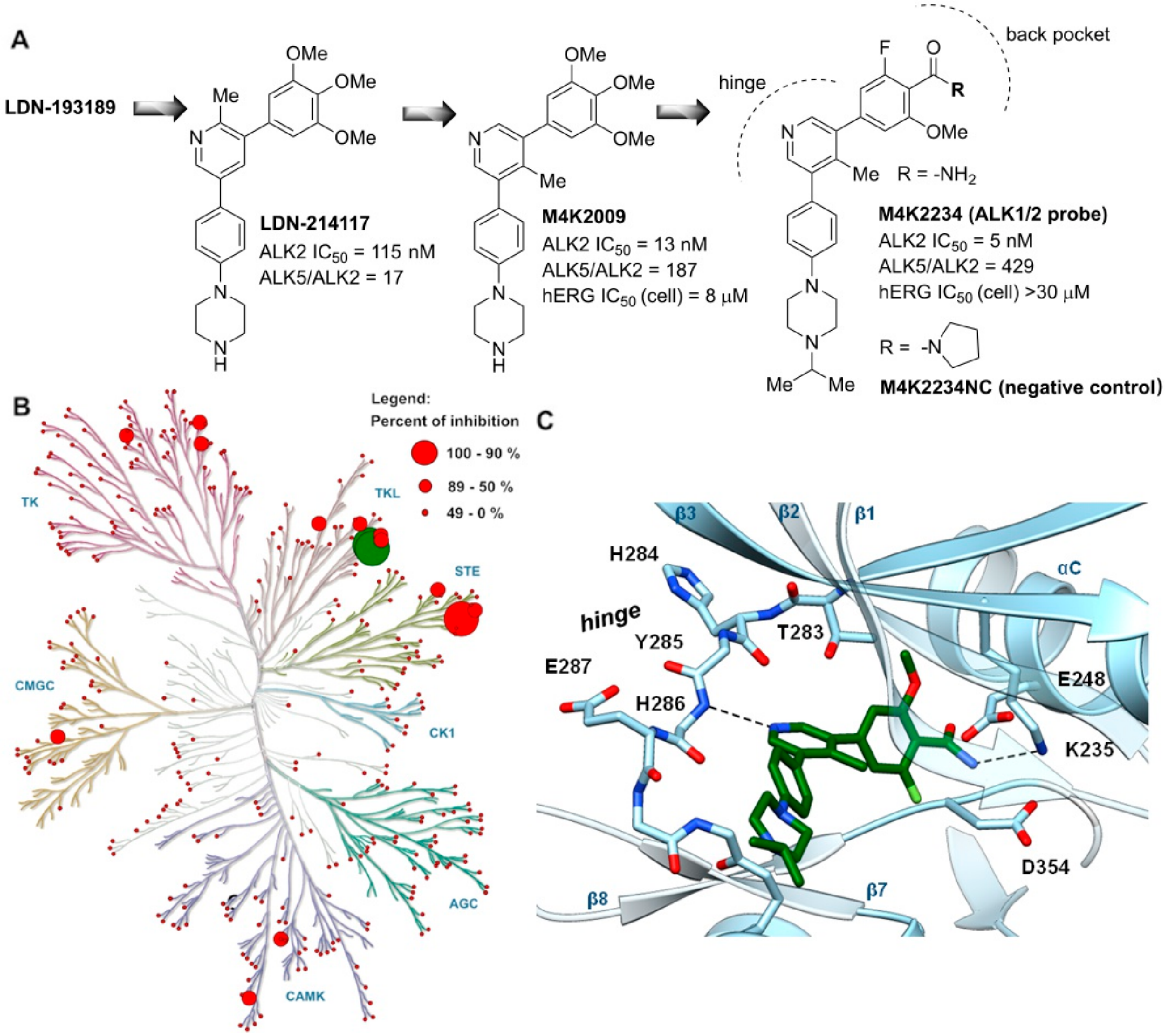
Structure and selectivity profile of **M4K2234**. (**A**) Development of **M4K2234** from previous generations of 3,5-diphenylpyridine-based inhibitors. (**B**) The compound **M4K2234** was screened in a panel of 375 protein kinases at 1 μM concentration (Reaction Biology). Red circles represent percentage of inhibition; ALK1 and ALK2 kinases are marked in green. (**C**) X-ray cocrystal structure of **M4K2234** (green, PDB code: 8R7G) bound to the active site of ALK2 (the amide nitrogen and carbonyl positions appeared interchangeable in the electron density allowing hydrogen bonding to ALK2 residues K235 and E248).

Employing an open science drug discovery model and using **LDN-214117** as a lead (see Figure 2A below), the previously reported work on the development of CNS-penetrant ALK2 inhibitors for the treatment of DMG produced a set of 3,5-diphenylpyridine inhibitors (typified by **M4K2009** in Figure 2A) with excellent ALK1/2 potency, selectivity, and/or blood–brain barrier (BBB) penetration profile.^46^ Based on their favorable in vivo pharmacokinetic (PK) properties, these inhibitors were selected as advanced preclinical compounds for further development and evaluation in orthotopic models of DMG;^32^ and the subsequent studies with 11C-labeled analogues suitable for PET neuroimaging demonstrated their potential to directly penetrate the pons region of the brain.^50^ Although these inhibitors had good structural and physicochemical properties, they posed the risk of eliciting torsades de pointes arrythmia in vivo because of their moderate affinity to the protein product encoded by the human ether-a-go-go related gene (hERG).^47^ Subsequent efforts to mitigate or eliminate this undesired off-target activity focused on the SAR around the original trimethoxyphenyl-substituted 3,5-diphenylpyridine inhibitor core, leading to the identification of benzamide analogues such as **M4K2234** with minimized hERG inhibition (Figure 2A).^47^

Of particular relevance to our probe development was the improved selectivity of **M4K2234** toward ALK5, as well as the overall selectivity profile across the kinome in comparison with **LDN-193189** (Figure 1B) which served as a literature reference compound. **M4K2234** was profiled in a comprehensive kinase selectivity panel, analogous to that used for characterizing **MU1700** previously (375 kinases, 1 μM, Reaction Biology). Pleasingly, the compound also exhibited excellent kinome-wide selectivity (Figure 2B and Table S3), with only one significant off target, the STE family member TNIK (TRAF2 and NCK Interacting Kinase). A crystal structure of the **M4K2234**-ALK2 complex (PDB-ID: 8R7G and Table S2) revealed a hydrogen bond interaction between the pyridine nitrogen and the kinase hinge residue His286 (Figure 2C). The pendant benzamide moiety was positioned to form further direct hydrogen bond interactions with K235 and E248 in contrast to the water-mediated interaction of **MU1700** (Figure 2C). We selected **M4K2234** for further biochemical and biological profiling to assess its potential as a quality chemical probe. Simultaneously, we focused on the development of a proper structurally similar negative control compound, exploiting the known binding mode of **M4K2234** and its analogues.^47^ Specifically, we modified the amide functionality interacting with the conserved lysine residue located in the ATP back pocket. We hypothesized that introduction of a sterically hindered tertiary amide would block this key back pocket interaction preventing the binding to the active site cavity. Following this strategy, we identified the negative control compound **M4K2234NC** (Figure 2A), which can be prepared from **M4K2234** in one synthetic step (see the SI).

### Comprehensive Profiling Identified **MU1700** and **M4K2234** as Chemical Probes for ALK1/2

**MU1700** and **M4K2234** were further profiled in biochemical and cell-based assays, along with their negative controls **MU1700NC** and **M4K2234NC**, in order to evaluate the kinome-wide selectivity of these compounds in more detail. We confirmed all significant off targets of this single concentration screen in dose response titrations. Within the ALK1–6 family, **M4K2234** showed potent inhibition for ALK1, ALK2 and ALK6 with IC50 values of 7, 14, and 88 nM, respectively. Activity for ALK4/5 was more than 100-fold weaker. In comparison, **MU1700** had a similar activity spectrum (Table 2). Outside the ALK1–6 family, only a few targets were detected in the kinome-wide screening and generally had IC50 values that were at least 85-fold weaker. The potency for the main **M4K2234** off-target TNIK was 41 nM and other weak potencies detected for other kinases were deemed not significant and were not evaluated using enzyme kinetic assays.

**Table 2:** (Top) Enzyme Kinetic IC_50_ Values for the ALK1-7 Sub-family and Selected Off Targets Detected in Kinome-Wide Profiling; The Selectivity Ratio Was Calculated as the Ratio of the IC_50_ Value for the Corresponding Kinase to the ALK2 IC_50_; (Bottom) ALK1-6 and Off-target EC_50_ Values of the Indicated Compounds Obtained in the Target Engagement NanoBRET Assay in Living Cells

Biochemical profiling				
	**MU1700**		**M4K2234**	
Kinase	IC_50_ (nM)	Selectivity	IC_50_ (nM)	Selectivity
ALK1/*ACVRL1*	13	2.2	7	0.5
ALK2/*ACVR1*	6	1.0	14	1.00
ALK3/*BMPR1A*	425	72.1	168	12
ALK4/*ACVR1B*	inactive	–	1 660	119
ALK5/*TGFBR1*	inactive	–	1 950	375
ALK6/*BMPR1B*	41	6.9	88	6.3
DDR1	501	85	ND	–
FLT3	751	127	ND	–
KHS/MAP4K5	539	91	ND	–
TNIK	ND	–	41	2.9

In cellulo target engagement – NanoBRET EC_50_ (nM)					
Kinase	**MU1700**	**MU1700NC**	**M4K2234**	**M4K2234NC**	**LDN-193189**
ALK1/*ACVRL1*	225	>10 000	83	>10 000	47
ALK2/*ACVR1*	27	3 601	13	5 686	4
ALK3/*BMPR1A*	497	>10 000	536	>10 000	48
ALK4/*ACVR1B*	>10 000	>10 000	8424	>10 000	1 309
ALK5/*TGFBR1*	>10 000	>10 000	7932	>10 000	810
ALK6/*BMPR1B*	997	>10 000	1628	>10 000	50
DDR1	>20 000	>20 000	–	–	–
FLT3	28 600	>20 000	–	–	–
KHS/MAP4K5	10 700 (lysed)	–	–	–	–

Next, we profiled **MU1700** and **M4K2234** in the NanoBRET target engagement assay in intact cells. As shown in Table 2 and Figure S1, both compounds efficiently penetrated the cell membrane and bound to the ALK1/2 ATP binding pocket with high potency in cellulo. ALK2 EC_50_ values of **M4K2234** and **MU1700** were comparable also to the frequently used ALK2 inhibitor **LDN-193189**, but their selectivity within the ALK1–7 subfamily was significantly narrower. **M4K2234** and **MU1700** were at low concentrations essentially ALK1/2 dual inhibitors in cells with no activity on ALK4/5.

Both probe compounds and their negative controls were tested for cytotoxicity in a cell viability assay using the U2OS cell line, with no cytotoxic effect evident over 24 h up to 2.5 μM concentration for **MU1700** and **MU1700NC** and up to 50 μM for M4K2234 and M4K2234NC (Figure S12). We hypothesize that the mild cytotoxic effect of **MU1700** and **MU1700NC** that we observed at 5 μM concentrations or higher, is due to the limited aqueous solubility of the compounds and precipitation in the cell culture medium. Therefore, considering their cellular potency against ALK1/2, we recommend using both chemical probes, **MU1700** and **M4K2234**, at concentrations up to 1 μM in cellular assays to study the biological roles and functions of ALK1 and ALK2 kinases.

**Figure 3:**
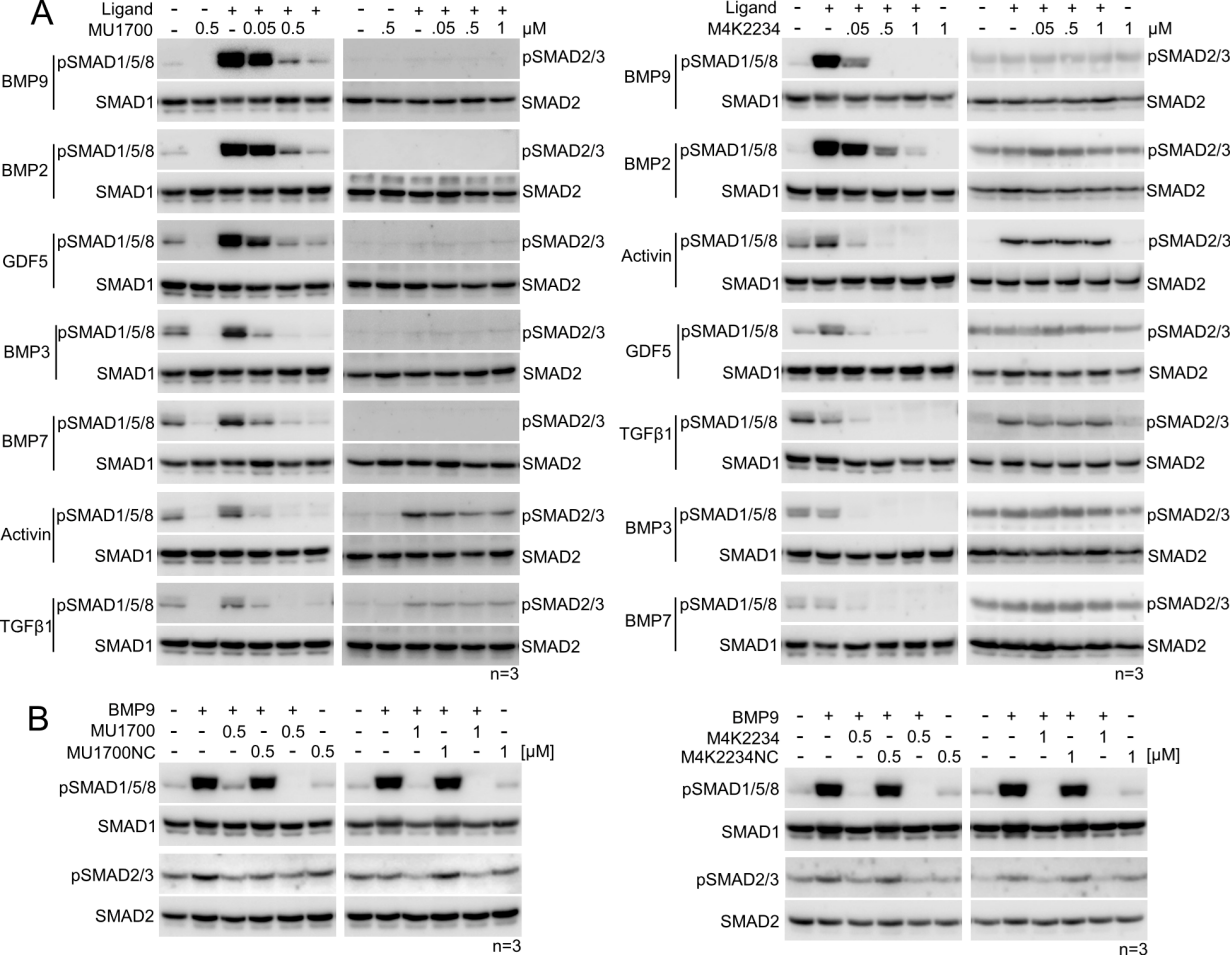
Western blot analysis of SMAD protein phosphorylation after receptor activation (**A**) **MU1700** and **M4K2234** specifically inhibited phosphorylation of SMAD1/5/8, but not SMAD2/3 in HEK293T cells in response to indicated ligands. The Western blot data are representative for three independent experiments (*n* = 3). (**B**) Side to side comparison of the inhibitory effect of the chemical probes **MU1700** and **M4K2234** (along with their negative control compounds **MU1700NC** and **M4K2234NC**) on BMP9-induced ALK2 phosphorylation of SMAD1/5/8 in HEK293T cells.

Activity of the developed chemical probes and their negative controls on endogenous ALK1–6 family members was first evaluated by monitoring the phosphorylation of SMAD proteins in HEK293T cells after receptor activation with appropriate ligands (Figure 3). Western blot analyses revealed that BMP and GDF family ligands induced phosphorylation of SMAD1/5/8 only, whereas activin A and TGF-β1 induced phosphorylation of both the SMAD1/5/8 and SMAD2/3 subfamilies. Interestingly, **MU1700** and **M4K2234** specifically inhibited phosphorylation of SMAD1/5/8, but not of SMAD2/3, consistent with reports of ALK5-mediated activation of ALK2 (Figure 3A). The weakest inhibition was observed using the BMP2 ligand which may be less biased toward ALK2 and more directed toward ALK3/6. No inhibition of SMAD phosphorylation was observed with the negative control compounds (Figure 3B).

**Figure 4:**
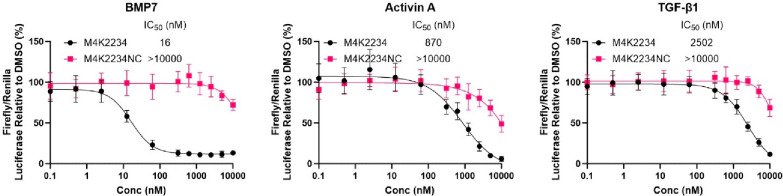
Inhibition of SMAD-dependent transcriptional reporters. BMP or CAGA-response element luciferase-based reporters in HEK293 cells were used to measure signaling induced by BMP7, activin A or TGF-β1, respectively. **M4K2234** exhibited low nanomolar inhibition of a BMP7 stimulated reporter, but only micromolar inhibition of an activin/TGF-β-responsive reporter. While the negative control compound **M4K2234NC** was largely inactive (IC50 values >10 μM) as expected, the orthogonal **MU1700** and **MU1700NC** exhibited assay interference preventing their use in this assay type. Data shown are from 3 independent experiments all performed in triplicate. Data plotted as mean ± standard deviation.

The cellular potency of the compounds was further tested by monitoring SMAD-dependent transcriptional activity using a dual luciferase-based reporter assay (Figure 4). Upon BMP7 stimulation, **M4K2234** potently inhibited a BMP-responsive reporter with an IC50 value of 16 nM (Figure 4), consistent with data from the NanoBRET target engagement assay. By comparison, a CAGA-response element reporter stimulated by activin A or TGF-β1 was inhibited 50 or 150-fold weaker, respectively (Figure 4). As expected, the negative control compound **M4K2234NC** was largely inactive against all ligands (IC50 > 10 μM). The orthogonal **MU1700** and **MU1700NC** negative control exhibited assay interference with apparent inhibition of the luciferase enzymes (data not shown). Further profiling of the dose responses of these compounds was therefore performed by Western blot detection of SMAD phosphorylation. SMAD1/5/8 phosphorylation induced by BMP7 was observed to decrease in the presence of 10 nM **MU1700** and was completely inhibited by 100 nM inhibitor (Figure S2). By contrast, inhibition of SMAD1/5/8 phosphorylation by the **MU1700NC** negative control was only observed at 10 μM concentration (Figure S2). Neither compound inhibited SMAD2 phosphorylation induced by activin A or TGF-β1 ligands (Figure S2). Overall, the cellular potency and selectivity of the probes against TGF-β superfamily ligands aligns well with the biochemical data suggesting their biased activity toward the ALK1/2 receptor kinases.

**Figure 5:**
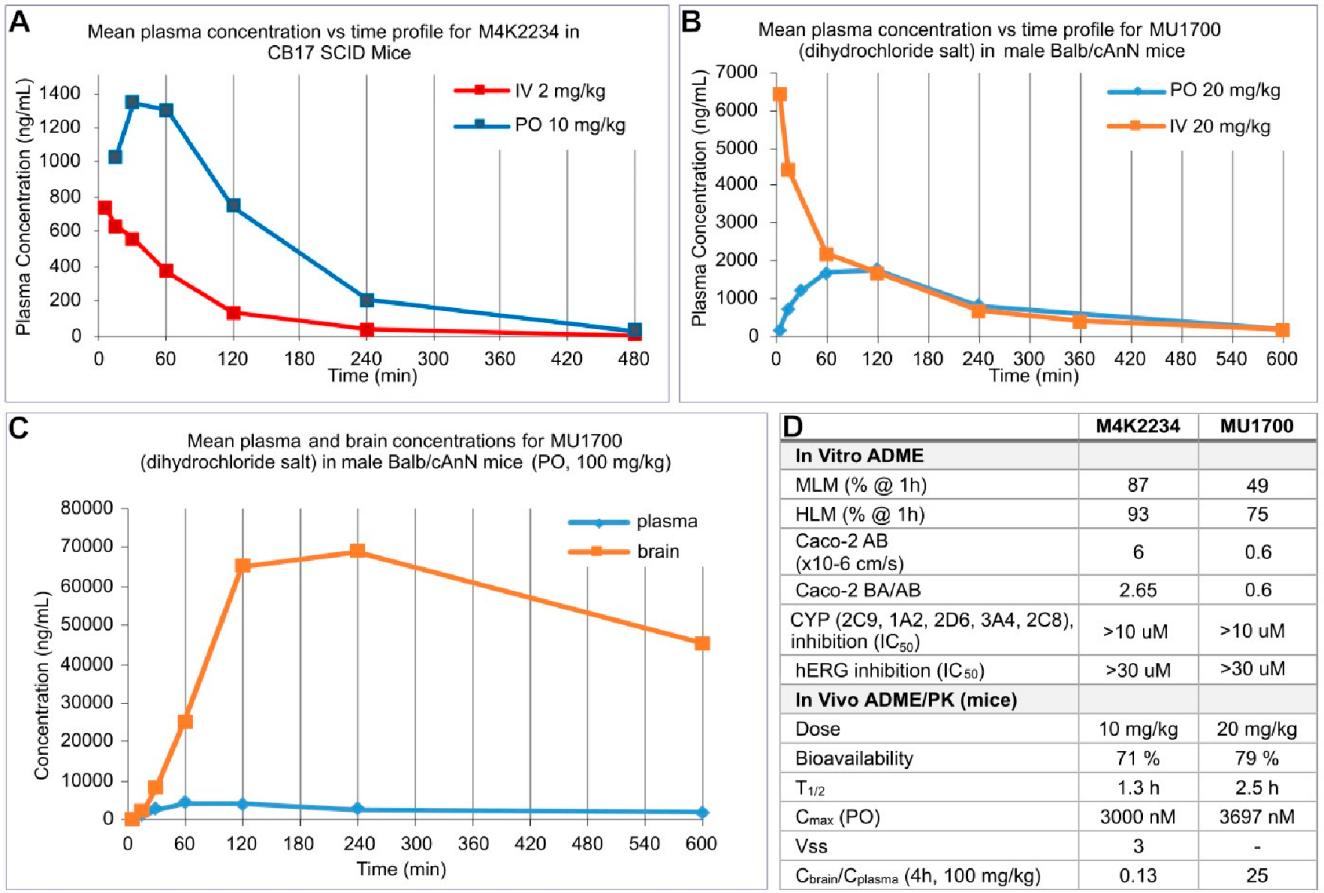
(**A**), (**B**) and (**C**) Pharmacokinetic profiles of **MU1700** (dosed as water-soluble dihydrochloride) and **M4K2234** in the mouse. The compounds were dosed as solutions in saline, the concentration values represent the averages obtained from samples from 4 animals, analyzed by LC/MS. Additional data have been compiled in SI. (**D**) ADME parameters of the compounds **M4K2234** and **MU1700**.

The stability of **M4K2234** and **MU1700** was profiled in vitro using human and mouse liver microsomes, revealing good and acceptable stability, respectively. Cell membrane penetration (Caco-2) was moderate for **M4K2234** and low for **MU1700**, with the BA/AB ratios 2.65 and 0.6, respectively (Figure 5D). In order to address the applicability of **MU1700** and **M4K2234** in vivo, we determined the pharmacokinetic (PK) profile of both compounds in mice. Gratifyingly, both chemical probes exhibited favorable PK profiles and very good bioavailability upon oral administration of 10 mg/kg (**M4K2234**) and 20 mg/kg (**MU1700**) (Figure 5A–C). Both compounds were well tolerated and for **MU1700** at the dose of 100 mg/kg in mice no signs of acute toxicity were observed.

Remarkably, **MU1700** exhibited excellent brain penetration, and its concentration in the brain was found to significantly exceed that in plasma (Figure 4). The brain:plasma ratio of **MU1700** was significantly higher than that observed for **LDN-193189**,^32^ which makes **MU1700** an especially attractive chemical probe for the investigation of CNS related ALK1/2 biology and pharmacology, such as validation of ALK2 as a therapeutic target for the treatment of DMG. Both compounds **M4K2234** and **MU1700** were found to be hERG-inactive, with IC_50_ values >30 μM (inhibiting hERG 32% and 8%, respectively, at 30 μM concentration).

## Conclusion

Chemical probes have become essential tools for studying the role of their protein targets in normal physiology as well as for their exploitation as drug targets and their role in disease pathogenesis. Because no small molecule, even after extensive profiling and optimization, can be expected to be absolutely selective for a single biological target even after extensive profiling and optimization, it is highly recommended that at least two orthogonal chemical probes be used, ideally along with their structurally related inactive negative controls.^51^ This is particularly important in the case of closely related target families such as ALK1–7 discussed in this study, as these targets may have overlapping unique functions in the complex signaling pathways that they modulate, and achieving selectivity for a single family member is challenging. The TGF-β superfamily induced signaling is highly context- and cell type-dependent, which represents an additional level of complexity of the signaling network.^52,53^

For example, some ligands of the TGF-β family can both inhibit and promote cell growth, maintain pluripotency or induce differentiation, and suppress or activate tumor cell growth, depending on the specific biological setting.^54^ Therefore, a key question in both basic biological research and the validation of an ALK family member as a drug target in a given disease is which members of the family are most relevant in regulating the diverse cellular signaling cascades that these receptors control.

In this study, we describe the identification and profiling of two chemically distinct highly selective inhibitors of kinases ALK1 and ALK2 - compounds **MU1700** and **M4K2234**, along with their structurally related negative controls. Compared to the heretofore used tool compound **LDN-193189**, both **MU1700** and **M4K2234** possess significantly improved kinome-wide selectivity, and within the ALK1–7 family they inhibit only ALK1 and ALK2 (and to a lesser extent ALK6).

Both compounds therefore fulfill community defined criteria for a quality chemical probe, efficiently inhibit the BMP pathway (which is mediated by SMAD1/5/8) via selective in cellulo inhibition of ALK1/2 kinases and exhibit favorable in vivo profiles in mice. Therefore, they represent highly selective chemical tools applicable in mechanistic studies of ALK1/ALK2-related signaling pathways that have been implicated in the development of many different diseases.^39^ M**U1700** and **M4K2234** represent a valuable addition to the small-molecule tool set of BMP modulators,^55^ and can be used for further pharmacological target validation of ALK1/2 for treatment of diseases such as DMG, FOP, MS or anemia of inflammation, along with other (pre)clinically profiled inhibitors.^56−62^ The probe **MU1700** is envisioned to be particularly suitable for neurological studies as it can pass through the blood-brain barrier and shows an exceptionally high accumulation in the brain. Based on its characteristics, the compounds **MU1700** and **M4K2234** have recently been reviewed and made available by the Structural Genomics Consortium as the state-of-the art chemical probes for ALK1/2 (https://www.thesgc.org/chemical-probes).^50,51,63,64^

The comparatively weaker interaction of the furo[3,2-*b*]pyridine core with the highly conserved kinase hinge region^48^ has been herein further experimentally supported by the crystal structure of **MU1700** in ALK2. We believe this is an important factor determining the high kinome-wide selectivity of **MU1700**, compared to the widely used inhibitor **LDN-193189**. Herein, we also provide data suggesting a wider application of the furo[3,2-*b*]pyridine scaffold for the development of selective inhibitors for the kinases ALK3–7.

## Experimental Section

### General Experimental Procedures

All reactions were performed in round-bottom flasks fitted with rubber septa. Reactions sensitive to air and/or moisture were performed under nitrogen or argon atmosphere. Air and moisture-sensitive liquids were transferred by syringe. Analytical thin-layer chromatography (TLC) was performed using aluminum plates precoated with silica gel (silica gel 60 F_254_, Merck). TLC plates were visualized by exposure to ultraviolet light. Flash-column chromatography was carried out on silica gel (60 Å, 20–45 μm, Fluorochem).

### Materials

All reagents were obtained from commercial suppliers and were used without further purification. Anhydrous solvents were used from commercial suppliers (Sigma-Aldrich, Acros Organics) and stored over 4 Å molecular sieves.

### Instrumentation

Nuclear magnetic resonance spectra were recorded using Bruker Avance 300 and 500 instrument at 30 °C. Data are represented the following way: chemical shift, multiplicity (s = singlet, d = doublet, t = triplet, q = quartet, sept = septet, m = multiplet and/or multiple resonances), coupling constant (*J*) in Hz. Proton chemical shifts are expressed in parts per million (ppm, δ scale) and are referenced to residual H in the NMR solvents (Chloroform-*d*, δ 7.26 ppm; DMSO-*d*_6_, δ 2.50 ppm; Methanol-*d*_4_, δ 3.31 ppm). Carbon chemical shifts are expressed in parts per million (ppm, δ scale) and are referenced to the carbon resonances of the NMR solvents (Chloroform-*d*, δ 77.2 ppm; DMSO-*d*_6_, δ 39.5 ppm; Methanol-*d*_4_, δ 49.0 ppm). Infrared (IR) spectra were obtained using an Alpha Bruker FT-IR Spectrometer (Platinum ATR). Data are represented the following way: frequency of absorption (cm^–1^). High-resolution mass spectra were obtained on Agilent 6224 Accurate-Mass TOF LC-MS with dual electrospray/chemical ionization mode or on MALDI-TOF Ultraflextreme (Bruker Daltonics) with positive ions detection. Melting points were determined with Stuart SMP40 automatic melting point apparatus. The purity of the synthesized target compounds was determined by HPLC analysis with UV detection (Ultimate 3000 LC analytical Systems–Thermo Scientific) and ^1^H NMR. All final compounds reported herein were >95% pure (unless stated otherwise).

### General Procedure A for Suzuki Cross-Coupling of 3-Bromo-6-chlorofuro[3,2-*b*]pyridine at Position 3

To a degassed solution of 3-bromo-6-chlorofuro[3,2-*b*]pyridine (0.43 mmol), K_3_PO_4_ (1.29 mmol), and boronic acid or ester (0.56 mmol) in a mixture of 1,4-dioxane/H_2_O (4:1; 1.25 mL per 0.1 mmol of 3-bromo-6-chlorofuro[3,2-*b*]pyridine) was added Pd(dppf)Cl_2_ (0.013 mmol), and the reaction mixture was stirred at 90 °C; the progress of the reaction was followed by TLC. After consumption of the starting material, the mixture was cooled to 25 °C, diluted with H_2_O (10 mL), and extracted with EtOAc (3 × 15 mL). The combined organic extracts were washed with brine (10 mL), dried over MgSO_4_ and filtered. The solvent was evaporated in vacuo and the residue was purified by flash chromatography.

### General Procedure B for Suzuki Cross-Coupling of 6-Chloro-3-Substituted Furo[3,2-*b*]pyridine at Position 6

To a degassed solution of 6-chloro-3-substituted furo[3,2-*b*]pyridine (0.229 mmol), K_3_PO_4_ (0.687 mmol), and boronic acid or ester (0.298 mmol) in a mixture of *n*-BuOH/H_2_O (4:1; 1.25 mL per 0.1 mmol of 6-chloro-3-substituted furo[3,2-*b*]pyridine) was added SPhos Pd G3 (0.007 mmol), and the reaction mixture was stirred at 110 °C (the progress of the reaction was followed by TLC). After consumption of the starting material, the mixture was cooled to 25 °C, diluted with H_2_O (10 mL), and extracted with EtOAc (3 × 15 mL). The combined organic extracts were washed with brine (10 mL), dried over MgSO_4_ and filtered. The solvent was evaporated in vacuo and the residue was purified by flash chromatography.

### General Procedure C for Deprotection of *N*-Boc-Protected Compounds

HCl (35% aq., 2 mL, 25.5 mmol) was added to a solution of the respective *N*-Boc-protected compound (0.186 mmol) in MeOH (2 mL) and the reaction mixture was stirred at 50 °C (the progress of the reaction was followed by TLC). After the time indicated for particular reaction, the mixture was cooled to 25 °C. The pH was adjusted to 8 with 2M NaOH (aq., 13 mL) and the resulting solution was extracted with EtOAc (3 × 30 mL). The combined organic extracts were washed with brine (10 mL), dried over MgSO_4_, and filtered. The solvent was evaporated in vacuo and the residue was purified by flash chromatography.

### 5-Chloro-2-iodopyridin-3-ol (**2**)

H_2_O (80 mL) was added to a mixture of 5-chloropyridin-3-ol (**1**, 5.12 g, 39.7 mmol), iodine (10.1 g, 39.7 mmol) and Na_2_CO_3_ (8.83 g, 83.3 mmol), and the resulting mixture was stirred under N_2_ at 25 °C for 3.5 h. The mixture was neutralized with 1M aqueous solution of HCl (120 mL) and extracted with EtOAc (120 + 70 + 70 mL). The combined organic extracts were washed with brine (80 mL), dried over MgSO_4_, filtered, and the solvent was evaporated. The product was obtained as a brown solid (10.1 g, 100% yield). ^1^H NMR (300 MHz, DMSO-*d*_6_) δ 11.38 (s, 1H), 7.95 (d, *J* = 2.3 Hz, 1H), 7.17 (d, *J* = 2.3 Hz, 1H). ^13^C NMR (126 MHz, DMSO) δ 154.56, 139.48, 130.81, 120.44, 108.53. FTIR (neat), cm^–1^: 2843, 2720, 2568, 1744, 1686, 1548, 1411, 1323, 1278, 1241, 1181, 1160, 1113, 1043, 865, 717, 590, 559, 537, 445. HRMS (APCI): calcd. for C_5_H_3_ClINO [M + H]^+^ = 255.9021, found [M + H]^+^ = 255.9020

### 6-Chloro-2-(trimethylsilyl)furo[3,2-*b*]pyridine (**3**)

To a degassed solution of 5-chloro-2-iodopyridin-3-ol (**2**, 5.60 g, 21.9 mmol) in dioxane (30 mL) and TEA (30 mL) were added ethynyltrimethylsilane (4.03 mL, 28.5 mmol), PdCl_2_(PPh_3_)_2_ (461 mg, 0.651 mmol) and CuI (250 mg, 1.31 mmol), and the resulting mixture was stirred at 45 °C for 105 min. The solvent was evaporated, and the residue was purified by column chromatography (*n*-hexane/EtOAc, gradient from 8% to 10% of EtOAc). The product was obtained as an orange solid (3.59 g, 73% yield). ^1^H NMR (500 MHz, Chloroform-*d*) δ 8.52 (dd, *J* = 1.9, 1.4 Hz, 1H), 7.81–7.76 (m, 1H), 7.16–7.12 (m, 1H), 0.40 (d, *J* = 1.0 Hz, 9H). ^13^C NMR (126 MHz, Chloroform-*d*) δ 170.30, 150.58, 146.96, 144.91, 127.25, 118.30, 117.11, −1.92. FTIR (neat), cm^–1^: 2958, 2918, 2851, 1453, 1381, 1250, 1043, 935, 839, 756, 635, 601. HRMS (APCI): calcd. for C_10_H_12_ClNOSi [M + H]^+^ = 226.0449, found [M + H]^+^ = 226.0458.

### 6-Chlorofuro[3,2-*b*]pyridine (**4**)

To a solution of 6-chloro-2-(trimethylsilyl)furo[3,2-*b*]pyridine (**3**, 3.59 g, 15.9 mmol) in methanol (25 mL) was added KF (2.77 g, 47.7 mmol) and the resulting mixture was stirred at 38 °C for 17 h. The solvent was evaporated in vacuo and the residue was purified by column chromatography (EtOAc/hexane, gradient from 1:10 to 3:10). The product was obtained as a white solid (2.18 g, 89% yield). ^1^H NMR (500 MHz, Chloroform-*d*) δ 8.58 (s, 1H), 7.85 (d, *J* = 2.3 Hz, 1H), 7.83–7.78 (m, 1H), 7.03–6.96 (m, 1H). ^13^C NMR (126 MHz, Chloroform-*d*) δ 149.87, 146.12, 145.39–145.20 (m), 118.66, 108.38. FTIR (neat), cm^–1^: 3342, 2973, 2926, 1379, 1269, 1087, 1045, 879, 736. HRMS (APCI): calcd. for C_7_H_4_ClNO [M + H]^+^**=** 154.0054, found = 154.0058.

### 3-Bromo-6-chlorofuro[3,2-*b*]pyridine (**5**)

Bromine (14.6 mL, 256 mmol) was added slowly at −20 °C to a stirred mixture of 6-chlorofuro[3,2-*b*]pyridine (**4**, 2.18 g, 14.2 mmol) and CCl_4_ (23 mL). The resulting mixture was stirred while allowed to warm to 25 °C over 75 min. Then, solution of Na_2_S_2_O_5_ (53 g) in water (100 mL) and ice (150 mL) were added and the resulting mixture was extracted with Et_2_O (2 × 150 mL). The combined organic extracts were dried over MgSO_4_, filtered, and the solvent was evaporated in vacuo (while keeping the temperature of bath at 30 °C). Then, toluene (25 mL) and DBU (6.36 mL, 42.6 mmol) were added to the residue and the resulting mixture was stirred at 80 °C for 45 min. The solvent was evaporated, and the residue was purified by column chromatography (EtOAc/hexane; from 1:8 to 1:5). The product was obtained as a white solid (2.46 g, 74% yield). ^1^H NMR (500 MHz, Chloroform-*d*) δ 8.61 (d, *J* = 2.0 Hz, 1H), 7.89 (s, 1H), 7.82 (d, *J* = 2.0 Hz, 1H). ^13^C NMR (126 MHz, Chloroform-*d*) δ 147.40, 147.27, 146.29, 143.52, 128.86, 119.34, 99.71. FTIR (neat), cm^–1^: 3094, 3041, 1536, 1457, 1379, 1285, 1074, 995, 910, 875, 772, 603, 496. HRMS (APCI): calcd. for C_7_H_3_BrClNO [M + H]^+^**=** 231.9159, found = 231.9162.

### 6-Chloro-3-(quinolin-4-yl)furo[3,2-*b*]pyridine (**6**)

To a degassed solution of 3-bromo-6-chlorofuro[3,2-*b*]pyridine (**5**, 347 mg, 1.49 mmol) in 1-butanol/H_2_O (5.0 + 1.0 mL) were added quinolin-4-ylboronic acid (310 mg, 1.79 mmol), K_3_PO_4_ (951 mg, 4.48 mmol) and Pd(PPh_3_)_4_ (85.0 mg, 73.5 μmol, 0.05 equiv), and the resulting mixture was stirred at 110 °C for 2 h. The solvent was evaporated in vacuo and the residue was purified by flash chromatography (Biotage; column: 25 g of 20–45 μm silica gel; eluent: cyclohexane/EtOAc, gradient from 30% to 60% of EtOAc). The product was obtained as a white solid (267 mg, 64% yield). ^1^H NMR (500 MHz, Methanol-*d*_4_) δ 8.95 (d, *J* = 4.5 Hz, 1H), 8.62–8.52 (m, 2H), 8.26 (d, *J* = 2.0 Hz, 1H), 8.17–8.06 (m, 2H), 7.87–7.79 (m, 2H), 7.66–7.61 (m, 1H). ^13^C NMR (126 MHz, Methanol-*d*_4_) δ 151.09, 150.86, 149.72, 149.27, 146.51, 145.66, 138.37, 131.29, 129.79, 129.59, 128.47, 128.23, 127.05, 123.77, 120.90, 119.85. FTIR (neat), cm^–1^: 3004, 1588, 1504, 1460, 1382, 1137, 1098, 907, 824, 800, 784, 753, 649, 601, 449, 423. HRMS (APCI): calcd. for C_16_H_9_ClN_2_O [M + H]^+^**=** 281.0476, found = 281.0475.

### 6-(4-(Piperazin-1-yl)phenyl)-3-(quinolin-4-yl)furo[3,2-*b*]pyridine (**7**, **MU1700**)

To a degassed solution of 6-chloro-3-(quinolin-4-yl)furo[3,2-*b*]pyridine (**6**, 112 mg, 0.399 mmol) in 1-butanol/H_2_O (5.0 mL + 1.0 mL) were added *tert*-butyl 4-(4-(4,4,5,5-tetramethyl-1,3,2-dioxaborolan-2-yl)phenyl)piperazine-1-carboxylate (201 mg, 0.518 mmol), K_3_PO_4_ (254 mg, 1.20 mmol), SPhos (8.19 mg, 0.0200 mmol) and SPhos Pd G3 (9.30 mg, 12.0 μmol, 0.03 equiv), and the resulting mixture was stirred for 35 min at 90 °C. Then, MeOH (2.0 mL) and 35% aqueous solution of HCl (0.822 mL, approximately 20 equiv) were added and the resulting mixture was stirred for 3.5 h at 25 °C. Then, additional MeOH (1.0 mL) and 35% aqueous solution of HCl (0.822 mL, approximately 20 equiv) were added and the resulting mixture was stirred for additional 2 h at 50 °C. Saturated aqueous solution of NaHCO_3_ (2.0 mL) was added, the solvent was evaporated in vacuo and the residue was purified by column chromatography (DCM/7 M solution of NH_3_ in MeOH, gradient from 5% to 15% of methanolic solution). The obtained material was triturated with EtOAc/MeOH (4 + 2 mL) and then with EtOAc (2 mL). The product was obtained as a white solid (97 mg, 60% yield). ^1^H NMR (500 MHz, DMSO-*d*_6_) δ 9.02 (d, *J* = 4.4 Hz, 1H), 8.88 (d, *J* = 1.8 Hz, 1H), 8.81 (s, 1H), 8.42 (d, *J* = 1.9 Hz, 1H), 8.20–8.09 (m, 2H), 7.88 (d, *J* = 4.4 Hz, 1H), 7.86–7.79 (m, 1H), 7.70 (d, *J* = 8.8 Hz, 2H), 7.67–7.59 (m, 1H), 7.06 (d, *J* = 8.9 Hz, 2H), 3.20–3.09 (m, 5H), 2.90–2.79 (m, 4H). ^13^C NMR (126 MHz, DMSO-*d*_6_) δ 151.42, 150.13, 149.00, 148.41, 148.17, 144.62, 143.63, 136.11, 132.95, 129.55, 129.48, 127.76, 126.86, 126.58, 126.09, 125.91, 122.28, 117.88, 115.80, 115.41, 48.90, 45.51. FTIR (neat), cm^–1^: 3286, 2818, 1596, 1522, 1479, 1450, 1372, 1237, 1203, 1144, 1096, 811, 769, 659, 544. HRMS (APCI): calcd. for C_26_H_22_N_4_O [M + H]^+^**=** 407.1866, found = 407.1869.

### 6-Chloro-3-(quinolin-8-yl)furo[3,2-*b*]pyridine (**8**)

The compound was prepared by the general procedure **A** using 224 mg (0.963 mmol) of 3-bromo-6-chlorofuro[3,2-*b*]pyridine (**5**) and 200 mg (1.156 mmol) of quinolin-8-ylboronic acid; the reaction mixture was stirred for 1 h; flash chromatography (Biotage; column: 10 g of 20–45 μm silica gel; eluent: cyclohexane/EtOAc, gradient from 0% to 20% of EtOAc) afforded the compound **8** as a pale yellow solid (192 mg, 71% yield). ^1^H NMR (500 MHz, Chloroform-*d*) δ 9.35 (s, 1H), 9.18 (dd, *J* = 7.3, 1.5 Hz, 1H), 8.99 (dd, *J* = 4.2, 1.8 Hz, 1H), 8.64 (d, *J* = 2.0 Hz, 1H), 8.22 (dd, *J* = 8.3, 1.8 Hz, 1H), 7.89–7.86 (m, 1H), 7.81 (dd, *J* = 8.1, 1.4 Hz, 1H), 7.71 (t, *J* = 7.7 Hz, 1H), 7.47 (dd, *J* = 8.2, 4.1 Hz, 1H). ^13^C NMR (126 MHz, Chloroform-*d*) δ 151.63, 149.83, 147.72, 145.96, 145.66, 144.77, 136.88, 130.25, 129.00, 128.88, 127.61, 127.25, 126.86, 121.20, 118.68, 117.31. FTIR (neat), cm^–1^: 3184, 3045, 1597, 1502, 1457, 1383, 1261, 1091, 1071, 938, 910, 865, 819, 791, 775, 642, 595, 435. HRMS (APCI): calcd. for C_16_H_9_ClN_2_O [M + H]^+^ = 281.0476; found 281.0473. MP: 150–151 °C

### *tert*-Butyl 4-(4-(3-(quinolin-8-yl)furo[3,2-*b*]pyridin-6-yl)phenyl)piperazine-1-carboxylate (**9**)

The compound was prepared by the general procedure **B** using 174 mg (0.620 mmol) of 6-chloro-3-(quinolin-8-yl)furo[3,2-*b*]pyridine (**8**) and 313 mg (0.806 mmol) of 4-(4-*tert*-butoxycarbonylpiperazinyl)phenylboronic acid pinacol ester; the reaction mixture was stirred for 2 h; flash chromatography (cyclohexane/EtOAc, gradient from 0% to 40% of EtOAc) afforded the compound **9** as a yellow foam (280 mg, 89% yield). ^1^H NMR (500 MHz, Chloroform-*d*) δ 9.34 (s, 1H), 9.28 (dd, *J* = 7.3, 1.5 Hz, 1H), 9.01 (dd, *J* = 4.1, 1.8 Hz, 1H), 8.91 (d, *J* = 1.9 Hz, 1H), 8.23 (dd, *J* = 8.2, 1.8 Hz, 1H), 7.98 (d, *J* = 2.0 Hz, 1H), 7.81 (dd, *J* = 8.2, 1.5 Hz, 1H), 7.74 (dd, *J* = 8.1, 7.2 Hz, 1H), 7.64–7.59 (m, 2H), 7.47 (dd, *J* = 8.2, 4.1 Hz, 1H), 7.08–7.03 (m, 2H), 3.70–3.60 (m, 4H), 3.23 (t, *J* = 5.2 Hz, 4H), 1.50 (s, 9H). ^13^C NMR (126 MHz, Chloroform-*d*) δ 154.91, 151.07, 151.00, 149.79, 148.59, 146.10, 145.66, 144.60, 136.88, 132.62, 130.27, 129.81, 129.67, 128.94, 128.35, 127.33, 126.97, 121.15, 117.32, 116.94, 116.03, 80.15, 49.17, 43.71, 28.60. FTIR (neat), cm^–1^: 2974, 1688, 1607, 1523, 1501, 1476, 1420, 1377, 1364, 1289, 1233, 1217, 1163, 1120, 1091, 1067, 1048, 938, 913, 819, 791, 779, 647, 537. HRMS (APCI): calcd. for C_31_H_30_N_4_O_3_ [M + H]^+^ = 507.2391; found [M + H]^+^ = 507.2394

### 6-(4-(Piperazin-1-yl)phenyl)-3-(quinolin-8-yl)furo[3,2-*b*]pyridine (**10**; **MU1700NC**)

The compound was prepared by the general procedure **C** using 140 mg (0.277 mmol) of *tert*-butyl 4-(4-(3-(quinolin-8-yl)furo[3,2-*b*]pyridin-6-yl)phenyl)piperazine-1-carboxylate (**9**); the reaction time was 2 h; flash chromatography (DCM/MeOH, gradient from 0% to 20% of MeOH) afforded the compound as a yellow solid (29 mg, 26% yield). ^1^H NMR (500 MHz, Chloroform-*d*) δ 9.34 (s, 1H), 9.29 (dd, *J* = 7.2, 1.5 Hz, 1H), 9.02 (dd, *J* = 4.1, 1.8 Hz, 1H), 8.92 (d, *J* = 1.9 Hz, 1H), 8.23 (dd, *J* = 8.2, 1.9 Hz, 1H), 7.99 (d, *J* = 1.9 Hz, 1H), 7.81 (dd, *J* = 8.2, 1.6 Hz, 1H), 7.74 (dd, *J* = 8.1, 7.3 Hz, 1H), 7.60 (d, *J* = 8.7 Hz, 2H), 7.47 (dd, *J* = 8.2, 4.2 Hz, 1H), 7.06 (d, *J* = 8.8 Hz, 2H), 3.29–3.20 (m, 4H), 3.10–3.05 (m, 4H). ^13^C NMR (126 MHz, Chloroform-*d*) δ 151.66, 150.94, 149.78, 148.61, 146.11, 145.54, 144.60, 136.87, 132.77, 130.26, 129.71, 129.19, 128.93, 128.26, 127.29, 126.98, 121.13, 117.30, 116.42, 115.96, 50.20, 46.29. FTIR (neat), cm^–1^: 3169, 3041, 2840, 1608, 1477, 1432, 1375, 1335, 1272, 1230, 1215, 1134, 1093, 819, 786, 777, 750, 643, 542. HRMS (APCI): calcd. for C_26_H_22_N_4_O [M + H]^+^ = 407.1866; found [M + H]^+^ = 407.1869. MP: 195–196

### 2-Methyl-4-(4,4,5,5-tetramethyl-1,3,2-dioxaborolan-2-yl)quinoline (**11**)

Pd(dppf)Cl_2_ (33 mg, 0.045 mmol, 0.02 equiv) was added to a degassed solution of 4-bromo-2-methylquinoline (500 mg, 2.27 mmol, 1.0 equiv), bis(pinacolato)diboron (866 mg, 3.41 mmol, 1.5 equiv) and KOAc (667 mg, 6.81 mmol, 3.0 equiv) in dry 1,4-dioxane (10 mL), and the resulting mixture was stirred at 90 °C for 1 h. (The progress of the reaction was followed by TLC). The mixture was cooled to 25 °C, diluted with H_2_O (20 mL), and extracted with EtOAc (3 × 20 mL). The combined organic extracts were washed with brine (10 mL), dried over MgSO_4_ and filtered. The solvent was evaporated in vacuo and the residue was purified by flash chromatography (cyclohexane/EtOAc, gradient from 30% to 100% of EtOAc). The product was obtained as a black solid (275 mg, 45% yield). ^1^H NMR (500 MHz, Chloroform-*d*) δ 8.58 (dd, *J* = 8.4, 1.4 Hz, 1H), 8.02 (dt, *J* = 8.3, 1.0 Hz, 1H), 7.75 (s, 1H), 7.66 (ddd, *J* = 8.4, 6.8, 1.5 Hz, 1H), 7.51 (ddd, *J* = 8.2, 6.8, 1.3 Hz, 1H), 2.74 (s, 3H), 1.44 (s, 12H). ^13^C NMR (126 MHz, Chloroform-*d*) δ 158.14, 147.85, 129.86, 129.38, 129.14, 129.12, 128.23, 126.03, 84.62, 25.28, 25.11. HRMS (APCI): calcd. for C_16_H_20_BNO_2_ [M + H]^+^ = 270.1663; found [M + H]^+^ = 270.1662.

### 6-Chloro-3-(2-methylquinolin-4-yl)furo[3,2-*b*]pyridine (**12**)

The compound was prepared by the general procedure **A** using 180 mg (0.774 mmol) of 3-bromo-6-chlorofuro[3,2-*b*]pyridine (**5**) and 250 mg (0.929 mmol) of 2-methyl-4-(4,4,5,5-tetramethyl-1,3,2-dioxaborolan-2-yl)quinoline (**11**); the reaction time was 1 h; flash chromatography (cyclohexane/EtOAc, gradient from 0% to 22% of EtOAc) afforded the compound **12** as a white solid (144 mg, 63% yield). ^1^H NMR (500 MHz, Chloroform-*d*) δ 8.60 (d, *J* = 2.1 Hz, 1H), 8.15 (s, 1H), 8.11 (dt, *J* = 8.3, 1.0 Hz, 1H), 7.98–7.91 (m, 2H), 7.72 (ddd, *J* = 8.4, 6.9, 1.4 Hz, 1H), 7.60 (s, 1H), 7.47 (ddd, *J* = 8.2, 6.8, 1.2 Hz, 1H), 2.81 (s, 3H). ^13^C NMR (126 MHz, Chloroform-*d*) δ 158.84, 148.73, 148.19, 148.14, 146.00, 144.69, 135.82, 129.75, 129.56, 128.39, 126.23, 125.15, 123.23, 119.47, 119.26, 25.56. FTIR (neat), cm-1:2978, 2931, 1372, 1331, 1288, 1202, 1172, 1122, 1091, 959, 847, 772, 741, 672, 577, 529. HRMS (APCI): calcd. for C_17_H_11_ClN_2_O [M + H]^+^ = 295.0633, found = 295.0635. MP: 128–129 °C

### *tert*-Butyl 4-(4-(3-(2-methylquinolin-4-yl)furo[3,2-*b*]pyridin-6-yl)phenyl)piperazine-1-carboxylate (**13**)

The compound was prepared by the general procedure **B** using 130 mg (0.441 mmol) of 6-chloro-3-(2-methylquinolin-4-yl)furo[3,2-*b*]pyridine (**12**) and 223 mg (0.573 mmol) of 4-(4-*tert*-butoxycarbonylpiperazinyl)phenylboronic acid pinacol ester; the reaction time was 1 h; flash chromatography (cyclohexane/EtOAc, gradient from 50% to 71% of EtOAc) afforded the compound **13** as an off-white solid (230 mg, 100% yield). ^1^H NMR (500 MHz, Chloroform-*d*) δ 8.85 (d, *J* = 1.9 Hz, 1H), 8.13 (s, 1H), 8.11 (d, *J* = 8.3 Hz, 1H), 8.04 (dd, *J* = 8.6, 1.4 Hz, 1H), 8.01 (d, *J* = 1.9 Hz, 1H), 7.71 (ddd, *J* = 8.4, 6.8, 1.4 Hz, 1H), 7.66 (s, 1H), 7.58 (d, *J* = 8.8 Hz, 2H), 7.47 (ddd, *J* = 8.2, 6.8, 1.3 Hz, 1H), 7.04 (d, *J* = 8.8 Hz, 2H), 3.64–3.58 (m, 4H), 3.23 (t, *J* = 5.2 Hz, 4H), 2.82 (s, 3H), 1.50 (s, 9H). ^13^C NMR (126 MHz, Chloroform-*d*) δ 158.81, 154.84, 151.21, 149.03, 148.68, 147.50, 145.67, 144.58, 136.61, 133.79, 129.63, 129.41, 129.14, 128.37, 126.10, 125.37, 125.32, 123.17, 119.31, 116.82, 116.35, 80.14, 48.99, 43.58, 28.57, 25.52. FTIR (neat), cm^–1^: 2974, 2818, 1699, 1606, 1523, 1473, 1458, 1421, 1383, 1366, 1340, 1290, 1269, 1228, 1179, 1159, 1128, 1092, 1047, 999, 914, 897, 866, 831, 812, 794, 780, 764, 631, 622, 537, 508. HRMS (APCI): calcd. for C_32_H_32_N_4_O_3_ [M + H]^+^ = 521.2547, found [M + H]^+^ = 521.2551. MP: 179–180 °C

### 3-(2-Methylquinolin-4-yl)-6-(4-(piperazin-1-yl)phenyl)furo[3,2-*b*]pyridine (**14**)

TFA (0.5 mL, 6.535 mmol) was added to a solution of *tert*-butyl 4-(4-(3-(2-methylquinolin-4-yl)furo[3,2-*b*]pyridin-6-yl)phenyl)piperazine-1-carboxylate (**13**; 100 mg, 0.192 mmol) in DCM (5 mL) and the reaction mixture was stirred at 23 °C for 2 h. All volatiles were evaporated in vacuo, the residue was dissolved in acetonitrile (5 mL), triethylamine (0.15 mL) was added, and the mixture was allowed to stir for 2 min. The product was collected by filtration as a white solid (79 mg, 98% yield). ^1^H NMR (500 MHz, Methanol-*d*_4_) δ 8.73 (d, *J* = 1.9 Hz, 1H), 8.44 (s, 1H), 8.22 (d, *J* = 1.9 Hz, 1H), 8.03 (ddd, *J* = 10.7, 8.3, 1.1 Hz, 2H), 7.76 (ddd, *J* = 8.4, 6.9, 1.4 Hz, 1H), 7.70 (s, 1H), 7.67–7.61 (m, 1H), 7.53 (ddd, *J* = 8.3, 6.9, 1.3 Hz, 1H), 7.16–7.07 (m, 2H), 3.39–3.34 (m, 4H), 3.23–3.18 (m, 4H), 2.78 (s, 3H). ^13^C NMR (126 MHz, Methanol-*d*_4_) δ 160.17, 152.37, 150.60, 150.16, 149.03, 145.80, 145.18, 138.80, 135.33, 131.14, 130.17, 129.20, 128.96, 127.50, 126.84, 126.59, 124.55, 119.64, 117.90, 48.93, 45.61, 24.69. FTIR (neat), cm^–1^: 2842, 1675, 1601, 1526, 1475, 1379, 1246, 1200, 1125, 1111, 920, 825, 800, 756, 720, 634, 533. HRMS (APCI): calcd. for C_27_H_24_N_4_O [M + H]^+^ = 421.2023; found [M + H]^+^ = 421.2026. MP: 175–176 °C

### 4-(4,4,5,5-Tetramethyl-1,3,2-dioxaborolan-2-yl)-2-(trifluoromethyl)quinoline (**15**)

Pd(dppf)Cl_2_ (40 mg, 0.054 mmol, 0.025 equiv) was added to a degassed solution of 4-chloro-2-(trifluoromethyl)quinoline (500 mg, 2.16 mmol, 1.0 equiv), bis(pinacolato)diboron (822 mg, 3.24 mmol, 1.5 equiv) and KOAc (635 mg, 6.48 mmol, 3.0 equiv) in dry 1,4-dioxane (10 mL), and the resulting mixture was stirred at 80 °C for 18 h. (The progress of the reaction was followed by TLC). The mixture was cooled to 25 °C, diluted with H_2_O (20 mL), and extracted with EtOAc (3 × 20 mL). The combined organic extracts were washed with brine (10 mL), dried over MgSO_4_ and filtered. The solvent was evaporated in vacuo and the residue was purified by flash chromatography (cyclohexane/EtOAc, gradient from 0% to 12% of EtOAc). The product was obtained as a white wax (390 mg, 56% yield). ^1^H NMR (500 MHz, Chloroform-*d*) δ 8.76–8.70 (m, 1H), 8.23 (d, *J* = 8.1 Hz, 1H), 8.18 (s, 1H), 7.80 (ddd, *J* = 8.4, 6.8, 1.4 Hz, 1H), 7.69 (ddd, *J* = 8.2, 6.9, 1.3 Hz, 1H), 1.45 (s, 12H). ^13^C NMR (126 MHz, Chloroform-*d*) δ 147.26 (q, *J*_*CF*_ = 34.3 Hz), 147.09, 132.08, 130.61, 130.40, 128.87, 128.52, 124.18 (q, *J*_*CF*_ = 2.2 Hz), 121.92 (q, *J*_*CF*_ = 275.4 Hz), 85.10, 25.11. FTIR (neat), cm^–1^: 2978, 2931, 1372, 1331, 1288, 1202, 1172, 1122, 1091, 959, 847, 772, 741, 672, 577, 529. HRMS (APCI): calcd. for C_16_H_17_BF_3_NO_2_ [M + H]^+^ = 324.1380; found [M + H]^+^ = 324.1382. MP: 77–78 °C

### 6-Chloro-3-(2-(trifluoromethyl)quinolin-4-yl)furo[3,2-*b*]pyridine (**16**)

The compound was prepared by the general procedure **A** using 170 mg (0.732 mmol) of 3-bromo-6-chlorofuro[3,2-*b*]pyridine (**5**) and 284 mg (0.879 mmol) of 4-(4,4,5,5-tetramethyl-1,3,2-dioxaborolan-2-yl)-2-(trifluoromethyl)quinoline (**15**); the reaction time was 1 h; flash chromatography (cyclohexane/EtOAc, gradient from 0% to 20% of EtOAc) afforded the compound **16** as a white solid (125 mg, 49% yield). ^1^H NMR (500 MHz, Chloroform-*d*) δ 8.62 (d, *J* = 2.0 Hz, 1H), 8.32 (dt, *J* = 8.5, 1.0 Hz, 1H), 8.22 (s, 1H), 8.09 (dd, *J* = 8.4, 1.3 Hz, 1H), 8.05 (s, 1H), 7.97 (d, *J* = 2.0 Hz, 1H), 7.87 (ddd, *J* = 8.5, 6.8, 1.4 Hz, 1H), 7.67 (ddd, *J* = 8.2, 6.8, 1.3 Hz, 1H). ^13^C NMR (126 MHz, Chloroform-*d*) δ 148.48, 148.23, 148.10, 148.06, 147.82, 146.34, 144.19, 138.42, 131.04, 129.12, 128.82, 127.53, 125.59, 122.80, 120.61, 119.47, 119.07, 118.06, 118.04, 45.79, 43.44, 8.65. ^19^F NMR (471 MHz, Chloroform-*d*) δ −67.47. FTIR (neat), cm^–1^: 3083, 2921, 1592, 1469, 1455, 1374, 1343, 1284, 1227, 1178, 1133, 1099, 1082, 957, 914, 885, 845, 811, 786, 777, 764, 748, 708, 669, 599, 474, 441. HRMS (APCI): calcd. for C_17_H_8_ClF_3_N_2_O [M + H]^+^ = 349.0350; found [M + H]^+^ = 349.0352. MP: 209–210 °C

### *tert*-Butyl 4-(4-(3-(2-(trifluoromethyl)quinolin-4-yl)furo[3,2-*b*]pyridin-6-yl)phenyl)piperazine-1-carboxylate (**17**)

The compound was prepared by the general procedure **B** using 125 mg (0.358 mmol) of 6-chloro-3-(2-(trifluoromethyl)quinolin-4-yl)furo[3,2-*b*]pyridine (**16**) and 181 mg (0.466 mmol) of 4-(4-*tert*-butoxycarbonylpiperazinyl)phenylboronic acid pinacol ester; the reaction mixture was stirred for 1 h; flash chromatography (cyclohexane/EtOAc, gradient from 0% to 20% of EtOAc) afforded the compound **17** as a white solid (136 mg, 66% yield). ^1^H NMR (500 MHz, Chloroform-*d*) δ 8.87 (d, *J* = 1.8 Hz, 1H), 8.32 (dt, *J* = 8.5, 1.0 Hz, 1H), 8.21 (s, 1H), 8.18 (dd, *J* = 8.6, 1.3 Hz, 1H), 8.11 (s, 1H), 8.05 (d, *J* = 1.9 Hz, 1H), 7.86 (ddd, *J* = 8.4, 6.8, 1.4 Hz, 1H), 7.68 (ddd, *J* = 8.3, 6.8, 1.3 Hz, 1H), 7.62–7.57 (m, 2H), 7.08–7.03 (m, 2H), 3.67–3.59 (m, 4H), 3.24 (t, *J* = 5.2 Hz, 4H), 1.50 (s, 9H). ^13^C NMR (126 MHz, Chloroform-*d*) δ 154.88, 151.33, 149.16, 148.11, 148.08, 147.83, 147.75, 146.01, 144.11, 139.24, 134.22, 130.96, 130.94, 128.97, 128.44, 127.72, 127.38, 125.87, 122.87, 120.68, 119.03, 118.02, 118.00, 116.94, 116.85, 116.53, 80.20, 49.00, 28.60. ^19^F NMR (471 MHz, Chloroform-*d*) δ −67.43. FTIR (neat), cm-1:2975, 2923, 2855, 2817, 1683, 1599, 1504, 1477, 1453, 1415, 1389, 1365, 1281, 1232, 1163, 1120, 1048, 1000, 919, 827, 811, 758, 730, 693, 531. HRMS (APCI): calcd. for C_32_H_29_F_3_N_4_O_3_ [M + H]^+^ = 575.2265; found [M + H]^+^ = 575.2267. MP: 164–165 °C.

### 6-(4-(Piperazin-1-yl)phenyl)-3-(2-(trifluoromethyl)quinolin-4-yl)furo[3,2-*b*]pyridine (**18**)

TFA (0.5 mL, 6.535 mmol) was added to a solution of *tert*-butyl 4-(4-(3-(2-(trifluoromethyl)quinolin-4-yl)furo[3,2-*b*]pyridin-6-yl)phenyl)piperazine-1-carboxylate (**17**; 100 mg, 0.174 mmol) in DCM (5 mL) and the reaction mixture was stirred at 23 °C for 2 h. All volatiles were evaporated in vacuo and the residue was dissolved in acetonitrile (5 mL), triethylamine (0.15 mL) was added, and the mixture was allowed to stir for 2 min. The product was collected by filtration as a yellow solid (26 mg, 31% yield). ^1^H NMR (500 MHz, Chloroform-*d*) δ 8.87 (d, *J* = 1.9 Hz, 1H), 8.32 (d, *J* = 8.4 Hz, 1H), 8.20 (s, 1H), 8.18 (dd, *J* = 8.5, 1.4 Hz, 1H), 8.11 (s, 1H), 8.04 (d, *J* = 1.9 Hz, 1H), 7.86 (ddd, *J* = 8.5, 6.8, 1.4 Hz, 1H), 7.68 (ddd, *J* = 8.3, 6.8, 1.3 Hz, 1H), 7.59 (d, *J* = 8.8 Hz, 2H), 7.06 (d, *J* = 8.8 Hz, 2H), 3.29–3.26 (m, 4H), 3.10 (dd, *J* = 6.2, 3.7 Hz, 4H). ^13^C NMR (126 MHz, Chloroform-*d*) δ 151.74, 149.18, 148.09, 147.97 (q, *J* = 34.7 Hz), 147.69, 146.01, 144.03, 139.27, 134.32, 130.95, 130.92, 128.96, 128.57, 128.37, 127.73, 125.88, 121.78 (q, *J* = 275.4 Hz), 119.03, 118.01, 118.00, 117.98, 116.47, 77.16, 49.73, 45.99. ^19^F NMR (471 MHz, Chloroform-*d*) δ −67.43. FTIR (neat), cm^–1^: 3042, 2846, 1675, 1604, 1524, 1478, 1375, 1348, 1253, 1181, 1135, 1101, 828, 766, 722, 531. HRMS (APCI): calcd. for C_27_H_21_F_3_N_4_O [M + H]^+^ = 475.1740; found [M + H]^+^ = 475.1743.

### 6-Chloro-3-(naphthalen-1-yl)furo[3,2-*b*]pyridine (**19**)

The compound was prepared by the general procedure **A** using 225 mg (0.969 mmol) of 3-bromo-6-chlorofuro[3,2-*b*]pyridine (**5**) and 200 mg (1.163 mmol) of naphthalen-1-ylboronic acid; the reaction time was 1 h; flash chromatography (cyclohexane/EtOAc, gradient from 0% to 20% of EtOAc) afforded the compound **19** as a white wax (167 mg, 62% yield).

^1^H NMR (500 MHz, Chloroform-*d*) δ 8.58 (d, *J* = 2.0 Hz, 1H), 8.06 (s, 1H), 7.96–7.92 (m, 3H), 7.91 (d, *J* = 2.0 Hz, 1H), 7.71 (dd, *J* = 7.0, 1.3 Hz, 1H), 7.58 (dd, *J* = 8.3, 7.0 Hz, 1H), 7.52 (ddd, *J* = 8.1, 6.7, 1.3 Hz, 1H), 7.47 (ddd, *J* = 8.3, 6.7, 1.4 Hz, 1H). ^13^C NMR (126 MHz, Chloroform-*d*) δ 148.00, 147.62, 145.63, 145.59, 134.16, 132.14, 129.09, 128.69, 128.36, 127.87, 126.87, 126.54, 126.22, 125.66, 125.63, 121.69, 118.99. FTIR (neat), cm^–1^: 3048, 1606, 1508, 1456, 1383, 1275, 1234, 1142, 1091, 1071, 939, 911, 878, 798, 773, 648, 599, 489. HRMS (APCI): calcd. for C_17_H_10_ClNO [M + H]^+^ = 280.0524; found [M + H]^+^ = 280.0526.

### *tert*-Butyl 4-(4-(3-(naphthalen-1-yl)furo[3,2-*b*]pyridin-6-yl)phenyl)piperazine-1-carboxylate (**20**)

The compound was prepared by the general procedure **B** using 140 mg (0.500 mmol) of 6-chloro-3-(naphthalen-1-yl)furo[3,2-*b*]pyridine (**19**) and 253 mg (0.650 mmol) of 4-(4-*tert*-butoxycarbonylpiperazinyl)phenylboronic acid pinacol ester; the reaction time was 1 h; flash chromatography (cyclohexane/EtOAc, gradient from 0% to 100% of EtOAc) afforded the compound **20** as a pale yellow solid (150 mg, 59% yield). ^1^H NMR (500 MHz, Chloroform-*d*) δ 8.82 (d, *J* = 1.9 Hz, 1H), 8.06 (s, 1H), 8.03 (dq, *J* = 8.3, 0.9 Hz, 1H), 8.00 (d, *J* = 1.9 Hz, 1H), 7.92 (dd, *J* = 8.3, 1.3 Hz, 2H), 7.76 (dd, *J* = 7.0, 1.2 Hz, 1H), 7.62–7.55 (m, 3H), 7.52 (ddd, *J* = 8.2, 6.8, 1.4 Hz, 1H), 7.47 (ddd, *J* = 8.2, 6.8, 1.4 Hz, 1H), 7.08–6.98 (m, 2H), 3.66–3.56 (m, 4H), 3.23 (t, *J* = 5.2 Hz, 4H), 1.50 (s, 9H). ^13^C NMR (126 MHz, Chloroform-*d*) δ 154.90, 151.14, 148.89, 146.96, 145.61, 145.43, 134.22, 133.32, 132.33, 129.60, 128.83, 128.63, 128.40, 128.33, 127.65, 126.44, 126.14, 125.93, 125.70, 121.62, 116.89, 116.25, 80.17, 49.11, 43.75, 28.60. FTIR (neat), cm^–1^: 2977, 2925, 1691, 1607, 1477, 1421, 1366, 1234, 1166, 1132, 800, 775. HRMS (APCI): calcd. for C_32_H_31_N_3_O_3_ [M + H]^+^ = 506.2438; found [M + H]^+^ = 506.2441. MP: 207–208 °C

### 3-(Naphthalen-1-yl)-6-(4-(piperazin-1-yl)phenyl)furo[3,2-*b*]pyridine (**21**)

The compound was prepared by the general procedure **C** using 140 mg (0.277 mmol) of *tert*-butyl 4-(4-(3-(naphthalen-1-yl)furo[3,2-*b*]pyridin-6-yl)phenyl)piperazine-1-carboxylate (**20**); the reaction time was 2 h; flash chromatography (DCM/MeOH, gradient from 0% to 20% of MeOH) afforded the compound as a white solid (60 mg, 53% yield). ^1^H NMR (500 MHz, DMSO-*d*_6_) δ 8.81 (d, *J* = 1.9 Hz, 1H), 8.60 (s, 1H), 8.37 (d, *J* = 1.9 Hz, 1H), 8.03 (dd, *J* = 8.3, 1.3 Hz, 2H), 7.97 (dd, *J* = 8.4, 1.1 Hz, 1H), 7.77 (dd, *J* = 7.1, 1.3 Hz, 1H), 7.74–7.67 (m, 2H), 7.64 (dd, *J* = 8.2, 7.0 Hz, 1H), 7.57 (ddd, *J* = 8.2, 6.8, 1.2 Hz, 1H), 7.50 (ddd, *J* = 8.2, 6.7, 1.4 Hz, 1H), 7.13–7.01 (m, 2H), 3.25–3.18 (m, 4H), 2.98–2.92 (m, 4H). ^13^C NMR (126 MHz, DMSO-*d*_6_) δ 150.97, 148.19, 147.87, 144.59, 144.29, 133.40, 132.47, 131.46, 128.33, 128.23, 128.15, 127.75, 127.46, 127.14, 126.27, 125.99, 125.76, 125.48, 120.29, 115.64, 115.60, 48.07, 44.83, 39.52. FTIR (neat), cm^–1^: 2823, 1605, 1521, 1477, 1450, 1376, 1335, 1238, 1216, 1134, 1096, 1060, 937, 915, 891, 829, 790, 781, 752, 661, 546, 522, 503, 428. HRMS (APCI): calcd. for C_27_H_23_N_3_O [M + H]^+^ = 406.1914; found [M + H]^+^ = 406.1918. MP: 209–210 °C

### 4,4,5,5-Tetramethyl-2-(3-methylnaphthalen-1-yl)-1,3,2-dioxaborolane (**22**)

29.3 mg (0.040 mmol) of bis(dibenzylideneacetone)palladium and 23.0 mg (0.040 mmol) of 2-dicyclohexylphosphino-2′,4′,6′-triisopropylbiphenyl were added to a degassed mixture of 353 mg (2.00 mmol) of 1-chloro-3-methylnaphthalene, 393 mg (4.00 mmol) of KOAc and 711 mg (2.80 mmol) of bis(pinacolato)diboron in 8.0 mL of 1,4-dioxane. The mixture was stirred for 17 h at 110 °C, cooled to ambient temperature, diluted with 20.0 mL EtOAc, filtered over Celite and evaporated in vacuo; flash chromatography (cyclohexane/EtOAc, gradient from 0% to 2% of EtOAc) afforded the compound as a white solid (250 mg, 47% yield). ^1^H NMR (500 MHz, Chloroform-*d*) δ 8.71 (d, *J* = 7.9 Hz, 1H), 7.92 (d, *J* = 1.9 Hz, 1H), 7.78–7.73 (m, 1H), 7.70 (br s, 1H), 7.49–7.39 (m, 2H), 2.51 (s, 3H), 1.43 (s, 12H). ^13^C NMR (126 MHz, Chloroform-*d*) δ 137.96, 135.36, 134.48, 133.74, 130.74, 128.33, 127.81, 125.66, 125.60, 83.85, 25.12, 21.56. FTIR (neat), cm^–1^: 2976, 1575, 1508, 1399, 1371, 1330, 1301, 1259, 1198, 1139, 1011, 964, 883, 847, 784, 752, 684, 628, 517. HRMS (APCI): calcd. for C_17_H_21_BO_2_ [M + H]^+^ = 269.1710; found [M + H]^+^ = 269.1703. MP: 97–98 °C.

### 6-Chloro-3-(3-methylnaphthalen-1-yl)furo[3,2-*b*]pyridine (**23**)

The compound was prepared by the general procedure **A** using 159 mg (0.685 mmol) of 3-bromo-6-chlorofuro[3,2-*b*]pyridine (**5**) and 239 mg (0.890 mmol) of 4,4,5,5-tetramethyl-2-(3-methylnaphthalen-1-yl)-1,3,2-dioxaborolane (**22**); the reaction time was 4 h; flash chromatography (cyclohexane/EtOAc, gradient from 0% to 2% of EtOAc) afforded the compound as a colorless oil (201 mg, quantitative yield). ^1^H NMR (500 MHz, Chloroform-*d*) δ 8.57 (d, J = 2.0 Hz, 1H), 8.04 (s, 1H), 7.91 (d, J = 2.0 Hz, 1H), 7.84 (dd, J = 15.8, 8.2 Hz, 1H), 7.70 (br s, 1H), 7.54 (d, J = 1.7 Hz, 1H), 7.47 (ddd, J = 8.2, 6.7, 1.2 Hz, 1H), 7.38 (ddd, J = 8.3, 6.8, 1.3 Hz, 1H), 2.57 (s, 3H). ^13^C NMR (126 MHz, Chloroform-*d*) δ 148.01, 147.65, 145.65, 145.61, 135.20, 134.48, 130.62, 130.51, 128.16, 128.03, 127.86, 126.65, 126.27, 125.66, 125.47, 121.73, 118.99, 21.76. FTIR (neat), cm^–1^: 2164, 1605, 1458, 1381, 1276, 1090, 916, 879, 813, 774, 748, 600, 528, 490. HRMS (APCI): calcd. for C_18_H_12_ClNO [M + H]^+^ = 294.0680; found [M + H]^+^ = 294.0683.

### *tert*-Butyl 4-(4-(3-(3-methylnaphthalen-1-yl)furo[3,2-*b*]pyridin-6-yl)phenyl)piperazine-1-carboxylate (**24**)

The compound was prepared by the general procedure **B** using 148 mg (0.504 mmol) of 6-chloro-3-(3-methylnaphthalen-1-yl)furo[3,2-*b*]pyridine (**23**) and 254 mg (0.655 mmol) of 4-(4-*tert*-butoxycarbonylpiperazinyl)phenylboronic acid pinacol ester; the reaction time was 5 h; flash chromatography (DCM/MeOH, gradient from 0% to 1% of MeOH) afforded the compound as a white solid (246 mg, 94% yield). ^1^H NMR (500 MHz, Chloroform-*d*) δ 8.82 (d, *J* = 1.8 Hz, 1H), 8.04 (s, 1H), 8.00 (d, *J* = 1.8 Hz, 1H), 7.95 (dd, *J* = 8.4, 1.1 Hz, 1H), 7.83 (d, *J* = 8.2 Hz, 1H), 7.70 (s, 1H), 7.61–7.57 (m, 3H), 7.47 (ddd, *J* = 8.1, 6.8, 1.2 Hz, 1H), 7.39 (ddd, *J* = 8.3, 6.8, 1.4 Hz, 1H), 7.07 (d, *J* = 8.3 Hz, 2H), 3.63 (t, *J* = 5.2 Hz, 4H), 3.24 (t, *J* = 5.2 Hz, 4H), 2.58 (s, 3H), 1.50 (s, 9H). ^13^C NMR (126 MHz, Chloroform-*d*) δ 154.87, 148.86, 147.02, 145.62, 145.34, 135.22, 134.49, 133.23, 130.66, 130.56, 128.65, 128.42, 127.96, 127.90, 127.34, 126.35, 126.18, 125.69, 125.55, 121.57, 117.00, 116.29, 80.19, 49.23, 43,47, 28.60, 21.81. FTIR (neat), cm^–1^: 2164, 2109, 1981, 1696, 1607, 1524, 1477, 1423, 1366, 1235, 1164, 1001, 914, 816, 535. HRMS (APCI): calcd. for C_33_H_33_N_3_O_3_ [M + H]^+^ = 520.2595; found [M + H]^+^ = 520.2599.

### 3-(3-Methylnaphthalen-1-yl)-6-(4-(piperazin-1-yl)phenyl)furo[3,2-*b*]pyridine (**25**)

2.0 mL HCl 36% was added to a solution of 170 mg (0.327 mmol) *tert*-butyl 4-(4-(3-(6-methylnaphthalen-1-yl)furo[3,2-*b*]pyridin-6-yl)phenyl)piperazine-1-carboxylate (**24**) in 2.0 mL MeOH. The mixture was stirred at 50 °C for 2 h and evaporated in vacuo. The residue was dissolved in 15.0 mL DCM/MeOH (9:1), 1.0 g Na_2_CO_3_ was added and the resulting suspension was stirred for 1 h, filtered through a paper filter, and evaporated in vacuo. Flash chromatography (DCM/MeOH, gradient from 0% to 6% of MeOH) afforded the compound as off-white solid (124 mg, 90%. yield). ^1^H NMR (500 MHz, Chloroform-*d*) δ 8.83 (d, *J* = 1.9 Hz, 1H), 8.03 (s, 1H), 8.00 (d, *J* = 1.9 Hz, 1H), 7.96 (dd, *J* = 8.5, 1.1 Hz, 1H), 7.83 (dd, *J* = 8.1, 1.3 Hz, 1H), 7.70 (s, 1H), 7.61–7.56 (m, 3H), 7.47 (ddd, *J* = 8.2, 6.7, 1.2 Hz, 1H), 7.39 (ddd, *J* = 8.2, 6.8, 1.3 Hz, 1H), 7.08–7.02 (m, 2H), 3.33–3.22 (m, 4H), 3.10 (t, *J* = 5.0 Hz, 4H), 2.68 (br s, 1H), 2.58 (s, 3H). ^13^C NMR (126 MHz, Chloroform-*d*) δ 151.47, 148.87, 146.91, 145.59, 145.42, 135.22, 134.49, 133.34, 130.67, 130.55, 129.28, 128.34, 127.94, 127.87, 127.43, 126.18, 125.72, 125.54, 121.59, 116.54, 116.18, 49.74, 45.91, 21.81. FTIR (neat), cm^–1^: 2165, 1609, 1528, 1481, 1384, 1249, 1217, 1143, 1088, 1015, 914, 814, 747, 694, 610, 526, 471. HRMS (APCI): calcd. for C_28_H_25_N_3_O [M + H]^+^ = 420.2070; found [M + H]^+^ = 420.2073.

### 6-Chloro-3-(isoquinolin-4-yl)furo[3,2-*b*]pyridine (**26**)

The compound was prepared by the general procedure **A** using 76 mg (0.327 mmol) of 3-bromo-6-chlorofuro[3,2-*b*]pyridine (**5**) and 100 mg (0.392 mmol) of 4-(4,4,5,5-tetramethyl-1,3,2-dioxaborolan-2-yl)isoquinoline; the reaction time was 2 h; flash chromatography (cyclohexane/EtOAc, gradient from 0% to 35% of EtOAc) afforded the compound **26** as a white wax (66 mg, 72% yield). ^1^H NMR (500 MHz, Chloroform-*d*) δ 9.33 (s, 1H), 8.71 (s, 1H), 8.58 (d, *J* = 2.0 Hz, 1H), 8.10 (s, 1H), 8.07 (dd, *J* = 7.5, 1.6 Hz, 1H), 7.94 (d, *J* = 2.0 Hz, 1H), 7.91 (d, *J* = 8.3 Hz, 1H), 7.70 (ddd, *J* = 8.3, 6.8, 1.6 Hz, 1H), 7.67 (ddd, *J* = 8.1, 6.8, 1.4 Hz, 1H). ^13^C NMR (126 MHz, Chloroform-*d*) δ 153.45, 148.20, 147.80, 145.86, 145.23, 143.99, 134.72, 130.99, 128.76, 128.25, 127.75, 125.00, 121.06, 119.24, 118.99. FTIR (neat), cm^–1^: 3065, 3030, 1714, 1605, 1567, 1499, 1459, 1399, 1386, 1357, 1282, 1248, 1225, 1113, 1078, 940, 912, 885, 857, 832, 803, 782, 774, 747, 654, 634, 620, 600, 565, 539, 526, 493, 465, 446, 416. MP: 190–191 °C

HRMS (APCI): calcd. for C_16_H_9_ClN_2_O [M + H]^+^ = 281.0476; found [M + H]^+^ = 281.0478.

### *tert*-Butyl 4-(4-(3-(isoquinolin-4-yl)furo[3,2-*b*]pyridin-6-yl)phenyl)piperazine-1-carboxylate (**27**)

The compound was prepared by the general procedure **B** using 61 mg (0.217 mmol) of 6-chloro-3-(isoquinolin-4-yl)furo[3,2-*b*]pyridine (**26**) and 110 mg (0.283 mmol) of 4-(4-*tert*-butoxycarbonylpiperazinyl)phenylboronic acid pinacol ester; the reaction time was 2 h; flash chromatography (cyclohexane/EtOAc, gradient from 0% to 100% of EtOAc) afforded the compound **27** as a yellow oil (92 mg, 84% yield). ^1^H NMR (500 MHz, Chloroform-*d*) δ 9.32 (d, *J* = 0.9 Hz, 1H), 8.82 (d, *J* = 1.9 Hz, 1H), 8.74 (s, 1H), 8.09 (s, 1H), 8.08–8.05 (m, 1H), 8.03–7.99 (m, 2H), 7.71 (ddd, *J* = 8.4, 6.8, 1.5 Hz, 1H), 7.66 (ddd, *J* = 8.1, 6.8, 1.2 Hz, 1H), 7.62–7.55 (m, 2H), 7.11–6.99 (m, 2H), 3.65–3.58 (m, 4H), 3.23 (t, *J* = 5.2 Hz, 4H), 1.50 (s, 9H). ^13^C NMR (126 MHz, Chloroform-*d*) δ 154.87, 153.16, 151.19, 149.06, 147.06, 145.58, 145.18, 143.90, 134.88, 133.65, 130.87, 129.33, 128.79, 128.40, 128.15, 127.63, 125.27, 121.78, 118.86, 116.85, 116.40, 80.16, 49.04, 43.51, 28.59, 25.01. FTIR (neat), cm^–1^: 2975, 1738, 1692, 1608, 1524, 1478, 1422, 1367, 1235, 1165, 944, 826, 779, 753, 733. HRMS (APCI): calcd. for C_31_H_30_N_4_O_3_ [M + H]^+^ = 507.2391; found [M + H]^+^ = 507.2389

### 3-(Isoquinolin-4-yl)-6-(4-(piperazin-1-yl)phenyl)furo[3,2-*b*]pyridine (**28**)

The compound was prepared by the general procedure **C** using 84 mg (0.166 mmol) of *tert*- butyl 4-(4-(3-(isoquinolin-4-yl)furo[3,2-*b*]pyridin-6-yl)phenyl)piperazine-1-carboxylate (**27**); the reaction time was 2 h; flash chromatography (DCM/MeOH, gradient from 0% to 20% of MeOH) afforded the compound as a yellow solid (27 mg, 40% yield). ^1^H NMR (500 MHz, Methanol-*d*_4_) δ 9.34 (s, 1H), 8.75 (d, *J* = 1.9 Hz, 1H), 8.68 (s, 1H), 8.45 (s, 1H), 8.30 (d, *J* = 1.8 Hz, 1H), 8.27–8.21 (m, 1H), 8.07–7.98 (m, 1H), 7.80 (dddd, *J* = 20.4, 8.1, 6.9, 1.3 Hz, 2H), 7.75–7.68 (m, 2H), 7.27–7.15 (m, 2H), 3.52 (dd, *J* = 6.6, 3.9 Hz, 4H), 3.44–3.38 (m, 4H). ^13^C NMR (126 MHz, Methanol-*d*_4_) δ 153.77, 151.57, 150.65, 149.96, 145.99, 145.74, 143.74, 136.32, 135.12, 132.72, 131.18, 130.11, 129.50, 129.39, 129.25, 126.05, 123.58, 118.80, 118.32, 118.11, 47.48, 44.80. FTIR (neat), cm^–1^: 2933, 2719, 2498, 1605, 1524, 1477, 1456, 1399, 1377, 1250, 1141, 1119, 1109, 946, 914, 906, 887, 820, 791, 775, 759, 621, 613, 548, 526, 510. HRMS (APCI): calcd. for C_26_H_22_N_4_O [M + H]^+^ = 407.1866; found [M + H]^+^ = 407.1867. MP: 229–230 °C

### 6-Chloro-3-(isoquinolin-5-yl)furo[3,2-*b*]pyridine (**29**)

The compound was prepared by the general procedure **A** using 103 mg (0.444 mmol) of 3-bromo-6-chlorofuro[3,2-*b*]pyridine (**5**) and 100 mg (0.578 mmol) of isoquinolin-5-ylboronic acid; the reaction time was 3 h; flash chromatography (cyclohexane/EtOAc, gradient from 0% to 100% of EtOAc) afforded the compound as a white solid (28 mg, 23% yield). ^1^H NMR (500 MHz, Chloroform-*d*) δ 9.34 (s, 1H), 8.59 (d, *J* = 2.1 Hz, 1H), 8.53 (d, *J* = 6.0 Hz, 1H), 8.10 (s, 1H), 8.06 (dd, *J* = 8.2, 1.1 Hz, 1H), 7.98 (dd, *J* = 7.1, 1.2 Hz, 1H), 7.94 (d, *J* = 2.0 Hz, 1H), 7.77 (d, *J* = 6.0 Hz, 1H), 7.73 (dd, *J* = 8.3, 7.1 Hz, 1H). ^13^C NMR (126 MHz, Chloroform-*d*) δ 153.18, 148.14, 147.67, 145.87, 145.03, 143.77, 134.72, 132.42, 129.29, 128.59, 128.28, 127.21, 126.24, 120.36, 119.25, 118.45. FTIR (neat), cm^–1^: 3072, 3026, 2922, 2852, 1726, 1619, 1592, 1460, 1387, 1282, 1263, 1096, 1073, 1060, 1039, 911, 879, 834, 816, 808, 798, 776, 757, 713, 664, 599, 490, 470, 413. HRMS (APCI): calcd. for C_16_H_9_ClN_2_O [M + H]^+^ = 281.0476; found [M + H]^+^ = 281.0475. MP: 239–240 °C.

### *tert*-Butyl 4-(4-(3-(isoquinolin-5-yl)furo[3,2-*b*]pyridin-6-yl)phenyl)piperazine-1-carboxylate (**30**)

The compound was prepared by the general procedure **B** using 50 mg (0.178 mmol) of 6-chloro-3-(isoquinolin-5-yl)furo[3,2-*b*]pyridine (**29**) and 90 mg (0.232 mmol) of 4-(4-*tert*-butoxycarbonylpiperazinyl)phenylboronic acid pinacol ester; the reaction time was 2 h; flash chromatography (cyclohexane/EtOAc, gradient from 0% to 72% of EtOAc) afforded the compound **30** as a white solid (59 mg, 65% yield). ^1^H NMR (500 MHz, Chloroform-*d*) δ 9.31 (d, *J* = 1.1 Hz, 1H), 8.82 (d, *J* = 1.9 Hz, 1H), 8.52 (d, *J* = 6.0 Hz, 1H), 8.06 (s, 1H), 8.02 (td, *J* = 7.3, 1.2 Hz, 2H), 7.99 (d, *J* = 1.9 Hz, 1H), 7.84 (dt, *J* = 6.0, 1.0 Hz, 1H), 7.71 (dd, *J* = 8.2, 7.2 Hz, 1H), 7.60–7.53 (m, 2H), 7.06–6.99 (m, 2H), 3.68–3.56 (m, 4H), 3.22 (dd, *J* = 6.2, 4.1 Hz, 4H), 1.49 (s, 9H). ^13^C NMR (126 MHz, Chloroform-*d*) δ 154.79, 153.05, 151.13, 148.93, 146.88, 145.49, 144.89, 143.65, 134.74, 133.59, 132.20, 129.25, 129.18, 128.31, 128.17, 127.13, 126.93, 120.19, 118.57, 116.77, 116.29, 80.08, 48.95, 43.67, 28.53. FTIR (neat), cm^–1^: 2975, 2931, 2824, 2215, 1684, 1604, 1522, 1461, 1412, 1379, 1365, 1334, 1283, 1253, 1231, 1158, 1133, 1101, 1061, 1039, 994, 904, 886, 838, 823, 814, 799, 776, 763, 742, 724, 671, 541, 476. HRMS (APCI): calcd. for C_31_H_30_N_4_O_3_ [M + H]^+^ = 507.2391; found [M + H]^+^ = 507.2393. MP: 175–176 °C

### 3-(Isoquinolin-5-yl)-6-(4-(piperazin-1-yl)phenyl)furo[3,2-*b*]pyridine (**31**)

The compound was prepared by the general procedure **C** using 50 mg (0.099 mmol) of *tert*-butyl 4-(4-(3-(isoquinolin-5-yl)furo[3,2-*b*]pyridin-6-yl)phenyl)piperazine-1-carboxylate (**30**). The reaction time was 2 h; flash chromatography (DCM/MeOH, gradient from 0% to 20% of MeOH). The product was obtained as pale yellow wax (18 mg, 45% yield). ^1^H NMR (500 MHz, DMSO-*d*_6_) δ 9.42 (d, *J* = 0.9 Hz, 1H), 8.85 (d, *J* = 1.9 Hz, 1H), 8.69 (s, 1H), 8.51 (d, *J* = 6.0 Hz, 1H), 8.39 (d, *J* = 1.9 Hz, 1H), 8.24 (dt, *J* = 8.3, 1.2 Hz, 1H), 8.12 (dd, *J* = 7.2, 1.2 Hz, 1H), 7.91–7.85 (m, 1H), 7.84 (dd, *J* = 8.2, 7.1 Hz, 1H), 7.72–7.68 (m, 2H), 7.08–7.04 (m, 2H), 3.20–3.12 (m, 4H), 2.91–2.84 (m, 4H). ^13^C NMR (126 MHz, DMSO-*d*_6_) δ 152.74, 151.31, 148.32, 148.16, 144.40, 144.05, 143.31, 133.67, 132.71, 132.13, 128.57, 127.93, 127.72, 127.11, 126.75, 126.58, 118.88, 118.43, 115.68, 115.44, 48.76, 45.38. FTIR (neat), cm^–1^: 1653, 1605, 1525, 1378, 1099, 818, 805, 607, 545, 502, 474. HRMS (APCI): calcd. for C_26_H_22_N_4_O [M + H]^+^ = 407.1866; found [M + H]^+^ = 407.1869. MP: 213-214 °C

### 6-Chloro-3-(quinolin-5-yl)furo[3,2-*b*]pyridine (**32**)

The compound was prepared by the general procedure **A** using 224 mg (0.963 mmol) of 3-bromo-6-chlorofuro[3,2-*b*]pyridine (**5**) and 200 mg (1.160 mmol) of quinolin-5-ylboronic acid; the reaction time was 2 h, flash chromatography (cyclohexane/EtOAc, gradient from 0% to 100% of EtOAc) afforded the compound **32** as a white solid (140 mg, 52% yield). ^1^H NMR (500 MHz, Chloroform-*d*) δ 8.96 (dd, *J* = 4.1, 1.7 Hz, 1H), 8.57 (d, *J* = 2.0 Hz, 1H), 8.27 (ddd, *J* = 8.6, 1.7, 0.9 Hz, 1H), 8.21 (dt, *J* = 8.4, 1.1 Hz, 1H), 8.06 (s, 1H), 7.93 (d, *J* = 2.0 Hz, 1H), 7.82 (dd, *J* = 8.4, 7.1 Hz, 1H), 7.77 (dd, *J* = 7.1, 1.3 Hz, 1H), 7.39 (dd, *J* = 8.5, 4.1 Hz, 1H). ^13^C NMR (126 MHz, Chloroform-*d*) δ 150.75, 148.93, 148.12, 147.67, 145.85, 145.21, 134.24, 130.61, 129.30, 128.75, 128.25, 127.35, 127.29, 121.46, 120.92, 119.23. FTIR (neat), cm^–1^: 3062, 1593, 1505, 1451, 1407, 1386, 1316, 1276, 1145, 1081, 1064, 1033, 938, 914, 874, 862, 826, 799, 777, 752, 663, 599, 537, 491, 447, 421, HRMS (APCI): calcd. for C_16_H_9_ClN_2_O [M + H]^+^ = 281.0476; found [M + H]^+^ = 281.0474. MP: 195–196 °C

### *tert*-Butyl 4-(4-(3-(quinolin-5-yl)furo[3,2-*b*]pyridin-6-yl)phenyl)piperazine-1-carboxylate (**33**)

The compound was prepared by the general procedure **B** using 100 mg (0.356 mmol) of 6-chloro-3-(quinolin-5-yl)furo[3,2-*b*]pyridine (**32**) and 166 mg (0.427 mmol) of 4-(4-*tert*-butoxycarbonylpiperazinyl)phenylboronic acid pinacol ester; the reaction time was 2 h; flash chromatography (cyclohexane/EtOAc, gradient from 0% to 67% of EtOAc) afforded the compound **33** as a white solid (163 mg, 90% yield). ^1^H NMR (500 MHz, Chloroform-*d*) δ 8.96 (dd, *J* = 4.2, 1.7 Hz, 1H), 8.82 (d, *J* = 1.9 Hz, 1H), 8.37 (ddd, *J* = 8.6, 1.7, 0.9 Hz, 1H), 8.20 (ddd, *J* = 7.3, 2.5, 0.9 Hz, 1H), 8.05 (s, 1H), 8.01 (d, *J* = 1.9 Hz, 1H), 7.86–7.78 (m, 1H), 7.81 (s, 1H), 7.58 (d, *J* = 8.7 Hz, 2H), 7.40 (dd, *J* = 8.6, 4.1 Hz, 1H), 7.05 (d, *J* = 8.8 Hz, 2H), 3.65–3.58 (m, 4H), 3.27–3.20 (m, 4H), 1.50 (s, 9H). ^13^C NMR (126 MHz, Chloroform-*d*) δ 154.89, 151.22, 150.64, 148.99, 148.94, 146.94, 145.57, 145.14, 134.53, 133.65, 130.27, 129.33, 128.64, 128.40, 128.07, 127.48, 121.36, 120.82, 116.86, 116.39, 80.17, 49.05, 43.62, 28.59. FTIR (neat), cm^–1^: 2971, 2928, 2824, 1684, 1604, 1521, 1475, 1459, 1410, 1378, 1364, 1339, 1232, 1156, 1129. HRMS (APCI): calcd. for C_31_H_30_N_4_O_3_ [M + H]^+^ = 507.2391; found [M + H]^+^ = 507.2394. MP: 179–180 °C

### 6-(4-(Piperazin-1-yl)phenyl)-3-(quinolin-5-yl)furo[3,2-*b*]pyridine (**34**)

The compound was prepared by the general procedure **C** using 90 mg (0.177 mmol) of *tert*- butyl 4-(4-(3-(quinolin-5-yl)furo[3,2-*b*]pyridin-6-yl)phenyl)piperazine-1-carboxylate (**33**); the reaction time was 2 h; flash chromatography (DCM/MeOH, gradient from 0% to 20% of MeOH) afforded the compound as a pale yellow solid (25 mg, 35% yield). ^1^H NMR (500 MHz, DMSO-*d*_6_) δ 8.97 (dd, *J* = 4.1, 1.6 Hz, 1H), 8.85 (d, *J* = 1.9 Hz, 1H), 8.67 (s, 1H), 8.42 (d, *J* = 1.9 Hz, 1H), 8.39 (ddd, *J* = 8.6, 1.5, 0.8 Hz, 1H), 8.16–8.11 (m, 2H), 7.93–7.86 (m, 1H), 7.89 (s, 1H), 7.75 (d, *J* = 8.9 Hz, 2H), 7.54 (dd, *J* = 8.6, 4.1 Hz, 1H), 7.13 (d, *J* = 8.9 Hz, 2H), 3.50–3.41 (m, 4H), 3.24–3.17 (m, 4H). ^13^C NMR (126 MHz, DMSO-*d*_6_) δ 150.54, 149.83, 148.32, 148.25, 148.04, 144.49, 144.44, 134.25, 132.39, 129.32, 129.08, 128.36, 128.09, 128.00, 127.91, 126.52, 121.49, 119.32, 116.18, 115.91, 45.37, 42.72. FTIR (neat), cm^–1^: 2848, 1606, 1524, 1479, 1459, 1376, 1247, 1132, 1098, 1052, 941, 917, 890, 832, 820, 807, 619, 548, 526, 508, 443. HRMS (APCI): calcd. for C_26_H_22_N_4_O [M + H]^+^ = 407.1866; found [M + H]^+^ = 407.1869. MP: 236–237 °C

### 6-Chloro-3-(isoquinolin-8-yl)furo[3,2-*b*]pyridine (**35**)

The compound was prepared by the general procedure **A** using 217 mg (0.93 mmol) of 3-bromo-6-chlorofuro[3,2-*b*]pyridine (**5**) and 194 mg (1.12 mmol) of isoquinolin-8-ylboronic acid; the reaction time was 3.5 h; flash chromatography (cyclohexane/EtOAc, gradient from 0% to 40% of EtOAc) afforded the compound **35** as a white solid (115 mg, 44% yield). ^1^H NMR (500 MHz, Chloroform-*d*) δ 9.39 (s, 1H), 8.61–8.56 (m, 2H), 8.12 (s, 1H), 7.94 (d, *J* = 2.1 Hz, 1H), 7.90 (d, *J* = 8.1 Hz, 1H), 7.84 (dd, *J* = 7.1, 1.4 Hz, 1H), 7.80 (dd, *J* = 8.1, 7.1 Hz, 1H), 7.73 (dd, *J* = 5.7, 1.0 Hz, 1H). ^13^C NMR (126 MHz, Chloroform-*d*) δ 150.75, 148.11, 147.82, 145.96, 145.11, 143.30, 136.70, 130.31, 129.58, 128.34, 127.80, 127.47, 127.09, 120.92, 120.13, 119.27, 77.16. FTIR (neat), cm^–1^: 3120, 3096, 3040, 1619, 1586, 1463, 1400, 1384, 1282, 1266, 1187, 1148, 1098, 1083, 1075, 1026, 942, 912, 879, 825, 796, 777, 754, 665, 597, 522, 494, 462, 445, 415. HRMS (APCI): calcd. for C_16_H_9_ClN_2_O [M + H]^+^ = 281.0476; found [M + H]^+^ = 281.0477. MP: 211–212 °C

### *tert*-Butyl 4-(4-(3-(isoquinolin-8-yl)furo[3,2-*b*]pyridin-6-yl)phenyl)piperazine-1-carboxylate (**36**)

The compound was prepared by the general procedure **B** using 85 mg (0.303 mmol) of 6-chloro-3-(isoquinolin-8-yl)furo[3,2-*b*]pyridine (**35**) and 141 mg (0.363 mmol) of 4-(4-*tert*-butoxycarbonylpiperazinyl)phenylboronic acid pinacol ester; the reaction time was 2 h; flash chromatography (cyclohexane/EtOAc, gradient from 0% to 60% of EtOAc) afforded the compound **36** as a white wax (146 mg, 95% yield). ^1^H NMR (500 MHz, Chloroform-*d*) δ 9.47 (s, 1H), 8.82 (d, *J* = 1.9 Hz, 1H), 8.58 (d, *J* = 5.7 Hz, 1H), 8.12 (s, 1H), 8.02 (d, *J* = 1.9 Hz, 1H), 7.90 (d, *J* = 7.7 Hz, 2H), 7.84–7.77 (m, 1H), 7.73 (d, *J* = 5.0 Hz, 1H), 7.58 (d, *J* = 8.8 Hz, 1H), 7.05 (d, *J* = 8.8 Hz, 1H), 3.62 (dd, *J* = 6.2, 4.2 Hz, 4H), 3.33–3.15 (m, 4H), 1.50 (s, 9H). ^13^C NMR (126 MHz, Chloroform-*d*) δ 154.87, 151.20, 150.92, 148.99, 147.10, 145.72, 145.01, 142.98, 136.76, 133.78, 130.45, 129.52, 129.33, 128.64, 128.43, 127.20, 127.16, 120.94, 120.00, 116.86, 116.45, 80.15, 49.05, 43.63, 28.59. FTIR (neat), cm^–1^: 3041, 2973, 2819, 1687, 1607, 1524, 1477, 1419, 1378, 1364, 1263, 1233, 1160, 1121, 1095, 1046, 999, 910, 820, 796, 752, 728, 670, 604, 537. HRMS (APCI): calcd. for C_31_H_30_N_4_O_3_ [M + H]^+^ = 507.2391; found [M + H]^+^ = 507.2395

### 3-(Isoquinolin-8-yl)-6-(4-(piperazin-1-yl)phenyl)furo[3,2-*b*]pyridine (**37**)

The compound was prepared by the general procedure **C** using 120 mg (0.237 mmol) of *tert*-butyl 4-(4-(3-(isoquinolin-8-yl)furo[3,2-*b*]pyridin-6-yl)phenyl)piperazine-1-carboxylate (**36**); the reaction time was 2 h; flash chromatography (DCM/MeOH, gradient from 0% to 20% of MeOH) provided impure product which was suspended in EtOAc (5 mL) and sonicated for 1 min. The solid was collected by filtration and washed on filter with H_2_O (2 mL) and then with Et_2_O (2 mL). The product was obtained as yellow solid (30 mg, 31% yield). ^1^H NMR (500 MHz, DMSO-*d*_6_) δ 9.36 (s, 1H), 8.84 (d, *J* = 1.9 Hz, 1H), 8.73 (s, 1H), 8.57 (d, *J* = 5.6 Hz, 1H), 8.40 (d, *J* = 1.9 Hz, 1H), 8.08 (dd, *J* = 7.6, 1.9 Hz, 1H), 8.04–7.85 (m, 3H), 7.71 (d, *J* = 8.5 Hz, 2H), 7.07 (d, *J* = 8.5 Hz, 2H), 3.21 (dd, *J* = 6.4, 3.7 Hz, 4H), 2.95 (dd, *J* = 6.3, 3.7 Hz, 4H). ^13^C NMR (126 MHz, DMSO-*d*_6_) δ 150.99, 150.58, 148.41, 148.30, 144.46, 144.22, 142.86, 135.79, 132.71, 130.22, 129.38, 128.04, 127.78, 127.06, 126.91, 126.16, 120.53, 118.91, 115.79, 115.60, 48.03, 44.81, 39.52. FTIR (neat), cm^–1^: 3296, 2943, 2831, 2467, 1604, 1523, 1479, 1377, 1236, 1218, 1141, 1090, 888, 832, 797, 778, 752, 674, 528, 508. HRMS (APCI): calcd. for C_26_H_22_N_4_O [M + H]^+^ = 407.1866; found [M + H]^+^ = 407.1863. MP: 217–218 °C

### 6-Chloro-3-(1*H*-pyrrolo[2,3-*b*]pyridin-4-yl)furo[3,2-*b*]pyridine (**38**)

The compound was prepared by the general procedure **A** using 100 mg (0.430 mmol) of 3-bromo-6-chlorofuro[3,2-*b*]pyridine (**5**) and 91 mg (0.515 mmol) of (1*H*-pyrrolo[2,3-*b*]pyridin-4-yl)boronic acid; the reaction time was 4 h; flash chromatography (cyclohexane/EtOAc, gradient from 40% to 60% of EtOAc) afforded the compound **38** as a white solid (82 mg, 71% yield). ^1^H NMR (500 MHz, DMSO-*d*_6_) δ 11.84 (s, 1H), 9.15 (s, 1H), 8.75 (d, *J* = 2.1 Hz, 1H), 8.50 (d, *J* = 2.1 Hz, 1H), 8.35 (d, *J* = 4.9 Hz, 1H), 8.21 (d, *J* = 4.9 Hz, 1H), 7.59 (dd, *J* = 3.5, 2.4 Hz, 1H), 6.89 (dt, *J* = 3.5, 1.0 Hz, 1H). ^13^C NMR (126 MHz, DMSO-*d*_6_) δ 150.05, 149.02, 148.87, 147.68, 144.95, 143.94, 142.61, 128.84, 127.01, 126.52, 126.35, 119.58, 118.17, 116.17, 114.26, 99.94. FTIR (neat), cm^–1^: 3135, 3105, 2920, 1897, 1727, 1668, 1605, 1462, 1382, 1329, 1280, 1233, 1126, 1104, 1074, 923, 897, 875, 810, 776, 701, 657, 640, 586, 539, 467. HRMS (APCI): calcd. for C_14_H_8_ClN_3_O [M + H]^+^ = 270.0429; found [M + H]^+^ = 270.0429. MP: 249–250 °C.

### 6-(4-(4-Methylpiperazin-1-yl)phenyl)-3-(1*H*-pyrrolo[2,3-*b*]pyridin-4-yl)furo[3,2-*b*]pyridine (**39**)

The compound was prepared by the general procedure **B** using 70 mg (0.260 mmol) of 6-chloro-3-(1*H*-pyrrolo[2,3-*b*]pyridin-4-yl)furo[3,2-*b*]pyridine (**38**) and 102 mg (0.370 mmol) of 1-methyl-4-(4-(4,4,5,5-tetramethyl-1,3,2-dioxaborolan-2-yl)phenyl)piperazine; the reaction time was 2 h; flash chromatography (EtOAc/MeOH, gradient from 0% to 20% of MeOH) afforded the compound **39** as a yellow solid (7 mg, 7% yield). ^1^H NMR (500 MHz, DMSO-*d*_6_) δ 11.80 (s, 1H), 9.07 (s, 1H), 8.98 (d, *J* = 1.9 Hz, 1H), 8.38 (d, *J* = 1.9 Hz, 1H), 8.37–8.33 (m, 2H), 7.72 (d, *J* = 8.9 Hz, 2H), 7.59 (dd, *J* = 3.5, 2.4 Hz, 1H), 7.13–7.05 (m, 2H), 6.93 (dd, *J* = 3.6, 1.6 Hz, 1H), 3.26–3.22 (m, 4H), 2.47 (d, *J* = 5.1 Hz, 4H), 2.24 (s, 3H). ^13^C NMR (126 MHz, DMSO-*d*_6_) δ 150.78, 149.03, 148.90, 148.55, 144.41, 143.37, 142.60, 132.57, 129.54, 127.76, 126.76, 126.31, 118.18, 116.15, 115.67, 115.50, 114.27, 100.04, 54.46, 47.63, 45.73. FTIR (neat), cm^–1^: 2843, 2801, 1599, 1583, 1525, 1478, 1380, 1342, 1292, 1244, 1214, 1121, 1007, 808, 716, 584, 524, 454. HRMS (APCI): calcd. for C_25_H_23_N_5_O [M + H]^+^ = 410.1975; found [M + H]^+^ = 410.1976. MP: > 250 °C.

### 3-(1-Benzofuran-3-yl)-6-chlorofuro[3,2-*b*]pyridine (**40**)

The compound was prepared by the general procedure **A** using 100 mg (0.430 mmol) of 3-bromo-6-chlorofuro[3,2-*b*]pyridine (**5**) and 84 mg (0.515 mmol) of (1-benzofuran-3-yl)boronic acid; the reaction time was 18 h; flash chromatography (cyclohexane/EtOAc, gradient from 0% to 10% of EtOAc) afforded the compound **40** as a white solid (88 mg, 76% yield). ^1^H NMR (500 MHz, Chloroform-*d*) δ 8.71 (s, 1H), 8.62 (d, *J* = 2.0 Hz, 1H), 8.31 (s, 1H), 7.84 (d, *J* = 2.0 Hz, 1H), 7.81–7.75 (m, 1H), 7.65–7.55 (m, 1H), 7.47–7.32 (m, 2H). ^13^C NMR (126 MHz, Chloroform-*d*) δ 155.38, 147.82, 145.39, 144.60, 144.44, 144.40, 128.13, 125.73, 124.98, 123.35, 120.48, 118.81, 114.40, 112.12, 110.26. FTIR (neat), cm^–1^: 1451, 1379, 1279, 1151, 1106, 1078, 924, 899, 870, 856, 784, 764, 738, 596, 580, 567, 451, 423. HRMS (APCI): calcd. for C_15_H_8_ClNO_2_ [M + H]^+^ = 270.0316; found [M + H]^+^ = 270.0318. MP: 152–153 °C

### 3-(Benzofuran-3-yl)-6-(4-(4-methylpiperazin-1-yl)phenyl)furo[3,2-*b*]pyridine (**41**)

The compound was prepared by the general procedure **B** using 87 mg (0.323 mmol) of 3-(1-benzofuran-3-yl)-6-chlorofuro[3,2-*b*]pyridine (**40**) and 127 mg (0.419 mmol) of 1-methyl-4-(4-(4,4,5,5-tetramethyl-1,3,2-dioxaborolan-2-yl)phenyl)piperazine; the reaction time was 2 h; flash chromatography (DCM/MeOH, gradient from 1:0 to 10:1 of MeOH) afforded the compound as a white solid (49 mg, 37% yield). ^1^H NMR (500 MHz, DMSO-*d*_6_) δ 9.06 (s, 1H), 8.96 (d, *J* = 1.9 Hz, 1H), 8.90 (s, 1H), 8.35 (d, *J* = 1.9 Hz, 1H), 8.18 (dd, *J* = 7.7, 1.5 Hz, 1H), 7.78–7.65 (m, 3H), 7.44 (dtd, *J* = 18.6, 7.3, 1.3 Hz, 2H), 7.10–7.05 (m, 2H), 3.27–3.20 (m, 4H), 2.49–2.45 (m, 4H), 2.24 (s, 3H). ^13^C NMR (126 MHz, DMSO-*d*_6_) δ 154.26, 150.41, 147.80, 144.98, 144.01, 143.09, 143.00, 132.53, 127.35, 126.60, 124.83, 124.51, 122.78, 120.83, 115.13, 115.02, 112.40, 111.07, 110.30, 54.07, 47.37, 45.15. FTIR (neat), cm^–1^: 2931, 2848, 2807, 1605, 1521, 1454, 1380, 1328, 1290, 1244, 1144, 1103, 1078, 1002, 923, 899, 857, 818, 733, 528, 447, 421. HRMS (APCI): calcd. for C_26_H_23_N_3_O_2_ [M + H]^+^ = 410.1863; found [M + H]^+^ = 410.1864. MP: 218–219 °C

### 3-(Benzo[*b*]thiophen-3-yl)-6-chlorofuro[3,2-*b*]pyridine (**42**)

The compound was prepared by the general procedure **A** using 150 mg (0.645 mmol) of 3-bromo-6-chlorofuro[3,2-*b*]pyridine (**5**) and 149 mg (0.839 mmol) of benzo[*b*]thiophen-3-ylboronic acid. The reacion time was 2 h. The reaction mixture was hot-filtered through a pad of the mixture Celite 535/SiO_2_ = 3:1 (4 g) and the filtrate was concentrated in vacuo. Flash chromatography (eluent: cyclohexane) provided the compound **42** as a white solid (142 mg, 77% yield)). ^1^H NMR (500 MHz, Chloroform-*d*) δ 8.63 (d, *J* = 2.0 Hz, 1H), 8.34 (s, 1H), 8.30 (s, 1H), 8.01–7.94 (m, 2H), 7.88 (d, *J* = 2.0 Hz, 1H), 7.44 (dddd, *J* = 21.1, 8.2, 7.0, 1.3 Hz, 2H). ^13^C NMR (126 MHz, Chloroform-*d*) δ 147.95, 145.54, 145.50, 144.92, 140.54, 137.56, 128.09, 126.66, 124.79, 124.77, 124.70, 123.29, 122.72, 118.93, 117.23. FTIR (neat), cm^–1^: 2949, 2820, 2803, 1454, 1381, 1247, 1067, 914, 869, 829, 780, 755, 725, 703, 596, 485. HRMS (APCI): calcd. For C_15_H_8_ClNOS [M + H]^+^ = 286.0088; found [M + H]^+^ = 286.0090. MP: 127–128 °C

### 3-(Benzo[*b*]thiophen-3-yl)-6-(4-(4-methylpiperazin-1-yl)phenyl)furo[3,2-*b*]pyridine (**43**)

The compound was prepared by the general procedure **B** using 100 mg (0.350 mmol) of 3-(benzo[*b*]thiophen-3-yl)-6-chlorofuro[3,2-*b*]pyridine (**42**) and 0.127 g (0.420 mmol) of 1-methyl-4-(4-(4,4,5,5-tetramethyl-1,3,2-dioxaborolan-2-yl)phenyl)piperazine. Mixture of 1,4-dioxane and water (4:1; 5 mL) was used as a solvent. The reaction time was 2 h. The reaction mixture was hot-filtered through a pad of Celite 535 (4 g) and the filtrate was concentrated in vacuo. Flash chromatography (DCM/MeOH, gradient from 0 to 4% of MeOH) afforded the product **43** as a white solid (79.7 mg, 54%). ^1^H NMR (500 MHz, DMSO-*d*_6_) δ 8.97 (s, 1H), 8.94 (d, *J* = 2.0 Hz, 1H), 8.66 (s, 1H), 8.36 (d, *J* = 1.8 Hz, 1H), 8.25–8.21 (m, 1H), 8.14–8.10 (m, 1H), 7.74–7.69 (m, 2H), 7.54–7.46 (m, 2H), 7.11–7.06 (m, 2H), 3.25–3.21 (m, 4H), 2.47 (d, *J* = 5.2 Hz, 4H), 2.24 (s, 3H). ^13^C NMR (126 MHz, DMSO-*d*_6_) δ 150.78, 148.12, 146.05, 144.33, 143.73, 139.51, 136.90, 132.69, 127.78, 126.85, 126.23, 125.02, 124.74, 124.65, 123.15, 115.61, 115.56, 115.52, 54.47, 47.64, 45.74. FTIR (neat), cm^–1^: 2935, 2839, 2798, 1601, 1448, 1378, 1290, 1239, 1185, 1139, 1095, 1056, 1007, 915, 887, 821, 781, 756, 729, 693, 530. HRMS (APCI): calcd. For C_26_H_23_N_3_OS [M + H]^+^ = 426.1635; found [M + H]^+^ = 426.1637. MP: 216–217 °C

### 6-Chloro-3-(2,6-dimethylpyridin-4-yl)furo[3,2-*b*]pyridine (**44**)

The compound was prepared by the general procedure **A** using 332 mg (1.430 mmol) of 3-bromo-6-chlorofuro[3,2-*b*]pyridine (**5**) and 400 mg (1.716 mmol) of 2,6-dimethyl-4-(4,4,5,5-tetramethyl-1,3,2-dioxaborolan-2-yl)pyridine; the reaction time was 1 h; flash chromatography (cyclohexane/EtOAc, gradient from 0% to 100% of EtOAc) afforded the compound **44** as a yellow solid (300 mg, 81% yield). ^1^H NMR (500 MHz, Chloroform-*d*) δ 8.62 (d, *J* = 2.0 Hz, 1H), 8.20 (s, 1H), 7.83 (d, *J* = 2.0 Hz, 1H), 7.62 (s, 2H), 2.60 (s, 6H). ^13^C NMR (126 MHz, Chloroform-*d*) δ 158.56, 148.60, 147.05, 145.65, 143.95, 138.13, 128.12, 120.08, 119.05, 117.94, 77.16, 24.81. FTIR (neat), cm^–1^: 3142, 3019, 2959, 2920, 2850, 1617, 1550, 1383, 1372, 1350, 1277, 1131, 1107, 1081, 910, 896, 880, 846, 806, 782, 597, 561, 546, 525, 456. HRMS (APCI): calcd. for C_14_H_11_ClN_2_O [M + H]^+^ = 259.0633, found = 259.0636. MP: 160–161 °C

### *tert*-Butyl 4-(4-(3-(2,6-dimethylpyridin-4-yl)furo[3,2-*b*]pyridin-6-yl)phenyl)piperazine-1-carboxylate (**45**)

The compound was prepared by the general procedure **B** using 112 mg (0.433 mmol) of 6-chloro-3-(2,6-dimethylpyridin-4-yl)furo[3,2-*b*]pyridine (**44**) and 202 mg (0.520 mmol) of 4-(4-*tert*-butoxycarbonylpiperazinyl)phenylboronic acid pinacol ester; the reaction time was 1 h; flash chromatography (cyclohexane/EtOAc, gradient from 20% to 60% of EtOAc) afforded the compound **45** as a white solid (122 mg, 58% yield). ^1^H NMR (500 MHz, Chloroform-*d*) δ 8.90 (d, *J* = 1.9 Hz, 1H), 8.22 (s, 1H), 7.93 (d, *J* = 1.9 Hz, 1H), 7.70 (s, 2H), 7.57 (d, *J* = 8.7 Hz, 2H), 7.04 (d, *J* = 8.8 Hz, 2H), 3.66–3.54 (m, 4H), 3.23 (t, *J* = 5.2 Hz, 4H), 2.62 (s, 6H), 1.50 (s, 9H). ^13^C NMR (126 MHz, Chloroform-*d*) δ 158.48, 154.87, 151.22, 149.57, 146.43, 145.41, 143.93, 138.86, 133.46, 129.23, 128.36, 120.03, 118.02, 116.85, 116.24, 80.17, 49.04, 43.55, 28.59, 24.85. FTIR (neat), cm^–1^: 2978, 1678, 1608, 1524, 1430, 1388, 1365, 1239, 1180, 826, 812, 546, 459, 413. HRMS (APCI): calcd. for C_29_H_32_N_4_O_3_ [M + H]^+^ = 485.2547, found = 485.2549. MP: 202–203 °C

### 3-(2,6-Dimethylpyridin-4-yl)-6-(4-(piperazin-1-yl)phenyl)furo[3,2-*b*]pyridine (**46**)

TFA (0.5 mL, 6.535 mmol) was added to a solution of *tert*-butyl 4-(4-(3-(2,6-dimethylpyridin-4-yl)furo[3,2-*b*]pyridin-6-yl)phenyl)piperazine-1-carboxylate (**45**; 100 mg, 0.206 mmol) in DCM (5 mL) and the reaction mixture was stirred at 23 °C for 2 h. All volatiles were evaporated in vacuo, the residue was dissolved in acetonitrile (5 mL), triethylamine (0.15 mL) was added, and the mixture was allowed to stir for 2 min. The product was collected by filtration as a yellow solid (56 mg, 71% yield). ^1^H NMR (500 MHz, Methanol-*d*_4_) δ 8.87 (d, *J* = 1.9 Hz, 1H), 8.64 (s, 1H), 8.12 (d, *J* = 1.9 Hz, 1H), 7.88 (s, 2H), 7.65 (d, *J* = 8.8 Hz, 2H), 7.13 (d, *J* = 8.9 Hz, 2H), 3.33–3.27 (m, 4H), 3.12–3.07 (m, 4H), 2.61 (s, 6H). ^13^C NMR (126 MHz, Methanol-*d*_4_) δ 159.05, 152.95, 151.05, 149.48, 145.84, 144.41, 141.31, 134.96, 129.72, 129.03, 120.13, 119.44, 117.61, 117.27, 50.21, 46.32, 23.84. FTIR (neat), cm^–1^: 1667, 1604, 1523, 1480, 1375, 1229, 1199, 1182, 1120, 1099, 827, 808, 539. HRMS (APCI): calcd. for C_24_H_24_N_4_O [M + H]^+^ = 385.2023; found [M + H]^+^ = 385.2025. MP: 210–211 °C

### 6-Chloro-3-(pyridin-4-yl)furo[3,2-*b*]pyridine (**47**)

The compound was prepared by the general procedure **A** using 200 mg (0.86 mmol) of 3-bromo-6-chlorofuro[3,2-*b*]pyridine (**5**) and 137 mg (1.120 mmol) of pyridin-4-ylboronic acid; the reaction time was 1 h; flash chromatography (cyclohexane/EtOAc, gradient from 0% to 50% of EtOAc) afforded the compound **47** as a yellow solid (108 mg, 54% yield). ^1^H NMR (500 MHz, Chloroform-*d*) δ 8.71 (d, *J* = 6.2 Hz, 2H), 8.65 (d, *J* = 2.0 Hz, 1H), 8.28 (s, 1H), 8.07–7.98 (m, 2H), 7.88 (d, *J* = 2.1 Hz, 1H). ^13^C NMR (126 MHz, Chloroform-*d*) δ 150.18, 148.74, 147.29, 145.91, 143.75, 138.22, 128.43, 121.47, 119.64, 119.25. FTIR (neat), cm^–1^: 3062, 1610, 1422, 1388, 1284, 1231, 1144, 1090, 995, 979, 914, 880, 824, 729, 655, 618, 600, 518, 427. HRMS (APCI): calcd. for C_12_H_7_ClN_2_O [M + H]^+^ = 231.0320; found [M + H]^+^ = 231.0319.

### *tert*-Butyl 4-(4-(3-(pyridin-4-yl)furo[3,2-*b*]pyridin-6-yl)phenyl)piperazine-1-carboxylate (**48**)

The compound was prepared by the general procedure **B** using 100 mg (0.434 mmol) of 6-chloro-3-(pyridin-4-yl)furo[3,2-*b*]pyridine (**47**) and 202 mg (0.520 mmol) of 4-(4-*tert*-butoxycarbonylpiperazinyl)phenylboronic acid pinacol ester; the reaction time was 2 h; flash chromatography (cyclohexane/EtOAc, gradient from 0% to 66% of EtOAc) afforded the compound **48** as a yellow solid (184 mg, 93% yield). ^1^H NMR (500 MHz, Chloroform-*d*) δ 8.90 (d, *J* = 1.9 Hz, 1H), 8.74–8.69 (m, 2H), 8.28 (s, 1H), 8.10–8.06 (m, 2H), 7.95 (d, *J* = 1.9 Hz, 1H), 7.61–7.55 (m, 2H), 7.11–7.01 (m, 2H), 3.62 (dd, *J* = 6.3, 4.1 Hz, 4H), 3.24 (dd, *J* = 6.2, 4.1 Hz, 4H), 1.50 (s, 9H). ^13^C NMR (126 MHz, Chloroform-*d*) δ 154.88, 151.28, 150.14, 149.68, 146.60, 145.59, 143.67, 138.86, 133.73, 129.08, 128.39, 121.50, 119.55, 116.85, 116.34, 80.18, 49.02, 43.70, 28.60. FTIR (neat), cm^–1^: 2973, 2929, 2833, 1683, 1603, 1524, 1480, 1411, 1379, 1363, 1342, 1263, 1239, 1203, 1158, 1124, 1108, 1046, 907, 823, 811, 775, 670, 649, 551, 532. HRMS (APCI): calcd. for C_27_H_28_N_4_O_3_ [M + H]^+^ = 457.2234; found [M + H]^+^ = 457.2238.

### 6-(4-(Piperazin-1-yl)phenyl)-3-(pyridin-4-yl)furo[3,2-*b*]pyridine (**49**)

The compound was prepared by the general procedure **C** using 90 mg (0.197 mmol) of *tert*-butyl 4-(4-(3-(pyridin-4-yl)furo[3,2-*b*]pyridin-6-yl)phenyl)piperazine-1-carboxylate (**48**); the reaction time was 2 h; flash chromatography (DCM/MeOH, gradient from 0% to 20% of MeOH) afforded the compound as a pale yellow solid (40 mg, 57% yield). ^1^H NMR (500 MHz, DMSO-*d*_6_) δ ^1^H NMR (500 MHz, DMSO-*d*_6_) δ 9.36 (s, 1H), 9.10 (s, 1H), 8.98 (d, *J* = 1.9 Hz, 1H), 8.69 (d, *J* = 5.1 Hz, 2H), 8.39 (d, *J* = 1.9 Hz, 1H), 8.26 (d, *J* = 5.3 Hz, 2H), 7.75 (d, *J* = 8.5 Hz, 2H), 7.13 (d, *J* = 8.6 Hz, 2H), 3.48 (t, *J* = 5.1 Hz, 4H), 3.20 (t, *J* = 5.1 Hz, 4H). ^13^C NMR (126 MHz, Chloroform-*d*) δ 150.07, 149.86, 149.08, 148.98, 144.69, 142.95, 137.90, 132.42, 127.92, 127.81, 120.72, 117.80, 116.14, 116.05, 45.13, 42.42. FTIR (neat), cm^–1^: 2926, 2753, 2699, 2613, 2490, 2470, 1604, 1527, 1383, 1251, 1211, 1146, 1124, 1103, 929, 806, 663, 526. HRMS (APCI): calcd. for C_22_H_20_N_4_O [M + H]^+^ = 357.1710; found [M + H]^+^ = 357.1708.

### 6-Chloro-3-(pyridin-3-yl)furo[3,2-*b*]pyridine (**50**)

The compound was prepared by the general procedure **A** using 200 mg (0.86 mmol) of 3-bromo-6-chlorofuro[3,2-*b*]pyridine (**5**) and 137 mg (1.120 mmol) of pyridin-3-ylboronic acid; the reaction time was 1 h; flash chromatography (cyclohexane/EtOAc, gradient from 0% to 50% of EtOAc) afforded the compound **50** as a yellow solid (104 mg, 52% yield). ^1^H NMR (500 MHz, Chloroform-*d*) δ 9.18 (d, *J* = 2.4 Hz, 1H), 8.67–8.60 (m, 2H), 8.49 (dt, *J* = 7.9, 2.0 Hz, 1H), 8.19 (s, 1H), 7.86 (d, *J* = 2.0 Hz, 1H), 7.47–7.39 (m, 1H). ^13^C NMR (126 MHz, Chloroform-*d*) δ 148.96, 148.52, 147.82, 145.73, 145.69, 144.10, 134.84, 128.24, 126.45, 123.94, 119.10, 119.02. FTIR (neat), cm^–1^: 3070, 3039, 1610, 1468, 1425, 1388, 1365, 1326, 1281, 1142, 1093, 1077, 1030, 968, 911, 891, 801, 787, 733, 704, 619, 597, 528. HRMS (APCI): calcd. for C_12_H_7_ClN_2_O [M + H]^+^ = 231.0320; found [M + H]^+^ = 231.0322.

### *tert*-Butyl 4-(4-(3-(pyridin-3-yl)furo[3,2-*b*]pyridin-6-yl)phenyl)piperazine-1-carboxylate (**51**)

The compound was prepared by the general procedure **B** using 96 mg (0.416 mmol) of 6-chloro-3-(pyridin-3-yl)furo[3,2-*b*]pyridine (**50**) and 194 mg (0.499 mmol) of 4-(4-*tert*-butoxycarbonylpiperazinyl)phenylboronic acid pinacol ester; the reaction time was 2 h; flash chromatography (cyclohexane/EtOAc, gradient from 0% to 66% of EtOAc) afforded the compound **51** as a yellow solid (171 mg, 90% yield). ^1^H NMR (500 MHz, Chloroform-*d*) δ 9.21 (dd, *J* = 2.3, 0.9 Hz, 1H), 8.88 (d, *J* = 1.9 Hz, 1H), 8.61 (dd, *J* = 4.9, 1.7 Hz, 1H), 8.57 (ddd, *J* = 7.9, 2.2, 1.7 Hz, 1H), 8.19 (s, 1H), 7.95 (d, *J* = 1.9 Hz, 1H), 7.58 (d, *J* = 8.8 Hz, 2H), 7.44 (ddd, *J* = 7.8, 4.8, 0.9 Hz, 1H), 7.05 (d, *J* = 8.9 Hz, 2H), 3.62 (dd, *J* = 6.3, 4.1 Hz, 4H), 3.23 (dd, *J* = 6.3, 4.1 Hz, 4H), 1.50 (s, 9H). ^13^C NMR (126 MHz, Chloroform-*d*) δ 154.88, 151.22, 149.42, 148.61, 147.78, 145.42, 145.04, 144.04, 134.90, 133.56, 129.25, 128.39, 127.13, 123.95, 118.89, 116.86, 116.26, 80.17, 49.05, 43.66, 28.60. FTIR (neat), cm^–1^ 2977, 2929, 2900, 2857, 2823, 1681, 1608, 1526, 1483, 1462, 1421, 1380, 1362, 1342, 1283, 1239, 1204, 1161, 1128, 1097, 1048, 966, 909, 821, 795, 765, 706, 546, 524. HRMS (APCI): calcd. for C_27_H_28_N_4_O_3_ [M + H]^+^ = 457.2234; found [M + H]^+^ = 457.2237. MP: 165–166 °C

### 6-(4-(Piperazin-1-yl)phenyl)-3-(pyridin-3-yl)furo[3,2-*b*]pyridine (**52**)

The compound was prepared by the general procedure **C** using 90 mg (0.197 mmol) of *tert*-butyl 4-(4-(3-(pyridin-3-yl)furo[3,2-*b*]pyridin-6-yl)phenyl)piperazine-1-carboxylate (**51**); the reaction time was 2 h; flash chromatography (DCM/MeOH, gradient from 0% to 20% of MeOH) afforded the compound as a pale yellow solid (71 mg, 100% yield). ^1^H NMR (300 MHz, DMSO-*d*_6_) δ 9.43 (d, *J* = 2.2 Hz, 1H), 8.96 (s, 2H), 8.62 (dd, *J* = 8.0, 2.1 Hz, 1H), 8.58 (dd, *J* = 4.8, 1.7 Hz, 1H), 8.35 (d, *J* = 1.9 Hz, 1H), 7.71 (d, *J* = 8.5 Hz, 2H), 7.54 (dd, *J* = 7.9, 4.8 Hz, 1H), 7.06 (d, *J* = 8.5 Hz, 2H), 3.16 (dd, *J* = 6.4, 3.7 Hz, 4H), 2.89 (t, *J* = 5.0 Hz, 4H). ^13^C NMR (126 MHz, DMSO-*d*_6_) δ 151.25, 148.79, 148.45, 147.41, 147.08, 144.45, 142.98, 133.56, 132.57, 127.72, 126.68, 126.57, 123.73, 117.33, 115.68, 115.45, 48.57, 45.23. FTIR (neat), cm^–1^: 3289, 3033, 2945, 2825, 2748, 1604, 1522, 1481, 1449, 1377, 1333, 1234, 1201, 1144, 1125, 1099, 966, 946, 884, 822, 803, 787, 704, 683, 608, 537. HRMS (APCI): calcd. for C_22_H_20_N_4_O [M + H]^+^ = 357.1710; found [M + H]^+^ = 357.1710.

### *tert*-Butyl (4-(6-chlorofuro[3,2-*b*]pyridin-3-yl)pyridin-2-yl)carbamate (**53**)

The compound was prepared by the general procedure **A** using 100 mg (0.430 mmol) of 3-bromo-6-chlorofuro[3,2-*b*]pyridine (**5**) and 179 mg (0.559 mmol) of *tert*-butyl (4-(4,4,5,5-tetramethyl-1,3,2-dioxaborolan-2-yl)pyridin-2-yl)carbamate; the reaction time was 2 h; flash chromatography (cyclohexane/EtOAc, isocratic 80% of EtOAc) afforded the compound **53** as a white solid (108 mg, 73% yield). ^1^H NMR (500 MHz, Chloroform-*d*) δ 8.65 (d, *J* = 2.0 Hz, 1H), 8.49–8.44 (m, 2H), 8.40 (br d, *J* = 5.2 Hz, 1H), 8.33 (s, 1H), 7.88 (dd, *J* = 5.2, 1.3 Hz, 1H), 7.85 (d, *J* = 2.0 Hz, 1H), 1.57 (s, 9H). ^13^C NMR (126 MHz, Chloroform-*d*) δ 152.93, 152.77, 148.64, 148.50, 147.76, 145.82, 143.89, 140.12, 128.15, 120.06, 119.12, 116.81, 109.35, 81.25, 28.54. FTIR (neat), cm^–1^: 3358, 1687, 1611, 1524, 1386, 1363, 1274, 1249, 1163, 1126, 1076, 1008, 909, 885, 812, 788, 718, 697, 606, 523, 453. HRMS (APCI): calcd. for C_17_H_16_ClN_3_O_3_ [M + H]+ = 346.0953; found [M + H]+ = 346.0958. MP: 104–105 °C.

### *tert*-Butyl 4-(4-(3-(2-((*tert*-butoxycarbonyl)amino)pyridin-4-yl)furo[3,2-*b*]pyridin-6-yl)phenyl)piperazine-1-carboxylate (**54**)

The compound was prepared by the general procedure **B** using 53 mg (0.155 mmol) of *tert*-butyl (4-(6-chlorofuro[3,2-*b*]pyridin-3-yl)pyridin-2-yl)carbamate (**53**) and 78 mg (0.202 mmol) of 4-(4-*tert*-butoxycarbonylpiperazinyl)phenylboronic acid pinacol ester; the reaction time was 2 h; flash chromatography (cyclohexane/EtOAc, gradient from 0% to 60% of EtOAc) afforded the compound **54** as white solid (80 mg, 90% yield). ^1^H NMR (500 MHz, Chloroform-*d*) δ 8.91 (d, *J* = 1.9 Hz, 1H), 8.49 (dd, *J* = 1.4, 0.8 Hz, 1H), 8.38 (dd, *J* = 5.3, 0.8 Hz, 1H), 8.33 (s, 1H), 7.99–7.92 (m, 2H), 7.88 (br s, 1H), 7.60–7.55 (m, 2H), 7.07–7.01 (m, 2H), 3.64–3.60 (m, 4H), 3.26–3.21 (m, 4H), 1.57 (s, 9H), 1.50 (s, 9H). ^13^C NMR (126 MHz, Chloroform-*d*) δ 154.92, 154.90, 152.69, 151.20, 149.58, 148.67, 147.06, 145.54, 143.85, 140.69, 133.46, 129.29, 128.38, 119.99, 119.24, 116.86, 116.28, 109.25, 81.19, 80.19, 51.18, 49.06, 28.60, 28.53. FTIR (neat), cm^–1^: 1687, 1609, 1524, 1424, 1365, 1230, 1154, 1116, 1058, 998, 910, 811, 773, 724, 677, 645, 539, 466. HRMS (APCI): calcd. for C_32_H_37_N_5_O_5_ [M + H]+ = 572.2867; found [M + H]+ = 572.2869. MP: 267–268 °C.

### 4-(6-(4-(Piperazin-1-yl)phenyl)furo[3,2-*b*]pyridin-3-yl)pyridin-2-amine (**55**)

The compound was prepared by the general procedure **C** using 53 mg (0.093 mmol) of *tert*-butyl 4-(4-(3-(2-((*tert*-butoxycarbonyl)amino)pyridin-4-yl)furo[3,2-*b*]pyridin-6-yl)phenyl)piperazine-1-carboxylate (**54**); the reaction time was 1 h; flash chromatography (DCM/7 M NH_3_ in MeOH; 8% of methanolic solution) afforded the compound **55** as a white solid (47 mg, 98% yield). ^1^H NMR (500 MHz, Chloroform-*d*) δ 8.75 (d, *J* = 1.9 Hz, 1H), 8.16 (s, 1H), 7.97–7.93 (m, 1H), 7.91–7.88 (m, 1H), 7.53–7.46 (m, 2H), 7.37 (s, 1H), 7.08–7.04 (m, 1H), 7.00–6.94 (m, 2H), 3.28 (p, *J* = 1.6 Hz, 1H), 3.23–3.17 (m, 4H), 3.05–2.98 (m, 4H). ^13^C NMR (126 MHz, DMSO-*d*_6_) δ 160.24, 150.96, 148.91, 148.20, 148.06, 144.30, 143.07, 138.50, 132.42, 127.79, 127.02, 118.75, 115.74, 115.63, 109.44, 105.11, 47.92, 44.73. FTIR (neat), cm^–1^: 1604, 1515, 1447, 1377, 1294, 1236, 1105, 1014, 923, 878, 811, 677, 541, 464. HRMS (APCI): calcd. for C_22_H_21_N_5_O [M + H]^+^ = 372.1819; found [M + H]^+^ = 372.1818. MP: decomposition

### 1-(4-(6-Chlorofuro[3,2-*b*]pyridin-3-yl)pyridin-2-yl)ethan-1-one (**56**)

The compound was prepared by the general procedure **A** using 300 mg (1.291 mmol) of 3-bromo-6-chlorofuro[3,2-*b*]pyridine (**5**) and 415 mg (1.678 mmol) of 1-(4-(4,4,5,5-tetramethyl-1,3,2-dioxaborolan-2-yl)pyridin-2-yl)ethan-1-one; the reaction time was 5 h. The reaction mixture was hot-filtered through a pad of the mixture Celite 535/SiO_2_ (3:1, 4 g) and the filtrate was concentrated. Flash chromatography (CH_2_Cl_2_/MeOH, gradient from 1% to 2% of MeOH) afforded the product **56** as an off-white solid (27 mg, 8% yield). ^1^H NMR (500 MHz, Chloroform-*d*) δ 8.80 (d, *J* = 4.9 Hz, 1H), 8.67 (d, *J* = 2.1 Hz, 1H), 8.55 (s, 1H), 8.45 (dd, *J* = 5.1, 1.8 Hz, 1H), 8.38 (s, 1H), 7.89 (d, *J* = 2.0 Hz, 1H), 2.79 (s, 3H). ^13^C NMR (126 MHz, Chloroform-*d*) δ 200.16, 154.12, 149.91, 148.74, 147.76, 146.03, 143.61, 139.16, 128.54, 124.88, 119.31, 119.28, 118.90, 26.10. FTIR (neat), cm^–1^: 3099, 3027, 1690, 1608, 1470, 1385, 1351, 1271, 1213, 1143, 1100, 1082, 992, 905, 851, 794, 717, 665, 590, 518, 459. HRMS (APCI): calcd. for C_14_H_9_ClN_2_O_2_ [M + H]^+^ = 273.0425; found [M + H]^+^ = 273.0426. MP: decomposition

### 1-(4-(6-(4-(4-Methylpiperazin-1-yl)phenyl)furo[3,2-*b*]pyridin-3-yl)pyridin-2-yl)ethan-1-one (**57**)

The compound was prepared by the general procedure **B** using 25 mg (0.0917 mmol) of 1-(4-(6-chlorofuro[3,2-*b*]pyridin-3-yl)pyridin-2-yl)ethan-1-one (**56**) and 36 mg (0.119 mmol) of 1-methyl-4-(4-(4,4,5,5-tetramethyl-1,3,2-dioxaborolan-2-yl)phenyl)piperazine; the reaction time was 5 h. The reaction mixture was hot-filtered through a pad of Celite 535/SiO_2_ (3:1, 4 g) and the filtrate was concentrated in vacuo. Flash chromatography (DCM/MeOH, gradient from 0% to 5% of MeOH) afforded the product **57** as an off-white solid (14 mg, 36% yield). ^1^H NMR (500 MHz, DMSO-*d*_6_) δ 9.22 (s, 1H), 9.01 (d, *J* = 1.8 Hz, 1H), 8.89 (dd, *J* = 1.8, 0.8 Hz, 1H), 8.83 (d, *J* = 5.0 Hz, 1H), 8.48 (dd, *J* = 5.0, 1.7 Hz, 1H), 8.37 (d, *J* = 2.0 Hz, 1H), 7.74–7.69 (m, 2H), 7.11–7.05 (m, 2H), 3.25–3.21 (m, 4H), 2.70 (s, 3H), 2.50–2.46 (m, 4H), 2.24 (s, 3H). ^13^C NMR (126 MHz, DMSO-*d*_6_) δ 199.51, 153.71, 150.83, 149.69, 149.62, 149.07, 144.78, 142.57, 139.38, 132.81, 127.78, 126.55, 123.95, 117.96, 117.37, 115.89, 115.51, 54.44, 47.59, 45.72, 25.74. FTIR (neat), cm^–1^: 3097, 2932, 2843, 2798, 1681, 1601, 1523, 1477, 1451, 1378, 1292, 1237, 1197, 1143, 1100, 1010, 922, 849, 825, 793, 675, 587, 546, 524. HRMS (APCI): calcd. For C_25_H_24_N_4_O_2_ [M + H]^+^ = 413.1972; found [M + H]^+^ = 413.1973. MP: decomposition

### 6-Chloro-3-phenylfuro[3,2-*b*]pyridine (**58**)

The compound was prepared by the general procedure **A** using 85 mg (0.365 mmol) of 3-bromo-6-chlorofuro[3,2-*b*]pyridine **(5)** and 53 mg (0.439 mmol) of phenylboronic acid; the reaction time was 2 h; flash chromatography (cyclohexane/EtOAc, gradient from 0% to 10% of EtOAc) afforded the compound **58** as a white solid (60 mg, 71% yield). ^1^H NMR (500 MHz, Chloroform-*d*) δ 8.62 (d, *J* = 2.0 Hz, 1H), 8.10 (s, 1H), 8.07–7.98 (m, 2H), 7.82 (d, *J* = 2.1 Hz, 1H), 7.48 (dd, *J* = 8.4, 7.0 Hz, 2H), 7.44–7.35 (m, 1H). ^13^C NMR (126 MHz, Chloroform-*d*) δ 148.54, 145.60, 145.31, 144.53, 129.97, 129.04, 128.17, 127.71, 127.26, 122.03, 118.83. FTIR (neat), cm^–1^: 3063, 1605, 1487, 1443, 1383, 1267, 1222, 1132, 1089, 1070, 965, 906, 880, 817, 777, 751, 691, 654, 617, 594, 520, 503. HRMS (APCI): calcd. for C_13_H_8_ClNO [M + H]^+^ = 230.0367; found [M + H]^+^ = 230.0370. MP: 106–107 °C

### *tert*-Butyl 4-(4-(3-phenylfuro[3,2-*b*]pyridin-6-yl)phenyl)piperazine-1-carboxylate (**59**)

The compound was prepared by the general procedure **B** using 60 mg (0.2612 mmol) of 6-chloro-3-phenylfuro[3,2-*b*]pyridine (**58**) and 122 mg (0.313 mmol) of 4-(4-*tert*-butoxycarbonylpiperazinyl)phenylboronic acid pinacol ester; the reaction time was 2 h; flash chromatography (cyclohexane/EtOAc, gradient from 0% to 10% of EtOAc) afforded the compound **59** as a yellow solid (120 mg, 100% yield). ^1^H NMR (500 MHz, Chloroform-*d*) δ 8.89 (d, *J* = 1.9 Hz, 1H), 8.12 (s, 1H), 8.09 (dd, *J* = 8.3, 1.2 Hz, 2H), 7.92 (d, *J* = 1.9 Hz, 1H), 7.58 (d, *J* = 8.8 Hz, 2H), 7.49 (dd, *J* = 8.3, 7.1 Hz, 2H), 7.42–7.32 (m, 1H), 7.12–7.01 (m, 2H), 3.69–3.56 (m, 4H), 3.23 (t, *J* = 5.2 Hz, 4H), 1.50 (s, 9H). ^13^C NMR (126 MHz, Chloroform-*d*) δ 154.87, 149.45, 145.08, 144.99, 144.50, 133.02, 130.64, 129.02, 128.37, 127.89, 127.29, 121.96, 116.95, 116.13, 80.16, 49.19, 43.66, 28.60. FTIR (neat), cm^–1^: 2972, 2929, 2864, 2818, 1686, 1605, 1521, 1478, 1420, 1378, 1364, 1340, 1283, 1249, 1229, 1200, 1158, 1122, 1099, 1044, 999, 966, 916, 828, 781, 756, 695, 669, 552, 529. HRMS (APCI): calcd. for C_28_H_29_N_3_O_3_ [M + H]^+^ = 456.2282; found [M + H]^+^ = 456.2285.

### 3-Phenyl-6-(4-(piperazin-1-yl)phenyl)furo[3,2-*b*]pyridine (**60**)

The compound was prepared by the general procedure **C** using 110 mg (0.241 mmol) of *tert*-butyl 4-(4-(3-phenylfuro[3,2-*b*]pyridin-6-yl)phenyl)piperazine-1-carboxylate (**59**); the reaction time was 2 h; flash chromatography (DCM/MeOH, gradient from 0% to 20% of MeOH) afforded the compound as a pale yellow solid (54 mg, 63% yield). ^1^H NMR (500 MHz, DMSO-*d*_6_) δ 8.93 (d, *J* = 1.9 Hz, 1H), 8.82 (s, 1H), 8.29 (d, *J* = 1.9 Hz, 1H), 8.26 (d, *J* = 7.6 Hz, 2H), 7.69 (d, *J* = 8.5 Hz, 2H), 7.50 (t, *J* = 7.6 Hz, 2H), 7.37 (t, *J* = 7.4 Hz, 1H), 7.05 (d, *J* = 8.5 Hz, 2H), 3.13 (t, *J* = 5.0 Hz, 4H), 2.85 (dd, *J* = 6.2, 3.8 Hz, 4H). ^13^C NMR (126 MHz, DMSO-*d*_6_) δ 151.37, 148.85, 146.54, 144.19, 143.30, 132.28, 130.43, 128.63, 127.68, 127.52, 126.70, 126.52, 120.04, 115.50, 115.40, 48.94, 45.49. FTIR (neat), cm^–1^: 2956, 2926, 1729, 1660, 1605, 1524, 1480, 1447, 1380, 1238, 1202, 1122, 1099, 966, 916, 891, 830, 781, 759, 697, 668, 546, 525. HRMS (APCI): calcd. for C_23_H_21_N_3_O [M + H]^+^ = 356.1757; found [M + H]^+^ = 356.1760. MP: 139–140 °C

### 6-Chloro-3-(4-fluorophenyl)furo[3,2-*b*]pyridine (**61**)

The compound was prepared by the general procedure **A** using 100 mg (0.430 mmol) of 3-bromo-6-chlorofuro[3,2-*b*]pyridine (**5**) and 78 mg (0.559 mmol) of (4-fluorophenyl)boronic acid; the reaction time was 2 h; flash chromatography (cyclohexane) afforded the compound **61** as a pale yellow solid (70 mg, 66% yield). ^1^H NMR (500 MHz, Chloroform-*d*) δ 8.61 (d, *J* = 2.0 Hz, 1H), 8.08 (s, 1H), 8.02 (dd, *J* = 8.8, 5.3 Hz, 2H), 7.83 (d, *J* = 2.0 Hz, 1H), 7.17 (t, *J* = 8.7 Hz, 2H). ^13^C NMR (126 MHz, Chloroform-*d*) δ 162.74 (d, *J* = 247.5 Hz), 148.53, 145.37, 145.30 (d, *J* = 1.4 Hz), 144.35, 128.99 (d, *J* = 8.0 Hz), 127.88, 126.08 (d, *J* = 3.4 Hz), 121.16, 118.94, 116.06 (d, *J* = 21.6 Hz). ^19^F NMR (471 MHz, Chloroform-*d*) δ −113.46. FTIR (neat), cm^–1^: 3151, 3075, 1568, 1504, 1461, 1386, 1267, 1217, 1163, 1134, 1087, 1071, 967, 912, 871, 833, 798, 783, 713, 611, 585, 521, 506. HRMS (APCI): calcd, for C_13_H_7_ClFNO [M + H]^+^ = 248.0273; found [M + H]^+^ = 248.0275. MP: 207–208 °C

### *tert*-Butyl 4-(4-(3-(4-fluorophenyl)furo[3,2-*b*]pyridin-6-yl)phenyl)piperazine-1-carboxylate (**62**)

The compound was prepared by the general procedure **B** using 85 mg (0.343 mmol) of 6-chloro-3-(4-fluorophenyl)furo[3,2-*b*]pyridine (**61**) and 173 mg (0.446 mmol) of 4-(4-*tert*-butoxycarbonylpiperazinyl)phenylboronic acid pinacol ester; the reaction time was 2 h; flash chromatography (cyclohexane/EtOAc, gradient from 0% to 30% of EtOAc) afforded the compound **62** as a white solid (130 mg, 80% yield). ^1^H NMR (500 MHz, Chloroform-*d*) δ 8.87 (d, *J* = 1.9 Hz, 1H), 8.16–8.01 (m, 3H), 7.93 (d, *J* = 1.9 Hz, 1H), 7.58 (d, *J* = 8.8 Hz, 2H), 7.18 (t, *J* = 8.7 Hz, 2H), 7.06 (d, *J* = 8.3 Hz, 2H), 3.66–3.55 (m, 4H), 3.23 (t, *J* = 5.2 Hz, 4H), 1.50 (s, 9H). ^13^C NMR (126 MHz, Chloroform-*d*) δ 162,60 (d, *J* = 247.2 Hz), 154.88, 151.07, 149.42, 145.12, 144.65, 144.31, 133.17, 128.96 (d, *J* = 7.9 Hz), 128.39, 126.74 (d, *J* = 3.4 Hz), 121.08, 116.95, 116.19, 115.99 (d, *J* = 21.6 Hz), 80.19, 49.18, 43.53, 28.61. ^19^F NMR (471 MHz, Chloroform-*d*) δ −114.02. FTIR (neat), cm^–1^: 2975, 2930, 2836, 1686, 1609, 1524, 1504, 1480, 1414, 1380, 1364, 1239, 1203, 1159, 1124, 1104, 1047, 912, 844, 823, 810, 773, 585, 548, 529. HRMS (APCI): calcd, for C_28_H_28_FN_3_O_3_ [M + H]^+^ = 474.2187; found [M + H]^+^ = 474.2187. MP: 122–123 °C

### 3-(4-Fluorophenyl)-6-(4-(piperazin-1-yl)phenyl)furo[3,2-*b*]pyridine (**63**)

The compound was prepared by the general procedure **C** using 85 mg (0.180 mmol) of *tert*-butyl 4-(4-(3-(4-fluorophenyl)furo[3,2-*b*]pyridin-6-yl)phenyl)piperazine-1-carboxylate (**62**); the reaction time was 2 h; flash chromatography (DCM/MeOH, gradient from 0% to 20% of MeOH) afforded the compound as a pale yellow solid (60 mg, 90% yield). ^1^H NMR (500 MHz, DMSO-*d*_6_) δ 8.94 (d, *J* = 1.9 Hz, 1H), 8.83 (s, 1H), 8.36–8.23 (m, 3H), 7.79–7.66 (m, 2H), 7.35 (t, *J* = 8.9 Hz, 2H), 7.21–7.05 (m, 2H), 3.32 (dd, *J* = 6.5, 3.8 Hz, 4H), 3.07 (dd, *J* = 6.6, 3.6 Hz, 4H). ^13^C NMR (126 MHz, DMSO-*d*_6_) δ 162.49, 160.54, 150.48, 148.77, 146.49, 144.26, 143.24, 132.19, 128.52, 128.45, 127.80, 127.45, 126.90, 126.88, 119.09, 115.85, 115.70, 115.65, 115.48, 46.86, 43.86. FTIR (neat), cm^–1^: 2931, 2835, 2700, 2612, 2496, 2475, 1605, 1573, 1524, 1504, 1481, 1455, 1378, 1340, 1245, 1217, 1201, 1162, 1123, 1095, 968, 916, 890, 846, 831, 801, 652, 584, 544, 525. HRMS (APCI): calcd, for C_23_H_20_FN_3_O [M + H]^+^ = 374.1663; found [M + H]^+^ = 374.1663. MP: > 250 °C

### 6-Chloro-3-(3-methoxyphenyl)furo[3,2-*b*]pyridine (**64**)

The compound was prepared by the general procedure **A** using 100 mg (0.430 mmol) of 3-bromo-6-chlorofuro[3,2-*b*]pyridine (**5**) and 85 mg (0.559 mmol) of (3-methoxyphenyl)boronic acid; the reaction time was 2 h; flash chromatography (cyclohexane/EtOAc, gradient from 0% to 8% of EtOAc) afforded the compound **64** as a white solid (68 mg, 61% yield). ^1^H NMR (500 MHz, Chloroform-*d*) δ 8.62 (d, *J* = 2.0 Hz, 1H), 8.10 (s, 1H), 7.81 (d, *J* = 2.0 Hz, 1H), 7.65 (dd, *J* = 2.6, 1.6 Hz, 1H), 7.58 (ddd, *J* = 7.6, 1.6, 0.9 Hz, 1H), 7.39 (t, *J* = 8.0 Hz, 1H), 6.93 (ddd, *J* = 8.3, 2.6, 0.9 Hz, 1H), 3.89 (s, 3H). ^13^C NMR (126 MHz, Chloroform-*d*) δ 160.14, 148.52, 145.77, 145.33, 144.48, 131.24, 130.03, 127.71, 121.87, 119.61, 118.80, 113.69, 113.07, 55.47. FTIR (neat), cm^–1^: 3108, 3068, 2964, 2942, 2838, 1611, 1584, 1564, 1480, 1451, 1438, 1387, 1343, 1304, 1287, 1274, 1234, 1209, 1183, 1170, 1128, 1096, 1084, 1071, 1051, 999, 987, 911, 887, 875, 859, 822, 780, 685, 654, 599, 569, 525, 457. HRMS (APCI): calcd. for C_14_H_10_ClNO_2_ [M + H]^+^ = 260.0473; found [M + H]^+^ = 260.0476. MP: 72–73 °C

### *tert*-Butyl 4-(4-(3-(3-methoxyphenyl)furo[3,2-*b*]pyridin-6-yl)phenyl)piperazine-1-carboxylate (**65**)

The compound was prepared by the general procedure **B** using 65 mg (0.250 mmol) of 6-chloro-3-(3-methoxyphenyl)furo[3,2-*b*]pyridine (**64**) and 126 mg (0.325 mmol) of 4-(4-*tert*-butoxycarbonylpiperazinyl)phenylboronic acid pinacol ester; the reaction time was 2 h; flash chromatography (cyclohexane/EtOAc, gradient from 0% to 30% of EtOAc) afforded the compound **65** as a yellow solid (88 mg, 72% yield). ^1^H NMR (500 MHz, Chloroform-*d*) δ 8.88 (d, *J* = 1.9 Hz, 1H), 8.11 (s, 1H), 7.92 (d, *J* = 2.0 Hz, 1H), 7.70 (dd, *J* = 2.6, 1.5 Hz, 1H), 7.66 (dt, *J* = 7.8, 1.2 Hz, 1H), 7.63–7.55 (m, 2H), 7.40 (t, *J* = 7.9 Hz, 1H), 7.05 (d, *J* = 8.6 Hz, 2H), 6.92 (ddd, *J* = 8.3, 2.6, 1.0 Hz, 1H), 3.90 (s, 3H), 3.67–3.57 (m, 4H), 3.23 (t, *J* = 5.1 Hz, 4H), 1.50 (s, 9H). ^13^C NMR (126 MHz, Chloroform-*d*) δ 160.15, 154.88, 149.44, 145.17, 145.11, 144.48, 133.02, 131.91, 130.02, 128.38, 121.85, 119.74, 116.96, 116.11, 113.53, 113.01, 80.18, 55.50, 49.21, 43.56, 28.60. FTIR (neat), cm^–1^: 2974, 2815, 1682, 1604, 1590, 1524, 1480, 1461, 1449, 1412, 1378, 1366, 1336, 1290, 1261, 1251, 1224, 1160, 1134, 1117, 1101, 1042, 996, 908, 887, 860, 826, 813, 791, 773, 693, 544. HRMS (APCI): calcd. for C_29_H_31_N_3_O_4_ [M + H]^+^ = 486.2387; found [M + H]^+^ = 486.2391. MP: 190–191 °C

### 3-(3-Methoxyphenyl)-6-(4-(piperazin-1-yl)phenyl)furo[3,2-*b*]pyridine (**66**)

The compound was prepared by the general procedure **C** using 80 mg (0.165 mmol) of *tert*-butyl 4-(4-(3-(3-methoxyphenyl)furo[3,2-*b*]pyridin-6-yl)phenyl)piperazine-1-carboxylate (**65**); the reaction time was 2 h; flash chromatography (DCM/MeOH, gradient from 0% to 15% of MeOH) afforded the compound as a white solid (43 mg, 68% yield). ^1^H NMR (500 MHz, DMSO-*d*_6_) δ 8.93 (d, *J* = 2.0 Hz, 1H), 8.85 (s, 1H), 8.29 (d, *J* = 1.9 Hz, 1H), 7.88 (dd, *J* = 2.6, 1.5 Hz, 1H), 7.85 (dt, *J* = 7.7, 1.2 Hz, 1H), 7.70 (d, *J* = 8.9 Hz, 2H), 7.41 (t, *J* = 7.9 Hz, 1H), 7.15–7.03 (m, 2H), 6.95 (ddd, *J* = 8.3, 2.6, 1.0 Hz, 1H), 3.84 (s, 3H), 3.21–3.13 (m, 4H), 2.93–2.86 (m, 4H). ^13^C NMR (126 MHz, DMSO-*d*_6_) δ 159.48, 151.17, 148.85, 146.85, 144.23, 143.30, 132.24, 131.67, 129.68, 127.70, 126.86, 119.88, 118.88, 115.52, 115.49, 112.94, 112.26, 55.12, 48.51, 45.18. FTIR (neat), cm^–1^: 2829, 1603, 1590, 1523, 1481, 1450, 1377, 1261, 1242, 1227, 1117, 1100, 1038, 910, 887, 859, 827, 814, 793, 693, 543, 517. HRMS (APCI): calcd. for C_24_H_23_N_3_O_2_ [M + H]^+^ = 386.1863; found [M + H]^+^ = 386.1864. MP: 165–166 °C

### 3-(6-Chlorofuro[3,2-*b*]pyridin-3-yl)-*N*-methylbenzamide (**67**)

The compound was prepared by the general procedure **A** using 100 mg (0.430 mmol) of 3-bromo-6-chlorofuro[3,2-*b*]pyridine (**5**) and 100 mg (0.559 mmol) of (3-(methylcarbamoyl)phenyl)boronic acid; the reaction time was 2 h; flash chromatography (cyclohexane/EtOAc, gradient from 0% to 79% of EtOAc) afforded the compound **67** as a pale yellow solid (96 mg, 78% yield). ^1^H NMR (500 MHz, Chloroform-*d*) δ 8.62 (d, *J* = 2.0 Hz, 1H), 8.43 (t, *J* = 1.8 Hz, 1H), 8.19 (d, *J* = 8.2 Hz, 2H), 7.84 (d, *J* = 2.0 Hz, 1H), 7.76 (dt, *J* = 7.8, 1.5 Hz, 1H), 7.54 (t, *J* = 7.7 Hz, 1H), 6.35 (s, 1H), 3.06 (d, *J* = 4.8 Hz, 3H). ^13^C NMR (126 MHz, Chloroform-*d*) δ 168.15, 148.61, 146.15, 145.37, 144.16, 135.51, 130.47, 129.97, 129.34, 127.99, 126.56, 125.59, 121.15, 119.12, 27.06. FTIR (neat), cm^–1^: 3314, 3102, 1632, 1607, 1586, 1536, 1389, 1357, 1322, 1311, 1293, 1276, 1135, 1095, 1070, 985, 916, 876, 842, 810, 779, 689, 597, 524, 474, 414. HRMS (APCI): calcd. for C_15_H_11_ClN_2_O_2_ [M + H]^+^ = 287.0582; found [M + H]^+^ = 287.0580. MP: 197–198 °C

### *tert*-Butyl 4-(4-(3-(3-(methylcarbamoyl)phenyl)furo[3,2-*b*]pyridin-6-yl)phenyl)piperazine-1-carboxylate (**68**)

The compound was prepared by the general procedure **B** using 85 mg (0.296 mmol) of 3-(6-chlorofuro[3,2-*b*]pyridin-3-yl)-*N*-methylbenzamide (**67**) and 150 mg (0.385 mmol) of 4-(4-*tert*-butoxycarbonylpiperazinyl)phenylboronic acid pinacol ester; the reaction time was 2 h; flash chromatography (cyclohexane/EtOAc, gradient from 0% to 20% of EtOAc) afforded the compound **68** as a pale yellow solid (121 mg, 80% yield). ^1^H NMR (500 MHz, Chloroform-*d*) δ 8.86 (d, *J* = 2.0 Hz, 1H), 8.47 (t, *J* = 1.8 Hz, 1H), 8.22 (dt, *J* = 7.8, 1.4 Hz, 1H), 8.16 (s, 1H), 7.92 (d, *J* = 1.8 Hz, 1H), 7.77 (dt, *J* = 7.8, 1.5 Hz, 1H), 7.62–7.47 (m, 3H), 7.11–6.97 (m, 2H), 6.55 (s, 1H), 3.67–3.53 (m, 4H), 3.22 (dd, *J* = 6.3, 4.0 Hz, 4H), 3.05 (d, *J* = 4.8 Hz, 3H), 1.50 (s, 9H). ^13^C NMR (126 MHz, Chloroform-*d*) δ 168.29, 154.87, 151.17, 149.49, 145.45, 145.03, 144.05, 135.46, 133.28, 131.05, 129.94, 129.27, 129.25, 128.33, 126.43, 125.48, 121.06, 116.83, 116.28, 80.17, 49.04, 43.62, 28.59, 27.01. FTIR (neat), cm^–1^: 3260, 2971, 2937, 2894, 2837, 1704, 1627, 1603, 1524, 1481, 1409, 1366, 1254, 1231, 1205, 1158, 1116, 1081, 929, 893, 803, 779, 694, 654, 548, 523. HRMS (APCI): calcd. for C_30_H_32_N_4_O_4_ [M + H]^+^ = 513.2496; found [M + H]^+^ = 513.2499. MP: 210–211 °C

### *N*-Methyl-3-(6-(4-(piperazin-1-yl)phenyl)furo[3,2-*b*]pyridin-3-yl)benzamide (**69**)

The compound was prepared by the general procedure **C** using 85 mg (0.166 mmol) of *tert*-butyl 4-(4-(3-(3-(methylcarbamoyl)phenyl)furo[3,2-*b*]pyridin-6-yl)phenyl)piperazine-1-carboxylate (**68**); the reaction time was 2 h; flash chromatography (DCM/MeOH, gradient from 0% to 20% of MeOH) afforded the compound as a pale yellow solid (60 mg, 88% yield). ^1^H NMR (500 MHz, DMSO-*d*_6_) δ 8.96 (d, *J* = 2.0 Hz, 1H), 8.87 (s, 1H), 8.64 (t, *J* = 1.8 Hz, 1H), 8.49 (q, *J* = 4.5 Hz, 1H), 8.42 (dt, *J* = 7.8, 1.4 Hz, 1H), 8.32 (d, *J* = 1.9 Hz, 1H), 7.80 (dt, *J* = 7.7, 1.5 Hz, 1H), 7.76–7.68 (m, 2H), 7.59 (t, *J* = 7.7 Hz, 1H), 7.14–6.94 (m, 2H), 3.26–3.21 (m, 4H), 3.01–2.93 (m, 4H), 2.83 (d, *J* = 4.5 Hz, 3H). ^13^C NMR (126 MHz, DMSO-*d*_6_) δ 166.69, 150.91, 148.85, 146.94, 144.33, 143.20, 135.26, 132.32, 130.49, 128.98, 128.59, 127.75, 127.08, 125.97, 125.39, 119.74, 115.64, 47.84, 44.64, 26.23. FTIR (neat), cm^–1^: 3352, 2969, 2940, 2829, 2795, 2726, 2493, 1668, 1604, 1587, 1522, 1481, 1449, 1374, 1258, 1244, 1201, 1139, 1117, 1103, 1084, 1045, 921, 905, 889, 831, 804, 789, 778, 750, 728, 689, 623, 541, 520, 489. HRMS (APCI): calcd. for C_25_H_24_N_4_O_2_ [M + H]^+^ = 413.1972; found [M + H]^+^ = 413.1971. MP: > 250 °C

### 6-(4-(4-Methylpiperazin-1-yl)phenyl)-3-phenylfuro[3,2-*b*]pyridine (**70**)

The compound was prepared by the general procedure **B** using 100 mg (0.435 mmol) of 6-chloro-3-phenylfuro[3,2-*b*]pyridine (**58**) and 158 mg (0.523 mmol) of 1-methyl-4-(4-(4,4,5,5-tetramethyl-1,3,2-dioxaborolan-2-yl)phenyl)piperazine; the reaction time was 2 h; flash chromatography (DCM/MeOH, gradient from 0% to 10% of MeOH) afforded the compound **70** as a white solid (153 mg, 95% yield). ^1^H NMR (500 MHz, Chloroform-*d*) δ 8.89 (d, *J* = 1.9 Hz, 1H), 8.11 (s, 1H), 8.11–8.06 (m, 2H), 7.92 (d, *J* = 1.8 Hz, 1H), 7.60–7.53 (m, 2H), 7.49 (dd, *J* = 8.4, 7.1 Hz, 2H), 7.38 (d, *J* = 7.4 Hz, 1H), 7.09–7.02 (m, 2H), 3.34 (t, *J* = 5.0 Hz, 4H), 2.66 (bs, 4H), 2.41 (s, 3H). ^13^C NMR (126 MHz, Chloroform-*d*) δ 151.11, 149.47, 145.12, 144.89, 144.42, 133.16, 130.70, 129.01, 128.29, 127.86, 127.28, 121.96, 116.39, 116.03, 55.22, 55.13, 53.54, 49.13, 48.76, 46.22. FTIR (neat), cm^–1^: 2940, 2843, 2800, 1604, 1525, 1478, 1445, 1381, 1293, 1245, 1201, 1159, 1140, 1122, 1098, 967, 918, 820, 789, 779, 752, 691, 671, 530, 504. HRMS (APCI): calcd, for C_24_H_23_N_3_O [M + H]^+^ = 370.1914; found [M + H]^+^ = 370.1911. MP: 184–185 °C

### 3-(6-Chlorofuro[3,2-*b*]pyridin-3-yl)benzaldehyde (**71**)

The compound was prepared by the general procedure **A** using 100 mg (0.430 mmol) of 3-bromo-6-chlorofuro[3,2-*b*]pyridine (**5**) and 84 mg (0.559 mmol) of 3-formylphenylboronic acid; the reaction time was 2 h; flash chromatography (cyclohexane/EtOAc, gradient from 0% to 10% of EtOAc) afforded the compound **71** as a white solid (84 mg, 76% yield). ^1^H NMR (500 MHz, Chloroform-*d*) δ 10.12 (s, 1H), 8.65 (d, *J* = 2.0 Hz, 1H), 8.54 (t, *J* = 1.7 Hz, 1H), 8.38 (ddd, *J* = 7.7, 1.8, 1.2 Hz, 1H), 8.22 (s, 1H), 7.90 (dt, *J* = 7.6, 1.4 Hz, 1H), 7.87 (d, *J* = 2.0 Hz, 1H), 7.67 (t, *J* = 7.7 Hz, 1H). ^13^C NMR (126 MHz, Chloroform-*d*) δ 192.26, 148.56, 146.07, 145.56, 144.08, 137.09, 132.98, 131.18, 129.75, 129.14, 128.25, 128.08, 120.79, 119.03. FTIR (neat), cm^–1^: 3138, 3106, 3068, 2858, 1686, 1602, 1565, 1484, 1461, 1386, 1359, 1270, 1239, 1176, 1126, 1097, 1077, 999, 915, 890, 877, 827, 791, 780, 719, 681, 651, 597, 522, 427, 411. HRMS (APCI): calcd, for C_14_H_8_ClNO_2_ [M + H]^+^ = 258.0316; found [M + H]^+^ = 258.0314. MP: 127–128 °C

### 3-(6-(4-(4-Methylpiperazin-1-yl)phenyl)furo[3,2-*b*]pyridin-3-yl)benzaldehyde (**72**)

The compound was prepared by the general procedure **B** using 80 mg (0.310 mmol) of 3-(6-chlorofuro[3,2-*b*]pyridin-3-yl)benzaldehyde (**71**) and 122 mg (0.404 mmol) of 1-methyl-4-(4-(4,4,5,5-tetramethyl-1,3,2-dioxaborolan-2-yl)phenyl)piperazine; the reaction time was 2 h; flash chromatography (DCM/MeOH, gradient from 0% to 10% of MeOH) afforded the compound **75** as a white solid (109 mg, 88% yield). ^1^H NMR (500 MHz, DMSO-*d*_6_) δ 10.11 (s, 1H), 9.02–8.91 (m, 2H), 8.84 (t, *J* = 1.7 Hz, 1H), 8.57 (dt, *J* = 7.7, 1.5 Hz, 1H), 8.33 (d, *J* = 1.9 Hz, 1H), 7.92 (dt, *J* = 7.6, 1.4 Hz, 1H), 7.75 (t, *J* = 7.6 Hz, 1H), 7.73–7.68 (m, 2H), 7.07 (d, *J* = 8.9 Hz, 2H), 3.25–3.21 (m, 4H), 2.49–2.43 (m, 4H), 2.24 (s, 3H). ^13^C NMR (126 MHz, DMSO-*d*_6_) δ 193.00, 150.76, 148.90, 147.36, 144.42, 143.01, 136.66, 135.66, 132.49, 132.20, 131.47, 129.56, 128.90, 127.73, 126.93, 126.75, 119.00, 115.68, 115.49, 113.69, 82.97, 54.44, 47.61, 45.72, 39.52, 24.63. HRMS (APCI): calcd, for C_25_H_23_N_3_O_2_ [M + H]^+^ = 398.1863; found [M + H]^+^ = 398.1863. 1203, 1175, 1140, 1118, 1107, 1008, 921, 887, 821, 799, 790, 688, 671, 655, 538, 521. FTIR (neat), cm^–1^ 2973, 2940, 2827, 2797, 1699, 1604, 1586, 1524, 1480, 1450, 1376, 1362, 1292, 1238. MP: 163–164 °C

### (*E*)-3-(6-(4-(4-Methylpiperazin-1-yl)phenyl)furo[3,2-*b*]pyridin-3-yl)benzaldehyde oxime (**73**)

Pyridine (40 μL, 0.503 mmol) and hydroxylamine hydrochloride (26 mg, 0.377 mmol) were added to a solution of 3-(6-(4-(4-methylpiperazin-1-yl)phenyl)furo[3,2-*b*]pyridin-3-yl)benzaldehyde (**72**; 100 mg, 0.252 mmol) in EtOH (5 mL) at 0 °C and the reaction mixture was stirred at 25 °C for 18 h. The solvent was evaporated in vacuo and the residue was diluted with EtOAc (10 mL) and extracted with H_2_O (3 × 10 mL). The organic layer was dried over MgSO_4_ and filtered. The solvent was evaporated in vacuo and the residue was purified by flash chromatography (DCM/MeOH, gradient from 0% to 20% of MeOH). The product was obtained as a white solid (83 mg, 80% yield). ^1^H NMR (500 MHz, DMSO-*d*_6_) δ 11.32 (s, 1H), 10.36 (s, 1H), 8.97 (d, *J* = 1.9 Hz, 1H), 8.87 (s, 1H), 8.56 (t, *J* = 1.8 Hz, 1H), 8.35 (d, *J* = 1.9 Hz, 1H), 8.29–8.15 (m, 2H), 7.82–7.71 (m, 2H), 7.62–7.49 (m, 2H), 7.23–7.07 (m, 2H), 3.29 (s, 4H), 2.82 (s, 3H). ^13^C NMR (126 MHz, DMSO-*d*_6_) δ 149.29, 148.81, 147.92, 147.04, 144.37, 143.41, 133.51, 132.05, 130.83, 129.05, 128.11, 127.93, 127.30, 125.95, 124.17, 119.67, 116.17, 115.82, 51.98, 45.18, 42.07. FTIR (neat), cm^–1^: 3401, 3186, 2966, 2688, 2593, 1605, 1523, 1476, 1457, 1378, 1245, 1185, 1107, 1017, 987, 971, 949, 920, 826, 801, 696, 682, 639, 545, 521. HRMS (APCI): calcd, for C_25_H_24_N_4_O_2_ [M + H]^+^ = 413.1972; found [M + H]^+^ = 413.1973. MP: > 250 °C

### 6-Chloro-3-(3-(methylsulfonyl)phenyl)furo[3,2-*b*]pyridine (**74**)

The compound was prepared by the general procedure **A** using 100 mg (0.430 mmol) of 3-bromo-6-chlorofuro[3,2-*b*]pyridine (**5**) and 103 mg (0.515 mmol) of (3-(methylsulfonyl)phenyl)boronic acid; the reaction time was 2 h; flash chromatography (cyclohexane/EtOAc, gradient from 0% to 25% of EtOAc) afforded the compound **74** as a white solid (96 mg, 73% yield). ^1^H NMR (500 MHz, Chloroform-*d*) δ 8.64 (d, *J* = 2.0 Hz, 1H), 8.58 (t, *J* = 1.7 Hz, 1H), 8.46 (dt, J = 7.8, 1.4 Hz, 1H), 8.24 (s, 1H), 7.94 (ddd, J = 7.8, 1.8, 1.1 Hz, 1H), 7.87 (d, *J* = 2.0 Hz, 1H), 7.70 (t, *J* = 7.8 Hz, 1H), 3.13 (s, 3H). ^13^C NMR (126 MHz, Chloroform-*d*) δ 148.59, 146.38, 145.76, 143.83, 141.49, 132.29, 131.78, 130.14, 128.29, 126.63, 125.74, 120.30, 119.15, 44.68. FTIR (neat), cm^–1^: 1608, 1467, 1385, 1284, 1128, 1095, 993, 886, 837, 783, 748, 680, 650, 598, 560, 537, 490. HRMS (APCI): calcd. for C_14_H_10_ClNO_3_S [M + H]^+^ = 308.0143; found [M + H]^+^ = 308.0147. MP: 207–208 °C.

### 6-(4-(4-Methylpiperazin-1-yl)phenyl)-3-(3-(methylsulfonyl)phenyl)furo[3,2-*b*]pyridine (**75**)

The compound was prepared by the general procedure **B** using 59 mg (0.192 mmol) of 6-chloro-3-(3-(methylsulfonyl)phenyl)furo[3,2-*b*]pyridine (**74**) and 79 mg (0.261 mmol) of 1-methyl-4-(4-(4,4,5,5-tetramethyl-1,3,2-dioxaborolan-2-yl)phenyl)piperazine; the reaction time was 2 h; flash chromatography (DCM/MeOH, gradient from 0% to 7% of MeOH) afforded the compound **78** as a white solid (54 mg, 63% yield). ^1^H NMR (500 MHz, Chloroform-*d*) δ 8.89 (d, *J* = 1.9 Hz, 1H), 8.62 (t, *J* = 1.8 Hz, 1H), 8.54 (dt, *J* = 7.8, 1.4 Hz, 1H), 8.23 (s, 1H), 7.97–7.90 (m, 2H), 7.70 (t, *J* = 7.8 Hz, 1H), 7.60–7.55 (m, 2H), 7.08–7.02 (m, 2H), 3.36 (t, *J* = 4.9 Hz, 4H), 3.14 (s, 3H), 2.69 (t, *J* = 5.0 Hz, 4H), 2.43 (s, 3H). ^13^C NMR (126 MHz, Chloroform-*d*) δ 151.21, 149.53, 145.61, 145.47, 143.66, 141.34, 133.69, 132.50, 132.36, 130.09, 128.65, 128.31, 126.28, 125.67, 120.20, 116.37, 116.22, 55.09, 48.68, 46.21, 44.70. FTIR (neat), cm^–1^: 3936, 2842, 2794, 2165, 1982, 1604, 1524, 1478, 1380, 1289, 1243, 1202, 1145, 1094, 1039, 992, 957, 919, 883, 820, 781, 686, 661, 618, 533, 486. HRMS (APCI): calcd. for C_25_H_25_N_3_O_3_S [M + H]^+^ = 448.1689; found [M + H]^+^ = 448.1692. MP: 216–217 °C.

### 1-(4-(6-Chlorofuro[3,2-*b*]pyridin-3-yl)phenyl)urea (**76**)

The compound was prepared by the general procedure **A** using 100 mg (0.430 mmol) of 3-bromo-6-chlorofuro[3,2-*b*]pyridine (**5**) and 147 mg (0.559 mmol) of [4-(4,4,5,5-tetramethyl-1,3,2-dioxaborolan-2-yl)phenyl]urea; the reaction time was 2 h; flash chromatography (cyclohexane/EtOAc, gradient from 0% to 100% of EtOAc) afforded the compound **76** as a pale yellow solid (70 mg, 57% yield). ^1^H NMR (500 MHz, DMSO-*d*_6_) δ 8.77 (s, 1H), 8.67 (d, *J* = 2.1 Hz, 1H), 8.64 (s, 1H), 8.38 (d, *J* = 2.1 Hz, 1H), 8.04 (d, *J* = 8.7 Hz, 2H), 7.51 (d, *J* = 8.7 Hz, 2H), 5.86 (s, 2H). ^13^C NMR (126 MHz, DMSO-*d*_6_) δ 155.81, 147.85, 146.61, 144.51, 144.03, 140.30, 126.98, 126.52, 122.35, 120.06, 119.21, 117.73. FTIR (neat), cm^–1^: 3433, 3305, 1665, 1592, 1543, 1384, 1340, 1268, 1132, 1096, 1072, 967, 914, 872, 837, 805, 783, 595, 527. HRMS (APCI): calcd. for C_14_H_10_ClN_3_O_2_ [M + H]^+^ = 288.0534; found [M + H]^+^ = 288.0535. MP: > 250 °C

### (4-{6-[4-(4-Methylpiperazin-1-yl)phenyl]furo[3,2-*b*]pyridin-3-yl}phenyl)urea (**77**)

The compound was prepared by the general procedure **B** using 58 mg (0.202 mmol) of (4-{6-[4-(4-methylpiperazin-1-yl)phenyl]furo[3,2-*b*]pyridin-3-yl}phenyl)urea (**76**) and 73 mg (0.242 mmol) of 1-methyl-4-(4-(4,4,5,5-tetramethyl-1,3,2-dioxaborolan-2-yl)phenyl)piperazine; the reaction time was 3 h; flash chromatography (DCM/MeOH, gradient from 0% to 20% of MeOH) afforded the compound **77** as a pale yellow solid (63 mg, 73% yield). ^1^H NMR (500 MHz, DMSO-*d*_6_) δ 8.91 (d, *J* = 2.0 Hz, 1H), 8.69 (s, 1H), 8.63 (s, 1H), 8.26 (d, *J* = 2.0 Hz, 1H), 8.11 (d, *J* = 8.7 Hz, 2H), 7.69 (d, *J* = 8.9 Hz, 2H), 7.52 (d, *J* = 8.7 Hz, 2H), 7.07 (d, *J* = 8.9 Hz, 2H), 5.86 (s, 2H), 3.25–3.21 (m, 4H), 2.49–2.48 (m, 4H), 2.25 (s, 3H). ^13^C NMR (126 MHz, DMSO-*d*_6_) δ 155.85, 150.67, 148.73, 145.39, 144.02, 143.52, 140.04, 132.06, 127.69, 126.99, 126.90, 123.10, 120.02, 117.73, 115.52, 115.40, 54.42, 47.61, 45.66. FTIR (neat), cm^–1^: 3376, 3313, 3201, 2940, 2818, 1678, 1591, 1538, 1450, 1417, 1377, 1293, 1241, 1200, 1124, 1090, 1001, 968, 915, 837, 817, 802, 764, 536, 444. HRMS (APCI): calcd. for C_25_H_25_N_5_O_2_ [M + H]^+^ = 428.2081; found [M + H]^+^ = 428.2086. MP: > 250 °C

### Methyl 4-(6-chlorofuro[3,2-*b*]pyridin-3-yl)benzoate (**78**)

The compound was prepared by the general procedure **A** using 200 mg (0.860 mmol) of 3-bromo-6-chlorofuro[3,2-*b*]pyridine (**5**) and 201 mg (1.119 mmol) of 4-(methoxycarbonyl)phenylboronic acid. The reaction time was 3 h. The reaction mixture was hot-filtered through a pad of Celite 535 (4 g) and the filtrate was concentrated in vacuo. Flash chromatography (cyclohexane/EtOAc, gradient from 0% to 5% of EtOAc) afforded the compound **78** as a white solid (194 mg, 78% yield). ^1^H NMR (500 MHz, Chloroform-*d*) δ 8.64 (d, *J* = 2.0 Hz, 1H), 8.20 (s, 1H), 8.15 (s, 4H), 7.85 (d, *J* = 2.0 Hz, 1H), 3.95 (s, 3H). ^13^C NMR (126 MHz, Chloroform-*d*) δ 166.98, 148.67, 146.51, 145.64, 144.14, 134.66, 130.37, 129.64, 128.09, 126.96, 121.18, 119.05, 52.35. FTIR (neat), cm^–1^: 3107, 3065, 2953, 1715, 1610, 1438, 1385, 1270, 1181, 1077, 964, 909, 886, 846, 779, 761, 697, 598, 529, 503. HRMS (APCI): calcd. For C_15_H_10_ClNO_3_ [M + H]^+^ = 288.0422; found [M + H]^+^ = 288.0420. MP: 170–171 °C

### (4-(6-Chlorofuro[3,2-*b*]pyridin-3-yl)phenyl)methanol (**79**)

0.67 mL of 2 M LiBH_4_ in THF (1.390 mmol) was added to the mixture of 160 mg (0.521 mmol) of methyl 4-(6-chlorofuro[3,2-*b*]pyridin-3-yl)benzoate (**78**) in 15.0 mL of anhydrous THF at 0 °C. The mixture was stirred at 0 °C for 30 min. Ice bath was removed and the mixture was stirred for additional 23.5 h at 22 °C. The reaction mixture was diluted with 2.0 mL (111.0 mmol) of water and 15 mL of EtOAc, stirred 1 h, and concentrated in vacuo. Flash chromatography (cyclohexane/EtOAc, gradient from 5% to 30% of EtOAc) provided the compound **79** as a white solid (69 mg, 48% yield). ^1^H NMR (500 MHz, Chloroform-*d*) δ 8.62 (d, *J* = 2.0 Hz, 1H), 8.12 (s, 1H), 8.05–8.02 (m, 2H), 7.83 (d, *J* = 2.0 Hz, 1H), 7.51–7.47 (m, 2H), 4.76 (d, *J* = 6.0 Hz, 2H), 1.65 (t, *J* = 6.0 Hz, 1H). ^13^C NMR (126 MHz, Chloroform-*d*) δ 148.56, 145.61, 145.36, 144.52, 140.87, 129.37, 127.78, 127.64, 127.44, 121.74, 118.88, 65.31. FTIR (neat), cm^–1^: 2854, 2805, 1703, 1613, 1528, 1427, 1384, 1284, 1249, 1208, 1131, 1095, 1043, 967, 916, 874, 807, 778, 747, 682, 595, 525, 503. HRMS (APCI): calcd. for C_14_H_10_ClNO_2_ [M + H]^+^ = 260.0473; found [M + H]^+^ = 260.0474. MP: 128–129 °C

### (4-(6-(4-(4-Methylpiperazin-1-yl)phenyl)furo[3,2-*b*]pyridin-3-yl)phenyl)methanol (**80**)

The compound was prepared by the general procedure **B** using 50 mg (0.193 mmol) of (4-(6-chlorofuro[3,2-*b*]pyridin-3-yl)phenyl)methanol (**79**) and 70 mg (0.231 mmol) of 1-methyl-4-(4-(4,4,5,5-tetramethyl-1,3,2-dioxaborolan-2-yl)phenyl)piperazine. Mixture of 1,4-dioxane and water (4:1, 5 mL) was used as a solvent. The reaction time was 2 h. The reaction mixture was hot-filtered through a pad of Celite 535 (4 g) and the filtrate was concentrated in vacuo. Flash chromatography (DCM/MeOH, gradient from 0% to 5% of MeOH) afforded the product **80** as a white solid (55 mg, 71% yield). ^1^H NMR (500 MHz, Chloroform-*d*) δ 8.89 (d, *J* = 1.8 Hz, 1H), 8.12 (s, 1H), 8.10 (d, *J* = 8.2 Hz, 2H), 7.92 (d, *J* = 2.0 Hz, 1H), 7.57 (d, *J* = 8.7 Hz, 2H), 7.50 (d, *J* = 8.2 Hz, 2H), 7.05 (d, *J* = 8.9 Hz, 2H), 4.76 (s, 2H), 3.34–3.27 (m, 4H), 2.66–2.58 (m, 4H), 2.38 (s, 3H). ^13^C NMR (126 MHz, Chloroform-*d*) δ 151.20, 149.49, 145.15, 144.89, 144.38, 140.52, 133.25, 130.15, 128.93, 128.29, 127.66, 127.46, 121.65, 116.37, 116.06, 65.44, 55.19, 48.82, 46.31. FTIR (neat), cm^–1^: 3153, 2807, 1604, 1522, 1481, 1453, 1376, 1295, 1234, 1198, 1124, 1096, 1032, 1002, 968, 920, 836, 801, 625, 544, 532. HRMS (APCI): calcd. for C_25_H_25_N_3_O_2_ [M + H]^+^ = 400.2020; found [M + H]^+^ = 400.2021. MP: 236–237 °C

### 5-Chloro-3-(cinnamyloxy)-2-iodopyridine (**81**)

Cinnamyl bromide (463 mg, 2.349 mmol) was added to a mixture of 5-chloro-2-iodopyridin-3-ol (**2**; 500 mg, 1.957 mmol) and K_2_CO_3_ (649 mg, 4.698 mmol) in acetone (16 mL). The resulting reaction mixture was stirred under reflux for 24 h. After cooling to ambient temperature, water (30 mL) was added and the mixture was extracted with EtOAc (3 × 40 mL). The organic parts were washed with brine (50 mL), dried over Na_2_SO_4_, filtered, and the solvent was evaporated in vacuo. Flash chromatography (cyclohexane/EtOAc, gradient from 0% to 5% of EtOAc) afforded the product as pale yellow oil (712 mg, 98% yield). ^1^H NMR (500 MHz, Chloroform-*d*) δ 8.03 (d, *J* = 2.1 Hz, 1H), 7.47–7.40 (m, 2H), 7.39–7.33 (m, 3H), 7.33–7.28 (m, 1H), 7.03 (d, *J* = 2.1 Hz, 1H), 6.82 (d, *J* = 16.0 Hz, 1H), 6.38 (dt, *J* = 15.9, 5.6 Hz, 1H), 4.79 (dd, *J* = 5.6, 1.6 Hz, 2H). ^13^C NMR (126 MHz, Chloroform-*d*) δ 154.83, 141.48, 136.08, 134.45, 132.21, 128.89, 128.87, 128.51, 126.89, 122.27, 118.94, 109.30, 70.41. FTIR (neat), cm^–1^: 1717, 1556, 1411, 1270, 1192, 1116, 1044, 701, 452. HRMS (APCI): calcd. For C_14_H_11_ClINO [M + H]^+^ = 371.9647; found [M + H]^+^ = 371.9647.

### 3-Benzyl-6-chlorofuro[3,2-*b*]pyridine (**82**)

Pd(OAc)_2_ (5.4 mg, 0.024 mmol) was added to a degassed mixture of 5-chloro-3-(cinnamyloxy)-2-iodopyridine (**81**; 150 mg, 0.404 mmol), K_2_CO_3_ (140 mg, 1.009 mmol), HCOONa (28 mg, 0.404 mmol), and tetrabutylammonium chloride (123 g, 0.444 mmol) in *N*,*N*-dimethylformamide (3 mL) and the reaction mixture was stirred at 80 °C for 3 h. The reaction mixture was diluted with EtOAc (30 mL) and extracted with brine (6 × 30 mL). The organic layer was dried over Na_2_SO_4_, filtered, and the solvent was evaporated in vacuo. Flash chromatography (cyclohexane) afforded the product **82** as a yellow oil (38 mg, 39% yield). ^1^H NMR (500 MHz, Chloroform-*d*) δ 8.14 (d, *J* = 2.0 Hz, 1H), 7.43 (t, *J* = 7.7 Hz, 2H), 7.35 (t, *J* = 3.2 Hz, 1H), 7.29 (dd, *J* = 9.9, 8.3 Hz, 3H), 7.16 (d, *J* = 2.0 Hz, 1H), 5.58 (d, *J* = 3.2 Hz, 2H). ^13^C NMR (126 MHz, Chloroform-*d*) δ 156.96, 146.50, 141.73, 136.18, 132.55, 131.74, 129.11, 128.92, 128.89, 128.78, 128.01, 120.90, 117.21, 75.60. FTIR (neat), cm^–1^: 3061, 3030, 2967, 2926, 2857, 1694, 1605, 1524, 1452, 1401, 1236, 1201, 1166, 1079, 967, 914, 820, 767, 692. HRMS (APCI): calcd. For C_14_H_10_ClNO [M + H]^+^ = 244.0524; found [M + H]^+^ = 244.0525.

### 3-Benzyl-6-(4-(4-methylpiperazin-1-yl)phenyl)furo[3,2-*b*]pyridine (**83**)

The compound was prepared by the general procedure **B** using 30 mg (0.123 mmol) 3-benzyl-6-chlorofuro[3,2-*b*]pyridine (**82**) and 45 mg (0.148 mmol) of 1-methyl-4-(4-(4,4,5,5-tetramethyl-1,3,2-dioxaborolan-2-yl)phenyl)piperazine; the reaction time was 3 h; flash chromatography (DCM/MeOH, gradient from 0% to 10% of MeOH) afforded the compound as a pale yellow solid (16 mg, 34% yield). ^1^H NMR (500 MHz, Chloroform-*d*) δ 8.42 (d, *J* = 1.8 Hz, 1H), 7.55–7.49 (m, 2H), 7.43 (dd, *J* = 8.3, 7.1 Hz, 2H), 7.36 (t, *J* = 3.2 Hz, 1H), 7.34–7.28 (m, 4H), 7.05–6.97 (m, 2H), 5.58 (d, *J* = 3.2 Hz, 2H), 3.33 (t, *J* = 5.2 Hz, 4H), 2.64 (s, 4H), 2.40 (s, 3H). ^13^C NMR (126 MHz, Chloroform-*d*) δ 157.24, 151.23, 145.84, 141.62, 137.47, 136.69, 133.92, 129.04, 128.79, 128.05, 127.56, 119.44, 116.25, 114.31, 75.08, 55.05, 48.60, 46.14. FTIR (neat), cm^–1^: 2935, 2839, 2793, 1591, 1524, 1445, 1390, 1292, 1242, 1144, 977, 921, 820, 766, 689, 516. HRMS (APCI): calcd. For C_25_H_25_N_3_O [M + H]^+^ = 384.2070; found [M + H]^+^ = 384.2067. MP: decomposition

### 6-Chloro-3-(1-phenylvinyl)furo[3,2-*b*]pyridine (**84**)

The compound was prepared by the general procedure **A** using 150 mg (0.645 mmol) of 3-bromo-6-chlorofuro[3,2-*b*]pyridine (**5**) and 124 mg (0.839 mmol) of 1-(vinylphenyl)boronic acid, the reaction time was 3 h. The reaction mixture was hot-filtered through a pad of the mixture Celite 535/SiO_2_ (3:1, 4 g), and the filtrate was concentrated in vacuo. Flash chromatography (cyclohexane/EtOAc, gradient from 0% to 1% of EtOAc) afforded the product **84** as a white solid (145 mg, 88% yield). ^1^H NMR (500 MHz, Chloroform-*d*) δ 8.63 (d, *J* = 2.0 Hz, 1H), 7.82 (d, *J* = 2.1 Hz, 1H), 7.66 (s, 1H), 7.51–7.46 (m, 2H), 7.44–7.36 (m, 3H), 6.62 (d, *J* = 1.5 Hz, 1H), 5.64 (d, *J* = 1.5 Hz, 1H). ^13^C NMR (126 MHz, Chloroform-*d*) δ 148.35, 148.22, 145.12, 144.70, 140.91, 138.32, 128.46, 128.00, 127.91, 127.59, 121.90, 118.64, 117.82. FTIR (neat), cm^–1^: 3158, 3079, 3052, 3041, 3024, 1624, 1494, 1460, 1384, 1268, 1162, 1076, 1026, 900, 868, 811, 782, 766, 711, 693, 634, 598, 546, 483. HRMS (APCI): calcd. For C_15_H_10_ClNO [M + H]^+^ = 256.0524; found [M + H]^+^ = 256.0523. MP: decomposition

### 6-(4-(4-Methylpiperazin-1-yl)phenyl)-3-(1-phenylvinyl)furo[3,2-*b*]pyridine (**85**)

The compound was prepared by the general procedure **B** using 82 mg, (0.321 mmol) of 6-chloro-3-(1-phenylvinyl)furo[3,2-*b*]pyridine (**84**) and 107 mg, (0.353 mmol) of 1-methyl-4-(4-(4,4,5,5-tetramethyl-1,3,2-dioxaborolan-2-yl)phenyl)piperazine. The reaction time was 1 h. The reaction mixture was hot-filtered through a pad of a mixture Celite 535/SiO_2_ = 3:1 (4 g). The filtrate was concentrated in vacuo. Flash chromatography (DCM/MeOH, gradient from 0% to 4% of MeOH) afforded the compound (**85**) as an off-white solid (43 mg, 34% yield). ^1^H NMR (500 MHz, Chloroform-*d*) δ 8.89 (d, *J* = 2.0 Hz, 1H), 7.92 (d, *J* = 1.8 Hz, 1H), 7.65 (s, 1H), 7.59 (d, *J* = 8.9 Hz, 2H), 7.52 (dd, *J* = 8.1, 1.5 Hz, 2H), 7.45–7.36 (m, 3H), 7.07 (d, *J* = 8.9 Hz, 2H), 6.69 (d, *J* = 1.8 Hz, 1H), 5.65 (d, *J* = 1.7 Hz, 1H), 3.36–3.30 (m, 4H), 2.67–2.61 (m, 4H), 2.40 (s, 3H). ^13^C NMR (126 MHz, Chloroform-*d*) δ 151.02, 149.25, 147.52, 144.90, 144.58, 141.28, 138.84, 133.04, 128.79, 128.39, 128.13, 128.00, 127.87, 121.84, 117.33, 116.19, 115.81, 55.03, 48.66, 46.14. HRMS (APCI): calcd. For C_26_H_25_N_3_O [M + H]^+^ = 396.2070; found [M + H]^+^ = 396.2067. FTIR (neat), cm^–1^: 2934, 2846, 2813, 1605, 1525, 1479, 1449, 1382, 1340, 1291, 1234, 1147, 1105, 1000, 904, 814, 789, 727, 697, 654, 619, 524. MP: 186–187 °C

### 6-(4-(4-Methylpiperazin-1-yl)phenyl)-3-(1-phenylethyl)furo[3,2-*b*]pyridine (**86**)

The mixture of 100 mg (0.253 mmol) of 6-(4-(4-methylpiperazin-1-yl)phenyl)-3-(1-phenylvinyl)furo[3,2-*b*]pyridine (**85**) and 300 mg (0.282 mmol) of 10% palladium on carbon in 20.0 mL of MeOH was hydrogenated under pressure 10 bar at 23 °C for 2 h. The reaction mixture was filtered and the filtrate was concentrated in vacuo. Flash chromatography (DCM/MeOH, gradient from 0% to 4% of MeOH) provided the compound as an off-white solid (13 mg, 13% yield). ^1^H NMR (500 MHz, Chloroform-*d*) δ 8.78 (d, *J* = 2.0 Hz, 1H), 7.84 (d, *J* = 2.0 Hz, 1H), 7.54 (d, *J* = 8.9 Hz, 2H), 7.50 (s, 1H), 7.44 (d, *J* = 7.8 Hz, 2H), 7.35 (t, *J* = 7.7 Hz, 2H), 7.27–7.22 (m, 1H), 7.04 (d, *J* = 8.9 Hz, 2H), 4.52 (q, *J* = 6.9 Hz, 1H), 3.39–3.30 (m, 4H), 2.72–2.61 (m, 4H), 2.42 (s, 3H), 1.82 (d, *J* = 7.2 Hz, 3H). ^13^C NMR (126 MHz, Chloroform-*d*) δ 150.77, 148.97, 145.32, 145.30, 144.64, 144.49, 132.75, 129.23, 128.50, 128.11, 127.43, 126.70, 126.46, 116.27, 115.67, 54.93, 48.56, 45.99, 34.93, 21.26. HRMS (APCI): calcd. For C_26_H_27_N_3_O [M + H]^+^ = 398.2227; found [M + H]^+^ = 398.2225. FTIR (neat), cm^–1^: 2966, 2935, 2841, 2796, 1605, 1524, 1449, 1377, 1291, 1242, 1160, 1084, 1052, 1008, 919, 818, 764, 724, 699, 598, 530. MP: decomposition

### 6-Chloro-3-(1-methyl-1*H*-pyrazol-4-yl)furo[3,2-*b*]pyridine (**87**)

The compound was prepared by the general procedure **A** using 100 mg (0.430 mmol) of 3-bromo-6-chlorofuro[3,2-*b*]pyridine (**5**) and 116 mg (0.559 mmol) of 1-methylpyrazole-4-boronic acid pinacol ester; the reaction time was 2 h; flash chromatography (cyclohexane/EtOAc, gradient from 0% to 35% of EtOAc) afforded the compound **87** as a white solid (59 mg, 59% yield). ^1^H NMR (500 MHz, Chloroform-*d*) δ 8.55 (d, *J* = 2.1 Hz, 1H), 8.17 (s, 1H), 7.97 (s, 1H), 7.86 (s, 1H), 7.76 (d, *J* = 2.1 Hz, 1H), 3.97 (s, 3H). ^13^C NMR (126 MHz, Chloroform-*d*) δ 148.00, 145.07, 144.44, 143.85, 137.09, 128.77, 127.71, 118.66, 114.69, 110.40, 39.18. FTIR (neat), cm^–1^: 3132, 3088, 2926, 1524, 1459, 1385, 1277, 1197, 1126, 1073, 986, 925, 902, 883, 836, 782, 773, 719, 658, 619, 588, 525, 420. HRMS (APCI): calcd. for C_11_H_8_ClN_3_O [M + H]^+^ = 234.0429; found [M + H]^+^ = 234.0428. MP: 134–135 °C

### *tert*-Butyl 4-(4-(3-(1-methyl-1*H*-pyrazol-4-yl)furo[3,2-*b*]pyridin-6-yl)phenyl)piperazine-1-carboxylate (**88**)

The compound was prepared by the general procedure **B** using 45 mg (0.193 mmol) 6-chloro-3-(1-methyl-1H-pyrazol-4-yl)furo[3,2-*b*]pyridine (**87**) and 97 mg (0.250 mmol) of 4-(4-*tert*-butoxycarbonylpiperazinyl)phenylboronic acid pinacol ester; the reaction time was 2 h; flash chromatography (cyclohexane/EtOAc, gradient from 0% to 60% of EtOAc) afforded the compound **88** as a pale yellow solid (73 mg, 82% yield). ^1^H NMR (500 MHz, Chloroform-*d*) δ 8.83 (d, *J* = 1.8 Hz, 1H), 8.25 (s, 1H), 8.00 (s, 1H), 7.89 (dd, *J* = 4.9, 1.3 Hz, 2H), 7.56 (d, *J* = 8.7 Hz, 2H), 7.04 (d, *J* = 8.8 Hz, 2H), 3.99 (s, 3H), 3.76–3.53 (m, 4H), 3.22 (t, *J* = 5.2 Hz, 4H), 1.50 (s, 9H). ^13^C NMR (126 MHz, Chloroform-*d*) δ 154.86, 151.07, 148.96, 144.84, 143.31, 137.15, 137.13, 133.15, 129.60, 128.80, 128.78, 128.35, 119.29, 118.50, 116.88, 116.07, 114.56, 110.99, 80.15, 49.11, 43.69, 39.19, 28.59. FTIR (neat), cm^–1^: 2978, 2930, 1677, 1607, 1526, 1484, 1461, 1420, 1384, 1363, 1340, 1281, 1263, 1237, 1224, 1170, 1130, 1085, 1065, 1047, 997, 982, 928, 909, 869, 844, 823, 793, 765, 731, 714, 690, 662, 648, 631, 607, 594, 549, 526, 502, 454, 426, 412. HRMS (APCI): calcd. for C_26_H_29_N_5_O_3_ [M + H]^+^ = 460.2343; found [M + H]^+^ = 460.2347. MP: 197–198 °C

### 3-(1-Methyl-1*H*-pyrazol-4-yl)-6-(4-(piperazin-1-yl)phenyl)furo[3,2-*b*]pyridine (**89**)

The compound was prepared by the general procedure **C** using 60 mg (0.131 mmol) of *tert*-butyl 4-(4-(3-(1-methyl-1*H*-pyrazol-4-yl)furo[3,2-*b*]pyridin-6-yl)phenyl)piperazine-1-carboxylate (**88**); the reaction time was 2 h; flash chromatography (DCM/MeOH, gradient from 0% to 20% of MeOH) afforded the compound as a beige solid (26 mg, 55% yield). ^1^H NMR (500 MHz, Chloroform-*d*) δ 8.83 (d, *J* = 1.9 Hz, 1H), 8.24 (s, 1H), 7.99 (s, 1H), 7.93–7.85 (m, 2H), 7.55 (d, *J* = 8.7 Hz, 2H), 7.03 (d, *J* = 8.8 Hz, 2H), 3.99 (s, 3H), 3.26–3.19 (m, 4H), 3.16–3.04 (m, 4H). ^13^C NMR (126 MHz, Chloroform-*d*) δ 151.62, 148.96, 144.91, 144.36, 143.18, 137.14, 133.27, 129.05, 128.75, 128.25, 116.38, 115.94, 114.55, 111.06, 50.02, 46.15, 39.18. FTIR (neat), cm^–1^: 3307, 2937, 2817, 2686, 2467, 1605, 1524, 1482, 1451, 1423, 1381, 1344, 1239, 1202, 1190, 1174, 1146, 1120, 1079, 984, 927, 902, 885, 829, 816, 796, 786, 751, 664, 594, 540, 528. HRMS (APCI): calcd, for C_21_H_21_N_5_O [M + H]^+^ = 360.1819; found [M + H]^+^ = 360.1820. MP: 194–195 °C

### 6-Phenyl-3-(pyridin-4-yl)furo[3,2-*b*]pyridine (**90**)

The compound was prepared by the general procedure **B** using 50 mg (0.217 mmol) of 6-chloro-3-(pyridin-4-yl)furo[3,2-*b*]pyridine (**47**) and 34 mg (0.282 mmol) of phenylboronic acid; the reaction time was 2 h; flash chromatography (cyclohexane/EtOAc, gradient from 0% to 20% of EtOAc) afforded the compound **90** as a white solid (56 mg, 95% yield). ^1^H NMR (500 MHz, Chloroform-*d*) δ 8.93 (d, *J* = 1.9 Hz, 1H), 8.73 (bs, 2H), 8.30 (s, 1H), 8.07 (d, *J* = 5.7 Hz, 2H), 8.00 (d, *J* = 1.8 Hz, 1H), 7.69–7.62 (m, 2H), 7.52 (t, *J* = 7.6 Hz, 2H), 7.48–7.40 (m, 1H). ^13^C NMR (126 MHz, Chloroform-*d*) δ 150.43, 149.48, 146.87, 145.93, 144.42, 138.43, 137.96, 133.97, 129.38, 128.33, 127.68, 121.47, 119.64, 117.16. FTIR (neat), cm^–1^: 3043, 1602, 1384, 1367, 1204, 1098, 979, 882, 829, 787, 756, 699, 680, 633, 536, 525, 508. HRMS (APCI): calcd. for C_18_H_12_N_2_O [M + H]^+^ = 273.1022; found [M + H]^+^ = 273.1020.

### 6-(6-(Piperidin-1-yl)pyridin-3-yl)-3-(pyridin-4-yl)furo[3,2-*b*]pyridine (**91**)

The compound was prepared by the general procedure **B** using 60 mg (0.260 mmol) of 6-chloro-3-(pyridin-4-yl)furo[3,2-*b*]pyridine (**47**) and 97 mg (0.338 mmol) of 6-(piperidin-1-yl)pyridine-3-boronic acid pinacol ester; the reaction time was 2 h; flash chromatography (cyclohexane/EtOAc, gradient from 0% to 100% of EtOAc) afforded the compound **91** as a pale yellow solid (71 mg, 77% yield). ^1^H NMR (500 MHz, Chloroform-*d*) δ 8.86 (d, *J* = 1.9 Hz, 1H), 8.75–8.69 (m, 2H), 8.49 (dd, *J* = 2.6, 0.7 Hz, 1H), 8.27 (s, 1H), 8.10–8.02 (m, 2H), 7.92 (d, *J* = 1.9 Hz, 1H), 7.74 (dd, *J* = 8.9, 2.6 Hz, 1H), 6.78 (dd, *J* = 8.9, 0.8 Hz, 1H), 3.64 (dd, *J* = 5.5, 3.3 Hz, 4H), 1.69 (dd, *J* = 7.5, 3.5 Hz, 6H). ^13^C NMR (126 MHz, Chloroform-*d*) δ 159.08, 150.40, 149.65, 146.53, 145.05, 143.78, 138.57, 136.39, 131.51, 121.75, 121.45, 119.63, 115.84, 107.18, 46.46, 25.69, 24.87. FTIR (neat), cm^–1^: 2934, 2852, 1602, 1510, 1477, 1450, 1408, 1247, 1206, 1127, 809. HRMS (APCI): calcd, for C_22_H_20_N_4_O [M + H]^+^ = 357.1710; found [M + H]^+^ = 357.1709. MP: 184–185 °C

### 5-Chloro-2-iodopyridin-3-yl acetate (**92**)

5-Chloro-2-iodopyridin-3-ol (**2**; 1.79 g. 7.008 mmol) was mixed with acetic anhydride (5.0 mL, 53.0 mmol) and the mixture was stirred at 125 °C for 30 min. A saturated aqueous solution of NaHCO_3_ (80 mL) was added and the mixture was extracted with ethyl acetate (100 mL). The organic phase was washed with sat. aq. solution of NaHCO_3_ (50 mL, until evolution of CO_2_ ceased), the organic part was concentrated and all volatiles were evaporated in vacuo. The product was obtained as pale yellow solid (1.554 g, 75% yield). ^1^H NMR (500 MHz, Chloroform-*d*) δ 8.27 (d, *J* = 2.3 Hz, 1H), 7.41 (d, *J* = 2.3 Hz, 1H), 2.40 (s, 3H). ^13^C NMR (126 MHz, Chloroform-*d*) δ 167.81, 148.88, 146.84, 132.05, 130.31, 112.26, 21.32. FTIR (neat), cm^–1^: 3058, 2920, 1766, 1415, 1368, 1177, 1106, 1042, 1012, 930, 900, 857, 695, 654, 590, 543, 504. HRMS (APCI): calcd. for C_7_H_5_ClINO_2_ [M + H]^+^ = 297.9126; found [M + H]^+^ = 297.9127.

### 5-chloro-2-(pent-1-yn-1-yl)pyridin-3-yl acetate (**93**)

To a degassed solution of 5-chloro-2-iodopyridin-3-yl acetate (**92**; 756 mg, 2.541 mmol) in 1,4-dioxane (10 mL) and TEA (10 mL) were added pent-1-yne (0.326 mL, 3.304 mmol), PdCl_2_(PPh_3_)_2_ (54 mg, 0.076 mmol), and CuI (29 mg, 0.152 mmol), and the resulting mixture was stirred at 45 °C for 2 h. The solvent was evaporated in vacuo and the residue was purified by flash chromatography (cyclohexane/EtOAc, gradient from 1:0 to 10:1). The product was obtained as brown oil (604 mg, 100% yield). ^1^H NMR (500 MHz, Chloroform-*d*) δ 8.39 (d, *J* = 2.1 Hz, 1H), 7.48 (d, *J* = 2.2 Hz, 1H), 2.45 (t, *J* = 7.0 Hz, 2H), 2.35 (s, 3H), 1.65 (q, *J* = 7.2 Hz, 2H), 1.06 (t, *J* = 7.4 Hz, 3H). ^13^C NMR (126 MHz, Chloroform-*d*) δ 168.11, 148.36, 146.20, 136.24, 130.47, 130.16, 97.68, 75.64, 21.92, 21.66, 20.91, 13.65. FTIR (neat), cm^–1^: 2964, 2935, 2873, 2231, 1773, 1442, 1396, 1187, 1155, 1095, 1010, 932. HRMS (APCI): calcd. for C_12_H_12_ClNO_2_ [M + H]^+^ = 238.0629; found [M + H]^+^ = 238.0627.

### 6-Chloro-3-iodo-2-propylfuro[3,2-*b*]pyridine (**94**)

To a solution of 5-chloro-2-(pent-1-yn-1-yl)pyridin-3-yl acetate (**93**; 584 mg, 2.457 mmol) in MeOH (10 mL) were added solution of iodine (1.871 g, 7.371 mmol) in MeOH (10 mL) and CsHCO_3_ (1.429 g, 7.371 mmol), and the resulting mixture was stirred at 40 °C for 2 h in a flask wrapped with aluminum foil. A solution of Na_2_S_2_O_3_ (2.439 g, 9.828 mmol) in H_2_O (5 mL) was added and the mixture was concentrated to dryness in vacuo. The residue was purified by flash chromatography (cyclohexane/EtOAc, gradient from 1:0 to 9:1) to afford the product as a white solid (556 mg, 70% yield). ^1^H NMR (500 MHz, Chloroform-*d*) δ 8.52 (d, *J* = 2.0 Hz, 1H), 7.68 (d, *J* = 2.0 Hz, 1H), 2.90 (t, *J* = 7.4 Hz, 2H), 1.81 (q, *J* = 7.4 Hz, 2H), 1.01 (t, *J* = 7.4 Hz, 3H). ^13^C NMR (126 MHz, Chloroform-*d*) δ 164.43, 147.44, 147.04, 145.34, 127.77, 118.23, 65.31, 30.65, 21.16, 13.77. FTIR (neat), cm^–1^: 2962, 2929, 2873, 1733, 1700, 1577, 1457, 1384, 1236, 1074, 986, 940, 878. HRMS (APCI): calcd. for C_10_H_9_ClINO [M + H]^+^ = 321.9490; found [M + H]^+^ = 321.9491.

### 6-Chloro-3-(1-methyl-1*H*-pyrazol-4-yl)-2-propylfuro[3,2-*b*]pyridine (**95**)

The compound was prepared by the general procedure **A** using 90 mg (0.280 mmol) of 6-chloro-3-iodo-2-propylfuro[3,2-*b*]pyridine (**94**) and 76 mg (0.364 mmol) of 1-methyl-4-(4,4,5,5-tetramethyl-1,3,2-dioxaborolan-2-yl)-1*H*-pyrazole; the reaction time was 2 h; flash chromatography (cyclohexane/EtOAc, gradient from 0% to 20% of EtOAc) afforded the compound as a yellow solid (43 mg, 44% yield). ^1^H NMR (500 MHz, Chloroform-*d*) δ 8.49 (d, *J* = 2.0 Hz, 1H), 8.12 (s, 1H), 7.82 (s, 1H), 7.69 (d, *J* = 2.0 Hz, 1H), 4.00 (s, 3H), 2.96 (t, *J* = 7.5 Hz, 2H), 1.90–1.80 (m, 2H), 1.03 (t, *J* = 7.4 Hz, 3H). ^13^C NMR (126 MHz, Chloroform-*d*) δ 159.41, 146.96, 146.24, 144.29, 137.53, 129.35, 126.68, 117.85, 111.12, 109.25, 39.29, 30.13, 21.23, 14.01. FTIR (neat), cm^–1^: 3407, 2963, 1547, 1461, 1404, 1375, 1331, 1271, 1237, 1172, 1077, 1043, 985, 931, 904, 859, 792, 753, 720, 701, 670, 603, 549. HRMS (APCI): calcd. for C_14_H_14_ClN_3_O [M + H]^+^ = 276.0898; found [M + H]^+^ = 276.0901. MP: 139–140 °C

### 3-(1-Methyl-1*H*-pyrazol-4-yl)-6-(4-(4-methylpiperazin-1-yl)phenyl)-2-propylfuro[3,2-*b*]pyridine (**96**)

The compound was prepared by the general procedure **B** using 26 mg (0.094 mmol) of 6-chloro-3-(1-methyl-1*H*-pyrazol-4-yl)-2-propylfuro[3,2-*b*]pyridine (**95**) and 37 mg (0.122 mmol) of 1-methyl-4-(4-(4,4,5,5-tetramethyl-1,3,2-dioxaborolan-2-yl)phenyl)piperazine; the reaction time was 4 h; flash chromatography (DCM/MeOH, gradient from 0% to 6% of MeOH) afforded the compound as a white solid (22 mg, 56% yield). ^1^H NMR (500 MHz, Chloroform-*d*) δ 8.75 (d, *J* = 1.9 Hz, 1H), 8.18 (s, 1H), 7.84 (s, 1H), 7.80 (d, *J* = 1.9 Hz, 1H), 7.57–7.52 (m, 2H), 7.07–7.01 (m, 2H), 4.01 (s, 3H), 3.38–3.26 (m, 4H), 2.98 (t, *J* = 7.5 Hz, 6H), 2.72–2.54 (m, 4H), 2.40 (br s, 3H), 1.87 (h, *J* = 7.5 Hz, 2H), 1.05 (t, *J* = 7.4 Hz, 3 H). ^13^C NMR (126 MHz, Chloroform-*d*) δ 158.40, 150.91, 147.75, 146.35, 144.24, 137.64, 132.26, 129.25, 128.21, 116.49, 115.09, 111.78, 109.15, 55.11, 48.78, 46.16, 39.24, 30.16, 21.38, 14.06. FTIR (neat), cm^–1^: 2961, 2163, 2051, 1608, 1524, 1480, 1378, 1289, 1244, 1088, 985, 930, 821, 686, 535, 490. HRMS (APCI): calcd. for C_25_H_29_N_5_O [M + H]^+^ = 416.2445; found [M + H]^+^ = 416.2444. MP: 194–195 °C

### (2-Fluoro-4-(5-(4-(4-isopropylpiperazin-1-yl)phenyl)-4-methylpyridin-3-yl)-6-methoxyphenyl)(pyrrolidin-1-yl)methanone (**97**, M4K2234NC)

To a degassed solution of 2-fluoro-6-methoxy-4-[4-methyl-5-[4-(4-propan-2-ylpiperazin-1-yl)phenyl]pyridin-3-yl]benzamide (50 mg, 0.108 mmol) in DMF (2.0 mL) was added sodium hydride (5.71 mg, 0.238 mmol) at 0 °C and the reaction mixture was stirred for 10 min. Then, 1,4-dibromobutane (23.3 mg, 0.108 mmol) was added and the reaction mixture was stirred and warmed up to 25 °C over 3 h and then it was stirred for additional 3 h at 25 °C; the progress of the reaction was followed by TLC. After consumption of the starting material, the mixture was diluted with 0.2 mL of saturated aqueous solution of NH_4_Cl. Then, volatiles were evaporated and the residues were adsorbed on Celite and purified by RP-flash chromatography (water/MeCN, gradient from 5% to 100% of MeCN). The product was obtained as a white solid (25 mg, 45% yield). ^1^H NMR (500 MHz, DMSO-*d*_6_) δ 8.37 (d, *J* = 0.8 Hz, 2H), 7.30 (d, *J* = 8.8 Hz, 2H), 7.07–6.98 (m, 4H), 3.86 (s, 3H), 3.50–3.43 (m, *J* = 6.7 Hz, 2H), 3.25–3.13 (m, *J* = 11.8 Hz, 6H), 2.70 (s, 1H), 2.66–2.55 (m, 4H), 2.19 (s, 3H), 1.92–1.80 (m, 4H), 1.02 (d, *J* = 6.5 Hz, 6H). ^13^C NMR (126 MHz, DMSO-*d*_6_) δ 160.97, 158.91, 156.98, 156.20, 156.13, 150.46, 148.92, 147.44, 141.66, 140.81, 140.73, 137.43, 136.30, 130.10, 127.30, 114.91, 114.60, 114.42, 109.26, 109.13, 108.95, 56.47, 48.11, 46.80, 45.11, 25.24, 24.10, 18.23, 18.00. HRMS (ESI): calcd. for C_31_H_37_FN_4_O_2_ [M + H]^+^ = 517.2973; found [M + H]^+^ = 517.2993.

## Supporting Information Available

^1^H NMR, ^13^C NMR, HRMS, and IR spectra of intermediates and final compounds; additional figures, tables, details of pharmacokinetic/biochemical/kinome-wide profiling, crystallization, Western blots, and inhibition activity (PDF)
Molecular formula strings (CSV)

## Accession Codes

The PDB code for MU1700 with ALK2(172–499)-FKBP12 is 8POD. The PDB code for M4K2234 with ALK2(201–499) is 8R7G.

## ABBREVIATIONS

ALK1–7: activin receptor-like kinases 1–7
ACVR1: activin A receptor type 1 human recombinant
ACVR1C: activin A receptor
ACVR1B: activin receptor type-1B
ACVRL1: activin receptor-like kinase 1
AMPK: adenosine monophosphate-activated protein kinase
ATP: adenosine triphosphate
BBB: blood–brain barrier
BMP: bone morphogenetic protein
BMPR1A: bone morphogenetic protein receptor type-IA
BMPR1B: bone morphogenetic protein receptor type-1B
CNS: central nervous system
DCM: dichloromethane
DMF: *N*,*N*-dimethylformamide
DMG: diffuse midline glioma
DMSO: dimethyl sulfoxide
ENG: endoglin
EtOAc: ethyl acetate
FKBP12: peptidyl-prolyl *cis*–*trans* isomerase
FOP: fibrodysplasia ossificans progressiva
FTIR: Fourier-transform infrared spectroscopy
GS: glycine-serine rich
HHT: hereditary hemorrhagic telangiectasia
HRMS: high-resolution mass spectrometry
hERG: human ether-a-go-go related gene
LC/MS: Liquid chromatography–mass spectrometry
LDL: low-density lipoprotein
MeCN: acetonitrile
MeOH: methanol
MP: melting point
NCK: noncatalytic region of tyrosine kinase adaptor protein 1
NMR: nuclear magnetic resonance
RP: reversed phase
STE TEA: triethylamine
TLC: thin layer chromatography
TNIK: TRAF2 and NCK-interacting protein kinase
TGF-β: transforming growth factor
TGFBR1: transforming growth factor beta receptor I
TLKs: tyrosine kinase-like kinases
TRAF2: TNF receptor-associated factor 2
VEGF-A: Vascular endothelial growth factor A
