# Supplementary material for: Discovery of Two Highly Selective Structurally Orthogonal Chemical Probes for Activin Receptor-like Kinases 1 and 2
Source: J Med Chem. 2024 Jul 18;67(15):12632–59. doi: 10.1021/acs.jmedchem.4c00629 (PMC11320582; doi:10.1021/acs.jmedchem.4c00629)
Supplement: Supplementary file 1 — jm4c00629_si_001.pdf [file jm4c00629_si_001.pdf]

Supporting Information

Discovery of two highly selective structurally orthogonal chemical probes for Activin receptor-like kinases 1 and 2

Václav Němec[1, 2,#], Marek Remeš[2,#], Petr Beňovský[2], Michael C. Böck[2], Eliška Šranková[2], Jong Fu Wong[3], Julien Cros[3], Eleanor Williams[3], Lap Hang Tse[3], David Smil[4], Deeba Ensan[4], Methvin B. Isaac[4], Rima Al-Awar[4,5], Regina Gomolková[6,7], Vlad-Constantin Ursachi[6,8], Bohumil Fafílek[6,7,8], Zuzana Kahounová[9], Ráchel Víchová[9], Ondřej Vacek[8,9], Benedict-Tilman Berger[1], Carrow I. Wells[10], Cesear R. Corona[11], James D. Vasta[11], Matthew B. Robers[11], Pavel Krejci[6,7,8], Karel Souček[8,9], Alex N. Bullock[3], Stefan Knapp[1,*], Kamil Paruch[2,7,*]

[1]Institute for Pharmaceutical Chemistry, Structural Genomics Consortium, Johann Wolfgang Goethe-University, Max-von-Laue-Strasse 9, Frankfurt am Main, 60438, Germany

[2]Department of Chemistry, Masaryk University, Brno 625 00, Czech Republic

[3]Centre for Medicines Discovery, Nuffield Department of Medicine, University of Oxford, Oxford OX3 7FZ, UK

[4]Drug Discovery Program, Ontario Institute for Cancer Research, 661 University Avenue, Toronto, Ontario M5G 0A3, Canada

[5]Department of Pharmacology and Toxicology, University of Toronto, Toronto, Ontario M5S 1A8, Canada

[6]Department of Biology, Faculty of Medicine, Masaryk University, 625 00 Brno, Czech Republic

[7]Institute of Animal Physiology and Genetics of the Czech Academy of Sciences, 602 00 Brno, Czech Republic

[8]International Clinical Research Center, St. Anne's University Hospital, 602 00 Brno, Czech Republic

[9]Institute of Biophysics of the Czech Academy of Sciences, Královopolská 135, 612 00 Brno Czech Republic

[10]Structural Genomics Consortium, UNC Eshelman School of Pharmacy, The University of North Carolina at Chapel Hill, Chapel Hill, NC, 27599, USA

[11]Promega Corporation, Madison, WI, USA

# these authors contributed equally

*Correspondence: knapp@pharmchem.uni-frankfurt.de (S.K.), paruch@chemi.muni.cz (K.P.)

Supporting Information

Table of contents

1. **Kinome-wide profiling of MU1700 and LDN-193189......3**
2. **Crystallization of MU1700-ALK2 complex and M4K2234-ALK2 complex. 8**
3. **Kinome-wide profiling of M4K2234 ......11**
4. **Biochemical profiling of M4K2234 ......13**
5. **Profiling MU1700 and M4K2234 in the NanoBRET^{TM} target engagement assay in intact cells 15**
6. **Inhibition of SMAD-dependent transcriptional reporters......18**
7. **Western blot analysis of SMAD protein phosphorylation after receptor activation 19**
8. **Pharmacokinetic profile of MU1700.2HCl......21**
9. **Determination of IC_{50} for *in vitro* CYP450 inhibition in human liver microsomes for MU1700......43**
10. **Identification of hERG Potassium Channel Inhibition 60**
11. **Assessment of Metabolic Stability in Human and Mouse Liver Microsomes for MU1700......64**
12. **Assessment of Caco-2 Permeability for Compound MU17002HCl......70**
13. **Comparison of the chemical probes MU1700 and M4K2234 with selected ALK2 inhibitors. 77**
14. **Cell viability assessment......78**
15. **Liver microsomal metabolic stability assay. 79**
16. **The inhibitory activity against ALK1-6: Kinase profiling of MU1700 and its analogs......81**
17. **NMR, FTIR, and HRMS spectra of prepared compounds 115**
18. **HPLC traces of MU1700 and M4K2234......312**
19. **Molecular formula strings 323**
20. **References 324**

1. Kinome-wide profiling of MU1700 and LDN-193189 (Reaction Biology)

Table S1: Data from kinome-wide profiling of MU1700 and LDN-193189 against the indicated kinases at 1 μM concentration (radiometric assays, Reaction Biology). The compounds were tested in single dose duplicate mode at a concentration of 1 μM. The control compound staurosporine was tested in 10-dose IC_{50} mode with 4-fold serial dilution starting at 20 or 100 μM. The other control compounds were tested in 10-dose IC_{50} mode with 3-fold or 4-fold serial dilution starting at 10, 20, or 100 μM. The reactions were carried out at 10 μM ATP.

Kinase	% Enzyme Activity (relative to DMSO controls)				IC50 (M) Control Cmpd	Control Cmpd ID
	MU1700 (1μM)		LDN193189 (1μM)			
	Data 1	Data 2	Data 1	Data 2		
FLT3	44.42	44.24	-0.76	-0.83	1.51E-09	STAUROSPORINE
ALK2/ACVR1	1.19	1.07	-0.56	-0.66	1.49E-08	LDN193189
ALK1/ACVRL1	2.24	1.81	0.18	-0.18	1.56E-08	LDN193189
TNIK	42.06	40.68	0.45	0.43	8.09E-10	STAUROSPORINE
KHS/MAP4K5	28.48	28.01	0.60	0.44	3.61E-10	STAUROSPORINE
ALK6/BMPR1B	7.51	7.30	1.05	0.21	1.08E-08	LDN193189
SIK2	54.84	54.68	2.21	2.01	1.44E-09	STAUROSPORINE
DDR1	28.73	27.70	3.57	3.53	6.65E-09	STAUROSPORINE
ABL1	51.55	50.27	3.90	3.22	9.08E-08	STAUROSPORINE
ZAK/MLTK	65.67	63.74	4.61	4.44	1.30E-06	GW5074
ABL2/ARG	73.42	72.02	8.53	8.40	3.65E-08	STAUROSPORINE
MYO3b	87.40	86.40	9.08	8.71	9.24E-09	STAUROSPORINE
TGFBR2	71.39	70.69	9.81	9.74	1.13E-07	LDN193189
BRK	59.90	58.08	9.97	9.82	2.49E-07	STAUROSPORINE
MINK/MINK1	107.25	106.97	11.23	11.12	1.72E-09	STAUROSPORINE
YES/YES1	69.73	69.20	13.13	12.70	2.80E-09	STAUROSPORINE
LIMK1	76.06	71.85	14.74	14.65	2.32E-09	STAUROSPORINE
HGK/MAP4K4	101.21	98.25	15.71	14.73	1.78E-09	STAUROSPORINE
ALK4/ACVR1B	92.79	90.07	16.18	15.92	4.32E-07	LDN193189
ARK5/NUAK1	88.35	85.58	17.92	17.29	1.86E-09	STAUROSPORINE
MARK2/PAR-1Ba	80.79	79.19	20.07	16.21	4.94E-11	STAUROSPORINE
FGR	66.49	59.27	18.49	18.24	1.46E-09	STAUROSPORINE
CDK7/cyclin H	89.50	87.56	18.72	18.50	7.69E-08	STAUROSPORINE
MELK	83.31	83.10	19.84	19.70	6.59E-10	STAUROSPORINE
FYN	83.03	80.95	20.26	20.05	2.43E-09	STAUROSPORINE
SIK1	98.02	94.88	21.94	18.38	1.86E-09	STAUROSPORINE
CLK1	102.85	102.34	20.71	20.61	1.02E-08	STAUROSPORINE
ALK5/TGFBR1	88.95	88.49	22.31	21.62	5.93E-07	LDN193189
DYRK1B	95.11	94.86	22.72	22.37	2.71E-09	STAUROSPORINE
LYN	78.73	76.23	22.98	22.87	2.12E-09	STAUROSPORINE
SIK3	95.53	94.24	23.30	23.08	2.12E-09	STAUROSPORINE
DYRK1/DYRK1A	95.64	95.34	23.81	23.56	5.51E-09	STAUROSPORINE
FRK/PTK5	77.65	77.49	25.85	22.76	6.51E-09	STAUROSPORINE
ALK3/BMPR1A	41.68	39.57	25.84	24.31	2.29E-08	LDN193189
KDR/VEGFR2	106.28	103.13	26.08	25.52	8.22E-09	STAUROSPORINE
MARK1	90.29	89.98	26.65	26.27	3.94E-10	STAUROSPORINE
MARK3	100.71	100.32	27.64	26.38	1.52E-10	STAUROSPORINE
MARK4	98.20	95.36	27.90	27.82	1.93E-10	STAUROSPORINE
GCK/MAP4K2	99.48	96.54	28.39	27.73	7.67E-10	STAUROSPORINE
LOK/STK10	91.74	90.34	28.41	27.82	1.14E-07	RO-31-8220
CK1a1	80.49	80.31	28.40	27.93	3.54E-06	STAUROSPORINE
TESK2	80.27	75.94	29.90	27.09	2.24E-05	STAUROSPORINE
c-Kit	85.61	82.23	29.96	29.35	1.16E-09	STAUROSPORINE
TYRO3/SKY	76.78	75.19	30.39	29.78	8.15E-09	STAUROSPORINE
MLCK2/MYLK2	86.75	86.31	30.59	29.91	5.20E-08	STAUROSPORINE
RIPK2	64.73	64.29	31.67	29.70	1.17E-07	STAUROSPORINE
NLK	87.12	85.03	32.30	31.25	8.95E-08	STAUROSPORINE
CAMKK2	94.40	92.88	32.06	31.95	4.43E-08	STAUROSPORINE
BRAF	71.38	68.65	33.32	32.25	4.77E-09	GW5074
GLK/MAP4K3	104.44	98.52	33.30	33.05	3.08E-10	STAUROSPORINE
ACK1	98.31	92.30	36.78	35.44	3.10E-08	STAUROSPORINE
CLK2	118.46	113.12	37.56	36.12	7.28E-09	STAUROSPORINE
CDK6/cyclin D3	91.27	87.18	39.00	38.53	3.42E-08	STAUROSPORINE
PDGFRb	104.80	97.12	39.09	38.60	2.63E-09	STAUROSPORINE
LCK	103.88	100.09	39.48	39.06	3.82E-09	STAUROSPORINE
LRRK2	93.67	93.10	39.88	39.85	1.38E-08	STAUROSPORINE
STK16	96.53	95.10	41.92	40.56	3.17E-07	STAUROSPORINE
STK39/STLK3	77.66	76.94	43.86	42.15	2.49E-08	STAUROSPORINE

FLT4/VEGFR3	75.65	72.87	45.99	42.95	3.66E-09	STAUROSPORINE
CLK4	94.73	93.83	45.76	43.81	8.08E-08	STAUROSPORINE
FLT1/VEGFR1	78.80	77.91	47.05	46.52	1.14E-08	STAUROSPORINE
LYN B	93.25	89.85	49.48	48.14	6.56E-09	STAUROSPORINE
PRKX	95.52	93.68	49.00	48.85	1.94E-09	STAUROSPORINE
PHKg1	79.87	78.44	50.35	48.53	1.68E-09	STAUROSPORINE
FGFR1	104.79	102.89	50.53	48.35	9.79E-09	STAUROSPORINE
TRKC	76.62	72.99	50.75	49.37	3.83E-10	STAUROSPORINE
FGFR2	102.41	101.94	50.34	50.09	5.76E-09	STAUROSPORINE
CDK5/P25	102.66	99.09	51.56	49.48	1.37E-09	STAUROSPORINE
ULK2	116.21	115.91	52.22	52.04	4.54E-09	STAUROSPORINE
c-Src	94.86	92.87	52.77	52.10	5.60E-09	STAUROSPORINE
LIMK2	97.16	90.04	52.75	52.31	1.73E-07	STAUROSPORINE
MNK1	98.64	96.97	53.03	52.96	9.99E-08	STAUROSPORINE
MNK2	101.34	99.89	54.49	53.76	2.59E-08	STAUROSPORINE
STK33	104.85	103.98	57.11	54.90	5.57E-08	STAUROSPORINE
BLK	114.79	114.47	56.55	56.39	4.40E-09	STAUROSPORINE
PKCnu/PRKD3	94.95	92.50	58.13	58.11	1.41E-09	STAUROSPORINE
TESK1	93.05	92.31	59.19	57.36	8.05E-08	STAUROSPORINE
PKCmu/PRKD1	81.59	81.16	60.08	57.73	2.19E-09	STAUROSPORINE
CK1d	98.28	94.28	62.05	57.47	2.00E-07	D4476
DDR2	125.73	117.94	60.51	60.43	8.67E-10	STAUROSPORINE
AURORA C	89.41	88.47	61.54	59.76	2.25E-09	STAUROSPORINE
CDK19/cyclin C	96.32	95.89	61.92	59.90	8.15E-10	STAUROSPORINE
DCAMKL2	64.91	64.75	64.02	58.80	1.67E-08	STAUROSPORINE
RAF1	90.83	88.91	61.69	61.38	3.20E-09	GW5074
CK1g1	102.51	98.00	63.47	60.34	1.80E-05	STAUROSPORINE
HCK	95.33	92.83	64.10	60.01	2.25E-09	STAUROSPORINE
CDK6/cyclin D1	90.81	90.38	63.12	61.56	1.20E-08	STAUROSPORINE
MYO3A	99.09	96.61	62.61	62.46	4.28E-08	STAUROSPORINE
CAMKK1	107.99	107.17	67.83	58.84	8.07E-08	STAUROSPORINE
MUSK	94.30	93.96	63.86	62.93	8.36E-09	STAUROSPORINE
TIE2/TEK	86.03	85.99	64.52	64.29	2.59E-08	STAUROSPORINE
SNARK/NUAK2	109.94	109.40	64.54	64.40	2.15E-09	STAUROSPORINE
Aurora B	88.74	86.15	65.36	64.17	2.23E-08	STAUROSPORINE
TXK	82.97	80.78	65.74	65.64	3.84E-08	STAUROSPORINE
PDGFRa	88.29	88.22	66.40	65.10	4.22E-09	STAUROSPORINE
HPK1/MAP4K1	102.09	100.51	66.08	65.48	8.70E-08	RO-31-8220
CDK2/cyclin E2	92.31	91.63	66.39	65.41	3.12E-09	STAUROSPORINE
EPHB2	97.66	96.41	66.97	66.30	5.44E-08	STAUROSPORINE
TBK1	95.43	94.36	67.99	66.60	1.95E-09	STAUROSPORINE
SYK	80.08	78.95	67.42	67.39	1.98E-10	STAUROSPORINE
Aurora A	87.75	86.65	67.82	67.36	1.78E-09	STAUROSPORINE
CDK4/cyclin D3	99.59	96.42	68.58	67.13	3.04E-08	STAUROSPORINE
ARAF	84.52	82.45	68.51	67.61	1.54E-08	GW5074
CDK8/cyclin C	91.47	87.09	69.25	67.53	1.79E-10	STAUROSPORINE
CK1epsilon	95.79	94.64	69.14	68.87	3.39E-07	D4476
PAK1	80.75	78.04	72.12	66.03	2.66E-10	STAUROSPORINE
TNK1	100.90	99.81	70.75	67.91	7.55E-09	STAUROSPORINE
PKN3/PRK3	78.68	77.33	71.27	67.93	4.98E-09	STAUROSPORINE
NEK11	71.78	70.01	70.52	69.20	5.77E-07	STAUROSPORINE
FMS	91.50	89.85	72.15	68.64	3.29E-09	STAUROSPORINE
PKD2/PRKD2	97.19	93.40	71.58	69.95	3.60E-09	STAUROSPORINE
BMX/ETK	91.41	89.49	72.91	70.17	9.63E-09	STAUROSPORINE
EPHA1	91.24	90.19	73.14	70.96	3.98E-07	STAUROSPORINE
FES/FPS	56.26	54.95	75.10	70.19	3.71E-09	STAUROSPORINE
CDK2/cyclin O	102.80	102.51	74.15	72.04	3.35E-09	STAUROSPORINE
PKN1/PRK1	80.68	78.71	73.60	72.76	2.27E-10	STAUROSPORINE
TRKB	82.36	72.10	76.74	70.42	6.76E-10	STAUROSPORINE
CDK3/cyclin E	97.88	95.91	74.24	73.49	2.71E-09	STAUROSPORINE
IKKa/CHUK	110.33	105.86	75.21	72.83	1.23E-07	STAUROSPORINE
CK1a1L	91.72	89.93	74.47	74.07	5.38E-06	STAUROSPORINE
MLK1/MAP3K9	100.05	98.78	75.79	72.75	1.39E-09	STAUROSPORINE
CSK	118.93	110.78	81.26	67.97	3.03E-08	STAUROSPORINE
BMPR2	92.78	86.59	75.62	74.07	9.60E-08	STAUROSPORINE
CDK1/cyclin E	97.98	96.18	76.61	73.10	7.93E-09	STAUROSPORINE
CDK5/p35	106.02	105.46	75.72	74.84	3.68E-09	STAUROSPORINE
TYK2	105.86	105.31	77.87	73.67	2.05E-10	STAUROSPORINE
IRR/INSRR	78.93	77.95	77.06	74.72	1.68E-08	STAUROSPORINE
YSK4/MAP3K19	96.49	94.45	77.08	75.23	1.15E-08	STAUROSPORINE
NEK4	96.30	94.94	77.00	75.46	2.79E-07	STAUROSPORINE
RSK3	98.94	98.26	77.58	75.24	3.39E-10	STAUROSPORINE
BRSK1	92.93	92.32	78.38	76.05	1.69E-10	STAUROSPORINE
CDK16/cyclin Y (PCTAIRE)	98.91	85.98	84.59	70.46	2.54E-08	STAUROSPORINE
RET	100.17	99.45	79.09	76.07	6.91E-09	STAUROSPORINE
RSK4	106.91	105.53	78.43	77.91	4.19E-10	STAUROSPORINE
CDK2/cyclin A1	101.95	99.56	78.84	77.86	1.74E-09	STAUROSPORINE
EPHA8	95.51	91.34	79.71	77.99	1.62E-07	STAUROSPORINE
ALK	96.18	95.25	79.99	78.96	3.41E-09	STAUROSPORINE
BTK	82.93	77.89	80.37	79.24	3.30E-08	STAUROSPORINE

CDK4/cyclin D1	92.49	89.50	80.53	79.48	1.47E-08	STAUROSPORINE
PKCiota	96.23	89.38	80.79	79.35	1.44E-08	STAUROSPORINE
RSK1	91.59	89.37	80.79	79.68	3.94E-10	STAUROSPORINE
CDK2/CYCLIN E	97.99	96.72	80.65	80.21	3.68E-09	STAUROSPORINE
CK1G3	94.28	92.34	81.87	79.82	3.51E-06	STAUROSPORINE
DYRK2	95.57	95.18	81.11	80.73	1.81E-07	STAUROSPORINE
FGFR4	100.19	98.68	81.11	80.82	1.15E-07	STAUROSPORINE
CAMK2d	95.10	93.54	81.80	80.78	5.50E-11	STAUROSPORINE
CDK3/cyclin E2	102.09	99.16	81.60	81.32	3.82E-09	STAUROSPORINE
ULK1	103.51	97.94	82.75	80.64	1.10E-08	STAUROSPORINE
MST3/STK24	106.66	93.62	82.27	81.32	3.72E-09	STAUROSPORINE
TRKA	88.17	87.77	82.15	81.84	4.14E-09	STAUROSPORINE
DAPK1	81.96	78.56	83.54	81.00	2.71E-08	STAUROSPORINE
IR	80.44	80.17	83.78	81.45	1.07E-08	STAUROSPORINE
MLK2/MAP3K10	90.14	87.39	83.13	82.30	6.05E-09	STAUROSPORINE
DCAMKL1	85.09	83.56	84.72	80.81	1.32E-07	STAUROSPORINE
EPHA2	95.11	95.09	85.22	80.39	1.22E-07	STAUROSPORINE
NEK1	102.27	100.65	82.95	82.67	1.35E-08	STAUROSPORINE
EPHB1	88.72	84.10	83.79	81.87	7.94E-08	STAUROSPORINE
ROCK2	86.51	79.67	85.58	81.58	1.28E-09	STAUROSPORINE
DYRK3	104.15	92.13	83.96	83.63	7.21E-08	STAUROSPORINE
CDK2/cyclin A	97.59	97.57	84.87	83.20	8.86E-10	STAUROSPORINE
CDK9/cyclin T2	93.02	92.63	85.25	83.68	5.22E-09	STAUROSPORINE
TAK1	98.99	98.78	85.91	83.73	1.78E-07	STAUROSPORINE
EPHA7	94.82	93.16	85.20	85.05	3.54E-08	STAUROSPORINE
MEK2	90.16	87.22	87.62	82.96	7.95E-08	STAUROSPORINE
IRAK1	97.82	95.94	86.25	85.08	1.14E-07	STAUROSPORINE
EPHB4	92.68	90.23	85.92	85.65	3.33E-07	STAUROSPORINE
SGK3/SGKL	87.53	82.32	87.90	85.16	3.60E-08	STAUROSPORINE
EPHA5	103.16	103.10	87.40	86.01	1.08E-08	STAUROSPORINE
ERBB4/HER4	98.35	97.69	88.54	85.33	5.74E-07	STAUROSPORINE
PHKg2	99.88	96.82	89.73	86.34	1.89E-09	STAUROSPORINE
CDK14/cyclin Y (PFTK1)	96.91	96.11	89.72	87.61	1.56E-07	STAUROSPORINE
CDK9/CYCLIN K	92.75	91.55	88.83	88.69	3.63E-08	STAUROSPORINE
CDC7/DBF4	101.65	100.64	90.74	87.48	3.86E-08	STAUROSPORINE
NEK5	101.85	99.01	89.44	89.03	7.82E-08	STAUROSPORINE
SLK/STK2	99.50	99.29	90.12	88.53	1.17E-08	STAUROSPORINE
MAST3	96.95	95.28	90.46	88.36	3.73E-07	STAUROSPORINE
TEC	93.93	91.85	91.33	87.74	1.13E-07	STAUROSPORINE
WNK1	88.41	88.14	90.56	88.62	9.34E-06	STAUROSPORINE
EPHA3	97.16	93.60	91.41	88.03	5.63E-08	STAUROSPORINE
PAK3	91.74	84.05	90.20	89.86	3.65E-10	STAUROSPORINE
PKCzeta	84.66	84.30	90.46	89.69	6.78E-08	STAUROSPORINE
ERK7/MAPK15	113.74	112.54	92.75	87.60	1.29E-08	STAUROSPORINE
MST1/STK4	103.71	102.74	91.99	88.72	1.49E-09	STAUROSPORINE
IGF1R	102.63	95.70	91.40	89.58	2.42E-08	STAUROSPORINE
SBK1	95.22	92.78	91.19	90.16	1.10E-07	STAUROSPORINE
BRSK2	96.91	95.52	92.50	88.86	2.34E-09	STAUROSPORINE
PKCb2	96.93	94.72	90.93	90.70	2.41E-09	STAUROSPORINE
SRPK2	96.45	95.36	90.93	90.70	1.42E-07	STAUROSPORINE
NEK3	103.43	95.86	91.83	89.95	1.20E-07	JNK-IN-7
PIM1	94.98	92.86	92.73	89.22	8.75E-09	STAUROSPORINE
ERK2/MAPK1	99.07	95.55	92.47	89.49	4.98E-10	SCH772984
MEKK6	113.03	106.67	91.26	91.12	5.06E-07	STAUROSPORINE
VRK1	92.75	88.13	92.60	89.85	1.22E-06	RO-31-8220
WEE1	101.87	99.48	92.56	90.56	7.28E-08	WEE-1 INHIBITOR
KSR1	102.82	97.79	95.88	87.29	9.09E-06	STAUROSPORINE
PKG1b	89.73	89.28	92.55	90.78	3.29E-09	STAUROSPORINE
STK22D/TSSK1	104.73	104.22	92.23	91.46	1.09E-10	STAUROSPORINE
VRK2	90.83	90.46	92.34	91.49	1.26E-05	RO-31-8220
PKCd	94.15	92.93	92.34	91.57	2.14E-10	STAUROSPORINE
CAMK2b	99.39	99.18	92.26	91.79	7.85E-11	STAUROSPORINE
CDK9/cyclin T1	102.64	102.03	95.79	88.30	1.36E-08	STAUROSPORINE
MYLK4	97.67	97.00	92.38	91.76	9.51E-08	STAUROSPORINE
EGFR	88.27	87.74	93.95	90.29	3.00E-07	STAUROSPORINE
ERN1/IRE1	95.96	95.78	92.51	91.78	1.30E-07	STAUROSPORINE
MASTL	103.31	102.88	92.36	92.08	9.33E-08	STAUROSPORINE
DAPK2	103.25	99.85	92.50	91.96	7.74E-09	STAUROSPORINE
MAPKAPK2	90.41	88.00	93.10	91.90	1.93E-07	STAUROSPORINE
IKKe/IKBKE	108.50	103.21	92.79	92.30	5.71E-10	STAUROSPORINE
PKCeta	108.71	102.79	94.44	90.87	4.13E-10	STAUROSPORINE
Haspin	97.49	94.31	93.28	92.23	1.93E-08	STAUROSPORINE
CAMK1g	88.03	86.26	93.27	92.31	1.23E-08	STAUROSPORINE
PKCg	96.84	96.24	94.46	91.39	3.15E-10	STAUROSPORINE
LKB1	98.33	96.15	94.37	91.65	5.13E-08	STAUROSPORINE
PKN2/PRK2	93.77	90.87	94.73	91.50	2.04E-09	STAUROSPORINE
MEKK1	98.95	97.25	95.26	90.97	1.72E-06	STAUROSPORINE
P38a/MAPK14	102.54	95.85	93.39	92.89	1.23E-08	SB202190
LATS1	99.04	97.43	94.03	92.55	6.07E-09	STAUROSPORINE
ITK	90.59	89.11	96.53	90.19	1.13E-08	STAUROSPORINE

CK2a2	98.40	95.97	94.08	93.60	2.01E-06	STAUROSPORINE
CHK2	105.40	102.99	94.11	93.80	8.47E-09	STAUROSPORINE
TAOK2/TAO1	96.28	95.57	94.13	93.86	5.27E-09	STAUROSPORINE
c-MER	105.23	100.93	95.78	92.58	2.11E-08	STAUROSPORINE
PKCa	97.92	96.39	95.44	93.02	3.26E-10	STAUROSPORINE
SGK2	92.93	90.88	95.50	93.43	3.91E-08	STAUROSPORINE
CAMK4	96.32	95.88	94.78	94.31	3.76E-07	STAUROSPORINE
PLK4/SAK	95.85	95.23	96.65	92.58	3.43E-08	STAUROSPORINE
CDK1/cyclin B	97.20	94.08	95.86	93.52	4.18E-09	STAUROSPORINE
P38g	113.39	103.65	95.14	94.29	3.21E-07	STAUROSPORINE
MEKK2	104.16	99.16	95.03	94.66	4.05E-08	STAUROSPORINE
ERN2/IRE2	91.18	91.01	99.17	90.71	4.75E-08	STAUROSPORINE
MKK7	101.13	95.97	96.74	93.38	1.29E-06	STAUROSPORINE
SNRK	92.85	88.66	96.00	94.28	5.78E-08	STAUROSPORINE
TSSK3/STK22C	95.75	94.75	95.81	94.61	9.39E-09	STAUROSPORINE
CAMK1a	85.68	82.97	96.86	93.68	5.37E-09	STAUROSPORINE
KSR2	106.13	105.04	96.77	93.81	6.09E-06	STAUROSPORINE
STK21/CIT	102.47	100.13	96.09	95.46	4.31E-07	STAUROSPORINE
COT1/MAP3K8	89.62	88.42	97.66	93.96	6.87E-06	RO-31-8220
JNK1	102.45	102.15	100.97	90.83	6.26E-07	STAUROSPORINE
PKA	98.04	96.19	96.32	95.63	2.25E-09	STAUROSPORINE
JNK2	89.61	89.56	97.69	94.85	2.98E-06	STAUROSPORINE
MEK1	95.55	90.45	96.34	96.24	2.38E-08	STAUROSPORINE
DMPK	100.40	98.85	96.54	96.44	1.74E-08	STAUROSPORINE
GRK7	95.48	94.19	97.19	95.91	8.44E-09	STAUROSPORINE
CAMK2g	103.24	99.52	96.69	96.47	1.06E-09	STAUROSPORINE
CK1g2	108.32	105.03	96.98	96.34	4.10E-06	STAUROSPORINE
TLK2	97.72	96.06	99.31	94.18	5.82E-09	STAUROSPORINE
EPHB3	99.55	99.32	98.43	95.16	2.37E-06	STAUROSPORINE
LATS2	97.11	95.08	99.41	94.53	3.30E-09	STAUROSPORINE
MLK3/MAP3K11	119.92	115.91	105.16	88.78	2.89E-09	STAUROSPORINE
PLK3	99.91	99.40	97.79	96.39	6.14E-09	BI2536
HIPK4	96.58	95.87	97.57	96.65	1.14E-06	STAUROSPORINE
c-MET	105.59	98.69	97.51	97.07	1.73E-07	STAUROSPORINE
ROCK1	97.34	96.20	97.76	96.85	1.20E-09	STAUROSPORINE
CTK/MATK	96.62	94.43	99.43	95.33	2.46E-07	STAUROSPORINE
RSK2	101.34	99.53	98.81	95.96	2.67E-10	STAUROSPORINE
PKCepsilon	107.70	105.05	97.75	97.25	1.63E-10	STAUROSPORINE
JAK2	98.24	95.69	97.61	97.41	7.43E-10	STAUROSPORINE
P38d/MAPK13	100.32	100.06	98.49	96.54	2.53E-07	STAUROSPORINE
EPHA6	100.12	99.95	97.78	97.36	3.35E-08	STAUROSPORINE
CDK1/cyclin A	99.65	99.51	98.76	96.54	5.85E-09	STAUROSPORINE
MAK	101.94	100.71	98.16	97.18	3.76E-08	STAUROSPORINE
STK25/YSK1	95.66	90.65	99.60	96.00	3.08E-09	STAUROSPORINE
DRAK1/STK17A	110.56	109.14	98.11	97.51	4.97E-08	STAUROSPORINE
TAOK1	116.31	112.65	97.94	97.89	1.00E-09	STAUROSPORINE
CHK1	91.24	89.60	98.50	97.47	2.17E-10	STAUROSPORINE
MAPKAPK5/PRAK	126.00	117.26	98.98	97.51	1.00E-06	STAUROSPORINE
MLCK/MYLK	99.97	98.89	99.43	97.10	3.55E-08	STAUROSPORINE
CDK17/cyclin Y (PCTK2)	108.35	108.26	99.59	97.11	3.26E-08	STAUROSPORINE
GRK1	100.98	96.24	100.00	96.71	1.34E-07	STAUROSPORINE
PASK	101.54	99.76	98.73	98.26	1.51E-08	STAUROSPORINE
p70S6K/RPS6KB1	98.96	96.50	99.23	97.92	9.51E-10	STAUROSPORINE
PLK1	104.52	103.89	98.70	98.50	2.47E-07	STAUROSPORINE
NEK7	97.53	91.93	99.65	97.67	9.56E-06	PKR INHIBITOR
RIPK5	97.11	95.64	98.86	98.46	6.87E-08	STAUROSPORINE
CAMK2a	102.59	99.99	99.30	98.05	4.81E-11	STAUROSPORINE
MSSK1/STK23	103.62	102.53	99.23	98.68	1.53E-06	STAUROSPORINE
AKT2	88.52	87.90	100.30	97.64	2.94E-08	STAUROSPORINE
MEK5	108.13	102.80	101.69	96.71	7.87E-08	STAUROSPORINE
ZAP70	103.47	99.94	100.51	98.27	1.70E-08	STAUROSPORINE
MYLK3	108.94	107.60	100.81	98.10	1.10E-07	STAUROSPORINE
LCK2/ICK	102.50	100.99	100.48	98.72	4.90E-08	STAUROSPORINE
WNK2	98.73	97.94	101.20	98.10	5.64E-06	STAUROSPORINE
TLK1	107.56	105.51	102.78	96.55	5.08E-08	STAUROSPORINE
GRK5	97.53	88.18	105.66	93.71	9.48E-08	STAUROSPORINE
PAK6	103.80	101.62	100.63	98.95	5.87E-09	STAUROSPORINE
PBK/TOPK	109.50	101.81	100.81	98.77	1.55E-07	STAUROSPORINE
PIM2	109.10	107.97	102.27	97.39	7.12E-08	STAUROSPORINE
HIPK2	107.08	106.03	100.73	99.49	5.31E-07	STAUROSPORINE
TYK1/LTK	107.92	105.82	100.31	100.19	1.85E-08	STAUROSPORINE
JAK3	105.71	104.38	101.12	99.61	1.60E-10	STAUROSPORINE
PKCb1	107.28	105.23	100.54	100.39	3.32E-09	STAUROSPORINE
CAMK1d	101.35	100.57	100.60	100.40	4.94E-10	STAUROSPORINE
MAPKAPK3	110.50	110.07	103.16	97.90	4.30E-06	STAUROSPORINE
CDK18/cyclin Y (PCTK3)	114.82	114.72	101.71	99.62	5.41E-08	STAUROSPORINE
p70S6Kb/RPS6KB2	99.03	93.66	101.19	100.17	3.44E-09	STAUROSPORINE
P38b/MAPK11	106.73	101.00	101.50	100.14	3.64E-08	SB202190
FER	107.23	104.36	101.78	99.97	2.04E-09	STAUROSPORINE

DMPK2	111.95	101.12	101.31	100.56	6.50E-10	STAUROSPORINE
SRPK1	96.85	96.63	101.12	100.87	2.76E-08	STAUROSPORINE
ZIPK/DAPK3	99.36	97.65	101.57	100.47	2.12E-08	STAUROSPORINE
MSK1/RPS6KA5	109.18	105.72	102.48	99.69	1.36E-09	STAUROSPORINE
GSK3b	99.89	99.53	101.87	100.41	1.02E-08	STAUROSPORINE
TSSK2	99.18	94.08	102.41	100.02	1.51E-08	STAUROSPORINE
CK2a	102.23	100.75	101.60	101.10	9.25E-08	GW5074
CAMK1b	104.93	101.97	102.40	100.39	2.69E-08	STAUROSPORINE
ERK1	103.48	101.66	101.87	101.00	4.43E-09	SCH772984
DYRK4	97.01	94.88	104.56	98.36	2.47E-06	GW5074
NEK6	96.67	96.21	102.12	101.39	3.44E-05	PKR INHIBITOR
PKAcg	91.87	91.04	104.56	99.02	5.35E-09	STAUROSPORINE
ROS/ROS1	103.23	102.30	102.90	100.71	2.74E-10	STAUROSPORINE
PKCtheta	104.78	103.56	104.41	99.21	6.67E-09	STAUROSPORINE
PDK1/PDPK1	103.26	99.04	102.33	101.66	1.35E-09	STAUROSPORINE
TAOK3/JIK	98.17	97.31	103.15	101.09	5.98E-09	STAUROSPORINE
STK32B/YANK2	114.43	101.39	106.39	98.59	2.92E-08	STAUROSPORINE
ASK1/MAP3K5	99.66	97.36	102.69	102.30	2.78E-08	STAUROSPORINE
NIM1	110.16	108.70	102.87	102.12	2.68E-07	STAUROSPORINE
GRK3	104.81	100.42	103.72	101.87	1.82E-06	STAUROSPORINE
GSK3a	105.08	103.20	103.81	101.93	8.73E-09	STAUROSPORINE
PAK4	102.01	101.56	102.93	102.86	1.14E-09	STAUROSPORINE
GRK6	98.92	98.68	103.57	102.36	7.91E-08	STAUROSPORINE
JAK1	108.14	106.82	104.60	102.15	1.62E-09	STAUROSPORINE
PKAcb	104.34	103.81	103.93	103.08	3.70E-09	STAUROSPORINE
AKT3	102.60	101.90	104.01	103.09	9.56E-09	STAUROSPORINE
MEKK3	100.50	96.44	104.58	102.61	3.62E-08	STAUROSPORINE
IRAK4	113.83	113.31	105.93	101.32	1.05E-08	STAUROSPORINE
FAK/PTK2	90.37	87.01	104.05	103.27	2.45E-08	STAUROSPORINE
WNK3	100.89	100.25	106.05	101.78	1.19E-06	WEE-1 INHIBITOR
PKG2/PRKG2	106.99	99.05	104.90	103.01	2.84E-09	STAUROSPORINE
JNK3	102.36	99.17	105.32	102.65	2.37E-08	JNKI VIII
EPHA4	117.51	116.24	106.74	101.56	3.59E-08	STAUROSPORINE
MKK4	105.33	105.20	104.36	104.31	4.17E-06	STAUROSPORINE
ERBB2/HER2	82.46	80.93	104.79	104.07	1.25E-07	STAUROSPORINE
IKKb/IKBKB	102.96	102.51	106.02	103.16	8.68E-07	STAUROSPORINE
MSK2/RPS6KA4	99.20	98.92	105.12	104.43	6.38E-09	STAUROSPORINE
MST2/STK3	107.49	98.74	106.88	102.68	5.86E-09	STAUROSPORINE
MRCKa/CDC42BPA	104.56	104.20	104.92	104.83	9.27E-09	STAUROSPORINE
HIPK3	102.16	97.92	105.85	104.35	1.49E-06	STAUROSPORINE
RIPK4	98.91	98.29	107.31	103.33	5.01E-07	STAUROSPORINE
STK32C/YANK3	118.90	114.76	105.93	104.80	2.22E-07	STAUROSPORINE
PAK5	106.89	104.90	106.08	104.88	9.35E-09	STAUROSPORINE
SSTK/TSSK6	93.45	92.60	106.10	105.69	5.56E-07	STAUROSPORINE
RON/MST1R	99.79	89.96	108.29	103.58	3.67E-07	STAUROSPORINE
STK38L/NDR2	108.43	105.84	106.22	105.79	2.51E-09	STAUROSPORINE
ERK5/MAPK7	104.29	103.40	106.48	105.68	3.55E-05	STAUROSPORINE
ULK3	106.18	105.43	106.70	106.21	1.13E-08	STAUROSPORINE
CLK3	120.97	112.55	109.63	103.50	3.20E-06	STAUROSPORINE
MKK6	97.89	95.10	108.11	105.09	9.17E-09	STAUROSPORINE
PLK2	116.58	106.96	106.80	106.46	5.54E-07	STAUROSPORINE
PAK2	108.99	108.11	109.07	105.00	2.82E-09	STAUROSPORINE
PYK2	107.62	104.84	107.99	106.46	1.05E-08	STAUROSPORINE
GRK4	102.39	102.12	109.85	104.73	6.75E-08	STAUROSPORINE
TTBK2	110.86	104.35	108.10	106.48	4.47E-06	SB202190
AKT1	102.35	102.34	108.85	105.99	7.96E-09	STAUROSPORINE
SGK1	108.14	106.89	110.95	104.69	2.55E-08	STAUROSPORINE
MLK4	105.60	101.72	108.75	107.14	4.18E-06	STAUROSPORINE
MST4	101.65	101.20	112.09	108.77	5.08E-09	STAUROSPORINE
OSR1/OXSR1	100.95	94.64	112.65	108.56	1.25E-07	STAUROSPORINE
STK38/NDR1	106.69	104.52	110.74	110.61	1.65E-09	STAUROSPORINE
GRK2	106.34	105.44	111.40	110.16	1.94E-06	STAUROSPORINE
MRCKb/CDC42BPB	108.03	106.11	113.28	110.62	7.29E-09	STAUROSPORINE
PKG1a	110.05	109.32	126.81	97.28	1.94E-09	STAUROSPORINE
MEK3	113.10	104.98	114.01	111.17	6.67E-08	STAUROSPORINE
NEK9	119.23	116.60	113.92	113.10	2.60E-07	STAUROSPORINE
HIPK1	121.24	120.32	117.74	112.79	3.39E-06	RO-31-8220
NEK2	110.96	110.33	116.60	115.00	3.28E-07	STAUROSPORINE
SRMS	102.00	98.62	123.18	122.09	3.25E-05	STAUROSPORINE
TTBK1	107.67	105.19	126.78	120.68	1.86E-05	SB202190

2. Crystallization of MU1700-ALK2 complex and M4K2234-ALK2 complex.

Protein expression and purification.

Full length human FKBP12 was cloned into the bacterial expression vector pGTVL2, which provides N-terminal GST and hexahistidine tags as well as a Tobacco Etch Virus (TEV) protease cleavage site. The FKBP12 plasmid was transformed into *Escherichia coli* strain BL21(DE3)R3-pRARE2. Cultures in LB media were induced with 0.5 mM isopropyl 1-thio-β-d-gasalactopyranoside overnight at 18°C, pelleted by centrifugation and stored at -20 °C. ALK2 constructs were cloned into the baculoviral expression vector pFB-LIC-Bse, which provides an N-terminal hexahistidine tag and TEV cleavage site as previously described.[1] The structure of the MU1700 complex used the GS and kinase domains of ALK2 spanning residues 172–499, whereas the structure of the M4K2234 complex used a construct containing the kinase domain of ALK2, including residues 201–499 and the activating mutation Q207D. ALK2 constructs were expressed in Sf9 insect cells at 27 °C with shaking at 110 rpm. Cells were harvested 72 h after infection, pelleted with centrifugation and stored at -20 °C.

Cells were lysed by ultrasonication with a lysis buffer (50 mM HEPES pH 7.5, 500 mM NaCl, 5mM imidazole, 5% glycerol) supplemented with 1 mM Tris(2-carboxyethyl)phosphine (TCEP) and protease inhibitor. Polyethylenimine (0.15%) was added to precipitate DNA. Insoluble material was excluded by centrifugation and the recombinant proteins were purified from the clear supernatant by nickel affinity chromatography. Relevant eluted proteins were subsequently cleaved overnight at 4 °C with TEV protease. Individual proteins were further purified by size-exclusion chromatography using a S75 HiLoad 16/60 Superdex column equilibrated in 50 mM HEPES pH 7.5, 300 mM NaCl, 0.5 mM TCEP. For the structure of the MU1700 complex, the ALK2 protein containing the GS and kinase domains was mixed with an excess of FKBP12 and a final clean up step for their complex was performed using the same size-exclusion chromatography procedure. Purity of all proteins was assessed by SDS-PAGE and the molecular weight verified by mass spectrometry analysis.

Crystallization.

The ALK2-FKBP12 complex was preincubated with 1 mM MU1700 at a protein concentration of 10 mg/mL. Crystallization was achieved using the sitting drop vapor diffusion method at 4 °C, with a reservoir solution containing 0.2 M sodium fluoride, 0.1 M bis-tris-propane pH 7.5, 10% ethylene glycol, 20% PEG3350. Viable crystals were obtained when the protein solution was mixed with the reservoir solution at 1:1 volume ratio. Crystals were cryoprotected with mother liquor solution supplemented with 25% ethylene glycol, prior to vitrification in liquid nitrogen.

For the M4K2334 complex, ALK2 was preincubated with 0.5 mM M4K2344 at a protein concentration of 12.5 mg/mL. Crystallization in sitting drops was achieved at 20 °C, with a reservoir solution containing 5% PEG1000, 40% ethanol, 0.1 M citrate pH 4.2. Viable crystals were obtained

when the protein solution was mixed with the reservoir solution at 1:2 volume ratio. Crystals were cryoprotected with mother liquor solution supplemented with 25% ethylene glycol, prior to vitrification in liquid nitrogen.

Data collection, phasing and refinement.

Diffraction data for the MU1700 complex were collected at Diamond Light Source, beamline I03 at a temperature of 100 K using monochromatic radiation at a wavelength of 0.92272 Å. Data were processed with Xia2 dials and subsequently scaled using the program AIMLESS from the CCP4 suite.[2] The structure was solved by molecular replacement using PHASER[3] and the coordinates of an ALK2-FKBP12 complex (PDB code 3H9R) as a search model. Atomic models were refined using phenix.refine[4] and manually improved using COOT.[5] Stereochemical validation of the final models were performed with MolProbity.[6] Data collection and refinement statistics are summarized in the Supporting Information, Table S2. The model was deposited in the PDB with accession code 8POD.

Diffraction data for the M4K2234 complex were collected at Diamond Light Source, beamline I03 at a temperature of 100 K using monochromatic radiation at a wavelength of 0.97950 Å. Data were processed with autoPROC dials and subsequently scaled using the program AIMLESS from the CCP4 suite.[7] The structure was solved by molecular replacement using PHASER[3] and trimmed coordinates of an ALK2-FKBP12 complex (PDB code 3H9R) as a search model with FKBP12 removed. Atomic models were refined using phenix.refine[4] and manually improved using COOT.[5] Stereochemical validation of the final models were performed with MolProbity.[6] Data collection and refinement statistics are summarized in the Table S2. The model was deposited in the PDB with accession code 8R7G.

Table S2: Diffraction data and refinement statistics.

**Data Collection Statistics**	**MU1700**	**M4K2234**
Radiation source	Diamond I03	Diamond I03
Wavelength (Å)	0.92272	0.97950
Spacegroup	$P2_12_12_1$	$P12_11$
Cell dimensions:		
a, *b*, *c* (Å)	43.34, 76.62, 138.54	82.82, 100.59, 83.58
α, *β*, *γ* (°)	90, 90, 90	90, 118.3, 90
Number of molecules/asymmetric unit	1	4
Resolution range (Å)	51.39-2.59 (2.63-2.59)	73.71-2.09 (2.11-2.09)
Total observations	200622 (10382)	504723 (25453)
Unique reflections	14991 (724)	71938 (3565)
Completeness (%)	99.91 (99.86)	100.00 (100.00)
Multiplicity	13.38 (14.34)	7.00 (7.10)
$R_{merge}{}^{a}$	0.4598 (2.1013)	0.0940 (2.9390)
Average I/σ *(I)*	3.09 (0.50)	11.50 (0.70)

$CC_{1/2}$ (%)	97.14 (59.12)	99.90 (34.70)
**Refinement and model statistics**		
Resolution range (Å)	51.39-2.59	73.71-2.09
Number of reflections used	14936	71733
$R_{work}^{b}/R_{free}^{c}$ (%)	23.53/25.53	22.97/25.33
Ligand	MU1700	M4K2234
Average B values (Å^2)		
All atoms	43.407	60.522
Protein atoms	43.688	60.682
Ligand	33.964	54.844
Fluoride	19.720	-
H_2O	35.637	53.959
Root mean square deviation from ideality		
Bond lenghts (Å)	0.002	0.005
Bond angles (°)	0.46	0.78
Ramachandran analysis		
Favored / Allowed / Outliers (% residues)	97.86/2.14/0.00	97.45/2.46/0.09
Rotamer outliers (%)	0.92	1.03
Clashscore	3.48	4.80
Number of atoms		
Protein atoms	3233	8782
Ligand	31	136
Fluoride	1	-
H_2O	76	97
**PDB Code**	8POD	8R7G

[a] $R_{merge} = \Sigma\Sigma|I_{h,i} - \langle I\rangle_h|_{ih}\Sigma\Sigma I_{h,iih}/$ where $\langle I\rangle_h$ is the mean intensity of the symmetry-equivalent reflections.

[b] $R_{work} = \Sigma||F_o| - |F_c||_h\Sigma|F_o|_h/$,where F_o and F_c are the observed and calculated structure factor amplitudes, respectively, for reflection h.

[c] R_{free} is the R value for a subset of 5% of the reflection data, which were not included in the crystallographic refinement.

3. Kinome-wide profiling of M4K2234 (Reaction Biology)

Table S3: Data from kinome-wide profiling of M4K2234 against the indicated kinases at 1 μM concentration (radiometric assays, Reaction Biology). The compound was tested in single dose duplicate mode at a concentration of 1 μM. The control compound staurosporine was tested in 10-dose IC_{50} mode with 3 or 4-fold serial dilution starting at 20 or 100 μM. Other control compounds were tested in 10-dose IC_{50} mode with 3 or 4-fold serial dilution starting at 10, 20, 50 or 100 μM. The reactions were carried out at 10 μM ATP.

**Kinase**	**M4K2234 (1uM)**				**M4K2234 (1uM)**				**M4K2234 (1uM)**		
	**Data 1**	**Data 2**	**Average**	**Kinase**	**Data 1**	**Data 2**	**Average**	**Kinase**	**Data 1**	**Data 2**	**Average**
TNIK	5.27	4.57	4.92	DMPK2	87.02	85.28	86.15	MUSK	90.30	88.04	89.17
ALK1/ACVRL1	2.90	2.55	2.73	PKCtheta	102.63	95.99	99.31	P38a/MAPK14	99.75	99.05	99.40
ALK2/ACVR1	7.69	5.67	6.68	MARK3	93.00	91.46	92.23	MARK2/PAR-1Ba	93.74	92.98	93.36
ALK6/BMPR1B	27.92	27.02	27.47	CAMK2b	89.04	86.76	87.90	P38b/MAPK11	101.90	99.99	100.95
NLK	44.71	44.67	44.69	TYRO3/SKY	97.39	95.17	96.28	ERN1/IRE1	98.26	96.56	97.41
HGK/MAP4K4	29.70	29.61	29.66	PKCb1	87.68	86.84	87.26	SNARK/NUAK2	93.54	92.65	93.10
MINK/MINK1	37.11	36.56	36.84	ULK1	86.28	82.29	84.29	JNK2	91.34	90.94	91.14
BRAF	44.45	44.03	44.24	CDK8/cyclin C	90.11	89.57	89.84	DLK/MAP3K12	94.50	94.38	94.44
ARAF	52.62	51.73	52.18	PHKg1	96.68	92.81	94.75	HCK	95.09	93.44	94.27
ZAK/MLTK	38.53	36.39	37.46	BRSK1	87.89	84.48	86.19	JAK2	95.80	92.56	94.18
SIK2	36.68	35.99	36.34	CAMK2a	89.93	89.17	89.55	LCK	96.99	96.67	96.83
DDR1	40.20	39.34	39.77	MSK1/RPS6KA5	99.70	96.47	98.09	MKK7	100.41	98.52	99.47
ALK3/BMPR1A	39.92	39.34	39.63	PYK2	83.76	81.78	82.77	PDK1/PDPK1	96.26	93.89	95.08
KHS/MAP4K5	42.81	39.41	41.11	STK32C/YANK3	92.80	90.70	91.75	TBK1	87.00	83.46	85.23
DYRK1B	53.64	52.79	53.22	NEK1	100.04	93.20	96.62	CDK16/cyclin Y	93.46	91.35	92.41
DYRK1/DYRK1A	57.20	56.39	56.80	MST4	90.69	90.53	90.61	CDK5/P25	93.58	93.23	93.41
MNK2	68.96	63.87	66.42	ERK7/MAPK15	90.48	89.98	90.23	PKN2/PRK2	102.96	100.36	101.66
MNK1	60.95	59.43	60.19	CDK6/cyclin D3	89.55	88.85	89.20	TIE2/TEK	93.07	91.97	92.52
LOK/STK10	61.59	61.54	61.57	MLK2/MAP3K10	115.37	106.44	110.91	CAMKK1	92.70	88.83	90.77
CK1g1	61.14	60.03	60.59	MEK5	96.31	93.44	94.88	OSR1/OXSR1	96.08	95.05	95.57
HPK1/MAP4K1	56.39	55.64	56.02	MEK2	91.30	89.42	90.36	EPHB2	96.21	95.97	96.09
BRK	65.32	64.33	64.83	STK21/CIT	102.54	99.05	100.80	CK1epsilon	95.99	91.70	93.85
SIK3	72.61	71.66	72.14	GSK3b	99.45	96.28	97.87	ROCK1	96.97	96.20	96.59
LRRK2	74.06	73.65	73.86	ROS/ROS1	95.56	95.52	95.54	CAMK1a	94.69	91.83	93.26
TAOK1	38.45	36.37	37.41	CLK4	94.89	93.34	94.12	EPHA4	101.47	99.89	100.68
GCK/MAP4K2	68.25	67.48	67.87	STK32B/YANK2	86.51	82.92	84.72	GRK1	95.38	93.08	94.23
BRSK2	73.81	71.41	72.61	NEK3	96.09	95.23	95.66	MEKK1	89.43	87.93	88.68
DCAMKL2	70.71	69.99	70.35	FER	83.86	83.48	83.67	PASK	90.71	89.01	89.86
LIMK1	77.94	76.31	77.13	MARK1	92.51	91.67	92.09	CDK1/cyclin A	84.48	83.71	84.10
CK1G3	71.45	69.81	70.63	TAOK2/TAO1	82.27	81.16	81.72	PAK5	96.74	96.36	96.55
ACK1	66.77	61.59	64.18	NEK11	92.05	89.23	90.64	CK1a1	99.28	99.26	99.27
MLCK2/MYLK2	82.26	81.15	81.71	VRK2	88.02	85.61	86.82	PLK4/SAK	94.00	93.89	93.95
RIPK2	80.92	79.92	80.42	GLK/MAP4K3	83.93	83.55	83.74	EPHB1	91.48	91.06	91.27
ALK4/ACVR1B	81.34	79.81	80.58	PAK3	90.17	89.47	89.82	CK2a2	98.13	91.24	94.69
TAOK3/JIK	68.90	65.47	67.19	BMPR2	94.33	94.05	94.19	TESK2	93.29	93.23	93.26
CAMKK2	79.40	79.29	79.35	CDK3/cyclin E2	85.07	84.43	84.75	ARK5/NUAK1	92.50	92.31	92.41
RIPK4	79.68	77.02	78.35	WNK3	92.11	90.95	91.53	CDK1/cyclin B	90.35	89.90	90.13
ULK2	81.06	80.11	80.59	STK39/STLK3	97.61	94.07	95.84	PKAcg	91.97	88.31	90.14
c-Kit	80.81	76.90	78.86	MST3/STK24	113.21	107.63	110.42	EPHB4	91.05	85.87	88.46
ABL1	84.48	80.78	82.63	PKCmu/PRKD1	93.33	90.68	92.01	MAST3	97.45	97.29	97.37
FLT3	90.68	85.34	88.01	IKKe/IKBKE	92.20	82.11	87.16	PHKg2	95.85	95.06	95.46
PDGFRb	90.14	88.87	89.51	DYRK3	93.39	92.56	92.98	KSR1	93.57	92.67	93.12
CK1g2	73.25	70.17	71.71	ERBB2/HER2	87.21	85.10	86.16	SBK1	96.31	96.20	96.26
CDK19/cyclin C	82.42	82.07	82.25	COT1/MAP3K8	94.82	93.40	94.11	SGK1	91.09	90.95	91.02
RAF1	85.03	84.08	84.56	JNK1	86.44	83.52	84.98	GRK3	97.93	96.85	97.39
FGR	93.41	91.32	92.37	PKCa	88.30	85.60	86.95	FAK/PTK2	96.05	87.69	91.87
FMS	49.28	48.82	49.05	STK33	97.29	96.42	96.86	PKN3/PRK3	100.61	98.77	99.69
LYN	84.03	82.25	83.14	RSK3	101.40	99.21	100.31	PLK3	96.05	93.88	94.97
GRK4	91.85	90.10	90.98	PKCzeta	92.41	91.33	91.87	RET	92.55	89.18	90.87
LKB1	92.49	92.06	92.28	PBK/TOPK	95.05	94.31	94.68	PKCeta	96.80	96.60	96.70
IRAK4	88.83	88.31	88.57	CDK9/cyclin T1	93.52	92.35	92.94	KDR/VEGFR2	97.27	95.43	96.35
MYO3b	84.96	84.87	84.92	TRKA	91.94	89.07	90.51	CDK2/cyclin O	92.35	92.30	92.33
DCAMKL1	84.74	83.59	84.17	STK38/NDR1	91.48	88.77	90.13	GRK7	100.81	100.06	100.44
AXL	81.11	81.04	81.08	EPHA5	94.67	94.61	94.64	MRCKb/CDC42BPB	97.02	95.88	96.45
PDGFRa	80.05	79.03	79.54	NEK2	93.02	91.46	92.24	ERK5/MAPK7	99.60	98.66	99.13
CDK3/cyclin E	85.83	85.35	85.59	ULK3	94.55	93.28	93.92	MST2/STK3	98.35	93.52	95.94
ALK5/TGFBR1	84.09	83.42	83.76	NEK9	87.78	82.66	85.22	BTK	97.71	97.62	97.67
MKK6	68.55	64.71	66.63	CLK2	88.31	87.08	87.70	AKT3	98.54	97.15	97.85
RIPK5	84.75	83.86	84.31	PLK1	98.82	97.79	98.31	ASK1/MAP3K5	101.03	99.17	100.10
PKCnu/PRKD3	144.24	142.24	143.24	AKT2	94.01	93.58	93.80	EPHA8	102.03	95.79	98.91
SIK1	88.51	87.47	87.99	CK2a	88.72	85.73	87.23	CAMK2d	93.56	93.45	93.51

MYO3A	99.37	95.48	97.43	MAPKAPK5/PRAK	96.08	95.81	95.95	JNK3	98.83	95.11	96.97
IKKa/CHUK	85.62	84.25	84.94	TYK2	103.25	100.78	102.02	CTK/MATK	95.25	94.38	94.82
Haspin	84.75	83.53	84.14	CDC7/DBF4	98.14	94.08	96.11	KSR2	97.23	96.38	96.81
MARK4	83.45	83.34	83.40	ERK2/MAPK1	89.35	88.25	88.80	DAPK1	98.50	98.30	98.40
CDK7/cyclin H	103.42	101.47	102.45	PKCepsilon	97.83	97.53	97.68	SLK/STK2	105.63	104.60	105.12
EPHA3	101.11	100.47	100.79	NEK8	101.75	100.17	100.96	AURORA B	112.26	110.21	111.24
MLK4	91.19	89.41	90.30	CDK14/cyclin Y	97.79	97.41	97.60	IR	101.24	100.67	100.96
MEKK2	82.92	81.13	82.03	PEAK1	112.02	110.20	111.11	CAMK2g	96.32	96.27	96.30
WNK2	98.86	98.34	98.60	CAMK1d	100.93	98.72	99.83	MEK3	104.87	103.04	103.96
EPHB3	94.79	92.25	93.52	PAK4	103.71	103.30	103.51	TRKC	118.59	117.99	118.29
STK25/YSK1	92.82	91.63	92.23	DDR2	92.60	91.94	92.27	IRAK1	100.81	100.16	100.49
FGFR2	97.84	97.43	97.64	PKCd	93.40	92.22	92.81	MAPKAPK2	99.92	98.48	99.20
ZAP70	99.62	98.79	99.21	RSK1	97.01	95.84	96.43	MRCKa/CDC42BPA	104.45	102.98	103.72
PKAcb	99.42	98.30	98.86	MAK	111.75	110.09	110.92	DAPK2	104.25	103.70	103.98
DYRK2	97.52	95.78	96.65	IGF1R	100.72	99.60	100.16	VRK1	108.53	108.29	108.41
CDK5/p35	95.63	95.08	95.36	FYN	121.72	119.07	120.40	TNK1	108.03	102.51	105.27
MEKK3	99.81	92.94	96.38	CDK4/cyclin D3	93.74	93.67	93.71	BMX/ETK	102.78	102.35	102.57
CDK17/cyclin Y	94.88	93.99	94.44	HIPK2	101.68	96.91	99.30	DYRK4	97.15	96.54	96.85
RSK4	97.30	94.31	95.81	CDK4/cyclin D1	100.09	99.89	99.99	IKKb/IKBKB	108.96	108.24	108.60
SRMS	98.82	97.08	97.95	MASTL	94.94	93.05	94.00	HIPK4	104.07	103.79	103.93
EGFR	101.33	101.05	101.19	Aurora A	93.85	91.68	92.77	PAK1	103.65	103.63	103.64
TTBK2	97.99	94.77	96.38	CDK2/cyclin A	92.33	92.29	92.31	ZIPK/DAPK3	108.04	105.01	106.53
PKD2/PRKD2	105.17	102.86	104.02	PIM1	105.63	103.63	104.63	PKCiota	100.39	95.18	97.79
NIM1	94.49	93.25	93.87	MSSK1/STK23	98.18	91.96	95.07	RIPK3	112.17	111.71	111.94
SRPK2	97.17	95.75	96.46	MELK	99.63	97.92	98.78	AKT1	101.90	99.01	100.46
DRAK1/STK17A	97.03	96.54	96.79	STK38L/NDR2	95.32	93.58	94.45	IRR/INSRR	123.57	121.01	122.29
ERK1	98.69	97.39	98.04	NEK6	100.84	100.11	100.48	Aurora C	100.58	98.40	99.49
TLK2	95.15	94.99	95.07	CDK6/cyclin D1	101.19	100.82	101.01	EPHA6	103.23	101.59	102.41
CLK1	93.61	92.82	93.22	MLK3/MAP3K11	106.15	101.95	104.05	GRK2	106.03	105.76	105.90
CK1d	100.50	99.03	99.77	c-MET	98.44	98.41	98.43	HIPK1	100.16	99.62	99.89
CHK1	106.02	105.34	105.68	MAPKAPK3	99.55	97.50	98.53	WEE1	128.65	120.95	124.80
TSSK3/STK22C	95.08	94.89	94.99	MYLK3	102.98	101.18	102.08	JAK3	102.64	101.59	102.12
c-Src	98.33	98.08	98.21	TYK1/LTK	100.49	99.87	100.18	ITK	99.45	95.85	97.65
ABL2/ARG	98.76	98.48	98.62	ERBB4/HER4	100.83	100.25	100.54	CDK2/cyclin E	106.40	104.69	105.55
BLK	101.20	100.60	100.90	P38d/MAPK13	103.50	101.10	102.30	YES/YES1	100.50	98.27	99.39
PKCg	98.34	97.90	98.12	CSK	94.12	92.13	93.13	SNRK	113.18	107.97	110.58
MYLK4	95.87	95.46	95.67	CAMK4	103.80	101.77	102.79	LATS2	110.37	109.35	109.86
CAMK1b	100.78	99.74	100.26	TSSK2	107.44	102.75	105.10	STK16	102.11	99.20	100.66
MSK2/RPS6KA4	96.00	95.45	95.73	CDK9/cyclin T2	102.05	102.02	102.04	LCK2/ICK	89.44	86.13	87.79
EPHA1	97.99	97.40	97.70	CHK2	95.42	95.41	95.42	MLCK/MYLK	106.35	106.15	106.25
FGFR1	101.69	101.63	101.66	FES/FPS	97.02	96.09	96.56	FLT1/VEGFR1	98.17	95.86	97.02
TTBK1	96.89	93.79	95.34	PIM3	107.18	106.04	106.61	PKCb2	104.64	103.20	103.92
NEK7	96.75	93.87	95.31	PRKX	96.97	96.87	96.92	CDK2/cyclin E2	104.97	104.46	104.72
p70S6Kb/RPS6KB2	102.82	98.03	100.43	PAK6	98.78	98.70	98.74	WNK1	101.95	100.13	101.04
CDK9/cyclin K	105.53	104.69	105.11	ALK	103.35	102.24	102.80	TEC	127.66	124.33	126.00
STK22D/TSSK1	101.05	99.56	100.31	EPHA7	102.78	102.43	102.61	GRK6	104.08	103.22	103.65
p70S6K/RPS6KB1	97.82	94.87	96.35	CDK2/Cyclin A1	111.93	111.68	111.81	TLK1	111.42	109.03	110.23
LATS1	101.28	99.48	100.38	MKK4	94.33	94.30	94.32	P38g	94.38	90.04	92.21
PIM2	98.05	96.36	97.21	c-MER	96.98	95.70	96.34	SRPK1	104.82	102.86	103.84
ERN2/IRE2	104.16	103.94	104.05	EPHA2	102.33	98.97	100.65	DMPK	111.79	110.69	111.24
PKA	97.99	97.30	97.65	TGFBR2	111.40	110.01	110.71	SSTK/TSSK6	110.28	107.83	109.06
MEK1	103.50	102.97	103.24	PKG1b	99.52	98.95	99.24	MLK1/MAP3K9	103.62	102.91	103.27
MEKK6	95.17	89.86	92.52	TRKB	109.41	105.99	107.70	TESK1	108.31	108.28	108.30
PKN1/PRK1	94.64	94.01	94.33	RSK2	97.43	97.24	97.34	ROCK2	121.91	119.86	120.89
CDK1/cyclin E	100.40	99.30	99.85	PAK2	106.75	104.17	105.46	SGK2	95.78	94.93	95.36
YSK4/MAP3K19	105.08	101.54	103.31	PKMYT1	124.81	121.94	123.38	GRK5	99.91	97.81	98.86
HIPK3	87.90	85.10	86.50	SYK	104.83	99.79	102.31	CDK18/cyclin Y	101.52	100.66	101.09
PKG1a	103.94	103.73	103.84	CAMK1g	98.99	97.85	98.42	FLT4/VEGFR3	96.83	96.06	96.45
RON/MST1R	113.67	108.77	111.22	MST1/STK4	100.28	99.22	99.75	NEK5	105.78	96.39	101.09
FGFR4	95.18	94.51	94.85	LIMK2	105.28	102.22	103.75	FRK/PTK5	100.39	99.17	99.78
LYN B	95.08	91.23	93.16	JAK1	96.67	96.26	96.47	GSK3a	102.65	100.51	101.58
CK1a1L	103.89	102.82	103.36	FGFR3	104.85	104.10	104.48	NEK4	100.85	99.62	100.24
TAK1	98.69	97.41	98.05	TXK	103.85	100.24	102.05				
PLK2	93.59	92.87	93.23	SGK3/SGKL	86.00	83.45	84.73				
PKG2/PRKG2	95.65	91.81	93.73	CLK3	92.75	91.99	92.37				

4. Biochemical profiling of M4K2234

M4K2234 was tested against 6 kinases. Compound M4K2234 was received as powder and dissolved to 10 mM in DMSO. Compound was tested in 10-dose IC50 mode with a 3-fold serial dilution starting at 3 µM. Control compound LDN193189 was tested in 10-dose IC50 mode with 3-fold serial dilution starting at 10 µM. Reactions were carried out at 10 µM ATP. Enzyme activity (relative to DMSO controls) and IC50 values were obtained from curve fits.

*Curve fits were performed where the enzyme activities at the highest concentration of compounds were less than 65%

Table S4: IC_{50} Summary of M4K2234 against ALK1-6

	**Compound IC50* (M):**	**IC50 (M) Control Cmpd**	**Control Cmpd ID**
**Kinase:**	**M4K2234**		
**ALK1/ACVRL1**	**6,57E-09**	**2,09E-08**	**LDN193189**
**ALK2/ACVR1**	**1,41E-08**	**3,88E-08**	**LDN193189**
**ALK3/BMPR1A**	**1,68E-07**	**1,16E-08**	**LDN193189**
**ALK4/ACVR1B**	**1,66E-06**	**1,91E-07**	**LDN193189**
**ALK5/TGFBR1**	**1,95E-06**	**5,89E-07**	**LDN193189**
**ALK6/BMPR1B**	**8,78E-08**	**1,18E-08**	**LDN193189**

**ALK1/ACVRL1 (%Activity)**		**ALK2/ACVR1 (%Activity)**		**ALK3/BMPR1A (%Activity)**	
**Conc.(M)**	**M4K2234**	**Conc.(M)**	**M4K2234**	**Conc.(M)**	**M4K2234**
**3,00E-06**	3,80	**3,00E-06**	0,75	**3,00E-06**	25,13
**1,00E-06**	3,74	**1,00E-06**	1,09	**1,00E-06**	31,12
**3,33E-07**	7,33	**3,33E-07**	2,67	**3,33E-07**	38,56
**1,11E-07**	9,70	**1,11E-07**	5,73	**1,11E-07**	47,37
**3,70E-08**	20,03	**3,70E-08**	15,86	**3,70E-08**	57,56
**1,23E-08**	38,72	**1,23E-08**	51,23	**1,23E-08**	72,49
**4,12E-09**	65,26	**4,12E-09**	90,82	**4,12E-09**	84,63
**1,37E-09**	98,91	**1,37E-09**	98,89	**1,37E-09**	77,16
**4,57E-10**	120,05	**4,57E-10**	93,75	**4,57E-10**	76,49
**1,52E-10**	112,43	**1,52E-10**	94,76	**1,52E-10**	77,08
**DMSO**	107,05	**DMSO**	95,10	**DMSO**	99,28
**HILLSLOPE**	**-1,03**	**HILLSLOPE**	**-1,82**	**HILLSLOPE**	**-0,37**
**IC50 (M)**	**6,57E-09**	**IC50 (M)**	**1,41E-08**	**IC50 (M)**	**1,68E-07**

**ALK4/ACVR1B (%Activity)**		**ALK5/TGFBR1 (%Activity)**		**ALK6/BMPR1B (%Activity)**	
**Conc.(M)**	**M4K2234**	**Conc.(M)**	**M4K2234**	**Conc.(M)**	**M4K2234**
**3,00E-06**	32,64	**3,00E-06**	38,34	**3,00E-06**	6,35
**1,00E-06**	68,23	**1,00E-06**	71,51	**1,00E-06**	14,73
**3,33E-07**	94,82	**3,33E-07**	99,60	**3,33E-07**	29,70
**1,11E-07**	96,72	**1,11E-07**	102,75	**1,11E-07**	44,25
**3,70E-08**	92,59	**3,70E-08**	103,75	**3,70E-08**	63,65
**1,23E-08**	97,43	**1,23E-08**	111,42	**1,23E-08**	83,37
**4,12E-09**	96,84	**4,12E-09**	105,62	**4,12E-09**	99,37
**1,37E-09**	107,56	**1,37E-09**	100,10	**1,37E-09**	100,94
**4,57E-10**	118,69	**4,57E-10**	104,55	**4,57E-10**	85,63

**1,52E-10**	109,95	**1,52E-10**	102,06	**1,52E-10**	98,72
**DMSO**	103,30	**DMSO**	98,71	**DMSO**	108,09
**HILLSLOPE**	**-1,24**	**HILLSLOPE**	**-1,39**	**HILLSLOPE**	**-0,75**
**IC50 (M)**	**1,66E-06**	**IC50 (M)**	**1,95E-06**	**IC50 (M)**	**8,78E-08**

5. Profiling MU1700 and M4K2234 in the NanoBRET™ target engagement assay in intact cells

ALK1-6

ALK1-6-nanoluciferase fusion constructs were mixed 1:9 with Transfection Carrier DNA (mass/mass). Transfected cells were trypsinized and resuspended in Opti-MEM at a density of 200,000 cells per mL. Cells (17 μL) were dispensed into each well of a Corning® NBS™ 384 well microplate. Working solutions (20 ×) of target engagement tracers were prepared by diluting DMSO stock in tracer dilution buffer (12.5 mM HEPES pH 7.5, 31.25% PEG-400). ALK1 assays used 250 nM ALK family tracer (gift from Promega; PBI-7428), ALK2 assays used 125 nM K11 tracer; ALK3 assays used 250 nM ALK family tracer (gift from Promega; PBI-7428); ALK4 assays used 500 nM K14 tracer; ALK5 assays used 500 nM K14 tracer; ALK6 assays used 500 nM SGC-1 tracer. Stocks (1000 ×) of test compounds in DMSO were diluted further in Opti-MEM for 10 × working solutions. After the addition of 1 μL of 20 × target engagement tracer and 2 μL of 10 × working solutions, contents of the wells were thoroughly mixed by agitating the plate at 500 rpm for 1 min. Cells were incubated in a humidified, 37 °C incubator with 5% CO_2 for 2 h prior to bioluminescence resonance energy transfer (BRET) measurement. For BRET measurement, the NanoBRET NanoGlo Substrate and Extracellular NanoLuc Inhibitor were diluted 166 × and 500 ×, respectively, in Opti-MEM to produce 3 × working stock. A PHERAstar FSX microplate reader with the LUM 610-LP 460-80 optical module was used to measure the intensity of dual emission. A measurement interval of 1 s and gain settings of 3600 and 1879 for 610 and 460 nm, respectively, were used. Milli-BRET units (mBU) were calculated by dividing the signal measured at 610 nm with the signal measured at 460 nm and multiplying by 1000. The apparent EC_{50} values of test compounds were estimated using the [Inhibitor] *versus* response (three parameter) nonlinear regression curve fitting function of GraphPad Prism.

DDR1, FLT3, KHS/MAP4K5

The assay was performed as described previously.[8] In brief: full-length kinases were obtained as plasmids cloned in frame with a terminal NanoLuc-fusion (Table S5). Plasmids were transfected into HEK293T cells using FuGENE HD, and proteins were allowed to express for 20 h. Serially diluted inhibitor and NanoBRET™ Kinase Tracer (Table S5) at a concentration determined previously as the Tracer EC_{50} (Table S5) were pipetted into white 384-well plates using an Echo 550 acoustic dispenser. Corresponding protein-transfected cells were added and reseeded at a density of 2 x 10^5 cells/mL after trypsinization and resuspending in Opti-MEM without phenol red. The system was allowed to equilibrate for 2 h at 37 °C/5% CO_2 prior to BRET measurements. To measure BRET, NanoBRET™ NanoGlo Substrate + Extracellular NanoLuc Inhibitor was added as per the manufacturer's protocol, and filtered luminescence was measured on a PHERAstar FSX plate reader equipped with a luminescence filter pair (450 nm BP filter (donor) and 610 nm LP filter (acceptor)). For

permeabilized-mode NanoBRET experiments, digitonin was added as per the manufacturer's instructions to a final concentration of 50 ng/mL. Competitive displacement data were then graphed using GraphPad Prism 10 software using a normalized 3-parameter curve fit with the following equation: Y=100/(1+10^(X-LogIC50)).

Table S5: Vectors and tracers for nanoBRET assay.

Kinase	Vector catalog #	Tracer	Tracer catalog #	[Tracer], [M]
ALK1	NV2391	ALK family tracer	on request	2.50E-07
ALK2	NV2341	K11	N2850	1.25E-07
ALK3	NV2471	ALK family tracer	on request	2.50E-07
ALK4	NV1021	K14	on request	5.00E-07
ALK5	kind gift of Promega	K14	on request	5.00E-07
ALK6	kind gift of Promega	SGC-1 tracer	N/A	5.00E-07
DDR1	N2451	K4	N2540	4.09E-08
FLT3	NV1391	K5	N2530	1.30E-07
KHS/MAP4K5	NV3501	K10	N2840	2.52E-07

Figure S1: Plots obtained in the NanoBRET assays; the EC_{50} values are summarized in Table 2.

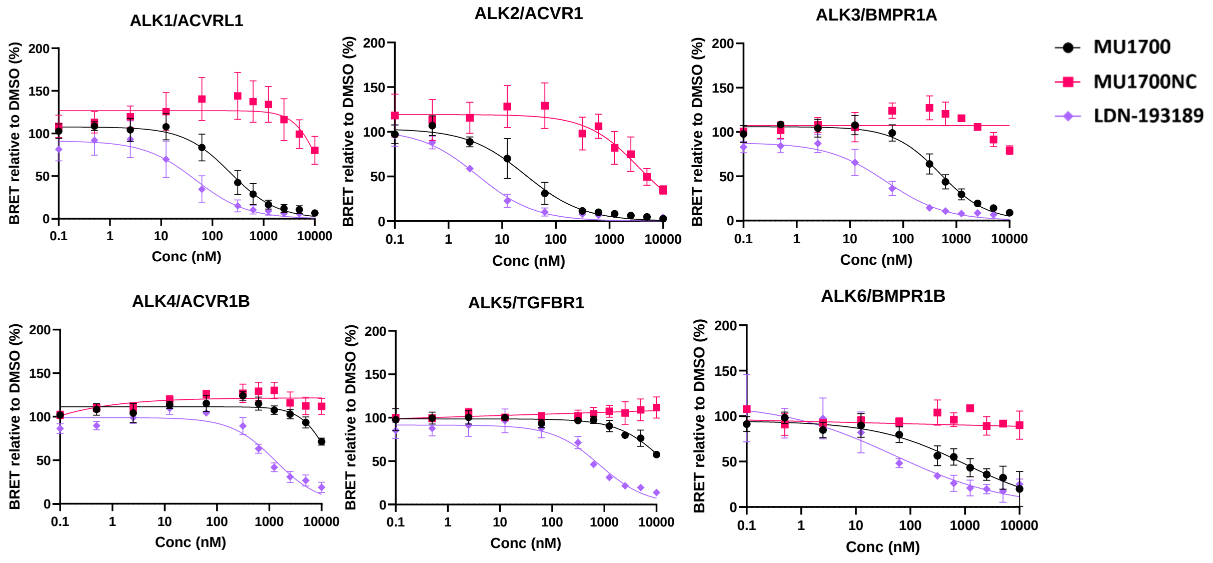

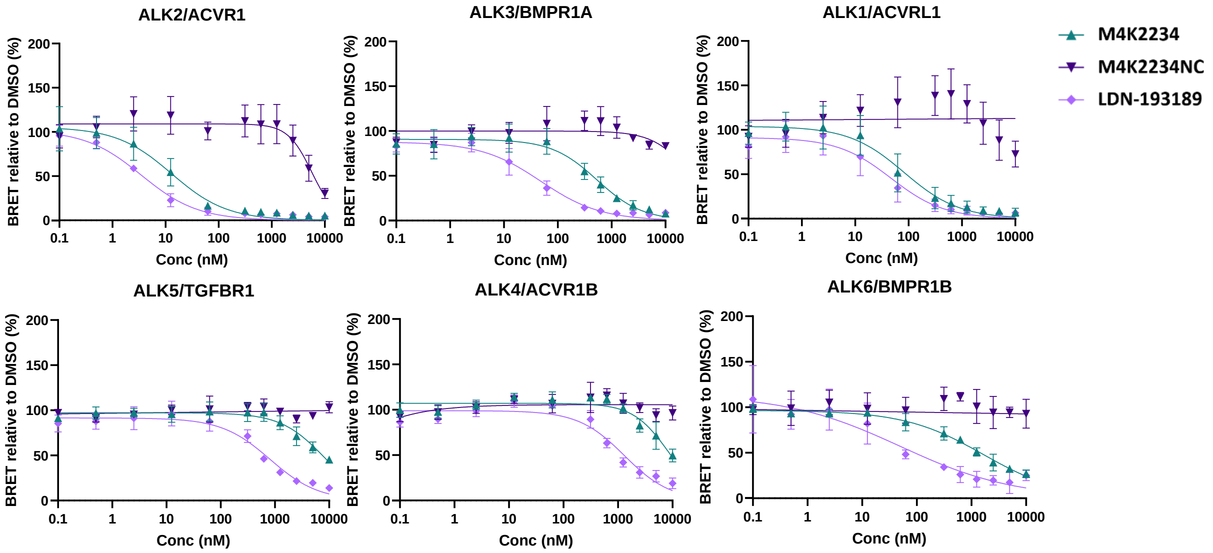

ALK2/ACVR1
ALK3/BMPR1A
ALK1/ACVRL1
ALK5/TGFBR1
ALK4/ACVR1B
ALK6/BMPR1B
BRET relative to DMSO (%)
Conc (nM)
0
50
100
150
200
0.1
1
10
100
1000
10000
M4K2234
M4K2234NC
LDN-193189

6. Inhibition of SMAD-dependent transcriptional reporters

Dual Luciferase Reporter Assay

HEK293 cells were co-transfected with BRE-Luc and Renilla luciferase pRLTK for measurements of BMP pathway signaling, or with CAGA-Luc and Renilla luciferase for measurements of activin or TGF-β pathway signalling. The BRE-Luc plasmid contains a BMP-responsive element from the Id1 promoter fused to a luciferase reporter gene.[9] CAGA-Luc contains a TGF-β responsive element from the PAI-1 promoter fused to luciferase reporter gene.[10] 10,000 transfected cells were seeded into each well of a 96-well plate (Corning) cultured in cultured in DMEM/10% FBS. 16 h after transfection, cells were starved for 7 h in DMEM containing 1% FBS. Cells were then incubated with 10 ng/mL BMP7, 10 ng/mL activin A or 10 ng/mL TGF-β1 (Peprotech, 100-21-10) and test compounds simultaneously at the indicated concentrations in a humidified, 37 °C incubator with 5% CO_2. Another 24 h later, the cells were harvested, lysed, and processed for the measurement of luciferase activity using the Dual-Luciferase Reporter Assay System (Promega) according to the manufacturer's instructions. Culture medium was aspirated and cells were lysed in 50 µL of 1× PLB with 300 rpm agitation for 30 min. The cell lysate (10 µL) was dispensed into each well of a 384-well flat-bottom polypropylene plate (Greiner). The luminescent signal of firefly- and Renilla-luciferase activity was measured sequentially using a PHERAstar FS microplate reader (BMG Labtech) after the addition of 25 µL of LARII and Stop & Glo, respectively. A measurement interval of 2 s and gain setting of 3600 were used. The firefly-luciferase signal was normalized to the cell number by division with the Renilla-luciferase signal. The relative luciferase unit (RLU) was obtained by further division with the signal from cells without ligand stimulation. The apparent EC_{50} values of test compounds were estimated using the [Inhibitor] versus response (three-parameter) nonlinear regression curve fitting function of GraphPad Prism 7. Results are the means ± S.D. of at least three independent experiments, each performed in triplicate.

7. Western blot analysis of SMAD protein phosphorylation after receptor activation

For Figure 5, Human embryonic kidney 293T cells were propagated in DMEM media supplemented with 10% FBS and antibiotics. 160.000 cells/well were seeded in 12-well tissue culture plates and pretreated with **MU1700** or **M4K2234** for 30 minutes before adding ligands for 60 minutes. The following recombinant ligands were used: BMP9 (20 ng/ml), BMP2 (20 ng/ml), GDF5 (100 ng/ml), Activin (100 ng/ml), BMP3 (100 ng/ml), BMP7 (60 ng/ml), TGFβ1 (20ng/ml). Cells were lysed in Laemmli buffer, protein lysates were resolved on SDS-PAGE and SMAD.

Further dose response profiling (Figure S2) was performed using HEK293 cells propagated in DMEM and 10% FBS overnight in a 24-well plate. The medium was then exchanged to OptiMEM to starve the cells for 1 hour. Serial dilutions of the indicated compounds were prepared in OptiMEM together with the indicated ligands and the mixtures added to the cells for a further 30-60 minutes. Cells were aspirated and then lysed in a buffer containing 50 mM Tris-HCl pH 7.4, 150 mM NaCl, 1% Triton-X100, protease inhibitor cocktail, 25 mM NaF, 2 mM sodium orthovanadate, 10 µg/ml benzonase. Protein lysates were resolved on SDS-PAGE and Western blot detection performed.

Figure S2: Western blot analysis showing selective inhibition of BMP7-induced SMAD1/5/8 phosphorylation by chemical probes **M4K2234** and **MU1700**.

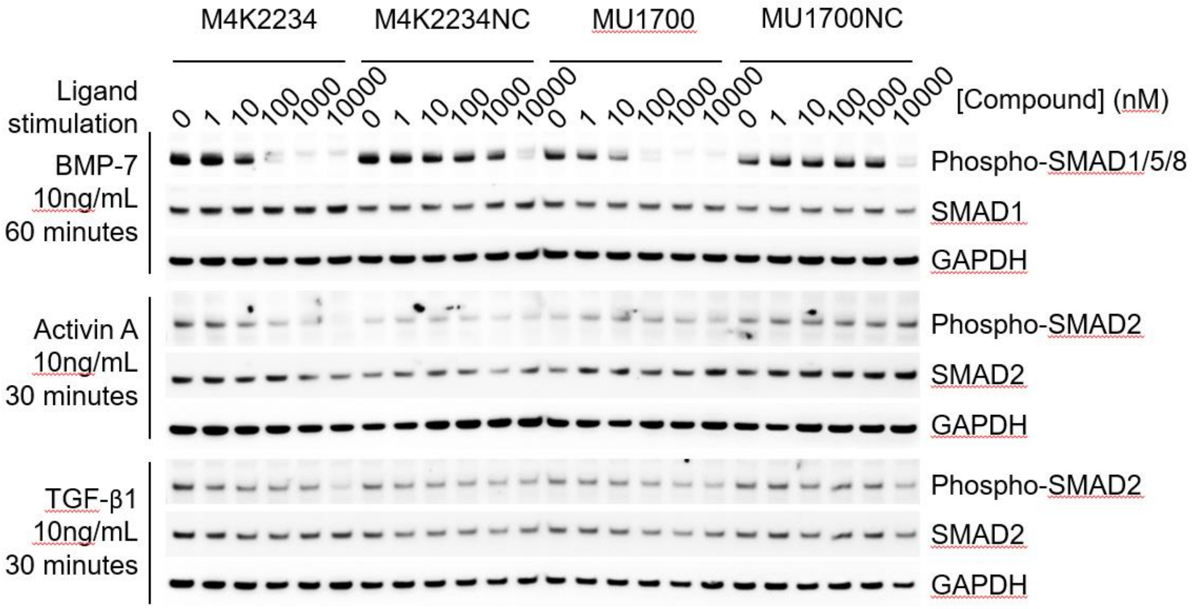

HEK293 cells were stimulated with BMP7, activin A or TGF-β1 in the presence of the indicated inhibitors. SMAD1/5/8 phosphorylation induced by BMP7 was observed to decrease in the presence of 10 nM **M4K2234** or **MU1700** and was completely inhibited by 100 nM inhibitor. By contrast, inhibition of SMAD1/5/8 phosphorylation by the **M4K2234NC** and **MU1700NC** negative controls was only observed at 10 μM concentration. SMAD2 phosphorylation induced by activin A or TGF-β1 was also inhibited by 10 μM **M4K2234**, but not by other compounds confirming the selectivity of the compounds.

8. Pharmacokinetic profile of MU1700.2HCl

STUDY REPORT P092920a – BIENTA ENAMINE BIOLOGY SERVICES

Study responsibilities

Study Director	Anna Kondratiuk, Ph.D.
In Vivo Scientists	Iryna Pishel, Ph.D.
	Alexander Tarasov, M.S.
	Maria Sokolenko, L.T.
Scientists	Volodymyr Iurchenko, Ph.D.
	Yulya Tokaryeva, M.S.
	Illia Petrov, M.S.

Study objective

The purpose of this study was to determine the pharmacokinetic characteristics of compound **MU1700.2HCL** in Balb/cAnN mice following intravenous (IV) and peroral (PO) administration. Levels of **MU1700.2HCL** were determined in blood plasma over time after a single dose.

Reagents and consumables

Compound **Sertraline** was used as internal standard (**IS**)

Compound **MU1700.2HCL** was supplied as dry powder. The batches of working formulations were prepared 30 min prior to the *in vivo* study. The vehicle was physiological saline. To prepare the formulation, the test compound was dissolved in saline. The mixture was vortexed for 2 minutes and sonicated for 5 min at 40ºC. The compound was fully dissolved in the formulation vehicle.

Equipment

Gradient HPLC system (Shimadzu, Japan)

MS/MS detector API 3000 PE with TurboIonSpray Electrospray module (PE Sciex, Canada)

VWR Membrane Nitrogen Generators N2-04-L1466. nitrogen purity 99%+ (VWR, USA)

Water purification system Millipore Milli-Q Gradient A10 (Millipore, France)

Fixed Speed Vortex Mixer “IKA Lab Dancer” (IKA®-Werke GmbH & Co. KG, Germany; IP-40)

Centrifuge 4-15C (Qiagen) (Sigma, Germany)

Ultrasonic bath (Daihan, Korea; WUC-A03H)

Centrifuge 5417R (Eppendorf, Germany)

Study design

Study design, animal selection, handling and treatment were all in accordance with the Enamine PK study protocols and the Institutional Animal Care and Use Guidelines. Animal treatment and plasma samples preparation were conducted by the Animal Laboratory personnel at Enamine/Bienta. Male Balb/cAnN mice (9-10 weeks old, body weight 18.0 g to 24.6 g and average body weight across all groups 20.6 g SD = 1.3 g) were used in this study. The animals were randomly assigned to the treatment groups before the pharmacokinetic study; all animals were fasted for 4 h before dosing. Seven time points (5, 15, 60, 120, 240, 360, and 600 min for IV; 5, 15, 30, 60, 120, 240, and 600 min for PO) were set for this pharmacokinetic study. Each of the time point treatment group included 4 animals. There was also control group of 2 animals. Dosing was done according to the treatment schedules outlined in Table S6. Mice were injected IP with 2,2,2-tribromoethanol at the dose of 150 mg/kg prior to drawing the blood. Blood collection was performed from the orbital sinus in microtainers containing K_2EDTA. Animals were sacrificed by cervical dislocation after the blood samples collection. All samples were immediately processed, flash-frozen and stored at -70°C until subsequent analysis.

Table S6. Study design.

Number of Mice (male)	Compound ID	Formulation	Delivery Route	Target Dose Level (mg/kg)	Target Dose Concentration (mg/ml)	Target Dose Volume (ml/kg)
28	**MU1700.2HCL**	Physiological saline	IV	20*	4*	5
28	**MU1700.2HCL**		PO	20*	4*	5
1	Vehicle dosed	Physiological saline	IV	0	0	5
1	Vehicle dosed		PO	0	0	5

*the dose was calculated for free base

Samples processing

Plasma samples (50 µl) were mixed with 200 µl of **IS** solution. After mixing by pipetting and centrifuging for 4 min at 6,000 rpm, 2 µl of each supernatant was injected into LC-MS/MS system. Solution of compound **Sertraline** (400 ng/ml in acetonitrile-methanol mixture 1:1, v/v) was used as internal standard (**IS**) for quantification of **MU1700.2HCL** in plasma samples.

Samples analysis

Analyses of plasma samples were conducted by the Bioanalytical Laboratory personnel at Enamine/Bienta. Plasma concentrations of **MU1700.2HCL** were determined using high performance liquid chromatography/tandem mass spectrometry (HPLC-MS/MS). Shimadzu HPLC system consisted of controller Prominence CBM20A2, isocratic pumps LC-10ADvp, an autosampler Prominence SIL-20AC, a sub-controller FCV-14AH, and a degasser DGU-14A. Mass spectrometric analysis was performed using API 3000 (triple-quadrupole) instrument from AB Sciex (Canada) with

an electro-spray (ESI) interface. The data acquisition and system control was performed using Analyst 1.5.2 software (AB Sciex, Canada).

HPLC-MS/MS Conditions

Chromatographic Conditions:

Column: Phenomenex Luna C18(2) (50 x 2 mm, 5μm)

Mobile phase A: Acetonitrile : Water : Formic acid = 50 : 950 : 1

Mobile phase B: Acetonitrile : Formic acid = 100 : 0.1

Linear gradient: 0 min 5% B, 0.90 min 100% B, 1.10 min 100% B, 1.11 min 5% B, 2.5 min stop

Elution rate: 400 μL/min. A divert valve directed the flow to the detector from 1.4 to 1.9 min

Column temperature: 30°C

MS/MS Detection:

Scan type: Positive MRM, Ion source: Turbo spray, Ionization mode: ESI

Nebulize gas: 15 L/min, Curtain gas: 8 L/min, Collision gas: 4 L/min

Ionspray voltage: 5000 V, Temperature: 400°C

Table S7. Other MS parameters

Compound ID	Parent, m/z	Daughter, m/z	Time, ms	DP, V	FP, V	EP, V	CE, V	CXP, V
**MU1700.2HCl**	204.106	364.0	60	76	400	11	21	10
**Sertraline**	306.097	275.1	60	31	400	11	17	46

Preparation of calibration standards

Compound **MU1700.2HCL** was dissolved in DMSO, and resulting solution with concentration of 2 mg/ml was used for calibration standards preparation (stock solution). A series of calibration standards was prepared by consecutive dilution of the stock compound solution with blank mouse plasma to a final concentration of 40 000, 20 000, 10 000, 5 000, 2 500, 1 000, 500, 250, 100, 50, and 20 ng/ml. Standard plasma samples (50 μl) were mixed with 200 μl of **IS**. After mixing by pipetting and centrifuging for 4 min at 6,000 rpm, 2 μl of each supernatant was injected into LC-MS/MS system.

Method Validation Results

Specificity: Mice blank plasma had no interference with compound **MU1700.2HCL** and IS, as shown in Figure S2.

Figure S2. Chromatographic Graphs

Mice blank plasma

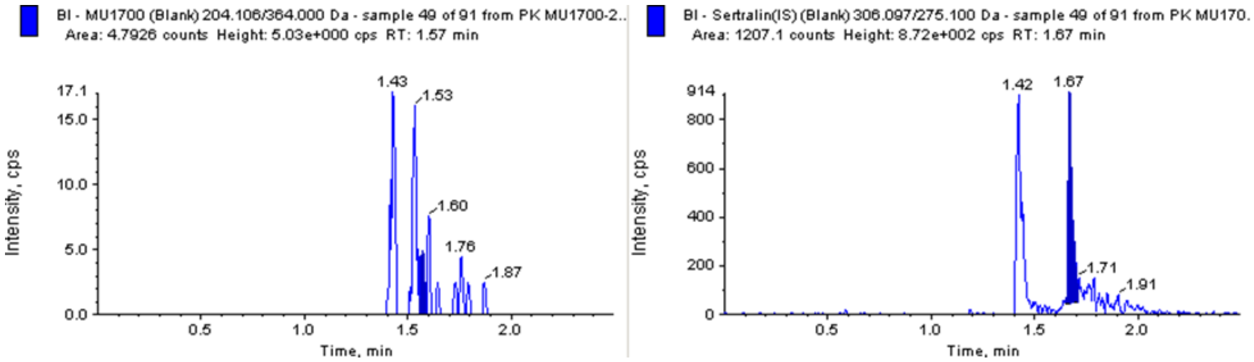

Blank plasma spiked with compound **MU1700.2HCL** (20 ng/ml) and IS

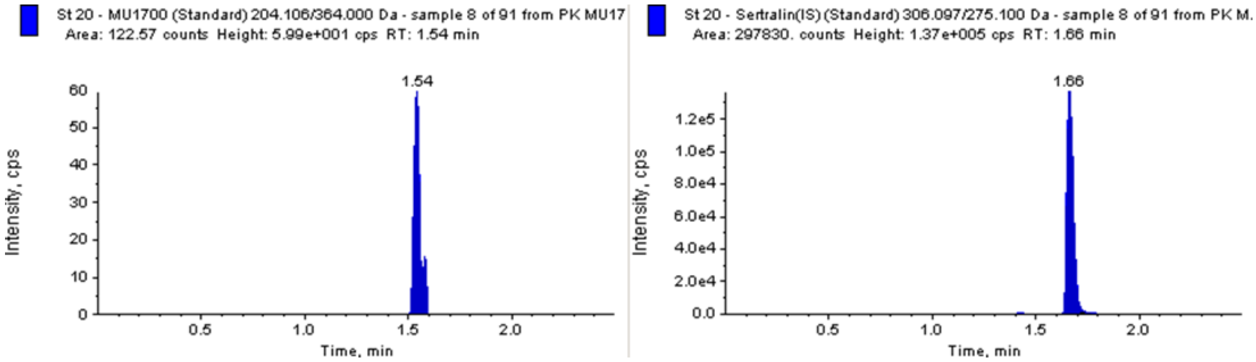

Calibration curve

The regression analysis of compound **MU1700.2HCL** was performed by plotting the peak area ratio (y) against the compound concentration in calibration standards (x, ng/ml). The validity of the calibration curve (relationship between peak area ratio and compound concentration) is proved by the correlation coefficient (R) calculated for the quadratic regression (Figure S3).

Figure S3. Calibration curve for the quantification of MU1700.2HCL (weight=1/x)

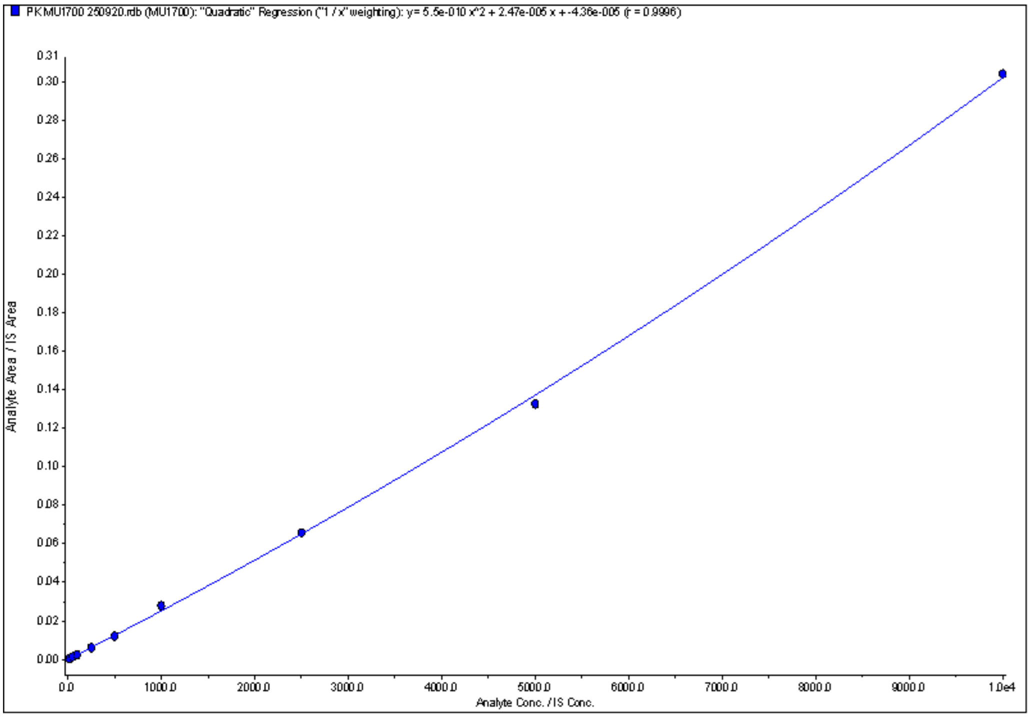

Correlation coefficient = 0.9996

Pharmacokinetic Method Analysis

The concentrations of **MU1700.2HCL** in plasma samples below the lower limit of quantitation (LLOQ – 20 ng/ml) were designated as zero. The pharmacokinetic data analysis was performed using noncompartmental, bolus injection or extravascular input analysis models in WinNonlin 5.2 (PharSight). Data below LLOQ were presented as missing to improve validity of T½ calculations. One outlier data point was removed from calculations.

The oral bioavailability was calculated as:

$$F\ (\%) = \frac{Dose_{\ IV} \times\ AUC_{(0-\infty)PO}}{Dose_{PO} \times AUC_{(0-\infty)IV}} \times 100\%$$

Results

The individual and average **MU1700.2HCL** concentrations data in plasma for perorally- and intravenously-dosed groups are listed in Tables S8 and S10 and graphically presented in Figures S4-S5. Selected noncompartmental pharmacokinetic parameters for plasma are listed in Tables S9 and S11. Please note that elimination curve approximation is performed automatically by WinNonlin PK program using a standardized procedure. Resulting calculated elimination rate K_{el} and the parameters derived from it, such as terminal elimination half-life $T_{1/2}$, may not always properly reflect the observed pharmacokinetic processes. In some cases, alternative analyses of the observed concentration-time dependency may be required. Any comparison of resulting PK parameter values and conclusions about compound and/or formulation properties must be based on full PK curve data and take into account specific experimental designs.

Table S8. Plasma concentrations of compound MU1700.2HCL in male Balb/cAnN mice following intravenous (20 mg/kg) administration

**Sample collection time point, min**	**Plasma concentration (ng/ml)**						
	**Mouse A**	**Mouse B**	**Mouse C**	**Mouse D**	**Mean**	**SD**	**SE**
0	BQL				**BQL**	ND	ND
5	6761	6589	5568	6803	**6430**	582	291
15	3964	3855	4376	5544	**4435**	773	386
60	2165	2233	2992*	2188	**2195**	35	20
120	1389	1500	1581	2314	**1696**	419	210
240	720	653	681	658	**678**	31	15
360	464	277	286	558	**396**	138	69
600	110	152	234	152	**162**	52	26

BQL - Below the lower limit of quantitation (LLOQ)
ND - Not determined
*Grubbs' outlier test: Significant outlier. $P < 0.05$

Table S9. Selected pharmacokinetic parameters for compound MU1700.2HCL in male Balb/cAnN mice following intravenous (20 mg/kg) administration

Animal	Administration	Dose, mg/kg	Pharmacokinetic Parameters									
			Tmax, min	C_0, ng/ml l	$AUC_{0\rightarrow t=600min}$ (AUClast) ng*min/ml	$AUC_{0\rightarrow\infty}$ (AUCINF_obs), ng*min/ml	$T_{1/2}$ (HL_Lambda_z), min	K_{el} (Lambda_z), min^{-1}	MRT (MRTlast), min	MRT (MRTinf), min	V_d (Vz_obs), ml/kg	CL (Cl_obs), ml/min/kg
Mice	IV	20	-	7743	630000	671000	176	0.00394	134	178	8000	30

Figure S4. Plasma concentration-time curve of compound MU1700.2HCL in male Balb/cAnN mice following intravenous (20 mg/kg) administration (n=4)

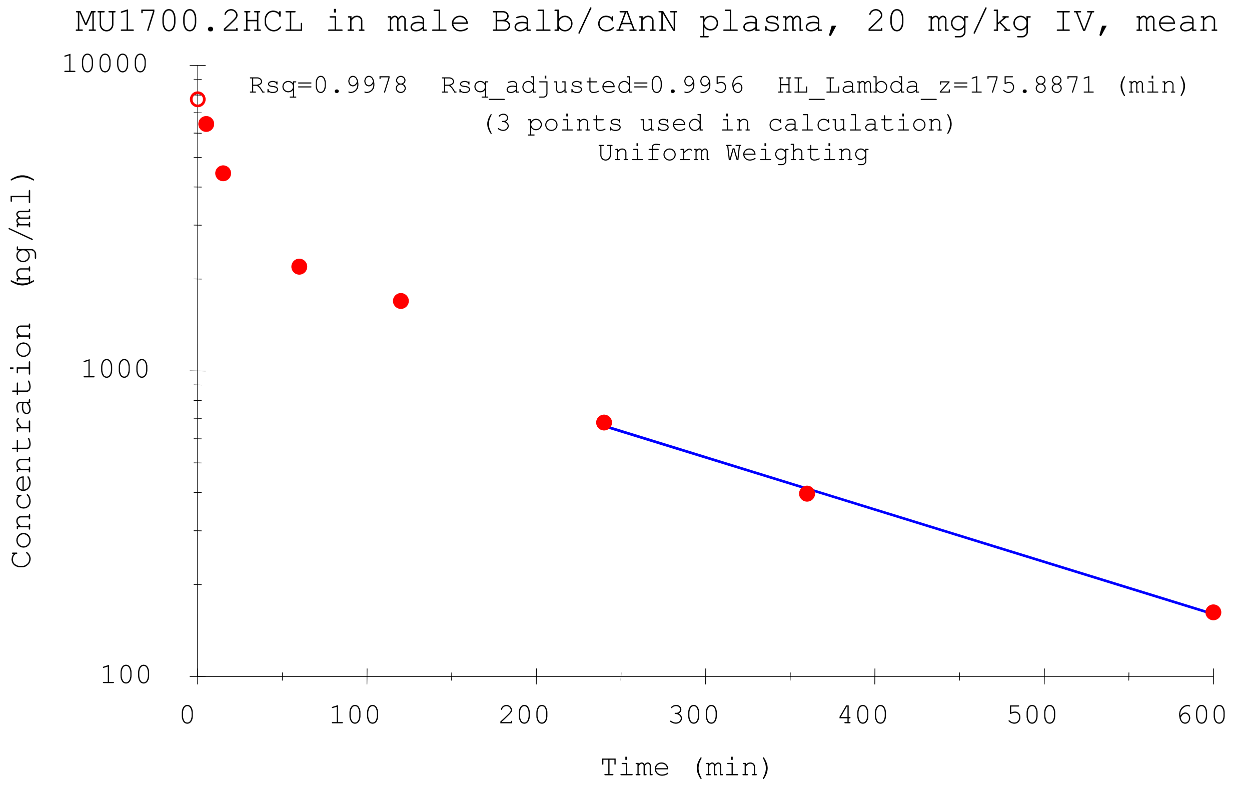

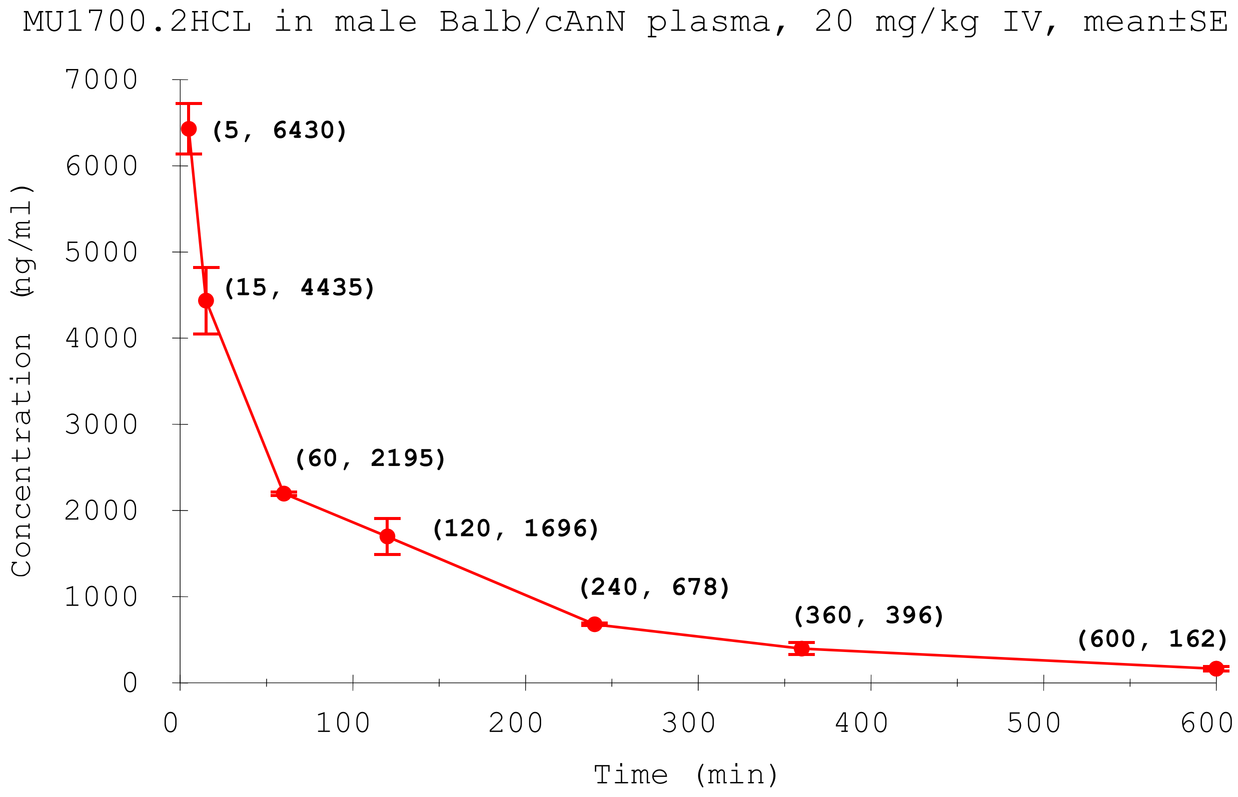

Table S10. Plasma concentrations of compound MU1700.2HCL in male Balb/cAnN mice following peroral (20 mg/kg) administration

Sample collection time point, min	Plasma concentration (ng/ml)						
	**Mouse A**	**Mouse B**	**Mouse C**	**Mouse D**	**Mean**	**SD**	**SE**
0	BQL				**BQL**	ND	ND
5	408	48	175	43	**168**	171	85
15	685	557	1088	647	**744**	235	118
30	1518	1185	1117	999	**1205**	223	111
60	1825	1983	1848	1050	**1677**	423	212
120	2019*	1749	1762	1763	**1758**	8	5
240	792	933	662	882	**817**	119	59
600	196	140	221	113	**168**	50	25

BQL - Below the lower limit of quantitation (LLOQ)
ND - Not determined

Table S11. Selected pharmacokinetic parameters for compound MU1700.2HCL in male Balb/cAnN mice following peroral (20 mg/kg) administration

Animal	Administration	Dose, mg/kg	Pharmacokinetic Parameters							
			Tmax, min	Cmax, ng/ml	$AUC_{0\rightarrow t=600min}$ (AUClast) ng*min/ml	$AUC_{0\rightarrow\infty}$ (AUCINF_obs), ng*min/ml	$T_{1/2}$ (HL_Lambda_z), min	K_{el} (Lambda_z), min^{-1}	MRT (MRTlast), min	MRT (MRTinf), min
Mice	PO	20	240	1760	498000	533000	145	0.00478	180	221

Figure S5. Plasma concentration-time curve of compound MU1700.2HCL in male Balb/cAnN mice following peroral (20 mg/kg) administration (n=4)

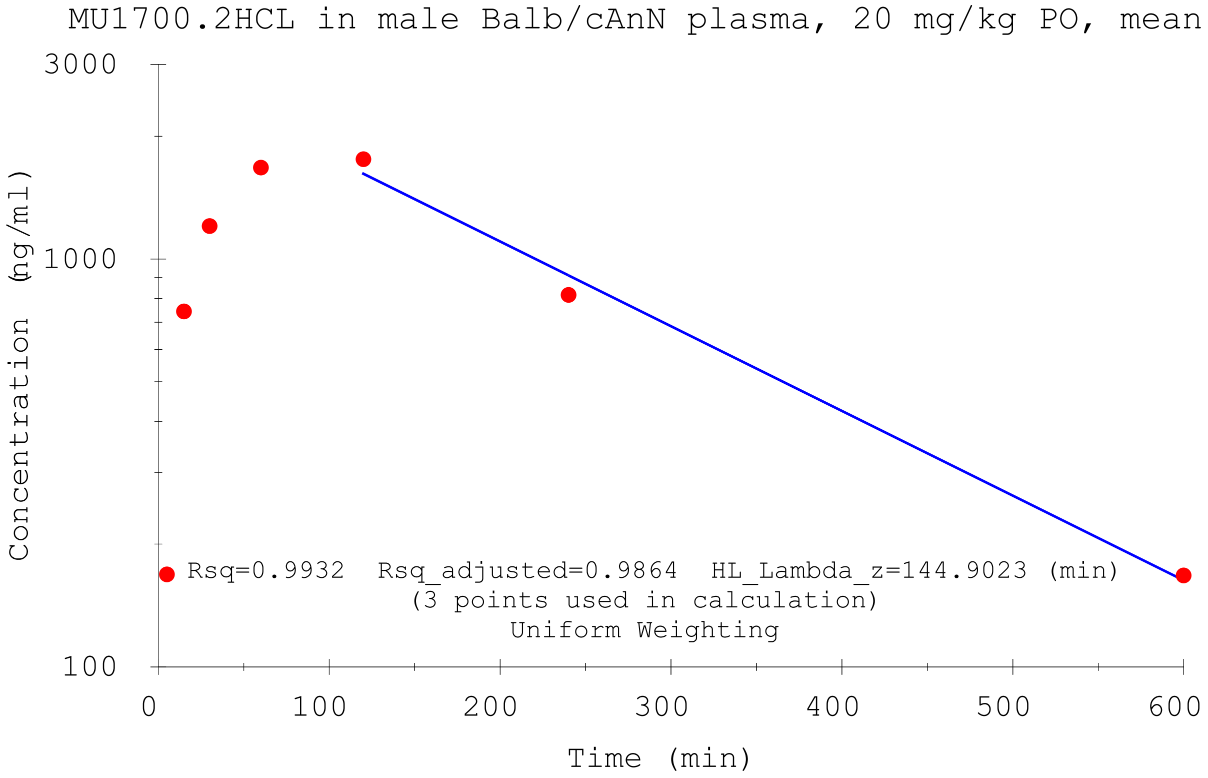

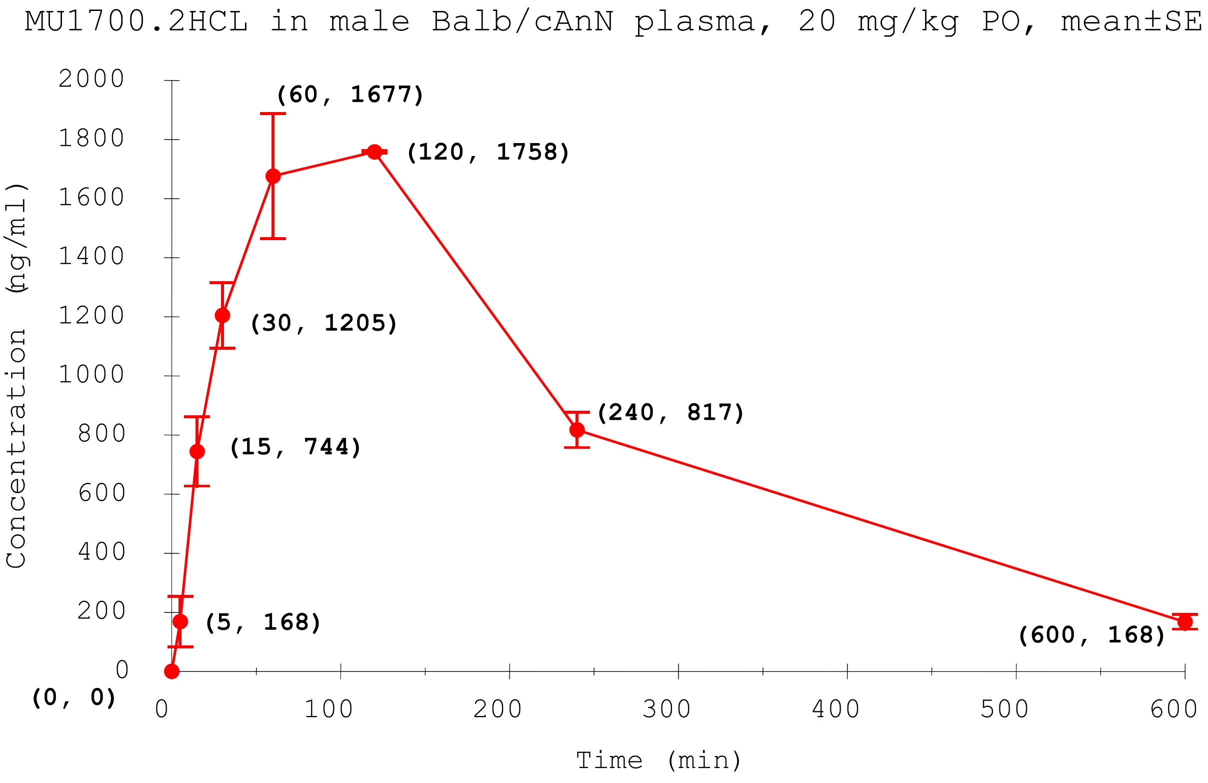

Summary: The pharmacokinetic parameters for **MU1700.2HCL** in blood plasma are shown in the summary table below (Table S12). The calculated oral bioavailability for compound **MU1700.2HCL** is **79%**. Figure S6 summarizes the results of PK study for compound **MU1700.2HCL** in mice.

No obvious adverse effects were observed during this PK study.

Table S12. Selected pharmacokinetic parameters for MU1700.2HCL in male Balb/cAnN mice

Sample	Administration	Dose, mg/kg	Pharmacokinetic Parameters							
			Tmax, min	Cmax, ng/ml	$AUC_{0\to t\,min}$ (AUClast) ng*min/ml	$AUC_{0\to\infty}$ (AUCINF_obs) ng*min/ml	$T_{1/2}$ (HL_Lambda_z), min	K_{el} (Lambda_z), min^{-1}	V_d (Vz_obs) ml/kg	Bioavailability, %
Plasma	IV	20	-	6430	630000	671000	176	0.00394	8000	**79**
	PO	20	240	1760	498000	533000	145	0.00478	ND	

Note: in Table S12, C_{max} is indicated for IV route, in contrast to C_0 from Table S9

Figure S6. Concentration-time curves for MU1700.2HCL in male Balb/cAnN mice following IV and PO administration

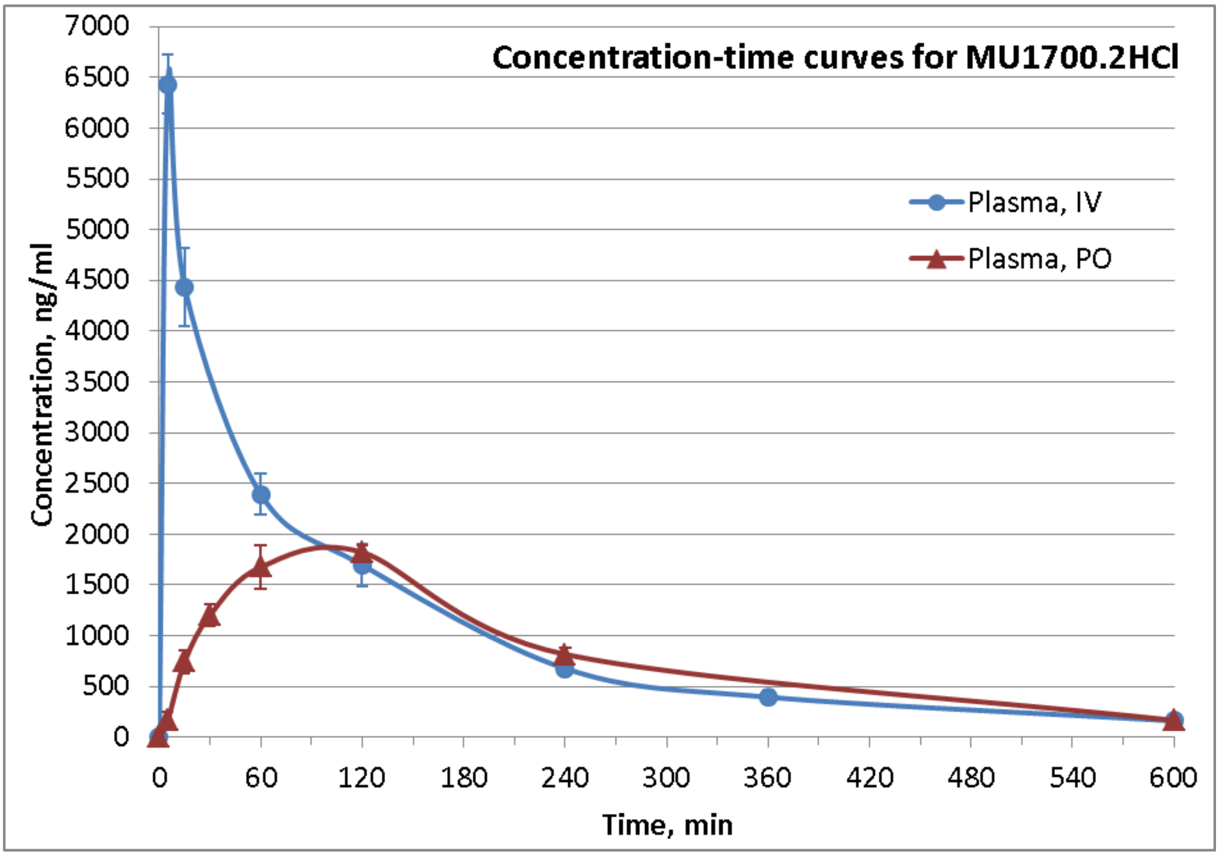

STUDY REPORT P111221a – BIENTA ENAMINE BIOLOGY SERVICES

Study responsibilities

Study Director	Yuliia Holota, Ph.D.
In Vivo Scientists	Alexander Tarasov, M.S.
	Maria Sokolenko, L.T.
	Arabadzhy Viktoriia, M.S.
	Kateryna Ivanchenko M.S.
Scientists	Volodymyr Iurchenko, Ph.D.
	Yulya Tokaryeva, M.S.
	Karina Turenko, M.S.
	Illia Petrov, M.S.

Study objective

The purpose of this study was to determine the pharmacokinetic characteristics of compound **MU1700.2HCL** in Balb/cAnN mice following peroral (PO) administration. Levels of **MU1700.2HCL** were determined in blood plasma and brain over time after a single dose.

Reagents and consumables

Compound **Sertraline** was used as internal standard (**IS**)

Compound **MU1700.2HCL** (dihydrochloride salt) was supplied as a dry powder. The batches of working formulations were prepared 1 min prior to the *in vivo* study. The vehicle was physiological saline. To prepare the formulation, the test compound was mixed with the calculated volume of saline, the mixture was vortexed for 30 sec and sonicated for 3 min at 40 ºC (the solution was with flakes). Vortexing of the mixture for 10 sec and sonication for 2 min at 40 ºC were repeated four times. The resulting solution was clear (pH= 2.2).

Equipment

Gradient HPLC system (Shimadzu, Japan)

MS/MS detector API 3000 PE with TurboIonSpray Electrospray module (PE Sciex, Canada)

IMT-PN 1280 OG Nitrogen Generator (INMATEC Technologies GmbH, Germany)

Water purification system Arium mini (Sartorius, Germany)

VWR Analog Vortex Mixer VM 3000 (VWR, USA)

Centrifuge 4-15C (Qiagen) (Sigma, Germany)

Centrifuge 5417R (Eppendorf, Germany)

Dispersing system The Bullet Blender®, model BB24-AU (Next Advance, USA)

Study design

Study design, animal selection, handling and treatment were all in accordance with the Enamine PK study protocols and the Institutional Animal Care and Use Guidelines. Animal treatment and plasma samples preparation were conducted by the Animal Laboratory personnel at Enamine/Bienta. Male Balb/cAnN mice (12 weeks old, body weight 18.1 g to 23.2 g and average body weight across all groups 20.6 g SD = 1.4 g) were used in this study. The animals were randomly assigned to the treatment groups before the pharmacokinetic study; all animals were fasted for 4 h before dosing. Seven time points (5, 15, 30, 60, 120, 240 and 600 min) were set for this pharmacokinetic study. Each of the time point treatment group included 4 animals. There was also a control group of one animal. Dosing was done according to the treatment schedule outlined in Table S13. Mice were injected IP with 2,2,2-tribromoethanol at the dose of 150 mg/kg prior to drawing the blood. Blood collection was performed from the orbital sinus in microtainers containing K_2EDTA. Animals were sacrificed by cervical dislocation after the blood samples collection. Brain samples (left lobe) were collected and weighed. All samples were immediately processed, flash-frozen and stored at -70 °C until subsequent analysis.

Table S13. Study design.

Number of Mice (male)	Compound ID	Formulation	Delivery Route	Target Dose Level (mg/kg)	Target Dose Concentration (mg/ml)	Target Dose Volume (ml/kg)
28	**MU1700.2HCL**	Physiological saline	PO	100*	10*	10
1	Vehicle dosed		PO	0	0	10

*Dose calculated for the free base of the compound

Samples processing

Plasma samples (40 µl) were mixed with 200 µl of **IS** solution. After mixing by pipetting and centrifuging for 4 min at 6,000 rpm, 2 µl of each supernatant was injected into LC-MS/MS system.

Solution of compound **Sertraline** (400 ng/ml in water-methanol mixture 1:9, v/v) was used as internal standard (**IS**) for quantification of **MU1700.2HCL** in plasma samples.

Brain samples (weight 115 mg – 161 mg) were homogenized with 5 parts of **IS** solution (1 w + 5 v) using zirconium oxide beads (115 mg ± 5 mg) in The Bullet Blender® homogenizer for 30 seconds at speed 8. After this, the samples were centrifuged for 4 min at 14,000 rpm, and 2 µl of each supernatant was injected into LC-MS/MS system.

Solution of compound **Sertraline** (400 ng/ml in water-methanol mixture, 1:4, v/v) was used as internal standard (**IS**) for quantification of **MU1700.2HCL** in the brain samples.

Samples analysis

Analyses of plasma and brain samples were conducted by the Bioanalytical Laboratory personnel at Enamine/Bienta. The concentration of **MU1700.2HCL** in plasma and brain samples was determined

using high performance liquid chromatography/tandem mass spectrometry (HPLC-MS/MS). Shimadzu HPLC system comprised 2 isocratic pumps LC-10ADvp, an autosampler SIL-20AC, a sub-controller FCV-14AH and a degasser DGU-14A. Mass spectrometric analysis was performed using an API 3000 (triple-quadrupole) instrument from AB Sciex (Canada) with an electro-spray (ESI) interface. The data acquisition and system control were performed using Analyst 1.6.3 software from AB Sciex.

HPLC-MS/MS Conditions

Chromatographic Conditions:

Column: Luna C18(2) (50 x 2.0 mm, 5µm)

Mobile phase A: Acetonitrile : Water : Formic acid = 50 : 950 : 1

Mobile phase B: Acetonitrile : Formic acid = 100 : 0.1

Linear gradient: 0 min 10% B, 0.9 min 100% B, 1.10 min 100% B, 1.11 min 10% B, 2.5 min stop

Elution rate: 400 µL/min. A divert valve directed the flow to the detector from 1.25 to 1.7 min

Column temperature: 30°C

MS/MS Detection:

Scan type: Positive MRM, Ion source: Turbo spray, Ionization mode: ESI

Nebulize gas: 15 L/min, Curtain gas: 8 L/min, Collision gas: 4 L/min

Ionspray voltage: 5000 V, Temperature: 400°C

Table S14. Other MS parameters

Compound ID	Parent, m/z	Daughter, m/z	Time, ms	DP, V	FP	EP, V	CE, V	CXP, V
**MU1700 2HCl**	204.106	364.0	100	76	400	11	21	10
Sertraline	306.097	275.1	100	31	400	11	17	46

Preparation of calibration standards

Calibration standards for quantification of MU1700.2HCL in plasma samples. Compound **MU1700.2HCL** was dissolved in DMSO and resulting solution with concentration of 2 mg/ml (for free base) was used for calibration standards preparation (stock solution). The stock solution was consecutively diluted with **IS** to get a series of calibration solutions with final concentrations of 10 000, 4 000, 2 000, 1 000, 400, 200, 100, 40, 20, 10, 4 and 2 ng/ml. Calibration curve was constructed using blank mouse plasma samples. To obtain calibration standards, blank plasma samples (40 µl) were mixed with 200 µl of corresponding calibration solution. After mixing by pipetting and centrifugation for 4 min at 6000 rpm, 2 µl of each supernatant was injected into LC-MS/MS system.

Calibration standards for quantification of MU1700.2HCL in brain samples. The stock solution of compound **MU1700.2HCL** was consecutively diluted with **IS** to get a series of calibration solutions with final concentrations of 10 000, 2 000, 1 000, 400, 200, 100, 40, 20, 10 and 4 ng/ml. Calibration curve was constructed using blank mouse brain samples. To obtain calibration standards, blank brain

samples (weight 100 mg ± 1 mg) were homogenized in 500 µl of corresponding calibration solution using zirconium oxide beads (115 mg ± 5 mg) in The Bullet Blender® homogenizer for 30 seconds at speed 8. After this, the samples were centrifuged for 4 min at 14 000 rpm, and 2 µl of each supernatant was injected into LC-MS/MS system.

Method Validation Results

Specificity: Figure S7 shows that the blank mouse plasma and brain had no interference with compound **MU1700.2HCL** and IS.

Figure S7. Chromatographic Graphs

Mouse blank plasma

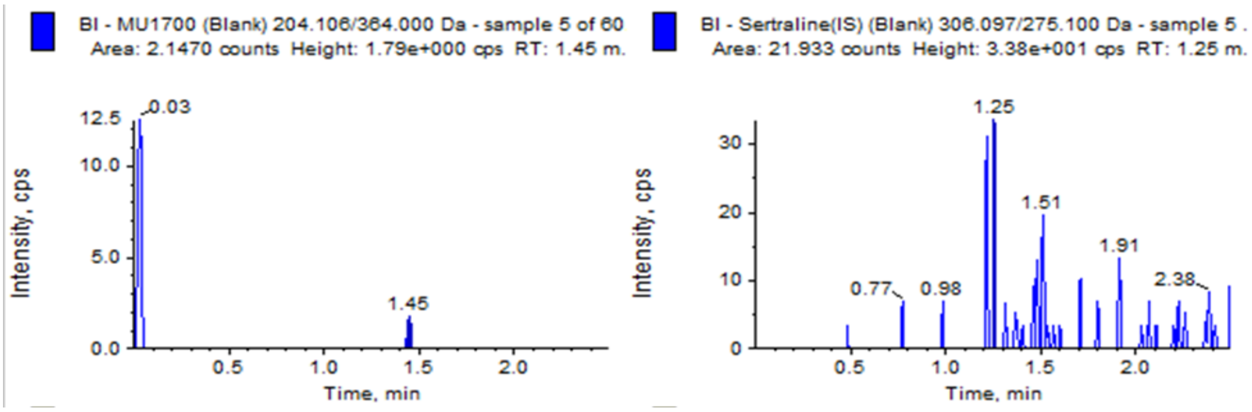

Blank plasma spiked with compound **MU1700.2HCL** (10 ng/ml) and IS

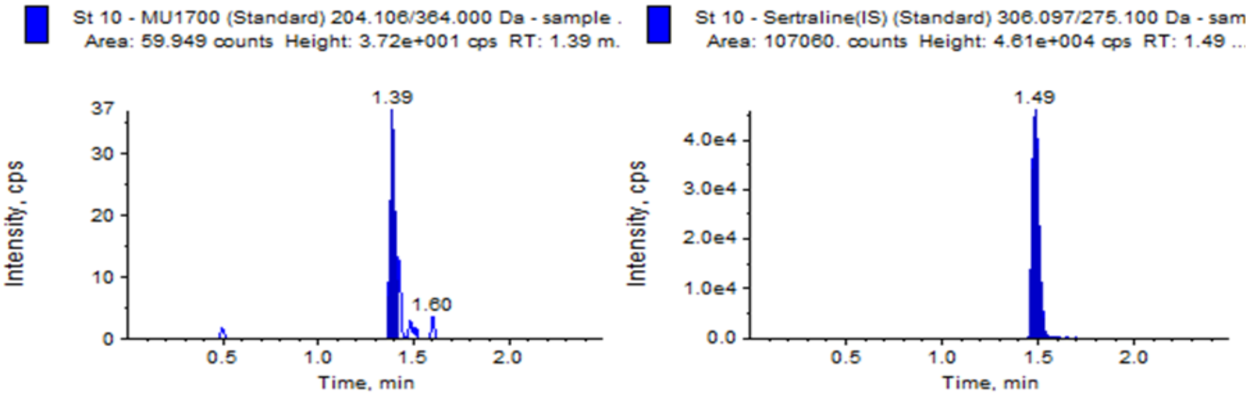

Mouse blank brain

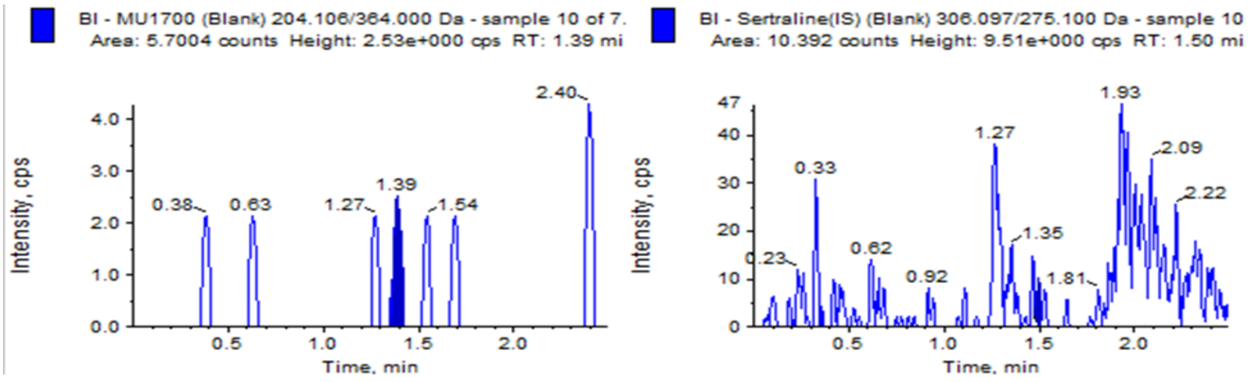

Blank brain sample spiked with compound **MU1700.2HCL** (20 ng/g) and IS

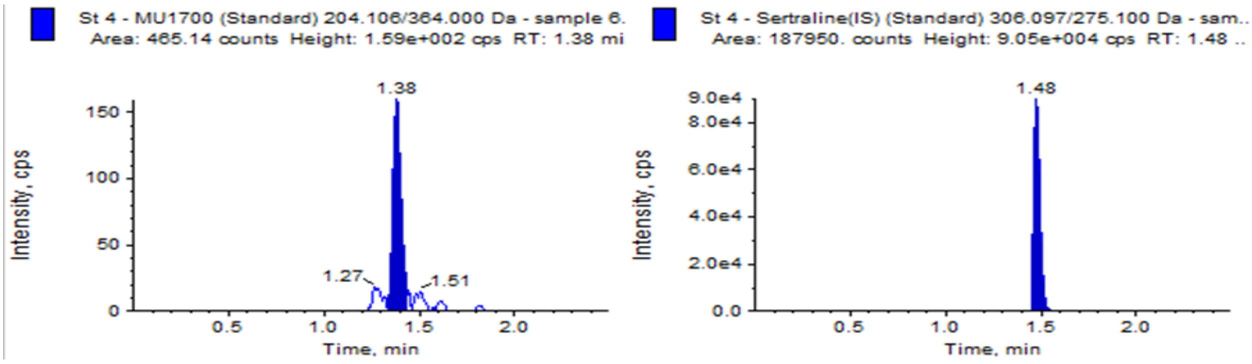

Calibration curves

The regression analysis of compound **MU1700.2HCL** was performed by plotting the peak area ratio (y) against the compound concentration in calibration solutions (x, ng/ml). The validity of the calibration curves (relationship between peak area ratio and compound concentration) is proved by the correlation coefficients (R) calculated for the quadratic regression (Figures S8A-C).

Figure S8A. Calibration curve for the quantification of MU1700.2HCL in plasma samples, time points 5-15 min (weight=1/x)

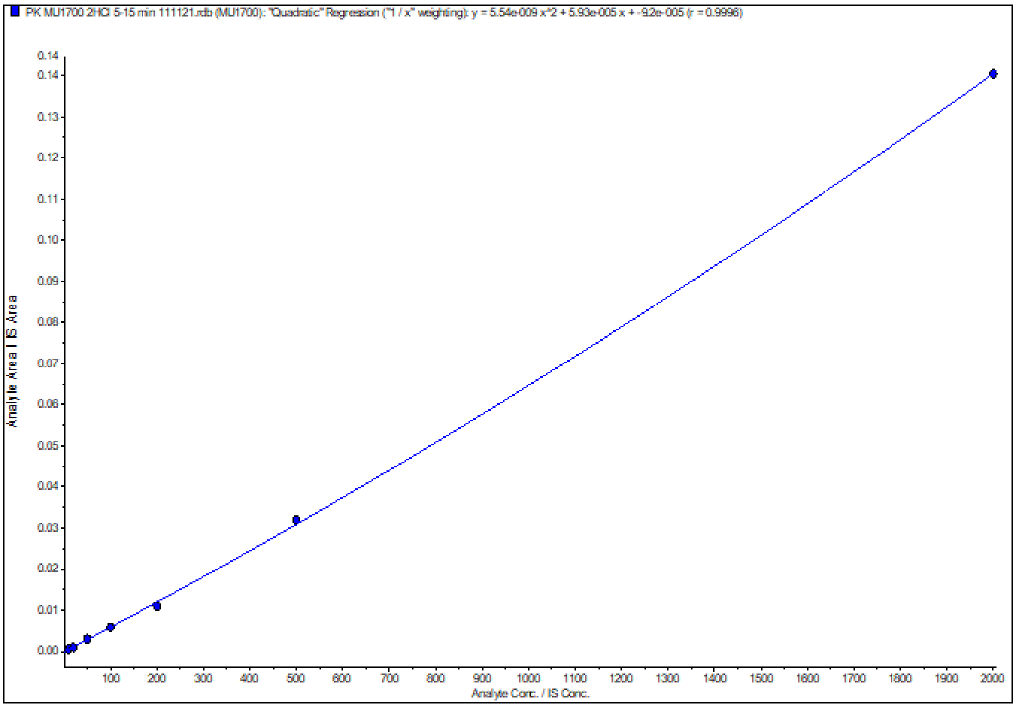

Correlation coefficient = 0.9996

Figure S8B. Calibration curve for the quantification of MU1700.2HCL in the plasma samples, time points 30-600 min (weight=1/x)

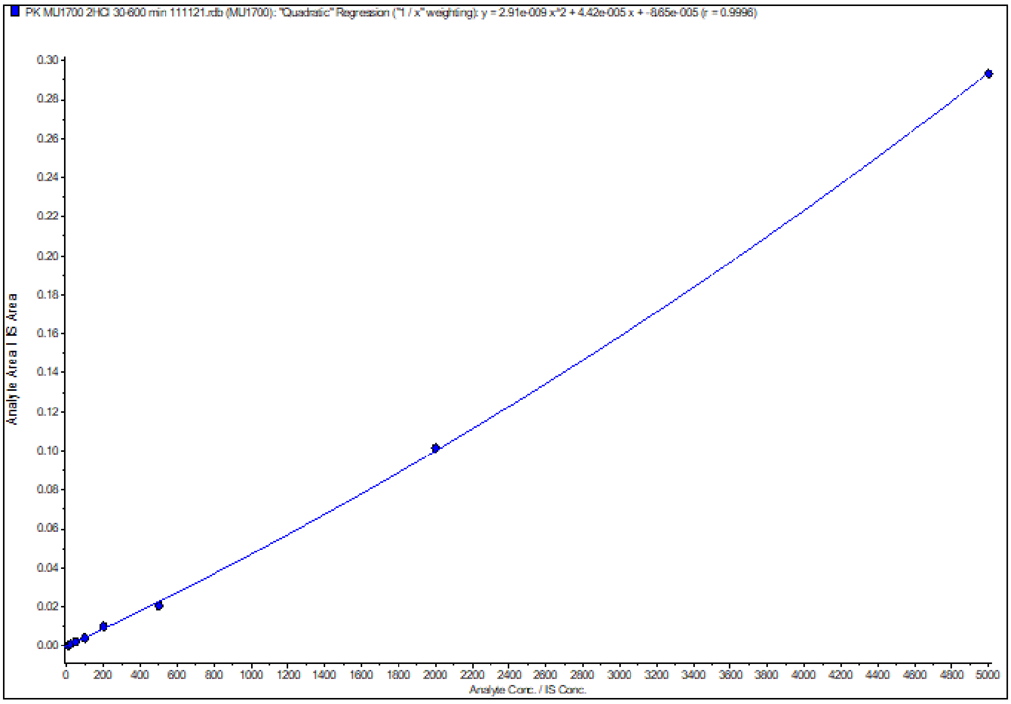

Correlation coefficient = 0.9996

Figure S8C. Calibration curve for the quantification of MU1700.2HCL in the brain samples (weight=1/x)

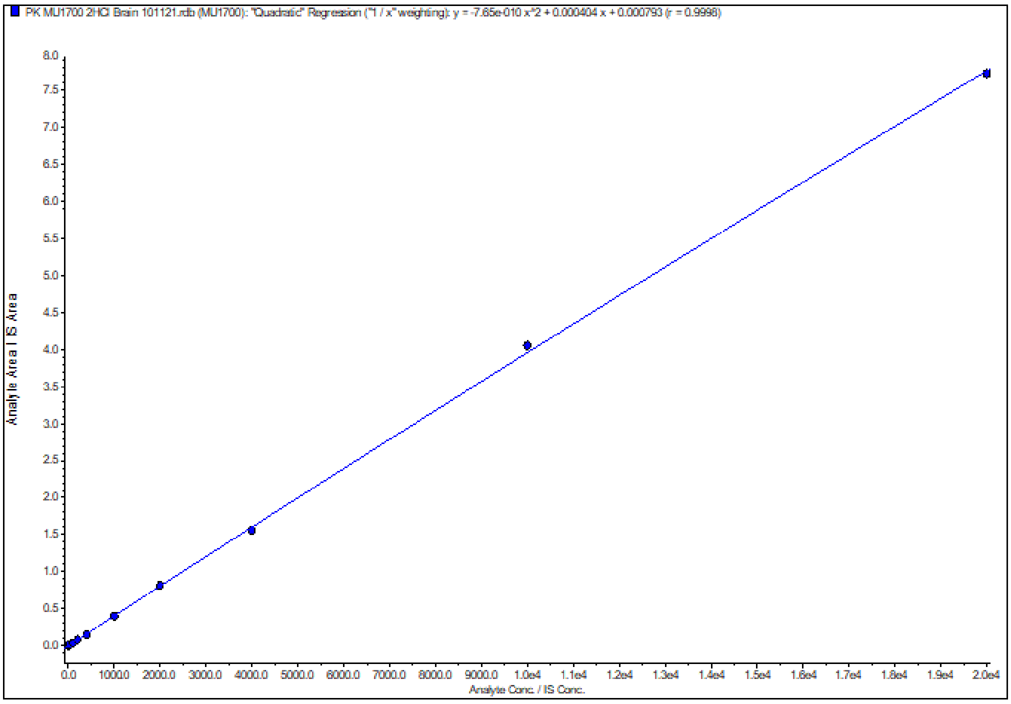

Correlation coefficient = 0.9998

Pharmacokinetic Method Analysis

The concentrations of **MU1700.2HCL** below the lower limit of quantitation (LLOQ = 10 ng/ml for plasma and 20 ng/g for brain samples) were designated as zero. The pharmacokinetic data analysis was performed using noncompartmental, bolus injection or extravascular input analysis models in

WinNonlin 5.2 (PharSight). Data below LLOQ were presented as missing to improve validity of T½ calculations.

Results

The individual and average **MU1700.2HCL** concentrations data in plasma and brain are listed in Tables S15 and S17 and graphically presented in Figures S9-10. Selected noncompartmental pharmacokinetic parameters for plasma are listed in Tables S16 and S18. Please note that elimination curve approximation is performed automatically by WinNonlin PK program using a standardized procedure. Resulting calculated elimination rate K_{el} and the parameters derived from it, such as terminal elimination half-life $T_{1/2}$, may not always properly reflect the observed pharmacokinetic processes. In some cases, alternative analyses of the observed concentration-time dependency may be required. Any comparison of resulting PK parameter values and conclusions about compound and/or formulation properties must be based on full PK curve data and take into account specific experimental designs.

Table S15. Plasma concentrations of compound MU1700.2HCL in male Balb/cAnN mice following PO (100 mg/kg) administration

Sample collection time point, min	Plasma concentration (ng/ml)						
	Mouse A	Mouse B	Mouse C	Mouse D	Mean	SD	SE
0	BQL				**BQL**	ND	ND
5	466	190	90	145	**223**	167	84
15	1272	1378	1307	1237	**1299**	60	30
30	2820	3154	3715	1834	**2881**	790	395
60	3551	3923	5166	4612	**4313**	719	359
120	3745	4158	4593	4622	**4280**	415	207
240	2307	3143	2818	2771	**2760**	344	172
600	1717	1782	2085	1856	**1860**	160	80

BQL - Below the lower limit of quantitation (LLOQ)
ND - Not determined

Table S16. Selected pharmacokinetic parameters (plasma) for compound MU1700.2HCL in male Balb/cAnN mice following PO (100 mg/kg) administration

Animal	Administration	Dose, mg/kg	Pharmacokinetic Parameters							
			Tmax, min	Cmax, ng/ml	$AUC_{0\rightarrow t=600min}$ (AUClast) ng*min/ml	$AUC_{0\rightarrow\infty}$ (AUCINF_obs), ng*min/ml	$T_{1/2}$ (HL_Lambda_z), min	K_{el} (Lambda_z), min^{-1}	MRT (MRTlast), min	MRT (MRTinf), min
Mice	PO	100	60.0	4310	1660000	2810000	431	0.00161	253	651

Figure S9. Plasma concentration-time curve of compound MU1700.2HCL in male Balb/cAnN mice following PO (100 mg/kg) administration (n=4)

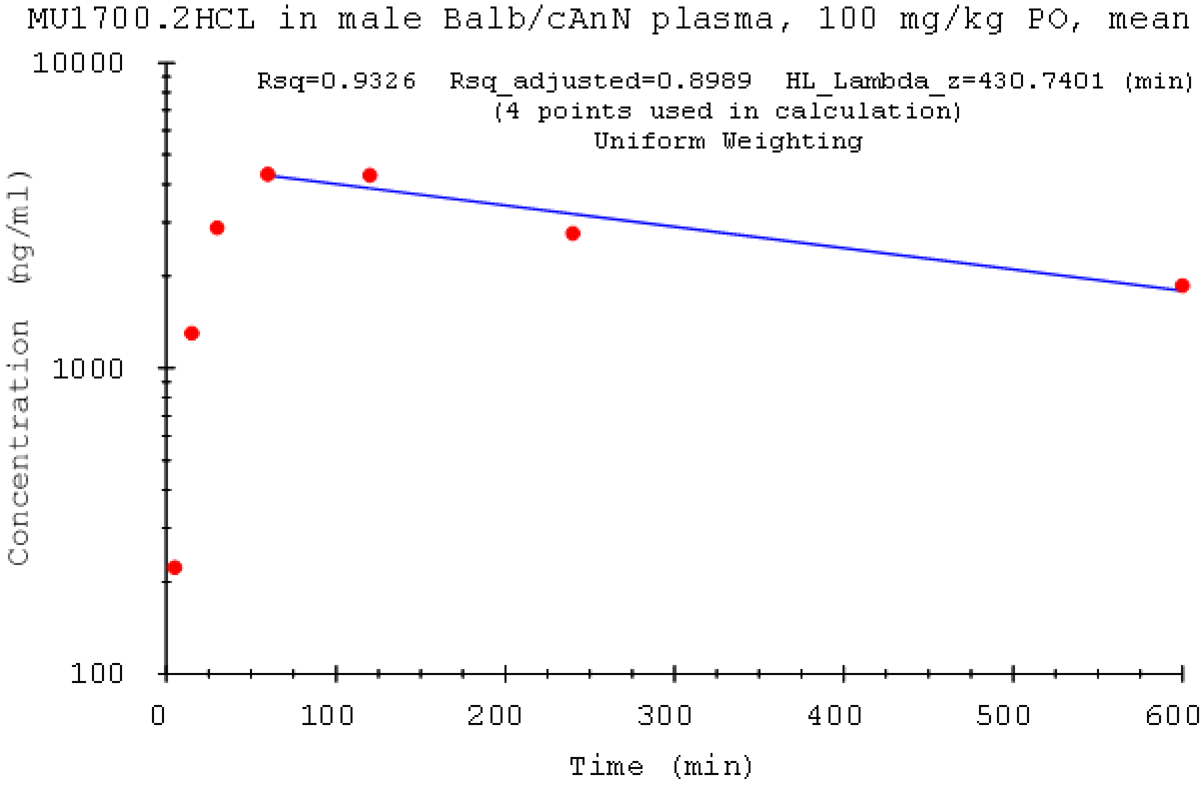

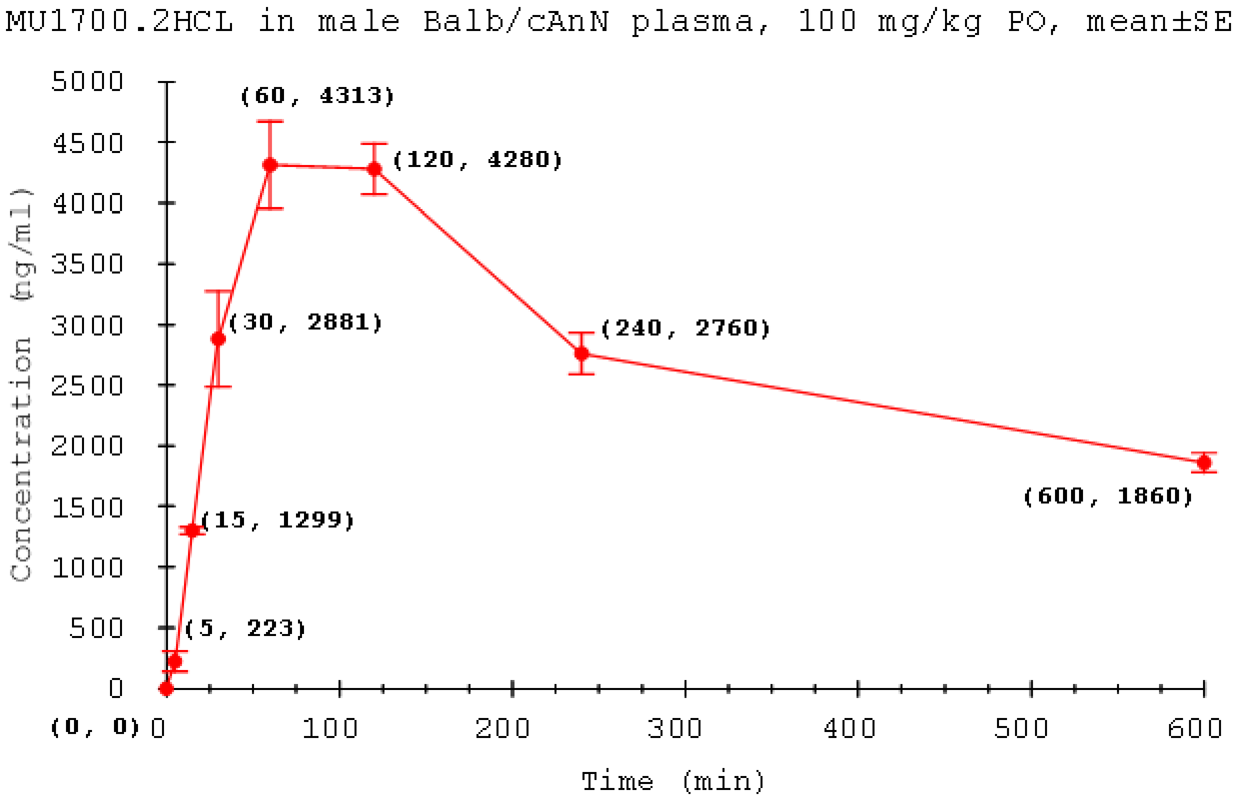
MU1700.2HCL in male Balb/cAnN plasma, 100 mg/kg PO, mean±SE
Concentration (ng/ml)
Time (min)
(0, 0)
(5, 223)
(15, 1299)
(30, 2881)
(60, 4313)
(120, 4280)
(240, 2760)
(600, 1860)
0
500
1000
1500
2000
2500
3000
3500
4000
4500
5000
0
100
200
300
400
500
600

Table S17. Brain concentrations of compound MU1700.2HCL in male Balb/cAnN mice following PO (100 mg/kg) administration

Sample collection time point, min	Brain concentration (ng/g)						
	**Mouse A**	**Mouse B**	**Mouse C**	**Mouse D**	**Mean**	**SD**	**SE**
0	BQL				**BQL**	BQL	BQL
5	128	64	37	45	**68**	41	21
15	2002	2557	2342	1890	**2198**	307	154
30	8540	14095	7345	4314	**8574**	4088	2044
60	21260	31950	21940	25940	**25273**	4907	2454
120	48410	62150	69400	79900	**64965**	13225	6613
240	64050	77600	65950	68250	**68963**	6009	3004
600	35855	41085	57850	46025	**45204**	9398	4699

BQL - Below the lower limit of quantitation (LLOQ)
ND - Not determined

Table S18. Selected pharmacokinetic parameters (brain) for compound MU1700.2HCL in male Balb/cAnN mice following PO (100 mg/kg) administration

Animal	Administration	Dose, mg/kg	Pharmacokinetic Parameters							
			Tmax, min	Cmax, ng/ml	$AUC_{0 \to t=600min}$ (AUClast) ng*min/ml	$AUC_{0 \to \infty}$ (AUCINF_obs), ng*min/ml	$T_{1/2}$ (HL_Lambda_z), min	K_{el} (Lambda_z), min^{-1}	MRT (MRTlast), min	MRT (MRTinf), min
Mice	PO	100	240	69000	31900000	70400000	591	0.00117	302	931

Figure S10. Brain concentration-time curve of compound MU1700.2HCL in male Balb/cAnN mice following PO (100 mg/kg) administration (n=4)

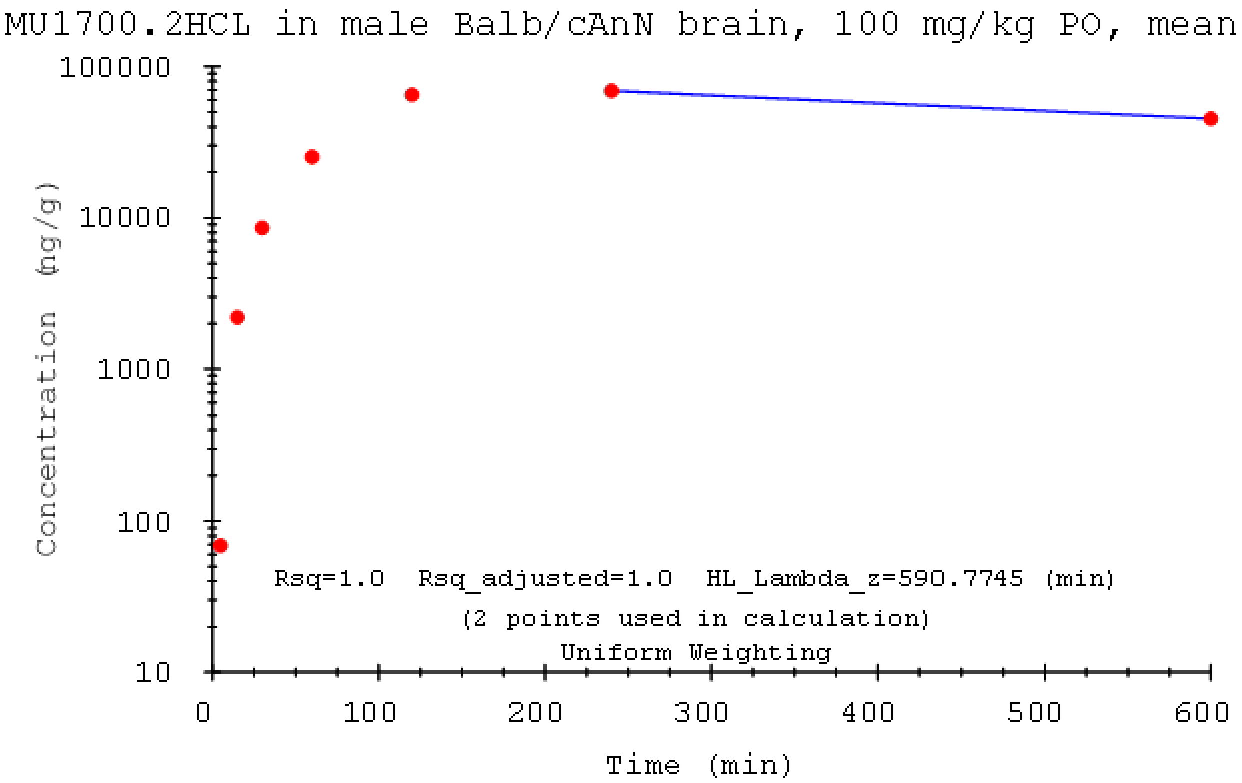

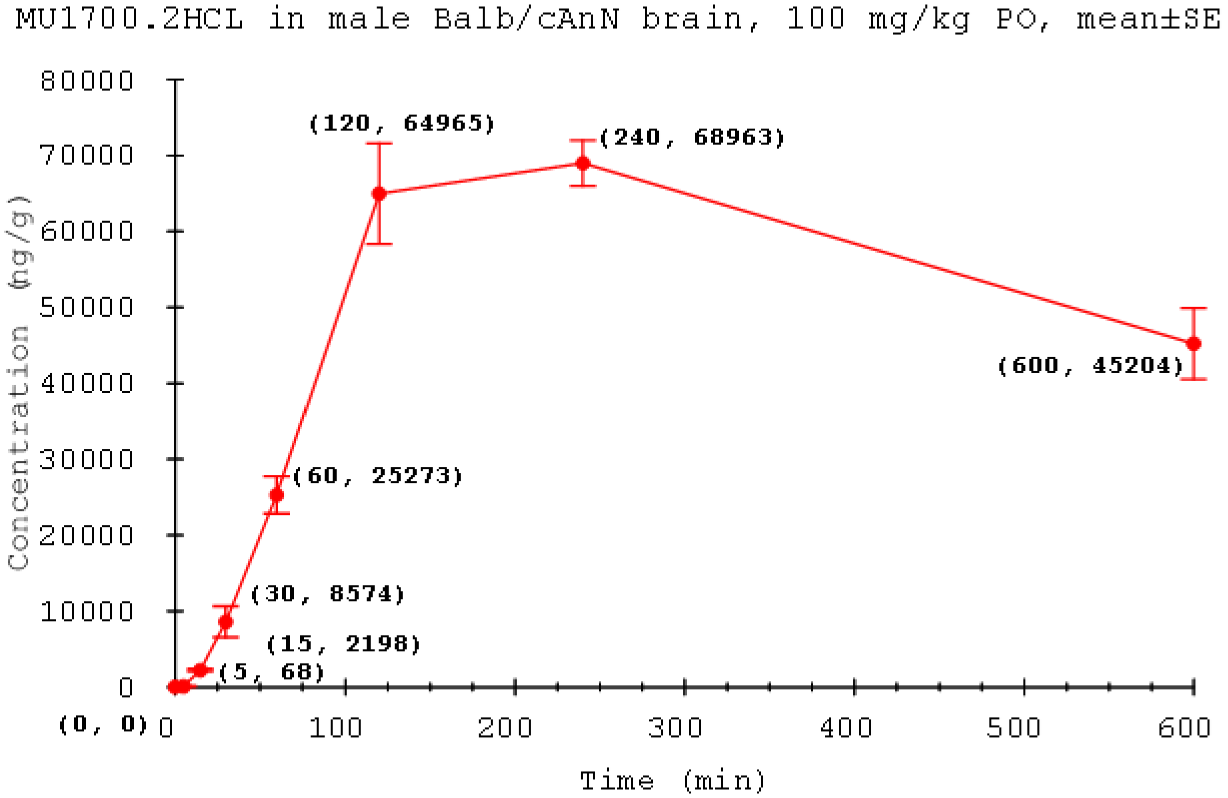

Summary: The pharmacokinetic parameters for **MU1700.2HCL** in blood plasma and brain are shown in the summary table below (Table S19). Figure 13 summarizes the results of PK study for compound **MU1700.2HCL** in mice.

No obvious adverse effects were observed during this PK study.

Table S19. Selected pharmacokinetic parameters for MU1700.2HCL in male Balb/cAnN mice

Sample	Administration	Dose, mg/kg	Pharmacokinetic Parameters							
			Tmax, min	Cmax, ng/ml(g)	AUC$_{0\to t\ min}$ (AUClast) ng*min/ml(g)	AUC$_{0\to\infty}$ (AUCINF_obs) ng*min/ml(g)	$T_{1/2}$ (HL_Lambda_z), min	K_{el} (Lambda_z), min^{-1}	MRT (MRTlast), min	MRT (MRTinf), min
Plasma	PO	100	60.0	4310	1660000	2810000	431	0.00161	253	651
Brain		100	240	69000	31900000	70400000	591	0.00117	302	931

Figure S11. Concentration-time curves for compound MU1700.2HCL in male Balb/cAnN mice following PO administration

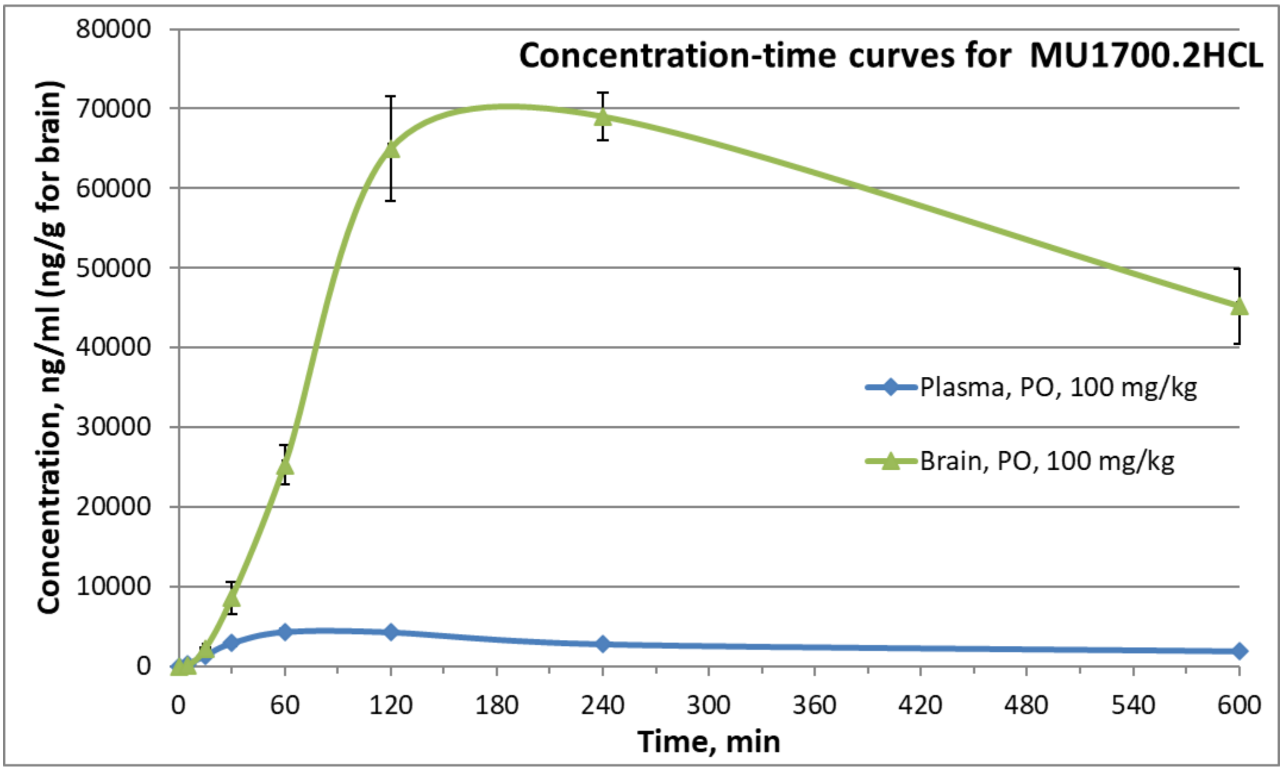

9. Determination of IC_{50} for *in vitro* CYP450 inhibition in human liver microsomes for MU1700

STUDY REPORT ADME051924j – BIENTA ENAMINE BIOLOGY SERVICES

Study Responsibilities

Study Director Yuliia Holota, Ph.D.

Scientist Lina Bortnichuk, M.S.

Study Objective

Assessment of IC_{50} for CYP450 (panel of 2C8) inhibition by **MU1700-2HCL** in human liver microsomes using LC-MS based assay.

Materials

Reagents and consumables

DMSO Chromasolv Plus, HPLC grade, ≥99.7% (Sigma-Aldrich, USA; Cat #34869)

Acetonitrile Chromasolv, HPLC grade, ≥99.9% (Sigma-Aldrich, USA; Cat #34851)

Methanol, HiPerSolv, HPLC grade, ≥99.9% (VWR Chemicals, USA, Cat #20864.320)

Potassium phosphate monobasic (Bio-Basic, Canada; Lot #N9016010)

Potassium phosphate dibasic (Bio-Basic, Canada; Lot #MA7100050)

Magnesium chloride hexahydrate (Santa Cruz Biotechnology, Inc., USA; Cat #sc-203126A)

NADPH tetrasodium salt (BLD Pharmatech Ltd., Cat #BD116582)

XTreme 200 Human Liver Microsomes: pooled, mixed gender (XenoTech, H2610/lot #1910096)

Human Liver Microsomes: pooled, mixed gender (XenoTech, H0630/lot #1210097)

Amodiaquine from Enamine stock

Montelukast from Enamine stock

Rac 8-Hydroxyefavirenz (Santa Cruz Biotechnology, Inc., USA; Cat #sc-208270)

Ticlopidine hydrochloride (Sigma-Aldrich, USA; Cat #T6654)

Agilent InfinityLab Poroshell 120 EC-C18, 2.1 x 50 mm, 4 µm (Cat #699770-902)

Matrix™ 0.75 ml blank tubes (Cat #4170), pipettor tips (Thermo Scientific).

Equipment

Gradient HPLC system (Shimadzu)

Triple quadrupole mass-detector API 4000 with Turbo V Ion Source (AB Sciex, Canada)
Nitrogen generator N2-04-L1466, nitrogen purity 99%+ (Whatman)
Incubator/Shaker Innova 4080 (New Brunswick Scientific, USA)
Water purification system Millipore Milli-Q Gradient A10 (Millipore, France)
Multichannel pipettors 1-30 μL, 2-125 μL, 30-850 μL (Thermo Scientific)

Analytical System

All measurements were performed using Shimadzu HPLC system including vacuum degasser, gradient pumps, reverse phase HPLC column, column oven, and autosampler. Mass spectrometric analysis was performed using a Triple quadrupole mass-detector API 4000 with Turbo V Ion Source (AB Sciex, Canada). The TurboIonSpray ion source was used in both positive and negative ion modes. The data acquisition and system control was performed using Analyst 1.6.3 software from AB Sciex.

Methods

The potential for CYP450 inhibition of cytochrome 2C8 was assessed using LC-MS/MS based assay, in which biotransformations of the CYP450 specific substrates (Table 1) were used as markers to quantify the enzymatic activity (Walsky et al, Drug metabolism and disposition, 2004, p647-660). The enzymatic reactions were performed under linear conditions with substrate concentration below Km values and conversion of substrate below 20%.

Table S20. Isoform-specific CYP450 substrates and metabolites

CYP450	Substrate	Km of substrate	Metabolite
2C8	Amodiaquine	1.9 uM	Desethylamodiaquine

General procedure. The incubations medium contained: test compounds (final DMSO concentration 0.15-0.2%), 50 and 100 mM phosphate buffer (pH 7.4), 3.3 mM $MgCl_2$, 1.3 mM NADPH and human liver microsomes (0.1-0.15 mg/ml). Control incubations were performed without NADPH. Test compounds were preincubated with enzyme and substrate at 37 °C during 10 min. The enzymatic reaction was initiated by addition of NADPH, the mixture was incubated at 37 °C during 9-40 min shaking at 100 rpm. Incubations were performed in duplicates. Incubations were terminated by addition of one volume of acetonitrile containing internal standard, followed by protein sedimentation by centrifuging at

5000 rpm for 5 minutes. Supernatants were analyzed using the HPLC system coupled with tandem mass spectrometer.

Validation procedure.

In CYP2C8 inhibition assay Amodiaquine dealkylation to Desethylamodiaquine was used as a marker to quantify the enzymatic activity. Montelukast, which was used as reference compound to assess inhibition of CYP2C8, exhibited IC_{50}= 107 uM corresponding to the literature data for microsomal CYP2C8 (Walsky et al, Drug metabolism and disposition, 2005, p 413-418).

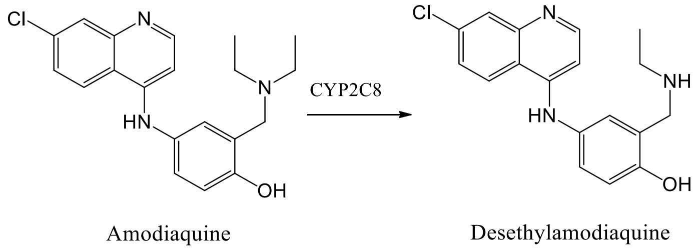

Scheme S1. Amodiaquine dealkylation by CYP2C8

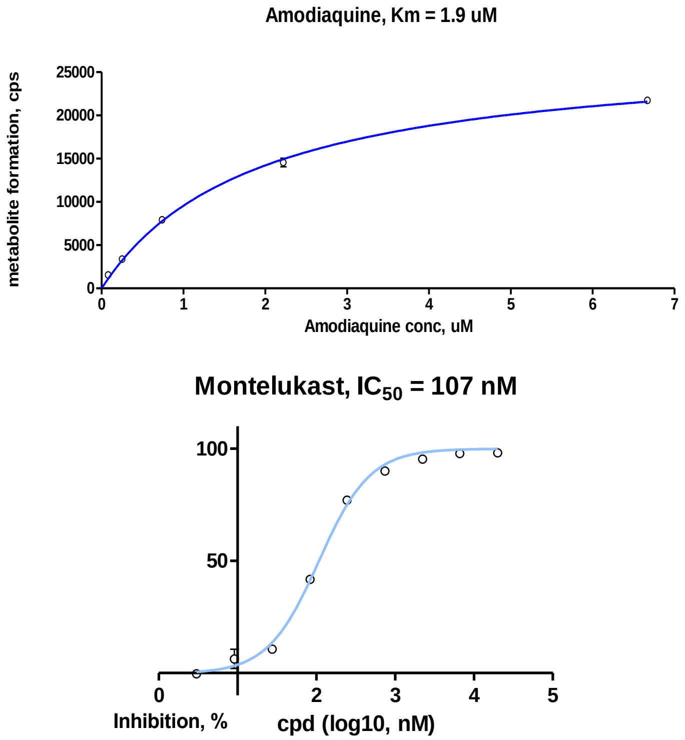

Figure S12. Determination of Km for CYP2C8 substrate Amodiaquine and IC_{50} for reference inhibitor Montelukast

Quantification of a decrease in the formation of a metabolite in the presence of an inhibitor was used to determine the CYP450 inhibition. Reference inhibitors specific for each CYP enzyme were used to assess inhibition in the control experiments for every batch of tested compounds. Test concentrations of the reference compounds corresponded to approximately 5x fold of IC_{50} values for each CYP450, which is expected to produce 80-100% inhibition in the properly performing assay (Table 2).

Table S21. Isoform-specific CYP450 inhibition by reference inhibitors

**CYP450**	**Reference inhibitor**	**Reference inhibitor concentration, 5x IC_{50}**	**CYP inhibition, %**
**2C8**	Montelukast	535 nM	**87**

The mean values and the log inhibitor vs. response (variable slope, three parameters; top "100" fitting constraints) function were calculated with Jupyter (IPython) Notebook web-based interactive applications using several open-source libraries. The data is presented as IC50 values. Please note that calculated IC50s may not always fully correctly reflect the actual potential inhibition properties, depending upon the complexity of the data, mechanism of action of the compounds, assay response range, and the fitting algorithm used by the program. A more precise interpretation of the data and full assessment of the inhibition potential for the test article may require arbitrary adjustment of curve fitting parameters, and assay parameters.

Results

CYP450 inhibition data for test article are shown in the tables below.

Table S22. CYP2C8 inhibition by **MU1700-2HCL** in HLM

Concentration of **MU1700-2HCL**, nM	$Log_{10}Conc$	Inhibition, %		Average Inhibition, %	SD
		1	2		
100000	5.00	54.0	57.8	**55.9**	2.7
33333.3	4.52	40.4	35.9	**38.2**	3.2
11111.1	4.05	21.9	22.8	**22.4**	0.6
3703.7	3.57	9.3	2.5	**5.9**	4.8
1234.6	3.09	-2.9	3.4	**0.2**	4.5
411.5	2.61	2.5	-5.2	**-1.4**	5.4
137.2	2.14	-5.6	-12.9	**-9.3**	5.1
45.7	1.66	1.6	3.4	**2.5**	1.3

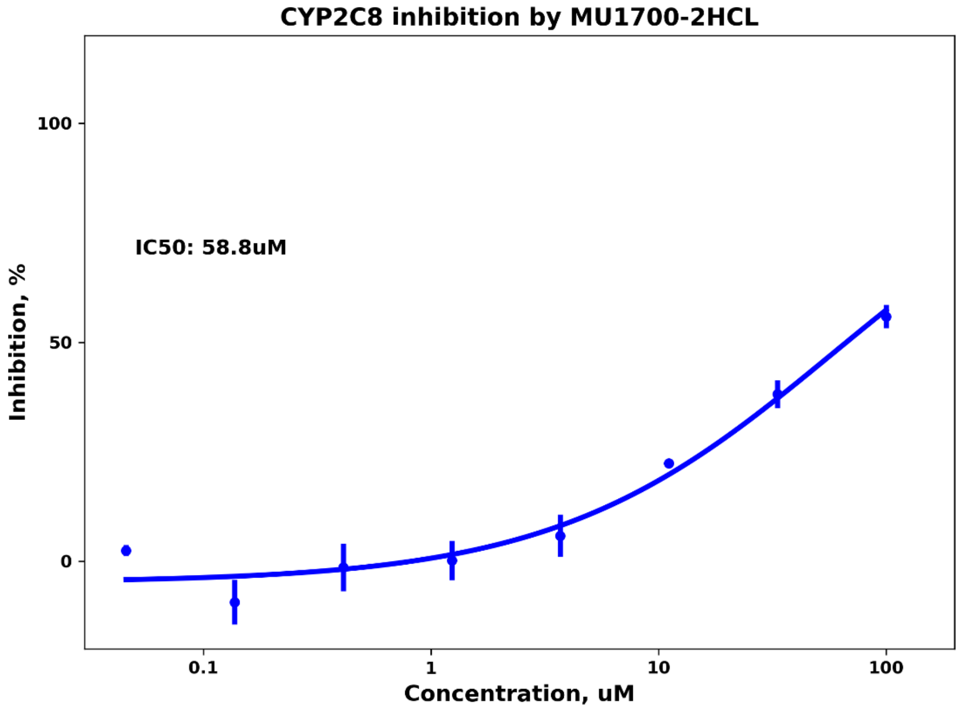

Figure S13. Inhibition of CYP2C8 by **MU1700-2HCL** in HLM

Study Responsibilities

Study Director Yuliia Holota, Ph.D.

Scientist Lina Bortnichuk, M.S.

Study Objective

Assessment of IC_{50} for CYP450 (panel of 4: CYP3A4, 2D6, 1A2, 2C9) inhibition by **MU1700-2HCL** in human liver microsomes using LC-MS based assay.

Materials

Reagents and consumables

DMSO Chromasolv Plus, HPLC grade, ≥99.7% (Sigma-Aldrich, USA; Cat #34869)
Acetonitrile Chromasolv, HPLC grade, ≥99.9% (Sigma-Aldrich, USA; Cat #34851)
Methanol, HiPerSolv, HPLC grade, ≥99.9% (VWR Chemicals, USA, Cat #20864.320)
Potassium phosphate monobasic (Bio-Basic, Canada; Lot #N9016010)
Potassium phosphate dibasic (Bio-Basic, Canada; Lot #MA7100050)
Magnesium chloride hexahydrate (Santa Cruz Biotechnology, Inc., USA; Cat #sc-203126A)
NADPH tetrasodium salt (BLD Pharmatech Ltd., Cat #BD116582)
XTreme 200 Human Liver Microsomes: pooled, mixed gender (XenoTech, H2610/lot #1910096)
Human Liver Microsomes: pooled, mixed gender (XenoTech, H0630/lot #1210097)
Testosterone from Enamine stock
6β-Hydroxy Testosterone (Corning, Cat #451012)
Ketoconazole (Sigma-Aldrich, USA; Cat #K1003)
Dextromethorphan hydrobromide monohydrate (Sigma-Aldrich, USA; Cat #D2531)
Dextrorphan tartrate (Sigma-Aldrich, USA; Cat #D127)
Quinidine (Sigma-Aldrich, USA; Cat #Q3625)
4-Hydroxy Mephenytoin (Cayman Chemical, Cat #16124)
S-Mephenytoin (Santa Cruz Biotechnology, Inc., USA; Cat #sc-200975)
Tranylcypromine from Enamine stock
Diclofenac from Enamine stock
4-Hydroxy diclofenac (Conning, Cat #45174)
Sulfaphenazole (Sigma-Aldrich, USA; Cat #S0758)

Phenacetin from Enamine stock

Acetaminophen from Enamine stock

Furafylline (Abcam, Cat #ab146021)

Agilent InfinityLab Poroshell 120 EC-C18, 2.1 x 50 mm, 4 μm (Cat #699770-902)

Matrix™ 0.75 ml blank tubes (Cat #4170), pipettor tips (Thermo Scientific).

Equipment

Gradient HPLC system (Shimadzu)

Triple quadrupole mass-detector API 4000 with Turbo V Ion Source (AB Sciex, Canada)

Nitrogen generator N2-04-L1466, nitrogen purity 99%+ (Whatman)

Incubator/Shaker Innova 4080 (New Brunswick Scientific, USA)

Water purification system Millipore Milli-Q Gradient A10 (Millipore, France)

Multichannel pipettors 1-30 μL, 2-125 μL, 30-850 μL (Thermo Scientific)

Analytical System

All measurements were performed using Shimadzu HPLC system including vacuum degasser, gradient pumps, reverse phase HPLC column, column oven, and autosampler. Mass spectrometric analysis was performed using a Triple quadrupole mass-detector API 4000 with Turbo V Ion Source (AB Sciex, Canada). The TurboIonSpray ion source was used in both positive and negative ion modes. The data acquisition and system control was performed using Analyst 1.6.3 software from AB Sciex.

Methods

The potential for CYP450 inhibition of 4 major cytochromes 3A4, 2D6, 1A2, and 2C9 was assessed using LC-MS/MS based assay, in which biotransformations of the CYP450 specific substrates (Table 1) were used as markers to quantify the enzymatic activity (Walsky et al, Drug metabolism and disposition, 2004, p647-660). The enzymatic reactions were performed under linear conditions with substrate concentration below Km values and conversion of substrate below 20%.

Table S23. Isoform-specific CYP450 substrates and metabolites

CYP450	Substrate	Km of substrate	Metabolite
1A2	Phenacetin	60 uM	Acetaminophen
3A4	Testosterone	56 uM	6β-Hydroxy Testosterone
2C9	Diclofenac	12 uM	4’-Hydroxydiclofenac
2D6	Dextromethorphan	5.6 uM	Dextrorphan

General procedure. The incubations medium contained: test compounds (final DMSO concentration 0.15-0.2%), 50 and 100 mM phosphate buffer (pH 7.4), 3.3 mM $MgCl_2$, 1.3 mM NADPH and human liver microsomes (0.1-0.15 mg/ml). Control incubations were performed without NADPH. Test compounds were preincubated with enzyme and substrate at 37 °C during 10 min. The enzymatic reaction was initiated by addition of NADPH, the mixture was incubated at 37 °C during 9-40 min shaking at 100 rpm. Incubations were performed in duplicates. Incubations were terminated by addition of one volume of acetonitrile containing internal standard, followed by protein sedimentation by centrifuging at 5000 rpm for 5 minutes. Supernatants were analyzed using the HPLC system coupled with tandem mass spectrometer.

Validation procedure. In CYP3A4 inhibition assay Testosterone 6-hydroxylation was used as a marker to quantify the CYP3A4 activity. Testosterone is a substrate of CYP3A4, metabolized to 6β-Hydroxy Testosterone, which was quantified by LC-MS. Ketoconazole, which was used as reference compound to assess CYP3A4 inhibition, exhibited IC_{50} = 69 nM in this assay, which is consistent with the literature data for microsomal CYP3A4 (Fig. 1).

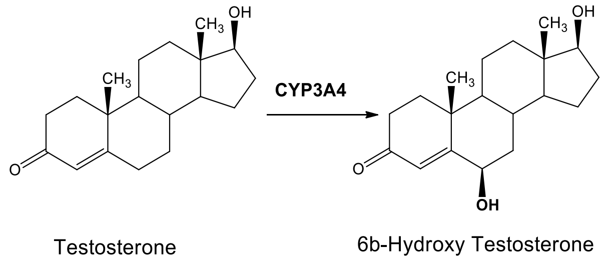

Scheme S2. Hydroxylation of Testosterone by CYP3A4

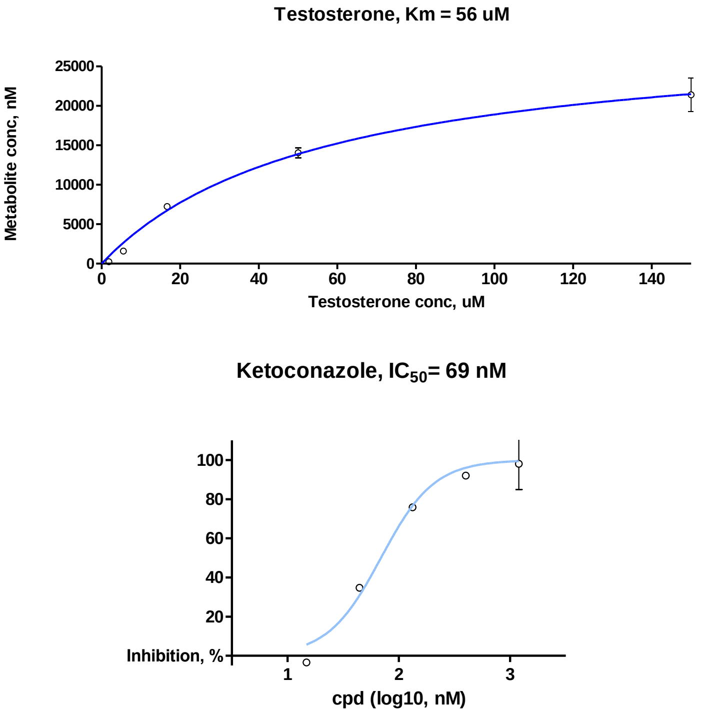

Figure S14. Determination of Km for CYP3A4 substrate Testosterone and IC_{50} for reference inhibitor Ketoconazole.

In CYP2D6 inhibition assay Dextromethorphan demethylation was used as a marker to quantify the CYP2D6 activity. Dextromethorphan is a substrate of CYP2D6, metabolized to Dextrorphan, which was quantified by LC-MS. Quinidine was used as reference compound to assess CYP2D6 inhibition. In this assay Quinidine exhibited IC_{50} = 45 nM (Fig. 2).

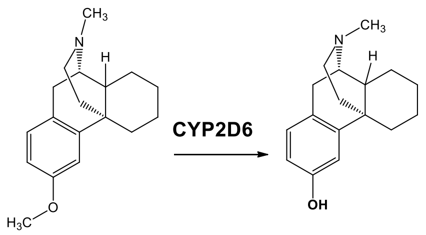

Dextromethorphan Dextrorphan

Scheme S3. Dextromethorphan demethylation by CYP2D6

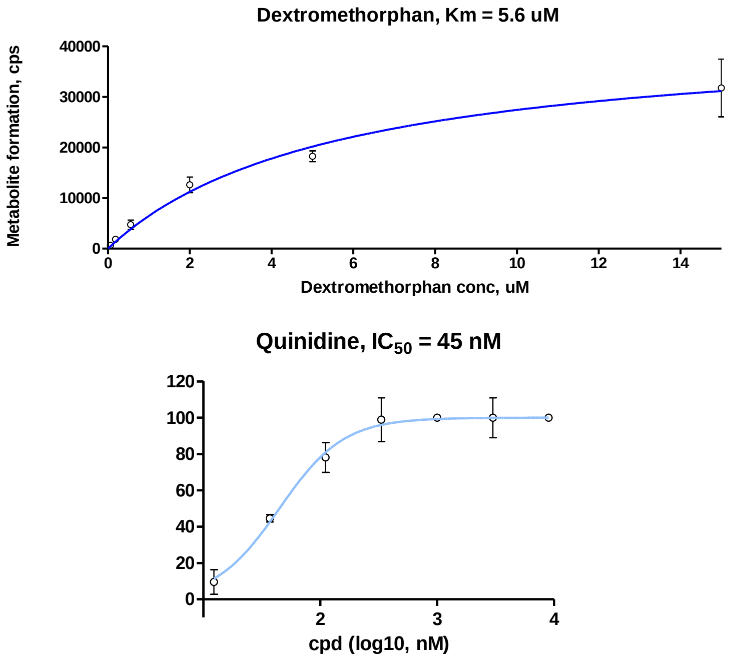

Figure S15. Determination of Km for CYP2D6 substrate Dextromethorphan and IC_{50} for reference inhibitor Quinidine.

Phenacetin O-deethylation was used as a marker to quantify the CYP1A2 activity. Product of this reaction Acetaminophen was quantified by LC-MS (Fig. 3). Reference inhibitor Furafylline exhibited IC_{50} = 3 uM in this assay.

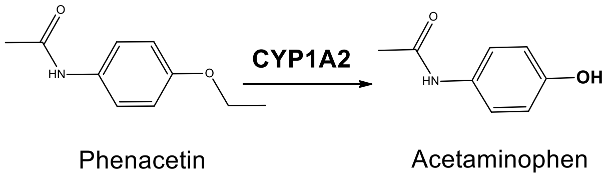

Scheme S4. Phenacetin O-deethylation by CYP1A2

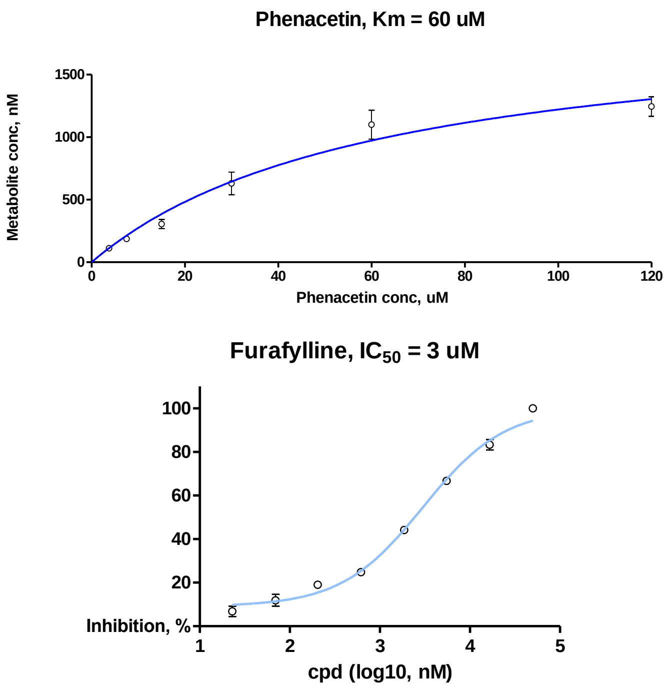

Figure S16. Determination of Km for CYP1A2 substrate Phenacetin and IC_{50} for reference inhibitor Furafylline.

In CYP2C9 inhibition assay Diclofenac 4'-hydroxylation was used as a marker to quantify the CYP2C9 activity. Sulphaphenazole was used as reference compound to assess inhibition of CYP2C9 (Fig. 5).

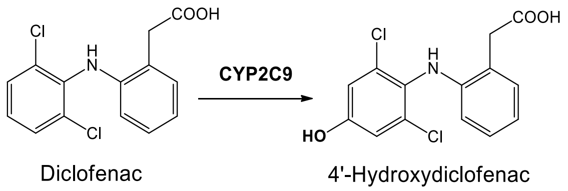

Scheme S5. Hydroxylation of Diclofenac by CYP2C9

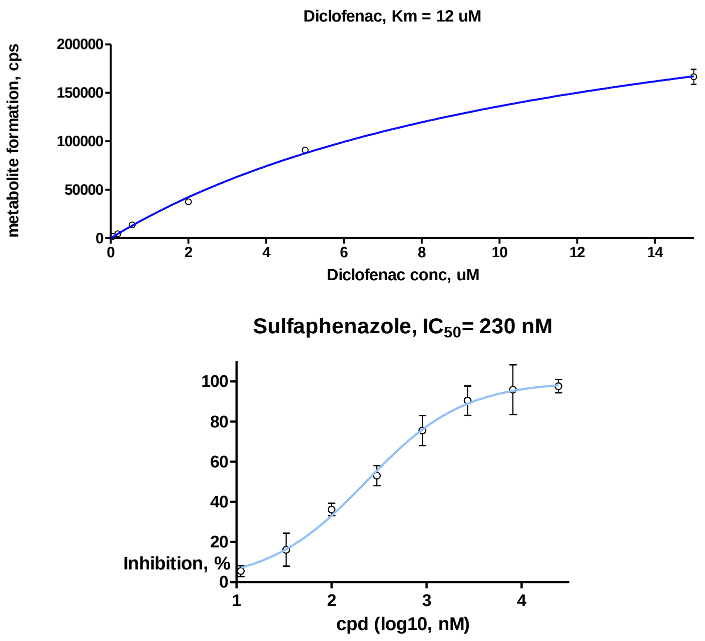

Figure S17. Determination of Km for CYP2C9 substrate Diclofenac and IC_{50} for reference inhibitor Sulphaphenazole.

Quantification of a decrease in the formation of a metabolite in the presence of an inhibitor was used to determine the CYP450 inhibition. Reference inhibitors specific for each CYP enzyme were used to assess inhibition in the control experiments for every batch of tested compounds. Test concentrations of the reference compounds corresponded to approximately 5x fold of IC_{50} values for each CYP450, which is expected to produce 80-100% inhibition in the properly performing assay (Table S24).

Table S24. Isoform-specific CYP450 inhibition by reference inhibitors

**CYP450**	**Reference inhibitor**	**Reference inhibitor concentration, 5x IC_{50}**	**CYP inhibition, %**
**3A4**	Ketoconazole	345 nM	**97**
**3A4**	Ketoconazole	345 nM	**96**
**2D6**	Quinidine	225 nM	**84**
**2D6**	Quinidine	225 nM	**83**
**1A2**	Furafylline	15 uM	**87**
**1A2**	Furafylline	15 uM	**88**
**2C9**	Sulphaphenazole	1150 nM	**80**
**2C9**	Sulphaphenazole	1150 nM	**82**

The mean values and the log inhibitor vs. response (variable slope, three parameters; top "100" fitting constraints) function were calculated with Jupyter (IPython) Notebook web-based interactive applications using several open-source libraries. The data is presented as IC50 values. Please note that calculated IC50s may not always fully correctly reflect the actual potential inhibition properties, depending upon the complexity of the data, mechanism of action of the compounds, assay response range, and the fitting algorithm used by the program. A more precise interpretation of the data and full assessment of the inhibition potential for the test article may require arbitrary adjustment of curve fitting parameters, and assay parameters.

Results

CYP450 inhibition data for test articles are shown in the tables below.

Table S25. CYP3A4 inhibition by **MU1700-2HCL** in HLM

Concentration of **MU1700-2HCL**, nM	$Log_{10}Conc$	Inhibition, %		Average Inhibition, %	SD
		1	2		
100000	5.00	55.6	55.5	**55.6**	0.0
33333.3	4.52	54.8	51.5	**53.2**	2.3
11111.1	4.05	39.7	37.8	**38.7**	1.3
3703.7	3.57	18.3	15.4	**16.9**	2.0
1234.6	3.09	5.9	5.5	**5.7**	0.3
411.5	2.61	2.6	-1.2	**0.7**	2.7
137.2	2.14	0.2	0.2	**0.2**	0.0
45.7	1.66	0.2	-3.1	**-1.4**	2.4

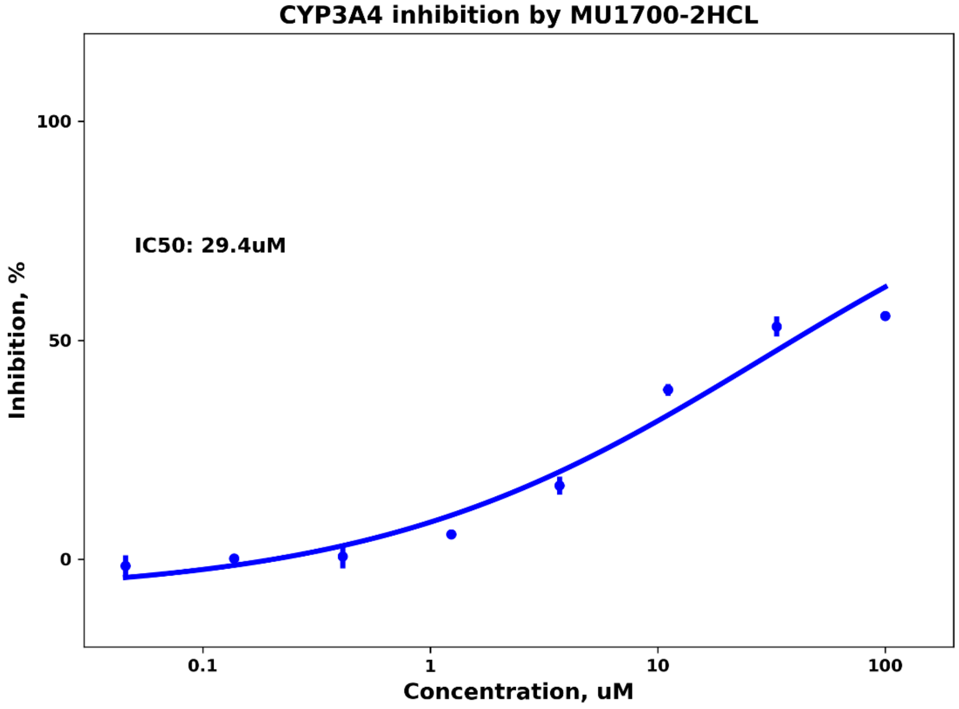

Figure S18. Inhibition of CYP3A4 by **MU1700-2HCL** in HLM

Table S26. CYP2D6 inhibition by **MU1700-2HCL** in HLM

Concentration of **MU1700-2HCL**, nM	$Log_{10}Conc$	Inhibition, %		Average Inhibition, %	SD
		1	2		
100000	5.00	39.9	39.9	**39.9**	0.0
33333.3	4.52	30.3	30.8	**30.5**	0.4
11111.1	4.05	24.3	23.4	**23.9**	0.6
3703.7	3.57	16.9	15.3	**16.1**	1.2
1234.6	3.09	15.4	10.4	**12.9**	3.5
411.5	2.61	12.9	8.9	**10.9**	2.8
137.2	2.14	14.8	13.9	**14.3**	0.6
45.7	1.66	14.3	10.8	**12.5**	2.5

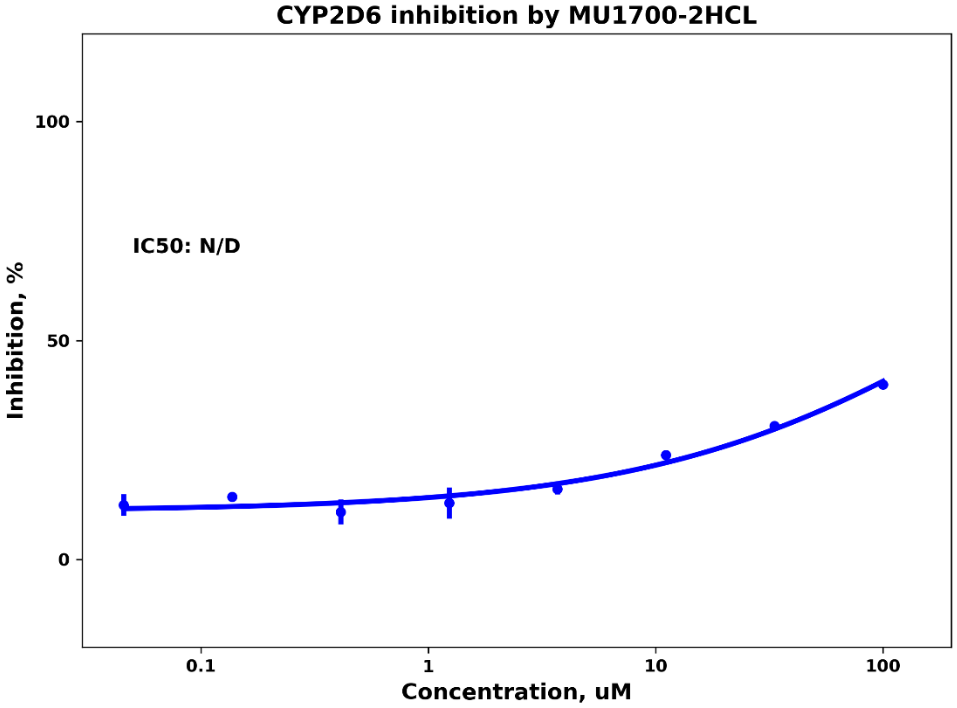

Figure S19. Inhibition of CYP2D6 by **MU1700-2HCL** in HLM

Table S27. CYP1A2 inhibition by **MU1700-2HCL** in HLM

Concentration of **MU1700-2HCL**, nM	$Log_{10}Conc$	Inhibition, %		Average Inhibition, %	SD
		1	2		
100000	5.00	38.3	40.1	**39.2**	1.3
33333.3	4.52	36.7	32.0	**34.3**	3.3
11111.1	4.05	15.6	18.0	**16.8**	1.7
3703.7	3.57	10.6	12.4	**11.5**	1.3
1234.6	3.09	11.6	11.2	**11.4**	0.3
411.5	2.61	6.7	7.1	**6.9**	0.3
137.2	2.14	8.3	7.1	**7.7**	0.8
45.7	1.66	2.2	4.1	**3.2**	1.4

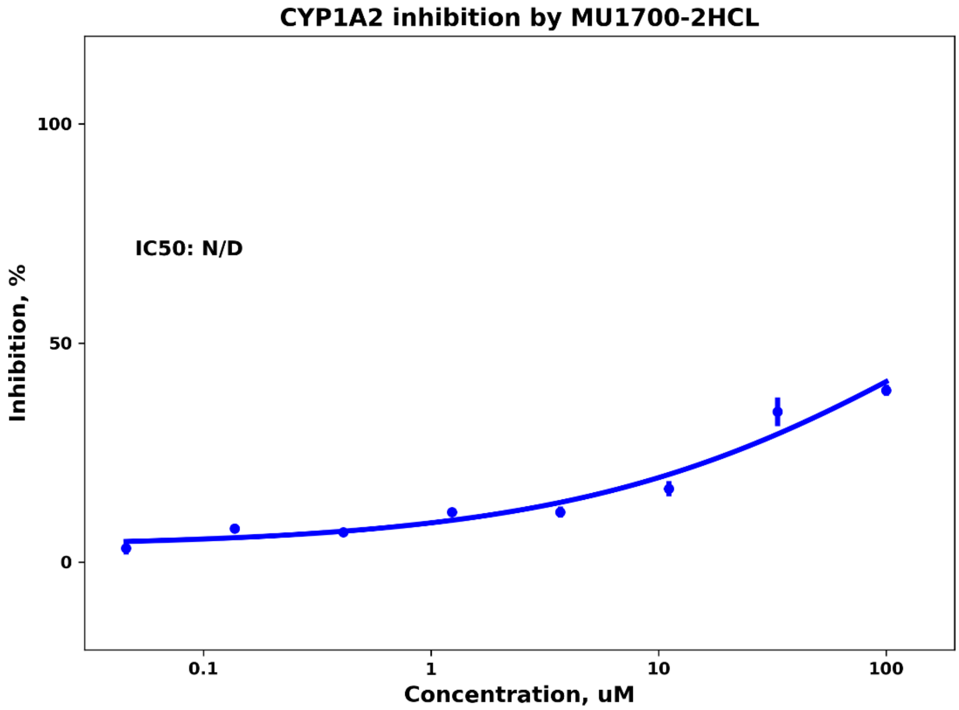

Figure S20. Inhibition of CYP1A2 by **MU1700-2HCL** in HLM

Table S28. CYP2C9 inhibition by **MU1700-2HCL** in HLM

Concentration of **MU1700-2HCL**, nM	$Log_{10}Conc$	Inhibition, %		Average Inhibition, %	SD
		1	2		
100000	5.00	49.5	43.1	**46.3**	4.5
33333.3	4.52	39.7	31.8	**35.8**	5.5
11111.1	4.05	30.4	26.4	**28.4**	2.8
3703.7	3.57	12.0	11.4	**11.7**	0.4
1234.6	3.09	11.6	4.6	**8.1**	5.0
411.5	2.61	7.8	1.4	**4.6**	4.5
137.2	2.14	11.0	-2.0	**4.5**	9.2
45.7	1.66	-1.4	-2.2	**-1.8**	0.6

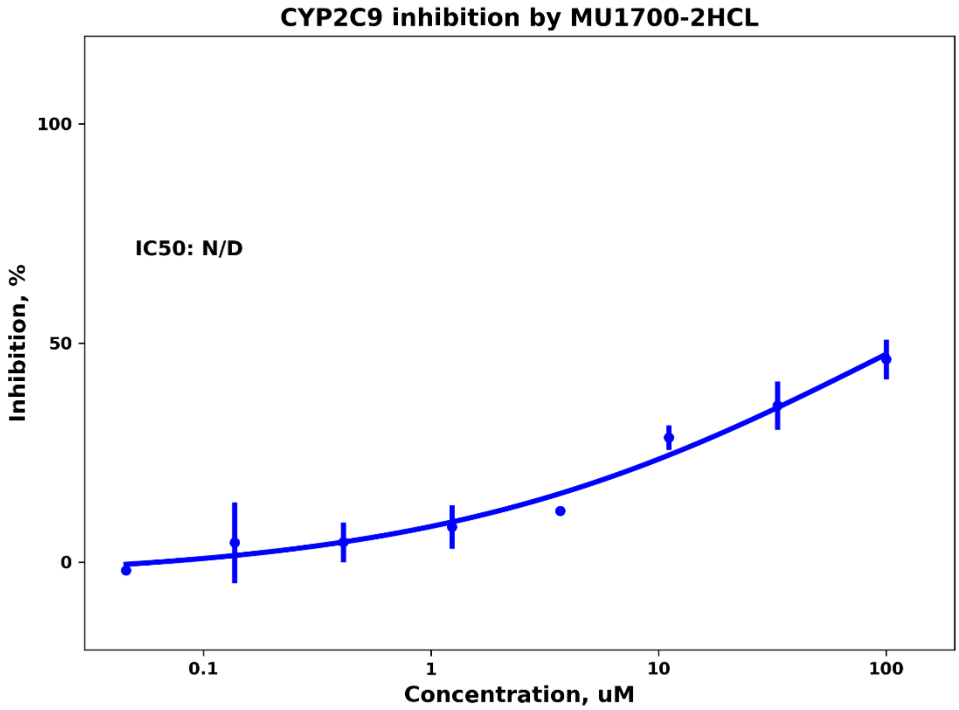

Figure S21. Inhibition of CYP2C9 by **MU1700-2HCL** in HLM

10. Identification of hERG Potassium Channel Inhibition

STUDY REPORT ADME050224e – BIENTA ENAMINE BIOLOGY SERVICES

Study responsibilities

Study Director	Y. Holota, Ph.D.
Scientist	A. Rodnichenko, Ph.D.
	L. Dmytrovska, M.Sc.

Study objective

The purpose of this study was to test the potential inhibition properties of the compound **MU1700** to hERG (human Ether-a-go-go Related Gene) potassium channel in the Tl Flux FLIPR-Based Assay. The assay provides valuable information about the possible blocking of the potassium channel with the test compound and a potential QT prolongation on the Electrocardiogram.

Materials and methods

Reagents and consumables

Centrifuge Tubes, 50 ml (Santa Cruz, USA; Cat# sc-200251)
Poly-D-Lysine-coated 384-well microplates (Corning, Cat#356663)
Disposable pipettor tips (Thermo Scientific, Fisherbrand, Eppendorf USA)
DMEM/F-12 with L-glutamine (HyClone, Cat# SH30004.04)
DMSO Chromasolv Plus, HPLC grade, ≥99.7% (Sigma-Aldrich, USA; Cat# 34869)
Dulbecco's PBS (1x) without Ca & Mg (PAA, UK; Cat# H15-002)
Fetal Bovine Serum (Sigma, Cat #F7524)
Flipr 384 Pipette Tips (Molecular Devices, Cat# 9000-0512)
Brilliant Thallium Flex (ION BIOSCIENCES, Cat# 11000-10)
G418 (Gibco, Cat#10131-035)
Haloperidol (Enamine, Ukraine)
Penicillin/Streptomycin (100x) (PAA, UK; Cat# P11-010)
Serological Pipettes 5 ml, 10 ml, 25 ml (Greiner Bio-One)
Trypsin-EDTA (10x) in PBS (PAA, Cat# L11-003)
Tissue Culture Treated T-75 Flask (UltraCrus, Cat# SC-200263)

Equipment

Centrifuge 5804R (Eppendorf, USA)
Cell culture CO_2 incubator, model CCL-170B-8 (ESCO, Singapore)
Etched Hemacytometer, dark line counting chamber (Hausser Scientific, USA; Cat#3500)

FLIPR Tetra High Throughput Cellular Screening System (Molecular Devices Corp., USA)
Labculture Biological Safety Cabinet, Class II, Type A2 (ESCO)
Inverted Microscope, Model CK2 (Olympus Optical Co., Ltd., Japan)
Microscope Leica DM LS2 (Leica Microsystems Wetzlar GmbH, Germany)
Multichannel manual pipette (Thermo Labsystems Finnpipette, FA16-50R)
PIPETMAN pipettes 2-20 µl, 50-200 µl, 200-1000 µl (Gilson, USA)
Water purification system Millipore Milli-Q Gradient A10 (Millipore, France)

Thallium flux FLIPR-Based Assay

HEK293-hERG cells (stably expressing human potassium channel hERG) were cultured in DMEM/F-12 medium supplemented with 10% FBS, 100 U/mL penicillin, 100 ug/mL streptomycin, and 500 ug/mL G418. The assay was performed according to the manufacturer's instructions. Cells were seeded into 384-well poly-D-lysine, clear-bottom microtiter plate (8000 cells/well) in DMEM/F-12 medium supplemented with 5% FBS, 100 U/mL penicillin, 100 ug/mL streptomycin 24 h prior to assay. After 24 h of cell growth, the conditioned medium was substituted with the Dye Loading Solution comprising thallium-sensitive dye followed by incubation for 1 hour in a CO_2 incubator. After the incubation, Dye Loading Solution was substituted with a 15 uL/well mixture of the Assay Buffer and the TRS. Then, 5 uL of the test compound diluted in the assay buffer (0.5% DMSO final concentration) was added to each well of the plate and incubated at room temperature for 30 minutes. hERG inhibition by test compound at the final concentrations ranging from 0.045 µM to 100.0 µM (8 points, 3-fold serial dilutions). All test points were performed in quadruplicates. Then, the Stimulation buffer containing Thallium and Potassium was added to each well and the intracellular fluorescence was measured in a kinetic assay at 470-495 nm excitation and 515-575 emission filter set (every 3 seconds for 3 minutes). DMSO was used as a negative control, and Haloperidol was used as a reference compound to verify the assay validity. Haloperidol was dissolved in 100% DMSO at a concentration of 20 mM and stored at -20°C (stored no more than a month). Haloperidol was assessed at the final concentrations ranging from 0.023 µM to 50.0 µM (8 points, 3-fold serial dilutions). The test compounds were dissolved in 100% DMSO at a concentration of 20 mM and stored at -20°C until use.

Experimental setup parameters for FLIPR Tetra

Exposure (sec): 0.4
Camera Gain: 110
Dispensing Volume (µL): 5

Excitation LED (nm): 470-495
Emission Filter (nm): 515-575
Intensity (%): 50
Height (μL): 10
Dispensing Speed (μL/sec): 10
Tip Up Speed (mm/sec): 10
Kinetic reduction: Area under the curve
Time course: 180 seconds, with stimulation buffer added 30 seconds after the start of reading.

Data analysis

The area under the curve (AUC) for each well was analyzed using Molecular Devices ScreenWorks® System Control Software (Molecular Devices). The median values and the log inhibitor vs. response (variable slope, four parameters) function were calculation with Jupyter (IPython) Notebook web-based interactive applications using several open-source libraries. The data is presented as IC_{50} values. Please note that calculated IC_{50s} shown in Table 1 may not always fully correctly reflect the actual potential inhibition properties, depending upon the complexity of the data, mechanism of action of the compounds, assay response range and fitting algorithm used by the program. More precise interpretation of the data and full assessment of the inhibition potential for test article may require arbitrary adjustment of curve fitting parameters, assay parameters.

Results

IC_{50} values of hERG channel activity inhibition for test and reference compounds are presented in Table 1. The dose-response curves for the reference compound Haloperidol and test compound are shown in Figures 1-2.

Table S29. IC_{50} values for test and reference compounds

Compound	IC50 value, μM
Haloperidol	**0.99**
MU1700	**N/D**

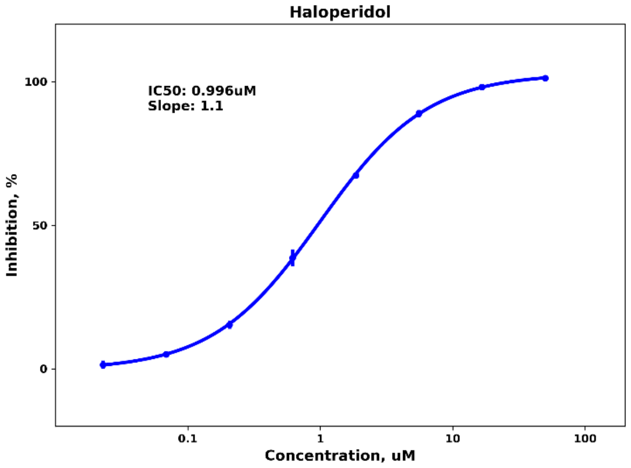

Figure S22. Haloperidol dose-response curve. The concentration range of 0.023 - 50 μM (8 points, 3-fold serial dilutions)

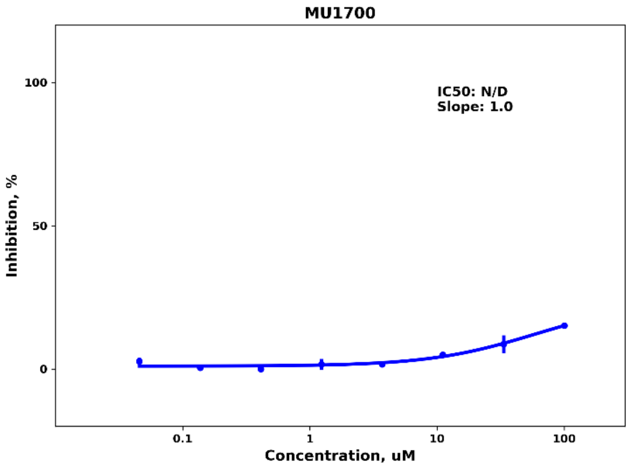

Figure S23. Dose-response curve for **MU1700**. Concentration range of 0.045 – 100 μM (8 points, 3-fold serial dilutions)

Conclusion: The test compound **MU1700** exhibited no significant inhibition of the hERG potassium channel. The IC50 values for this compound cannot be defined. The observed IC_{50} value of the control ligand (Haloperidol) was consistent with the literature data (Huang, Xi-Ping, et al. "Identification of human Ether-a-go-go related gene modulators by three screening platforms in an academic drug-discovery setting." Assay and drug development technologies 8.6 (2010): 727-742).

11. Assessment of Metabolic Stability in Human and Mouse Liver Microsomes for MU1700

STUDY REPORT ADME051924i – BIENTA ENAMINE BIOLOGY SERVICES

Study Responsibilities

Study Director	Yuliia Holota, Ph.D.
Scientists	Anna Pashchenko, B.Sc.;
	Mariia Pavlichenko, B.S.

Study Objective

The objective of this study was to determine metabolic stability of **MU1700** and reference compounds in human and mouse liver microsomes at five time points over 90 minutes using HPLC-MS. Metabolic stability is defined as the percentage of parent compound lost over time in the presence of a metabolically active test system.

Materials

Reagents and consumables

DMSO Chromasolv Plus, HPLC grade, ≥99.7% (Sigma-Aldrich, USA; Cat# 34869)

Acetonitrile Chromasolv, gradient grade, for HPLC, ≥99.9% (Sigma-Aldrich, USA; Cat# 34851)

Methanol, HiPerSolv, HPLC-gradient grade, ≥99.9% (VWR Chemicals, USA, Cat# 20864.320)

Potassium phosphate monobasic (Bio-Basic, Canada; Lot #N9016010)

Potassium phosphate dibasic (Bio-Basic, Canada; Lot #MA7100050)

Magnesium chloride hexahydrate (Santa Cruz Biotechnology, Inc., USA; sc-203126A)

Human Liver Microsomes: pooled, mixed gender (XenoTech, H0630/lot N#2110263)

Mouse Liver Microsomes: pooled, male Balb/c mice (XenoTech, M3000/lot #2010026)

Glucose-6-phosphate dehydrogenase from baker's yeast, type XV (Sigma-Aldrich, USA; Cat #G6378)

D-Glucose-6-phosphate monosodium salt (EMD Milipore Corp., USA; Cat #346764-5GM)

NADPH tetrasodium salt (BLD Pharmatech Ltd., Cat #BD116582)

Formic acid (Sigma-Aldrich, 94318)

Verapamil hydrochloride (Sigma Aldrich, USA; Cat #V4629)

Niclosamide (Sigma-Aldrich, USA; Cat #N3510)

DMSO stock solutions of the tested compounds 20mM

(+,-) Propranolol hydrochloride (Sigma-Aldrich, P0884)

Imipramine hydrochloride (Sigma-Aldrich, I7379)

Diclofenac sodium salt (Sigma-Aldrich, D6899)

InfinityLab Poroshell 120 EC-C18, 2.1 x 50 mm, 4 μm (Cat#699770-902)

Matrix™ 0.75 ml blank tubes (Cat #4170), pipettor tips (Thermo Scientific).

Equipment

Gradient HPLC system (Shimadzu)

Triple quadrupole mass-detector API 3000 with TurboIonSpray Ion Source (AB Sciex, Canada)

Nitrogen generator N2-04-L1466, nitrogen purity 99%+ (Whatman)

Environmental Incubator Shaker G24; Digital Refrigerated Incubator/Shaker Innova 4330 (New Brunswick Scientific)

Water purification system Millipore Milli-Q Gradient A10 (Millipore, France)

Multichannel pipettors 1-30 μL, 2-125 μL, 30-850 μL (Thermo Scientific)

Analytical System

All measurements were performed using Shimadzu HPLC system including vacuum degasser, gradient pumps, reverse phase HPLC column, column oven, and autosampler. Mass spectrometric analysis was performed using an API 3000 mass spectrometer from Applied Biosystems/ MDS Sciex (AB Sciex) with TurboIonspray interface. The TurboIonSpray ion source was used in both positive and negative ion modes. The data acquisition and system control was performed using Analyst 1.6.3 software from AB Sciex.

Methods

Microsomal incubations were carried out in 96-well plates in 5 aliquots of 30 μL each (one for each time point). Liver microsomal incubation medium comprised of phosphate buffer (100 mM, pH 7.4), $MgCl_2$ (3.3 mM), NADPH (3 mM), glucose-6-phosphate (5.3 mM), glucose-6-phosphate dehydrogenase (0.67 units/ml) with 0.42 mg of liver microsomal protein per ml. In the control reactions, the NADPH-cofactor system was substituted with phosphate buffer. Test compounds (2 μM, final acetonitrile concentration 1.6 %) were incubated with microsomes at 37°C, shaking at 100 rpm. Five time points over 90 minutes were analyzed. The reactions were stopped by adding 5 volumes of acetonitrile with internal standard to incubation aliquots, followed by protein sedimentation by centrifuging at 5500 rpm for 5 minutes. Each reaction was performed in

duplicates. Supernatants were analyzed using the HPLC system coupled with a tandem mass spectrometer.

The elimination constant (k_{el}), half-life ($t_{1/2}$), and intrinsic clearance (Cl_{int}) were determined in a plot of ln(AUC) versus time, using linear regression analysis:[a]

$$k_{el} = -slope \qquad t_{1/2} = \frac{0.693}{k} \qquad Cl_{int} = \frac{0.693}{t_{1/2}} \times \frac{\mu l_{incubation}}{mg_{microsomes}}$$

[a] In order to indicate the quality of the linear regression analysis, the R^2 (determination coefficient) values are provided. In some cases, the last time point is excluded from the calculations to ensure acceptable logarithmic linearity of decay.

Results

Human and mouse microsomal stability data for reference and test compounds are provided in the tables below.

Table S30. Human microsomal stability

Compound ID	Time, min	Analyte Peak Area		Analyte Peak Area, Mean of 2	% Remaining, Mean of 2	R^2	k_{el}, min^{-1}	$t_{1/2}$, min	Cl_{int}, µl/min/mg	% Remaining without cofactor, Mean of 2
		Inc. 1	Inc. 2							
Diclofenac human	0	1.37E+00	1.50E+00	1.44E+00	100	0.996	0.090	7.7	217	100
	15	4.27E-01	4.72E-01	4.50E-01	31	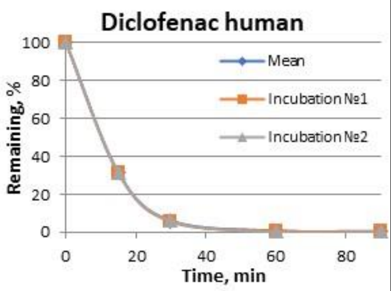				
	30	7.44E-02	9.24E-02	8.34E-02	6					
	60	8.11E-03	5.83E-03	6.97E-03	0					
	90	4.32E-03	4.86E-03	4.59E-03	0					93
Propranolol human	0	6.22E-02	6.61E-02	6.41E-02	100	0.986	0.009	79.4	21	100
	15	5.53E-02	5.58E-02	5.56E-02	87	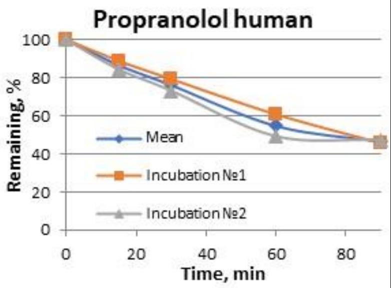				
	30	4.94E-02	4.84E-02	4.89E-02	76					
	60	3.79E-02	3.25E-02	3.52E-02	55					
	90	2.86E-02	3.13E-02	3.00E-02	47					101
**MU1700 human**	0	4.38E-02	4.14E-02	4.26E-02	100	0.967	0.004	178.0	9	100
	15	4.09E-02	3.62E-02	3.86E-02	90	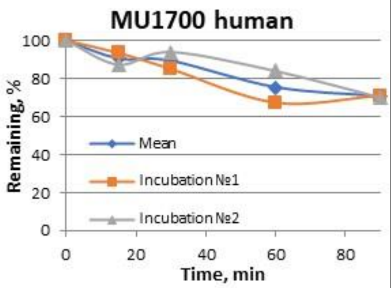				
	30	3.72E-02	3.89E-02	3.81E-02	89					
	60	2.94E-02	3.48E-02	3.21E-02	75					
	90	3.10E-02	2.91E-02	3.01E-02	71					85

Table S31. Mouse microsomal stability

Compound ID	Time, min	Analyte Peak Area		Analyte Peak Area, Mean of 2	% Remaining, Mean of 2	R^2	k_{el}, min^{-1}	$t_{1/2}$, min	Cl_{int}, µl/min/mg	% Remaining without cofactor, Mean of 2
		Inc. 1	Inc. 2							
Propranolol mouse	0	7.07E-02	7.53E-02	7.30E-02	100	0.897	0.033	20.9	80	100
	15	1.89E-02	1.95E-02	1.92E-02	26	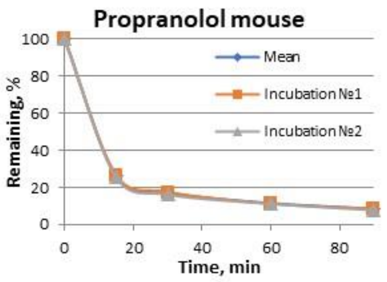				
	30	1.23E-02	1.24E-02	1.23E-02	17					
	60	7.80E-03	8.64E-03	8.22E-03	11					
	90	5.81E-03	6.10E-03	5.96E-03	8					109
Imipramine mouse	0	3.25E+00	3.55E+00	3.40E+00	100	0.962	0.085	8.2	204	100
	15	7.23E-01	7.84E-01	7.54E-01	22	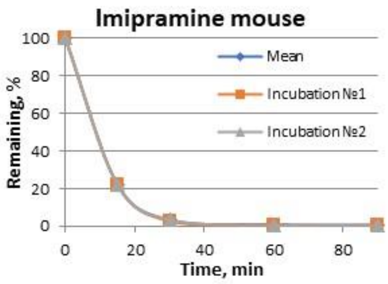				
	30	8.80E-02	1.28E-01	1.08E-01	3					
	60	1.76E-02	2.52E-02	2.14E-02	1					
	90	1.32E-02	1.31E-02	1.32E-02	0					103
**MU1700 mouse**	0	3.52E-02	3.55E-02	3.53E-02	100	0.991	0.012	59.6	28	100
	15	3.25E-02	3.16E-02	3.21E-02	91	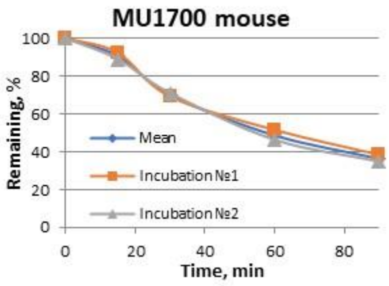				
	30	2.44E-02	2.52E-02	2.48E-02	70					
	60	1.82E-02	1.64E-02	1.73E-02	49					
	90	1.36E-02	1.23E-02	1.29E-02	37					100

Interpretation of microsomal stability assay data

The test compounds can be classified in terms of their microsomal stability into low, medium and high clearance groups. Intrinsic Clearance (*in vitro*) can be recalculated to Intrinsic Clearance (*in vivo*) using literature data for liver weight and liver blood flow with the next equation[1]:

$$Predicted\ in\ vivo\ CL_{int,u} = \frac{in\ vitro\ CL_{int} \times PBSF \times LW}{f_{u\ mic}\ or\ f_{u\ heps}}$$

where,

$in\ vivo\ CL_{int,u}$ – predicted *in vivo* intrinsic clearance, mL/min/kg

$in\ vitro\ CL_{int}$ – *in vitro* microsomal clearance, mL/min/mg

$PBSF$ – physiologically based scaling factor – the microsomal average recovery factor for microsomal predictions and hepatocellularity for hepatocyte predictions, mg/g

LW – liver weight/kg bodyweight, g/kg

$f_{u\ mic}\ or\ f_{u\ heps}$ – fraction unbound in either microsomes or hepatocytes (can be determined from Plasma Protein Binding study or assumed as 1, if it is unknown)

Using $in\ vivo\ Cl_{int}$ hepatic clearance can be predicted based on a "well-stirred" liver model using the next formula[2]:

$$CL_H = \frac{Q_H \times f_u \times CL_{int}}{Q_H + f_u \times CL_{int}}$$

where,

CL_H – predicted hepatic clearance, mL/min/kg

Q_H – liver blood flow, mL/min/kg

f_u – fraction unbound in the blood

CL_{int} – predicted *in vivo* clearance, mL/min/kg

---

[1]. Wood, F. L., Houston, J. B., & Hallifax, D. (2017). Clearance prediction methodology needs fundamental improvement: Trends common to rat and human hepatocytes/microsomes and implications for experimental methodology. Drug Metabolism and Disposition, 45(11), 1178–1188. https://doi.org/10.1124/dmd.117.077040

[2]. Laveé, T., & Funk, C. (2007). In vivo absorption, distribution, metabolism, and excretion studies in Discovery and Development. Comprehensive Medicinal Chemistry II, 31–50. https://doi.org/10.1016/b0-08-045044-x/00118-8

[3]. Barter Z.E., *et al*., Scaling factors for the extrapolation of *in vivo* metabolic drug clearance from *in vitro* data: reaching a consensus on values of human microsomal protein and hepatocellularity per gram of liver, *Current Drug Metabolism, 2007, 8,* 33-45.

[4]. Iwatsubo T., *et al*., Prediction of species differences (rats, dogs, humans) in the *in vivo* metabolic clearance of YM796 by the liver from *in vitro* data, *Journal of Pharmacology and Experimental Therapeutics, 1997,* **283,** 462-469.

12...Assessment of Caco-2 Permeability for Compound MU17002HCl

STUDY REPORT ADME050224g – BIENTA ENAMINE BIOLOGY SERVICES

Study Responsibilities

Study Director	Yuliia Holota, Ph.D.
Scientists	Anzhela Rodnichenko, Ph.D.
	Natalia Chyhrynova, M.S.
	Mariia Pavlichenko, M.S.

Study Objective

The purpose of this study was to evaluate the permeability of compound **MU17002HCl** in the bidirectional Caco-2 assay.

Materials

Reagents and consumables

DMEM/High glucose with L-Glutamine (HyClone, USA; Cat# SH30003.04)

Fetal Bovine Serum (Sigma-Aldrich, USA; Cat# F7524)

Sodium bicarbonate (Sigma-Aldrich, USA; Cat# S5761)

Penicillin/Streptomycin (100x) (Sigma-Aldrich, USA; Cat# P4333)

Puromycin dihydrochloride, ≥98% (Santa Cruz, USA; Cat# sc-108071)

Trypsin-EDTA solution (10x) (Sigma-Aldrich, USA; Cat# T4174)

HEPES, High Purity Grade (Helicon, Am-0485-0.1)

Dulbecco's PBS (1x) without Ca & Mg (Gibco, Cat# 21600-044)

Hanks' BSS (1x) without Ca & Mg without Phenol Red (Sigma, Cat# H4891)

Multiwell Insert system, PET, 1 µm (Millicell, USA; Cat# PSRP010R5)

24 well plate Multiwell™ (Millicell, USA; Cat# PSMW010R5)

Centrifuge Tubes, 50 ml (Santa Cruz, USA; Cat# sc-200251)

Serological Pipettes 5 ml, 10 ml, 25 ml (Greiner Bio-One)

Disposable pipettor tips (Thermo Scientific, Fisherbrand, Eppendorf USA)

1.4 ml microtubes (Thermo Scientific, USA, Cat# 4140)

Ketoprofen (Sigma-Aldrich, USA; Cat# PHR1375)

Atenolol (Sigma-Aldrich, USA; Cat# 74327)

Digoxin (Enamine, Ukraine, EN300-6492954)

DMSO Chromasolv Plus, HPLC grade, ≥99.7% (Sigma-Aldrich, USA; Cat# 34869)

Acetonitrile Chromasolv, gradient grade, for HPLC, ≥99.9% (Sigma-Aldrich, USA; Cat# 34851)

Formic acid for mass spectrometry, ~98% (Fluka, USA; Cat# 94318)

Methanol, for HPLC, ≥99.9% (Sigma-Aldrich, Cat# 34860)

Heptafluorobutyric acid (Sigma-Aldrich, Cat# 52411)

Equipment

Cell culture CO_2 incubator, model CCL-170B-8 (ESCO, Singapore)

Centrifuge 5804R (Eppendorf, USA)

Centrifuge 4-15C (Qiagen) (Sigma, Germany)

Etched Hemacytometer, dark line counting chamber (Hausser Scientific, USA; Cat# 3500)

Innova 4080 Incubator Shaker (New Brunswick Scientific, USA)

Millicell-ERS system ohm meter (Millipore, Cat# MERS 000 01)

Agilent InfinityLab Poroshell 120 EC-C18, 2.1 x 50 mm, 4 µm (Cat #699770-902)

VWR Membrane Nitrogen Generators N2-04-L1466, nitrogen purity 99%+ (VWR, USA)

Multichannel manual pipette (Thermo Labsystems Finnpipette, FA16-50R)

Multichannel Electronic Pipettes 2-125 µL, 5-250 µL, 15-1250 µL, Matrix (Thermo Scientific, USA)

PIPETMAN pipettes 2-20 µl, 50-200 µl, 200-1000 µl (Gilson, USA)

Water purification system Millipore Milli-Q Gradient A10 (Millipore, France)

Opentrons OT-2 (Opentrons Labworks, USA)

Analytical System

All measurements were performed using the Shimadzu HPLC system including vacuum degasser, gradient pumps, reverse phase HPLC column, column oven, and autosampler. Mass spectrometric analysis was performed using a Triple quadrupole mass-detector API 3000 with TurboIonSpray Ion Source (AB Sciex, Canada) and TurboIonspray interface. The TurboIonSpray ion source was used in both positive and negative ion modes. The data acquisition and system control were performed using Analyst 1.6.3 software from AB Sciex.

Methods

Caco-2 cells were cultured in 75 cm^2 flasks to 80-90% confluence according to the ATCC and Millipore recommendations (Arena A. et al., 2003) in a humidified atmosphere at 37°C and 5% CO_2. Cells were detached with Trypsin/EDTA solution and resuspended in the complete medium containing DMEM high glucose (4500 mg/l) with L-glutamine (4 mM) supplemented with 10% heat-inactivated Fetal Bovine Serum, 1% non-essential amino acids, and 730 nM puromycin and seeded at a density 5 $\times 10^5$ cells in 75 cm^2 flask (Sevin E. et al., 2013). After 5 days, cells were trypsinized and resuspended in the complete medium to a final concentration of 600 $\times 10^3$ cells/ml. 400 µl of the cell suspension was added to each well of the HTS 24-Multiwell Insert System and 25 ml of prewarmed complete medium was added to the feeder tray. Caco-2 cells were incubated in Multiwell Insert System for 6-10 days before the transport experiments. The medium in the filter plate and feeder tray was refreshed every other day. Prior to the transport experiment, the integrity of the monolayer was verified by measuring the transepithelial electrical resistance (TEER) for every well using the Millicell-ERS system ohm meter. The final TEER values were within the range of 150-600 $\Omega \times cm^2$ (Srinivasan B. et al., 2015) as required for the assay conditions. The 24-well insert plate was removed from its feeder plate and placed in a new sterile 24-well transport analysis plate. The inserts were washed with PBS after medium aspiration.

Ketoprofen, Atenolol, and Digoxin were used as reference compounds.

To determine the rate of compounds transport in apical (A)-to-basolateral (B) direction, 300 µL of the test compound dissolved in transport buffer (Hanks' BSS (9.5 g/L) and NaHCO3 (0.35 g /L) with MgSO4 to final concentration 0.81 mM, CaCl2 to final concentration 1.26 mM, HEPES to final concentration 25 mM. pH adjusted to 7.4) was added into the filter wells; 1000 µL of transport buffer was added to transport analysis plate wells.

To determine transport rates in the basolateral (B)-to-apical (A) direction, 1000 µL of the test compound solutions was added into the wells of the transport analysis plate, the wells in the filter plate were filled with 300 µL of buffer (apical compartment).

The final concentration of test and reference compounds was 10 µM.

The plates were incubated for 90 min at 37°C under continuous shaking at 100 rpm. 75 μL aliquots were taken from the donor and receiver compartments for LC-MS/MS analysis. All samples were mixed with 2 volumes of acetonitrile followed by protein sedimentation by centrifuging at 10000 rpm for 10 minutes. Supernatants were analyzed using the HPLC system coupled with a tandem mass spectrometer.

All solutions of test and reference compounds were prepared manually, and further manipulations with the solutions were performed with automation using Opentrons.

The apparent permeability (P_{app}) was calculated for the Caco-2 permeability assay using the following equation:

$$\boldsymbol{P_{app}} = \frac{\boldsymbol{V_A}}{\boldsymbol{Area} \times \boldsymbol{Time}} \times \frac{[\boldsymbol{drug}]_{\boldsymbol{acc}}}{[\boldsymbol{drug}]_{\boldsymbol{initial,d}}}$$

V_A – volume of transport buffer in acceptor well,

Area – surface area of the insert (equals to the effective growth area of the insert - 0.7 sq.cm),

Time – time of the assay,

$[drug]_{acc}$ – amount of test compound in acceptor well,

$[drug]_{initial,d}$ – initial amount of test compound in a donor well.

P_{app} is expressed in 10^{-6}cm/sec.

Efflux ratio (P_{app}(BA)/P_{app}(AB)) reveals the difference in P_{app} as a result of active transport. If the efflux ratio is greater than 2, this indicates the occurred active efflux.

The % recovery can be useful in interpreting the Caco-2 data. If the recovery is very low, this may indicate poor solubility, binding of the compound to the test plate materials, metabolism by the Caco-2 cells, or accumulation of the compound in the cell monolayer. The % recovery was calculated using the following equation:

$$\%\,\boldsymbol{recovery} = \frac{\boldsymbol{C_{acc}} \times \boldsymbol{V_{acc}} + \boldsymbol{C_d} \times \boldsymbol{V_d}}{\boldsymbol{C_{initial,d}} \times \boldsymbol{V_d}} \times \mathbf{100},$$

V_{acc} – volume of compound solution in acceptor well (cm^3),

V_d – volume of compound solution in donor well (cm^3),

C_{acc} – peak area of the test compound in acceptor well,

C_d – peak area of the test compound in donor well,

$C_{initial,d}$ – initial peak area of the test compound in a donor well.

Results and discussion

A-B and B-A permeability data as well as the efflux ratio (P_{app}B-A/P_{app}A-B) data for the test and reference compounds are listed in the tables below.

Table S32. A-B and B-A permeability data

Test compound	P_{app} (AB), 10^{-6} cm/s				P_{app} (BA), 10^{-6} cm/s				Efflux ratio*
	1	2	Mean	SD	1	2	Mean	SD	
Ketoprofen	17.5	16.7	**17.1**	0.6	16.5	19.0	**17.7**	1.7	**1.0**
Atenolol	0.7	0.8	**0.8**	0.1					
Digoxin	0.9	0.5	**0.7**	0.3	12.9	14.1	**13.5**	0.8	**18.7**
MU17002HCl	0.7	0.5	**0.6**	0.1	0.3	0.4	**0.4**	0.0	**0.6**

* Efflux ratio is expressed as the quotient of P_{app}(BA) to P_{app}(AB)

A-B permeability data for all the reference compounds correspond to the literature data (Y. Shirasaka et al. 2008; Zeynep S. Teksin et al, 2010), thus validating this study.

The efflux ratio for Digoxin in the absence of Verapamil corresponds to the literature data thus validating this assay.

Recovery values are indicated in the table below (Table 2).

Table S33. Recovery values for test and reference compounds

Compound ID	Recovery, %					
	A-B			B-A		
	1	2	Mean	1	2	Mean
Ketoprofen	91	90	**90**	98	104	**101**
Atenolol	97	95	**96**			
Digoxin	100	82	**91**	90	95	**93**
MU17002HCl	*18*	*27*	***23***	*61*	*63*	***62***

Conclusions

The test compound under the condition of the experiment exhibits low permeability in the A-B direction.

It should be noted that the recovery values (Table 2) for compound are low. This might be due to the poor solubility of this compound in transport solution, metabolism by the Caco-2 cells, and accumulation of this compound in the cell monolayer or binding to the test plate materials. Therefore, the permeability values for this test compound should be considered as approximate.

References

1. Arena A and Phillips J. Optimization of Caco-2 cell growth and differentiation for drug transport assay studies using a 96 well MultiScreen Caco-2 Assay System. Millipore protocol note PC1060EN00P, rev. 08/2003.
2. Srinivasan B., Kolli AR., Esch MB., Abaci HE., Shuler ML., Hickman JJ. TEER measurement techniques for *in vitro* barrier model systems. J Lab Autom. 2015 Apr;20(2):107-26.
3. Rautio J., Humphreys JE., Webster LO., Balakrishnan A., Keogh JP. Kunta JR., Serabjit-Singh CJ. Polli JW. In vitro, p-glycoprotein inhibition assays for assessment of clinical drug interaction potential of new drug candidates: a recommendation for probe substrates. Drug Metab Dispos. 2006 May;34(5):786-92.
4. http://www.cyprotex.com/admepk/in-vitro-permeability/caco-2-permeability
5. Yoshiyuki Shirasaka., Toshiyasu Sakane., Shinji Yamashita. Effect of P-Glycoprotein Expression Levels on the Concentration-Dependent Permeability of Drugs to the Cell Membrane J. of Pharmaceutical Sciences. V. 97. No. 1. 2008.
6. Sevin E., Dehouck L., Fabulas-da Costa A et al. Accelerated Caco-2 cell permeability model for drug discovery. Journal of Pharmacological and Toxicological Methods. V. 68. No. 3. 2013.
7. Zeynep S., Teksin., Paul R., Seo. James E., Poli. Comparison of Drug Permeabilities and BCS Classification: Three Lipid-Component PAMPA System Method versus Caco-2 Monolayers. AAPS J. 2010; 12(2): 238-241.

13. Comparison of the chemical probes MU1700 and M4K2234 with selected ALK2 inhibitors.

Table S34: Activity is expressed as biochemical IC_{50} values (nM) or as percentage of residual enzyme activity (compound concentration in parentheses). The compounds were tested in radiometric assays (Reaction Biology) in 10-dose IC_{50} mode with a 3-fold serial dilution starting at 3 μM and using 10 μM ATP concentration.

	MU1700	M4K2234	LDN-193189	K02288	LDN-212854	ML347	DMH1	Cmp 23	Comp 23-b
ALK1	13	7	0.8	1.8	2.4	46	27	51	120
ALK2	6	14	0.8	1.1	1.3	32	107.9	9	12
ALK3/ BMPR1A	425	168	5.3	34	85.8	10800	<5	6439	280
ALK4	>3000	1660	101	302	2133	Inactive	9622	18330	ND
ALK5/ TFGbR1	>3000	1950	350	321	9276	Inactive	Inactive	18650	2200
ALK6/ BMPR1B	41	88	16.7	6.4	ND	9830	47.6	13140	2100
ALK7	ND	ND	ND	ND	ND	ND	ND	ND	ND
ACTR2a	ND	ND	210	220	ND	ND	ND	2370	ND
ACTR2b	ND	ND	ND	ND	ND	ND	ND	ND	ND
TGFbR2	71 % (1 uM)	93 % (1 uM)	9.8 % (1 uM)	ND	ND	inactive	inactive	698	ND
MISR2	ND	ND	ND	ND	ND	ND	ND	ND	ND
BMPR2	89 % (1 uM)	91 % (1 uM)	75 % (1 uM)	ND	ND	inactive	inactive	inactive	ND
Kinome wide selectivity*	0/369	1/375	28/369	4/200	7/198	ND	ND	5/252	3/468
Ref.			10.1371/journal.pone.0062721	10.1021/cb300655w	10.1021/cb300655w	10.1016/j.bmcl.2013.03.113	10.1016/j.bmcl.2013.03.113	10.1016/j.bmcl.2022.128667	10.1016/j.bmcl.2018.09.006

*Selectivity is quantified as number of protein kinase off-targets outside ALK1-6 subfamily vs number of protein kinases tested at 1 μM concentration of a corresponding inhibitor. As the off targets were counted protein kinases with recidual activity below 25 %

14. Cell viability assessment

U-2 OS cells were seeded at 7500 cells per well in a volume of 50 µl and allowed to proliferate for 24 h at 37 °C/5% CO_2. The following day respective compounds were added to the plate, titrated between a range of 0.1 and 50 µM and incubated for 24 h. Following compound incubation time 5 µl of alamarBlue® reagent was added to each respective well and incubated at 37 °C/5% CO_2 for 2 h. Fluorescent values were determined using the PhERAstar® FSX (BMG Labtech) with excitation and emission wavelengths of 540/590nm respectively. Technical triplicates were averaged and background corrected. Averages were normalised against DMSO (100%) and wells containing no cells (0%). Values were plotted as % Growth control against compound concentration and IC50s were calculated via non-linear regression, log(inhibitor) vs normalised response with a variable slope (Graphpad Prism 9).

Figure S24: Cytotoxicity in a cell viability assay using the U2OS cell line and Alamar Blue as indicator at 24-hour time point.

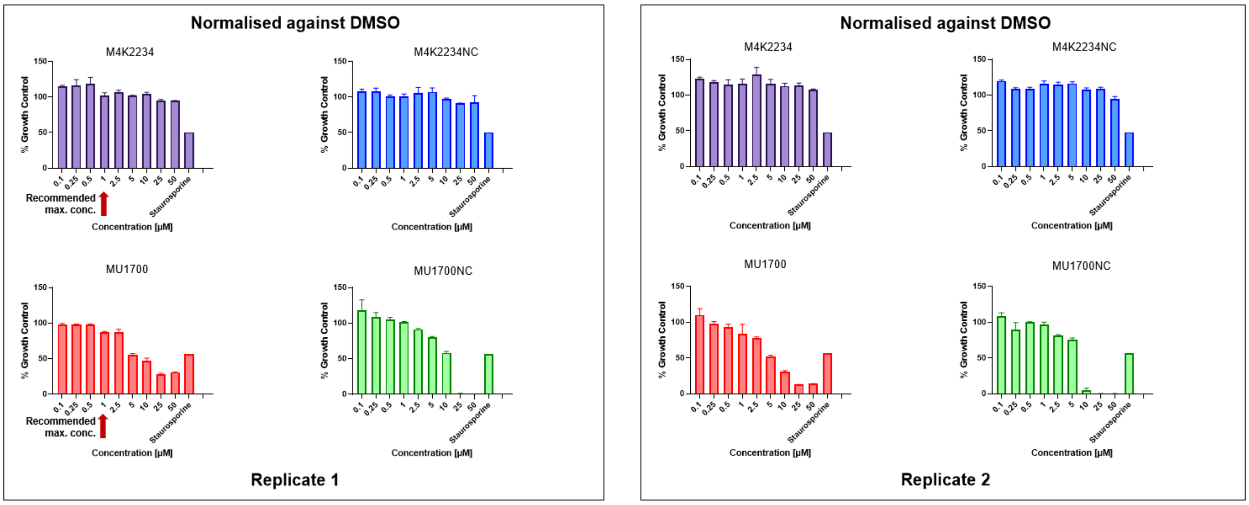

15. Liver microsomal metabolic stability assay.

For this assay, stock solutions of test compounds in DMSO (1 mM) were initially diluted to a concentration of 40.0 μM using 0.1 M potassium phosphate buffer (pH 7.4). Test compounds were then added to reaction wells at a final concentration of 1 μM, which was assumed to be well below K_m values to ensure linear reaction conditions (*i.e.*, avoid saturation). The final DMSO concentration was kept constant at 0.1%. Each compound was tested in duplicate for both time points (0 and 60 min). CD-1 mouse (male) or pooled human liver microsomes were added to the reaction wells at a final concentration of 0.5 mg/mL (protein). The final volume for each reaction was 100 μL, which included the NADPH-regeneration solution (NRS) mix. This NRS mix comprised glucose 6-phosphate dehydrogenase, $NADP^+$, $MgCl_2$, and glucose 6-phosphate. Reactions were carried out at 37 °C in an orbital shaker at 175 rpm. Upon completion of the 60 min time point, reactions were terminated by the addition of two volumes (200 μL) of ice-cold acetonitrile containing 0.5% formic acid and an internal standard (synthesized in-house). Samples were then centrifuged at 4000 rpm for 10 min to remove debris and precipitated proteins. Approximately 150 *μ*L of supernatant was subsequently transferred to a new 96-well microplate for LC/MS analysis. Narrow-window mass extraction LC/MS analysis was performed for all samples using a Waters Acquity UPLC system with a Waters Xevo quadrupole time-of-flight (Q-TOF) mass spectrometer to determine relative peak areas of the parent compounds. Chromatographic separations were performed on a Waters Acquity UPLC HSS T3 column (2.1 × 100 mm, 100 Å, 1.8 μm, part no. 186003539) at 30 °C. Mobile phase A and B were 0.1% formic acid in water and 0.1% formic acid in acetonitrile, respectively. The sample temperature was kept at 10 °C. The typical injection volume was 2 *μ*L. Chromatographic gradients used are shown in the table S35 below.

Table S35: Chomatographic gradients.

Time (min)	Flow Rate (*m*L/min)	%A	%B
0.0	0.450	100	0
2.5	0.450	5	95
3.0	0.450	5	95
3.5	0.450	100	0
4.5	0.450	100	0

Data acquisition was performed in the electrospray (ES) positive ion mode in the mass range of 100-1000 m/Z. The following source parameters were used: capillary voltage, 0.8 kV; cone voltage, 25 V; source temperature, 150 °C; desolvation temperature, 500 °C; cone gas flow, 150 L/h; desolvation gas flow, 600 L/h. Accurate masses were measured in lock-spray automated exact mass measurement mode. The fragment ions of leucine-enkephalin were used as reference substances (lock-mass) in the acquisition mode. The following lock-spray configuration was used: frequency, 10 s; cone voltage, 25

V; collision energy, 22 V. Data processing was performed using MassLynx 4.1 software. The percentage remaining values were calculated using the following equation:

$$\text{Percent remaining} = (A/A_0) \times 100$$

where A is area response after incubation and A_0 is area response at initial time point.

16. The inhibitory activity against ALK1-6: Kinase profiling of MU1700 and its analogs (Reaction Biology)

MU1700 and its derivatives were tested against 6 kinases (in duplicate or singlicate)

Compounds were tested in 10-dose IC_{50} mode with 3-fold serial dilution starting at 3 µM.

Control compound, LDN193189, was tested in 10-dose IC_{50} mode with 3-fold serial dilution starting at 10 µM. Reactions were carried out at 10 µM ATP.

Data tables include % enzyme activity (relative to DMSO controls) and curve fits.

*Curve fits were performed where the enzyme activities at the highest concentration of compounds were less than 65%.

Table S36: The inhibitory activity of **MU1700** against ALK1-6

	Compound IC50 (M)		IC50 (M) Control Cmpd	Control Cmpd ID
**Kinase**	**MU1700**	**MU1700**		
ALK1/ACVRL1	1,44E-08	1,17E-08	8,05E-09	LDN193189
ALK2/ACVR1	5,68E-09	6,12E-09	9,90E-09	LDN193189
ALK3/BMPR1A	3,63E-07	4,87E-07	3,71E-08	LDN193189
ALK4/ACVR1B			2,30E-07	LDN193189
ALK5/TGFBR1			8,10E-07	LDN193189
ALK6/BMPR1B	4,41E-08	3,70E-08	8,40E-09	LDN193189

ALK1/ACVRL1 (%Activity)

Conc.(M)	MU1700	MU1700
3,00E-06	158,36	133,00
1,00E-06	3,16	2,80
3,33E-07	8,39	5,47
1,11E-07	14,04	12,90
3,70E-08	27,46	28,01
1,23E-08	51,56	49,04
4,12E-09	89,78	78,61
1,37E-09	106,54	99,20
4,57E-10	38,98	41,97
1,52E-10	75,89	103,33
DMSO	100,00	107,07
HILLSLOPE	-1,15	-0,95
IC50 (M)	1,44E-08	1,17E-08

*Data point excluded to allow curve fit

ALK2/ACVR1 (%Activity)

Conc.(M)	MU1700	MU1700
3,00E-06	80,51	89,45
1,00E-06	1,13	-0,79
3,33E-07	0,48	1,26
1,11E-07	3,14	4,42
3,70E-08	11,00	11,18
1,23E-08	29,76	30,18
4,12E-09	59,21	62,23
1,37E-09	82,70	83,87
4,57E-10	19,96	21,94
1,52E-10	99,40	100,19
DMSO	99,62	99,06
HILLSLOPE	-1,12	-1,15
IC50 (M)	5,68E-09	6,12E-09

*Data point excluded to allow curve fit

ALK3/BMPR1A (%Activity)

Conc.(M)	MU1700	MU1700
3,00E-06	122,66	126,16
1,00E-06	46,39	51,26
3,33E-07	56,98	55,47
1,11E-07	53,48	59,26
3,70E-08	68,46	69,31
1,23E-08	84,32	79,16
4,12E-09	95,27	88,86
1,37E-09	97,90	94,04
4,57E-10	75,86	101,63
1,52E-10	103,37	91,98
DMSO	95,97	101,24
HILLSLOPE	-0,40	-0,32
IC50 (M)	3,63E-07	4,87E-07

*Data point excluded to allow

ALK4/ACVR1B (%Activity)

Conc.(M)	MU1700	MU1700
3,00E-06	150,32	139,70
1,00E-06	117,26	111,09
3,33E-07	145,67	146,60
1,11E-07	153,51	147,92
3,70E-08	115,10	122,45
1,23E-08	116,10	111,77
4,12E-09	107,49	99,78

ALK5/TGFBR1 (%Activity)

Conc.(M)	MU1700	MU1700
3,00E-06	70,15	73,06
1,00E-06	82,67	78,06
3,33E-07	82,65	72,43
1,11E-07	92,41	83,26
3,70E-08	92,76	85,48
1,23E-08	98,46	85,47
4,12E-09	99,59	85,11

ALK6/BMPR1B (%Activity)

Conc.(M)	MU1700	MU1700
3,00E-06	101,01	103,15
1,00E-06	5,36	5,05
3,33E-07	12,87	13,07
1,11E-07	27,78	26,94
3,70E-08	50,77	47,52
1,23E-08	72,79	67,24
4,12E-09	89,39	83,63

**1,37E-09**	102,10	102,06	**1,37E-09**	105,72	92,88	**1,37E-09**	88,06	96,14
**4,57E-10**	98,43	105,72	**4,57E-10**	107,29	93,43	**4,57E-10**	69,04	62,62
**1,52E-10**	79,63	95,43	**1,52E-10**	104,66	89,86	**1,52E-10**	90,15	93,85
**DMSO**	100,00	95,58	**DMSO**	100,00	95,70	**DMSO**	100,00	92,99
**HILLSLOPE**			**HILLSLOPE**			**HILLSLOPE**	**-0,94**	**-0,88**
**IC50 (M)**			**IC50 (M)**			**IC50 (M)**	**4,41E-08**	**3,70E-08**
						*Data point excluded to allow		

Table S37: The inhibitory activity of **MU1700NC** against ALK1-6

	Compound IC50 (M)		IC50 (M) Control Cmpd	Control Cmpd ID
**Kinase**	**MU1700NC**	**MU1700NC**		
**ALK1/ACVRL1**	**2,33E-06**	**1,97E-06**	**1,30E-08**	**LDN193189**
**ALK2/ACVR1**	**9,98E-07**	**1,15E-06**	**1,78E-08**	**LDN193189**
**ALK3/BMPR1A**			**2,80E-08**	**LDN193189**
**ALK4/ACVR1B**			**3,30E-07**	**LDN193189**
**ALK5/TGFBR1**			**3,98E-07**	**LDN193189**
**ALK6/BMPR1B**			**1,49E-08**	**LDN193189**

**ALK1/ACVRL1 (%Activity)**			**ALK2/ACVR1 (%Activity)**			**ALK3/BMPR1A (%Activity)**		
**Conc.(M)**	**MU1700**	**MU1700N**	**Conc.(M)**	**MU1700N**	**MU1700NC**	**Conc.(M)**	**MU1700NC**	**MU1700N**
**3,00E-06**	39,58	39,17	**3,00E-06**	17,01	19,94	**3,00E-06**	94,72	96,19
**1,00E-06**	75,31	69,69	**1,00E-06**	48,86	51,42	**1,00E-06**	104,15	110,93
**3,33E-07**	111,94	103,48	**3,33E-07**	89,27	104,80	**3,33E-07**	97,09	104,21
**1,11E-07**	113,63	112,91	**1,11E-07**	115,61	127,51	**1,11E-07**	96,06	106,27
**3,70E-08**	96,33	101,90	**3,70E-08**	109,68	115,54	**3,70E-08**	111,11	107,35
**1,23E-08**	93,16	94,52	**1,23E-08**	99,67	106,05	**1,23E-08**	97,72	102,02
**4,12E-09**	87,83	97,16	**4,12E-09**	92,01	92,00	**4,12E-09**	99,35	108,09
**1,37E-09**	90,35	97,71	**1,37E-09**	96,61	94,95	**1,37E-09**	96,61	107,01
**4,57E-10**	98,87	103,07	**4,57E-10**	103,73	101,48	**4,57E-10**	101,43	113,23
**1,52E-10**	99,47	108,18	**1,52E-10**	106,68	93,03	**1,52E-10**	101,55	99,02
**DMSO**	96,41	109,34	**DMSO**	100,00	101,11	**DMSO**	107,26	104,94
**HILLSLOPE**	**-1,91**	**-1,48**	**HILLSLO**	**-1,79**	**-2,03**	**HILLSLOPE**		
**IC50 (M)**	**2,33E-06**	**1,97E-06**	**IC50 (M)**	**9,98E-07**	**1,15E-06**	**IC50 (M)**		
			*Data point excluded to allow curve fit					

**ALK4/ACVR1B (%Activity)**			**ALK5/TGFBR1 (%Activity)**			**ALK6/BMPR1B (%Activity)**		
**Conc.(M)**	**MU1700**	**MU1700**	**Conc.(M)**	**MU1700**	**MU1700N**	**Conc.(M)**	**MU1700N**	**MU1700N**
**3,00E-06**	94,98	90,12	**3,00E-06**	102,54	106,41	**3,00E-06**	68,23	69,24
**1,00E-06**	95,44	94,88	**1,00E-06**	99,91	99,55	**1,00E-06**	93,52	94,78
**3,33E-07**	91,41	90,89	**3,33E-07**	100,22	102,32	**3,33E-07**	110,22	104,85
**1,11E-07**	93,53	90,72	**1,11E-07**	97,67	101,03	**1,11E-07**	108,63	105,59
**3,70E-08**	89,71	95,61	**3,70E-08**	99,77	105,47	**3,70E-08**	97,96	109,76
**1,23E-08**	90,34	95,12	**1,23E-08**	97,49	100,31	**1,23E-08**	99,48	97,72
**4,12E-09**	87,84	95,37	**4,12E-09**	92,33	123,80	**4,12E-09**	97,56	93,12
**1,37E-09**	93,38	98,33	**1,37E-09**	99,11	103,74	**1,37E-09**	97,93	97,90
**4,57E-10**	95,93	97,13	**4,57E-10**	97,15	99,04	**4,57E-10**	98,02	101,25
**1,52E-10**	101,30	102,29	**1,52E-10**	95,11	98,46	**1,52E-10**	100,61	92,68
**DMSO**	96,02	96,48	**DMSO**	95,97	102,04	**DMSO**	102,45	97,12
**HILLSLOPE**			**HILLSLOPE**			**HILLSLOPE**		
**IC50 (M)**			**IC50 (M)**			**IC50 (M)**		

Table S38: The inhibitory activity of **14** against ALK1-6

	Compound IC50 (M):		IC50 (M) Control Cmpd	Control Cmpd ID
**Kinase:**	**Compound 14**			
**ALK1/ACVRL1**	**3,88E-08**	**4,52E-08**	**1,13E-08**	**LDN193189**
**ALK2/ACVR1**	**2,62E-08**	**2,77E-08**	**1,50E-08**	**LDN193189**
**ALK3/BMPR1A**	**1,28E-06**	**1,55E-06**	**7,84E-08**	**LDN193189**
**ALK4/ACVR1B**	**3,39E-07**	**2,33E-07**	**7,63E-08**	**LDN193189**
**ALK5/TGFBR1**	**6,20E-07**	**6,63E-07**	**2,31E-07**	**LDN193189**
**ALK6/BMPR1B**	**1,19E-07**	**1,26E-07**	**1,42E-08**	**LDN193189**

ALK1/ACVRL1 (%Activity)

Conc.(M)	8	8
**3,00E-06**	0,96	0,87
**1,00E-06**	2,32	2,27
**3,33E-07**	8,47	8,73
**1,11E-07**	24,39	27,40
**3,70E-08**	52,16	54,47
**1,23E-08**	88,44	85,35
**4,12E-09**	103,29	100,32
**1,37E-09**	99,98	100,97
**4,57E-10**	104,42	102,86
**1,52E-10**	113,92	100,59
**DMSO**	104,96	98,87
**HILLSLO**	**-1,23**	**-1,22**
**IC50 (M)**	**3,88E-08**	**4,52E-08**

ALK2/ACVR1 (%Activity)

Conc.(M)	8	8
**3,00E-06**	0,93	0,70
**1,00E-06**	1,17	0,95
**3,33E-07**	3,50	3,10
**1,11E-07**	12,26	10,04
**3,70E-08**	31,31	32,61
**1,23E-08**	79,81	77,13
**4,12E-09**	95,50	90,56
**1,37E-09**	94,10	90,91
**4,57E-10**	101,61	86,99
**1,52E-10**	95,30	92,32
**DMSO**	100,00	100,92
**HILLSLOPE**	**-1,74**	**-1,78**
**IC50 (M)**	**2,62E-**	**2,77E-08**

ALK3/BMPR1A (%Activity)

Conc.(M)	8	8
**3,00E-06**	44,04	45,90
**1,00E-06**	52,34	51,43
**3,33E-07**	76,97	77,58
**1,11E-07**	94,54	88,80
**3,70E-08**	99,11	96,38
**1,23E-08**	99,80	96,52
**4,12E-09**	108,76	104,05
**1,37E-09**	120,61	106,96
**4,57E-10**	107,09	104,16
**1,52E-10**	103,22	100,70
**DMSO**	100,99	99,10
**HILLSLOPE**	**-0,68**	**-0,64**
**IC50 (M)**	**1,28E-06**	**1,55E-**

ALK4/ACVR1B (%Activity)

Conc.(M)	8	8
**3,00E-06**	8,93	7,84
**1,00E-06**	21,19	17,73
**3,33E-07**	46,49	44,63
**1,11E-07**	71,93	68,36
**3,70E-08**	88,82	80,53
**1,23E-08**	97,82	88,90
**4,12E-09**	103,98	102,06
**1,37E-09**	84,20	102,22
**4,57E-10**	96,08	96,35
**1,52E-10**	81,73	97,74
**DMSO**	96,28	101,57
**HILLSLOP**	**-1,17**	**-0,91**
**IC50 (M)**	**3,39E-07**	**2,33E-**

ALK5/TGFBR1 (%Activity)

Conc.(M)	8	8
**3,00E-06**	17,40	19,83
**1,00E-06**	39,04	38,71
**3,33E-07**	76,77	79,13
**1,11E-07**	102,22	116,18
**3,70E-08**	112,65	115,41
**1,23E-08**	117,66	116,05
**4,12E-09**	109,37	119,84
**1,37E-09**	117,52	119,34
**4,57E-10**	115,00	114,74
**1,52E-10**	115,19	116,22
**DMSO**	103,83	95,35
**HILLSLO**	**-1,23**	**-1,40**
**IC50 (M)**	**6,20E-07**	**6,63E-07**

ALK6/BMPR1B (%Activity)

Conc.(M)	8	8
**3,00E-06**	4,64	5,03
**1,00E-06**	10,40	10,76
**3,33E-07**	27,81	31,53
**1,11E-07**	54,34	56,90
**3,70E-08**	80,71	81,99
**1,23E-08**	99,90	100,39
**4,12E-09**	111,02	113,04
**1,37E-09**	105,97	110,29
**4,57E-10**	107,81	111,08
**1,52E-10**	105,21	104,69
**DMSO**	99,73	101,33
**HILLSLOP**	**-1,07**	**-1,03**
**IC50 (M)**	**1,19E-07**	**1,26E-07**

Table S39: The inhibitory activity of **18** against ALK1-6

	Compound IC50 (M):	IC50 (M) Control Cmpd	Control Cmpd ID	
**Kinase:**	**Compound 18**			
**ALK1/ACVRL1**	**1,29E-06**	**1,32E-06**	**1,13E-08**	**LDN193189**
**ALK2/ACVR1**	**7,27E-07**	**6,66E-07**	**1,50E-08**	**LDN193189**
**ALK3/BMPR1A**			**7,84E-08**	**LDN193189**
**ALK4/ACVR1B**	**4,17E-07**	**3,55E-07**	**7,63E-08**	**LDN193189**
**ALK5/TGFBR1**	**1,64E-06**	**1,48E-06**	**2,31E-07**	**LDN193189**
**ALK6/BMPR1B**			**1,42E-08**	**LDN193189**

ALK1/ACVRL1 (%Activity)

**Conc.(M)**	**9**	**9**
**3,00E-06**	25,08	22,80
**1,00E-06**	57,33	58,85
**3,33E-07**	95,94	96,31
**1,11E-07**	114,11	112,36
**3,70E-08**	114,79	114,45
**1,23E-08**	106,55	114,15
**4,12E-09**	99,29	100,18
**1,37E-09**	95,84	88,91
**4,57E-10**	95,94	87,77
**1,52E-10**	98,62	94,49
**DMSO**	100,12	96,68
**HILLSLO**	**-1,66**	**-1,78**
**IC50 (M)**	**1,29E-06**	**1,32E-**

ALK2/ACVR1 (%Activity)

**Conc.(M)**	**9**	**9**
**3,00E-06**	12,70	9,95
**1,00E-06**	38,83	35,64
**3,33E-07**	77,32	81,04
**1,11E-07**	115,02	114,40
**3,70E-08**	121,28	123,92
**1,23E-08**	111,55	118,65
**4,12E-09**	107,04	113,11
**1,37E-09**	102,61	101,56
**4,57E-10**	91,30	97,47
**1,52E-10**	93,26	98,70
**DMSO**	99,89	104,50
**HILLSLO**	**-1,73**	**-1,83**
**IC50 (M)**	**7,27E-07**	**6,66E-07**

ALK3/BMPR1A (%Activity)

**Conc.(M)**	**9**	**9**
**3,00E-06**	114,19	120,85
**1,00E-06**	109,35	117,66
**3,33E-07**	107,74	106,75
**1,11E-07**	108,21	111,61
**3,70E-08**	105,86	107,37
**1,23E-08**	99,72	105,46
**4,12E-09**	103,94	110,54
**1,37E-09**	110,91	109,61
**4,57E-10**	104,82	105,91
**1,52E-10**	102,11	104,07
**DMSO**	100,00	100,08
**HILLSLOP**		
**IC50 (M)**		

ALK4/ACVR1B (%Activity)

**Conc.(M)**	**9**	**9**
**3,00E-06**	15,21	13,87
**1,00E-06**	32,56	28,44
**3,33E-07**	59,69	54,04
**1,11E-07**	71,76	72,56
**3,70E-08**	80,89	78,33
**1,23E-08**	85,25	90,10
**4,12E-09**	103,20	100,51
**1,37E-09**	94,10	91,54
**4,57E-10**	99,01	96,67
**1,52E-10**	97,79	101,51
**DMSO**	101,56	100,59
**HILLSLO**	**-0,73**	**-0,78**
**IC50 (M)**	**4,17E-07**	**3,55E-07**

ALK5/TGFBR1 (%Activity)

**Conc.(M)**	**9**	**9**
**3,00E-06**	38,56	36,27
**1,00E-06**	69,36	65,69
**3,33E-07**	99,23	102,88
**1,11E-07**	110,40	121,96
**3,70E-08**	113,42	111,94
**1,23E-08**	114,70	117,53
**4,12E-09**	109,80	115,27
**1,37E-09**	115,95	108,43
**4,57E-10**	115,87	115,53
**1,52E-10**	115,00	115,75
**DMSO**	98,92	103,46
**HILLSLO**	**-1,20**	**-1,34**
**IC50 (M)**	**1,64E-06**	**1,48E-06**

ALK6/BMPR1B (%Activity)

**Conc.(M)**	**9**	**9**
**3,00E-06**	79,66	83,92
**1,00E-06**	105,83	103,62
**3,33E-07**	118,68	122,74
**1,11E-07**	120,68	124,44
**3,70E-08**	116,55	119,41
**1,23E-08**	111,27	114,72
**4,12E-09**	114,71	119,72
**1,37E-09**	115,37	117,63
**4,57E-10**	114,16	119,71
**1,52E-10**	115,04	114,89
**DMSO**	99,16	99,78
**HILLSLO**		
**IC50 (M)**		

Table S40: The inhibitory activity of **21** against ALK1-6

	Compound IC50 (M)		IC50 (M) Control Cmpd	Control Cmpd ID
**Kinase**	**Compound 21**			
**ALK1/ACVRL1**	**7,55E-07**	**8,53E-07**	**1,30E-08**	**LDN193189**
**ALK2/ACVR1**	**2,53E-07**	**2,66E-07**	**1,78E-08**	**LDN193189**
**ALK3/BMPR1A**			**2,80E-08**	**LDN193189**
**ALK4/ACVR1B**			**3,30E-07**	**LDN193189**
**ALK5/TGFBR1**			**3,98E-07**	**LDN193189**
**ALK6/BMPR1B**	**1,22E-06**	**1,08E-06**	**1,49E-08**	**LDN193189**

ALK1/ACVRL1 (%Activity)

**Conc.(M)**	**RM-493**	**RM-493**
**3,00E-06**	17,26	16,25
**1,00E-06**	38,44	42,29
**3,33E-07**	80,19	84,12
**1,11E-07**	114,12	117,17
**3,70E-08**	117,28	120,31
**1,23E-08**	106,82	104,25
**4,12E-09**	106,39	105,26
**1,37E-09**	84,58	98,24
**4,57E-10**	87,30	99,96
**1,52E-10**	93,58	96,33
**DMSO**	95,81	105,27
**HILLSLOP**	**-1,65**	**-1,74**
**IC50 (M)**	**7,55E-07**	**8,53E-07**

*Data point excluded to allow curve fit

ALK2/ACVR1 (%Activity)

**Conc.(M)**	**RM-493**	**RM-493**
**3,00E-06**	5,37	4,30
**1,00E-06**	15,30	14,93
**3,33E-07**	37,18	36,61
**1,11E-07**	74,77	91,95
**3,70E-08**	105,11	118,63
**1,23E-08**	108,44	119,31
**4,12E-09**	102,77	103,58
**1,37E-09**	90,16	94,43
**4,57E-10**	92,00	97,75
**1,52E-10**	96,90	99,11
**DMSO**	99,56	100,44
**HILLSLOP**	**-1,57**	**-2,19**
**IC50 (M)**	**2,53E-07**	**2,66E-07**

ALK3/BMPR1A (%Activity)

**Conc.(M)**	**RM-493**	**RM-493**
**3,00E-06**	72,06	77,23
**1,00E-06**	93,50	99,77
**3,33E-07**	100,64	97,04
**1,11E-07**	93,34	91,71
**3,70E-08**	104,91	108,06
**1,23E-08**	105,43	95,31
**4,12E-09**	109,77	106,52
**1,37E-09**	106,92	107,72
**4,57E-10**	119,57	114,08
**1,52E-10**	113,95	105,39
**DMSO**	95,61	108,16
**HILLSLOPE**		
**IC50 (M)**		

ALK4/ACVR1B (%Activity)

**Conc.(M)**	**RM-493**	**RM-493**
**3,00E-06**	115,74	93,93
**1,00E-06**	110,46	102,70
**3,33E-07**	95,95	95,16
**1,11E-07**	97,64	89,87
**3,70E-08**	95,70	89,69
**1,23E-08**	93,58	88,68
**4,12E-09**	101,36	90,92
**1,37E-09**	94,98	92,04
**4,57E-10**	97,33	99,50
**1,52E-10**	102,48	100,49
**DMSO**	103,45	100,32
**HILLSLOPE**		
**IC50 (M)**		

ALK5/TGFBR1 (%Activity)

**Conc.(M)**	**RM-493**	**RM-493**
**3,00E-06**	84,53	97,27
**1,00E-06**	90,47	91,41
**3,33E-07**	96,94	103,21
**1,11E-07**	102,17	106,83
**3,70E-08**	106,61	101,68
**1,23E-08**	102,03	93,61
**4,12E-09**	99,87	100,30
**1,37E-09**	105,42	98,85
**4,57E-10**	104,30	99,61
**1,52E-10**	106,04	97,61
**DMSO**	108,05	96,14
**HILLSLO**		
**IC50 (M)**		

ALK6/BMPR1B (%Activity)

**Conc.(M)**	**RM-493**	**RM-493**
**3,00E-06**	29,56	24,12
**1,00E-06**	57,14	51,67
**3,33E-07**	93,30	81,30
**1,11E-07**	107,41	103,05
**3,70E-08**	112,08	107,08
**1,23E-08**	111,16	106,90
**4,12E-09**	115,89	100,34
**1,37E-09**	113,29	102,00
**4,57E-10**	106,81	93,01
**1,52E-10**	98,57	95,91
**DMSO**	101,20	102,69
**HILLSLOP**	**-1,29**	**-1,31**
**IC50 (M)**	**1,22E-06**	**1,08E-06**

Table S41: The inhibitory activity of **25** against ALK1-6

	Compound IC50 (M)	IC50 (M) Control Cmpd	Control Cmpd ID
**Kinase**	**Compound 25**		
**ALK1/ACVRL1**	**1,48E-06**	**7,34E-09**	**LDN193189**
**ALK2/ACVR1**	**5,69E-07**	**3,03E-08**	**LDN193189**
**ALK3/BMPR1A**		**5,32E-08**	**LDN193189**
**ALK4/ACVR1B**		**1,83E-07**	**LDN193189**
**ALK5/TGFBR1**		**2,55E-07**	**LDN193189**
**ALK6/BMPR1B**	**> 3.00E-06**	**1,08E-08**	**LDN193189**

ALK1/ACVRL1 (%Activity)

Conc.(M)	11
**3,00E-06**	31,55
**1,00E-06**	59,49
**3,33E-07**	87,91
**1,11E-07**	104,96
**3,70E-08**	106,64
**1,23E-08**	104,16
**4,12E-09**	103,16
**1,37E-09**	97,04
**4,57E-10**	94,35
**1,52E-10**	93,21
**DMSO**	100,38
**HILLSLOPE**	**-1,31**
**IC50 (M)**	**1,48E-06**

ALK2/ACVR1 (%Activity)

Conc.(M)	11
**3,00E-06**	13,91
**1,00E-06**	42,12
**3,33E-07**	64,96
**1,11E-07**	86,67
**3,70E-08**	104,24
**1,23E-08**	113,71
**4,12E-09**	108,82
**1,37E-09**	101,45
**4,57E-10**	104,64
**1,52E-10**	105,60
**DMSO**	101,42
**HILLSLOPE**	**-1,04**
**IC50 (M)**	**5,69E-07**

ALK3/BMPR1A (%Activity)

Conc.(M)	11
**3,00E-06**	82,02
**1,00E-06**	93,79
**3,33E-07**	97,49
**1,11E-07**	97,92
**3,70E-08**	98,47
**1,23E-08**	97,65
**4,12E-09**	101,73
**1,37E-09**	100,48
**4,57E-10**	99,81
**1,52E-10**	82,94
**DMSO**	99,53
**HILLSLOPE**	
**IC50 (M)**	

ALK4/ACVR1B (%Activity)

Conc.(M)	11
**3,00E-06**	88,54
**1,00E-06**	107,41
**3,33E-07**	102,52
**1,11E-07**	99,20
**3,70E-08**	98,56
**1,23E-08**	95,30
**4,12E-09**	92,07
**1,37E-09**	100,57
**4,57E-10**	94,64
**1,52E-10**	102,60
**DMSO**	96,76
**HILLSLOPE**	
**IC50 (M)**	

ALK5/TGFBR1 (%Activity)

Conc.(M)	11
**3,00E-06**	88,53
**1,00E-06**	104,91
**3,33E-07**	106,37
**1,11E-07**	108,38
**3,70E-08**	100,00
**1,23E-08**	104,70
**4,12E-09**	101,14
**1,37E-09**	104,26
**4,57E-10**	99,08
**1,52E-10**	102,10
**DMSO**	101,68
**HILLSLOPE**	
**IC50 (M)**	

ALK6/BMPR1B (%Activity)

Conc.(M)	11
**3,00E-06**	56,32
**1,00E-06**	92,65
**3,33E-07**	113,77
**1,11E-07**	109,41
**3,70E-08**	103,50
**1,23E-08**	103,69
**4,12E-09**	102,24
**1,37E-09**	107,65
**4,57E-10**	106,89
**1,52E-10**	103,36
**DMSO**	104,48
**HILLSLOPE**	**-1,91**
**IC50 (M)**	**3,17E-06**

Table S42: The inhibitory activity of **28** against ALK1-6

	Compound IC50 (M):		IC50 (M) Control Cmpd	Control Cmpd ID
**Kinase:**	**Compound 28**	**Compound 28**		
**ALK1/ACVRL1**	**1,20E-06**	**1,08E-06**	**5,95E-09**	**LDN193189**
**ALK2/ACVR1**	**5,31E-07**	**5,75E-07**	**1,06E-08**	**LDN193189**
**ALK3/BMPR1A**			**1,45E-08**	**LDN193189**
**ALK4/ACVR1B**			**1,65E-07**	**LDN193189**
**ALK5/TGFBR1**			**3,12E-07**	**LDN193189**
**ALK6/BMPR1B**	**>3.00E-06**	**>3.00E-06**	**5,81E-09**	**LDN193189**

ALK1/ACVRL1 (%Activity)

**Conc.(M)**	**12**	**12**
**3,00E-06**	25,59	25,14
**1,00E-06**	57,37	56,11
**3,33E-07**	82,12	82,04
**1,11E-07**	100,19	99,73
**3,70E-08**	101,69	107,54
**1,23E-08**	107,00	103,53
**4,12E-09**	100,21	106,62
**1,37E-09**	100,13	97,63
**4,57E-10**	112,37	109,57
**1,52E-10**	91,25	115,10
**DMSO**	102,12	98,01
**HILLSLO**	**-1,21**	**-1,15**
**IC50 (M)**	**1,20E-06**	**1,08E-06**

ALK2/ACVR1 (%Activity)

**Conc.(M)**	**12**	**12**
**3,00E-06**	12,87	14,11
**1,00E-06**	32,22	34,21
**3,33E-07**	66,96	69,61
**1,11E-07**	104,43	105,66
**3,70E-08**	120,82	120,34
**1,23E-08**	119,71	119,44
**4,12E-09**	111,23	112,48
**1,37E-09**	102,56	104,67
**4,57E-10**	101,22	98,62
**1,52E-10**	105,86	103,01
**DMSO**	100,48	101,04
**HILLSLOP**	**-1,49**	**-1,48**
**IC50 (M)**	**5,31E-07**	**5,75E-07**

ALK3/BMPR1A (%Activity)

**Conc.(M)**	**12**	**12**
**3,00E-06**	80,27	84,44
**1,00E-06**	98,15	98,90
**3,33E-07**	105,53	110,58
**1,11E-07**	113,30	118,34
**3,70E-08**	112,06	111,57
**1,23E-08**	114,01	118,65
**4,12E-09**	123,95	124,54
**1,37E-09**	110,36	113,55
**4,57E-10**	114,96	118,47
**1,52E-10**	109,34	112,39
**DMSO**	100,57	99,70
**HILLSLOP**		
**IC50 (M)**		

ALK4/ACVR1B (%Activity)

**Conc.(M)**	**12**	**12**
**3,00E-06**	70,16	68,64
**1,00E-06**	94,91	94,68
**3,33E-07**	105,44	110,41
**1,11E-07**	106,98	111,55
**3,70E-08**	106,29	107,49
**1,23E-08**	109,34	103,03
**4,12E-09**	106,05	106,69
**1,37E-09**	111,19	105,04
**4,57E-10**	111,32	108,02
**1,52E-10**	105,77	107,88
**DMSO**	112,36	105,22
**HILLSLOP**		
**IC50 (M)**		

ALK5/TGFBR1 (%Activity)

**Conc.(M)**	**12**	**12**
**3,00E-06**	93,33	94,32
**1,00E-06**	112,66	107,23
**3,33E-07**	111,40	111,57
**1,11E-07**	114,77	109,74
**3,70E-08**	110,65	115,22
**1,23E-08**	113,47	118,82
**4,12E-09**	112,34	112,57
**1,37E-09**	109,71	116,79
**4,57E-10**	110,73	108,66
**1,52E-10**	107,26	102,03
**DMSO**	102,88	98,98
**HILLSLO**		
**IC50 (M)**		

ALK6/BMPR1B (%Activity)

**Conc.(M)**	**12**	**12**
**3,00E-06**	59,85	56,95
**1,00E-06**	87,62	88,24
**3,33E-07**	110,45	103,11
**1,11E-07**	117,43	108,82
**3,70E-08**	114,83	99,71
**1,23E-08**	113,59	99,64
**4,12E-09**	112,15	103,85
**1,37E-09**	110,74	100,62
**4,57E-10**	102,63	96,23
**1,52E-10**	107,79	95,44
**DMSO**	101,28	93,40
**HILLSLOP**	**-1,38**	**-1,79**
**IC50 (M)**	**3,26E-06**	**3,47E-06**

Table S43: The inhibitory activity of **31** against ALK1-6

Kinase:	Compound IC50 (M): Compound 31	Compound IC50 (M): Compound 31	IC50 (M) Control Cmpd	Control Cmpd ID
ALK1/ACVRL1			5,95E-09	LDN193189
ALK2/ACVR1	1,65E-06	1,65E-06	1,06E-08	LDN193189
ALK3/BMPR1A			1,45E-08	LDN193189
ALK4/ACVR1B			1,65E-07	LDN193189
ALK5/TGFBR1			3,12E-07	LDN193189
ALK6/BMPR1B			5,81E-09	LDN193189

ALK1/ACVRL1 (%Activity)

Conc.(M)	13	13
3,00E-06	69,32	65,92
1,00E-06	87,25	84,93
3,33E-07	104,22	99,61
1,11E-07	105,86	102,17
3,70E-08	103,97	104,92
1,23E-08	102,36	103,33
4,12E-09	98,85	101,56
1,37E-09	104,92	103,06
4,57E-10	104,65	102,50
1,52E-10	106,41	112,17
DMSO	100,63	102,02
HILLSLOPE		
IC50 (M)		

ALK2/ACVR1 (%Activity)

Conc.(M)	13	13
3,00E-06	38,11	38,86
1,00E-06	65,50	61,74
3,33E-07	106,78	101,88
1,11E-07	123,72	121,70
3,70E-08	122,52	119,17
1,23E-08	113,42	112,27
4,12E-09	104,59	102,05
1,37E-09	104,30	103,29
4,57E-10	106,15	105,70
1,52E-10	105,99	104,75
DMSO	98,30	97,73
HILLSLOPE	-1,48	-1,28
IC50 (M)	1,65E-06	1,65E-06

*Value excluded from curve fit

ALK3/BMPR1A (%Activity)

Conc.(M)	13	13
3,00E-06	101,38	100,86
1,00E-06	97,22	99,95
3,33E-07	101,49	102,51
1,11E-07	106,32	105,37
3,70E-08	103,87	103,09
1,23E-08	108,43	106,97
4,12E-09	105,12	105,64
1,37E-09	102,84	103,82
4,57E-10	104,98	103,71
1,52E-10	104,89	103,98
DMSO	97,81	97,23
HILLSLOPE		
IC50 (M)		

ALK4/ACVR1B (%Activity)

Conc.(M)	13	13
3,00E-06	95,11	89,36
1,00E-06	97,92	85,23
3,33E-07	101,16	89,96
1,11E-07	102,25	93,28
3,70E-08	95,42	90,28
1,23E-08	103,58	96,28
4,12E-09	104,53	93,14
1,37E-09	106,09	91,97
4,57E-10	98,61	91,25
1,52E-10	103,50	93,08
DMSO	100,62	91,79
HILLSLOPE		
IC50 (M)		

ALK5/TGFBR1 (%Activity)

Conc.(M)	13	13
3,00E-06	111,10	111,42
1,00E-06	105,55	105,74
3,33E-07	104,37	107,75
1,11E-07	104,10	108,14
3,70E-08	101,48	105,40
1,23E-08	103,66	101,70
4,12E-09	112,60	109,92
1,37E-09	107,56	106,58
4,57E-10	100,89	97,12
1,52E-10	105,35	99,74
DMSO	98,79	94,85
HILLSLOPE		
IC50 (M)		

ALK6/BMPR1B (%Activity)

Conc.(M)	13	13
3,00E-06	102,79	109,72
1,00E-06	102,90	114,82
3,33E-07	116,16	116,18
1,11E-07	111,62	110,88
3,70E-08	98,27	106,38
1,23E-08	99,24	108,57
4,12E-09	97,35	102,44
1,37E-09	99,01	106,88
4,57E-10	100,51	109,89
1,52E-10	96,88	109,27
DMSO	94,17	101,30
HILLSLOPE		
IC50 (M)		

Table S44: The inhibitory activity of **34** against ALK1-6

	Compound IC50 (M)	IC50 (M) Control	Control Cmpd ID	
**Kinase**	**Compound 34**	**Compound 34**		
**ALK1/ACVRL1**	**1,26E-07**	**1,42E-07**	**2,48E-08**	**LDN193189**
**ALK2/ACVR1**	**1,26E-07**	**1,35E-07**	**3,04E-08**	**LDN193189**
**ALK3/BMPR1A**	**>3.00E-06**	**>3.00E-06**	**1,61E-08**	**LDN193189**
**ALK4/ACVR1B**			**3,46E-07**	**LDN193189**
**ALK5/TGFBR1**			**5,69E-07**	**LDN193189**
**ALK6/BMPR1B**	**9,73E-07**	**1,09E-06**	**1,26E-08**	**LDN193189**

ALK1/ACVRL1 (%Activity)

**Conc.(M)**	**14**	**14**
**3,00E-06**	5,44	3,92
**1,00E-06**	10,33	9,94
**3,33E-07**	25,29	24,69
**1,11E-07**	46,58	51,20
**3,70E-08**	83,38	86,87
**1,23E-08**	89,98	99,83
**4,12E-09**	107,38	97,36
**1,37E-09**	96,14	100,28
**4,57E-10**	88,15	87,72
**1,52E-10**	92,88	88,37
**DMSO**	99,42	100,92
**HILLSLOP**	**-1,20**	**-1,37**
**IC50 (M)**	**1,26E-07**	**1,42E-07**

ALK2/ACVR1 (%Activity)

**Conc.(M)**	**14**	**14**
**3,00E-06**	3,32	2,15
**1,00E-06**	8,05	8,69
**3,33E-07**	21,03	24,02
**1,11E-07**	51,62	54,11
**3,70E-08**	99,82	107,37
**1,23E-08**	122,63	128,79
**4,12E-09**	111,74	116,83
**1,37E-09**	108,92	110,73
**4,57E-10**	103,56	103,46
**1,52E-10**	94,65	96,68
**DMSO**	97,72	100,48
**HILLSLOPE**	**-1,67**	**-1,70**
**IC50 (M)**	**1,26E-07**	**1,35E-07**

*Value excluded from curve fit

ALK3/BMPR1A (%Activity)

**Conc.(M)**	**14**	**14**
**3,00E-06**	61,58	59,54
**1,00E-06**	87,03	84,43
**3,33E-07**	104,43	102,89
**1,11E-07**	97,06	97,20
**3,70E-08**	85,12	93,59
**1,23E-08**	93,98	104,43
**4,12E-09**	105,81	101,60
**1,37E-09**	94,65	92,85
**4,57E-10**	95,60	89,17
**1,52E-10**	107,71	89,21
**DMSO**	106,59	100,13
**HILLSLOPE**	**-1,48**	**-1,65**
**IC50 (M)**	**4,02E-06**	**3,93E-06**

*Value excluded from curve fit

ALK4/ACVR1B (%Activity)

**Conc.(M)**	**14**	**14**
**3,00E-06**	96,92	101,14
**1,00E-06**	102,63	94,63
**3,33E-07**	98,31	91,94
**1,11E-07**	91,36	89,39
**3,70E-08**	99,93	87,27
**1,23E-08**	93,73	90,74
**4,12E-09**	88,61	95,21
**1,37E-09**	95,98	94,82
**4,57E-10**	103,16	95,88
**1,52E-10**	99,77	94,89
**DMSO**	105,35	99,53
**HILLSLOP**		
**IC50 (M)**		

ALK5/TGFBR1 (%Activity)

**Conc.(M)**	**14**	**14**
**3,00E-06**	110,08	92,29
**1,00E-06**	112,05	92,85
**3,33E-07**	106,79	99,81
**1,11E-07**	109,34	87,58
**3,70E-08**	98,07	90,59
**1,23E-08**	108,81	93,00
**4,12E-09**	98,08	94,33
**1,37E-09**	105,22	94,13
**4,57E-10**	103,91	99,90
**1,52E-10**	104,42	96,21
**DMSO**	105,63	95,44
**HILLSLOPE**		
**IC50 (M)**		

ALK6/BMPR1B (%Activity)

**Conc.(M)**	**14**	**14**
**3,00E-06**	24,37	26,65
**1,00E-06**	48,18	52,67
**3,33E-07**	86,73	85,88
**1,11E-07**	101,97	103,48
**3,70E-08**	112,07	113,12
**1,23E-08**	105,63	106,39
**4,12E-09**	104,55	109,71
**1,37E-09**	108,67	105,34
**4,57E-10**	107,19	103,12
**1,52E-10**	106,44	99,22
**DMSO**	98,65	100,99
**HILLSLOPE**	**-1,30**	**-1,26**
**IC50 (M)**	**9,73E-07**	**1,09E-06**

Table S45: The inhibitory activity of **37** against ALK1-6

	Compound IC50 (M)	IC50 (M) Control	Control Cmpd ID	
**Kinase**	**Compound 37**	**Compound 37**		
**ALK1/ACVRL1**			**2,48E-08**	**LDN193189**
**ALK2/ACVR1**	**3,00E-06**	**2,99E-06**	**3,04E-08**	**LDN193189**
**ALK3/BMPR1A**			**1,61E-08**	**LDN193189**
**ALK4/ACVR1B**			**3,46E-07**	**LDN193189**
**ALK5/TGFBR1**			**5,69E-07**	**LDN193189**
**ALK6/BMPR1B**			**1,26E-08**	**LDN193189**

ALK1/ACVRL1 (%Activity)

Conc.(M)	15	15
**3,00E-06**	61,27	62,30
**1,00E-06**	103,04	93,68
**3,33E-07**	116,00	105,59
**1,11E-07**	106,44	101,63
**3,70E-08**	98,89	98,03
**1,23E-08**	99,62	95,48
**4,12E-09**	95,47	90,36
**1,37E-09**	96,96	87,66
**4,57E-10**	91,04	88,74
**1,52E-10**	88,07	85,14
**DMSO**	90,54	104,49
**HILLSLO**		
**IC50 (M)**		

ALK2/ACVR1 (%Activity)

Conc.(M)	15	15
**3,00E-06**	48,74	49,54
**1,00E-06**	100,61	107,59
**3,33E-07**	142,83	143,00
**1,11E-07**	132,89	141,34
**3,70E-08**	129,49	128,91
**1,23E-08**	109,53	111,59
**4,12E-09**	97,31	99,88
**1,37E-09**	90,57	100,83
**4,57E-10**	102,18	103,99
**1,52E-10**	93,83	92,41
**DMSO**	97,30	97,48
**HILLSLOP**	**-15,86**	**-18,65**
**IC50 (M)**	**3,00E-**	**2,99E-06**

*Value excluded from curve fit

ALK3/BMPR1A (%Activity)

Conc.(M)	15	15
**3,00E-06**	86,22	95,42
**1,00E-06**	97,42	90,00
**3,33E-07**	98,66	95,16
**1,11E-07**	83,44	82,09
**3,70E-08**	90,53	90,15
**1,23E-08**	88,29	97,15
**4,12E-09**	83,60	91,79
**1,37E-09**	85,56	91,73
**4,57E-10**	98,14	96,59
**1,52E-10**	117,45	92,78
**DMSO**	103,92	95,33
**HILLSLOPE**		
**IC50 (M)**		

ALK4/ACVR1B (%Activity)

Conc.(M)	15	15
**3,00E-06**	96,93	95,03
**1,00E-06**	100,76	83,57
**3,33E-07**	96,52	89,72
**1,11E-07**	94,51	89,39
**3,70E-08**	97,97	87,05
**1,23E-08**	106,95	89,44
**4,12E-09**	98,75	91,38
**1,37E-09**	106,93	93,78
**4,57E-10**	107,67	99,97
**1,52E-10**	104,17	95,30
**DMSO**	97,57	99,24
**HILLSLOP**		
**IC50 (M)**		

ALK5/TGFBR1 (%Activity)

Conc.(M)	15	15
**3,00E-06**	96,96	85,54
**1,00E-06**	95,48	82,02
**3,33E-07**	100,24	87,31
**1,11E-07**	89,27	84,39
**3,70E-08**	88,92	84,86
**1,23E-08**	89,38	89,89
**4,12E-09**	91,62	91,40
**1,37E-09**	94,88	92,16
**4,57E-10**	93,31	90,25
**1,52E-10**	95,79	93,95
**DMSO**	94,42	100,86
**HILLSLOP**		
**IC50 (M)**		

ALK6/BMPR1B (%Activity)

Conc.(M)	15	15
**3,00E-06**	95,88	91,26
**1,00E-06**	111,77	108,55
**3,33E-07**	115,67	111,51
**1,11E-07**	105,26	104,20
**3,70E-08**	107,36	104,24
**1,23E-08**	106,67	99,97
**4,12E-09**	100,47	93,07
**1,37E-09**	101,09	99,64
**4,57E-10**	98,43	94,12
**1,52E-10**	100,22	102,70
**DMSO**	102,73	98,37
**HILLSLOP**		
**IC50 (M)**		

Table S46: The inhibitory activity of **39** against ALK1-6

	Compound IC50 (M):	IC50 (M) Control Cmpd	Control Cmpd ID
Kinase:	Compound 39		
ALK1/ACVRL1	3,60E-08	1,14E-08	LDN193189
ALK2/ACVR1	1,48E-08	1,88E-08	LDN193189
ALK3/BMPR1A	6,80E-08	7,37E-08	LDN193189
ALK4/ACVR1B		1,60E-07	LDN193189
ALK5/TGFBR1		3,60E-07	LDN193189
ALK6/BMPR1B	1,44E-08	1,30E-08	LDN193189

ALK1/ACVRL1 (%Activity)

Conc.(M)	16
3,00E-06	14,07
1,00E-06	12,00
3,33E-07	15,44
1,11E-07	29,26
3,70E-08	52,14
1,23E-08	72,34
4,12E-09	90,11
1,37E-09	98,72
4,57E-10	101,26
1,52E-10	103,71
DMSO	103,73
HILLSLOPE	-0,73
IC50 (M)	3,60E-08

ALK2/ACVR1 (%Activity)

Conc.(M)	16
3,00E-06	6,06
1,00E-06	5,40
3,33E-07	6,24
1,11E-07	11,04
3,70E-08	29,16
1,23E-08	57,67
4,12E-09	90,62
1,37E-09	105,15
4,57E-10	107,30
1,52E-10	108,66
DMSO	108,15
HILLSLOPE	-1,11
IC50 (M)	1,48E-08

ALK3/BMPR1A (%Activity)

Conc.(M)	16
3,00E-06	39,30
1,00E-06	48,51
3,33E-07	37,92
1,11E-07	41,88
3,70E-08	52,55
1,23E-08	65,69
4,12E-09	78,68
1,37E-09	88,31
4,57E-10	90,04
1,52E-10	104,74
DMSO	98,88
HILLSLOPE	-0,26
IC50 (M)	6,80E-08

ALK4/ACVR1B (%Activity)

Conc.(M)	16
3,00E-06	87,39
1,00E-06	104,58
3,33E-07	91,03
1,11E-07	90,44
3,70E-08	99,44
1,23E-08	96,17
4,12E-09	95,33
1,37E-09	98,50
4,57E-10	93,60
1,52E-10	97,96
DMSO	103,46
HILLSLOPE	
IC50 (M)	

ALK5/TGFBR1 (%Activity)

Conc.(M)	16
3,00E-06	97,78
1,00E-06	105,53
3,33E-07	100,55
1,11E-07	96,33
3,70E-08	102,91
1,23E-08	104,40
4,12E-09	101,03
1,37E-09	108,47
4,57E-10	110,84
1,52E-10	102,52
DMSO	97,03
HILLSLOPE	
IC50 (M)	

ALK6/BMPR1B (%Activity)

Conc.(M)	16
3,00E-06	11,90
1,00E-06	18,37
3,33E-07	13,71
1,11E-07	19,84
3,70E-08	30,34
1,23E-08	54,31
4,12E-09	78,56
1,37E-09	93,13
4,57E-10	97,55
1,52E-10	103,33
DMSO	103,19
HILLSLOPE	-0,67
IC50 (M)	1,44E-08

Table S47: The inhibitory activity of **41** against ALK1-6

	Compound IC50 (M):	IC50 (M) Control Cmpd	Control Cmpd ID
**Kinase:**	**Compound 41**		
**ALK1/ACVRL1**		**1,14E-08**	**LDN193189**
**ALK2/ACVR1**		**1,88E-08**	**LDN193189**
**ALK3/BMPR1A**		**7,37E-08**	**LDN193189**
**ALK4/ACVR1B**		**1,60E-07**	**LDN193189**
**ALK5/TGFBR1**		**3,60E-07**	**LDN193189**
**ALK6/BMPR1B**		**1,30E-08**	**LDN193189**

ALK1/ACVRL1 (%Activity)

Conc.(M)	17
**3,00E-06**	86,28
**1,00E-06**	87,11
**3,33E-07**	99,94
**1,11E-07**	105,94
**3,70E-08**	101,39
**1,23E-08**	96,11
**4,12E-09**	103,88
**1,37E-09**	110,35
**4,57E-10**	104,14
**1,52E-10**	97,97
**DMSO**	97,46
**HILLSLOPE**	
**IC50 (M)**	

ALK2/ACVR1 (%Activity)

Conc.(M)	17
**3,00E-06**	67,68
**1,00E-06**	61,57
**3,33E-07**	90,02
**1,11E-07**	105,44
**3,70E-08**	104,96
**1,23E-08**	99,02
**4,12E-09**	100,63
**1,37E-09**	101,33
**4,57E-10**	101,11
**1,52E-10**	105,70
**DMSO**	98,94
**HILLSLOPE**	
**IC50 (M)**	

ALK3/BMPR1A (%Activity)

Conc.(M)	17
**3,00E-06**	99,00
**1,00E-06**	105,38
**3,33E-07**	111,47
**1,11E-07**	104,45
**3,70E-08**	97,76
**1,23E-08**	106,08
**4,12E-09**	104,29
**1,37E-09**	106,56
**4,57E-10**	104,99
**1,52E-10**	109,97
**DMSO**	98,09
**HILLSLOPE**	
**IC50 (M)**	

ALK4/ACVR1B (%Activity)

Conc.(M)	17
**3,00E-06**	83,23
**1,00E-06**	91,22
**3,33E-07**	86,74
**1,11E-07**	88,97
**3,70E-08**	93,04
**1,23E-08**	94,51
**4,12E-09**	86,65
**1,37E-09**	97,52
**4,57E-10**	94,06
**1,52E-10**	89,24
**DMSO**	97,17
**HILLSLOPE**	
**IC50 (M)**	

ALK5/TGFBR1 (%Activity)

Conc.(M)	17
**3,00E-06**	107,20
**1,00E-06**	104,33
**3,33E-07**	109,69
**1,11E-07**	100,64
**3,70E-08**	106,68
**1,23E-08**	111,68
**4,12E-09**	102,38
**1,37E-09**	108,70
**4,57E-10**	103,52
**1,52E-10**	101,26
**DMSO**	99,84
**HILLSLOPE**	
**IC50 (M)**	

ALK6/BMPR1B (%Activity)

Conc.(M)	17
**3,00E-06**	84,59
**1,00E-06**	80,04
**3,33E-07**	91,87
**1,11E-07**	101,74
**3,70E-08**	100,76
**1,23E-08**	109,38
**4,12E-09**	100,37
**1,37E-09**	88,78
**4,57E-10**	92,08
**1,52E-10**	95,13
**DMSO**	97,43
**HILLSLOPE**	
**IC50 (M)**	

Table S48: The inhibitory activity of **43** against ALK1-6

	Compound IC50 (M):	IC50 (M) Control Cmpd	Control Cmpd ID
**Kinase:**	**Compound 43**		
**ALK1/ACVRL1**	**2,19E-06**	**1,14E-08**	**LDN193189**
**ALK2/ACVR1**	**1,95E-07**	**1,88E-08**	**LDN193189**
**ALK3/BMPR1A**		**7,37E-08**	**LDN193189**
**ALK4/ACVR1B**		**1,60E-07**	**LDN193189**
**ALK5/TGFBR1**		**3,60E-07**	**LDN193189**
**ALK6/BMPR1B**		**1,30E-08**	**LDN193189**

ALK1/ACVRL1 (%Activity)

Conc.(M)	18
**3,00E-06**	59,79
**1,00E-06**	54,57
**3,33E-07**	68,10
**1,11E-07**	85,55
**3,70E-08**	95,69
**1,23E-08**	100,52
**4,12E-09**	108,41
**1,37E-09**	102,91
**4,57E-10**	104,00
**1,52E-10**	103,28
**DMSO**	104,61
**HILLSLOPE**	**-0,44**
**IC50 (M)**	**2,19E-06**

ALK2/ACVR1 (%Activity)

Conc.(M)	18
**3,00E-06**	27,63
**1,00E-06**	27,87
**3,33E-07**	35,13
**1,11E-07**	61,58
**3,70E-08**	88,54
**1,23E-08**	97,00
**4,12E-09**	102,24
**1,37E-09**	114,39
**4,57E-10**	107,71
**1,52E-10**	107,85
**DMSO**	100,36
**HILLSLOPE**	**-0,68**
**IC50 (M)**	**1,95E-07**

ALK3/BMPR1A (%Activity)

Conc.(M)	18
**3,00E-06**	94,45
**1,00E-06**	99,42
**3,33E-07**	101,72
**1,11E-07**	98,37
**3,70E-08**	96,65
**1,23E-08**	99,51
**4,12E-09**	98,07
**1,37E-09**	100,88
**4,57E-10**	104,00
**1,52E-10**	111,61
**DMSO**	102,56
**HILLSLOPE**	
**IC50 (M)**	

ALK4/ACVR1B (%Activity)

Conc.(M)	18
**3,00E-06**	88,83
**1,00E-06**	93,75
**3,33E-07**	82,17
**1,11E-07**	90,78
**3,70E-08**	96,77
**1,23E-08**	96,31
**4,12E-09**	89,61
**1,37E-09**	95,06
**4,57E-10**	92,84
**1,52E-10**	92,95
**DMSO**	98,09
**HILLSLOPE**	
**IC50 (M)**	

ALK5/TGFBR1 (%Activity)

Conc.(M)	18
**3,00E-06**	106,87
**1,00E-06**	108,68
**3,33E-07**	107,01
**1,11E-07**	100,87
**3,70E-08**	106,58
**1,23E-08**	103,75
**4,12E-09**	101,96
**1,37E-09**	105,98
**4,57E-10**	109,97
**1,52E-10**	99,81
**DMSO**	103,38
**HILLSLOPE**	
**IC50 (M)**	

ALK6/BMPR1B (%Activity)

Conc.(M)	18
**3,00E-06**	76,82
**1,00E-06**	75,17
**3,33E-07**	81,67
**1,11E-07**	94,86
**3,70E-08**	98,33
**1,23E-08**	105,69
**4,12E-09**	113,08
**1,37E-09**	99,75
**4,57E-10**	95,24
**1,52E-10**	103,71
**DMSO**	101,15
**HILLSLOPE**	
**IC50 (M)**	

Table S49: The inhibitory activity of **46** against ALK1-6

	Compound IC50 (M):		IC50 (M) Control Cmpd	Control Cmpd ID
**Kinase:**	**Compound 46**			
**ALK1/ACVRL1**	**1,32E-07**	**1,19E-07**	**1,94E-08**	**LDN193189**
**ALK2/ACVR1**	**7,06E-08**	**7,80E-08**	**1,70E-08**	**LDN193189**
**ALK3/BMPR1A**	**2,85E-06**	**2,96E-06**	**4,44E-08**	**LDN193189**
**ALK4/ACVR1B**		**>3.00E-06**	**2,16E-07**	**LDN193189**
**ALK5/TGFBR1**			**4,69E-07**	**LDN193189**
**ALK6/BMPR1B**	**1,11E-06**	**1,13E-06**	**1,33E-08**	**LDN193189**

ALK1/ACVRL1 (%Activity)

**Conc.(M)**	**19**	**19**
**3,00E-06**	4,09	3,61
**1,00E-06**	10,91	9,49
**3,33E-07**	25,67	28,03
**1,11E-07**	58,01	50,99
**3,70E-08**	85,16	92,46
**1,23E-08**	105,84	102,61
**4,12E-09**	106,88	108,07
**1,37E-09**	111,08	113,11
**4,57E-10**	106,28	110,97
**1,52E-10**	104,13	107,09
**DMSO**	98,15	103,17
**HILLSLOP**	**-1,21**	**-1,20**
**IC50 (M)**	**1,32E-07**	**1,19E-07**

ALK2/ACVR1 (%Activity)

**Conc.(M)**	**19**	**19**
**3,00E-06**	2,45	2,50
**1,00E-06**	6,08	6,10
**3,33E-07**	15,10	14,11
**1,11E-07**	38,67	38,45
**3,70E-08**	78,46	82,00
**1,23E-08**	117,15	108,15
**4,12E-09**	125,19	113,41
**1,37E-09**	116,77	113,67
**4,57E-10**	114,53	109,92
**1,52E-10**	116,40	106,63
**DMSO**	100,78	101,39
**HILLSLOPE**	**-1,49**	**-1,52**
**IC50 (M)**	**7,06E-08**	**7,80E-08**

ALK3/BMPR1A (%Activity)

**Conc.(M)**	**19**	**19**
**3,00E-06**	53,66	53,13
**1,00E-06**	74,51	75,51
**3,33E-07**	96,22	93,00
**1,11E-07**	107,51	100,96
**3,70E-08**	108,79	105,17
**1,23E-08**	102,71	114,11
**4,12E-09**	113,79	103,19
**1,37E-09**	101,12	102,40
**4,57E-10**	103,51	104,34
**1,52E-10**	105,17	104,40
**DMSO**	99,22	96,92
**HILLSLO**	**-1,04**	**-0,96**
**IC50 (M)**	**2,85E-**	**2,96E-06**

ALK4/ACVR1B (%Activity)

**Conc.(M)**	**19**	**19**
**3,00E-06**	71,49	62,70
**1,00E-06**	96,10	95,69
**3,33E-07**	114,32	114,75
**1,11E-07**	112,30	109,56
**3,70E-08**	111,01	105,63
**1,23E-08**	103,96	107,53
**4,12E-09**	105,70	103,28
**1,37E-09**	101,65	101,84
**4,57E-10**	102,45	99,94
**1,52E-10**	104,99	103,97
**DMSO**	104,09	99,34
**HILLSLOPE**		**-2,10**
**IC50 (M)**		**3,60E-06**

ALK5/TGFBR1 (%Activity)

**Conc.(M)**	**19**	**19**
**3,00E-06**	78,70	84,85
**1,00E-06**	108,79	106,13
**3,33E-07**	109,77	116,21
**1,11E-07**	113,02	112,12
**3,70E-08**	110,48	111,83
**1,23E-08**	111,60	107,79
**4,12E-09**	112,38	114,68
**1,37E-09**	111,19	110,42
**4,57E-10**	111,32	107,85
**1,52E-10**	112,83	109,89
**DMSO**	104,21	104,00
**HILLSLOPE**		
**IC50 (M)**		

ALK6/BMPR1B (%Activity)

**Conc.(M)**	**19**	**19**
**3,00E-06**	24,14	25,73
**1,00E-06**	54,26	55,12
**3,33E-07**	90,87	89,57
**1,11E-07**	109,01	108,28
**3,70E-08**	118,28	118,13
**1,23E-08**	105,99	105,14
**4,12E-09**	107,18	106,19
**1,37E-09**	102,89	104,12
**4,57E-10**	104,05	108,50
**1,52E-10**	104,30	104,26
**DMSO**	102,02	100,47
**HILLSL**	**-1,45**	**-1,38**
**IC50 (M)**	**1,11E-**	**1,13E-06**

Table S50: The inhibitory activity of **49** against ALK1-6

	Compound IC50 (M):	IC50 (M) Control Cmpd	Control Cmpd ID
Kinase:	Compound 49		
ALK1/ACVRL1	3,61E-07	1,18E-08	LDN193189
ALK2/ACVR1	2,06E-07	1,51E-08	LDN193189
ALK3/BMPR1A	2,31E-06	6,32E-08	LDN193189
ALK4/ACVR1B		1,98E-07	LDN193189
ALK5/TGFBR1		3,49E-07	LDN193189
ALK6/BMPR1B	1,38E-06	1,88E-08	LDN193189

ALK1/ACVRL1 (%Activity)		ALK2/ACVR1 (%Activity)		ALK3/BMPR1A (%Activity)	
Conc.(M)	20	Conc.(M)	20	Conc.(M)	20
3,00E-06	11,47	3,00E-06	6,80	3,00E-06	42,17
1,00E-06	28,07	1,00E-06	16,68	1,00E-06	61,67
3,33E-07	51,88	3,33E-07	37,00	3,33E-07	74,37
1,11E-07	84,44	1,11E-07	66,08	1,11E-07	88,53
3,70E-08	107,99	3,70E-08	101,61	3,70E-08	94,18
1,23E-08	106,93	1,23E-08	121,13	1,23E-08	94,45
4,12E-09	114,49	4,12E-09	110,22	4,12E-09	89,60
1,37E-09	108,84	1,37E-09	106,63	1,37E-09	86,45
4,57E-10	103,85	4,57E-10	99,41	4,57E-10	89,93
1,52E-10	100,34	1,52E-10	94,89	1,52E-10	92,90
DMSO	102,47	DMSO	100,70	DMSO	97,30
HILLSLOPE	-1,17	HILLSLOPE	-1,32	HILLSLOPE	-0,85
IC50 (M)	3,61E-07	IC50 (M)	2,06E-07	IC50 (M)	2,31E-06

ALK4/ACVR1B (%Activity)		ALK5/TGFBR1 (%Activity)		ALK6/BMPR1B (%Activity)	
Conc.(M)	20	Conc.(M)	20	Conc.(M)	20
3,00E-06	108,76	3,00E-06	82,82	3,00E-06	30,69
1,00E-06	113,75	1,00E-06	78,72	1,00E-06	52,41
3,33E-07	101,18	3,33E-07	89,50	3,33E-07	80,96
1,11E-07	97,35	1,11E-07	83,69	1,11E-07	90,63
3,70E-08	95,83	3,70E-08	91,36	3,70E-08	101,33
1,23E-08	94,75	1,23E-08	91,05	1,23E-08	94,48
4,12E-09	98,65	4,12E-09	93,68	4,12E-09	91,93
1,37E-09	95,21	1,37E-09	102,88	1,37E-09	95,71
4,57E-10	97,25	4,57E-10	96,94	4,57E-10	91,68
1,52E-10	95,89	1,52E-10	100,35	1,52E-10	91,44
DMSO	93,17	DMSO	102,28	DMSO	97,49
HILLSLOPE		HILLSLOPE		HILLSLOPE	-1,14
IC50 (M)		IC50 (M)		IC50 (M)	1,38E-06

Table S51: The inhibitory activity of **52** against ALK1-6

	Compound IC50 (M):	IC50 (M) Control Cmpd	Control Cmpd ID
**Kinase:**	**Compound 52**		
**ALK1/ACVRL1**		**1,18E-08**	**LDN193189**
**ALK2/ACVR1**	**>3.00E-06**	**1,51E-08**	**LDN193189**
**ALK3/BMPR1A**		**6,32E-08**	**LDN193189**
**ALK4/ACVR1B**		**1,98E-07**	**LDN193189**
**ALK5/TGFBR1**		**3,49E-07**	**LDN193189**
**ALK6/BMPR1B**		**1,88E-08**	**LDN193189**

ALK1/ACVRL1 (%Activity)		ALK2/ACVR1 (%Activity)		ALK3/BMPR1A (%Activity)	
**Conc.(M)**	**21**	**Conc.(M)**	**21**	**Conc.(M)**	**21**
**3,00E-06**	85,10	**3,00E-06**	62,03	**3,00E-06**	90,66
**1,00E-06**	117,20	**1,00E-06**	101,68	**1,00E-06**	104,40
**3,33E-07**	118,59	**3,33E-07**	126,28	**3,33E-07**	100,10
**1,11E-07**	121,16	**1,11E-07**	119,57	**1,11E-07**	100,74
**3,70E-08**	108,93	**3,70E-08**	111,71	**3,70E-08**	98,63
**1,23E-08**	106,69	**1,23E-08**	99,44	**1,23E-08**	98,26
**4,12E-09**	101,73	**4,12E-09**	99,78	**4,12E-09**	101,29
**1,37E-09**	111,77	**1,37E-09**	94,44	**1,37E-09**	102,97
**4,57E-10**	107,72	**4,57E-10**	99,98	**4,57E-10**	109,65
**1,52E-10**	101,46	**1,52E-10**	98,76	**1,52E-10**	107,82
**DMSO**	104,22	**DMSO**	104,96	**DMSO**	103,53
**HILLSLOPE**		**HILLSLOPE**	**-3,06**	**HILLSLOPE**	
**IC50 (M)**		**IC50 (M)**	**3,35E-06**	**IC50 (M)**	

ALK4/ACVR1B (%Activity)		ALK5/TGFBR1 (%Activity)		ALK6/BMPR1B (%Activity)	
**Conc.(M)**	**21**	**Conc.(M)**	**21**	**Conc.(M)**	**21**
**3,00E-06**	110,95	**3,00E-06**	86,13	**3,00E-06**	106,64
**1,00E-06**	110,15	**1,00E-06**	82,95	**1,00E-06**	110,13
**3,33E-07**	102,22	**3,33E-07**	77,50	**3,33E-07**	101,14
**1,11E-07**	100,02	**1,11E-07**	79,67	**1,11E-07**	97,27
**3,70E-08**	95,34	**3,70E-08**	86,93	**3,70E-08**	95,93
**1,23E-08**	98,50	**1,23E-08**	92,59	**1,23E-08**	100,62
**4,12E-09**	97,15	**4,12E-09**	94,83	**4,12E-09**	97,75
**1,37E-09**	97,62	**1,37E-09**	97,26	**1,37E-09**	95,72
**4,57E-10**	95,34	**4,57E-10**	98,14	**4,57E-10**	97,47
**1,52E-10**	102,33	**1,52E-10**	102,48	**1,52E-10**	94,28
**DMSO**	98,91	**DMSO**	94,40	**DMSO**	95,35
**HILLSLOPE**		**HILLSLOPE**		**HILLSLOPE**	
**IC50 (M)**		**IC50 (M)**		**IC50 (M)**	

Table S52: The inhibitory activity of **55** against ALK1-6

	Compound IC50 (M):	IC50 (M) Control Cmpd	Control Cmpd ID
**Kinase:**	**Compound 55**		
**ALK1/ACVRL1**	**1,52E-07**	**7,78E-09**	**LDN193189**
**ALK2/ACVR1**	**6,87E-08**	**7,23E-09**	**LDN193189**
**ALK3/BMPR1A**	**4,57E-07**	**1,79E-08**	**LDN193189**
**ALK4/ACVR1B**		**1,46E-07**	**LDN193189**
**ALK5/TGFBR1**		**3,61E-07**	**LDN193189**
**ALK6/BMPR1B**	**5,32E-07**	**6,46E-09**	**LDN193189**

ALK1/ACVRL1 (%Activity)

Conc.(M)	22
**3,00E-06**	6,09
**1,00E-06**	15,46
**3,33E-07**	32,74
**1,11E-07**	56,60
**3,70E-08**	83,85
**1,23E-08**	97,46
**4,12E-09**	115,15
**1,37E-09**	92,07
**4,57E-10**	99,62
**1,52E-10**	103,85
**DMSO**	102,96
**HILLSLOPE**	**-1,02**
**IC50 (M)**	**1,52E-07**

ALK2/ACVR1 (%Activity)

Conc.(M)	22
**3,00E-06**	1,69
**1,00E-06**	4,90
**3,33E-07**	14,27
**1,11E-07**	39,94
**3,70E-08**	65,67
**1,23E-08**	96,04
**4,12E-09**	104,55
**1,37E-09**	110,25
**4,57E-10**	104,69
**1,52E-10**	101,75
**DMSO**	96,85
**HILLSLOPE**	**-1,19**
**IC50 (M)**	**6,87E-08**

ALK3/BMPR1A (%Activity)

Conc.(M)	22
**3,00E-06**	30,24
**1,00E-06**	35,92
**3,33E-07**	53,61
**1,11E-07**	69,35
**3,70E-08**	79,11
**1,23E-08**	84,03
**4,12E-09**	93,12
**1,37E-09**	100,86
**4,57E-10**	94,84
**1,52E-10**	94,88
**DMSO**	100,44
**HILLSLOPE**	**-0,53**
**IC50 (M)**	**4,57E-07**

ALK4/ACVR1B (%Activity)

Conc.(M)	22
**3,00E-06**	106,90
**1,00E-06**	120,39
**3,33E-07**	107,14
**1,11E-07**	111,17
**3,70E-08**	107,13
**1,23E-08**	103,98
**4,12E-09**	103,45
**1,37E-09**	106,73
**4,57E-10**	105,27
**1,52E-10**	106,53
**DMSO**	104,37
**HILLSLOPE**	
**IC50 (M)**	

ALK5/TGFBR1 (%Activity)

Conc.(M)	22
**3,00E-06**	98,55
**1,00E-06**	94,93
**3,33E-07**	106,17
**1,11E-07**	105,40
**3,70E-08**	108,91
**1,23E-08**	104,59
**4,12E-09**	100,59
**1,37E-09**	96,73
**4,57E-10**	97,73
**1,52E-10**	96,06
**DMSO**	94,89
**HILLSLOPE**	
**IC50 (M)**	

ALK6/BMPR1B (%Activity)

Conc.(M)	22
**3,00E-06**	15,58
**1,00E-06**	32,82
**3,33E-07**	58,47
**1,11E-07**	89,93
**3,70E-08**	105,90
**1,23E-08**	100,07
**4,12E-09**	92,01
**1,37E-09**	105,64
**4,57E-10**	100,22
**1,52E-10**	92,14
**DMSO**	102,16
**HILLSLOPE**	**-1,20**
**IC50 (M)**	**5,32E-07**

Table S53: The inhibitory activity of **57** against ALK1-6

	Compound IC50 (M):	IC50 (M) Control Cmpd	Control Cmpd ID
**Kinase:**	**Compound 57**		
**ALK1/ACVRL1**		**1,58E-08**	**LDN193189**
**ALK2/ACVR1**	**9,36E-07**	**1,68E-08**	**LDN193189**
**ALK3/BMPR1A**		**6,02E-08**	**LDN193189**
**ALK4/ACVR1B**		**4,84E-07**	**LDN193189**
**ALK5/TGFBR1**		**5,23E-07**	**LDN193189**
**ALK6/BMPR1B**	**5,36E-07**	**1,41E-08**	**LDN193189**

ALK1/ACVRL1 (%Activity)

Conc.(M)	23
**3,00E-06**	68,67
**1,00E-06**	65,83
**3,33E-07**	90,63
**1,11E-07**	98,58
**3,70E-08**	105,21
**1,23E-08**	108,11
**4,12E-09**	105,17
**1,37E-09**	99,55
**4,57E-10**	101,35
**1,52E-10**	95,84
**DMSO**	96,40
**HILLSLOPE**	
**IC50 (M)**	

ALK2/ACVR1 (%Activity)

Conc.(M)	23
**3,00E-06**	38,95
**1,00E-06**	36,82
**3,33E-07**	63,12
**1,11E-07**	87,99
**3,70E-08**	101,78
**1,23E-08**	96,90
**4,12E-09**	92,08
**1,37E-09**	92,98
**4,57E-10**	95,99
**1,52E-10**	93,88
**DMSO**	99,79
**HILLSLOPE**	**-0,80**
**IC50 (M)**	**9,36E-07**

ALK3/BMPR1A (%Activity)

Conc.(M)	23
**3,00E-06**	68,00
**1,00E-06**	61,62
**3,33E-07**	71,49
**1,11E-07**	81,76
**3,70E-08**	87,58
**1,23E-08**	86,95
**4,12E-09**	98,16
**1,37E-09**	94,58
**4,57E-10**	94,33
**1,52E-10**	93,62
**DMSO**	102,01
**HILLSLOPE**	
**IC50 (M)**	

ALK4/ACVR1B (%Activity)

Conc.(M)	23
**3,00E-06**	128,90
**1,00E-06**	129,40
**3,33E-07**	122,63
**1,11E-07**	109,88
**3,70E-08**	112,02
**1,23E-08**	109,09
**4,12E-09**	106,79
**1,37E-09**	110,81
**4,57E-10**	110,19
**1,52E-10**	107,68
**DMSO**	102,08
**HILLSLOPE**	
**IC50 (M)**	

ALK5/TGFBR1 (%Activity)

Conc.(M)	23
**3,00E-06**	91,94
**1,00E-06**	97,24
**3,33E-07**	101,10
**1,11E-07**	95,98
**3,70E-08**	97,45
**1,23E-08**	94,25
**4,12E-09**	96,70
**1,37E-09**	95,87
**4,57E-10**	96,97
**1,52E-10**	97,22
**DMSO**	96,98
**HILLSLOPE**	
**IC50 (M)**	

ALK6/BMPR1B (%Activity)

Conc.(M)	23
**3,00E-06**	22,42
**1,00E-06**	36,33
**3,33E-07**	60,58
**1,11E-07**	83,46
**3,70E-08**	102,70
**1,23E-08**	107,78
**4,12E-09**	100,98
**1,37E-09**	104,25
**4,57E-10**	105,13
**1,52E-10**	101,31
**DMSO**	100,81
**HILLSLOPE**	**-0,93**
**IC50 (M)**	**5,36E-07**

Table S54: The inhibitory activity of **60** against ALK1-6

	Compound IC50 (M):	IC50 (M) Control Cmpd	Control Cmpd ID
Kinase:	Compound 60		
ALK1/ACVRL1	>3.00E-06	7,58E-09	LDN193189
ALK2/ACVR1	1,22E-06	1,57E-08	LDN193189
ALK3/BMPR1A		3,75E-08	LDN193189
ALK4/ACVR1B		2,45E-07	LDN193189
ALK5/TGFBR1		7,89E-07	LDN193189
ALK6/BMPR1B		1,66E-08	LDN193189

ALK1/ACVRL1 (%Activity)		ALK2/ACVR1 (%Activity)		ALK3/BMPR1A (%Activity)	
Conc.(M)	24	Conc.(M)	24	Conc.(M)	24
3,00E-06	58,07	3,00E-06	28,75	3,00E-06	89,91
1,00E-06	84,45	1,00E-06	66,37	1,00E-06	98,29
3,33E-07	94,85	3,33E-07	73,20	3,33E-07	99,46
1,11E-07	90,52	1,11E-07	109,73	1,11E-07	106,90
3,70E-08	81,83	3,70E-08	110,68	3,70E-08	117,45
1,23E-08	89,92	1,23E-08	110,34	1,23E-08	116,46
4,12E-09	98,18	4,12E-09	108,32	4,12E-09	109,32
1,37E-09	88,48	1,37E-09	105,48	1,37E-09	106,15
4,57E-10	114,22	4,57E-10	107,83	4,57E-10	113,99
1,52E-10	101,90	1,52E-10	106,78	1,52E-10	109,87
DMSO	101,85	DMSO	103,00	DMSO	96,91
HILLSLOPE	-1,461	HILLSLOPE	-1,05	HILLSLOPE	
IC50 (M)	4,06E-06	IC50 (M)	1,22E-06	IC50 (M)	
*Data excluded from curve fit					

ALK4/ACVR1B (%Activity)		ALK5/TGFBR1 (%Activity)		ALK6/BMPR1B (%Activity)	
Conc.(M)	24	Conc.(M)	24	Conc.(M)	24
3,00E-06	98,71	3,00E-06	82,94	3,00E-06	101,46
1,00E-06	89,25	1,00E-06	79,77	1,00E-06	102,82
3,33E-07	87,30	3,33E-07	86,42	3,33E-07	110,26
1,11E-07	86,64	1,11E-07	85,25	1,11E-07	116,10
3,70E-08	88,59	3,70E-08	86,39	3,70E-08	109,31
1,23E-08	89,69	1,23E-08	84,00	1,23E-08	102,87
4,12E-09	89,68	4,12E-09	98,59	4,12E-09	98,43
1,37E-09	90,52	1,37E-09	97,08	1,37E-09	95,03
4,57E-10	91,28	4,57E-10	97,78	4,57E-10	94,22
1,52E-10	85,19	1,52E-10	95,80	1,52E-10	95,57
DMSO	100,00	DMSO	101,53	DMSO	102,92
HILLSLOPE		HILLSLOPE		HILLSLOPE	
IC50 (M)		IC50 (M)		IC50 (M)	

Table S55: The inhibitory activity of **63** against ALK1-6

Kinase:	Compound IC50 (M): Compound 63	IC50 (M) Control Cmpd	Control Cmpd ID
ALK1/ACVRL1	>3.00E-06	1,64E-08	LDN193189
ALK2/ACVR1	5,10E-07	1,28E-08	LDN193189
ALK3/BMPR1A	>3.00E-06	9,80E-09	LDN193189
ALK4/ACVR1B		5,63E-08	LDN193189
ALK5/TGFBR1		2,40E-07	LDN193189
ALK6/BMPR1B	2,51E-06	7,75E-09	LDN193189

ALK1/ACVRL1 (%Activity)

Conc.(M)	25
3,00E-06	55,62
1,00E-06	94,04
3,33E-07	117,62
1,11E-07	120,05
3,70E-08	113,35
1,23E-08	102,76
4,12E-09	102,35
1,37E-09	94,95
4,57E-10	104,31
1,52E-10	109,53
DMSO	97,27
HILLSLOPE	-2,17
IC50 (M)	3,15E-06

*Point excluded from curve fit

ALK2/ACVR1 (%Activity)

Conc.(M)	25
3,00E-06	12,62
1,00E-06	31,92
3,33E-07	61,04
1,11E-07	88,48
3,70E-08	106,70
1,23E-08	105,50
4,12E-09	114,18
1,37E-09	99,57
4,57E-10	95,81
1,52E-10	96,44
DMSO	94,20
HILLSLOPE	-1,26
IC50 (M)	5,10E-07

ALK3/BMPR1A (%Activity)

Conc.(M)	25
3,00E-06	54,10
1,00E-06	76,63
3,33E-07	89,19
1,11E-07	91,58
3,70E-08	94,70
1,23E-08	97,53
4,12E-09	100,57
1,37E-09	103,32
4,57E-10	102,12
1,52E-10	103,01
DMSO	99,89
HILLSLOPE	-0,74
IC50 (M)	3,99E-06

ALK4/ACVR1B (%Activity)

Conc.(M)	25
3,00E-06	82,31
1,00E-06	90,67
3,33E-07	94,05
1,11E-07	92,31
3,70E-08	98,65
1,23E-08	76,23
4,12E-09	91,94
1,37E-09	97,05
4,57E-10	92,79
1,52E-10	101,16
DMSO	95,99
HILLSLOPE	
IC50 (M)	

ALK5/TGFBR1 (%Activity)

Conc.(M)	25
3,00E-06	77,01
1,00E-06	80,07
3,33E-07	85,64
1,11E-07	85,88
3,70E-08	88,10
1,23E-08	92,71
4,12E-09	94,58
1,37E-09	94,82
4,57E-10	103,87
1,52E-10	94,82
DMSO	93,51
HILLSLOPE	
IC50 (M)	

ALK6/BMPR1B (%Activity)

Conc.(M)	25
3,00E-06	40,80
1,00E-06	76,17
3,33E-07	89,90
1,11E-07	103,51
3,70E-08	112,94
1,23E-08	101,48
4,12E-09	101,99
1,37E-09	89,86
4,57E-10	80,66
1,52E-10	87,46
DMSO	96,39
HILLSLOPE	-1,62
IC50 (M)	2,51E-06

*Point excluded from curve fit

Table S56: The inhibitory activity of **66** against ALK1-6

	Compound IC50 (M):	IC50 (M) Control Cmpd	Control Cmpd ID
**Kinase:**	**Compound 66**		
**ALK1/ACVRL1**	**1,36E-06**	**1,46E-08**	**LDN193189**
**ALK2/ACVR1**	**5,34E-07**	**1,88E-08**	**LDN193189**
**ALK3/BMPR1A**		**1,88E-08**	**LDN193189**
**ALK4/ACVR1B**		**1,72E-07**	**LDN193189**
**ALK5/TGFBR1**		**1,98E-07**	**LDN193189**
**ALK6/BMPR1B**		**1,43E-08**	**LDN193189**

**ALK1/ACVRL1 (%Activity)**		**ALK2/ACVR1 (%Activity)**		**ALK3/BMPR1A (%Activity)**	
**Conc.(M)**	**26**	**Conc.(M)**	**26**	**Conc.(M)**	**26**
**3,00E-06**	30,80	**3,00E-06**	13,12	**3,00E-06**	72,13
**1,00E-06**	64,58	**1,00E-06**	33,27	**1,00E-06**	91,71
**3,33E-07**	86,33	**3,33E-07**	65,71	**3,33E-07**	84,94
**1,11E-07**	106,51	**1,11E-07**	92,27	**1,11E-07**	90,05
**3,70E-08**	109,79	**3,70E-08**	111,34	**3,70E-08**	84,06
**1,23E-08**	111,06	**1,23E-08**	113,91	**1,23E-08**	93,37
**4,12E-09**	108,19	**4,12E-09**	104,47	**4,12E-09**	98,06
**1,37E-09**	109,41	**1,37E-09**	108,87	**1,37E-09**	88,49
**4,57E-10**	106,75	**4,57E-10**	101,61	**4,57E-10**	99,03
**1,52E-10**	105,96	**1,52E-10**	104,81	**1,52E-10**	98,06
**DMSO**	101,64	**DMSO**	93,38	**DMSO**	94,09
**HILLSLOPE**	**-1,17**	**HILLSLOPE**	**-1,29**	**HILLSLOPE**	
**IC50 (M)**	**1,36E-06**	**IC50 (M)**	**5,34E-07**	**IC50 (M)**	
**ALK4/ACVR1B (%Activity)**		**ALK5/TGFBR1 (%Activity)**		**ALK6/BMPR1B (%Activity)**	
**Conc.(M)**	**26**	**Conc.(M)**	**26**	**Conc.(M)**	**26**
**3,00E-06**	117,02	**3,00E-06**	97,10	**3,00E-06**	73,68
**1,00E-06**	118,84	**1,00E-06**	104,17	**1,00E-06**	89,11
**3,33E-07**	114,22	**3,33E-07**	101,53	**3,33E-07**	143,51
**1,11E-07**	116,20	**1,11E-07**	102,25	**1,11E-07**	110,52
**3,70E-08**	115,92	**3,70E-08**	96,79	**3,70E-08**	106,47
**1,23E-08**	112,30	**1,23E-08**	93,46	**1,23E-08**	107,05
**4,12E-09**	107,95	**4,12E-09**	98,76	**4,12E-09**	104,15
**1,37E-09**	111,89	**1,37E-09**	94,46	**1,37E-09**	102,24
**4,57E-10**	104,14	**4,57E-10**	93,94	**4,57E-10**	109,57
**1,52E-10**	101,64	**1,52E-10**	101,22	**1,52E-10**	111,24
**DMSO**	96,61	**DMSO**	96,80	**DMSO**	99,43
**HILLSLOPE**		**HILLSLOPE**		**HILLSLOPE**	
**IC50 (M)**		**IC50 (M)**		**IC50 (M)**	

Table S57: The inhibitory activity of **69** against ALK1-6

	Compound IC50 (M):	IC50 (M) Control Cmpd	Control Cmpd ID
**Kinase:**	**Compound 69**		
**ALK1/ACVRL1**	**5,01E-07**	**7,58E-09**	**LDN193189**
**ALK2/ACVR1**	**2,53E-07**	**1,57E-08**	**LDN193189**
**ALK3/BMPR1A**		**3,75E-08**	**LDN193189**
**ALK4/ACVR1B**		**2,45E-07**	**LDN193189**
**ALK5/TGFBR1**		**7,89E-07**	**LDN193189**
**ALK6/BMPR1B**	**>3.00E-06**	**1,66E-08**	**LDN193189**

ALK1/ACVRL1 (%Activity)

Conc.(M)	27
**3,00E-06**	25,62
**1,00E-06**	37,82
**3,33E-07**	57,93
**1,11E-07**	75,76
**3,70E-08**	78,64
**1,23E-08**	86,37
**4,12E-09**	97,55
**1,37E-09**	95,30
**4,57E-10**	95,52
**1,52E-10**	100,68
**DMSO**	103,80
**HILLSLOPE**	**-0,58**
**IC50 (M)**	**5,01E-07**

ALK2/ACVR1 (%Activity)

Conc.(M)	27
**3,00E-06**	7,10
**1,00E-06**	16,64
**3,33E-07**	40,17
**1,11E-07**	65,17
**3,70E-08**	92,14
**1,23E-08**	97,82
**4,12E-09**	96,45
**1,37E-09**	92,37
**4,57E-10**	94,46
**1,52E-10**	92,82
**DMSO**	94,42
**HILLSLOPE**	**-1,20**
**IC50 (M)**	**2,53E-07**

ALK3/BMPR1A (%Activity)

Conc.(M)	27
**3,00E-06**	74,54
**1,00E-06**	82,14
**3,33E-07**	87,45
**1,11E-07**	94,18
**3,70E-08**	103,20
**1,23E-08**	105,69
**4,12E-09**	108,71
**1,37E-09**	108,04
**4,57E-10**	100,86
**1,52E-10**	118,71
**DMSO**	106,41
**HILLSLOPE**	
**IC50 (M)**	

ALK4/ACVR1B (%Activity)

Conc.(M)	27
**3,00E-06**	117,86
**1,00E-06**	92,20
**3,33E-07**	96,35
**1,11E-07**	90,80
**3,70E-08**	90,24
**1,23E-08**	86,84
**4,12E-09**	91,20
**1,37E-09**	86,76
**4,57E-10**	102,18
**1,52E-10**	97,76
**DMSO**	99,55
**HILLSLOPE**	
**IC50 (M)**	

ALK5/TGFBR1 (%Activity)

Conc.(M)	27
**3,00E-06**	86,18
**1,00E-06**	84,77
**3,33E-07**	92,25
**1,11E-07**	84,59
**3,70E-08**	85,46
**1,23E-08**	91,41
**4,12E-09**	94,89
**1,37E-09**	98,12
**4,57E-10**	100,60
**1,52E-10**	93,57
**DMSO**	106,69
**HILLSLOPE**	
**IC50 (M)**	

ALK6/BMPR1B (%Activity)

Conc.(M)	27
**3,00E-06**	62,85
**1,00E-06**	68,49
**3,33E-07**	91,13
**1,11E-07**	102,63
**3,70E-08**	103,95
**1,23E-08**	105,64
**4,12E-09**	102,10
**1,37E-09**	102,06
**4,57E-10**	101,25
**1,52E-10**	96,96
**DMSO**	103,61
**HILLSLOPE**	**-0,76**
**IC50 (M)**	**4,23E-06**

Table S58: The inhibitory activity of **72** against ALK1-6

Kinase:	Compound IC50 (M): Compound 72	IC50 (M) Control Cmpd	Control Cmpd ID
ALK1/ACVRL1	2,03E-06	1,64E-08	LDN193189
ALK2/ACVR1	2,63E-07	1,28E-08	LDN193189
ALK3/BMPR1A	>3.00E-06	9,80E-09	LDN193189
ALK4/ACVR1B	>3.00E-06	5,63E-08	LDN193189
ALK5/TGFBR1		2,40E-07	LDN193189
ALK6/BMPR1B	5,24E-07	7,75E-09	LDN193189

ALK1/ACVRL1 (%Activity)

Conc.(M)	28
3,00E-06	39,78
1,00E-06	74,76
3,33E-07	103,43
1,11E-07	125,32
3,70E-08	119,95
1,23E-08	113,21
4,12E-09	105,37
1,37E-09	90,72
4,57E-10	106,46
1,52E-10	100,97
DMSO	102,63
HILLSLOPE	-1,48
IC50 (M)	2,03E-06

*Point excluded from curve fit

ALK2/ACVR1 (%Activity)

Conc.(M)	28
3,00E-06	6,99
1,00E-06	19,32
3,33E-07	47,06
1,11E-07	78,00
3,70E-08	111,09
1,23E-08	111,95
4,12E-09	126,13
1,37E-09	120,24
4,57E-10	111,86
1,52E-10	111,88
DMSO	102,27
HILLSLOPE	-1,27
IC50 (M)	2,63E-07

*Point excluded from curve fit

ALK3/BMPR1A (%Activity)

Conc.(M)	28
3,00E-06	52,15
1,00E-06	65,53
3,33E-07	77,61
1,11E-07	90,88
3,70E-08	101,04
1,23E-08	90,24
4,12E-09	98,60
1,37E-09	95,38
4,57E-10	101,58
1,52E-10	101,50
DMSO	98,64
HILLSLOPE	-0,63
IC50 (M)	3,17E-06

ALK4/ACVR1B (%Activity)

Conc.(M)	28
3,00E-06	61,40
1,00E-06	78,41
3,33E-07	89,26
1,11E-07	94,74
3,70E-08	98,06
1,23E-08	93,54
4,12E-09	92,97
1,37E-09	94,16
4,57E-10	98,43
1,52E-10	105,34
DMSO	101,93
HILLSLOPE	-0,77
IC50 (M)	5,84E-06

ALK5/TGFBR1 (%Activity)

Conc.(M)	28
3,00E-06	81,74
1,00E-06	95,43
3,33E-07	87,92
1,11E-07	94,46
3,70E-08	96,48
1,23E-08	99,63
4,12E-09	100,92
1,37E-09	101,22
4,57E-10	100,58
1,52E-10	104,52
DMSO	106,16
HILLSLOPE	
IC50 (M)	

ALK6/BMPR1B (%Activity)

Conc.(M)	28
3,00E-06	15,16
1,00E-06	34,86
3,33E-07	49,03
1,11E-07	94,27
3,70E-08	103,73
1,23E-08	94,48
4,12E-09	100,36
1,37E-09	99,03
4,57E-10	85,37
1,52E-10	88,93
DMSO	101,19
HILLSLOPE	-1,22
IC50 (M)	5,24E-07

Table S59: The inhibitory activity of **73** against ALK1-6

	Compound IC50 (M):	IC50 (M) Control Cmpd	Control Cmpd ID
Kinase:	Compound 29		
ALK1/ACVRL1	>3.00E-06	1,76E-08	LDN193189
ALK2/ACVR1	1,92E-06	2,76E-08	LDN193189
ALK3/BMPR1A		5,65E-08	LDN193189
ALK4/ACVR1B		5,31E-07	LDN193189
ALK5/TGFBR1		8,41E-07	LDN193189
ALK6/BMPR1B		2,00E-08	LDN193189

ALK1/ACVRL1 (%Activity)		ALK2/ACVR1 (%Activity)		ALK3/BMPR1A (%Activity)	
Conc.(M)	29	Conc.(M)	29	Conc.(M)	29
3,00E-06	59,88	3,00E-06	40,42	3,00E-06	104,26
1,00E-06	80,14	1,00E-06	70,01	1,00E-06	113,38
3,33E-07	93,37	3,33E-07	91,18	3,33E-07	102,98
1,11E-07	104,44	1,11E-07	108,61	1,11E-07	108,06
3,70E-08	105,35	3,70E-08	108,53	3,70E-08	101,26
1,23E-08	104,83	1,23E-08	107,29	1,23E-08	105,23
4,12E-09	105,88	4,12E-09	105,89	4,12E-09	102,15
1,37E-09	104,29	1,37E-09	104,10	1,37E-09	102,45
4,57E-10	107,04	4,57E-10	101,28	4,57E-10	103,61
1,52E-10	106,22	1,52E-10	100,04	1,52E-10	98,61
DMSO	102,78	DMSO	102,00	DMSO	98,07
HILLSLOPE	-0,87	HILLSLOPE	-1,19	HILLSLOPE	
IC50 (M)	3,94E-06	IC50 (M)	1,92E-06	IC50 (M)	

ALK4/ACVR1B (%Activity)		ALK5/TGFBR1 (%Activity)		ALK6/BMPR1B (%Activity)	
Conc.(M)	29	Conc.(M)	29	Conc.(M)	29
3,00E-06	107,50	3,00E-06	113,96	3,00E-06	103,09
1,00E-06	105,03	1,00E-06	113,99	1,00E-06	109,96
3,33E-07	99,76	3,33E-07	109,11	3,33E-07	115,01
1,11E-07	102,01	1,11E-07	114,26	1,11E-07	111,74
3,70E-08	103,80	3,70E-08	112,66	3,70E-08	105,92
1,23E-08	108,79	1,23E-08	104,70	1,23E-08	104,41
4,12E-09	99,36	4,12E-09	112,96	4,12E-09	103,00
1,37E-09	104,53	1,37E-09	113,54	1,37E-09	100,93
4,57E-10	107,94	4,57E-10	110,09	4,57E-10	101,89
1,52E-10	102,46	1,52E-10	112,46	1,52E-10	102,05
DMSO	97,02	DMSO	99,68	DMSO	101,67
HILLSLOPE		HILLSLOPE		HILLSLOPE	
IC50 (M)		IC50 (M)		IC50 (M)	

Table S60: The inhibitory activity of **76** against ALK1-6

Kinase	Compound IC50 (M): Compound 76	IC50 (M) Control Cmpd	Control Cmpd ID
ALK1/ACVRL1		1,42E-08	LDN193189
ALK2/ACVR1	2,40E-07	7,72E-09	LDN193189
ALK3/BMPR1A		2,98E-08	LDN193189
ALK4/ACVR1B		3,31E-07	LDN193189
ALK5/TGFBR1		5,36E-07	LDN193189
ALK6/BMPR1B		1,25E-08	LDN193189

ALK1/ACVRL1 (%Activity)

Conc.(M)	30
3,00E-06	69,48
1,00E-06	71,10
3,33E-07	74,21
1,11E-07	70,00
3,70E-08	94,04
1,23E-08	104,75
4,12E-09	96,69
1,37E-09	94,94
4,57E-10	92,74
1,52E-10	86,36
DMSO	97,49
HILLSLOPE	
IC50 (M)	

ALK2/ACVR1 (%Activity)

Conc.(M)	30
3,00E-06	49,83
1,00E-06	37,53
3,33E-07	41,39
1,11E-07	51,92
3,70E-08	110,81
1,23E-08	111,23
4,12E-09	108,26
1,37E-09	108,46
4,57E-10	103,82
1,52E-10	100,32
DMSO	100,54
HILLSLOPE	-0,92
IC50 (M)	2,40E-07

*Point excluded from curve fit

ALK3/BMPR1A (%Activity)

Conc.(M)	30
3,00E-06	110,54
1,00E-06	99,79
3,33E-07	88,37
1,11E-07	83,58
3,70E-08	85,62
1,23E-08	91,86
4,12E-09	93,91
1,37E-09	100,23
4,57E-10	107,95
1,52E-10	106,78
DMSO	104,13
HILLSLOPE	
IC50 (M)	

ALK4/ACVR1B (%Activity)

Conc.(M)	30
3,00E-06	195,77
1,00E-06	136,43
3,33E-07	110,50
1,11E-07	106,34
3,70E-08	99,35
1,23E-08	100,80
4,12E-09	98,87
1,37E-09	100,62
4,57E-10	99,94
1,52E-10	98,23
DMSO	95,66
HILLSLOPE	
IC50 (M)	

ALK5/TGFBR1 (%Activity)

Conc.(M)	30
3,00E-06	102,46
1,00E-06	106,52
3,33E-07	102,03
1,11E-07	98,90
3,70E-08	101,97
1,23E-08	100,50
4,12E-09	99,05
1,37E-09	96,16
4,57E-10	98,21
1,52E-10	97,71
DMSO	99,61
HILLSLOPE	
IC50 (M)	

ALK6/BMPR1B (%Activity)

Conc.(M)	30
3,00E-06	101,63
1,00E-06	103,27
3,33E-07	97,97
1,11E-07	101,75
3,70E-08	104,19
1,23E-08	101,12
4,12E-09	102,80
1,37E-09	101,67
4,57E-10	95,75
1,52E-10	101,82
DMSO	95,71
HILLSLOPE	
IC50 (M)	

Table S61: The inhibitory activity of **78** against ALK1-6

	Compound IC50 (M):	IC50 (M) Control Cmpd	Control Cmpd ID
**Kinase:**	**Compound 78**		
**ALK1/ACVRL1**	**>3.00E-06**	**1,76E-08**	**LDN193189**
**ALK2/ACVR1**	**6,66E-07**	**2,76E-08**	**LDN193189**
**ALK3/BMPR1A**		**5,65E-08**	**LDN193189**
**ALK4/ACVR1B**		**5,31E-07**	**LDN193189**
**ALK5/TGFBR1**		**8,41E-07**	**LDN193189**
**ALK6/BMPR1B**		**2,00E-08**	**LDN193189**

ALK1/ACVRL1 (%Activity)		ALK2/ACVR1 (%Activity)		ALK3/BMPR1A (%Activity)	
**Conc.(M)**	**31**	**Conc.(M)**	**31**	**Conc.(M)**	**31**
**3,00E-06**	59,28	**3,00E-06**	24,67	**3,00E-06**	110,09
**1,00E-06**	63,64	**1,00E-06**	40,71	**1,00E-06**	111,38
**3,33E-07**	86,84	**3,33E-07**	67,80	**3,33E-07**	112,15
**1,11E-07**	99,54	**1,11E-07**	96,06	**1,11E-07**	97,48
**3,70E-08**	98,55	**3,70E-08**	110,77	**3,70E-08**	101,92
**1,23E-08**	105,59	**1,23E-08**	113,09	**1,23E-08**	104,49
**4,12E-09**	99,68	**4,12E-09**	109,75	**4,12E-09**	107,30
**1,37E-09**	97,18	**1,37E-09**	107,56	**1,37E-09**	104,55
**4,57E-10**	99,35	**4,57E-10**	103,52	**4,57E-10**	104,84
**1,52E-10**	101,50	**1,52E-10**	103,53	**1,52E-10**	103,69
**DMSO**	98,68	**DMSO**	102,69	**DMSO**	101,60
**HILLSLOPE**	**-0,72**	**HILLSLOPE**	**-1,05**	**HILLSLOPE**	
**IC50 (M)**	**3,66E-06**	**IC50 (M)**	**6,66E-07**	**IC50 (M)**	

ALK4/ACVR1B (%Activity)		ALK5/TGFBR1 (%Activity)		ALK6/BMPR1B (%Activity)	
**Conc.(M)**	**31**	**Conc.(M)**	**31**	**Conc.(M)**	**31**
**3,00E-06**	127,17	**3,00E-06**	104,33	**3,00E-06**	99,00
**1,00E-06**	116,64	**1,00E-06**	107,41	**1,00E-06**	106,25
**3,33E-07**	104,20	**3,33E-07**	102,37	**3,33E-07**	109,41
**1,11E-07**	100,90	**1,11E-07**	96,25	**1,11E-07**	104,58
**3,70E-08**	101,67	**3,70E-08**	102,61	**3,70E-08**	103,80
**1,23E-08**	102,43	**1,23E-08**	96,35	**1,23E-08**	103,54
**4,12E-09**	96,94	**4,12E-09**	103,56	**4,12E-09**	101,80
**1,37E-09**	98,09	**1,37E-09**	101,29	**1,37E-09**	100,84
**4,57E-10**	92,30	**4,57E-10**	100,47	**4,57E-10**	99,47
**1,52E-10**	100,26	**1,52E-10**	99,26	**1,52E-10**	99,05
**DMSO**	96,44	**DMSO**	98,74	**DMSO**	97,75
**HILLSLOPE**		**HILLSLOPE**		**HILLSLOPE**	
**IC50 (M)**		**IC50 (M)**		**IC50 (M)**	

Table S62: The inhibitory activity of **80** against ALK1-6

	Compound IC50 (M)	IC50 (M) Control Cmpd	Control Cmpd ID
**Kinase**	**Compound 80**		
**ALK1/ACVRL1**	**4,66E-07**	**1,33E-08**	**LDN193189**
**ALK2/ACVR1**	**2,32E-07**	**1,86E-08**	**LDN193189**
**ALK3/BMPR1A**		**1,10E-07**	**LDN193189**
**ALK4/ACVR1B**		**2,18E-07**	**LDN193189**
**ALK5/TGFBR1**		**3,92E-07**	**LDN193189**
**ALK6/BMPR1B**		**1,22E-08**	**LDN193189**

ALK1/ACVRL1 (%Activity)

Conc.(M)	32
3,00E-06	121,28
1,00E-06	104,58
3,33E-07	69,05
1,11E-07	94,38
3,70E-08	115,35
1,23E-08	113,20
4,12E-09	107,27
1,37E-09	108,59
4,57E-10	100,34
1,52E-10	97,46
DMSO	98,37
HILLSLOPE	-1,83
IC50 (M)	4,66E-07

*Point excluded from curve fit

ALK2/ACVR1 (%Activity)

Conc.(M)	32
3,00E-06	71,59
1,00E-06	61,76
3,33E-07	41,92
1,11E-07	66,52
3,70E-08	94,15
1,23E-08	106,05
4,12E-09	102,63
1,37E-09	99,06
4,57E-10	97,78
1,52E-10	97,62
DMSO	97,77
HILLSLOPE	-1,25
IC50 (M)	2,32E-07

*Point excluded from curve fit

ALK3/BMPR1A (%Activity)

Conc.(M)	32
3,00E-06	113,45
1,00E-06	118,55
3,33E-07	105,12
1,11E-07	97,18
3,70E-08	98,12
1,23E-08	109,03
4,12E-09	104,80
1,37E-09	98,95
4,57E-10	96,20
1,52E-10	95,46
DMSO	97,46
HILLSLOPE	
IC50 (M)	

ALK4/ACVR1B (%Activity)

Conc.(M)	32
3,00E-06	117,10
1,00E-06	104,80
3,33E-07	94,46
1,11E-07	97,38
3,70E-08	93,57
1,23E-08	97,74
4,12E-09	95,81
1,37E-09	107,28
4,57E-10	97,20
1,52E-10	93,76
DMSO	98,62
HILLSLOPE	
IC50 (M)	

ALK5/TGFBR1 (%Activity)

Conc.(M)	32
3,00E-06	101,27
1,00E-06	96,04
3,33E-07	98,46
1,11E-07	95,32
3,70E-08	95,84
1,23E-08	94,94
4,12E-09	97,26
1,37E-09	97,16
4,57E-10	100,66
1,52E-10	100,04
DMSO	99,10
HILLSLOPE	
IC50 (M)	

ALK6/BMPR1B (%Activity)

Conc.(M)	32
3,00E-06	127,88
1,00E-06	115,75
3,33E-07	108,06
1,11E-07	107,95
3,70E-08	102,55
1,23E-08	105,50
4,12E-09	97,26
1,37E-09	96,63
4,57E-10	97,11
1,52E-10	94,58
DMSO	96,70
HILLSLOPE	
IC50 (M)	

Table S63: The inhibitory activity of **83** against ALK1-6

	Compound IC50 (M):	IC50 (M) Control Cmpd	Control Cmpd ID
**Kinase:**	**Compound 83**		
**ALK1/ACVRL1**	**2,55E-08**	**6,57E-09**	**LDN193189**
**ALK2/ACVR1**	**2,41E-08**	**1,86E-08**	**LDN193189**
**ALK3/BMPR1A**		**6,05E-08**	**LDN193189**
**ALK4/ACVR1B**		**1,32E-07**	**LDN193189**
**ALK5/TGFBR1**		**2,52E-07**	**LDN193189**
**ALK6/BMPR1B**	**>3.00E-06**	**2,07E-08**	**LDN193189**

ALK1/ACVRL1 (%Activity)

Conc.(M)	33
**3,00E-06**	8,29
**1,00E-06**	8,24
**3,33E-07**	12,86
**1,11E-07**	25,05
**3,70E-08**	43,32
**1,23E-08**	58,38
**4,12E-09**	79,16
**1,37E-09**	89,16
**4,57E-10**	96,77
**1,52E-10**	94,81
**DMSO**	95,78
**HILLSLOPE**	**-0,72**
**IC50 (M)**	**2,55E-08**

ALK2/ACVR1 (%Activity)

Conc.(M)	33
**3,00E-06**	4,44
**1,00E-06**	4,38
**3,33E-07**	8,00
**1,11E-07**	18,78
**3,70E-08**	40,17
**1,23E-08**	61,53
**4,12E-09**	88,78
**1,37E-09**	92,58
**4,57E-10**	97,34
**1,52E-10**	97,07
**DMSO**	99,72
**HILLSLOPE**	**-0,95**
**IC50 (M)**	**2,41E-08**

ALK3/BMPR1A (%Activity)

Conc.(M)	33
**3,00E-06**	65,03
**1,00E-06**	66,55
**3,33E-07**	75,81
**1,11E-07**	98,28
**3,70E-08**	106,27
**1,23E-08**	98,32
**4,12E-09**	108,02
**1,37E-09**	103,35
**4,57E-10**	98,66
**1,52E-10**	104,86
**DMSO**	101,15
**HILLSLOPE**	
**IC50 (M)**	

ALK4/ACVR1B (%Activity)

Conc.(M)	33
**3,00E-06**	94,50
**1,00E-06**	95,76
**3,33E-07**	95,69
**1,11E-07**	98,94
**3,70E-08**	99,73
**1,23E-08**	98,02
**4,12E-09**	93,68
**1,37E-09**	95,21
**4,57E-10**	100,82
**1,52E-10**	98,48
**DMSO**	100,28
**HILLSLOPE**	
**IC50 (M)**	

ALK5/TGFBR1 (%Activity)

Conc.(M)	33
**3,00E-06**	93,25
**1,00E-06**	97,45
**3,33E-07**	97,20
**1,11E-07**	98,28
**3,70E-08**	95,05
**1,23E-08**	95,47
**4,12E-09**	104,56
**1,37E-09**	107,19
**4,57E-10**	99,93
**1,52E-10**	99,45
**DMSO**	104,10
**HILLSLOPE**	
**IC50 (M)**	

ALK6/BMPR1B (%Activity)

Conc.(M)	33
**3,00E-06**	63,81
**1,00E-06**	62,96
**3,33E-07**	80,64
**1,11E-07**	100,25
**3,70E-08**	107,26
**1,23E-08**	98,76
**4,12E-09**	104,17
**1,37E-09**	108,70
**4,57E-10**	103,21
**1,52E-10**	103,13
**DMSO**	100,71
**HILLSLOPE**	**-0,58**
**IC50 (M)**	**3,90E-06**

Table S64: The inhibitory activity of **86** against ALK1-6

	Compound IC50 (M):	IC50 (M) Control Cmpd	Control Cmpd ID
Kinase:	Compound 86		
ALK1/ACVRL1		1,40E-08	LDN193189
ALK2/ACVR1		2,25E-08	LDN193189
ALK3/BMPR1A		5,88E-08	LDN193189
ALK4/ACVR1B		2,59E-07	LDN193189
ALK5/TGFBR1		5,09E-07	LDN193189
ALK6/BMPR1B		1,07E-08	LDN193189

ALK1/ACVRL1 (%Activity)		ALK2/ACVR1 (%Activity)		ALK3/BMPR1A (%Activity)	
Conc.(M)	34	Conc.(M)	34	Conc.(M)	34
3,00E-06	113,19	3,00E-06	109,99	3,00E-06	93,17
1,00E-06	108,55	1,00E-06	99,89	1,00E-06	97,33
3,33E-07	105,27	3,33E-07	112,31	3,33E-07	91,96
1,11E-07	101,54	1,11E-07	101,42	1,11E-07	87,48
3,70E-08	103,46	3,70E-08	92,79	3,70E-08	92,97
1,23E-08	97,36	1,23E-08	85,85	1,23E-08	90,48
4,12E-09	103,38	4,12E-09	90,14	4,12E-09	102,81
1,37E-09	98,94	1,37E-09	88,00	1,37E-09	91,99
4,57E-10	93,42	4,57E-10	95,61	4,57E-10	100,76
1,52E-10	100,35	1,52E-10	104,48	1,52E-10	94,44
DMSO	100,52	DMSO	102,04	DMSO	96,84
HILLSLOPE		HILLSLOPE		HILLSLOPE	
IC50 (M)		IC50 (M)		IC50 (M)	

ALK4/ACVR1B (%Activity)		ALK5/TGFBR1 (%Activity)		ALK6/BMPR1B (%Activity)	
Conc.(M)	34	Conc.(M)	34	Conc.(M)	34
3,00E-06	100,98	3,00E-06	101,01	3,00E-06	98,47
1,00E-06	98,16	1,00E-06	101,09	1,00E-06	92,24
3,33E-07	97,07	3,33E-07	105,40	3,33E-07	93,17
1,11E-07	100,77	1,11E-07	112,36	1,11E-07	92,86
3,70E-08	99,97	3,70E-08	111,64	3,70E-08	97,84
1,23E-08	103,43	1,23E-08	108,34	1,23E-08	97,36
4,12E-09	100,68	4,12E-09	105,81	4,12E-09	94,34
1,37E-09	93,52	1,37E-09	103,55	1,37E-09	99,02
4,57E-10	98,81	4,57E-10	106,99	4,57E-10	101,27
1,52E-10	97,85	1,52E-10	107,27	1,52E-10	100,25
DMSO	101,66	DMSO	109,01	DMSO	99,43
HILLSLOPE		HILLSLOPE		HILLSLOPE	
IC50 (M)		IC50 (M)		IC50 (M)	

Table S65: The inhibitory activity of **89** against ALK1-6

	Compound IC50 (M):	IC50 (M) Control Cmpd	Control Cmpd ID
**Kinase:**	**Compound 89**		
**ALK1/ACVRL1**		**9,45E-09**	**LDN193189**
**ALK2/ACVR1**		**2,56E-08**	**LDN193189**
**ALK3/BMPR1A**		**5,08E-08**	**LDN193189**
**ALK4/ACVR1B**		**1,17E-07**	**LDN193189**
**ALK5/TGFBR1**		**2,37E-07**	**LDN193189**
**ALK6/BMPR1B**		**9,39E-09**	**LDN193189**

ALK1/ACVRL1 (%Activity)

Conc.(M)	35
3,00E-06	111,49
1,00E-06	115,31
3,33E-07	103,35
1,11E-07	95,19
3,70E-08	90,95
1,23E-08	93,74
4,12E-09	93,98
1,37E-09	93,66
4,57E-10	93,72
1,52E-10	90,72
DMSO	93,76
HILLSLOPE	
IC50 (M)	

ALK2/ACVR1 (%Activity)

Conc.(M)	35
3,00E-06	101,16
1,00E-06	111,65
3,33E-07	107,65
1,11E-07	107,27
3,70E-08	108,42
1,23E-08	105,02
4,12E-09	104,50
1,37E-09	103,70
4,57E-10	107,01
1,52E-10	106,33
DMSO	100,70
HILLSLOPE	
IC50 (M)	

ALK3/BMPR1A (%Activity)

Conc.(M)	35
3,00E-06	103,35
1,00E-06	95,39
3,33E-07	96,60
1,11E-07	93,99
3,70E-08	95,98
1,23E-08	99,85
4,12E-09	97,88
1,37E-09	104,84
4,57E-10	109,36
1,52E-10	99,05
DMSO	97,04
HILLSLOPE	
IC50 (M)	

ALK4/ACVR1B (%Activity)

Conc.(M)	35
3,00E-06	118,05
1,00E-06	109,57
3,33E-07	101,85
1,11E-07	102,25
3,70E-08	104,47
1,23E-08	105,50
4,12E-09	110,72
1,37E-09	104,19
4,57E-10	101,40
1,52E-10	106,78
DMSO	99,75
HILLSLOPE	
IC50 (M)	

ALK5/TGFBR1 (%Activity)

Conc.(M)	35
3,00E-06	96,90
1,00E-06	104,97
3,33E-07	105,90
1,11E-07	94,84
3,70E-08	101,13
1,23E-08	99,90
4,12E-09	102,10
1,37E-09	91,20
4,57E-10	91,13
1,52E-10	96,03
DMSO	94,93
HILLSLOPE	
IC50 (M)	

ALK6/BMPR1B (%Activity)

Conc.(M)	35
3,00E-06	95,08
1,00E-06	97,61
3,33E-07	102,41
1,11E-07	101,97
3,70E-08	106,79
1,23E-08	95,65
4,12E-09	100,13
1,37E-09	66,12
4,57E-10	109,13
1,52E-10	105,07
DMSO	96,91
HILLSLOPE	
IC50 (M)	

Table S66: The inhibitory activity of **92** against ALK1-6

Kinase:	Compound IC50 (M): Compound 92	IC50 (M) Control Cmpd	Control Cmpd ID
ALK1/ACVRL1	5,24E-07	9,88E-09	LDN193189
ALK2/ACVR1	3,19E-07	2,24E-08	LDN193189
ALK3/BMPR1A	2,34E-06	6,65E-08	LDN193189
ALK4/ACVR1B		8,20E-08	LDN193189
ALK5/TGFBR1		2,56E-07	LDN193189
ALK6/BMPR1B	1,12E-06	1,90E-08	LDN193189

ALK1/ACVRL1 (%Activity)

Conc.(M)	36
3,00E-06	18,53
1,00E-06	38,14
3,33E-07	62,78
1,11E-07	79,68
3,70E-08	92,57
1,23E-08	99,64
4,12E-09	100,99
1,37E-09	118,42
4,57E-10	99,34
1,52E-10	95,50
DMSO	100,86
HILLSLOPE	-0,85
IC50 (M)	5,24E-07

ALK2/ACVR1 (%Activity)

Conc.(M)	36
3,00E-06	9,52
1,00E-06	22,41
3,33E-07	56,09
1,11E-07	73,10
3,70E-08	93,09
1,23E-08	111,12
4,12E-09	118,15
1,37E-09	106,89
4,57E-10	100,99
1,52E-10	97,82
DMSO	96,29
HILLSLOPE	-1,06
IC50 (M)	3,19E-07

ALK3/BMPR1A (%Activity)

Conc.(M)	36
3,00E-06	51,38
1,00E-06	65,13
3,33E-07	73,20
1,11E-07	91,31
3,70E-08	91,42
1,23E-08	97,96
4,12E-09	105,66
1,37E-09	106,13
4,57E-10	104,90
1,52E-10	106,34
DMSO	106,52
HILLSLOPE	-0,47
IC50 (M)	2,34E-06

ALK4/ACVR1B (%Activity)

Conc.(M)	36
3,00E-06	91,85
1,00E-06	104,14
3,33E-07	101,95
1,11E-07	101,61
3,70E-08	105,08
1,23E-08	111,00
4,12E-09	108,54
1,37E-09	105,03
4,57E-10	98,79
1,52E-10	109,59
DMSO	100,79
HILLSLOPE	
IC50 (M)	

ALK5/TGFBR1 (%Activity)

Conc.(M)	36
3,00E-06	84,24
1,00E-06	80,62
3,33E-07	82,12
1,11E-07	78,46
3,70E-08	85,48
1,23E-08	83,12
4,12E-09	84,07
1,37E-09	86,02
4,57E-10	88,21
1,52E-10	91,96
DMSO	100,00
HILLSLOPE	
IC50 (M)	

ALK6/BMPR1B (%Activity)

Conc.(M)	36
3,00E-06	30,23
1,00E-06	51,96
3,33E-07	76,75
1,11E-07	101,80
3,70E-08	111,22
1,23E-08	111,68
4,12E-09	107,44
1,37E-09	90,69
4,57E-10	96,51
1,52E-10	100,58
DMSO	99,78
HILLSLOPE	-1,12
IC50 (M)	1,12E-06

Table S67: The inhibitory activity of **93** against ALK1-6

	Compound IC50* (M):	IC50 (M) Control Cmpd	Control Cmpd ID
**Kinase:**	**Compound 93**		
**ALK1/ACVRL1**	**>3.00E-06**	**1,46E-08**	**LDN193189**
**ALK2/ACVR1**	**2,82E-06**	**1,88E-08**	**LDN193189**
**ALK3/BMPR1A**	**>3.00E-06**	**1,88E-08**	**LDN193189**
**ALK4/ACVR1B**		**1,72E-07**	**LDN193189**
**ALK5/TGFBR1**		**1,98E-07**	**LDN193189**
**ALK6/BMPR1B**		**1,43E-08**	**LDN193189**

ALK1/ACVRL1 (%Activity)		ALK2/ACVR1 (%Activity)		ALK3/BMPR1A (%Activity)	
**Conc.(M)**	**37**	**Conc.(M)**	**37**	**Conc.(M)**	**37**
**3,00E-06**	56,58	**3,00E-06**	52,77	**3,00E-06**	60,12
**1,00E-06**	85,17	**1,00E-06**	91,20	**1,00E-06**	81,28
**3,33E-07**	98,87	**3,33E-07**	112,78	**3,33E-07**	80,32
**1,11E-07**	102,59	**1,11E-07**	109,85	**1,11E-07**	86,76
**3,70E-08**	94,80	**3,70E-08**	114,13	**3,70E-08**	90,29
**1,23E-08**	103,46	**1,23E-08**	117,28	**1,23E-08**	97,26
**4,12E-09**	100,02	**4,12E-09**	107,45	**4,12E-09**	100,83
**1,37E-09**	100,46	**1,37E-09**	111,42	**1,37E-09**	101,39
**4,57E-10**	103,58	**4,57E-10**	110,92	**4,57E-10**	94,10
**1,52E-10**	98,98	**1,52E-10**	106,03	**1,52E-10**	98,90
**DMSO**	96,78	**DMSO**	98,43	**DMSO**	101,43
**HILLSLOPE**	**-1,44**	**HILLSLOPE**	**-1,69**	**HILLSLOPE**	**-0,47**
**IC50 (M)**	**3,56E-06**	**IC50 (M)**	**2,82E-06**	**IC50 (M)**	**9,61E-06**

ALK4/ACVR1B (%Activity)		ALK5/TGFBR1 (%Activity)		ALK6/BMPR1B (%Activity)	
**Conc.(M)**	**37**	**Conc.(M)**	**37**	**Conc.(M)**	**37**
**3,00E-06**	108,56	**3,00E-06**	101,97	**3,00E-06**	81,62
**1,00E-06**	107,92	**1,00E-06**	110,67	**1,00E-06**	92,25
**3,33E-07**	104,08	**3,33E-07**	106,42	**3,33E-07**	98,91
**1,11E-07**	106,44	**1,11E-07**	104,00	**1,11E-07**	104,67
**3,70E-08**	106,43	**3,70E-08**	99,77	**3,70E-08**	105,78
**1,23E-08**	111,21	**1,23E-08**	104,13	**1,23E-08**	100,71
**4,12E-09**	107,66	**4,12E-09**	106,31	**4,12E-09**	101,47
**1,37E-09**	106,99	**1,37E-09**	104,24	**1,37E-09**	105,85
**4,57E-10**	105,93	**4,57E-10**	103,72	**4,57E-10**	102,68
**1,52E-10**	104,85	**1,52E-10**	96,57	**1,52E-10**	105,12
**DMSO**	93,42	**DMSO**	97,90	**DMSO**	101,23
**HILLSLOPE**		**HILLSLOPE**		**HILLSLOPE**	
**IC50 (M)**		**IC50 (M)**		**IC50 (M)**	

Table S68: The inhibitory activity of **94** against ALK1-6

	Compound IC50 (M):	IC50 (M) Control Cmpd	Control Cmpd ID
**Kinase:**	**Compound 94**		
**ALK1/ACVRL1**		**1,64E-08**	**LDN193189**
**ALK2/ACVR1**	**9,95E-07**	**1,28E-08**	**LDN193189**
**ALK3/BMPR1A**	**>3.00E-06**	**9,80E-09**	**LDN193189**
**ALK4/ACVR1B**		**5,63E-08**	**LDN193189**
**ALK5/TGFBR1**		**2,40E-07**	**LDN193189**
**ALK6/BMPR1B**	**>3.00E-06**	**7,75E-09**	**LDN193189**

ALK1/ACVRL1 (%Activity)

**Conc.(M)**	**38**
**3,00E-06**	78,92
**1,00E-06**	101,91
**3,33E-07**	119,27
**1,11E-07**	106,73
**3,70E-08**	107,52
**1,23E-08**	90,27
**4,12E-09**	98,29
**1,37E-09**	102,79
**4,57E-10**	103,14
**1,52E-10**	105,02
**DMSO**	102,80
**HILLSLOPE**	
**IC50 (M)**	

ALK2/ACVR1 (%Activity)

**Conc.(M)**	**38**
**3,00E-06**	25,98
**1,00E-06**	55,86
**3,33E-07**	83,76
**1,11E-07**	117,84
**3,70E-08**	119,67
**1,23E-08**	118,78
**4,12E-09**	120,76
**1,37E-09**	115,91
**4,57E-10**	108,65
**1,52E-10**	110,85
**DMSO**	100,00
**HILLSLOPE**	**-1,27**
**IC50 (M)**	**9,95E-07**

*Value excluded from curve fit

ALK3/BMPR1A (%Activity)

**Conc.(M)**	**38**
**3,00E-06**	52,01
**1,00E-06**	71,13
**3,33E-07**	86,11
**1,11E-07**	94,65
**3,70E-08**	95,92
**1,23E-08**	102,89
**4,12E-09**	98,49
**1,37E-09**	104,75
**4,57E-10**	107,15
**1,52E-10**	106,02
**DMSO**	101,71
**HILLSLOPE**	**-0,67**
**IC50 (M)**	**3,10E-06**

ALK4/ACVR1B (%Activity)

**Conc.(M)**	**38**
**3,00E-06**	78,06
**1,00E-06**	90,55
**3,33E-07**	93,35
**1,11E-07**	92,97
**3,70E-08**	94,83
**1,23E-08**	96,91
**4,12E-09**	94,81
**1,37E-09**	96,39
**4,57E-10**	95,57
**1,52E-10**	105,92
**DMSO**	100,49
**HILLSLOPE**	
**IC50 (M)**	

ALK5/TGFBR1 (%Activity)

**Conc.(M)**	**38**
**3,00E-06**	84,69
**1,00E-06**	79,63
**3,33E-07**	86,54
**1,11E-07**	90,01
**3,70E-08**	89,96
**1,23E-08**	98,15
**4,12E-09**	104,63
**1,37E-09**	109,22
**4,57E-10**	110,21
**1,52E-10**	106,00
**DMSO**	98,20
**HILLSLOPE**	
**IC50 (M)**	

ALK6/BMPR1B (%Activity)

**Conc.(M)**	**38**
**3,00E-06**	61,88
**1,00E-06**	73,70
**3,33E-07**	89,51
**1,11E-07**	103,45
**3,70E-08**	95,17
**1,23E-08**	90,74
**4,12E-09**	94,88
**1,37E-09**	100,42
**4,57E-10**	90,90
**1,52E-10**	101,24
**DMSO**	104,49
**HILLSLOPE**	**-0,83**
**IC50 (M)**	**5,17E-06**

Table S69: The inhibitory activity of **99** against ALK1-6

	Compound IC50 (M):	IC50 (M) Control Cmpd	Control Cmpd ID
Kinase	Compound 99		
ALK1/ACVRL1		1,33E-08	LDN193189
ALK2/ACVR1		1,86E-08	LDN193189
ALK3/BMPR1A		1,10E-07	LDN193189
ALK4/ACVR1B		2,18E-07	LDN193189
ALK5/TGFBR1		3,92E-07	LDN193189
ALK6/BMPR1B		1,22E-08	LDN193189

ALK1/ACVRL1 (%Activity)		ALK2/ACVR1 (%Activity)		ALK3/BMPR1A (%Activity)	
Conc.(M)	39	Conc.(M)	39	Conc.(M)	39
3,00E-06	109,36	3,00E-06	95,91	3,00E-06	87,71
1,00E-06	106,66	1,00E-06	108,05	1,00E-06	102,75
3,33E-07	98,67	3,33E-07	99,41	3,33E-07	97,10
1,11E-07	96,48	1,11E-07	99,46	1,11E-07	99,75
3,70E-08	103,27	3,70E-08	93,96	3,70E-08	107,52
1,23E-08	96,66	1,23E-08	99,33	1,23E-08	109,17
4,12E-09	98,66	4,12E-09	94,15	4,12E-09	104,87
1,37E-09	99,40	1,37E-09	104,16	1,37E-09	93,96
4,57E-10	96,74	4,57E-10	106,14	4,57E-10	103,01
1,52E-10	90,82	1,52E-10	107,05	1,52E-10	107,68
DMSO	98,80	DMSO	101,18	DMSO	96,99
HILLSLOPE		HILLSLOPE		HILLSLOPE	
IC50 (M)		IC50 (M)		IC50 (M)	

ALK4/ACVR1B (%Activity)		ALK5/TGFBR1 (%Activity)		ALK6/BMPR1B (%Activity)	
Conc.(M)	39	Conc.(M)	39	Conc.(M)	39
3,00E-06	102,05	3,00E-06	94,68	3,00E-06	97,86
1,00E-06	96,05	1,00E-06	94,04	1,00E-06	93,82
3,33E-07	92,84	3,33E-07	97,17	3,33E-07	94,67
1,11E-07	90,60	1,11E-07	94,34	1,11E-07	93,50
3,70E-08	91,03	3,70E-08	92,44	3,70E-08	90,98
1,23E-08	97,65	1,23E-08	97,10	1,23E-08	99,00
4,12E-09	94,35	4,12E-09	94,09	4,12E-09	94,27
1,37E-09	98,55	1,37E-09	103,20	1,37E-09	92,90
4,57E-10	94,73	4,57E-10	103,37	4,57E-10	94,31
1,52E-10	94,57	1,52E-10	108,27	1,52E-10	94,49
DMSO	96,43	DMSO	102,12	DMSO	97,81
HILLSLOPE		HILLSLOPE		HILLSLOPE	
IC50 (M)		IC50 (M)		IC50 (M)	

17. NMR, FTIR, and HRMS spectra of prepared compounds

^{1}H (500 MHz) and ^{13}C NMR (126 MHz) spectra of **2** in DMSO-d_6.

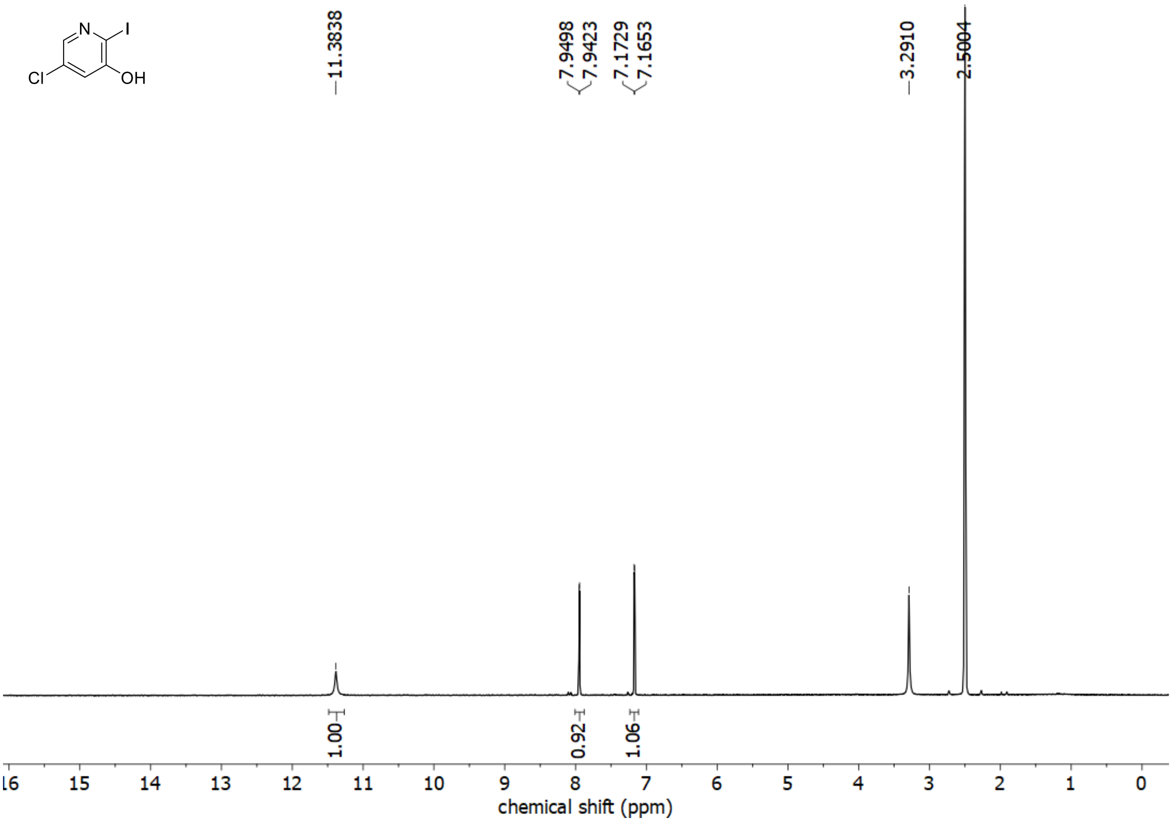

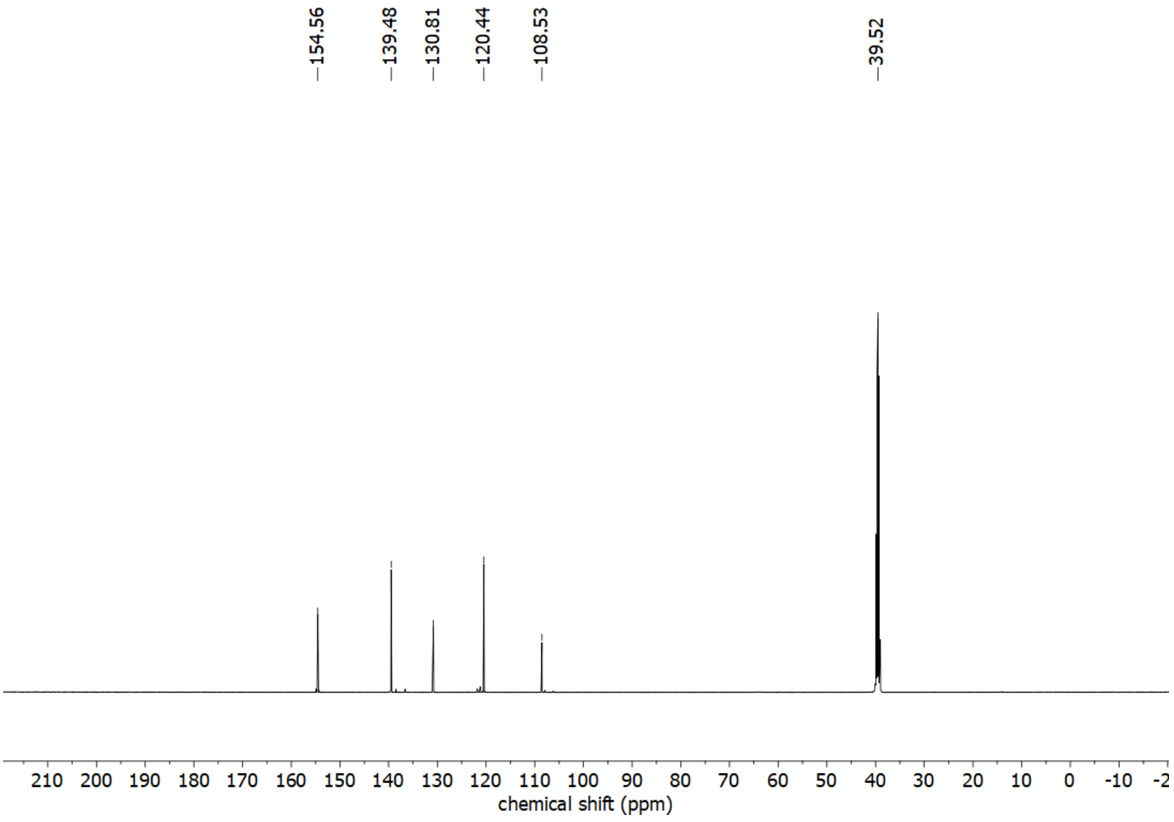

FT-IR spectrum (neat) of **2**

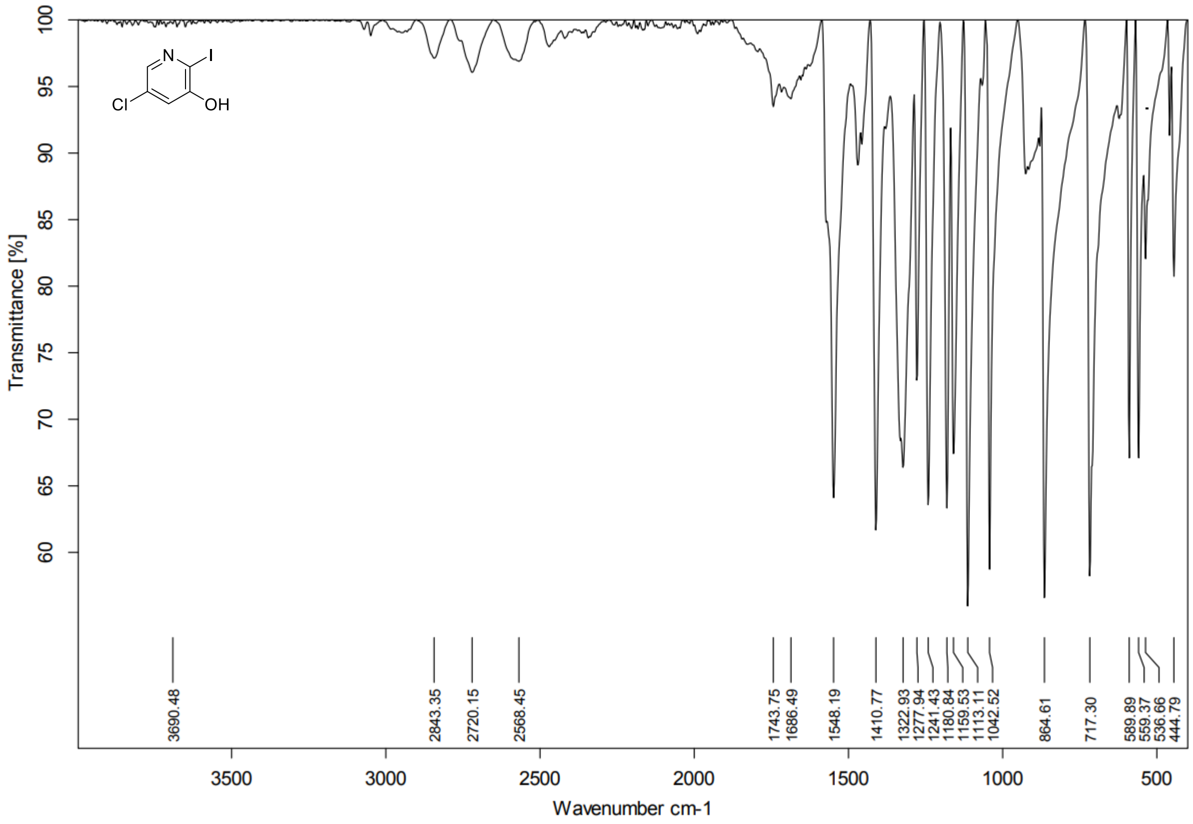

HRMS spectrum of **2**.

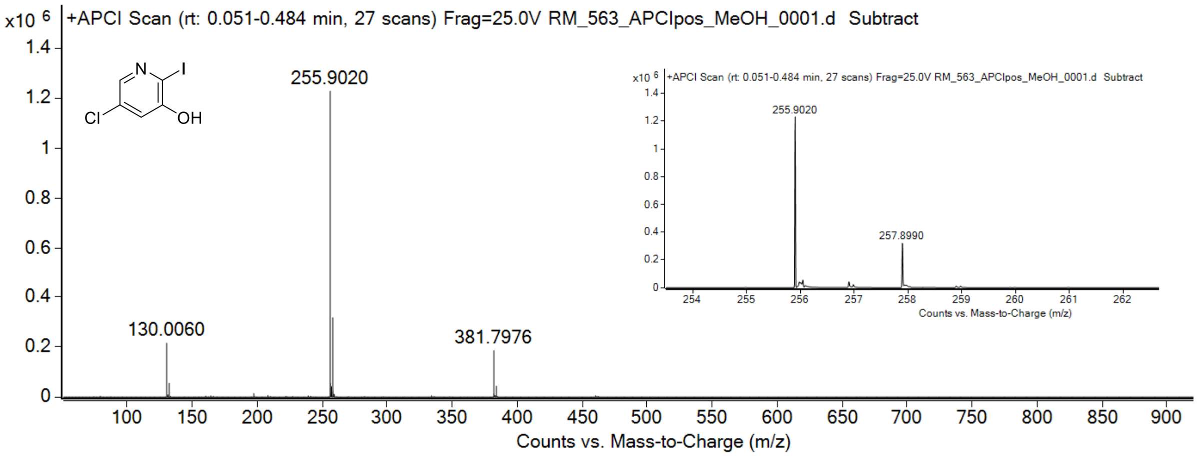

^{1}H (500 MHz) and ^{13}C NMR (126 MHz) spectra of **3** in chloroform-*d*.

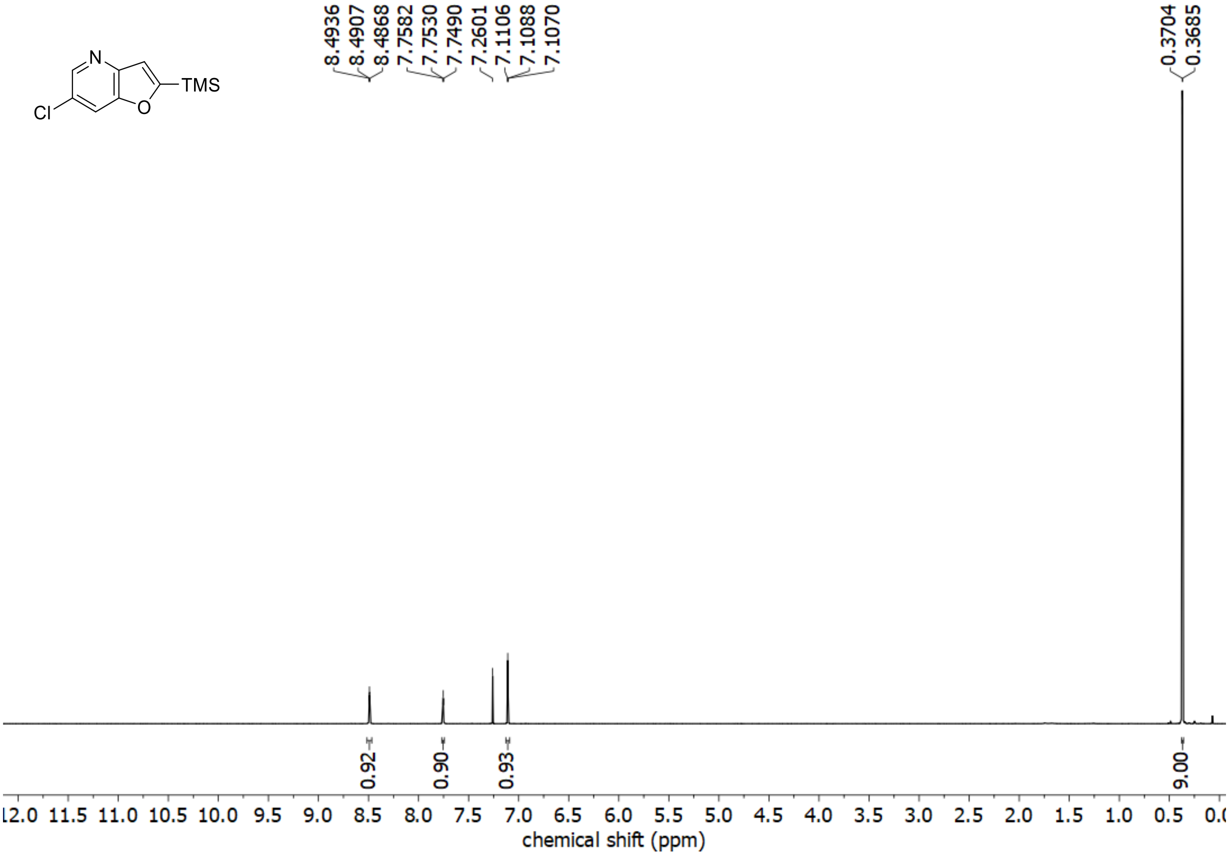

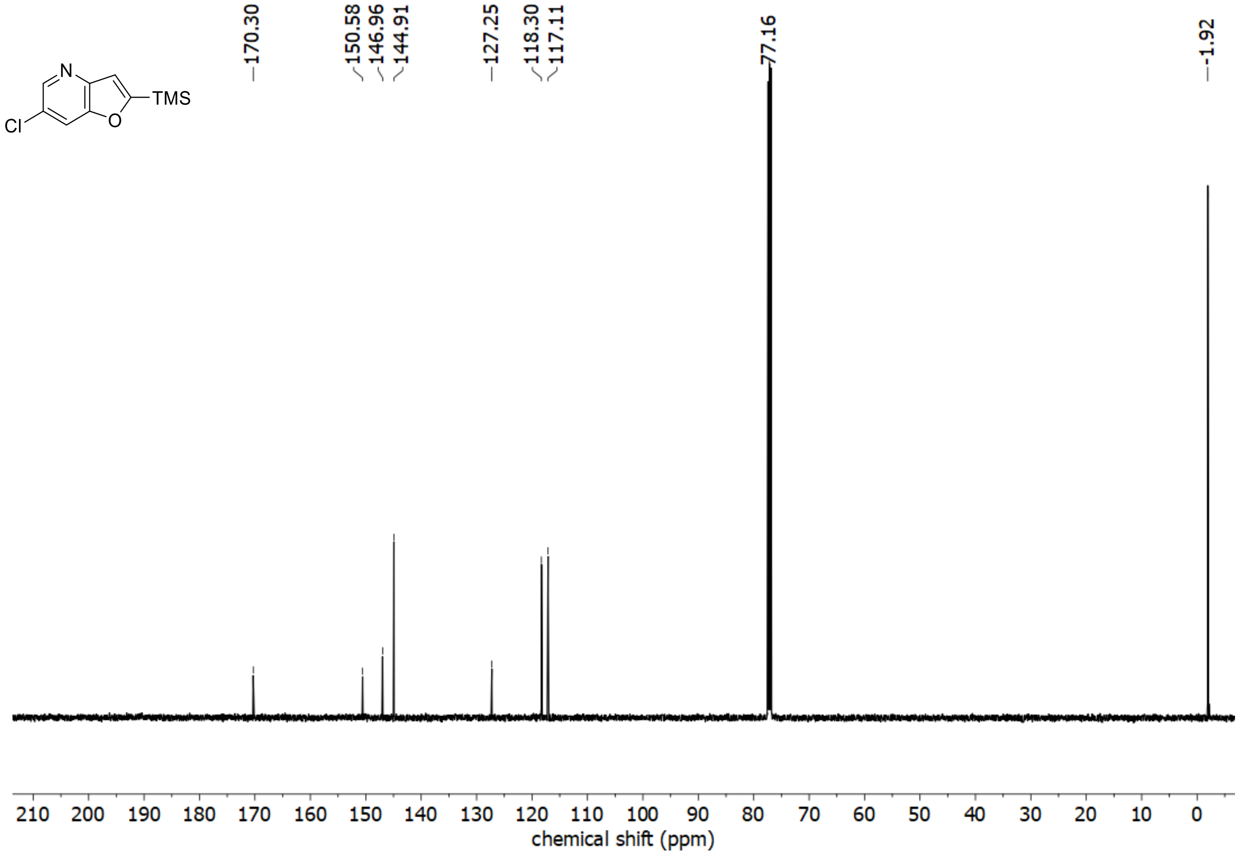

FT-IR spectrum (neat) of **3**.

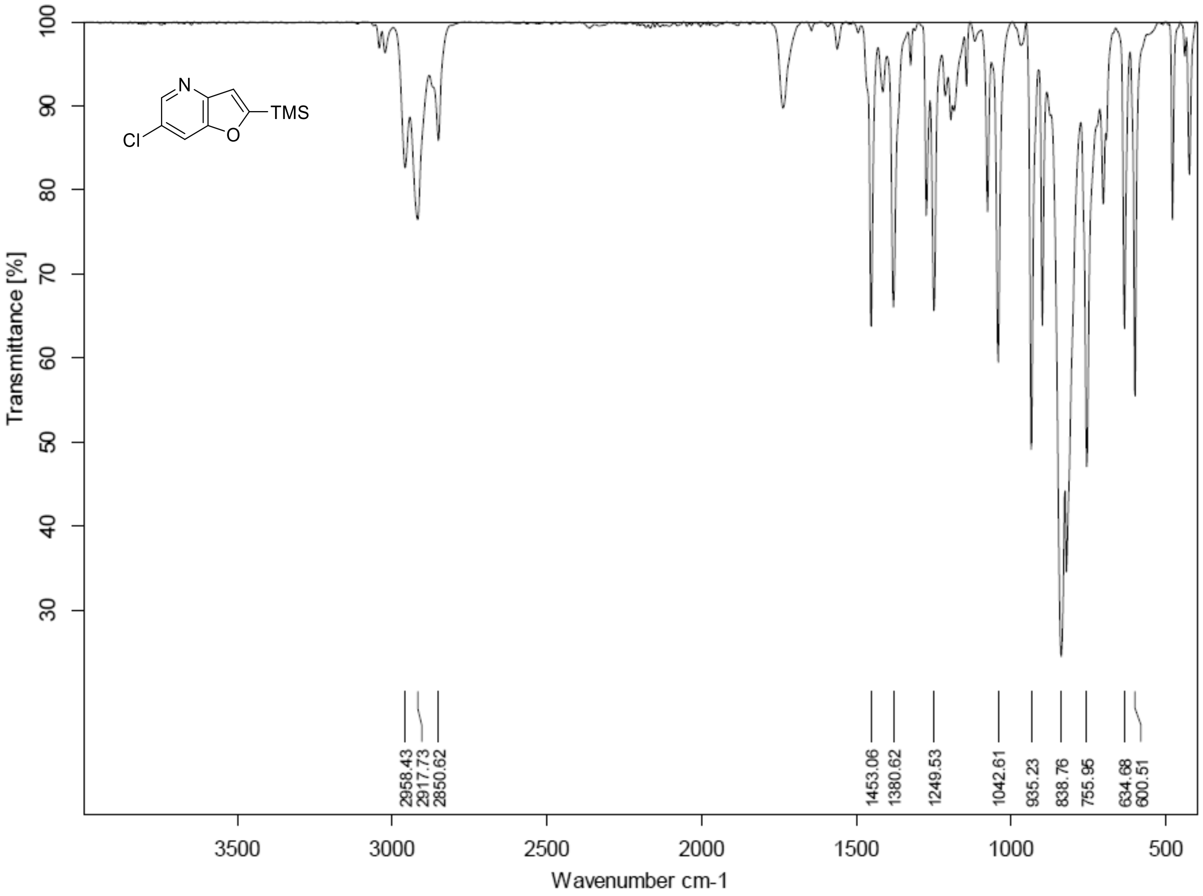

HRMS spectrum of **3**.

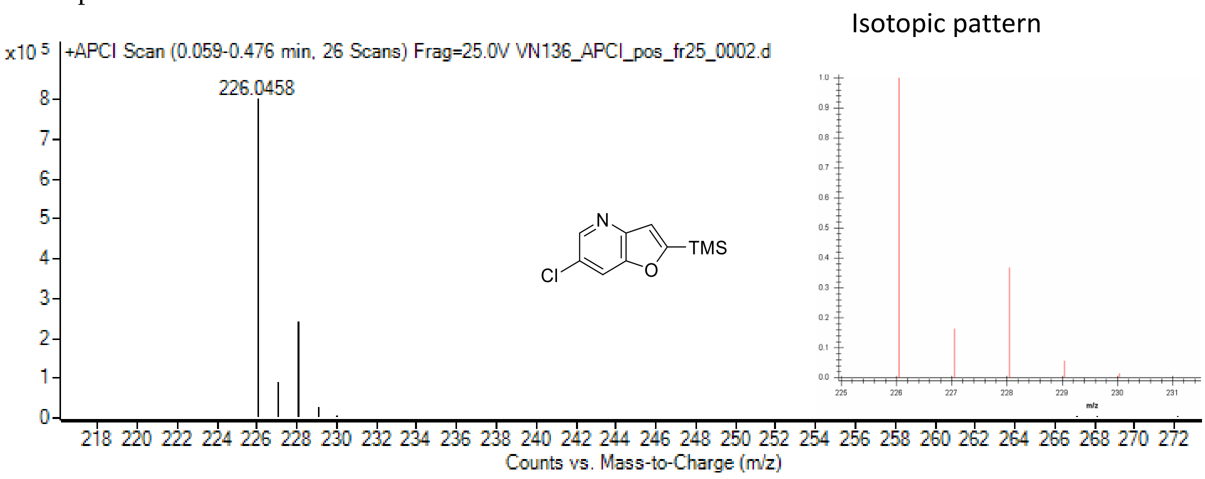

^{1}H (500 MHz) and ^{13}C NMR (126 MHz) spectra of **4** in chloroform-*d*.

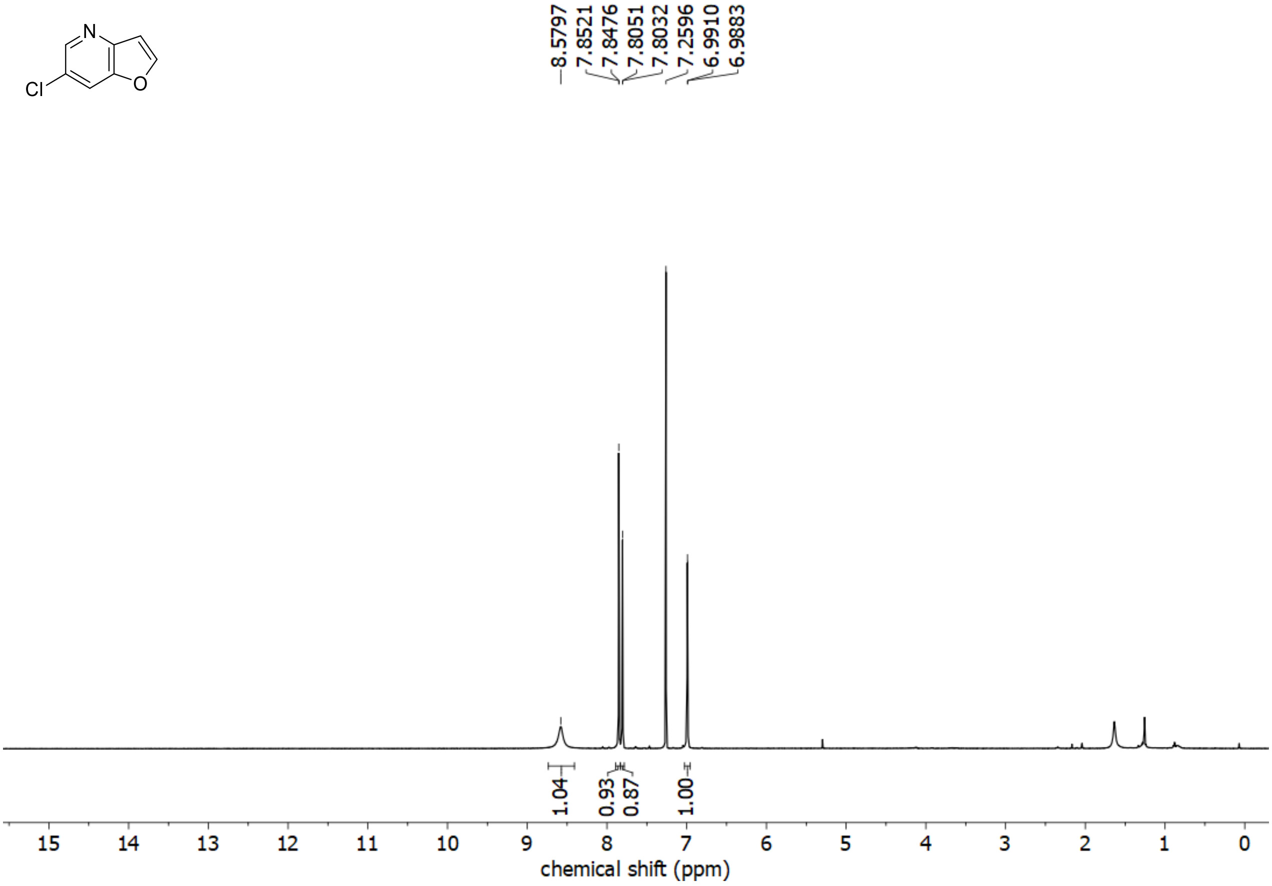

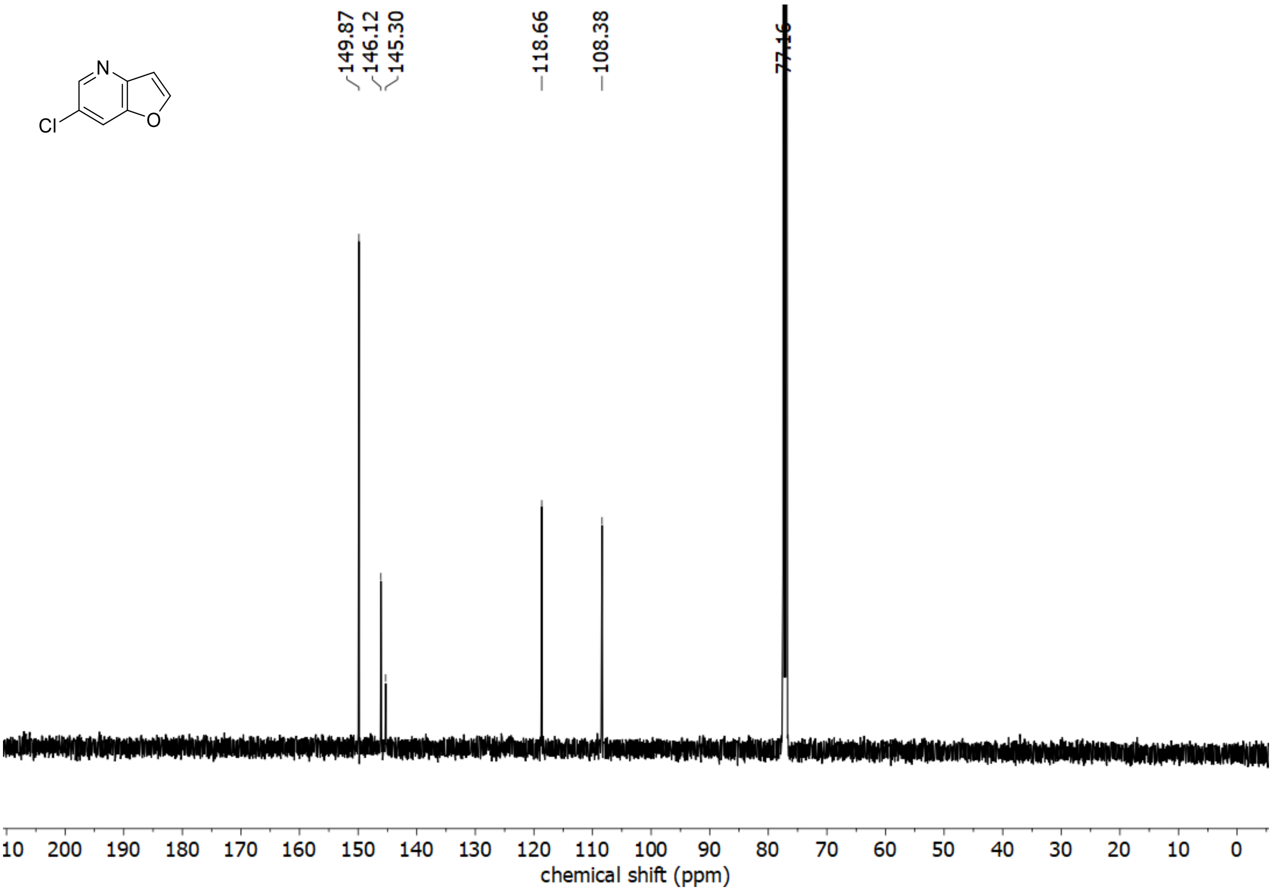

FT-IR spectrum (neat) of **4**.

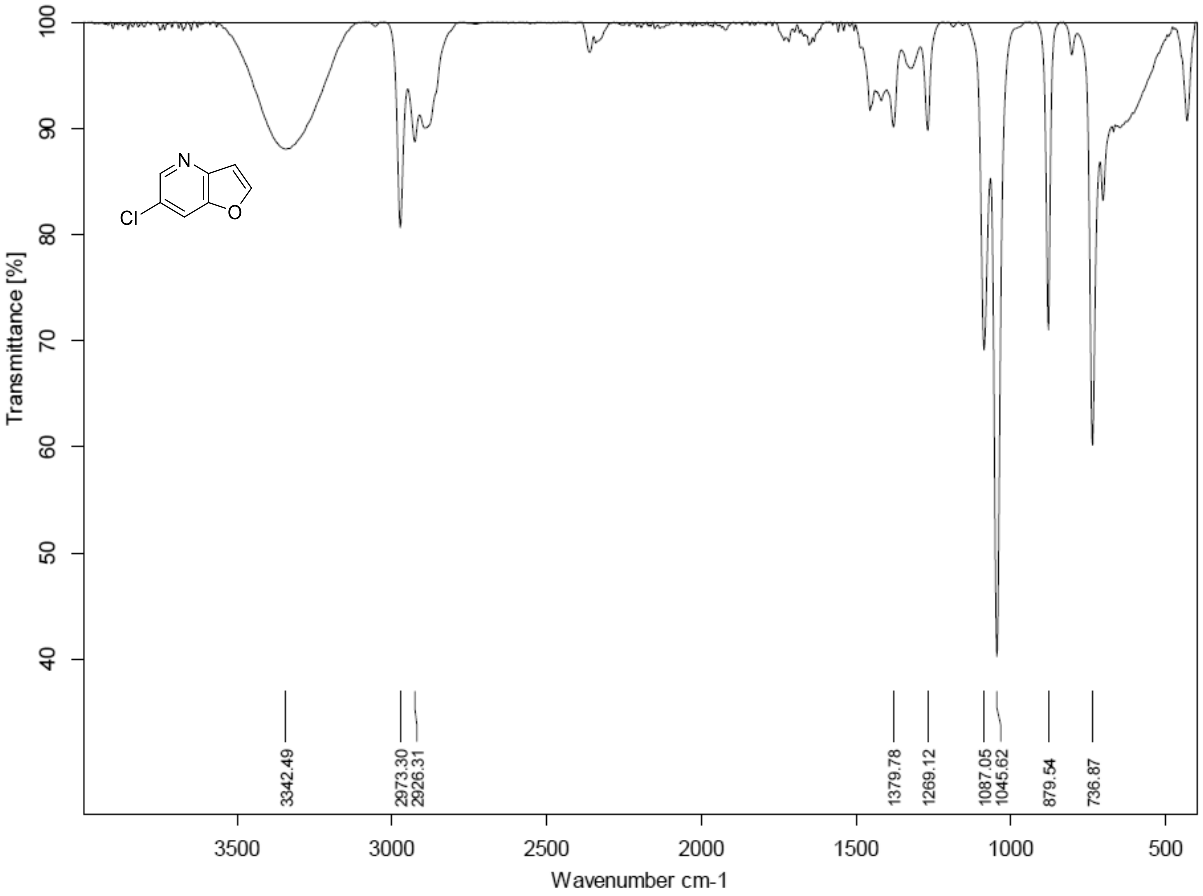

HRMS spectrum of **4**.

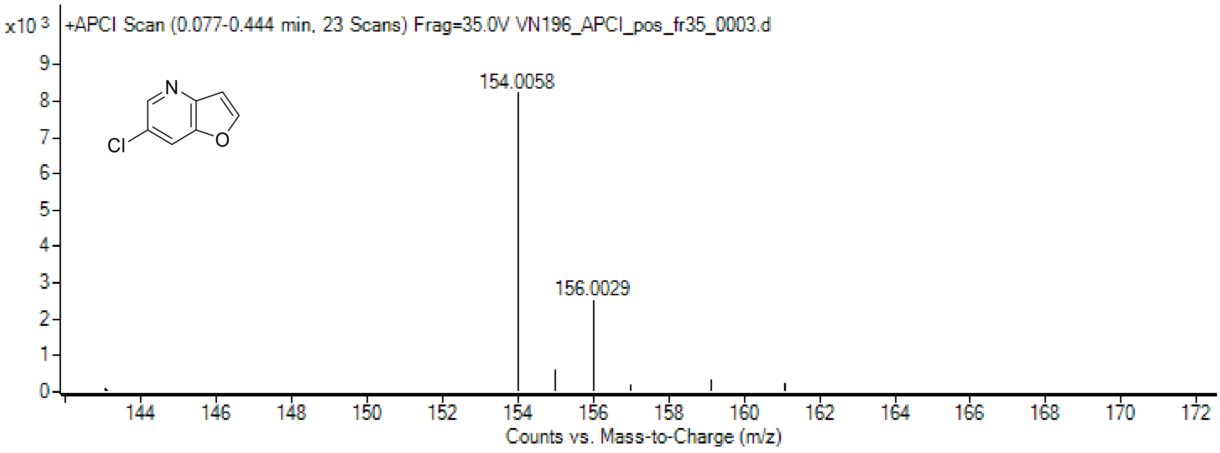

^{1}H (500 MHz) and ^{13}C NMR (126 MHz) spectra of **5** in chloroform-*d*.

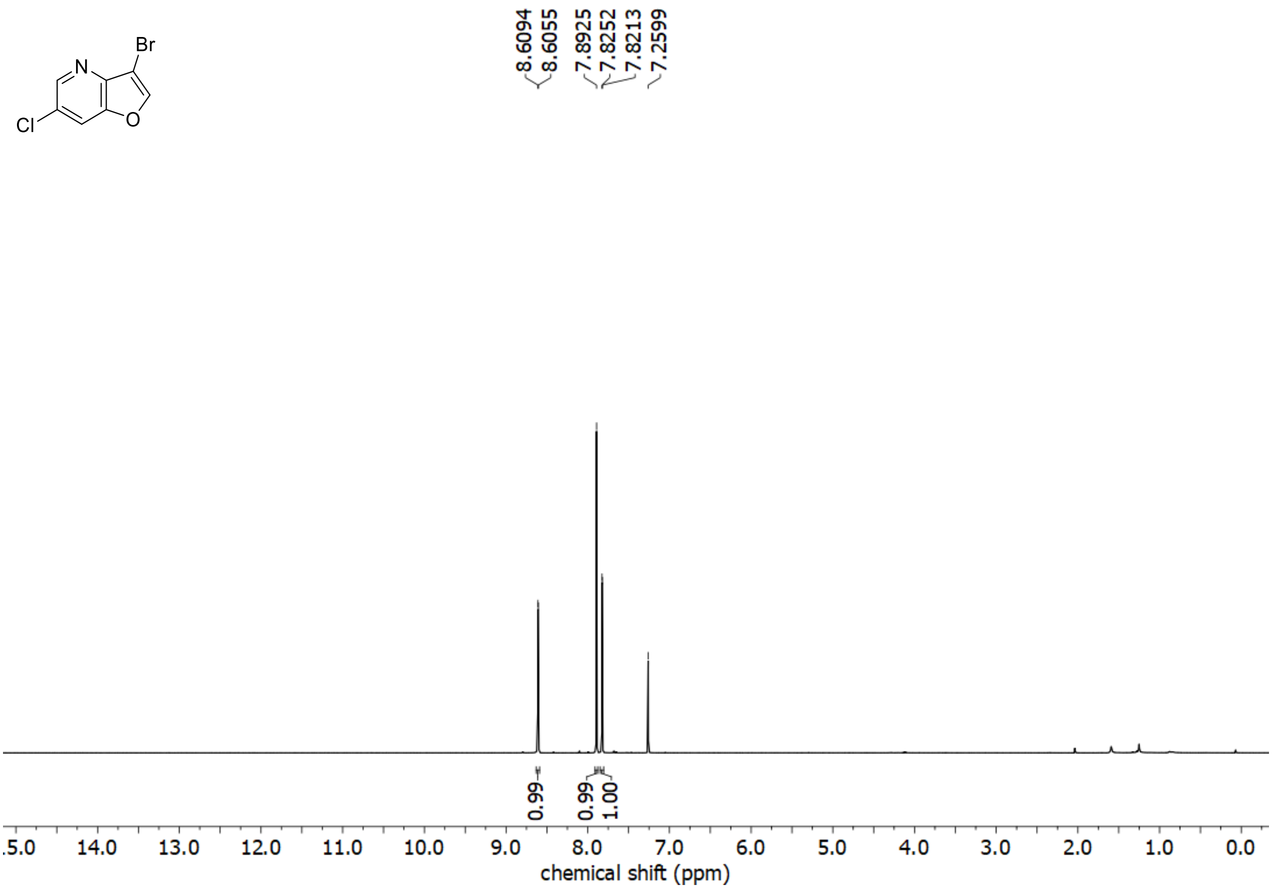

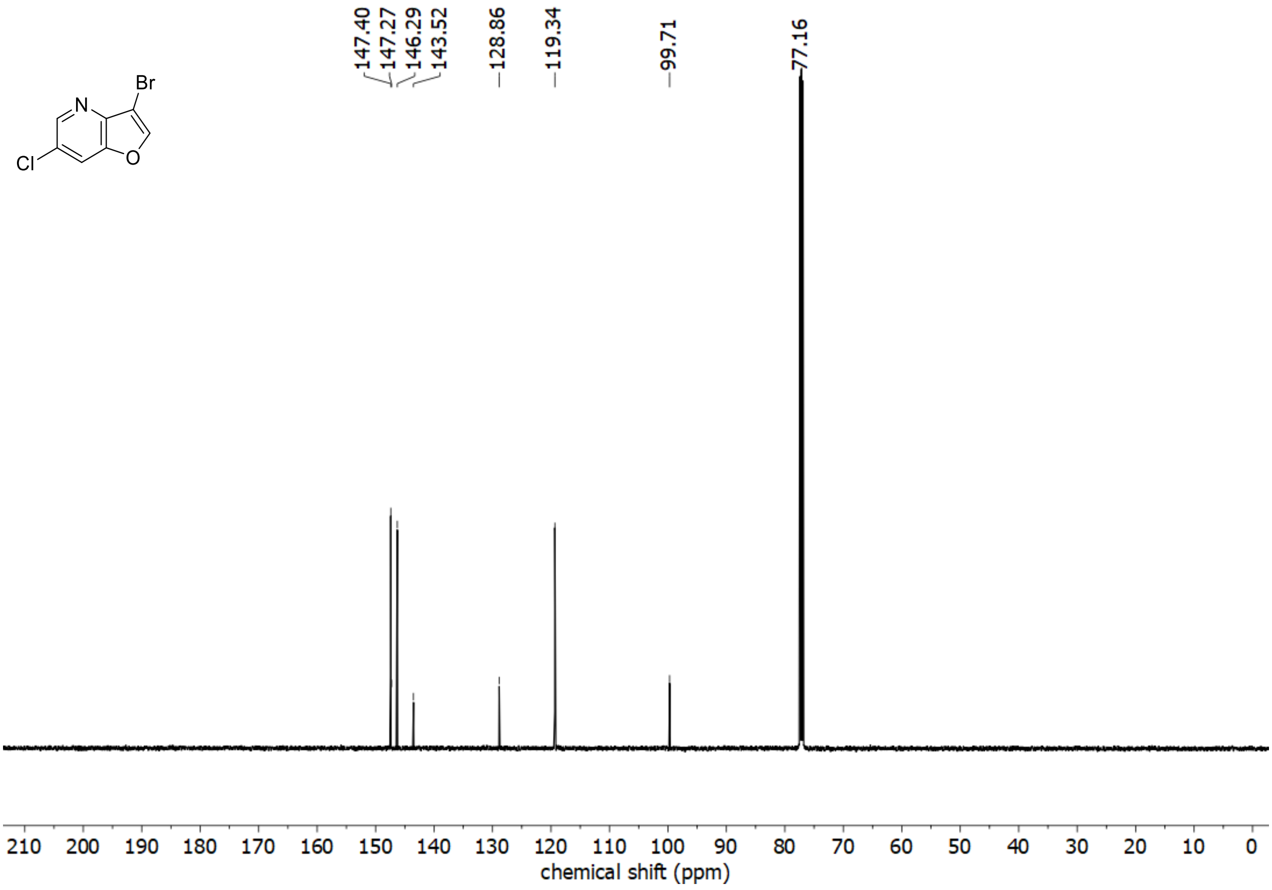

FT-IR spectrum (neat) of **5**.

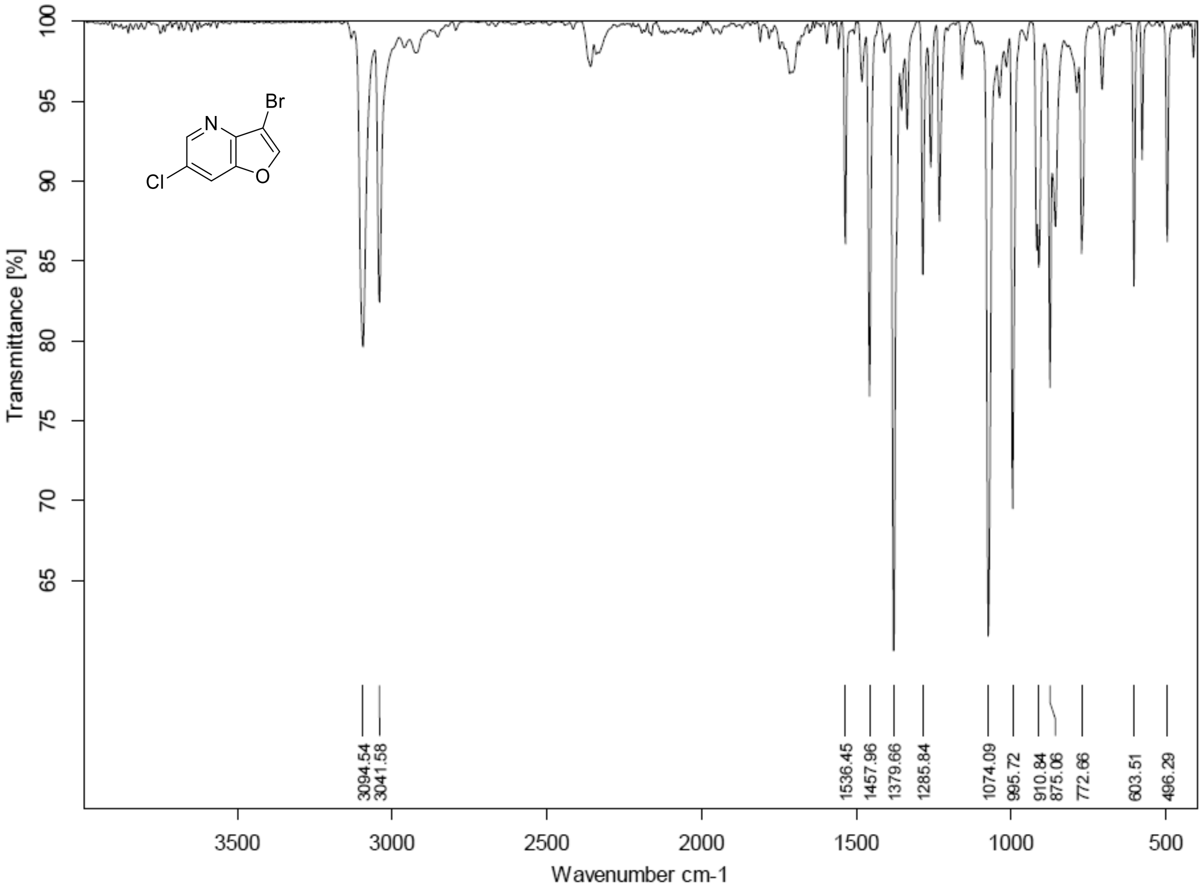

HRMS spectrum of **5**.

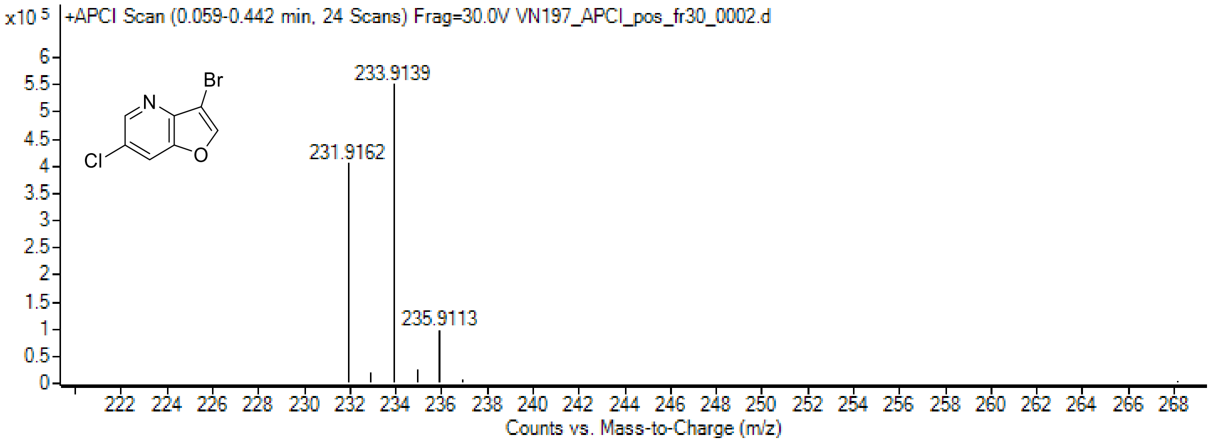

^{1}H (500 MHz) and ^{13}C NMR (126 MHz) spectra of **6** in methanol-d_4.

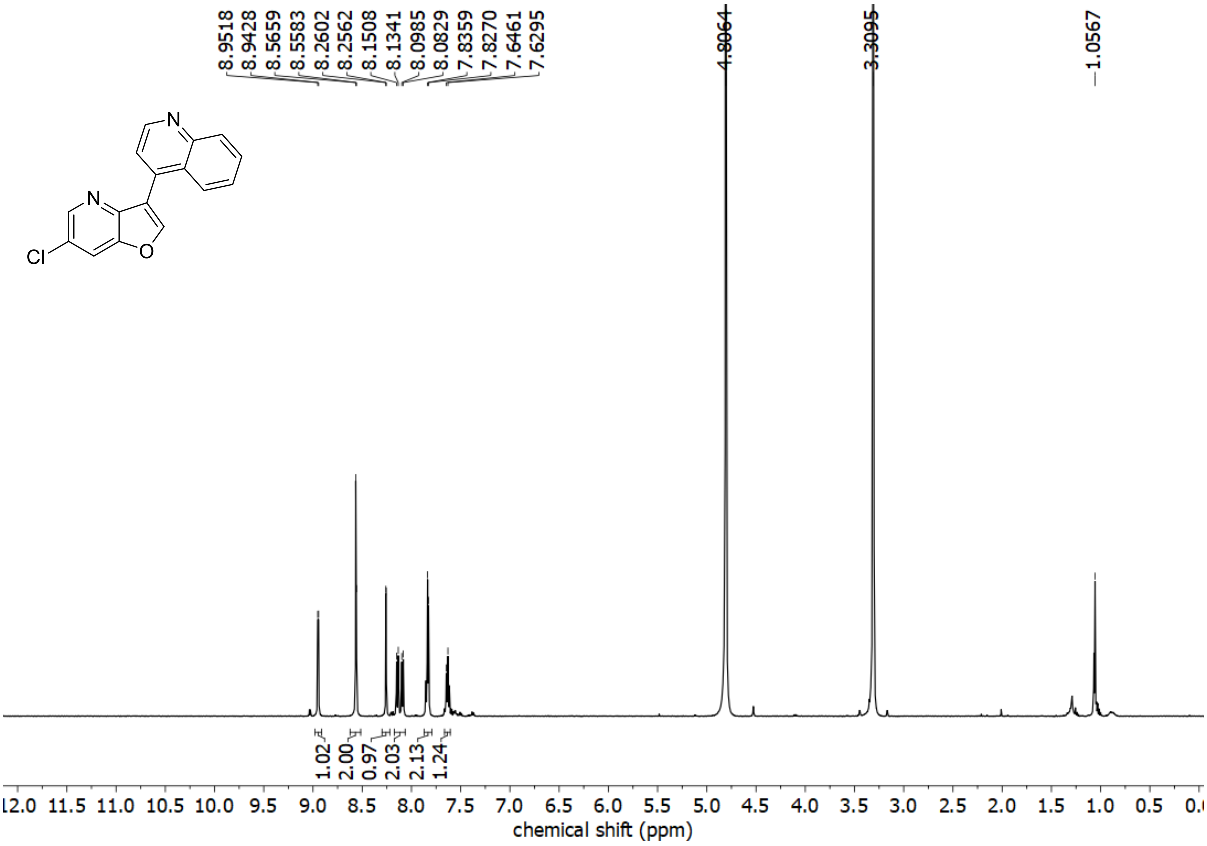

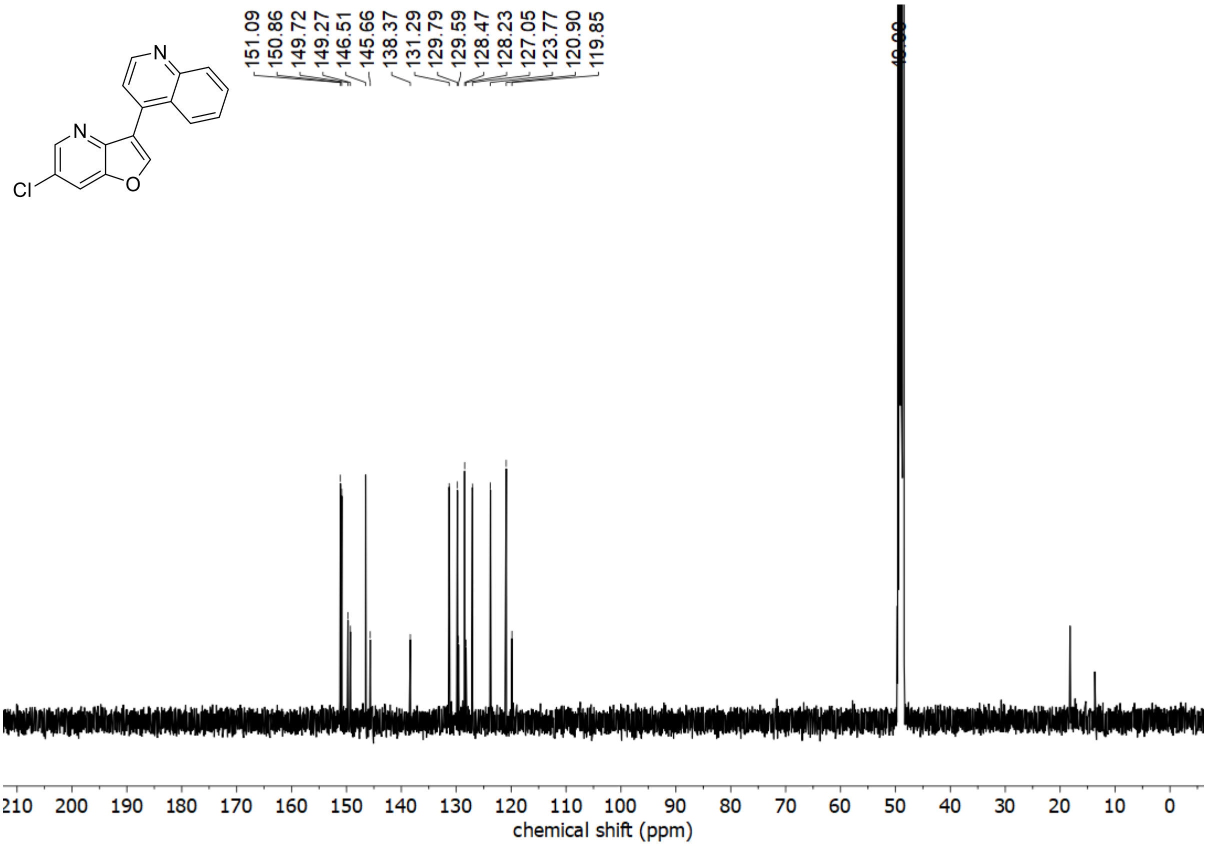

FT-IR spectrum (neat) of **6**.

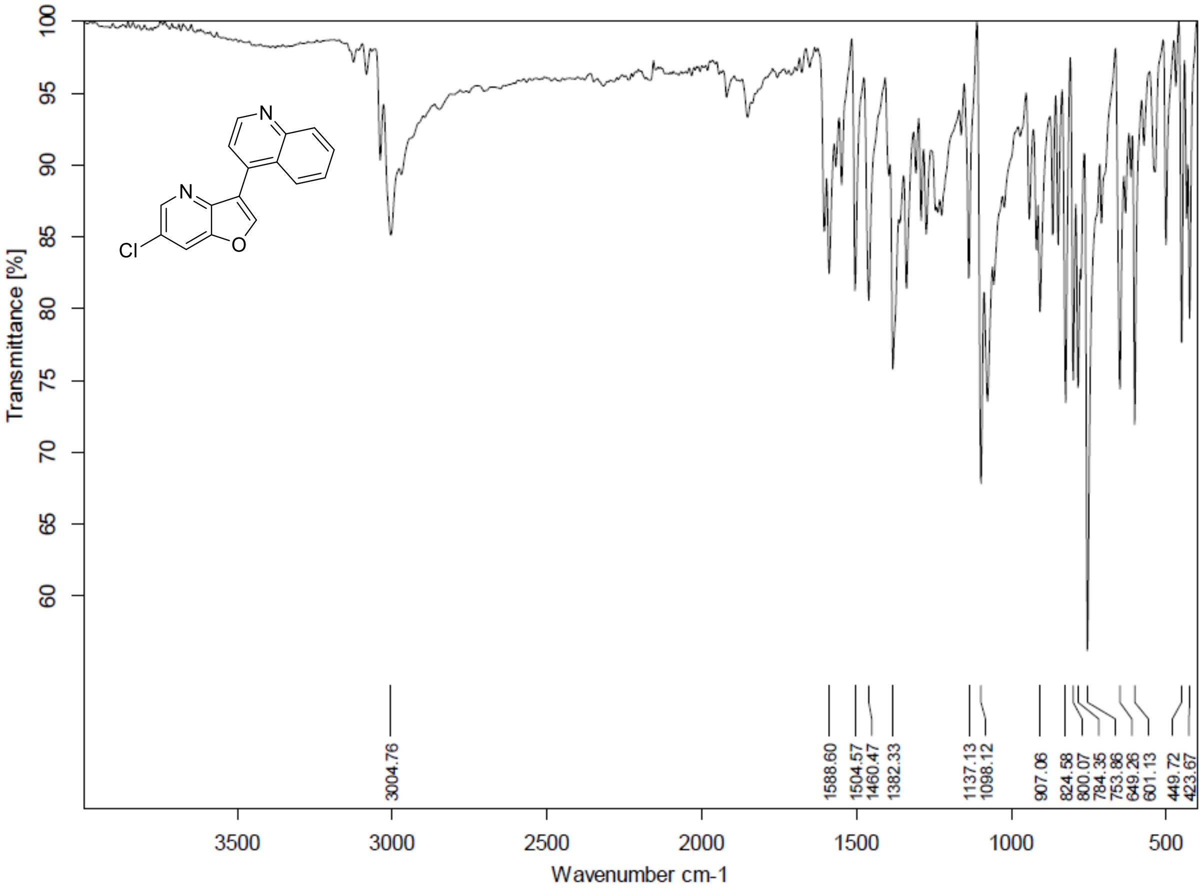

HRMS spectrum of **6**.

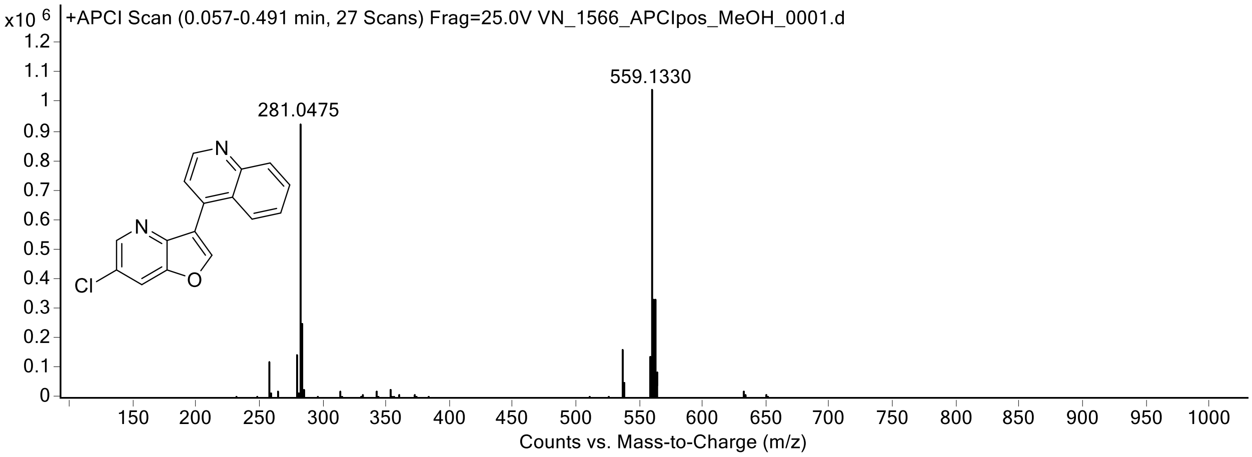

^{1}H (500 MHz) and ^{13}C NMR (126 MHz) spectra of **7** (**MU1700**) in DMSO-d_6.

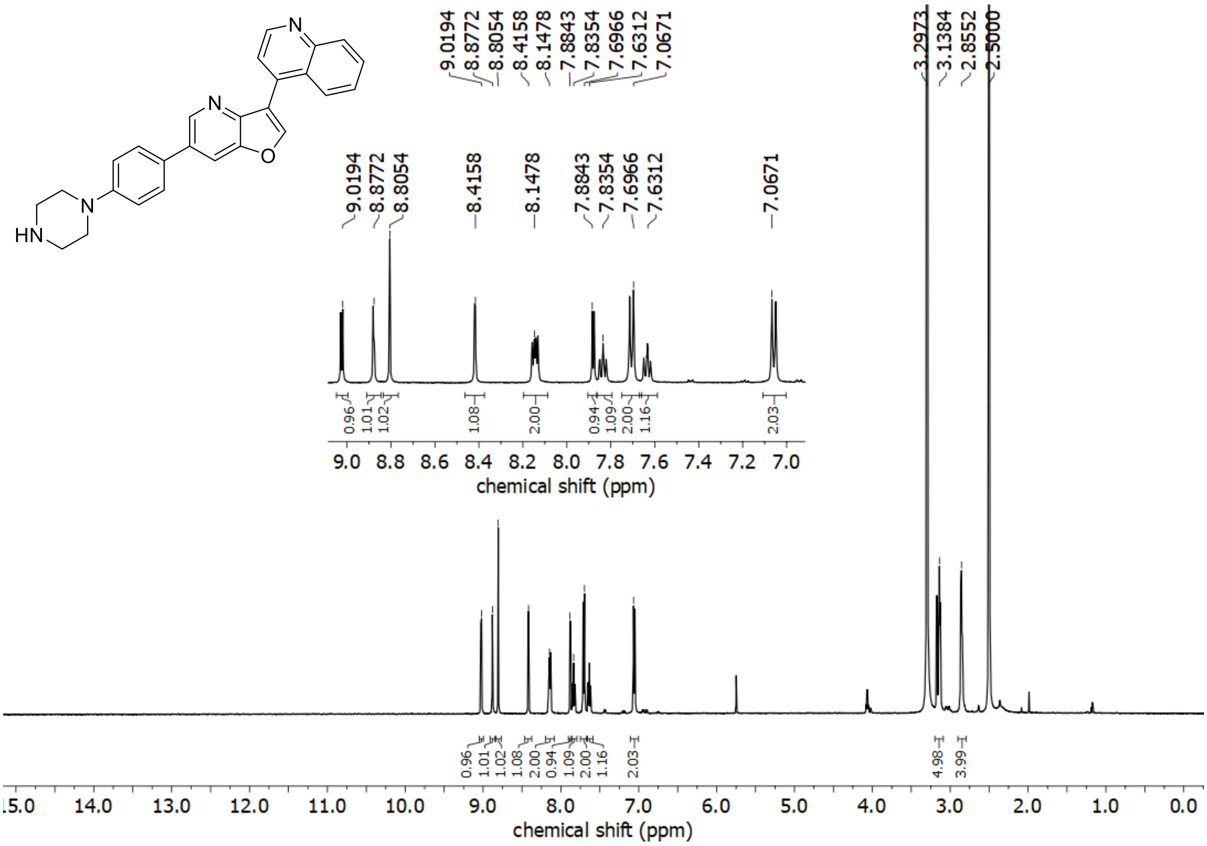

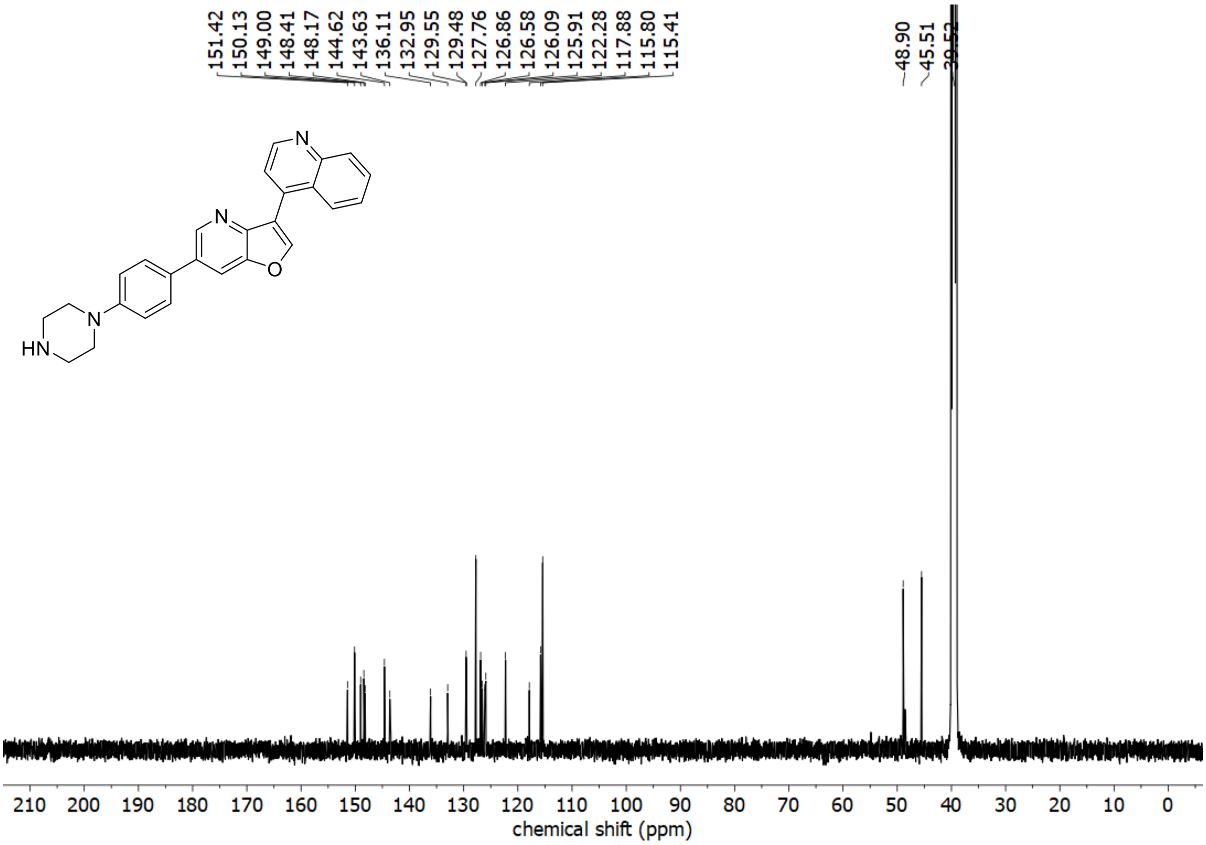

FT-IR spectrum (neat) of **7** (**MU1700**).

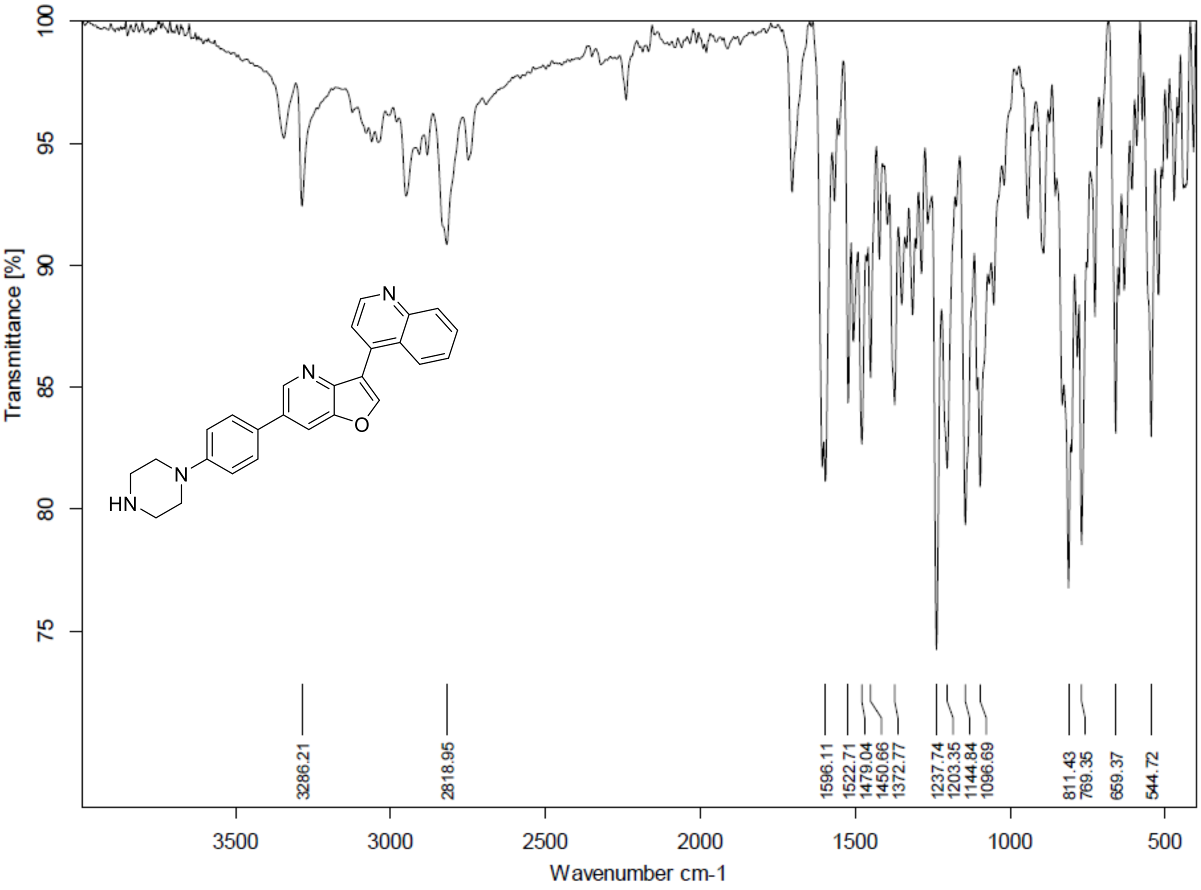

HRMS spectrum of **7** (**MU1700**).

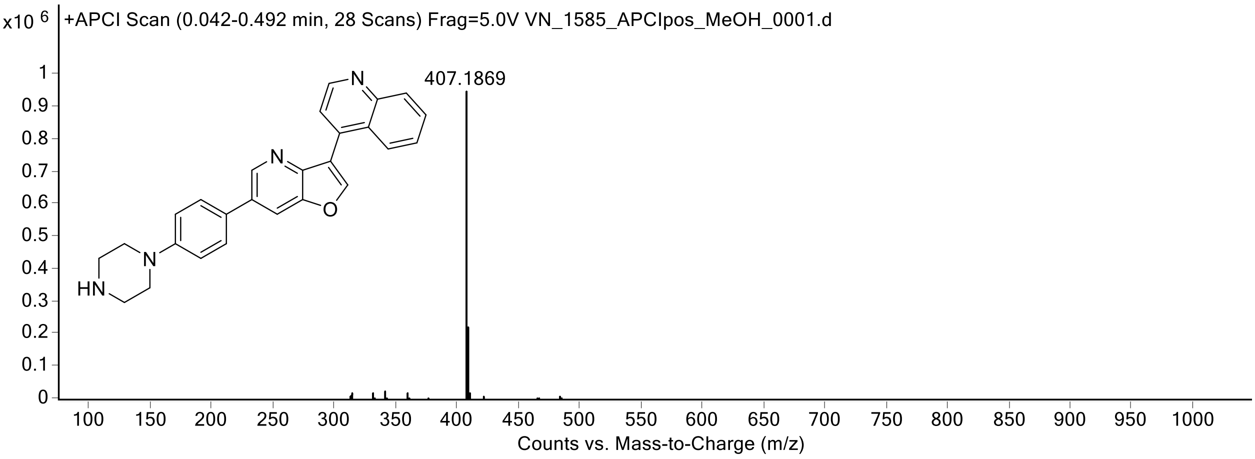

^{1}H (500 MHz) and ^{13}C NMR (126 MHz) spectra of **8** in chloroform-*d*.

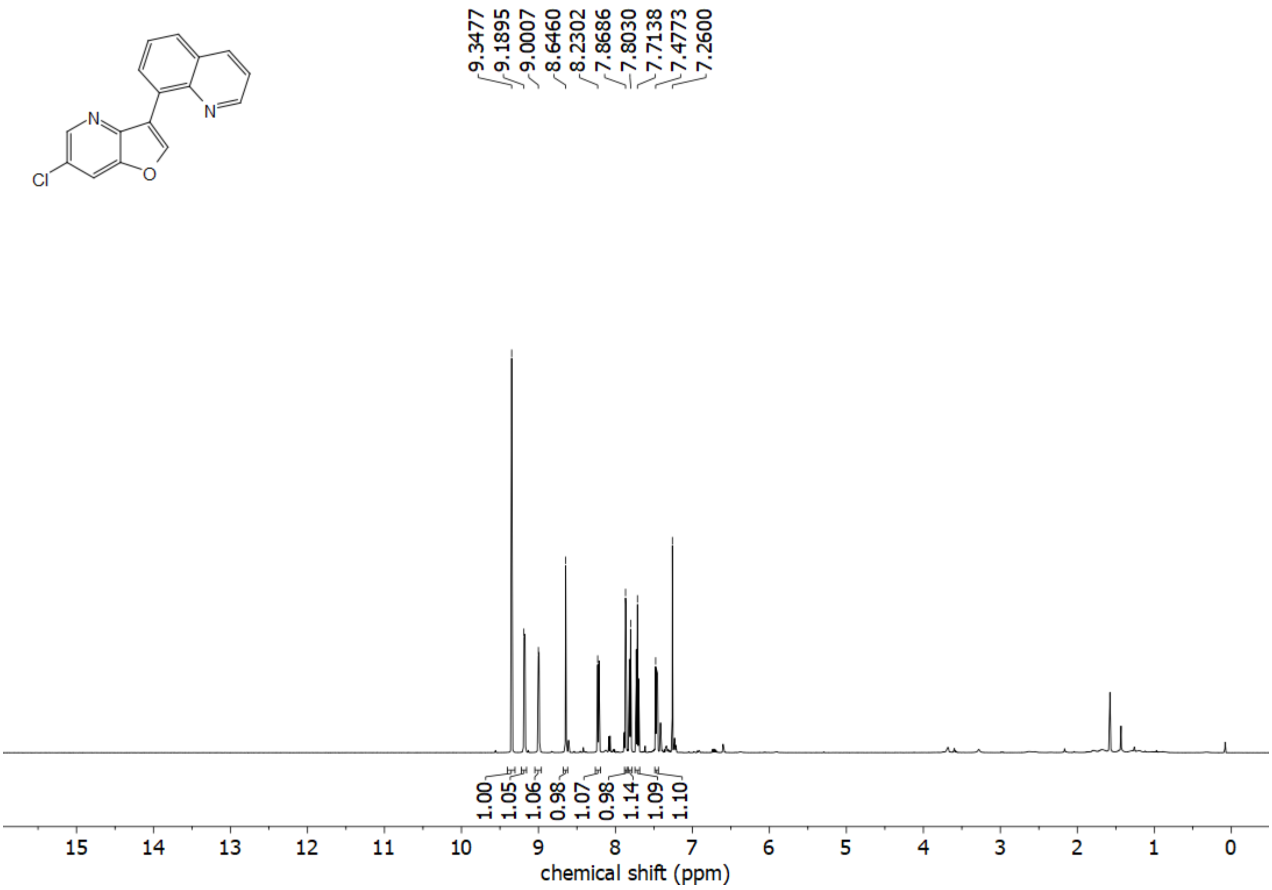

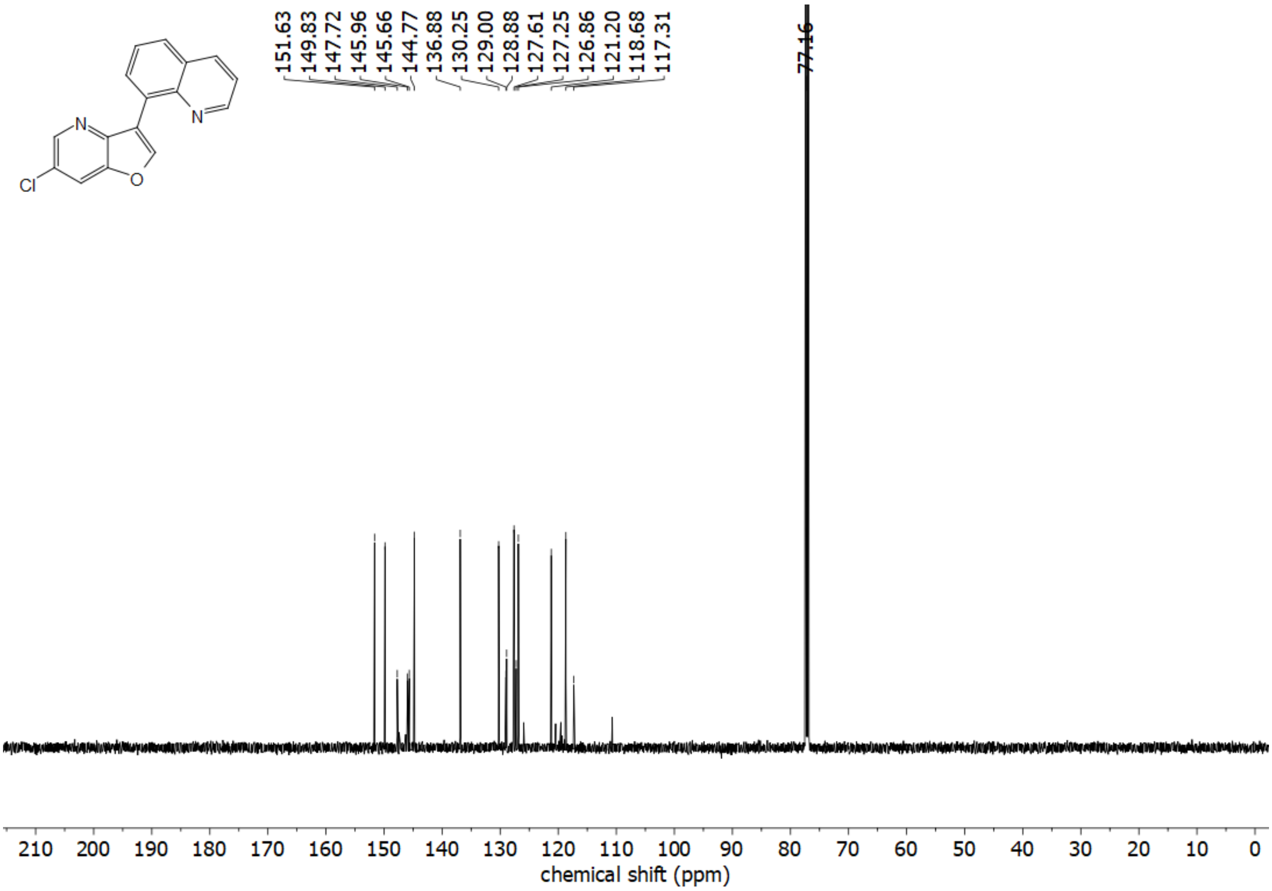

FT-IR spectrum (neat) of **8**.

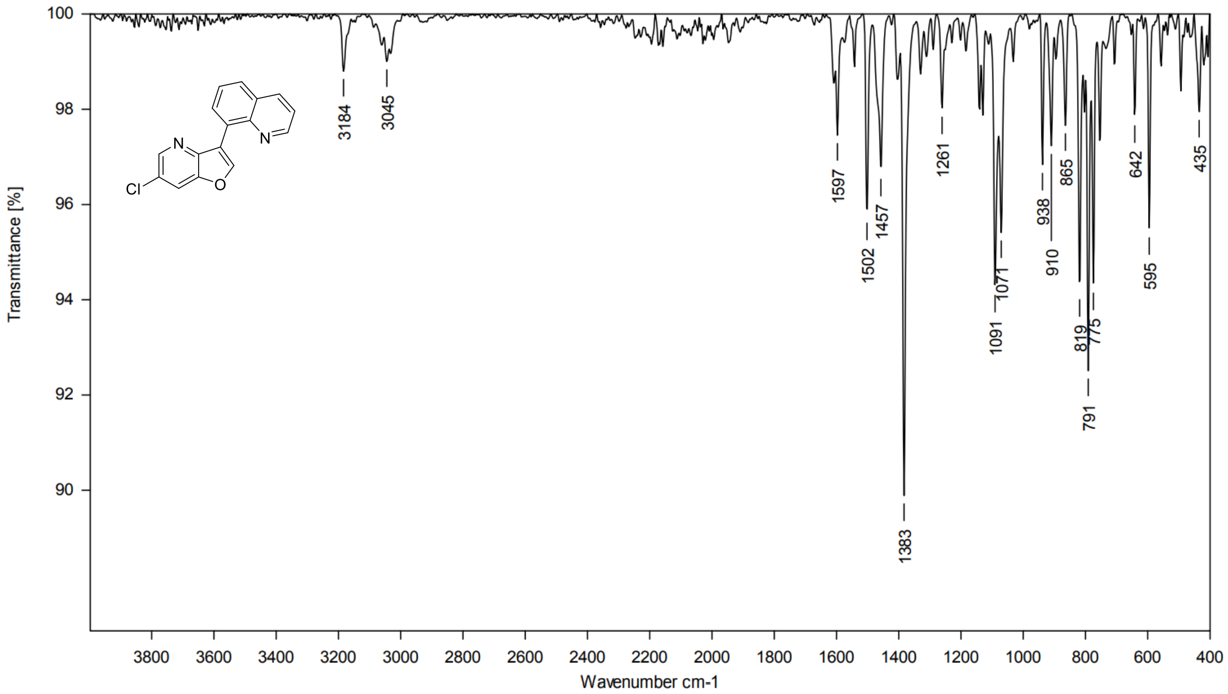

HRMS spectrum of **8**.

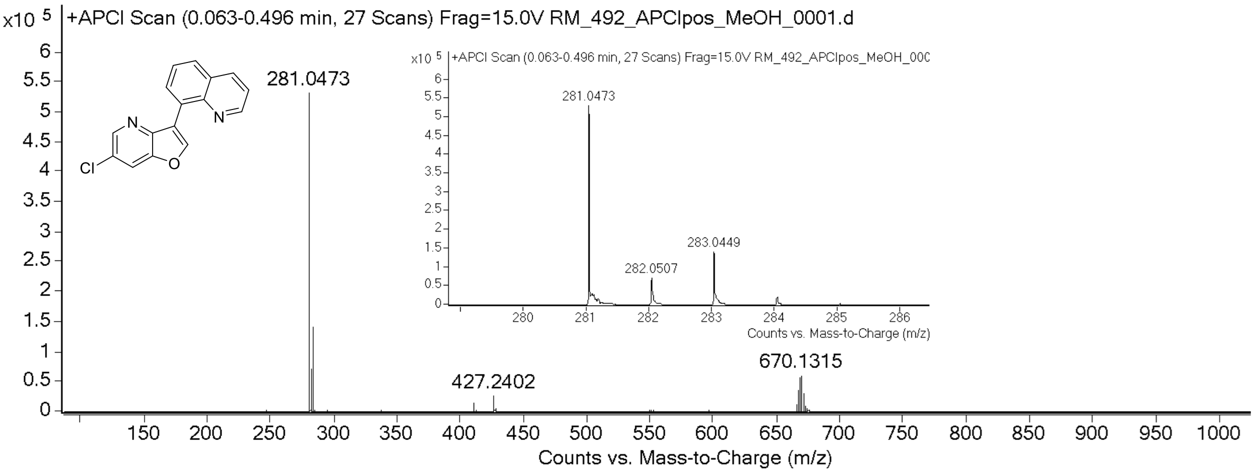

^{1}H (500 MHz) and ^{13}C NMR (126 MHz) spectra of **9** in chloroform-*d*.

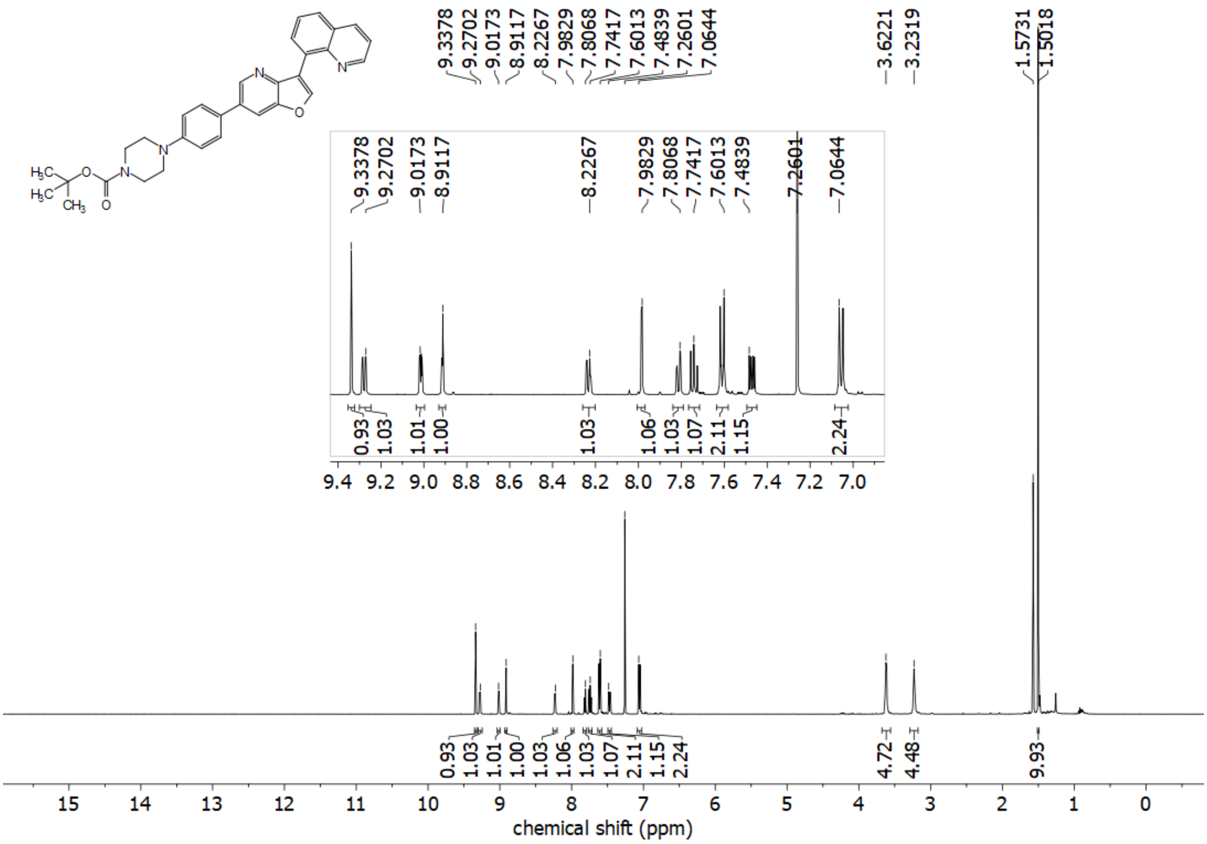

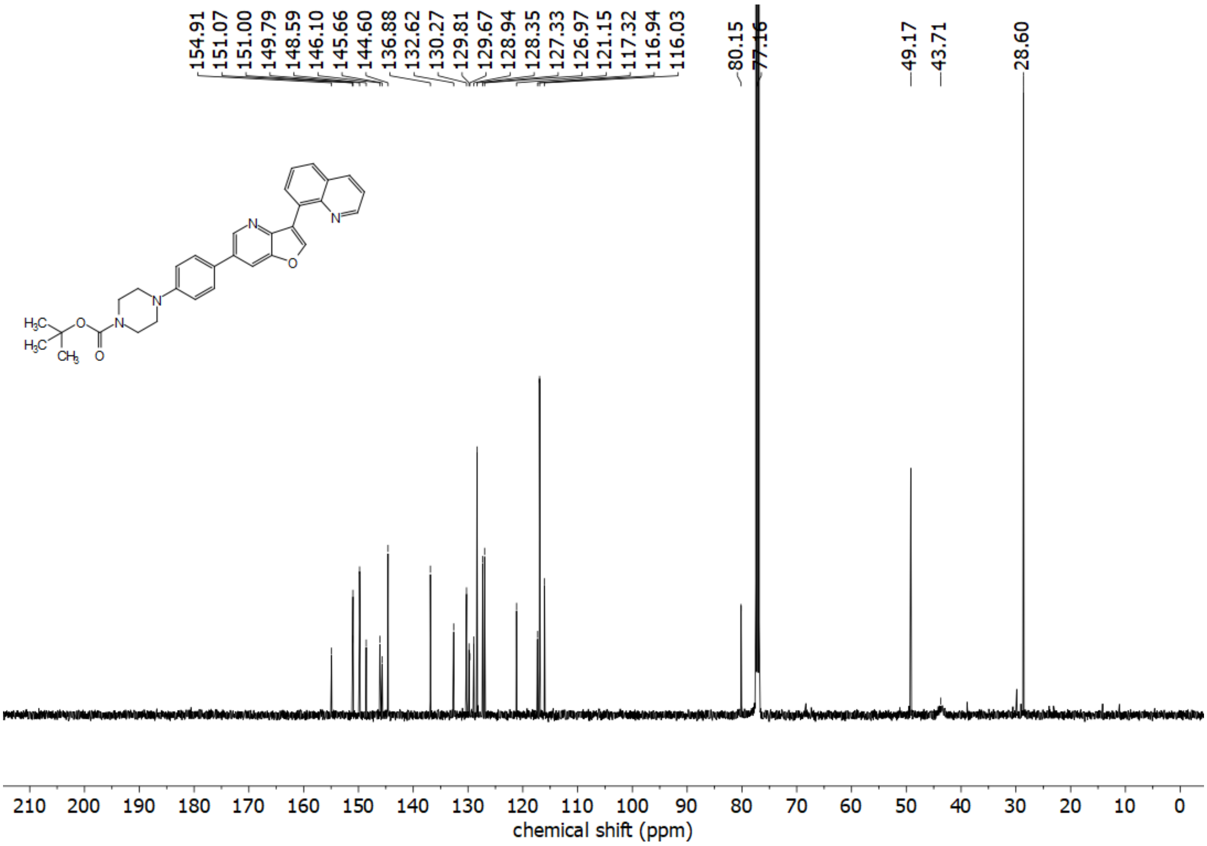

FT-IR spectrum (neat) of **9**.

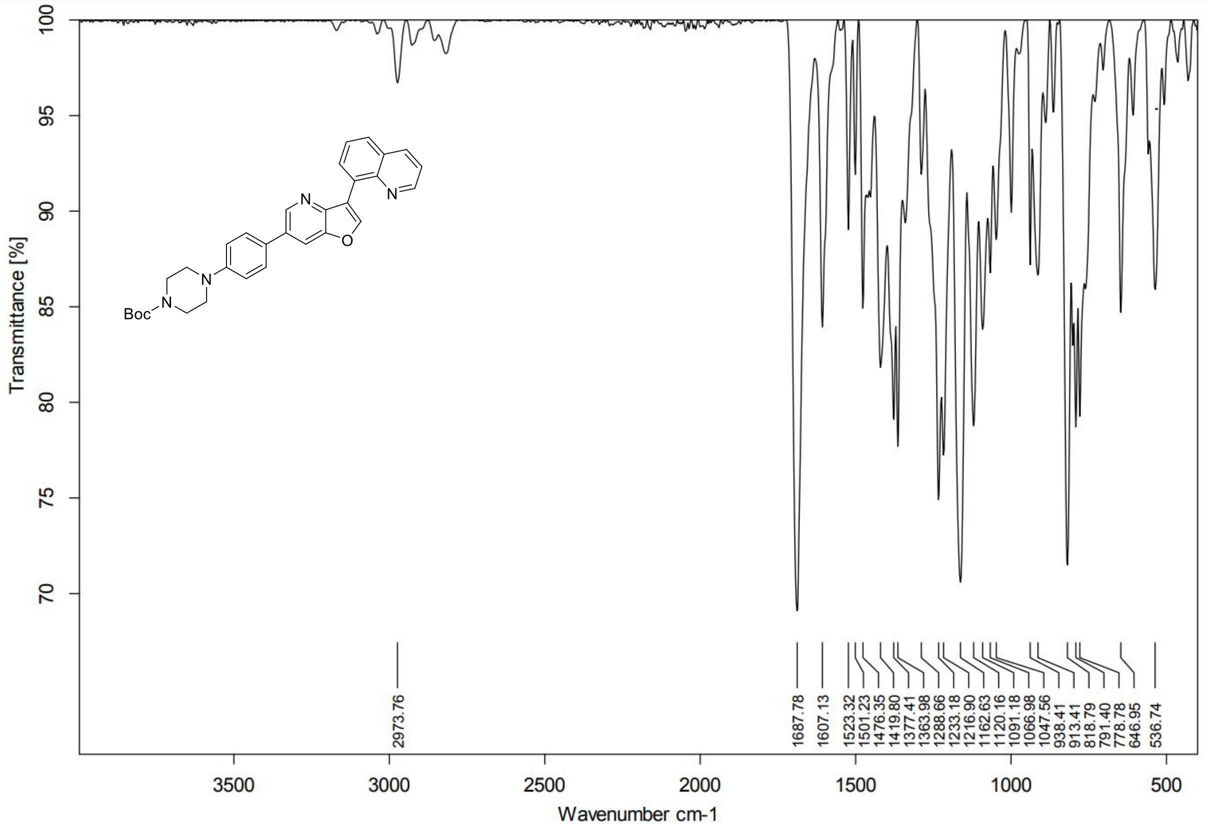

HRMS spectrum of **9**.

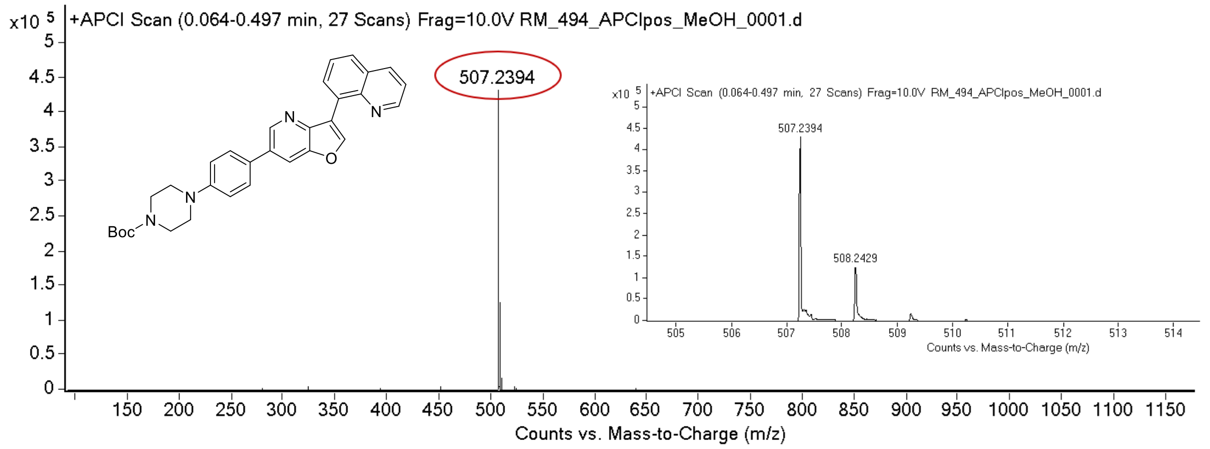

^{1}H (500 MHz) and ^{13}C NMR (126 MHz) spectra of **10** (**MU1700NC**) in chloroform-*d*.

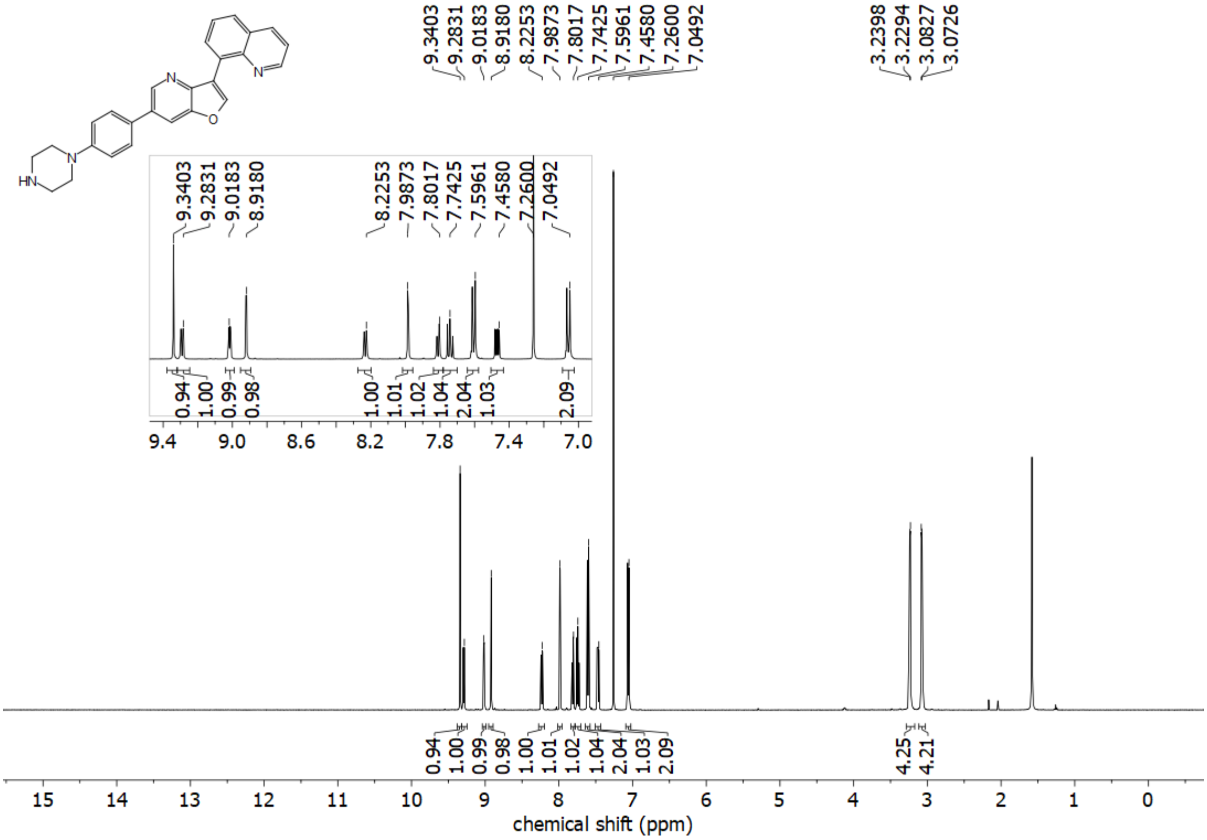

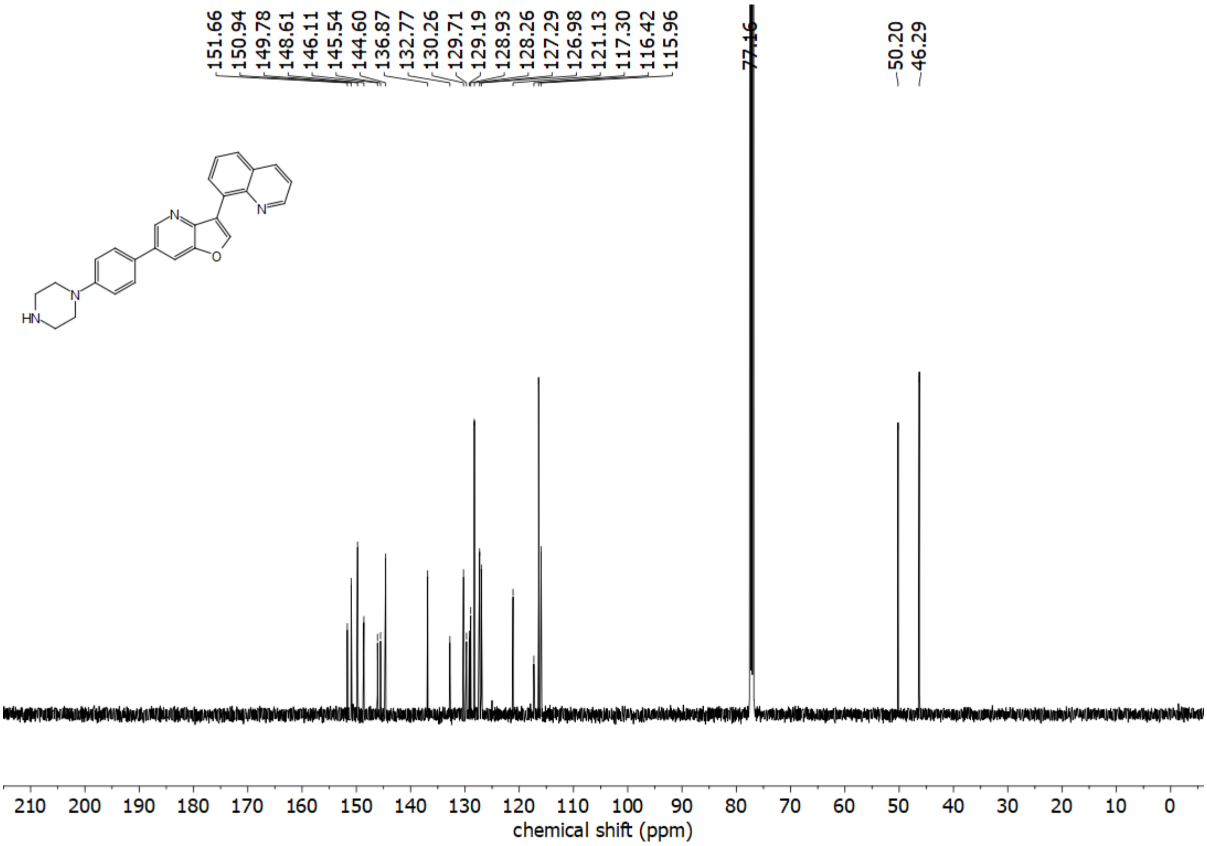

FT-IR spectrum (neat) of **10** (**MU1700NC**).

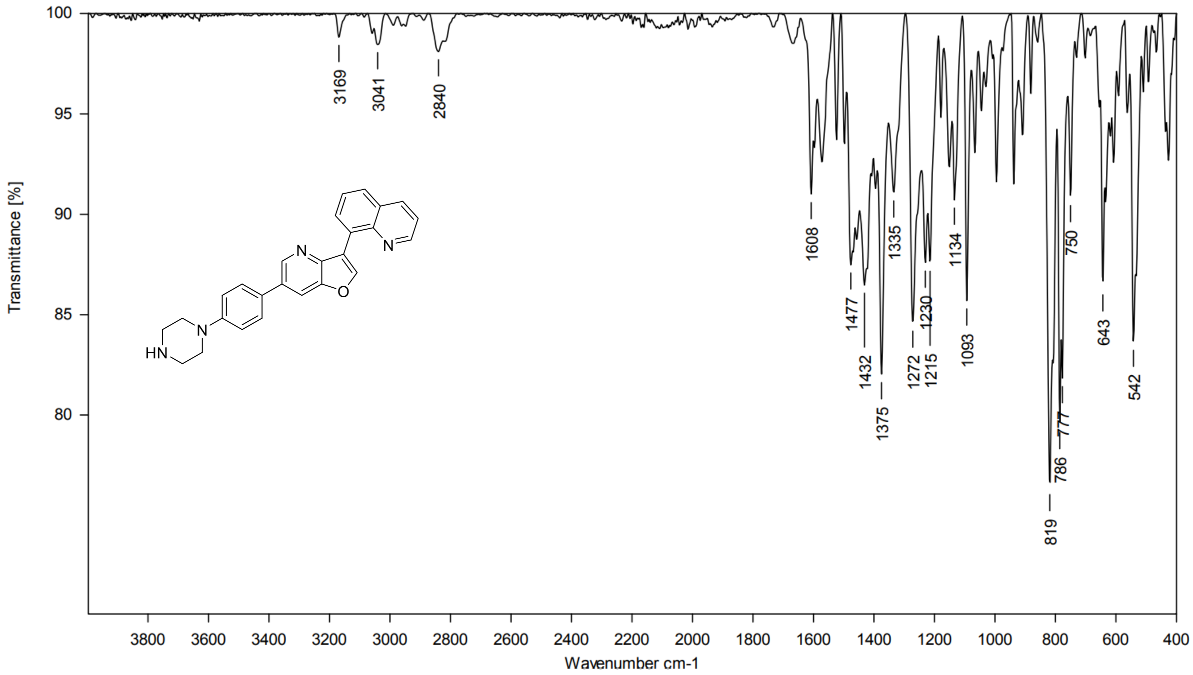

HRMS spectrum of **10** (**MU1700NC**).

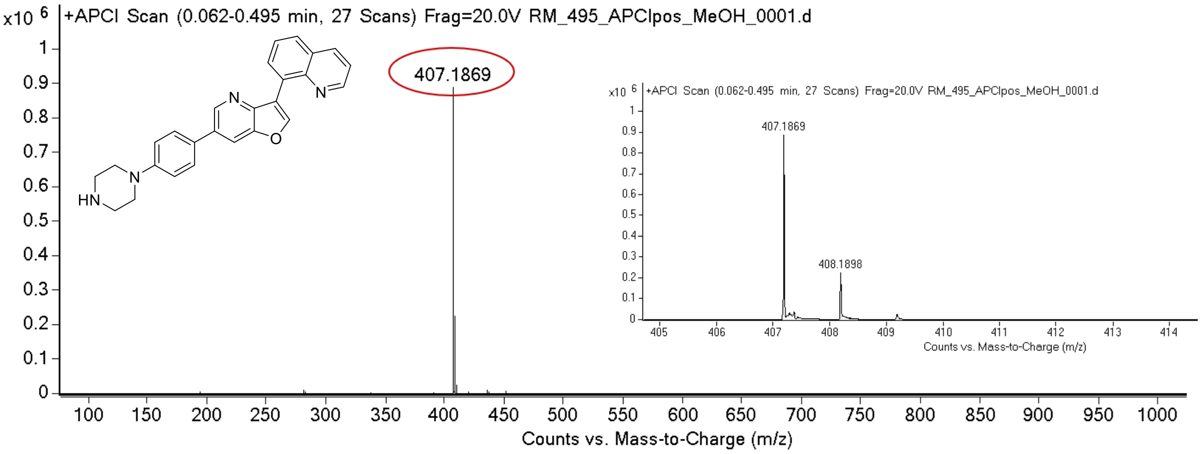

^{1}H (500 MHz) and ^{13}C NMR (126 MHz) spectra of **11** in chloroform-*d*.

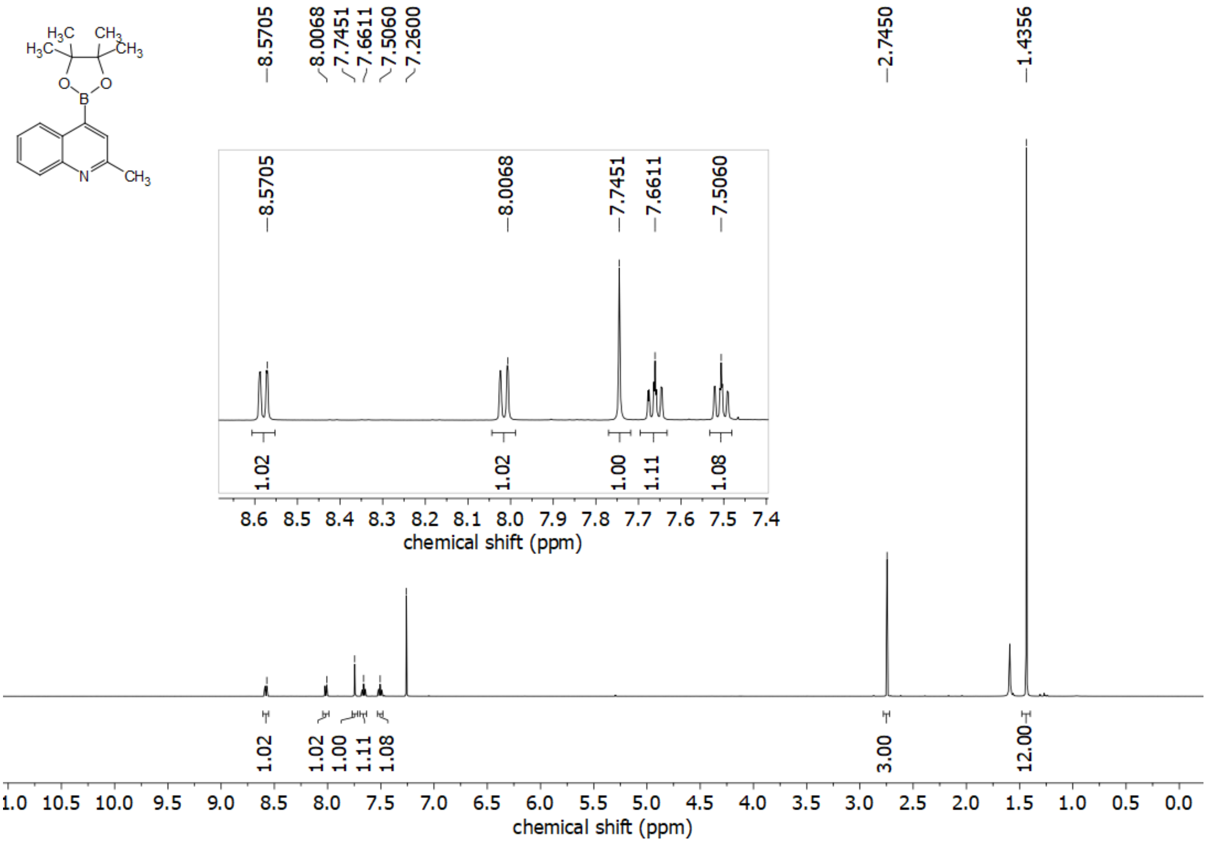

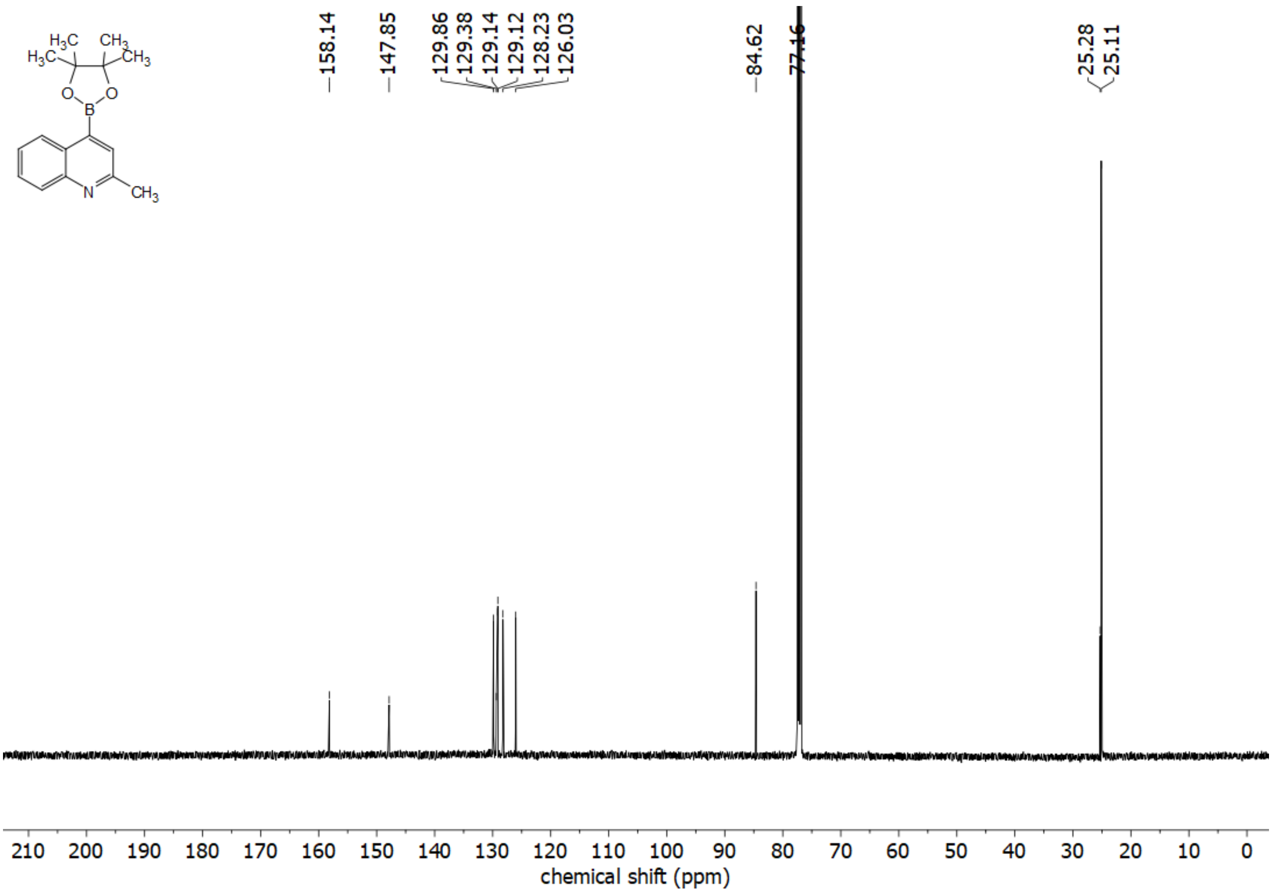

HRMS spectrum of **11**.

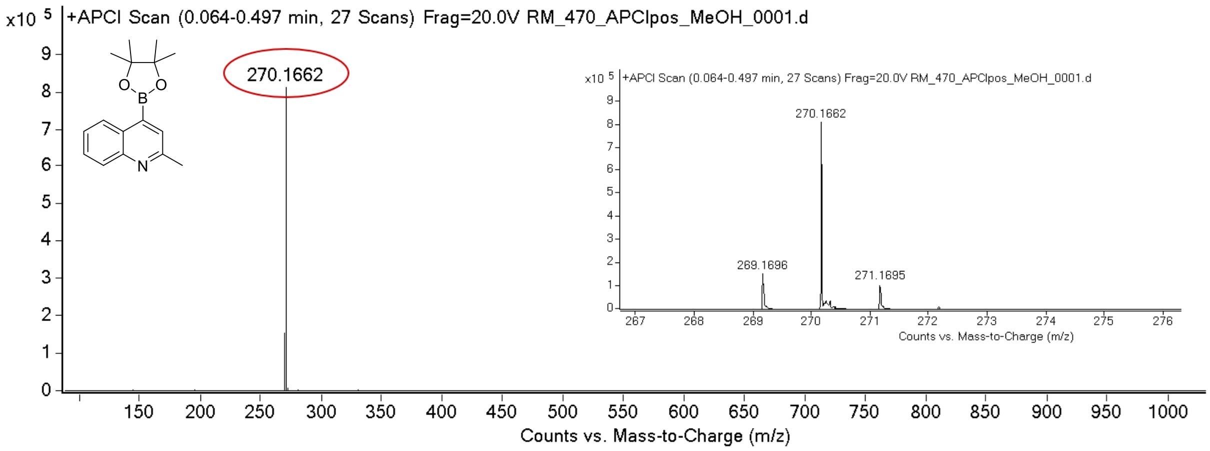

^{1}H (500 MHz) and ^{13}C NMR (126 MHz) spectra of **12** in chloroform-*d*.

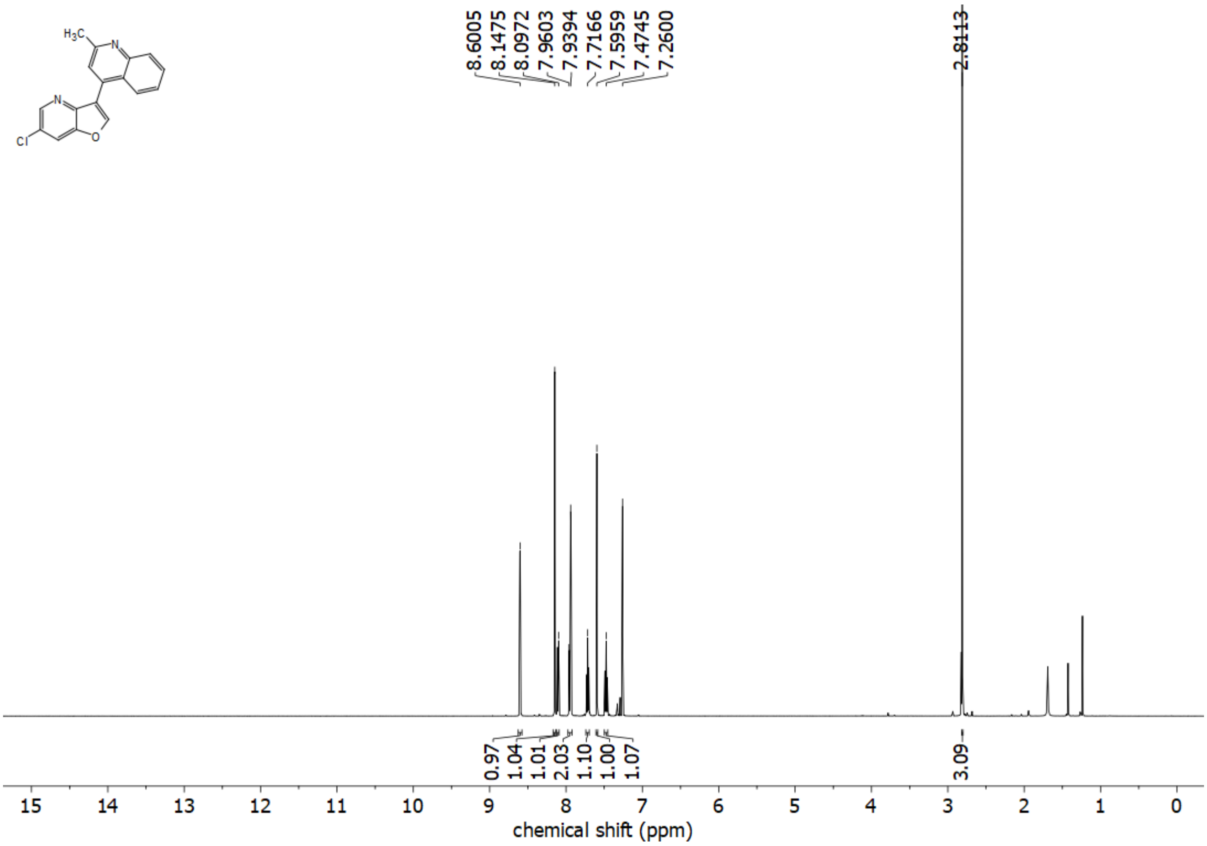

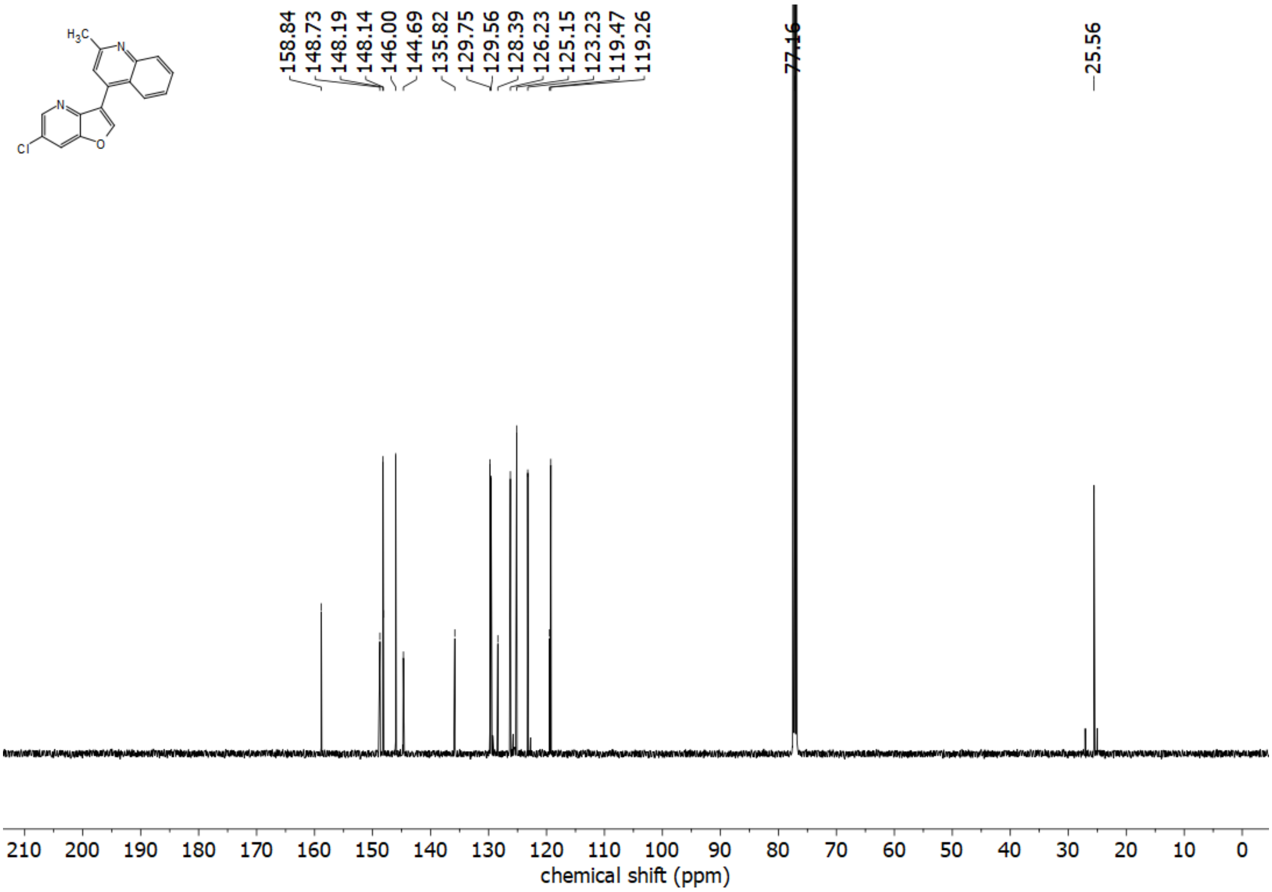

FT-IR spectrum (neat) of **12**.

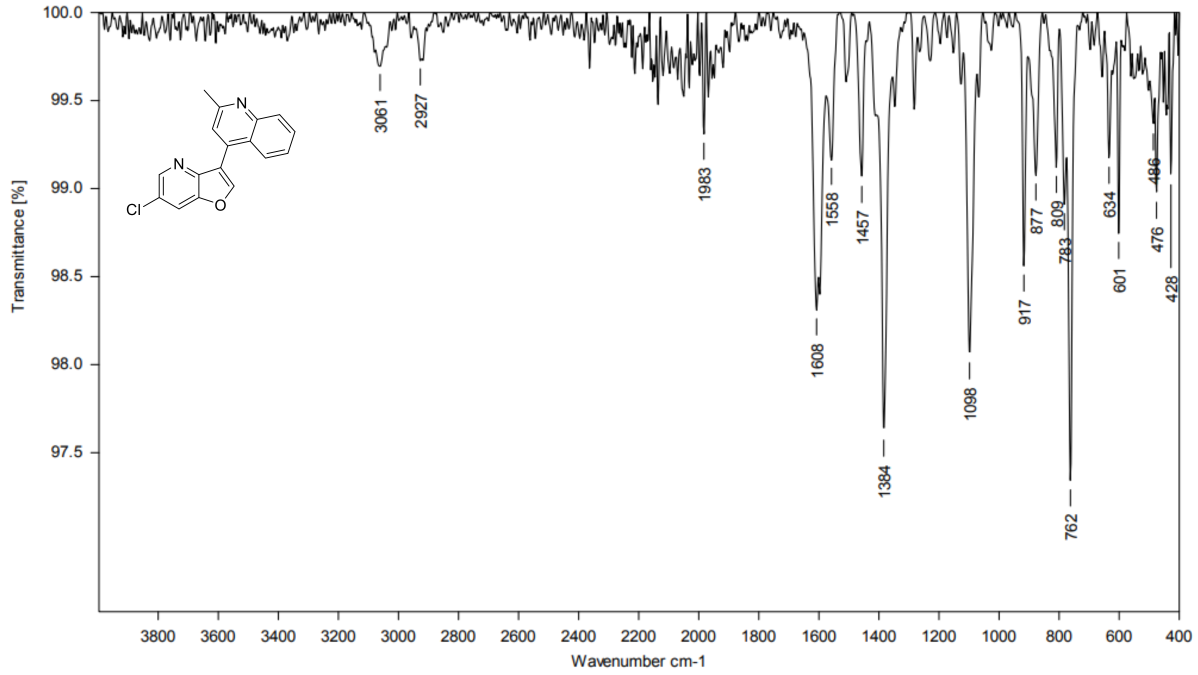

HRMS spectrum of **12**.

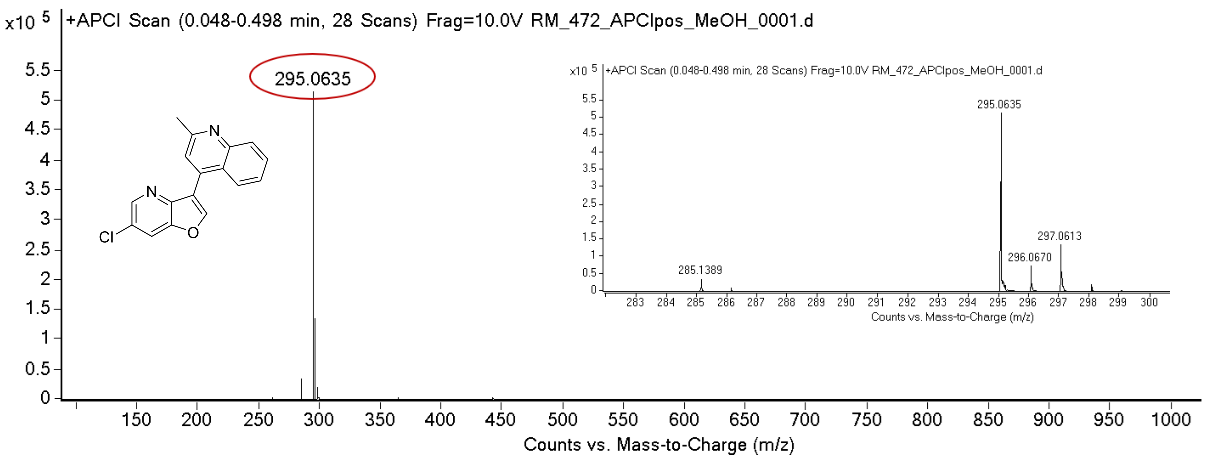

^{1}H (500 MHz) and ^{13}C NMR (126 MHz) spectra of **13** in chloroform-*d*.

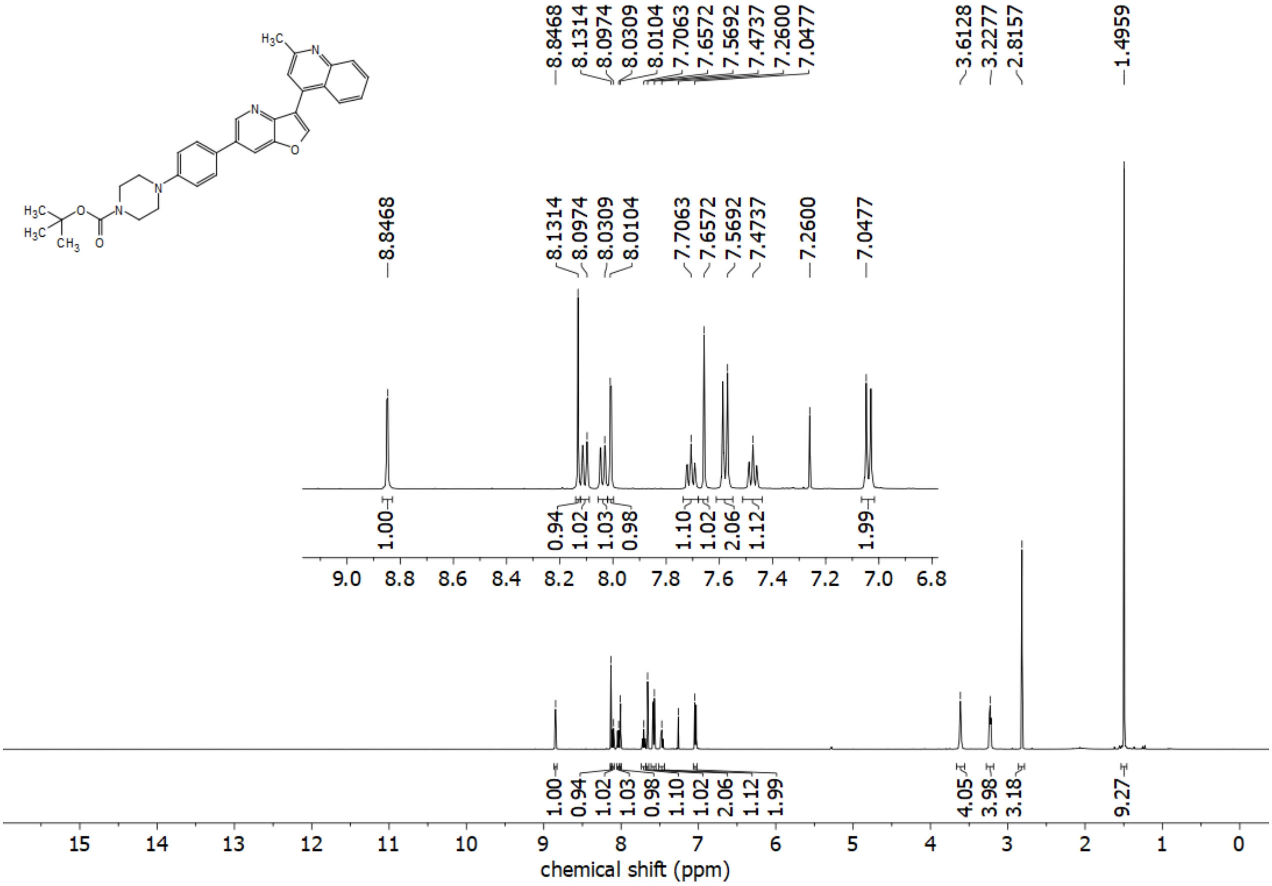

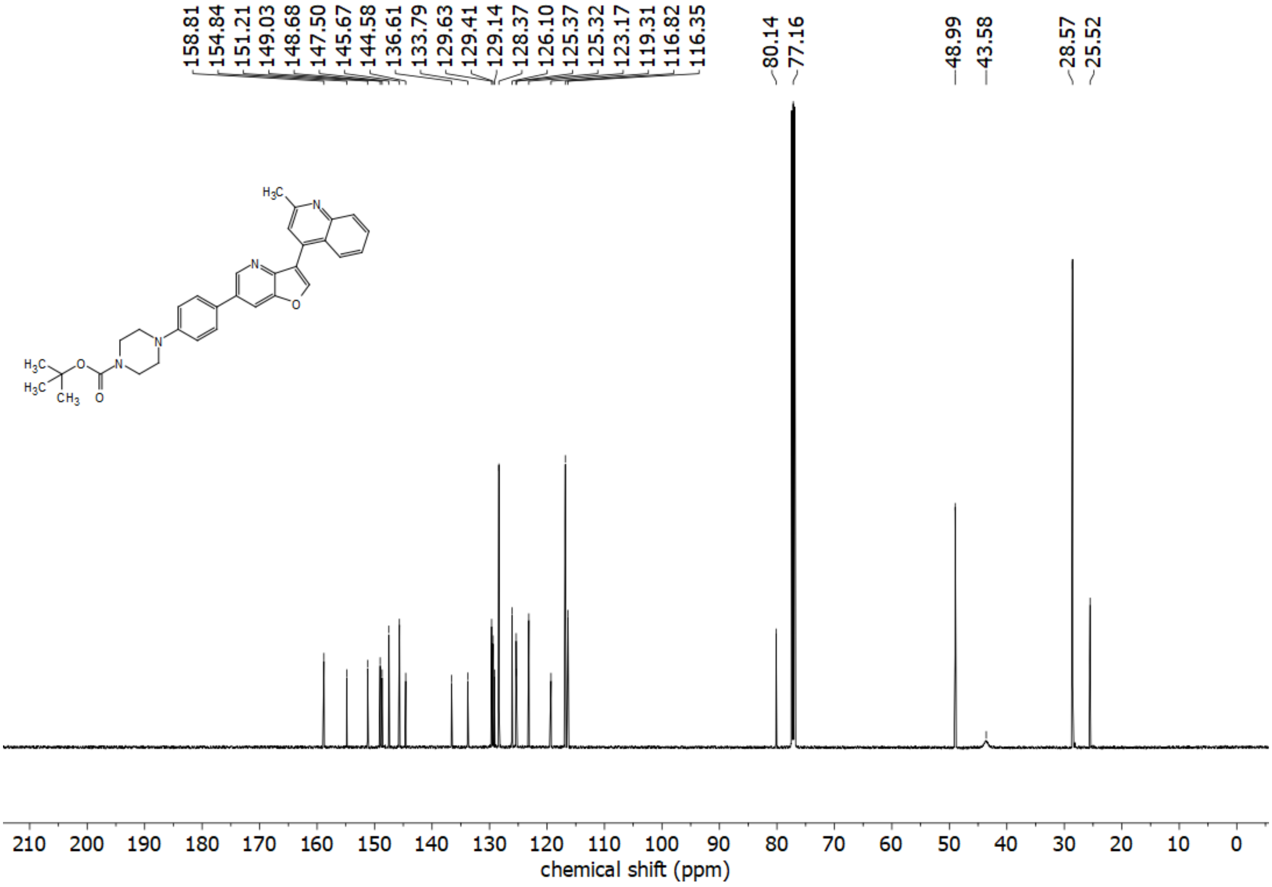

FT-IR spectrum (neat) of **13**.

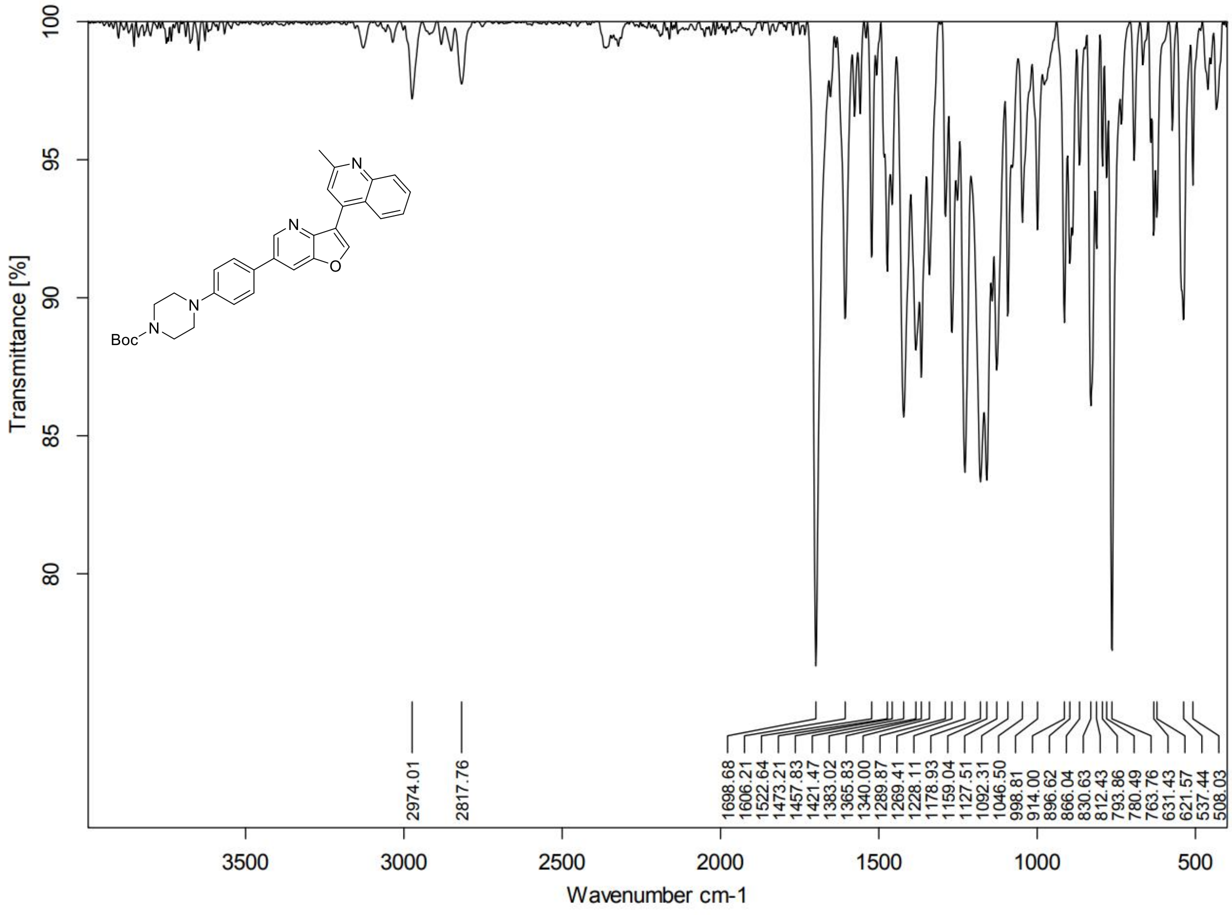

HRMS spectrum of **13**.

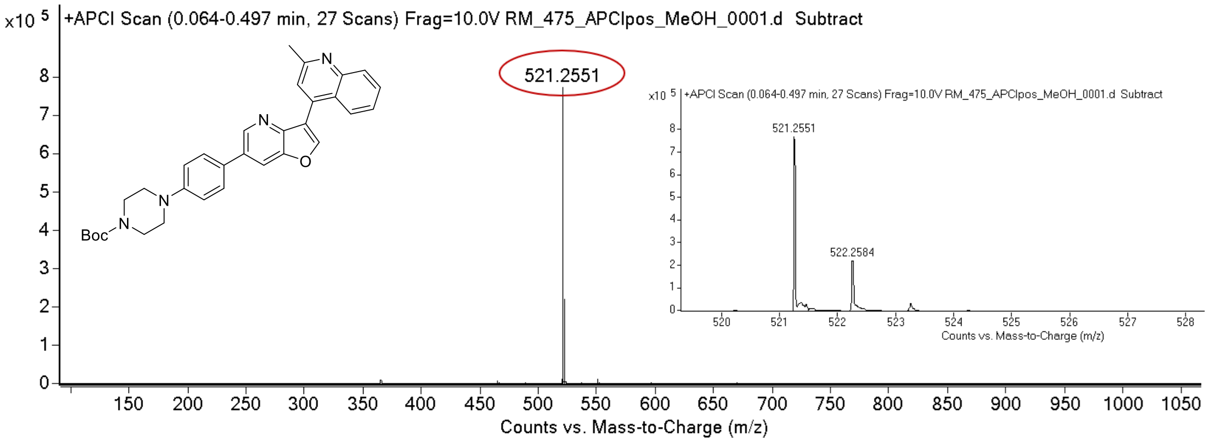

^{1}H (500 MHz) and ^{13}C NMR (126 MHz) spectra of **14** in methanol-d_4.

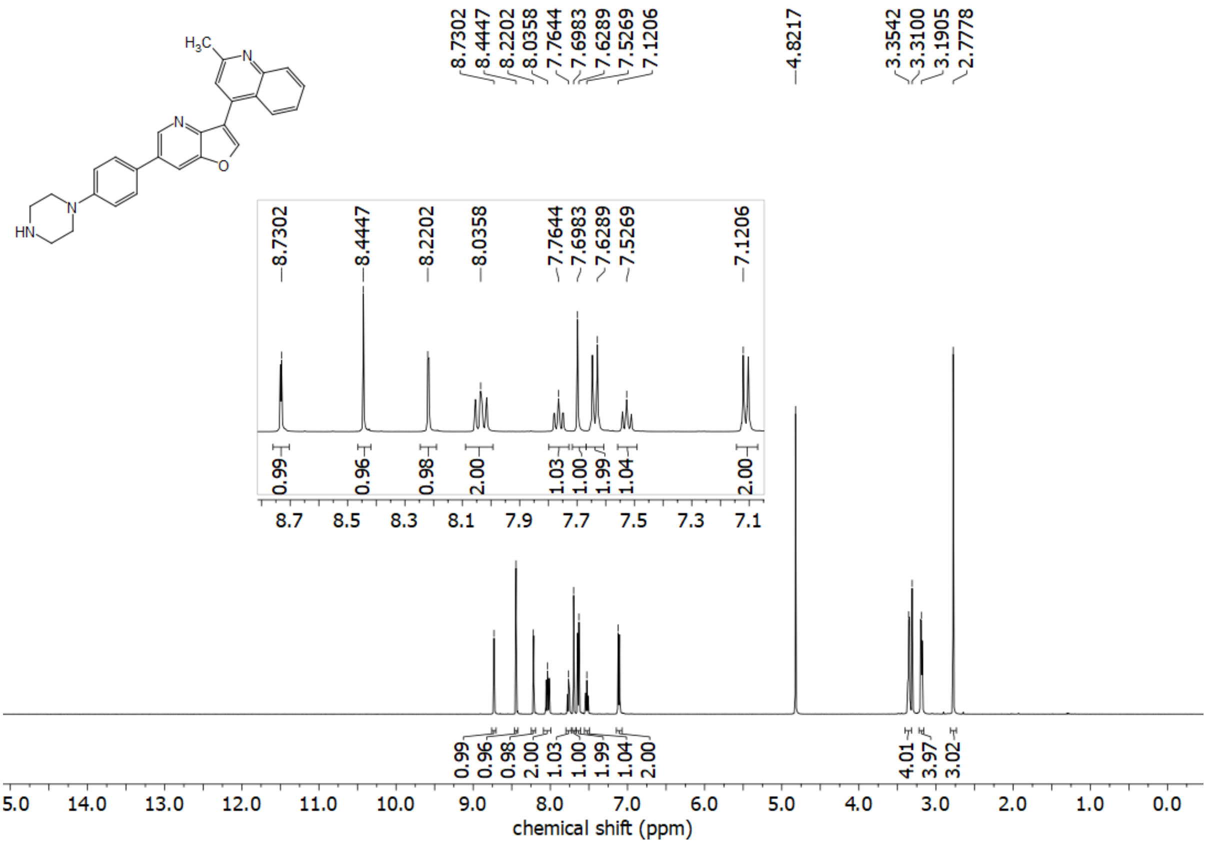

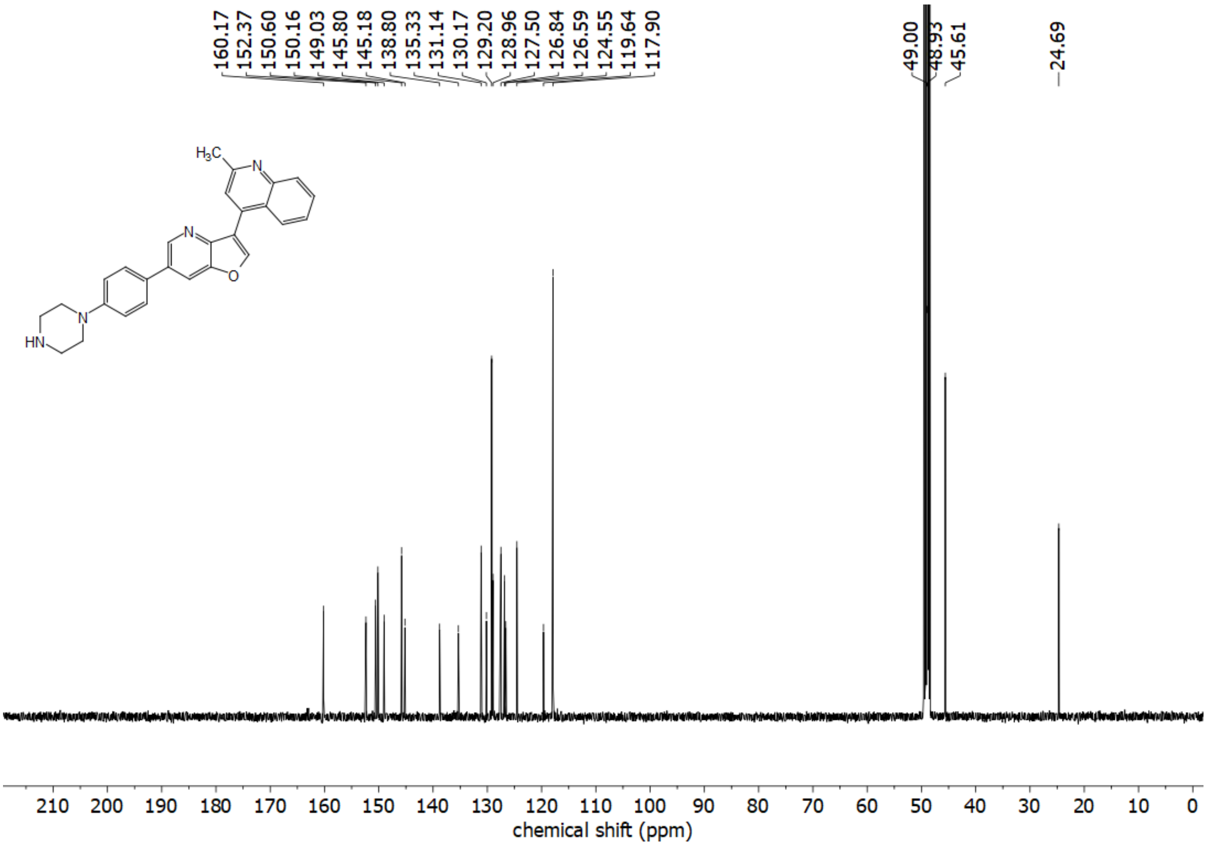

FT-IR spectrum (neat) of **14**.

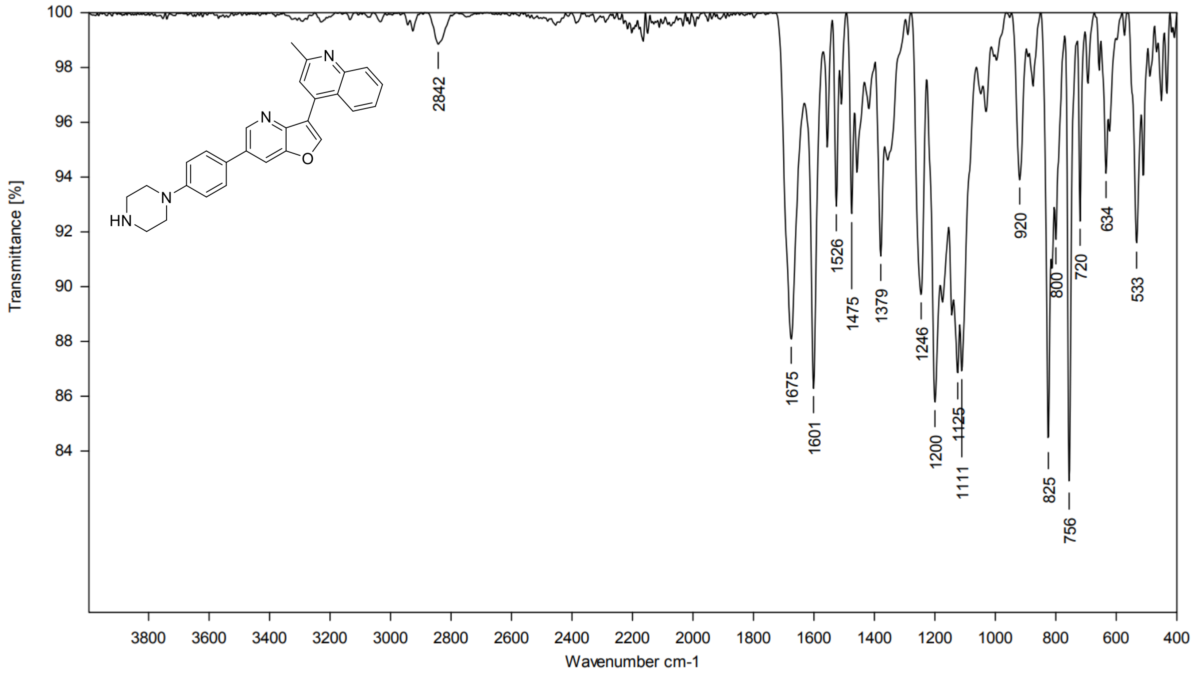

HRMS spectrum of **14**.

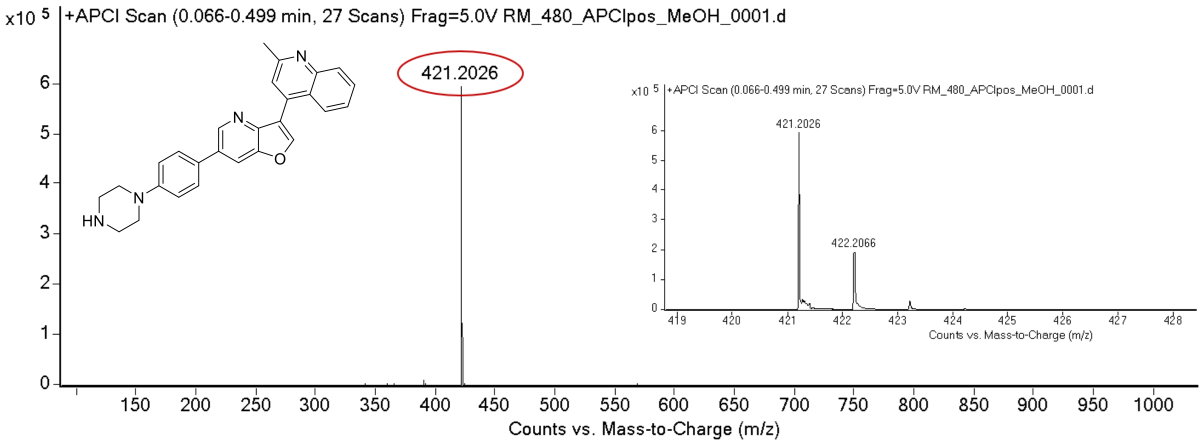

^{1}H (500 MHz) and ^{13}C NMR (126 MHz) spectra of **15** in chloroform-*d*.

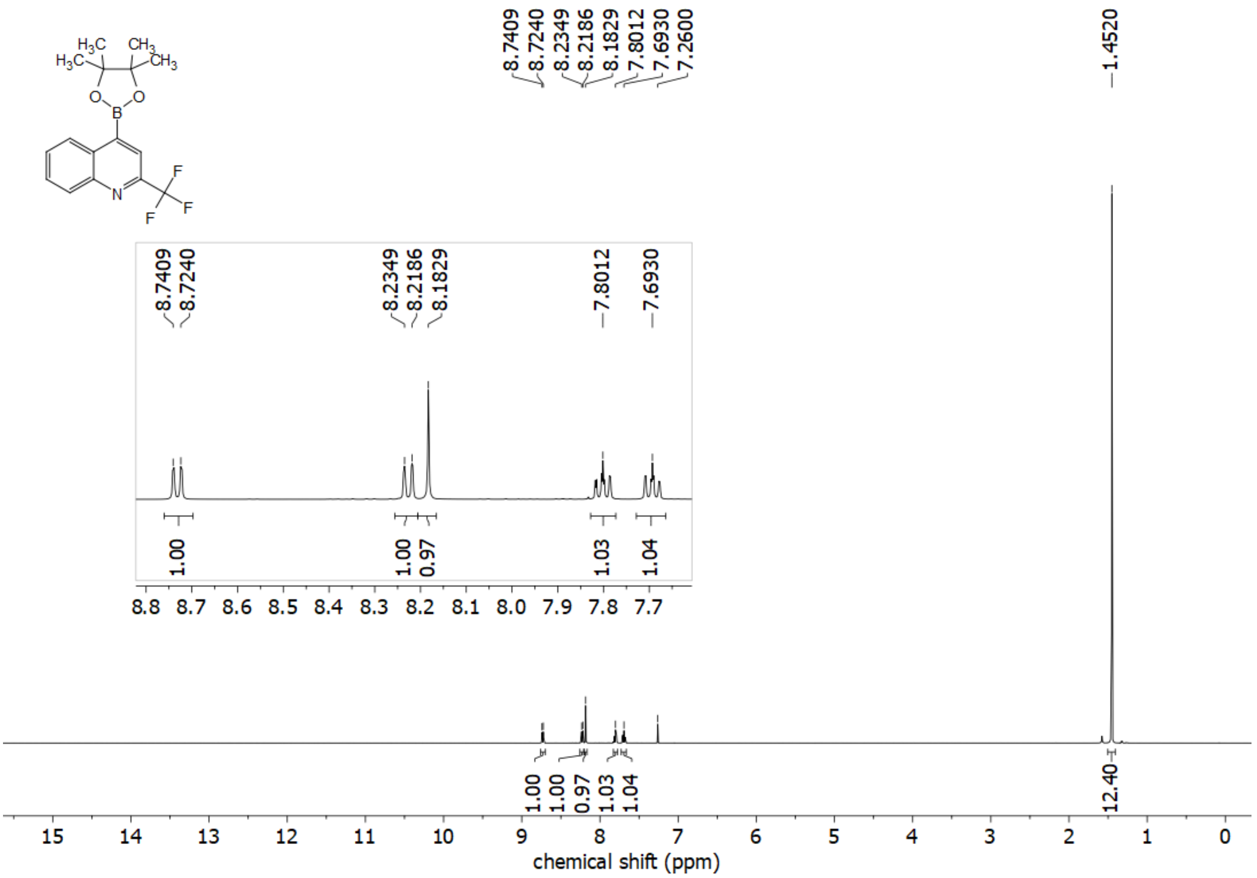

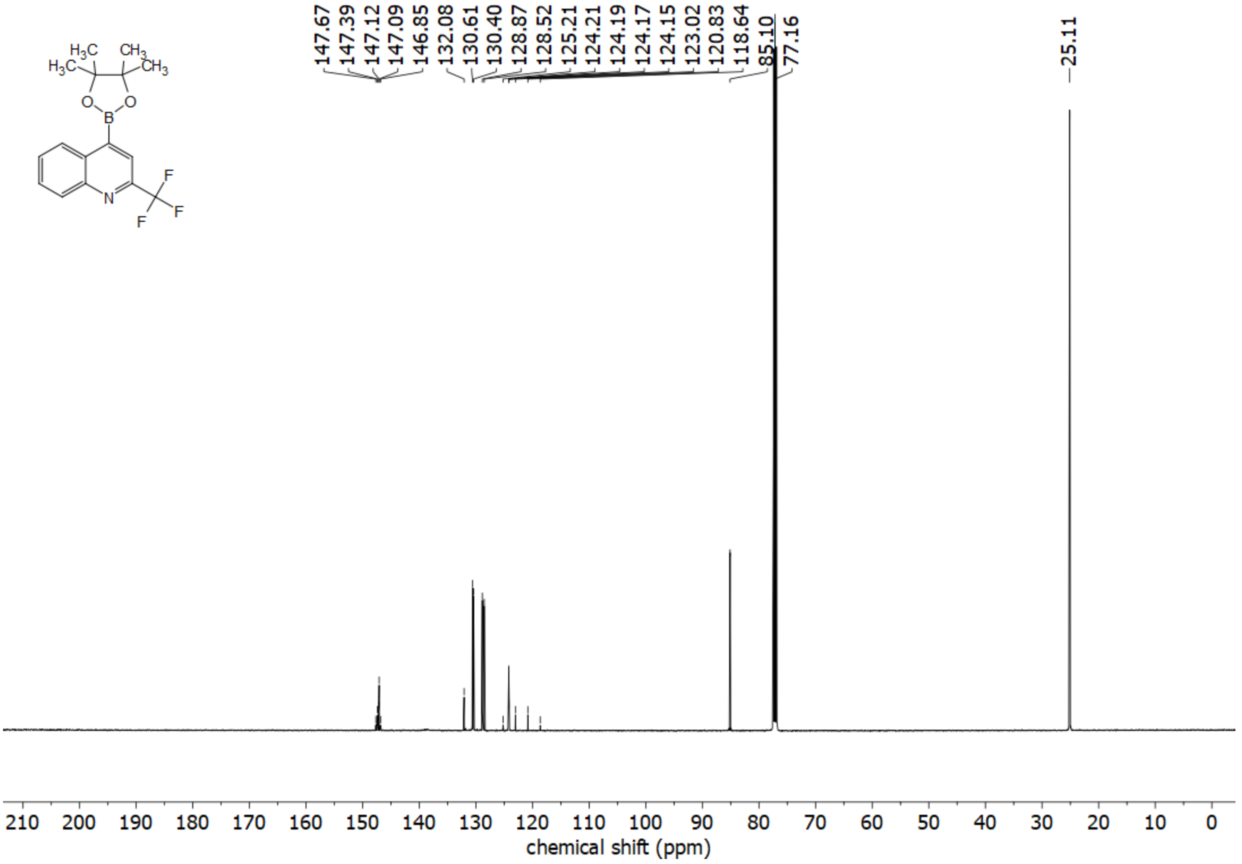

FT-IR spectrum (neat) of **15**.

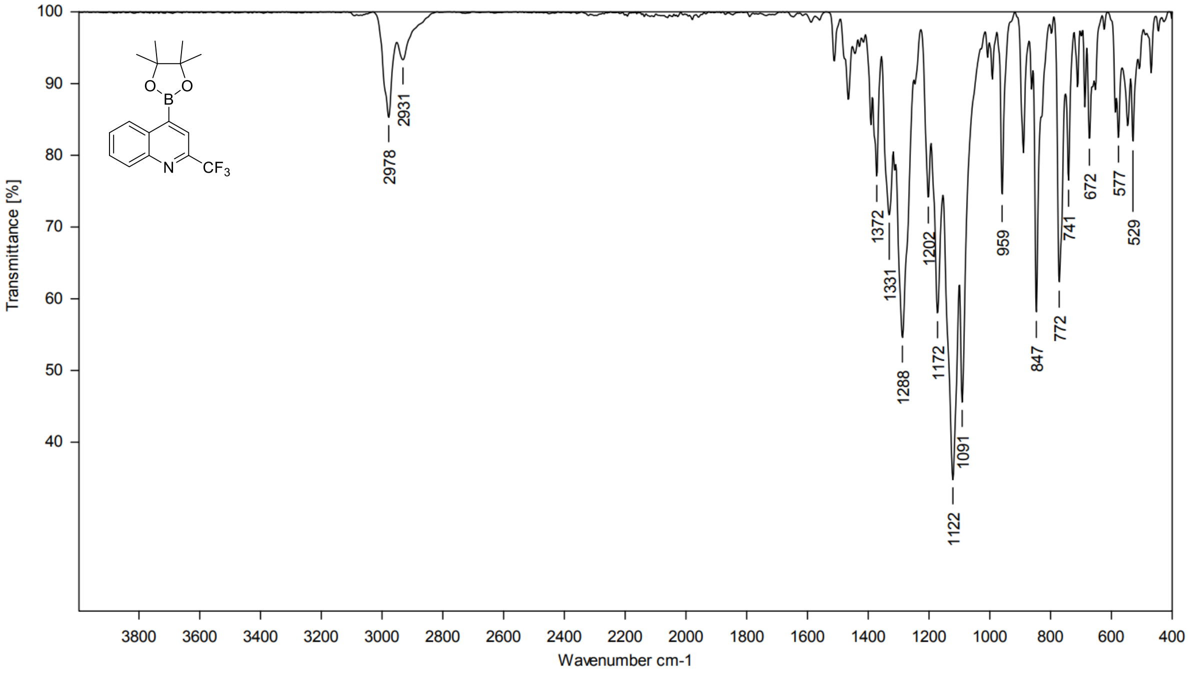

HRMS spectrum of **15**.

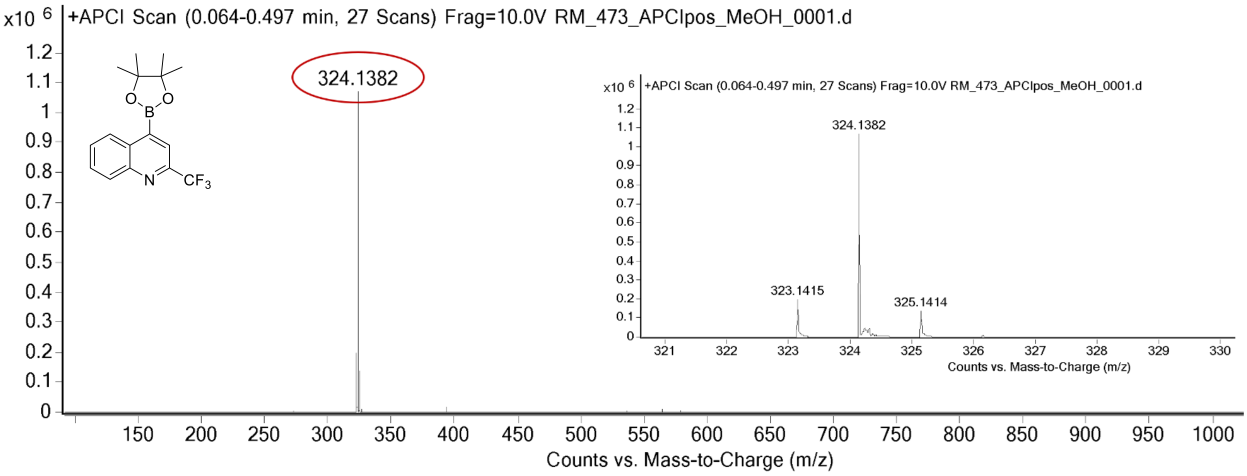

^{1}H (500 MHz) and ^{13}C NMR (126 MHz) spectra of **16** in chloroform-*d*.

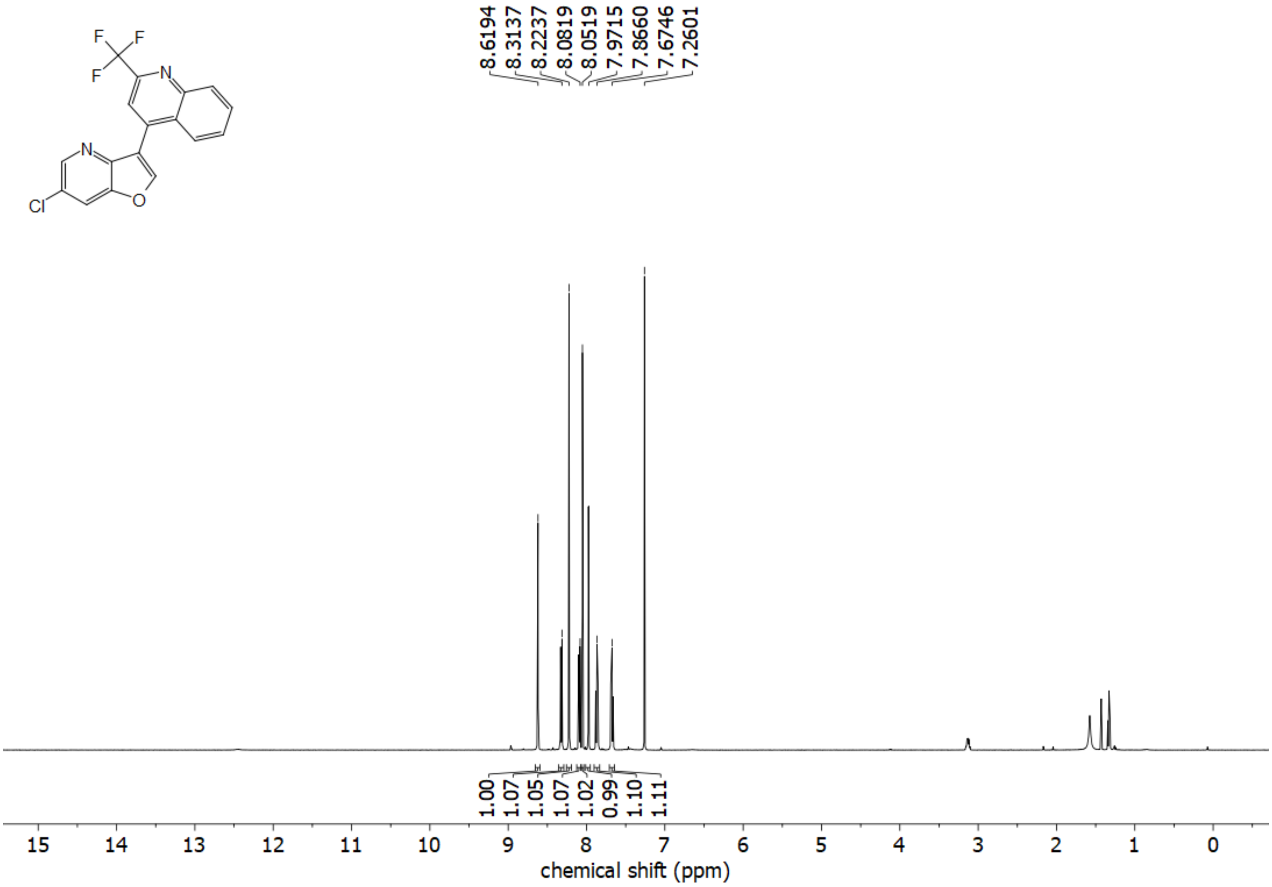

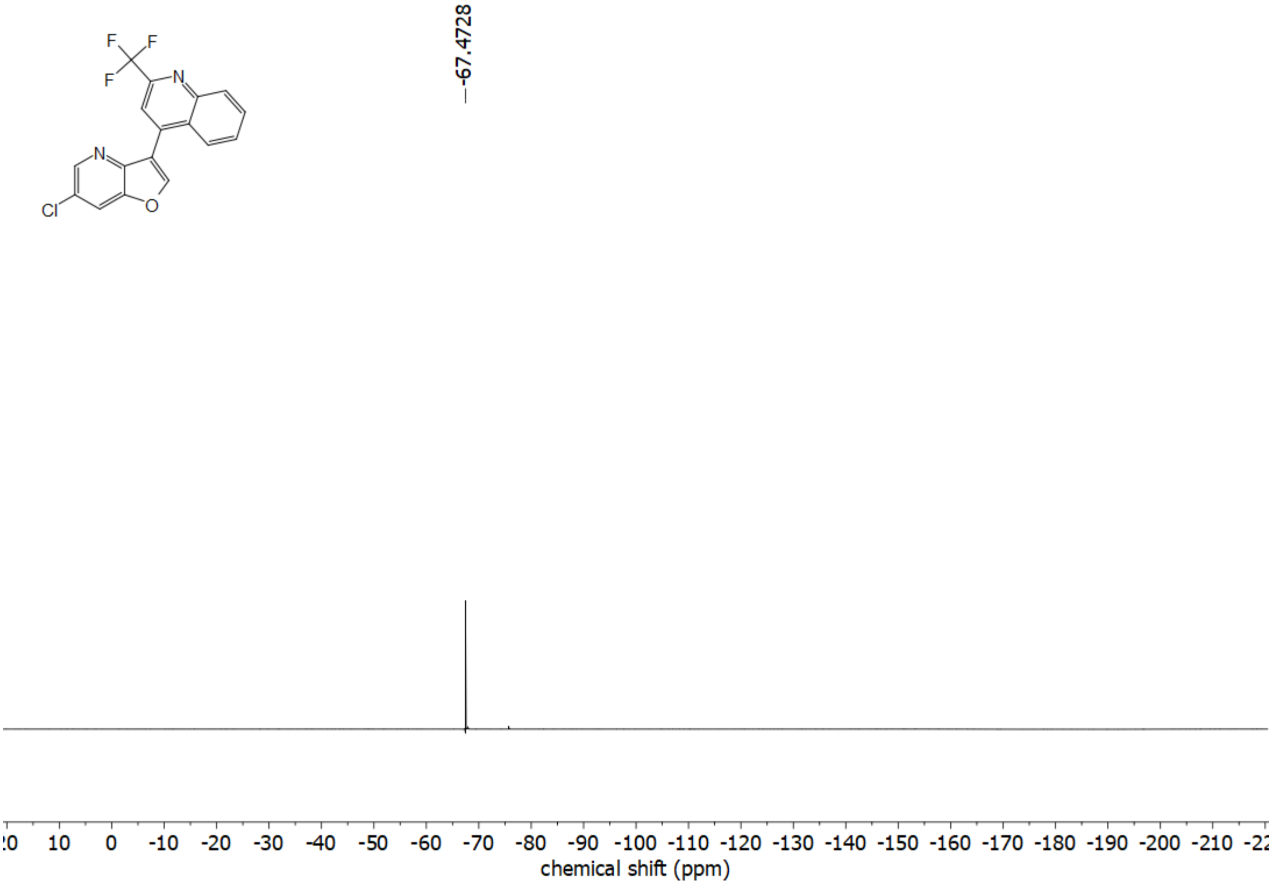

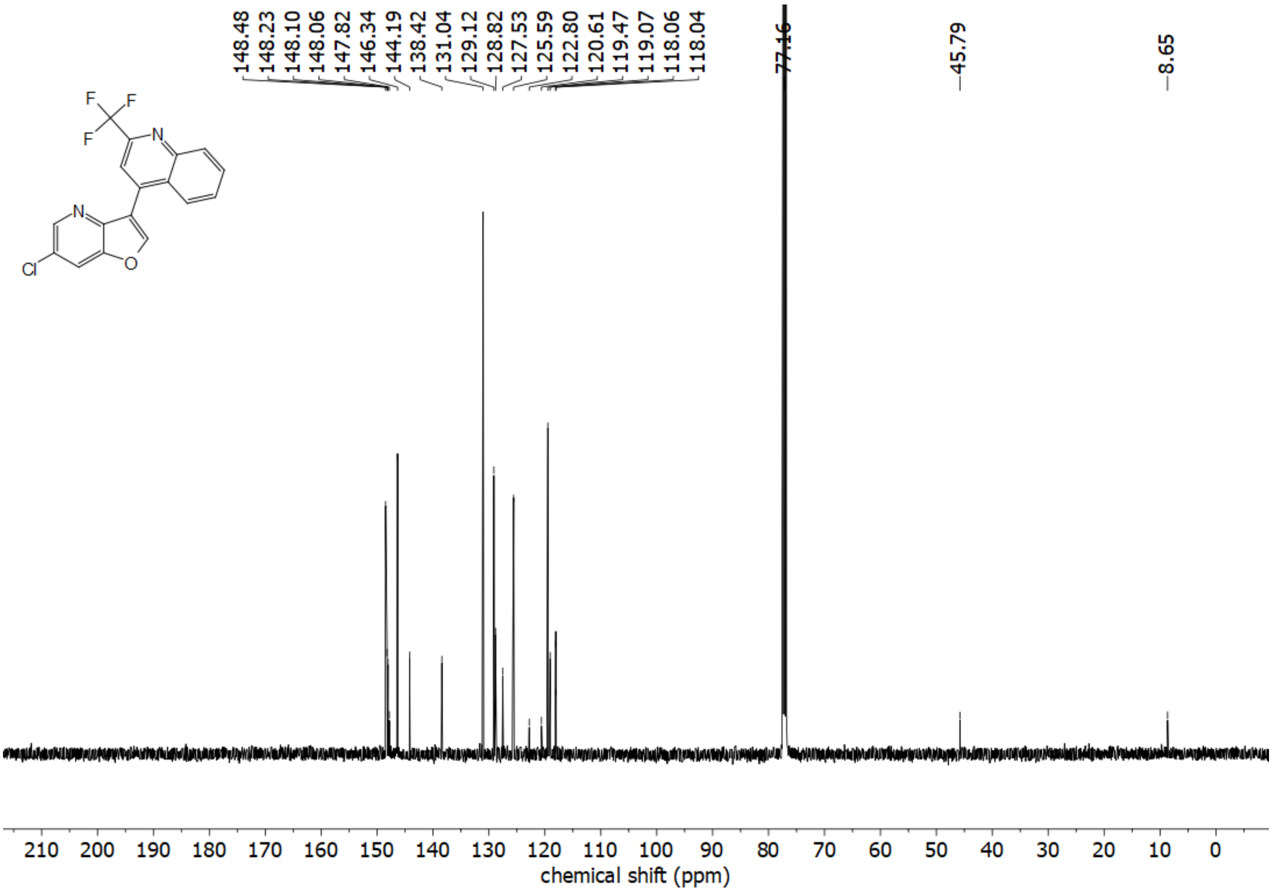

FT-IR spectrum (neat) of **16**.

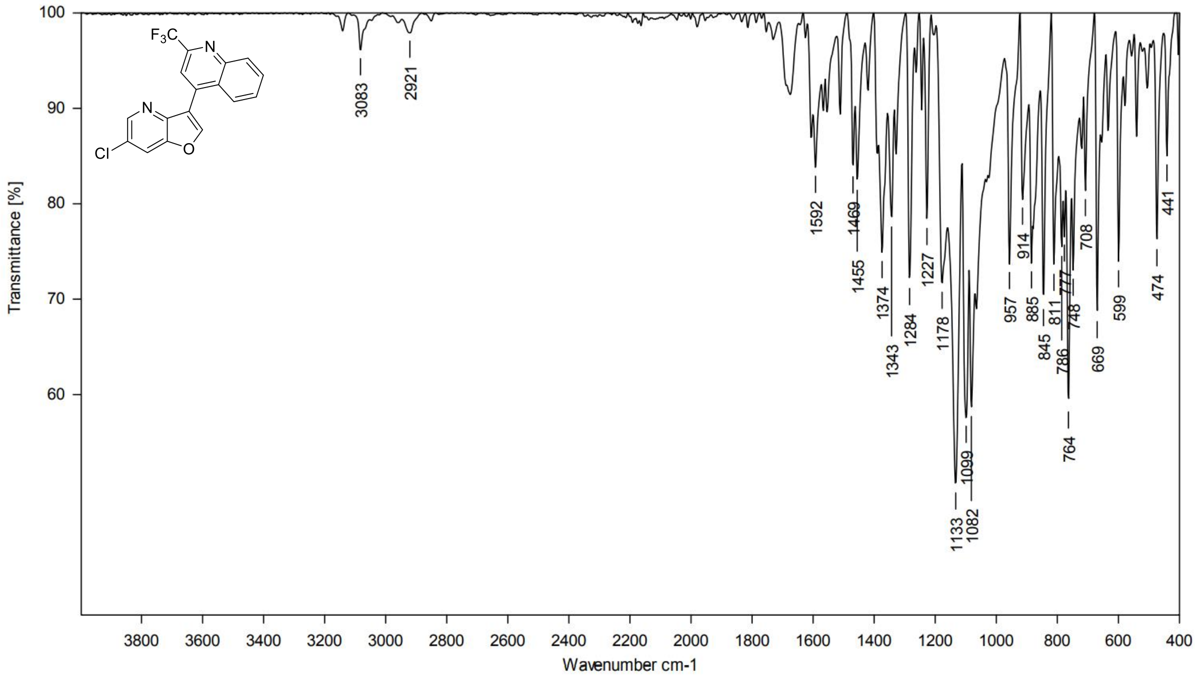

HRMS spectrum of **16**.

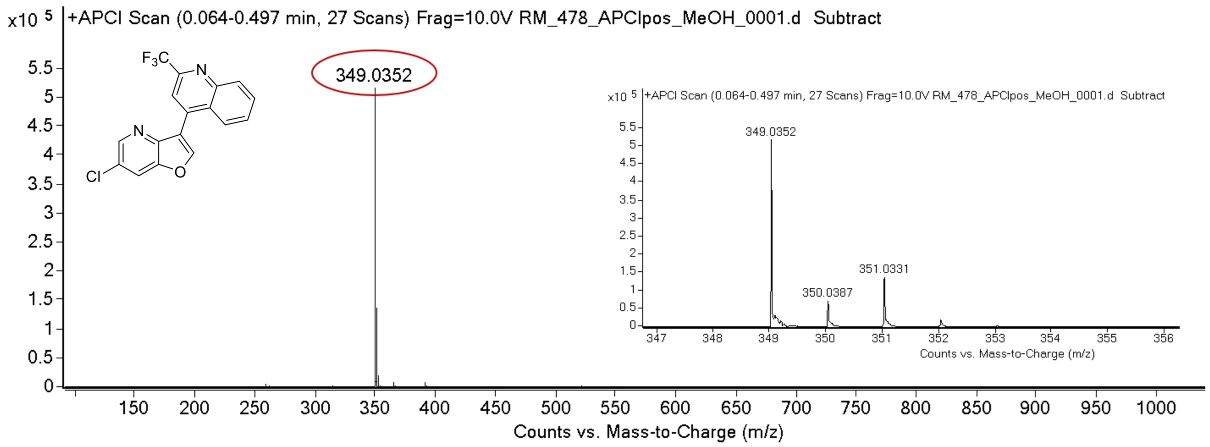

^{1}H (500 MHz), ^{13}C NMR (126 MHz), and ^{19}F NMR (471 MHz) spectra of **17** in chloroform-*d*.

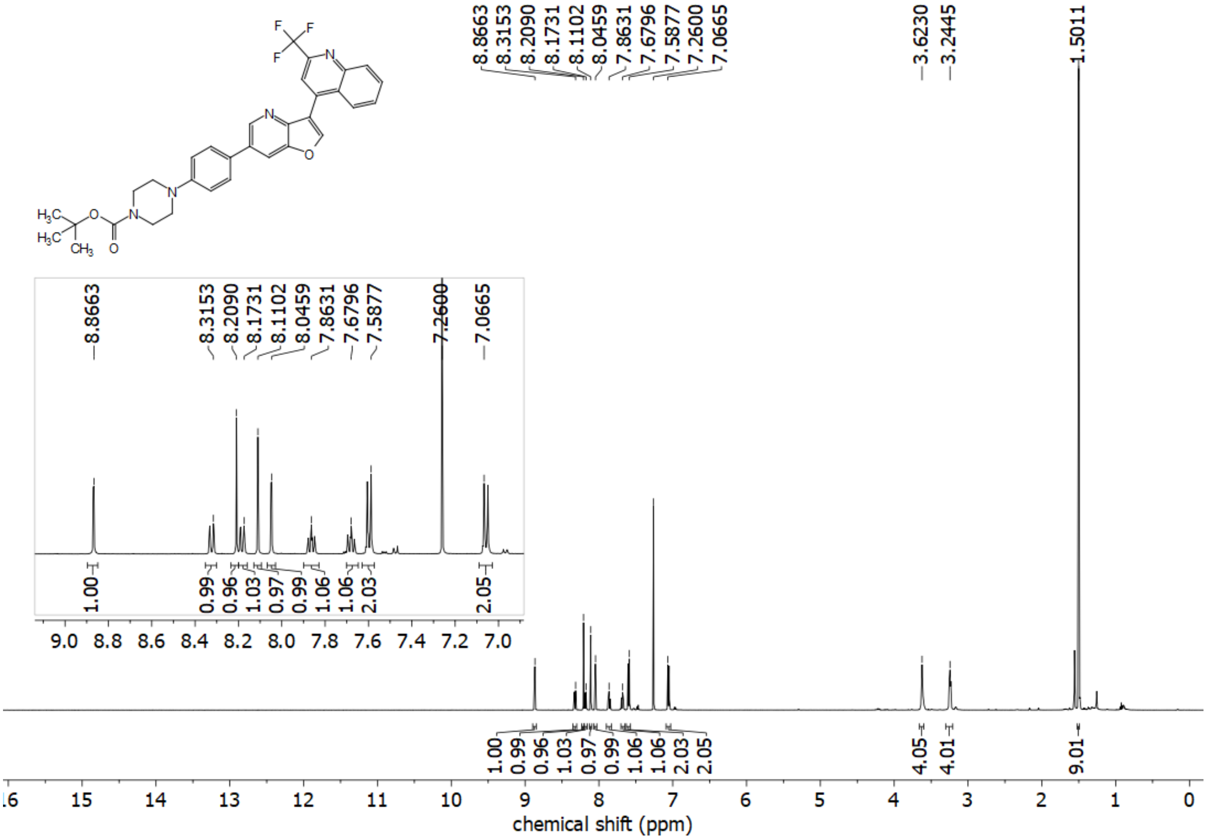

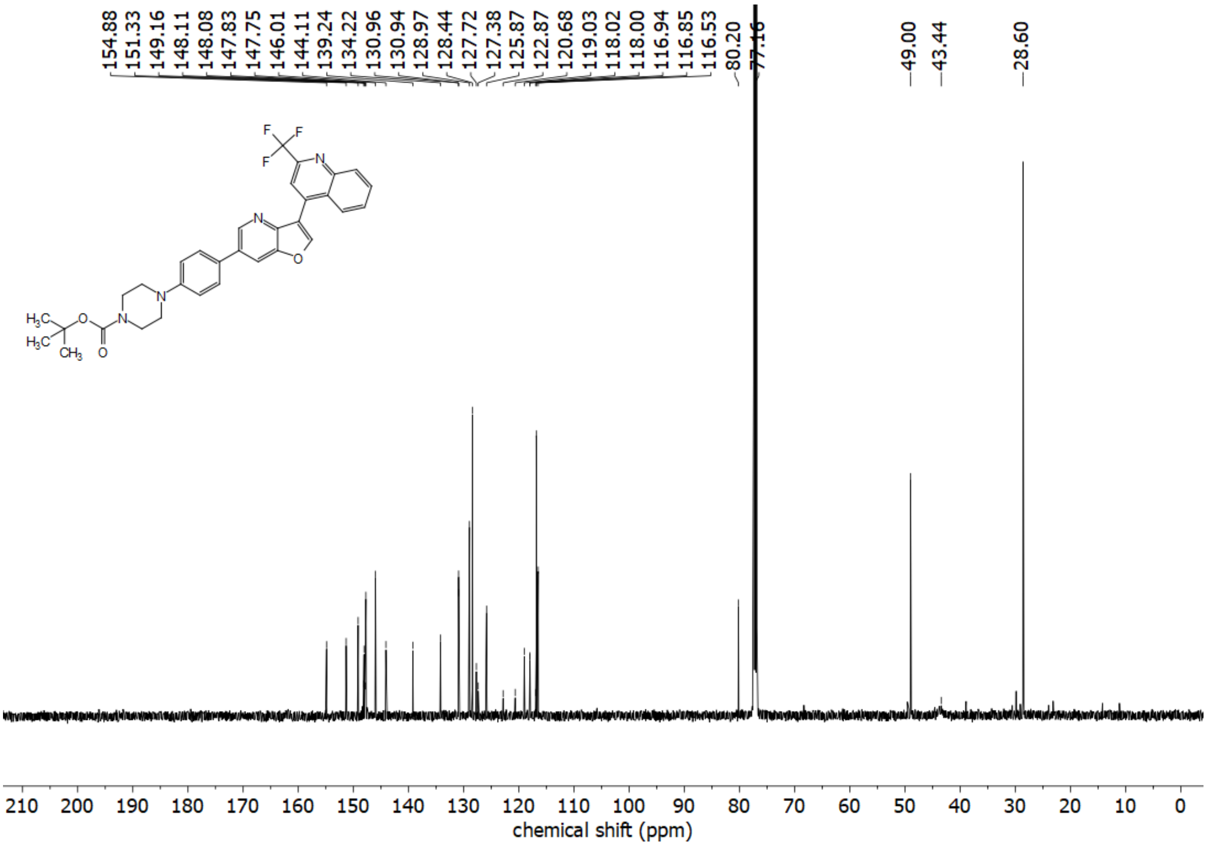

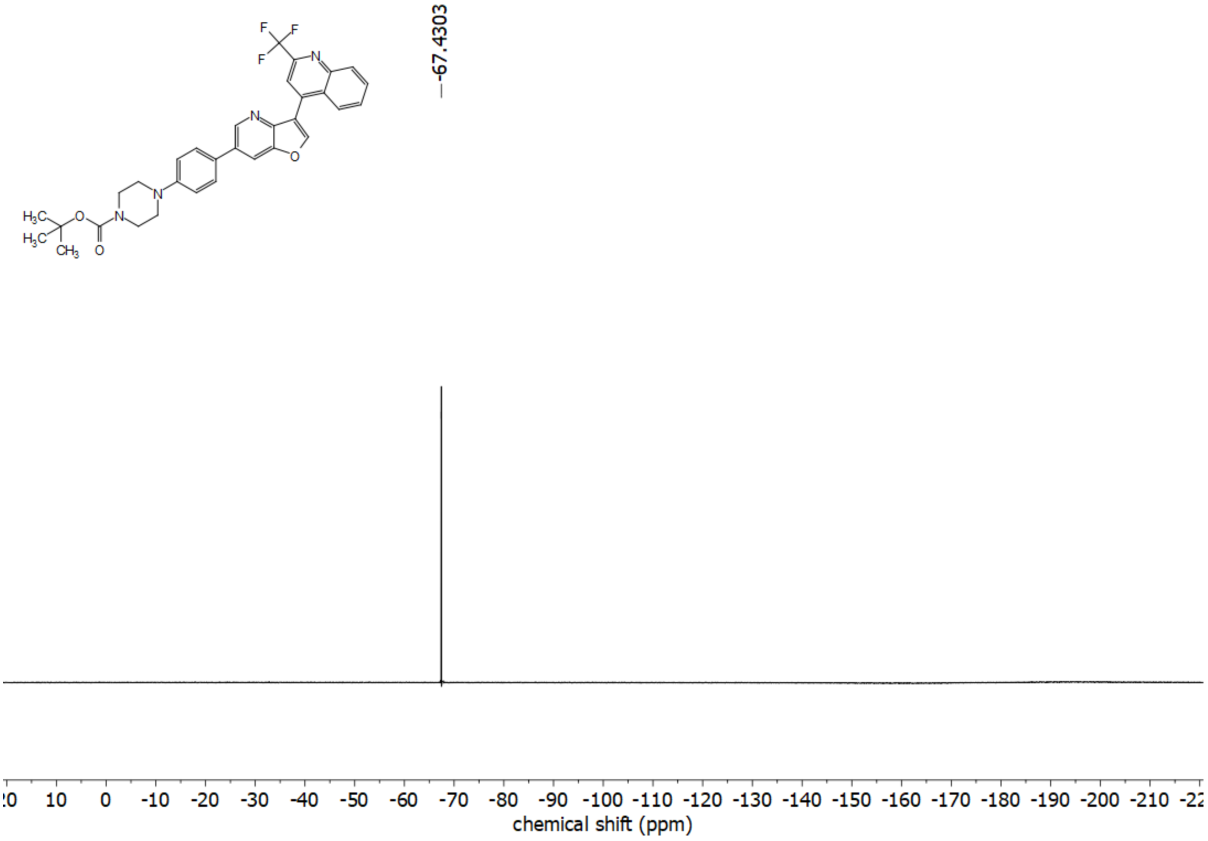

FT-IR spectrum (neat) of **17**.

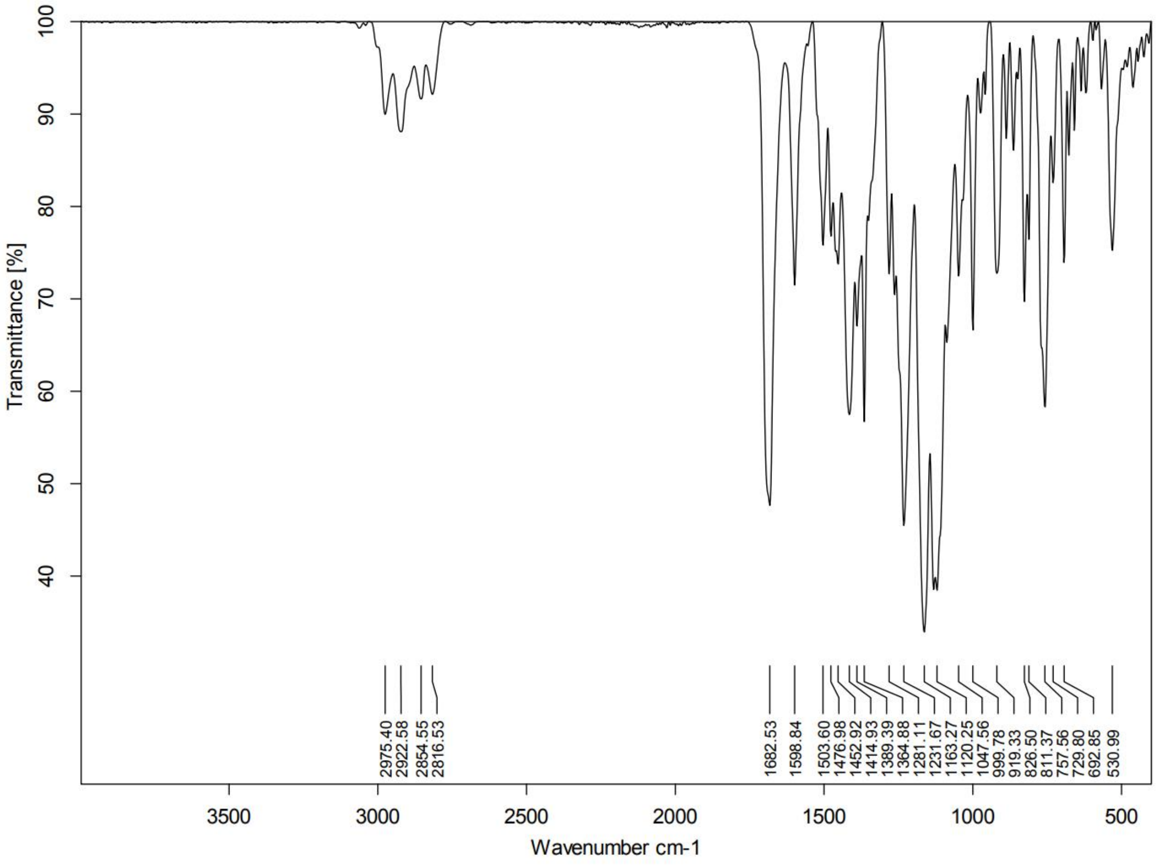

HRMS spectrum of **17**.

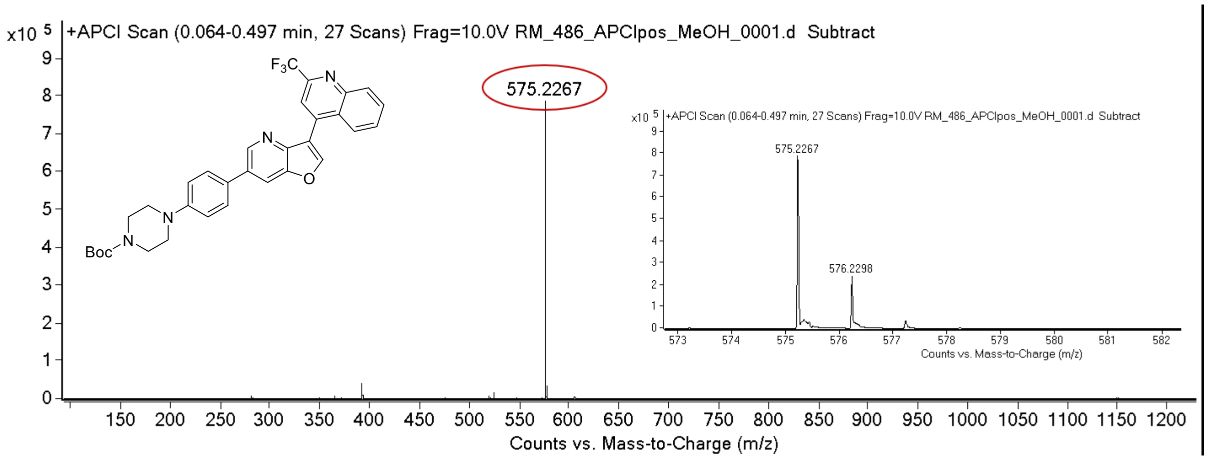

^{1}H (500 MHz), ^{13}C NMR (126 MHz), and ^{19}F NMR (471 MHz) spectra of **18** in chloroform-*d*.

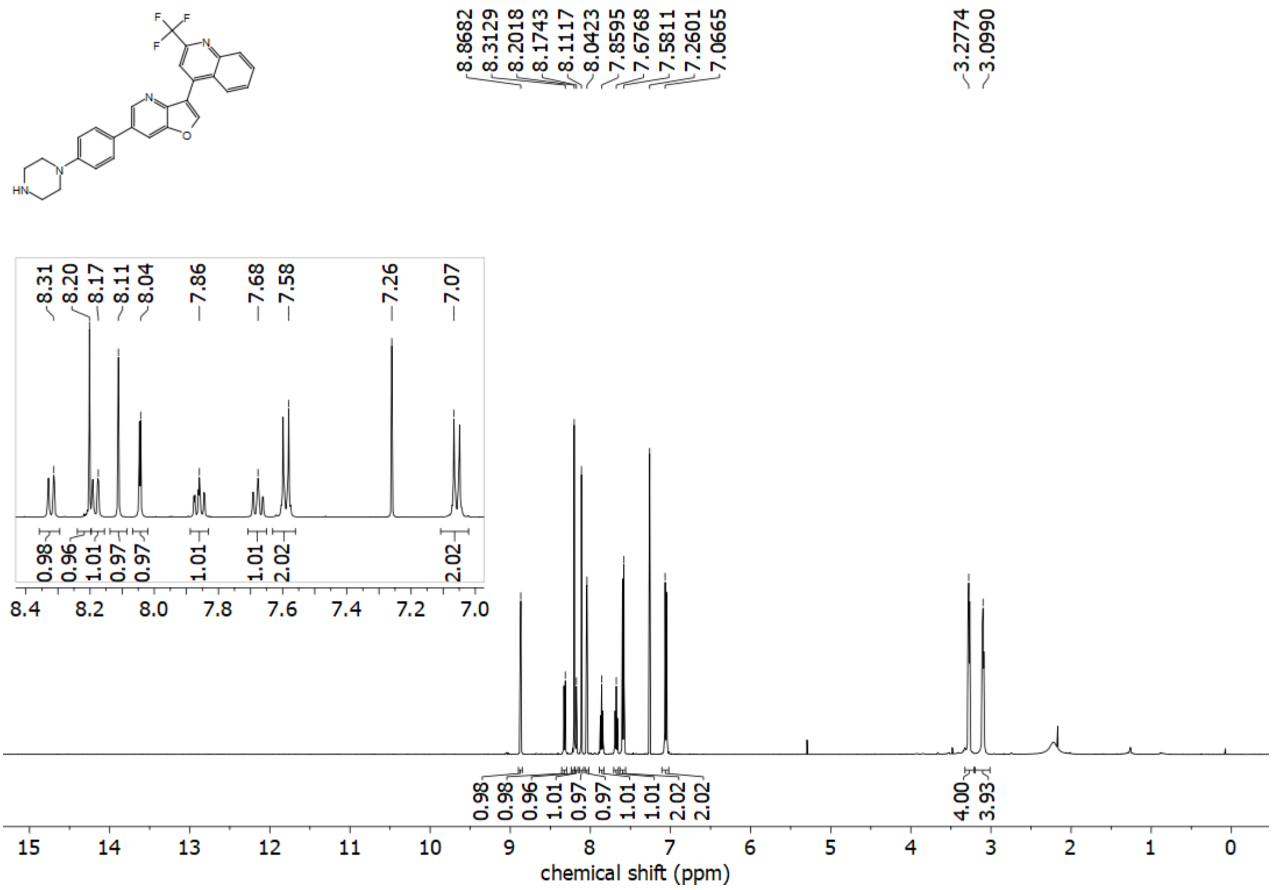

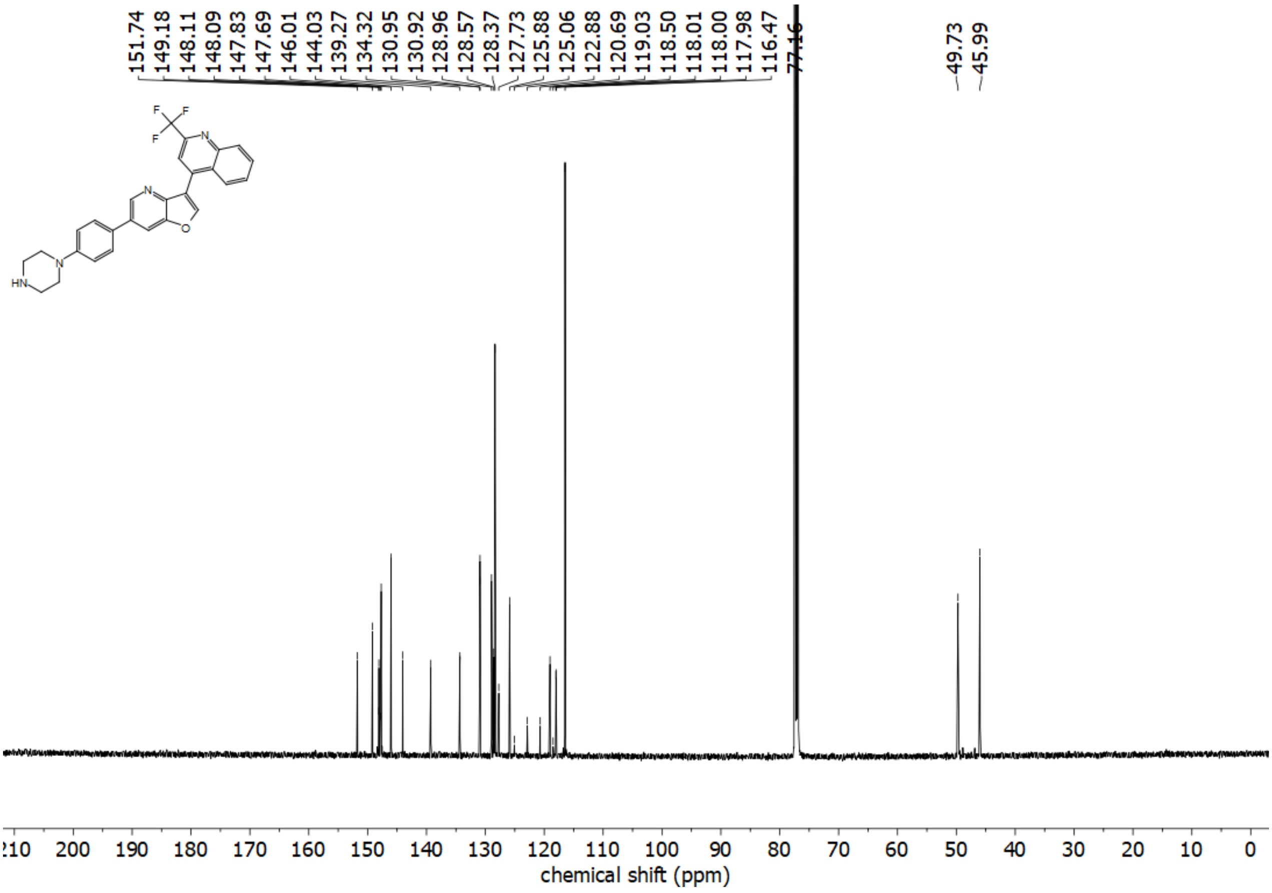

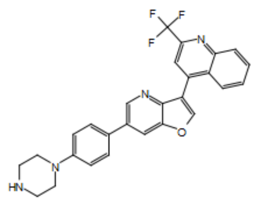

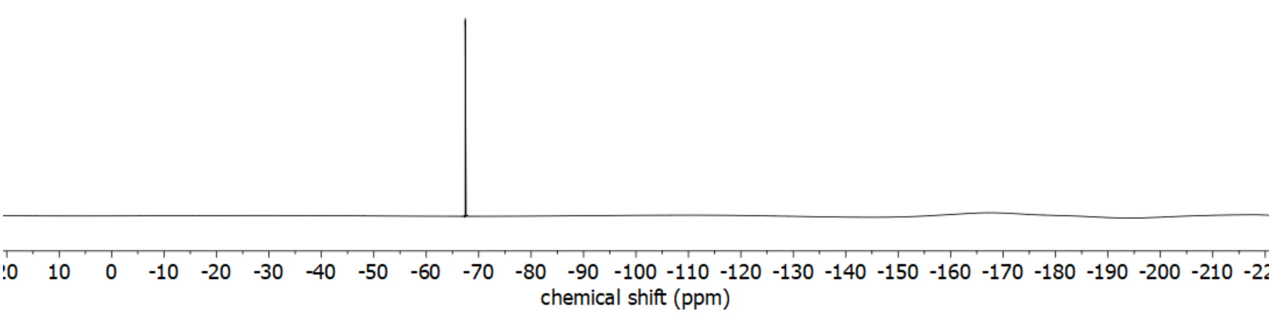

-67.43
20
10
0
-10
-20
-30
-40
-50
-60
-70
-80
-90
-100
-110
-120
-130
-140
-150
-160
-170
-180
-190
-200
-210
chemical shift (ppm)

FT-IR spectrum (neat) of **18**.

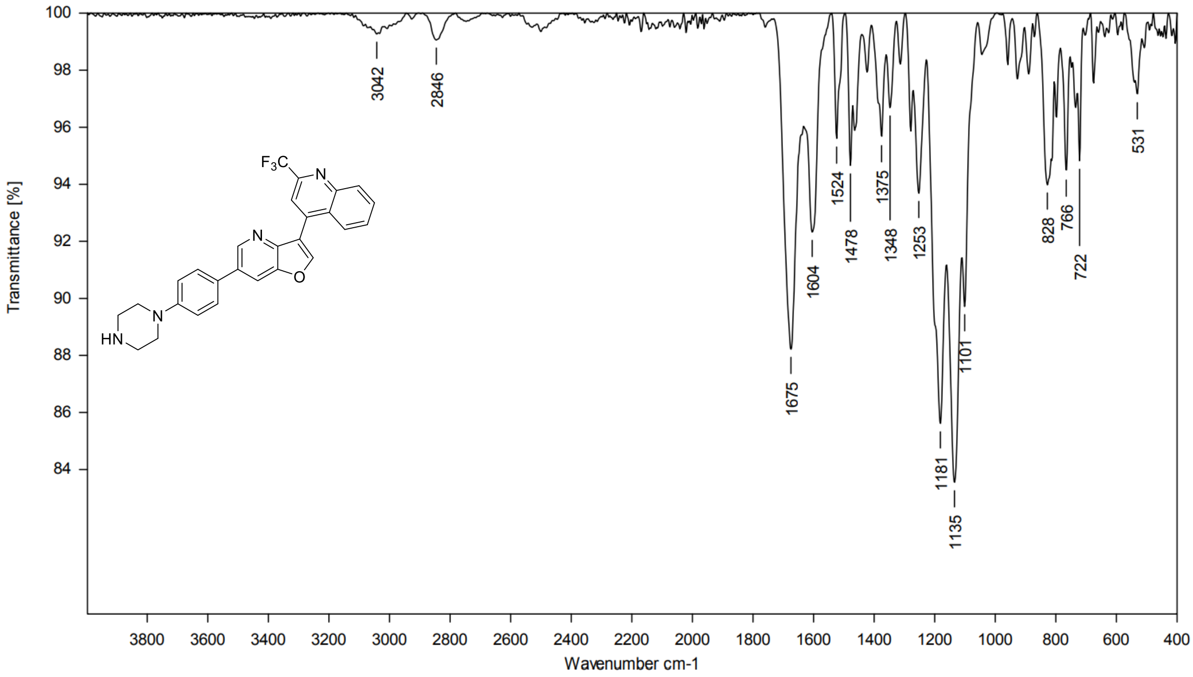

HRMS spectrum of **18**.

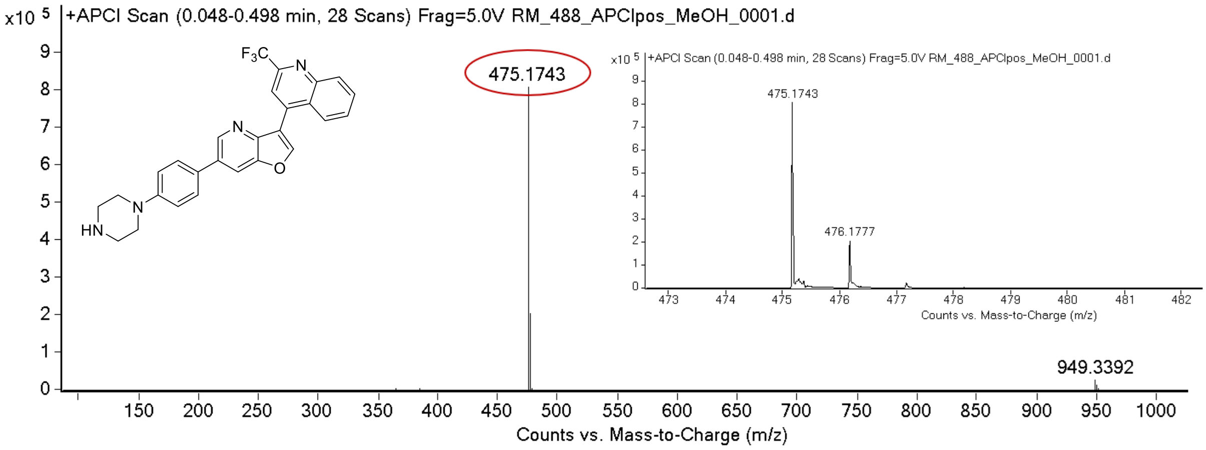

^{1}H (500 MHz) and ^{13}C NMR (126 MHz) spectra of **19** in chloroform-*d*.

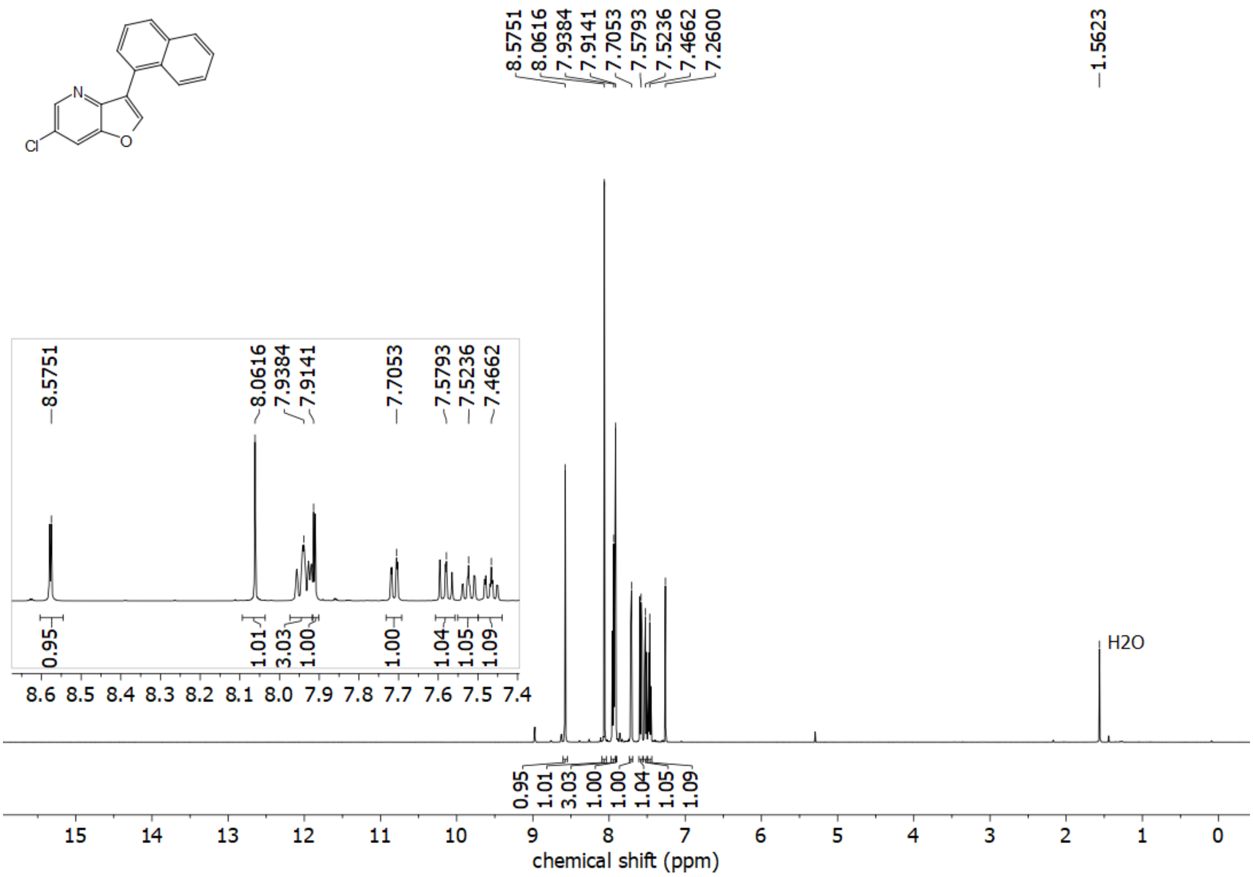

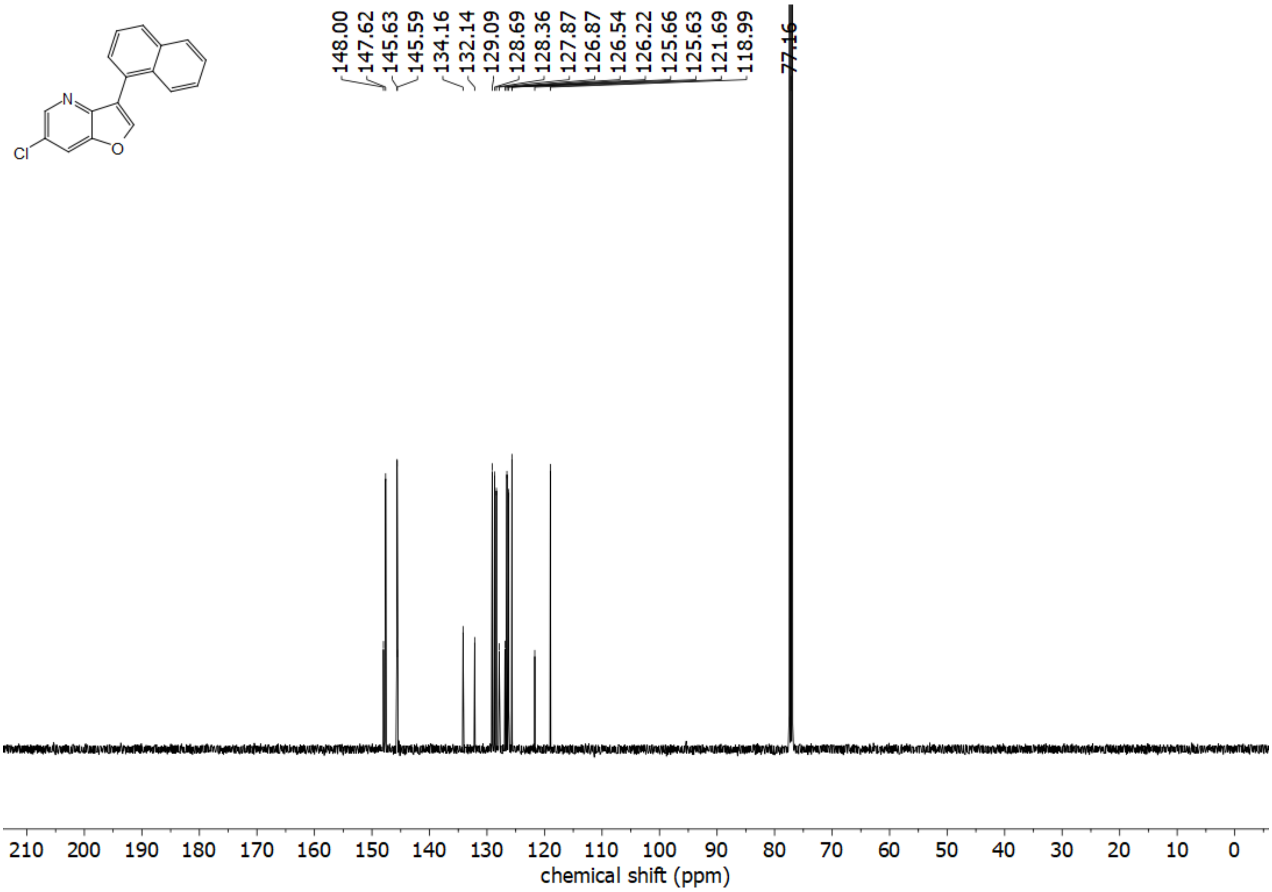

FT-IR spectrum (neat) of **19**.

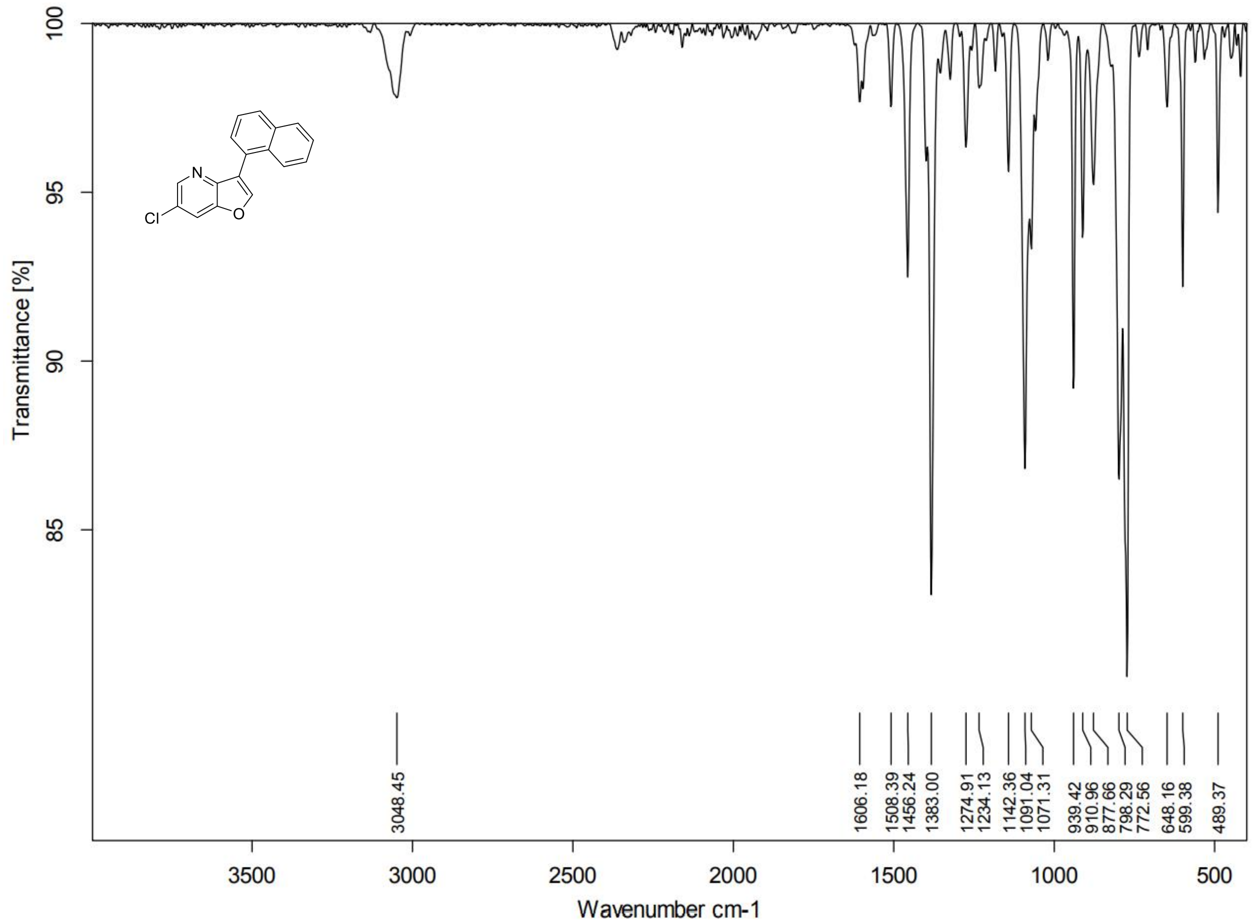

HRMS spectrum of **19**.

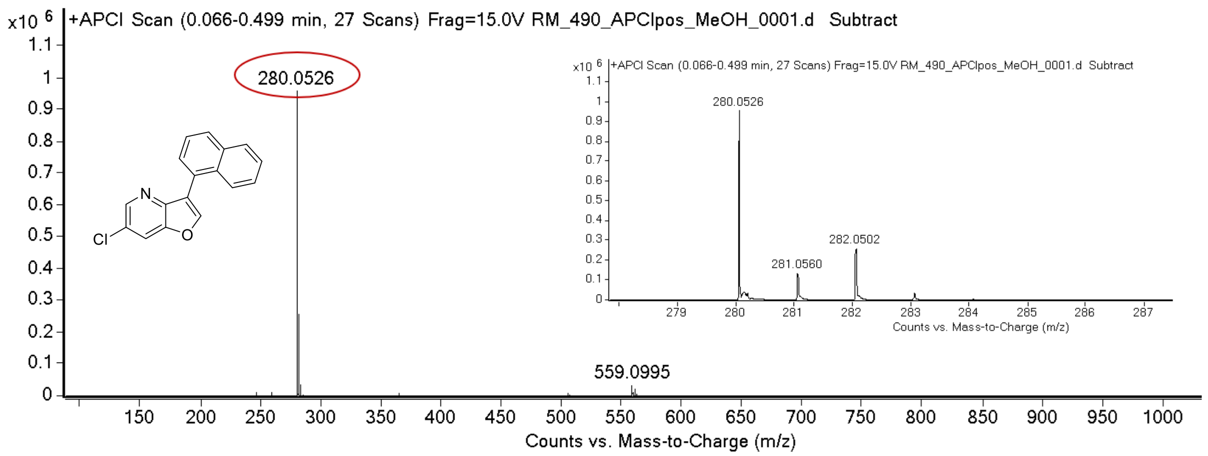

^{1}H (500 MHz) and ^{13}C NMR (126 MHz) spectra of **20** in chloroform-*d*.

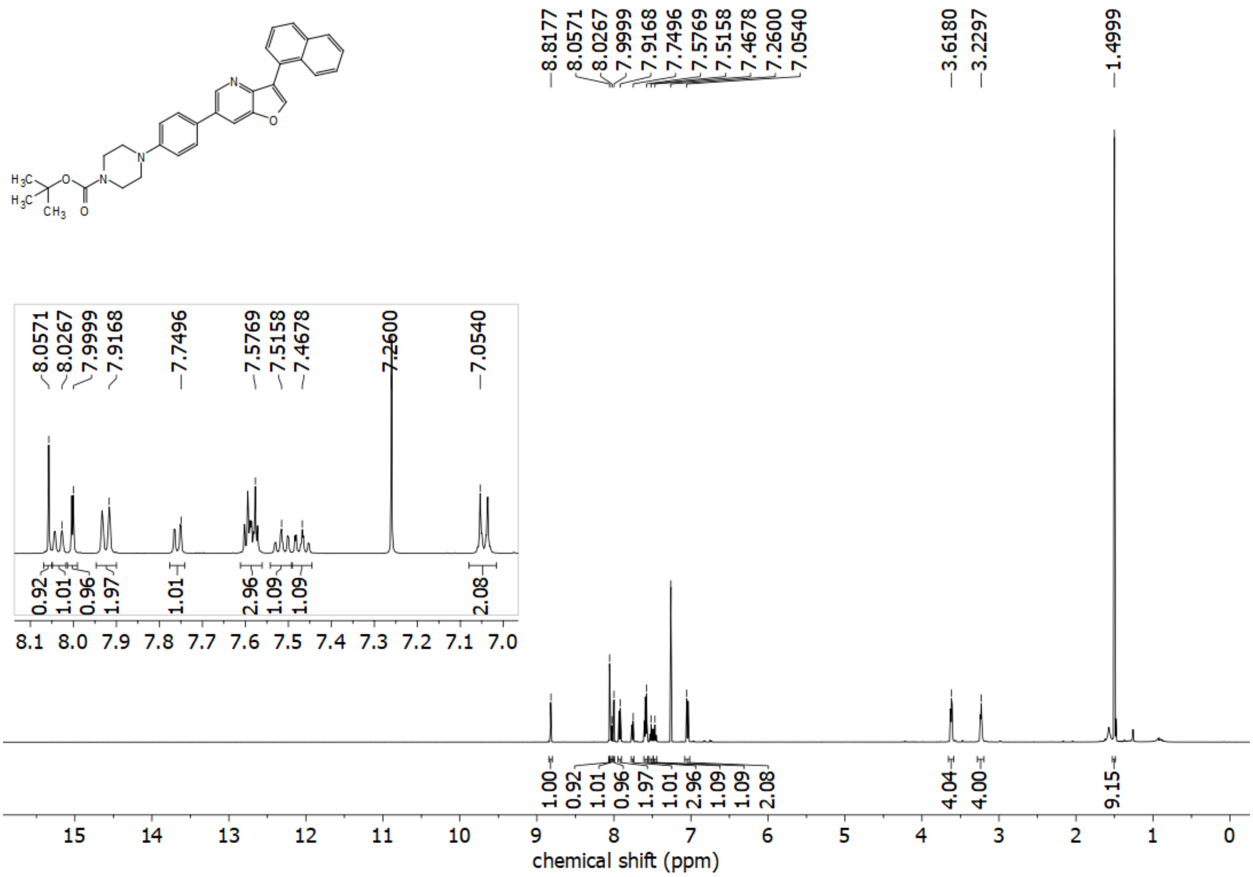

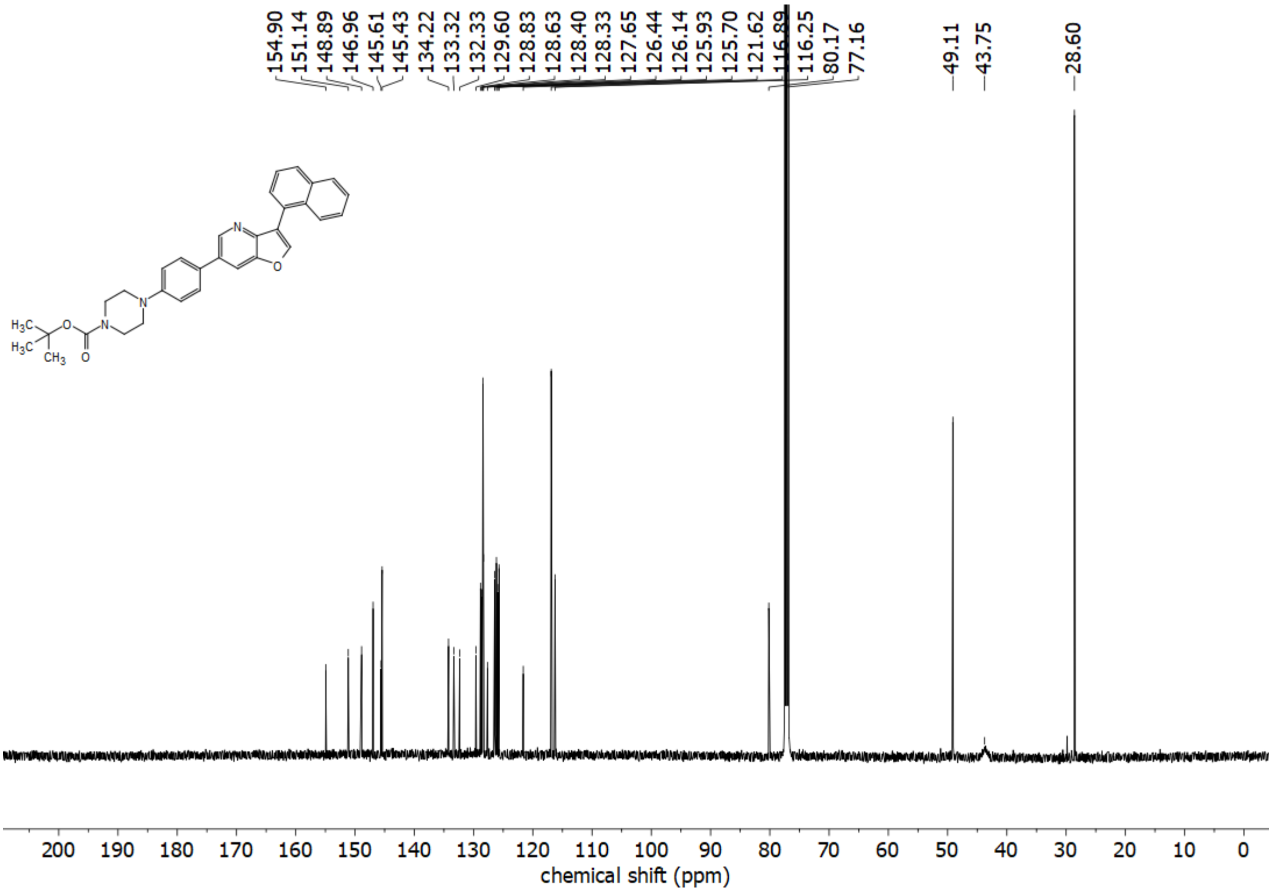

FT-IR spectrum (neat) of **20**.

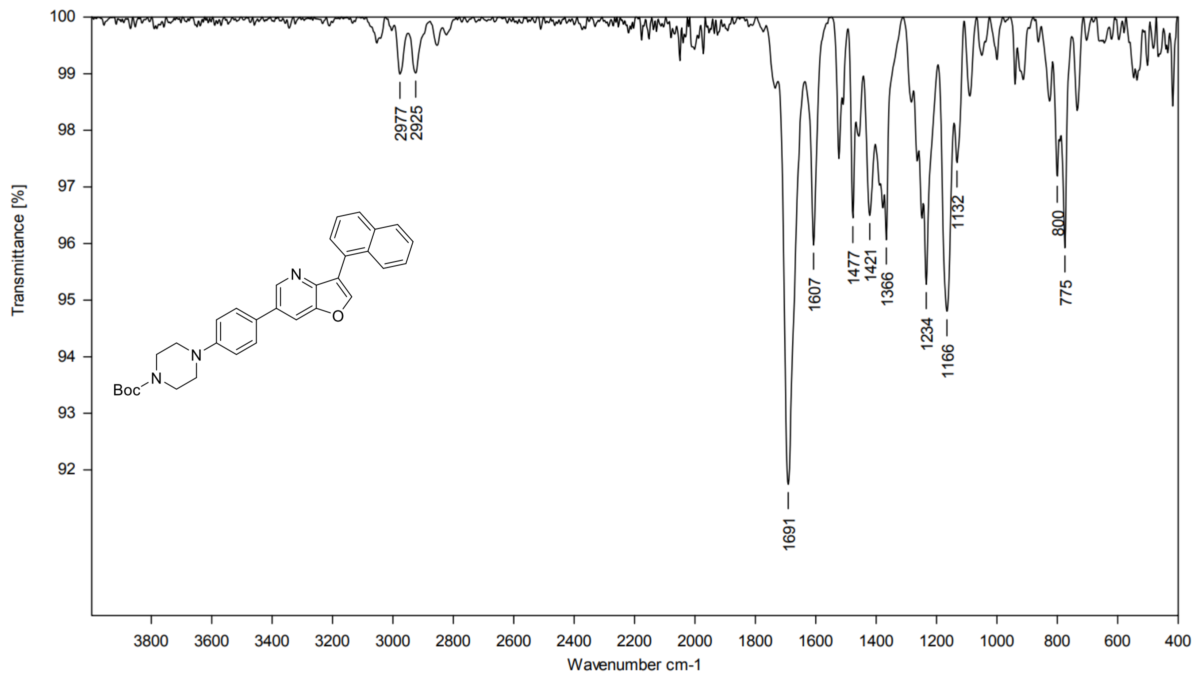

HRMS spectrum of **20**.

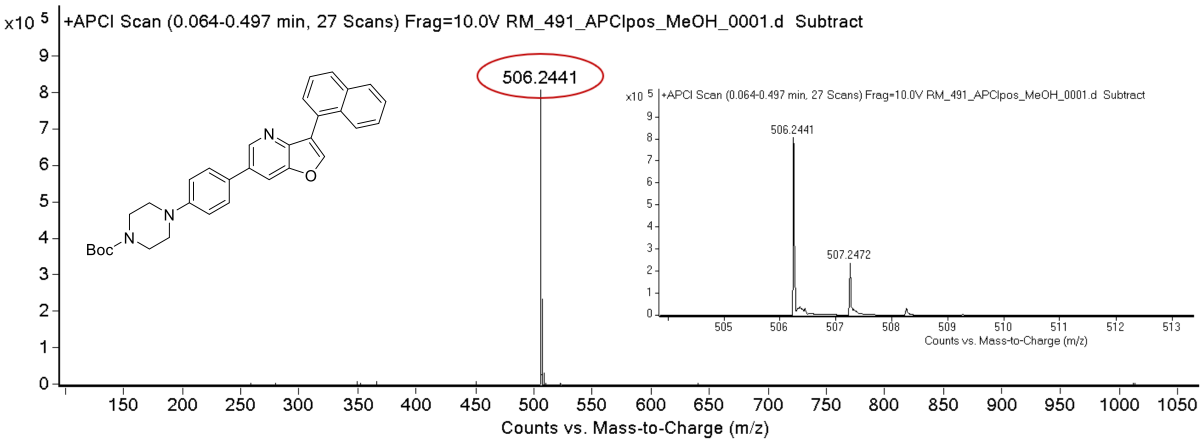

^{1}H (500 MHz) and ^{13}C NMR (126 MHz) spectra of **21** in DMSO-d_6.

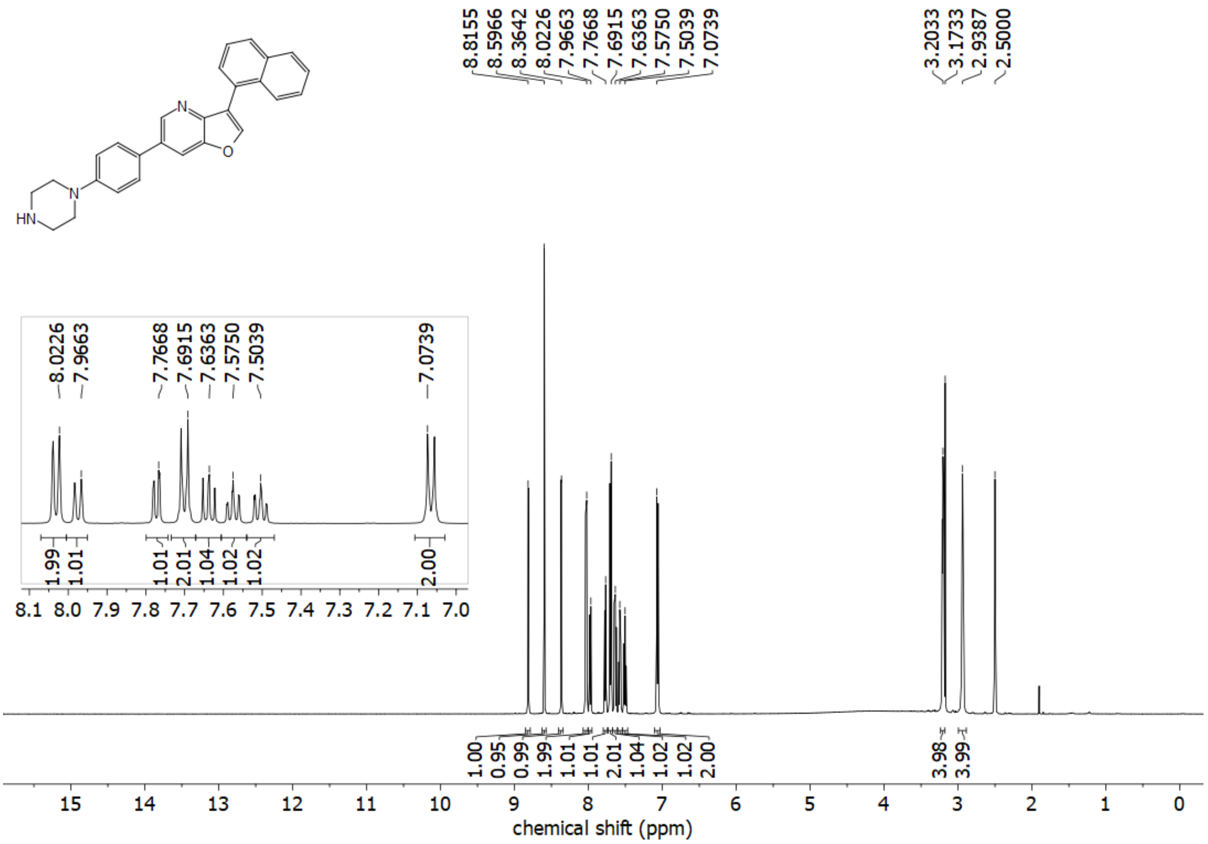

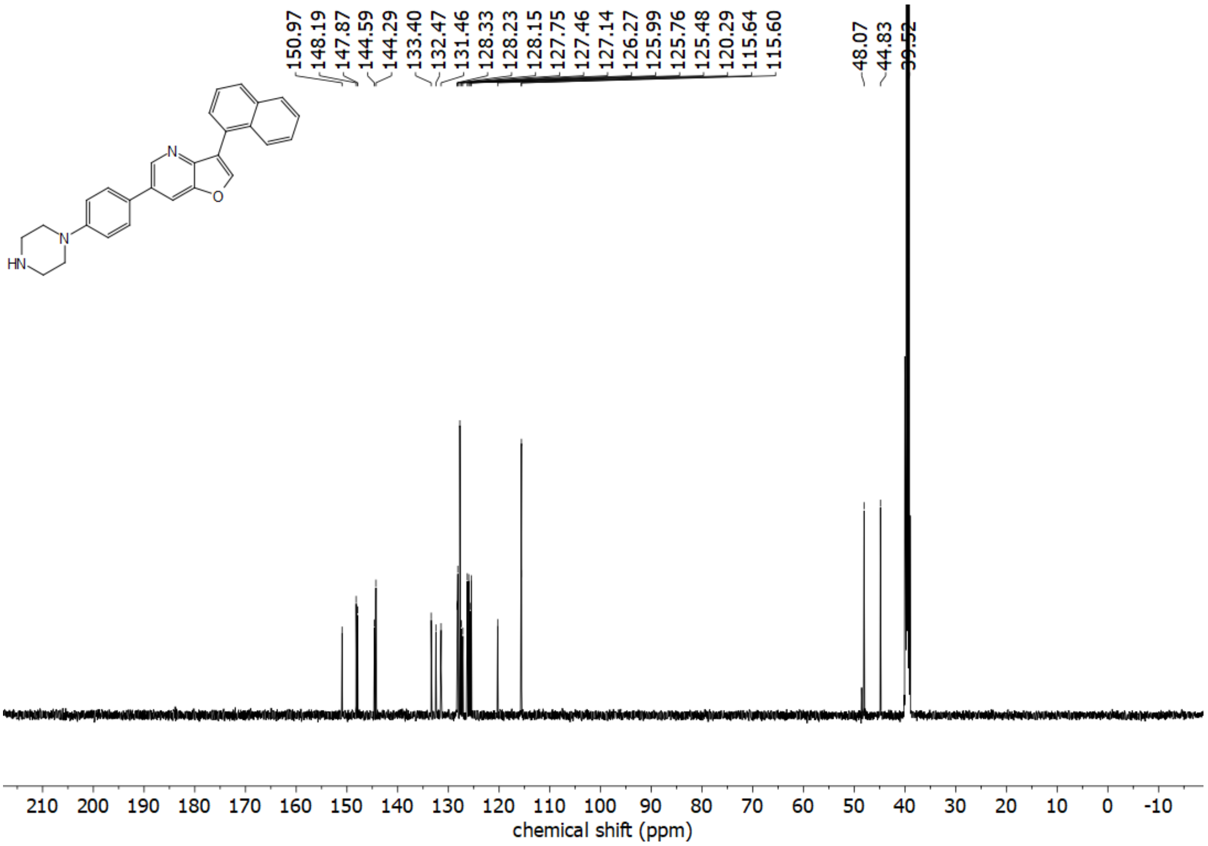

FT-IR spectrum (neat) of **21**.

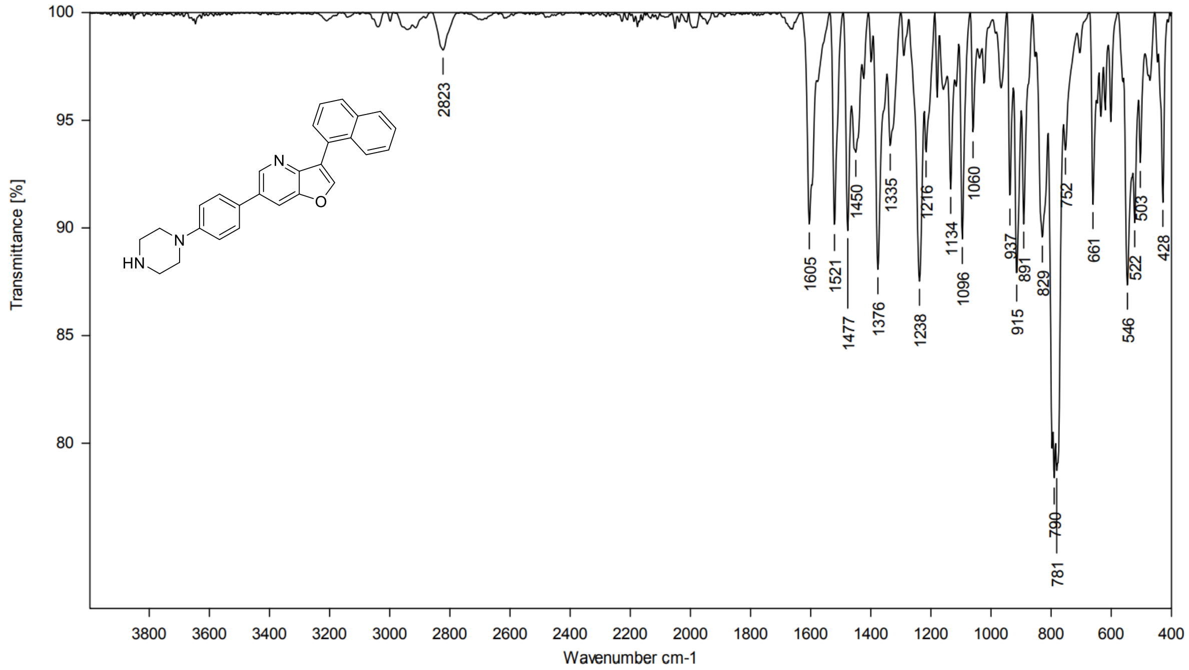

HRMS spectrum of **21**.

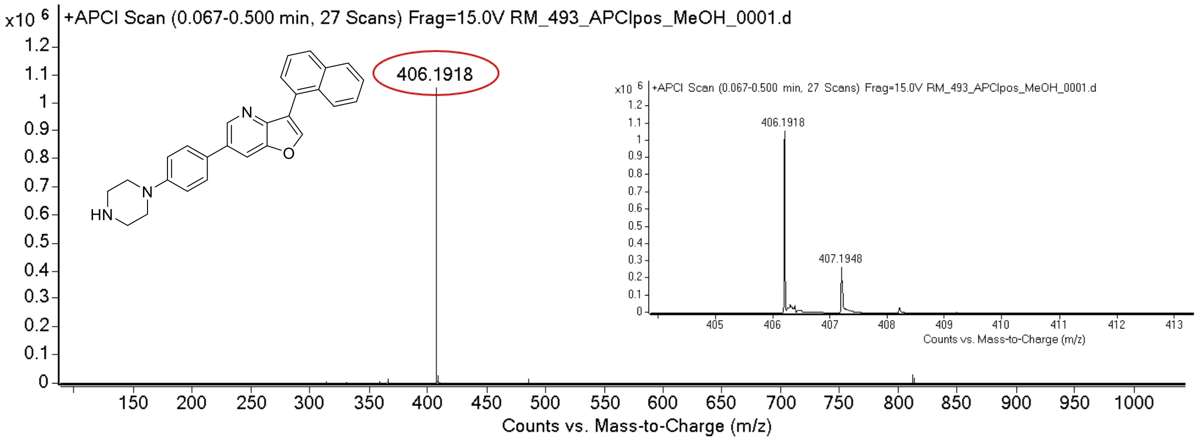

^{1}H (500 MHz) and ^{13}C NMR (126 MHz) spectra of **22** in chloroform-*d*.

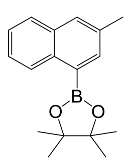

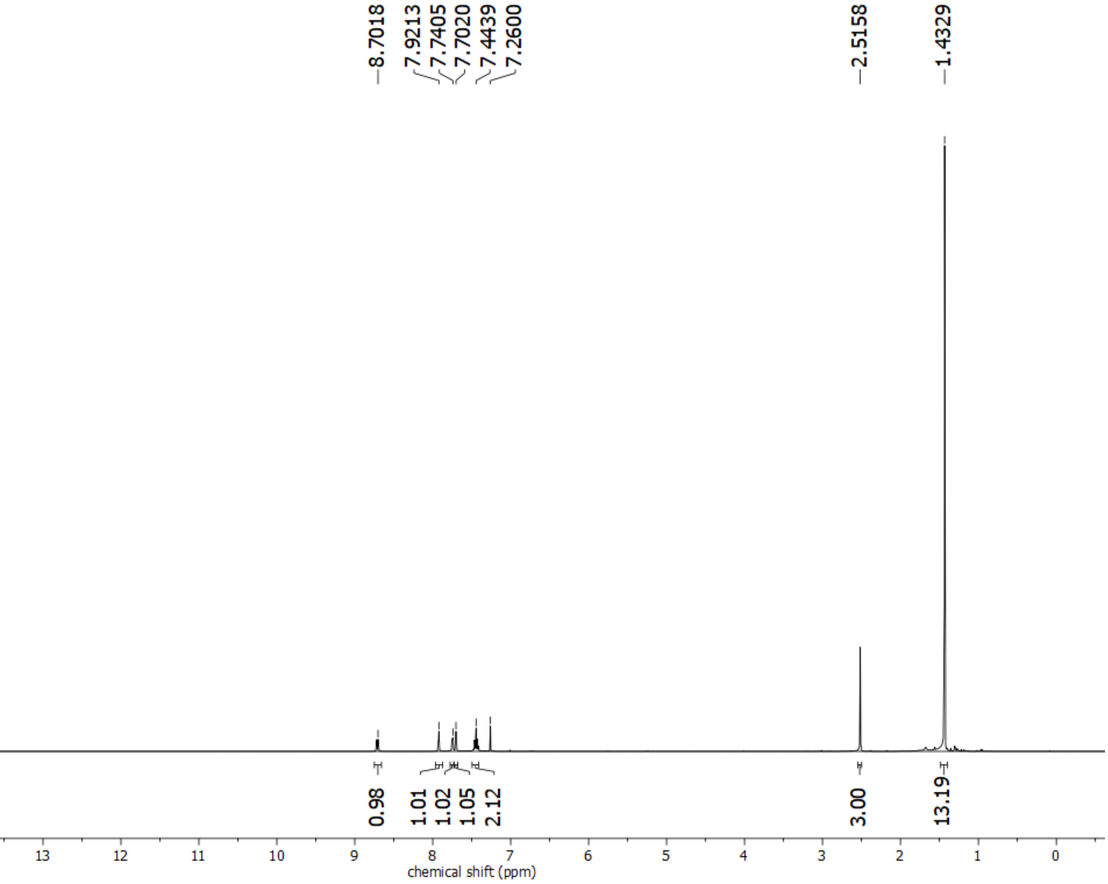

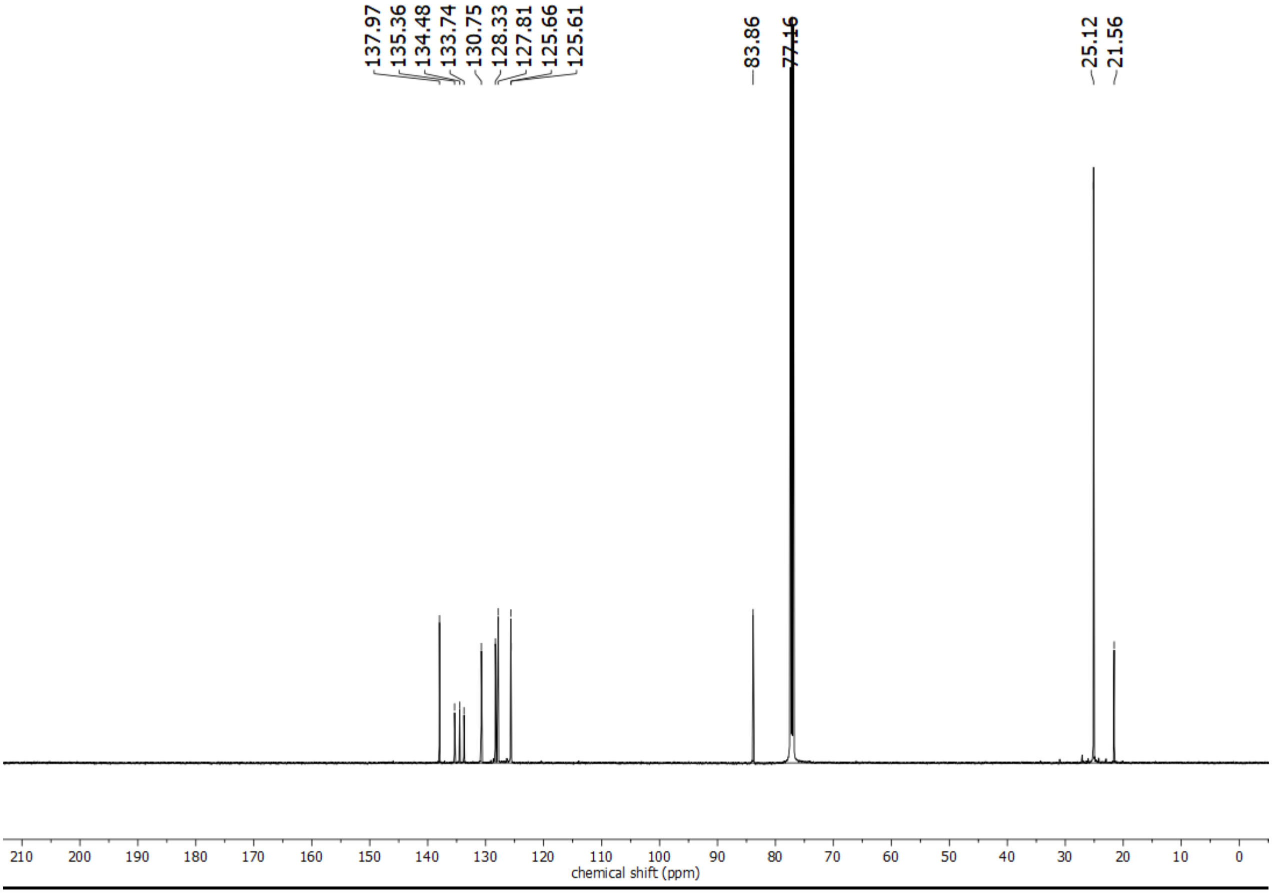

FT-IR spectrum (neat) of **22**.

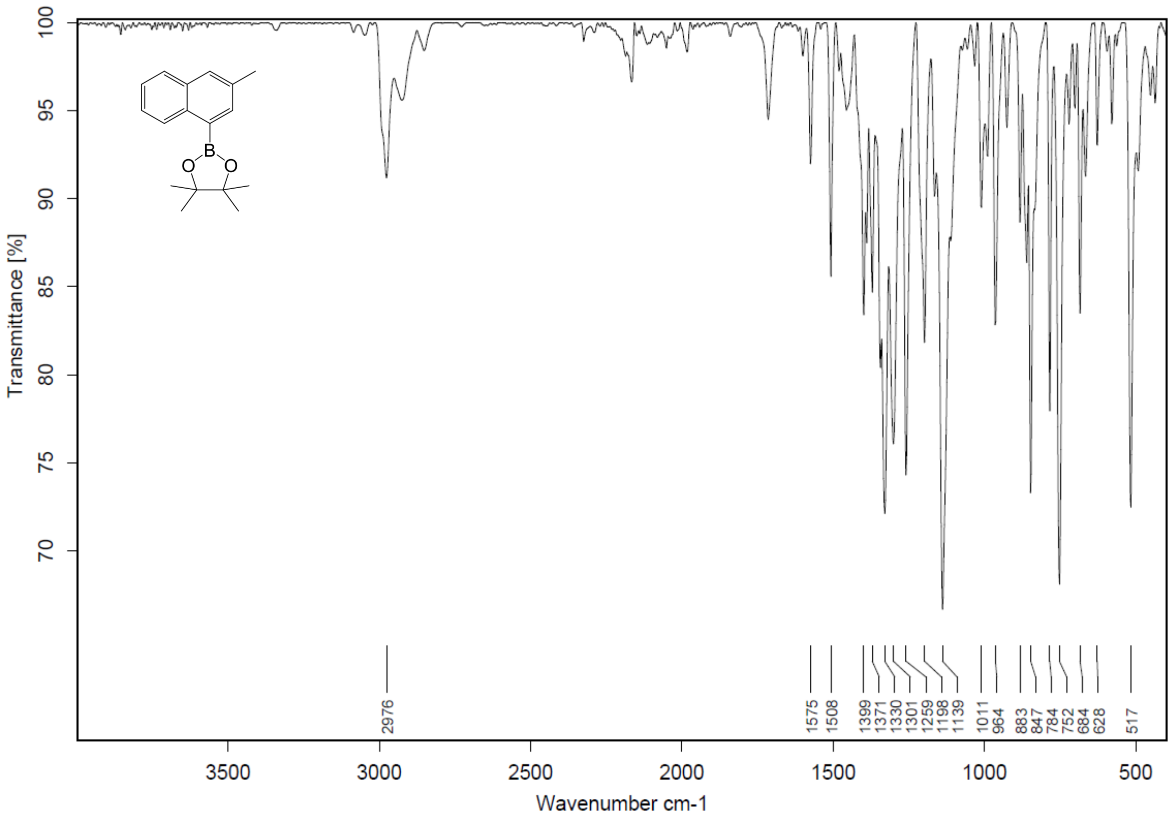

HRMS spectrum of **22**.

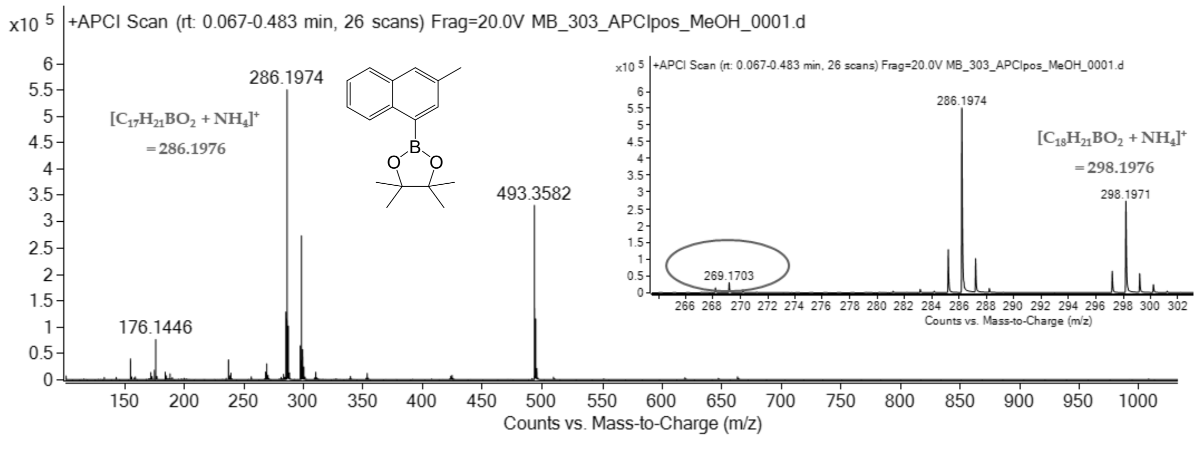

^{1}H (500 MHz) and ^{13}C NMR (126 MHz) spectra of **23** in chloroform-*d*.

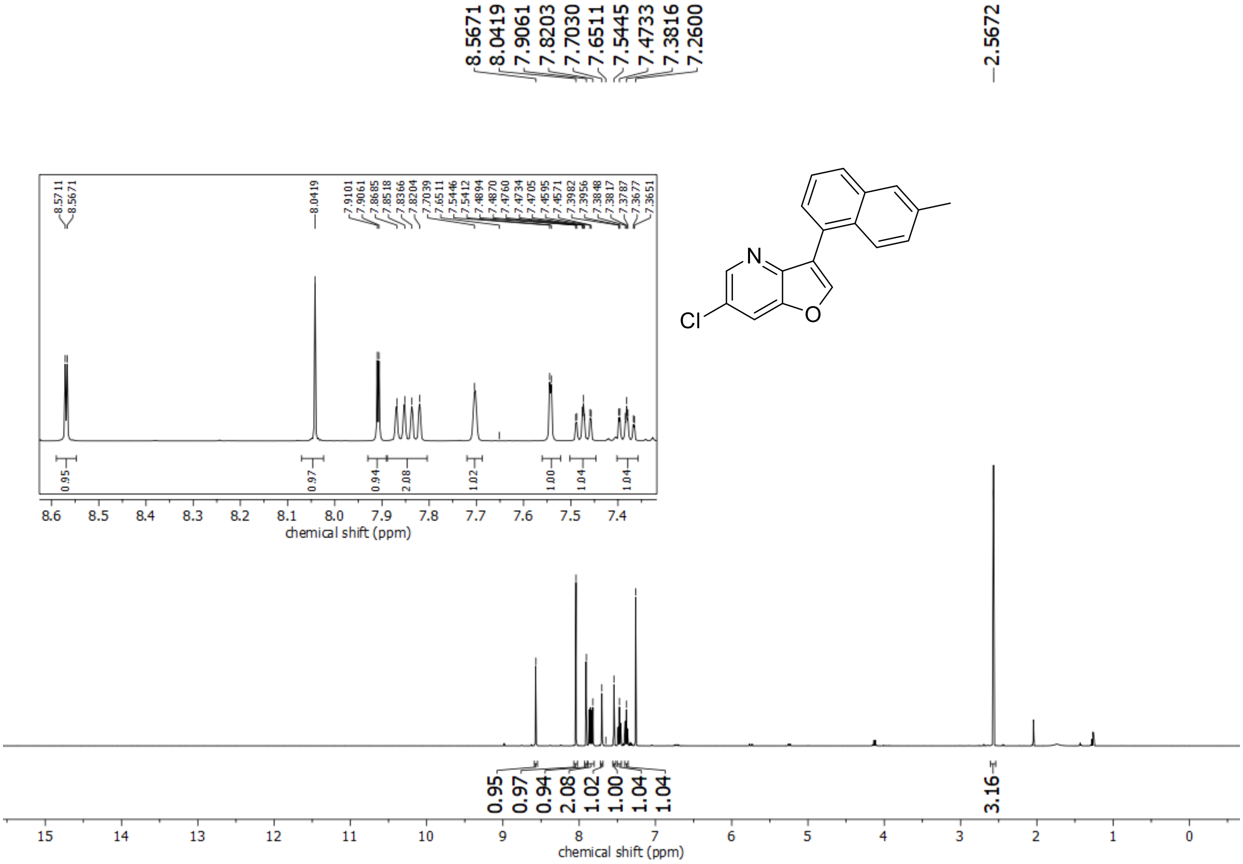

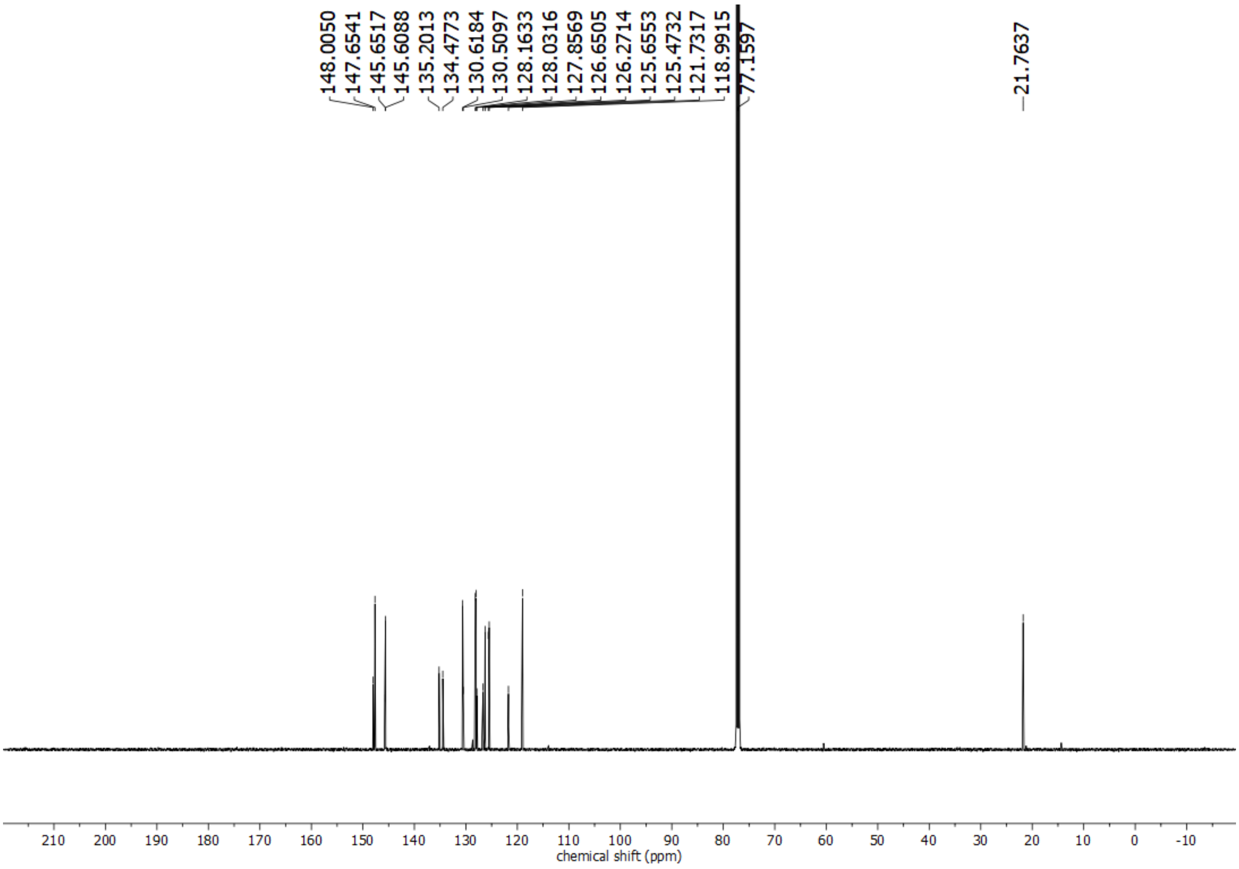

FT-IR spectrum (neat) of **23**.

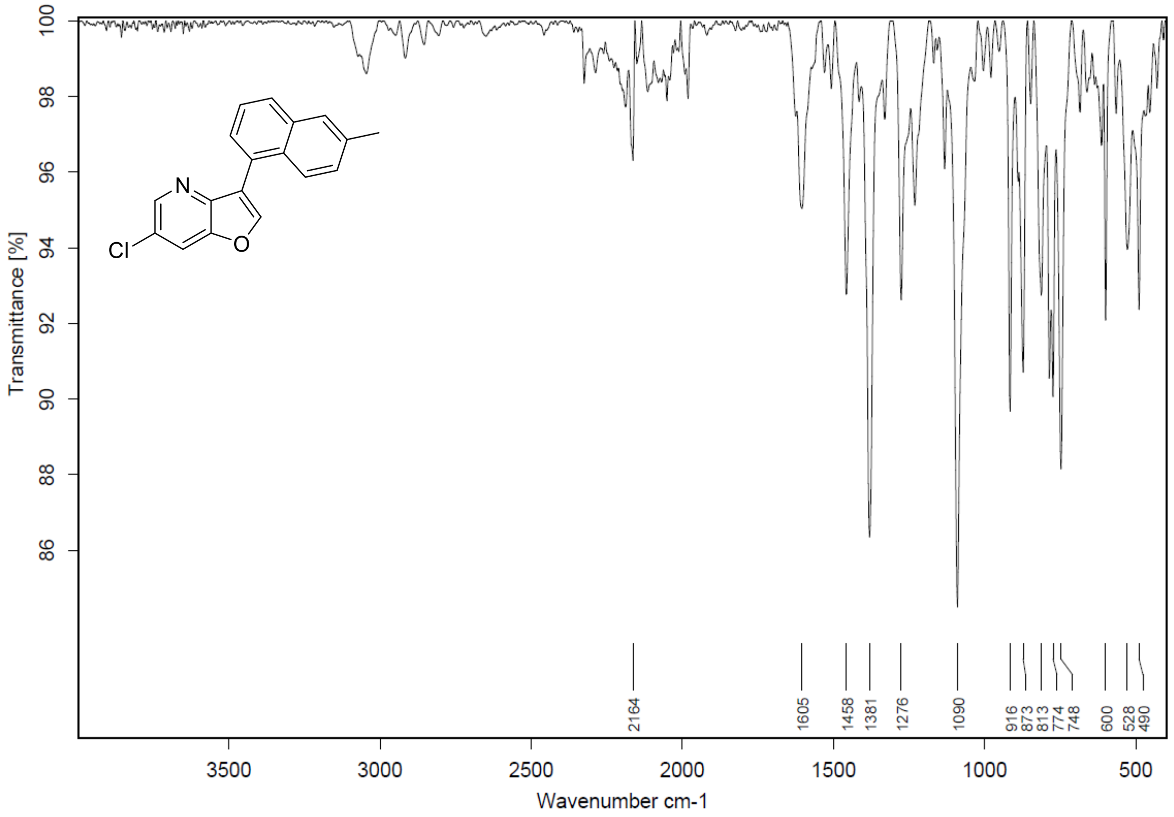

HRMS spectrum of **23**.

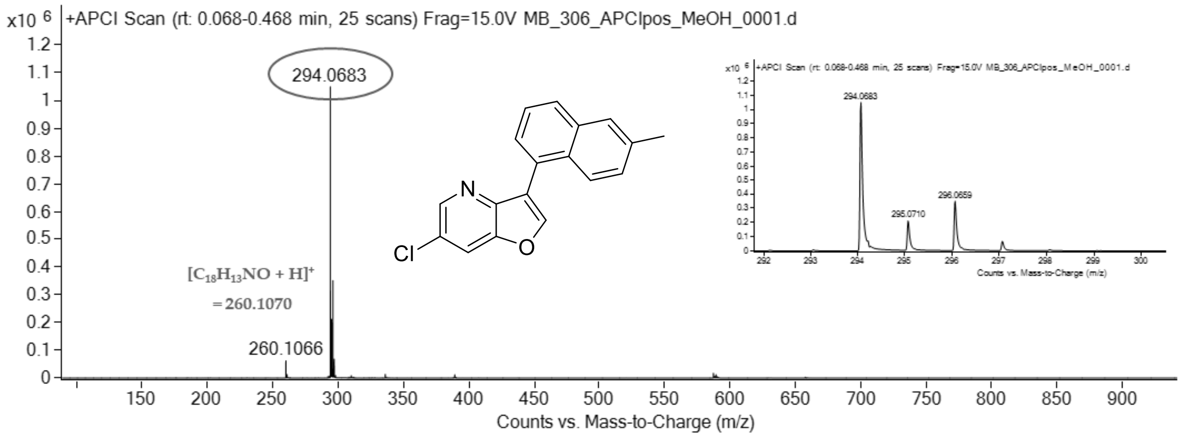

^{1}H (500 MHz) and ^{13}C NMR (126 MHz) spectra of **24** in chloroform-*d*.

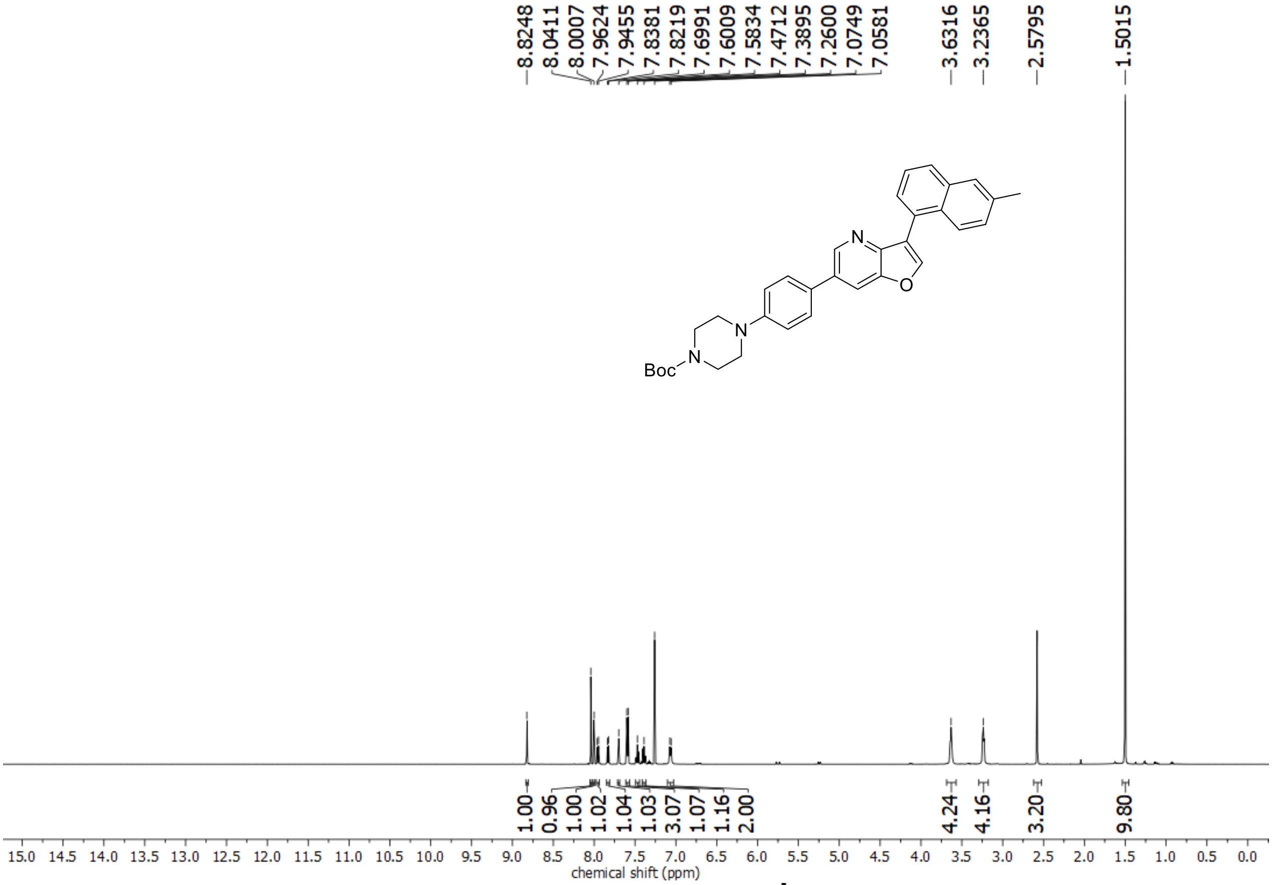

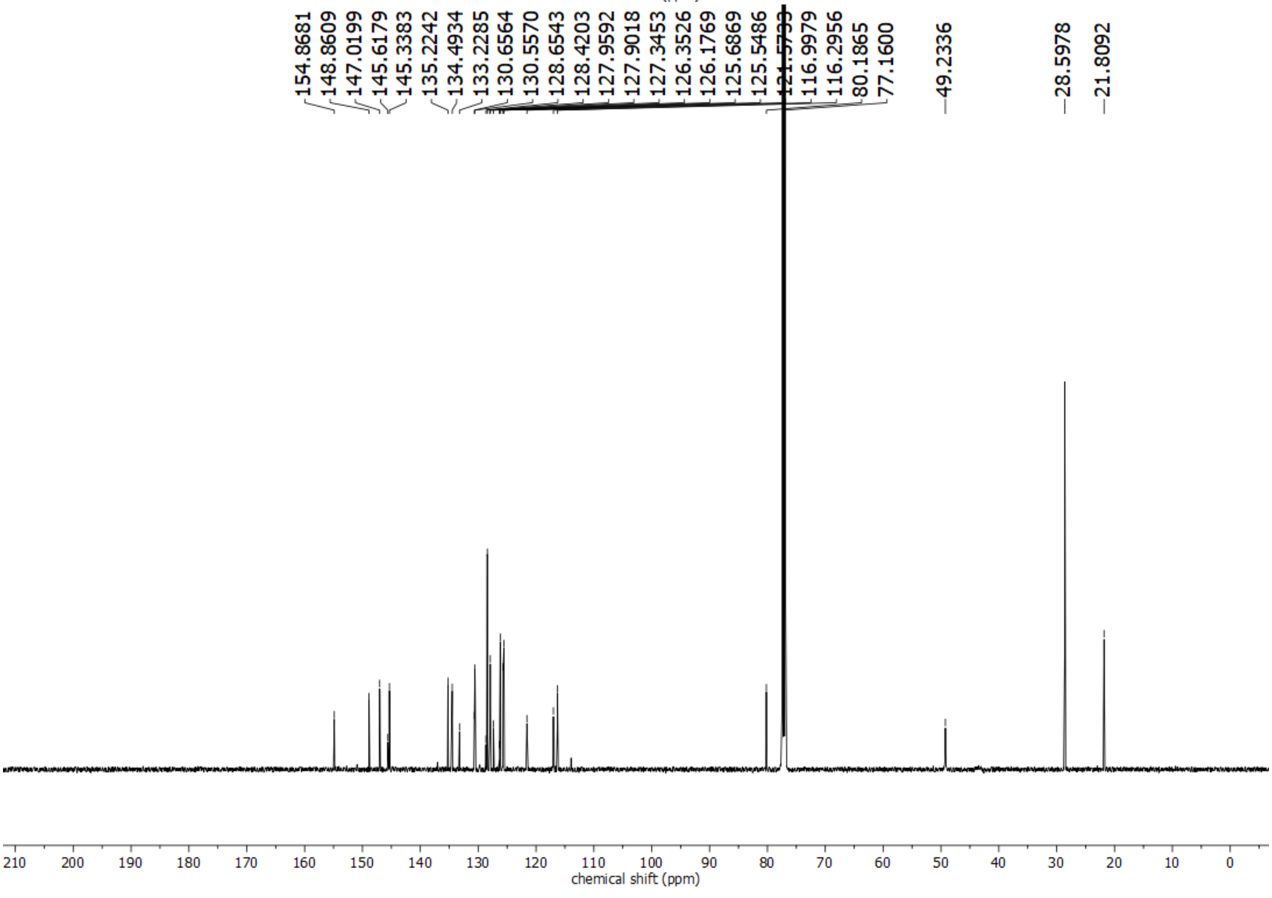

FT-IR spectrum (neat) of **24**.

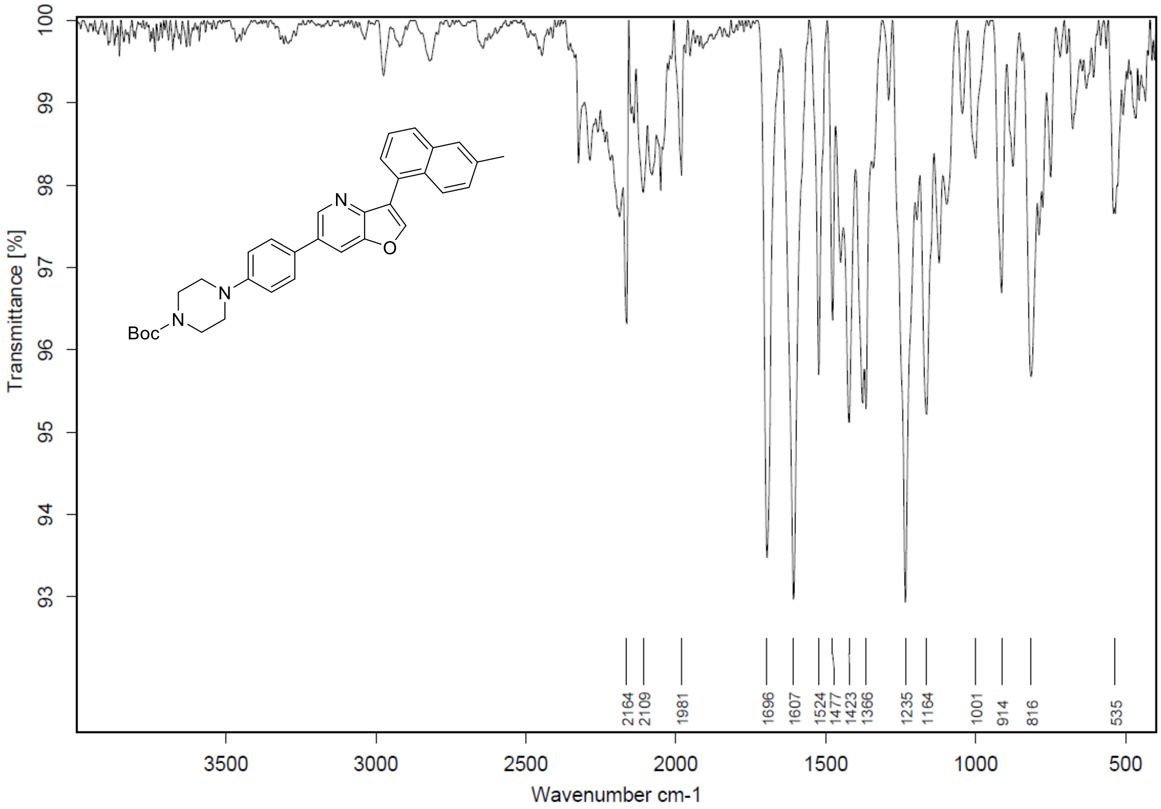

HRMS spectrum of **24.**

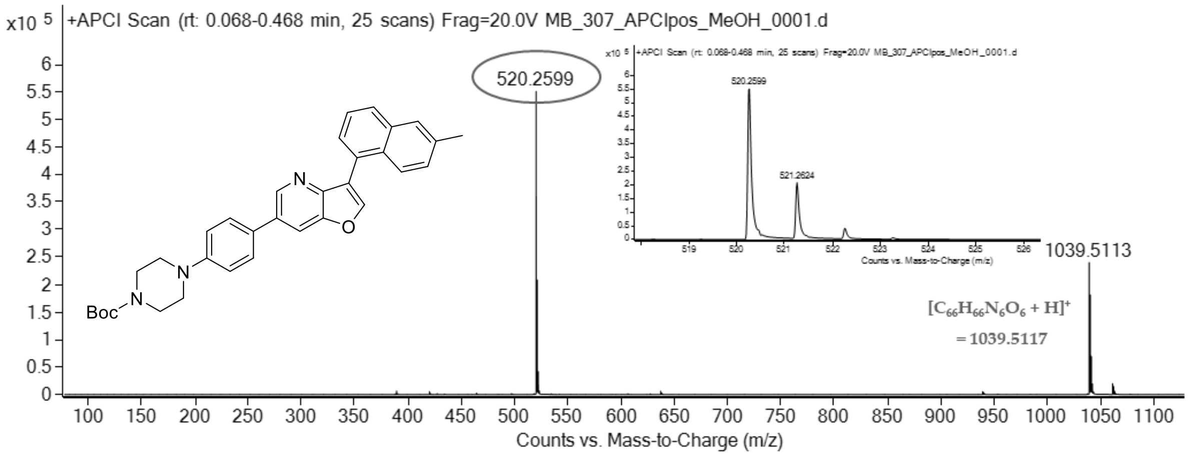

^{1}H (500 MHz) and ^{13}C NMR (126 MHz) spectra of **25** in chloroform-*d*.

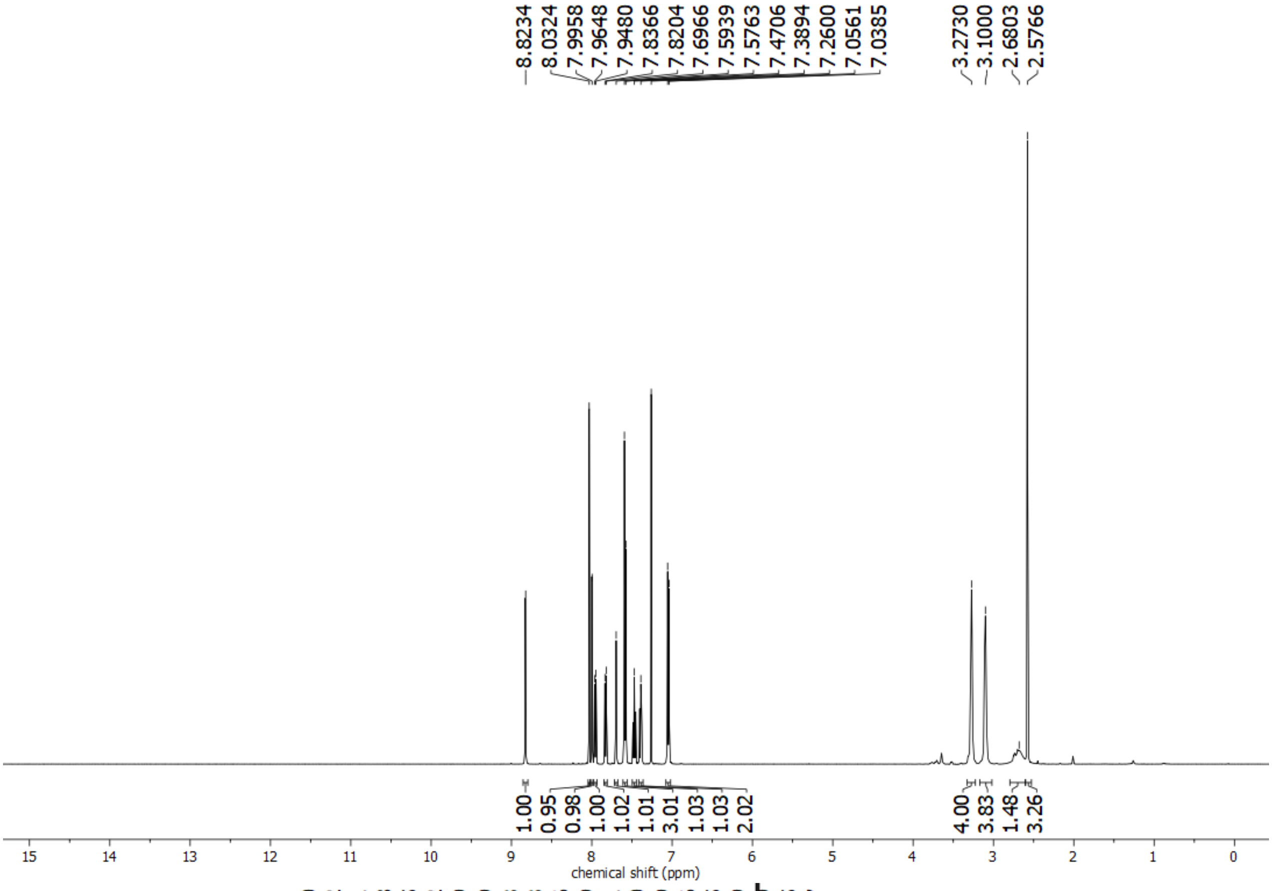

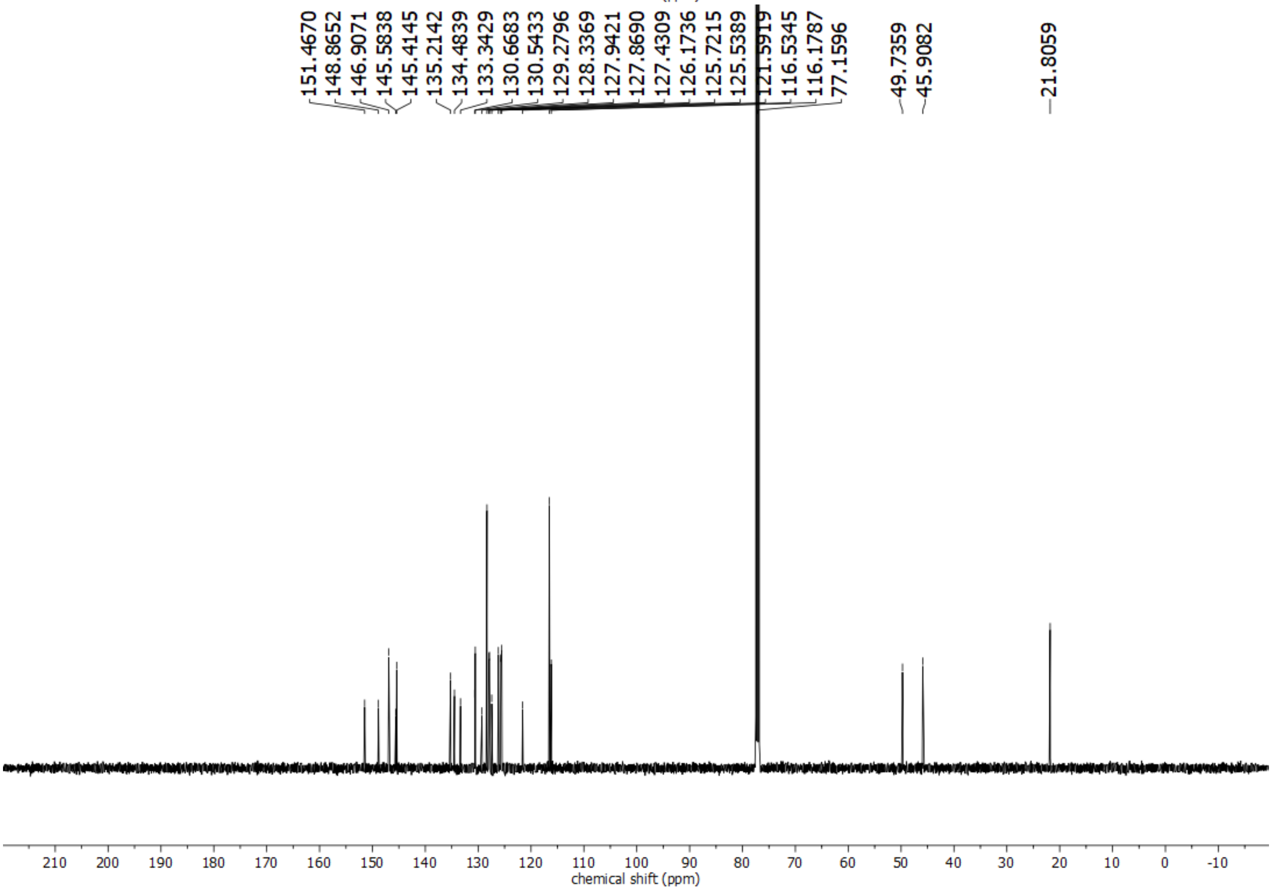

FT-IR spectrum (neat) of **25**.

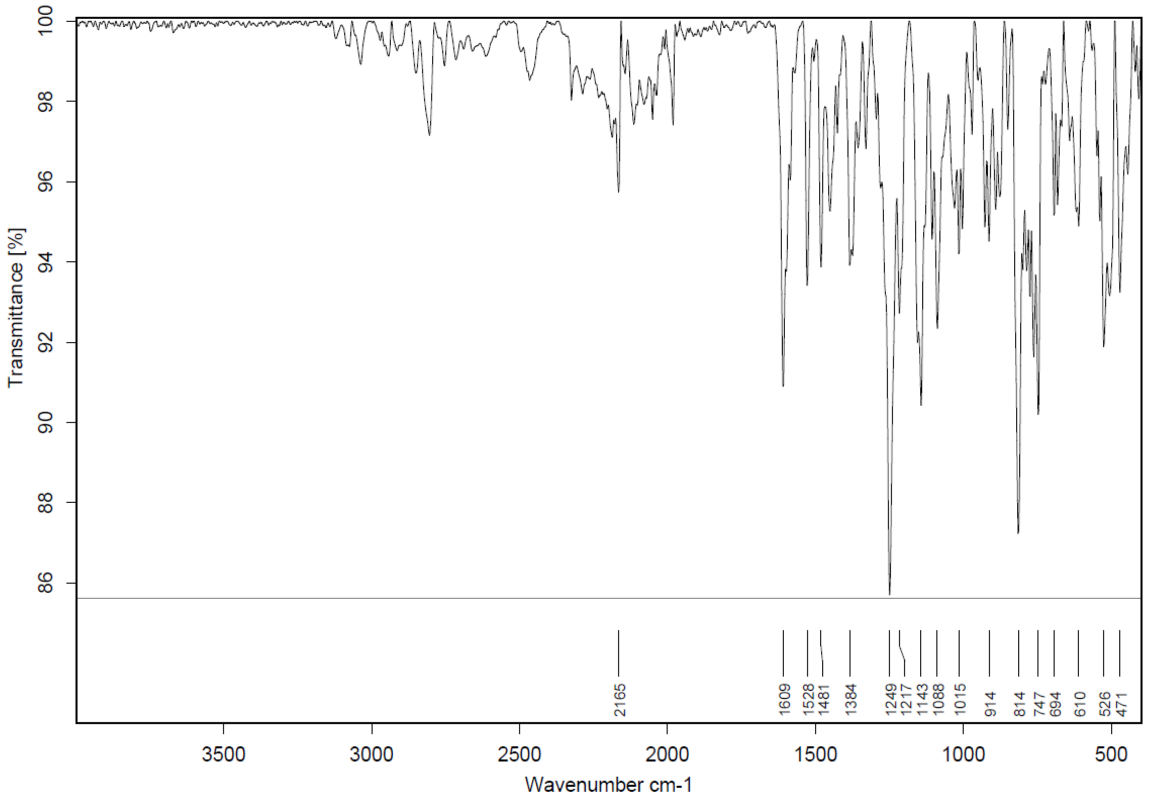

HRMS spectrum of **25.**

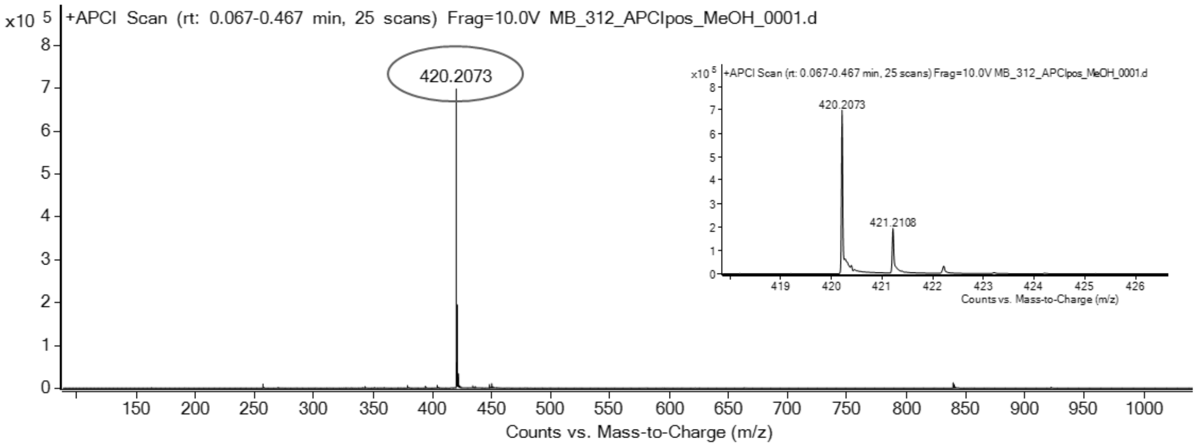

^{1}H (500 MHz) and ^{13}C NMR (126 MHz) spectra of **26** in chloroform-*d*.

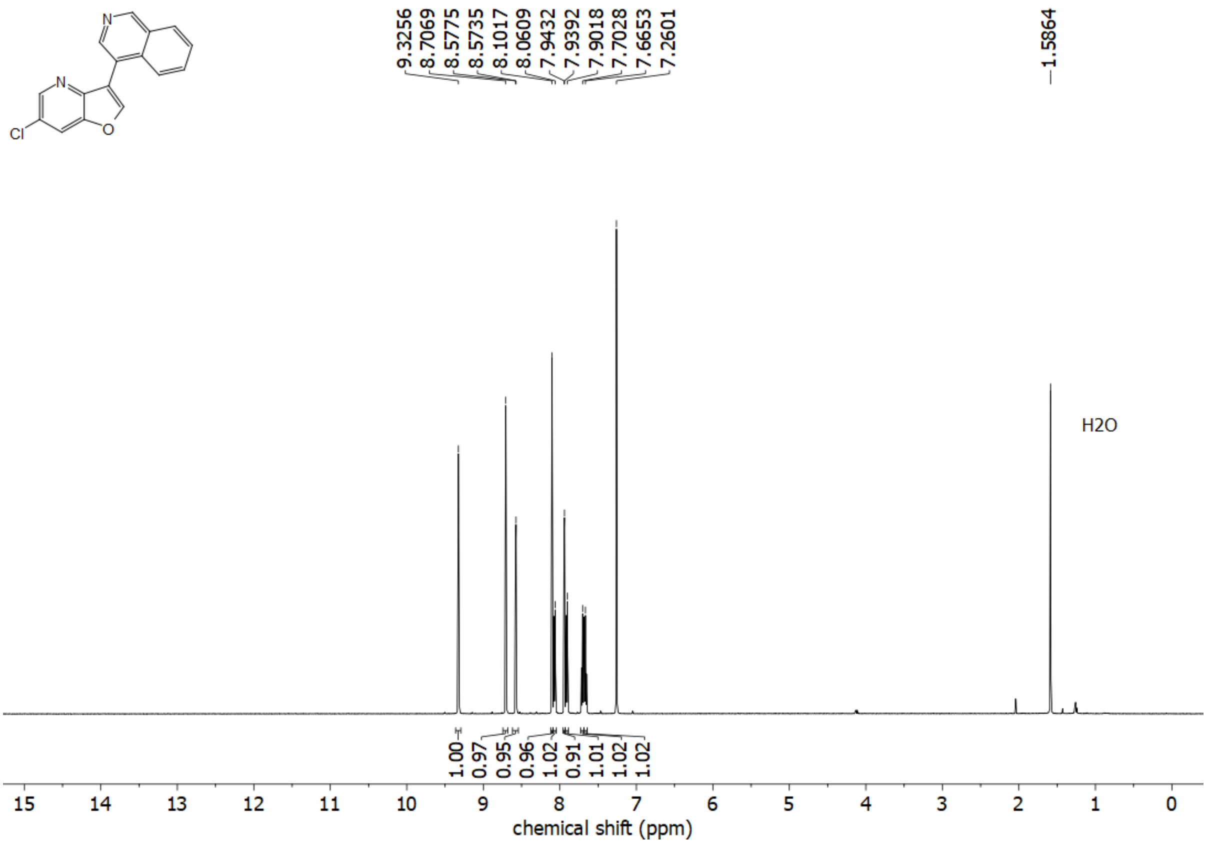

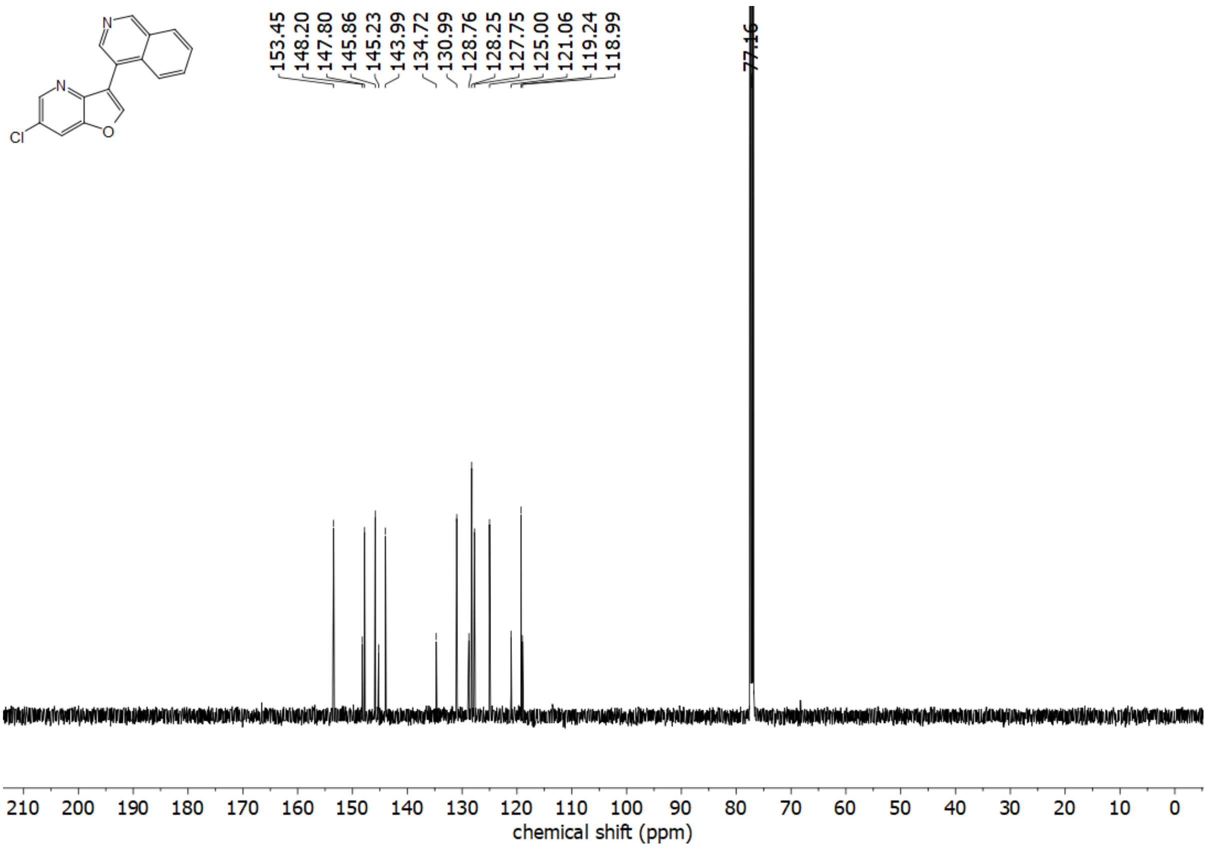

FT-IR spectrum (neat) of **26**.

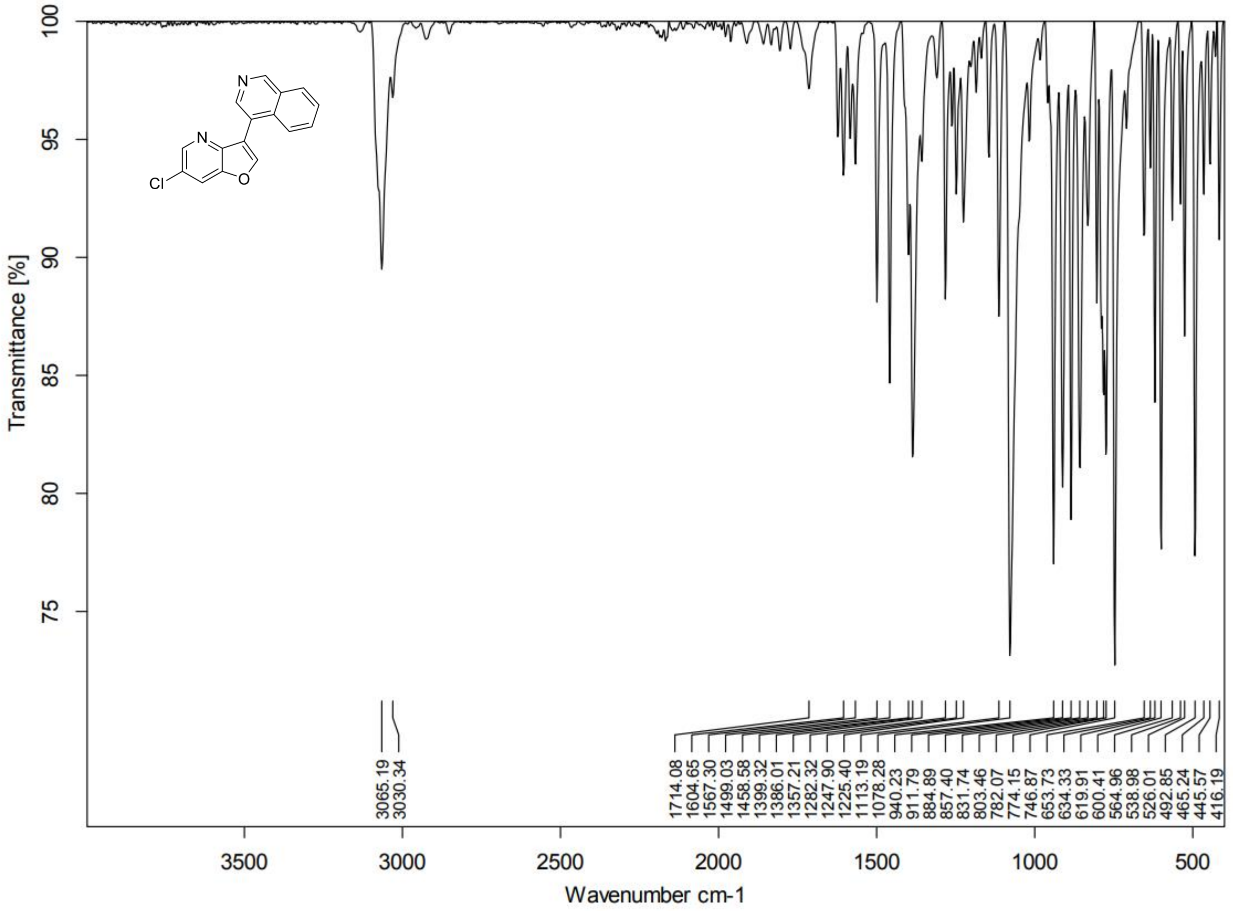

HRMS spectrum of **26**.

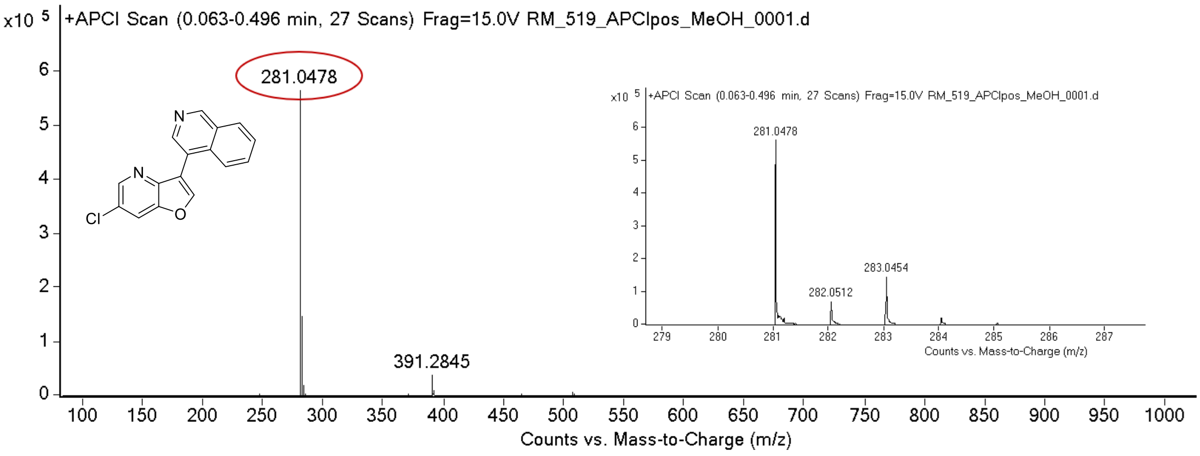

^{1}H (500 MHz) and ^{13}C NMR (126 MHz) spectra of **27** in chloroform-*d*.

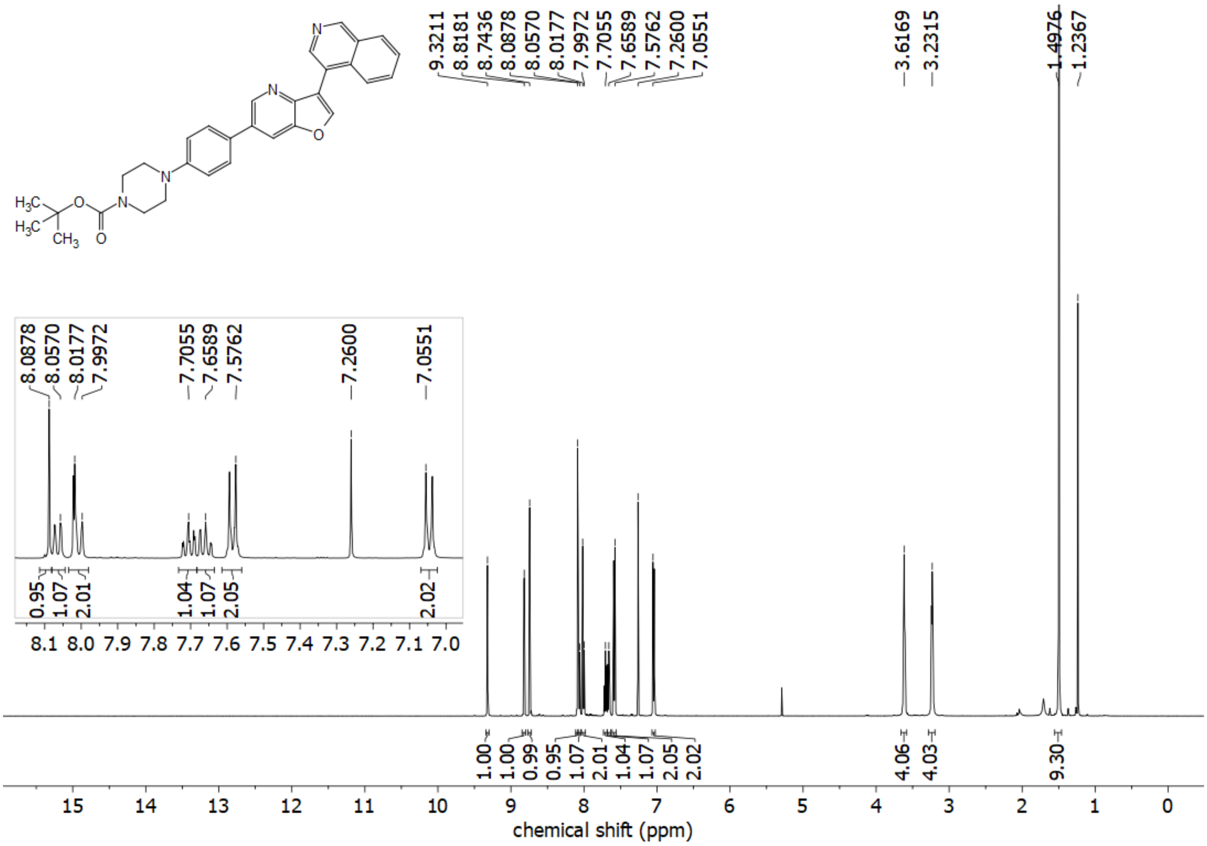

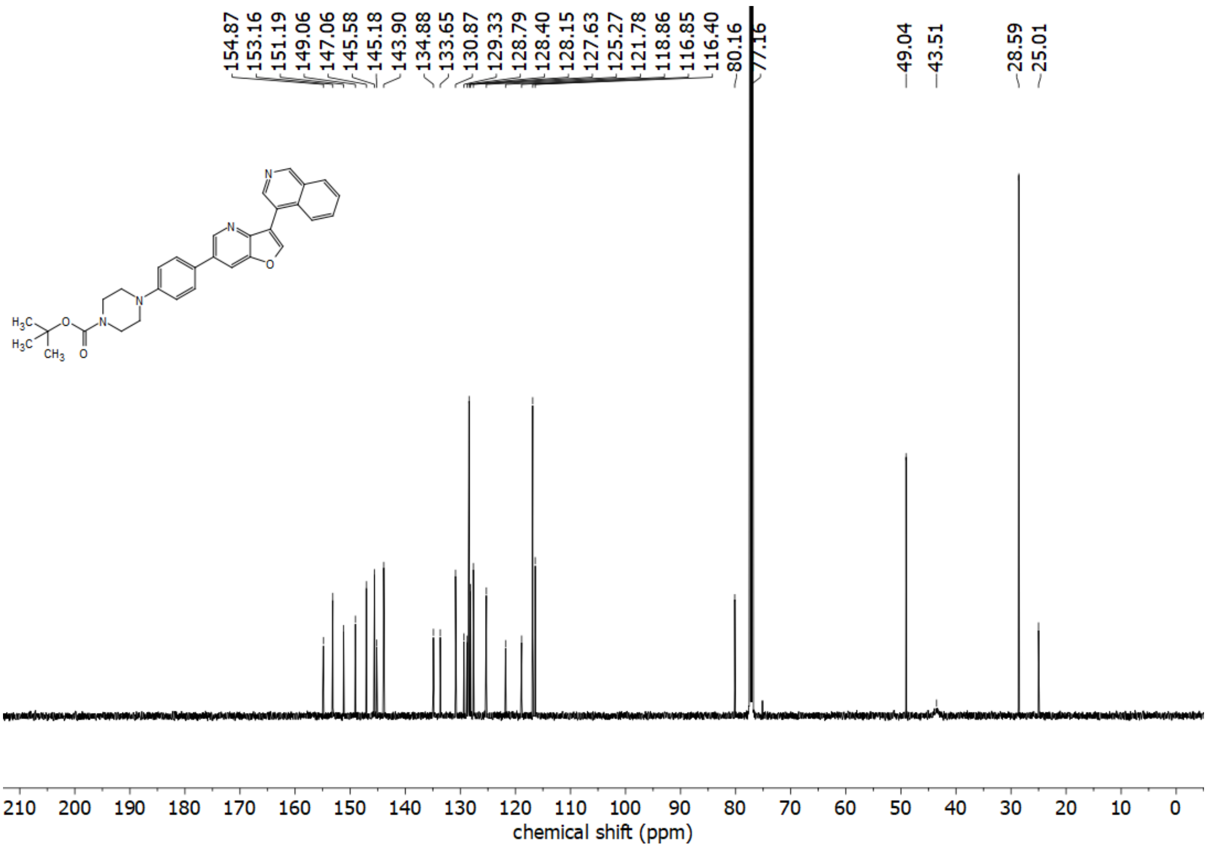

FT-IR spectrum (neat) of **27**.

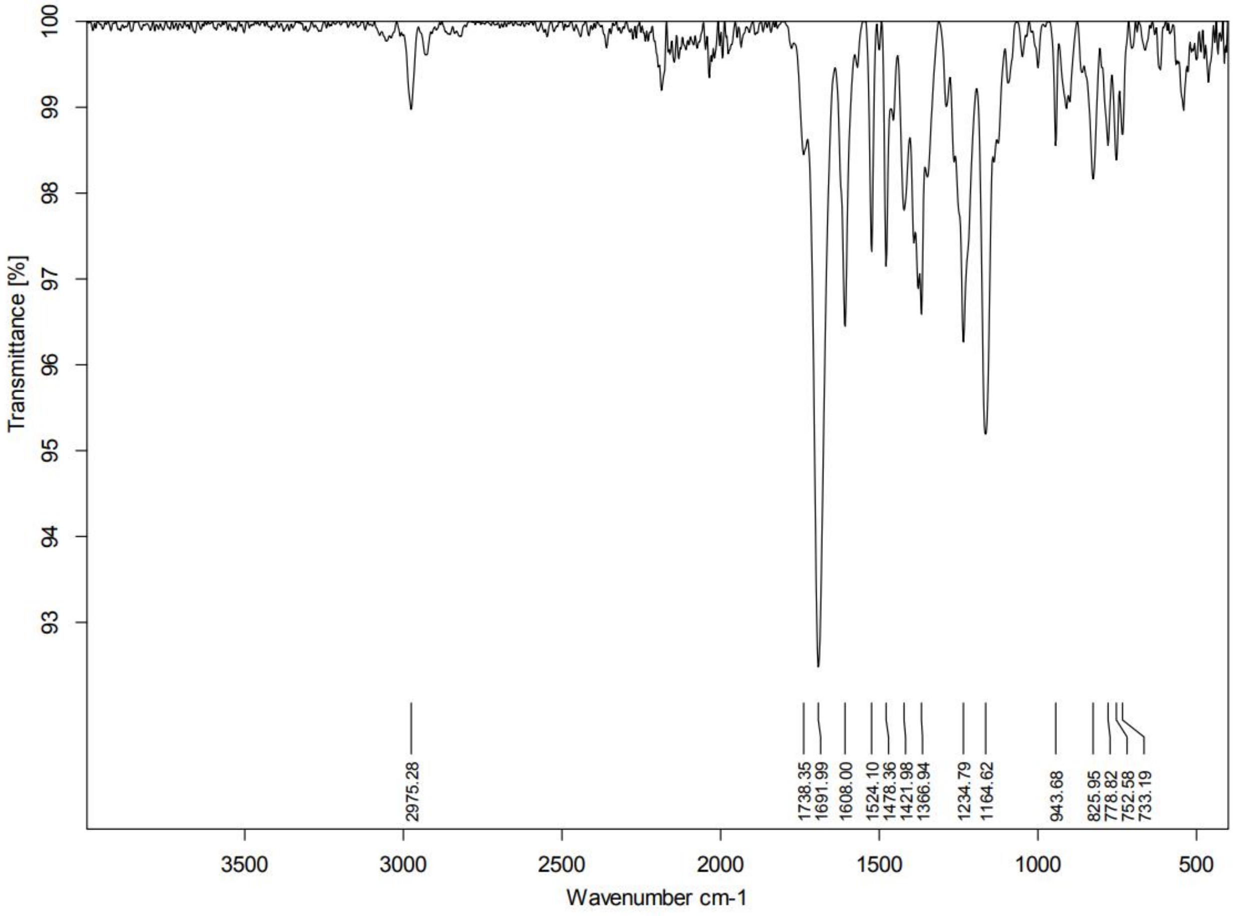

HRMS spectrum of **27**.

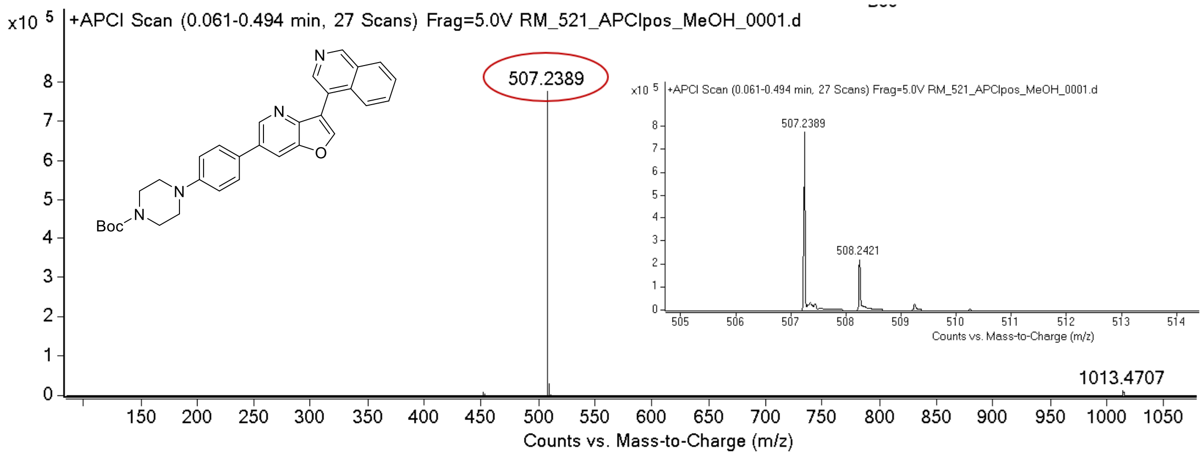

^{1}H (500 MHz) and ^{13}C NMR (126 MHz) spectra of **28** in methanol-d_4.

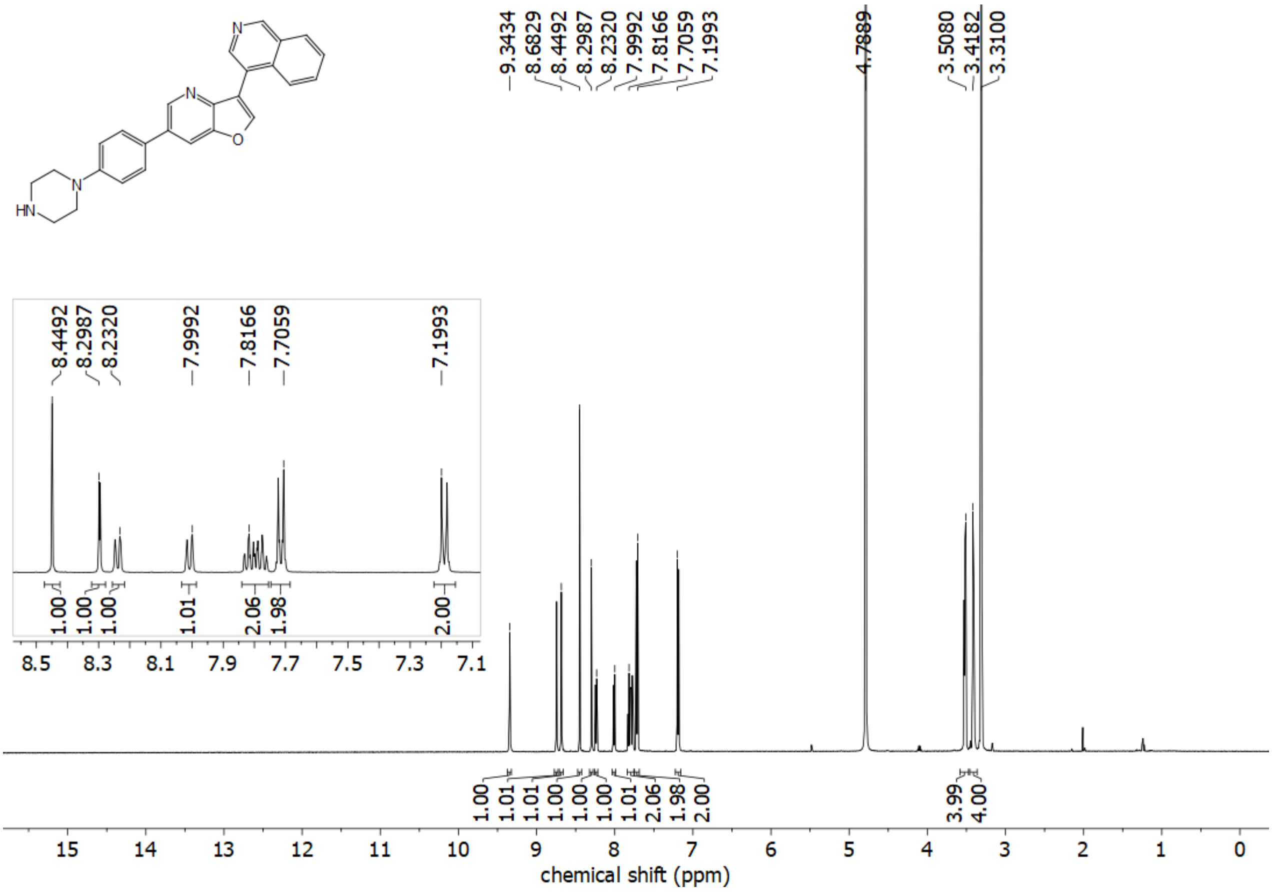

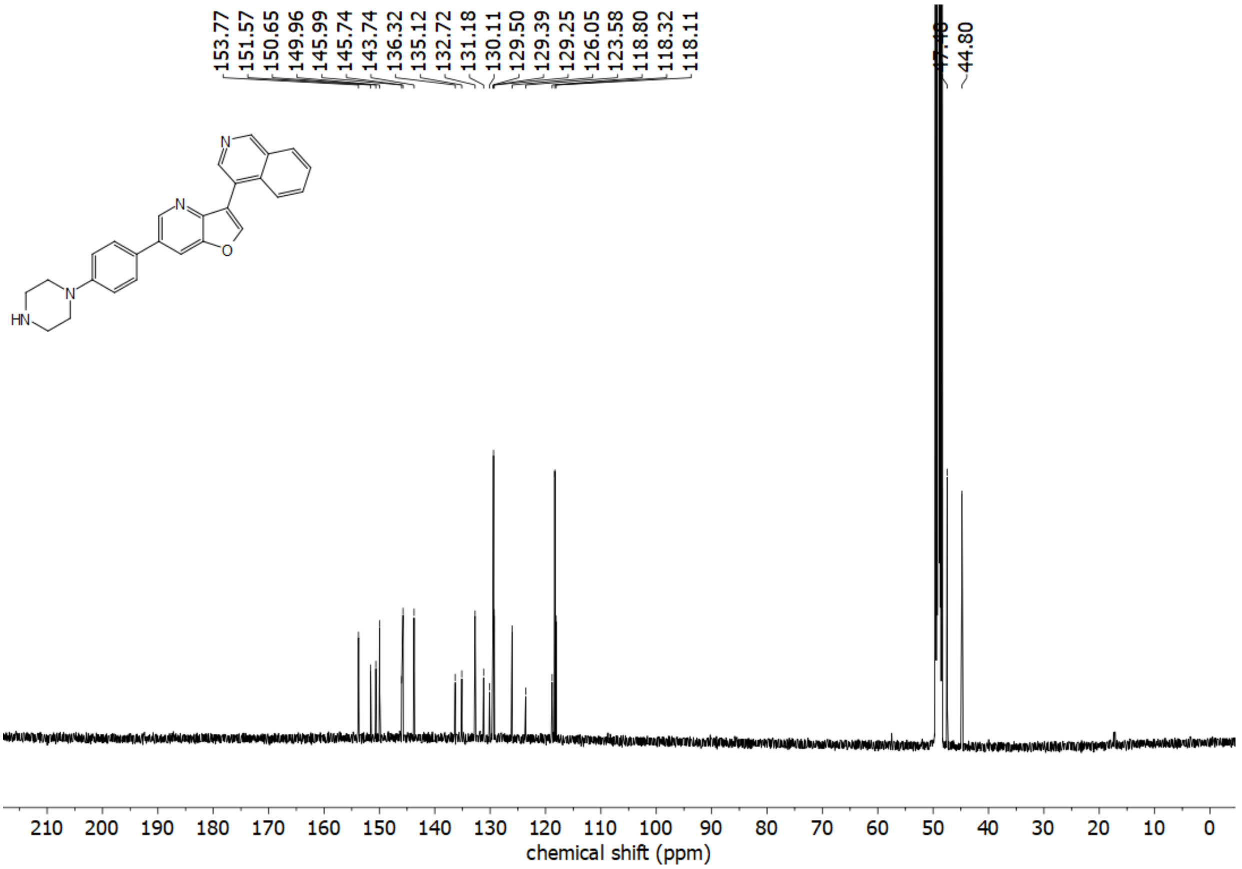

FT-IR spectrum (neat) of **28**.

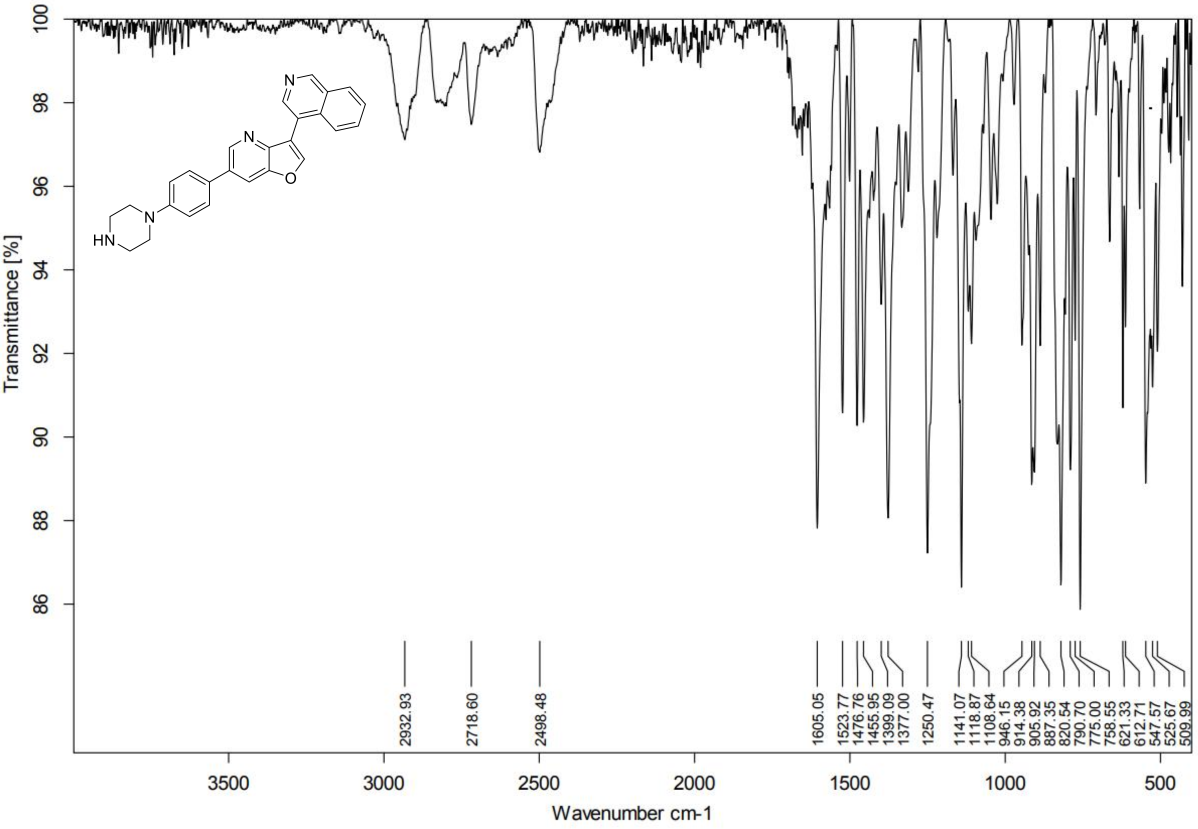

HRMS spectrum of **28**.

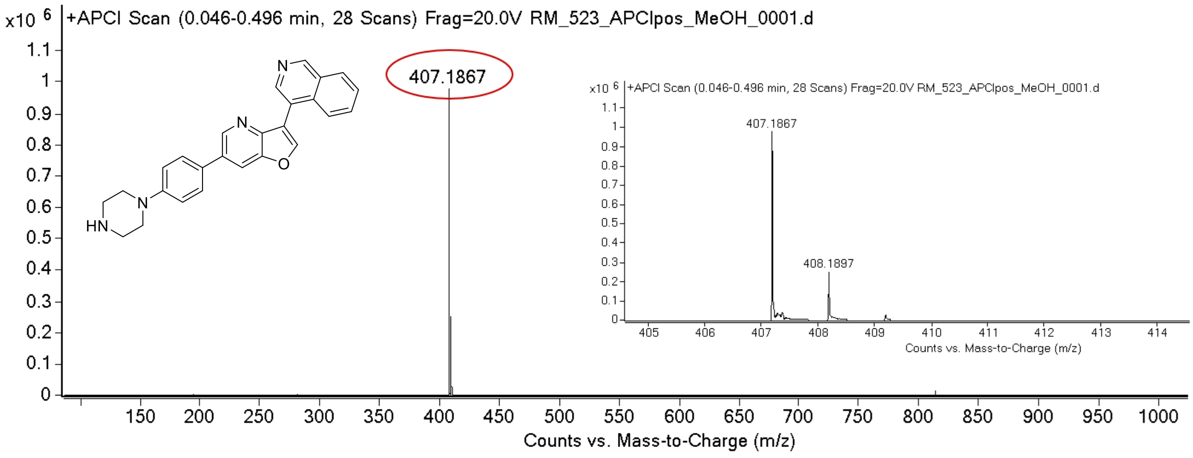

^{1}H (500 MHz) and ^{13}C NMR (126 MHz) spectra of **29** in chloroform-*d*.

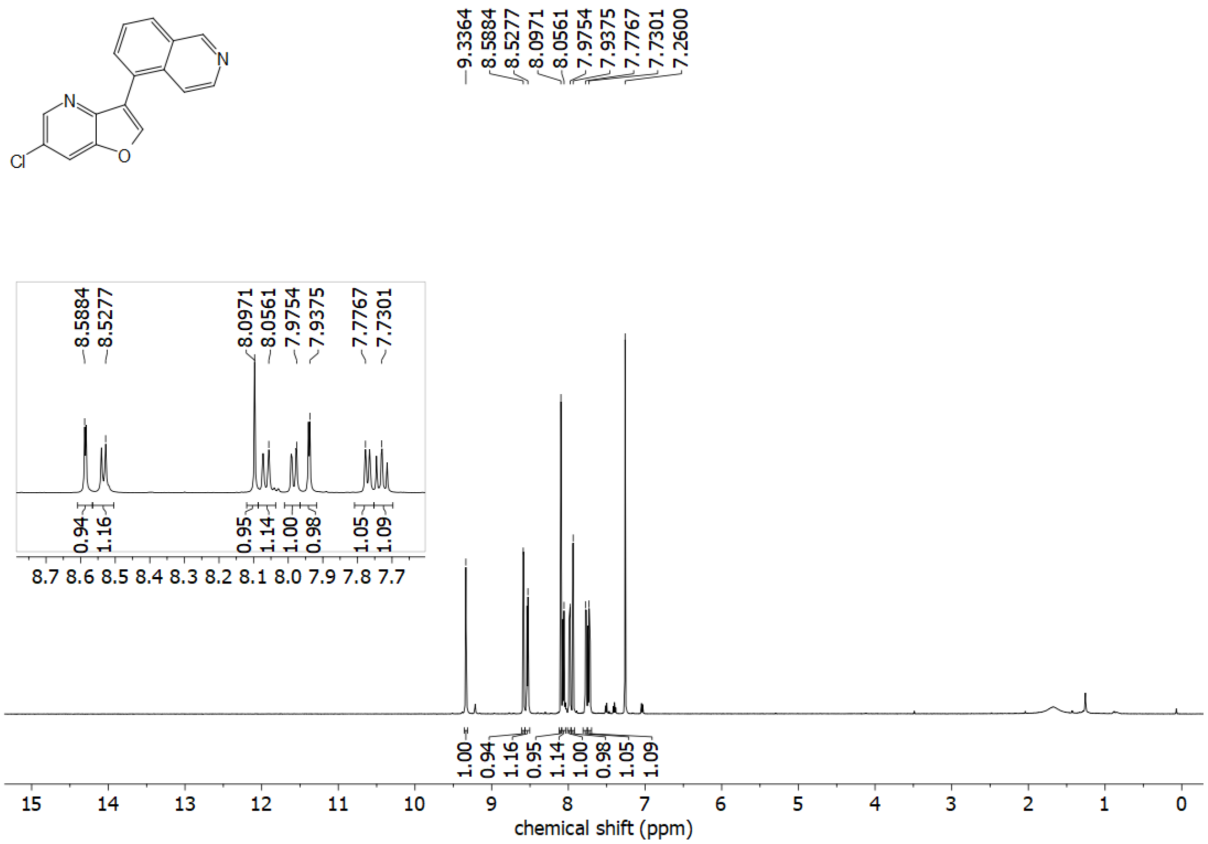

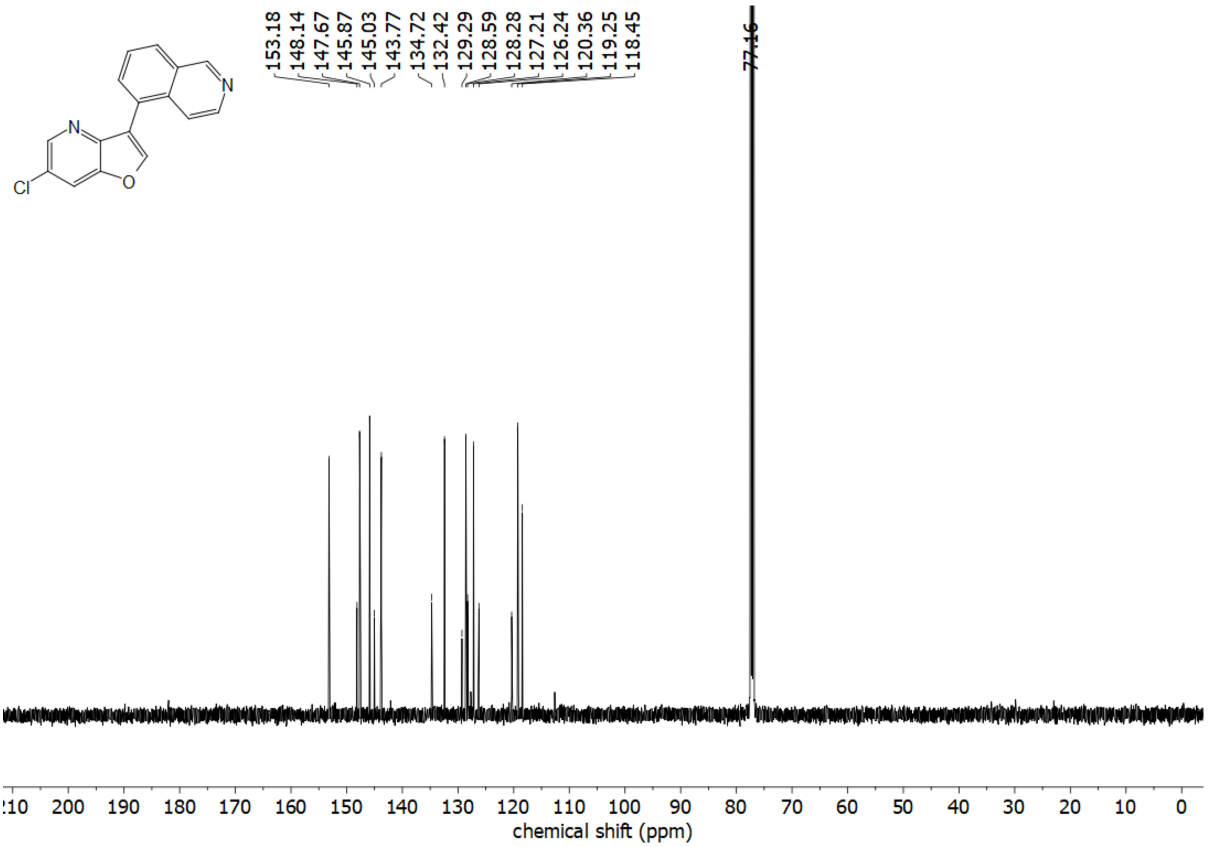

FT-IR spectrum (neat) of **29**.

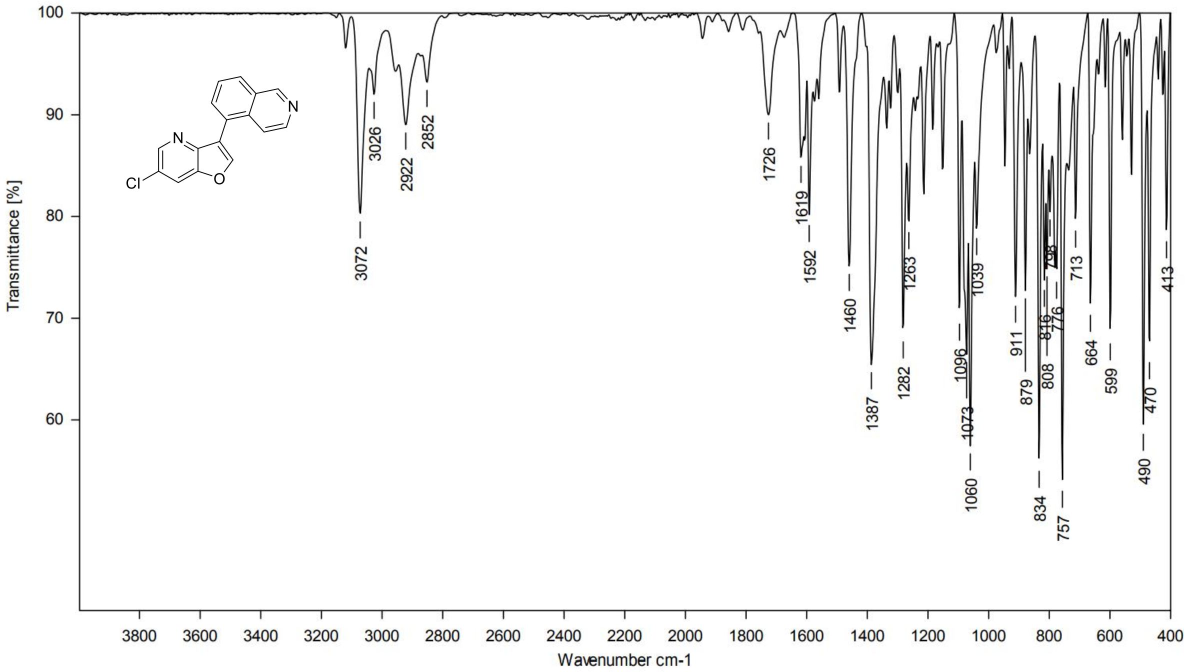

HRMS spectrum of **29**.

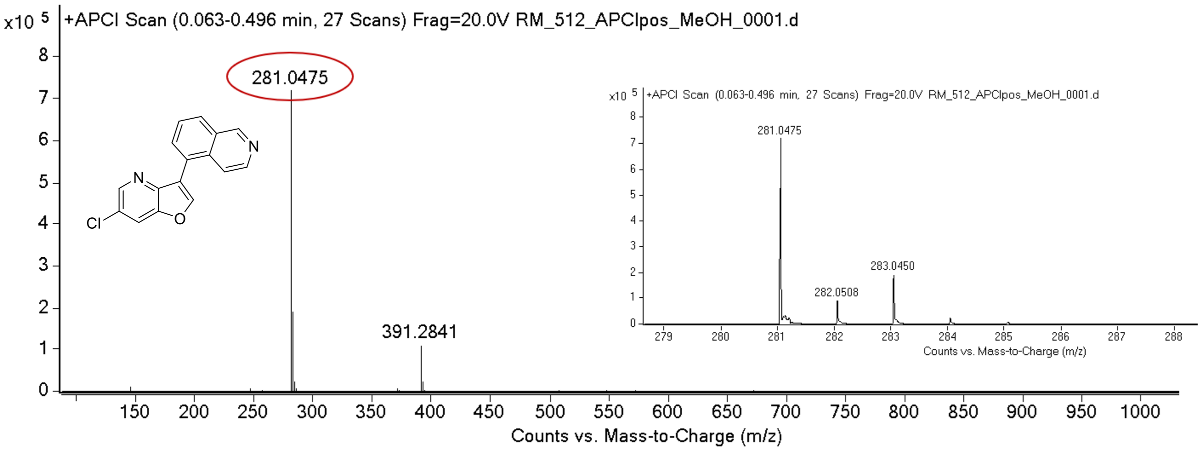

^{1}H (500 MHz) and ^{13}C NMR (126 MHz) spectra of **30** in chloroform-*d*.

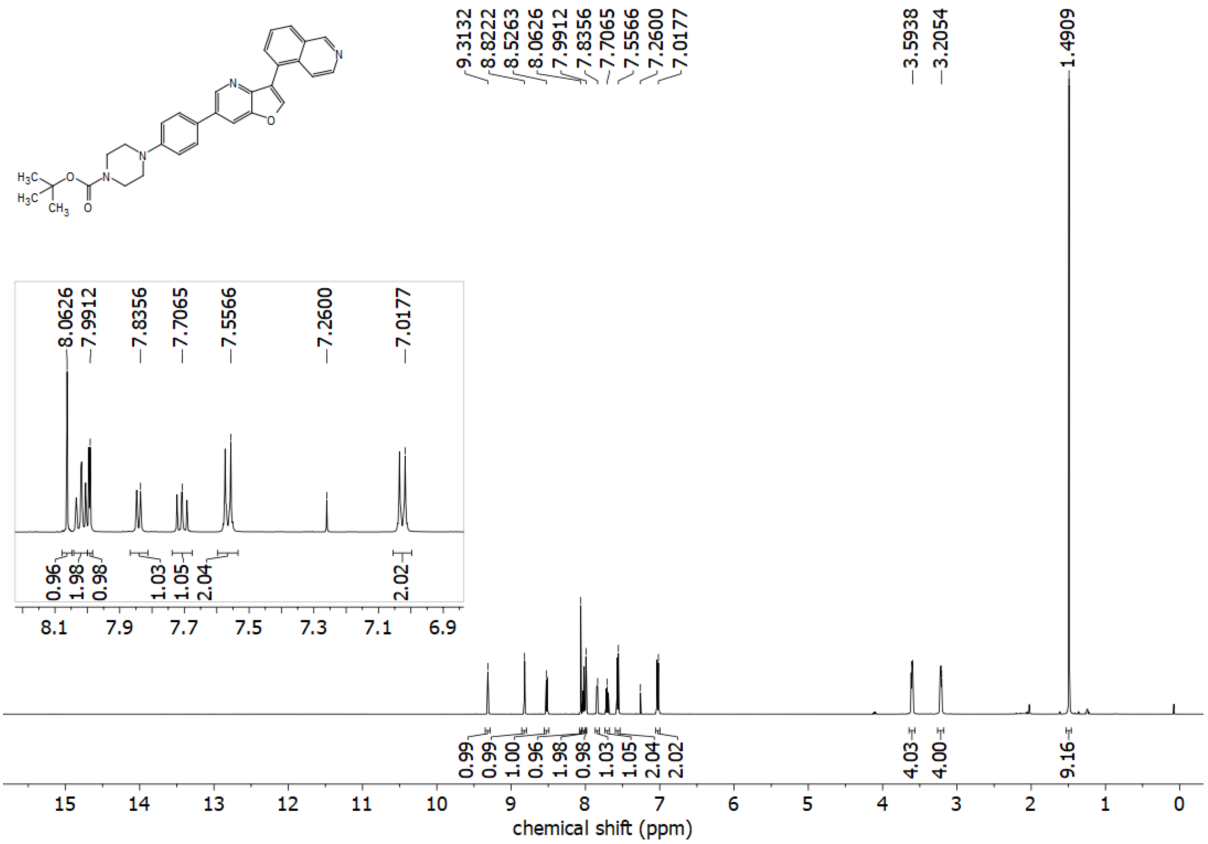

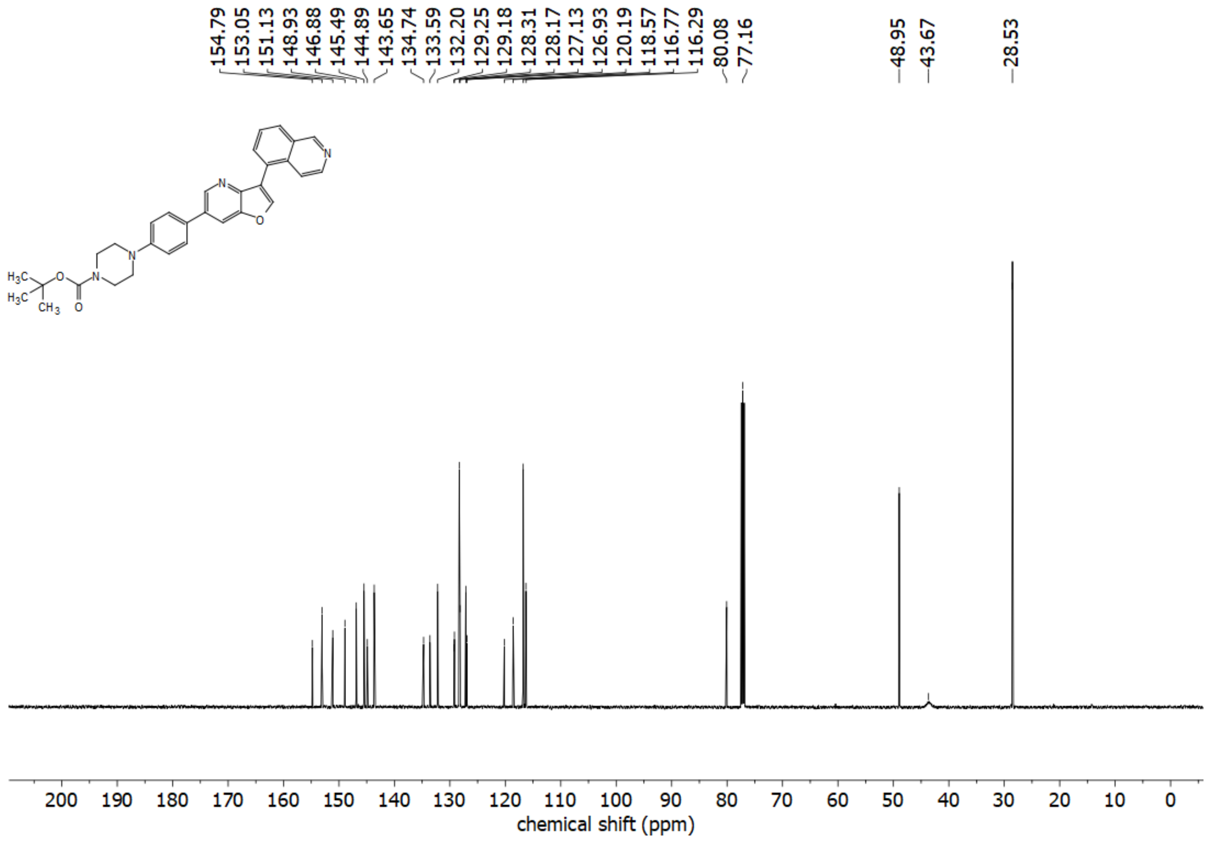

FT-IR spectrum (neat) of **30**.

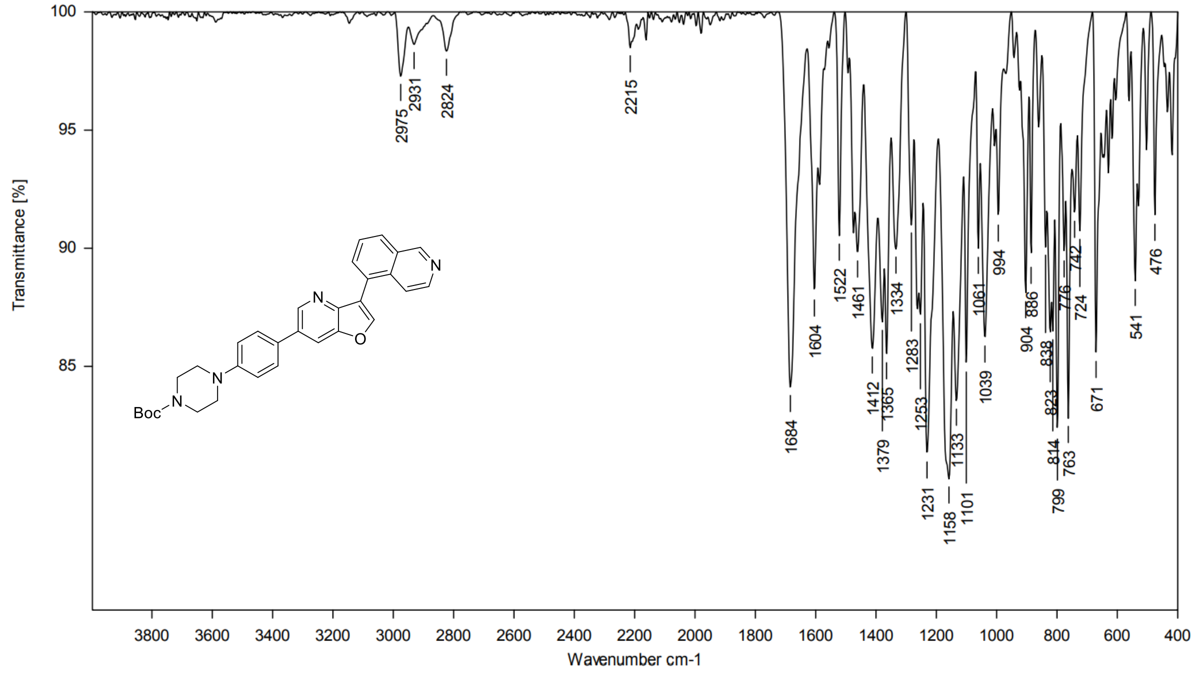

HRMS spectrum of **30**.

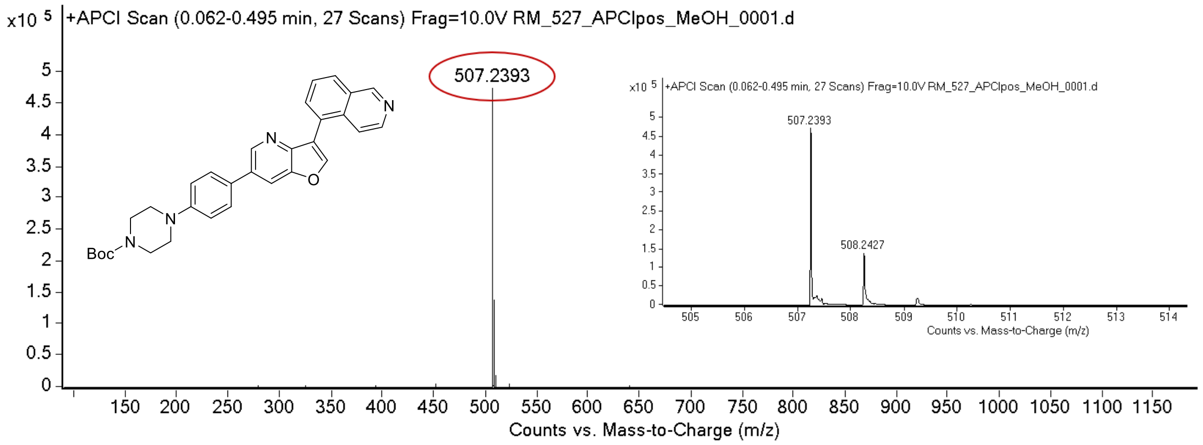

^{1}H (500 MHz) and ^{13}C NMR (126 MHz) spectra of **31** in DMSO-d_6.

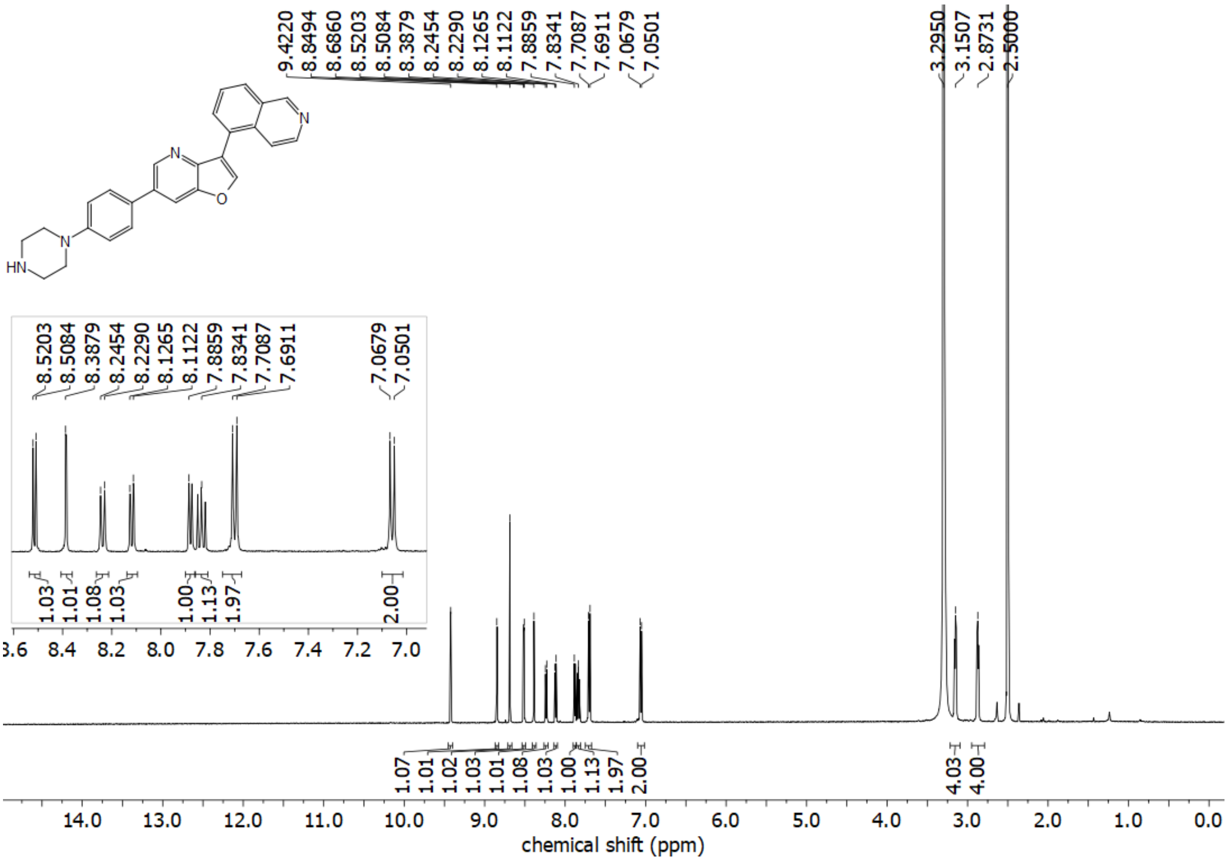

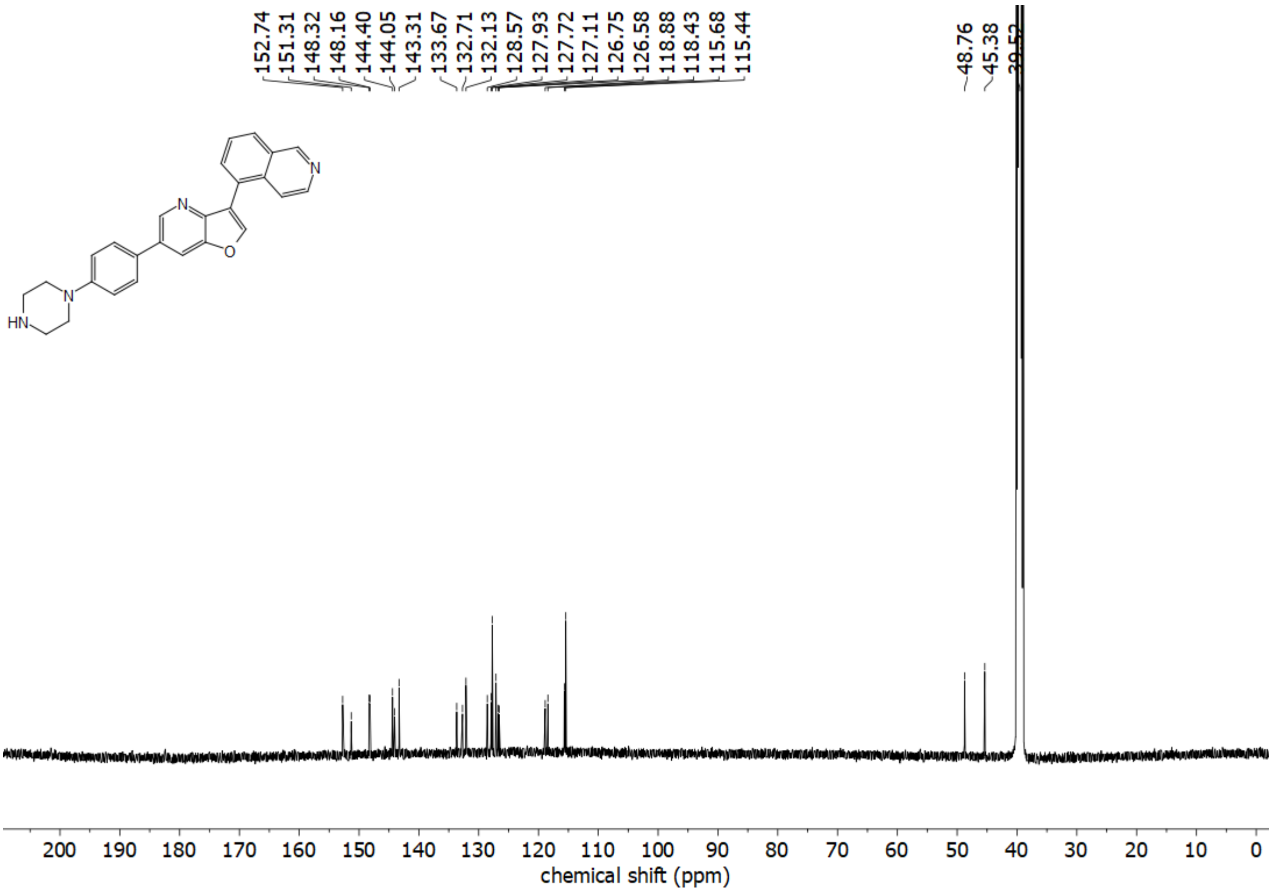

FT-IR spectrum (neat) of **31**.

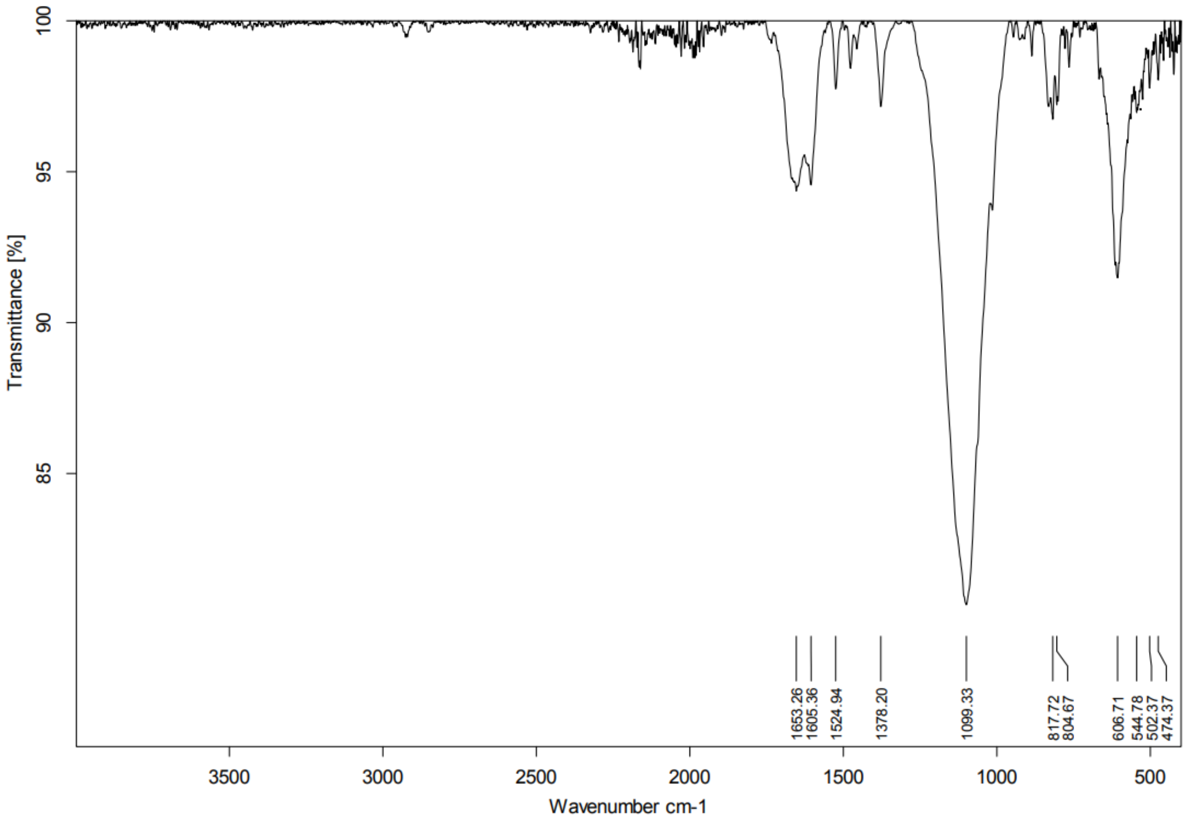

HRMS spectrum of **31**.

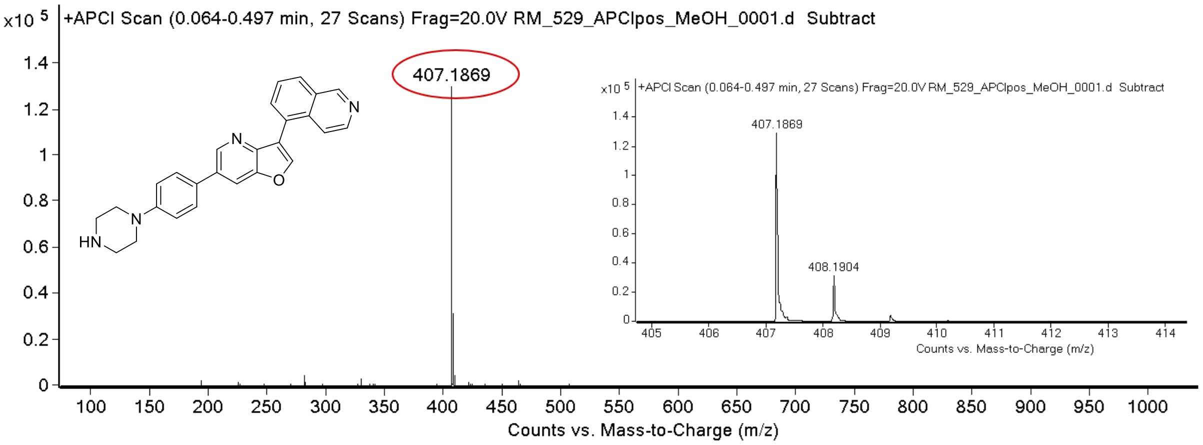

^{1}H (500 MHz) and ^{13}C NMR (126 MHz) spectra of **32** in chloroform-*d*.

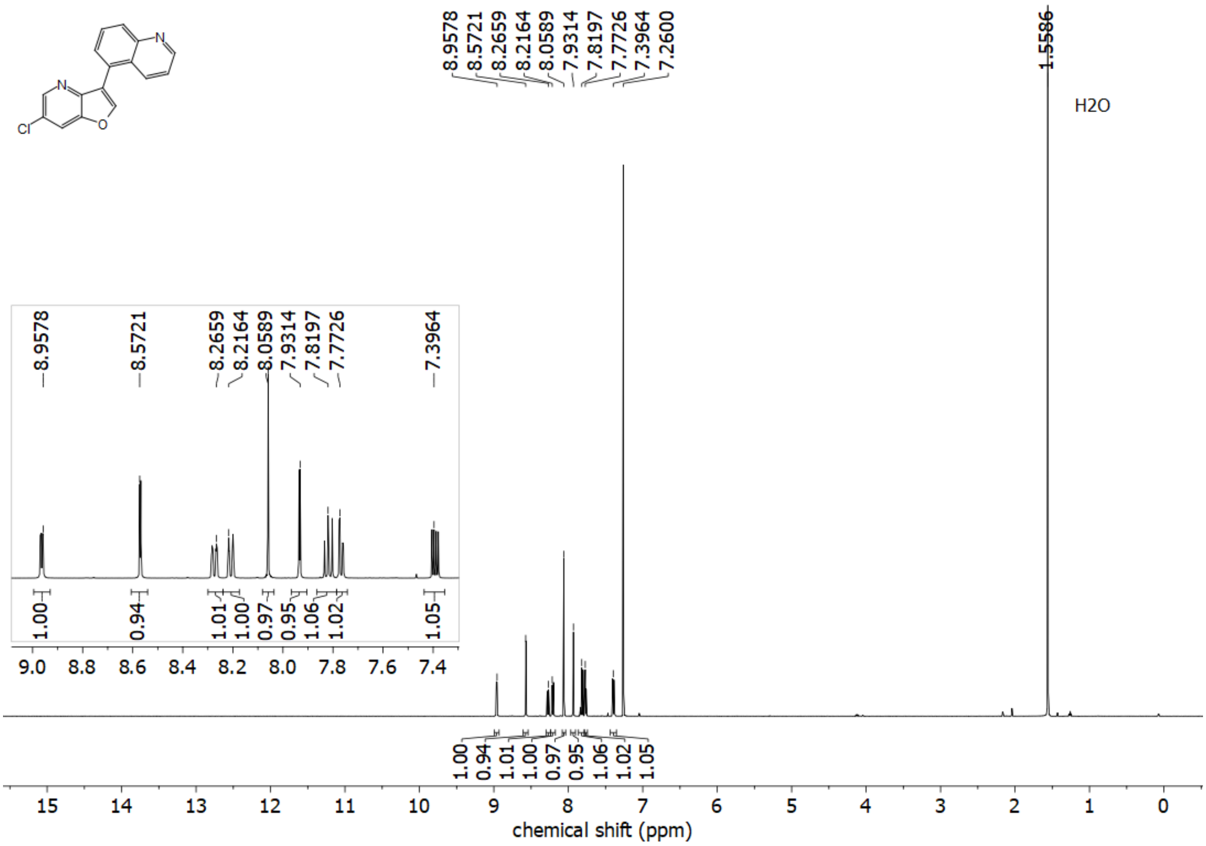

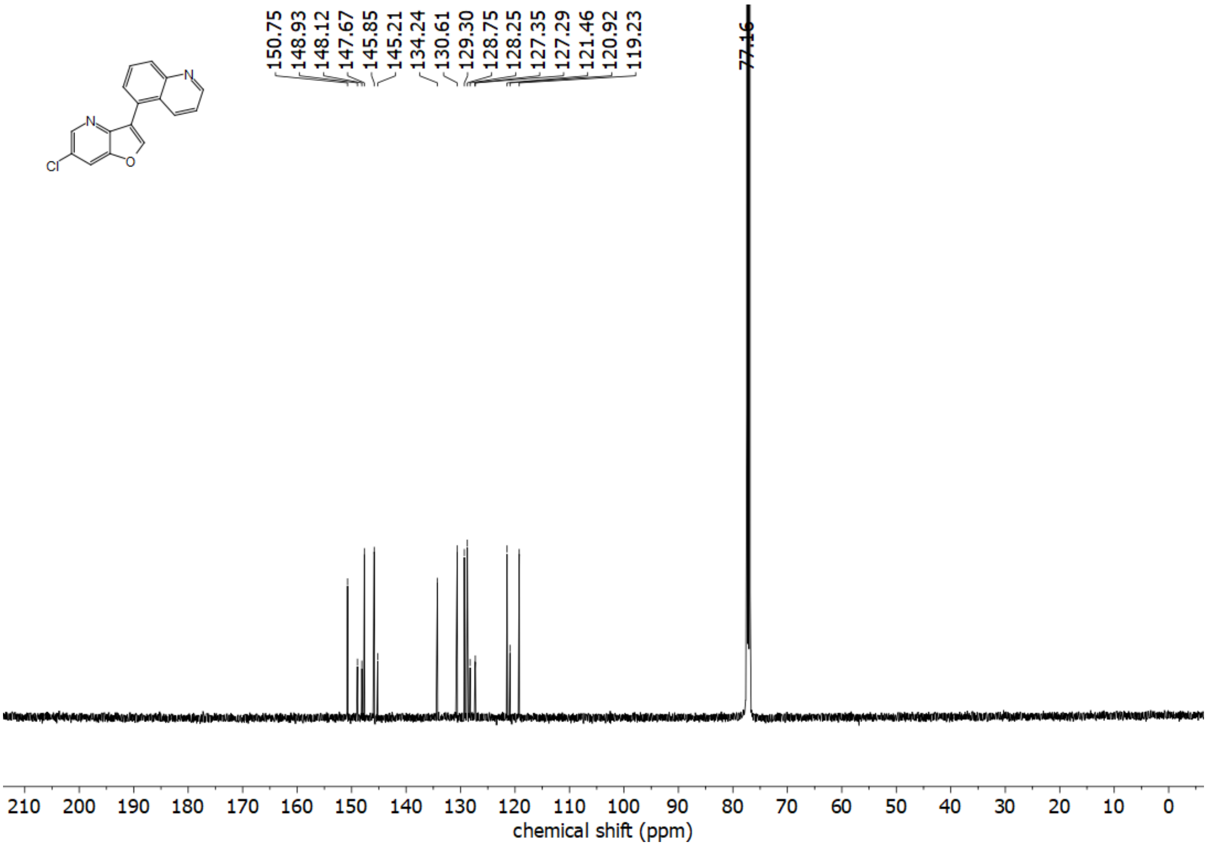

FT-IR spectrum (neat) of **32**.

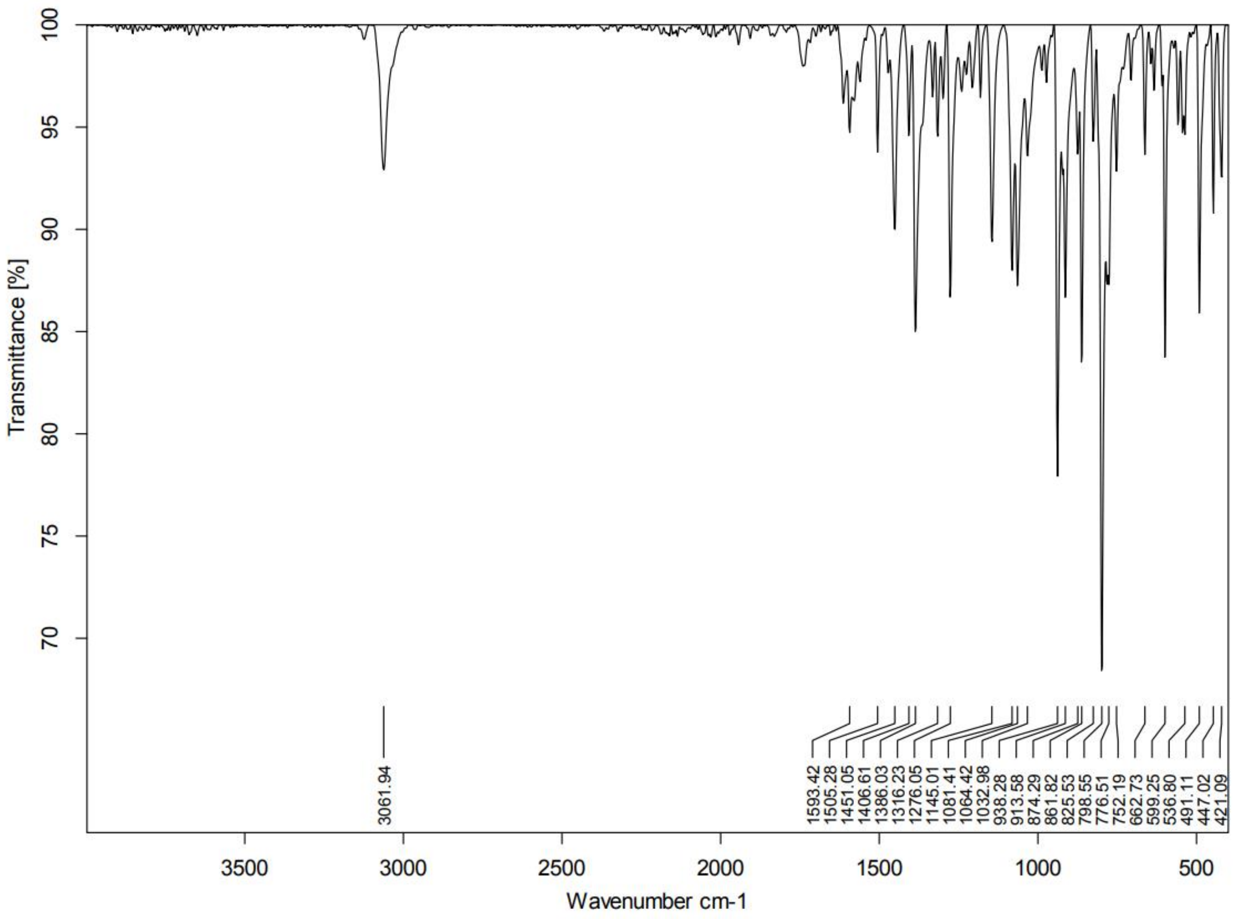

HRMS spectrum of **32**.

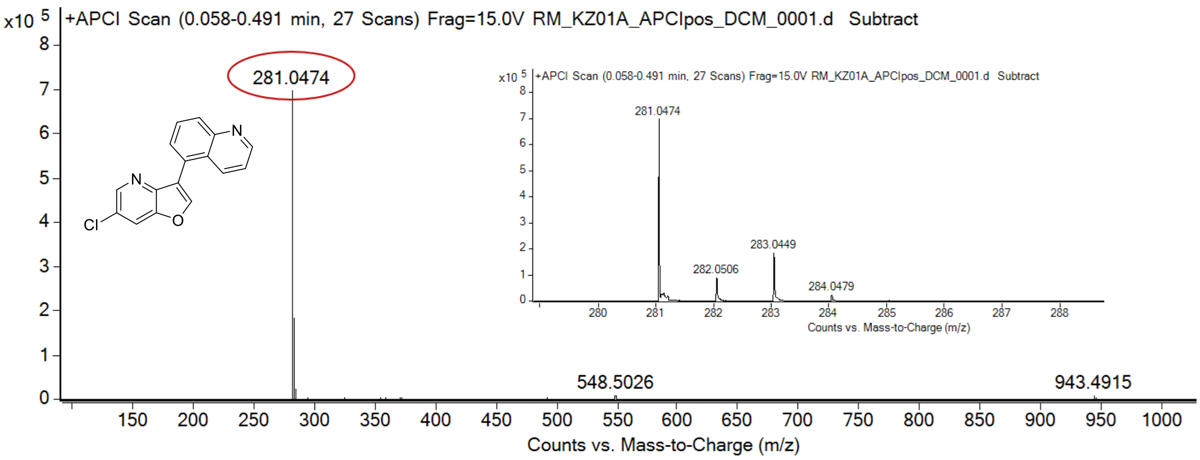

^{1}H (500 MHz) and ^{13}C NMR (126 MHz) spectra of **33** in chloroform-*d*.

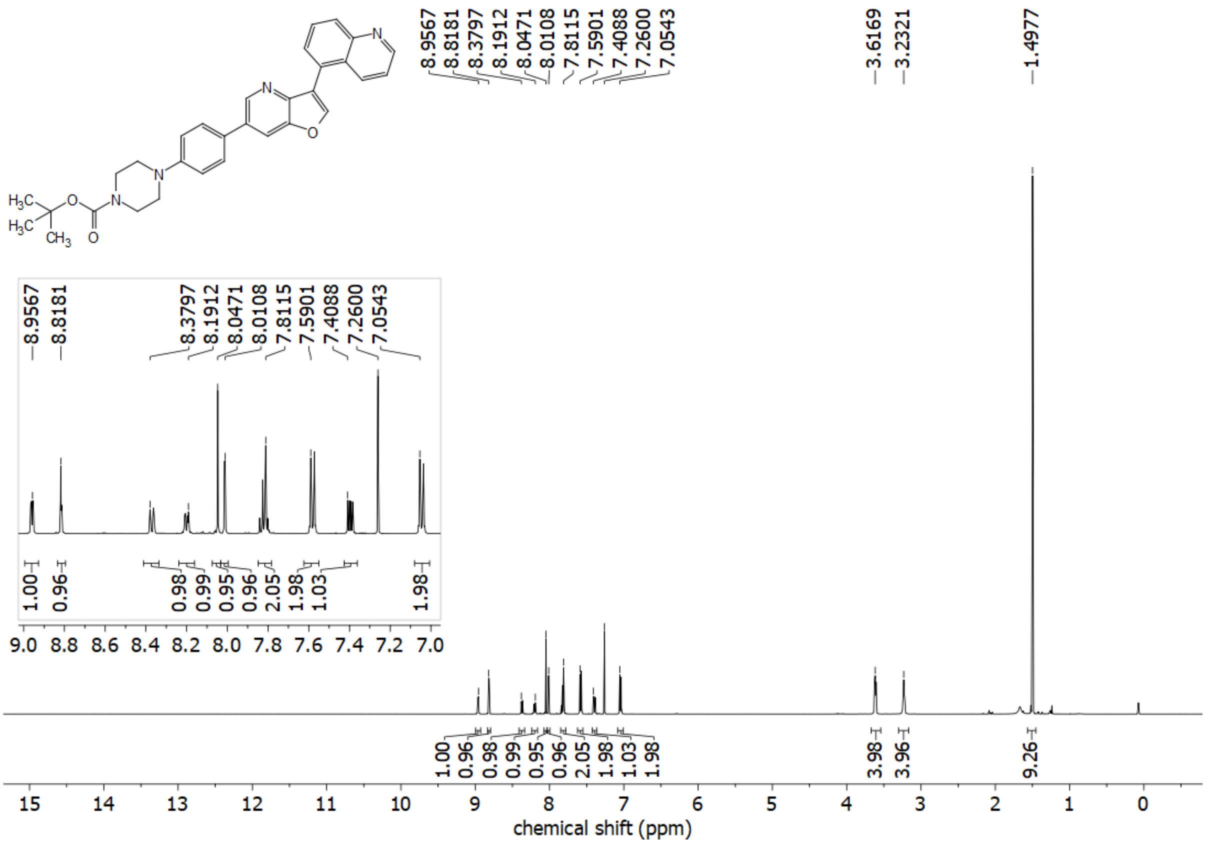

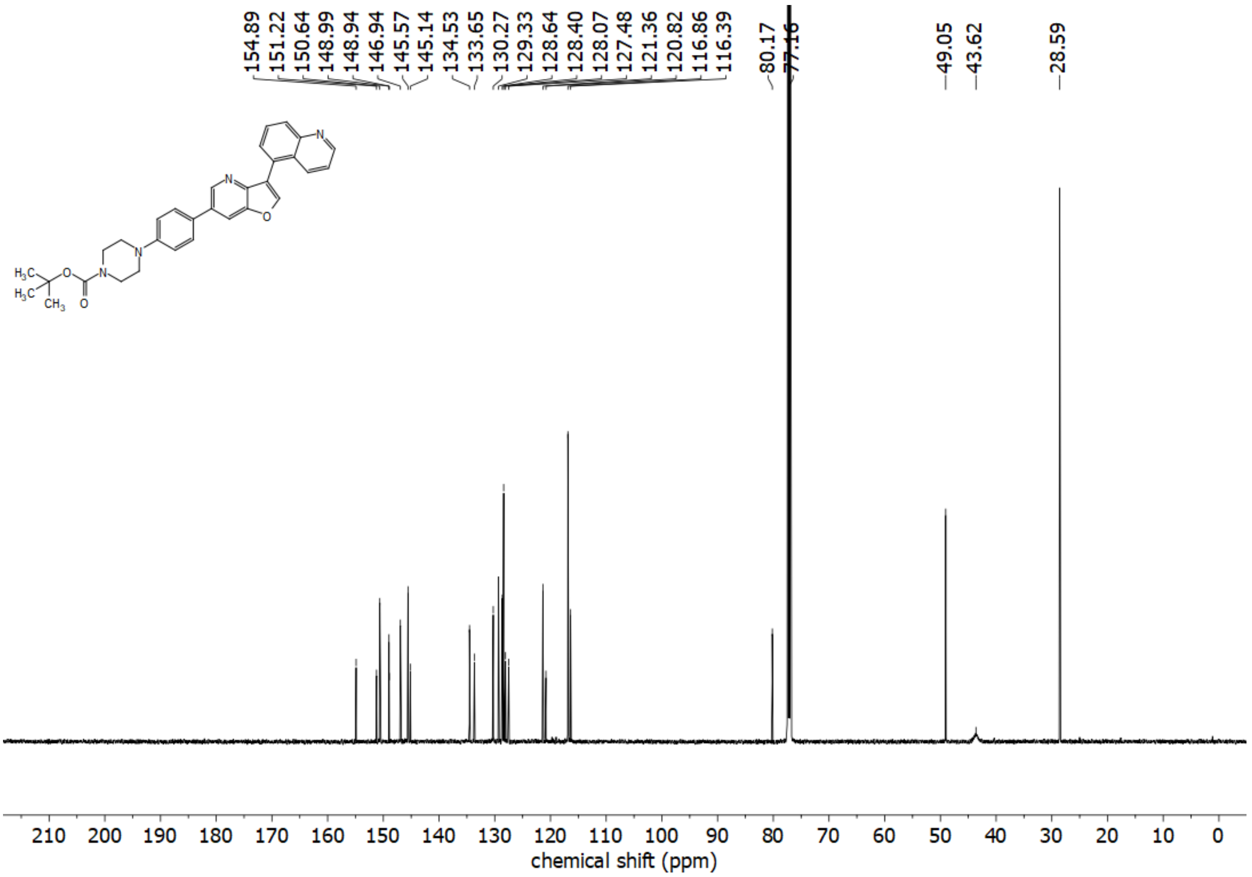

FT-IR spectrum (neat) of **33**.

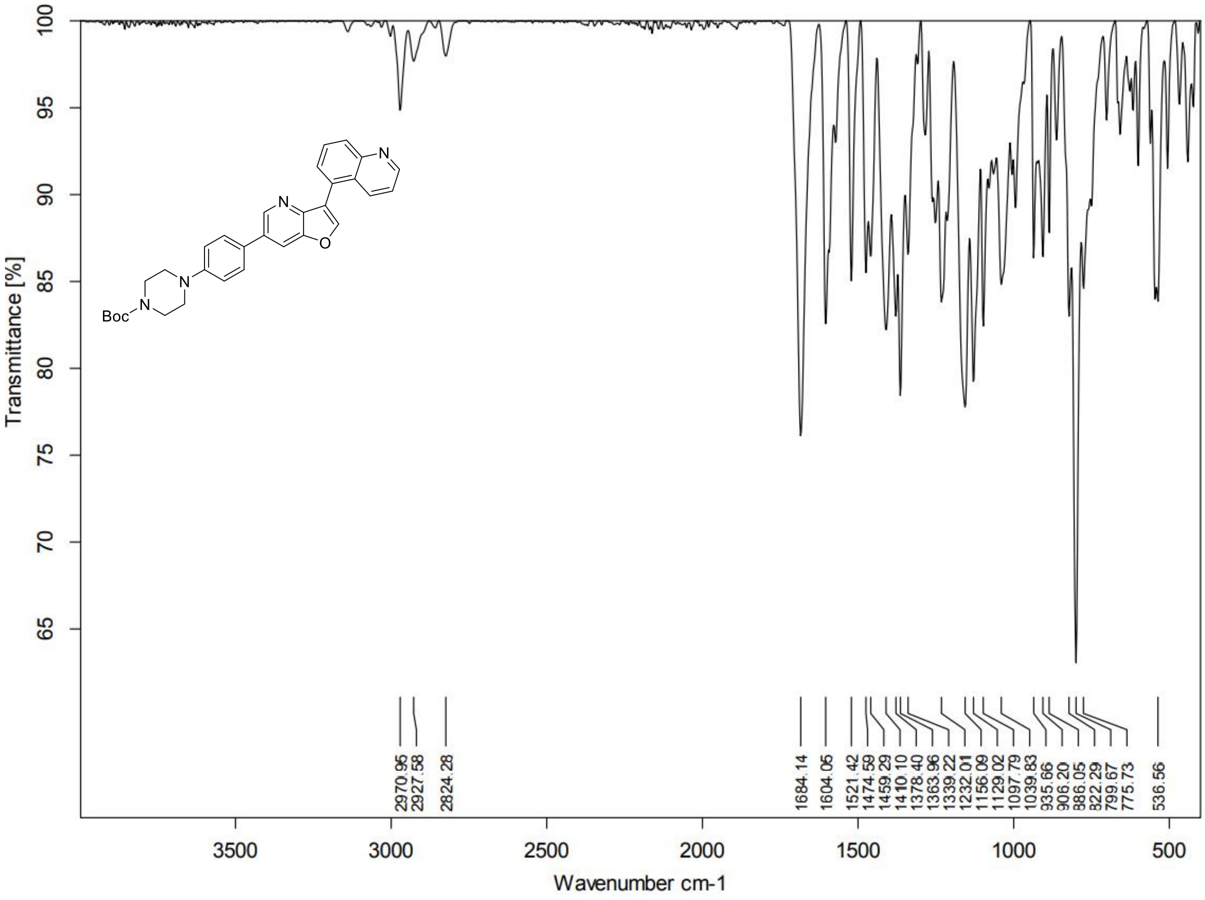

HRMS spectrum of **33**.

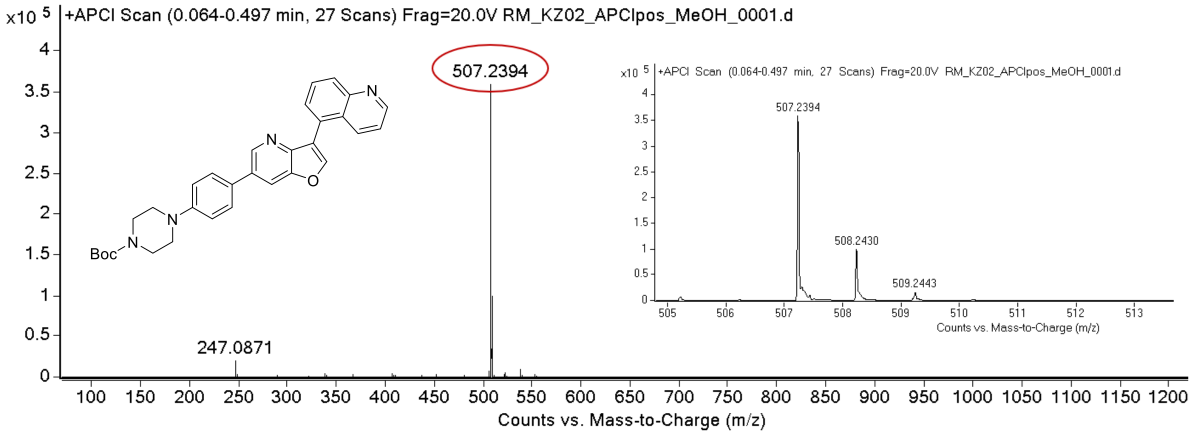

^{1}H (500 MHz) and ^{13}C NMR (126 MHz) spectra of **34** in chloroform-*d*.

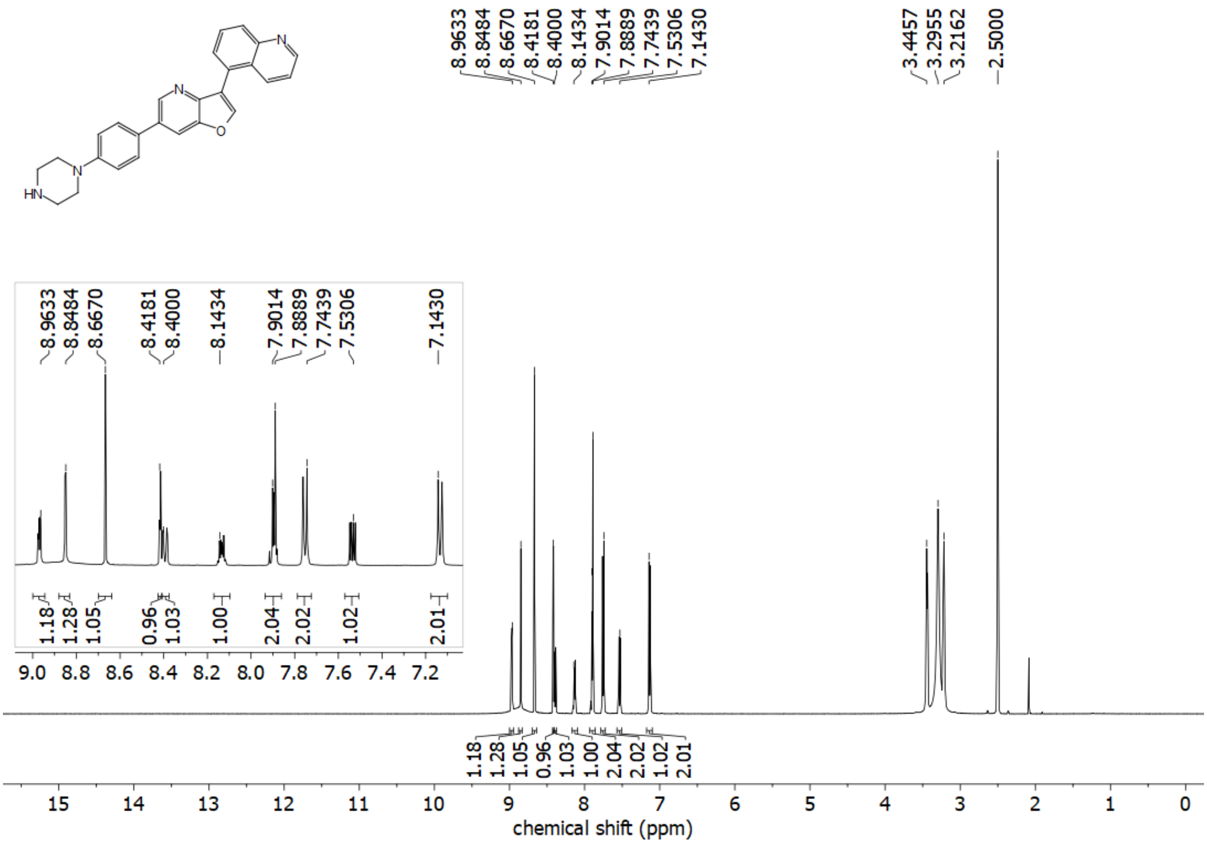

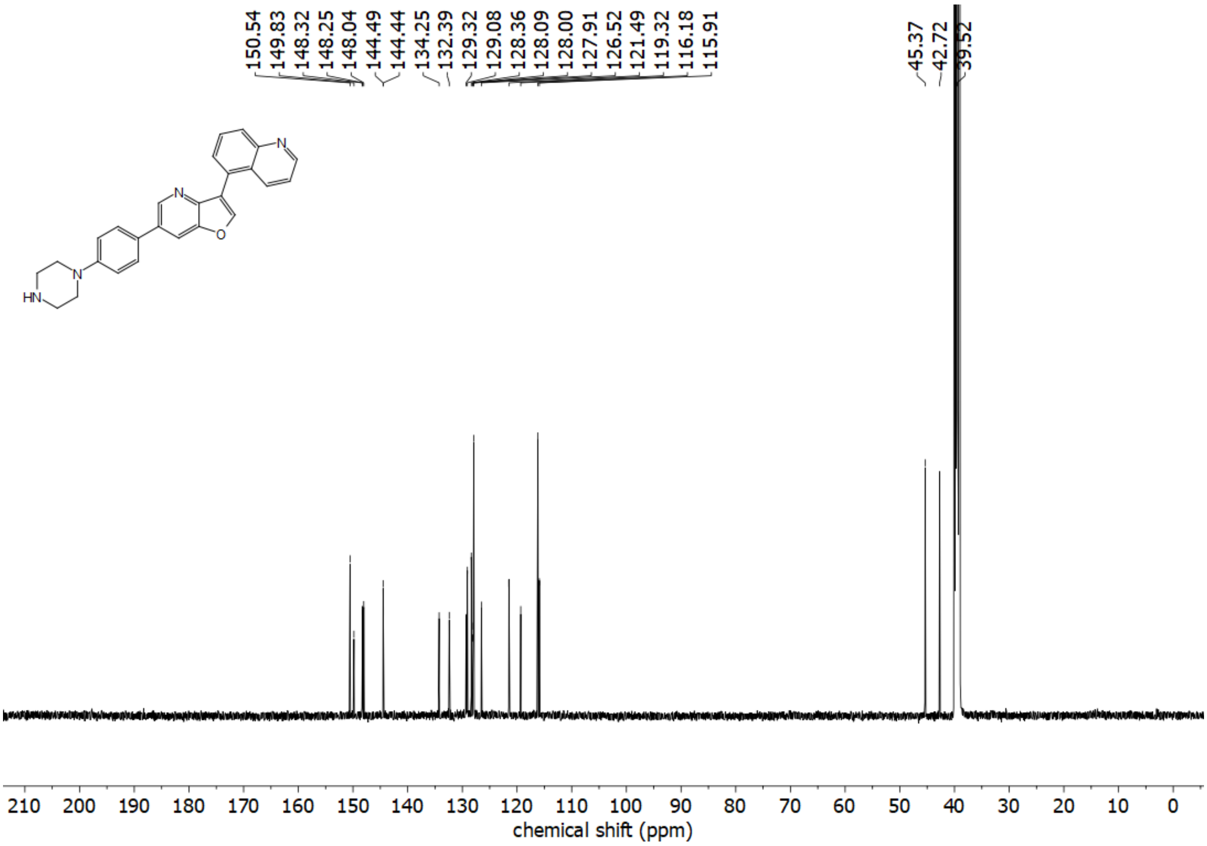

FT-IR spectrum (neat) of **34**.

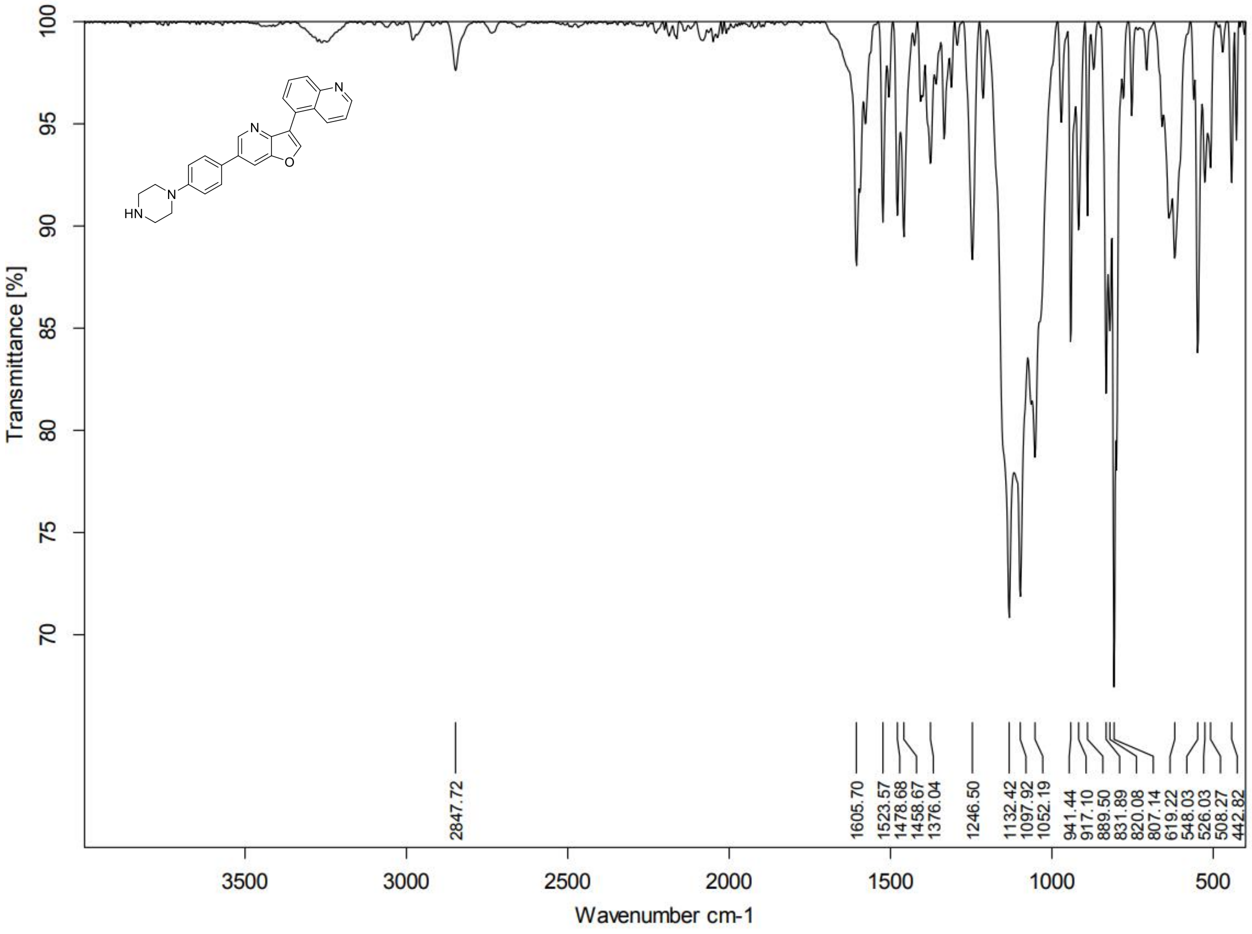

HRMS spectrum of **34**.

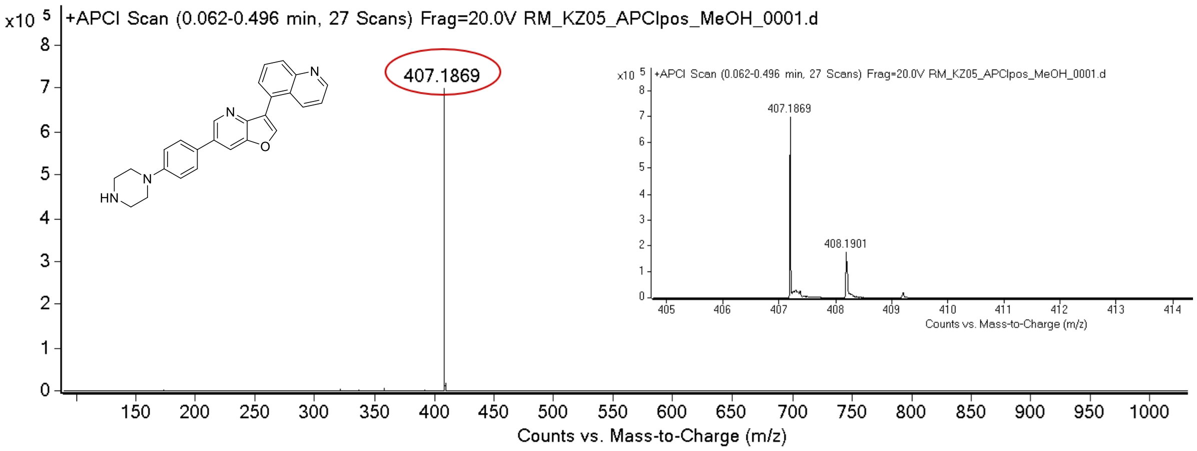

^{1}H (500 MHz) and ^{13}C NMR (126 MHz) spectra of **35** in chloroform-*d*.

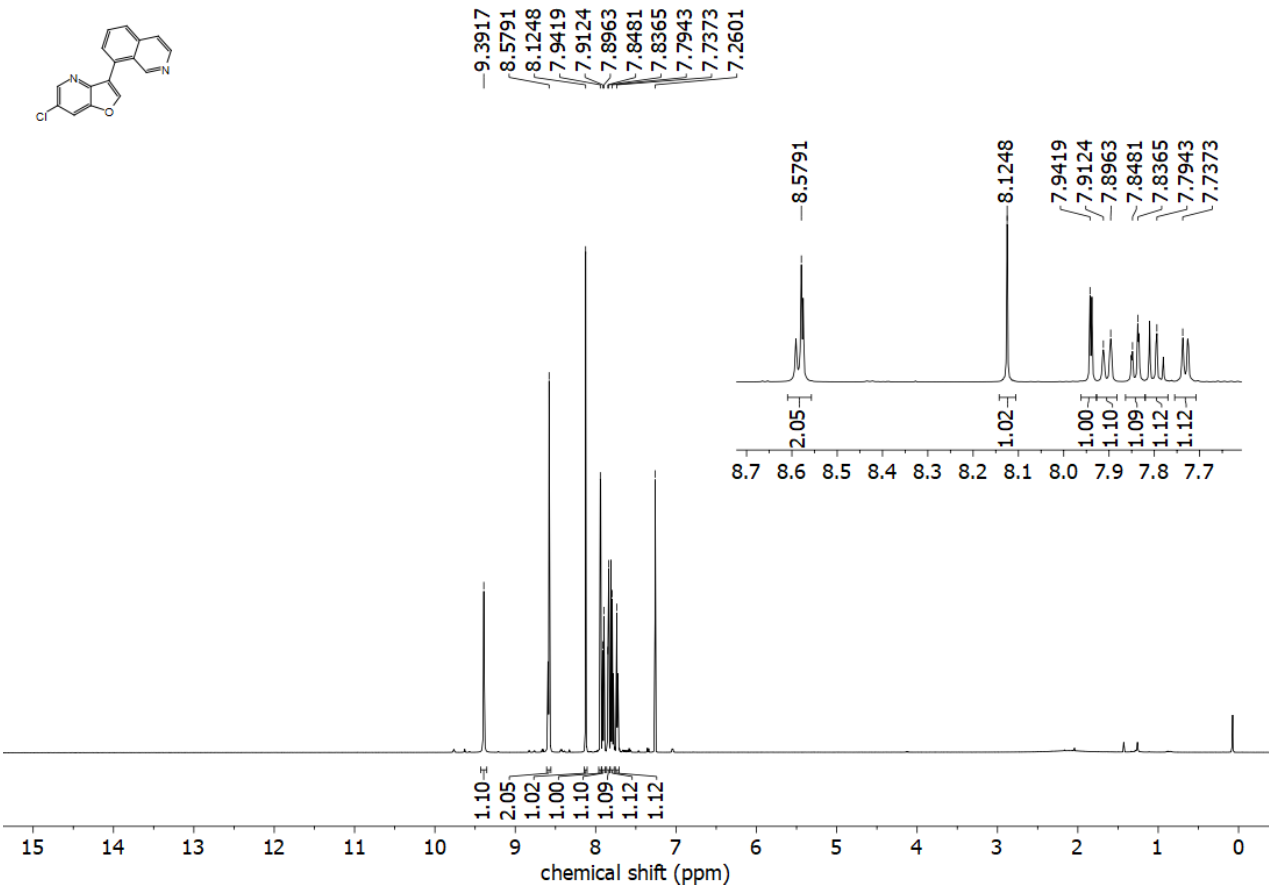

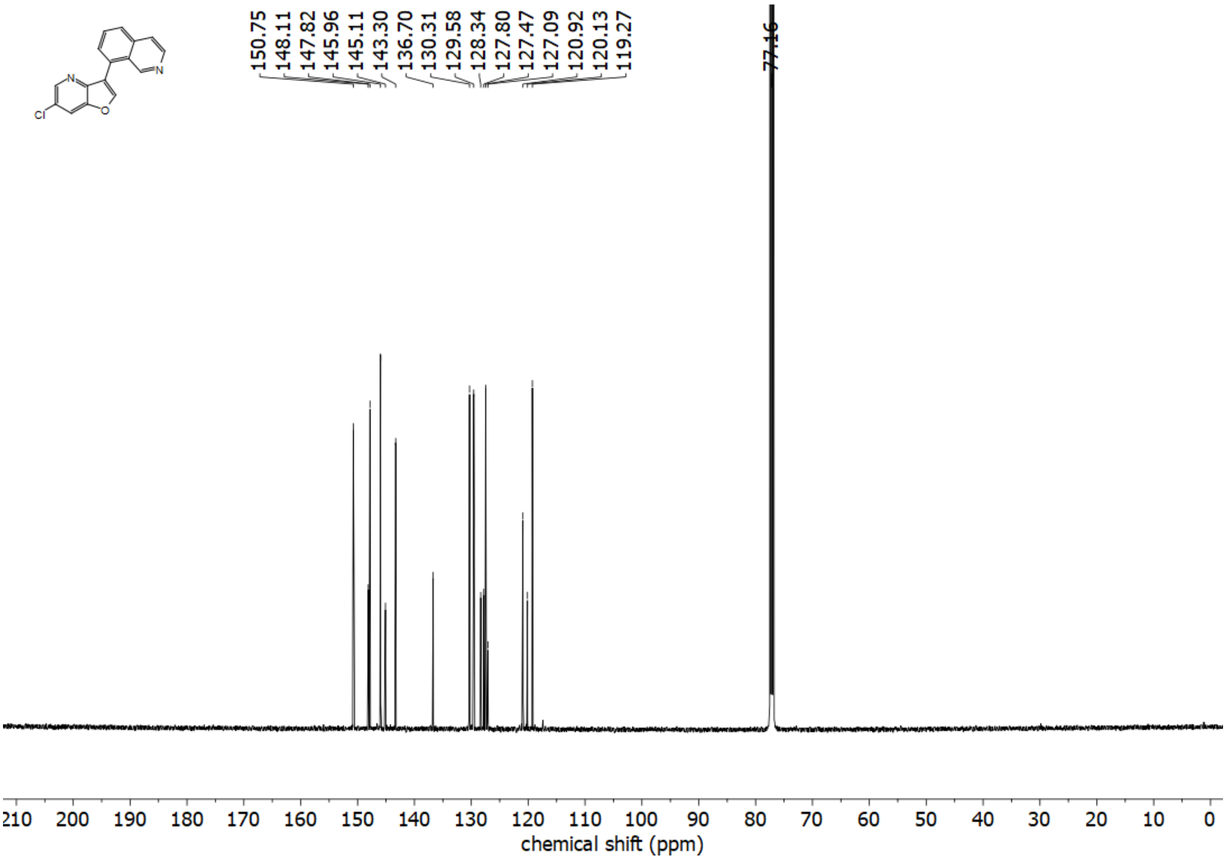

FT-IR spectrum (neat) of **35**.

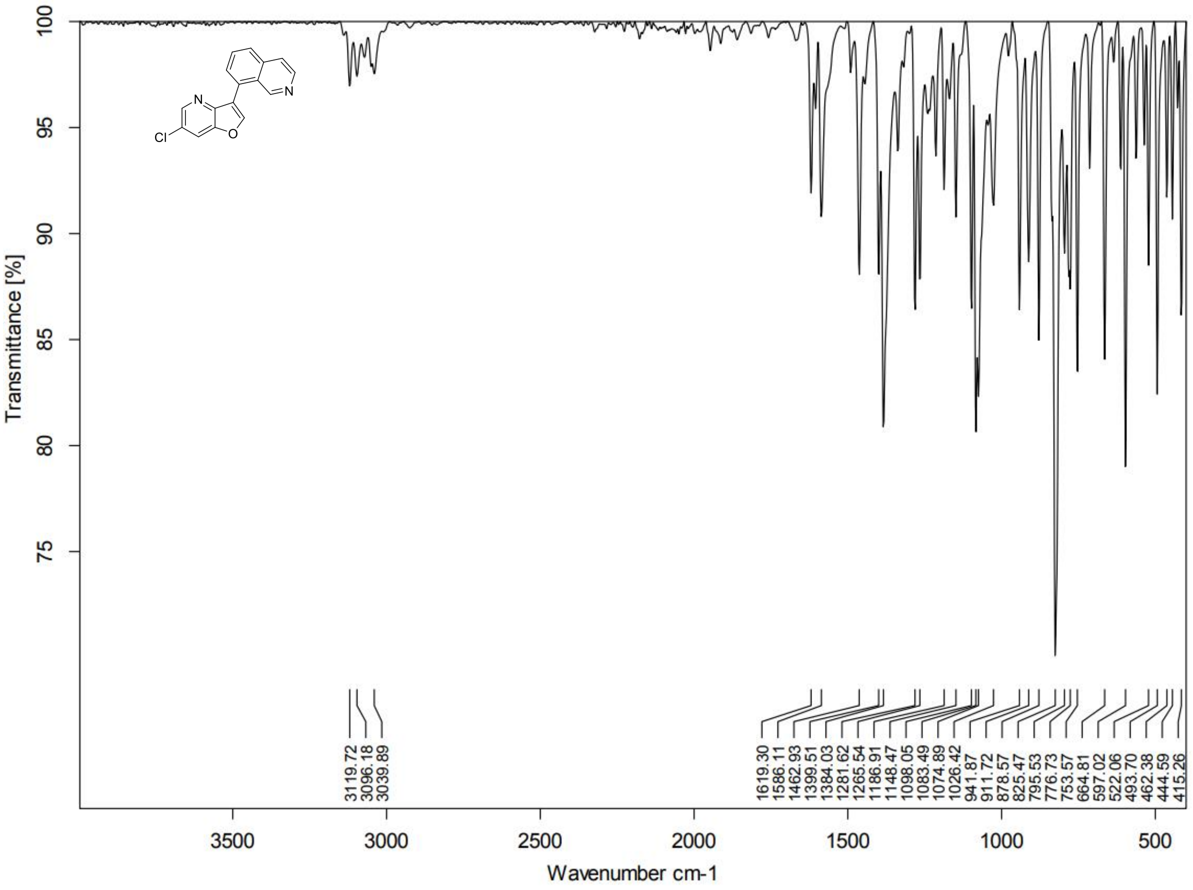

HRMS spectrum of **35**.

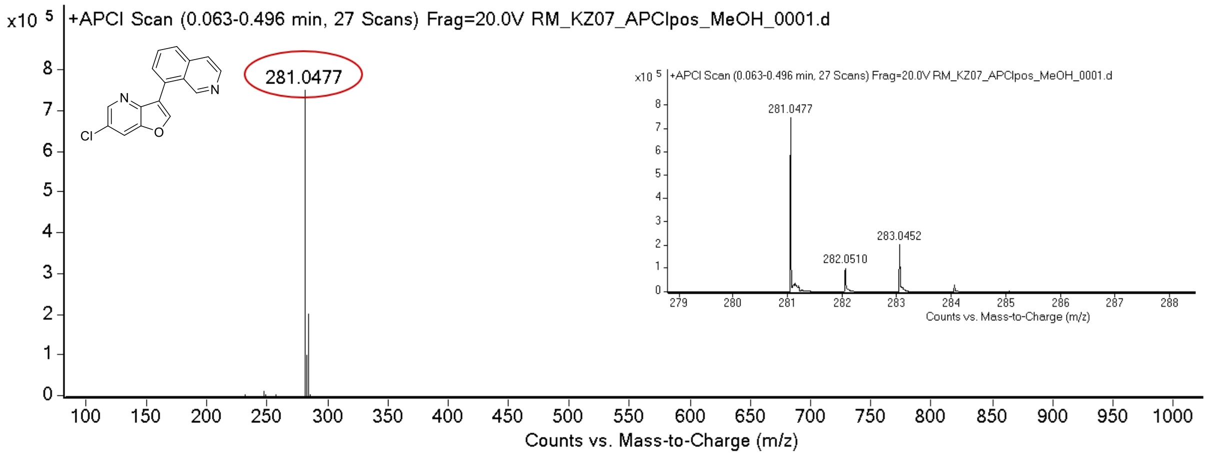

^{1}H (500 MHz) and ^{13}C NMR (126 MHz) spectra of **36** in chloroform-*d*.

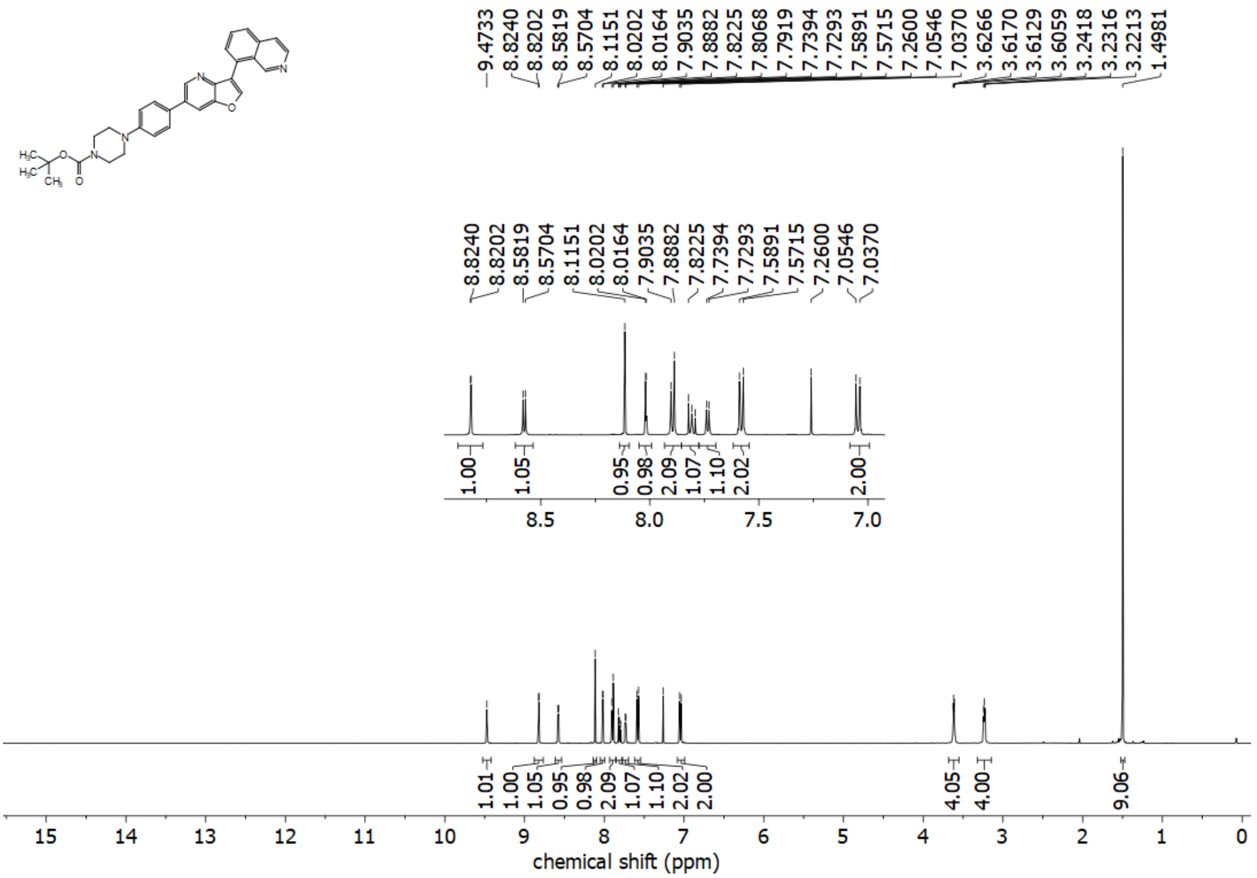

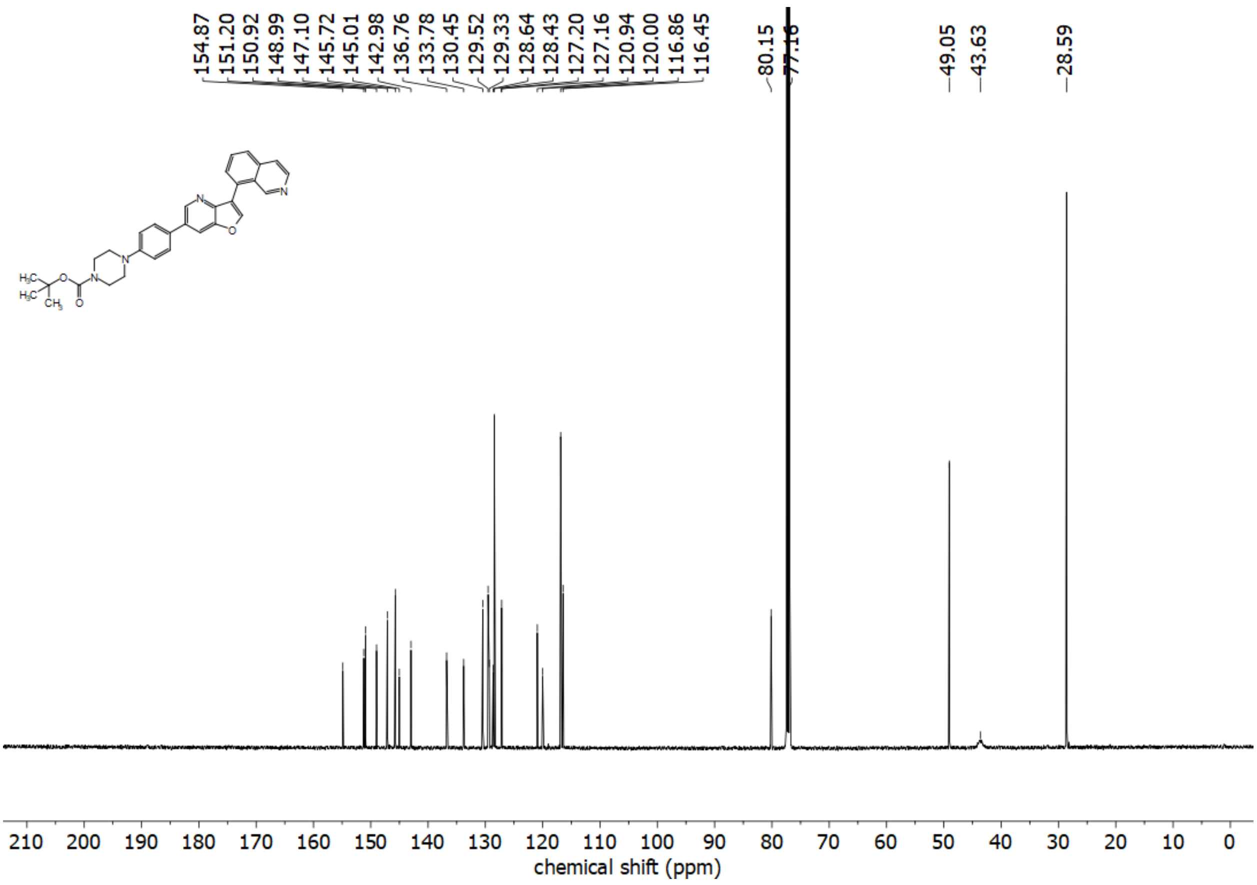

FT-IR spectrum (neat) of **36**.

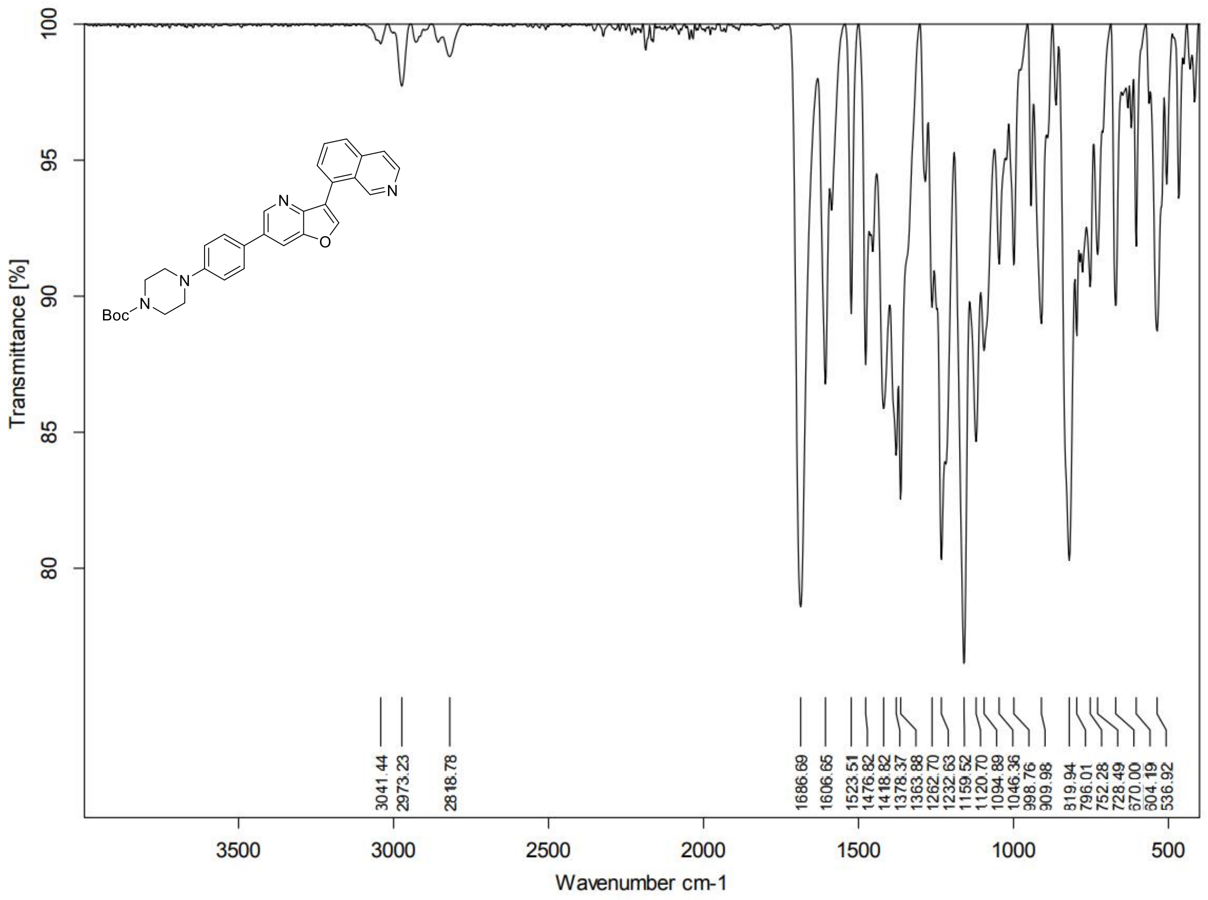

HRMS spectrum of **36**.

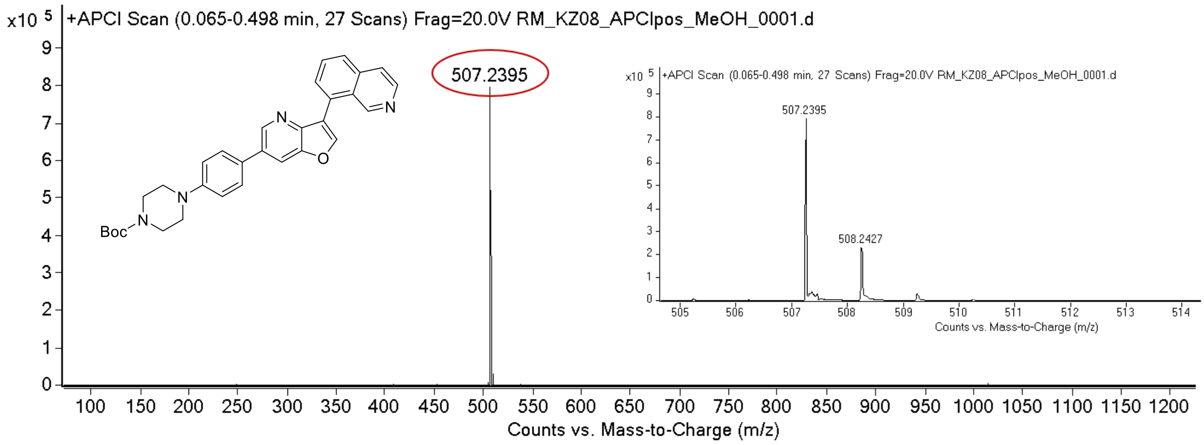

^{1}H (500 MHz) and ^{13}C NMR (126 MHz) spectra of **37** in DMSO-d_6.

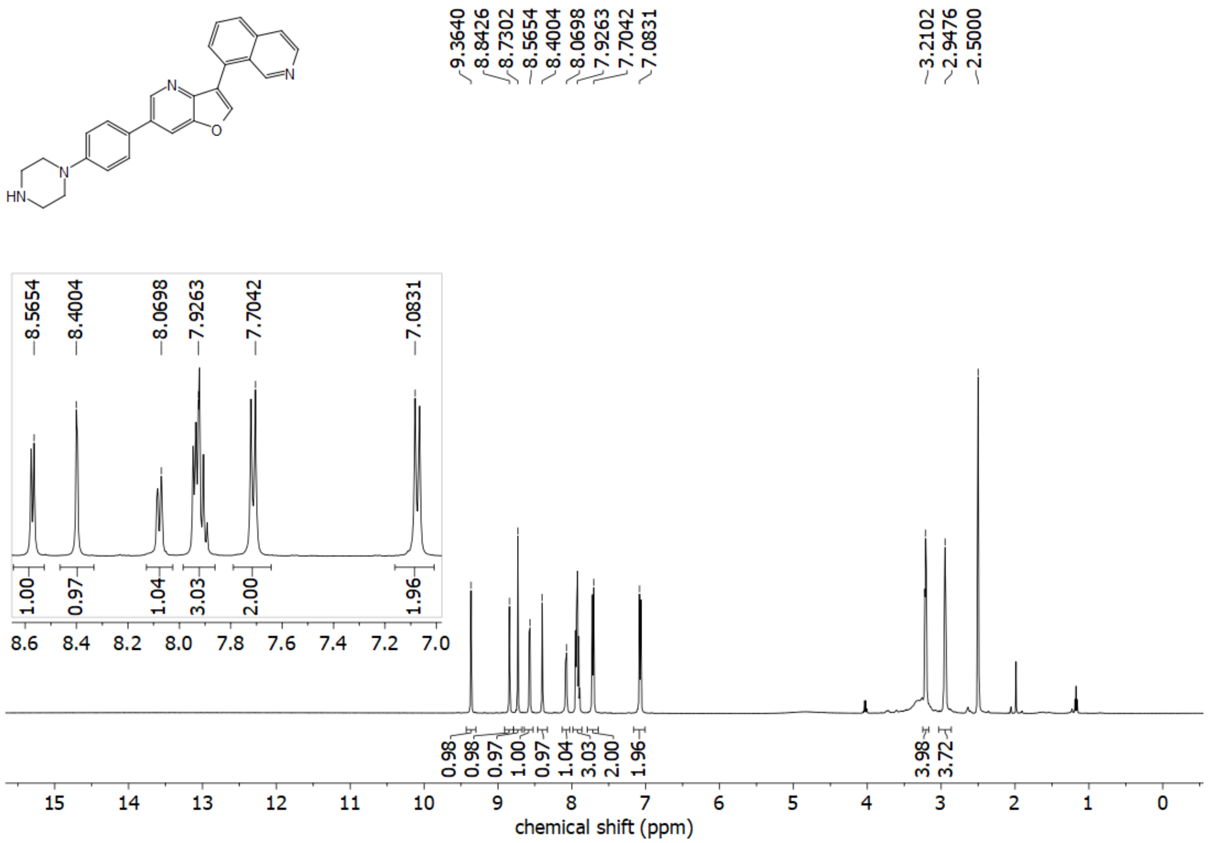

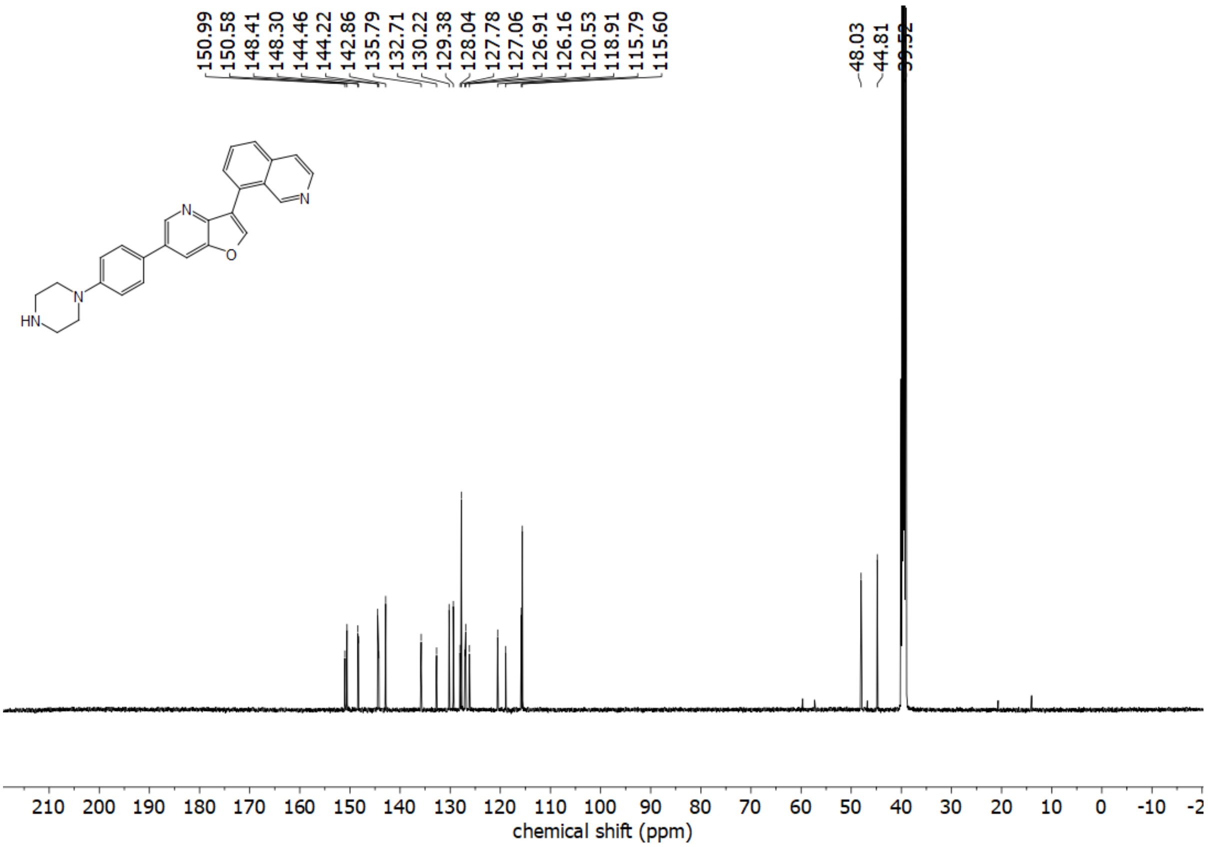

FT-IR spectrum (neat) of **37**.

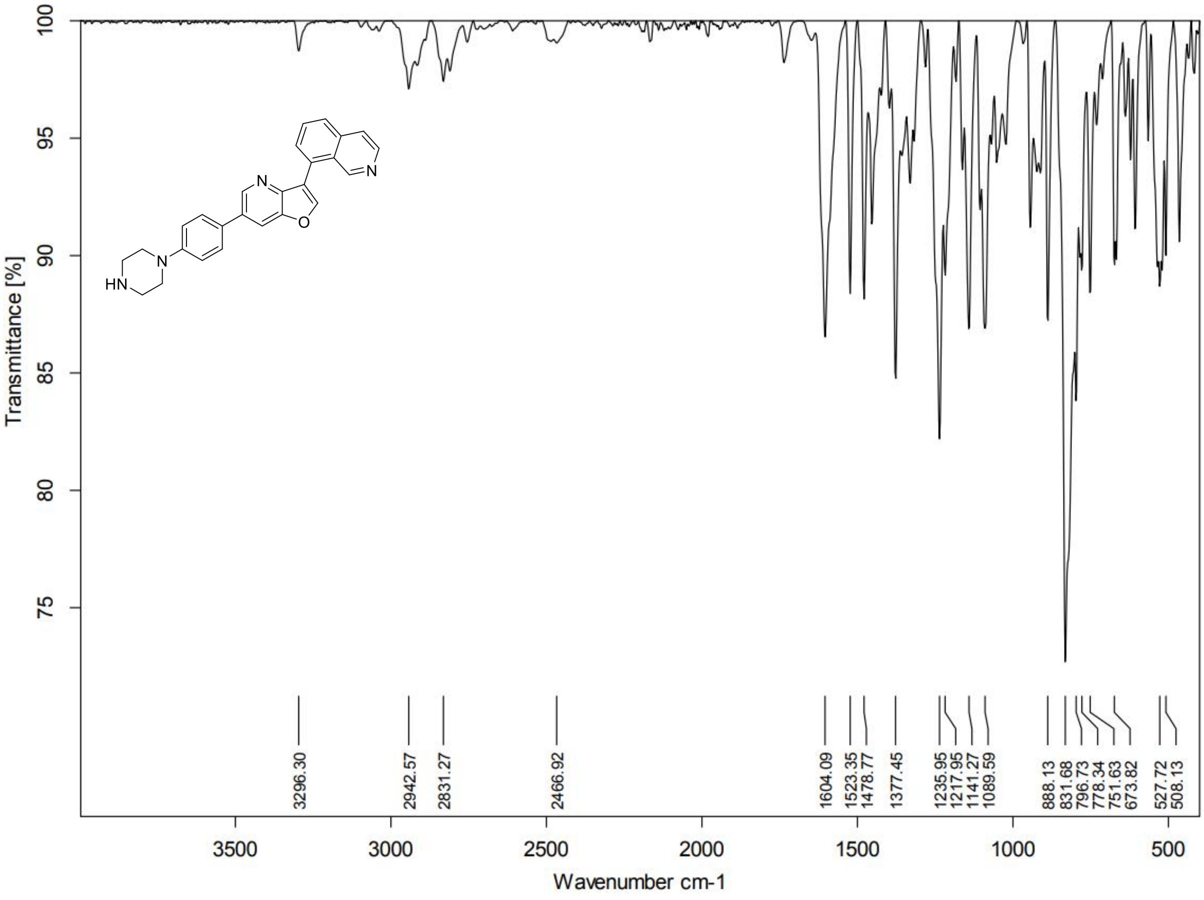

HRMS spectrum of **37**.

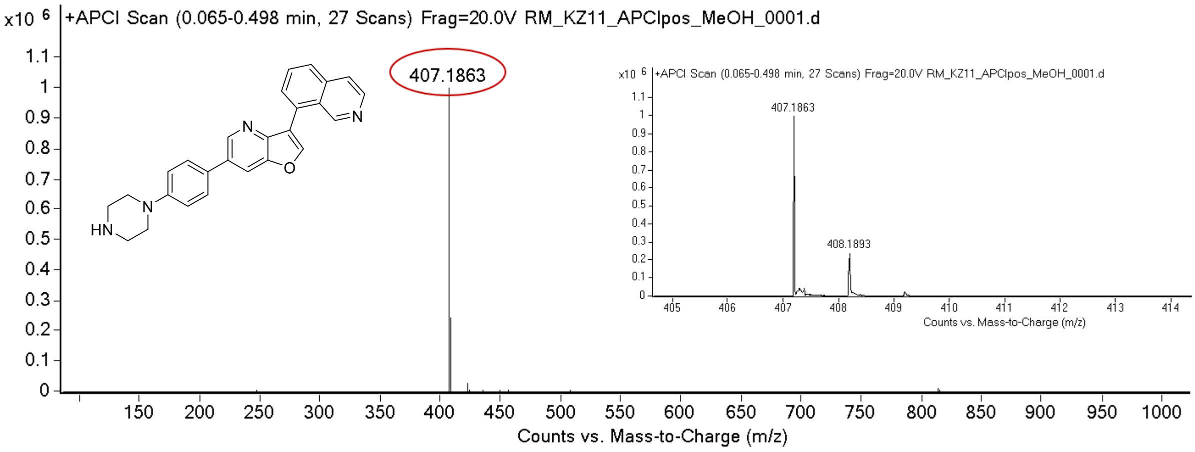

^{1}H (500 MHz) and ^{13}C NMR (126 MHz) spectra of **38** in DMSO-d_6.

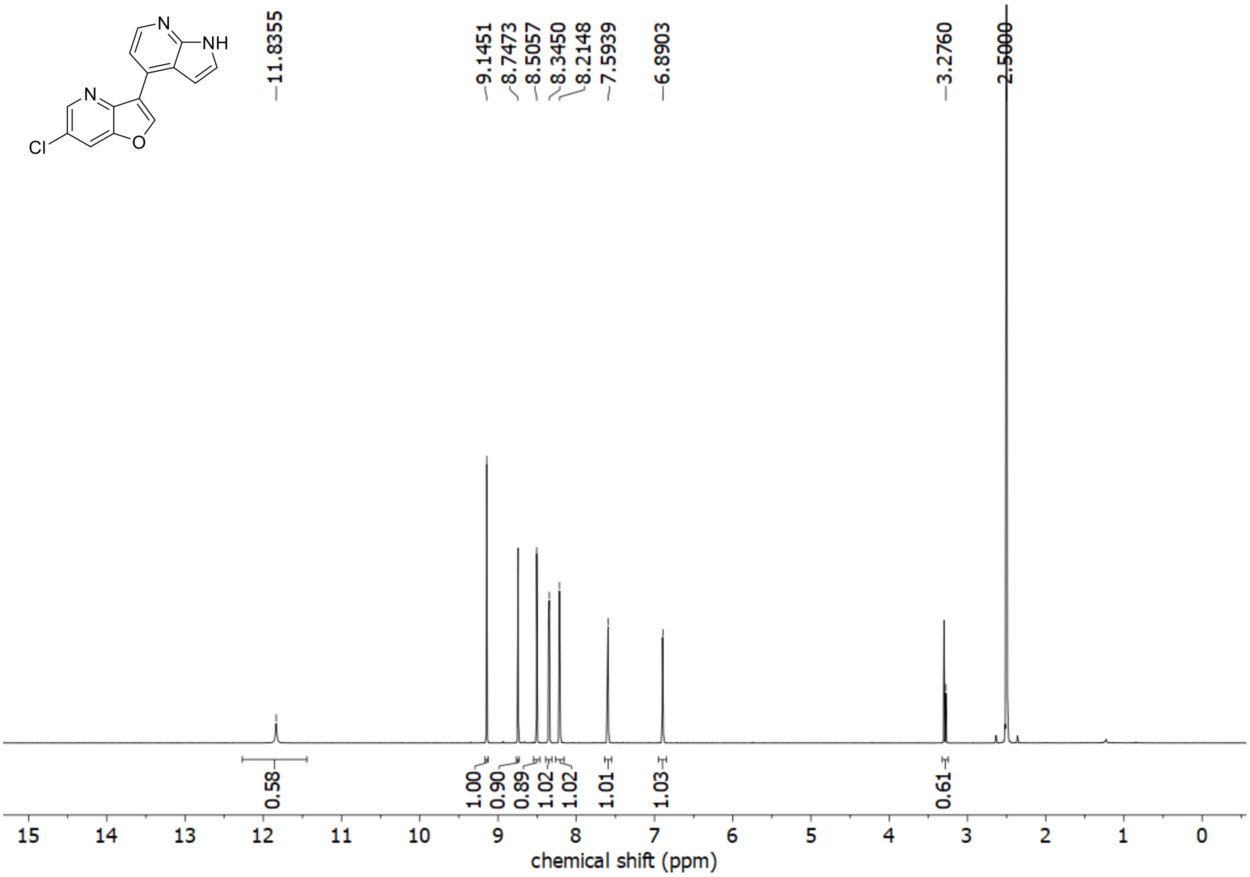

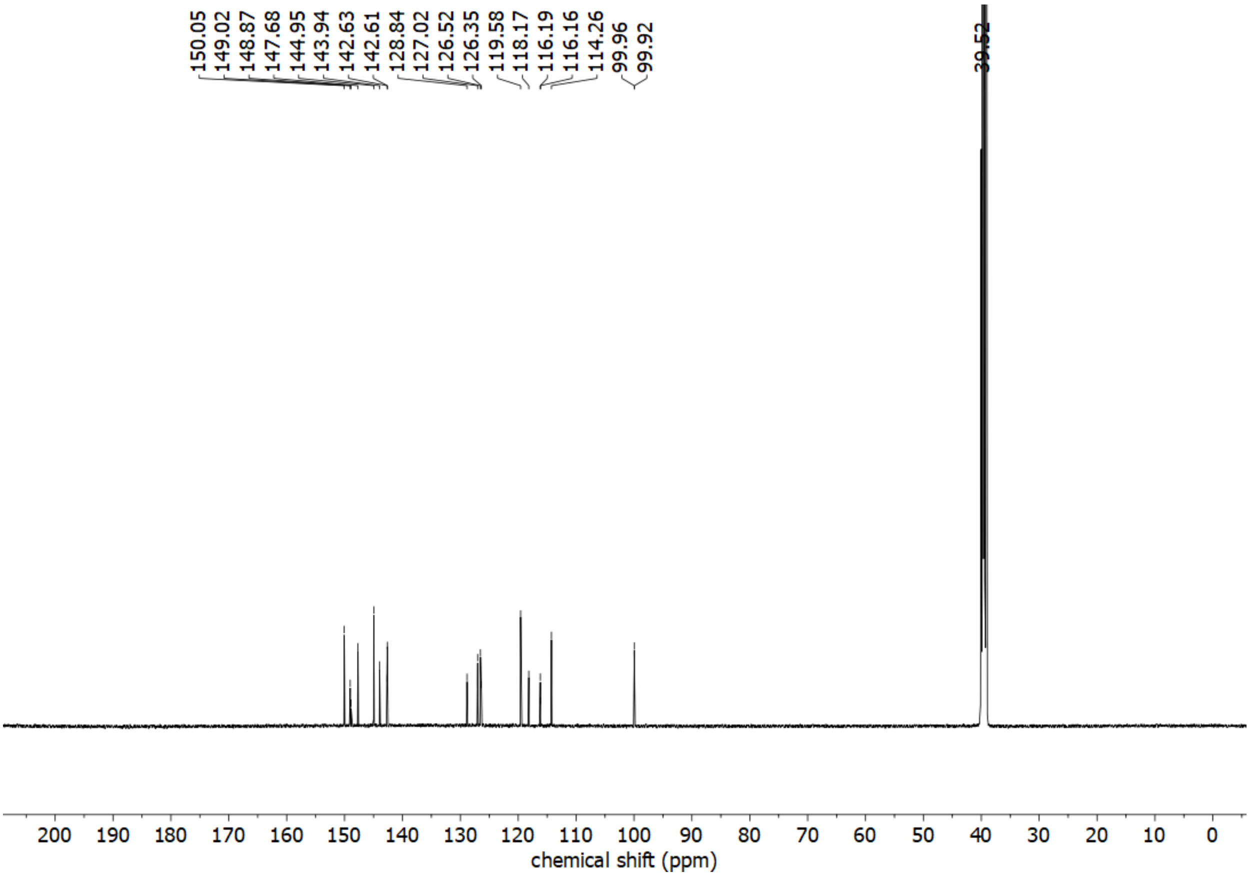

FT-IR spectrum (neat) of **38**.

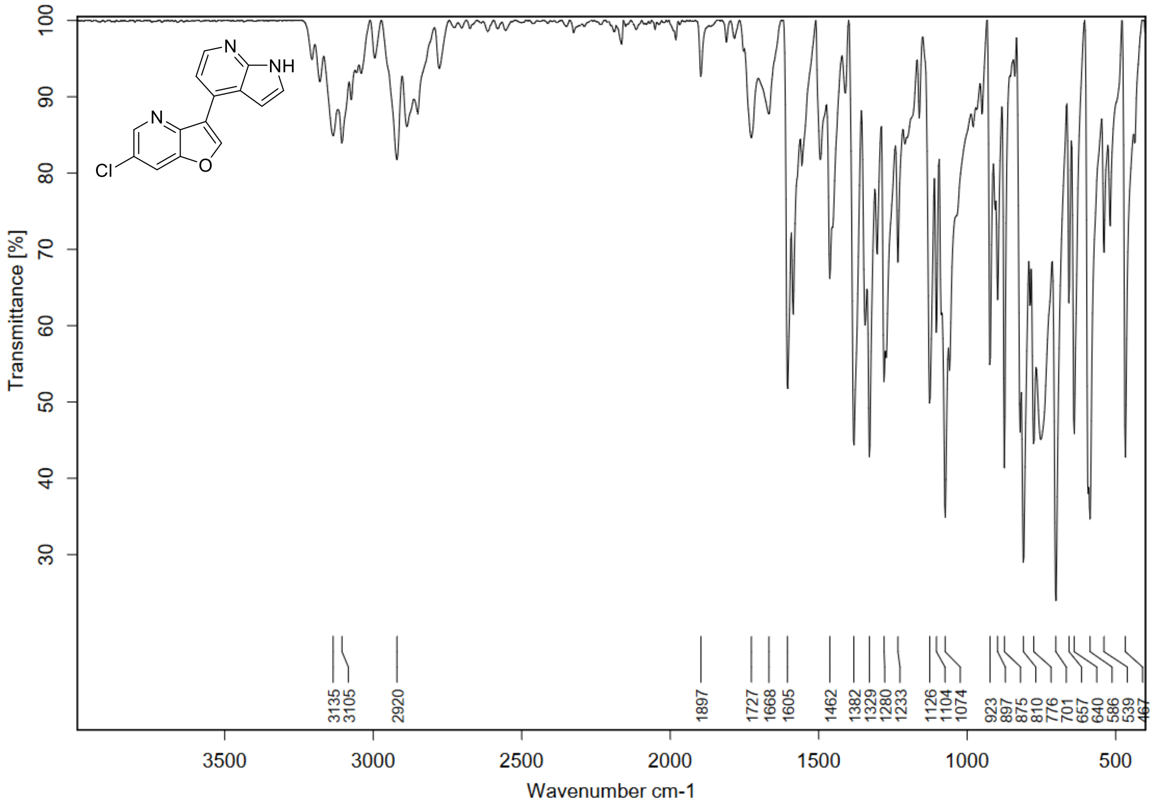

HRMS spectrum of **38.**

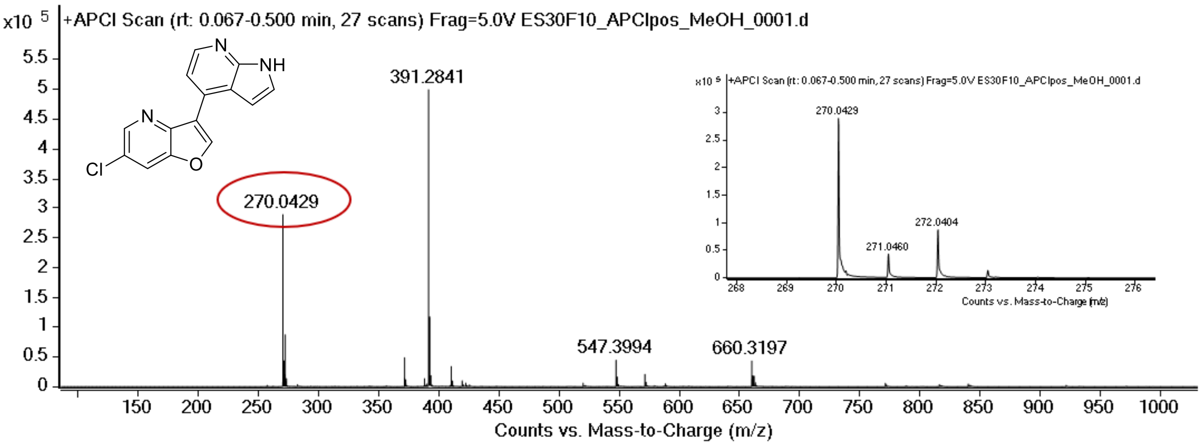

^{1}H (500 MHz) and ^{13}C NMR (126 MHz) spectra of **39** in DMSO-d_6.

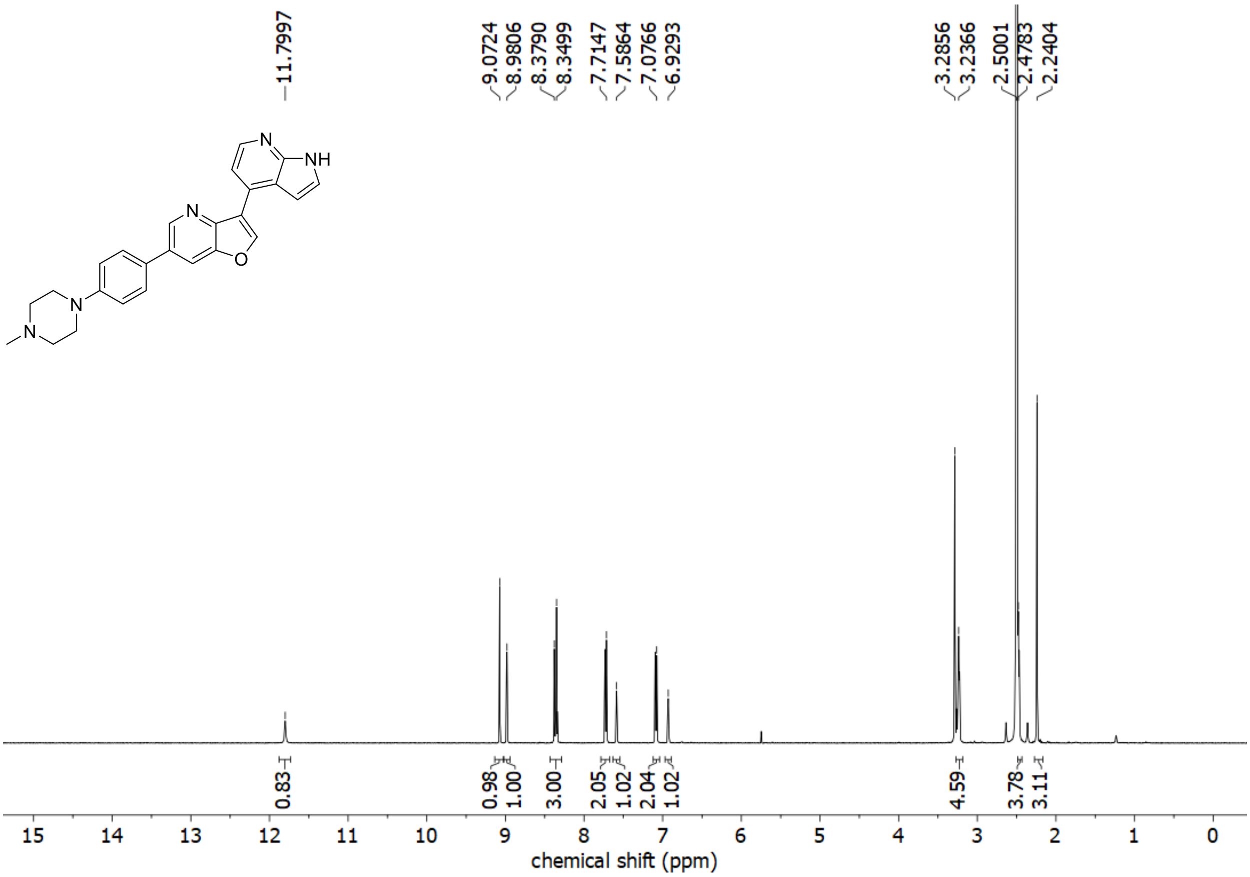

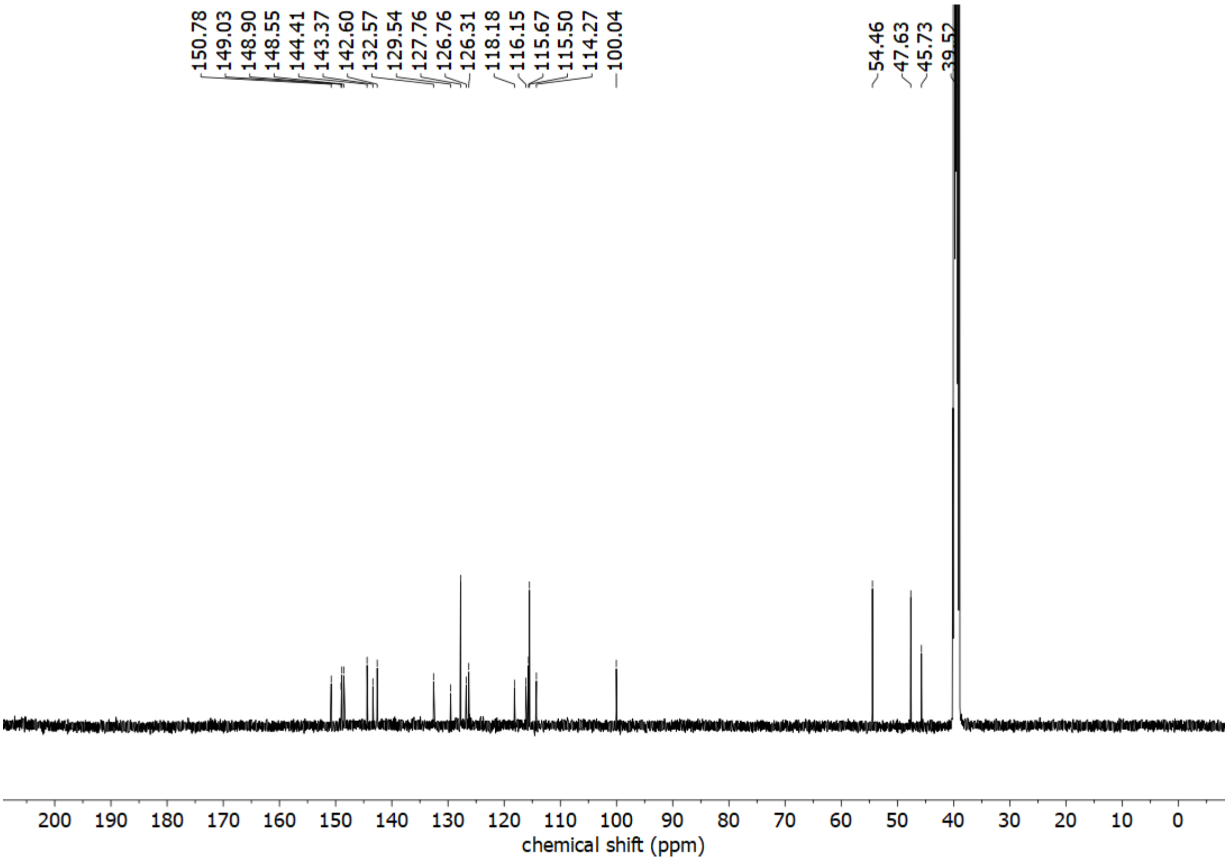

FT-IR spectrum (neat) of **39.**

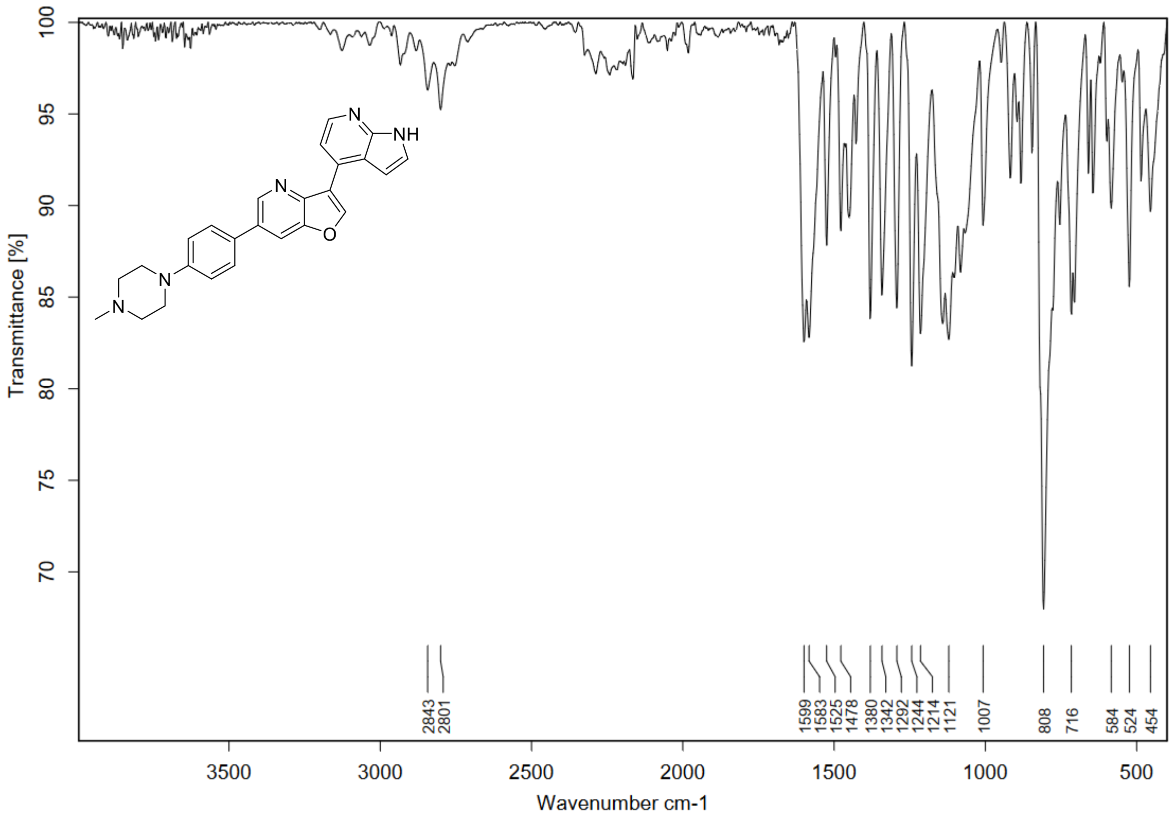

HRMS spectrum of **39.**

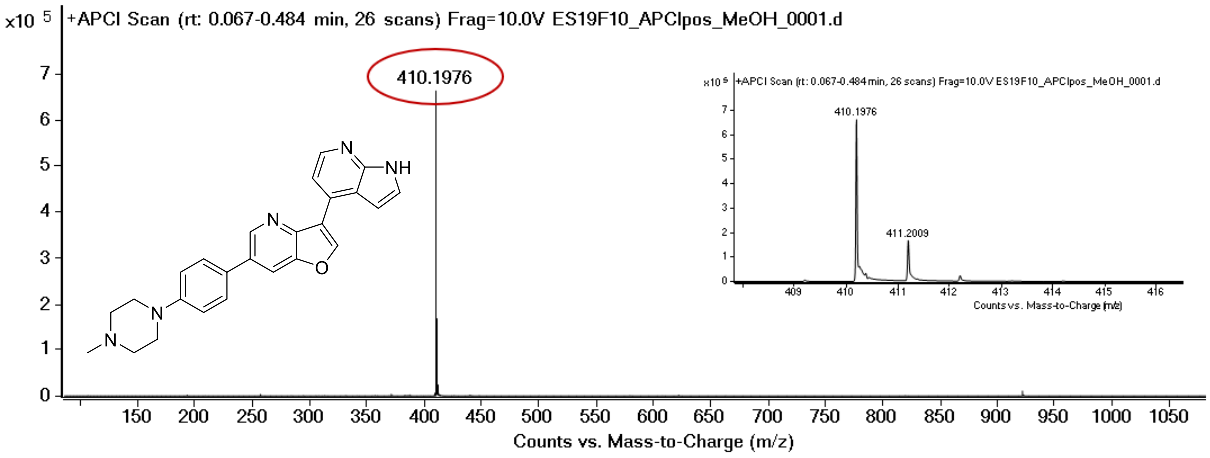

^{1}H (500 MHz) and ^{13}C NMR (126 MHz) spectra of **40** in chloroform-*d*.

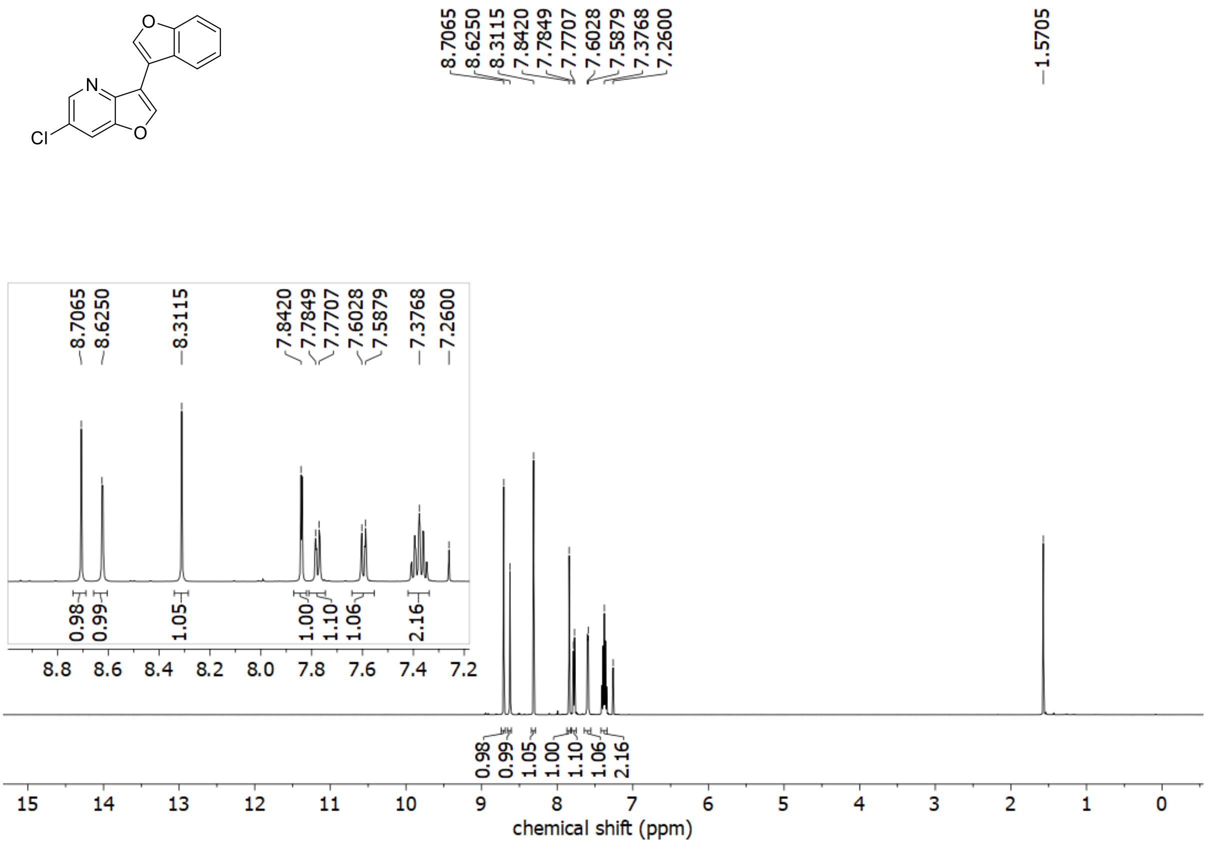

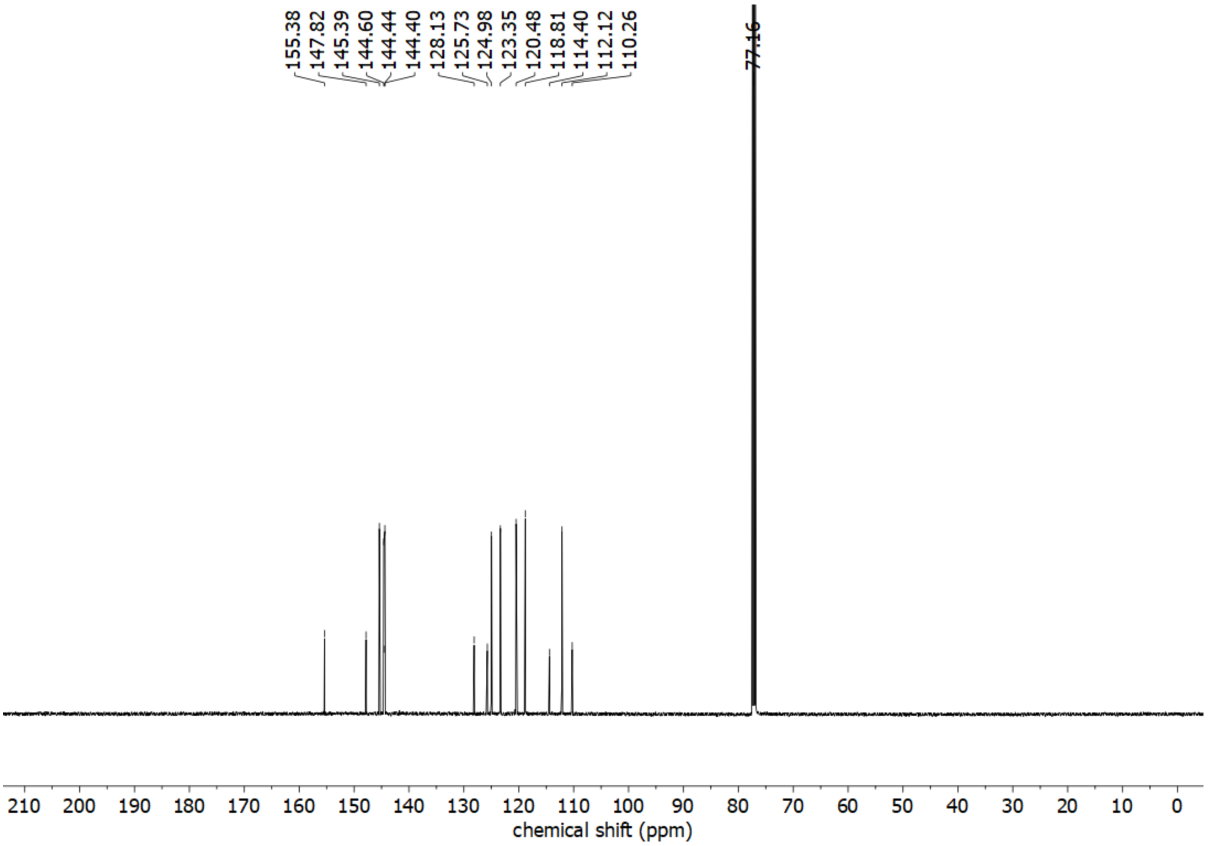

FT-IR spectrum (neat) of **40.**

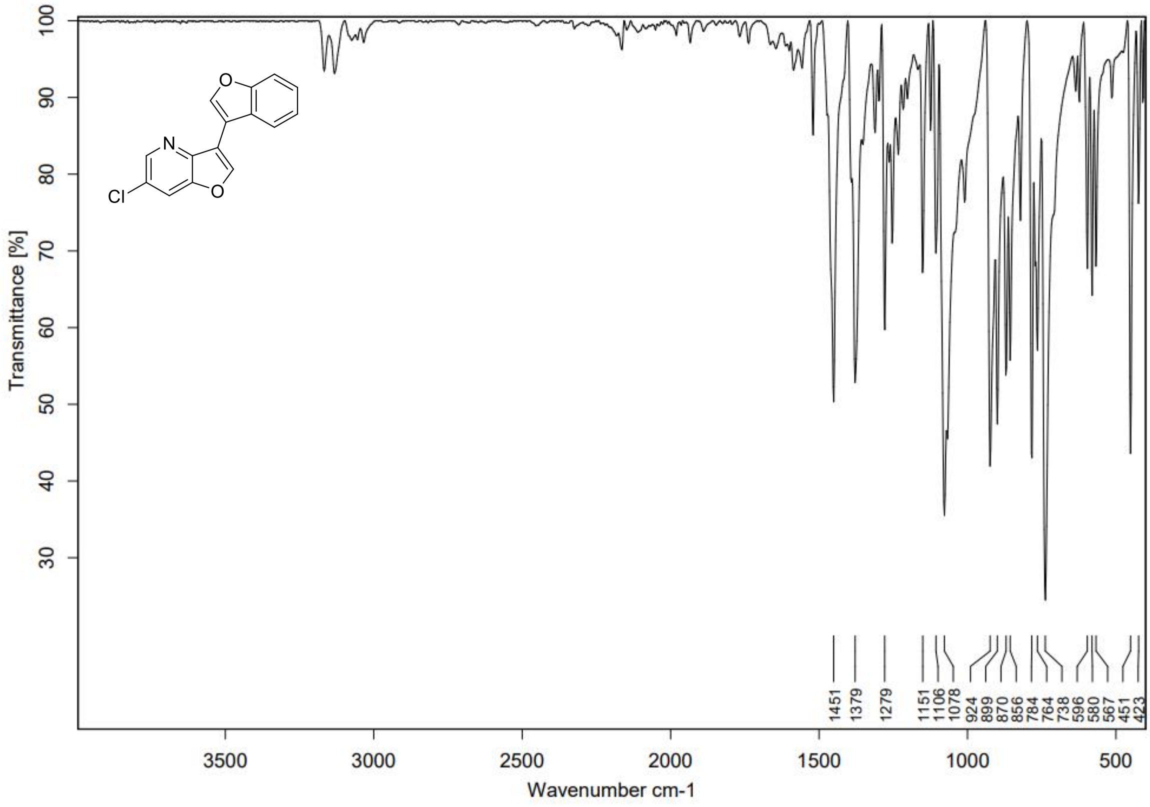

HRMS spectrum of **40**.

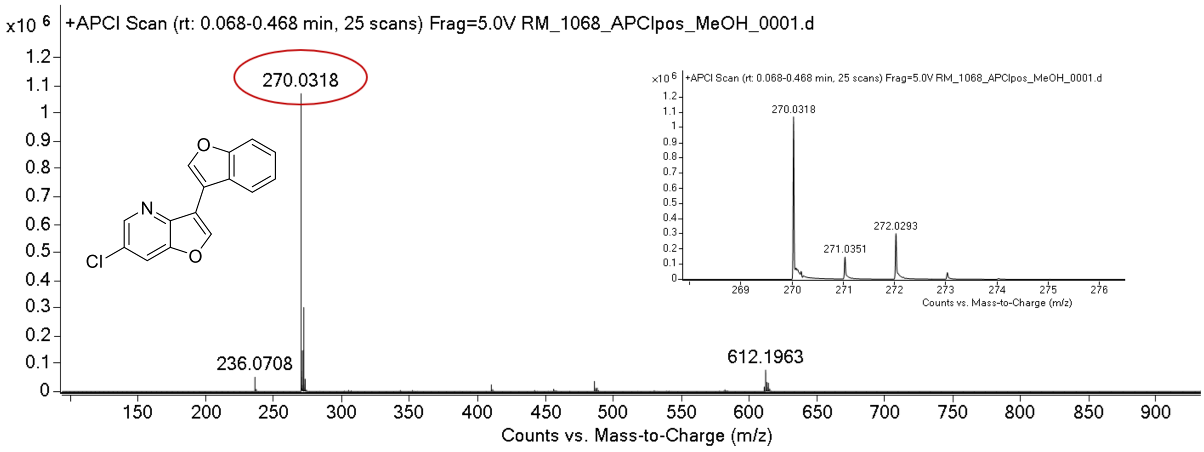

^{1}H (500 MHz) and ^{13}C NMR (126 MHz) spectra of **41** in DMSO-d_6.

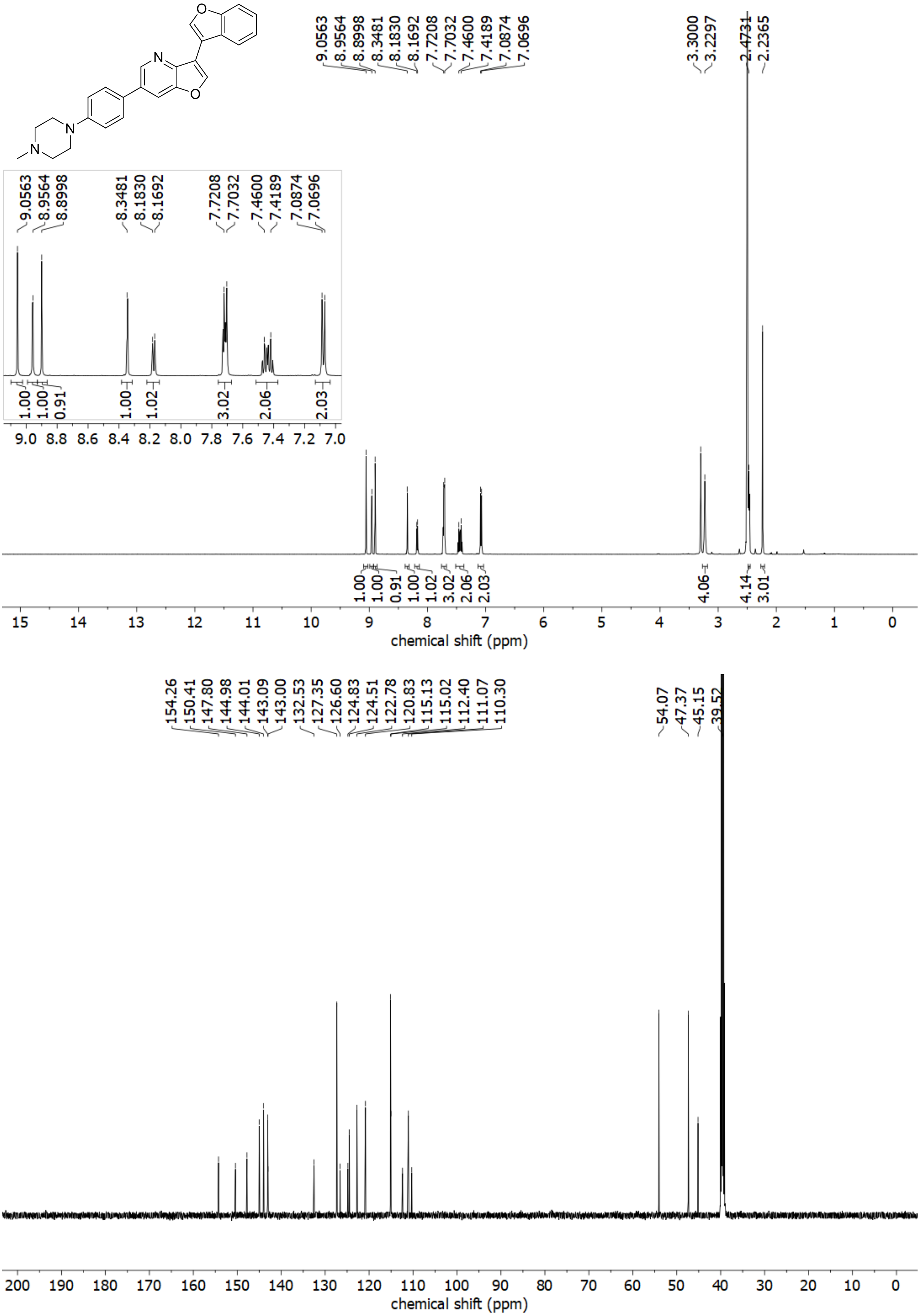

FT-IR spectrum (neat) of **41.**

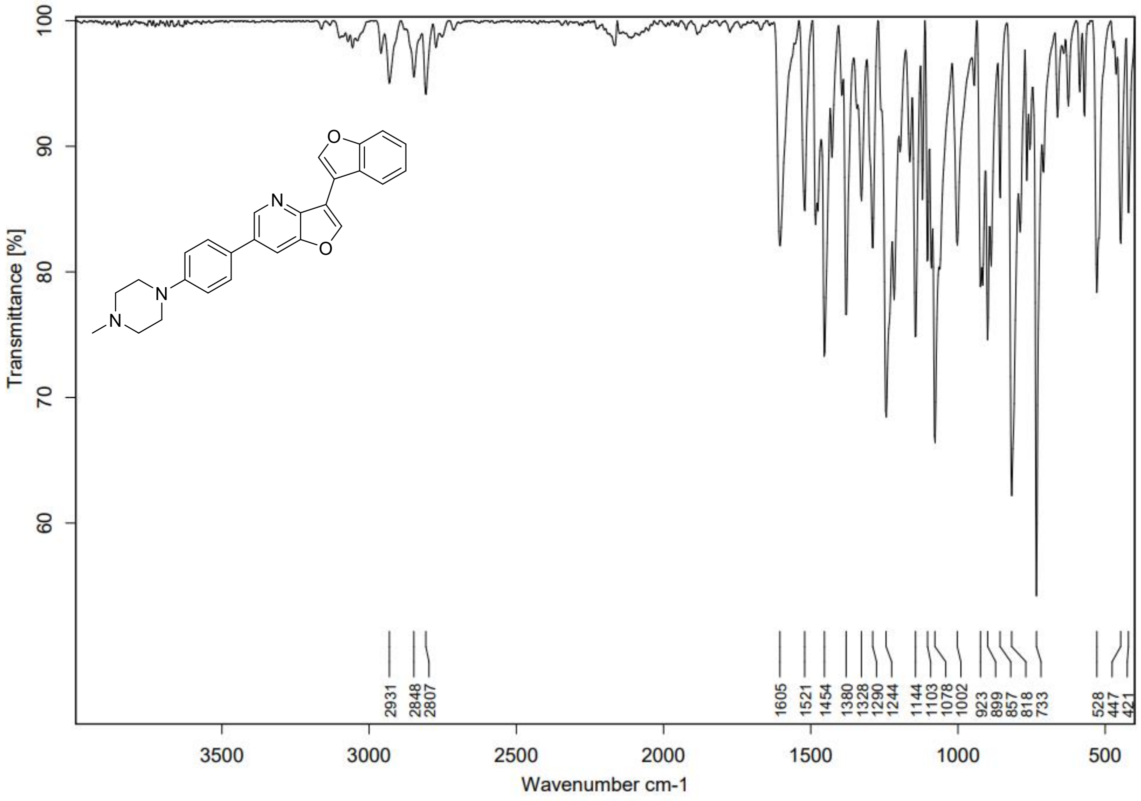

HRMS spectrum of **41.**

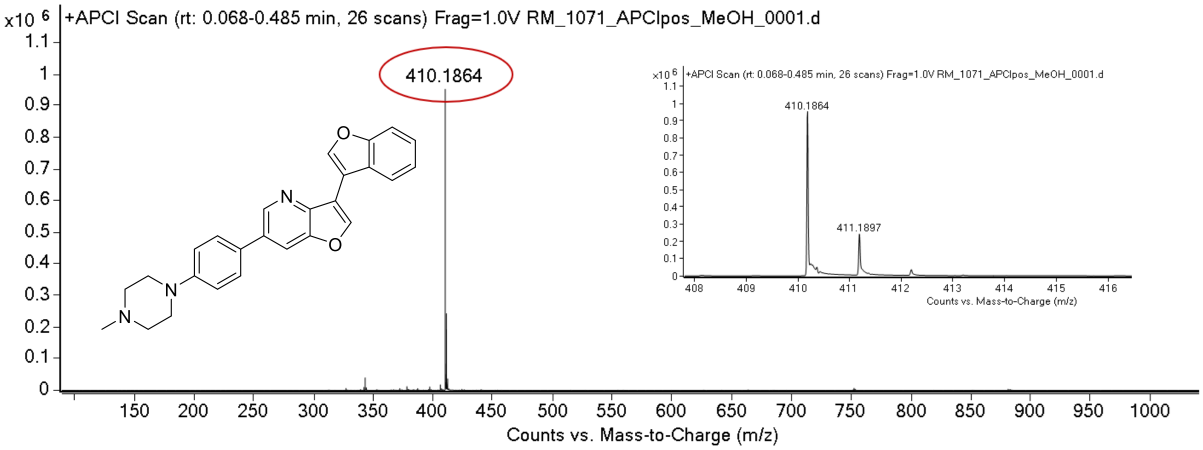

^{1}H (500 MHz) and ^{13}C NMR (126 MHz) spectra of **42** in chloroform-*d*.

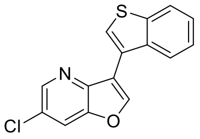

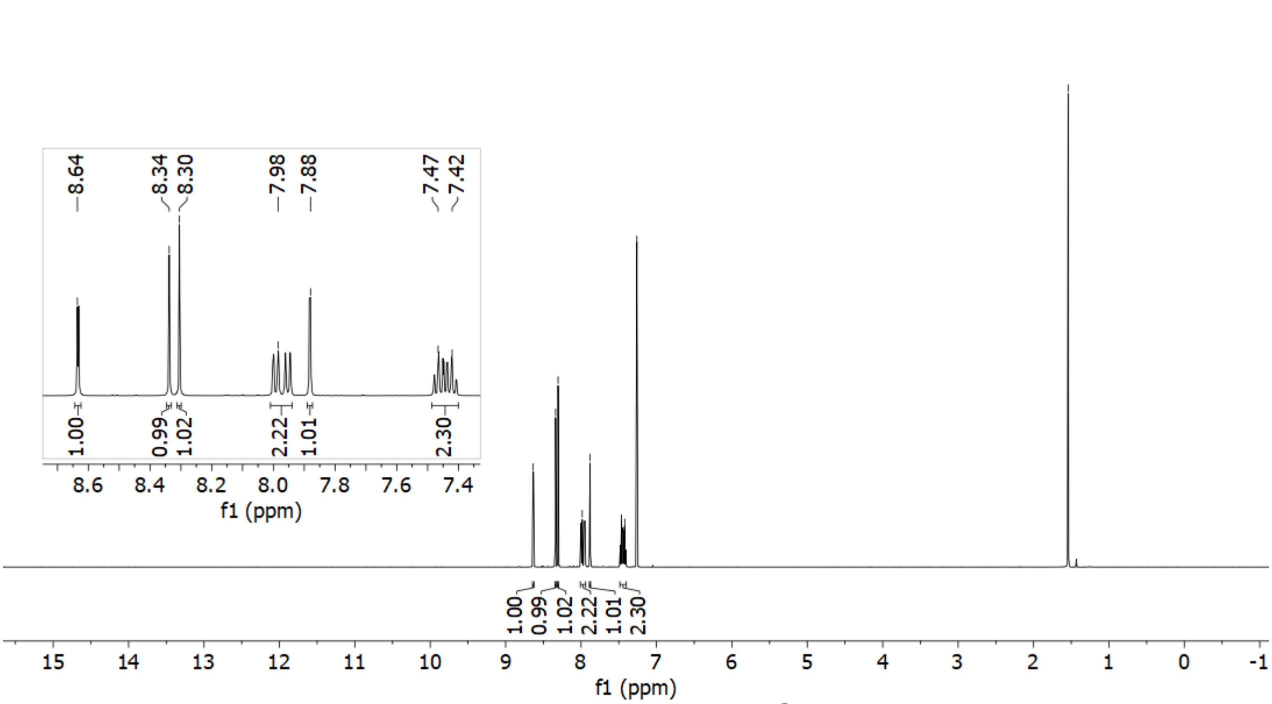

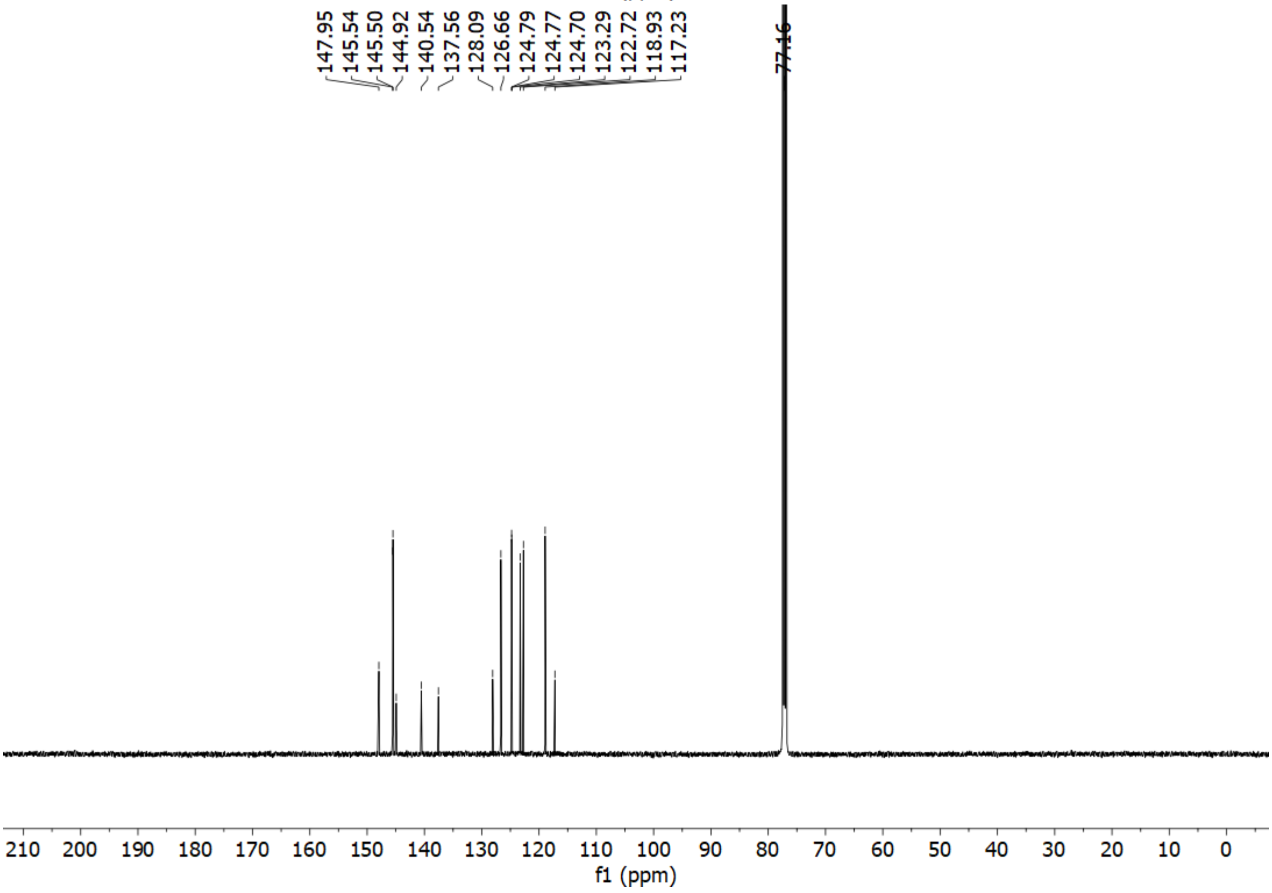

FT-IR spectrum (neat) of **42**

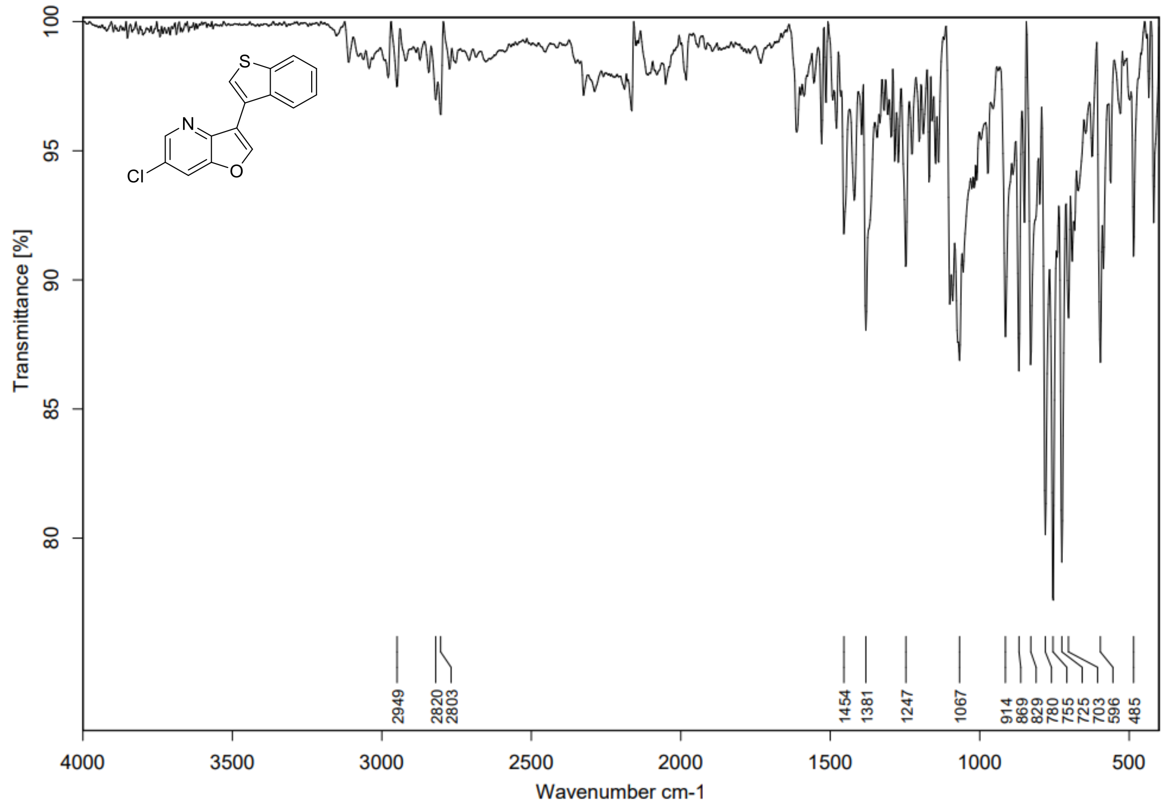

HRMS spectrum of **42.**

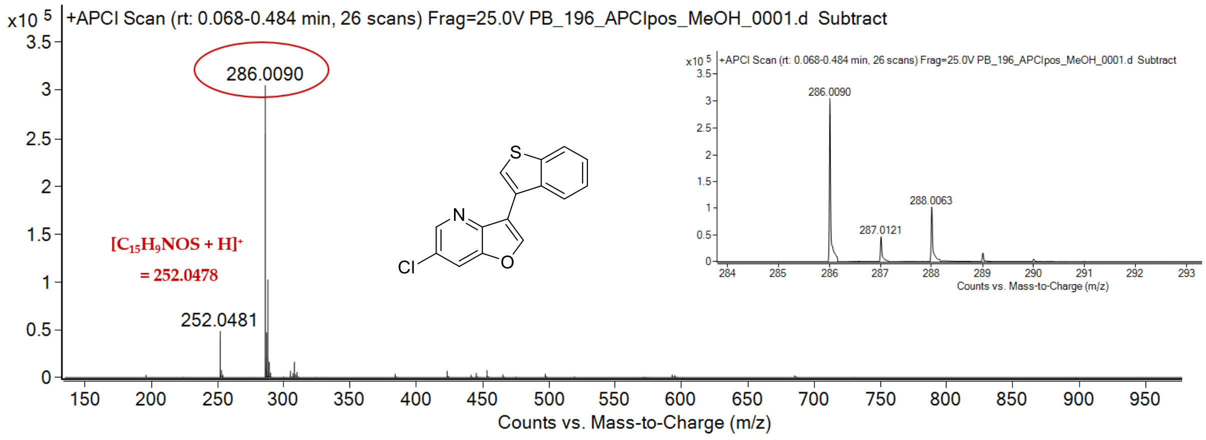

^{1}H (500 MHz) and ^{13}C NMR (126 MHz) spectra of **43** in DMSO-d_6.

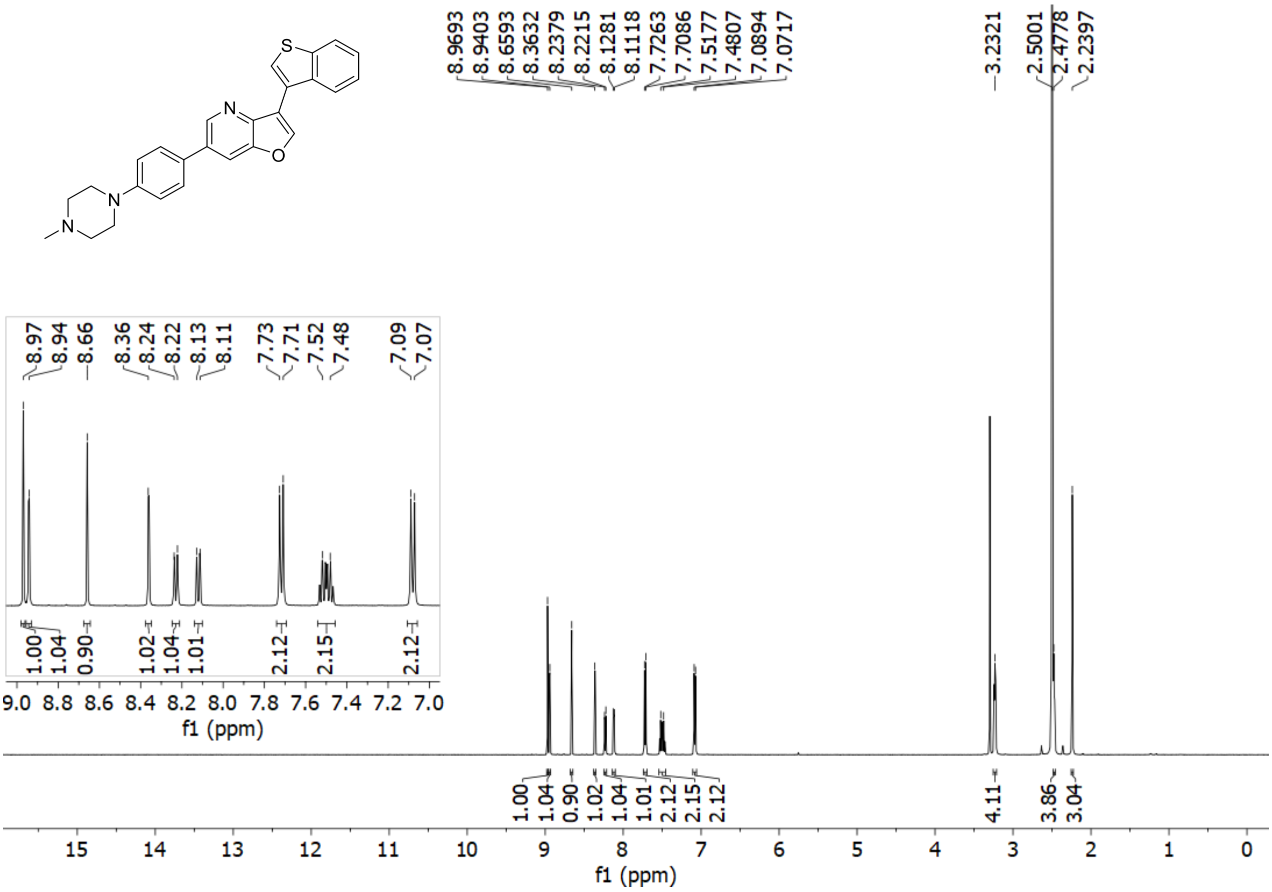

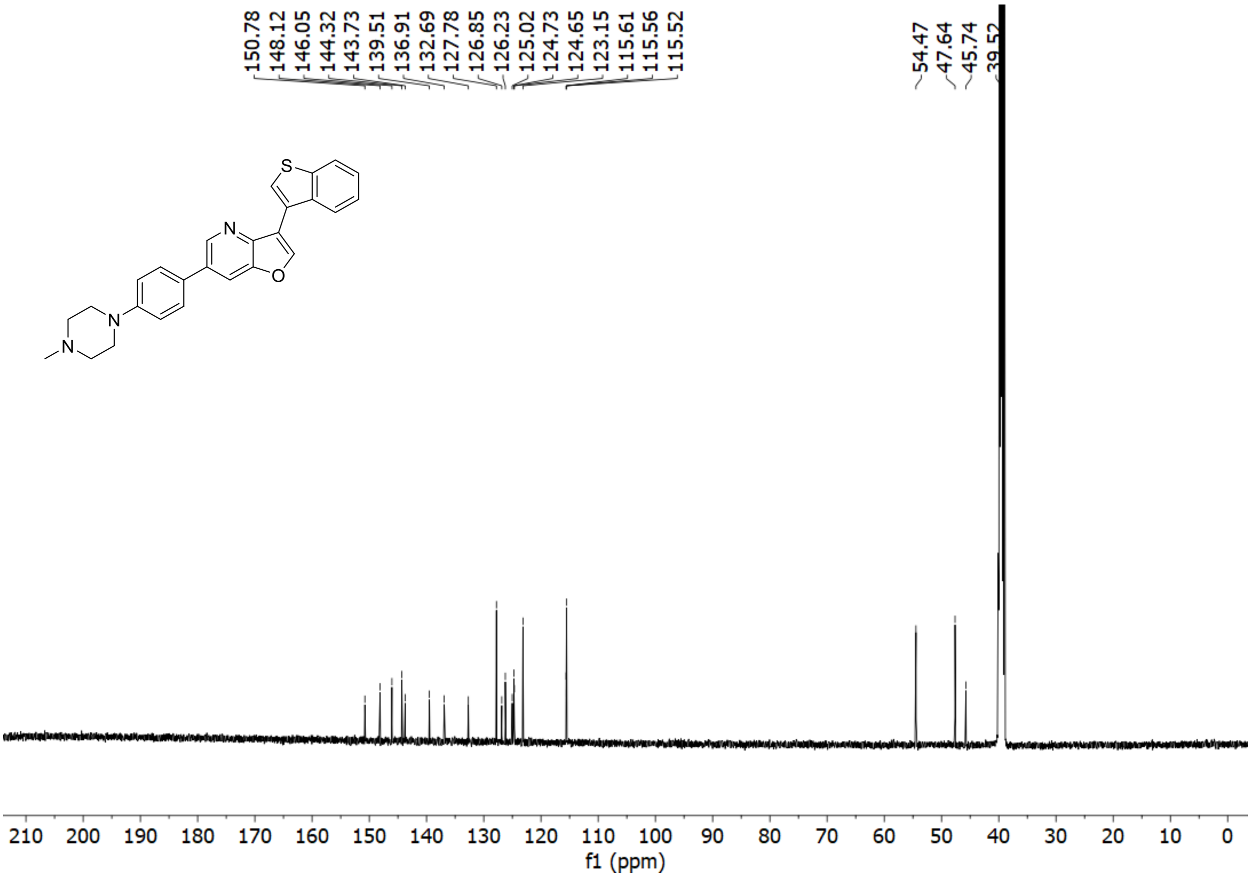

FT-IR spectrum (neat) of **43**.

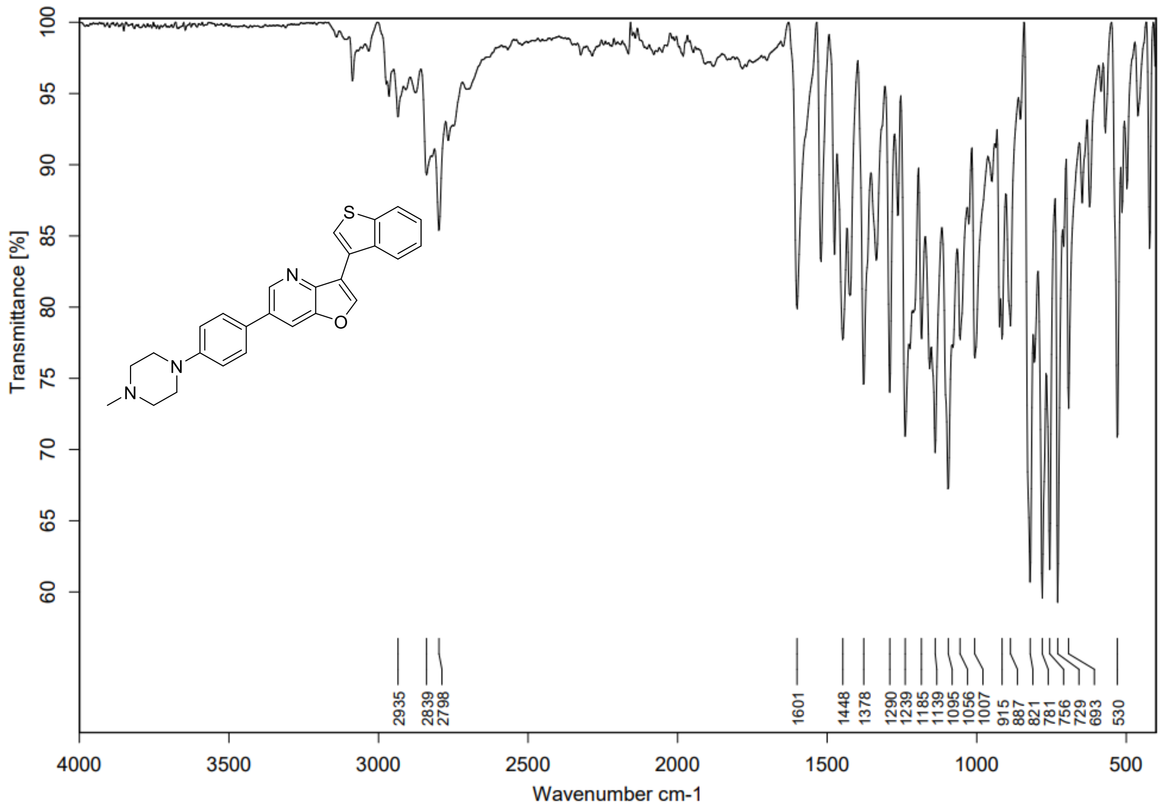

HRMS spectrum of **43.**

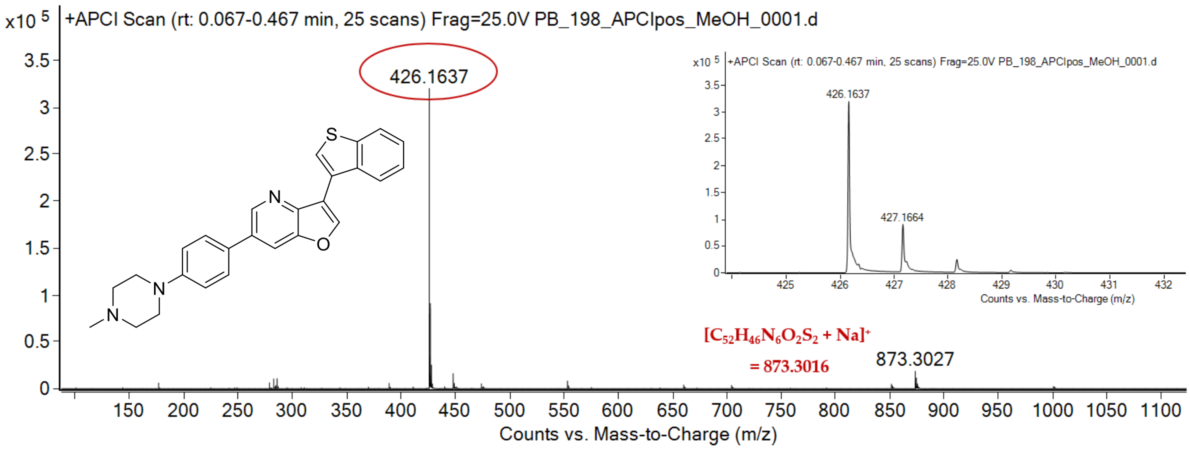

^{1}H (500 MHz) and ^{13}C NMR (126 MHz) spectra of **44** in chloroform-*d*.

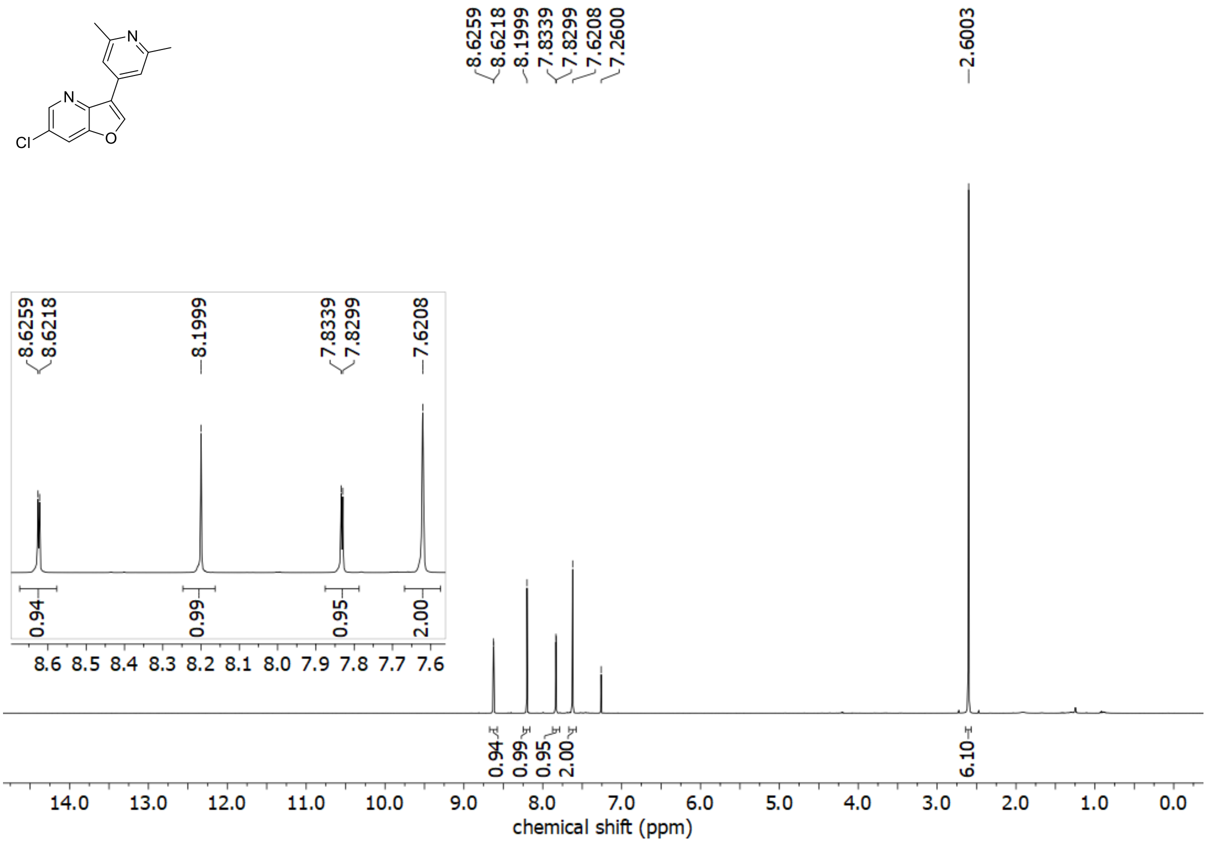

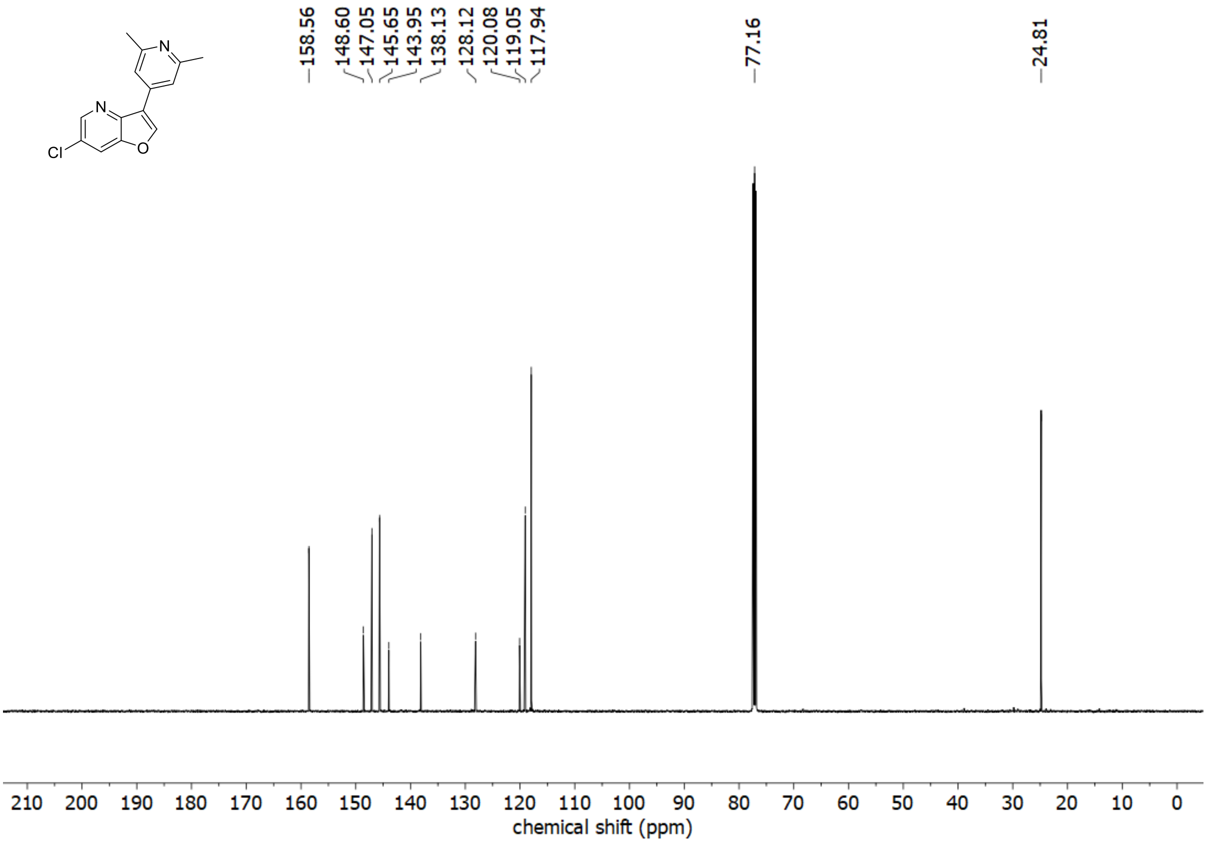

FT-IR spectrum (neat) of **44**.

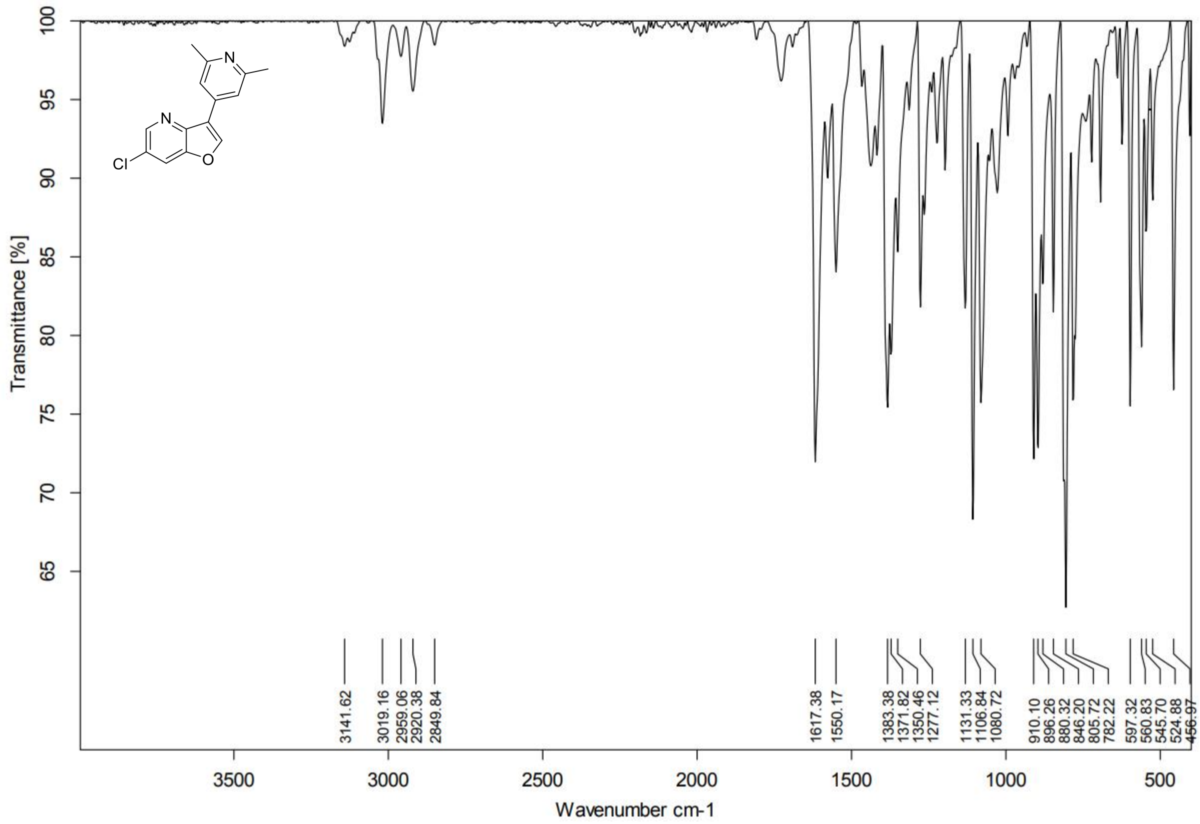

HRMS spectrum of **44**.

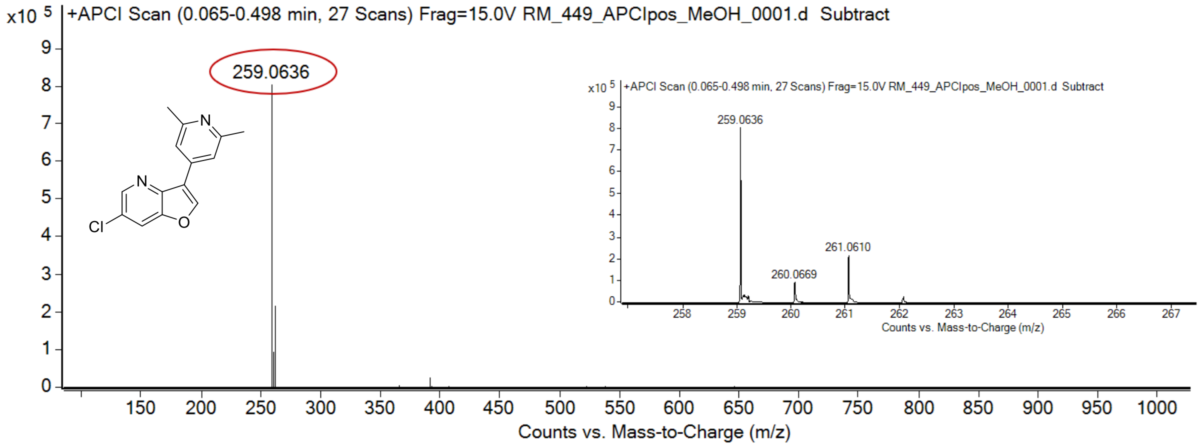

^{1}H (500 MHz) and ^{13}C NMR (126 MHz) spectra of **45** in chloroform-*d*.

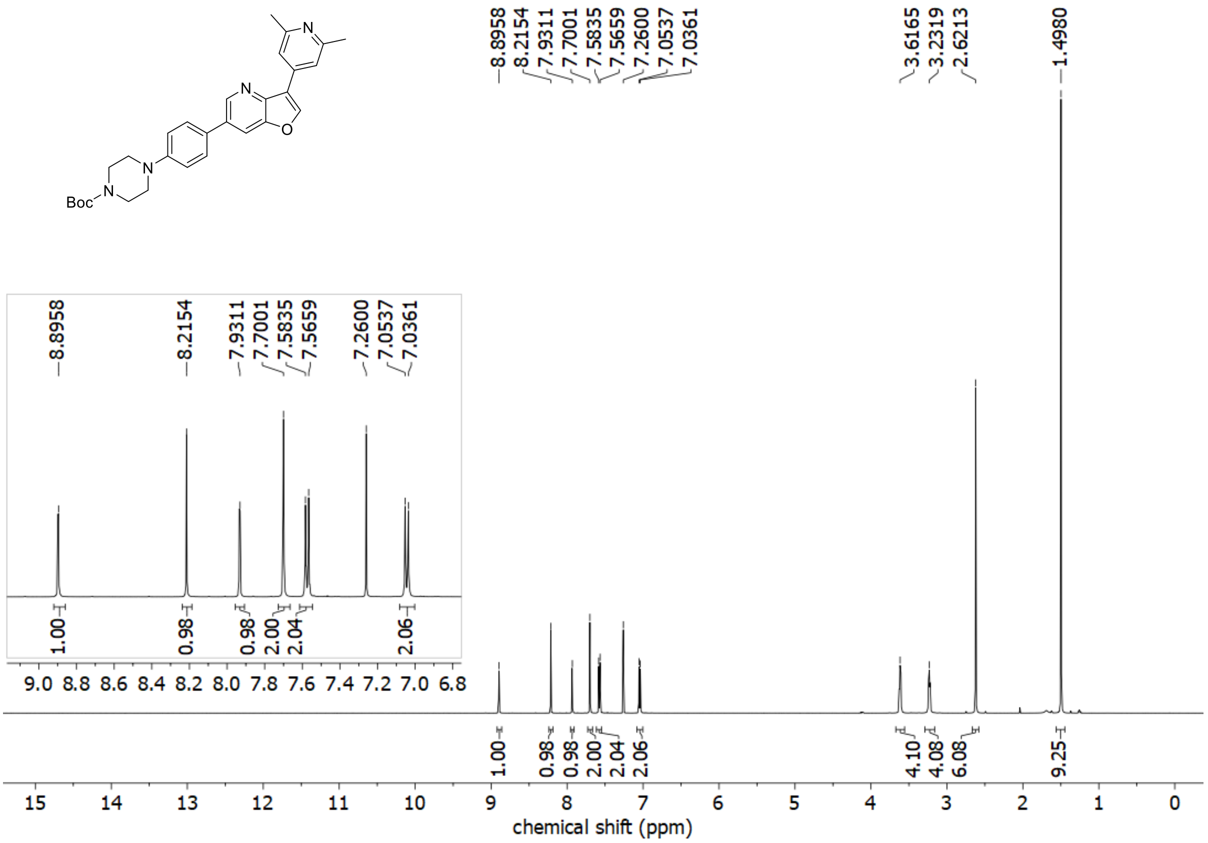

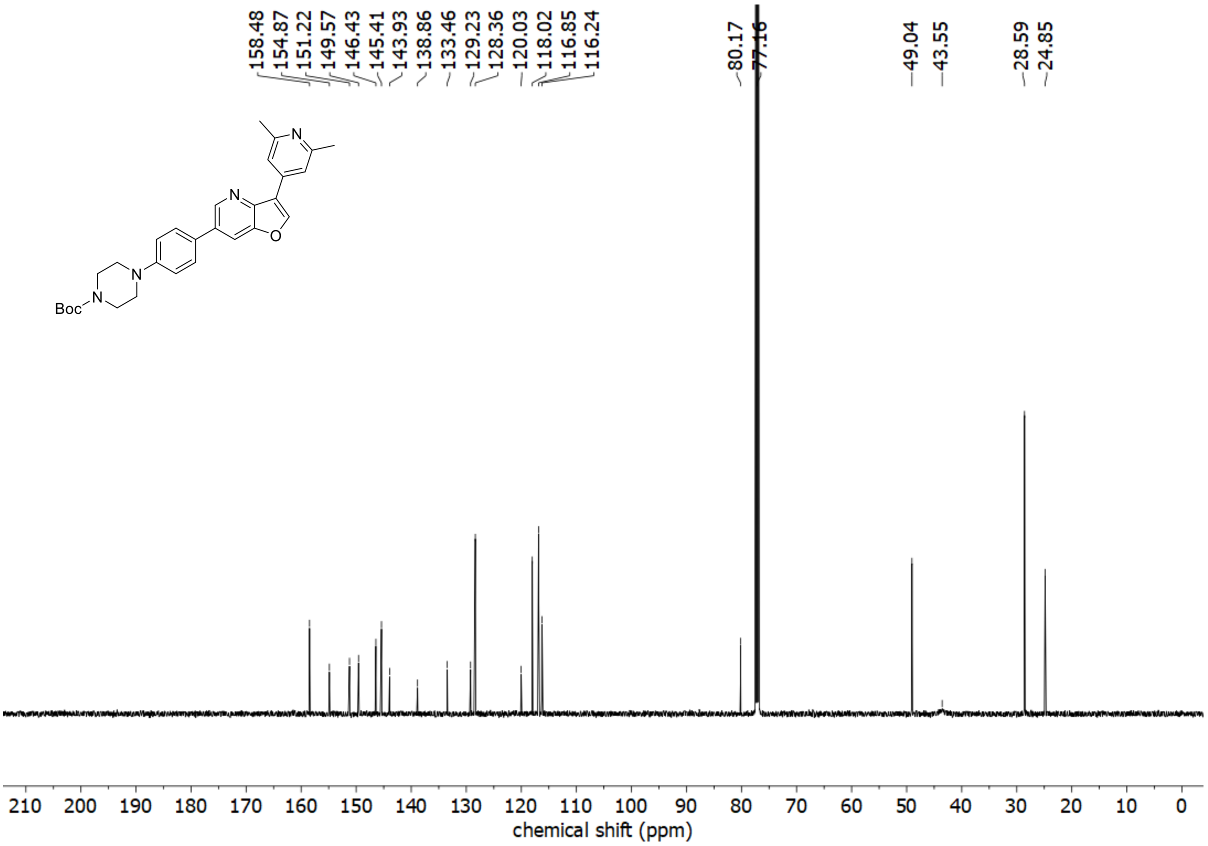

FT-IR spectrum (neat) of **45**.

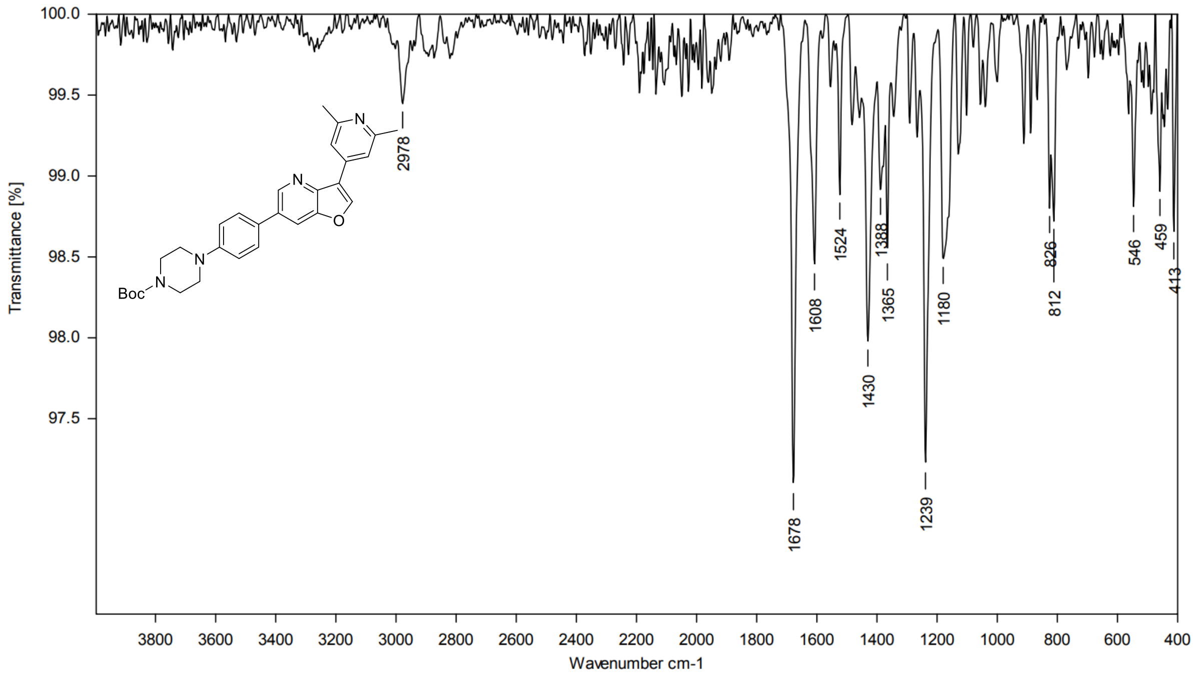

HRMS spectrum of **45**.

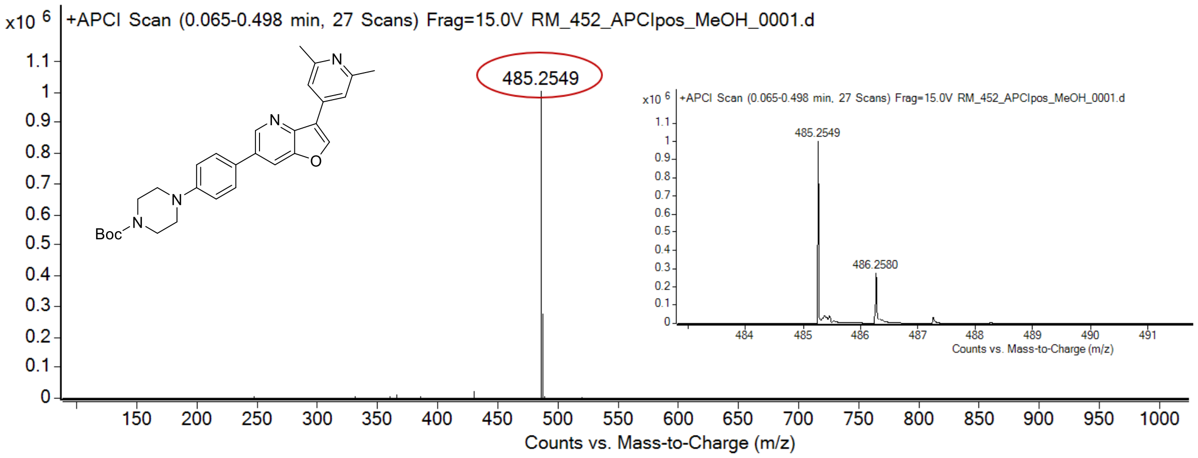

^{1}H (500 MHz) and ^{13}C NMR (126 MHz) spectra of **46** in MeOD-d_4.

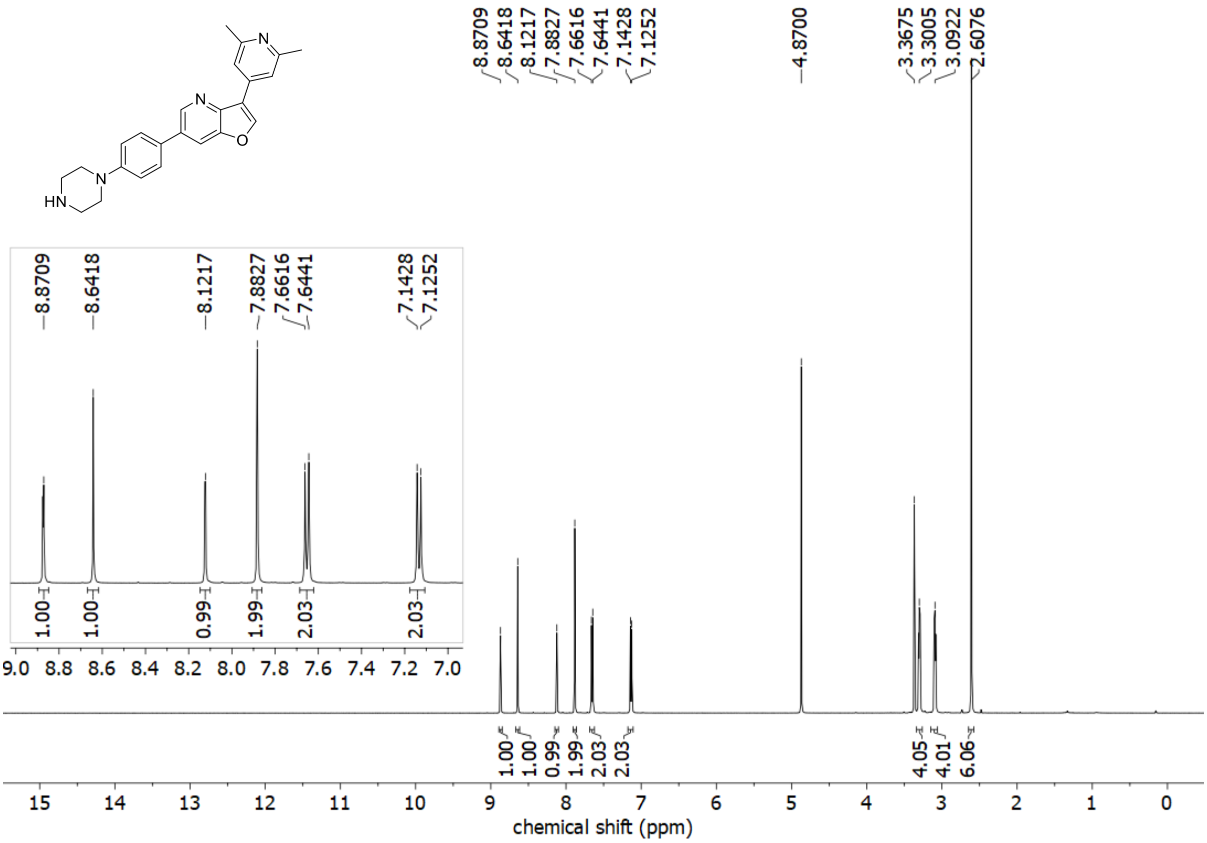

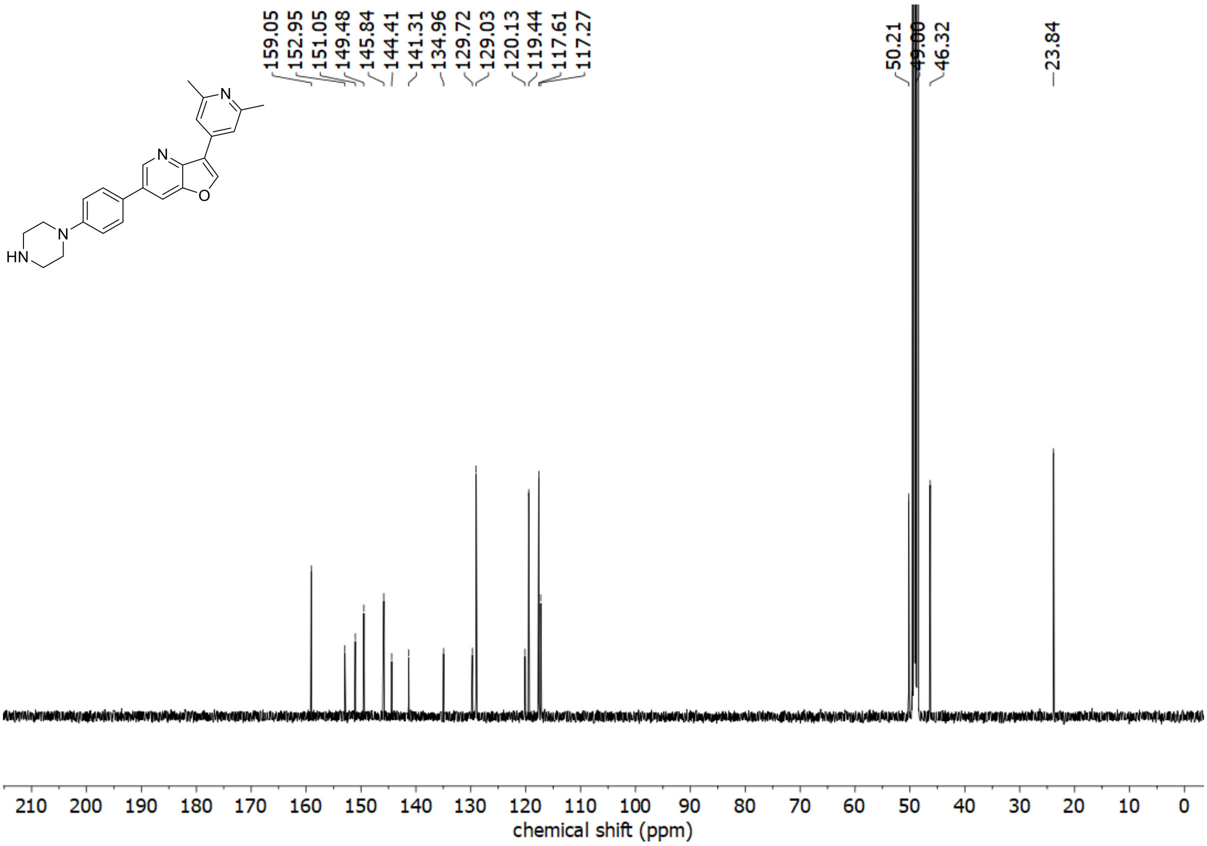

FT-IR spectrum (neat) of **46**.

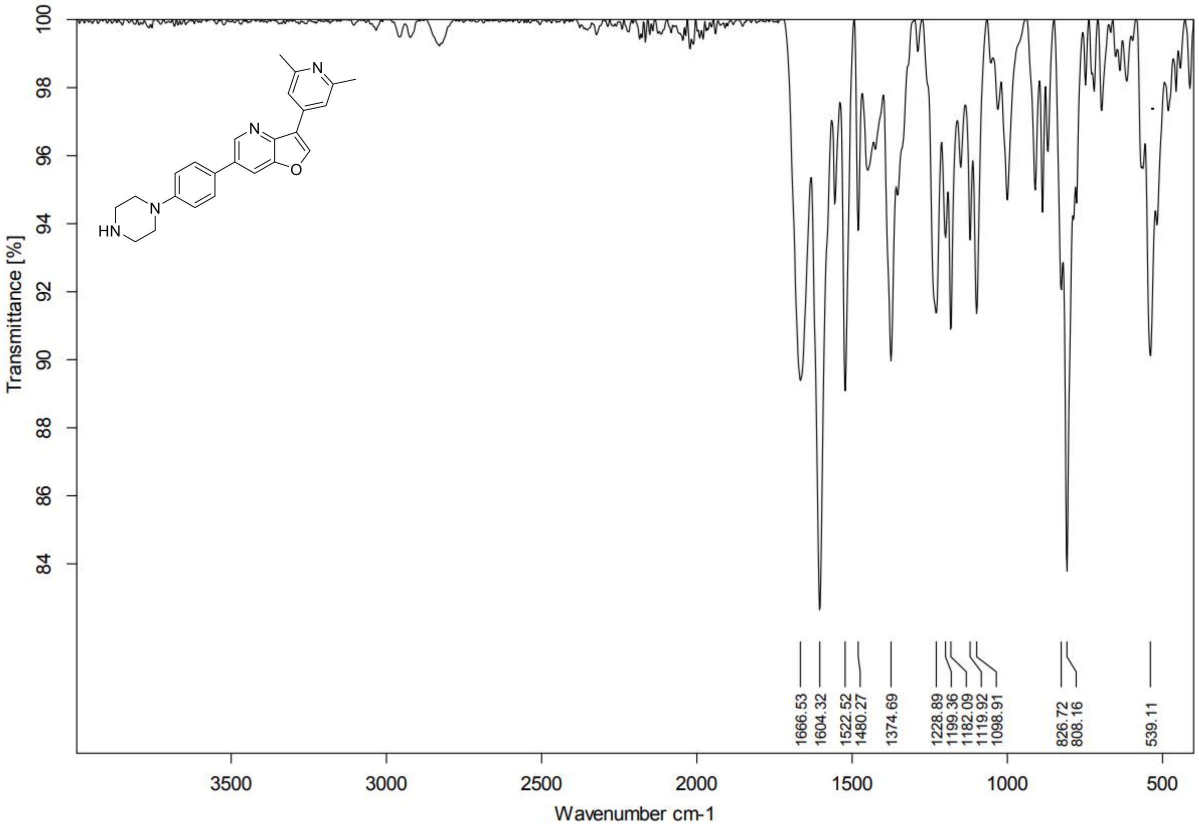

HRMS spectrum of **46**.

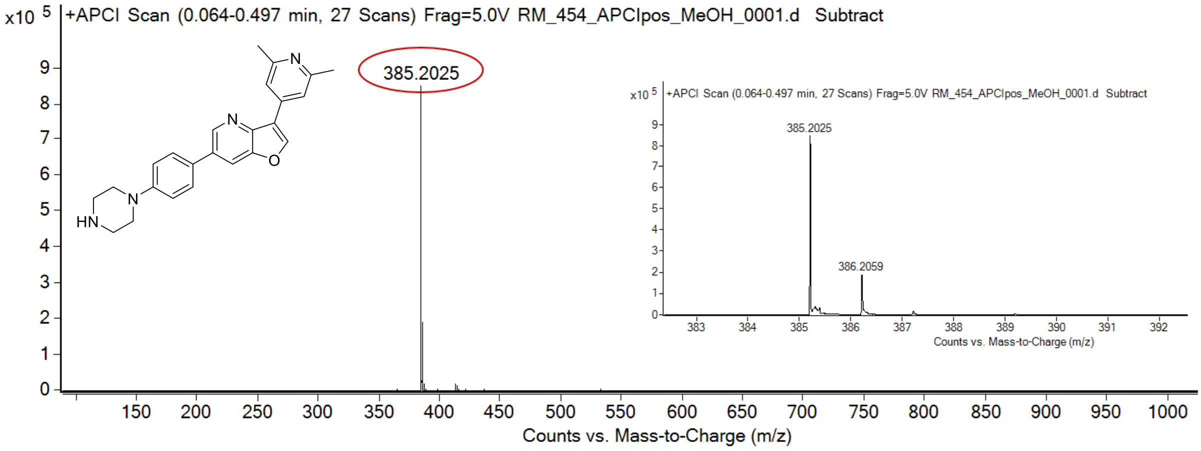

^{1}H (500 MHz) and ^{13}C NMR (126 MHz) spectra of **47** in chloroform-*d*.

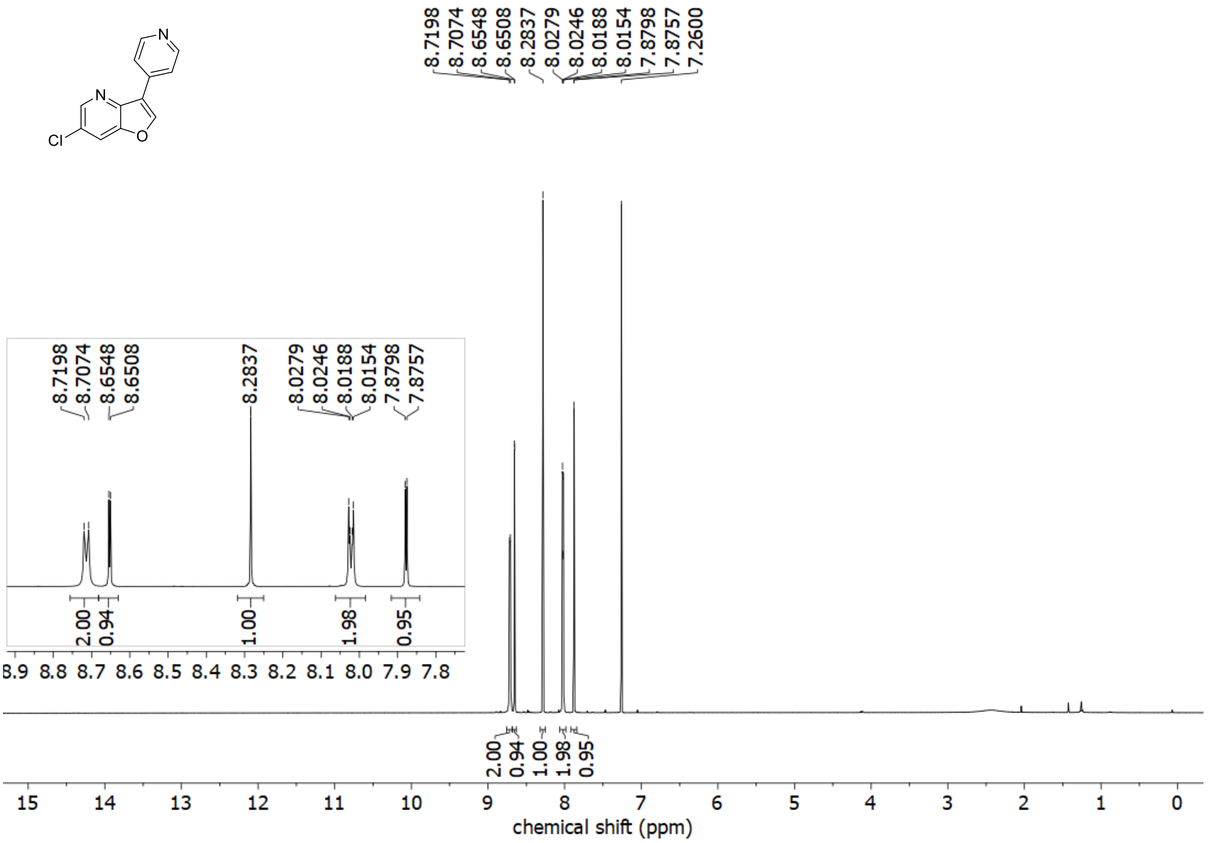

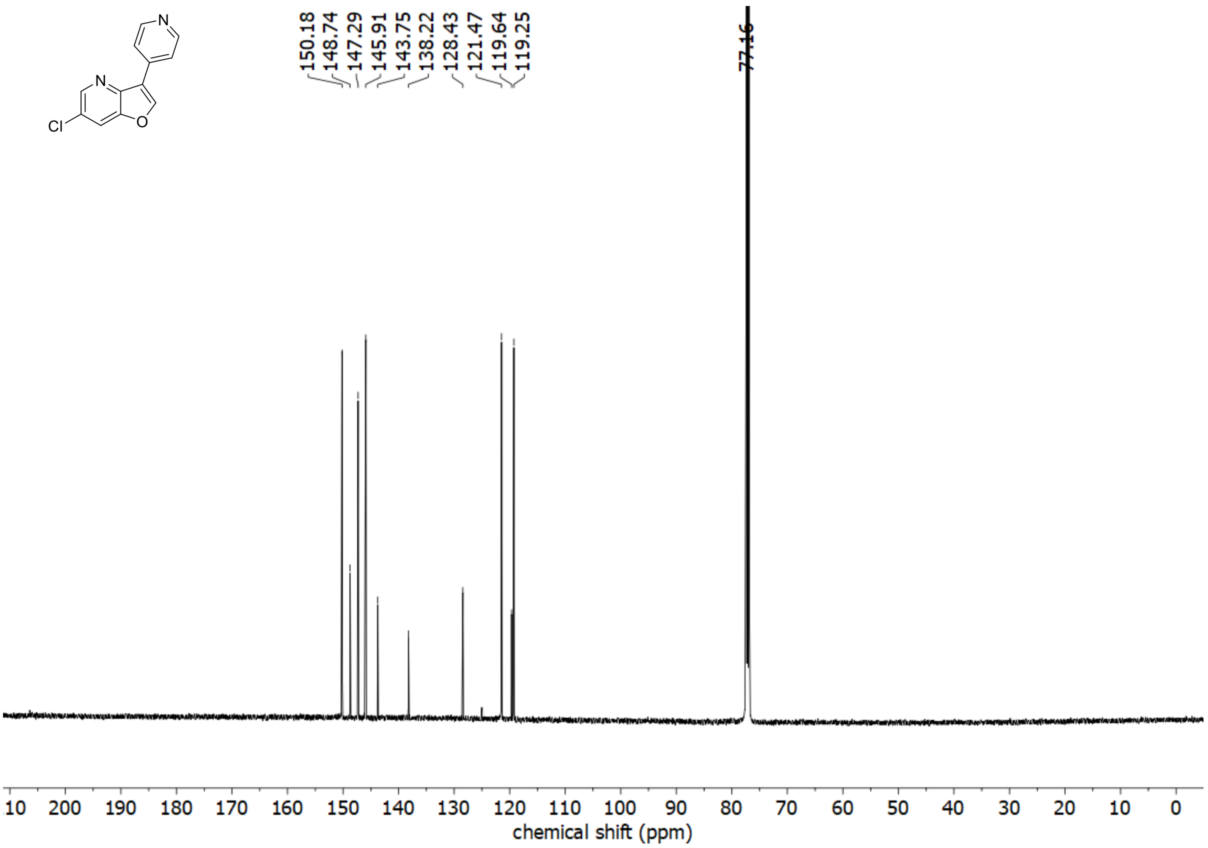

FT-IR spectrum (neat) of **47**.

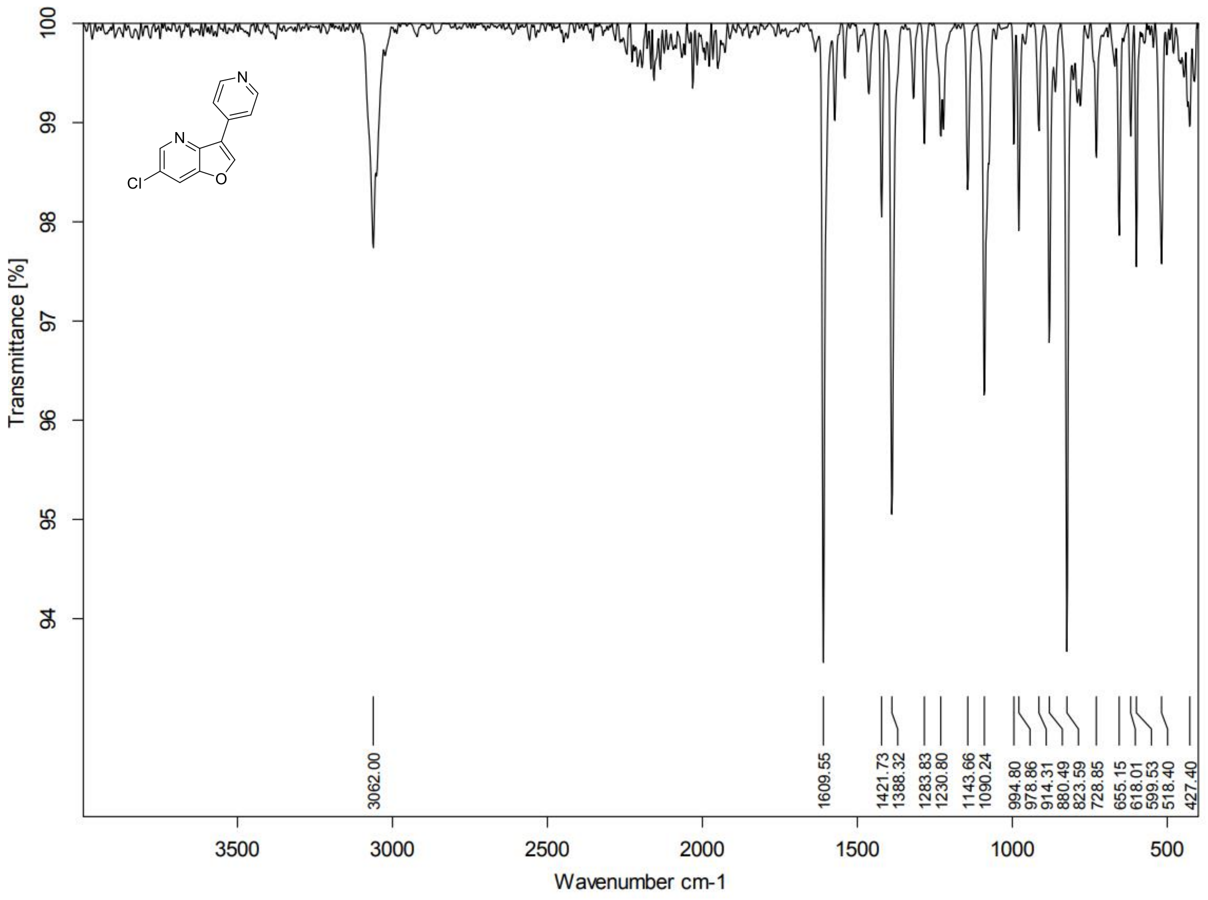

HRMS spectrum of **47**.

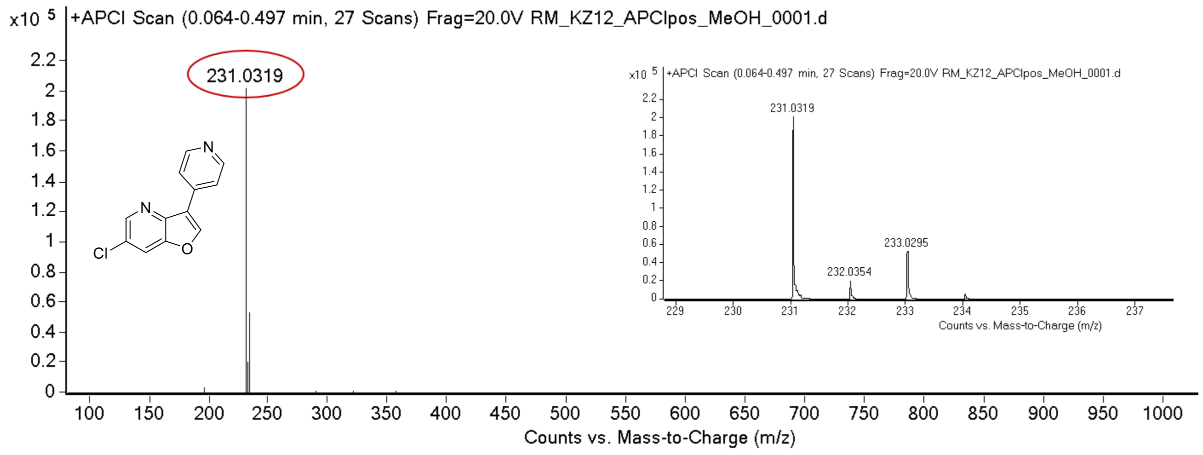

^{1}H (500 MHz) and ^{13}C NMR (126 MHz) spectra of **48** in chloroform-*d*.

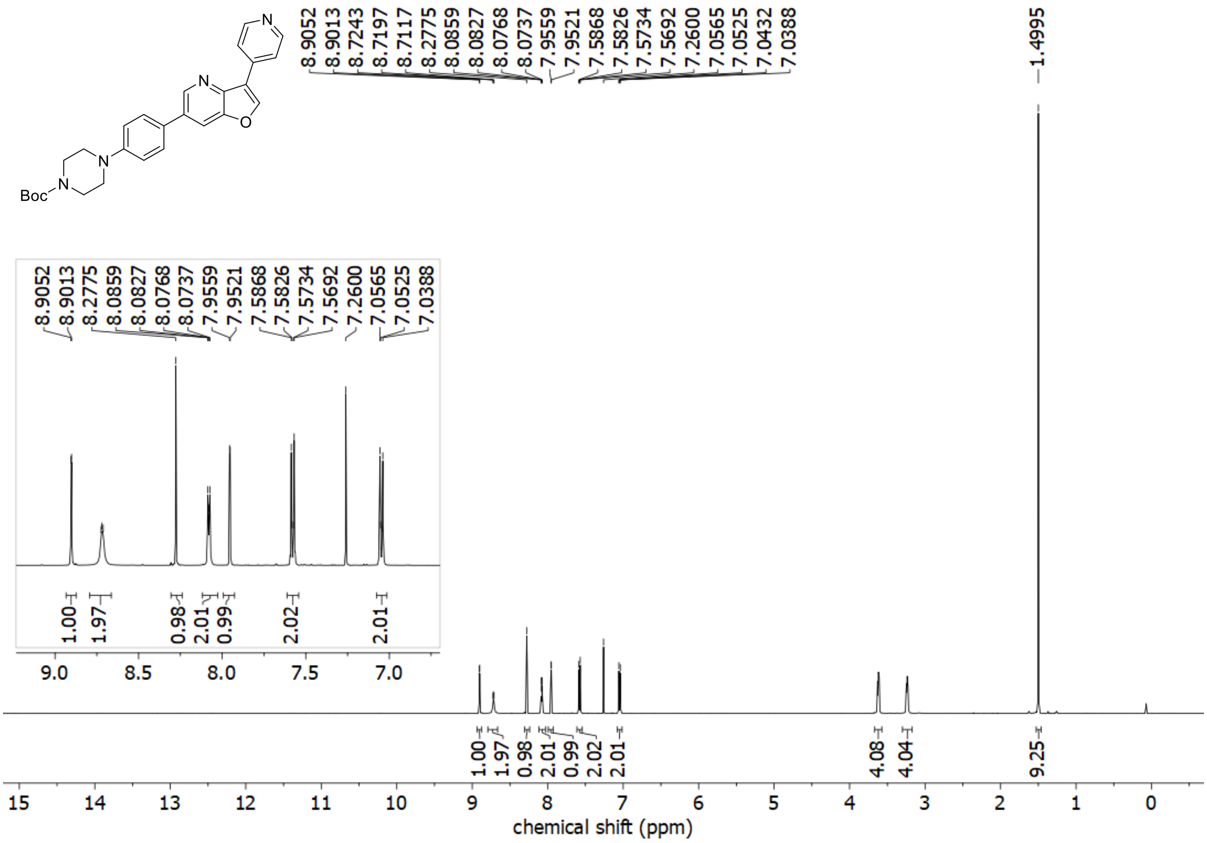

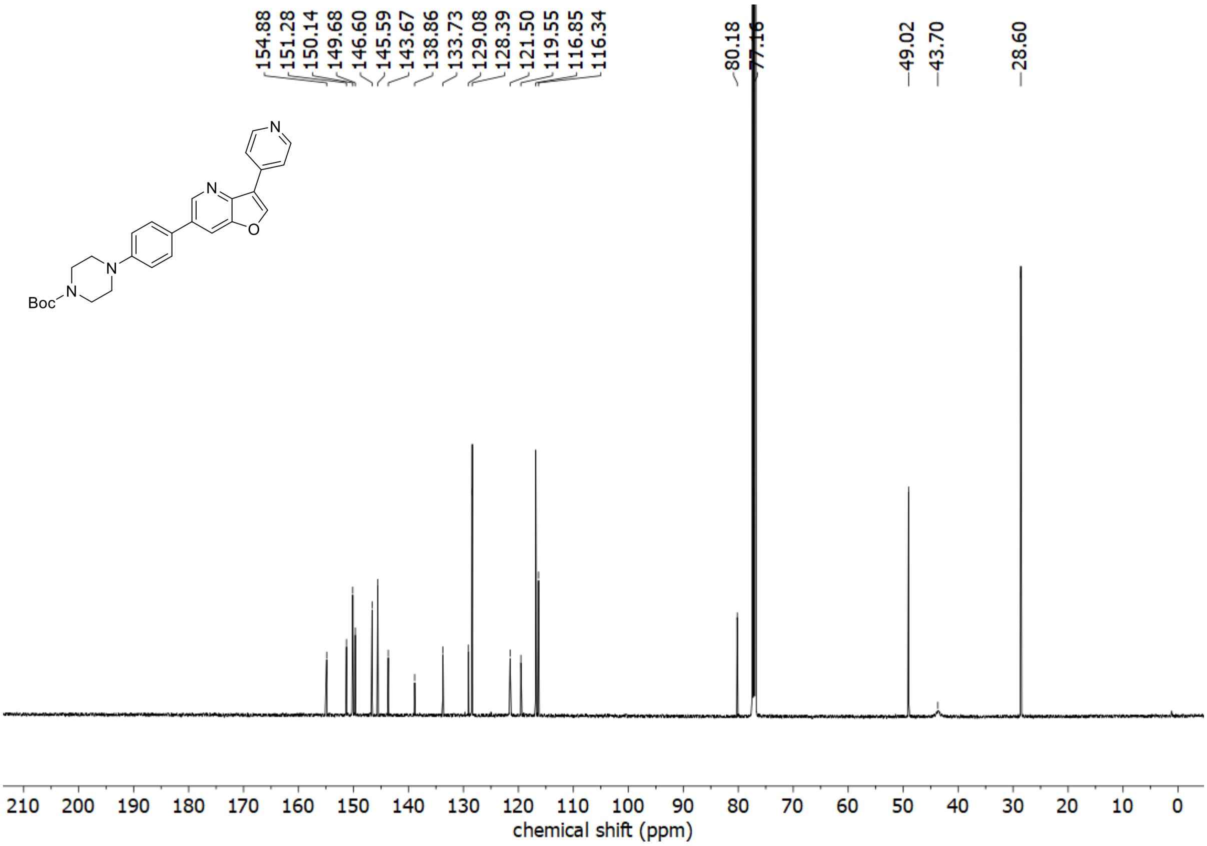

FT-IR spectrum (neat) of **48**.

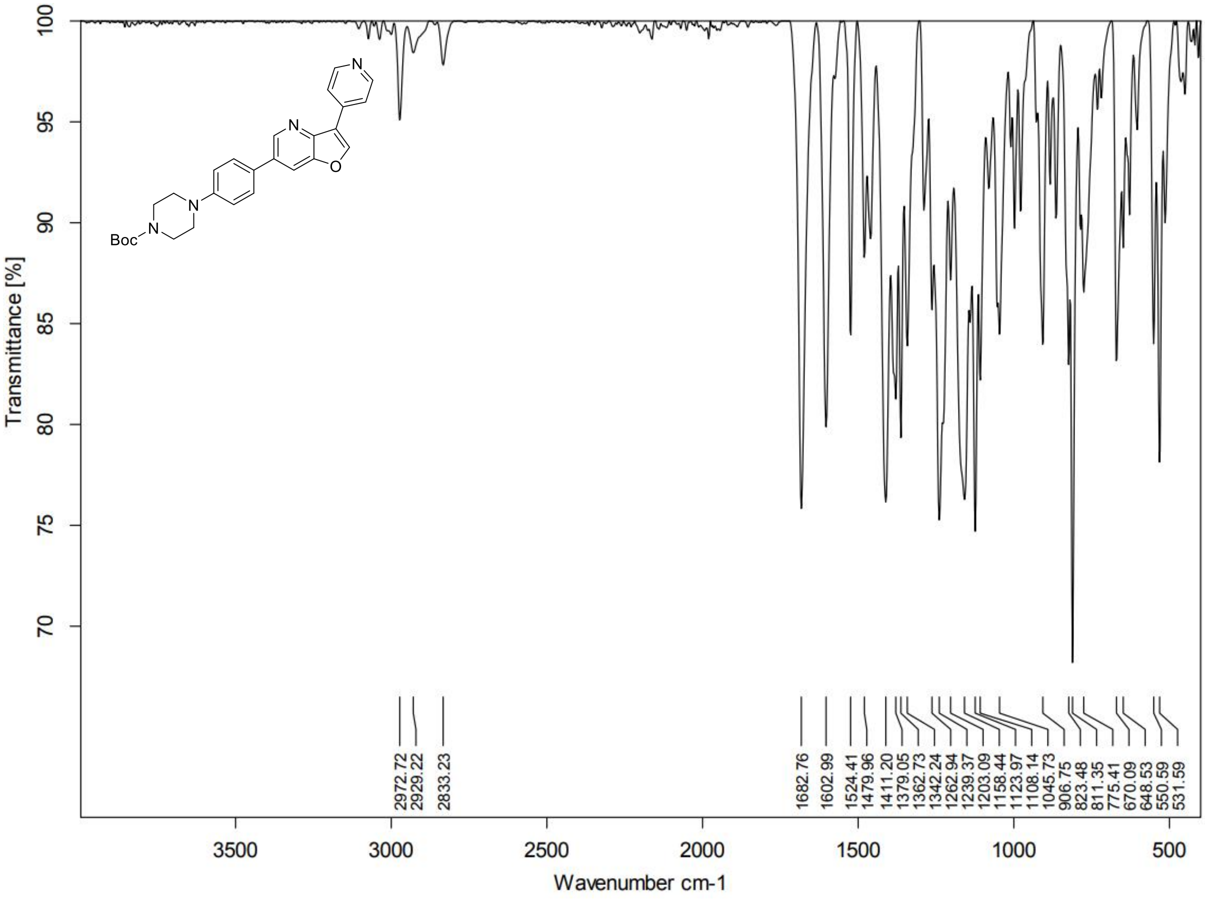

HRMS spectrum of **48**.

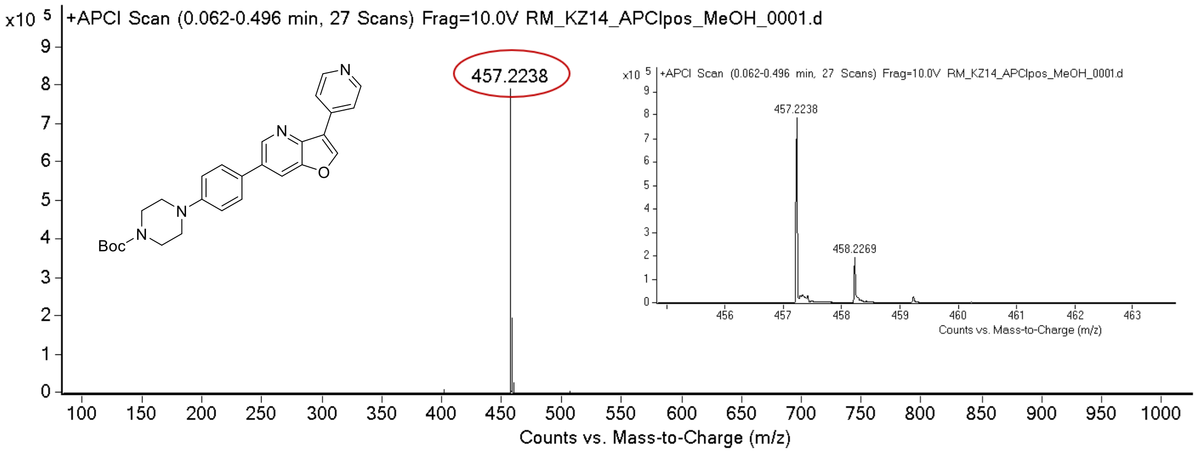

^{1}H (500 MHz) and ^{13}C NMR (126 MHz) spectra of **49** in DMSO-d_6.

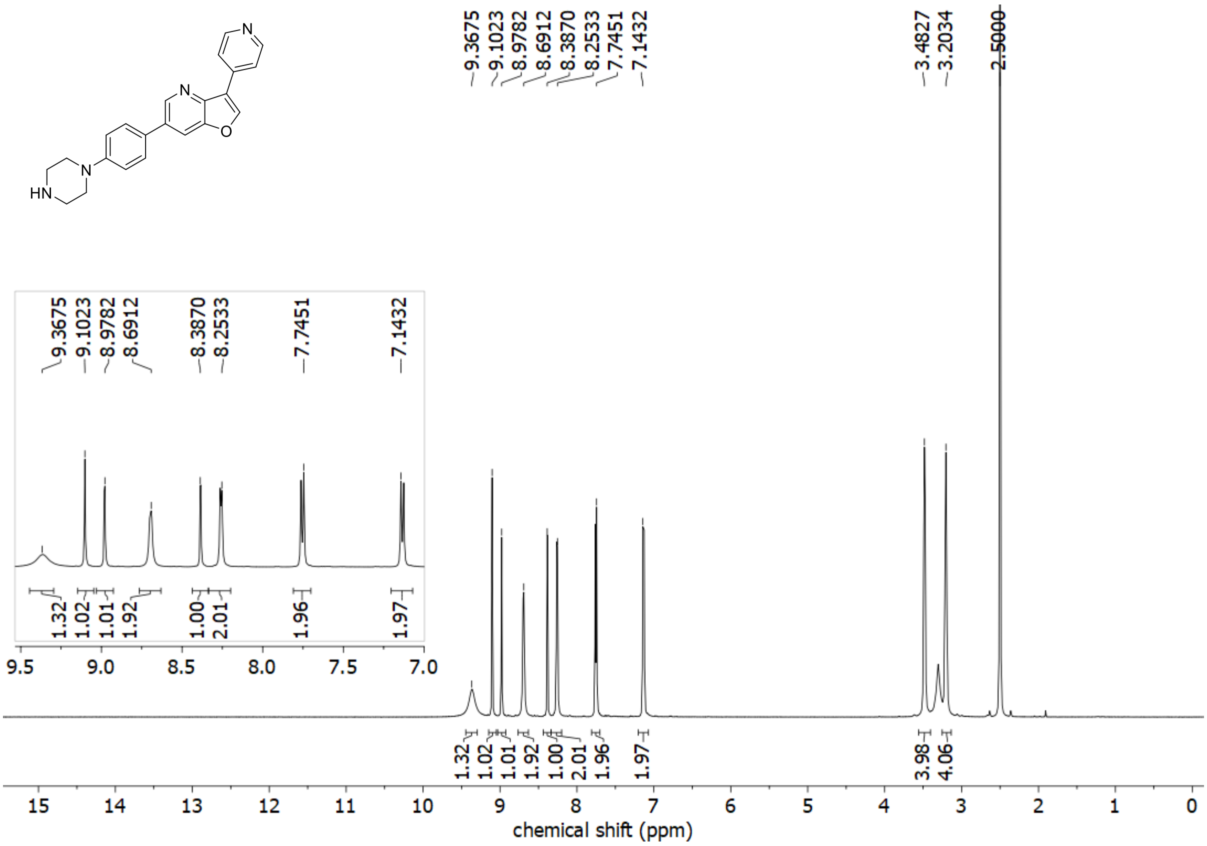

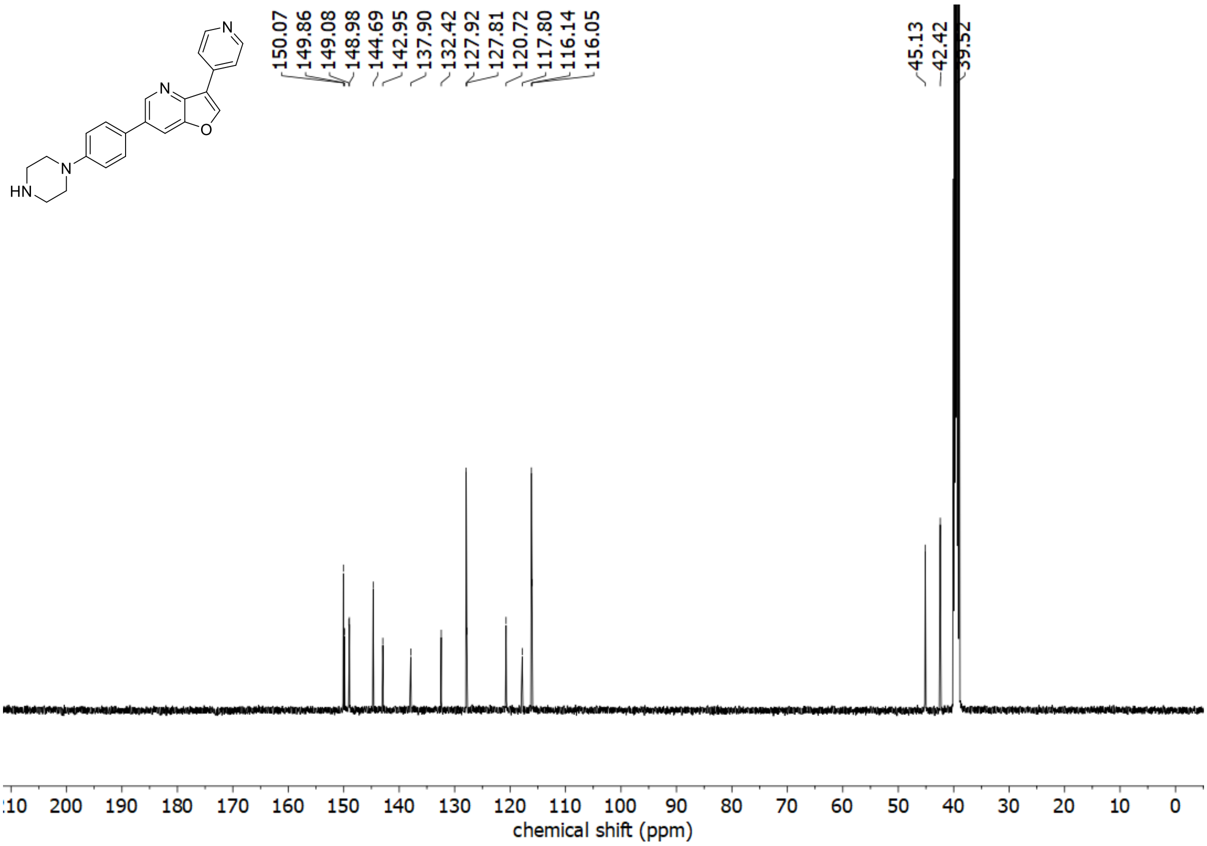

FT-IR spectrum (neat) of **49**.

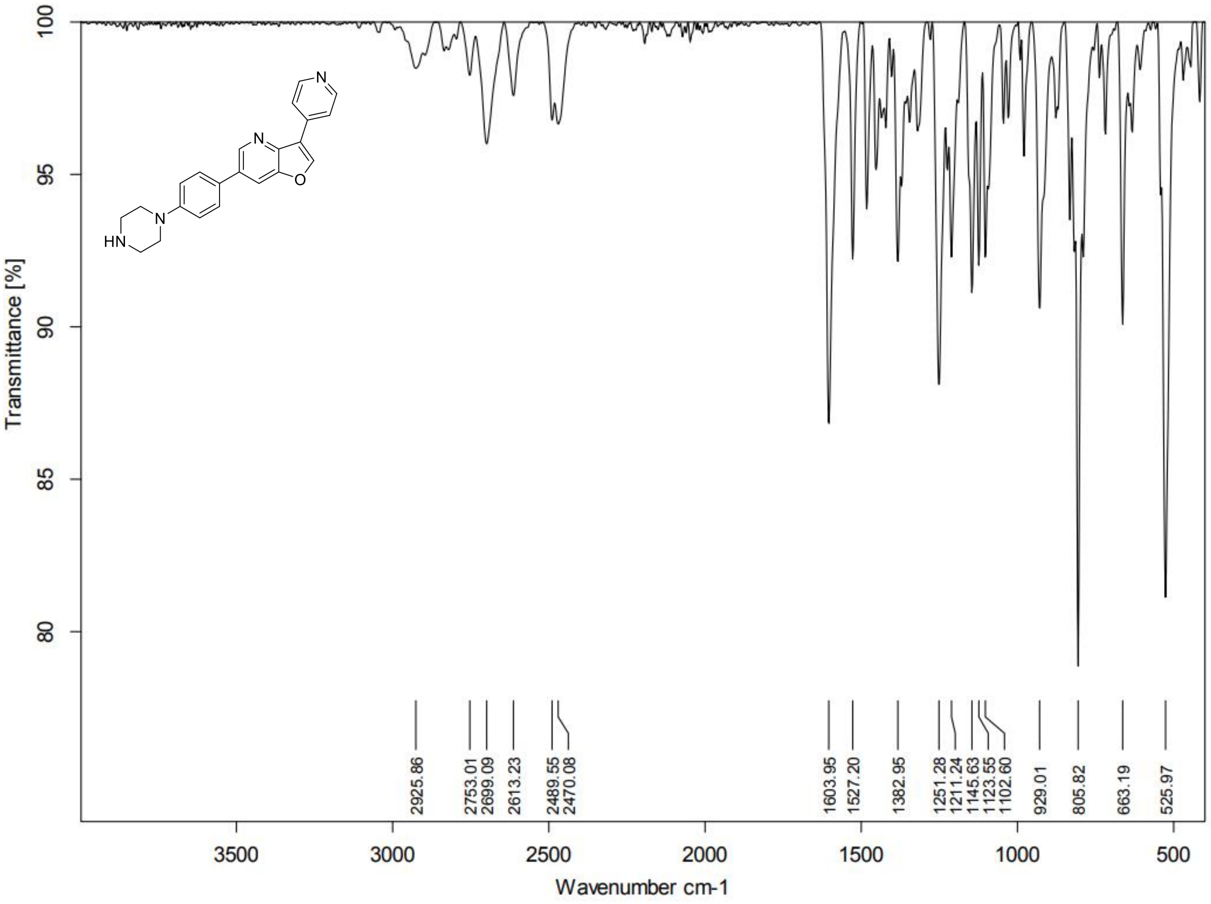

HRMS spectrum of **49**.

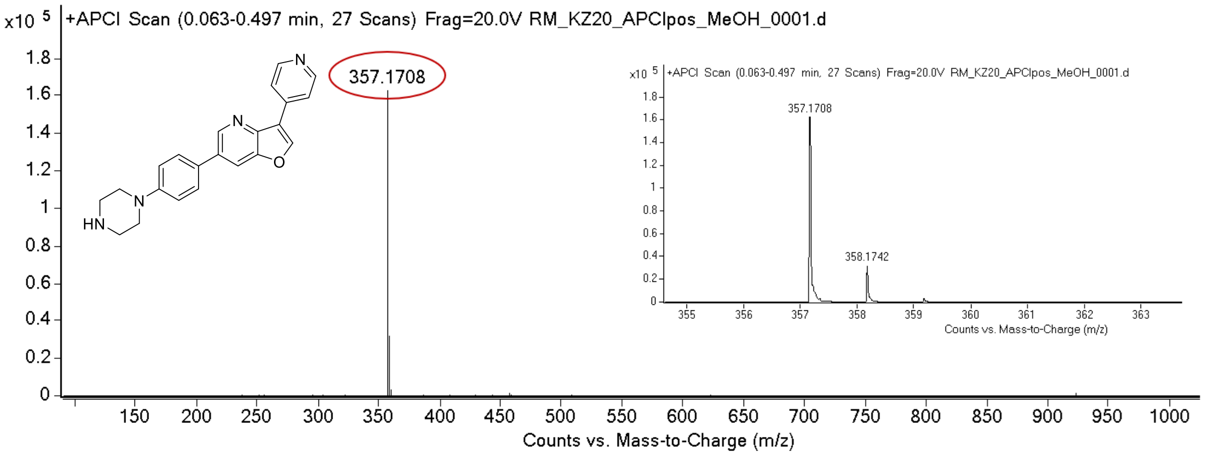

^{1}H (500 MHz) and ^{13}C NMR (126 MHz) spectra of **50** in chloroform-*d*.

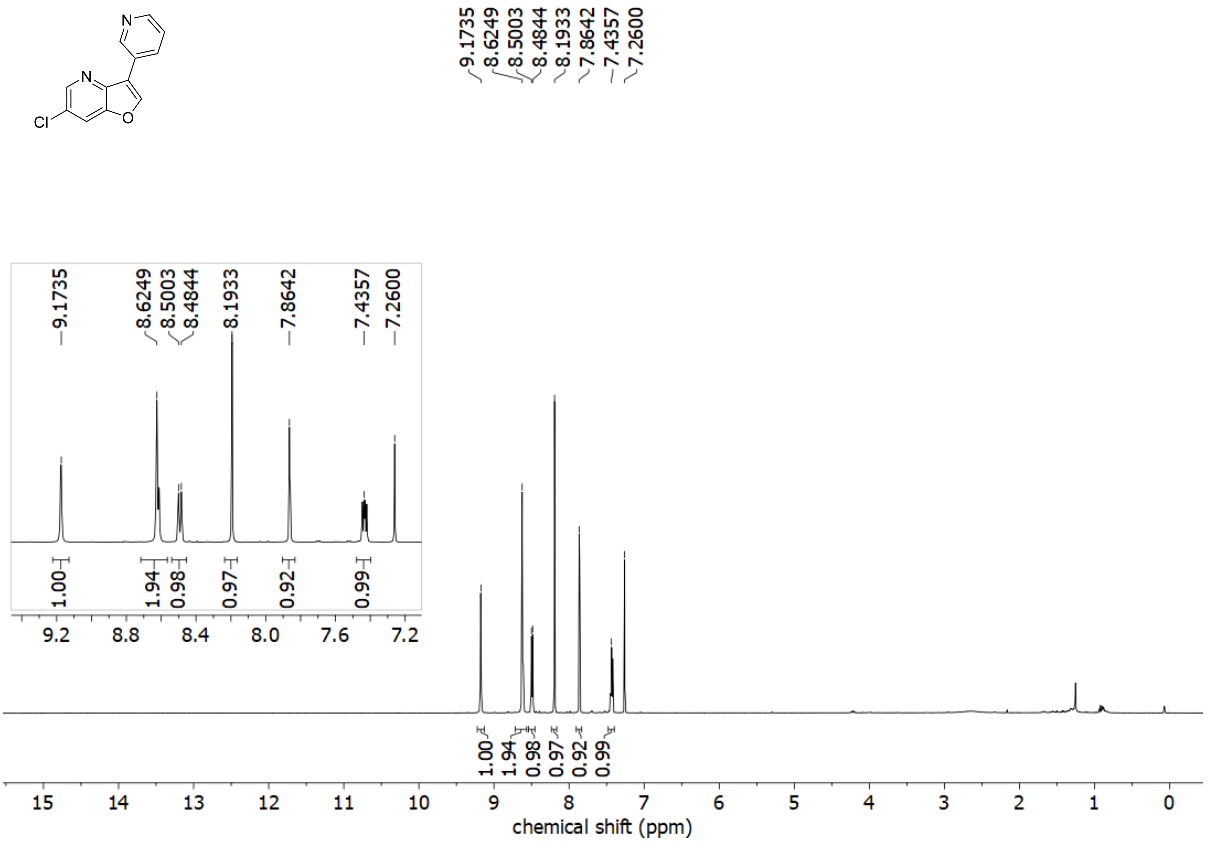

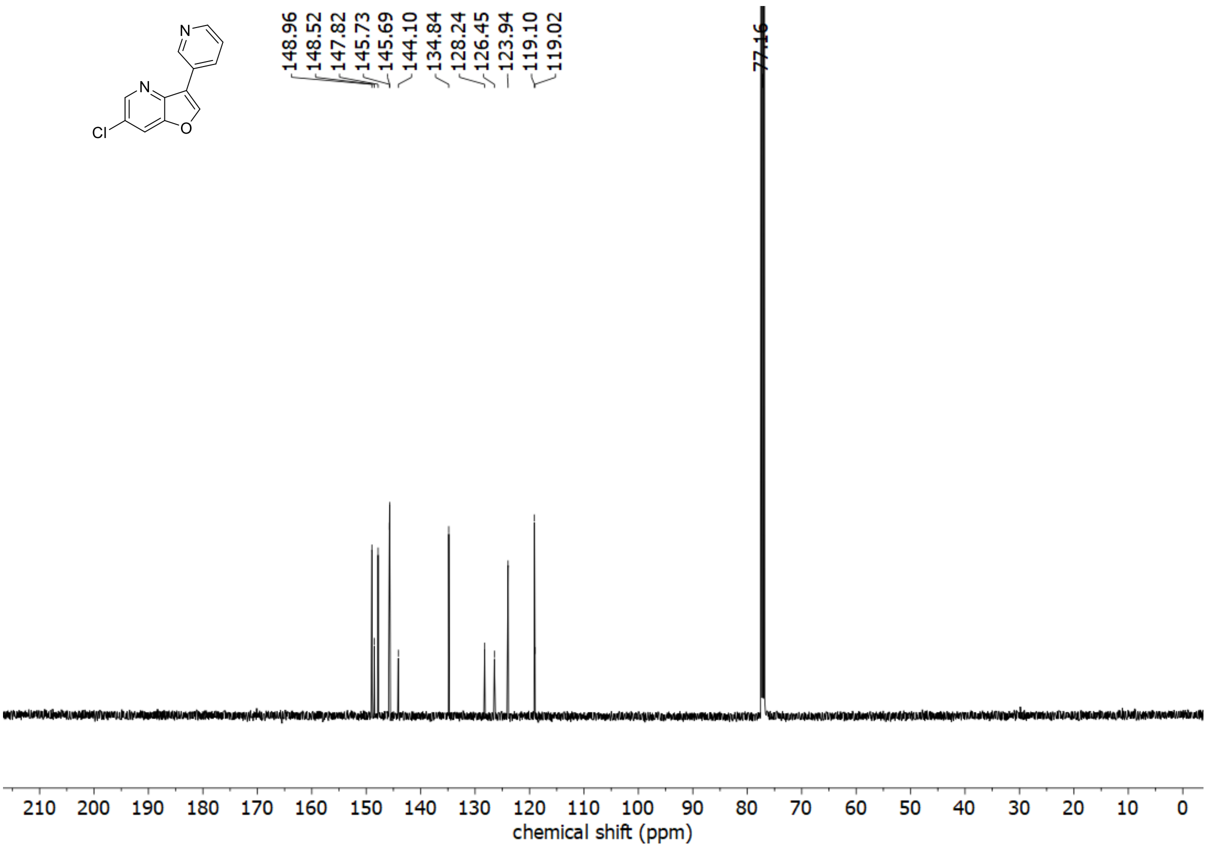

FT-IR spectrum (neat) of **50**.

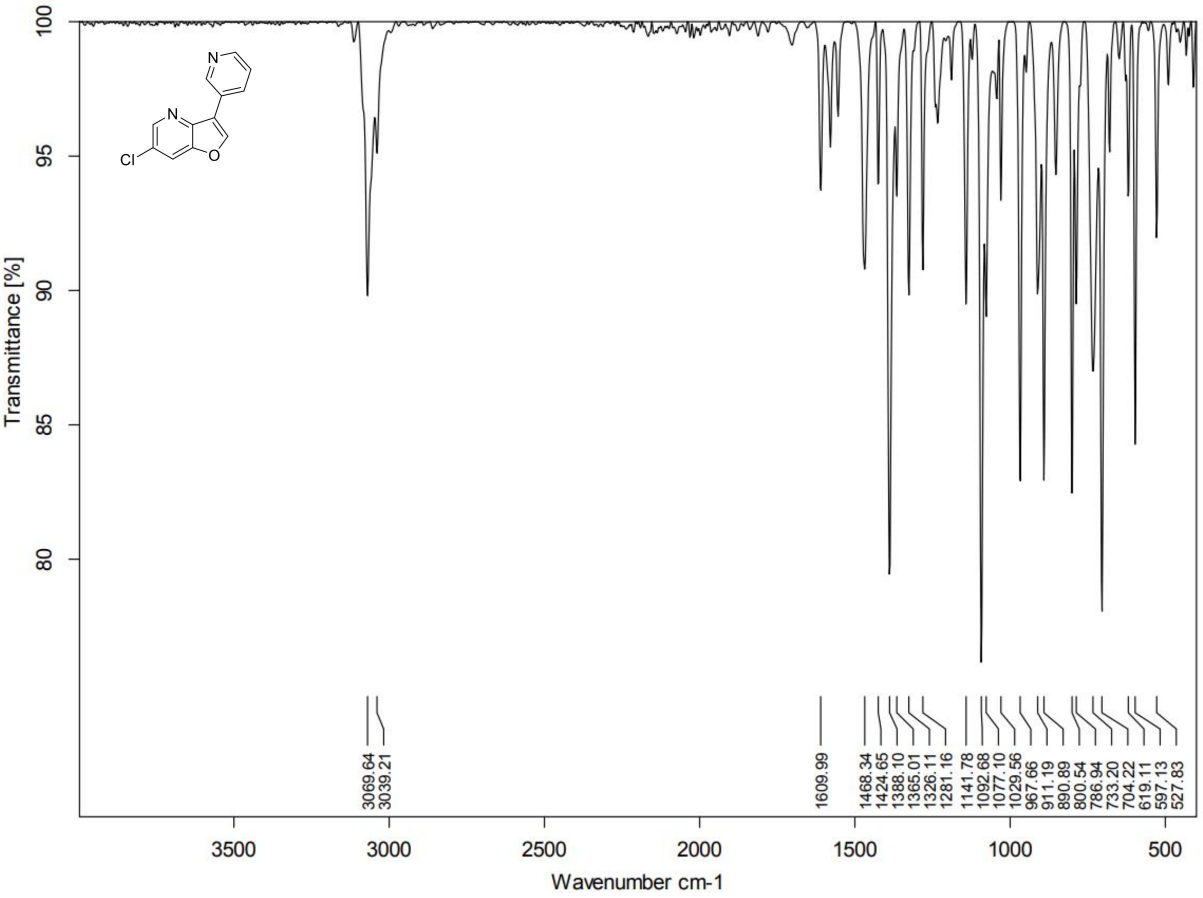

HRMS spectrum of **50**.

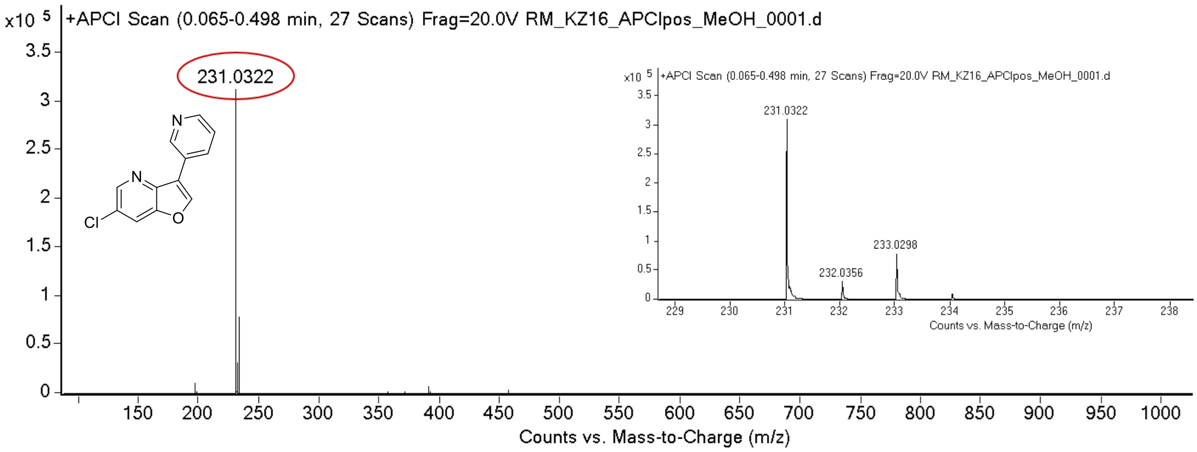

^{1}H (500 MHz) and ^{13}C NMR (126 MHz) spectra of **51** in chloroform-*d*.

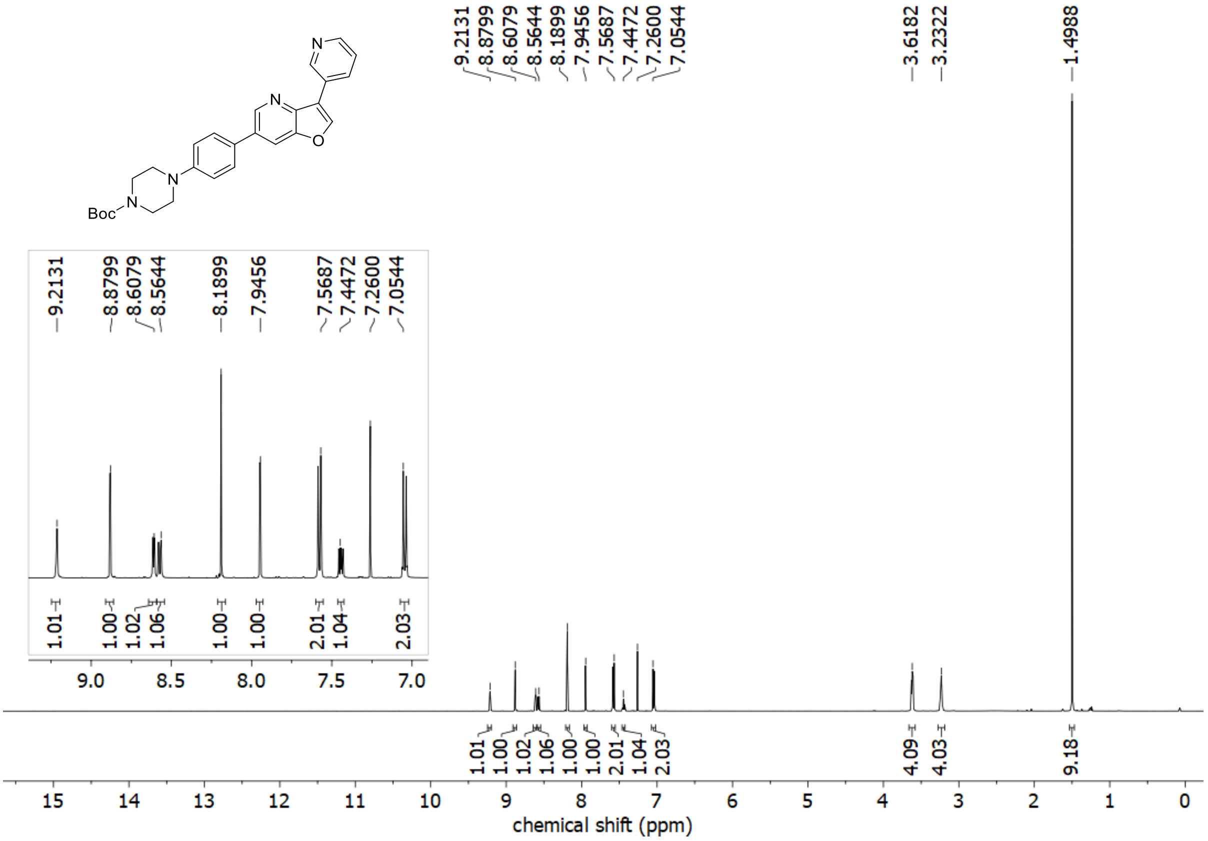

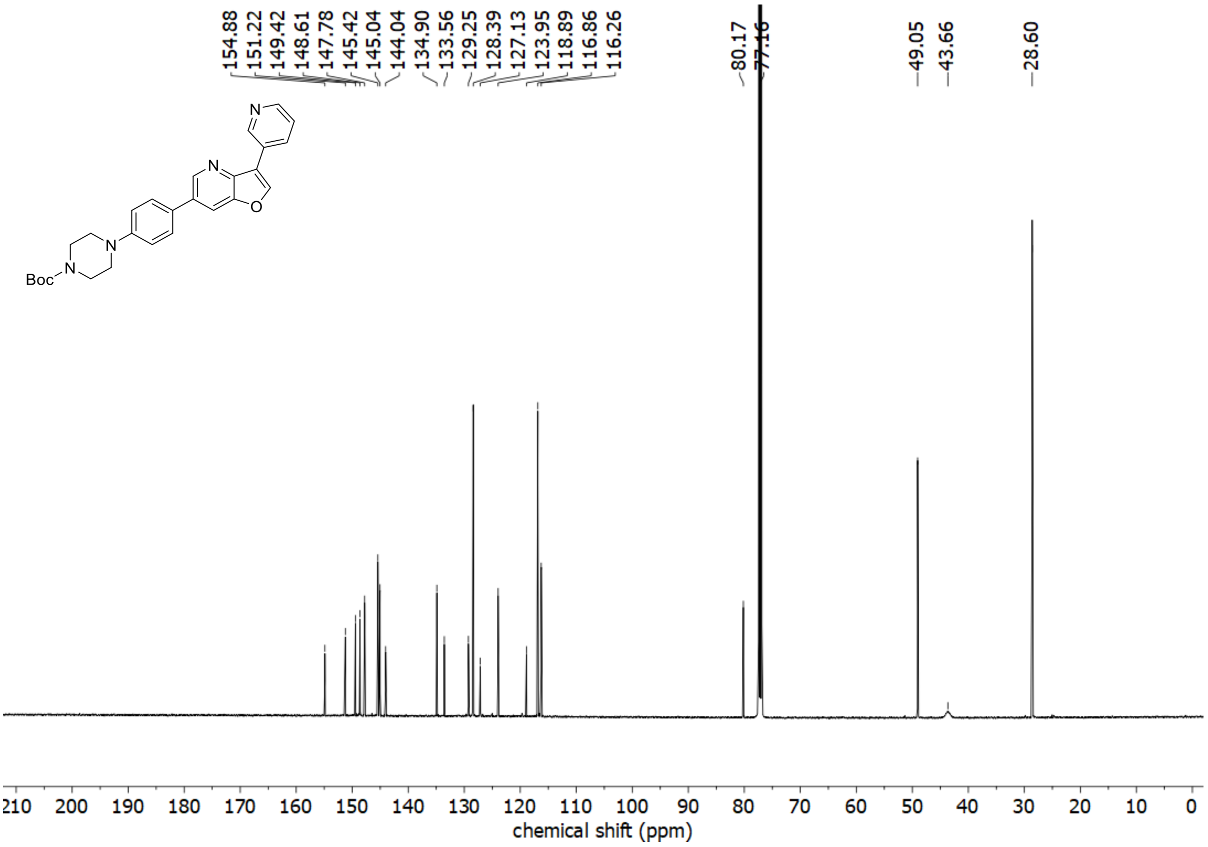

FT-IR spectrum (neat) of **51**.

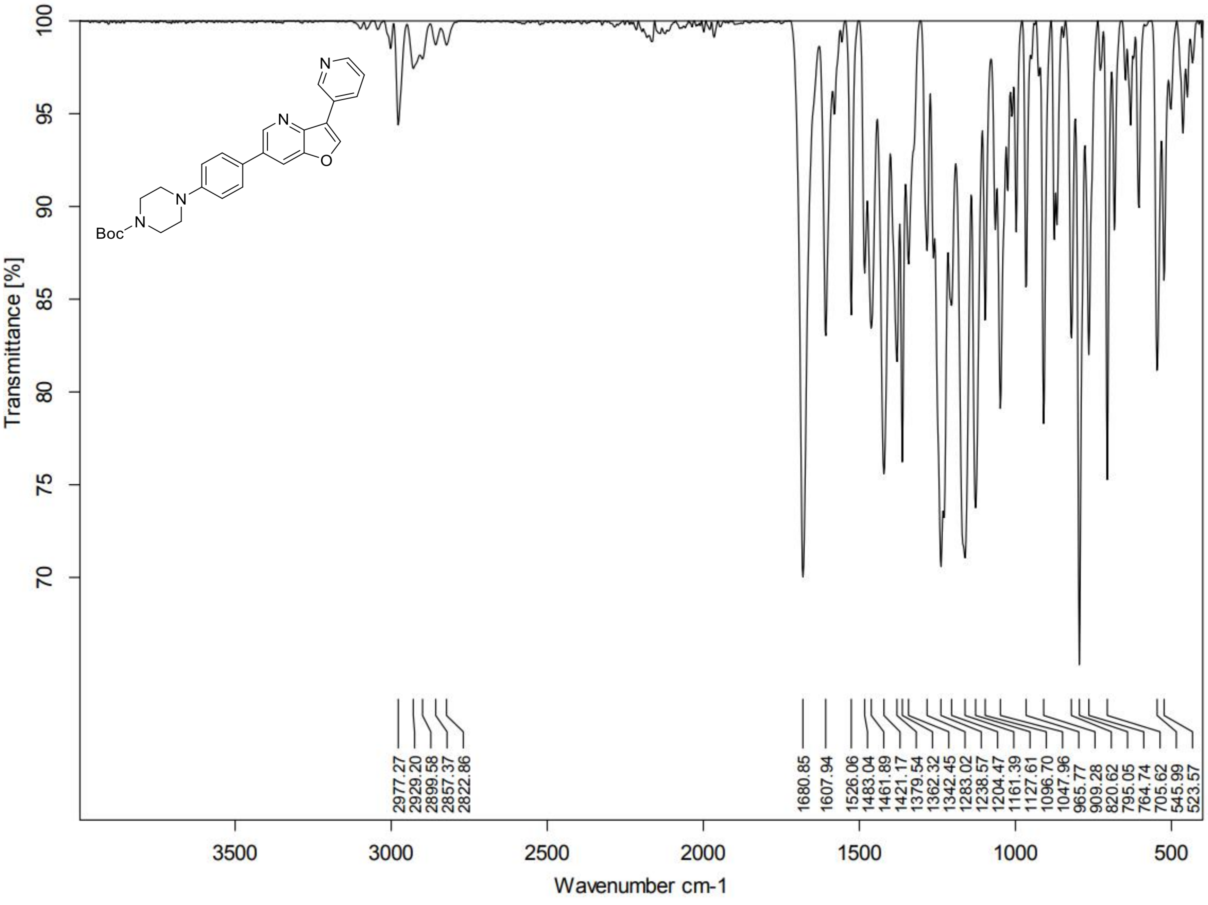

HRMS spectrum of **51**.

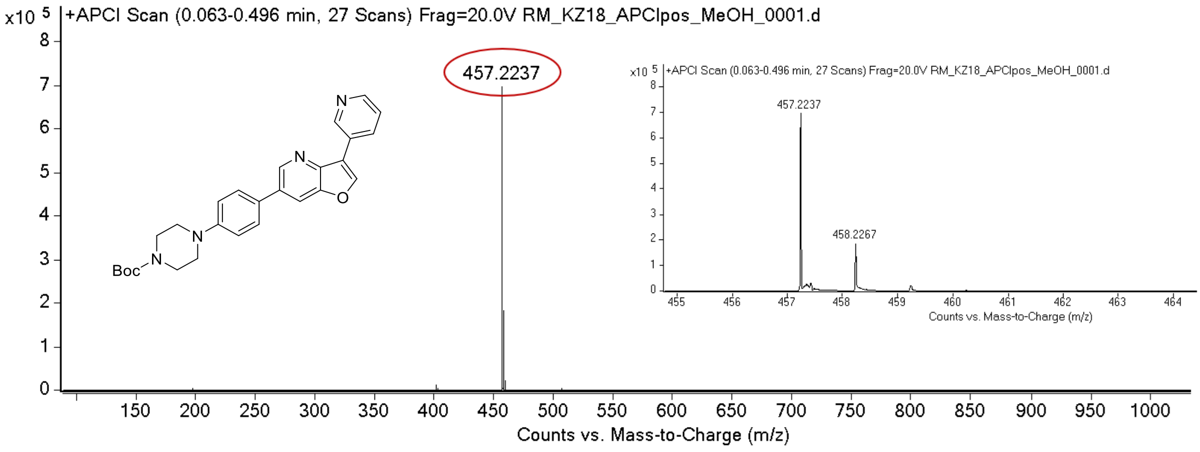

^{1}H (500 MHz) and ^{13}C NMR (126 MHz) spectra of **52** in DMSO-d_6.

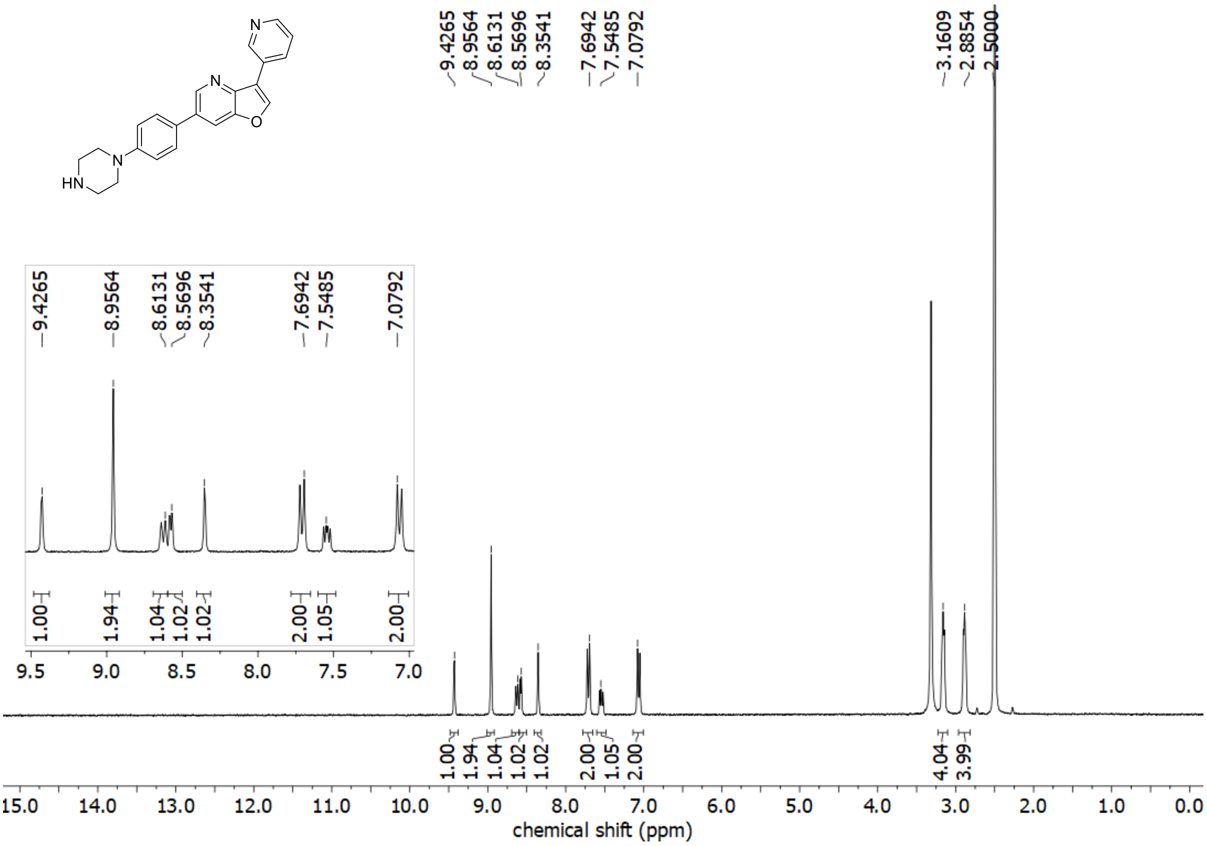

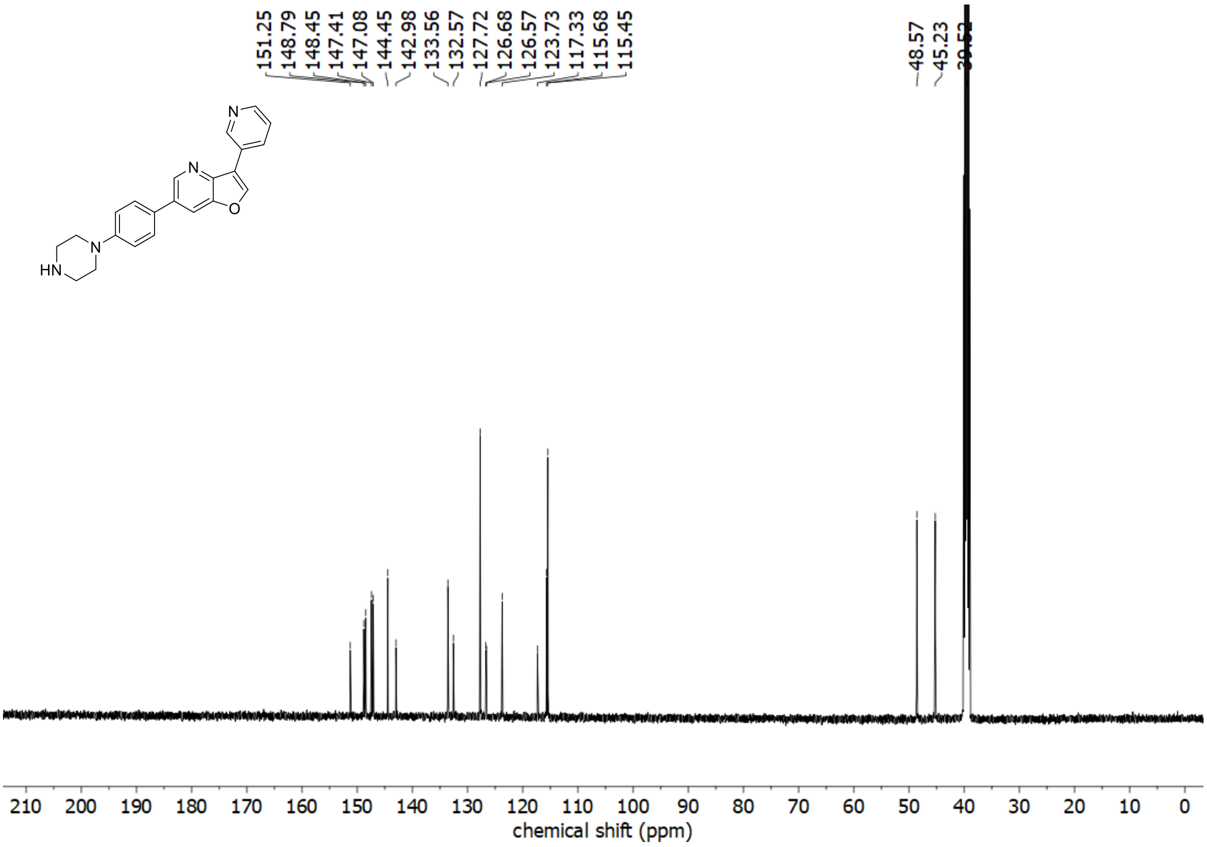

FT-IR spectrum (neat) of **52**.

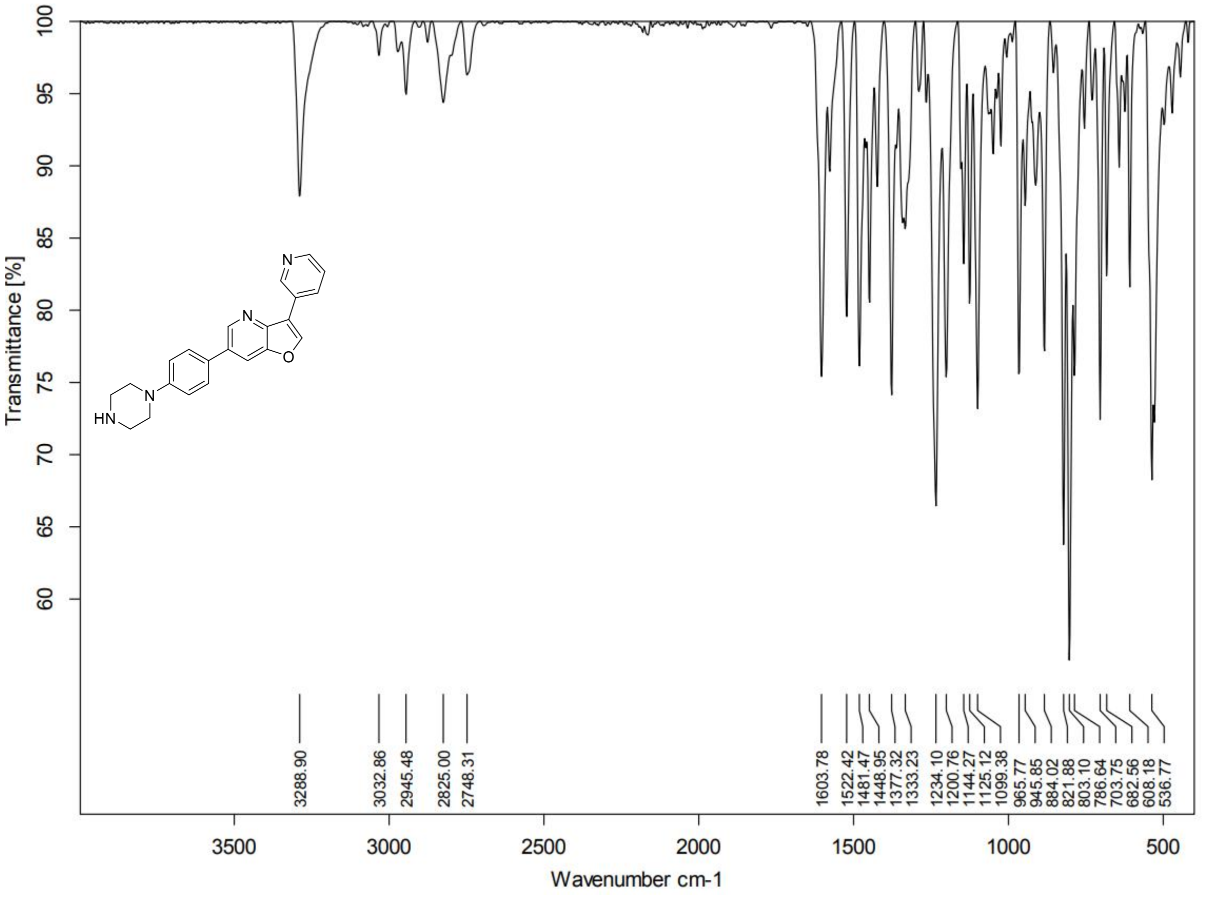

HRMS spectrum of **52**.

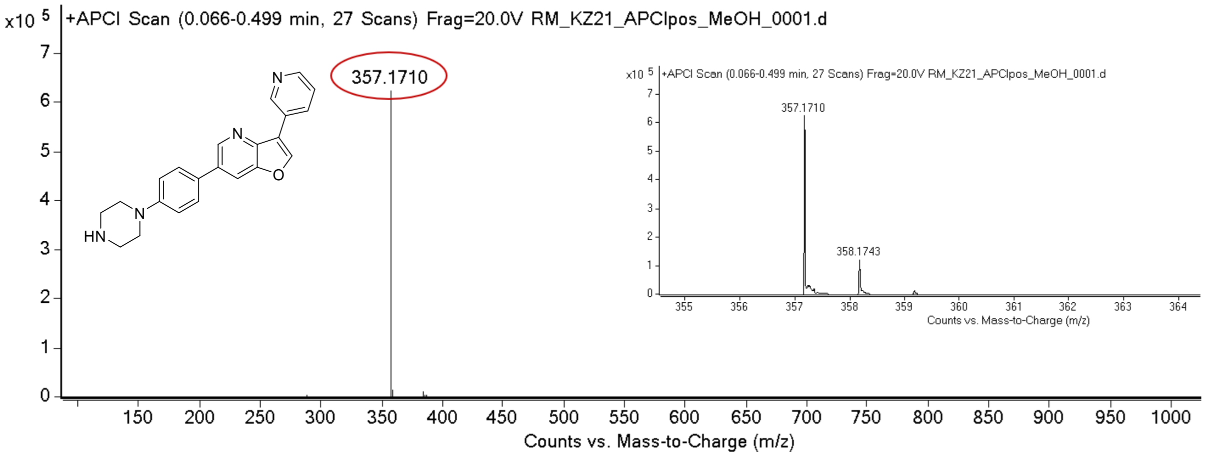

^{1}H (500 MHz) and ^{13}C NMR (126 MHz) spectra of **53** in chloroform-*d*.

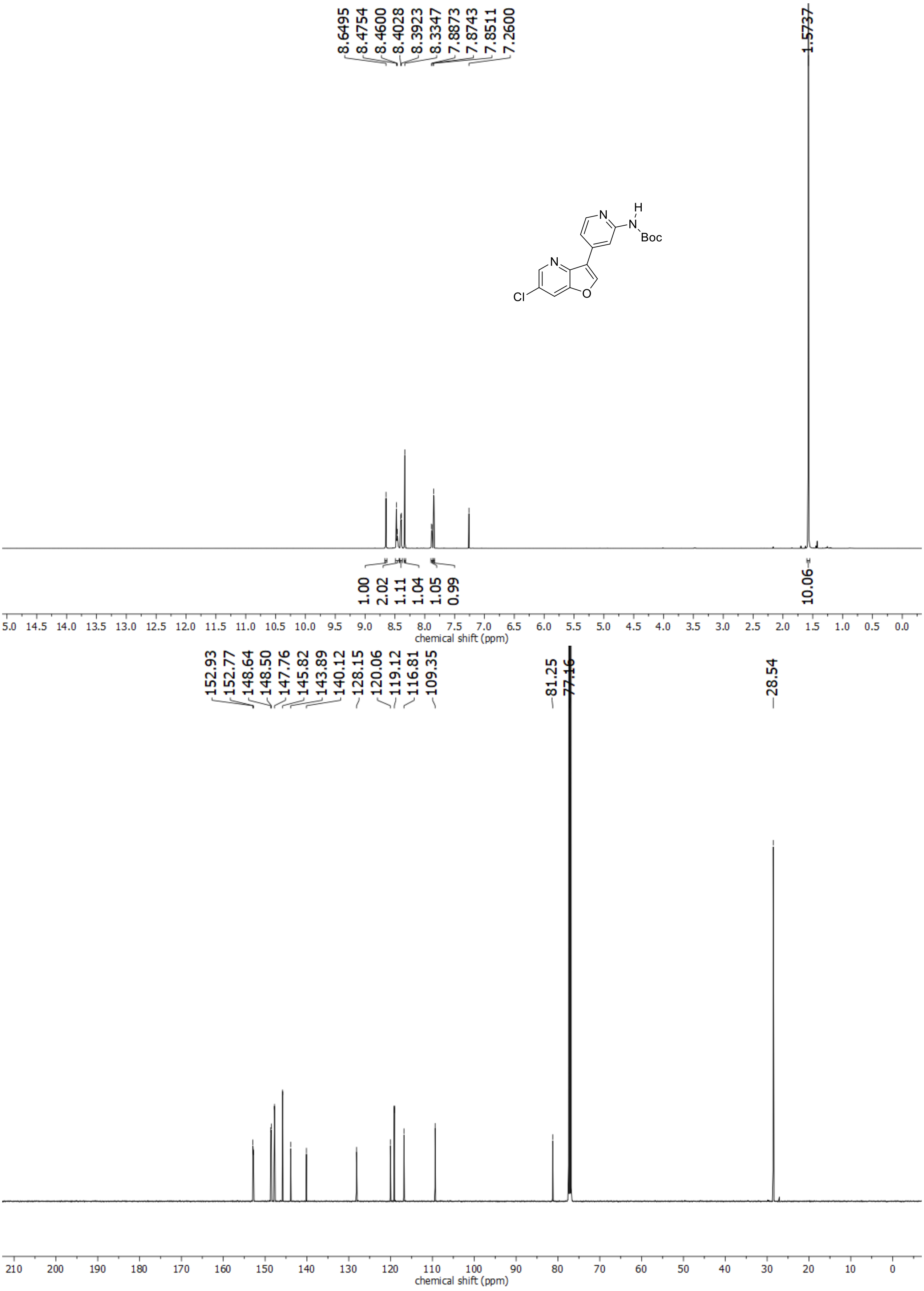

FT-IR spectrum (neat) of **53.**

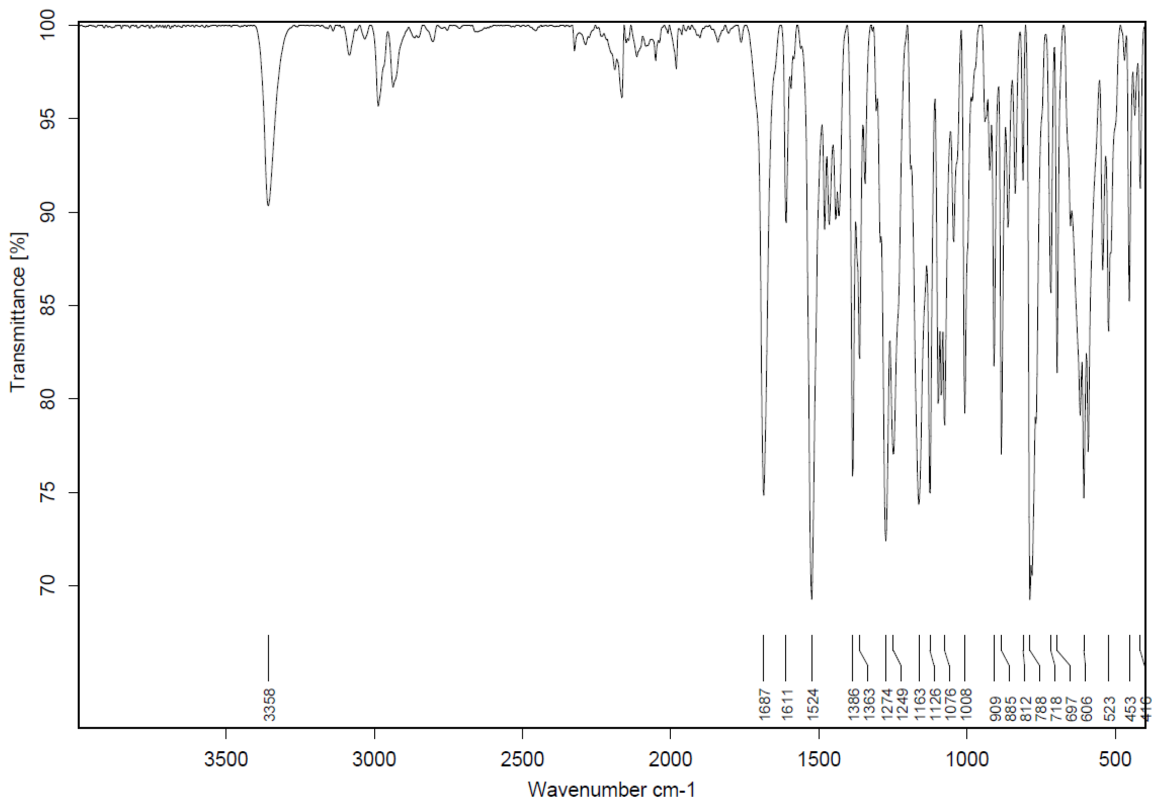

HRMS spectrum of **53.**

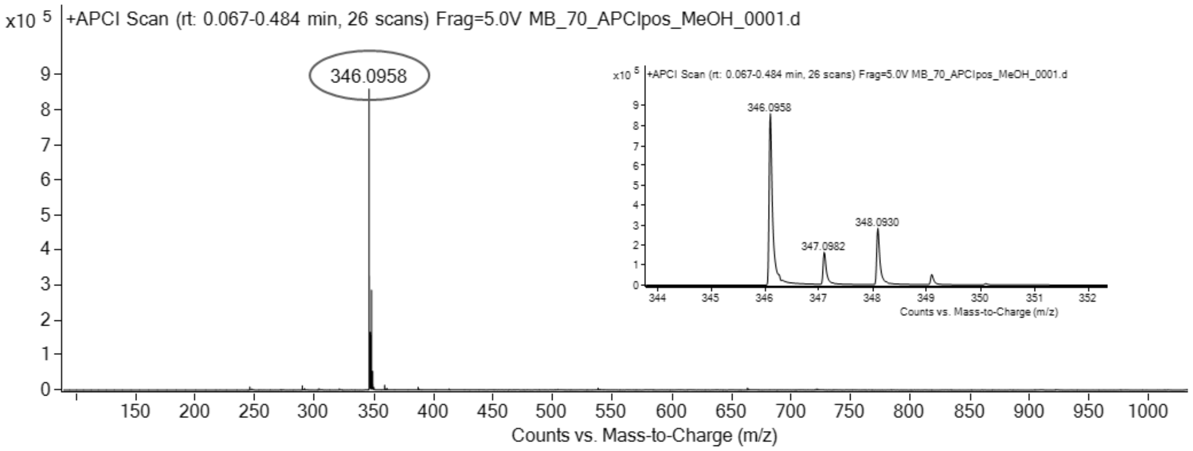

^{1}H (500 MHz) and ^{13}C NMR (126 MHz) spectra of **54** in chloroform-*d*.

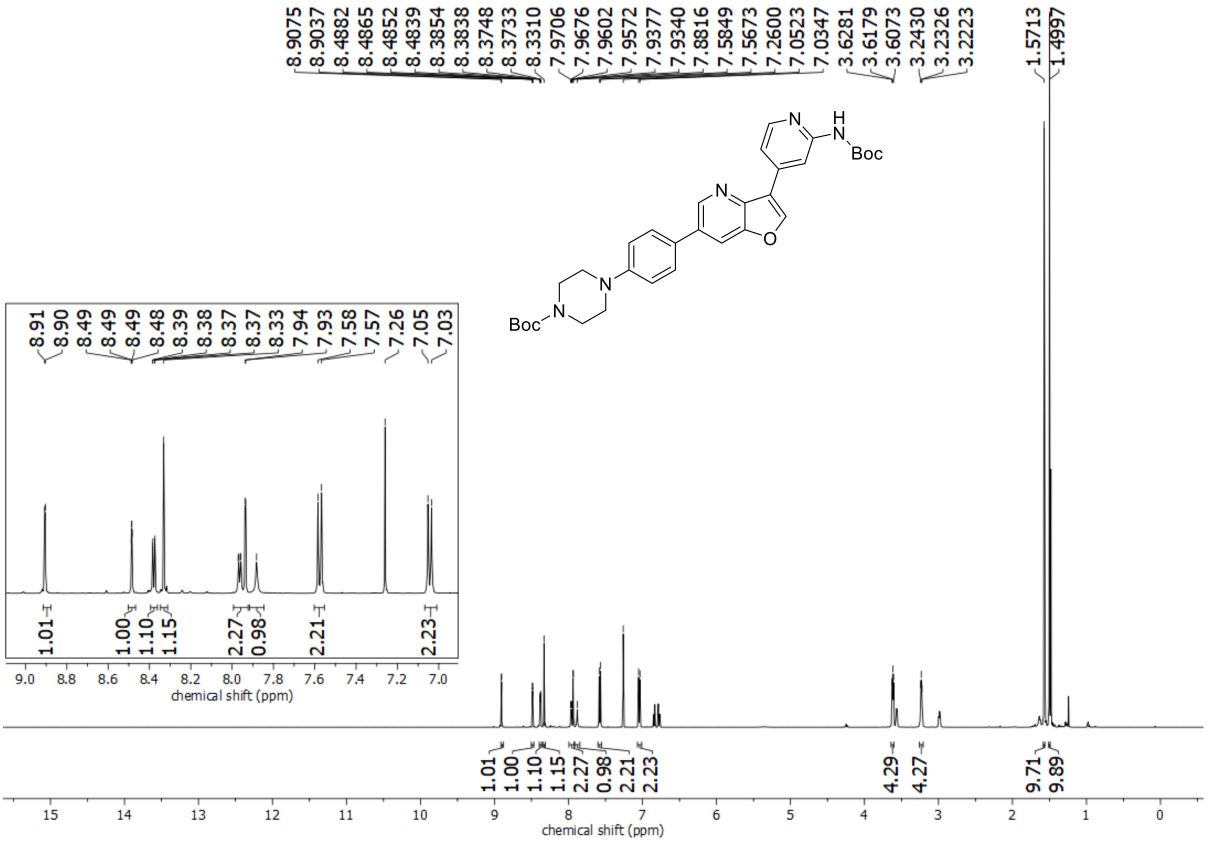

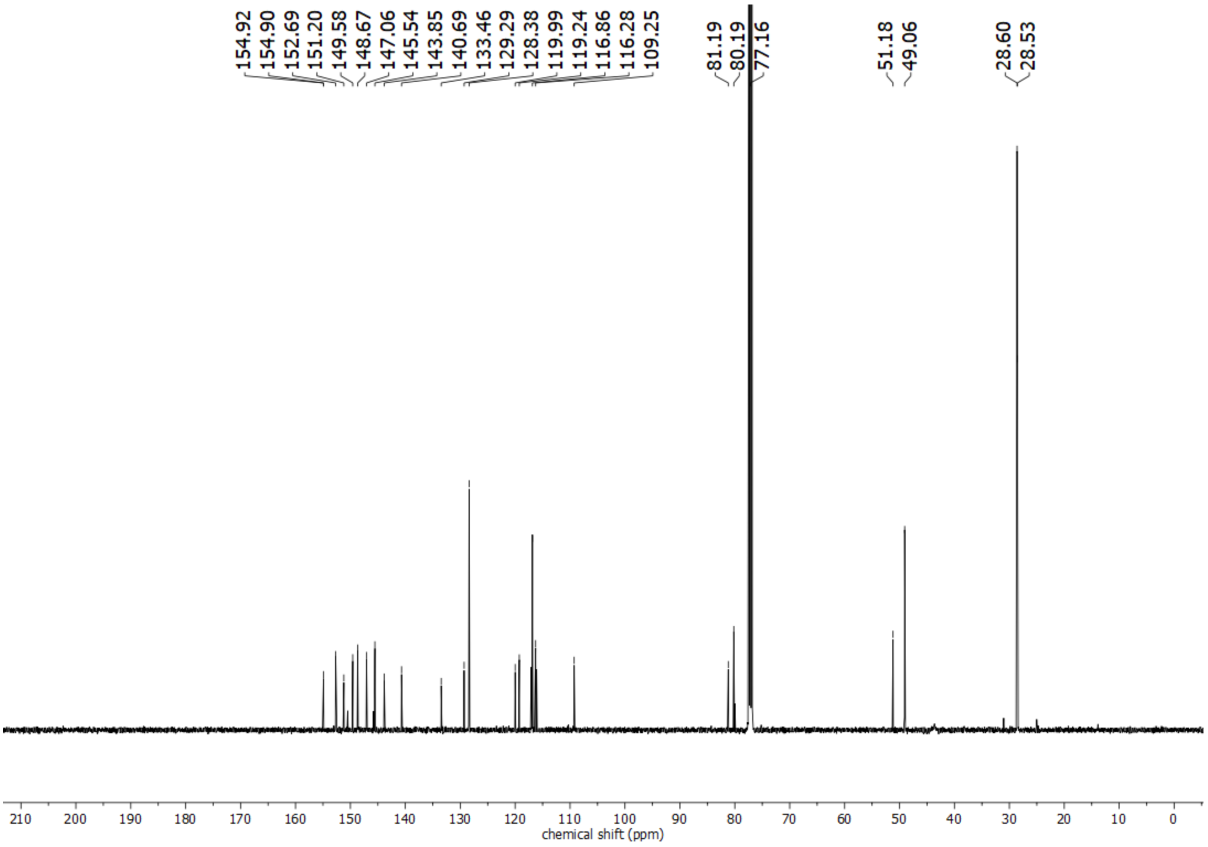

FT-IR spectrum (neat) of **54.**

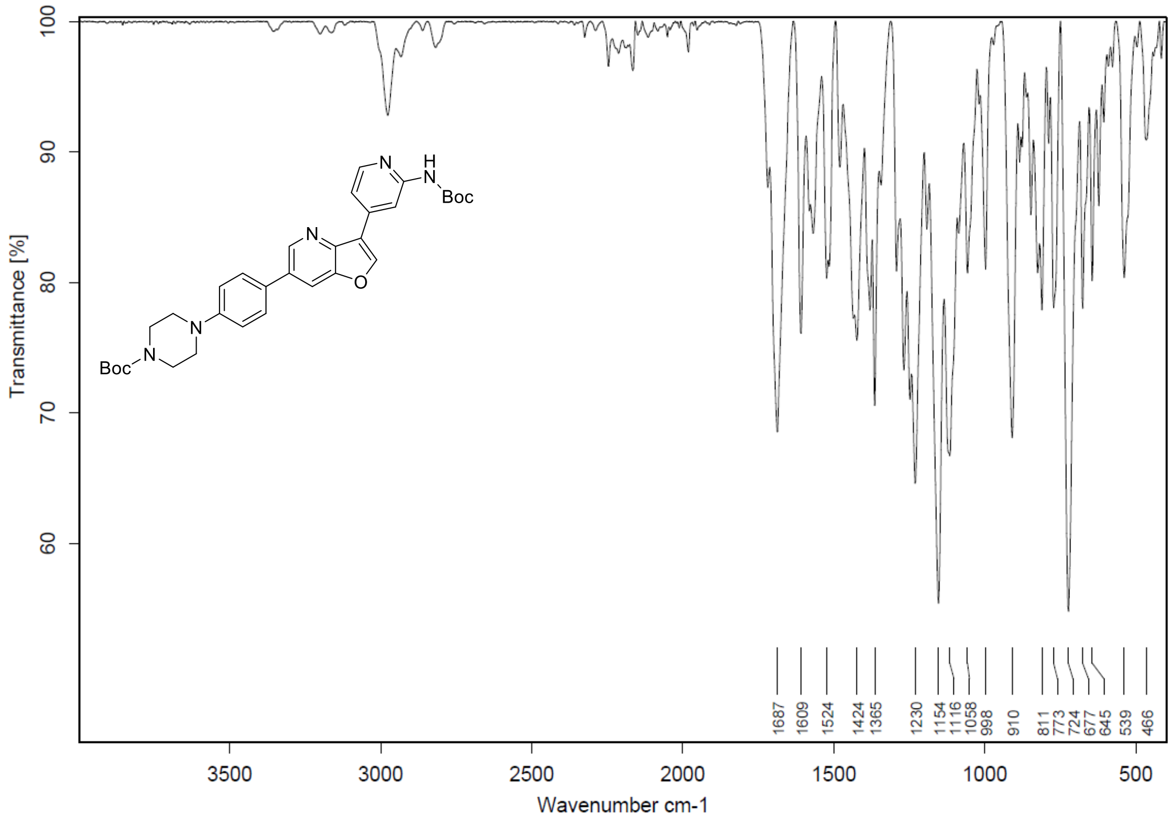

HRMS spectrum of **54.**

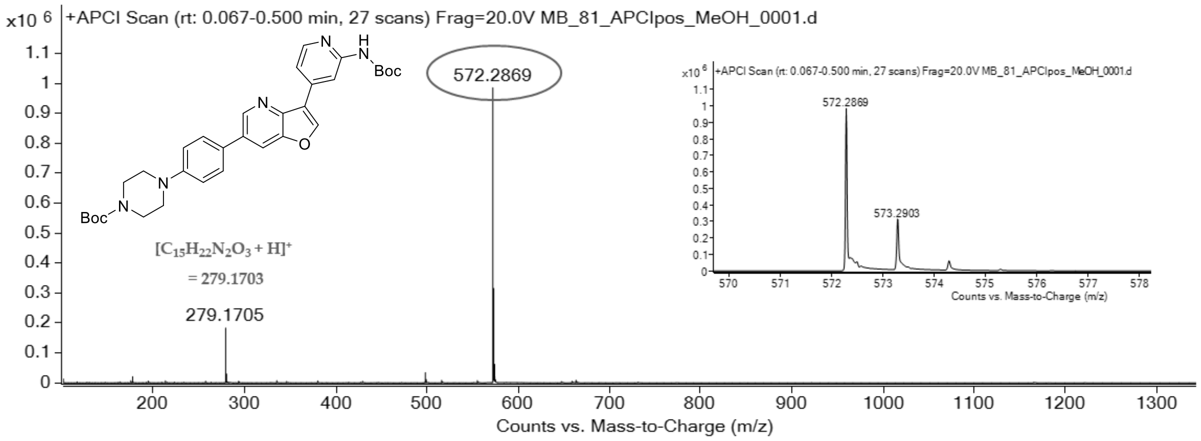

^{1}H (500 MHz) and ^{13}C NMR (126 MHz) spectra of **55** in chloroform-*d*.

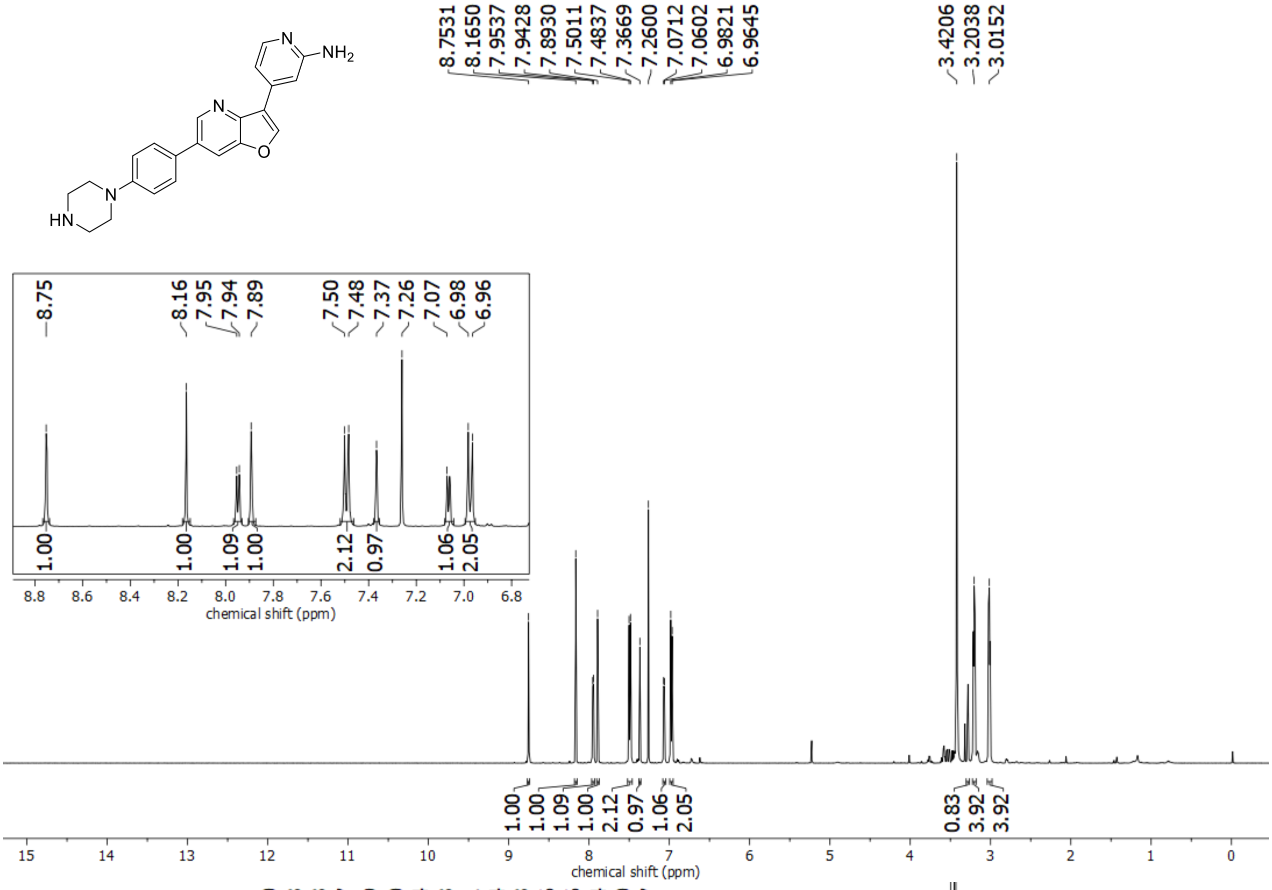

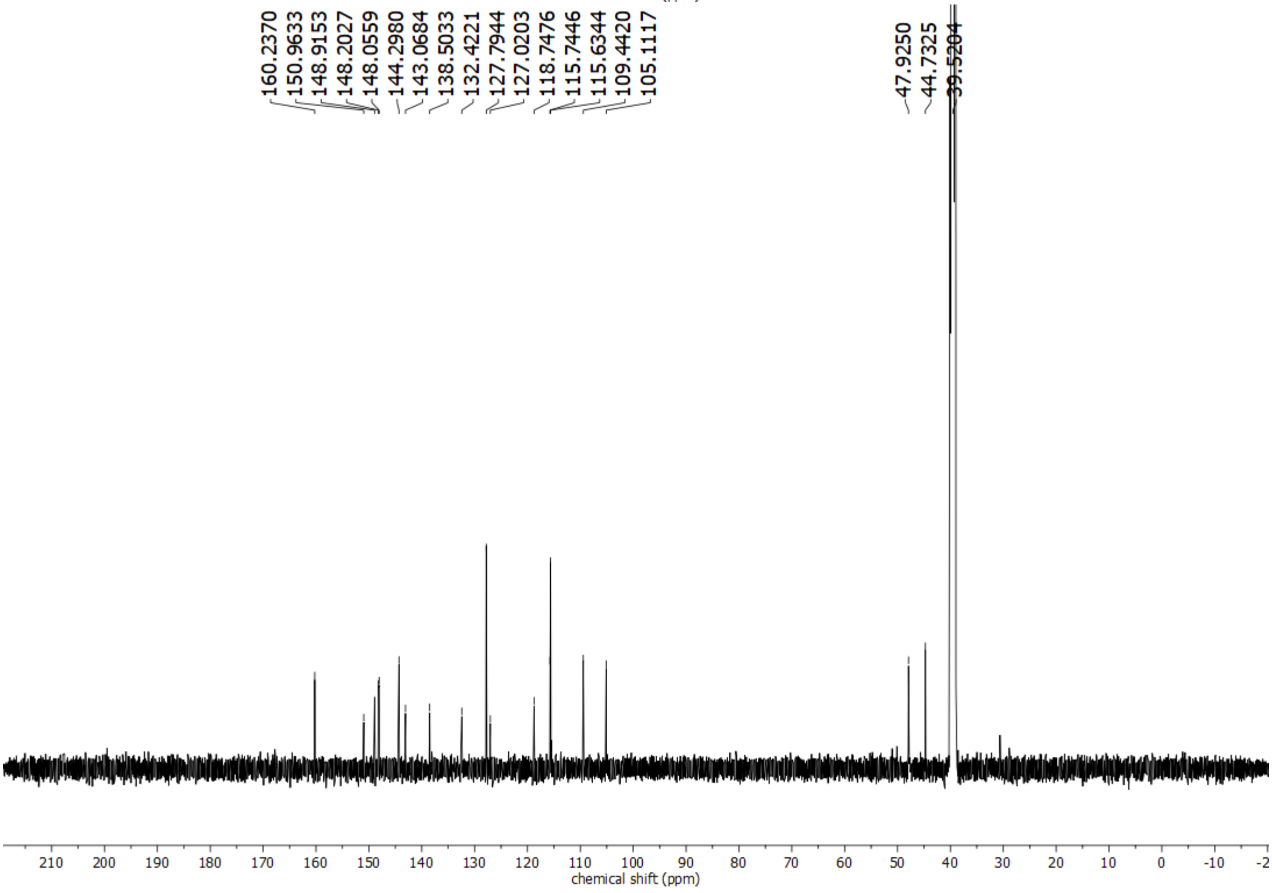

FT-IR spectrum (neat) of **55.**

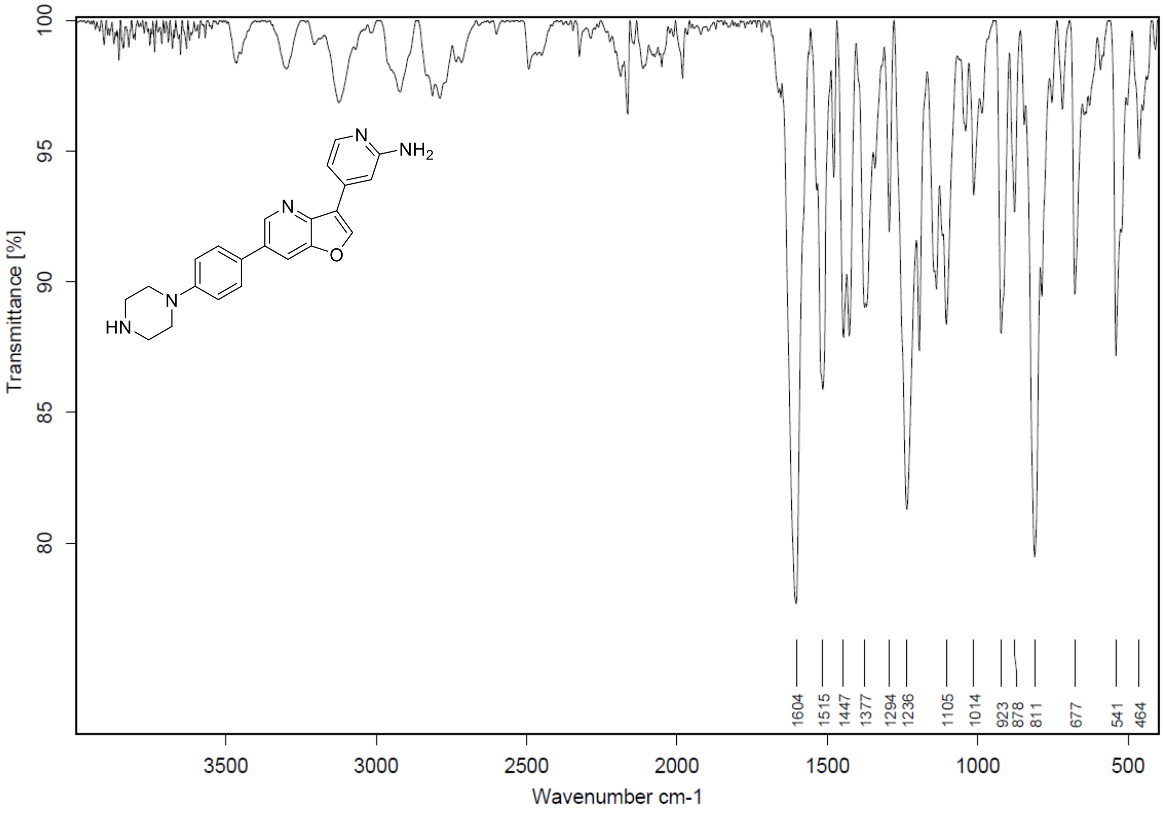

HRMS spectrum of **55.**

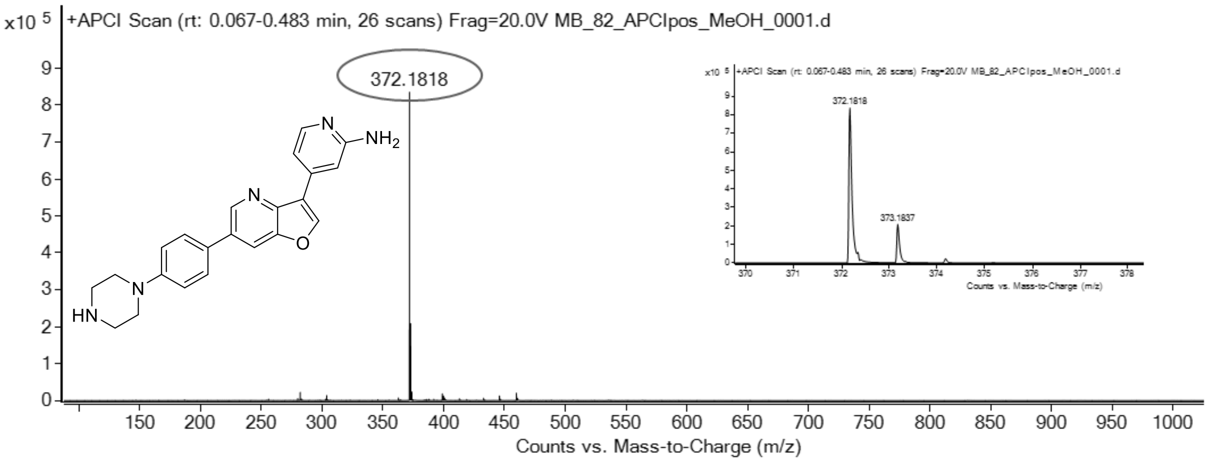

^{1}H (500 MHz) and ^{13}C NMR (126 MHz) spectra of **56** in chloroform-*d*.

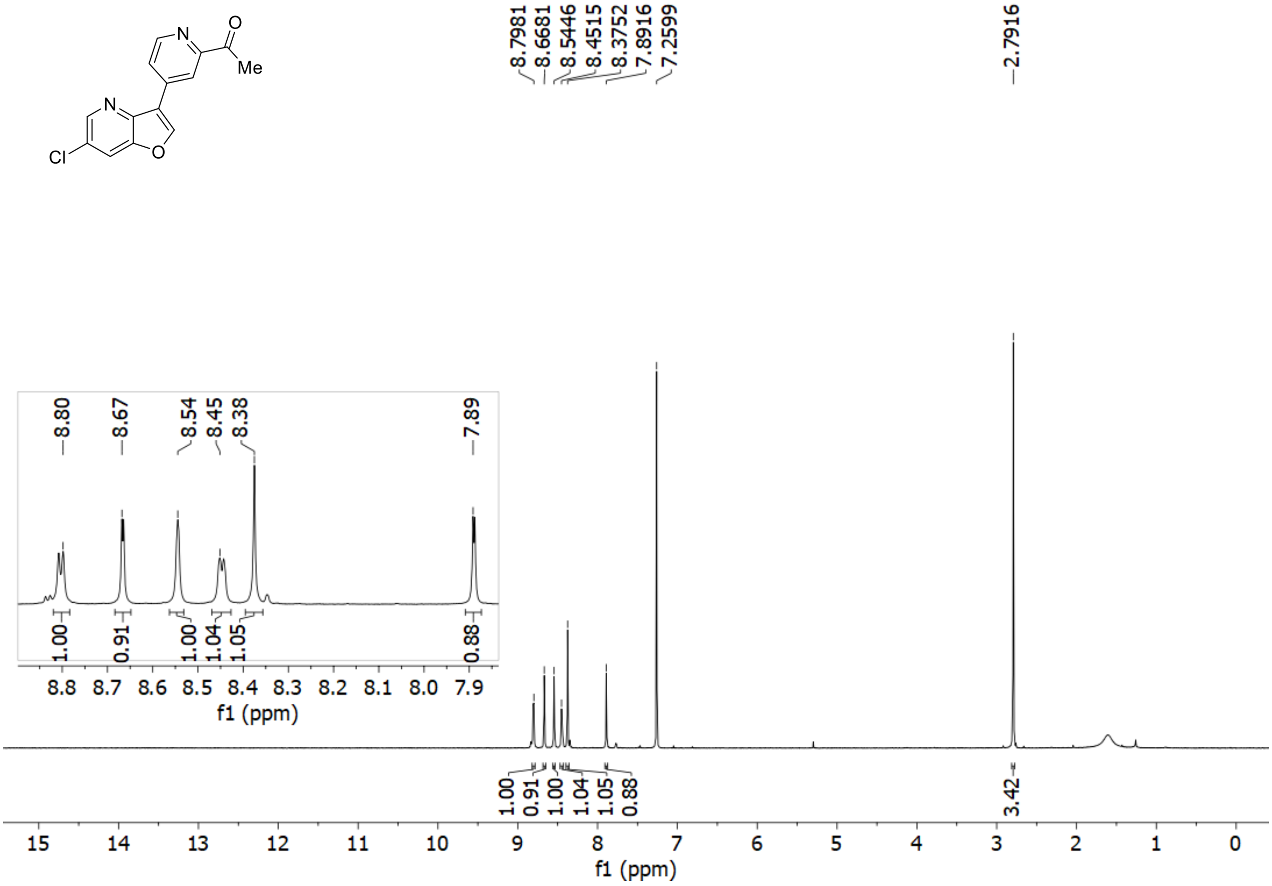

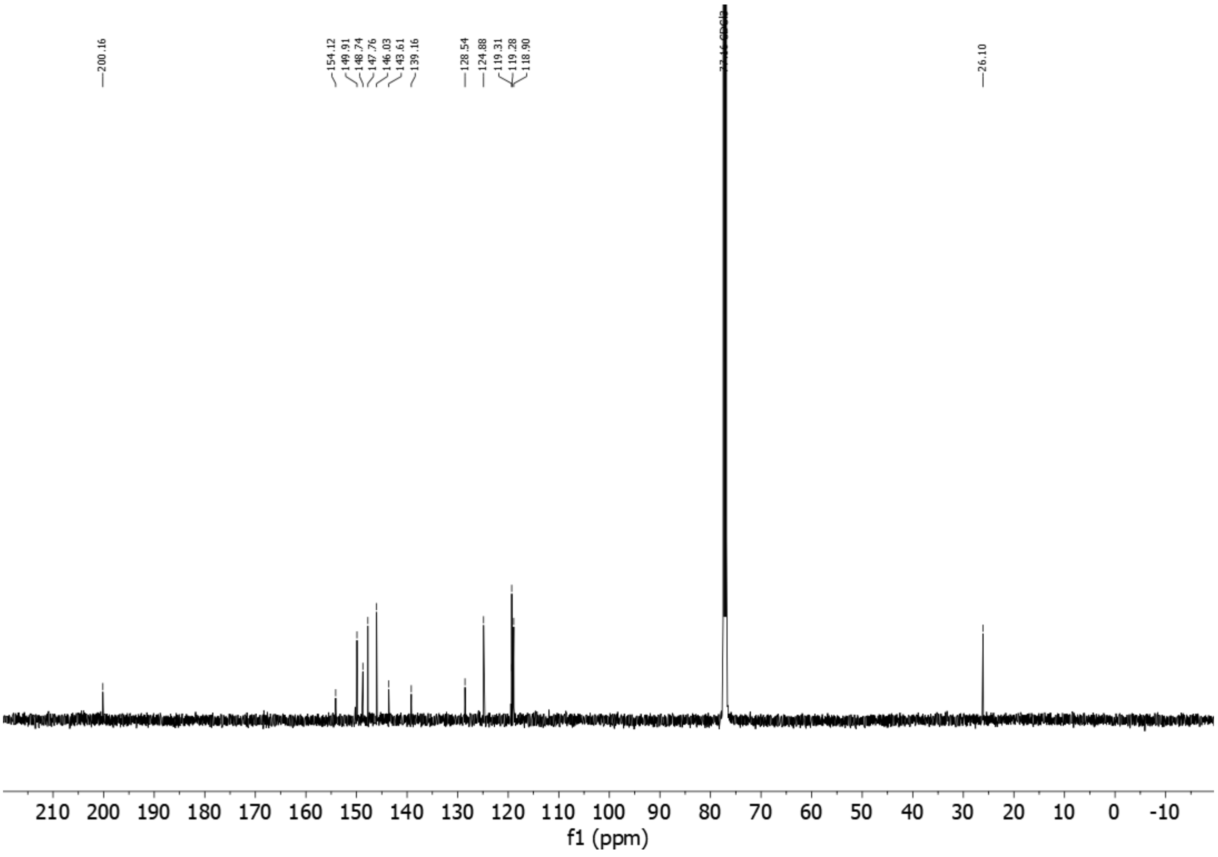

FT-IR spectrum (neat) of **56**.

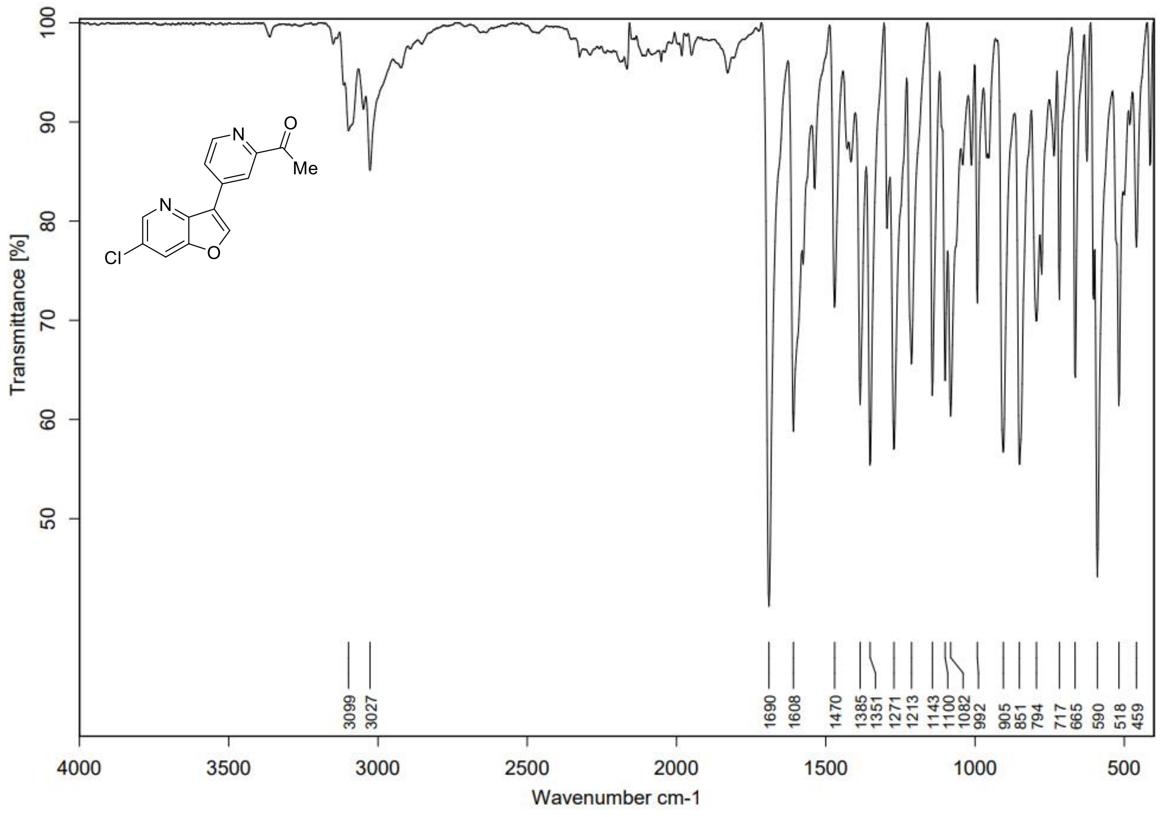

HRMS spectrum of **56.**

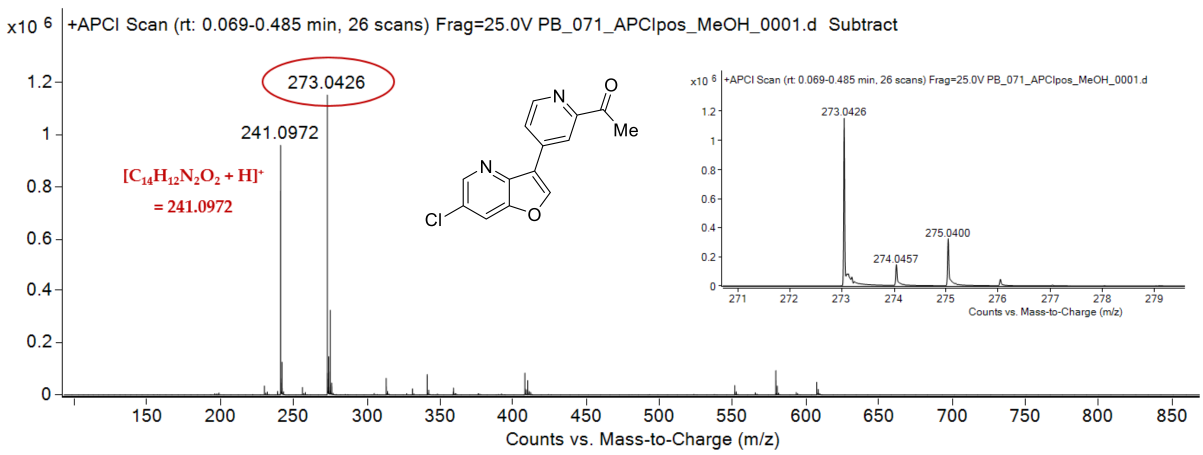

^{1}H (500 MHz) and ^{13}C NMR (126 MHz) spectra of **57** in DMSO-d_6.

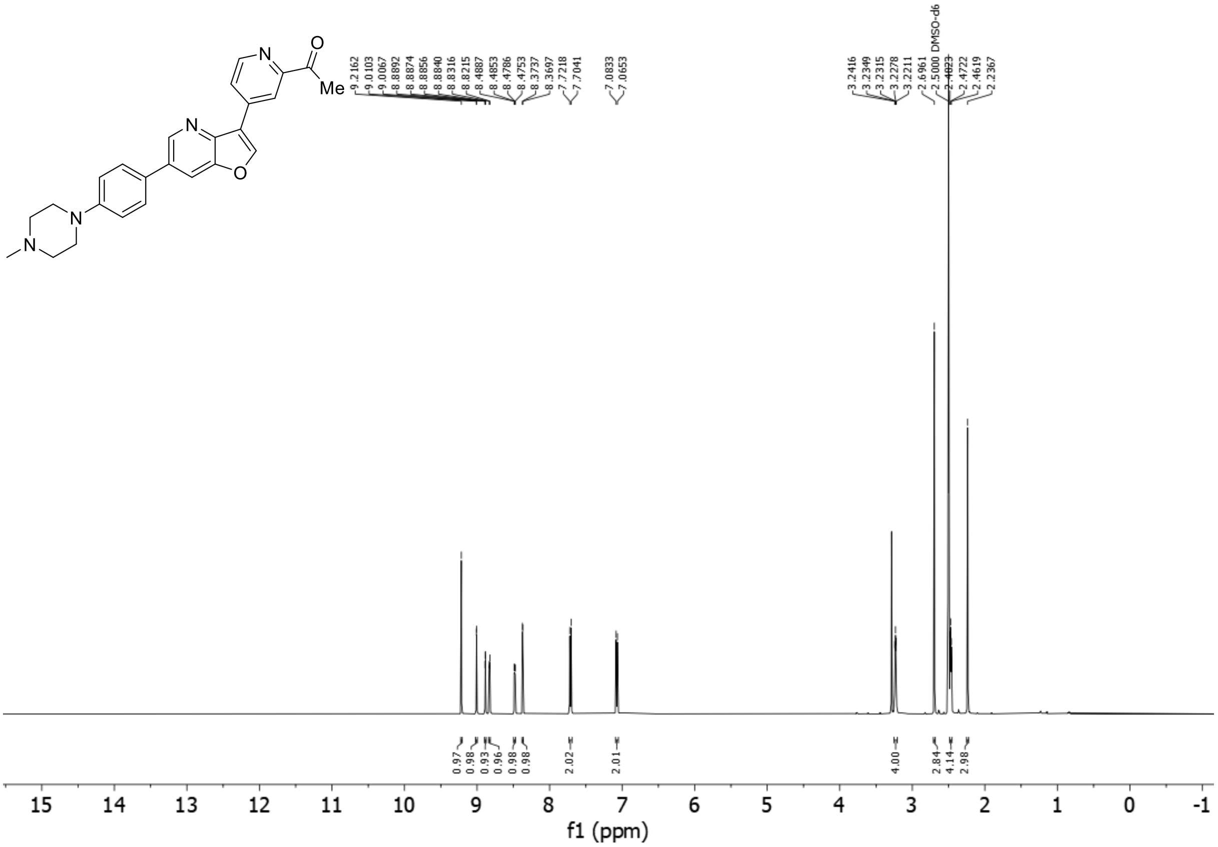

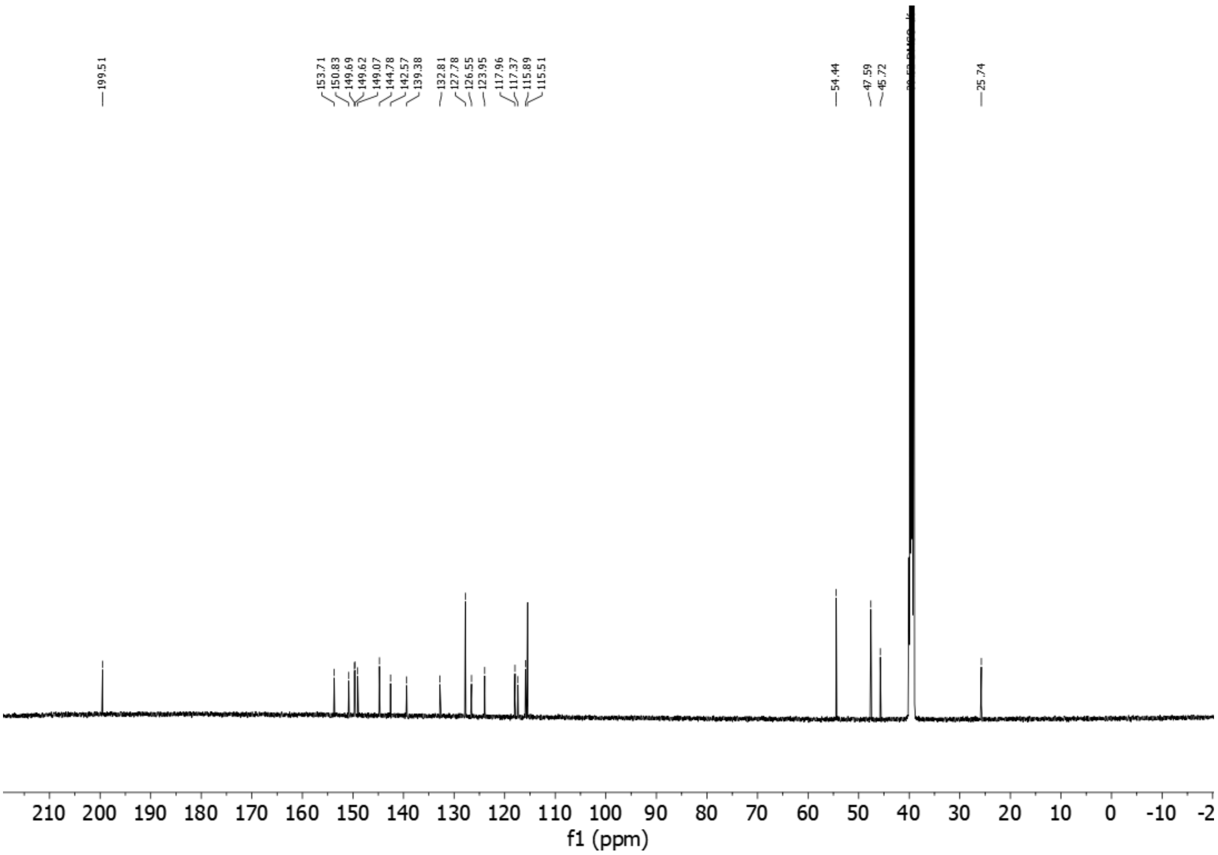

FT-IR spectrum (neat) of **57**.

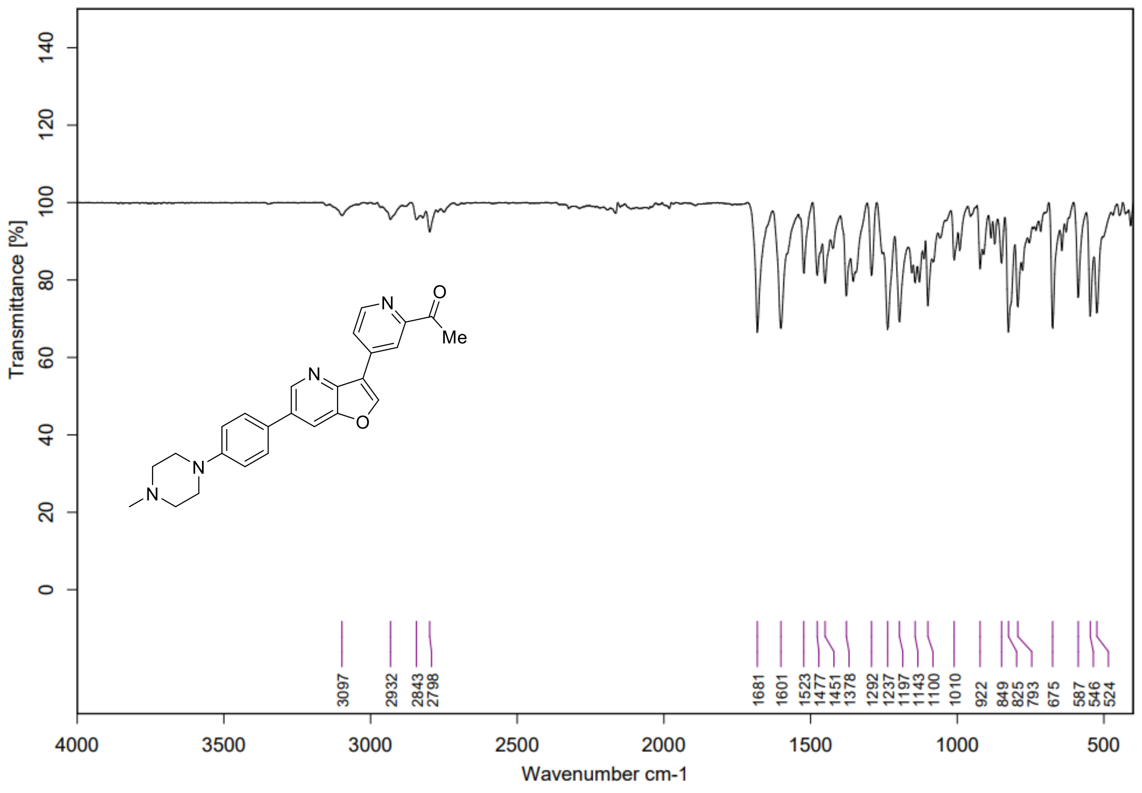

HRMS spectrum of **57.**

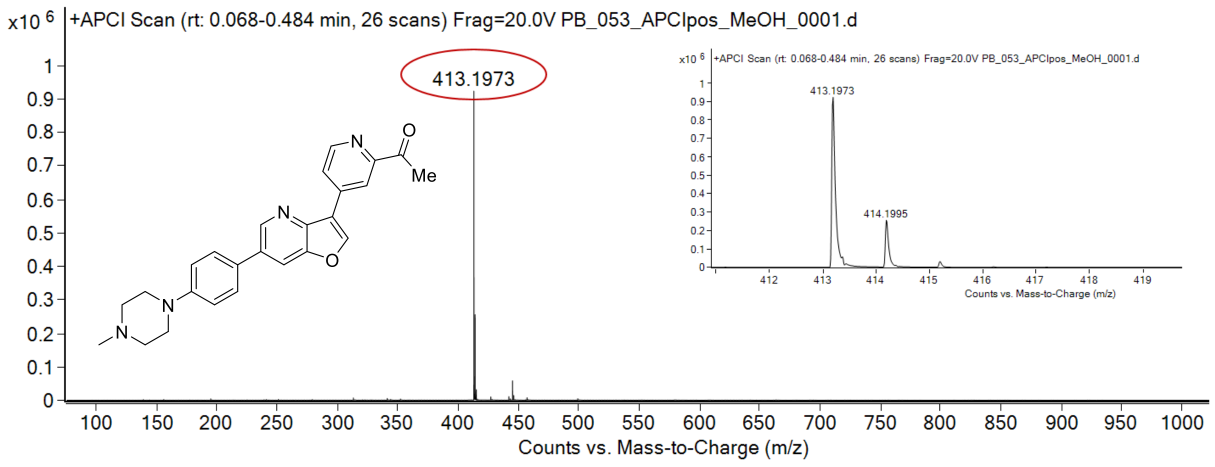

^{1}H (500 MHz) and ^{13}C NMR (126 MHz) spectra of **58** in chloroform-*d*.

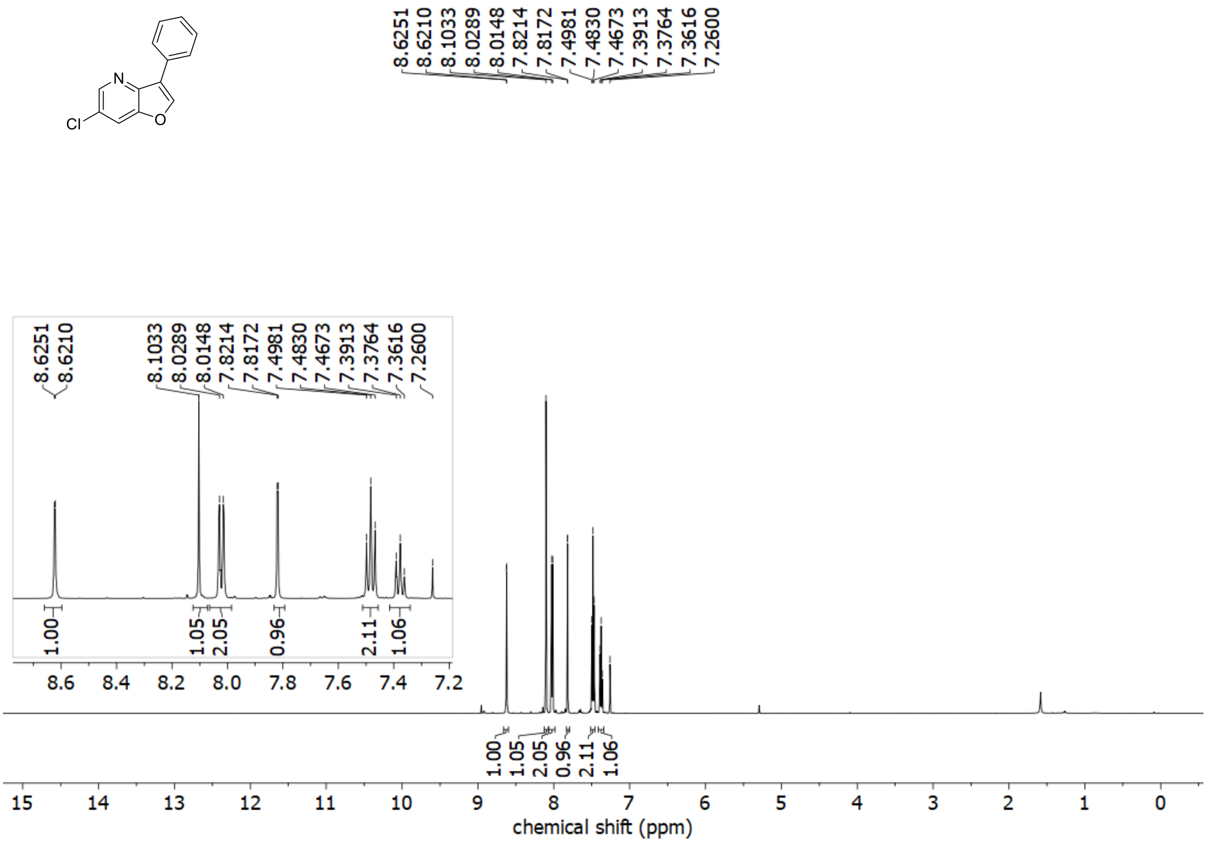

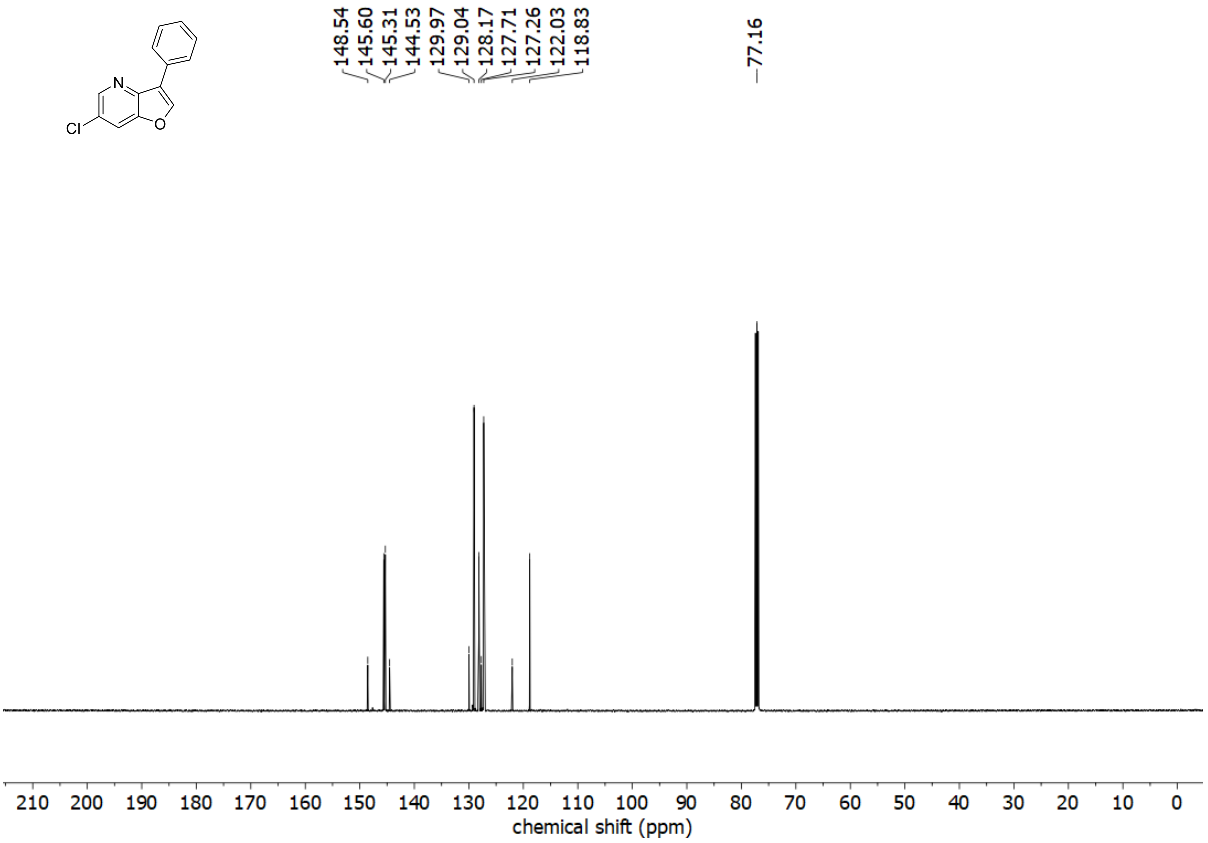

FT-IR spectrum (neat) of **58**.

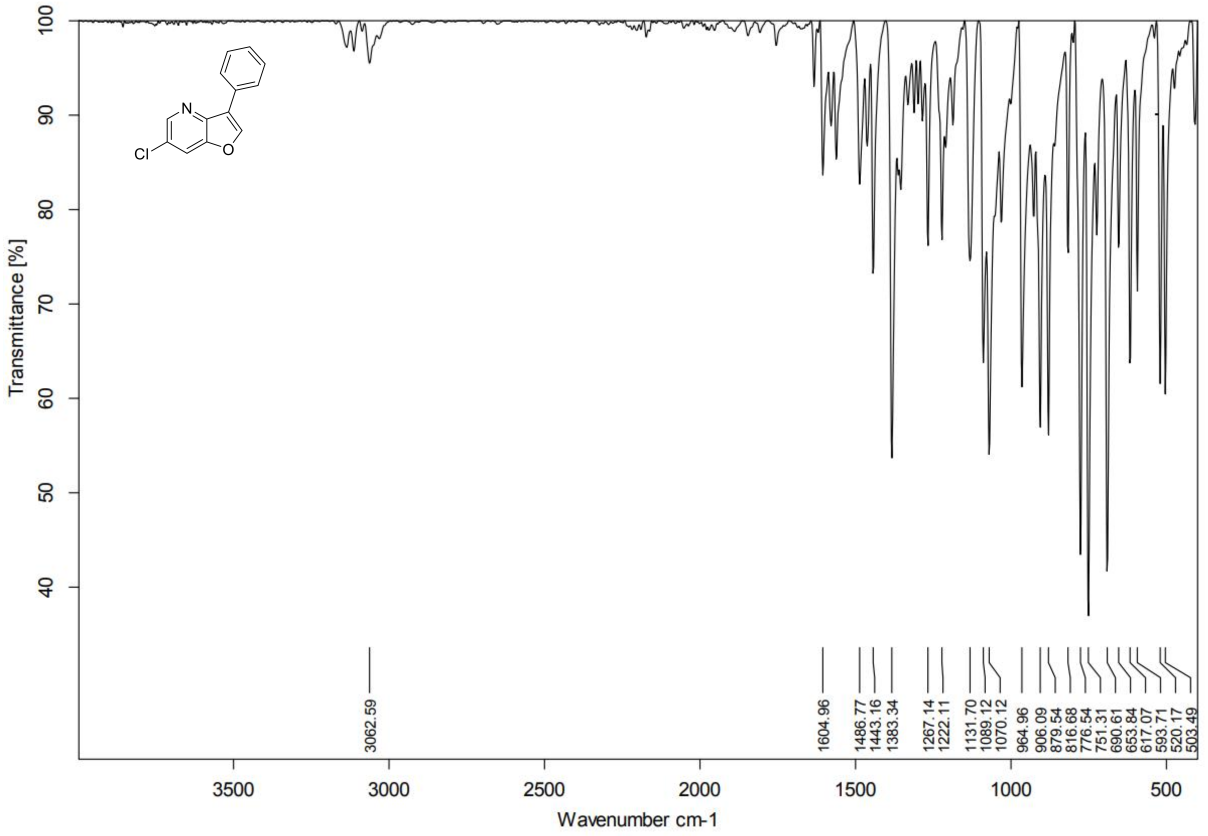

HRMS spectrum of **58**.

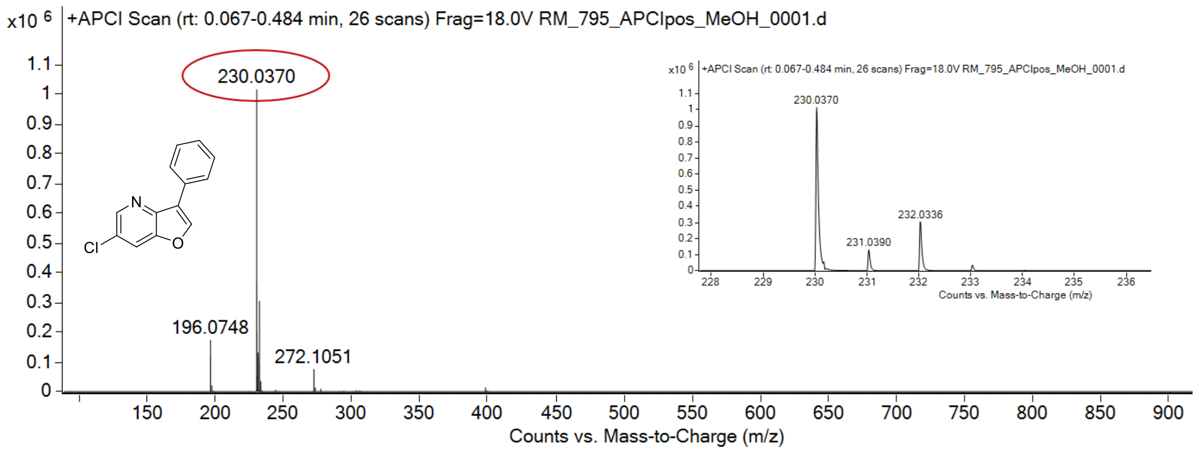

^{1}H (500 MHz) and ^{13}C NMR (126 MHz) spectra of **59** in chloroform-*d*.

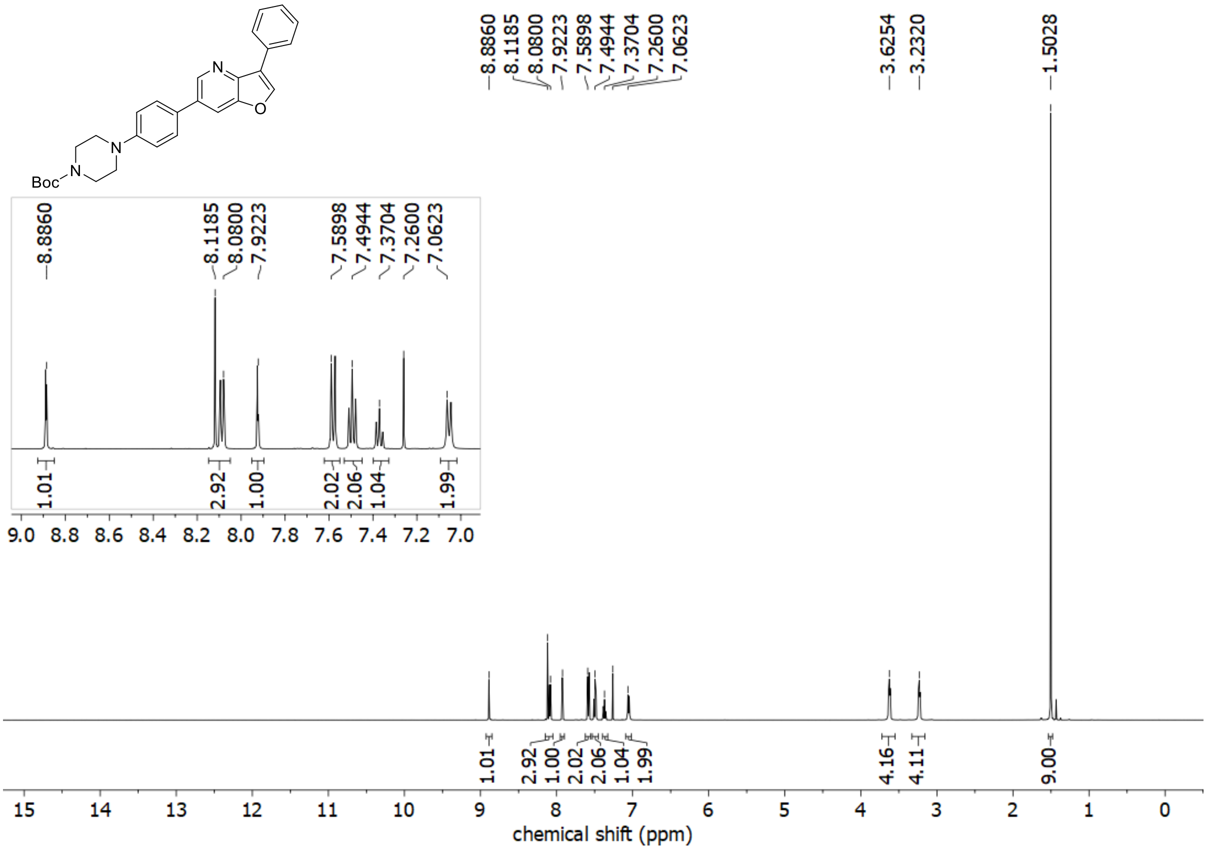

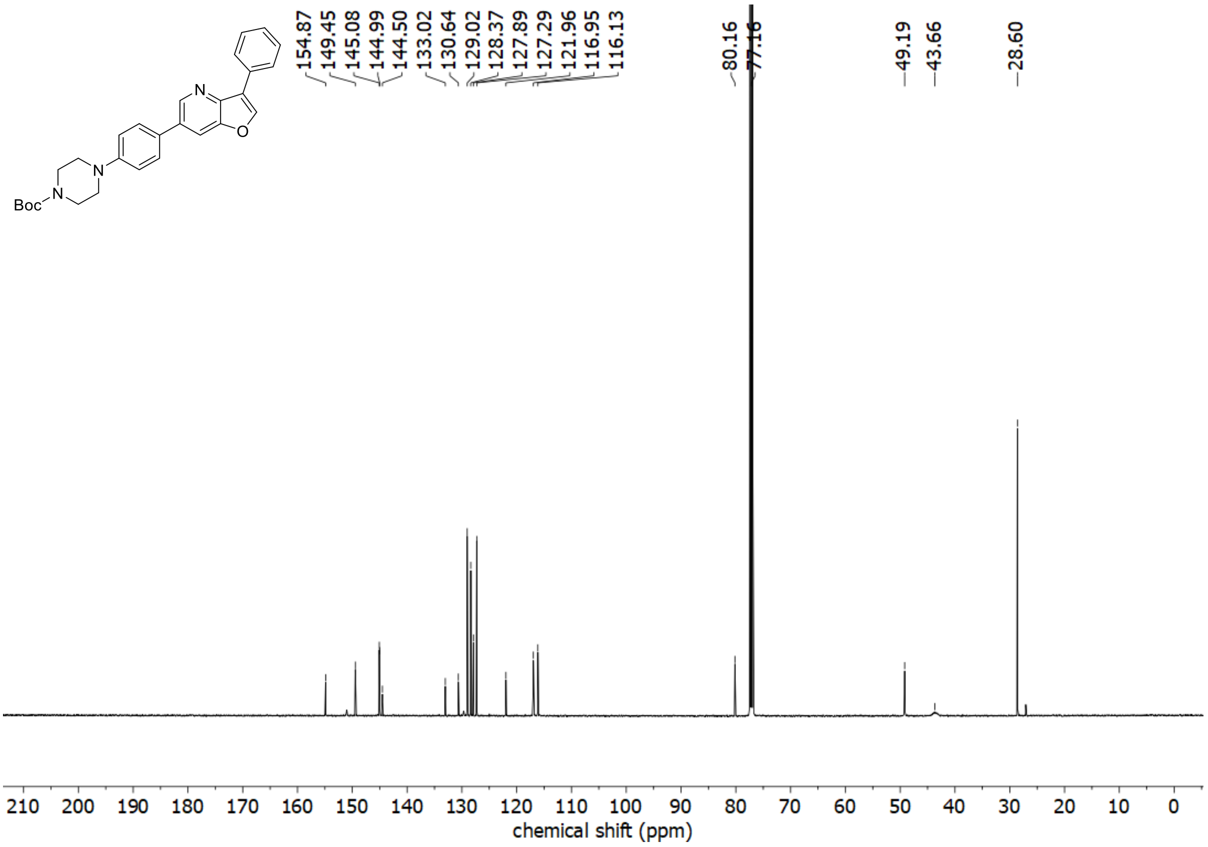

FT-IR spectrum (neat) of **59**.

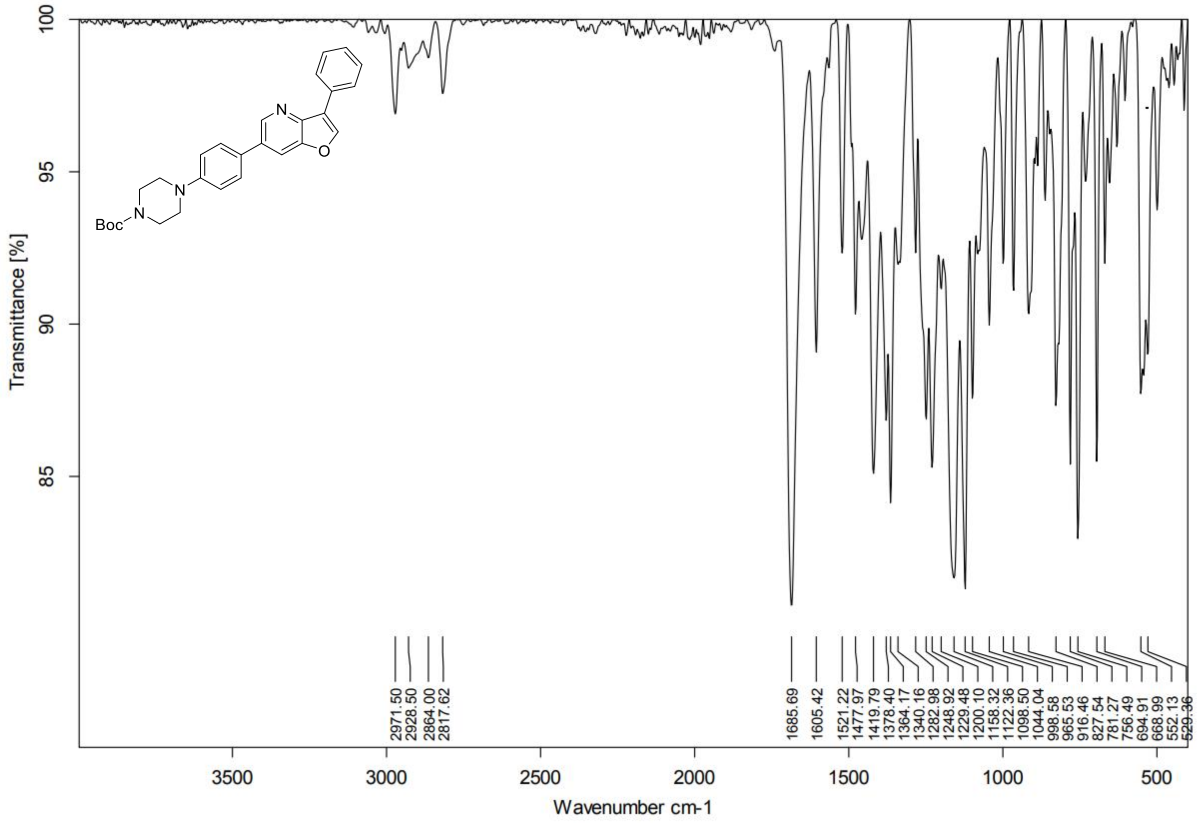

HRMS spectrum of **59**.

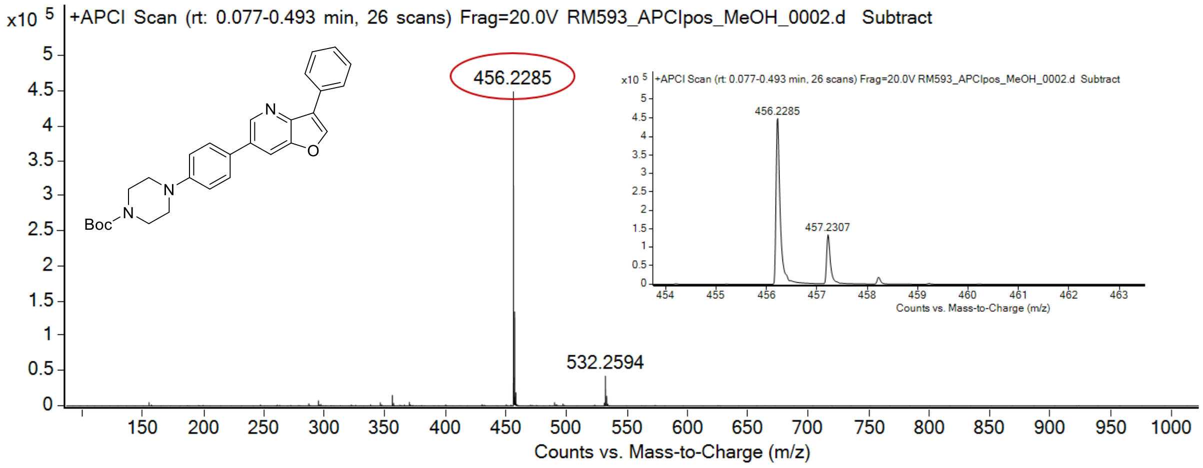

^{1}H (500 MHz) and ^{13}C NMR (126 MHz) spectra of **60** in DMSO-d_6.

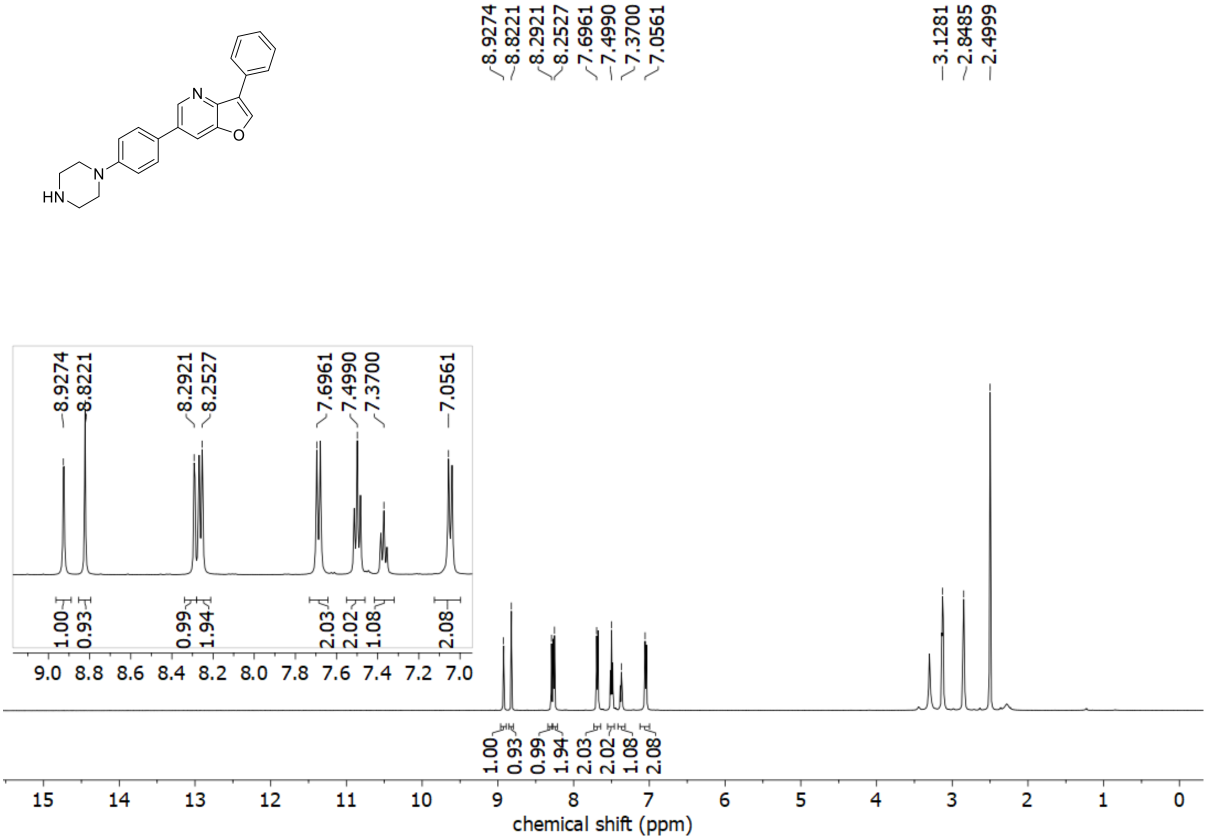

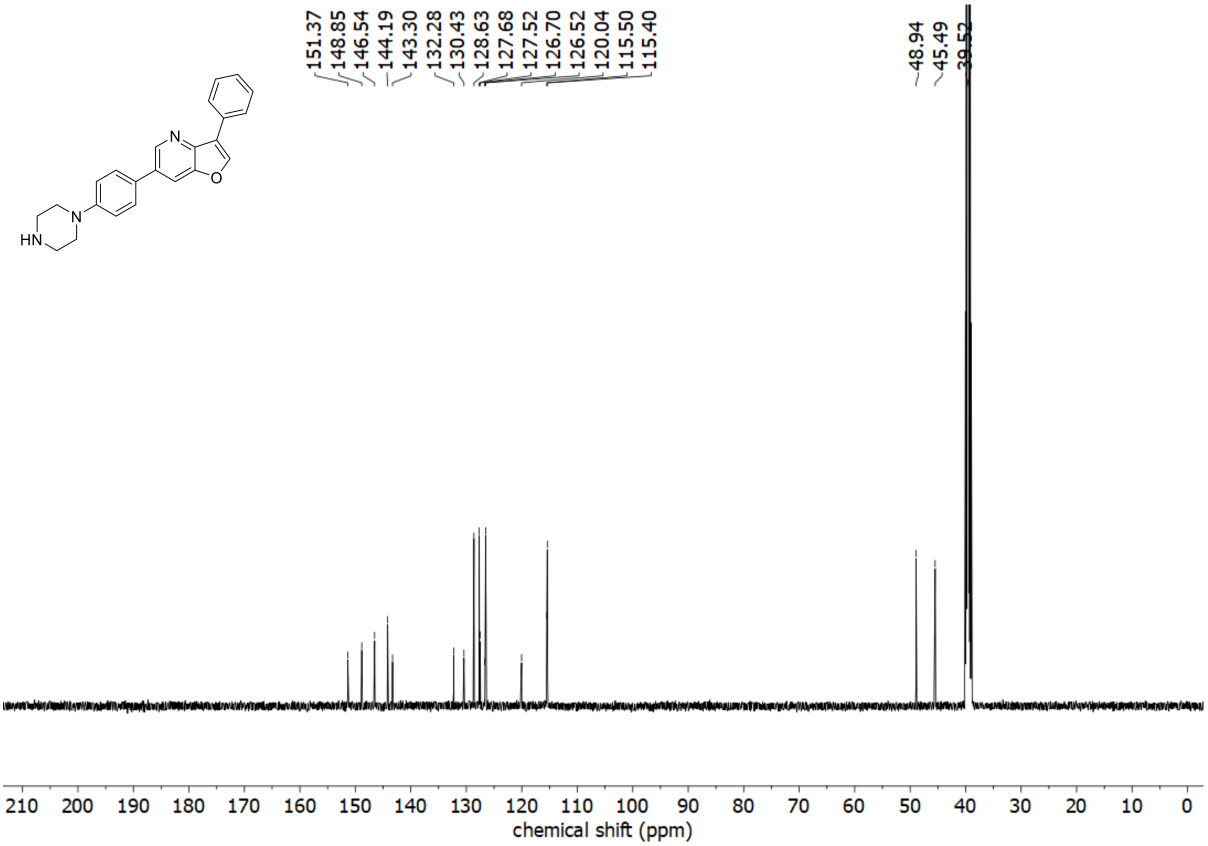

FT-IR spectrum (neat) of **60**.

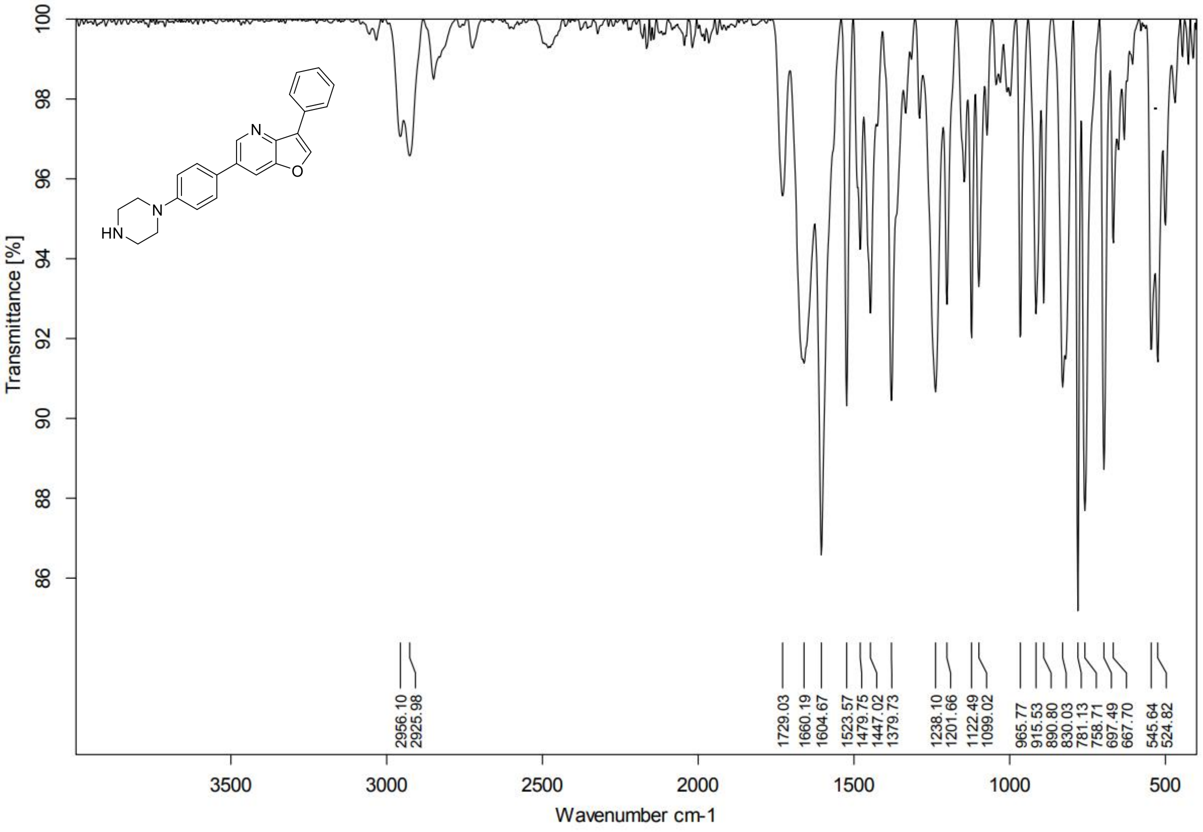

HRMS spectrum of **60**.

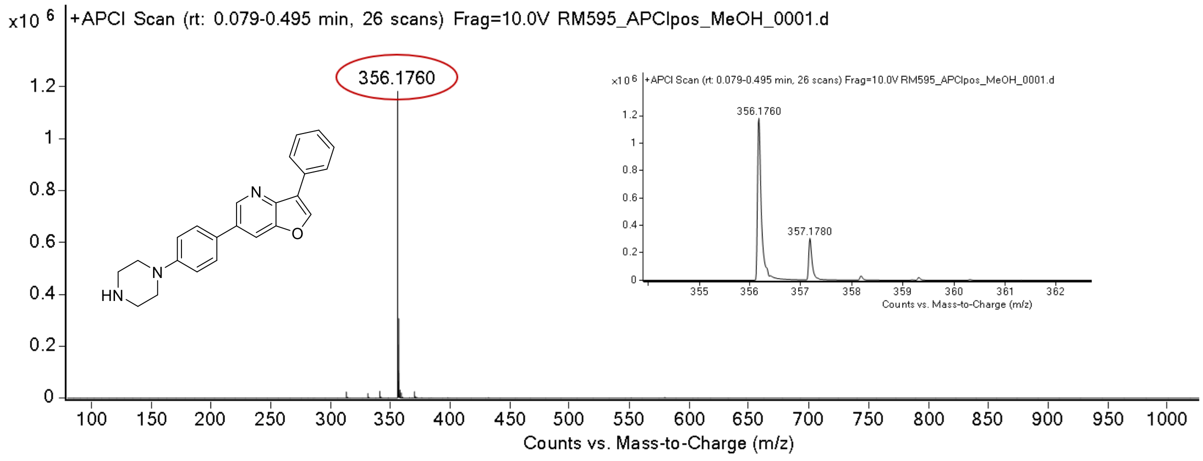

^{1}H (500 MHz) and ^{13}C NMR (126 MHz) spectra of **61** in chloroform-*d*.

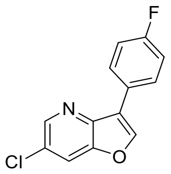

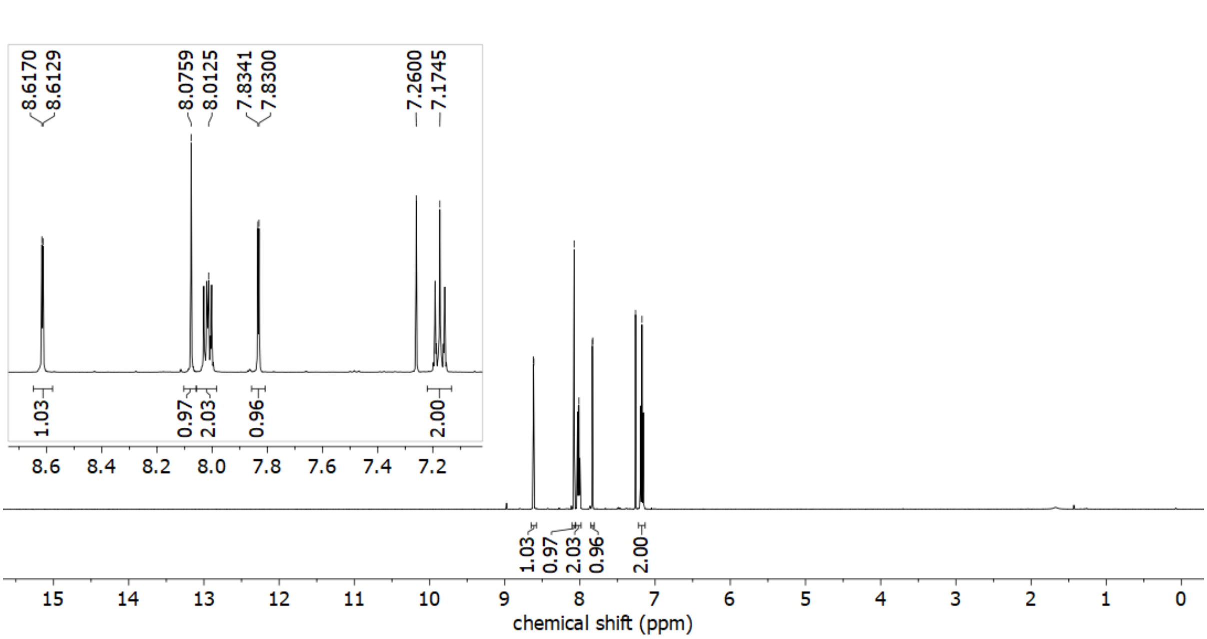

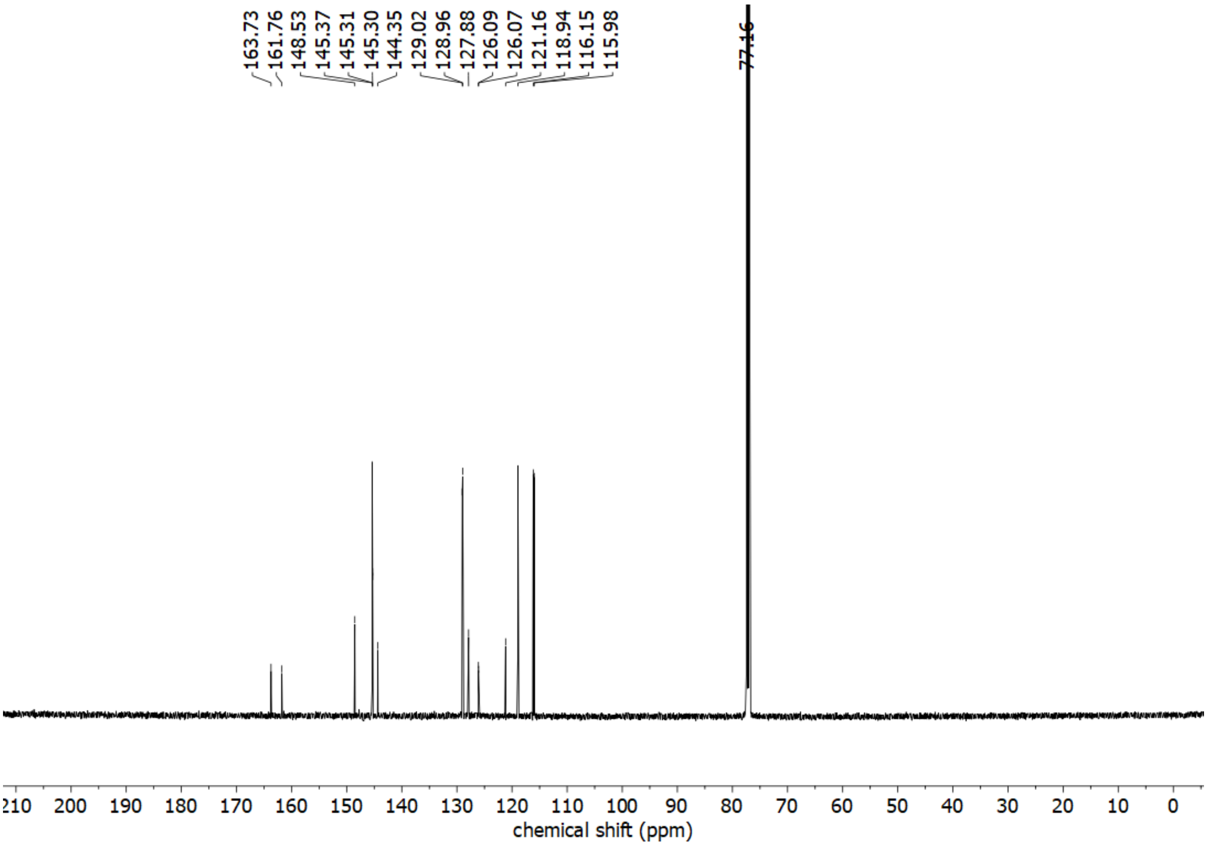

^{19}F NMR (471 MHz) spectrum of **61** in chloroform-*d*.

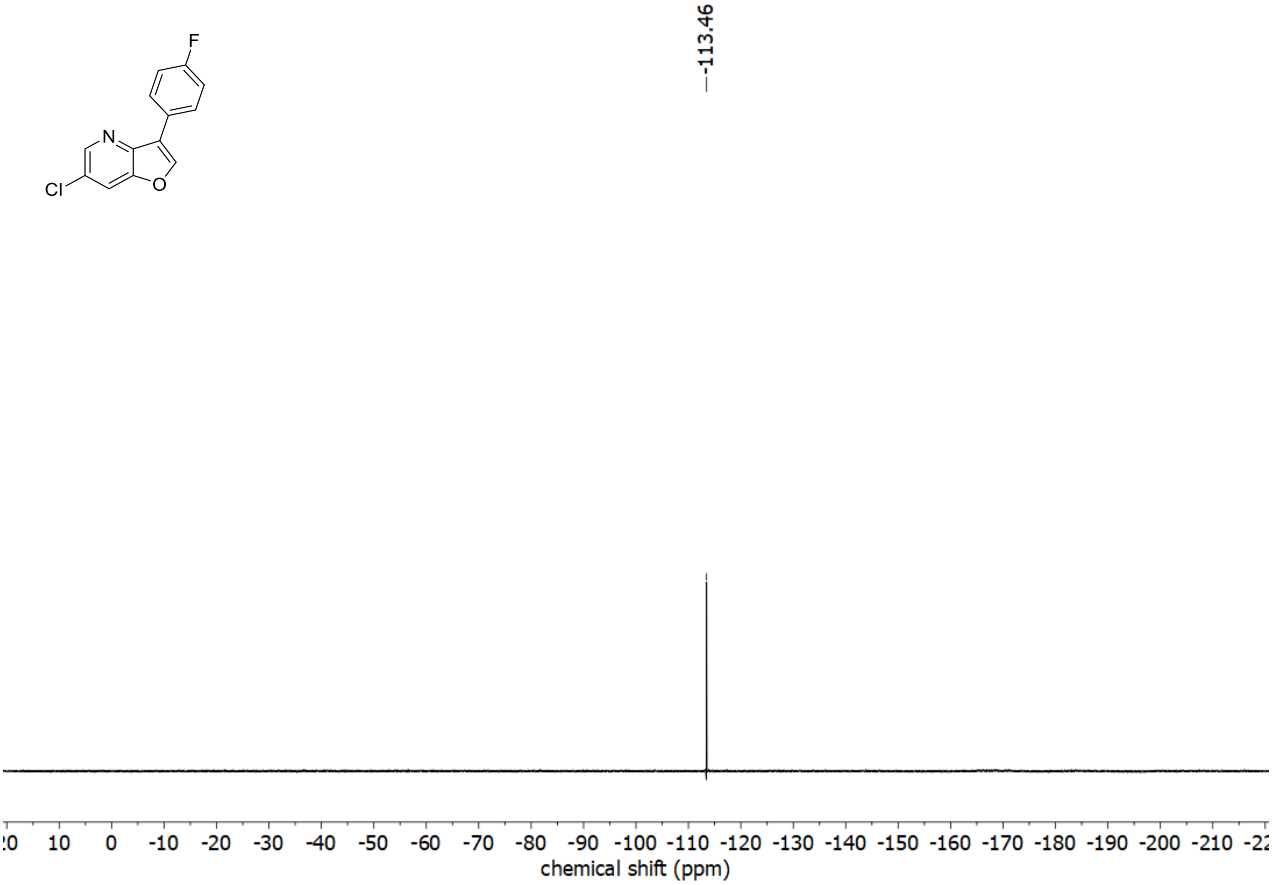

FT-IR spectrum (neat) of **61.**

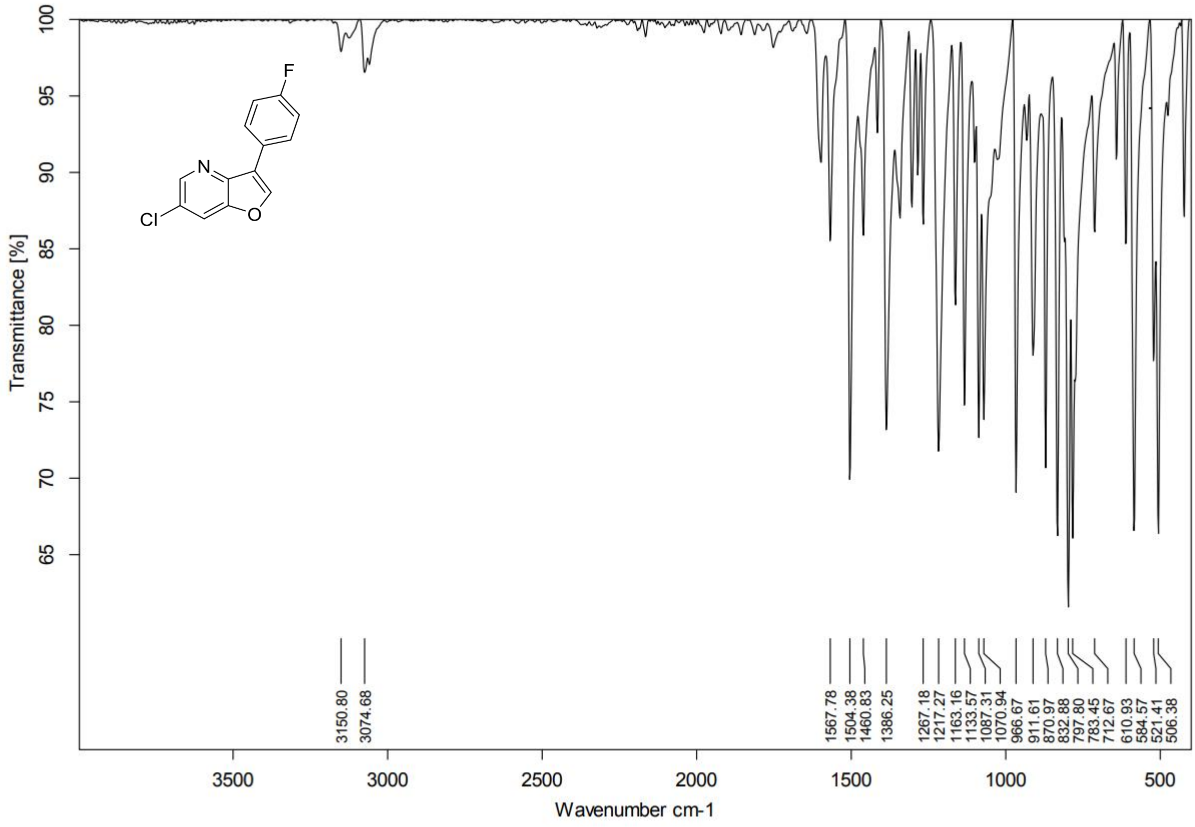

HRMS spectrum of **61.**

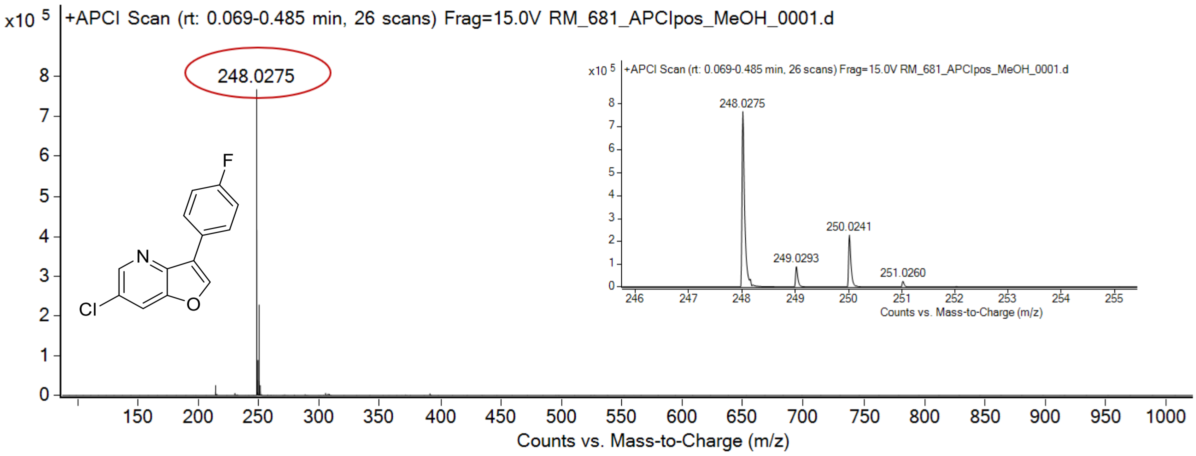

^{1}H (500 MHz) and ^{13}C NMR (126 MHz) spectra of **62** in chloroform-*d*.

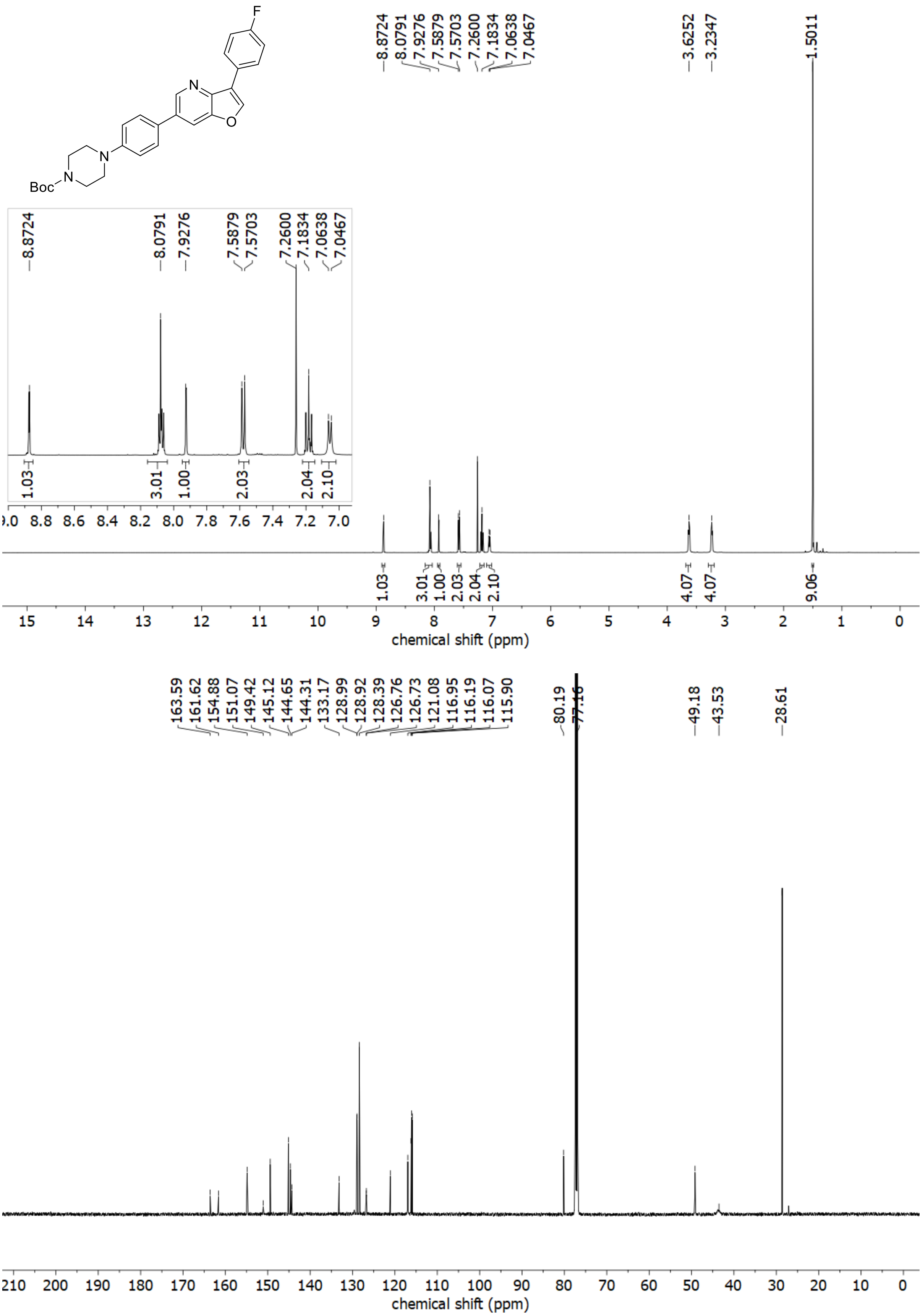

^{19}F NMR (471 MHz) of **62** in chloroform-*d*.

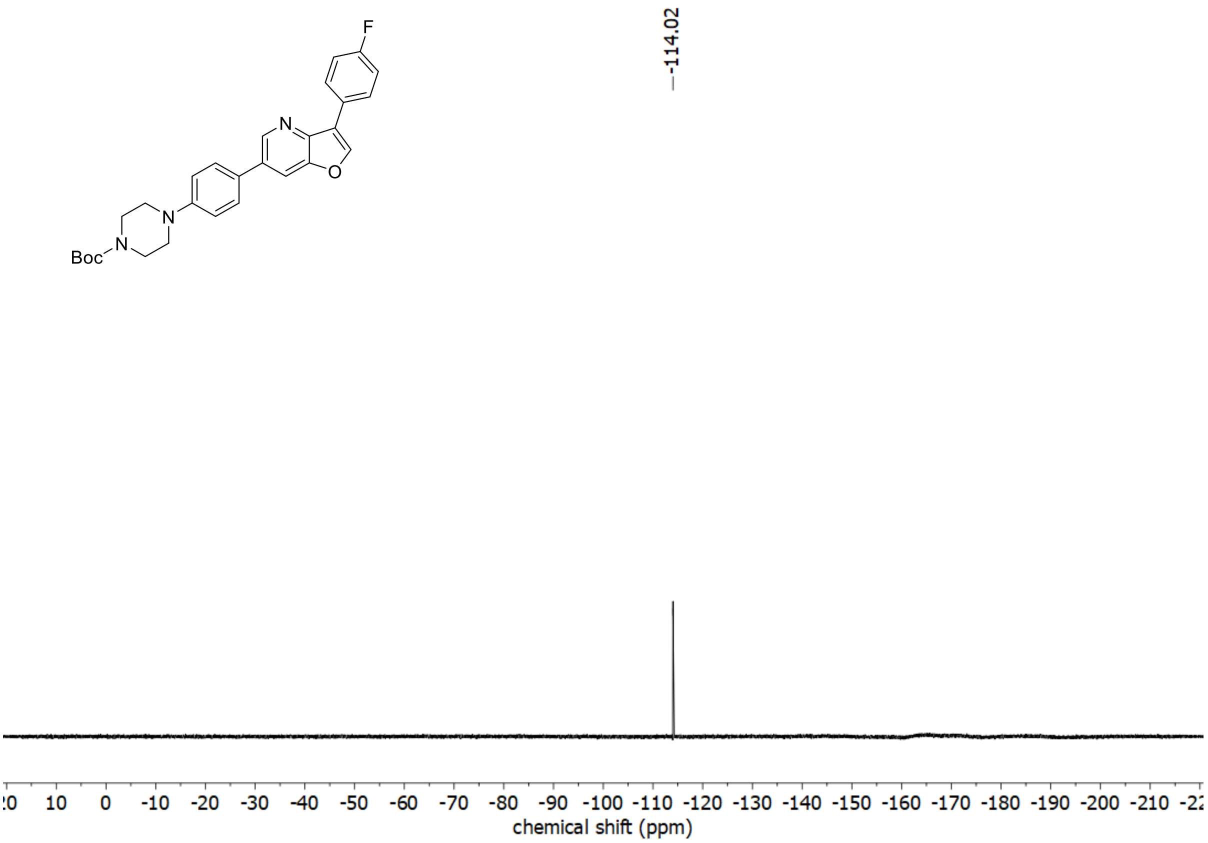

FT-IR spectrum (neat) of **62**.

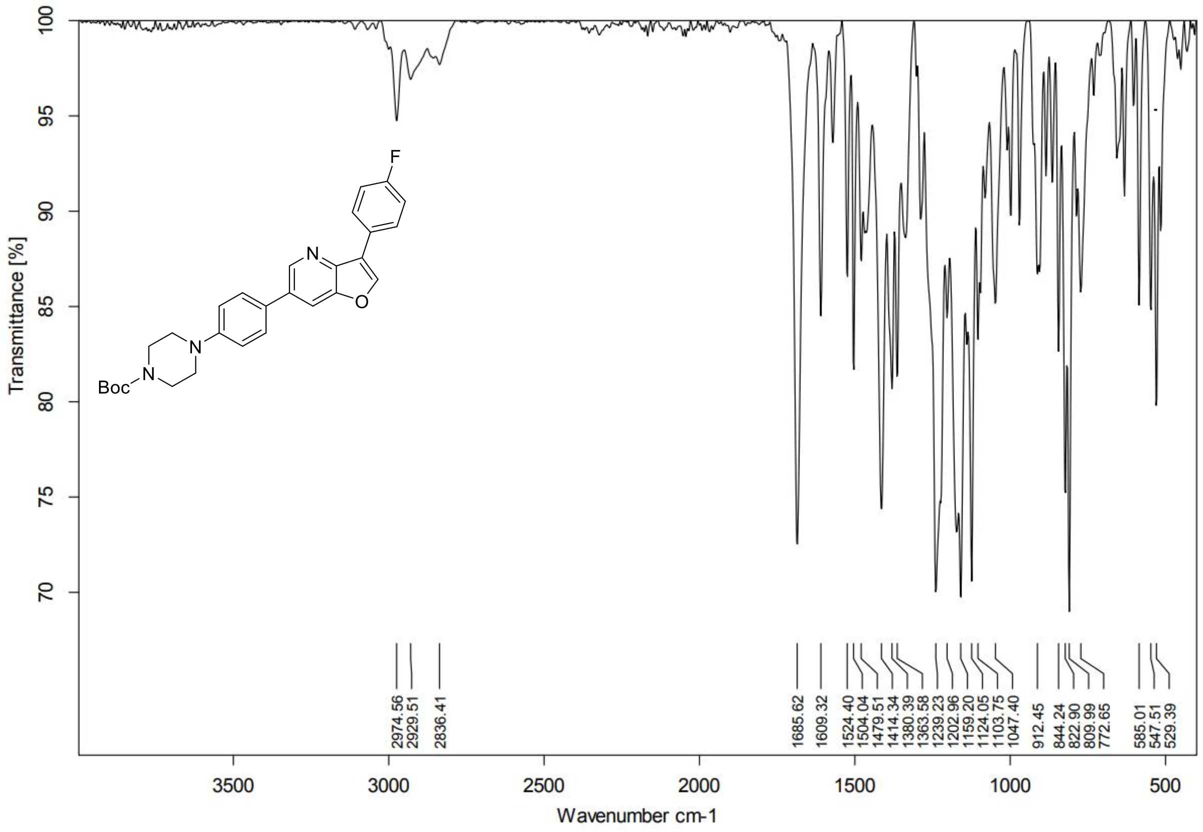

HRMS spectrum of **62.**

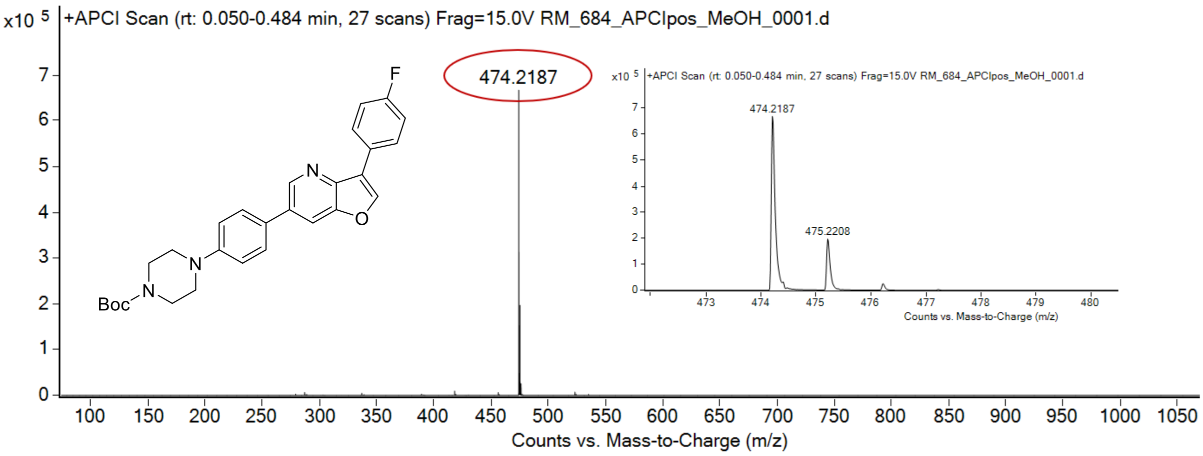

^{1}H (500 MHz) and ^{13}C NMR (126 MHz) spectra of **63** in DMSO-d_6.

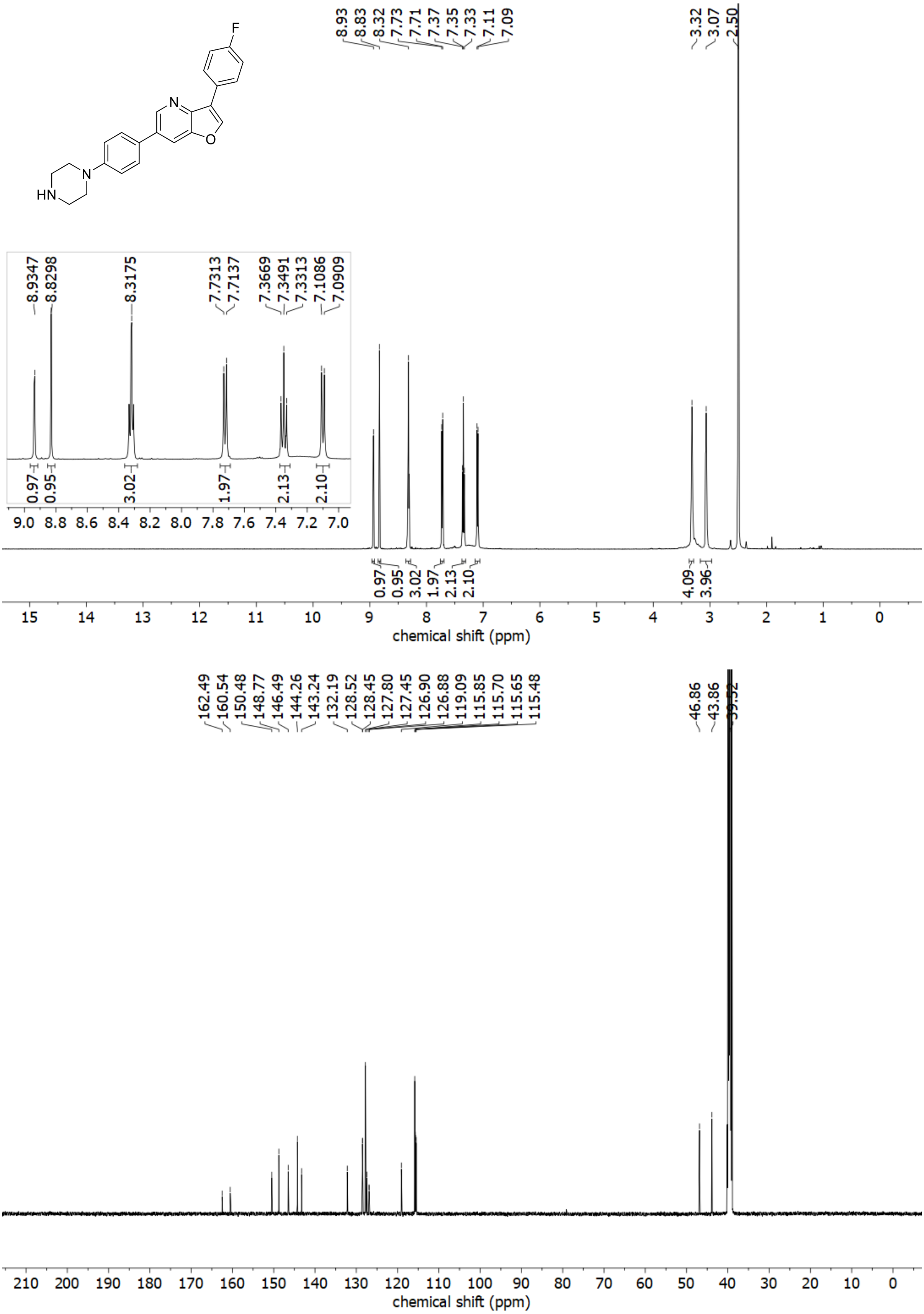

FT-IR spectrum (neat) of **63.**

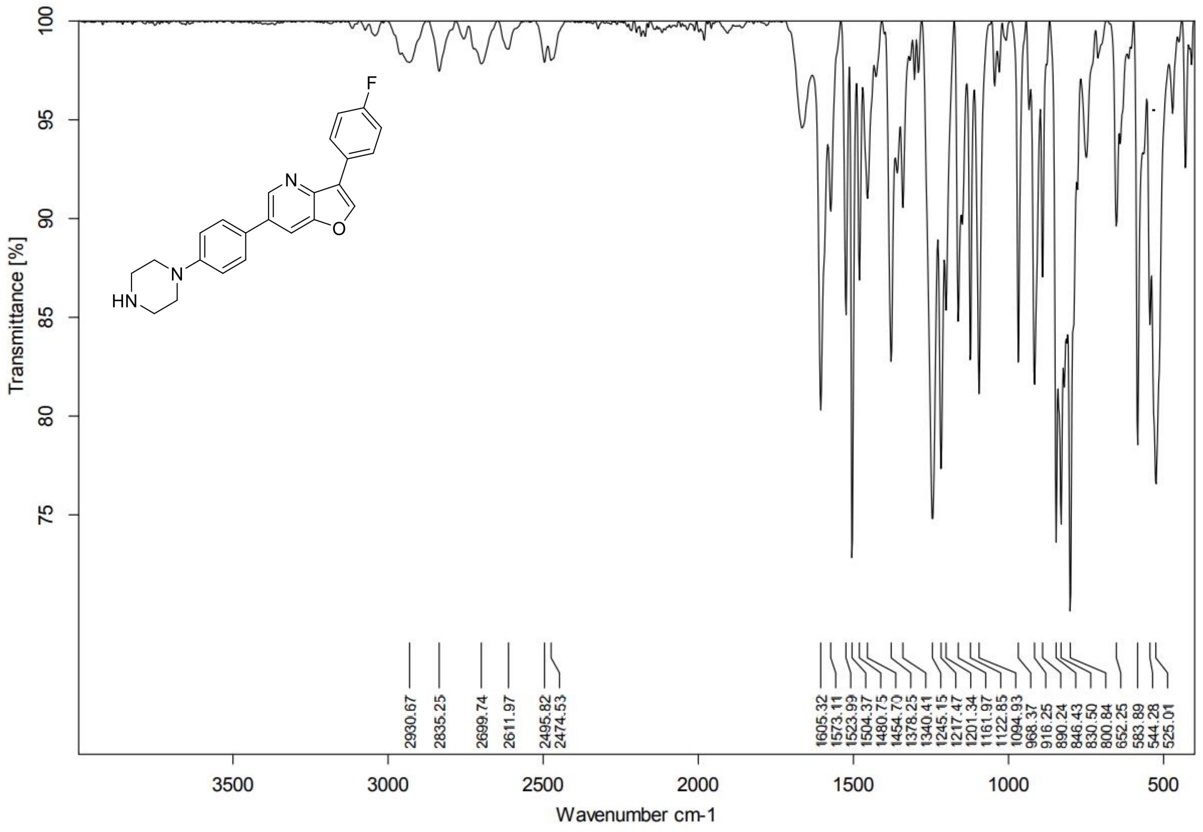

HRMS spectrum of **63.**

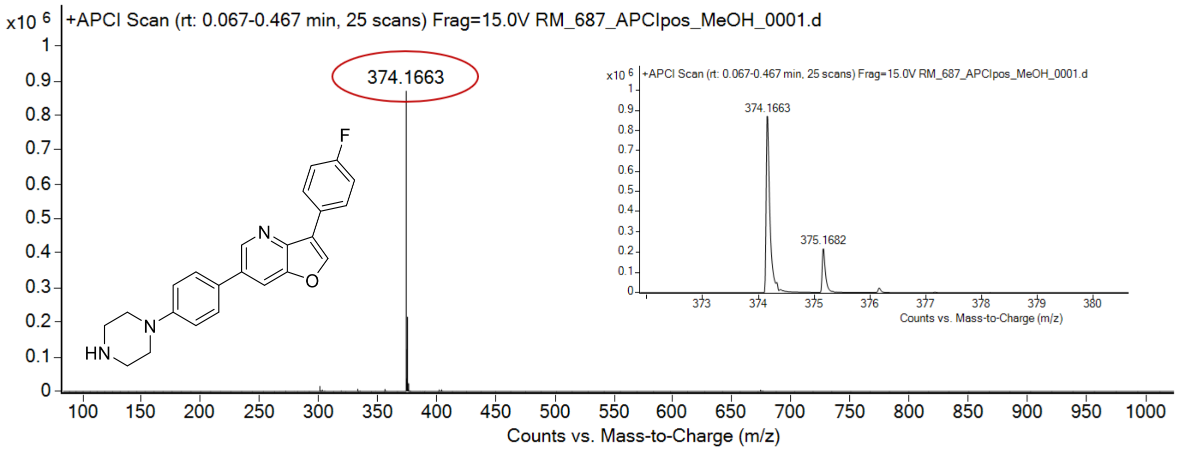

^{1}H (500 MHz) and ^{13}C NMR (126 MHz) spectra of **64** in chloroform-*d*.

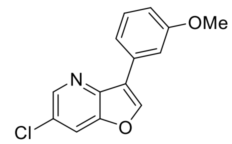

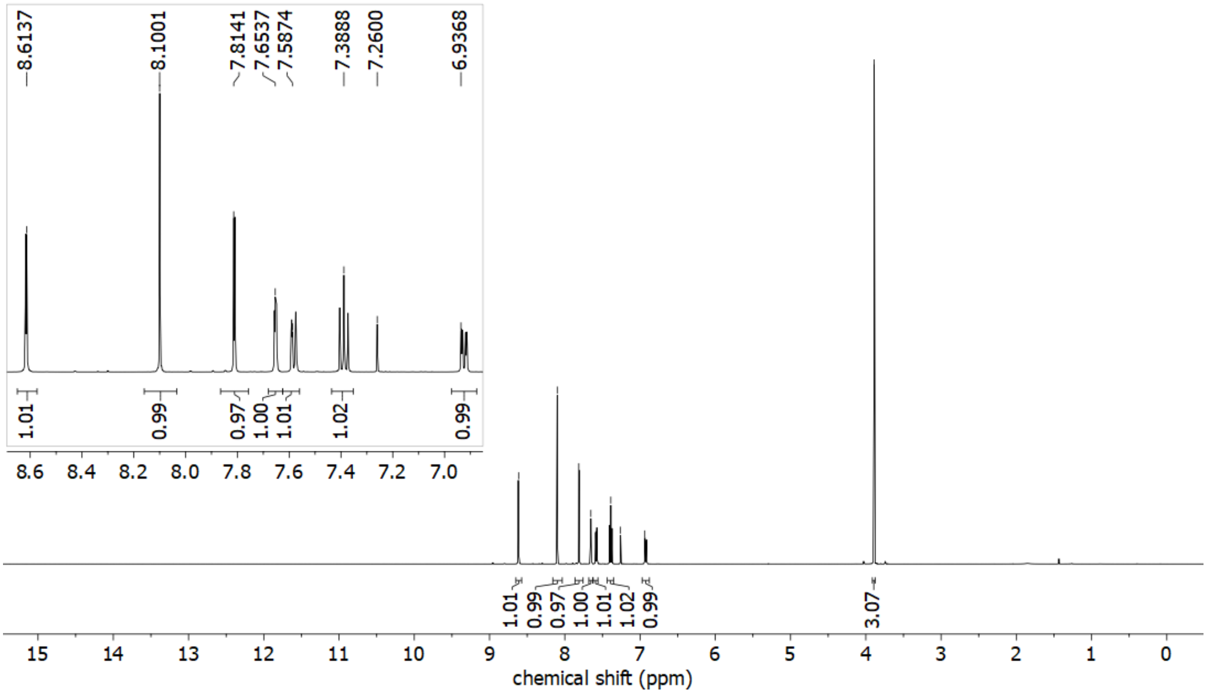

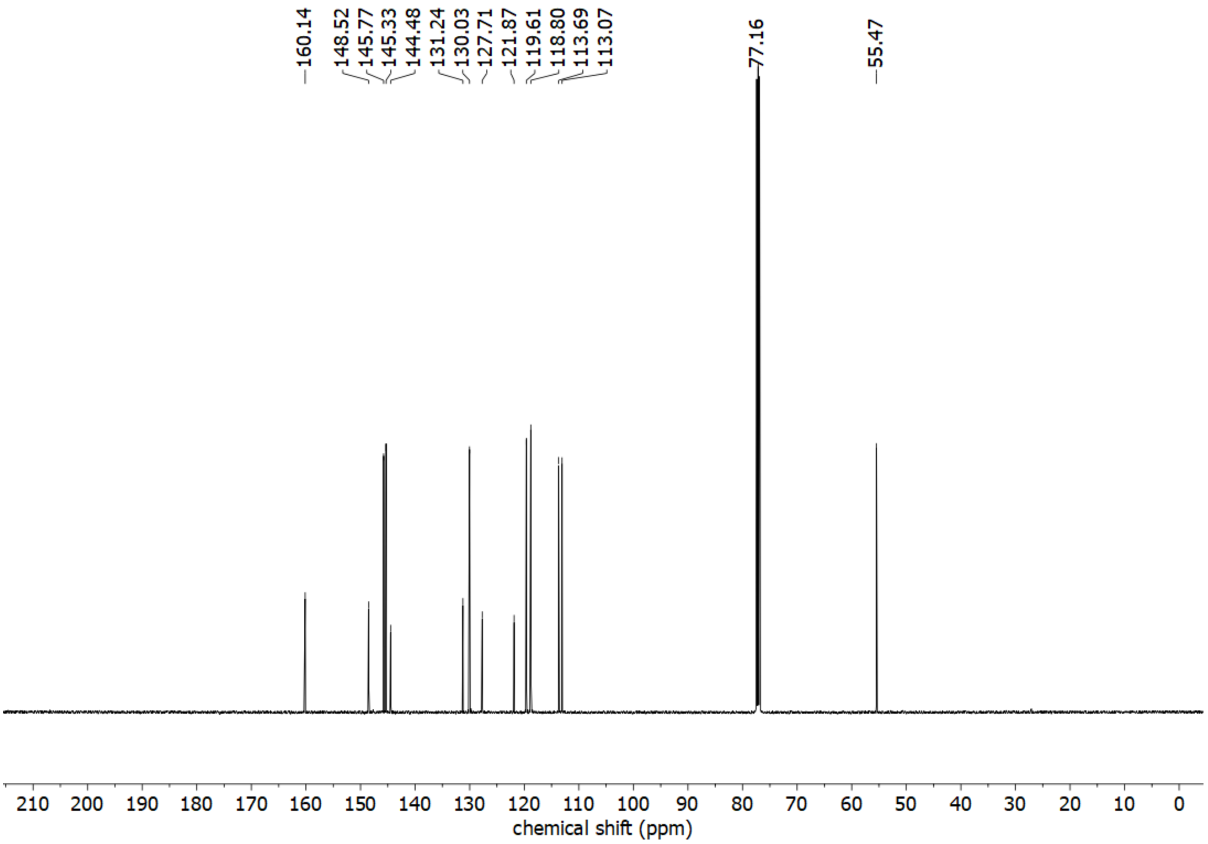

FT-IR spectrum (neat) of **64**.

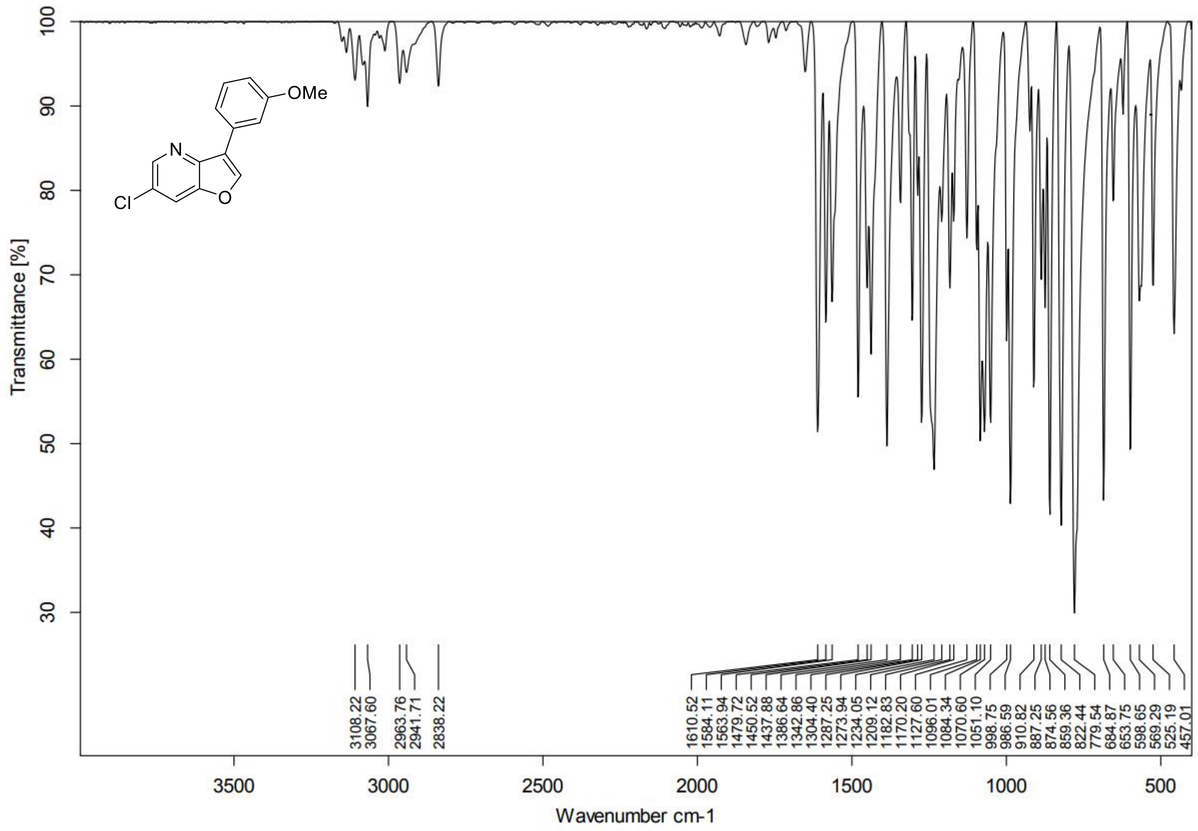

HRMS spectrum of **64.**

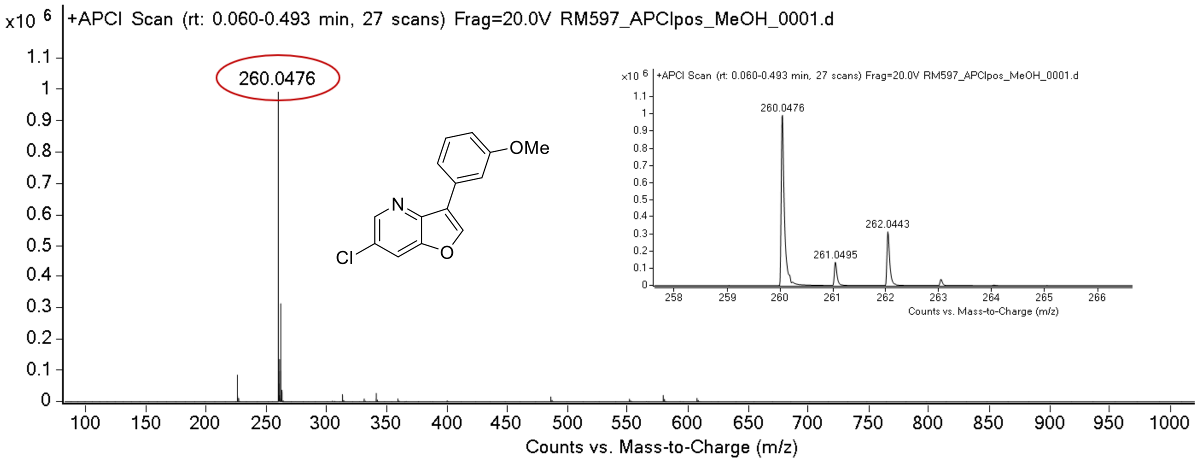

^{1}H (500 MHz) and ^{13}C NMR (126 MHz) spectra of **65** in chloroform-*d*.

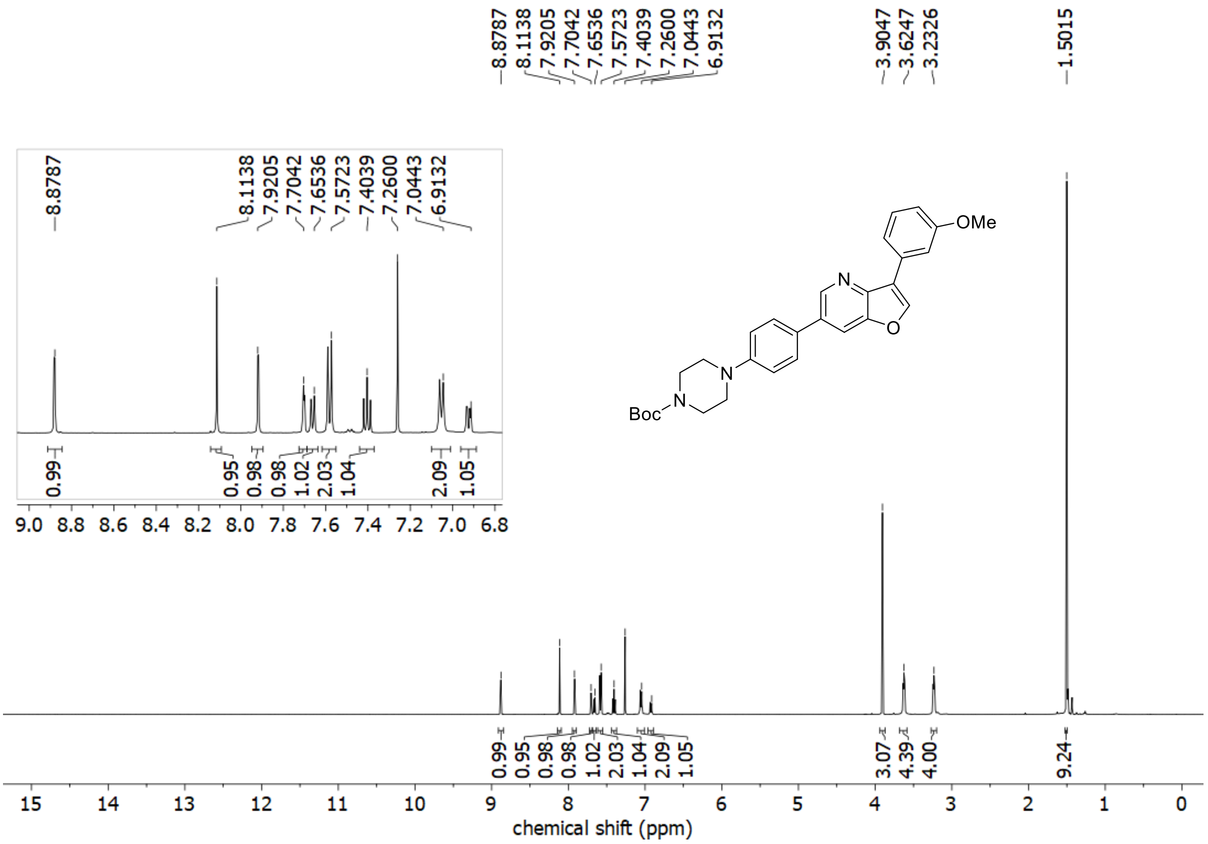

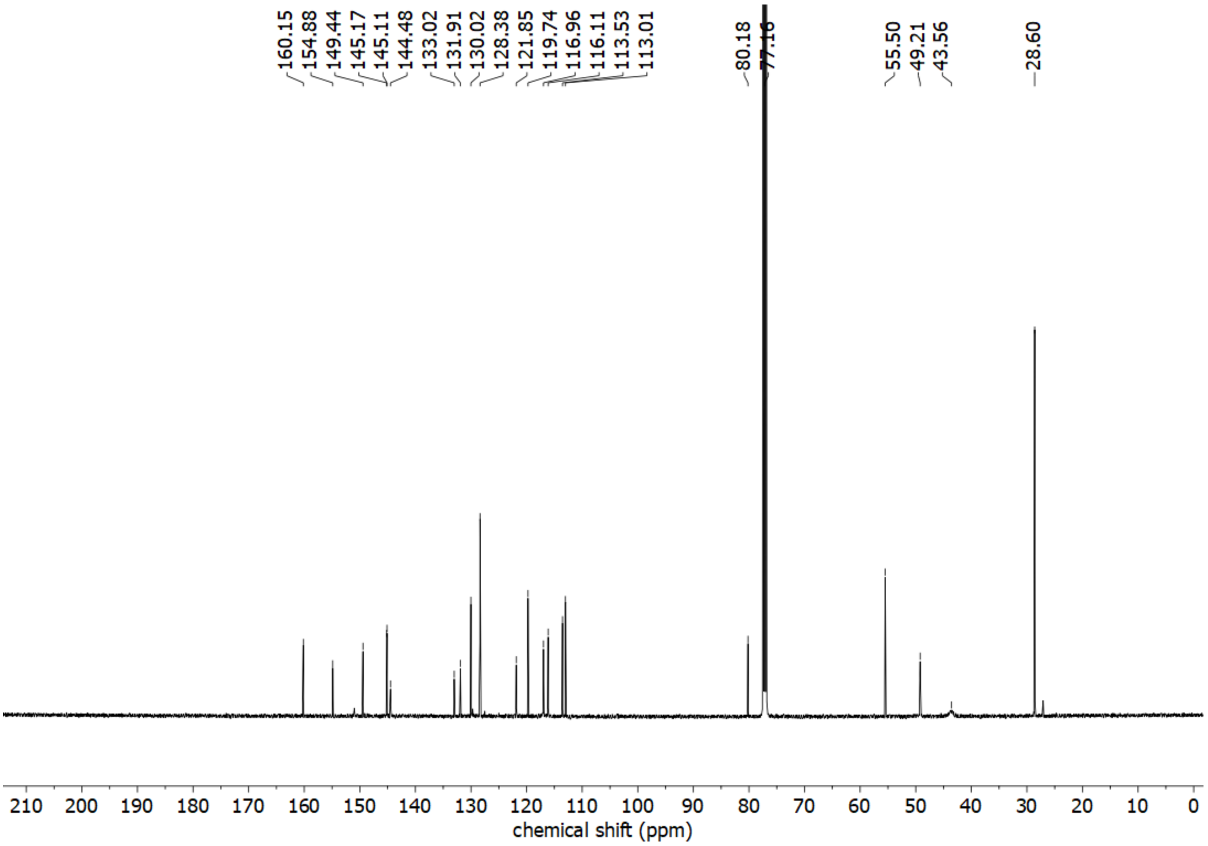

FT-IR spectrum (neat) of **65.**

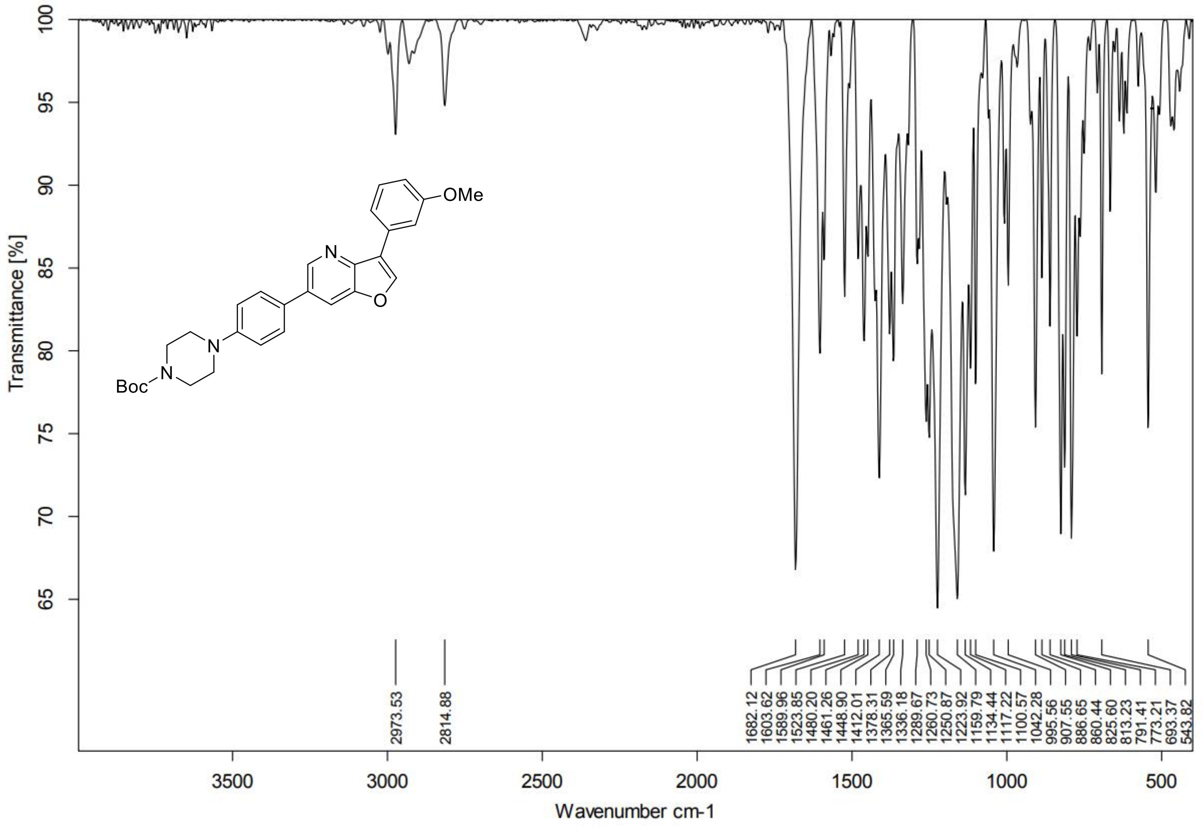

HRMS spectrum of **65.**

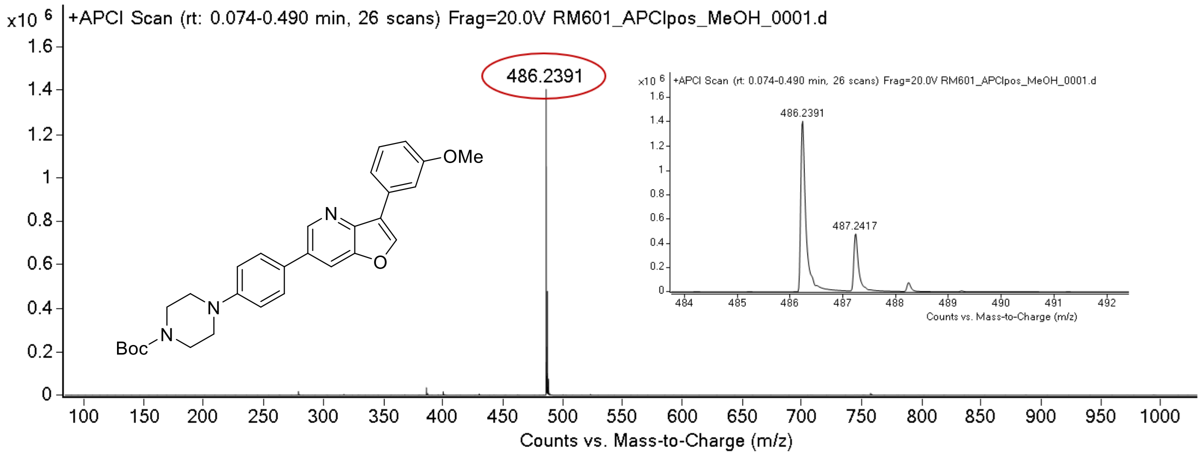

^{1}H (500 MHz) and ^{13}C NMR (126 MHz) spectra of **66** in DMSO-d_6.

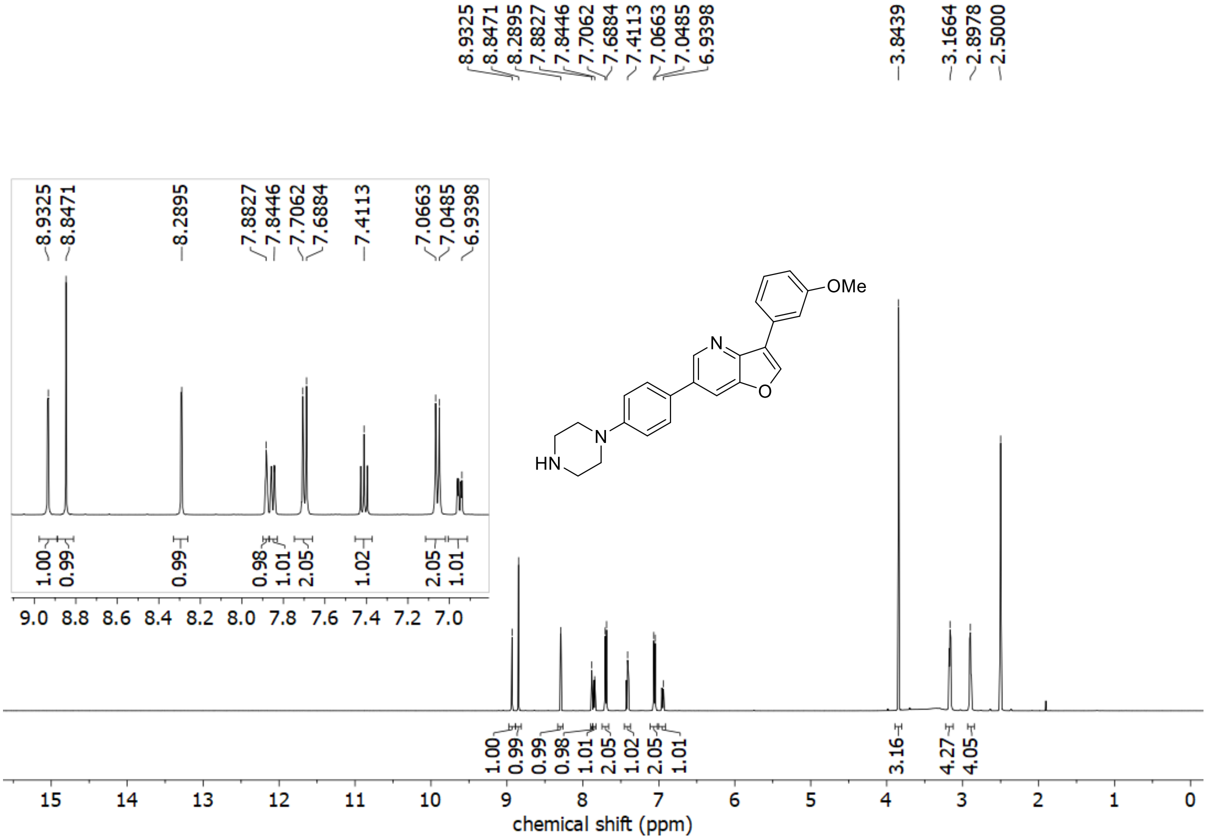

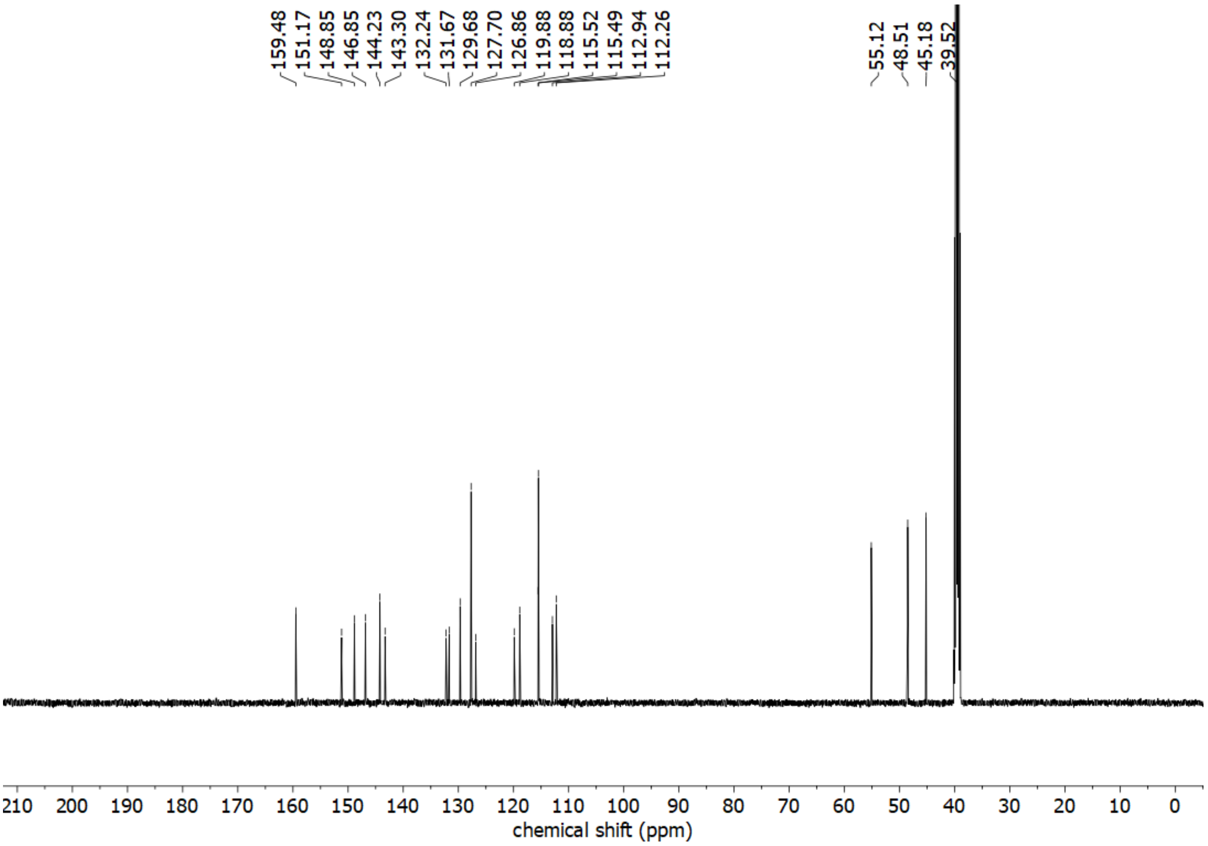

FT-IR spectrum (neat) of **66**.

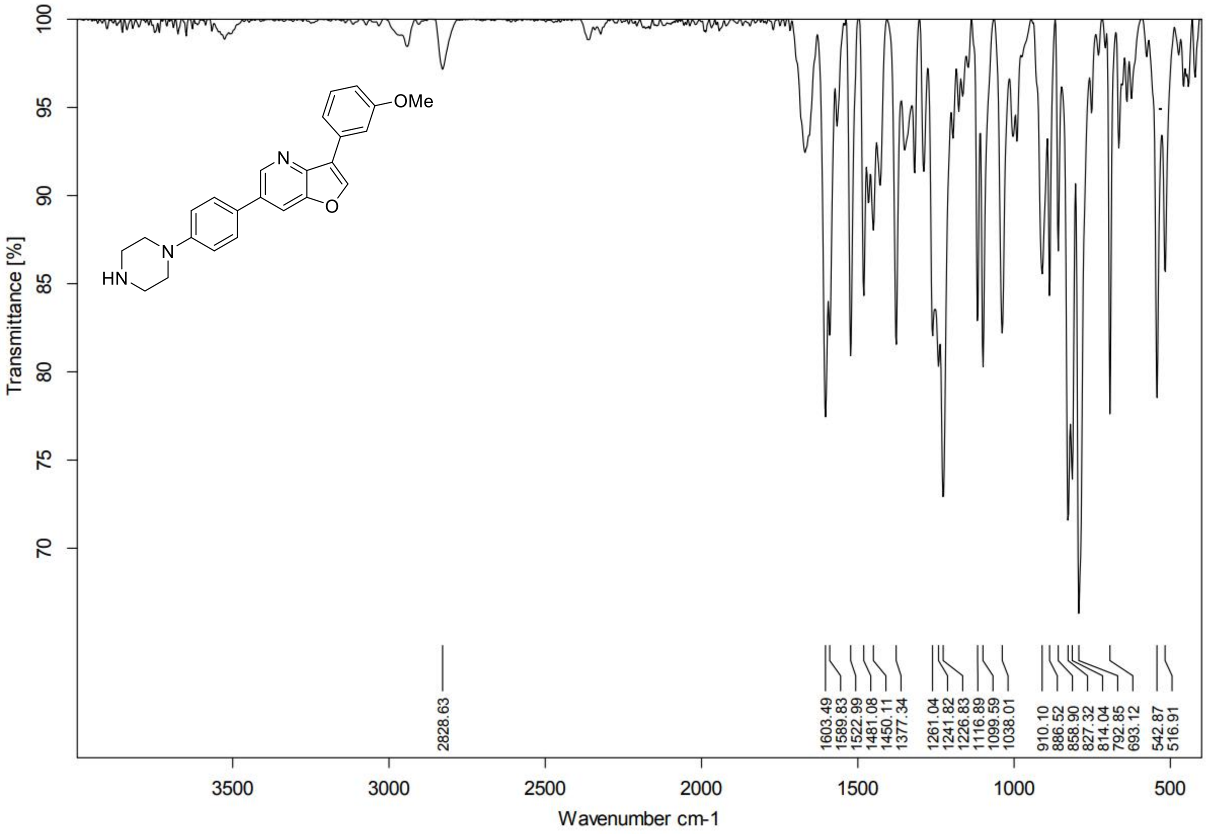

HRMS spectrum of **66.**

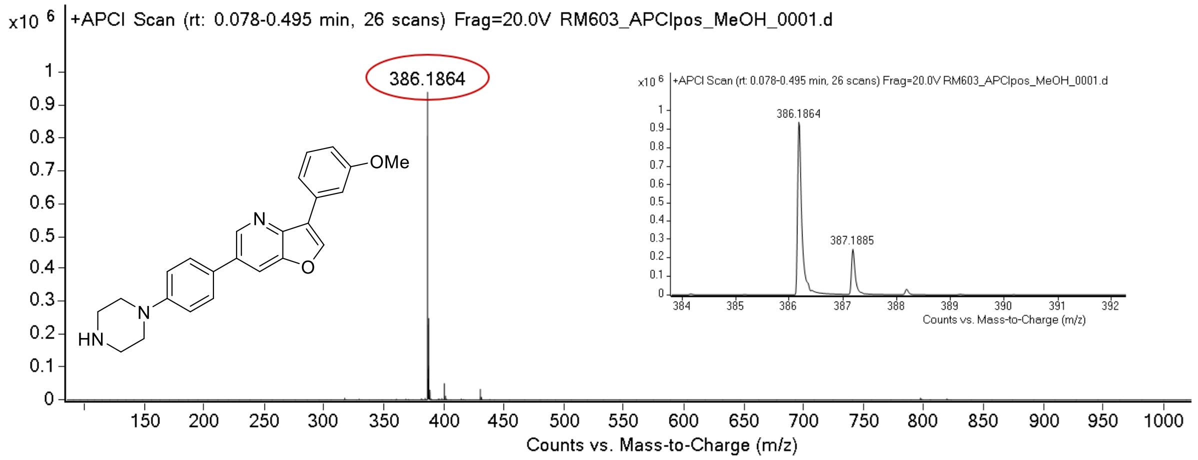

^{1}H (500 MHz) and ^{13}C NMR (126 MHz) spectra of **67** in chloroform-*d*.

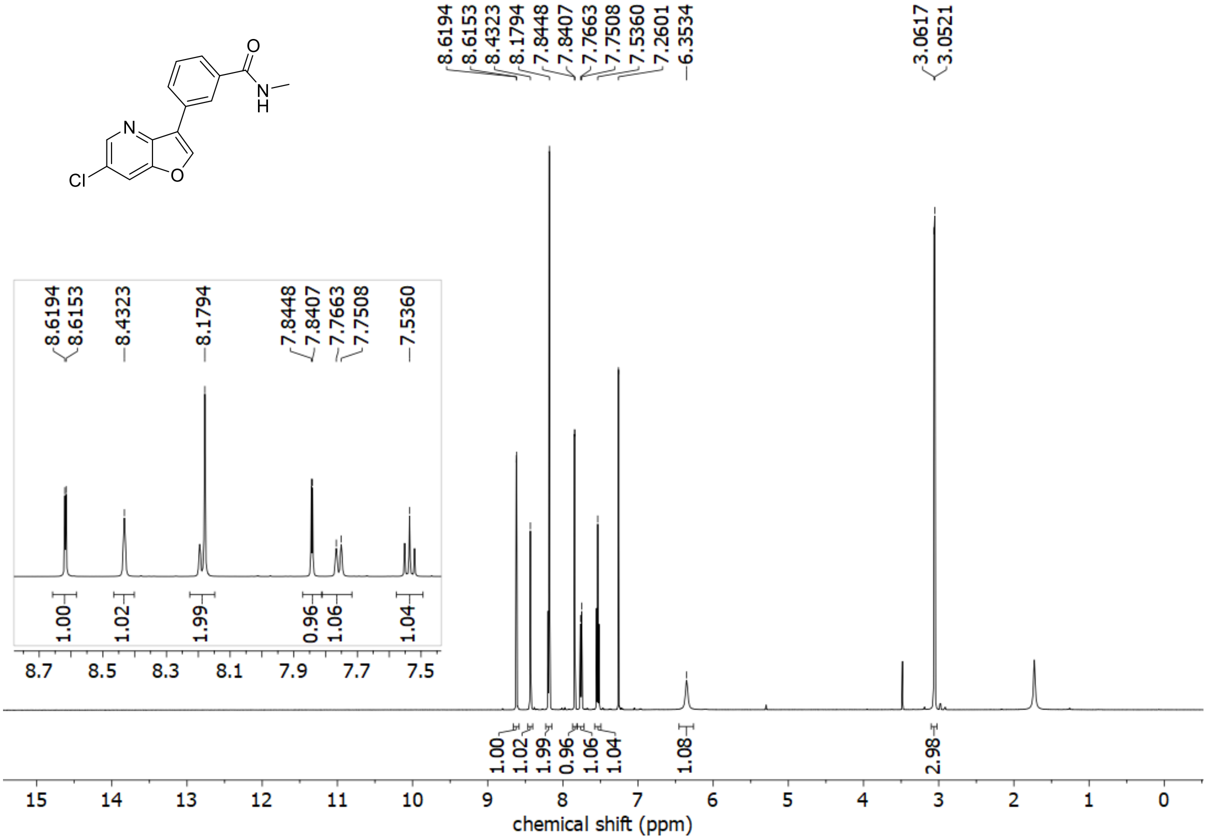

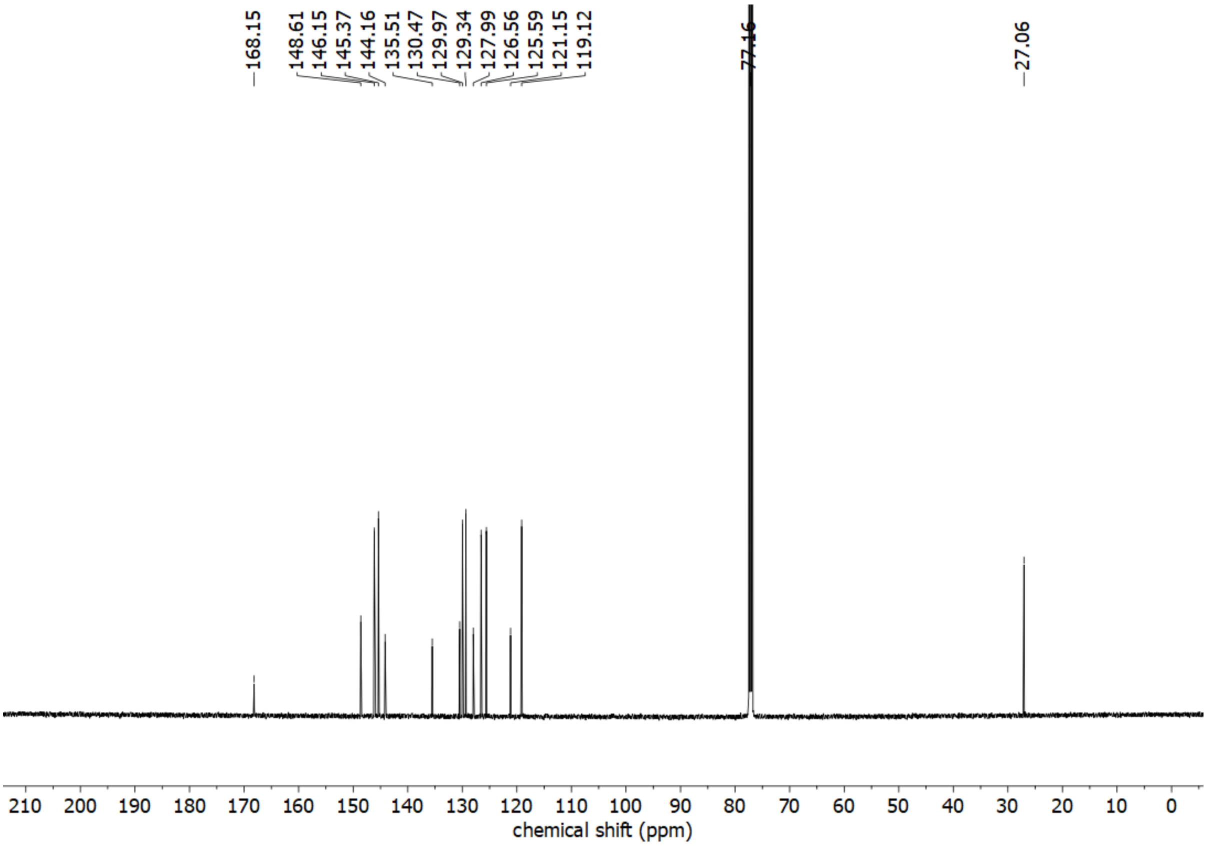

FT-IR spectrum (neat) of **67.**

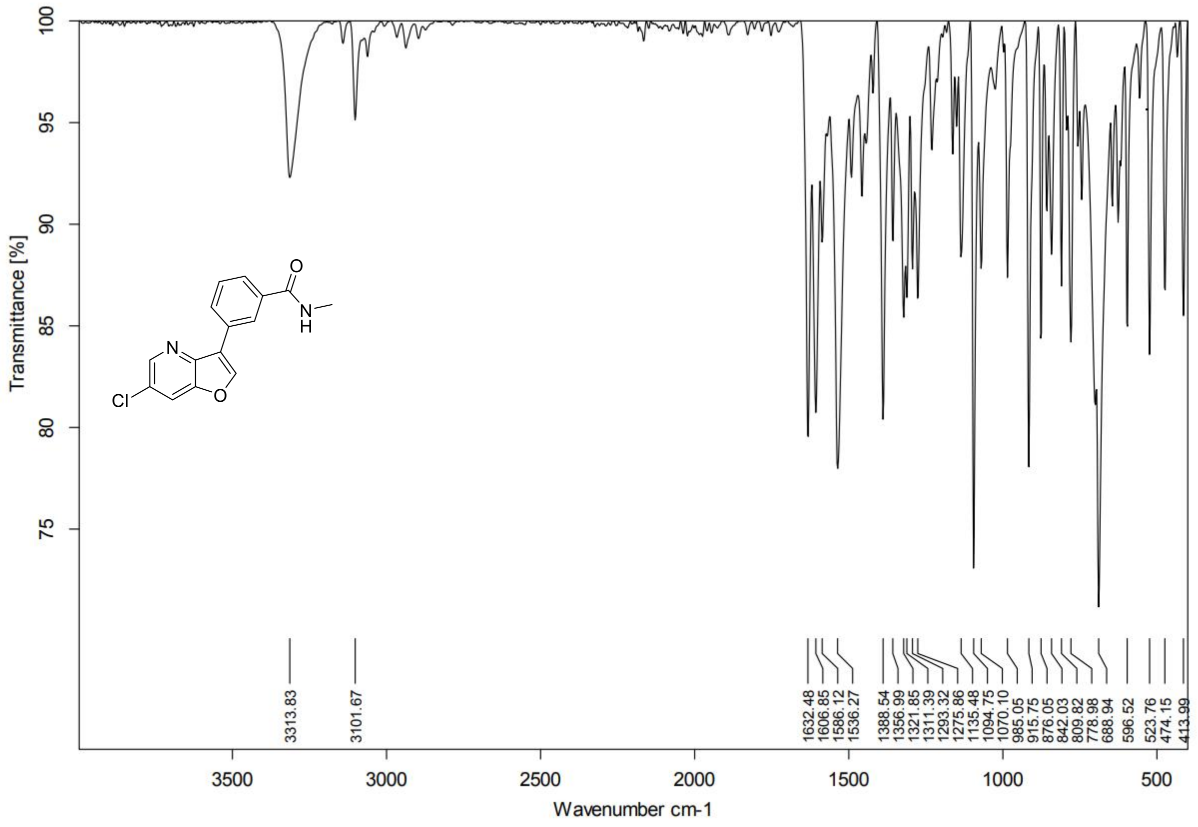

HRMS spectrum of **67.**

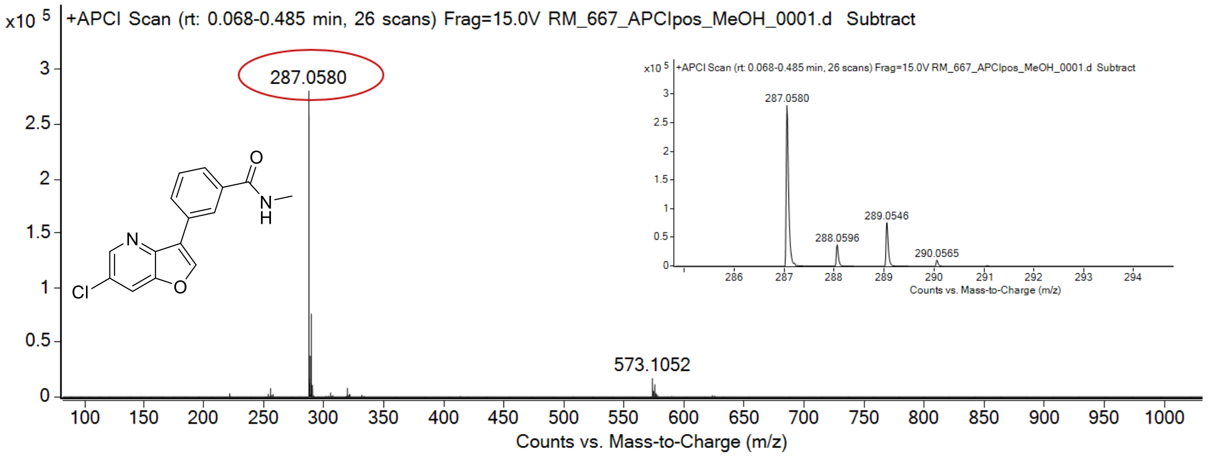

^{1}H (500 MHz) and ^{13}C NMR (126 MHz) spectra of **68** in chloroform-*d*.

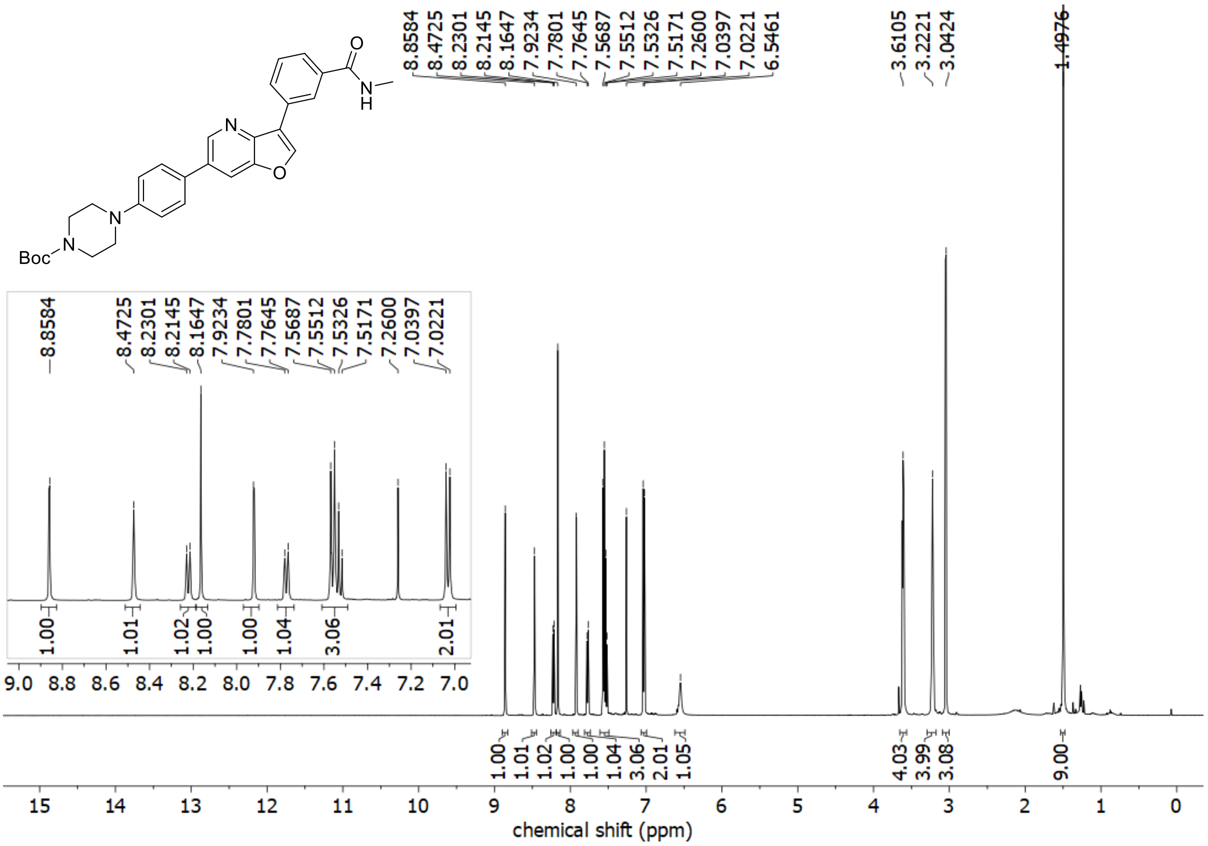

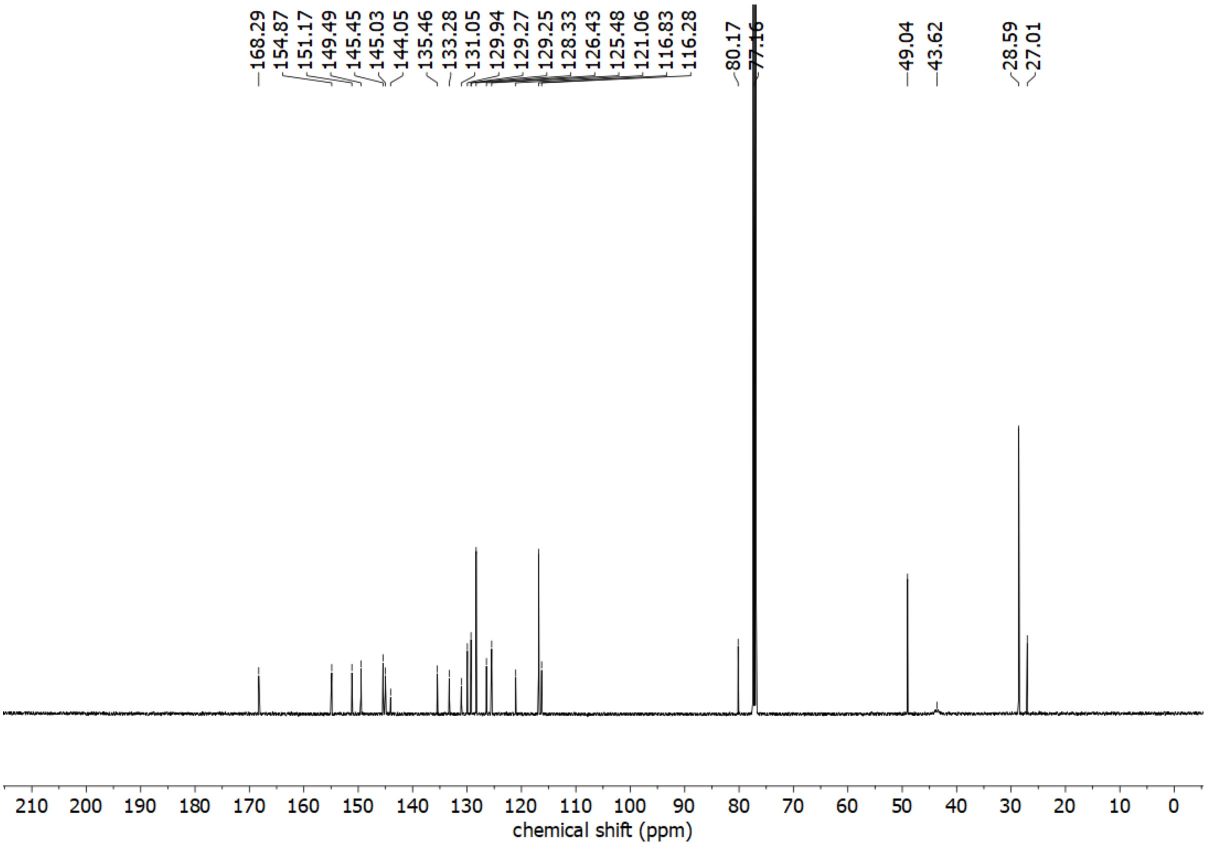

FT-IR spectrum (neat) of **68.**

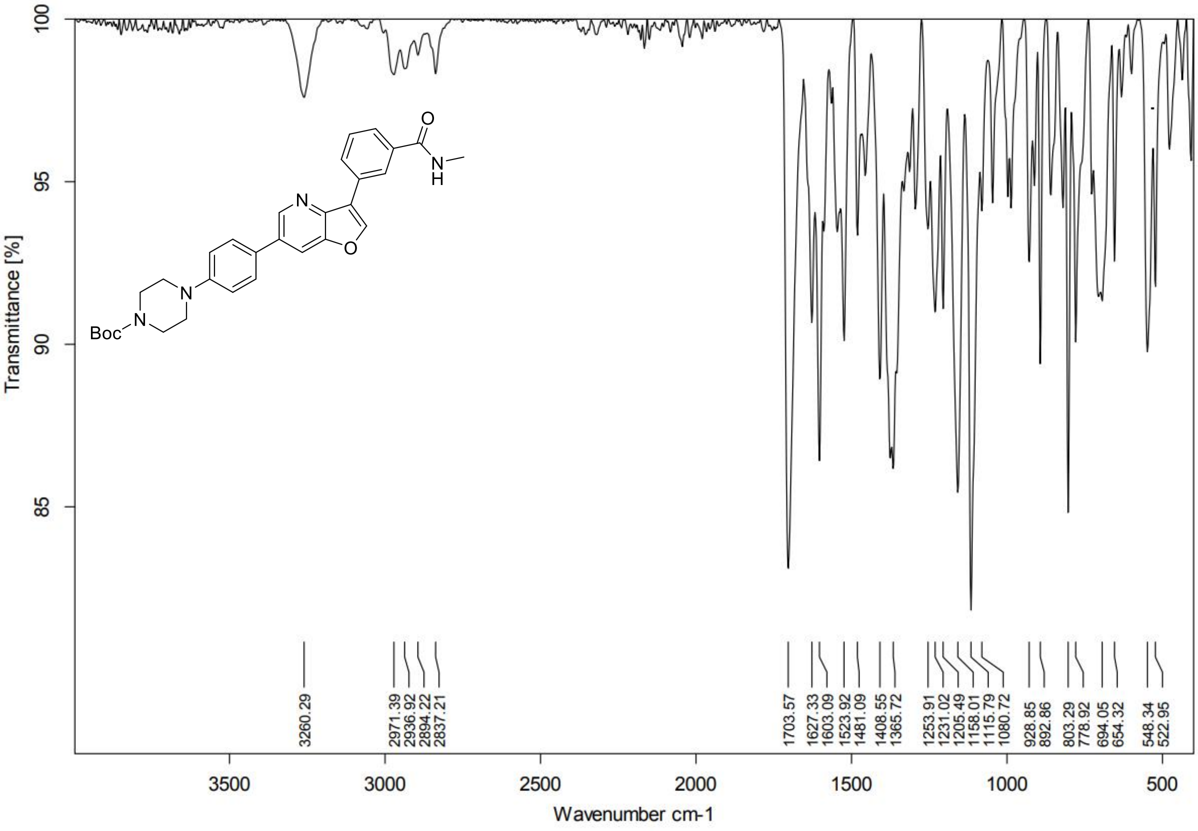

HRMS spectrum of **68.**

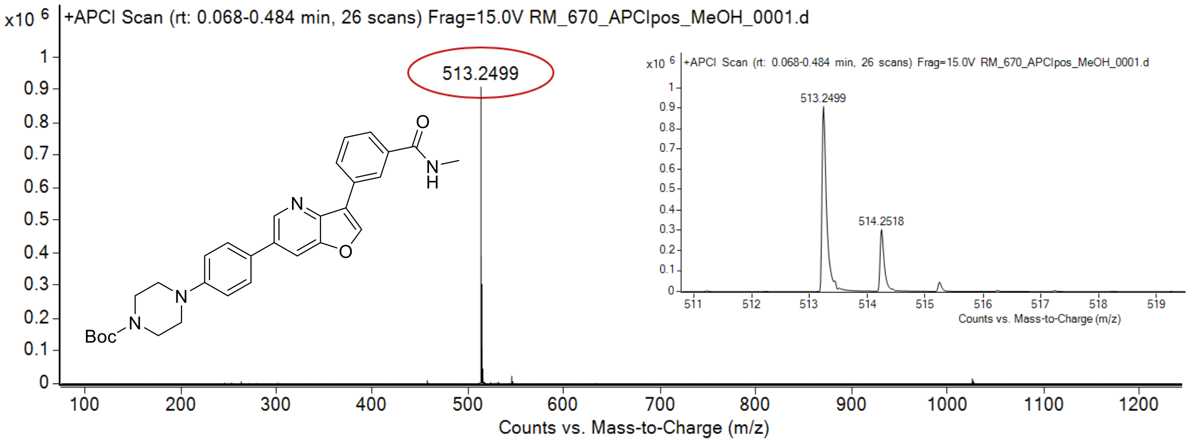

^{1}H (500 MHz) and ^{13}C NMR (126 MHz) spectra of **69** in DMSO-d_6.

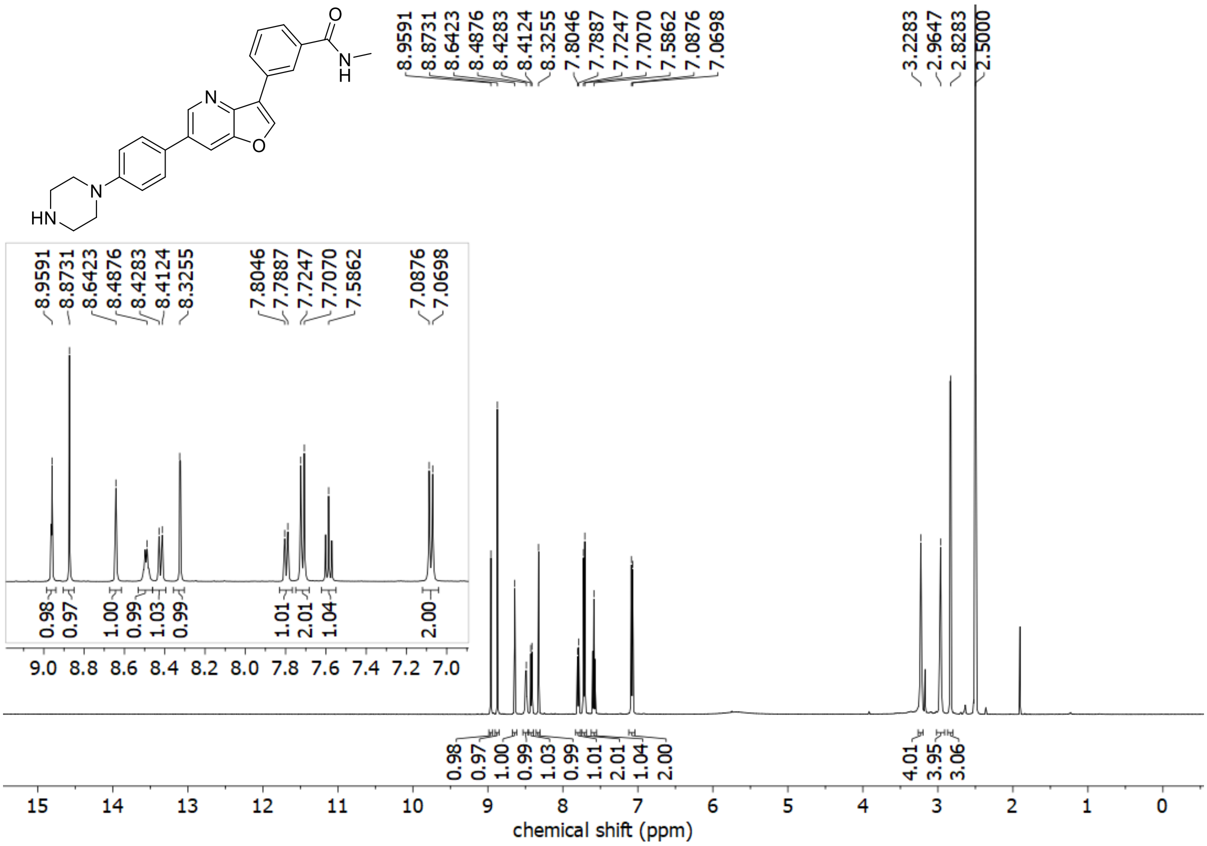

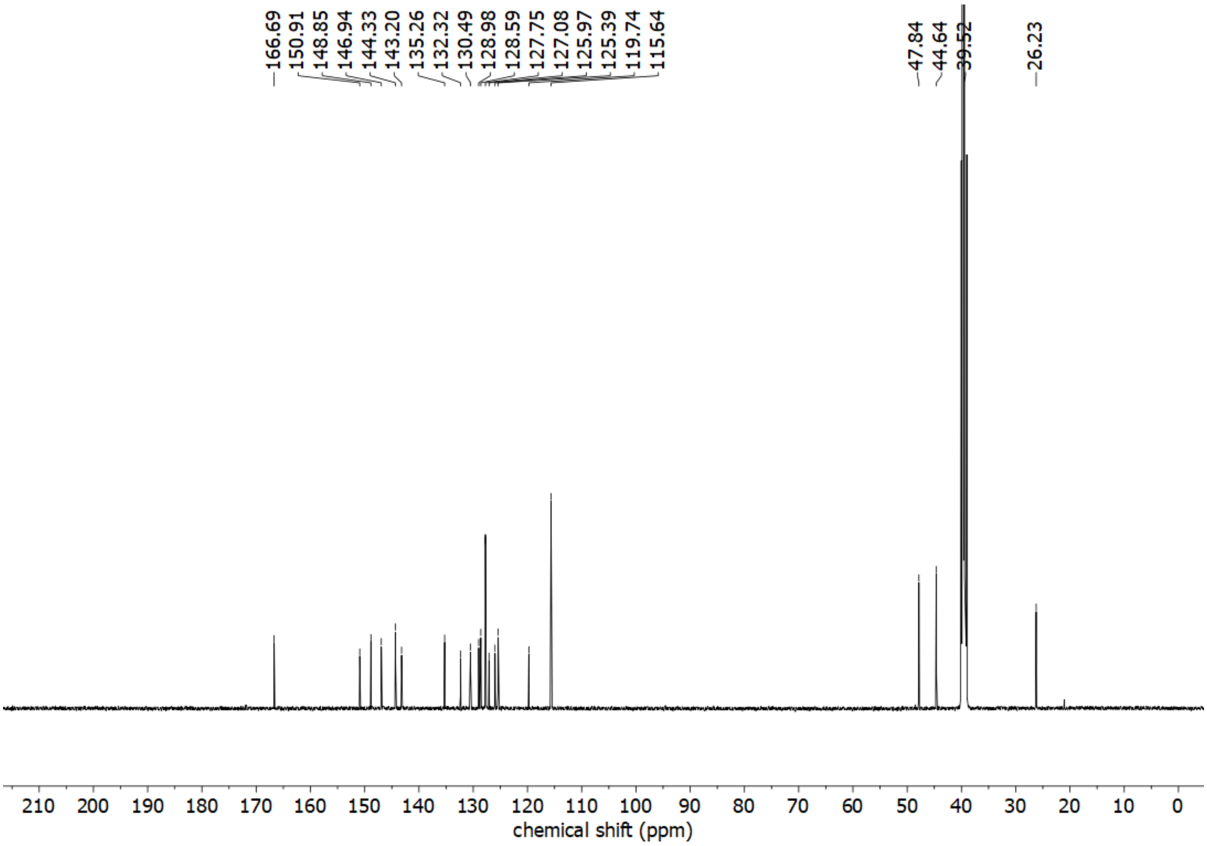

FT-IR spectrum (neat) of **69.**

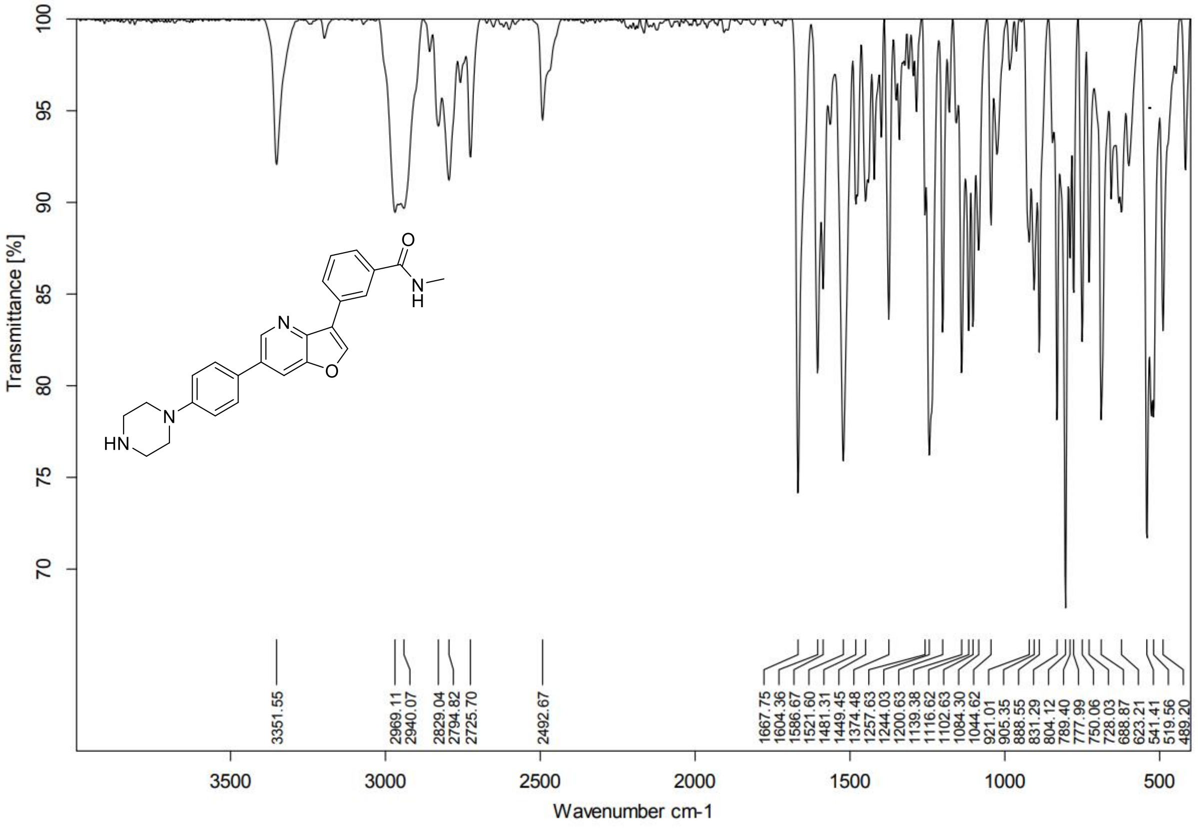

HRMS spectrum of **69.**

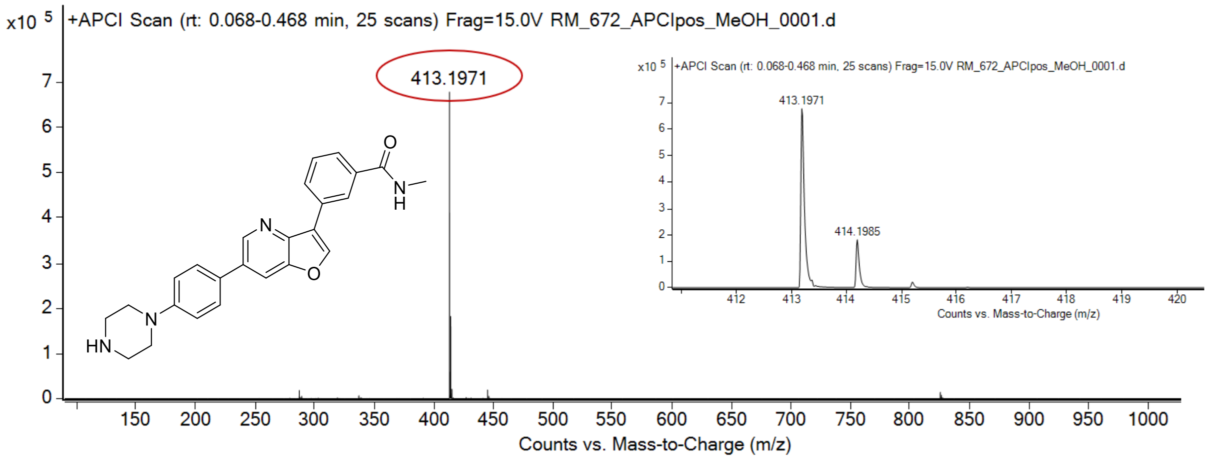

^{1}H (500 MHz) and ^{13}C NMR (126 MHz) spectra of **70** in chloroform-*d*.

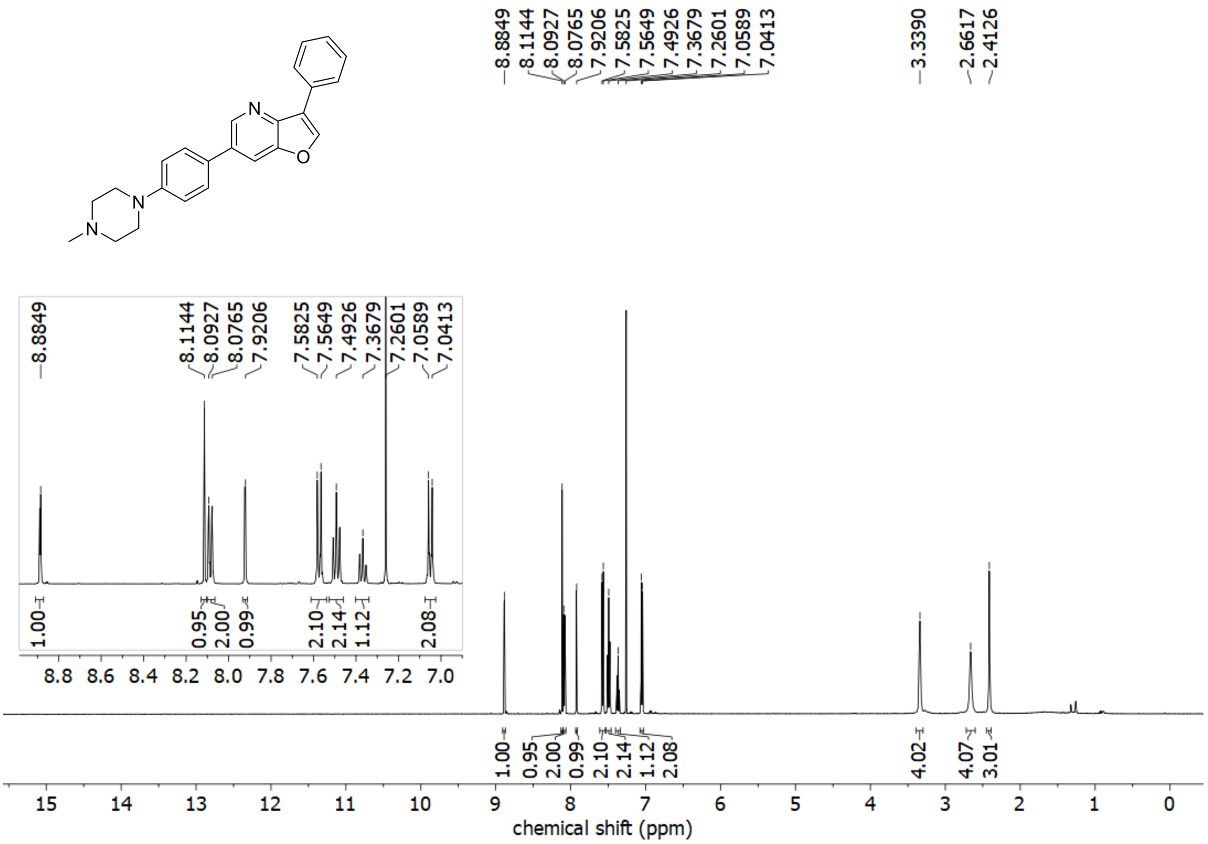

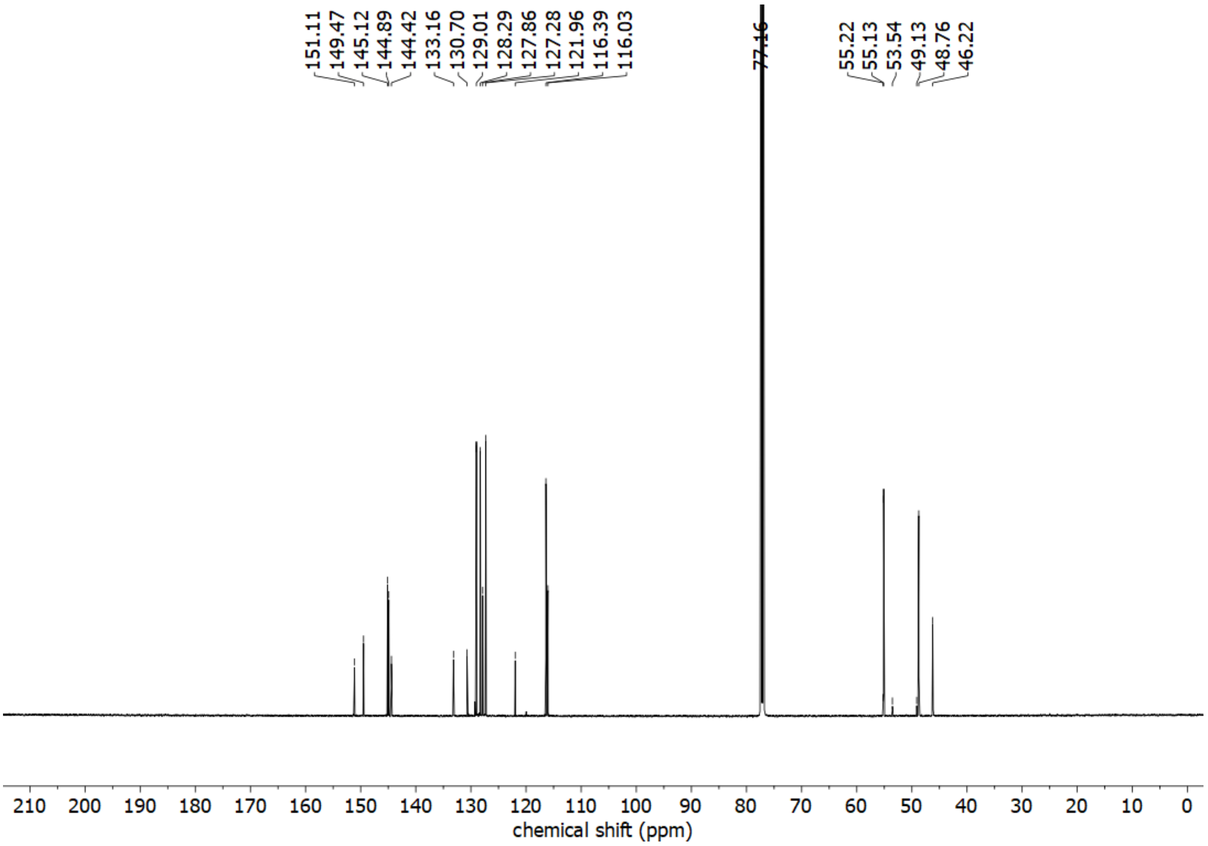

FT-IR spectrum (neat) of **70.**

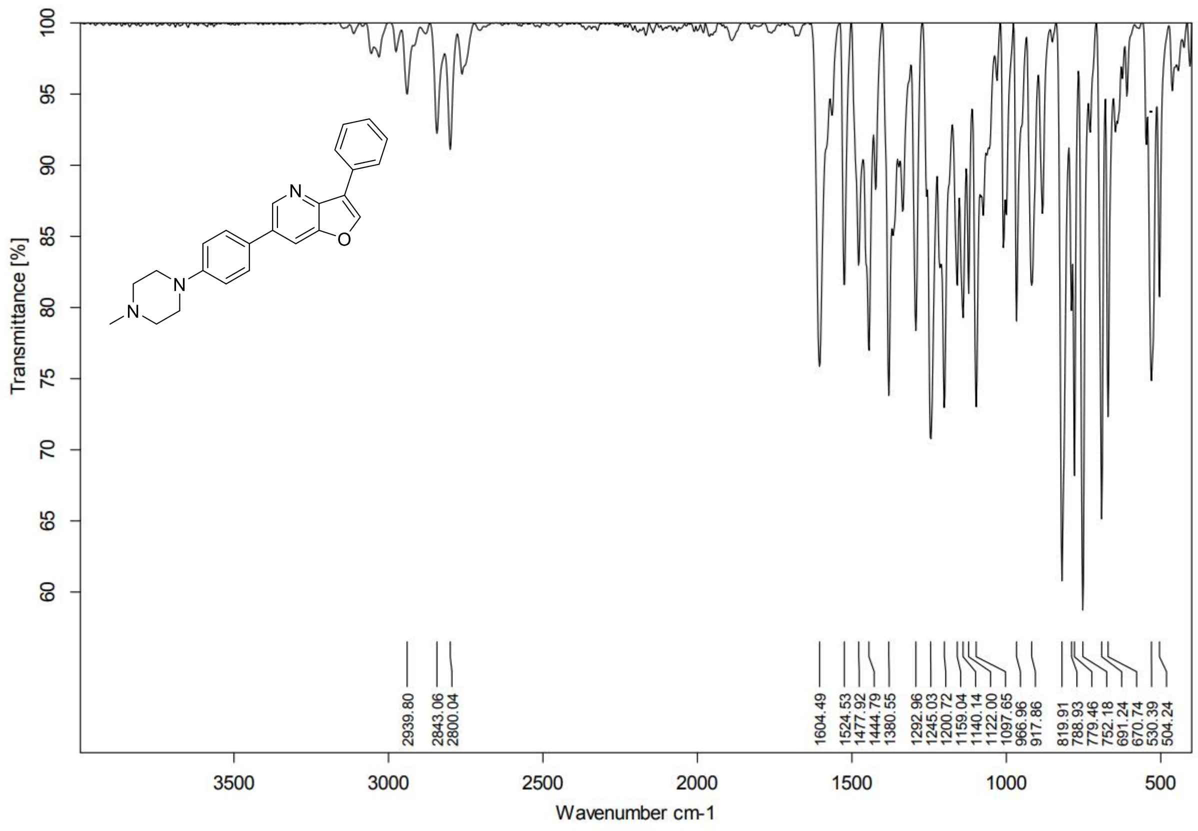

HRMS spectrum of **70.**

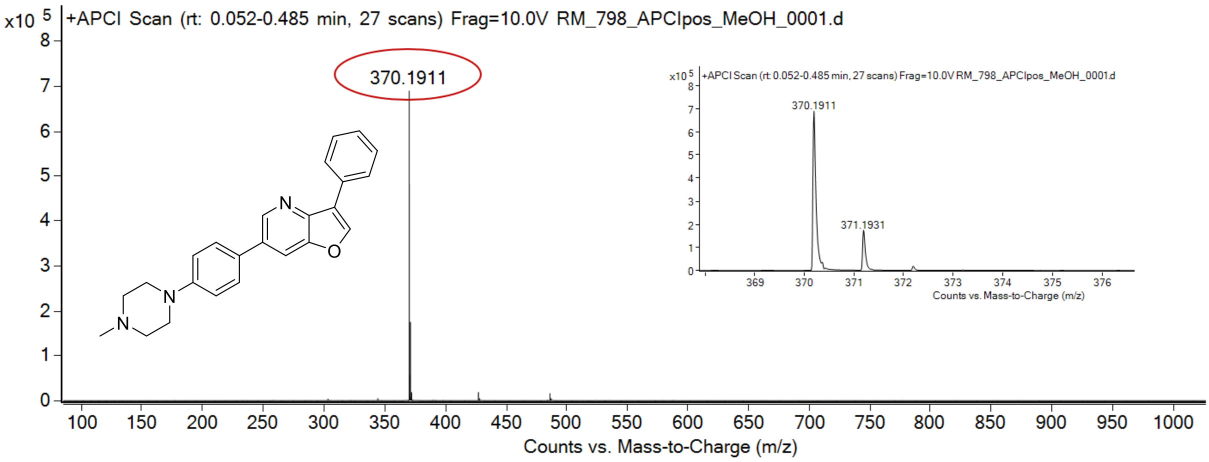

^{1}H (500 MHz) and ^{13}C NMR (126 MHz) spectra of **71** in chloroform-*d*.

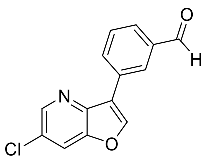

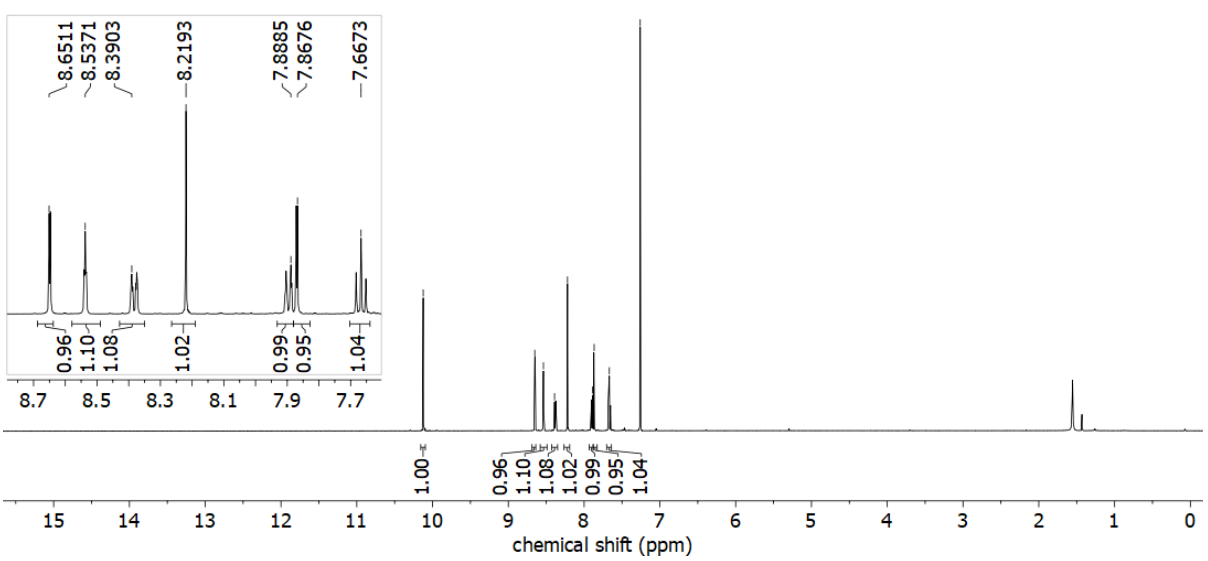

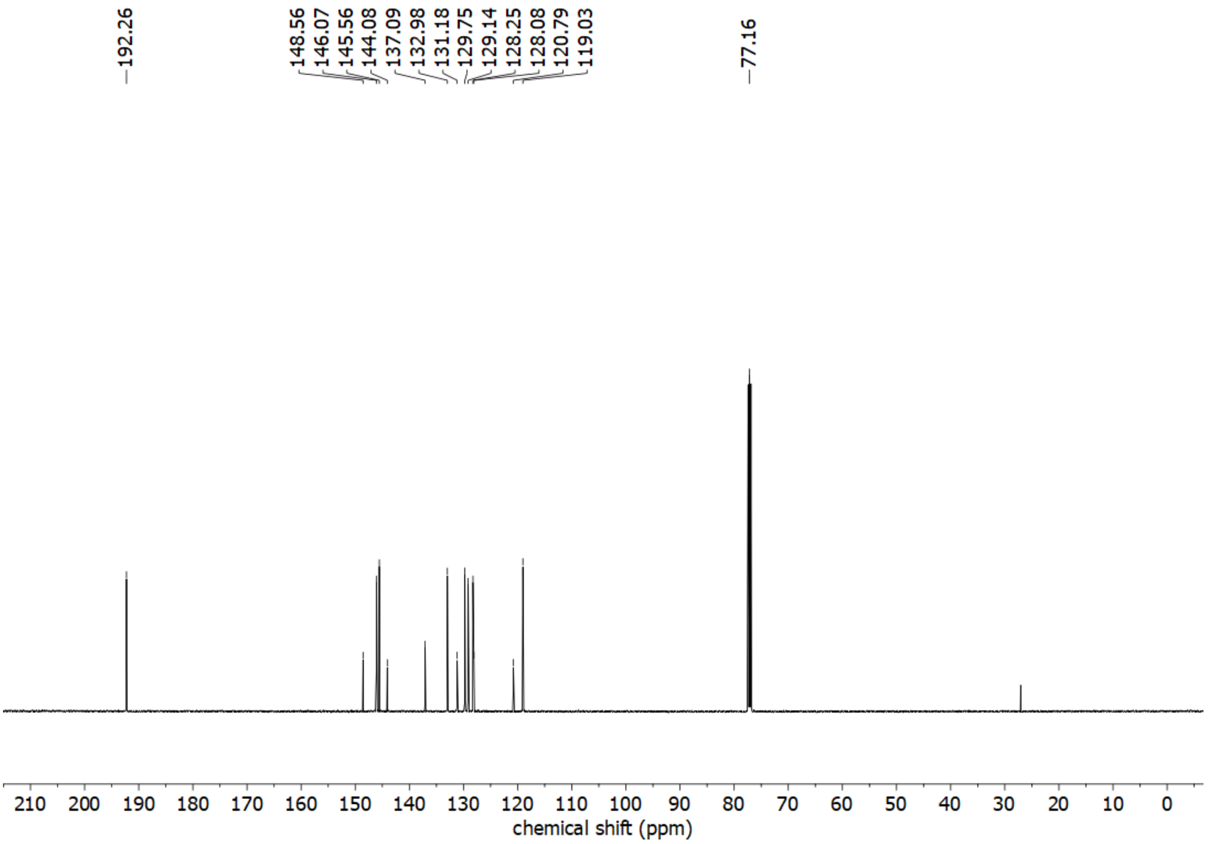

FT-IR spectrum (neat) of **71.**

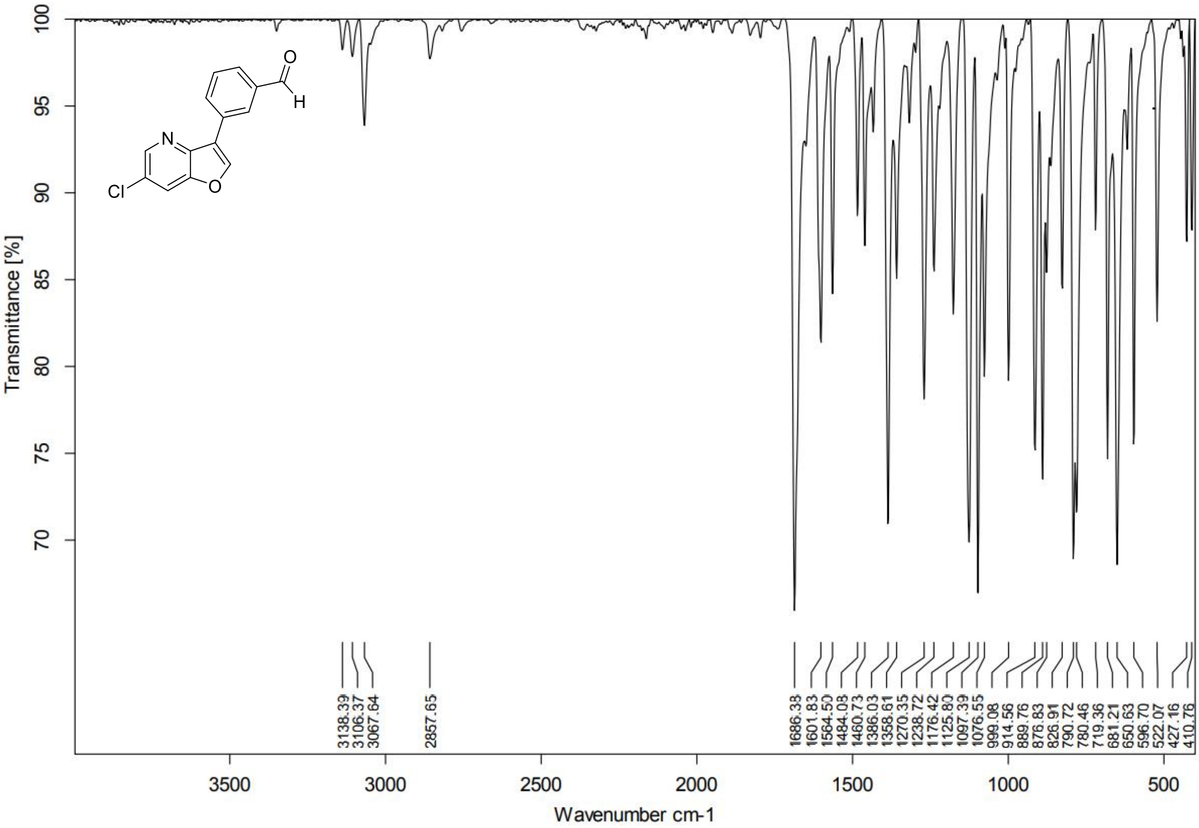

HRMS spectrum of **71.**

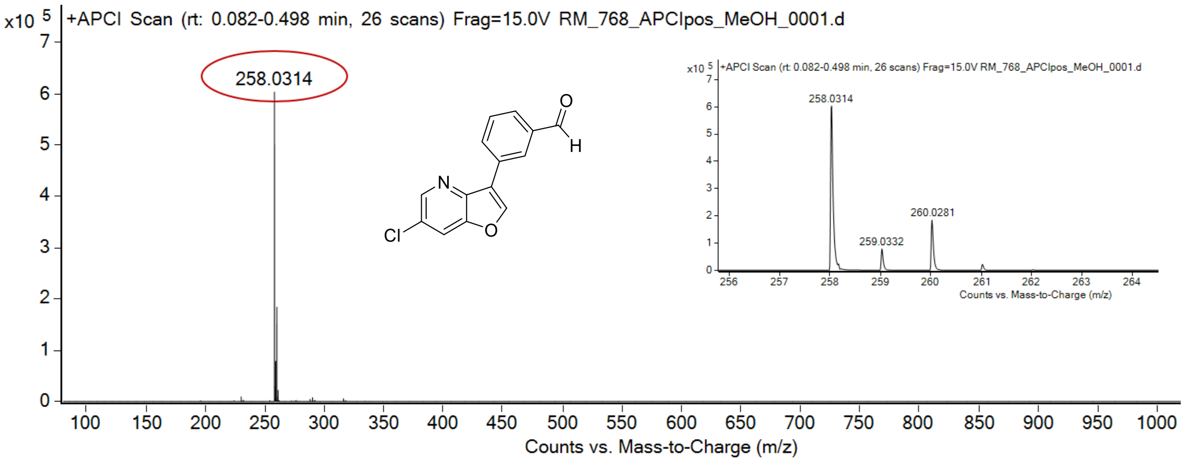

^{1}H (500 MHz) and ^{13}C NMR (126 MHz) spectra of **72** in DMSO-d_6.

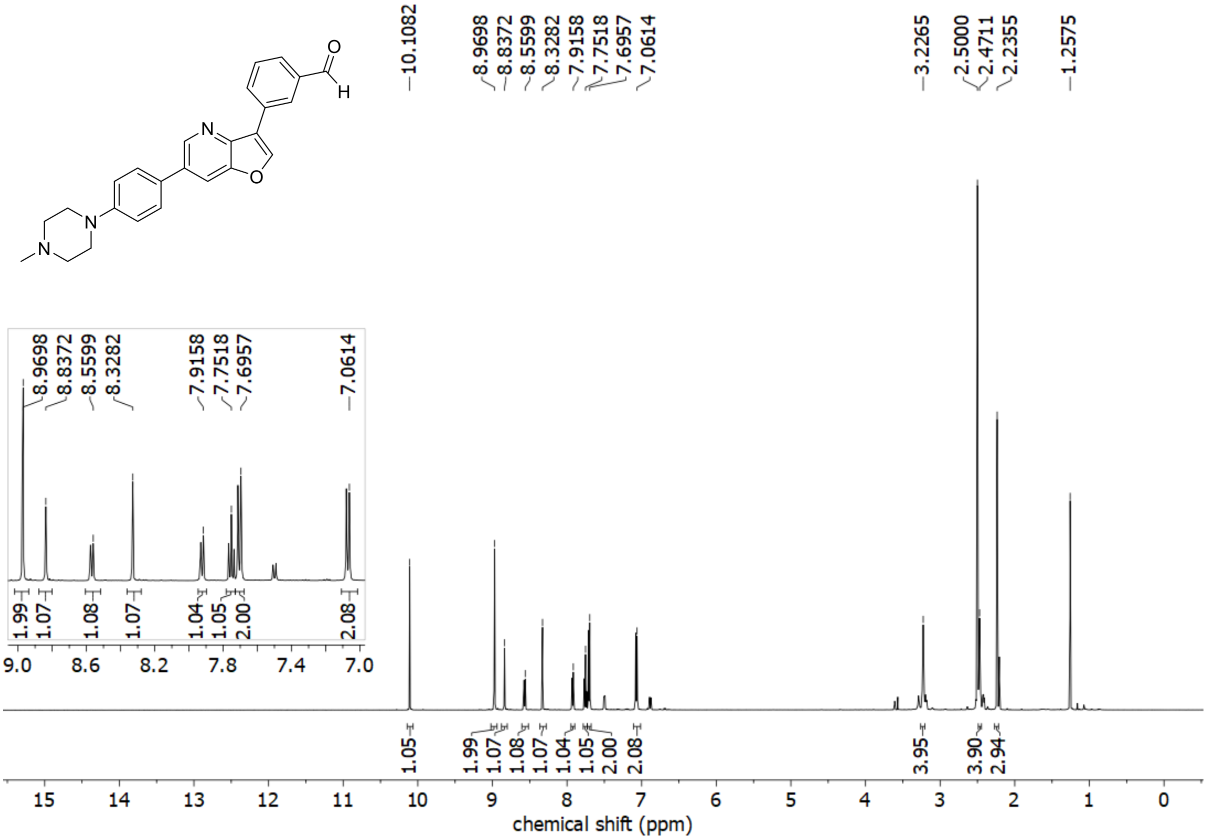

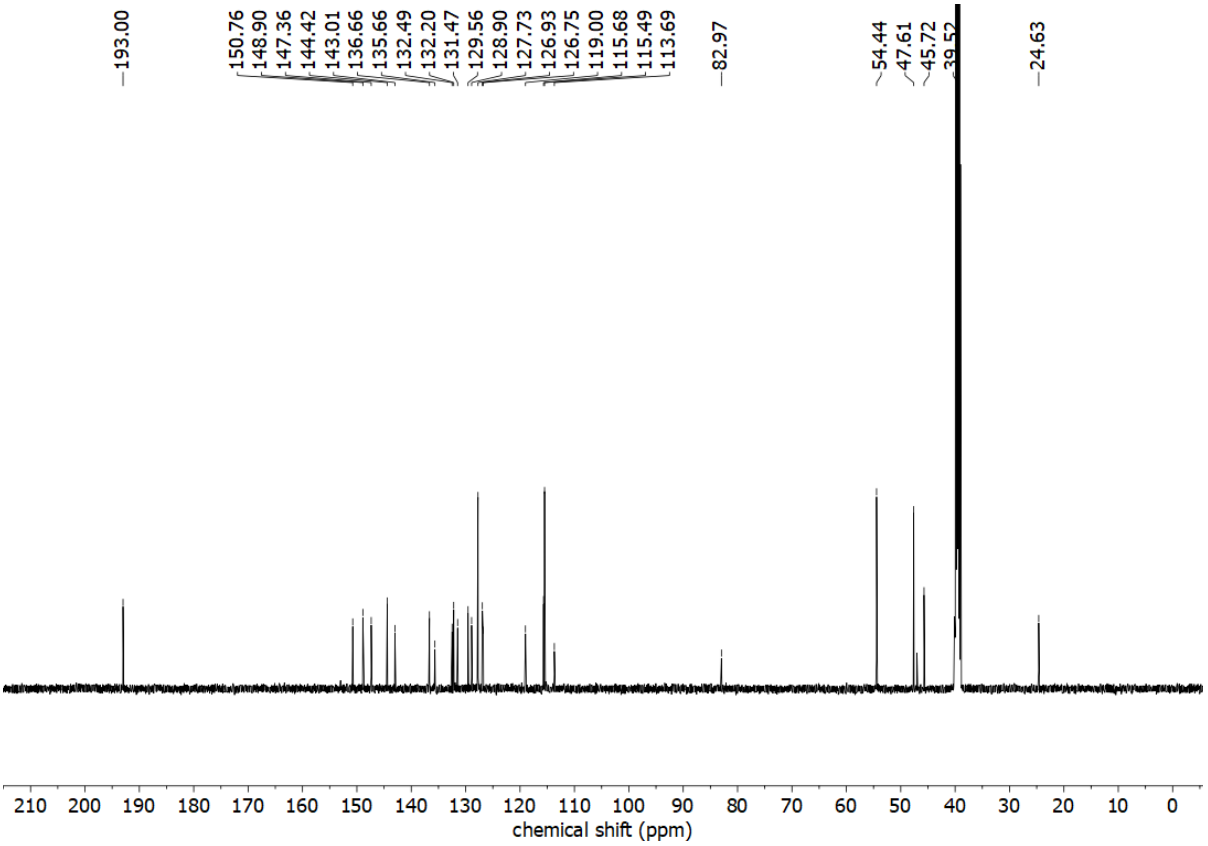

FT-IR spectrum (neat) of **72.**

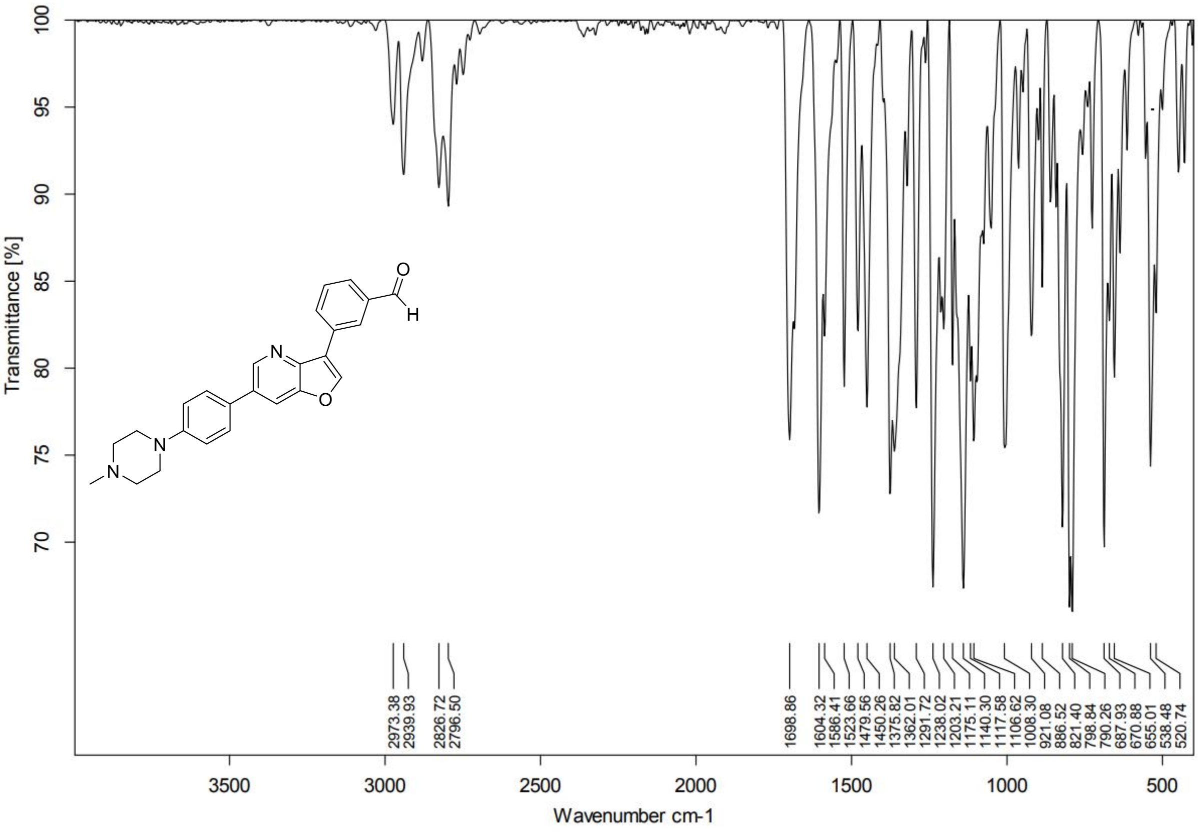

HRMS spectrum of **72.**

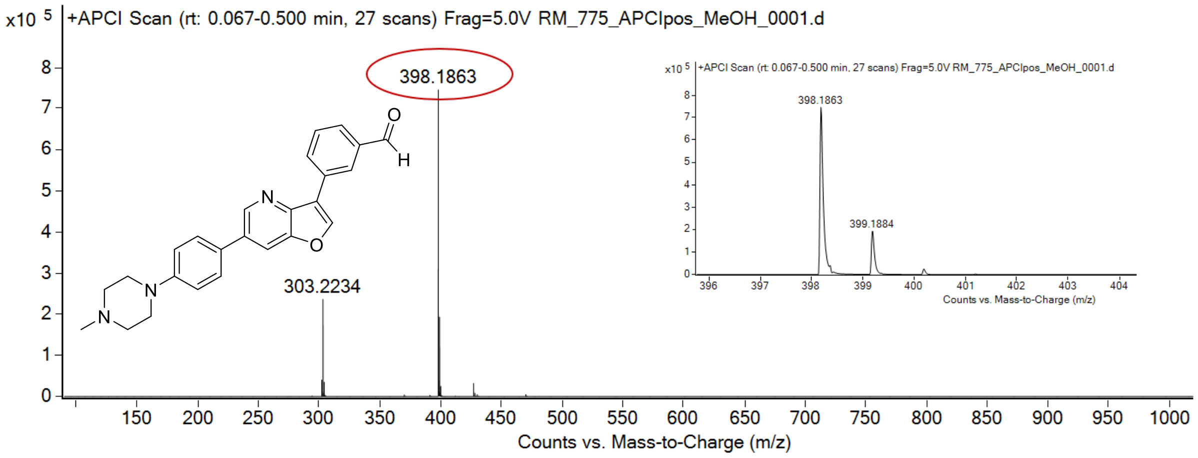

^{1}H (500 MHz) and ^{13}C NMR (126 MHz) spectra of **73** in DMSO-d_6.

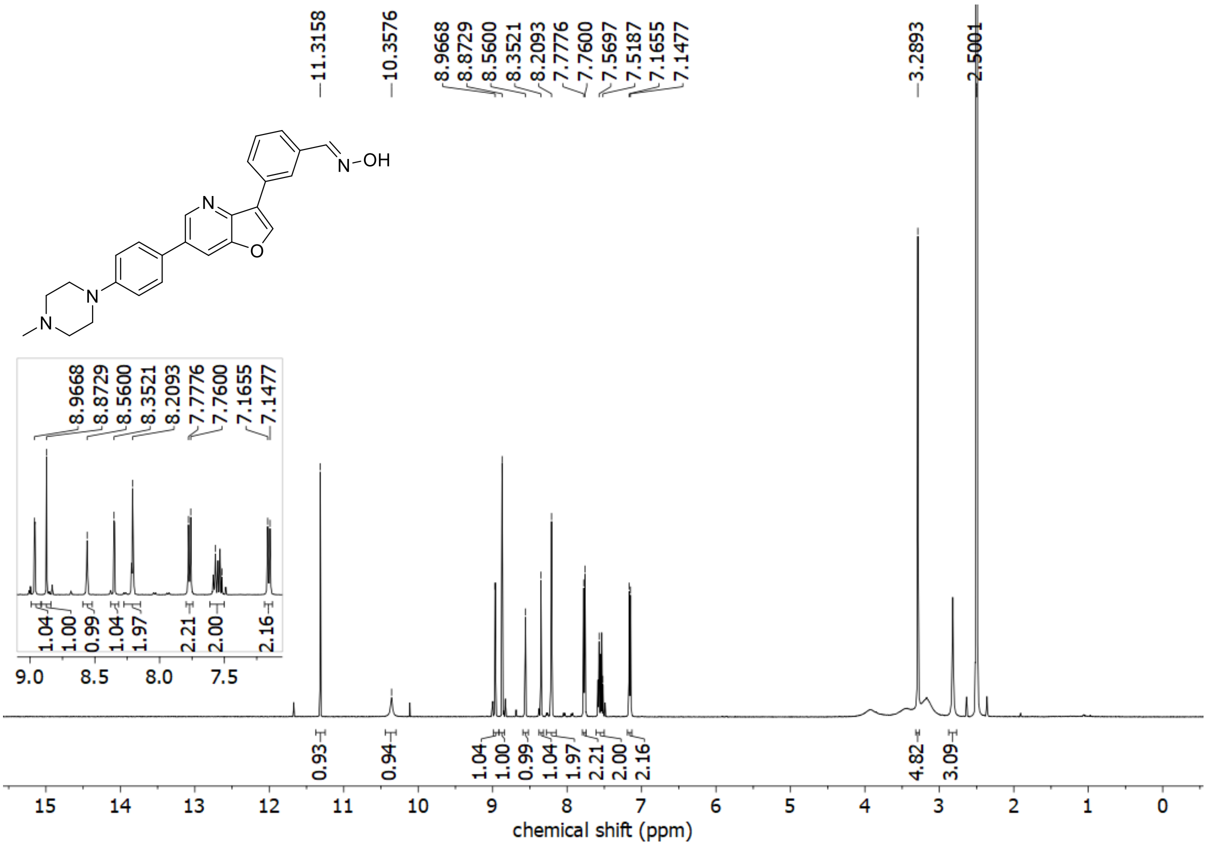

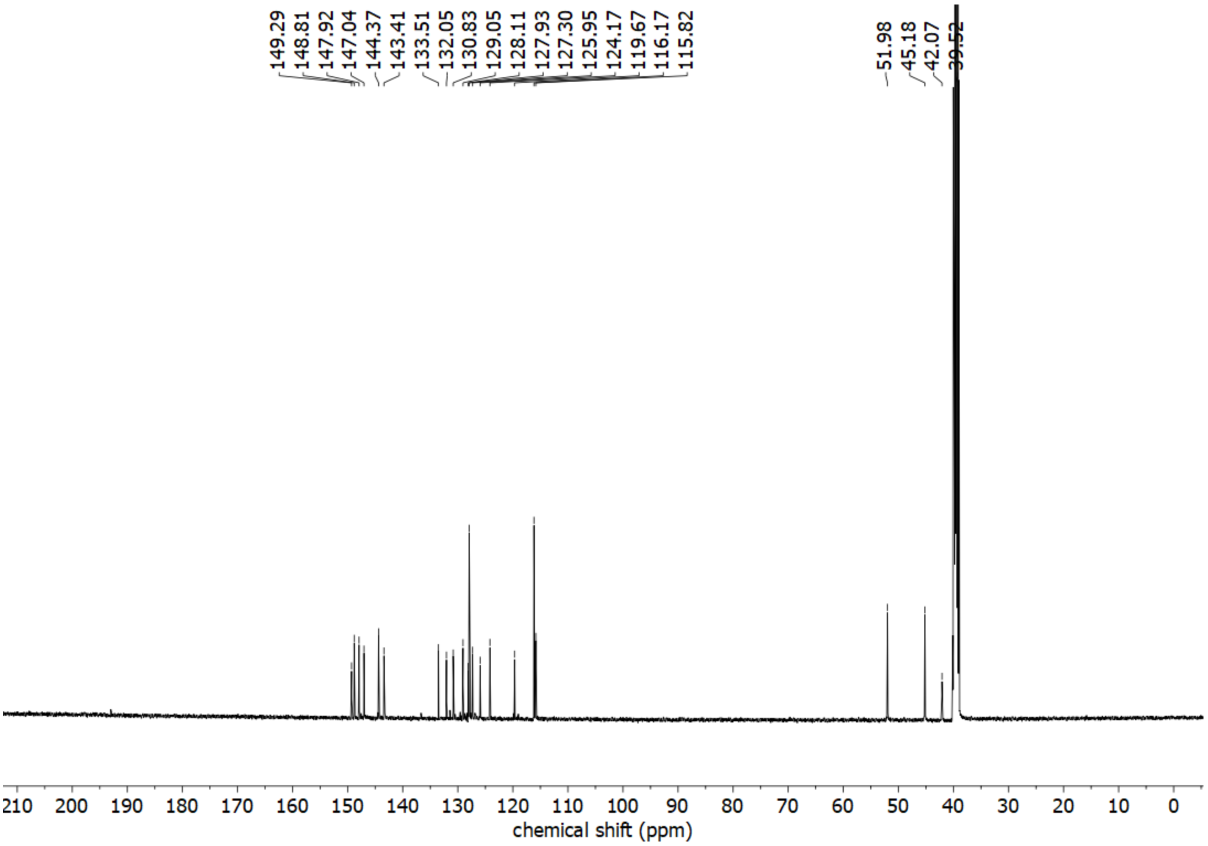

FT-IR spectrum (neat) of **73.**

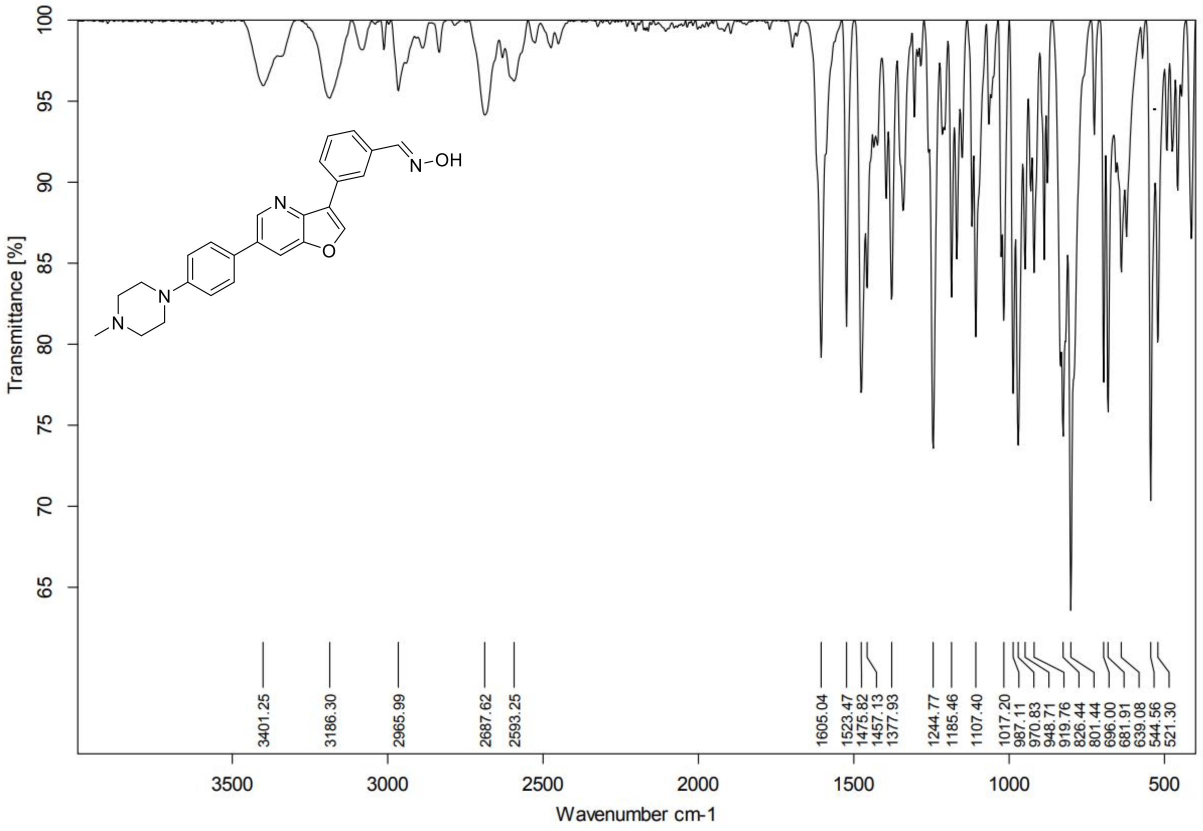

HRMS spectrum of **73.**

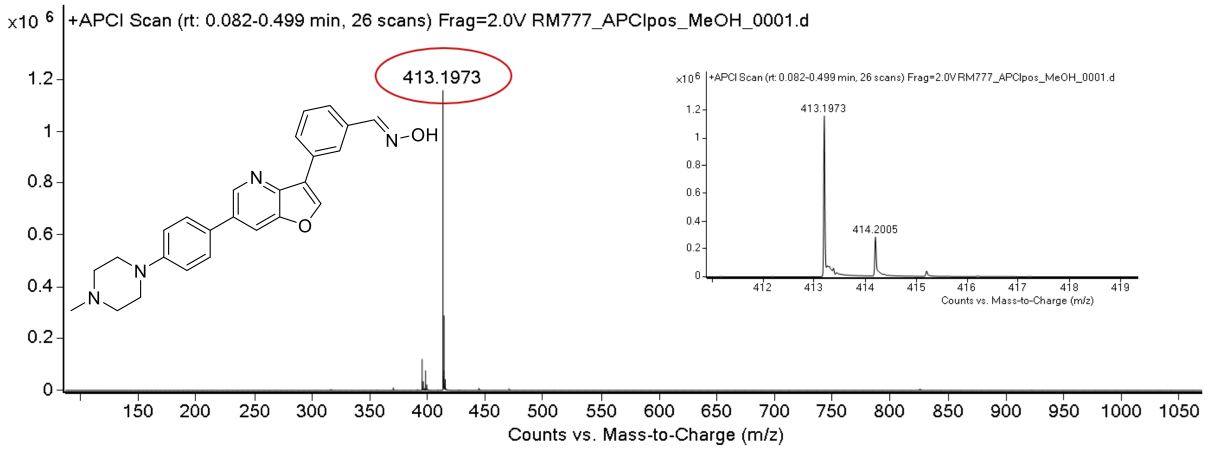

^{1}H (500 MHz) and ^{13}C NMR (126 MHz) spectra of **74** in chloroform-*d*.

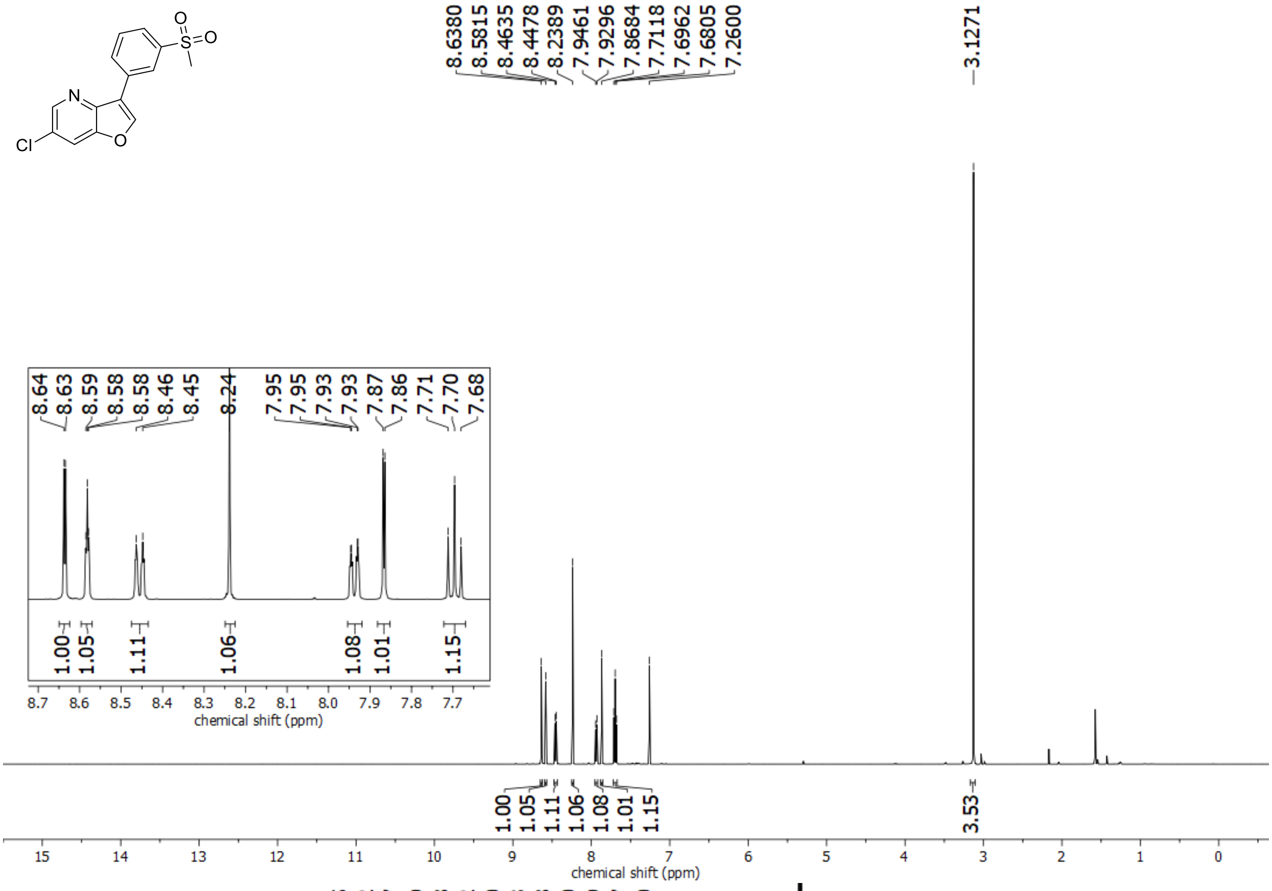

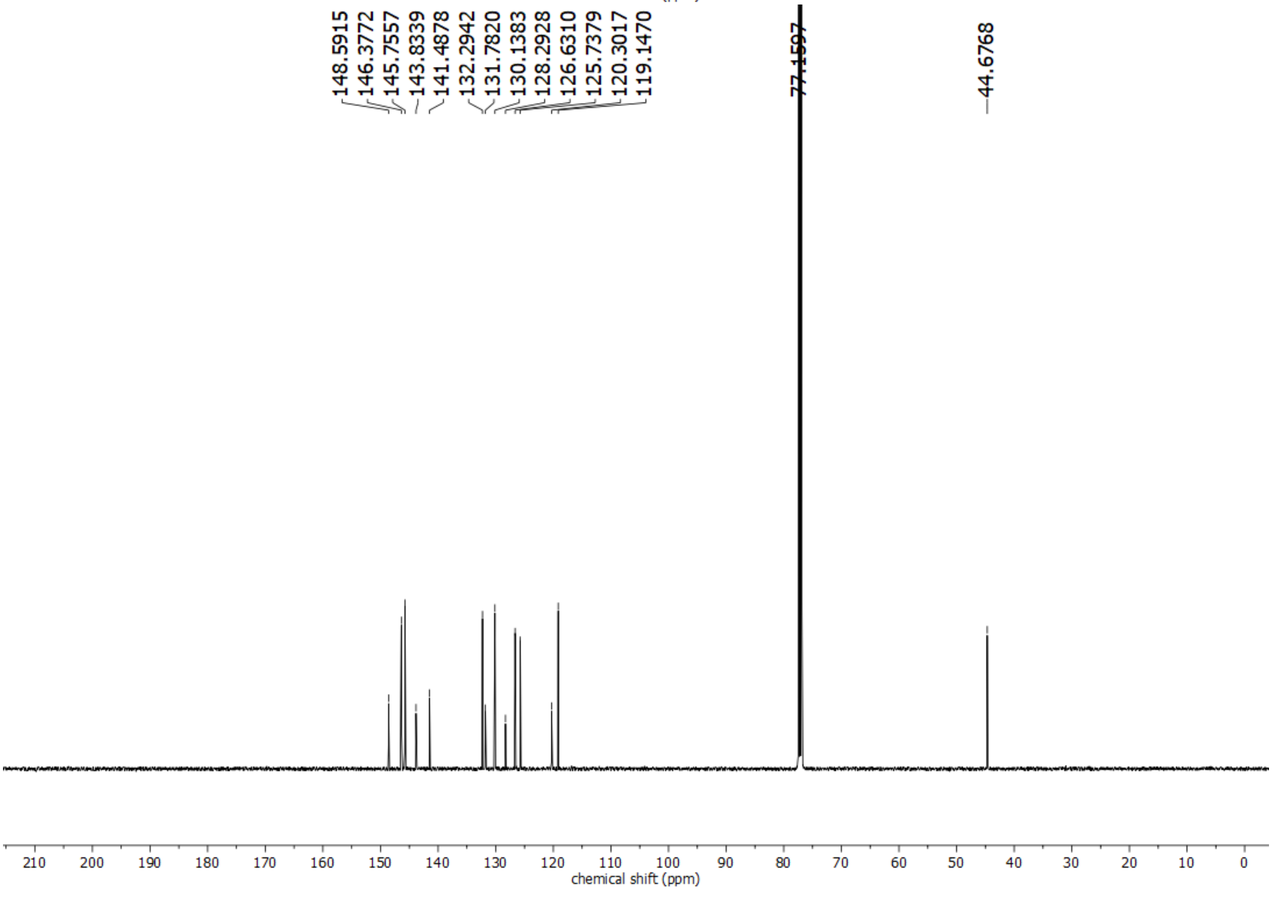

FT-IR spectrum (neat) of **74.**

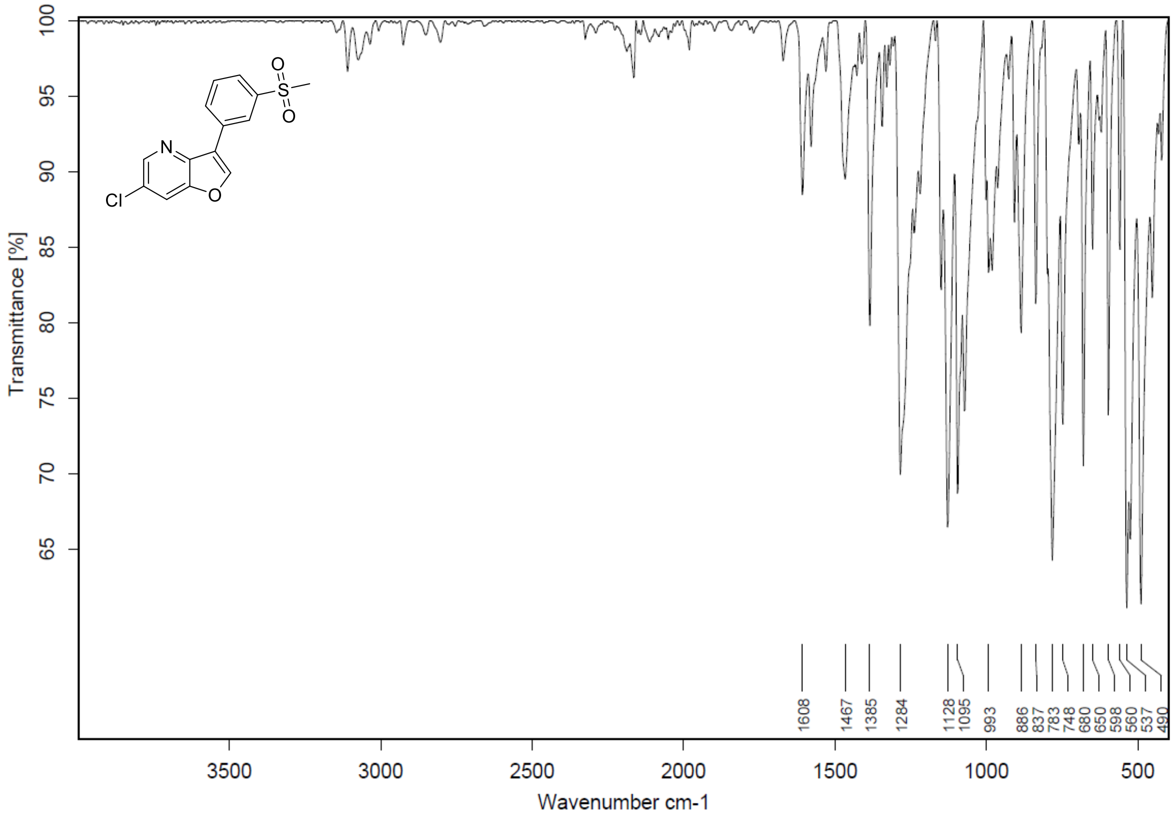

HRMS spectrum of **74.**

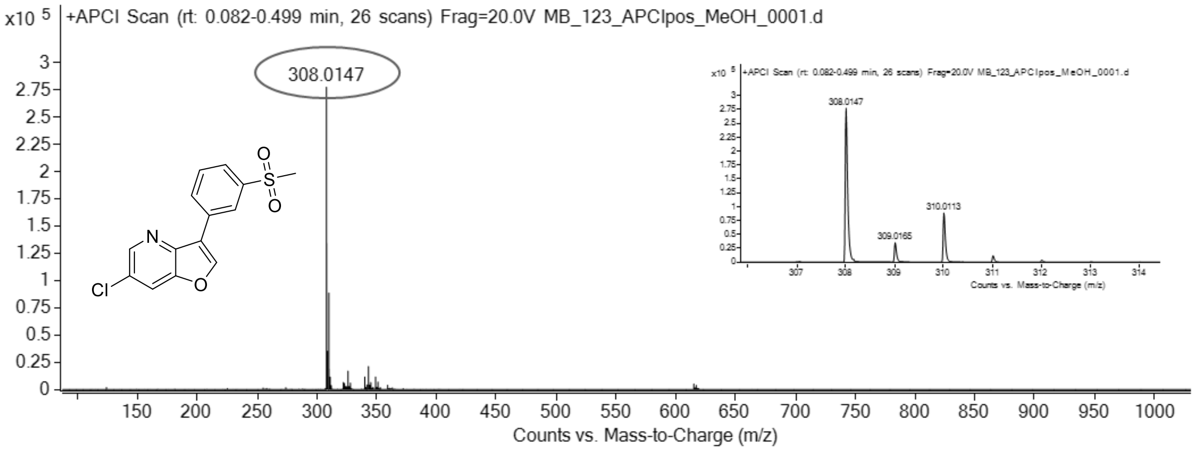

^{1}H (500 MHz) and ^{13}C NMR (126 MHz) spectra of **75** in chloroform-*d*.

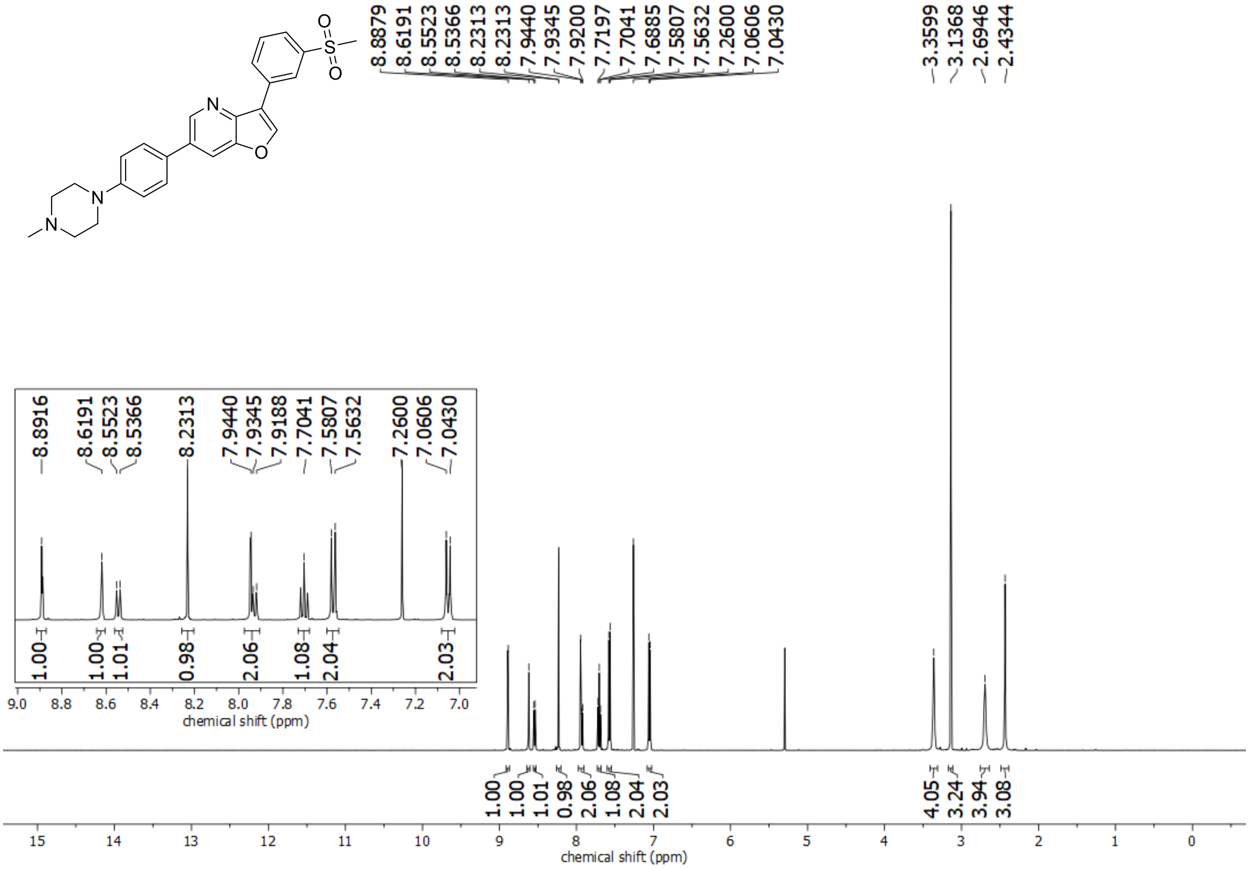

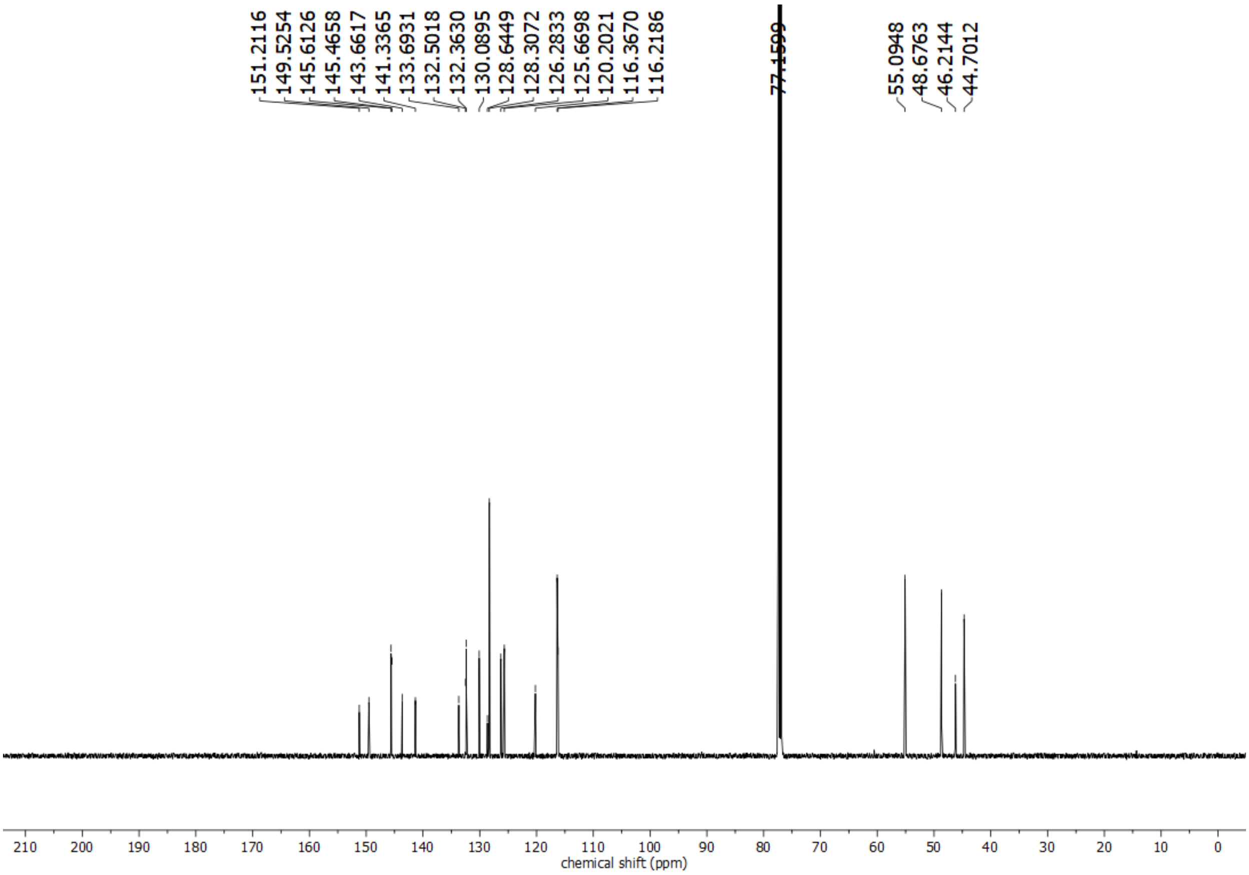

FT-IR spectrum (neat) of **75.**

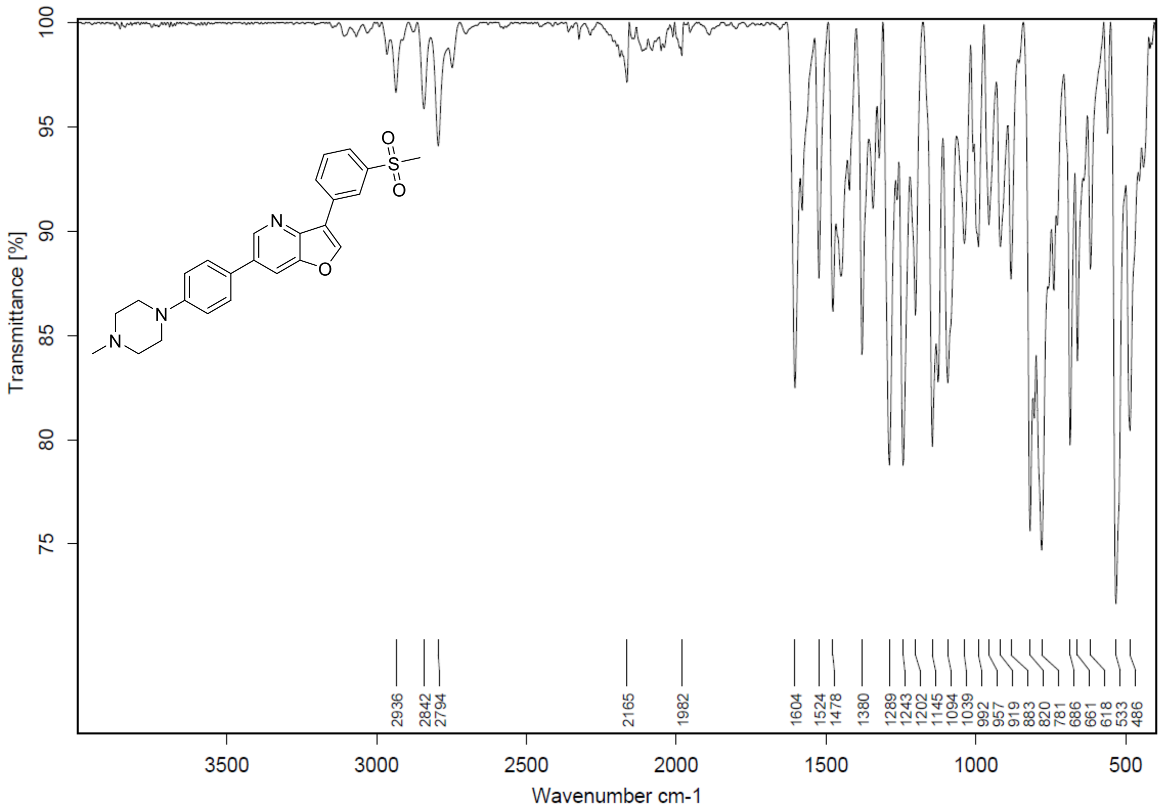

HRMS spectrum of **75.**

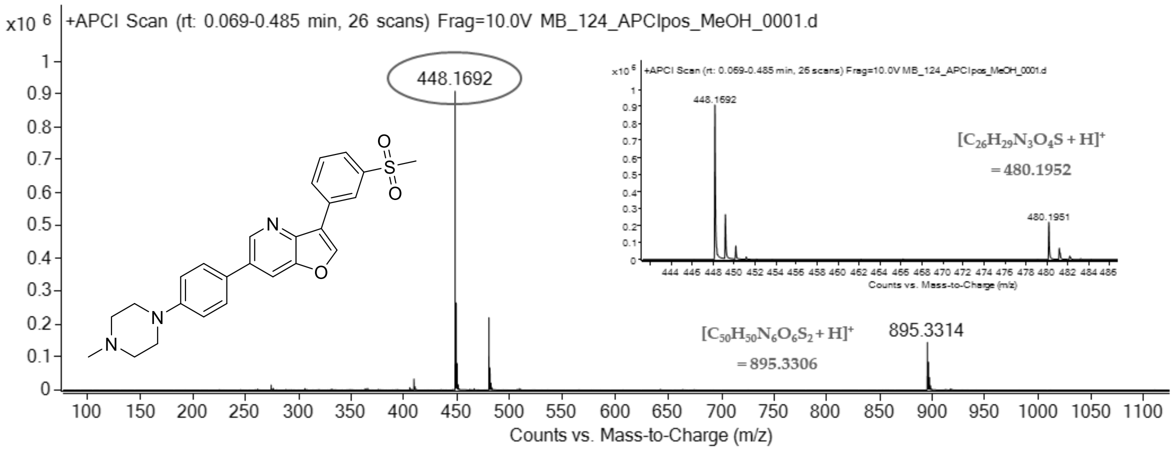

^{1}H (500 MHz) and ^{13}C NMR (126 MHz) spectra of **76** in DMSO-d_6.

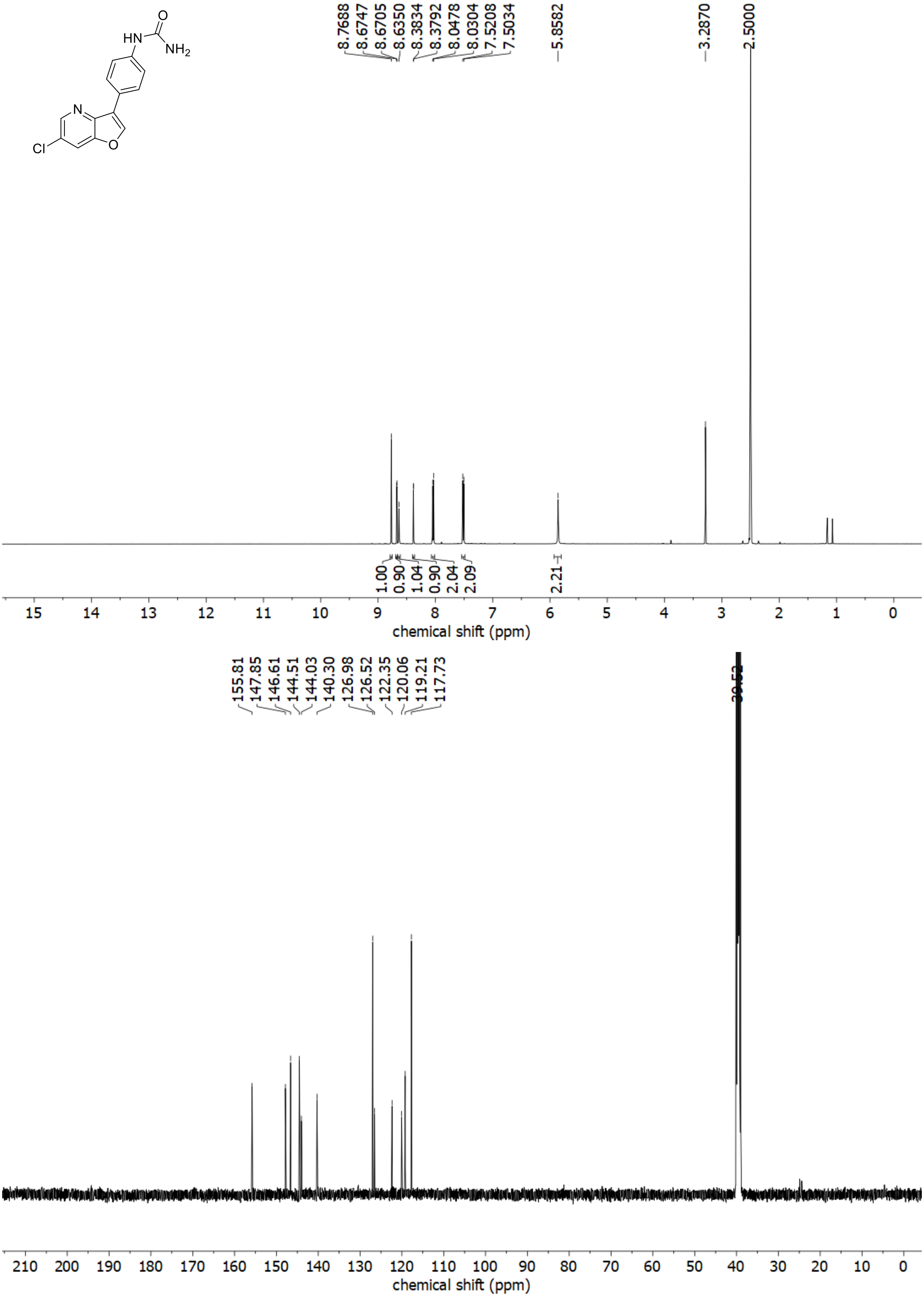

FT-IR spectrum (neat) of **76.**

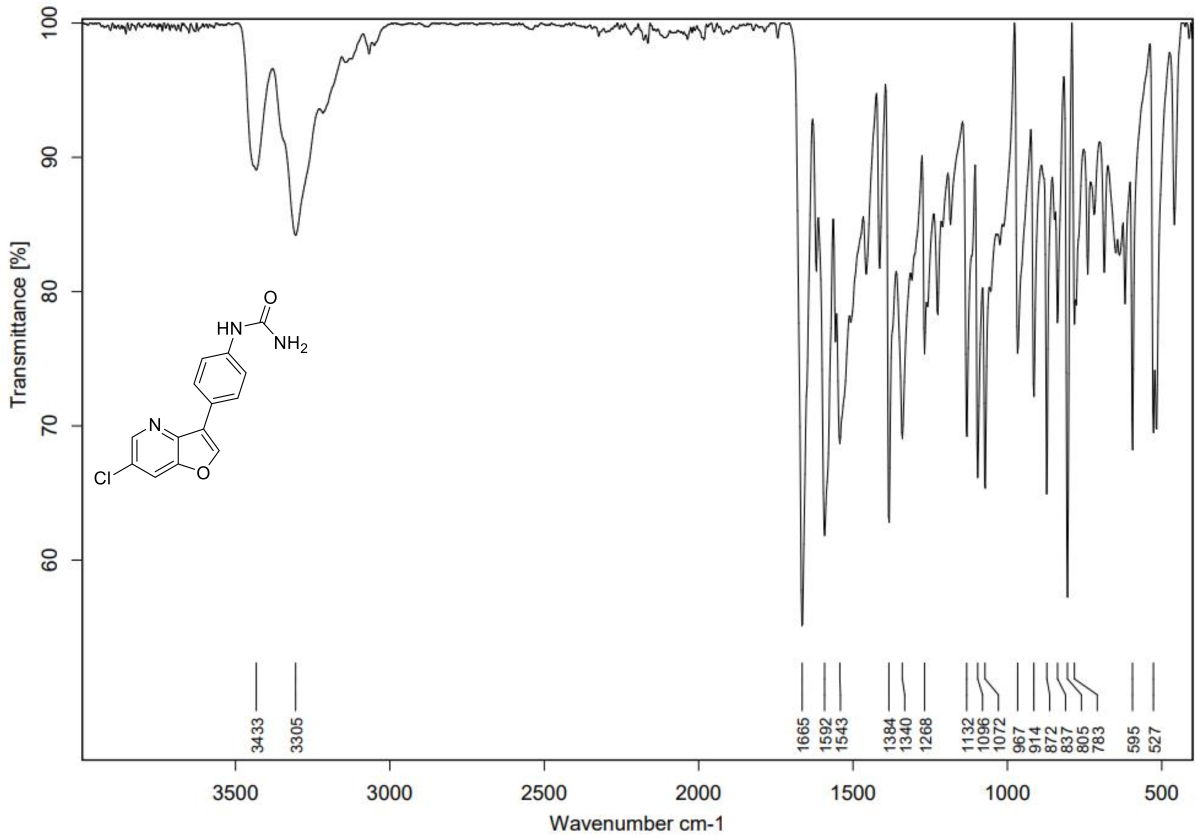

HRMS spectrum of **76.**

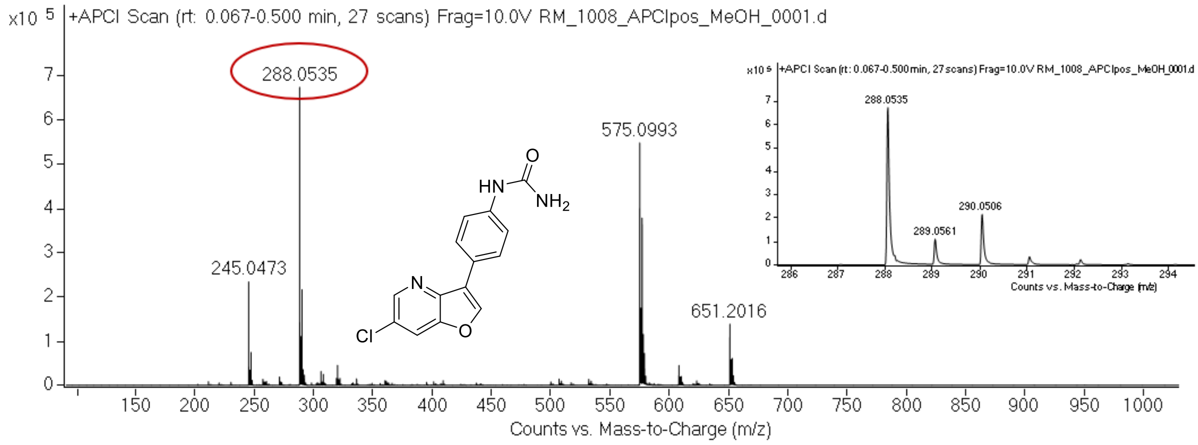

^{1}H (500 MHz) and ^{13}C NMR (126 MHz) spectra of **77** in DMSO-d_6.

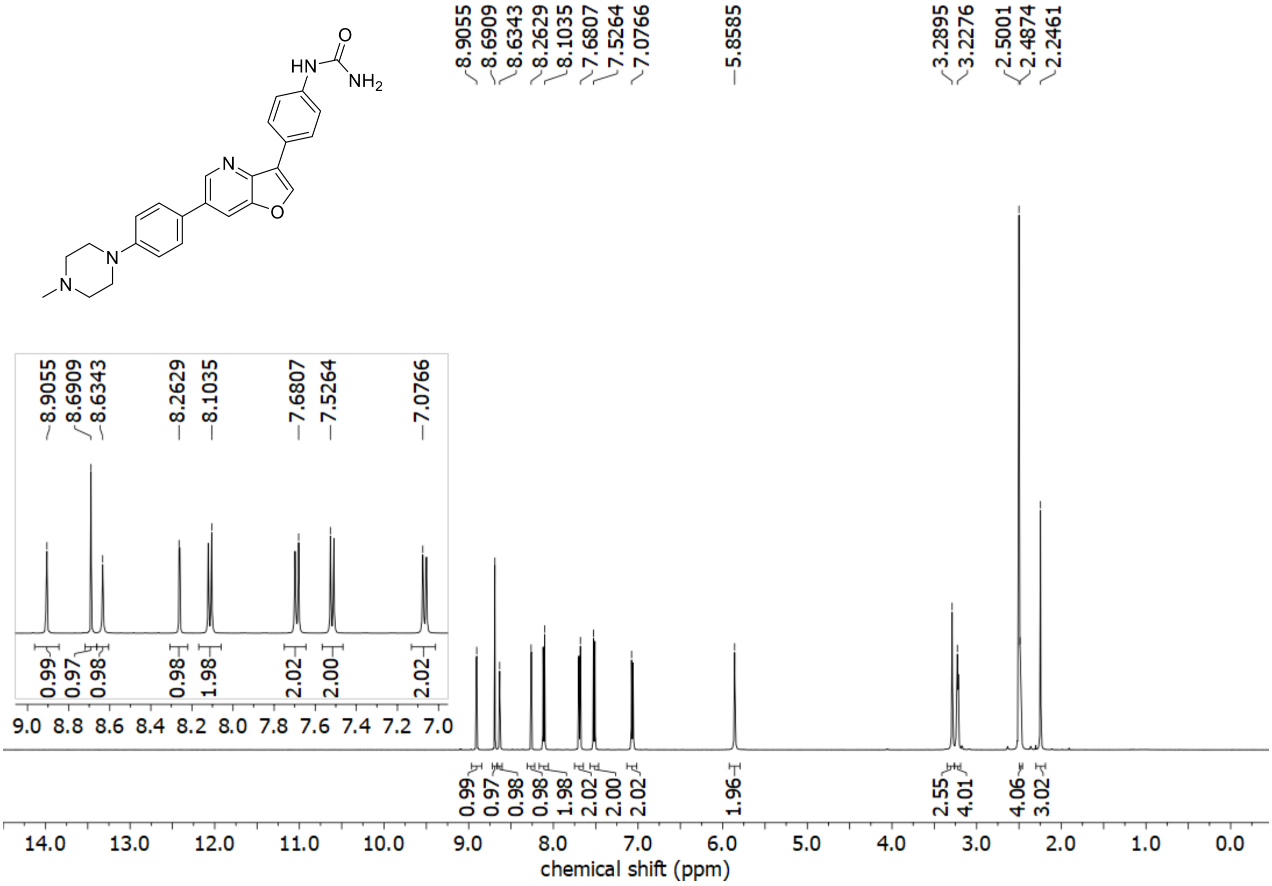

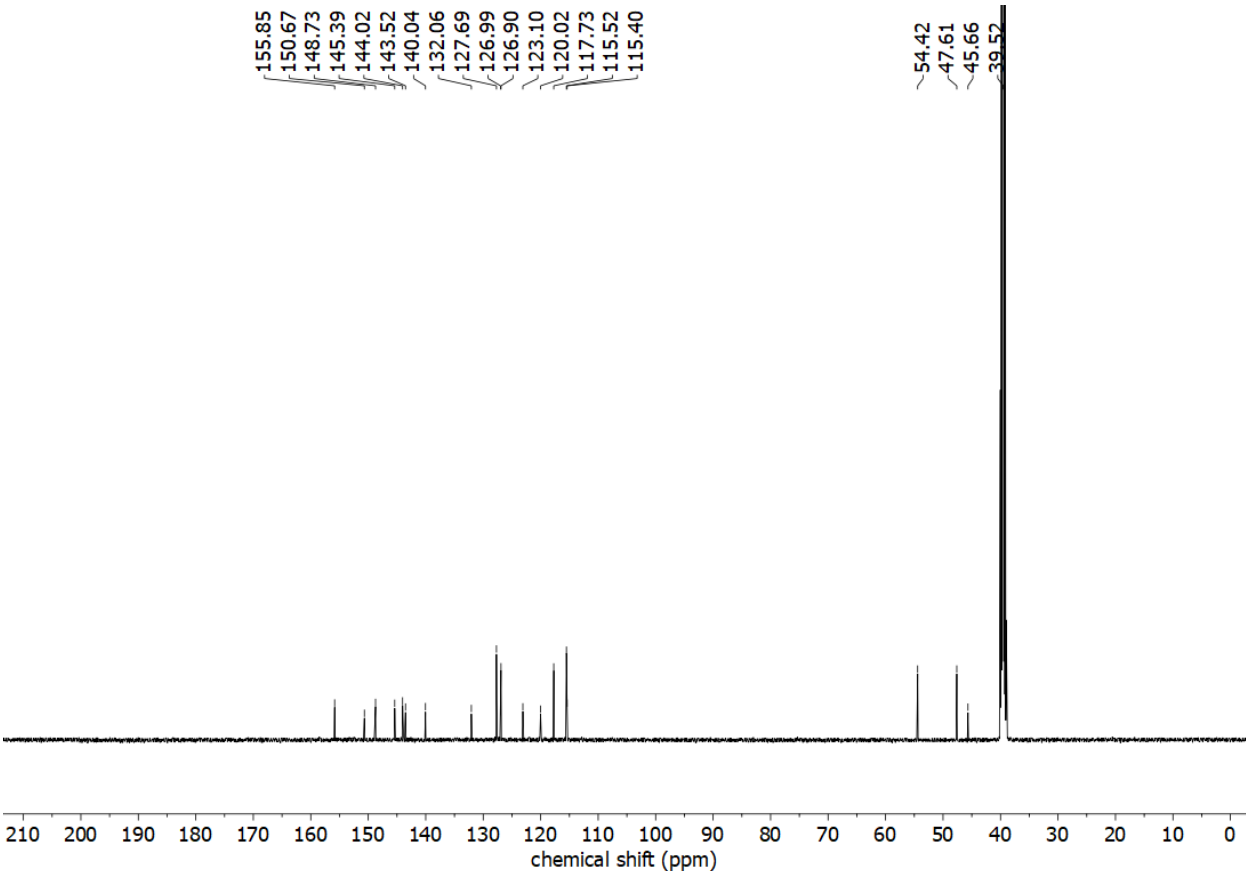

FT-IR spectrum (neat) of **77.**

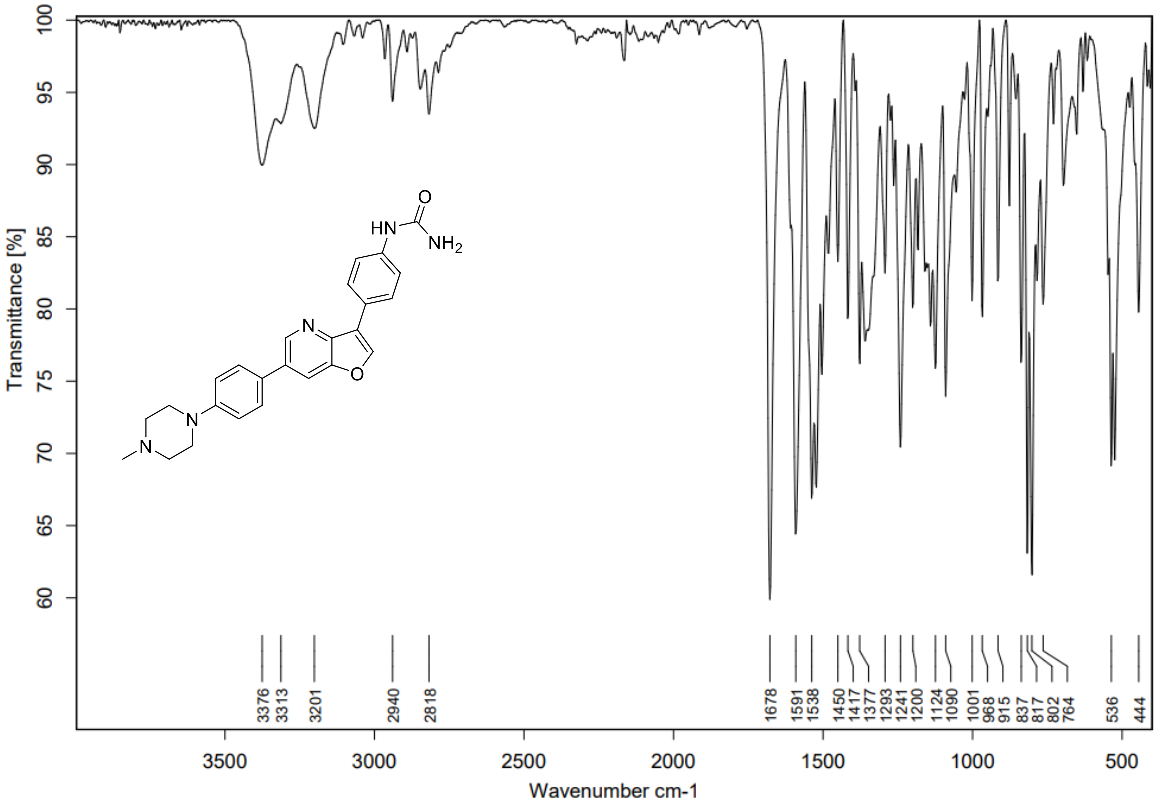

HRMS spectrum of **77.**

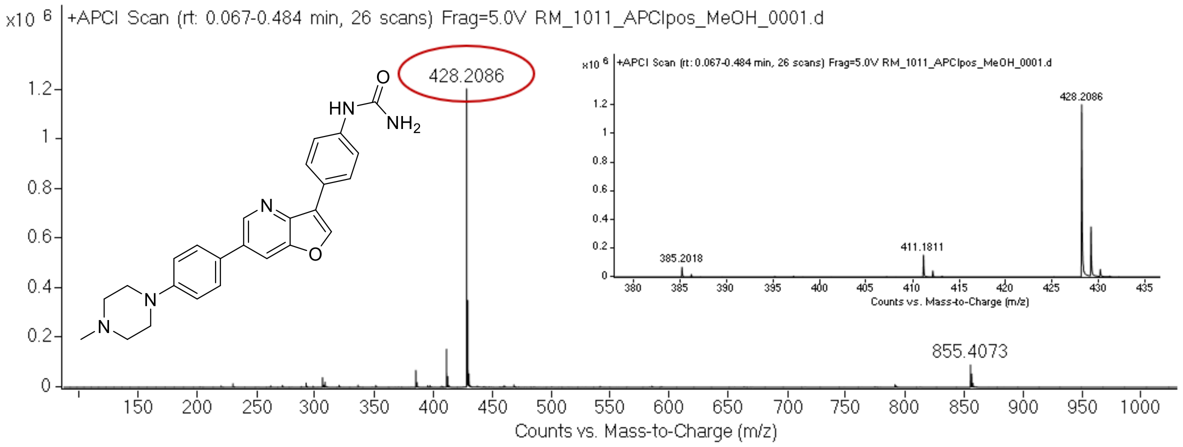

^{1}H (500 MHz) and ^{13}C NMR (126 MHz) spectra of **78** in chloroform-*d*.

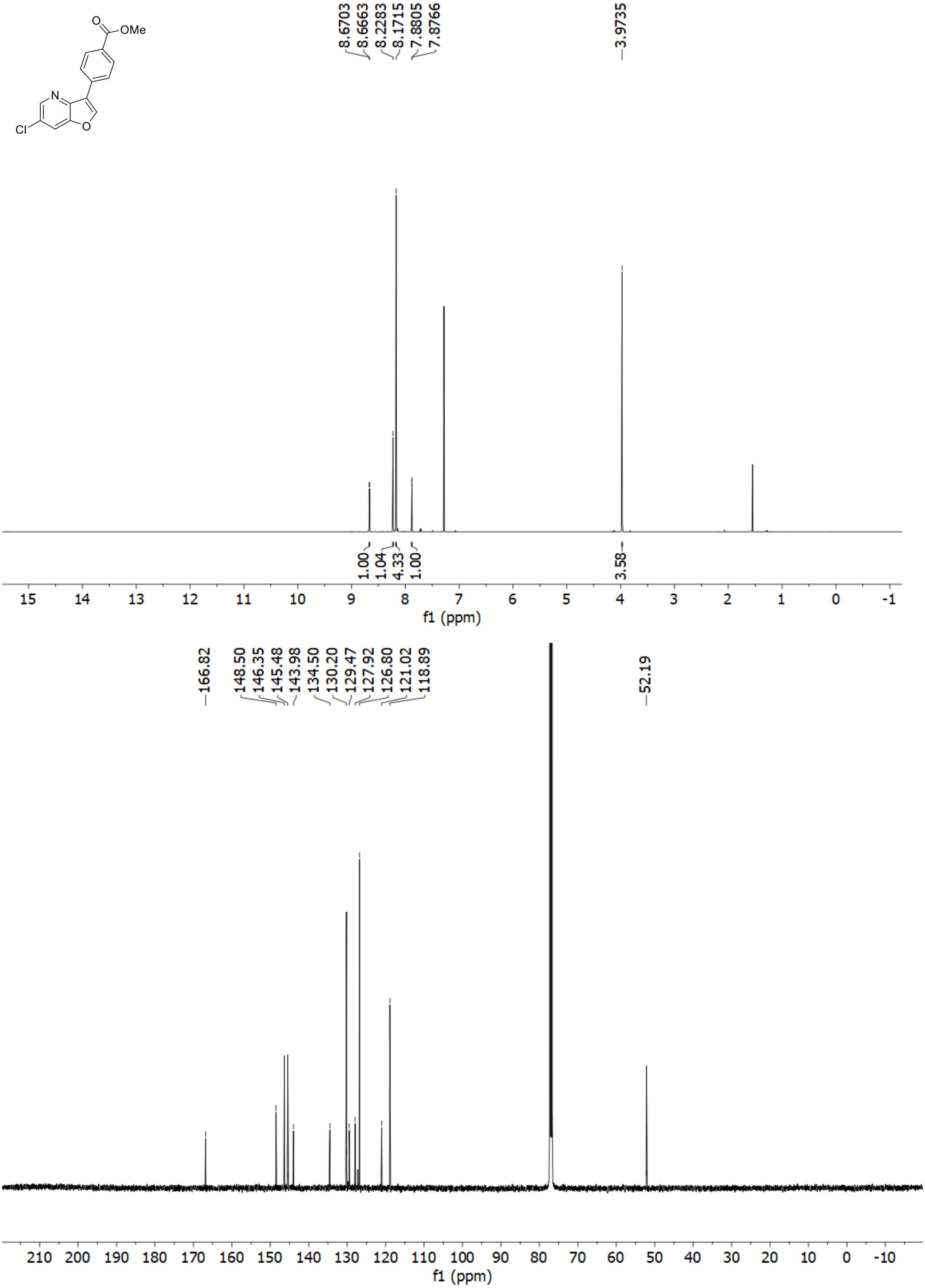

FT-IR spectrum (neat) of **78.**

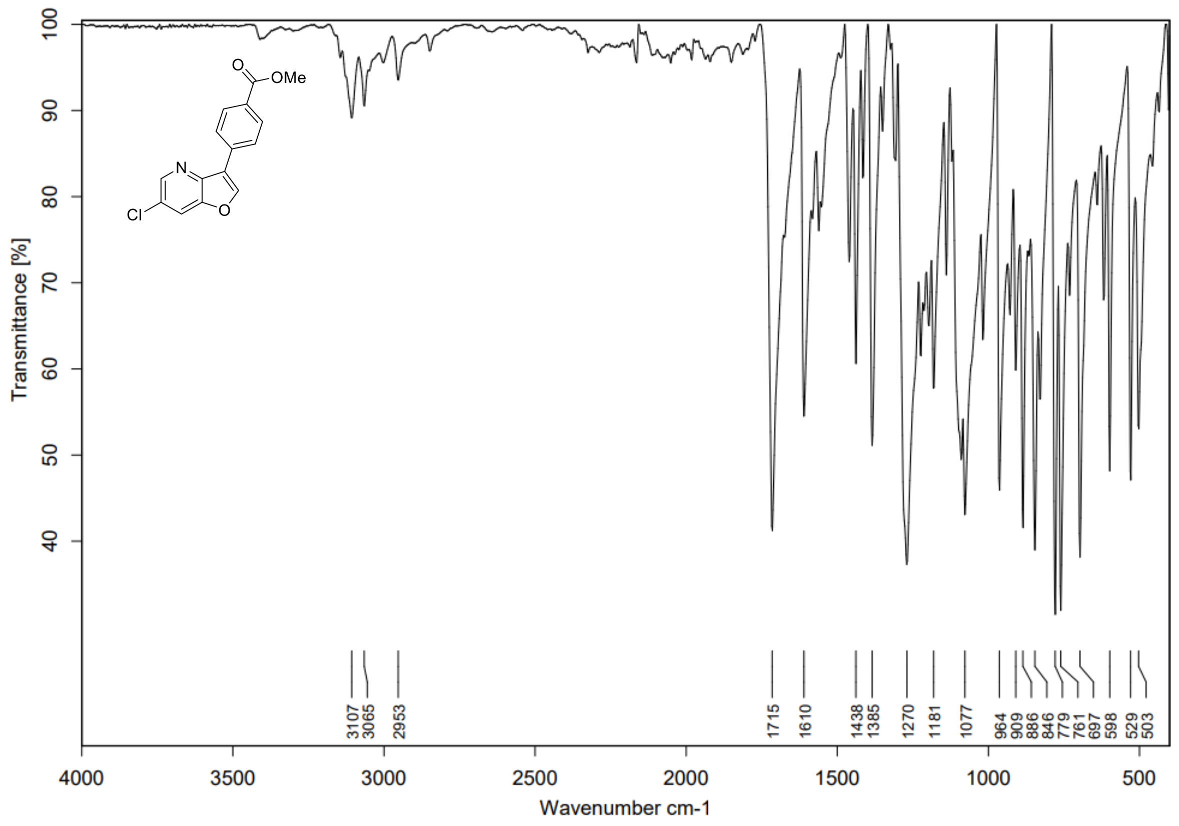

HRMS spectrum of **78.**

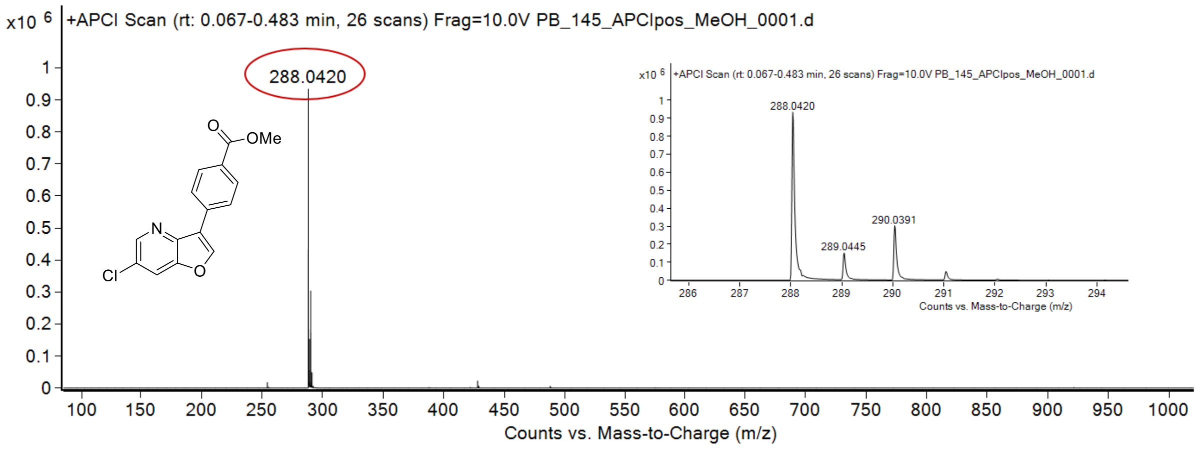

^{1}H (500 MHz) and ^{13}C NMR (126 MHz) spectra of **79** in chloroform-*d*.

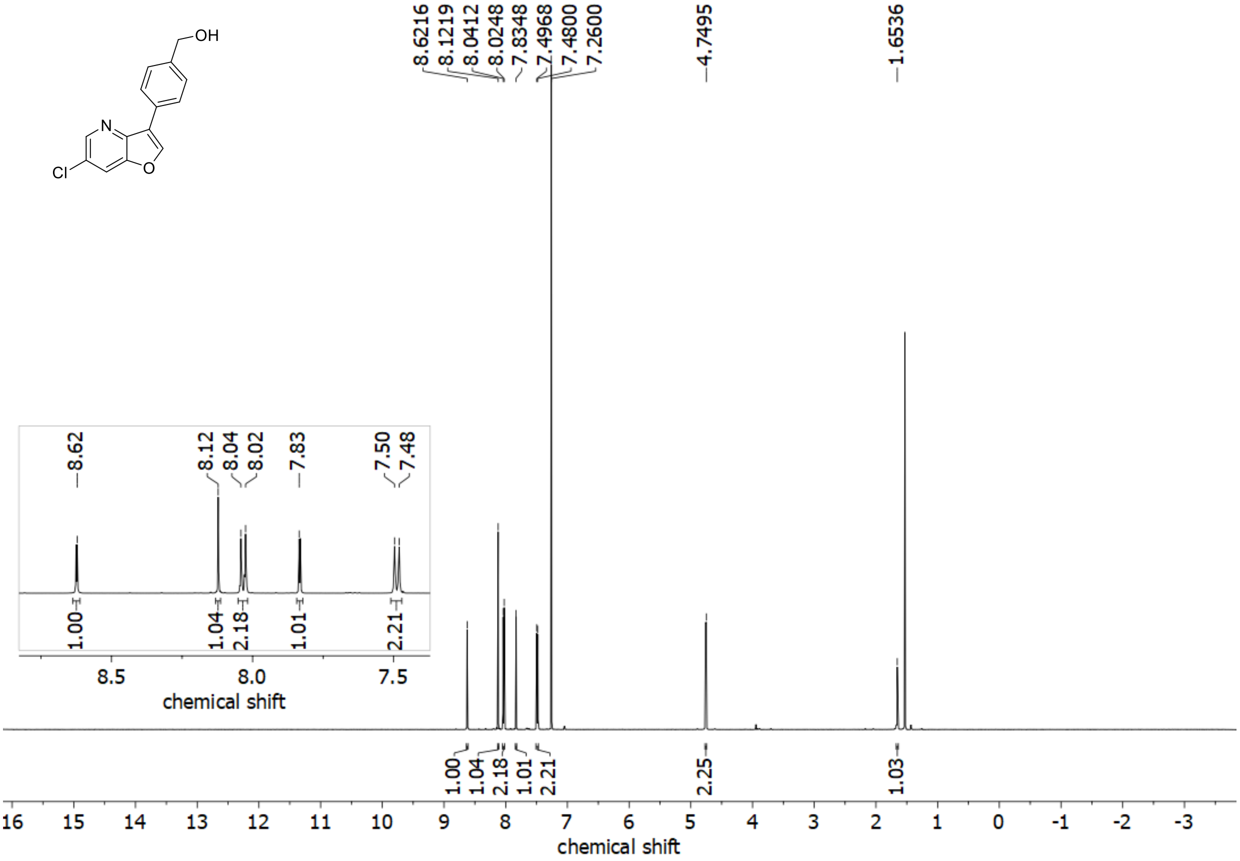

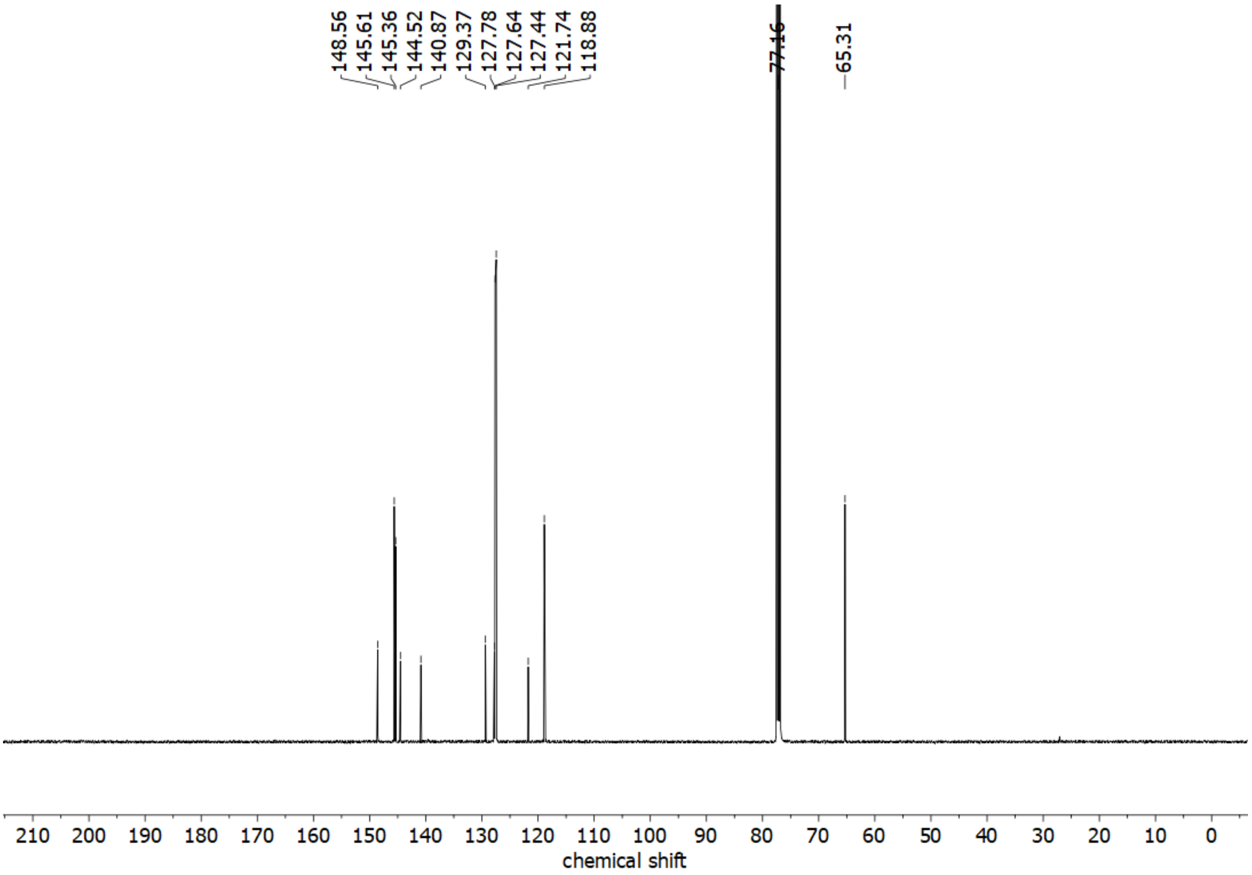

FT-IR spectrum (neat) of **79.**

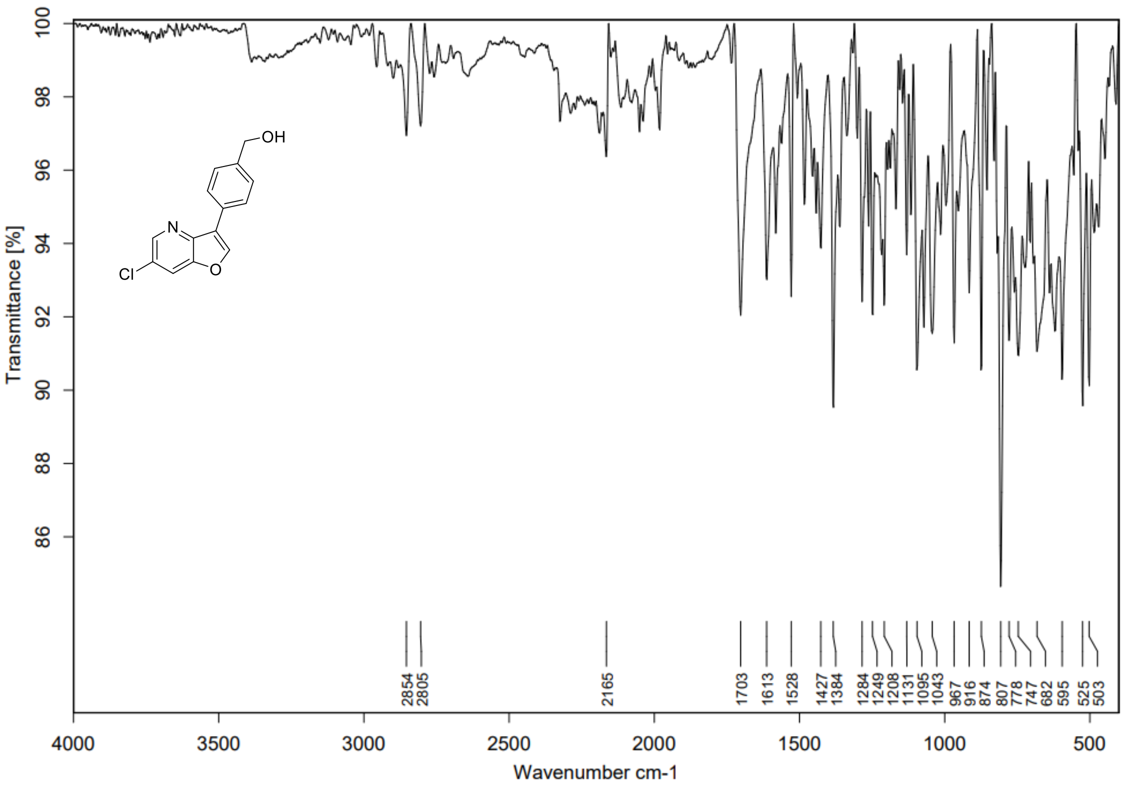

HRMS spectrum of **79.**

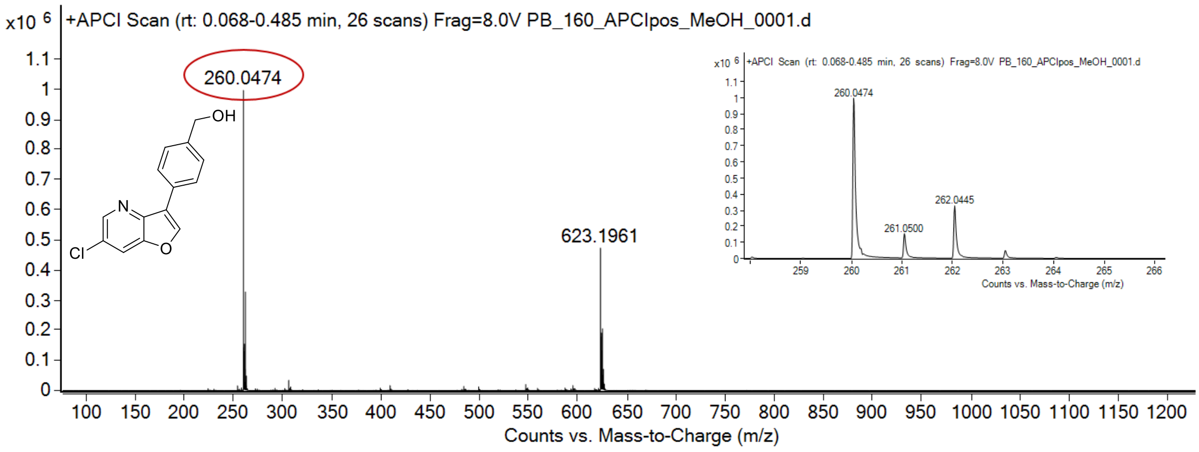

^{1}H (500 MHz) and ^{13}C NMR (126 MHz) spectra of **80** in chloroform-*d*.

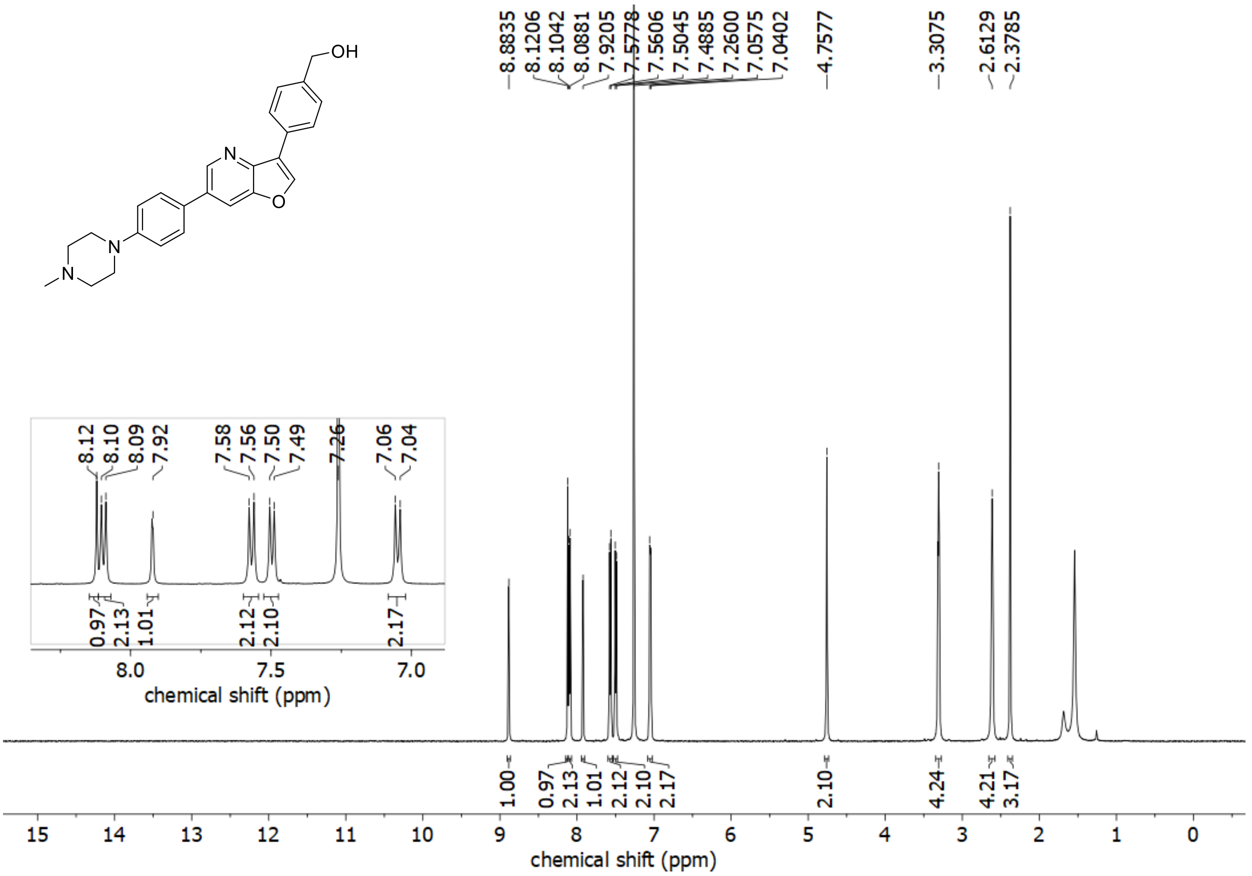

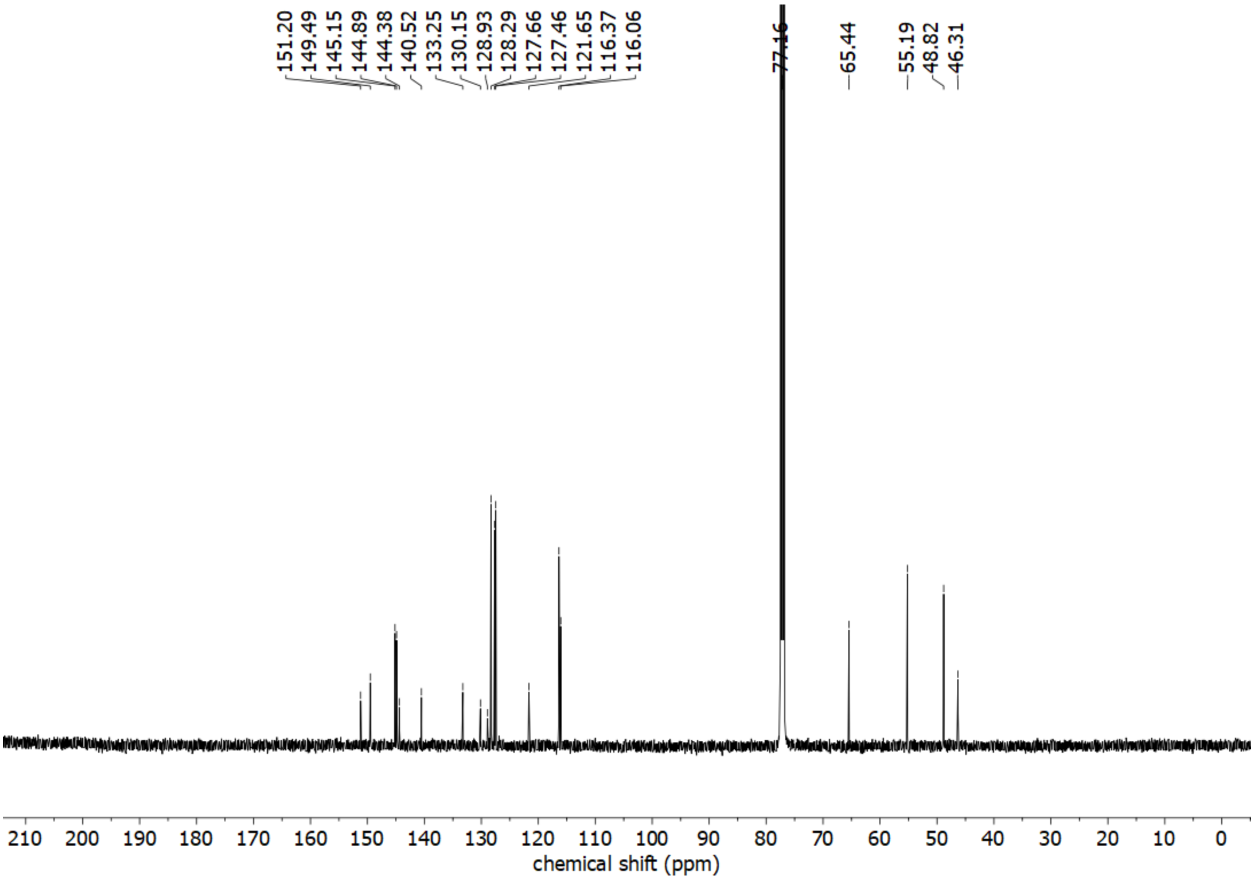

FT-IR spectrum (neat) of **80.**

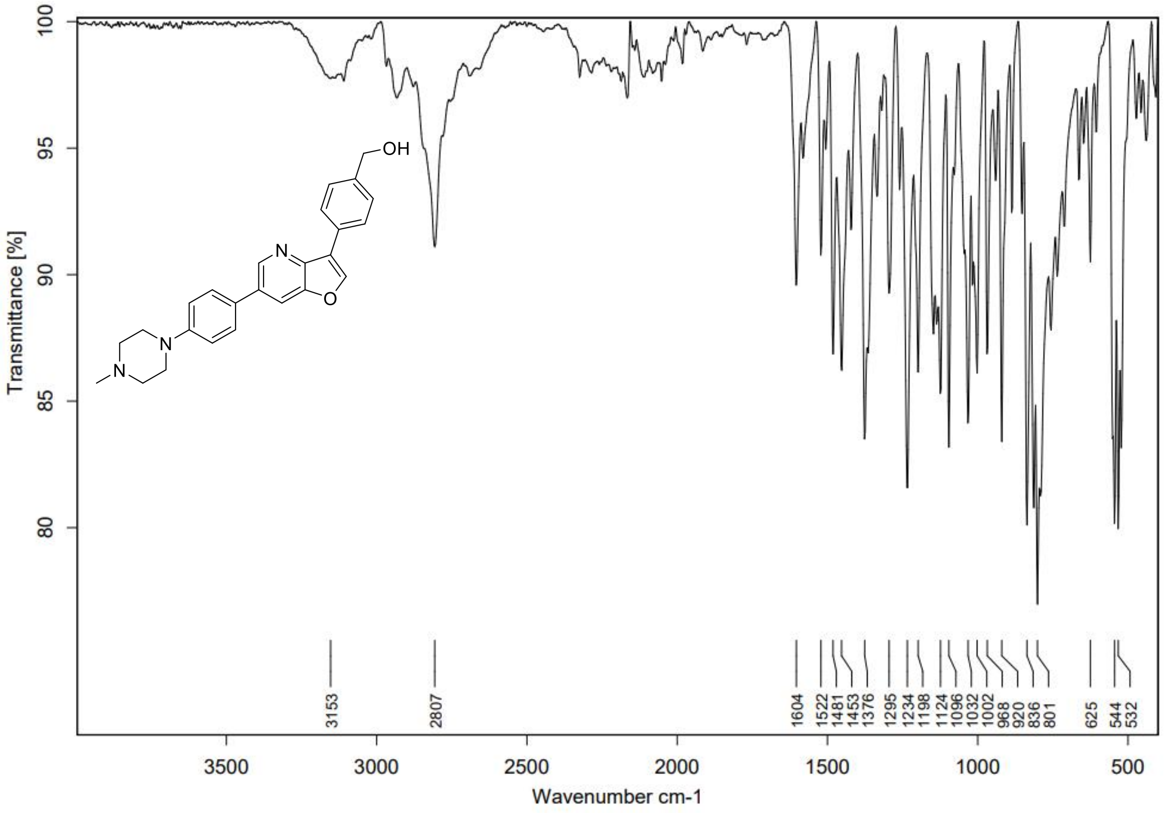

HRMS spectrum of **80.**

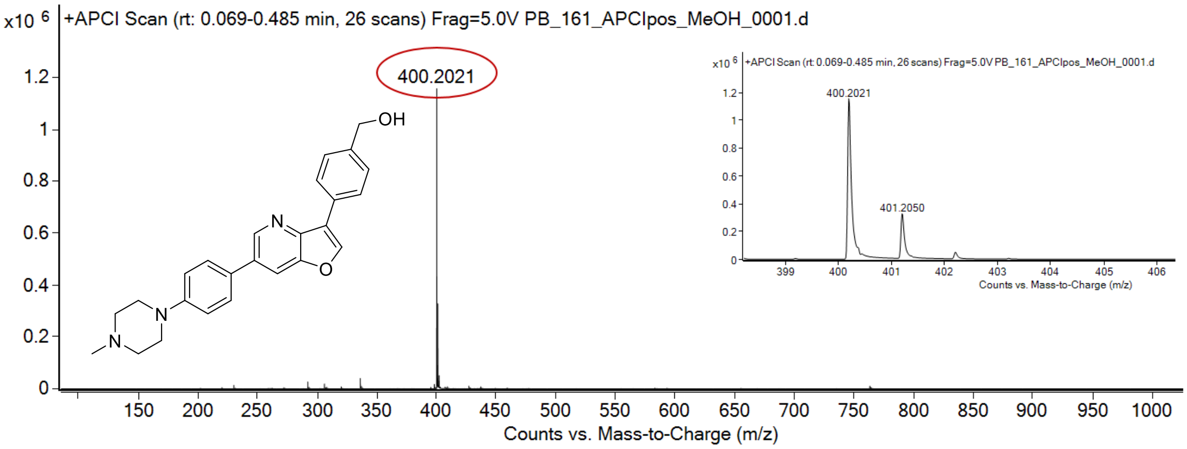

^{1}H (500 MHz) and ^{13}C NMR (126 MHz) spectra of **81** in chloroform-*d*.

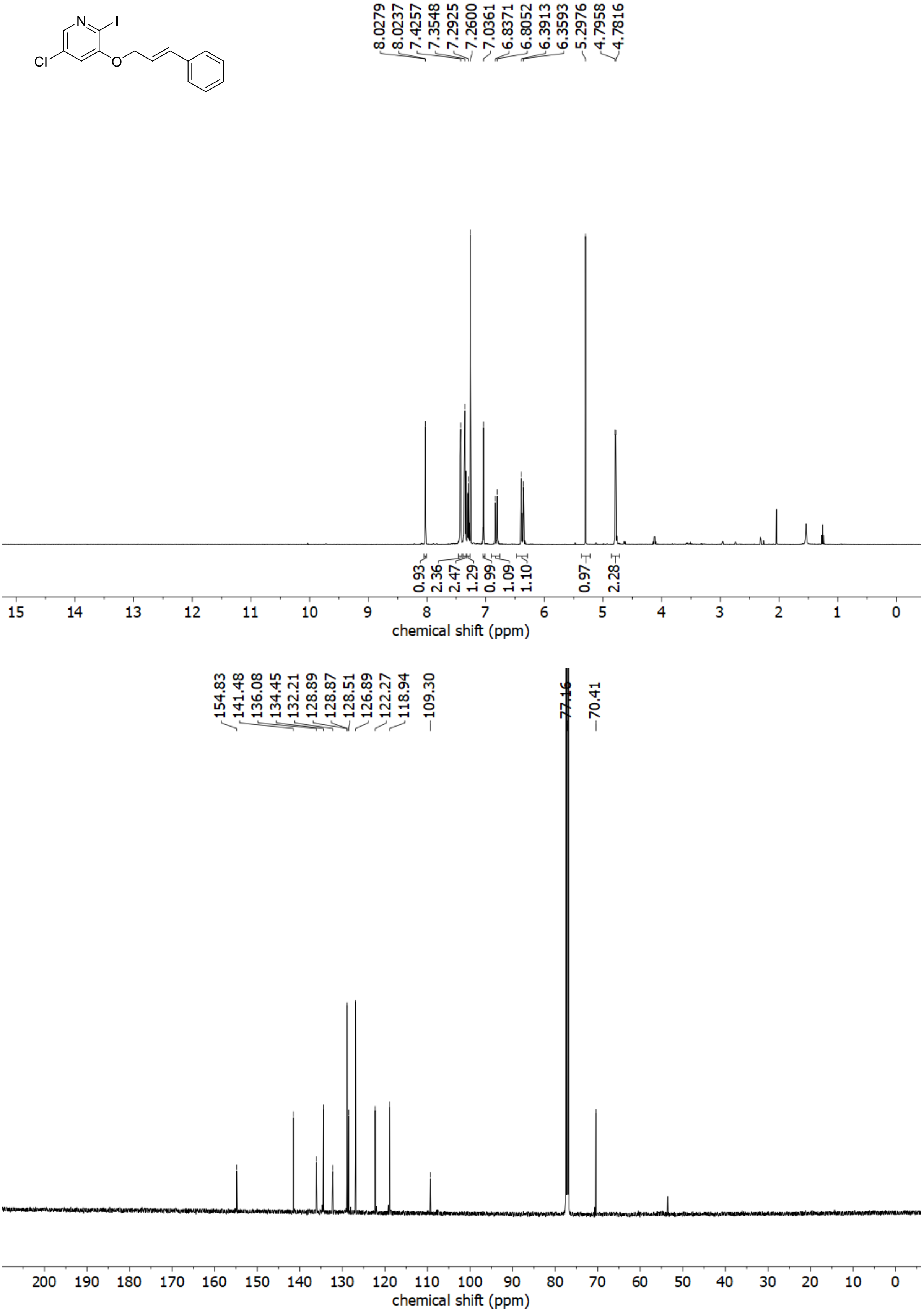

FT-IR spectrum (neat) of **81.**

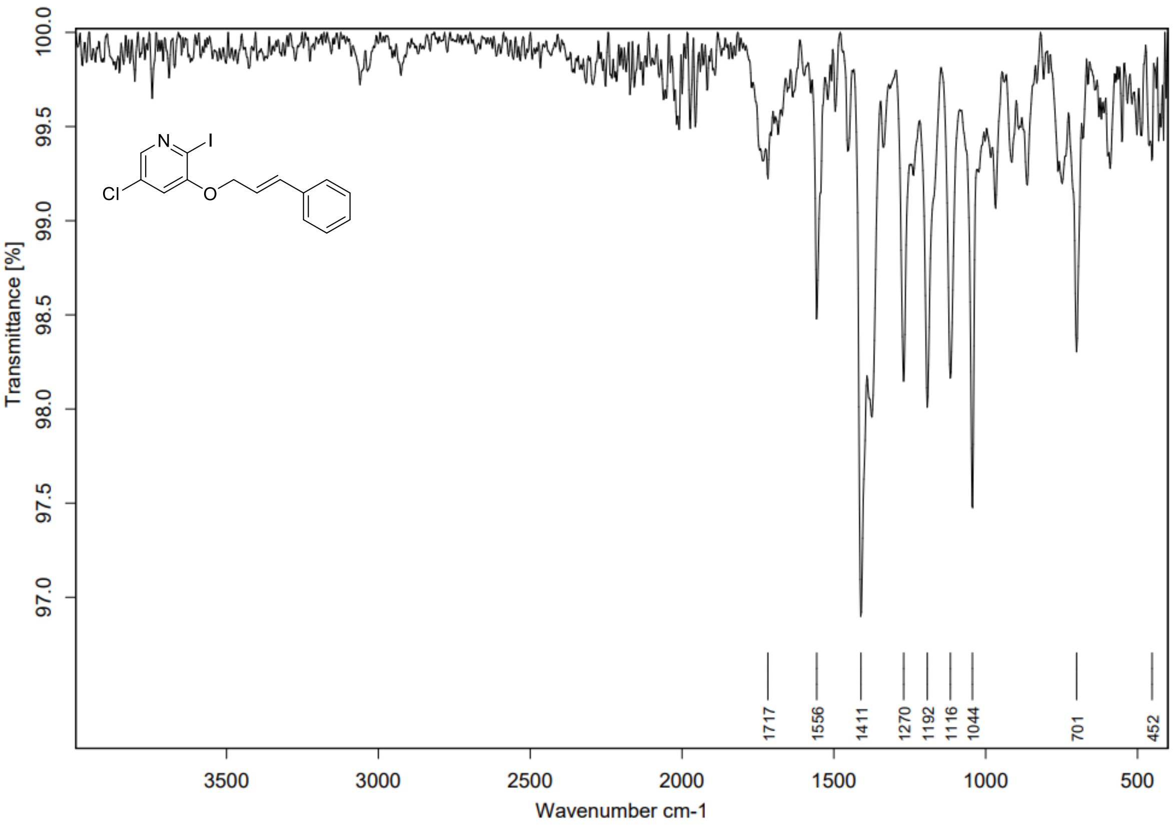

HRMS spectrum of **81**.

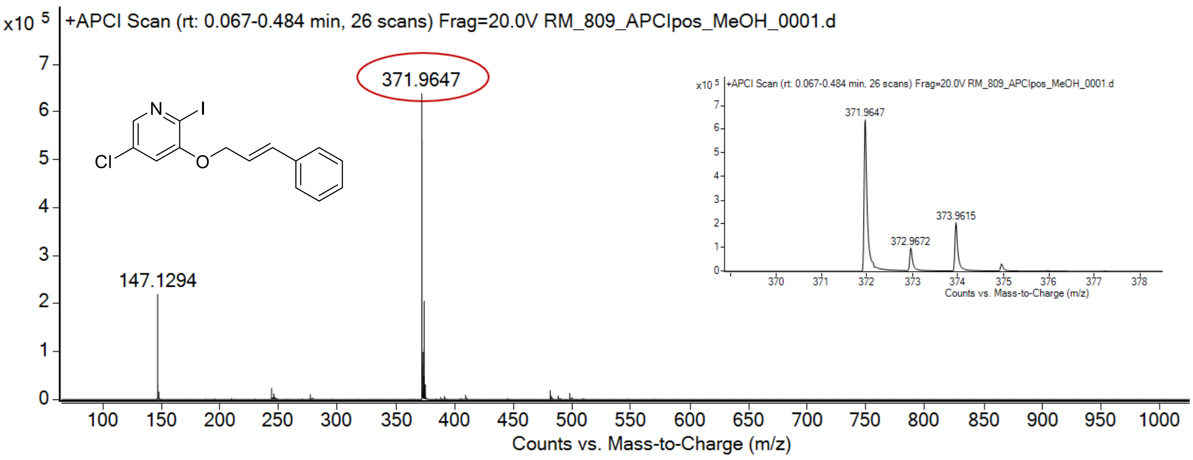

^{1}H (500 MHz) and ^{13}C NMR (126 MHz) spectra of **82** in chloroform-*d*.

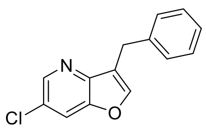

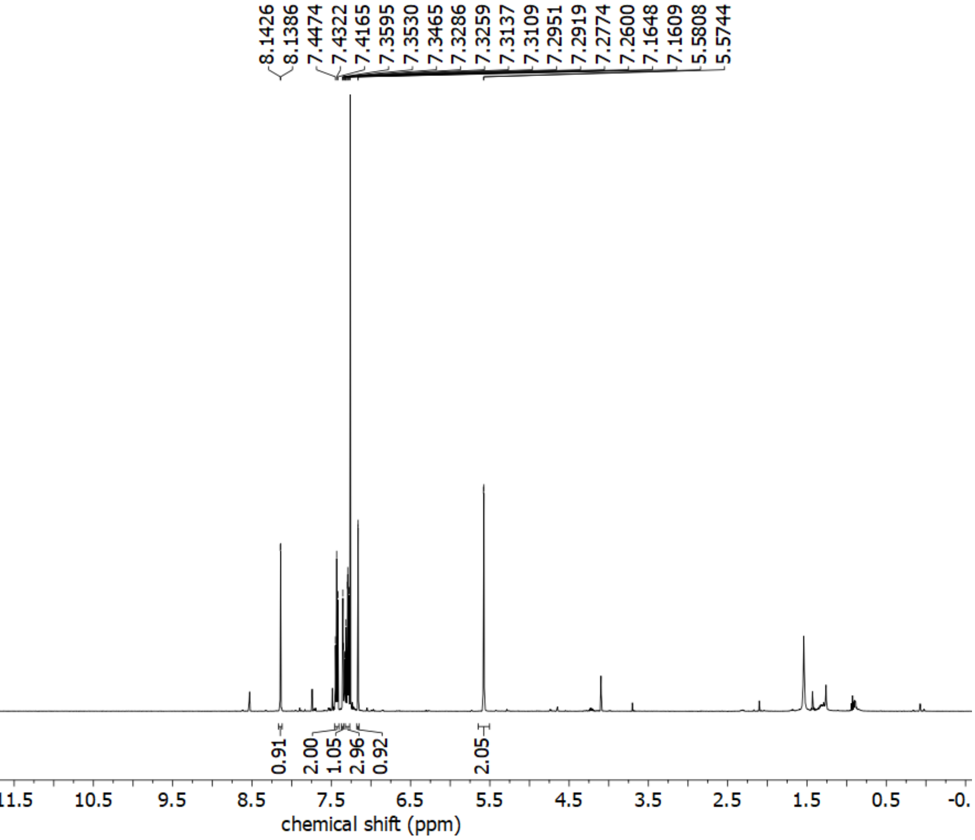

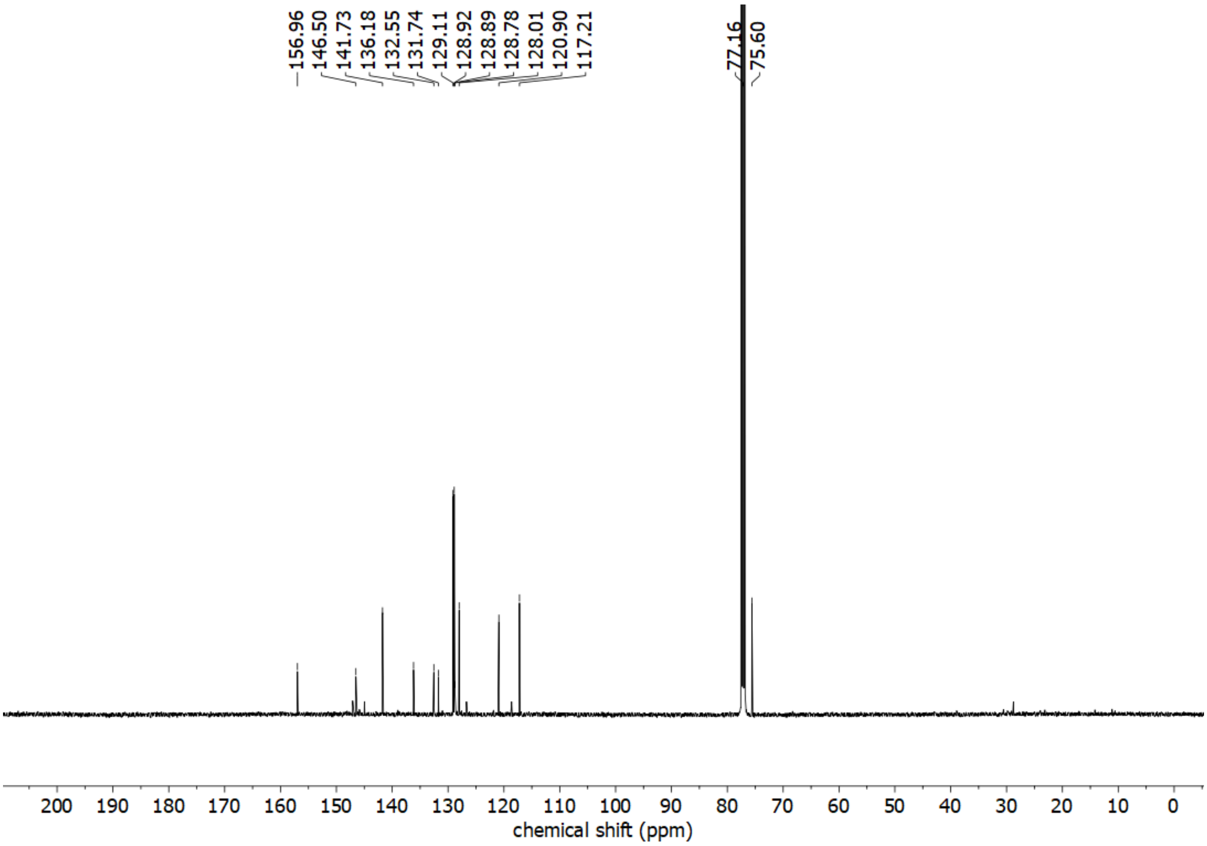

FT-IR spectrum (neat) of **82.**

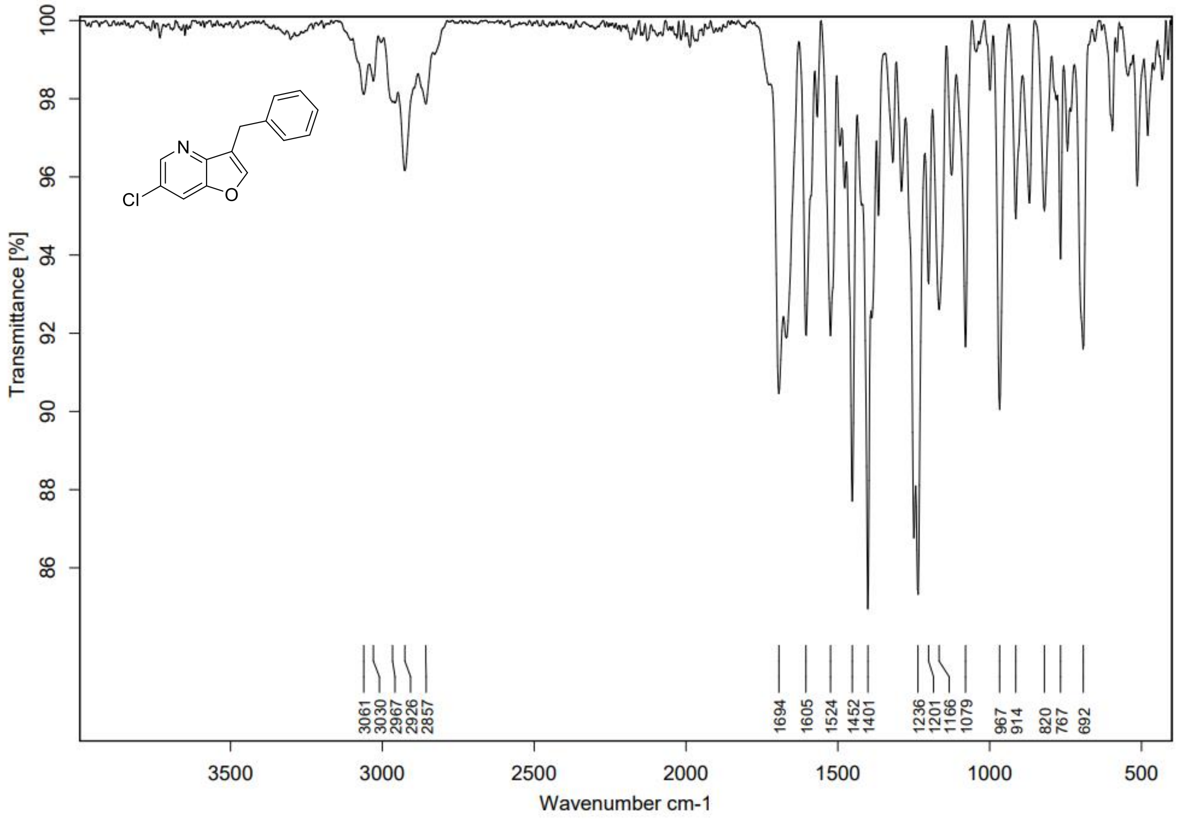

HRMS spectrum of **82**.

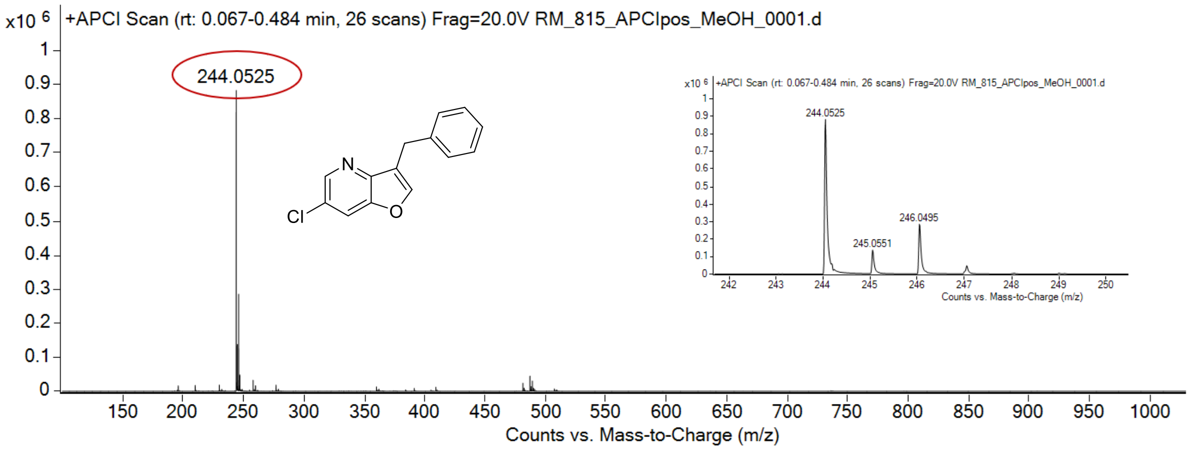

^{1}H (500 MHz) and ^{13}C NMR (126 MHz) spectra of **83** in chloroform-*d*.

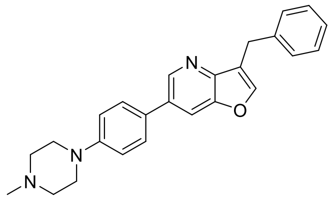

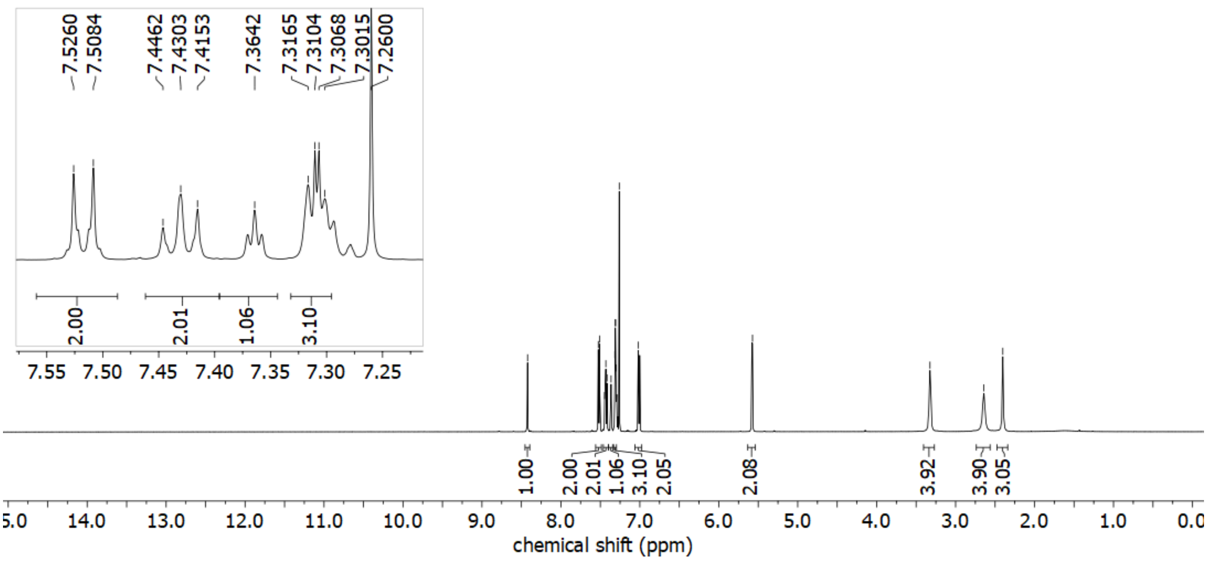

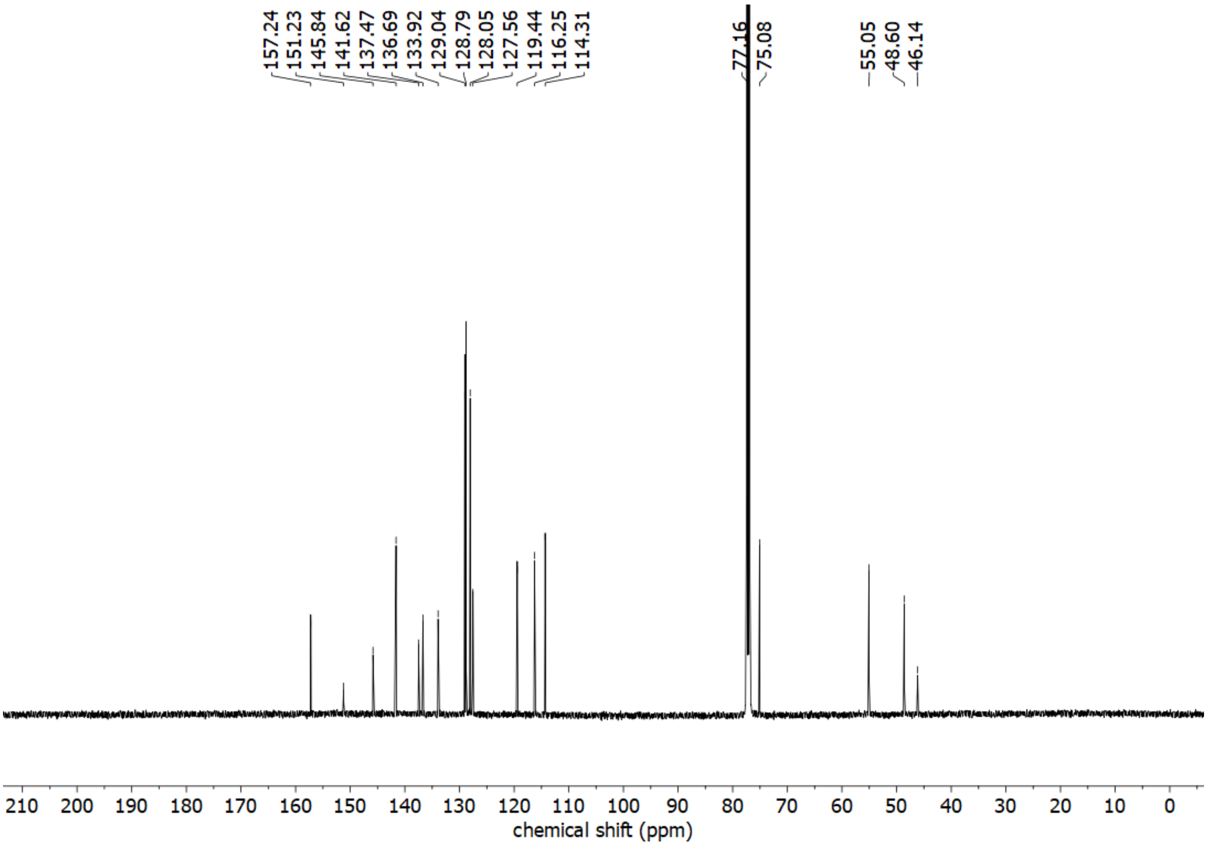

FT-IR spectrum (neat) of **83.**

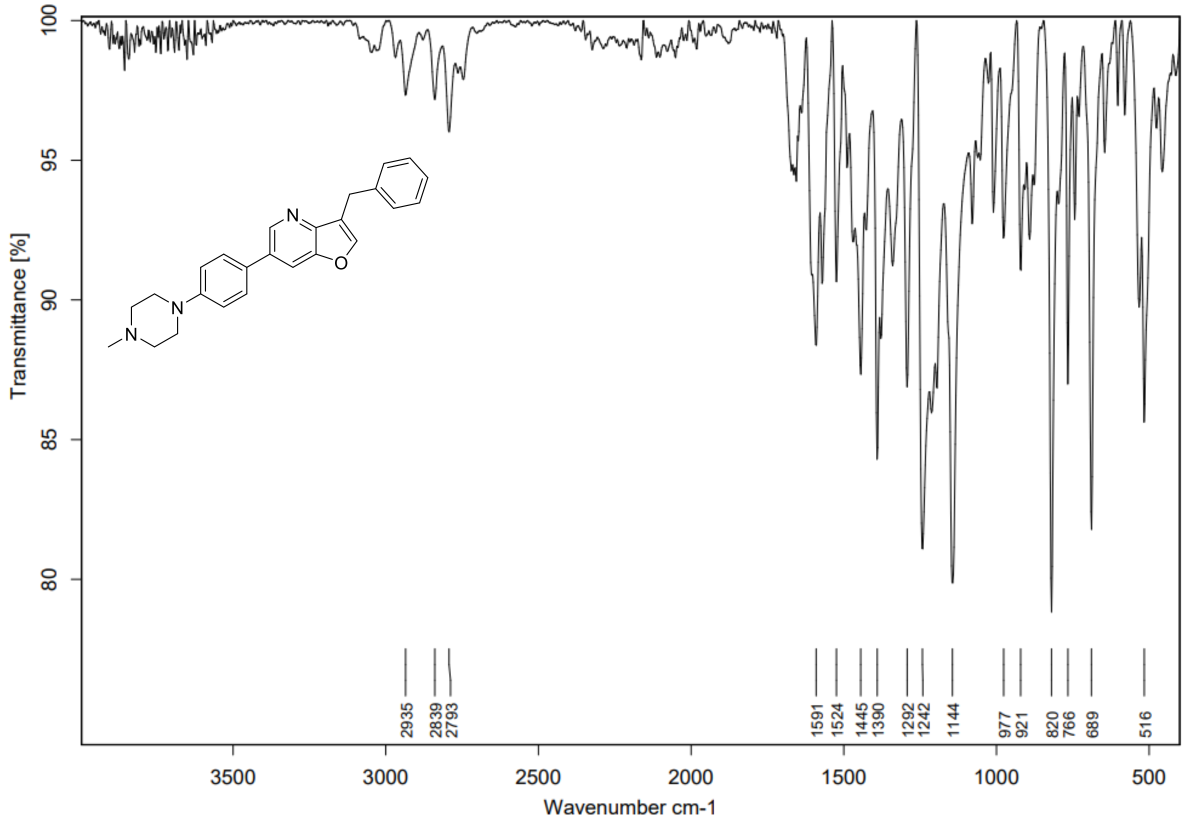

HRMS spectrum of **83.**

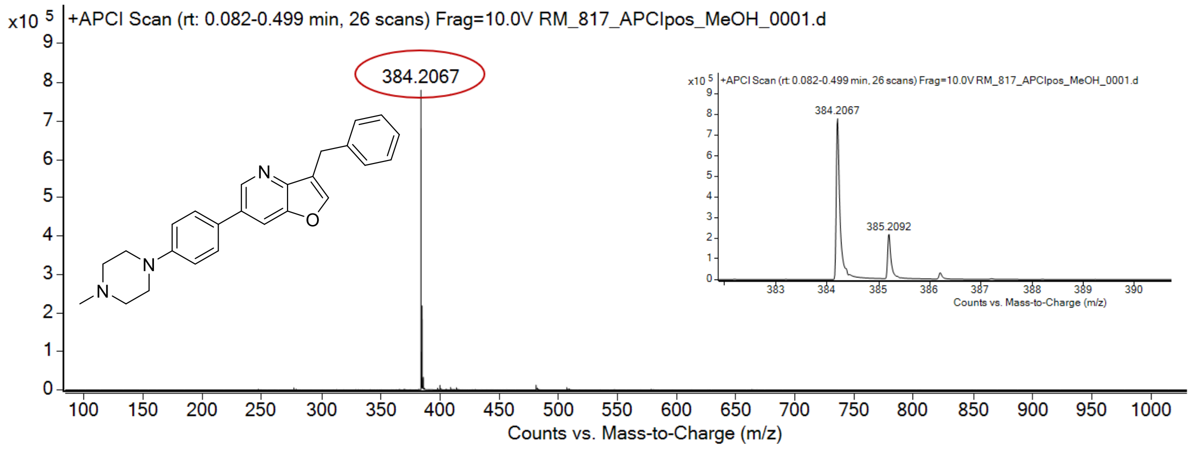

^{1}H (500 MHz) and ^{13}C NMR (126 MHz) spectra of **84** in chloroform-*d*.

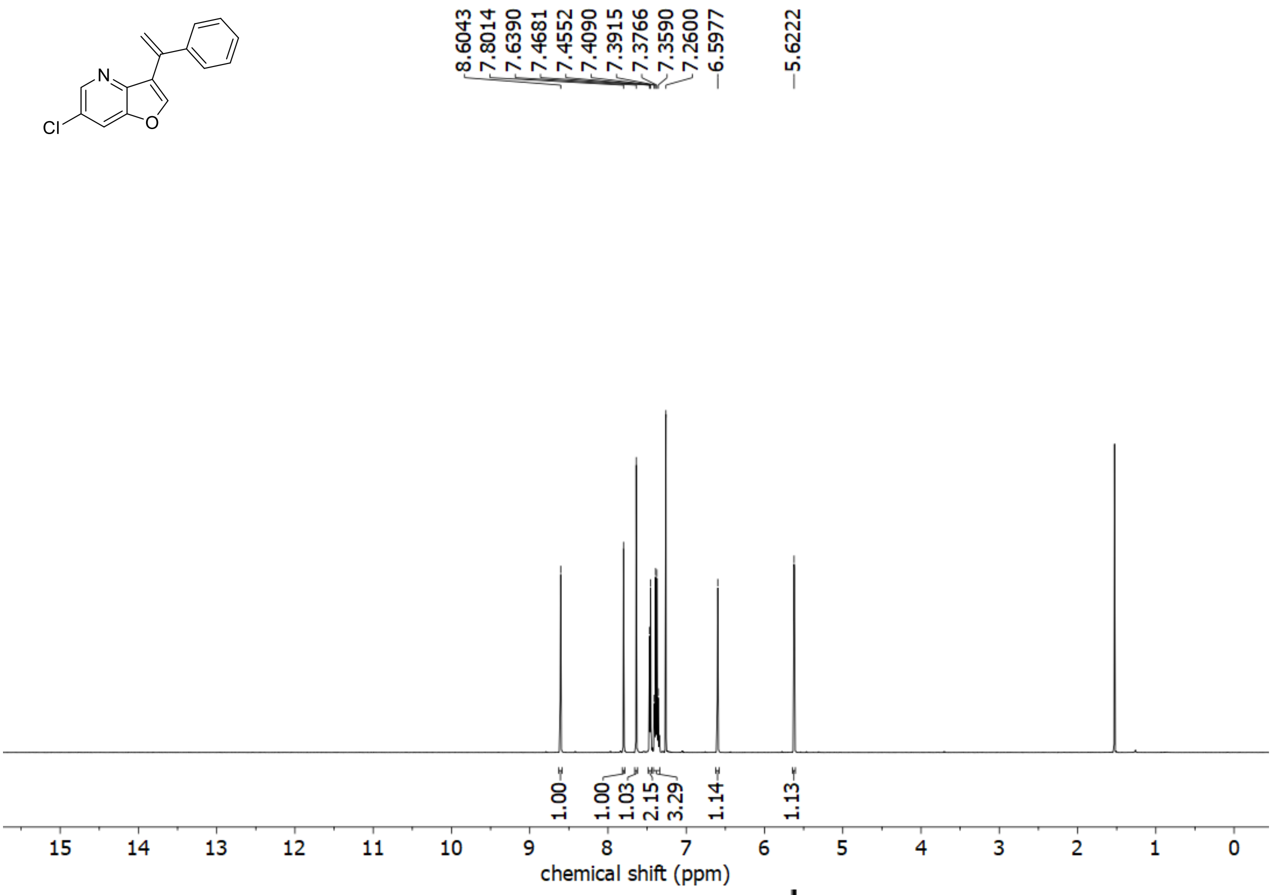

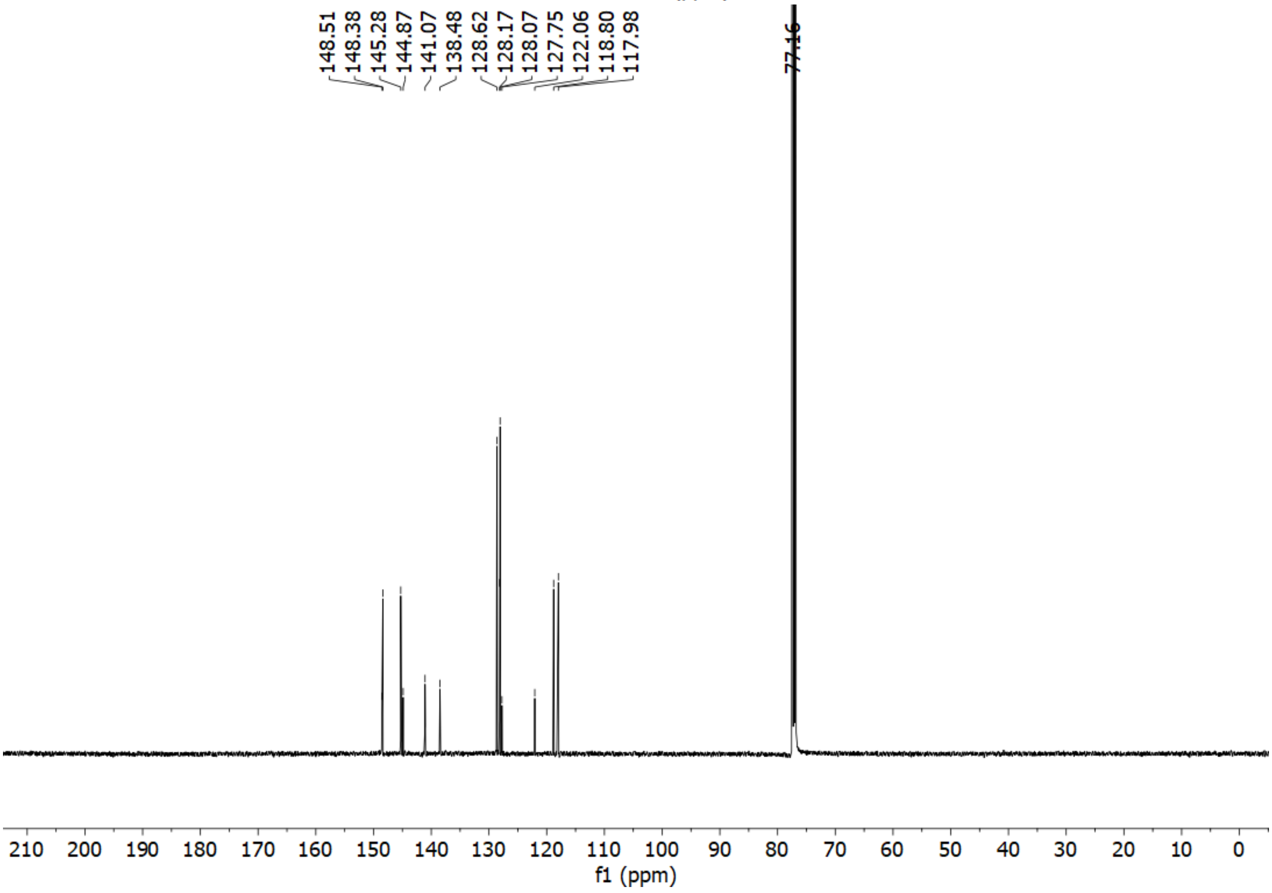

FT-IR spectrum (neat) of **84.**

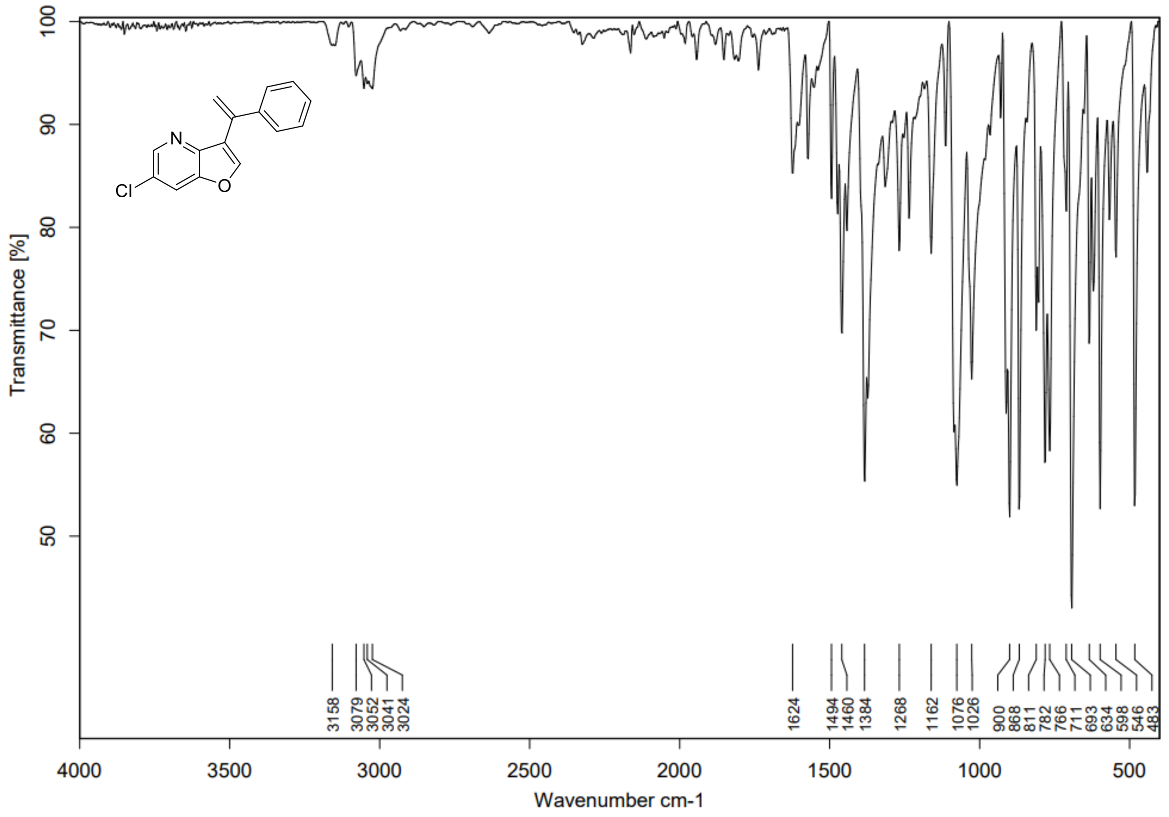

HRMS spectrum of **84.**

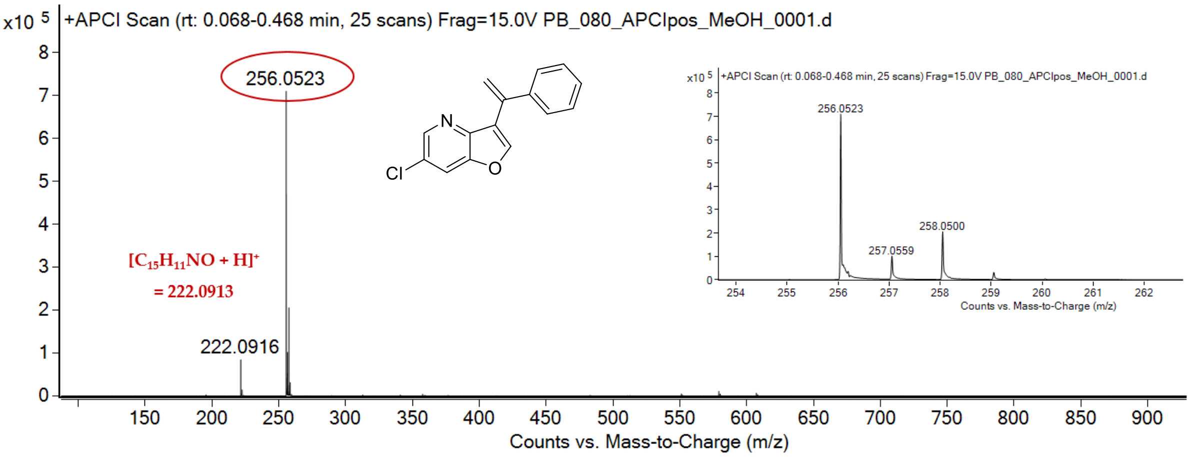

^{1}H (500 MHz) and ^{13}C NMR (126 MHz) spectra of **85** in chloroform-*d*.

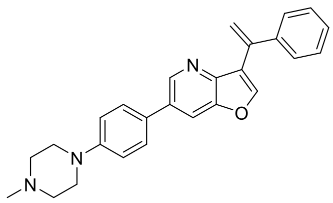

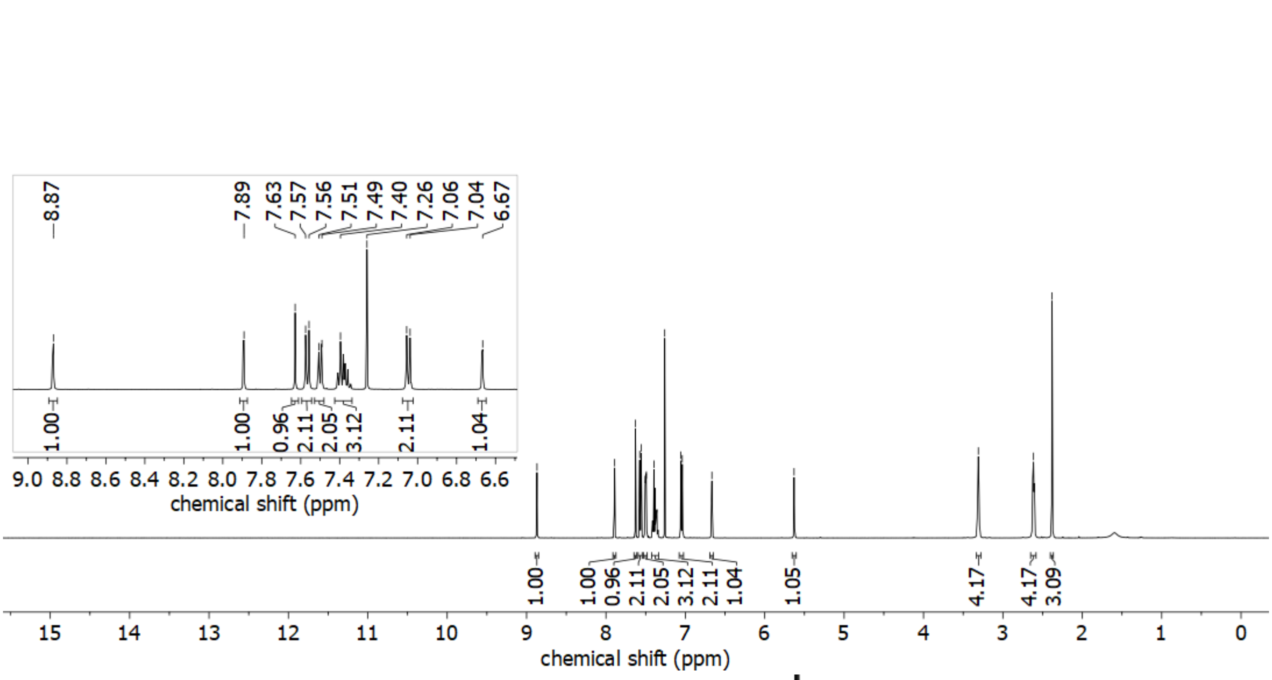

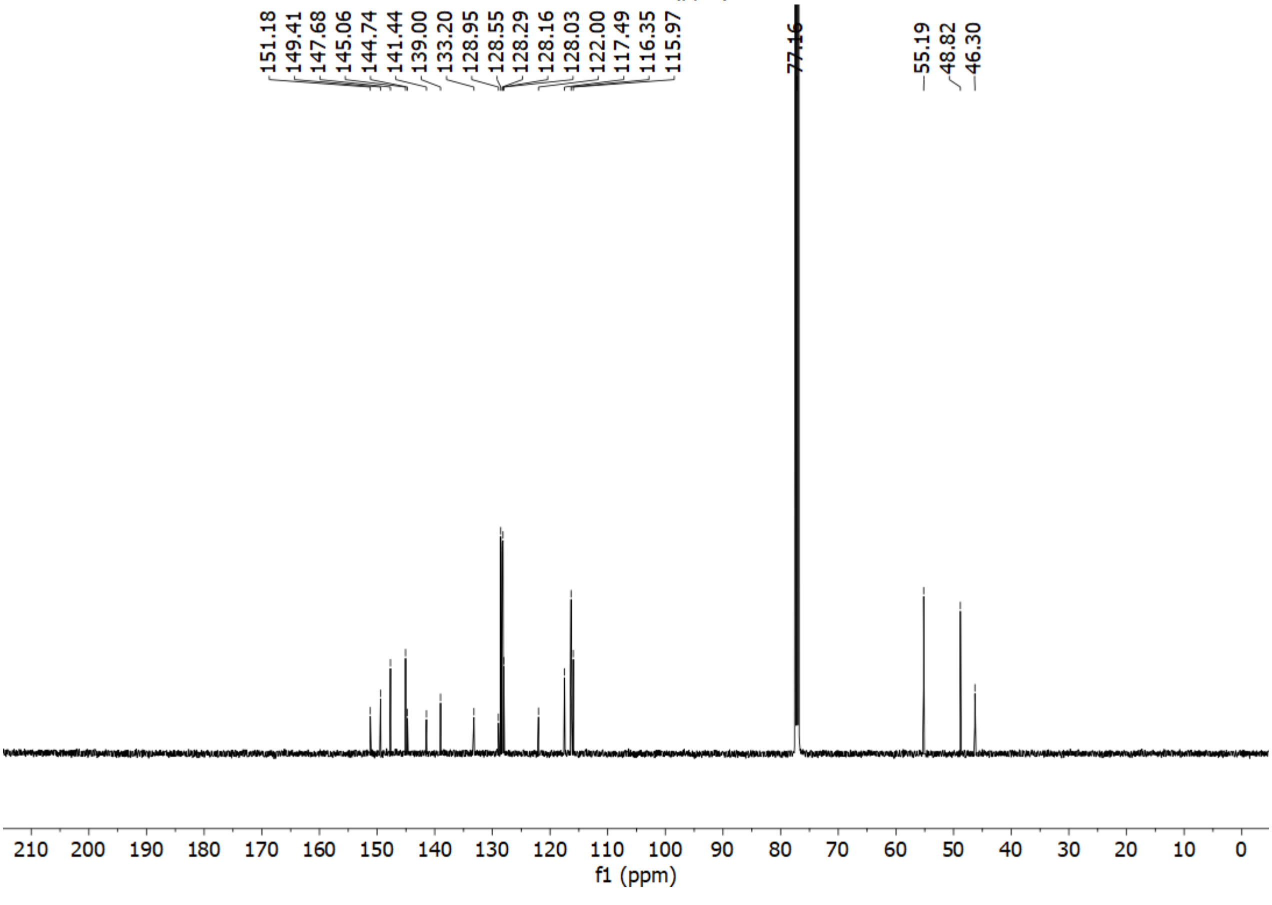

FT-IR spectrum (neat) of **85.**

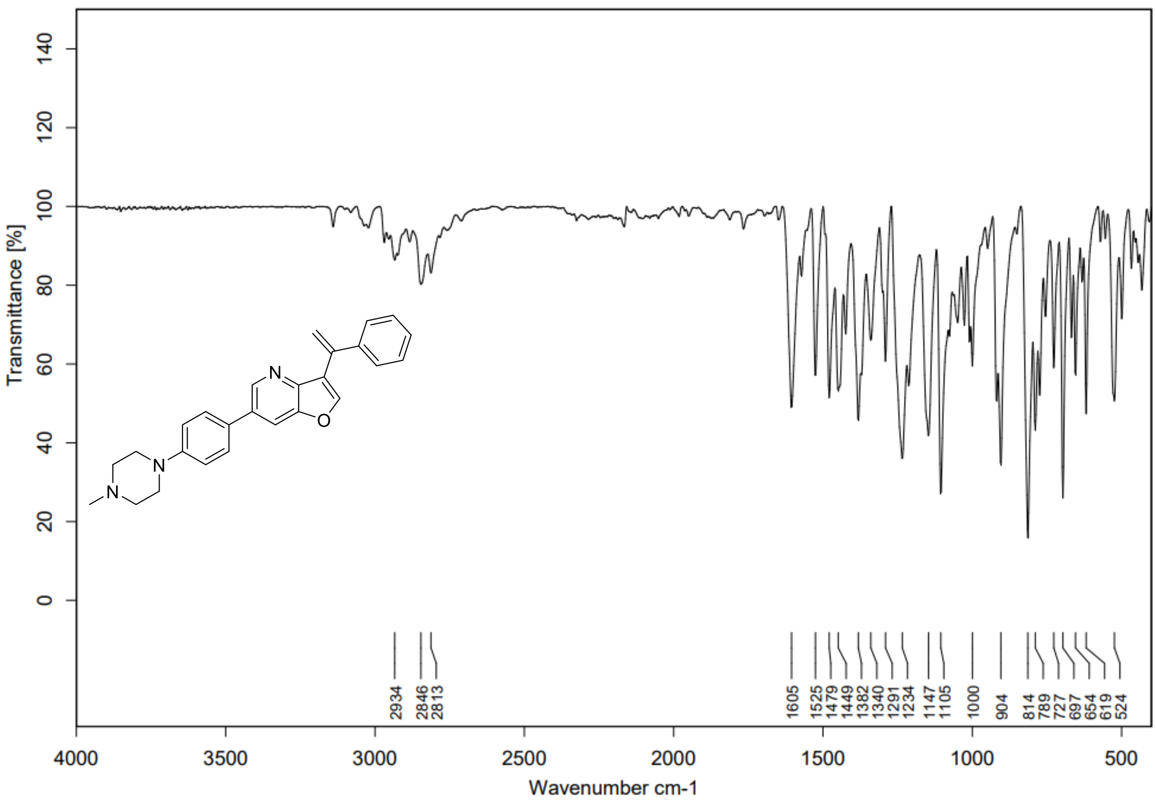

HRMS spectrum of **85.**

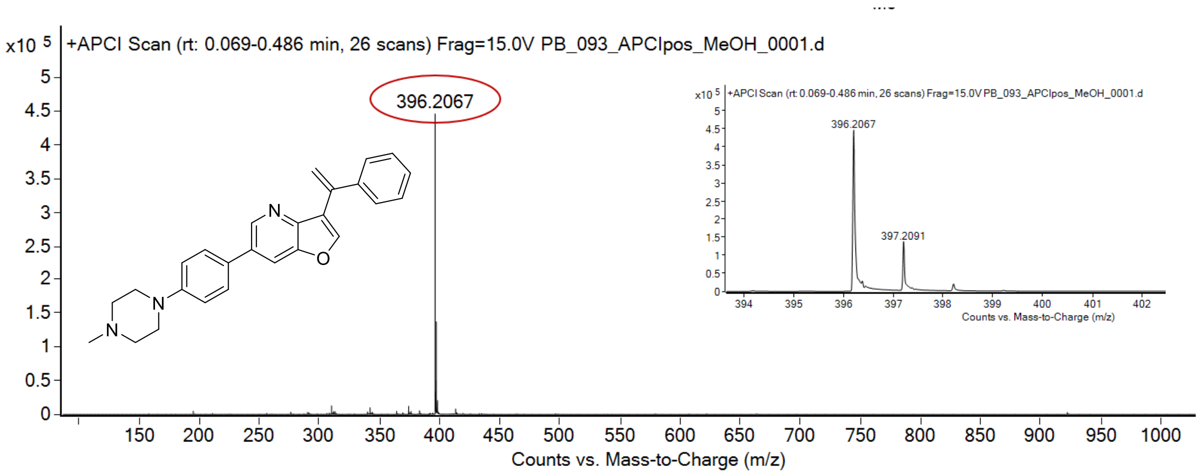

^{1}H (500 MHz) and ^{13}C NMR (126 MHz) spectra of **86** in chloroform-*d*.

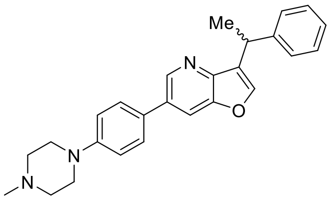

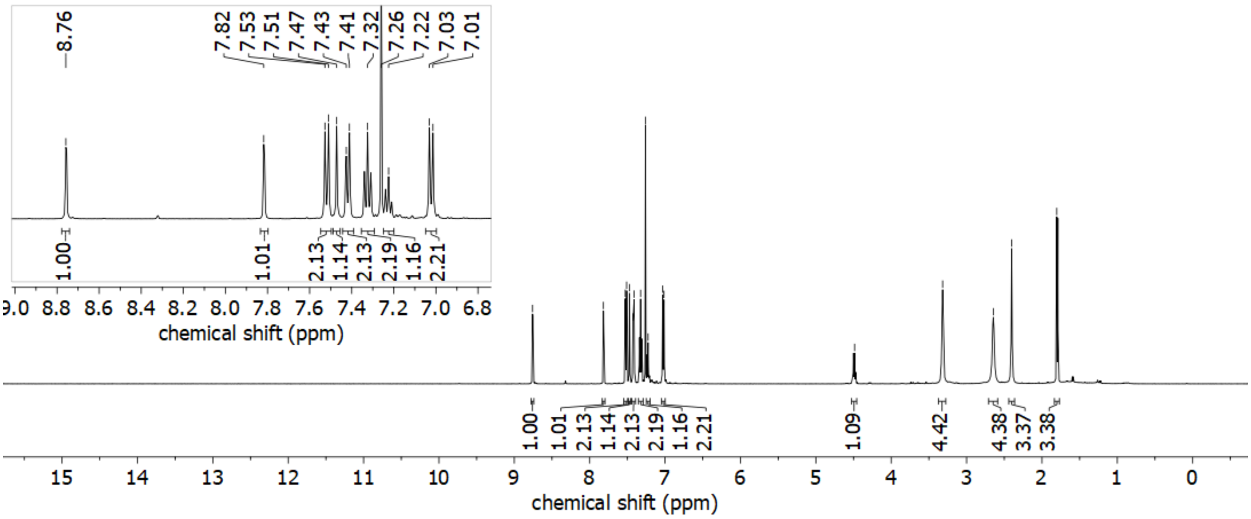

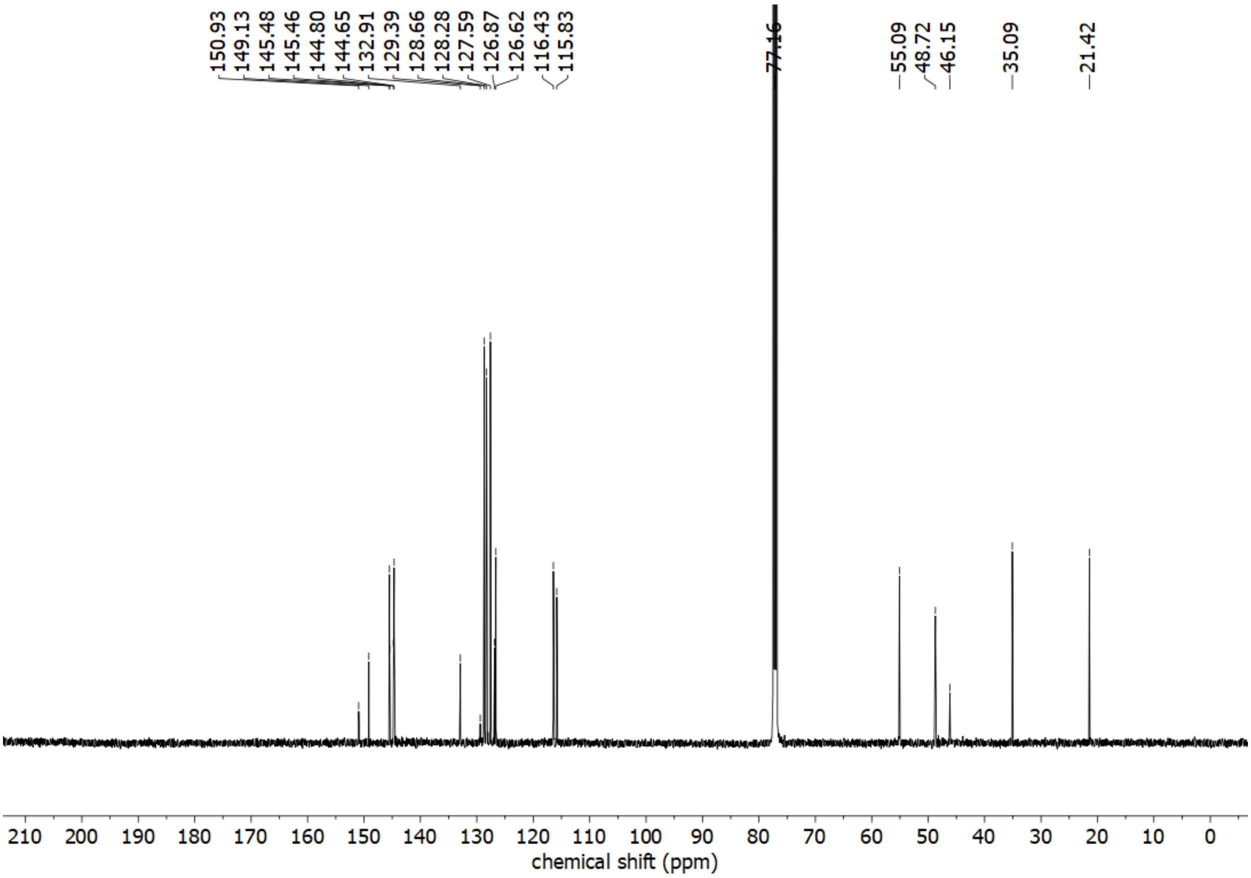

FT-IR spectrum (neat) of **86.**

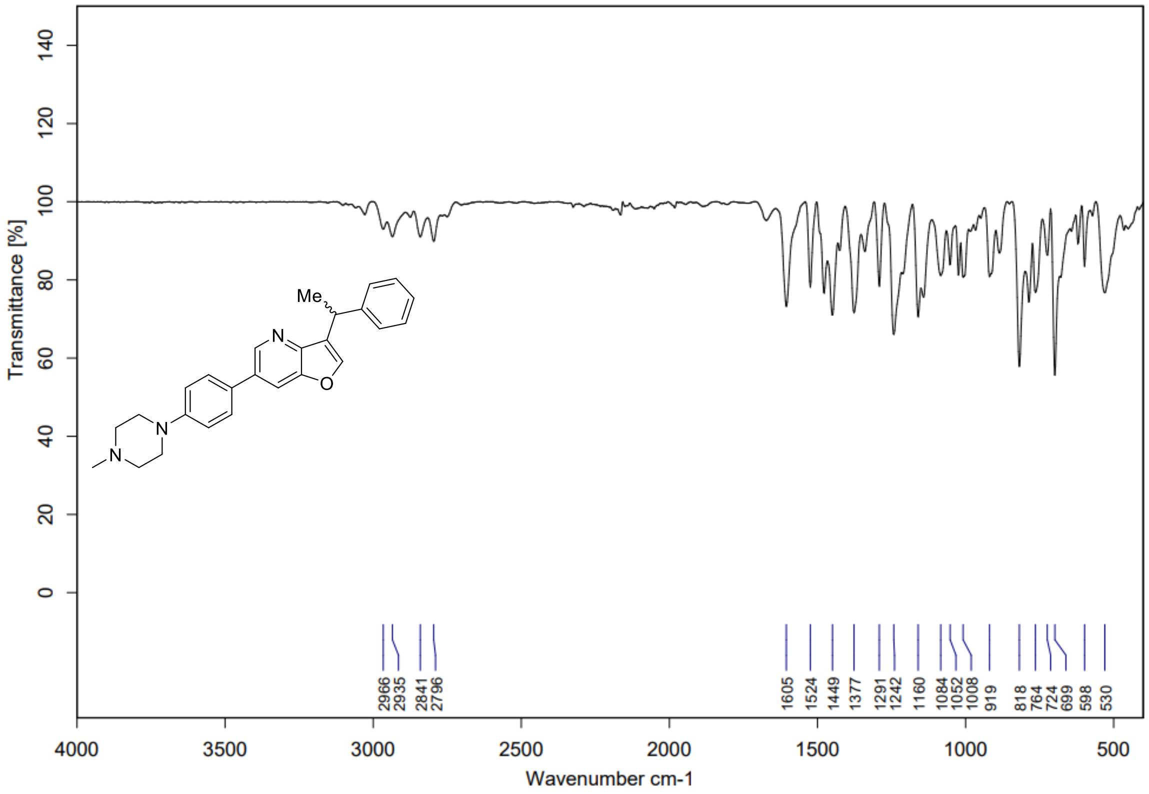

HRMS spectrum of **86.**

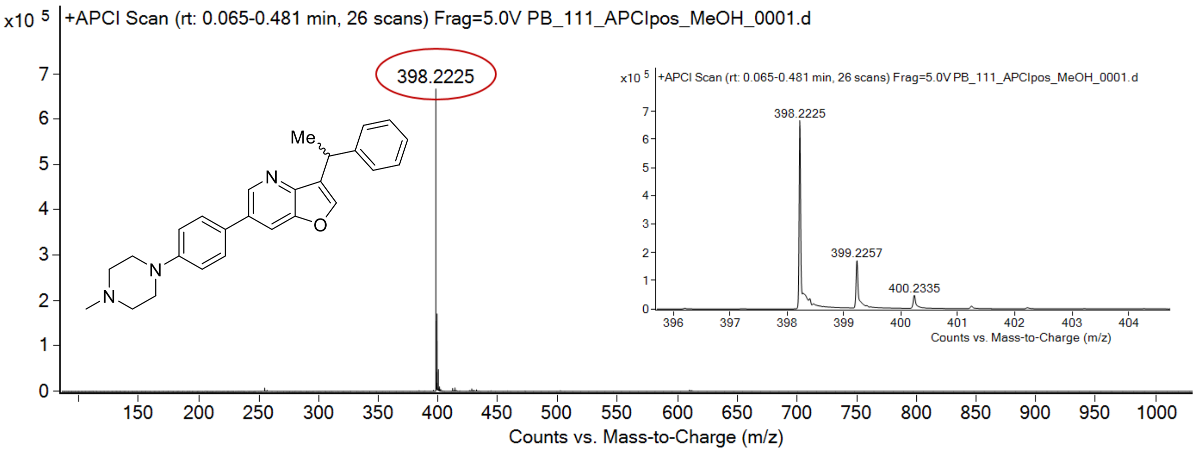

^{1}H (500 MHz) and ^{13}C NMR (126 MHz) spectra of **87** in chloroform-*d*.

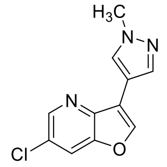

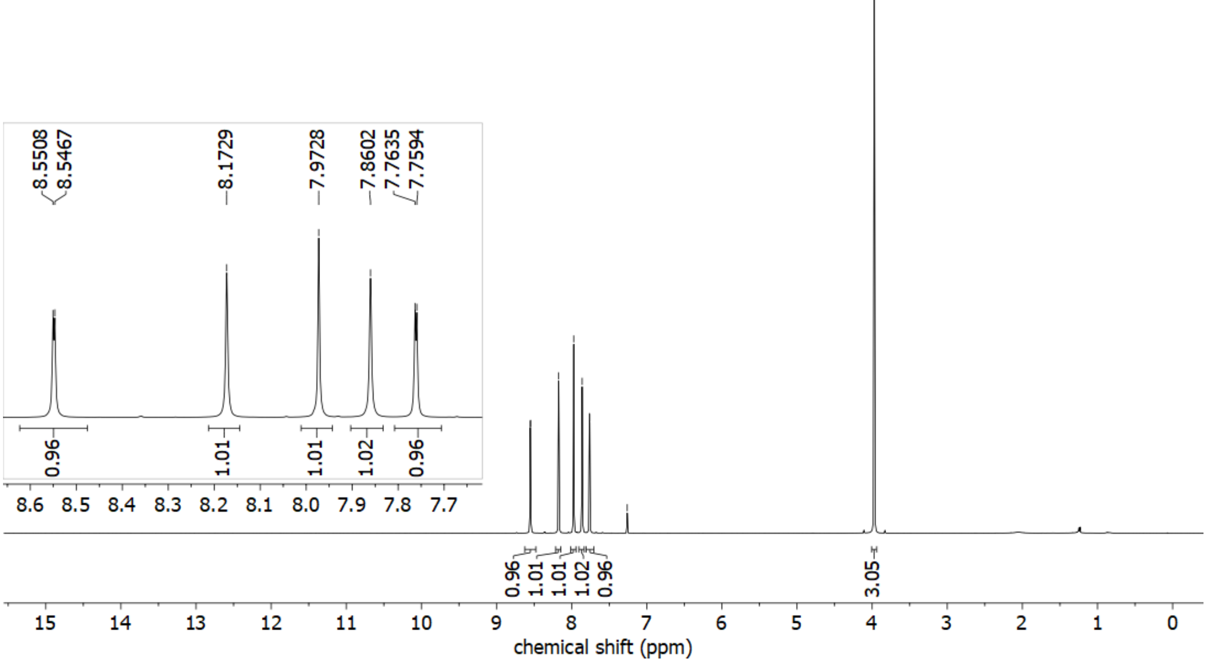

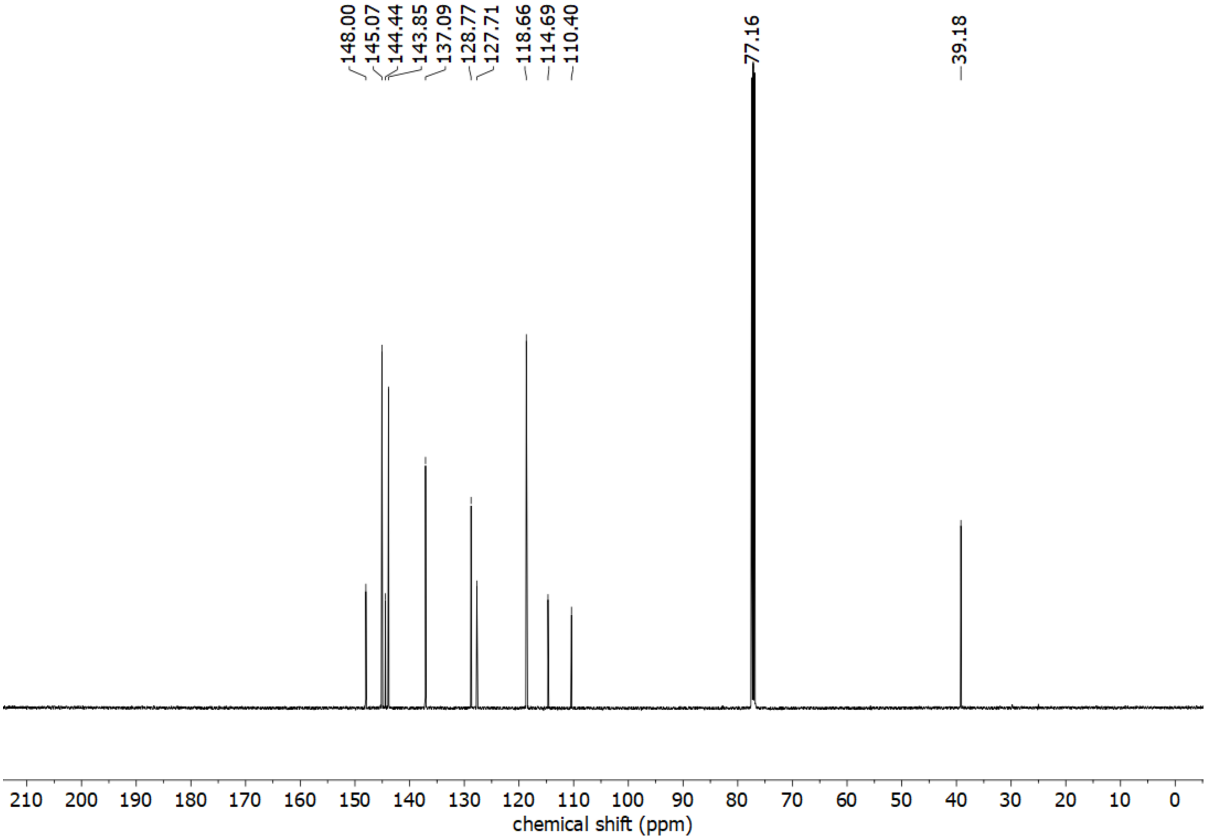

FT-IR spectrum (neat) of **87.**

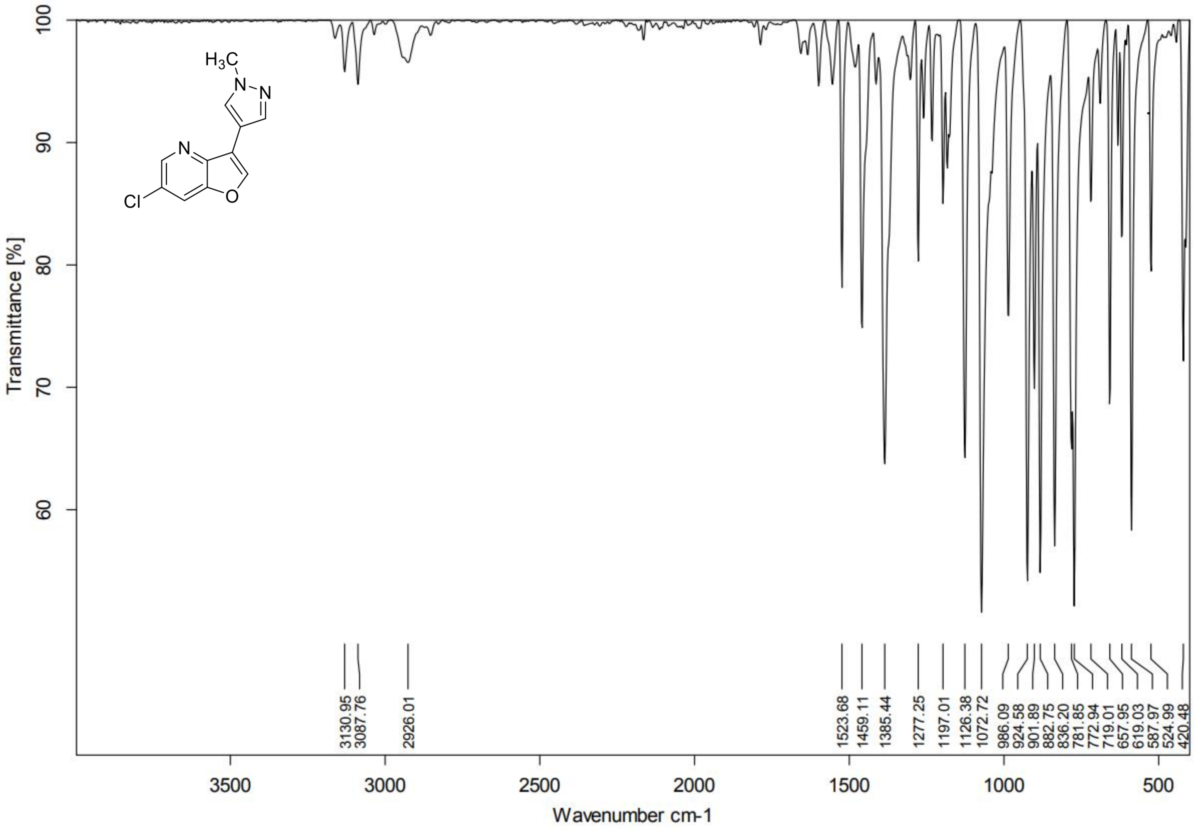

HRMS spectrum of **87.**

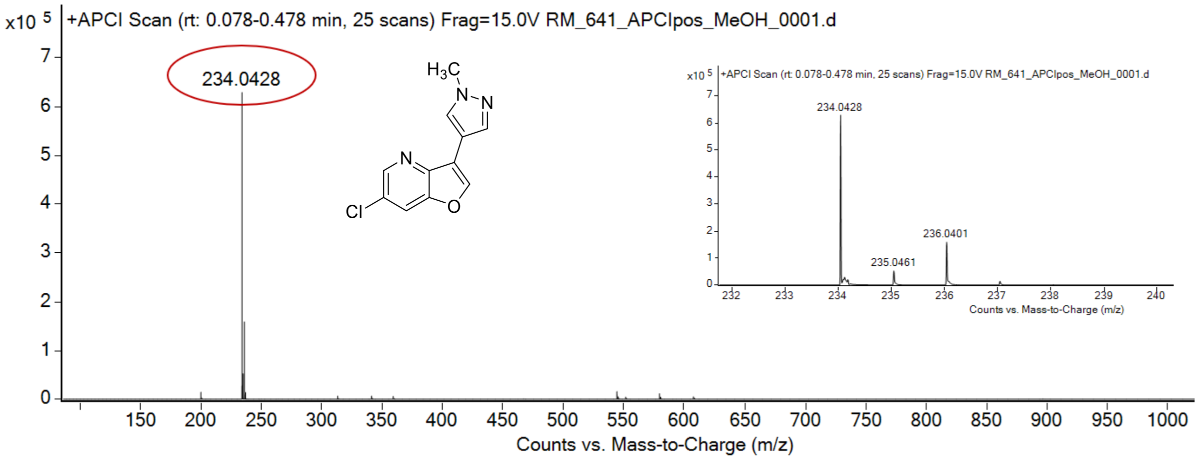

^{1}H (500 MHz) and ^{13}C NMR (126 MHz) spectra of **88** in chloroform-*d*.

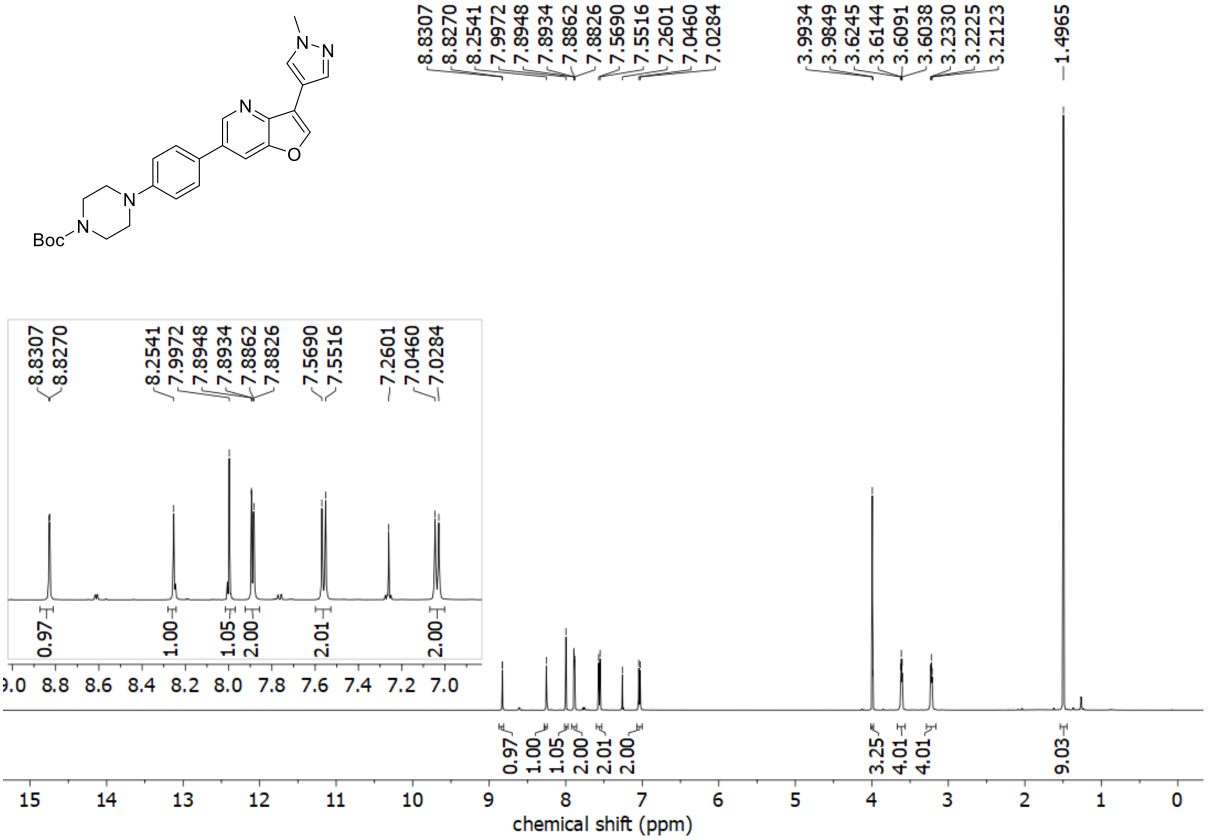

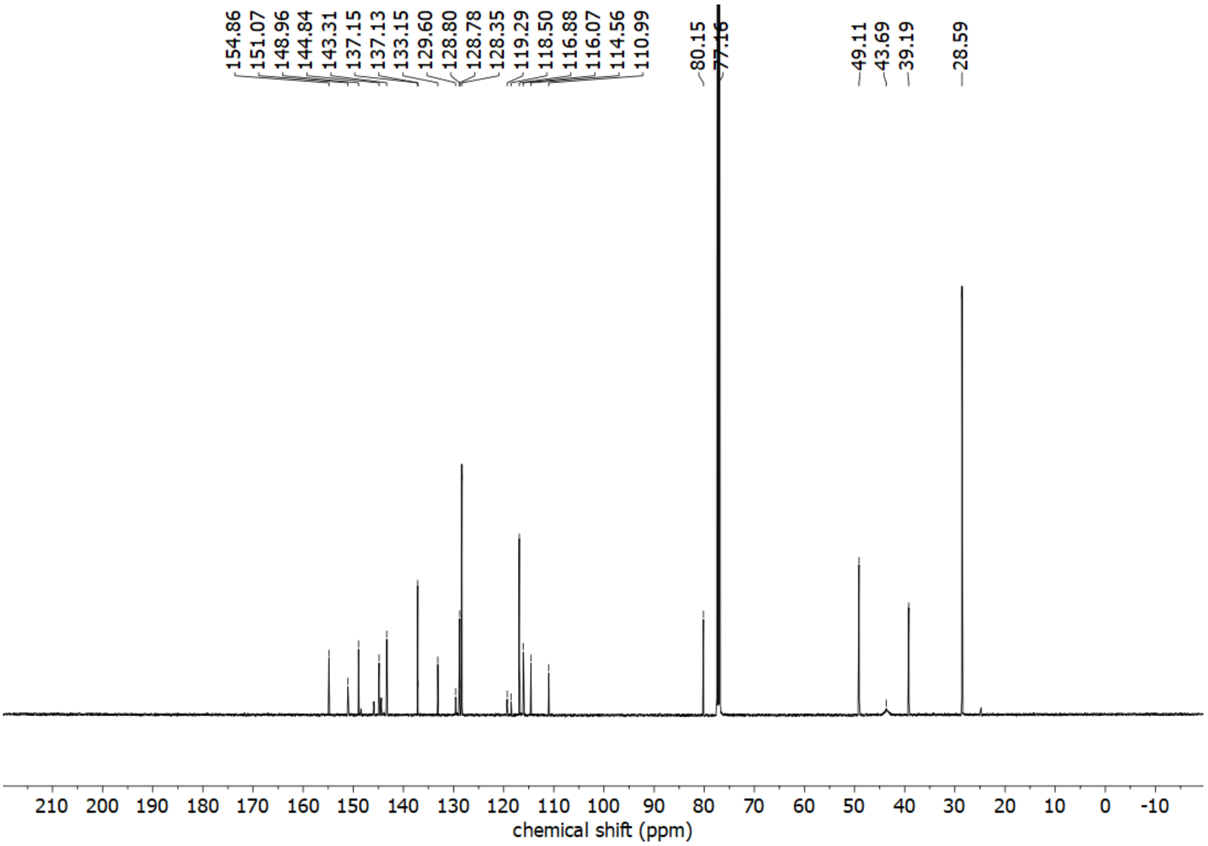

FT-IR spectrum (neat) of **88.**

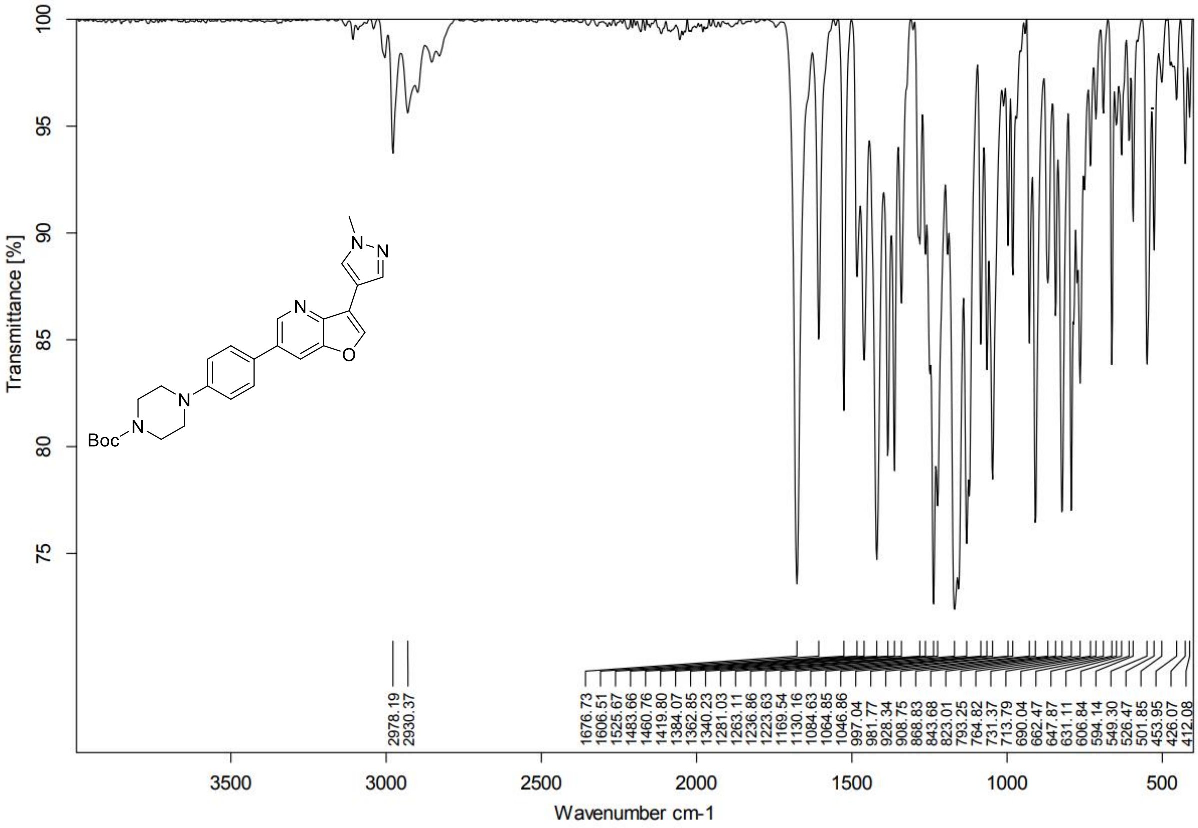

HRMS spectrum of **88.**

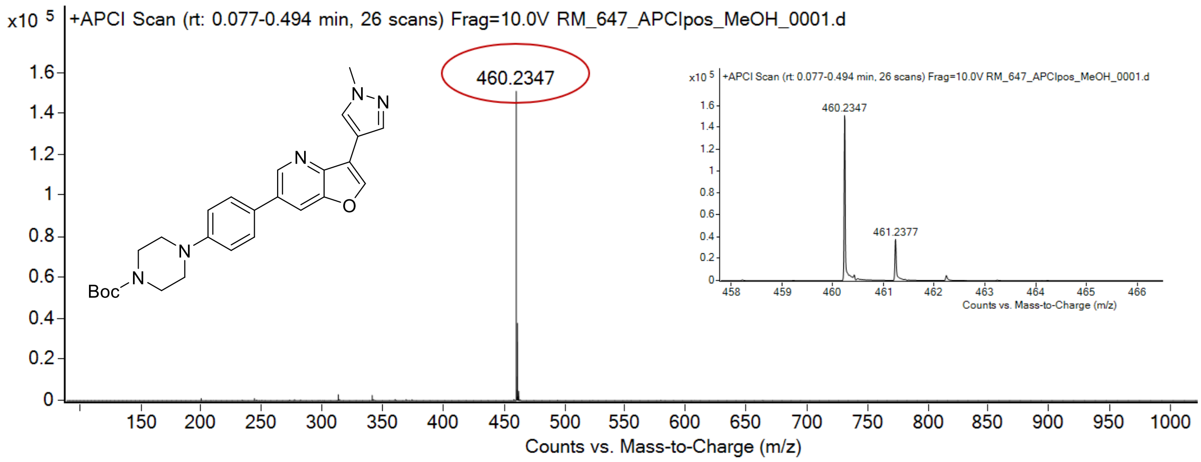

^{1}H (500 MHz) and ^{13}C NMR (126 MHz) spectra of **89** in chloroform-*d*.

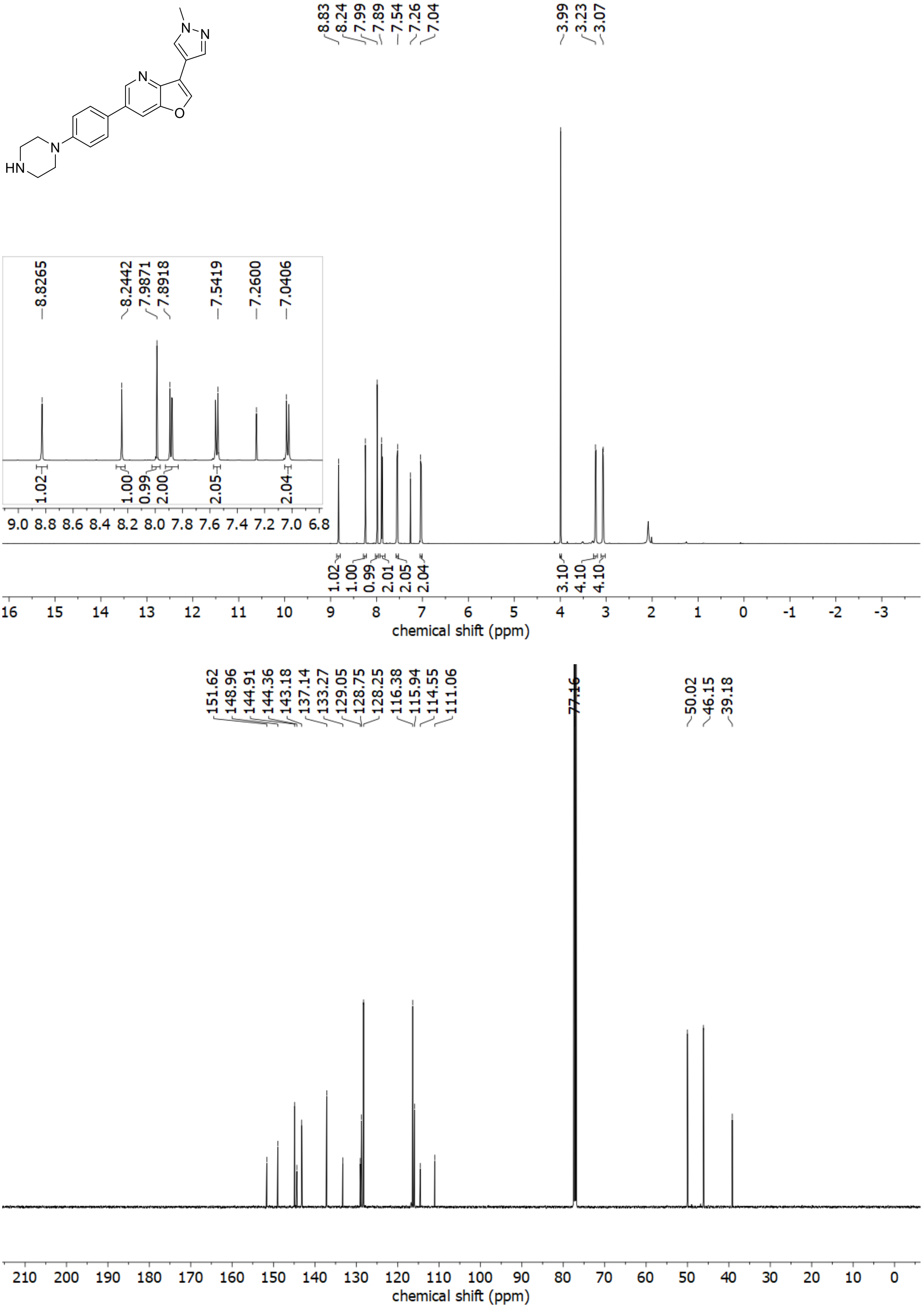

FT-IR spectrum (neat) of **89.**

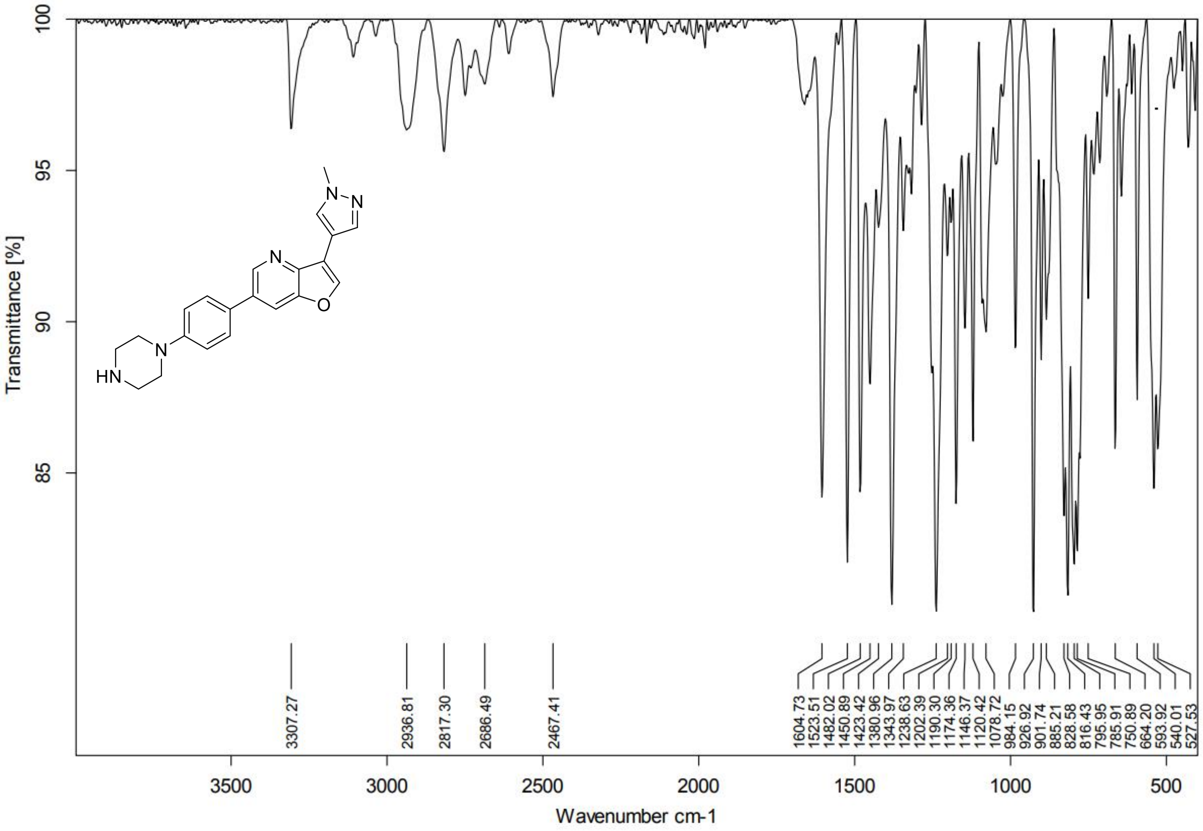

HRMS spectrum of **89.**

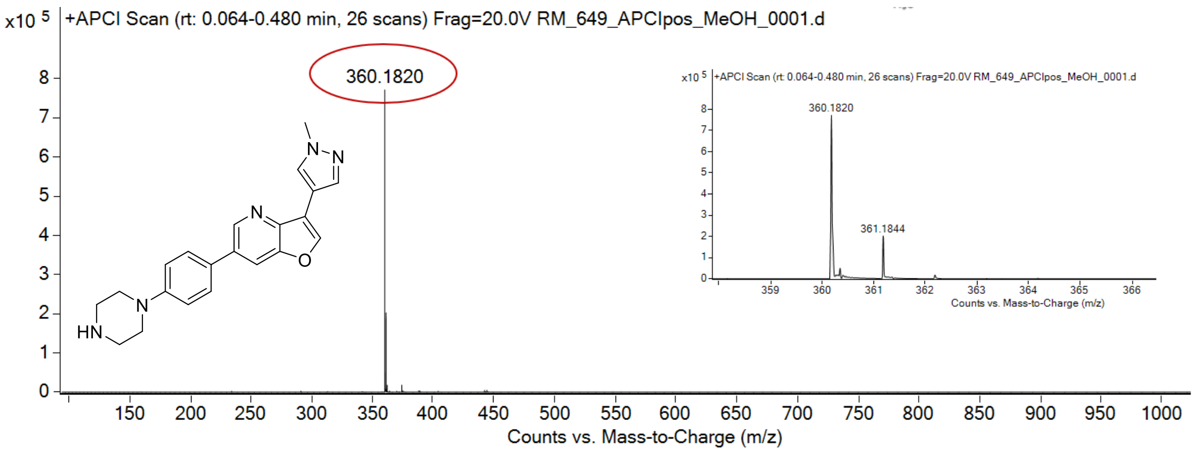

^{1}H (500 MHz) and ^{13}C NMR (126 MHz) spectra of **90** in chloroform-*d*.

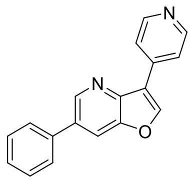

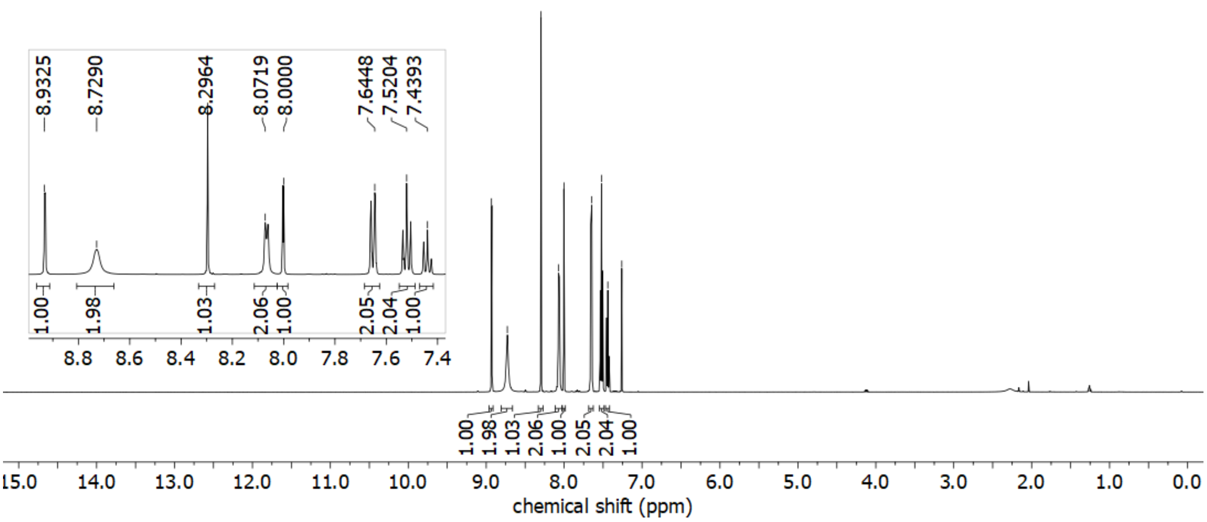

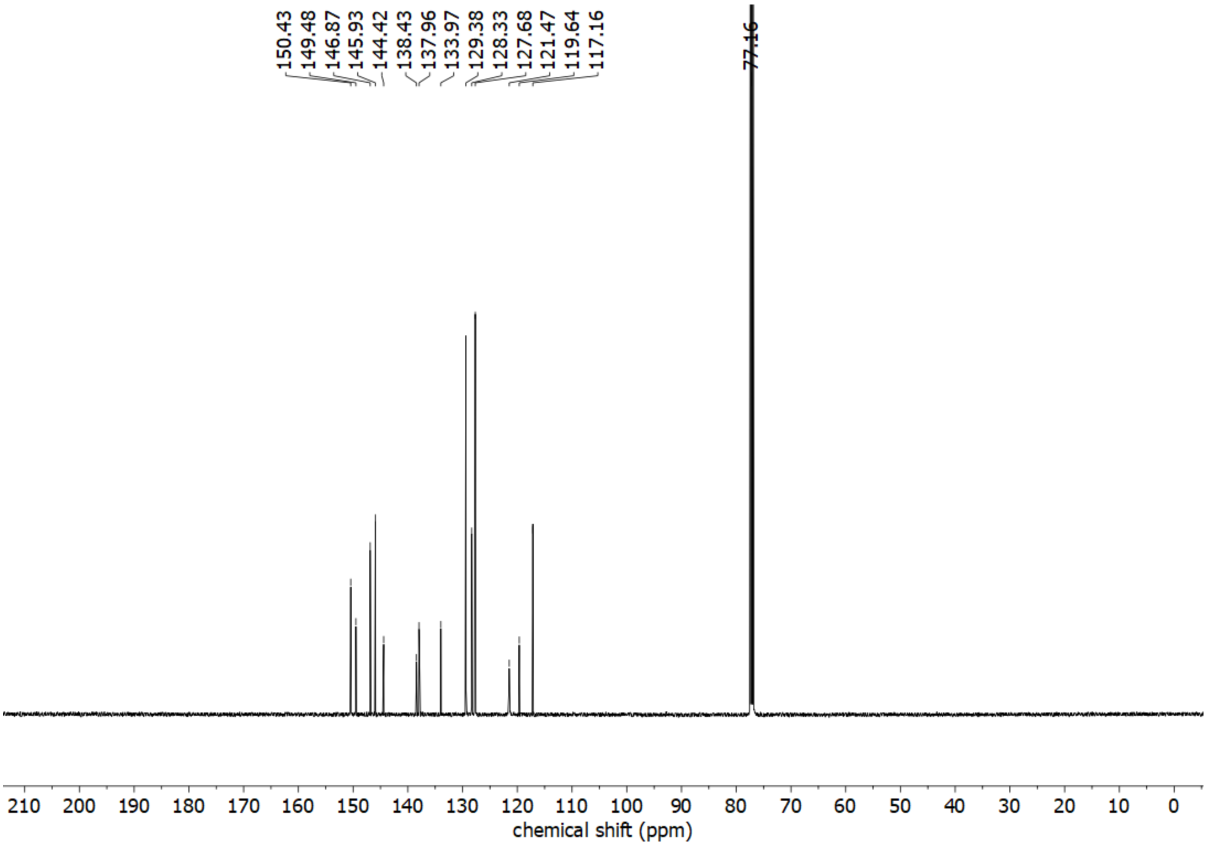

FT-IR spectrum (neat) of **90.**

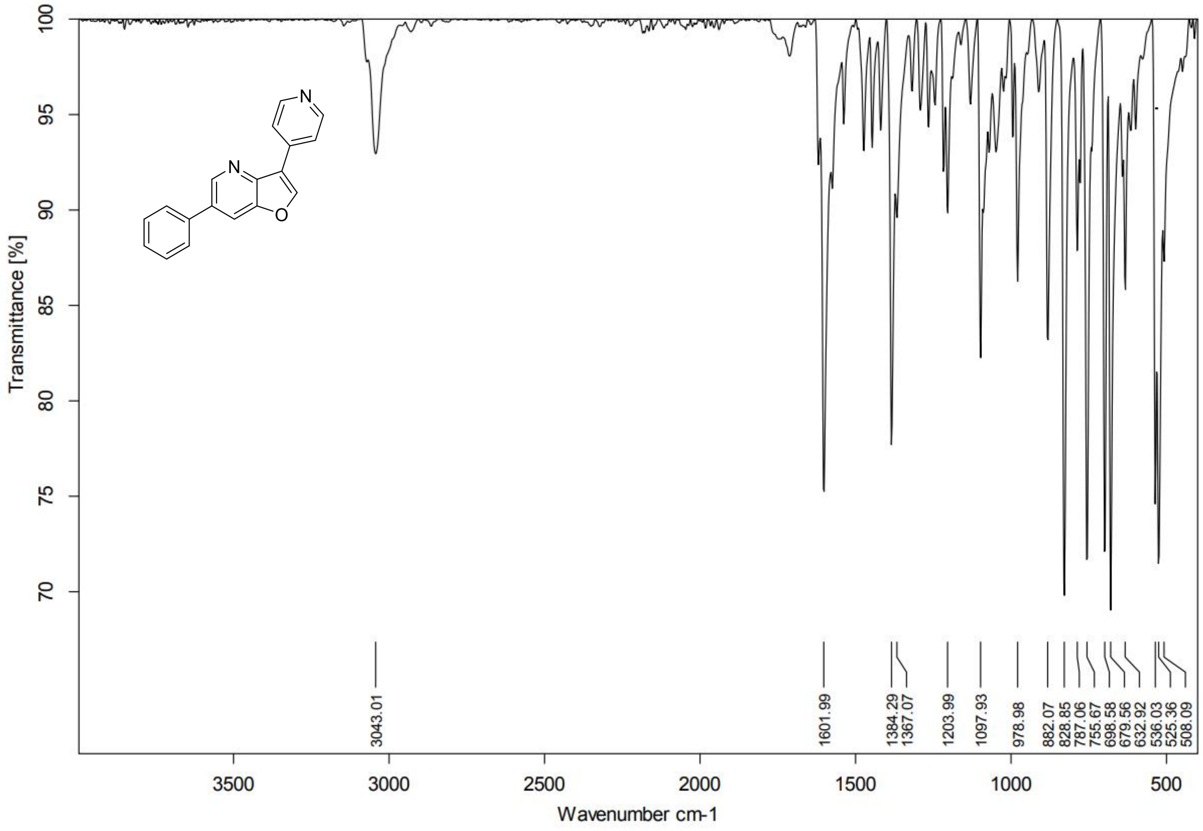

HRMS spectrum of **90.**

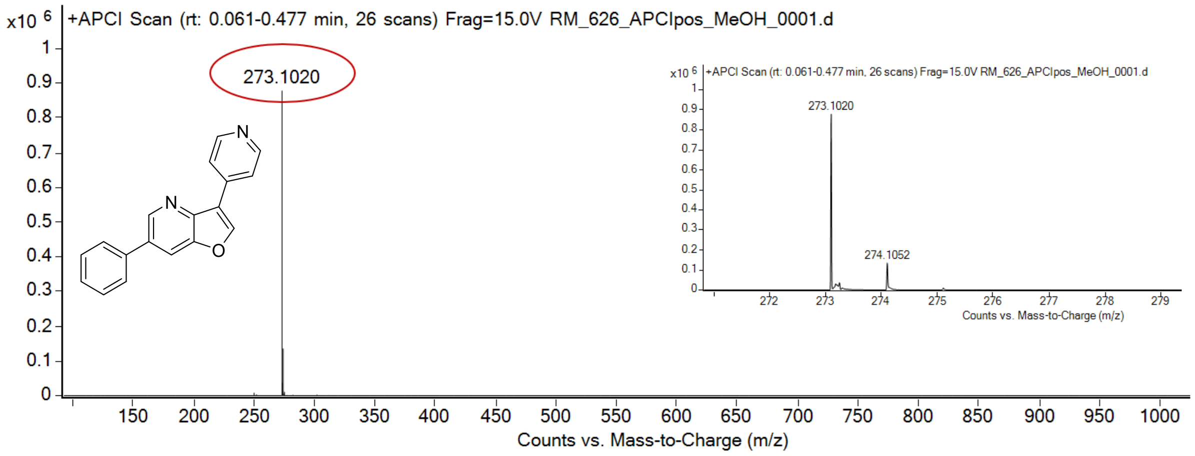

^{1}H (500 MHz) and ^{13}C NMR (126 MHz) spectra of **91** in chloroform-*d*.

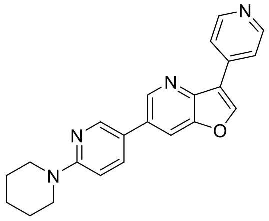

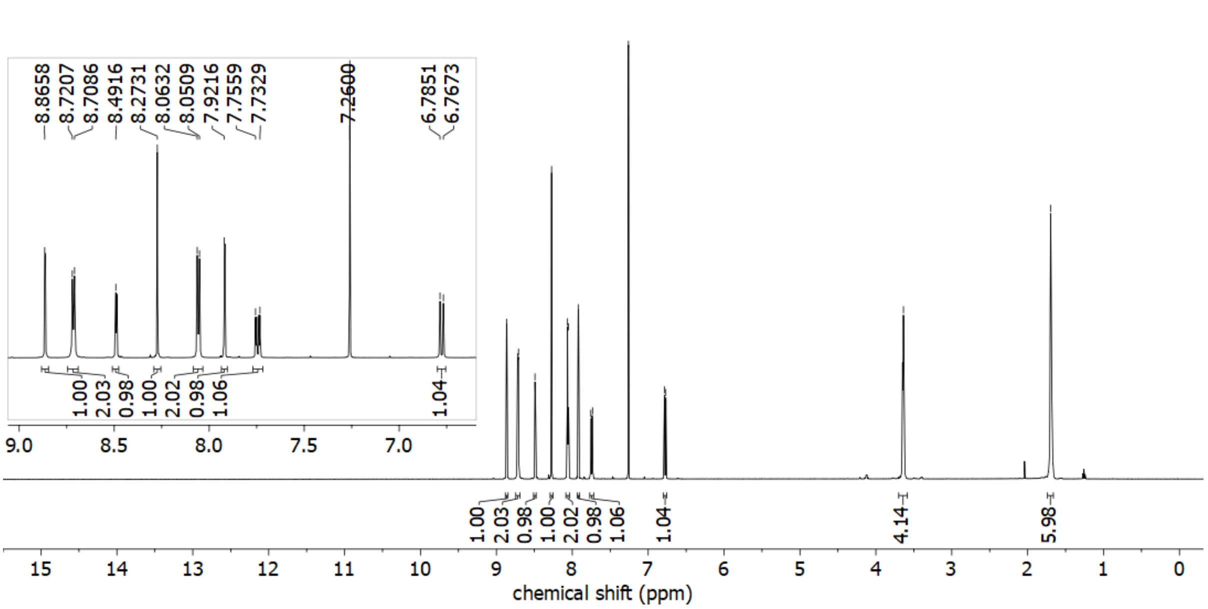

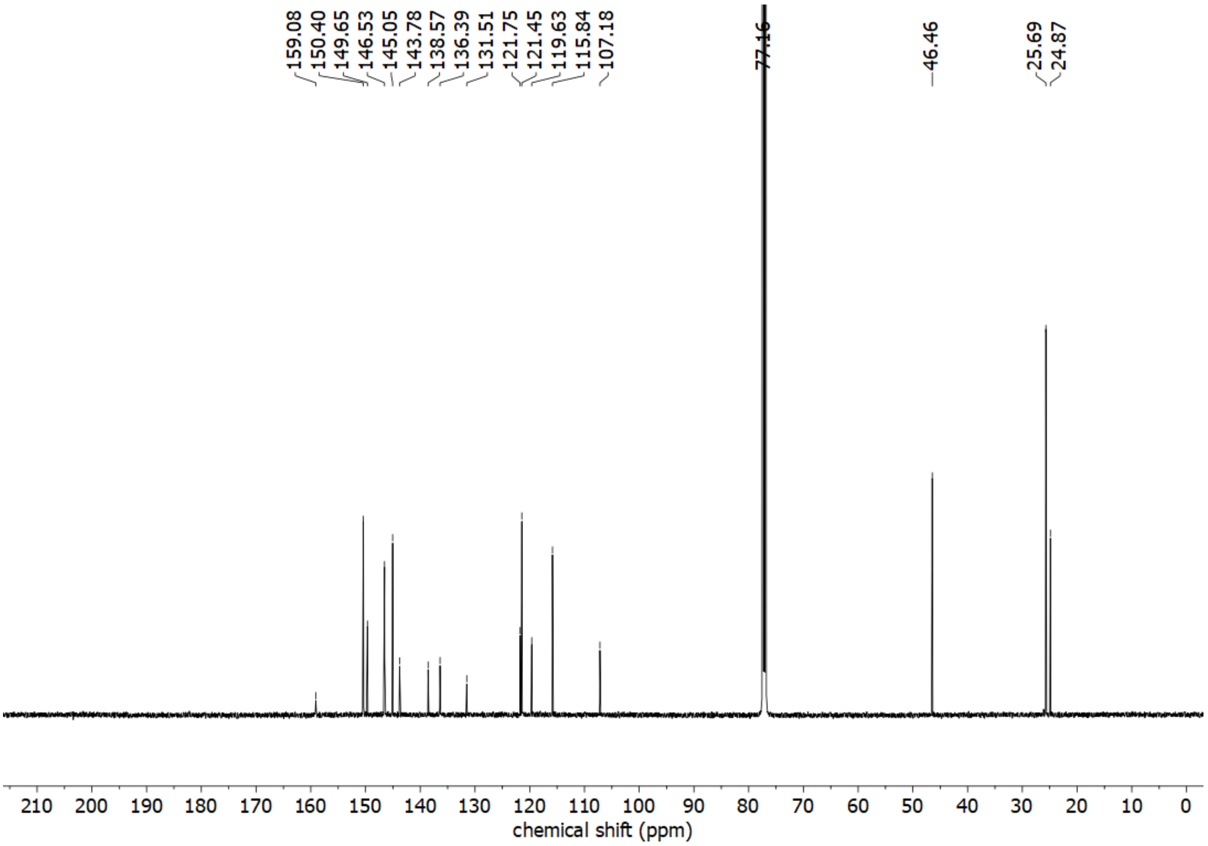

FT-IR spectrum (neat) of **91.**

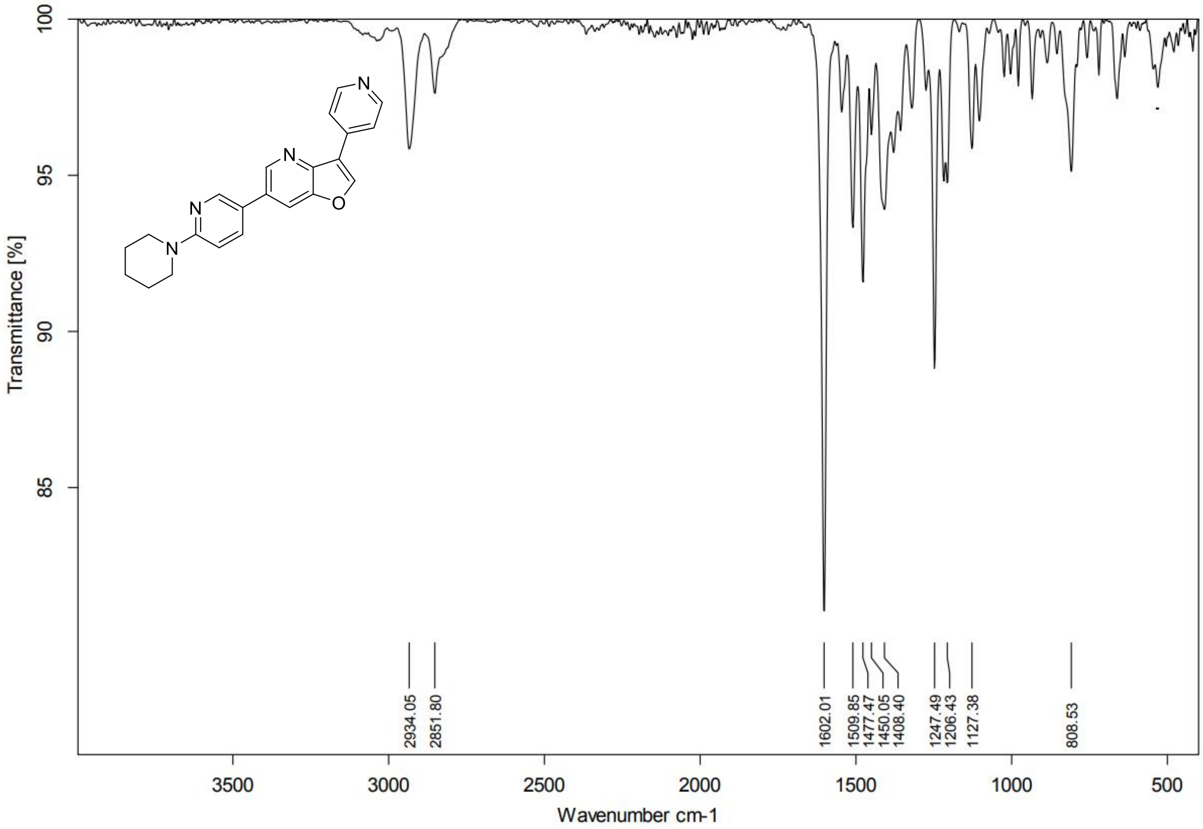

HRMS spectrum of **91.**

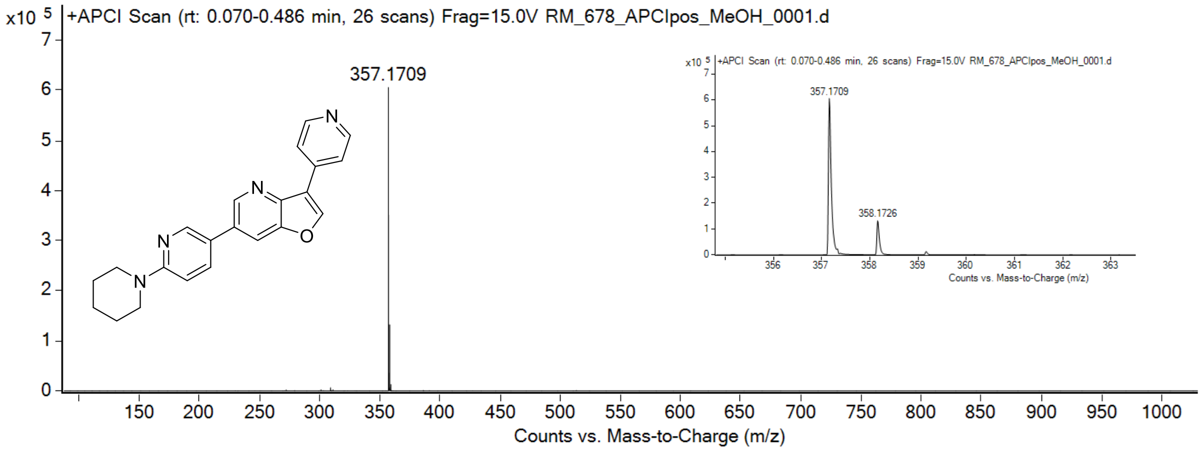

^{1}H (500 MHz) and ^{13}C NMR (126 MHz) spectra of **92** in chloroform-*d*.

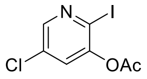

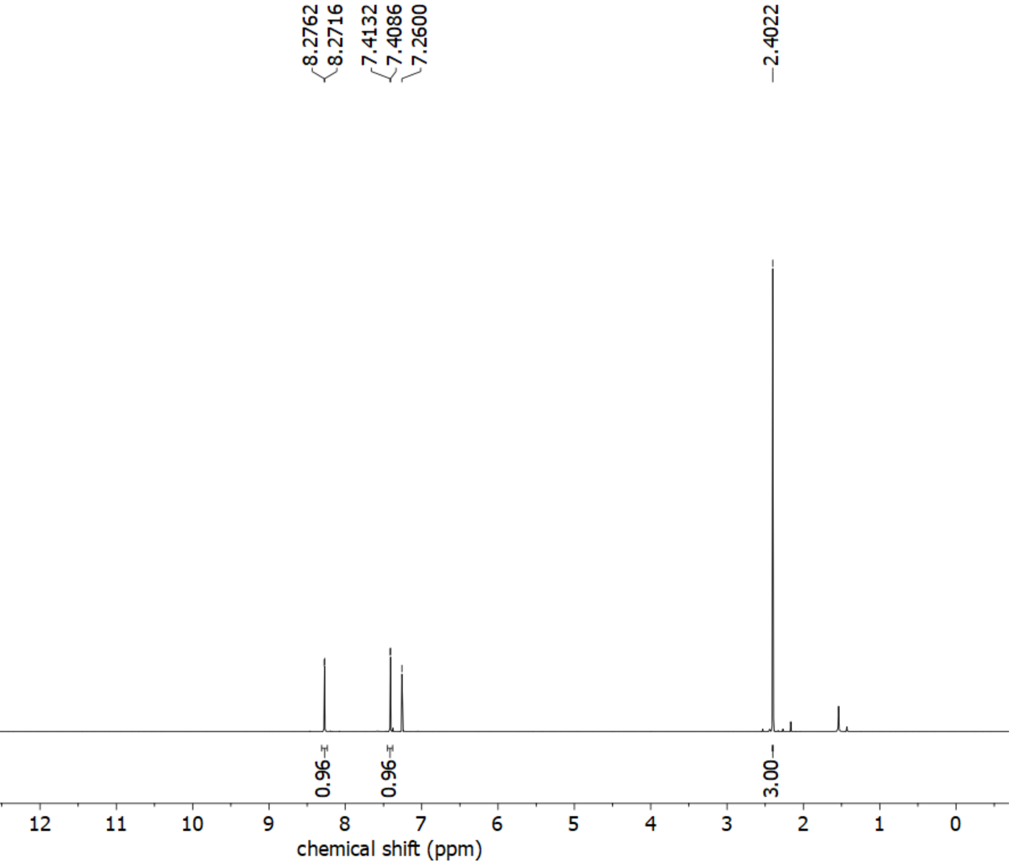

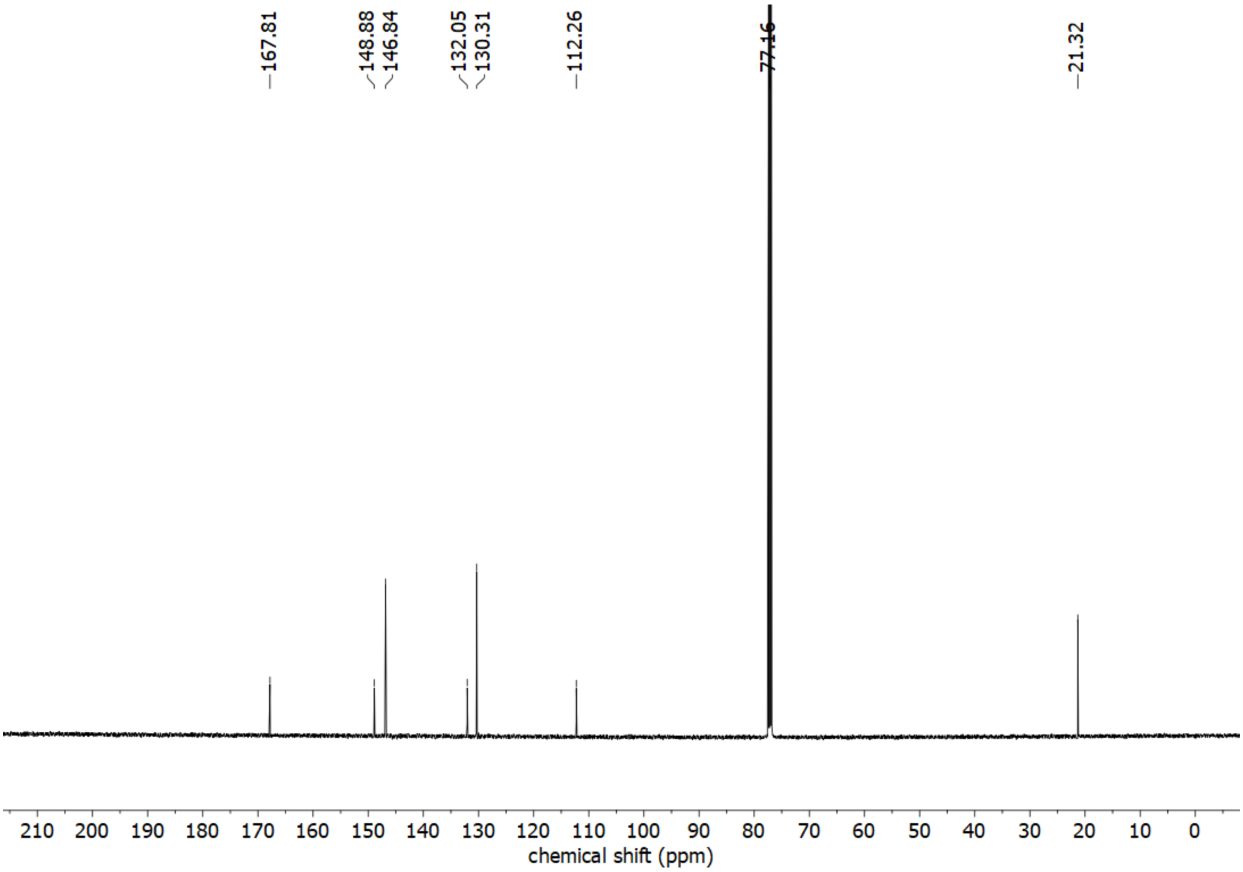

FT-IR spectrum (neat) of **92.**

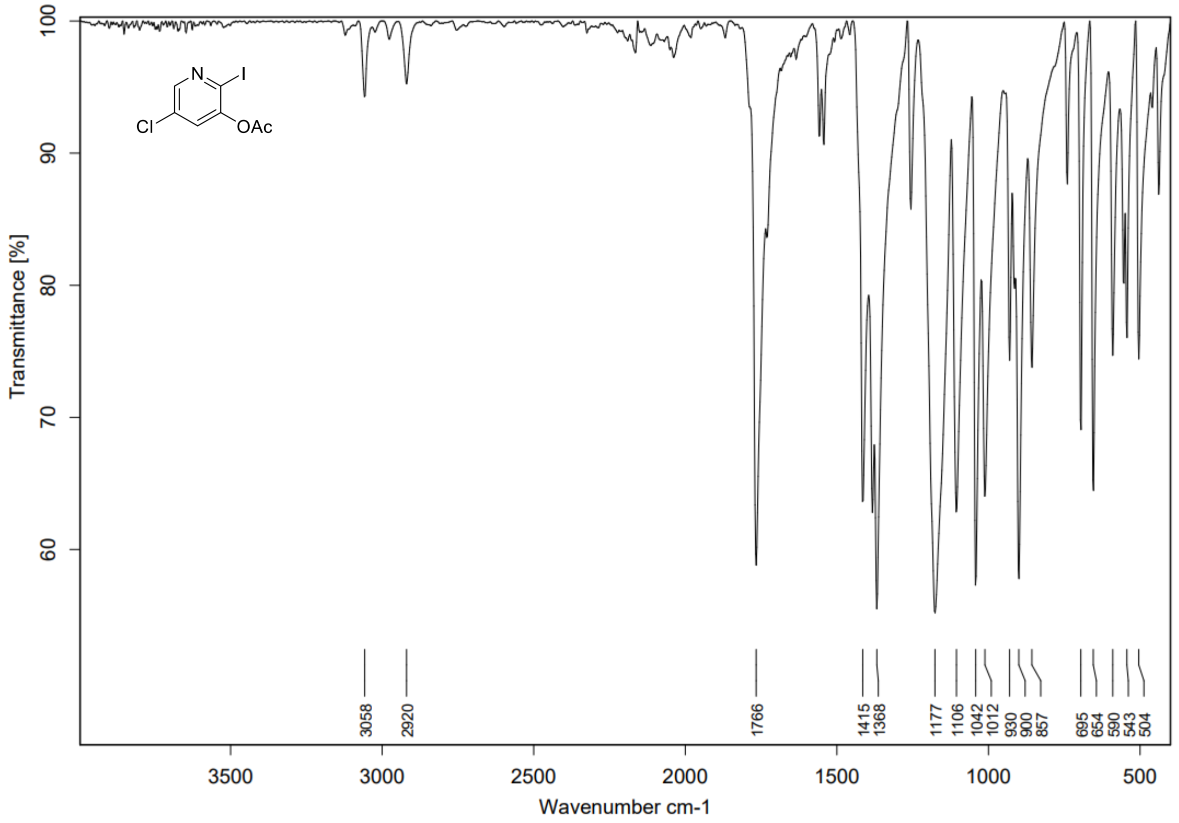

HRMS spectrum of **92.**

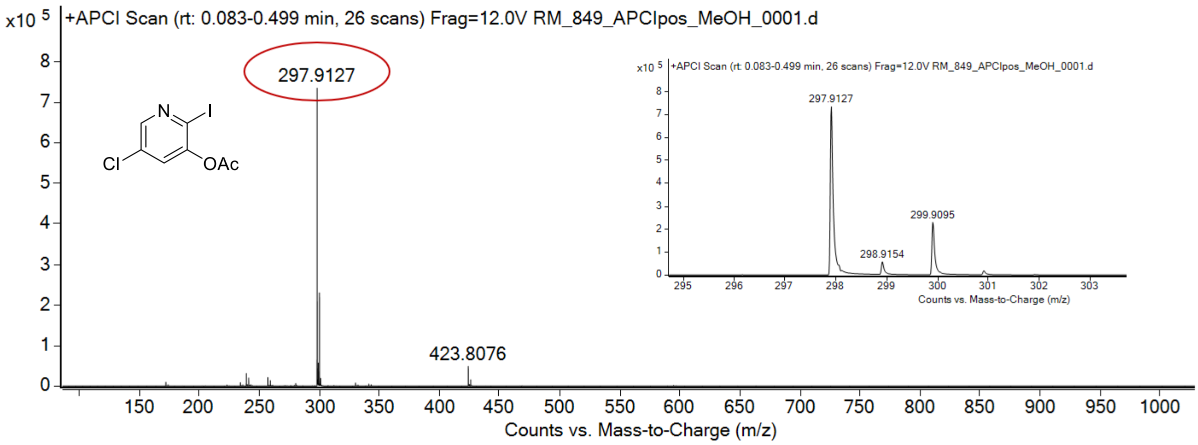

^{1}H (500 MHz) and ^{13}C NMR (126 MHz) spectra of **93** in chloroform-*d*.

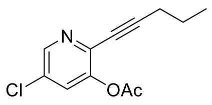

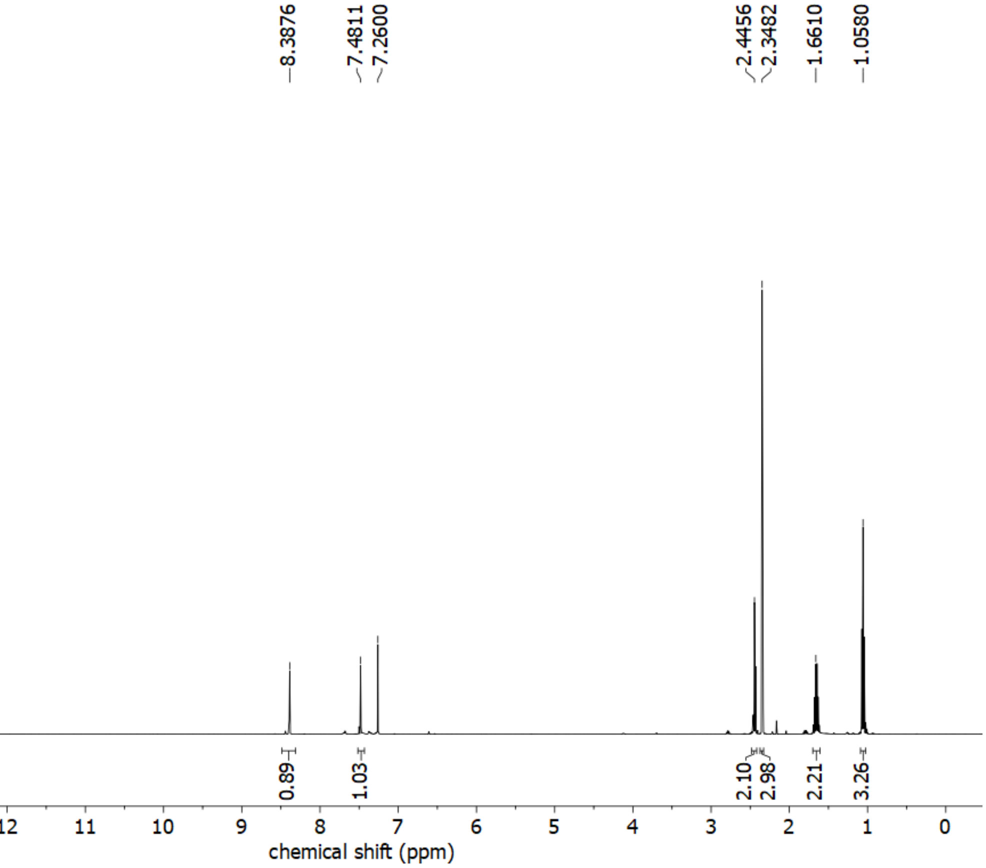

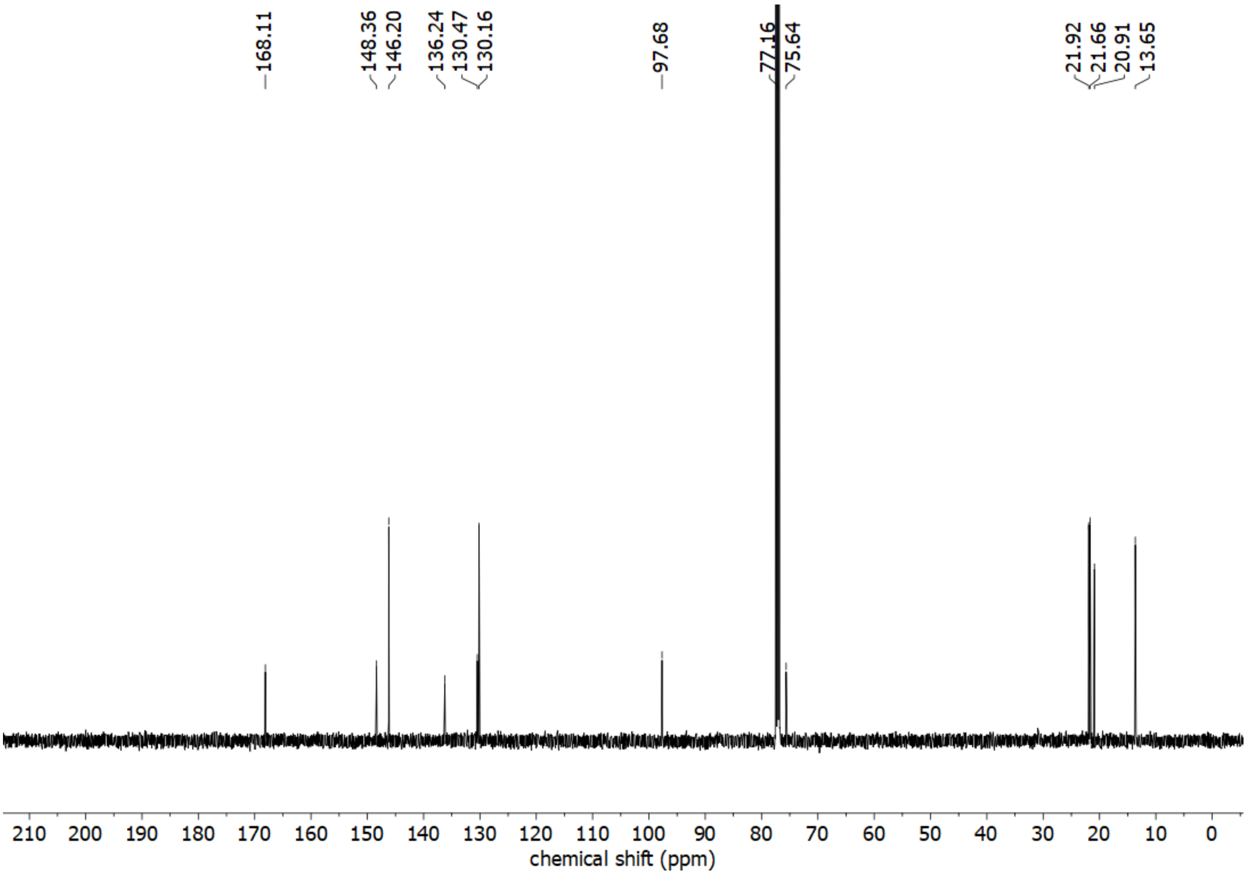

FT-IR spectrum (neat) of **93.**

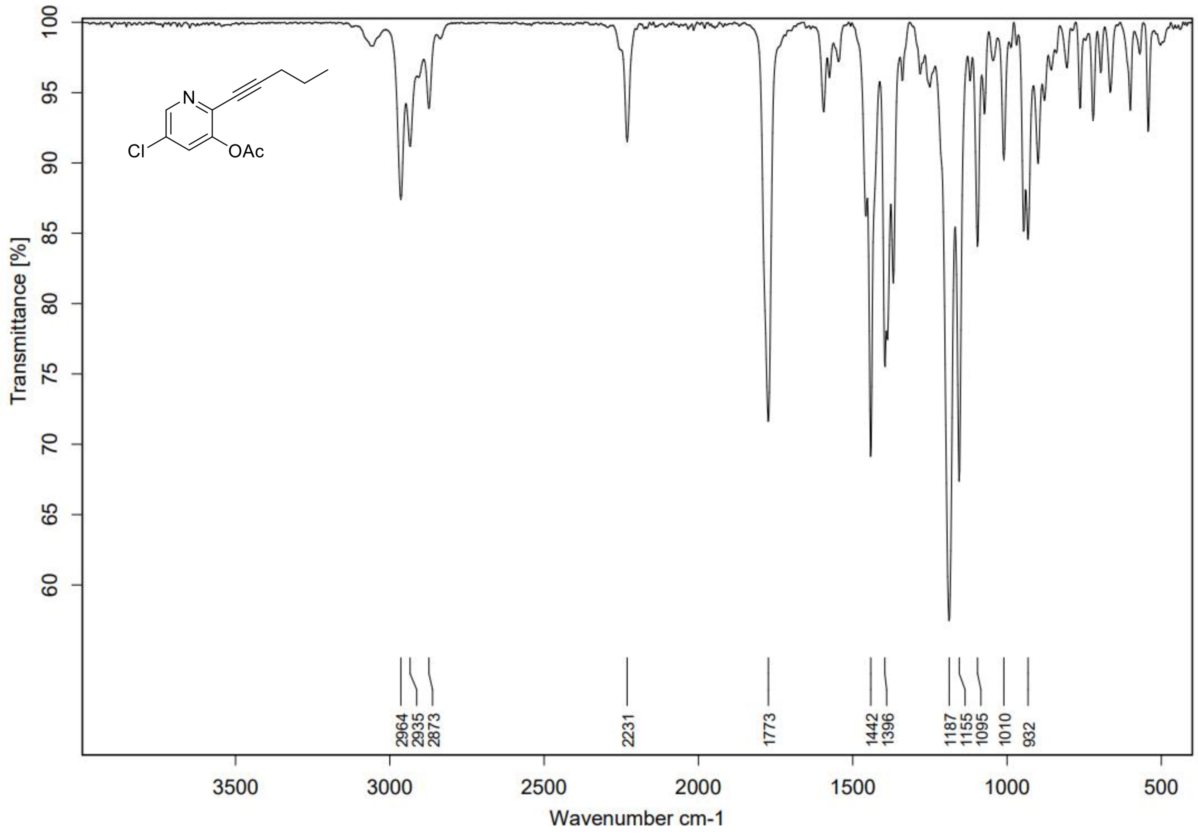

HRMS spectrum of **93.**

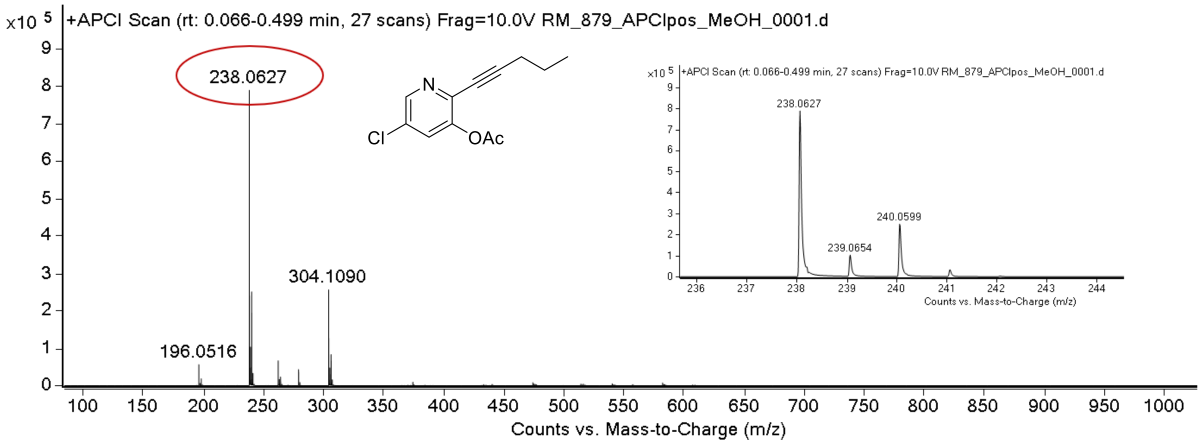

^{1}H (500 MHz) and ^{13}C NMR (126 MHz) spectra of **94** in chloroform-*d*.

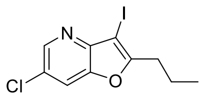

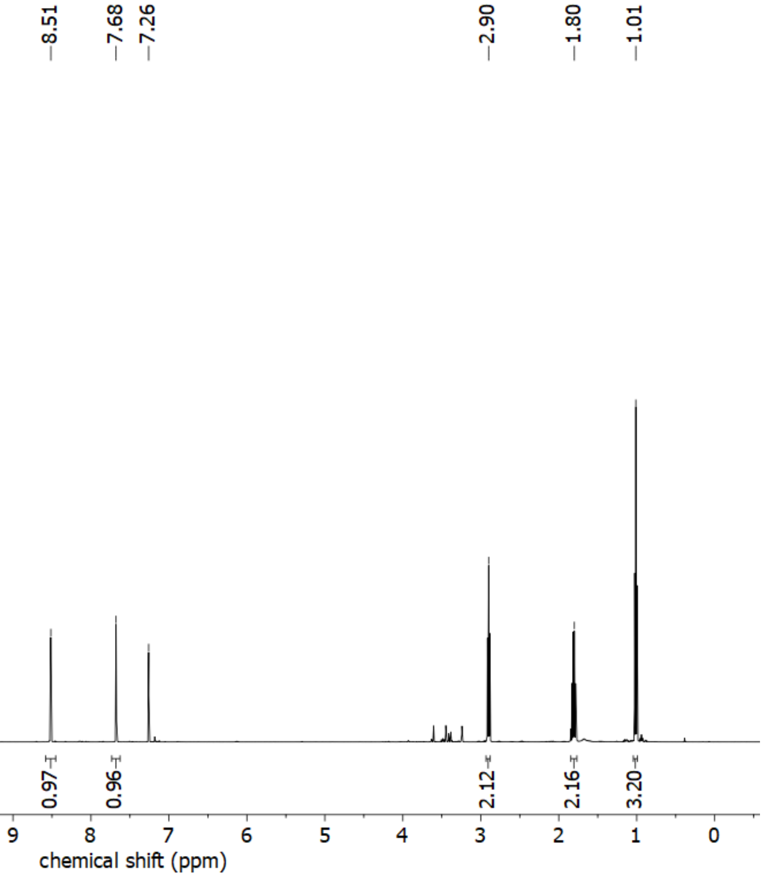

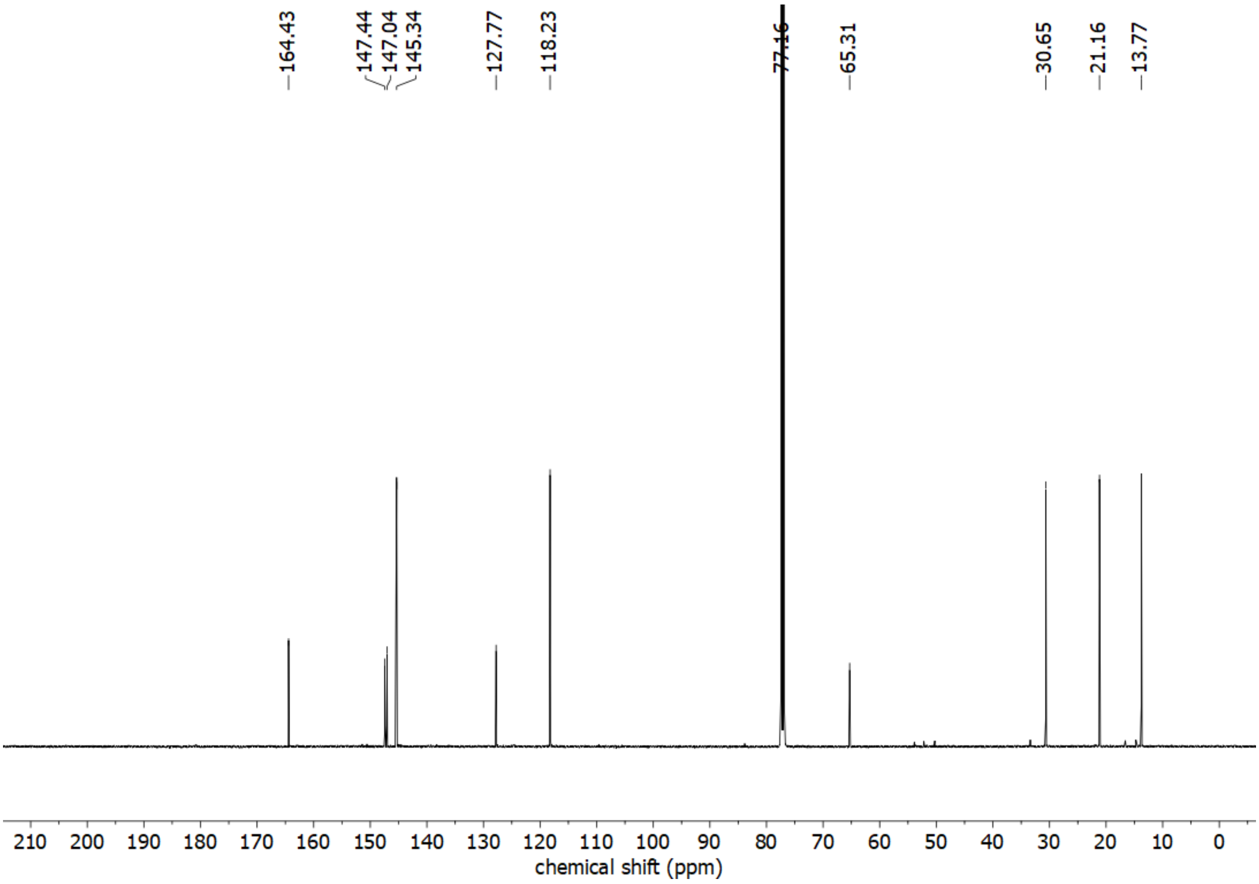

FT-IR spectrum (neat) of **94.**

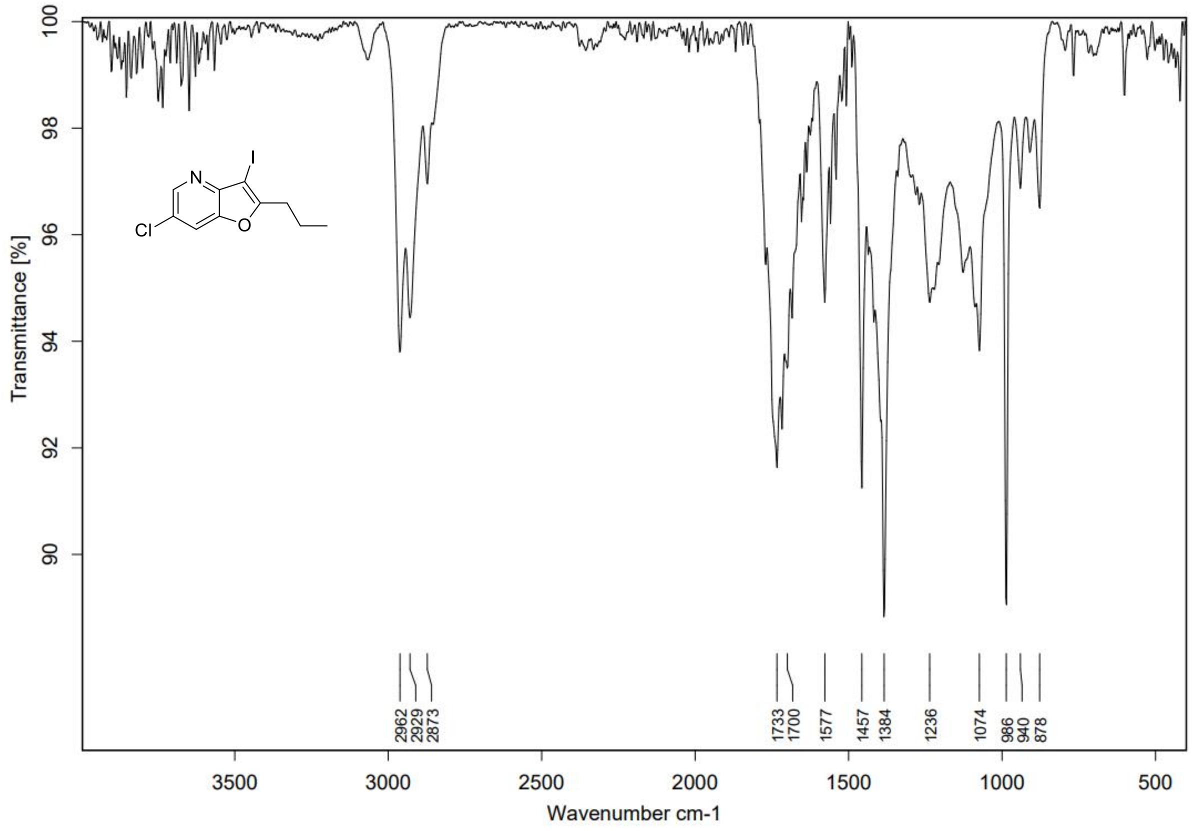

HRMS spectrum of **94.**

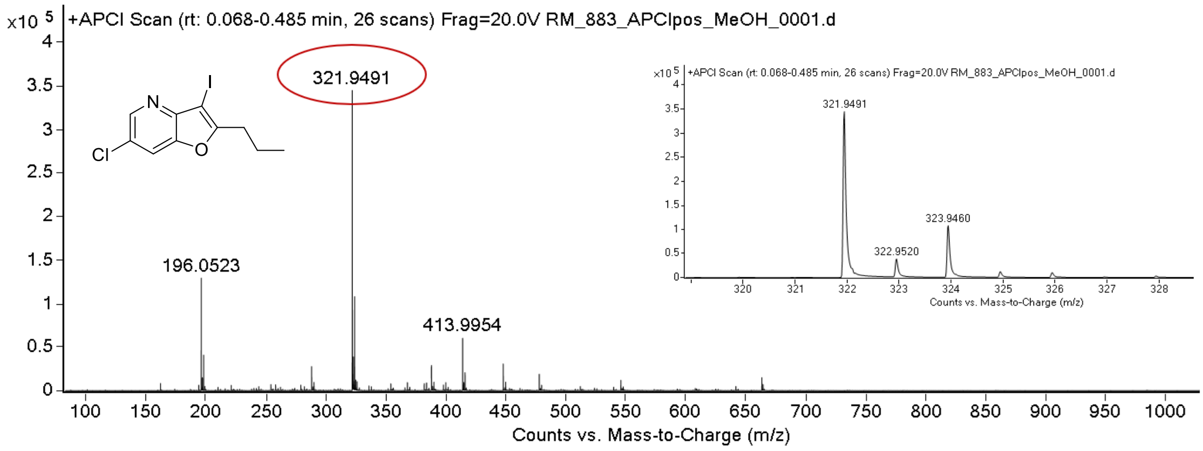

^{1}H (500 MHz) and ^{13}C NMR (126 MHz) spectra of **95** in chloroform-*d*.

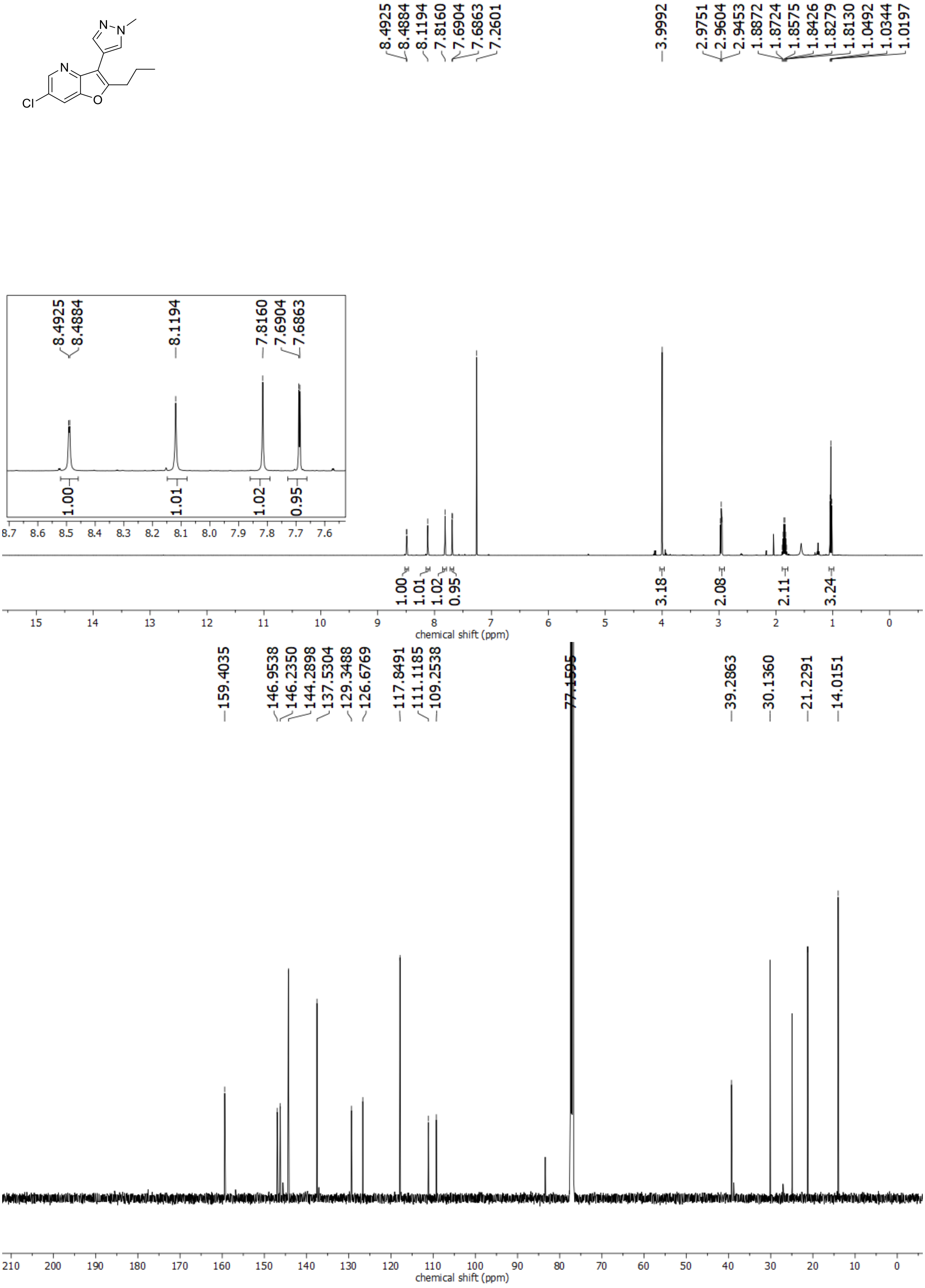

FT-IR spectrum (neat) of **95.**

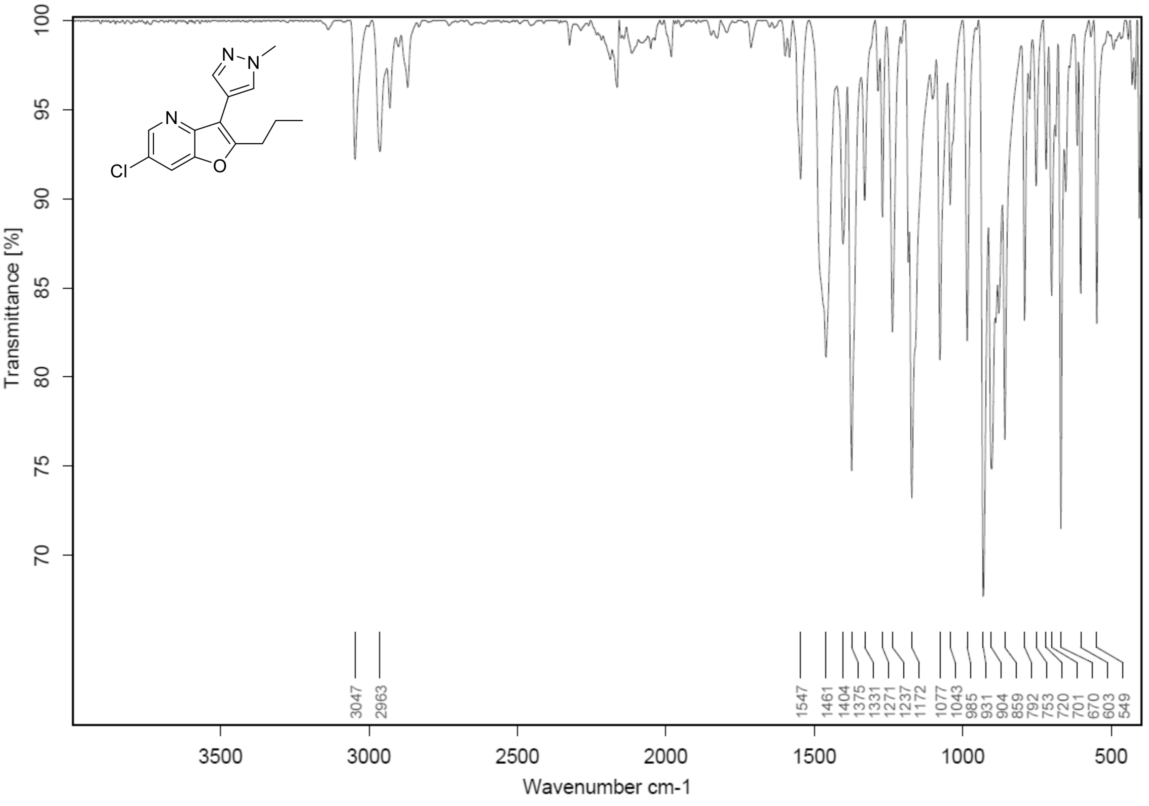

HRMS spectrum of **95.**

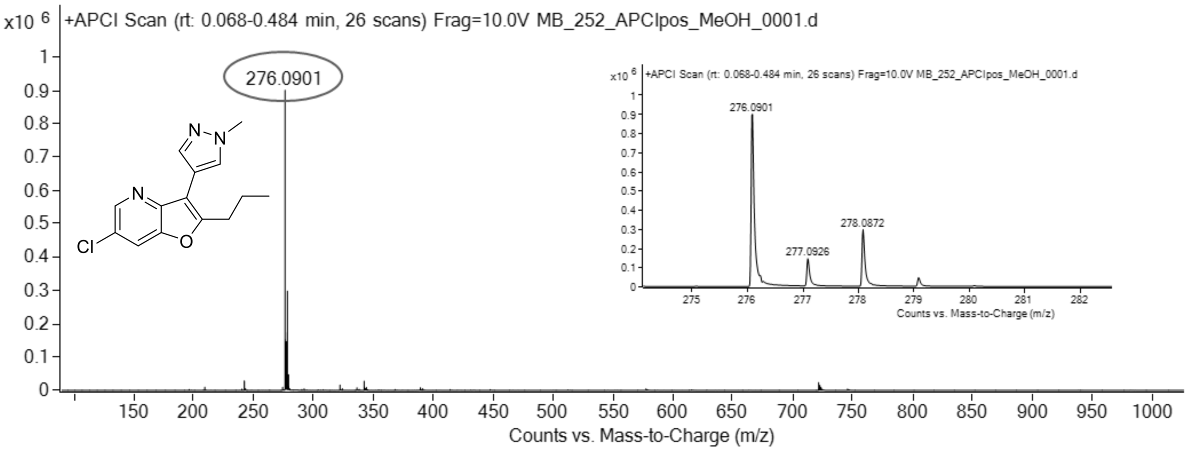

^{1}H (500 MHz) and ^{13}C NMR (126 MHz) spectra of **96** in chloroform-*d*.

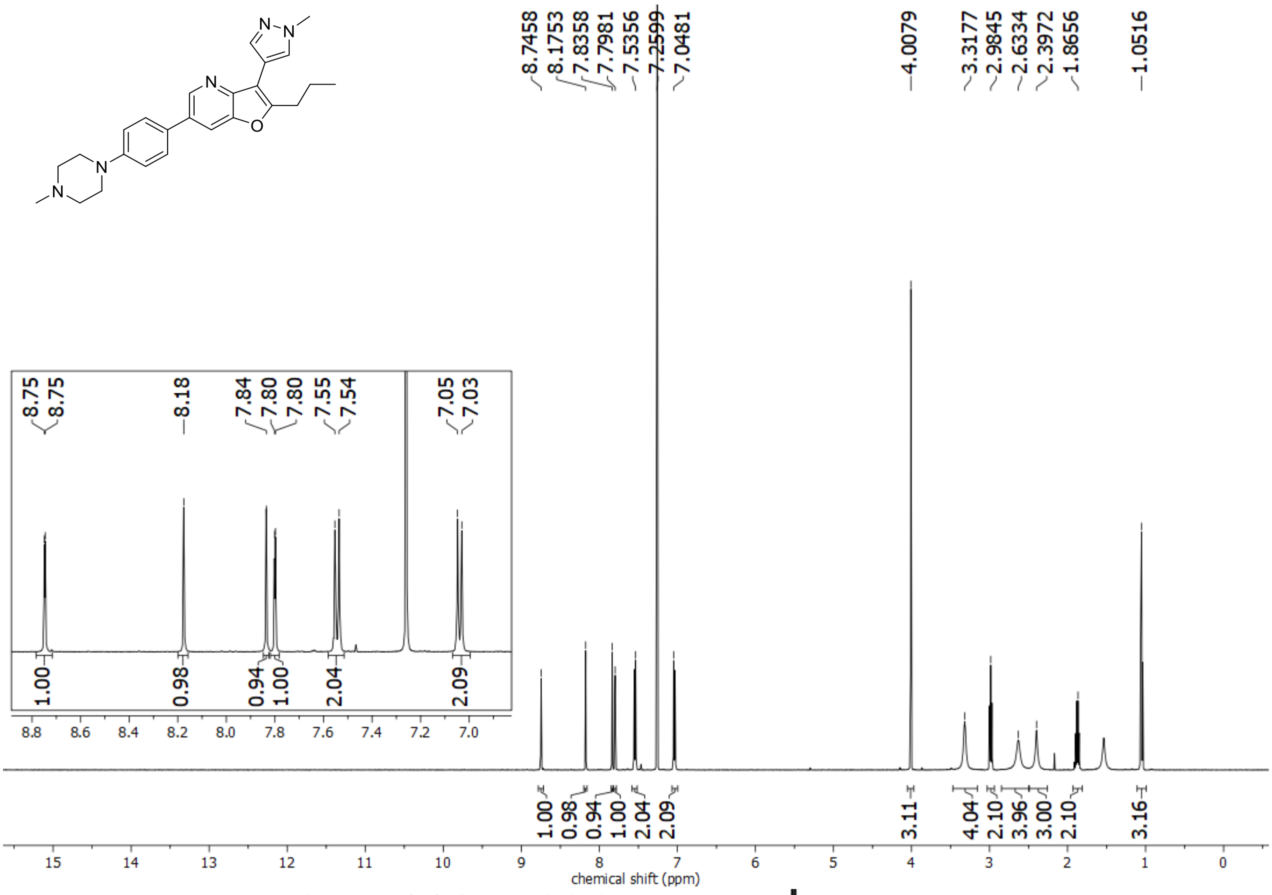

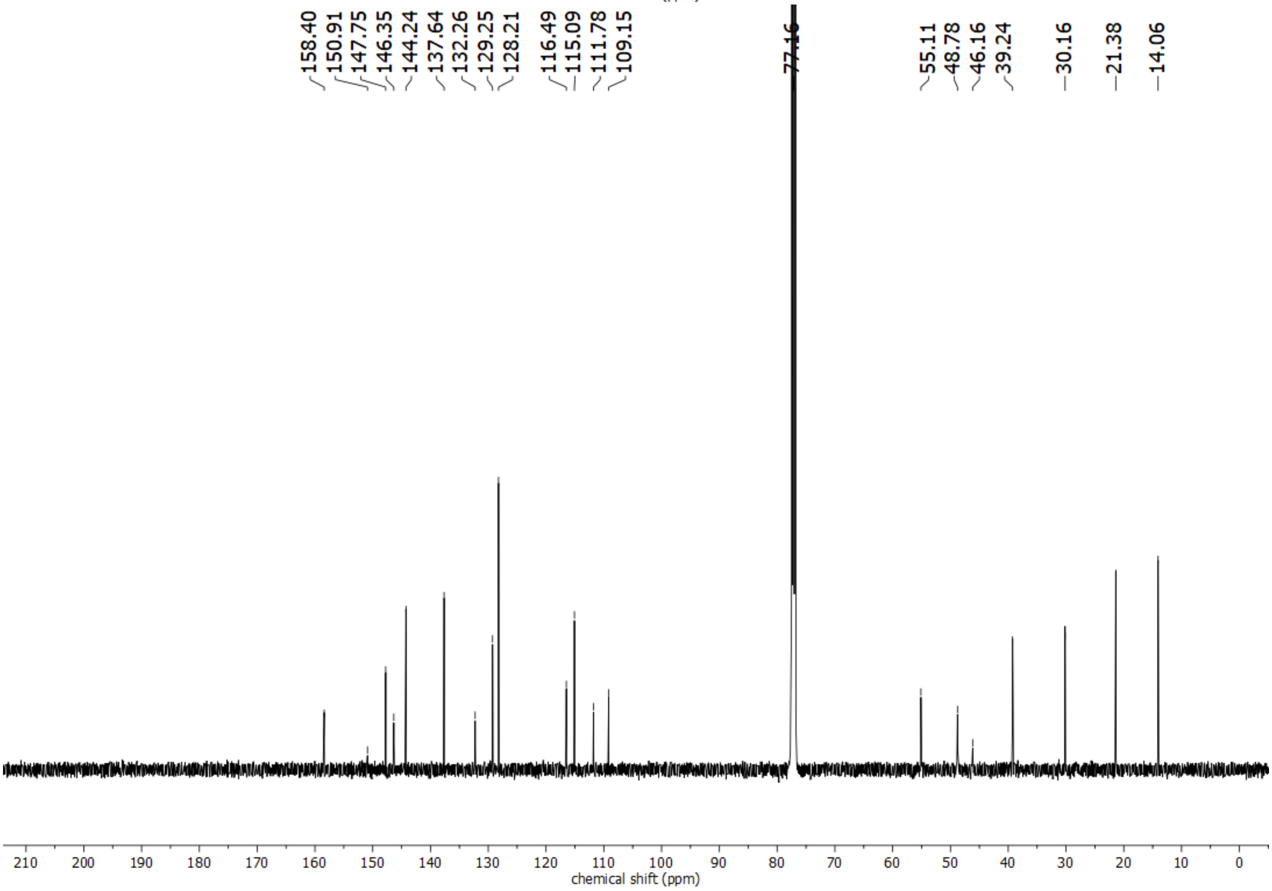

FT-IR spectrum (neat) of **96.**

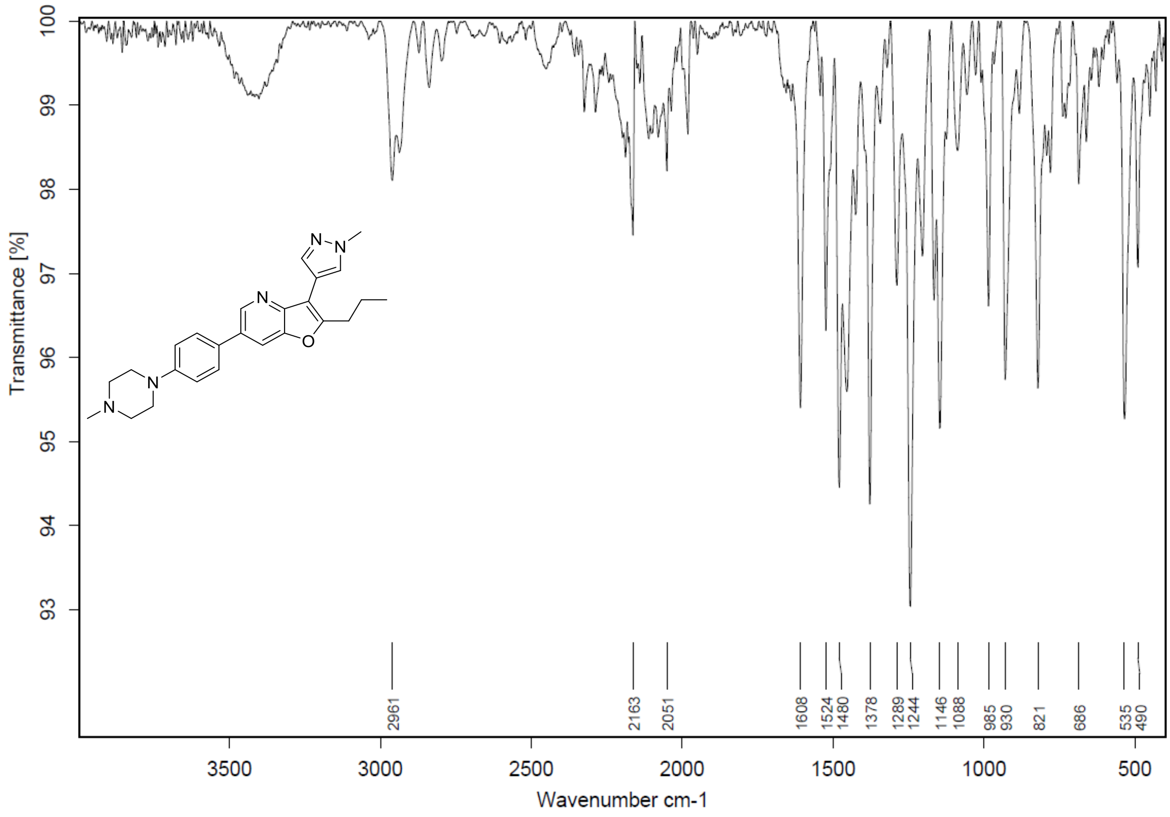

HRMS spectrum of **96.**

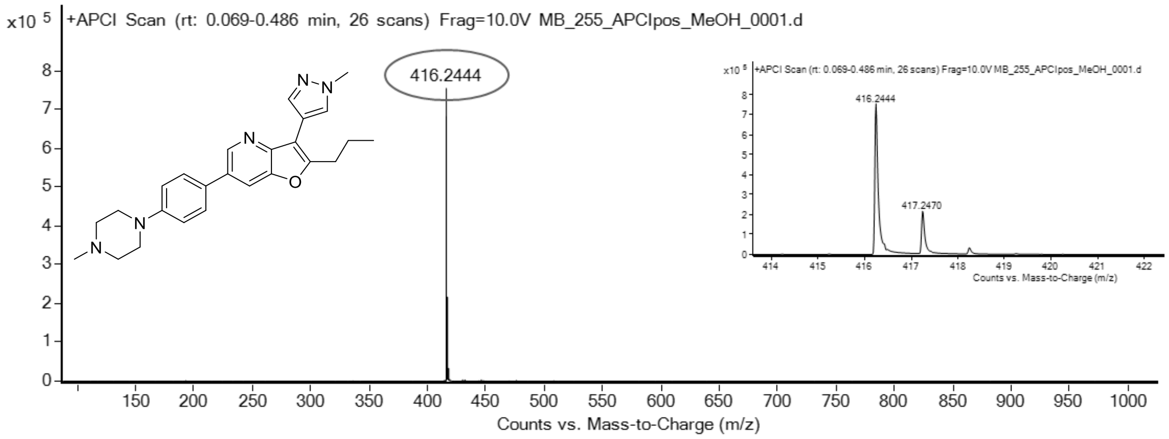

^{1}H (500 MHz) and ^{13}C NMR (126 MHz) spectra of **97** (**M4K2234NC**) in DMSO-*d6*.

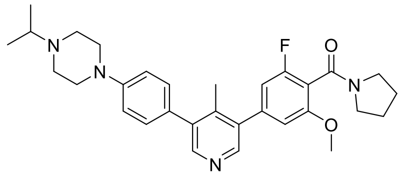

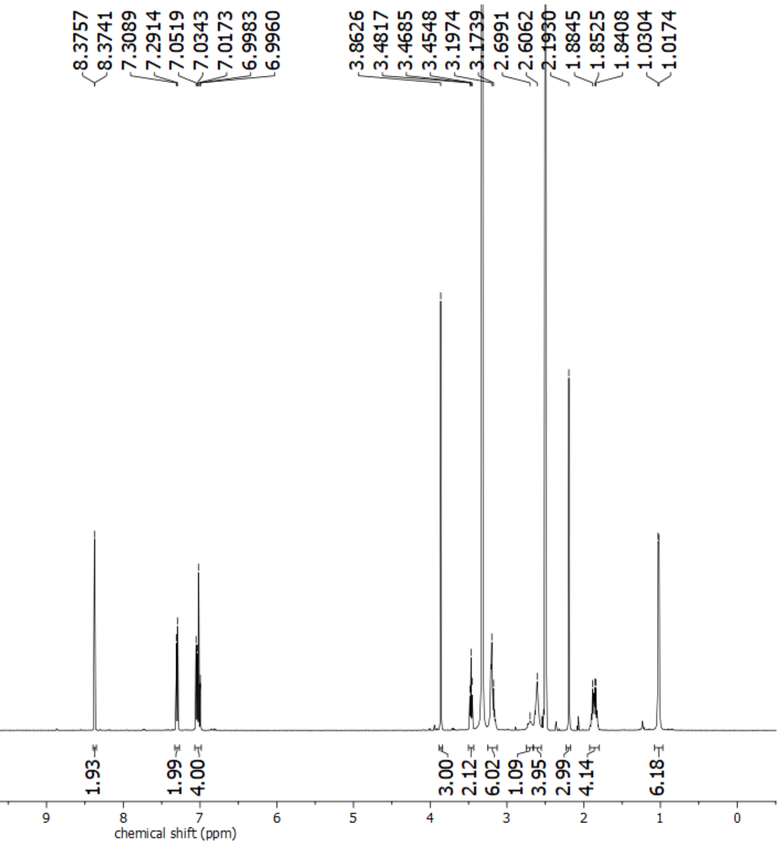

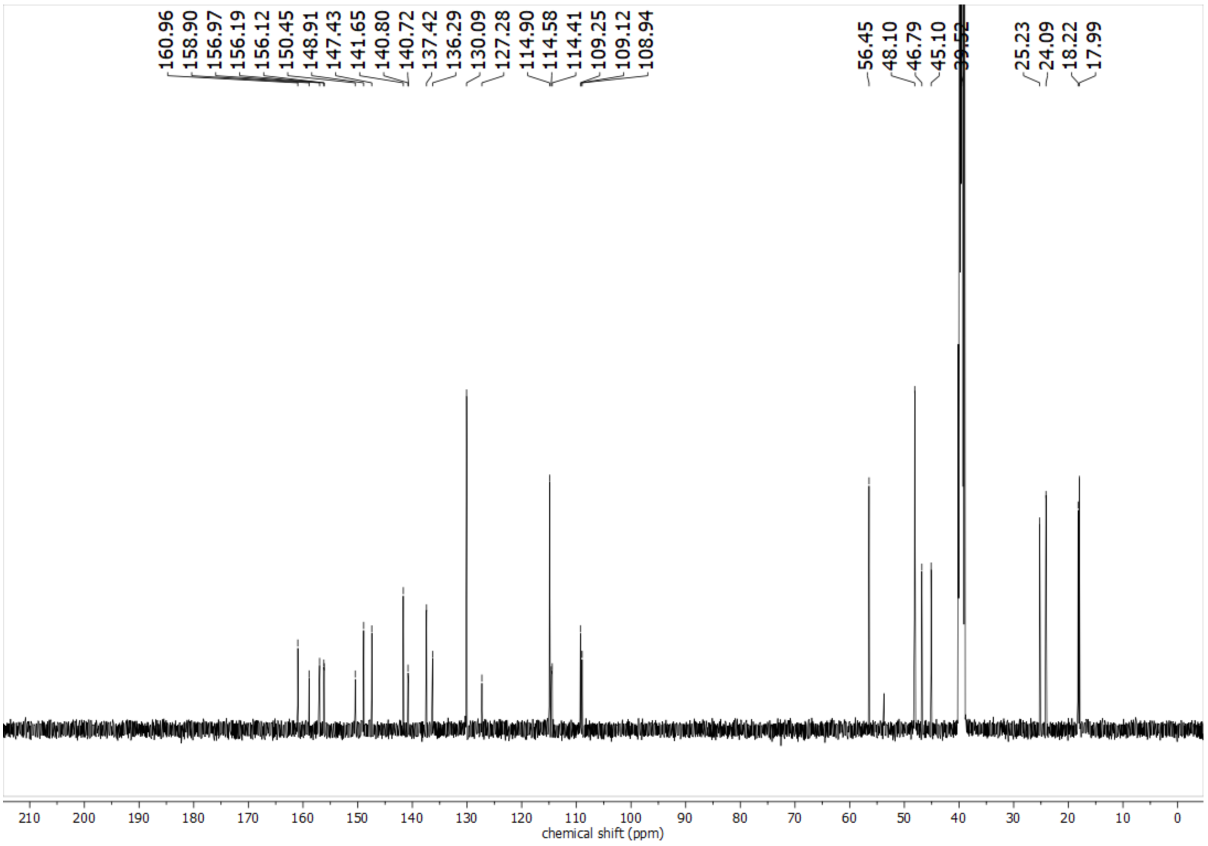

HRMS spectrum of **97** (**M4K2234NC.)**

Acquisition Parameter

Source Type	ESI	Ion Polarity	Positive	Set Nebulizer	1.8 Bar
Focus	Active	Set Capillary	4500 V	Set Dry Heater	220 °C
Scan Begin	50 m/z	Set End Plate Offset	-500 V	Set Dry Gas	8.0 l/min
Scan End	1300 m/z	Set Collision Cell RF	150.0 Vpp	Set Divert Valve	Waste

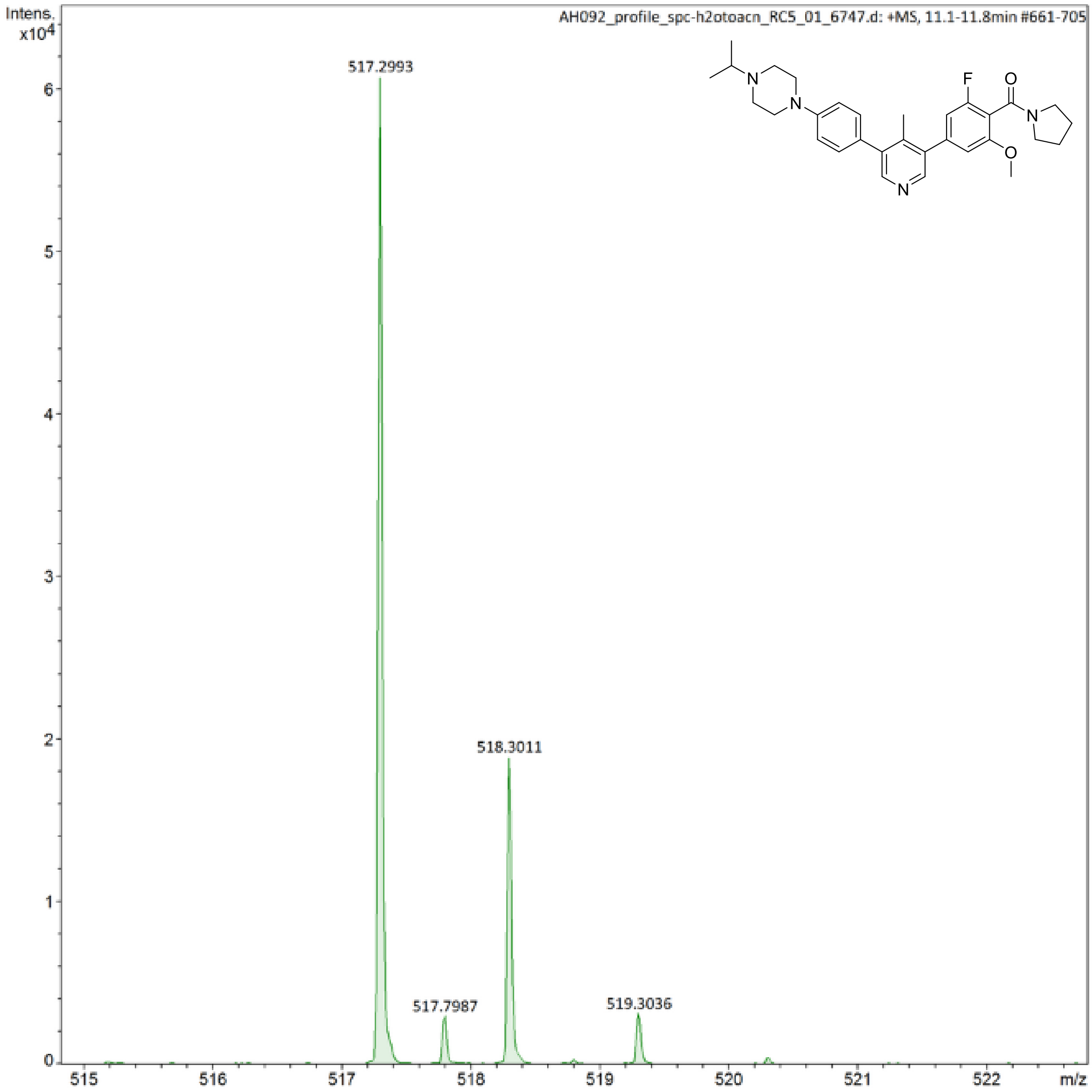

18. HPLC traces of MU1700 and M4K2234

Instrument:

HPLC Agilent 1260 Infinity II which was coupled to G6125B MS detector unit.

General conditions:

Flow:	0.600 mL/min
Low Pressure Limit:	5.00 bar
High Pressure Limit:	600.00 bar
Post time (equilibration between runs):	2.00 min
**Column Guard Description**	
Supplier name:	UHPLC Guard 3PK
Brand:	InfinityLab Poroshell
Product number:	120
Phase:	821725-925
Endcapped:	Bonus-RP
Inner diameter (ID):	Triple endcapped
Length:	2.1 mm
Particle Size:	5 mm
Pore Size:	2.7 µm
	120 Å
**Solvent composition**	
Solvent A:	Water + 0.1 % of formic acid
Solvent B:	MeCN + 0.1 % of formic acid
**Set wavelengths of DAD channels**	**Band Mid, bandwidth**
245-395 nm	320 nm, 150 nm
**MSD parameters**	
Ionization mode:	API-ES
Polarity:	Positive
Mass range:	100-1000 Da
Step size:	0.10 Da

Conditions for analysis of MU1700:
Solvent Gradient:

Time (min)	Solvent A (%)	Solvent B (%)
0.0	95	5
0.4	95	5
6.3	0	100
7.50	0	100

Column:

Supplier name:	InfinityLab Poroshell 120 Bonus-RP
Product number:	695768-901T
Phase:	Bonus-RP
Inner diameter:	2.1 mm
Length:	100.0 mm
Particle size:	2.7 µm

Conditions for analysis of M4K2234:

Solvent Gradient:

Time (min)	Solvent A (%)	Solvent B (%)
0.0	95	5
0.4	95	5
8.0	0	100
10.0	0	100

Column:

Supplier name:	Poroshell 120 EC-C18
Product number:	693575-302
Phase:	Reverse Phase
Inner diameter:	3.0 mm
Length:	150.0 mm
Particle size:	2.7 µm

Column Information

**Column name:**	Poroshell 120 EC-C18, 1000 bar		
**Serial #:**	USJYZ02256	**Length:**	150.0 mm
**Diameter:**	3.00 mm	**Void Time:**	min
**Dead volume:**	0.640 ml		

**Data file:**	W:\analytical_LCMS_DATA\VN\VN2090B (1) 2021-04-22 20-46-12.D		
**Sample name:**	VN2090B		
**Description:**			
**Sample amount:**	0.000	**Sample type:**	Sample
**Instrument:**	LCMS test	**Location:**	D1F-A9
**Injection date:**	2021-04-22 20:48:17+02:00	**Injection:**	1 of 1
		**Injection volume:**	1.000
**Acq. method:**	SLOW_General_Method_MS-PosScan-100-1000.M		
**Analysis method:**	condo.over_night.M		

**Module**	**Type**	**Part. No.**	**Serial No.**	**Firmware**
Agilent G6125B MSD	Detector	G6125B	<undef>	3.02.50
Quat. Pump	Pump	G7104C	DEAGZ01778	B.07.30 [0005]
Column Comp.	Column compartment	G7116A	DEAEM06531	D.07.30 [0005]
DAD	Detector	G7117C	DEAEK07274	D.07.30 [0005]
Multisampler	Auto sampler	G7167A	DEAGY01736	D.07.32 [0001]

Results

Sample Name VN2090B

Chromatograms

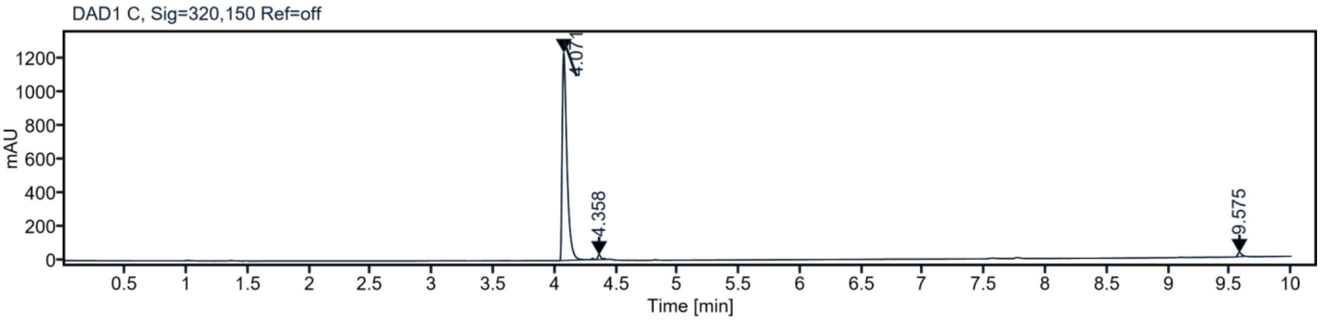

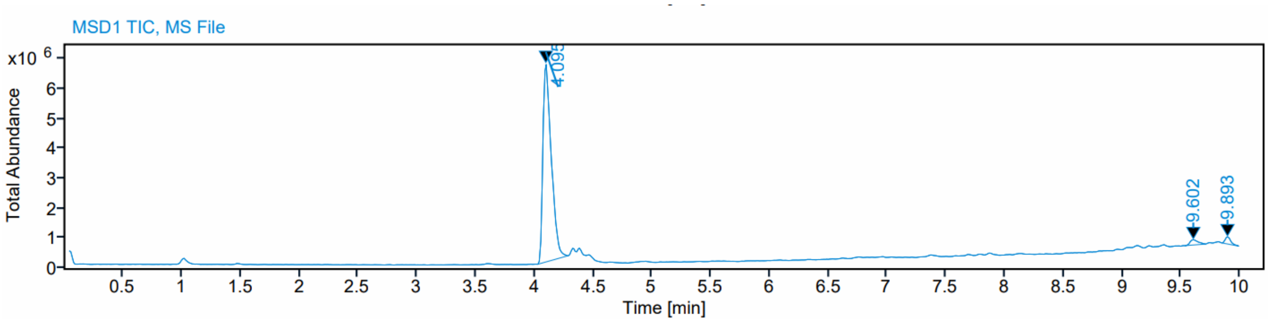

Sample Purity

Signal Description DAD1 C, Sig=320,150 Ref=off

Sample Name	Name	RT	Width	Area	Area%	Height
VN2090B		4.071	0.040	3310.9290	96.43	1232.5939
VN2090B		4.358	0.026	68.5149	2.00	29.5920
VN2090B		9.575	0.036	54.1653	1.58	24.6725

Max Area% 96.427

UV Signal Purity>95% Pass

UV Apex Spectra

RT:

4.071

Sample Name:

VN2090B

Signal Name:

DAD1C

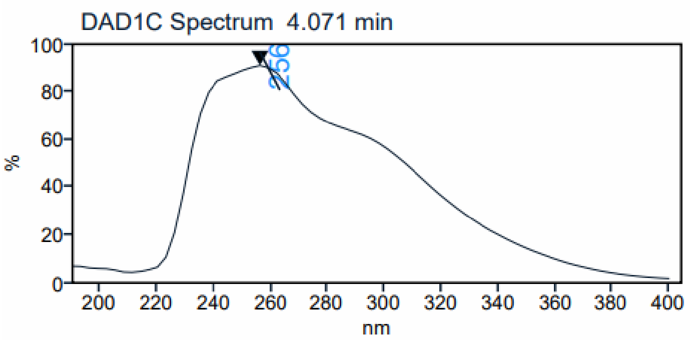

RT:

4.358

Sample Name:

VN2090B

Signal Name:

DAD1C

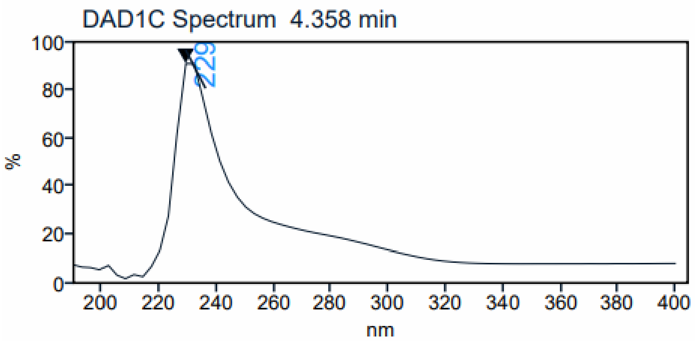

RT:

9.575

Sample Name:

VN2090B

Signal Name:

DAD1C

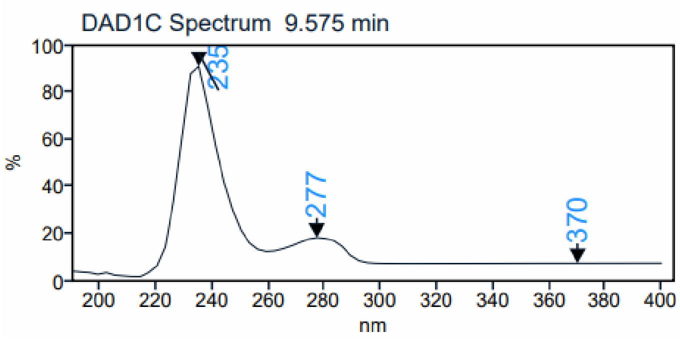

MSD Apex Spectra

RT:

4.095

Sample Name:

VN2090B

Signal Name:

MSD1TIC

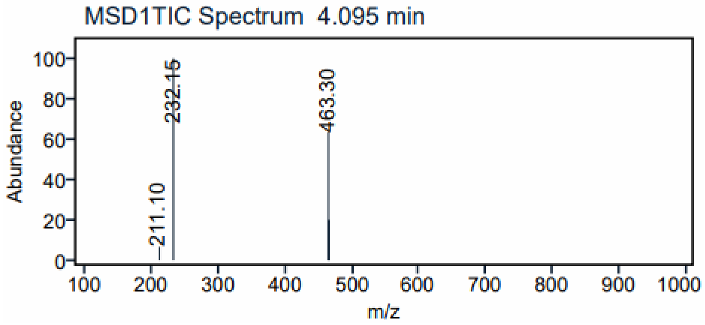

RT:

9.602

Sample Name:

VN2090B

Signal Name:

MSD1TIC

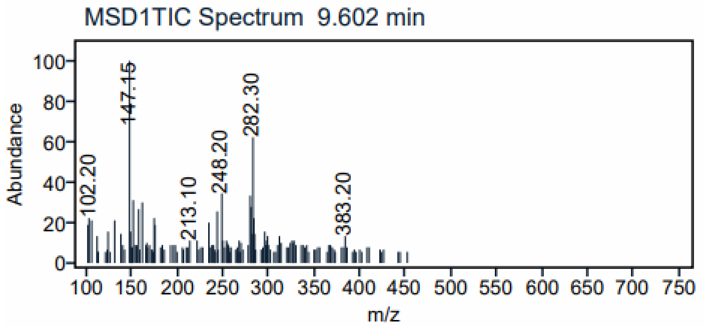

RT:

9.893

Sample Name:

VN2090B

Signal Name:

MSD1TIC

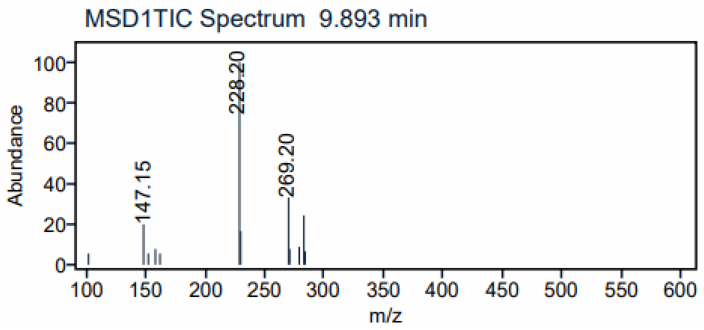

Sample Summary

Line#	Inj#	Location	Sample Name	Datafile	Injection Acq Method Name
2	1	D2F-B1	MU1700	002-D2F-B1-MU1700.D	CGL_FIRSTPASS_GENERALMETHOD_VIAL1+2_7-8-2023.M

Results

Sample Name MU1700

Chromatograms

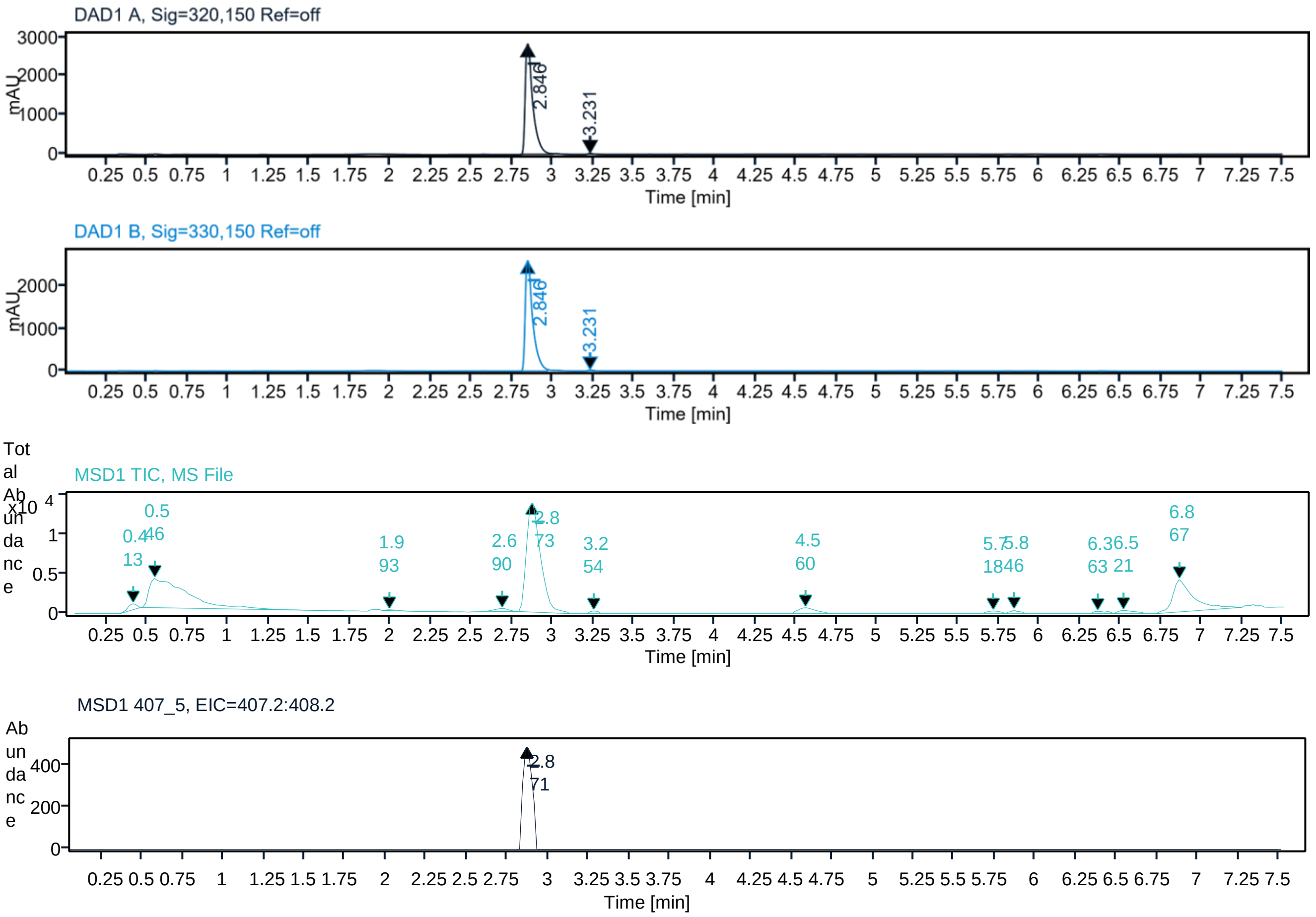

Sample Purity

Signal Description DAD1 A, Sig=320,150 Ref=off

Sample Name	Name	RT	Width	Area	Area%	Height
MU1700		2.846	0.046	9145.7822	99.82	2817.1724
MU1700		3.231	0.023	16.8498	0.18	11.5064

Max Area% 99.816

UV Signal Purity>95% Pass

Signal Description DAD1 B, Sig=330,150 Ref=off

Sample Name	Name	RT	Width	Area	Area%	Height
MU1700		2.846	0.045	8450.2910	99.81	2622.6477
MU1700		3.231	0.022	16.1874	0.19	10.6270

Max Area% 99.809

UV Signal Purity>95% Pass

UV Apex Spectra

RT:

2.846

Sample Name:

MU1700

Signal Name:

DAD1A

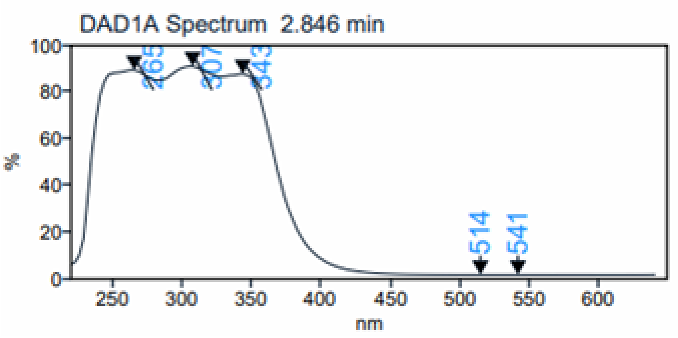

RT:

3.231

Sample Name:

MU1700

Signal Name:

DAD1A

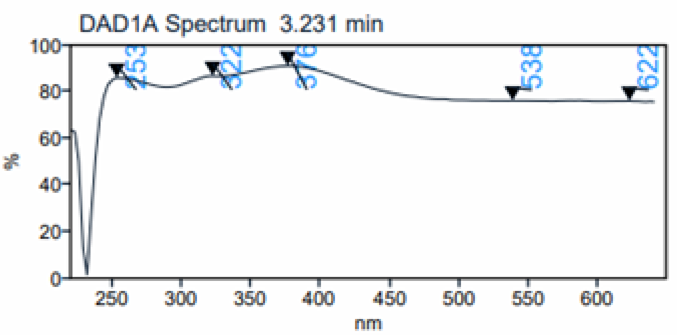

RT:

2.846

Sample Name:

MU1700

Signal Name:

DAD1B

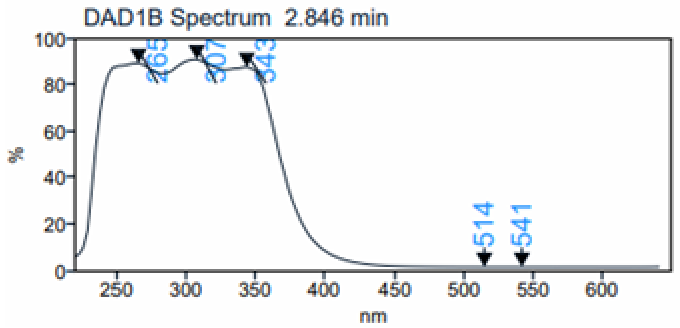

RT:

3.231

Sample Name:

MU1700

Signal Name:

DAD1B

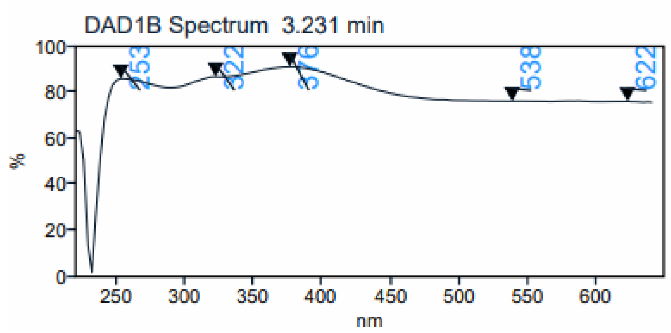

MSD Apex Spectra

RT:

0.413

Sample Name:

MU1700

Signal Name:

MSD1TIC

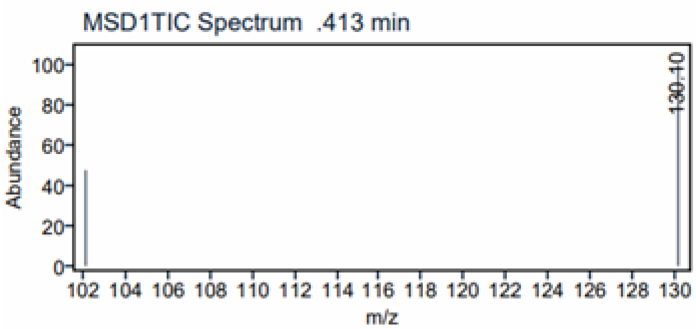

RT:

0.546

Sample Name:

MU1700

Signal Name:

MSD1TIC

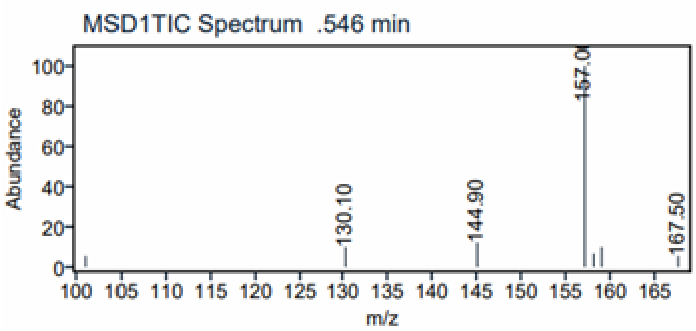

RT:

1.993

Sample Name:

MU1700

Signal Name:

MSD1TIC

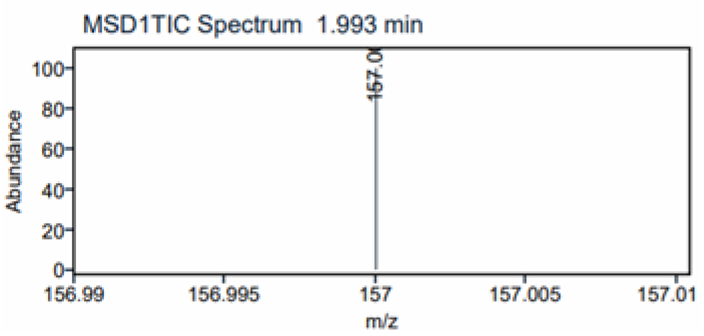

RT:

2.690

Sample Name:

MU1700

Signal Name:

MSD1TIC

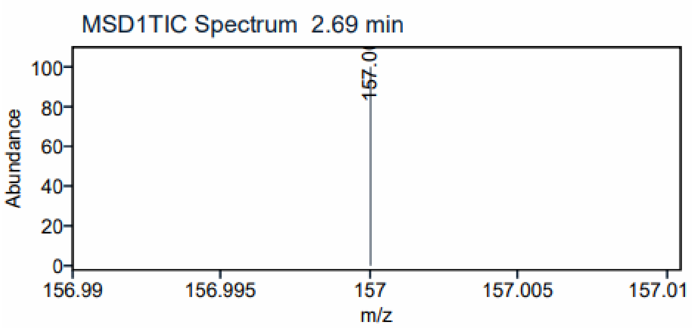

RT:

2.873

Sample Name:

MU1700

Signal Name:

MSD1TIC

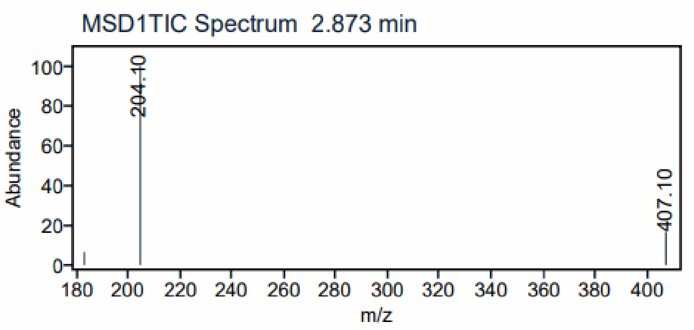

RT:

3.254

Sample Name:

MU1700

Signal Name:

MSD1TIC

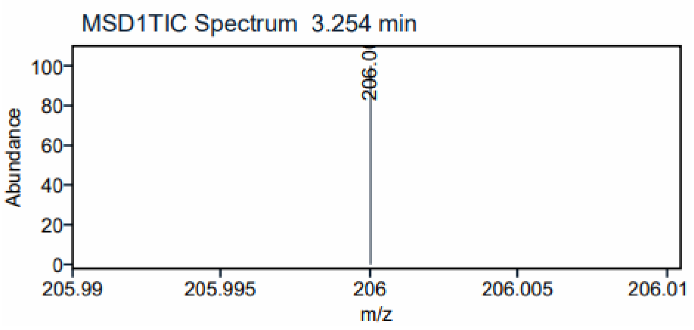

RT:

4.560

Sample Name:

MU1700

Signal Name:

MSD1TIC

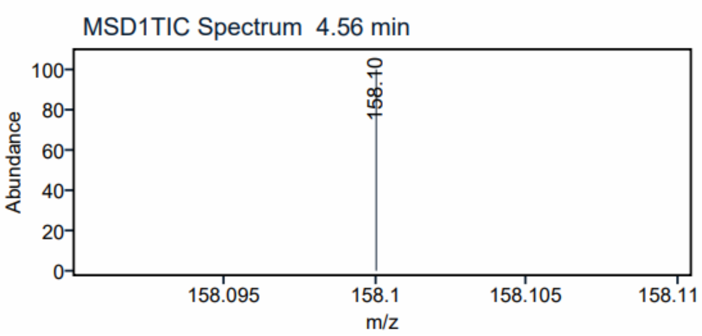

RT:

5.718

Sample Name:

MU1700

Signal Name:

MSD1TIC

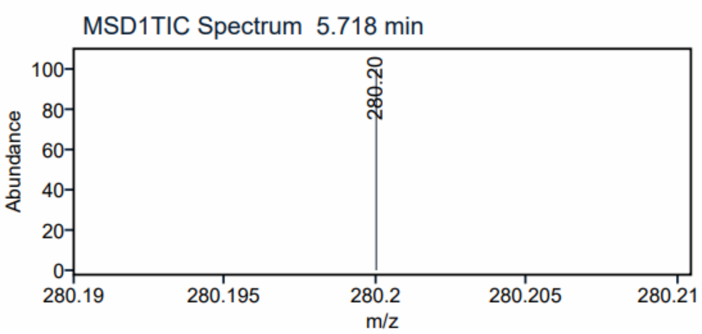

RT:

5.846

Sample Name:

MU1700

Signal Name:

MSD1TIC

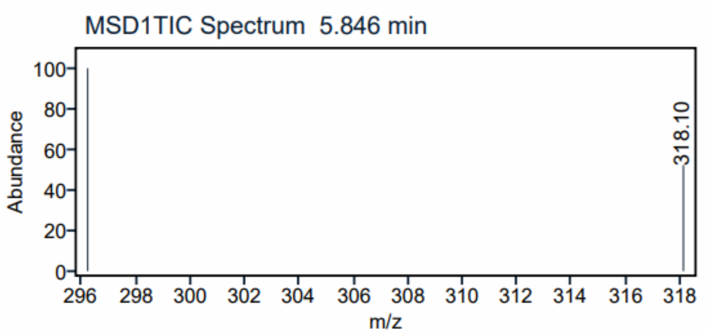

RT:

6.363

Sample Name:

MU1700

Signal Name:

MSD1TIC

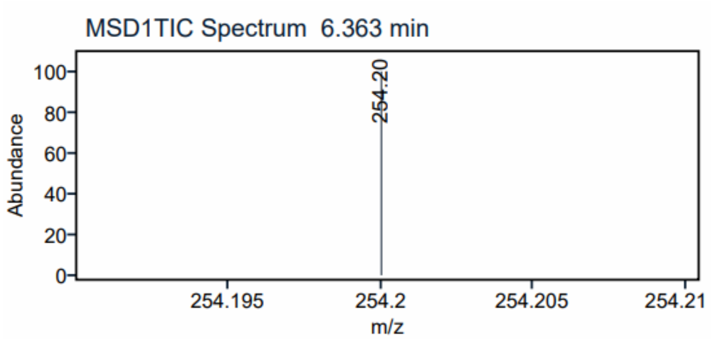

RT:

6.521

Sample Name:

MU1700

Signal Name:

MSD1TIC

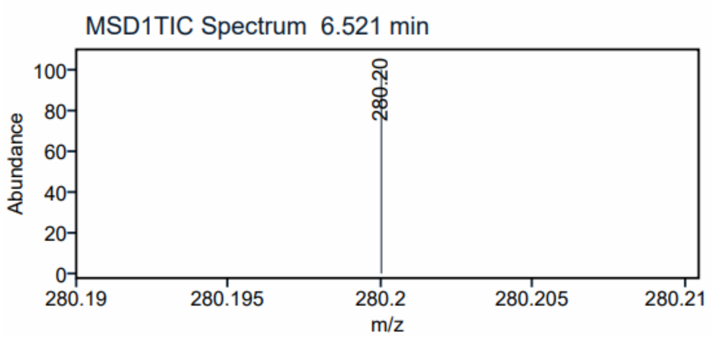

RT:

6.867

Sample Name:

MU1700

Signal Name:

MSD1TIC

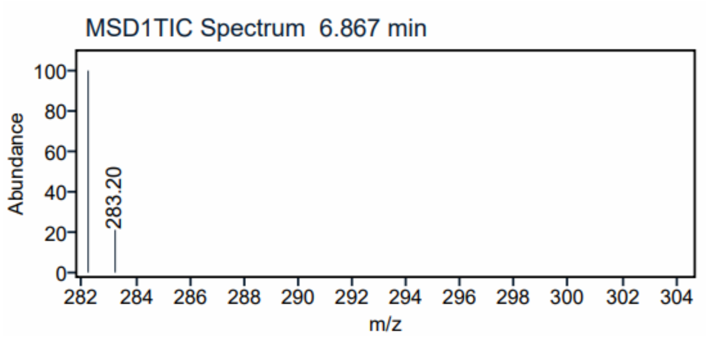

19. Molecular formula strings

**7**	**[H]N(CC1)CCN1C(C=C2)=CC=C2C3=CN=C4C(OC=C4C5=C(C=CC=C6)C6=NC=C5)=C3**
**10**	**[H]N(CC1)CCN1C(C=C2)=CC=C2C3=CN=C4C(OC=C4C5=C(N=CC=C6)C6=CC=C5)=C3**
**14**	**[H]N(CC1)CCN1C(C=C2)=CC=C2C3=CN=C4C(OC=C4C5=C(C=CC=C6)C6=NC(C)=C5)=C3**
**18**	**[H]N(CC1)CCN1C(C=C2)=CC=C2C3=CN=C4C(OC=C4C5=C(C=CC=C6)C6=NC(C(F)(F)F)=C5)=C3**
**21**	**[H]N(CC1)CCN1C(C=C2)=CC=C2C3=CN=C4C(OC=C4C5=C(C=CC=C6)C6=CC=C5)=C3**
**25**	**[H]N(CC1)CCN1C(C=C2)=CC=C2C3=CN=C4C(OC=C4C5=C(C=CC=C6)C6=CC(C)=C5)=C3**
**28**	**[H]N(CC1)CCN1C(C=C2)=CC=C2C3=CN=C4C(OC=C4C5=C(C=CC=C6)C6=CN=C5)=C3**
**31**	**[H]N(CC1)CCN1C(C=C2)=CC=C2C3=CN=C4C(OC=C4C5=C(C=CN=C6)C6=CC=C5)=C3**
**34**	**[H]N(CC1)CCN1C(C=C2)=CC=C2C3=CN=C4C(OC=C4C5=C(C=CC=N6)C6=CC=C5)=C3**
**37**	**[H]N(CC1)CCN1C(C=C2)=CC=C2C3=CN=C4C(OC=C4C5=C(C=NC=C6)C6=CC=C5)=C3**
**39**	**CN(CC1)CCN1C(C=C2)=CC=C2C3=CN=C4C(OC=C4C5=C(C=CN6)C6=NC=C5)=C3**
**41**	**CN(CC1)CCN1C(C=C2)=CC=C2C3=CN=C4C(OC=C4C5=COC6=C5C=CC=C6)=C3**
**43**	**CN(CC1)CCN1C(C=C2)=CC=C2C3=CN=C4C(OC=C4C5=CSC6=C5C=CC=C6)=C3**
**46**	**[H]N(CC1)CCN1C(C=C2)=CC=C2C3=CN=C4C(OC=C4C5=CC(C)=NC(C)=C5)=C3**
**49**	**[H]N(CC1)CCN1C(C=C2)=CC=C2C3=CN=C4C(OC=C4C5=CC=NC=C5)=C3**
**52**	**[H]N(CC1)CCN1C(C=C2)=CC=C2C3=CN=C4C(OC=C4C5=CC=CN=C5)=C3**
**55**	**[H]N(CC1)CCN1C(C=C2)=CC=C2C3=CN=C4C(OC=C4C5=CC(N)=NC=C5)=C3**
**57**	**CN(CC1)CCN1C(C=C2)=CC=C2C3=CN=C4C(OC=C4C5=CC(C(C)=O)=NC=C5)=C3**
**60**	**[H]N(CC1)CCN1C(C=C2)=CC=C2C3=CN=C4C(OC=C4C5=CC=CC=C5)=C3**
**63**	**[H]N(CC1)CCN1C(C=C2)=CC=C2C3=CN=C4C(OC=C4C5=CC=C(F)C=C5)=C3**
**66**	**[H]N(CC1)CCN1C(C=C2)=CC=C2C3=CN=C4C(OC=C4C5=CC(OC)=CC=C5)=C3**
**69**	**[H]N(CC1)CCN1C(C=C2)=CC=C2C3=CN=C4C(OC=C4C5=CC(C(NC)=O)=CC=C5)=C3**
**70**	**CN(CC1)CCN1C(C=C2)=CC=C2C3=CN=C4C(OC=C4C5=CC=CC=C5)=C3**
**73**	**CN(CC1)CCN1C(C=C2)=CC=C2C3=CN=C4C(OC=C4C5=CC(/C=N/O)=CC=C5)=C3**
**75**	**CN(CC1)CCN1C(C=C2)=CC=C2C3=CN=C4C(OC=C4C5=CC(S(=O)(C)=O)=CC=C5)=C3**
**77**	**CN(CC1)CCN1C(C=C2)=CC=C2C3=CN=C4C(OC=C4C5=CC=C(N(C(N)=O)[H])C=C5)=C3**
**80**	**CN(CC1)CCN1C(C=C2)=CC=C2C3=CN=C4C(OC=C4C5=CC=C(CO)C=C5)=C3**
**83**	**CN(CC1)CCN1C(C=C2)=CC=C2C3=CN=C4C(OC=C4C5=CC=C(CC6=CC=CC=C6)C=C5)=C3**
**86**	**CN(CC1)CCN1C(C=C2)=CC=C2C3=CN=C4C(OC=C4C5=CC=C(C(C)C6=CC=CC=C6)C=C5)=C3**
**89**	**[H]N(CC1)CCN1C(C=C2)=CC=C2C3=CN=C4C(OC=C4C5=CN(C)N=C5)=C3**
**90**	**C1(C2=CC=CC=C2)=CN=C3C(OC=C3C4=CC=NC=C4)=C1**
**91**	**C1(C2=CN=C(N3CCCCC3)C=C2)=CN=C4C(OC=C4C5=CC=NC=C5)=C1**
**96**	**CN(CC1)CCN1C(C=C2)=CC=C2C3=CC(OC(CCC)=C4C5=CN(C)N=C5)=C4N=C3**
**97**	**COC1=CC(C2=C(C)C(C3=CC=C(N4CCN(C(C)C)CC4)C=C3)=CN=C2)=CC(F)=C1C(N5CCCC5)=O**

20. References

1 a) Sanvitale, C. E.; Kerr, G.; Chaikuad, A.; Ramel, M. C.; Mohedas, A. H.; Reichert, S.; Wang, Y.; Triffitt, J. T.; Cuny, G. D.; Yu, P. B.; Hill, C. S.; Bullock, A. N. A new class of small molecule inhibitor of BMP signaling. *PloS One,* **2013**, *8*, e62721; b) Chaikuad, A.; Alfano, I.; Kerr, G.; Sanvitale, C. E.; Boergermann, J. H.; Triffitt, J. T.; von Delft, F.; Knapp, S.; Knaus, P.; Bullock, A. N. Structure of the bone morphogenetic protein receptor ALK2 and implications for fibrodysplasia ossificans progressiva. *J. Biol. Chem.*, **2012**, *287*, 36990.

2 a) Winter, G. xia2: an expert system for macromolecular crystallography data reduction. *J. Appl. Crystallogr.* **2010**, *43*, 186; b) Winn, M. D.; Ballard, C. C.; Cowtan, K. D.; Dodson, E. J.; Emsley, P.; Evans, P. R.; Keegan, R. M.; Krissinel, E. B.; Leslie, A. G. W.; McCoy, A.; McNicholas, S. J.; Murshudov, G. N.; Pannu, N. S.; Potterton, E. A.; Powell, H. R.; Read, R. J.; Vagin, A.; Wilson, K. S. Overview of the CCP4 suite and current developments. *Acta Crystallogr., Sect. D: Biol. Crystallogr.* **2011**, *67*, 235.

3 McCoy, A. J. Solving structures of protein complexes by molecular replacement with phaser. *Acta. Crystallogr., Sect. D. Biol. Crystallogr.* **2007**, *63*, 32.

4 Adams, P. D.; Afonine, P. V.; Bunkoczi, G.; Chen, V. B.; Davis, I. W.; Echols, N.; Headd, J. J.; Hung, L. W.; Kapral, G. J.; Grosse-Kunstleve, R. W.; McCoy, A. J.; Moriarty, N. W.; Oeffner, R.; Read, R. J.; Richardson, D. C.; Richardson, J. S.; Terwilliger, T. C.; Zwart, P. H. PHENIX: a comprehensive Python-based system for macromolecular structure solution. *Acta. Crystallogr., Sect. D. Biol. Crystallogr.*, **2010**, *66*, 213.

5 Emsley, P.; Cowtan, K. Coot: model-building tools for molecular graphics. *Acta. Crystallogr., Sect. D. Biol. Crystallogr.*, **2004**, *60*, 2126.

6 Davis, I. W.; Leaver-Fay, A.; Chen, V. B.; Block, J. N.; Kapral, G. J.; Wang, X.; Murray, L. W.; Arendall, W. B. 3rd; Snoeyink, J.; Richardson, J. S.; Richardson, D. C. MolProbity: all-atom contacts and structure validation for proteins and nucleic acids. *Nucleic Acids Res.*, **2007**, *35*, W375.

7 a) Winn, M. D.; Ballard, C. C.; Cowtan, K. D.; Dodson, E. J.; Emsley, P.; Evans, P. R.; Keegan, R. M.; Krissinel, E. B.; Leslie, A. G. W.; McCoy, A.; McNicholas, S. J.; Murshudov, G. N.; Pannu, N. S.; Potterton, E. A.; Powell, H. R.; Read, R. J.; Vagin, A.; Wilson, K. S. Overview of the CCP4 suite and current developments. *Acta Crystallogr., Sect. D: Biol. Crystallogr.*, **2011**, *67*, 235, b) Vonrhein, C.; Flensburg, C.; Keller, P.; Sharff, A.; Smart, O.; Paciorek, W.; Womack, T.; Bricogne, G.; Data processing and analysis with the autoPROC toolbox. *Acta Crystallogr., Sect. D: Biol. Crystallogr.*, **2011**, *67*, 293.

8 Vasta, J. D.; Corona, C. R.; Wilkinson, J.; Zimprich, C. A.; Hartnett, J. R.; Ingold, M. R.; Zimmerman, K.; Machleidt, T.; Kirkland, T. A.; Huwiler, K. G.; Ohana, R. F.; Slater, M.; Otto, P.; Cong, M.; Wells, C. I.; Berger, B. T.; Hanke, T.; Glas, C.; Ding, K.; Drewry, D. H.; Robers, M. B. Quantitative, wide-spectrum kinase profiling in live cells for assessing the effect of cellular ATP on target engagement. *Cell Chem. Biol.*, **2018**, *25*(2), 206.

9 Zilberberg, L.; ten Dijke, P.; Sakai, L. Y.; Rifkin, D. B. A rapid and sensitive bioassay to measure bone morphogenetic protein activity. *BMC cell biol.* **2007**, *8*, 41.

10 Oida, T.; Weiner, H. L. Murine CD4 T cells produce a new form of TGF-β as measured by a newly developed TGF-β bioassay. *PloS One*, **2011**, *6*(4), e18365.
